# Supplementary material for: Context Specificity in Causal Signaling Networks Revealed by Phosphoprotein Profiling
Source: Cell Syst. 2017 Jan 25;4(1):73–83.e10. doi: 10.1016/j.cels.2016.11.013 (PMC5279869; doi:10.1016/j.cels.2016.11.013)
Supplement: Document S1. Figures S1–S6 and Tables S4–S6 [file mmc1.pdf]

Cell Systems, Volume *4*

Supplemental Information

Context Specificity in Causal Signaling Networks Revealed by Phosphoprotein Profiling

Steven M. Hill, Nicole K. Nesser, Katie Johnson-Camacho, Mara Jeffress, Aimee Johnson, Chris Boniface, Simon E.F. Spencer, Yiling Lu, Laura M. Heiser, Yancey Lawrence, Nupur T. Pande, James E. Korkola, Joe W. Gray, Gordon B. Mills, Sach Mukherjee, and Paul T. Spellman

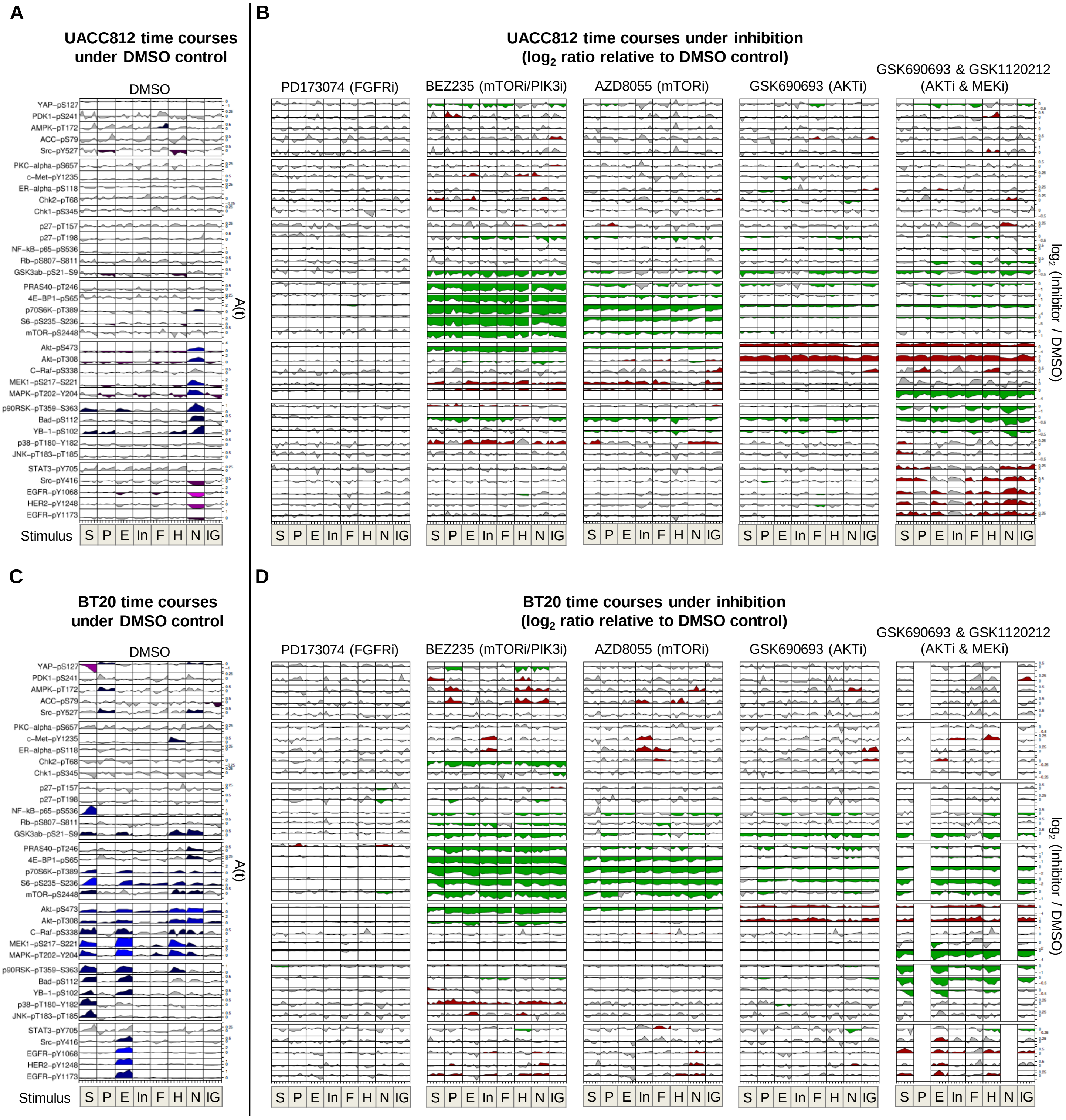

Figure S1 (continued on next page)

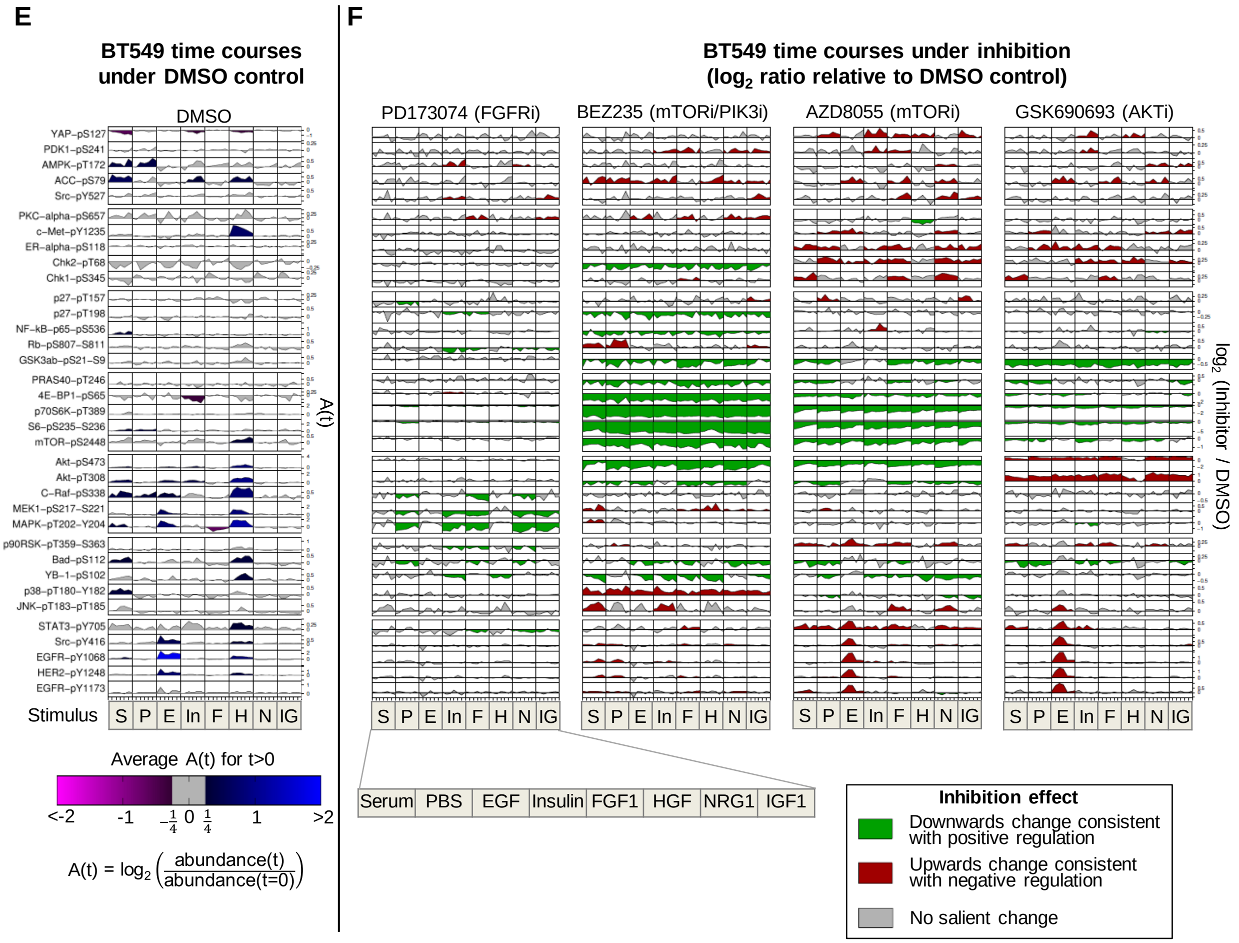

Figure S1. Phosphoprotein Time Course Data and Context-Specific Changes Under Inhibition for Breast Cancer Cell Lines UACC812, BT20 and BT549. Related to Figure 3.

(A,C,E) Phosphoprotein time courses under DMSO control for cell lines UACC812 (A), BT20 (C) and BT549 (E). Rows correspond to 35 phosphoproteins and columns correspond to the eight stimuli. Each time course shows $\log_2$ ratios of phosphoprotein abundance relative to abundance at $t = 0$. Shading represents average $\log_2$ ratio for $t > 0$. (B,D,F) Phosphoprotein time-courses under kinase inhibition for cell lines UACC812 (B), BT20 (D) and BT549 (F). Each of the five vertical blocks corresponds to a different inhibition regime. Within each block, rows and columns are as in (A,C,E). Each time course shows $\log_2$ ratios of phosphoprotein abundance under inhibition relative to abundance under DMSO control. Shading represents direction of changes in abundance due to inhibitor: Green denotes a decrease in abundance, red denotes an increase and gray denotes no salient change. See STAR Methods for details of statistical analysis. There is no data available for the AKTi & MEKi inhibition regime for the following contexts: *(BT549, all stimuli)* and *(BT20, PBS & NRG1)*. Plots were generated using a modified version of the DataRail software (Saez-Rodriguez, 2008). Each phosphoprotein is plotted on its own scale and phosphoproteins are ordered by hierarchical clustering of all data. See Figure 3 for corresponding plots for cell line MCF7.

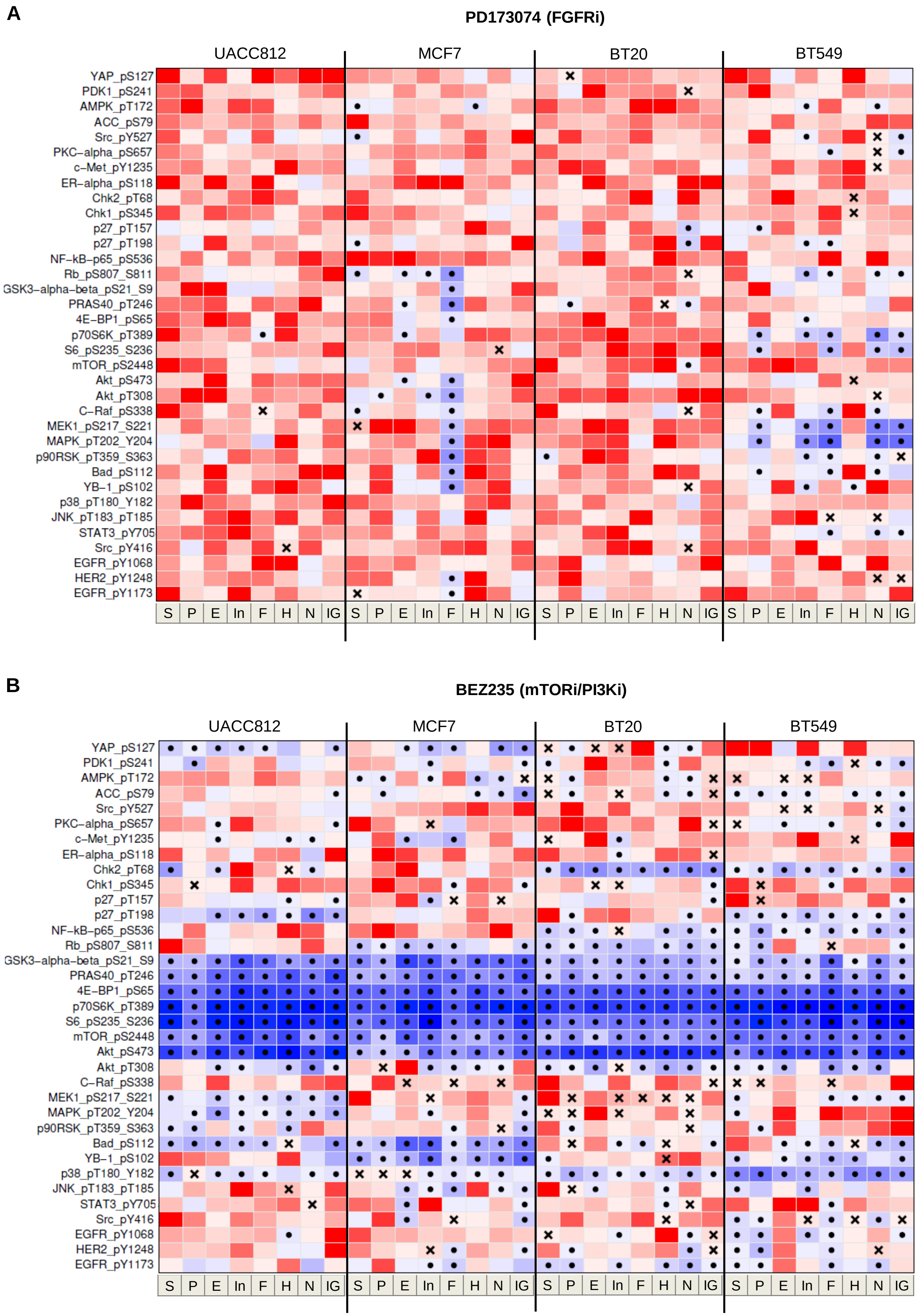

Figure S2 (continued on next page)

C

AZD8055 (mTORi)

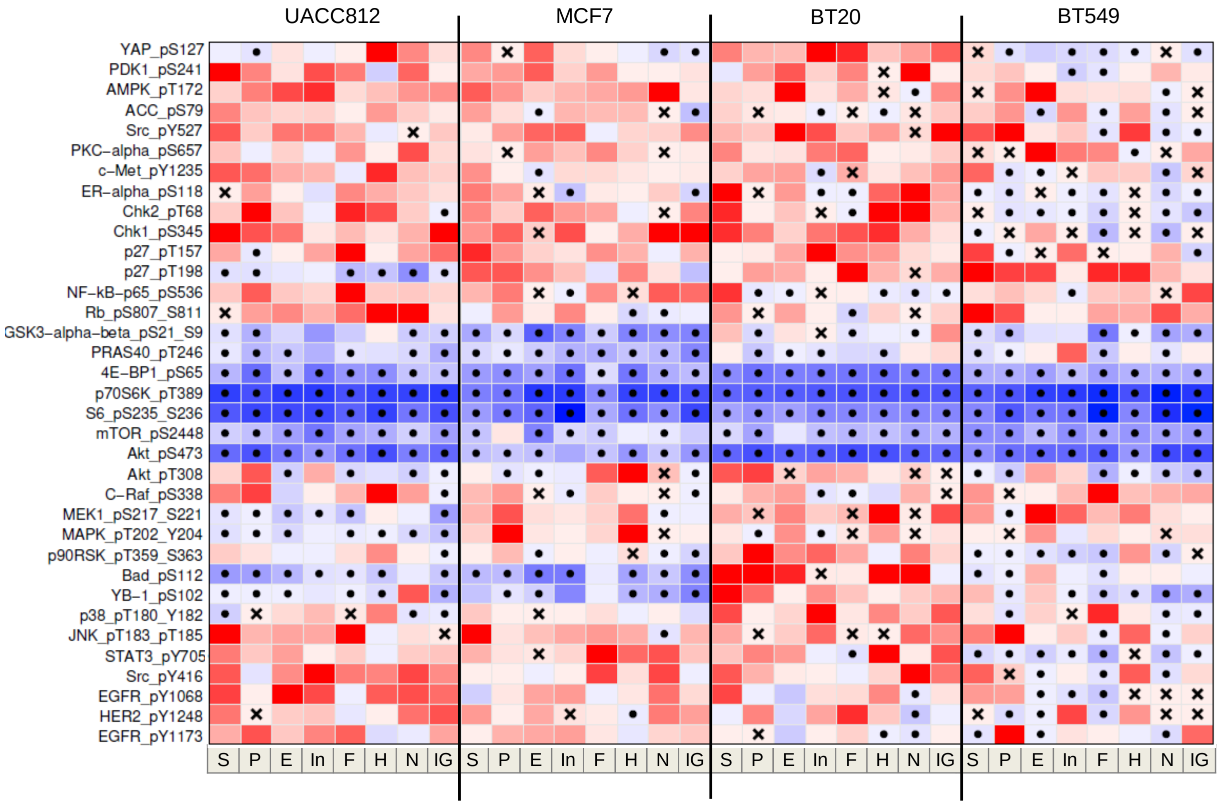

D

GSK690693 (AKTi)

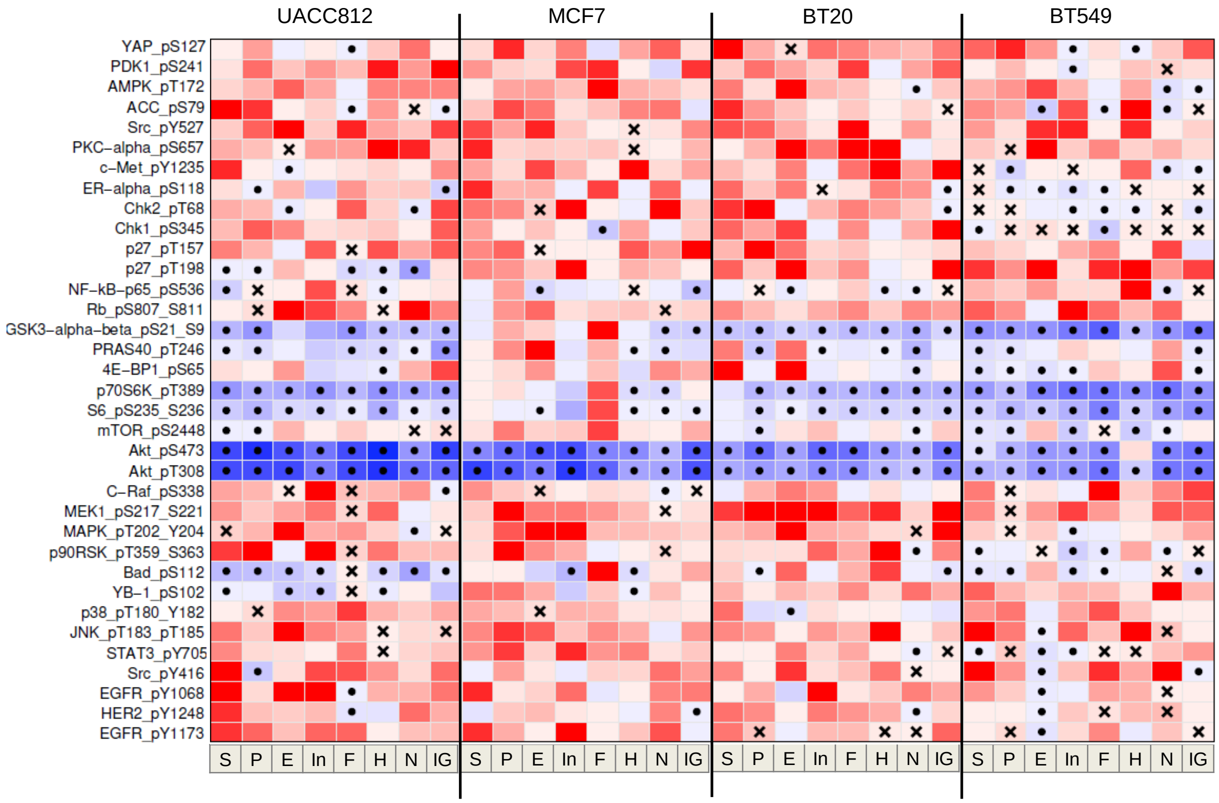

Figure S2 (continued on next page)

E

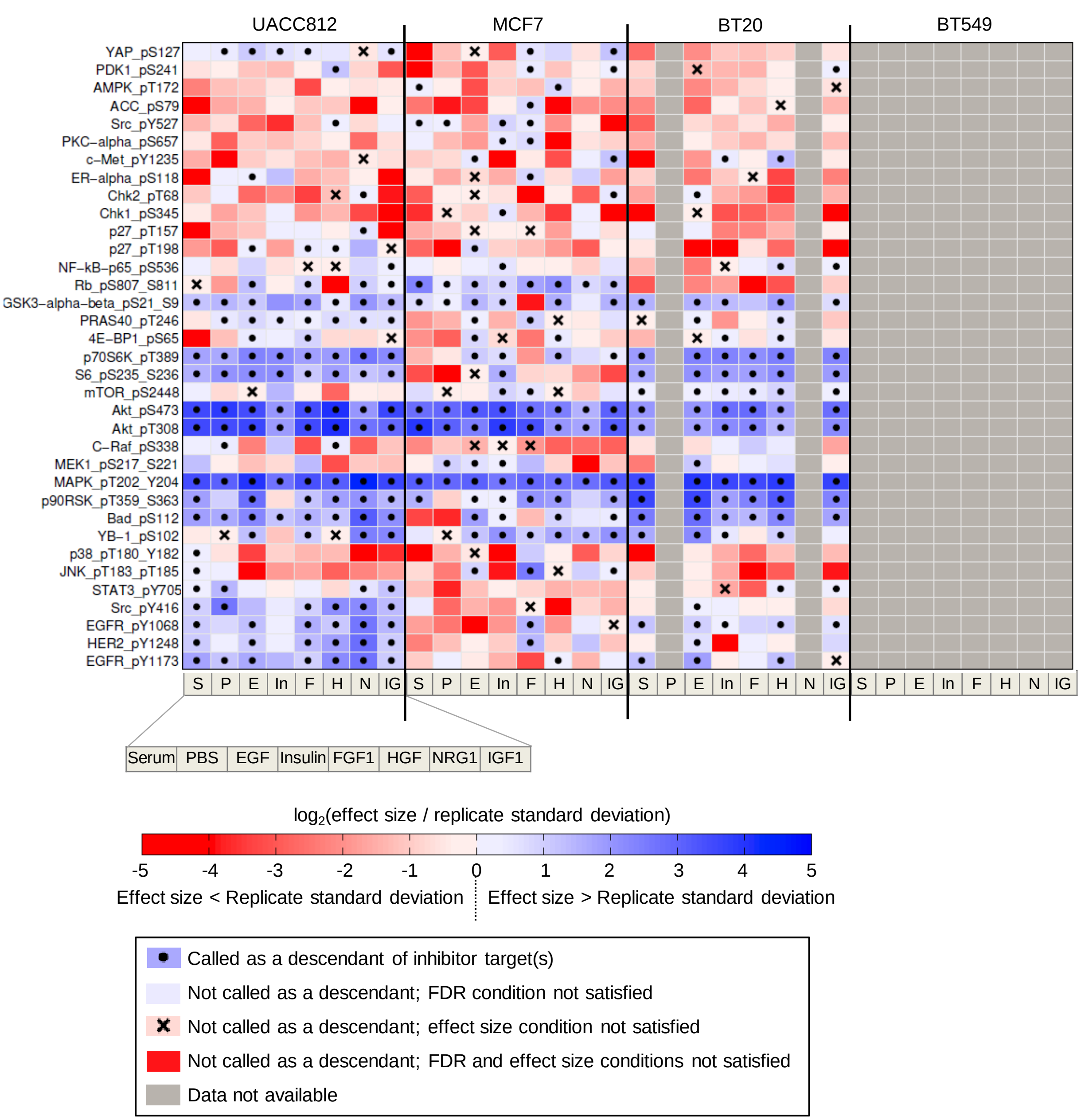

Figure S2. Change under inhibition effect sizes relative to replicate variation. Related to Figure 3. Log_2 ratio of change under inhibition effect size relative to replicate variation for each phosphoprotein in each of the 32 *(cell line, stimulus)* contexts under inhibition of: (A) FGFR (PD173074); (B) mTOR and PI3K (BEZ235); (C) mTOR (AZD8055); (D) AKT (GSK690693); (E) AKT and MEK (GSK690693 and GSK1120212). Rows corresponds to phosphoproteins and columns correspond to contexts. For a given context, a phosphoprotein was deemed to show a salient change under inhibition if two conditions were satisfied (see STAR Methods): (i) FDR < 5% (from paired *t*-test comparing DMSO and inhibitor time courses) and (ii) inhibition effect size > replicate standard deviation. Cells shaded blue indicate that condition (ii) is satisfied. Blue cells with black dots denote phosphoproteins that also satisfy condition (i) and are therefore called as causal descendants of (one of) the inhibitor target(s) in the signaling network for that context. Red cells with black crosses indicate phosphoproteins where condition (i) is satisfied only. Phosphoproteins ordered by output of hierarchical clustering of all data.

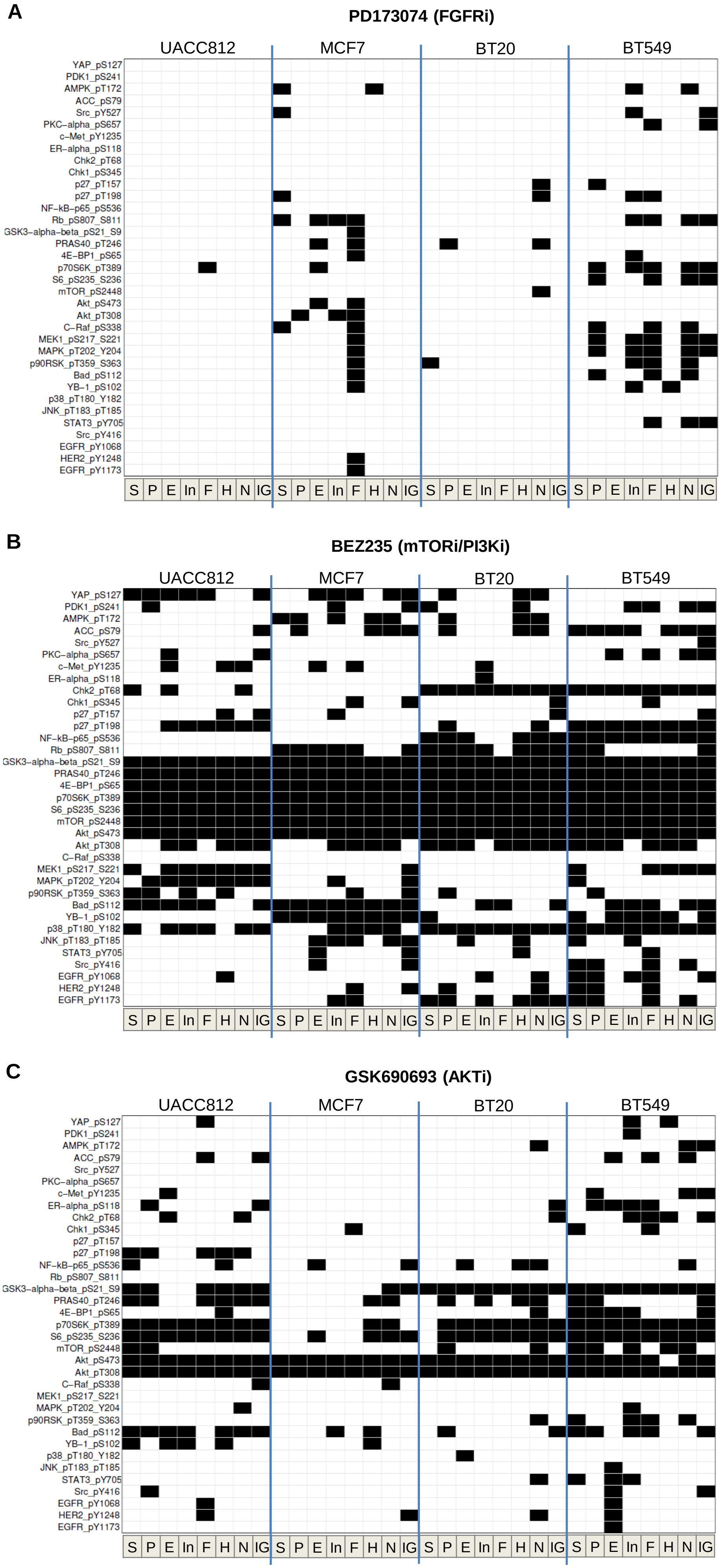

Figure S3 (continued on next page)

D

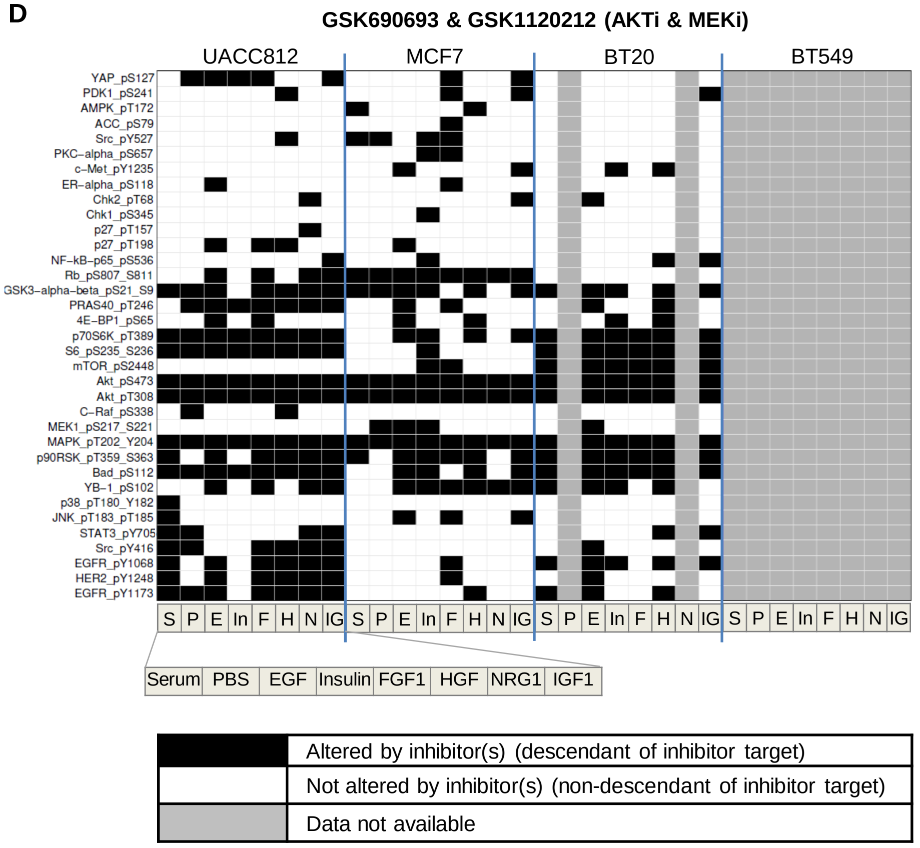

Figure S3. Context-Specific Signaling Revealed by Kinase Inhibition. Related to Figure 4.
Causal descendancy matrices showing causal effects observed in each of the 32 *(cell line, stimulus)* contexts under inhibition of: (A) FGFR (PD173074); (B) mTOR and PI3K (BEZ235); (C) AKT (GSK690693); (D) AKT and MEK (GSK690693 and GSK1120212). Rows correspond to phosphoproteins and columns correspond to contexts. Black cells indicate phosphoproteins that show a salient change under inhibition in a given context (see STAR Methods) and can therefore be regarded as causal descendants of (one of) the inhibitor target(s) in the signaling network for that context. Phosphoproteins ordered by output of hierarchical clustering of all data. Note that black cells correspond to blue cells with black dots in Figure S2. See Figure 4 for the causal descendancy matrix for mTOR inhibitor AZD8055.

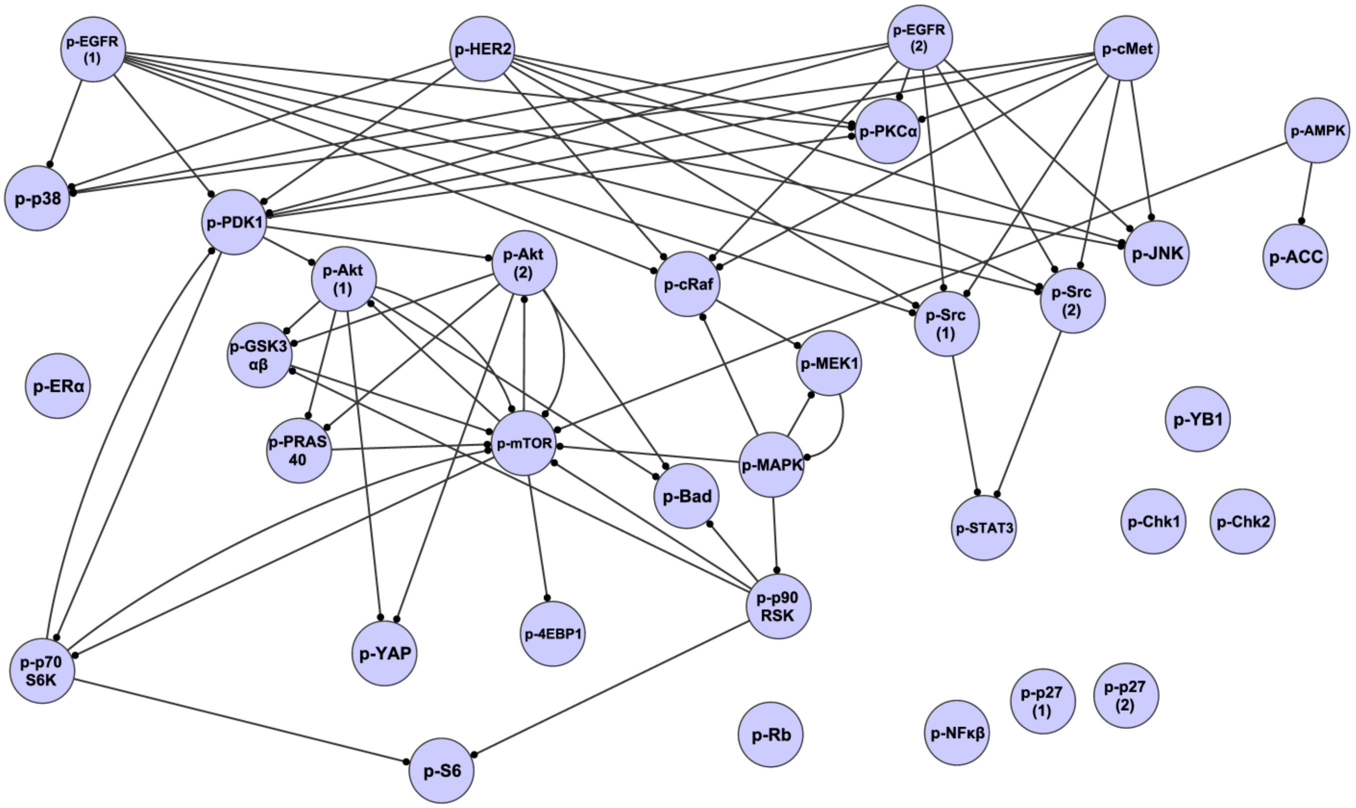

Figure S4. Prior Network Incorporated Within Network Learning. Related to Figure 5.
Known biology was incorporated into network learning using the prior network shown here. The same prior network was used for all contexts and contains canonical information manually curated with input from literature and online resources. Edges are directed with the child node indicated by a circle. The network includes edges describing indirect causal effects mediated via signaling components not included in our study. Full nodes names, including phosphorylation sites, are provided in Table S4.

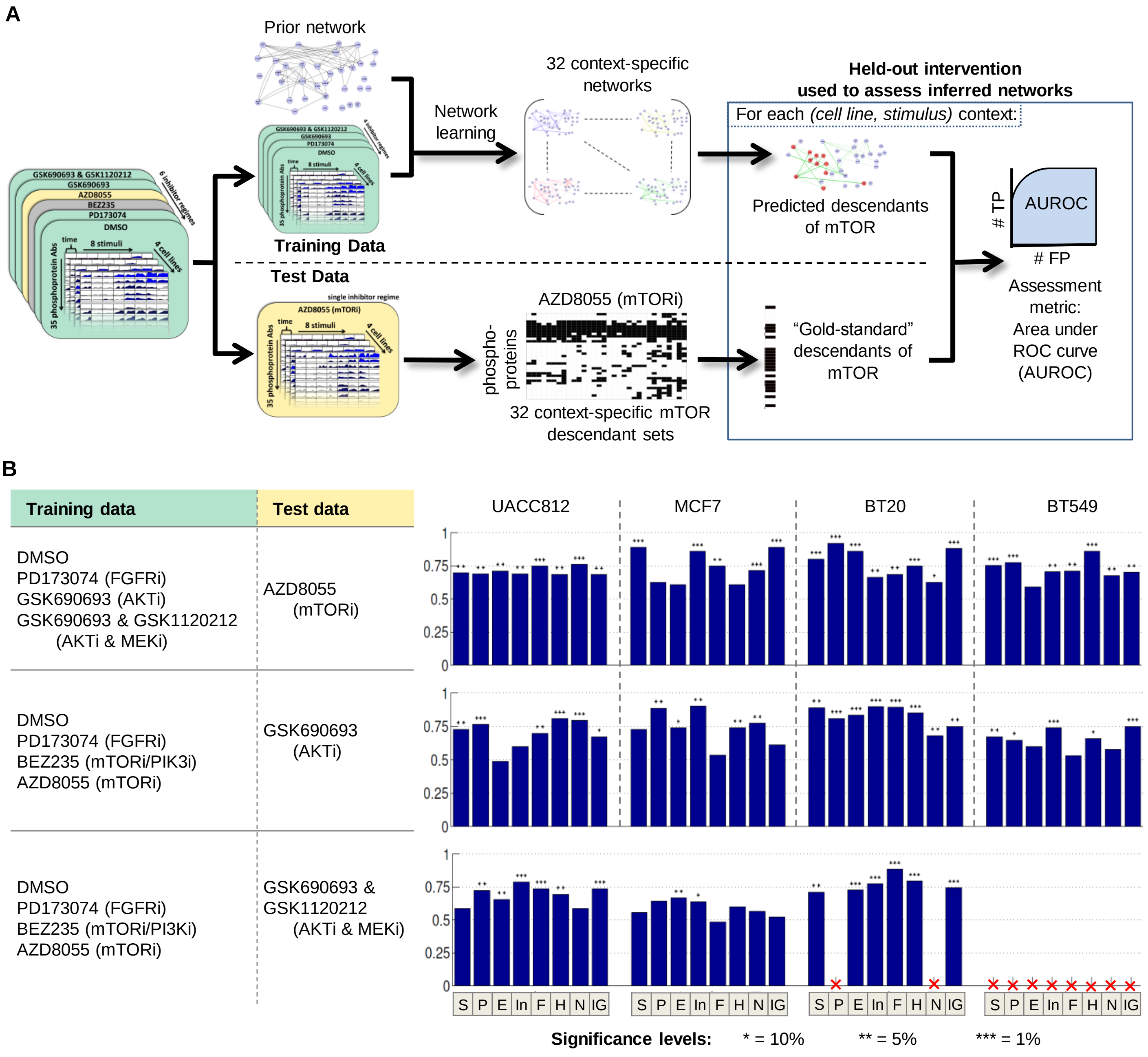

Figure S5. Assessment of Causal Network Learning. Related to STAR Methods.

(A) The ability of our approach to learn causal networks was assessed using an interventional train-and-test approach, following Hill et al. (2016). At each iteration, the data was split into a test set consisting of all data for a single inhibitor regime, and a training set comprising data for all other inhibition regimes (except those that target the same protein(s) as the test inhibitor). The example shown has AZD8055 (mTORi) as the test inhibitor. Thirty-two context-specific networks were inferred by applying the network learning procedure to the training data. The held-out test data were used to determine, for each context, descendants of the test set inhibitor target(s). These "gold-standard" descendant sets were then compared with descendant sets predicted by the inferred context-specific networks, resulting in an AUROC (area under the ROC curve) score for each context. See STAR Methods and Hill et al. (2016) for further details. (B) The procedure in (A) was repeated for three different train/test splits as shown in the table. The bar plots show the corresponding AUROC scores for the 32 contexts. Stars above the bars denote statistical significance of AUROC scores at the 10% (1 star), 5% (2 stars) and 1% (3 stars) level, with respect to a null distribution based on random edge scores. Red crosses indicate contexts where AUROC scores are unavailable due to missing data for the AKTi & MEKi inhibition regime.

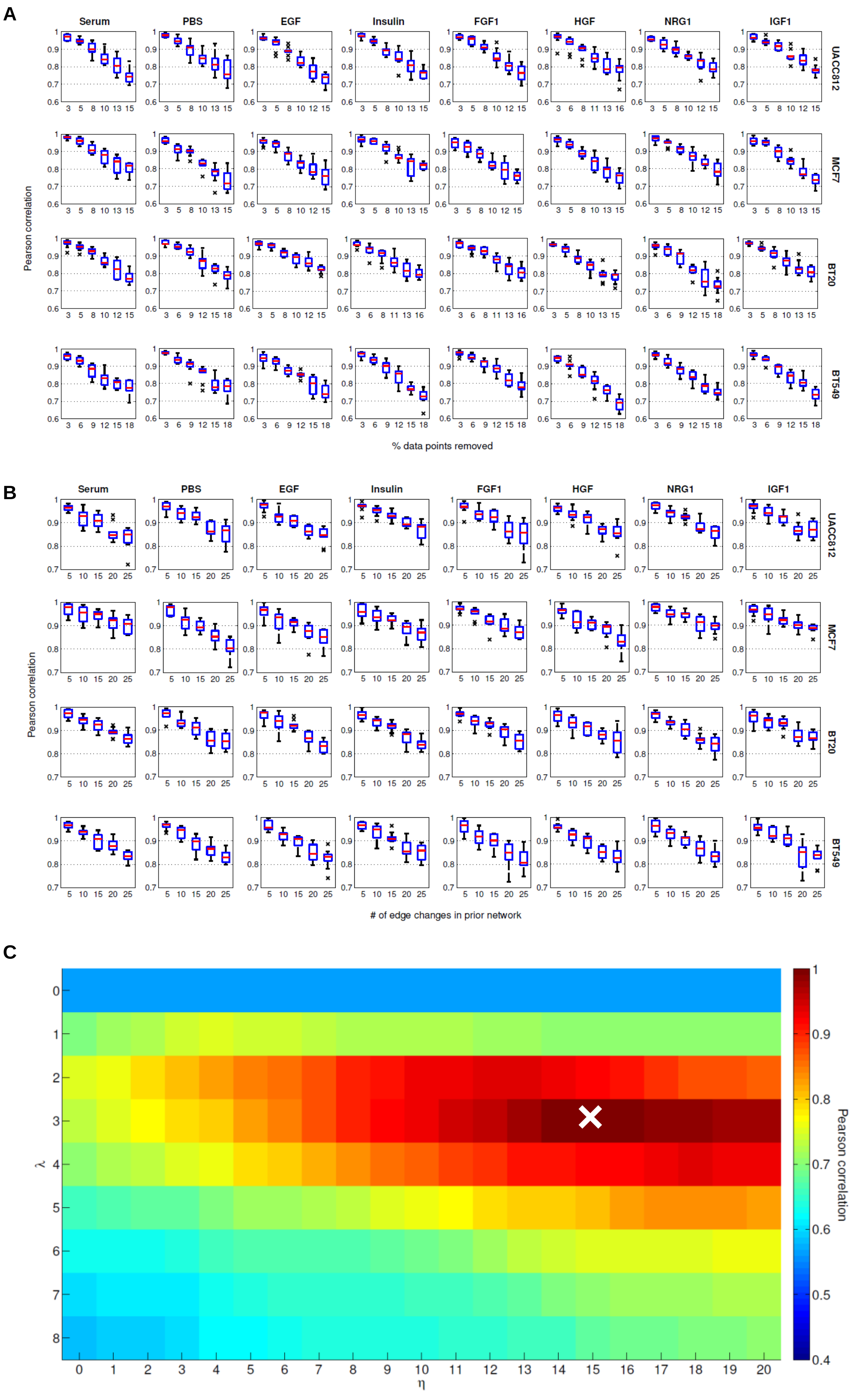

Figure S6 (legend on next page)

Figure S6. Sensitivity of Network Learning to Data Deletion and Prior Network Specification. Related to STAR Methods.

(A) Sensitivity to data deletion. For each *(cell line, stimulus)* context, data were deleted for all 35 phosphoproteins for between one and six time points, selected at random from the time courses for all available inhibitor regimes. Missing data were then imputed by linear interpolation using adjacent time points (for this reason the random selection for deletion excluded the first and last time points in each time series). For each context, Pearson correlation coefficients are shown between posterior edge probabilities inferred using the new, perturbed data sets and those obtained using the original data set. The *x*-axes show the percentage of data points removed. Results shown are over 10 iterations. (B) Sensitivity to prior network edge structure. The prior network (Figure S4) was perturbed by making random edge changes; an edge change consists of removing a randomly selected directed edge and then adding in (a different) randomly selected directed edge. Therefore the overall number of edges remains constant (65 edges). Edge probabilities obtained by incorporating the perturbed prior networks within network learning were compared with those obtained using the original, unperturbed prior network. For each context, Pearson correlations are shown as a function of number of edge changes, each for 10 iterations. (C) Sensitivity to prior strength parameters. Joint learning was done using the formulation in Oates et al. (2014). Parameters λ and η controlled the extent to which context-specific networks agreed with each other and with the prior network respectively. The heatmap shows average Pearson correlations between posterior edge probabilities reported in Main Text, obtained using $(\lambda = 3, \eta = 15)$ and those obtained using alternative (λ, η) values. Averages are calculated over the 32 *(cell line, stimulus)* contexts. The heatmap entry for $(\lambda = 3, \eta = 15)$ therefore has a value of unity and is indicated by a white cross. For $\eta = 0$, the prior network plays no role, while $\lambda = 0$ provides a flat prior distribution over networks (prior network plays no role and no agreement encouraged across contexts).

Table S1. RPPA Antibody List. Related to Figure 2 and STAR Methods.
Details of antibodies contained in each RPPA dataset. The presented analyses focused on phosphoprotein antibodies that were present across all three RPPA datasets, as indicated in the table.

(Supplied as an Excel file)

Table S2. Western Blot Validations of Context-Specific Changes Under Intervention. Related to Figures 4 and S3.
Western blots were carried out to validate a subset of the context-specific changes observed under kinase inhibition in the RPPA data (Figures 4A and S3). Each row in the table reports, for each of the four cell lines under a given stimulus, the change in abundance (relative to DMSO control) of a single phosphoprotein under a given inhibitor. Shown are: (i) changes in abundance determined using the RPPA time-course data (see STAR Methods), with green/red cells denoting a downwards/upwards effect under inhibition respectively and "NE" denotes no observed effect, and (ii) results of attempts to validate the RPPA observations by Western blot (see STAR Methods), with blue cells denoting observations that successfully validated and yellow cells denoting results that were inconsistent with the RPPA data (blank cells denote untested observations).

(Supplied as an Excel file)

Table S3. List of Directed Edges in Inferred Context-Specific Networks. Related to Figures 5 and 6.
A network specific to each of the 32 *(cell line, stimulus)* contexts is obtained by placing a threshold of 0.2 on the posterior edge probabilities that are the output of the network learning procedure. The table contains all directed edges that appear in at least one of these context-specific networks, together with edge probabilities for each context. Edges are sorted into three groups: (i) edges that are not in the prior network and are not self-edges (edges where the parent node is the same as the child node); (ii) edges that are in the prior network; (iii) self-edges (note that the prior network does not contain any self-edges). Within each of these groups, edges are sorted by average edge probability across all contexts. Also indicated are the edges appearing in the cell line-specific summary networks in Figure 5 and associated average edge probabilities (see columns with headings shaded blue). Gray cells denote (average) edge probabilities below a value of 0.2. Edges for which validation was attempted by Western blot (Figure 6) are highlighted in red. Summary counts for the number of edges in each context-specific network and percentage of "novel" edges (not in the prior network) are provided at the bottom of the table.

(Supplied as an Excel file)

Table S4. Full Antibody Names for Nodes in Network Visualizations. Related to Figures 4B, 5 and S4.

The networks shown in Figures 4B, 5 and S4 use abbreviated antibody names for node labels. The corresponding full antibody names are provided in this table.

Network node label	Full antibody name
p-4EBP1	4E-BP1_pS65
p-ACC	ACC_pS79
p-Akt (1)	Akt_pT308
p-Akt (2)	Akt_pS473
p-AMPK	AMPK_pT172
p-Bad	Bad_pS112
p-Chk1	Chk1_pS345
p-Chk2	Chk2_pT68
p-cMet	c-Met_pY1235
p-cRaf	C-Raf_pS338
p-EGFR (1)	EGFR_pY1173
p-EGFR (2)	EGFR_pY1068
p-ERα	ER-alpha_pS118
p-GSK3αβ	GSK3-alpha-beta_pS21_S9
p-HER2	HER2_pY1248
p-JNK	JNK_pT183_pT185
p-MAPK	MAPK_pT202_Y204
p-MEK1	MEK1_pS217_S221
p-mTOR	mTOR_pS2448
p-NFκβ	NF-kB-p65_pS536
p-p27 (1)	p27_pT157
p-p27 (2)	p27_pT198
p-p38	p38_pT180_Y182
p-p70 S6K	p70S6K_pT389
p-p90 RSK	p90RSK_pT359_S363
p-PDK1	PDK1_pS241
p-PKCα	PKC-alpha_pS657
p-PRAS 40	PRAS40_pT246
p-Rb	Rb_pS807_S811
p-S6	S6_pS235_S236
p-Src (1)	Src_pY527
p-Src (2)	Src_pY416
p-STAT3	STAT3_pY705
p-YAP	YAP_pS127
p-YB1	YB-1_pS102

Table S5. Antibody List for Western Blot Validations of Potentially Novel Edges. Related to Figure 6 and STAR Methods.
Details of the antibodies used in the western blot validations of potentially novel edges.

Desired Target	Company	Catalogue Number	Dilution Used
p27_pT198	Abcam	ab64949	1:500
p38_pT180/Y182	CST	9211	1:500
p70S6K_pT389	CST	9205	1:2,000
B-actin	Santa Cruz	sc-47778	1:30,000
Chk2_pT68	CST	2197	1:1,000
HSP90	BD Trans Labs	610419	1:20,000
JNK_pT183/T185	CST	4668	1:500
NFκβp65_pS653	CST	3033	1:500
YAP_pS127	CST	4911	1:500

Table S6. Samples Excluded from Analyses. Related to STAR Methods.
The table shows the samples excluded from analyses due to not passing quality control. For each sample, a justification for removal is provided (see STAR Methods for details). These samples are also highlighted in the data files in Data S1.

Cell line	Inhibitor	Stimulus	Time point	# replicates removed	Justification
MCF7	GSK690693	n/a	0	2 out of 16 replicates	Outlier (CF score)
UACC812	PD173074	n/a	0	1 out of 16 replicates	Outlier (CF score)
UACC812	GSK690693 & GSK1120212	Insulin	15min	1 out of 1 replicate	Outlier (variance)
UACC812	BEZ235	n/a	0	1 out of 13 replicates	Outlier (SNR analysis)
BT549	GSK690693 & GSK1120212	All	All	All	None of the expected effects of GSK1120212 (MEKi) were observed
BT20	GSK690693 & GSK1120212	PBS, NRG1	All	All	
BT20	GSK690693 & GSK1120212	n/a	0	4 out of 16 replicates	

Data S1. RPPA data. Related to STAR Methods.
A zip archive containing the reverse-phase protein array data generated in this study. See the README file included in the zip archive for further details.

Data S2. RPPA data time-course plots. Related to STAR Methods.
A zip archive containing time-course plots of the reverse-phase protein array data generated in this study. See the README file included in the zip archive for further details.
