# Supplementary material for: Context Specificity in Causal Signaling Networks Revealed by Phosphoprotein Profiling
Source: Cell Syst. 2017 Jan 25;4(1):73–83.e10. doi: 10.1016/j.cels.2016.11.013 (PMC5279869; doi:10.1016/j.cels.2016.11.013)
Supplement: Data S2. RPPA Data Time-Course Plots, Related to STAR Methods — A zip archive containing time-course plots of the reverse-phase protein array data generated in this study. See the README file included in the zip archive for further details. [file mmc6.zip › DataS2/BT20.pdf]

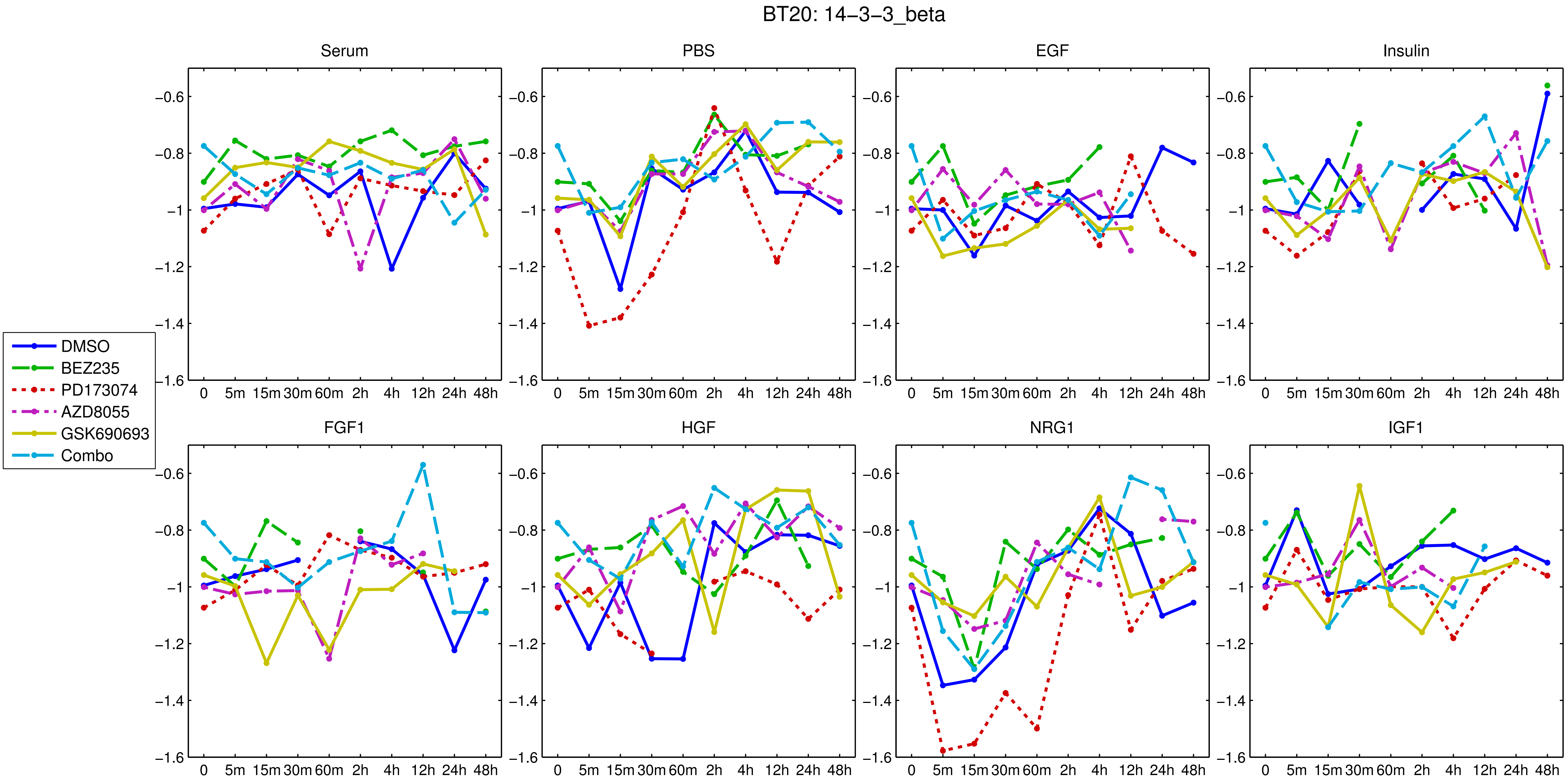
BT20: 14–3–3_beta
Serum
PBS
EGF
Insulin
FGF1
HGF
NRG1
IGF1
DMSO
BEZ235
PD173074
AZD8055
GSK690693
Combo
−0.6
−0.8
−1
−1.2
−1.4
−1.6
0
5m
15m
30m
60m
2h
4h
12h
24h
48h

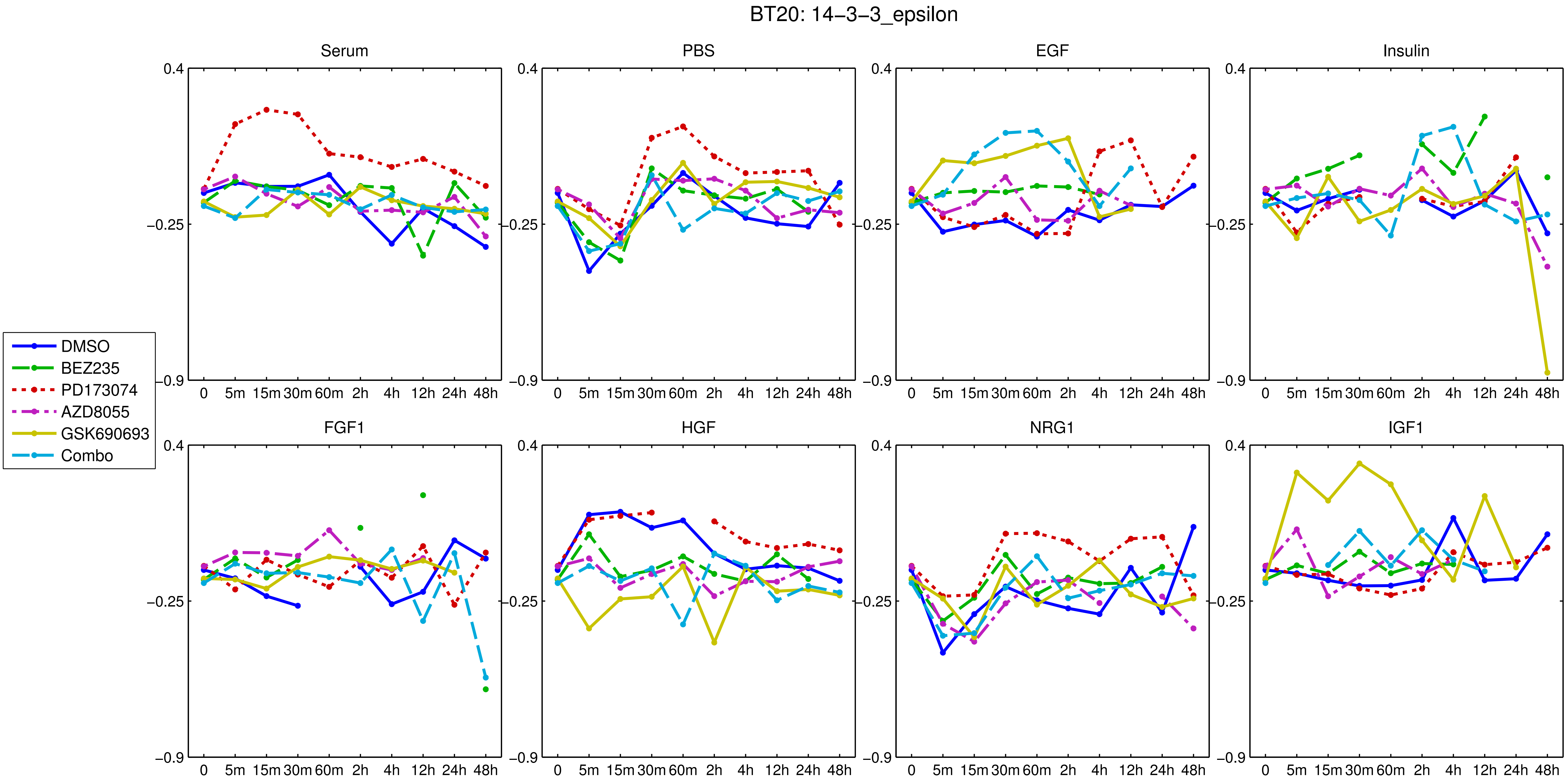
BT20: 14−3−3_epsilon
Serum
PBS
EGF
Insulin
FGF1
HGF
NRG1
IGF1
0.4
−0.25
−0.9
0
5m
15m
30m
60m
2h
4h
12h
24h
48h
DMSO
BEZ235
PD173074
AZD8055
GSK690693
Combo

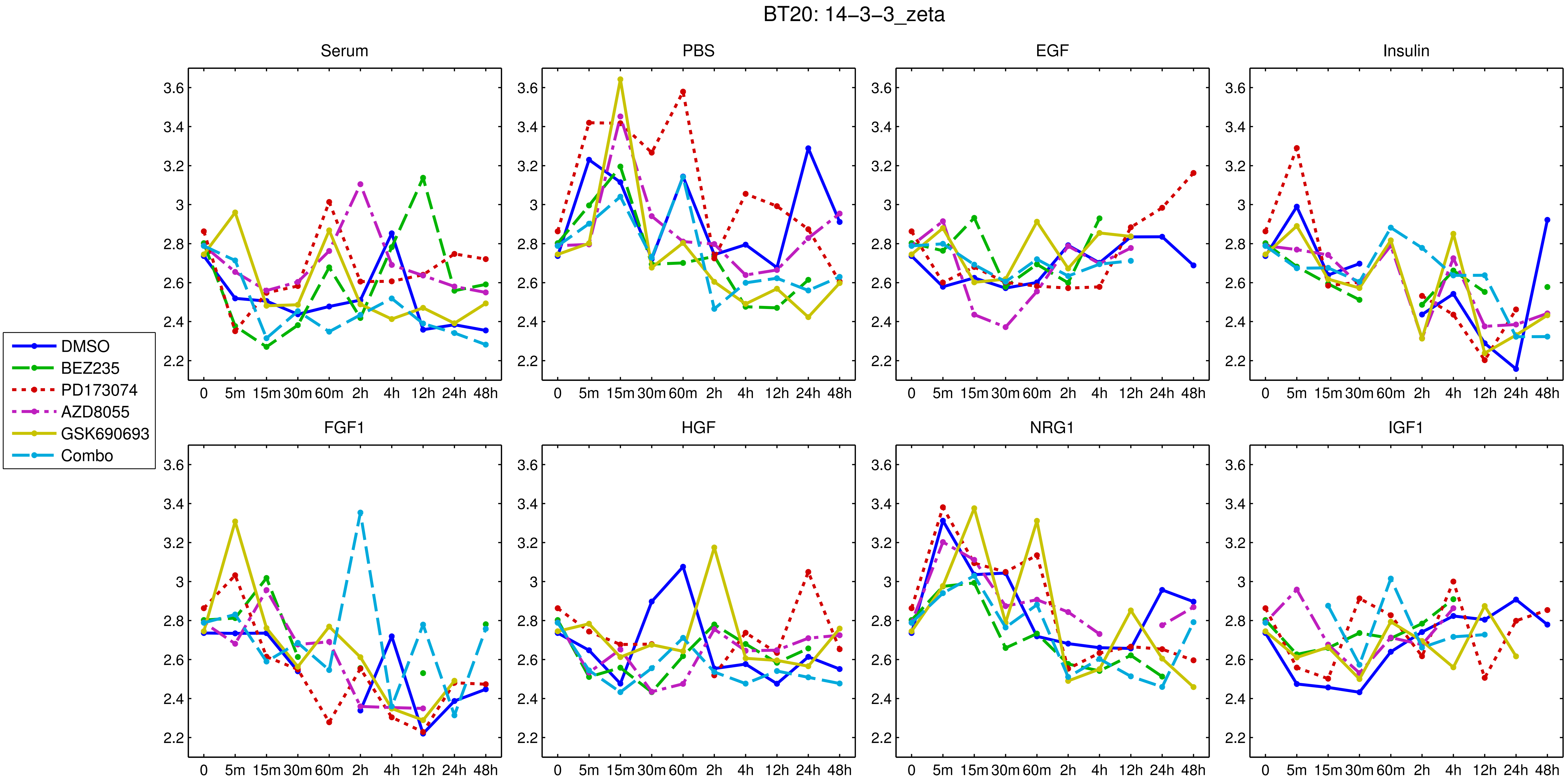
BT20: 14–3–3_zeta
Serum
PBS
EGF
Insulin
FGF1
HGF
NRG1
IGF1
DMSO
BEZ235
PD173074
AZD8055
GSK690693
Combo
3.6
3.4
3.2
3
2.8
2.6
2.4
2.2
0
5m
15m
30m
60m
2h
4h
12h
24h
48h

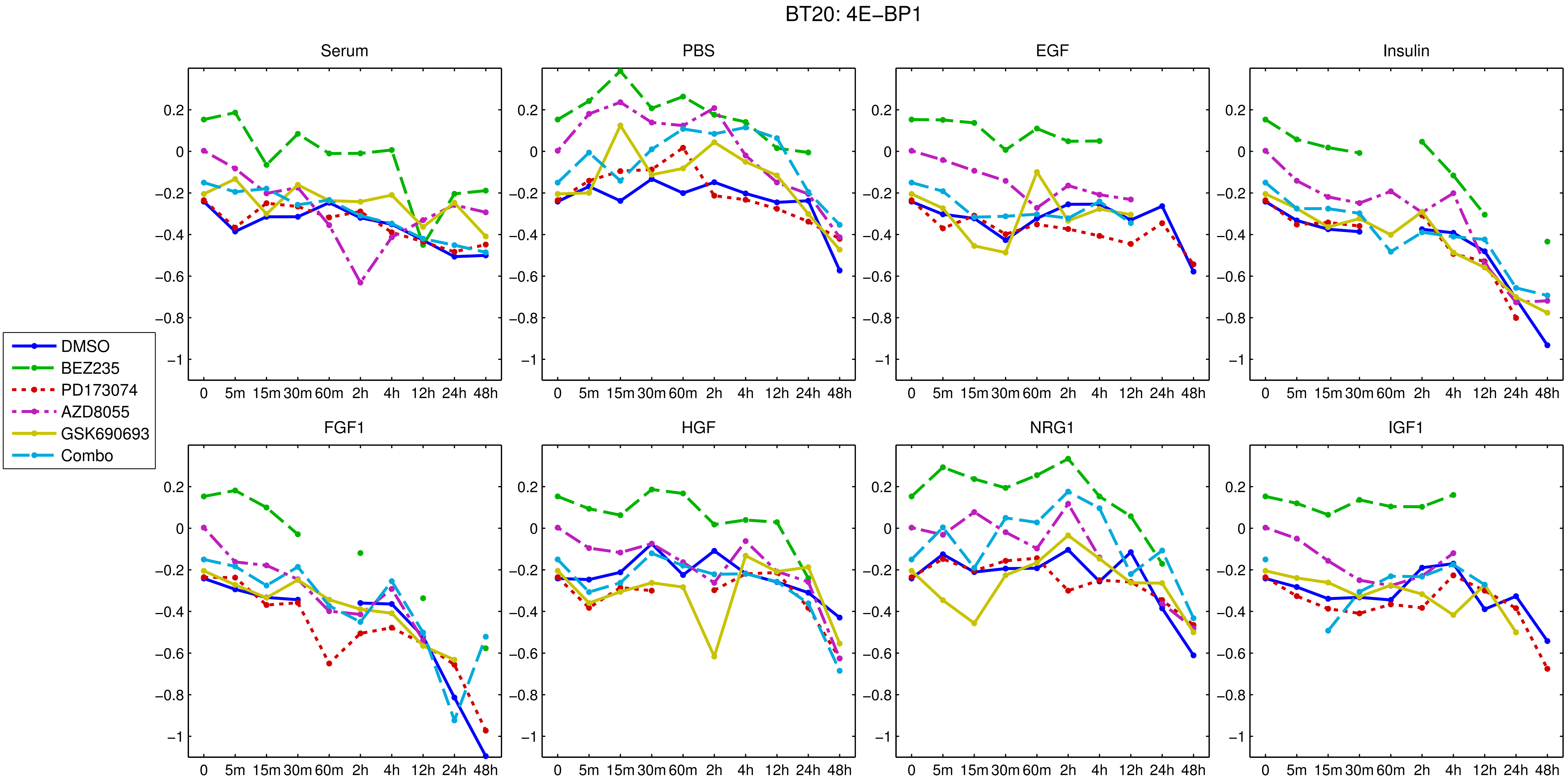

BT20: 4E−BP1
Serum
PBS
EGF
Insulin
FGF1
HGF
NRG1
IGF1
DMSO
BEZ235
PD173074
AZD8055
GSK690693
Combo
0.2
0
−0.2
−0.4
−0.6
−0.8
−1
0 5m 15m 30m 60m 2h 4h 12h 24h 48h

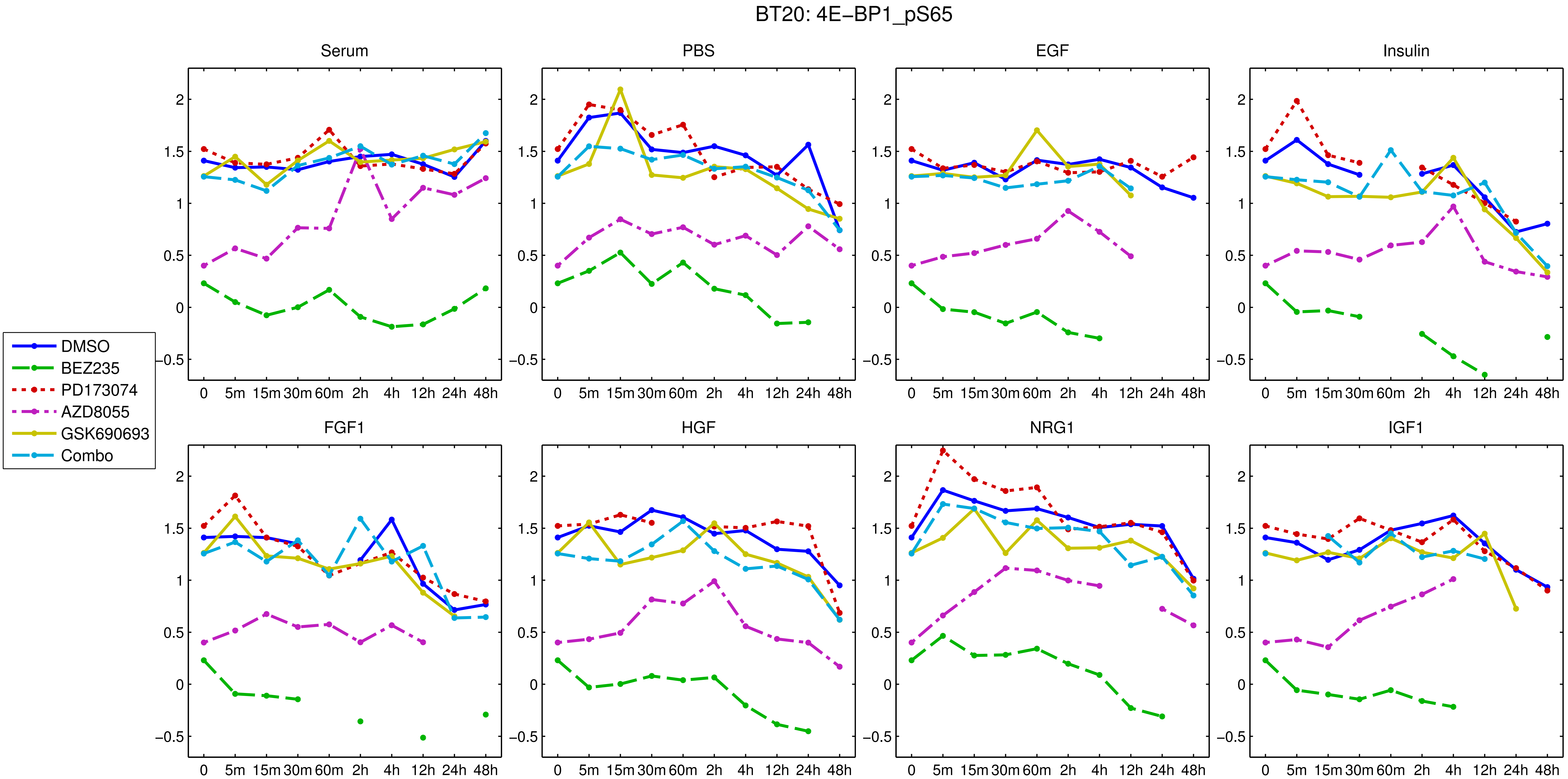
BT20: 4E−BP1_pS65
Serum
PBS
EGF
Insulin
FGF1
HGF
NRG1
IGF1
DMSO
BEZ235
PD173074
AZD8055
GSK690693
Combo
0 5m 15m 30m 60m 2h 4h 12h 24h 48h

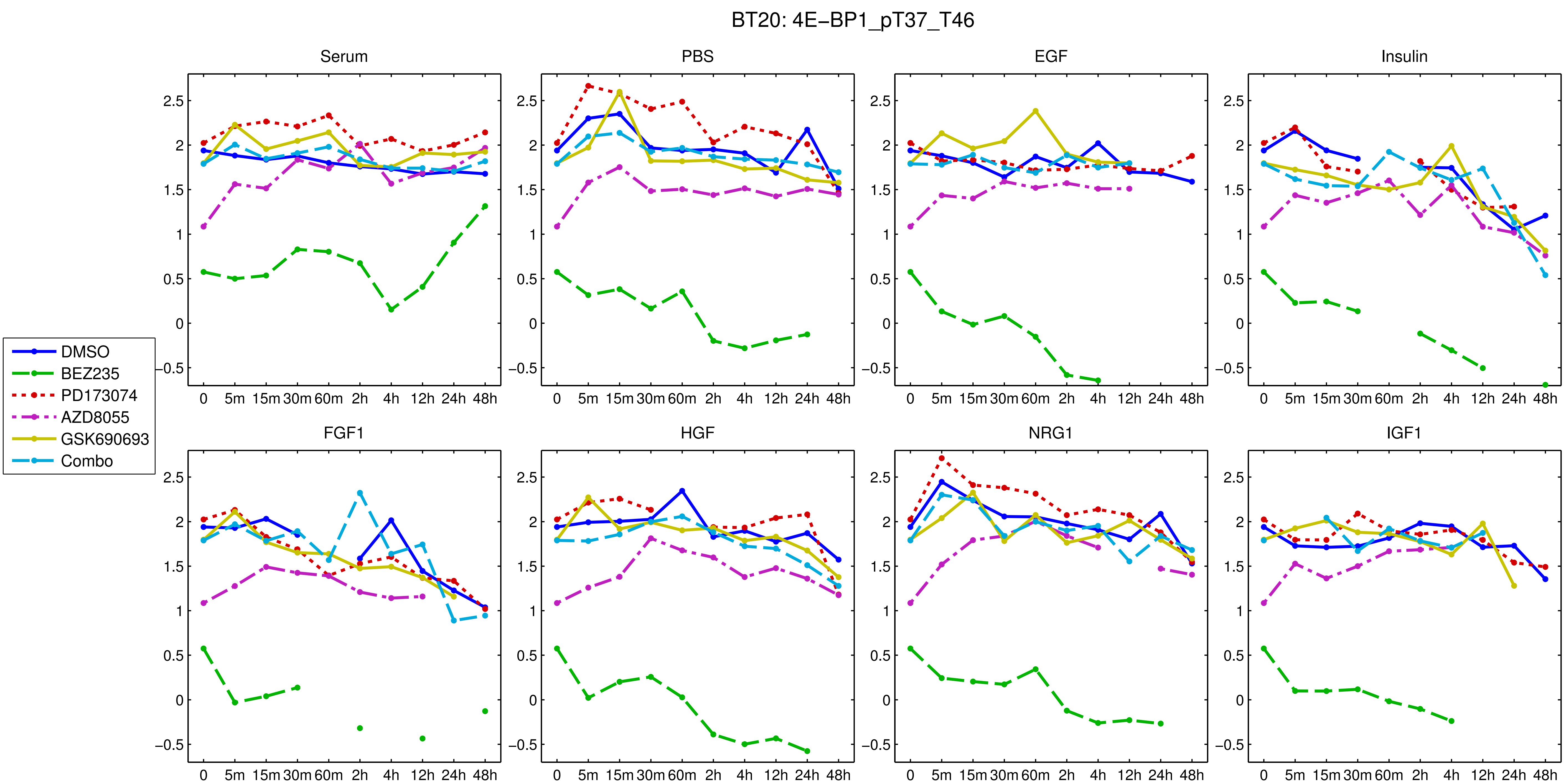
BT20: 4E−BP1_pT37_T46
Serum
PBS
EGF
Insulin
FGF1
HGF
NRG1
IGF1
DMSO
BEZ235
PD173074
AZD8055
GSK690693
Combo
0
5m
15m
30m
60m
2h
4h
12h
24h
48h
−0.5
0
0.5
1
1.5
2
2.5

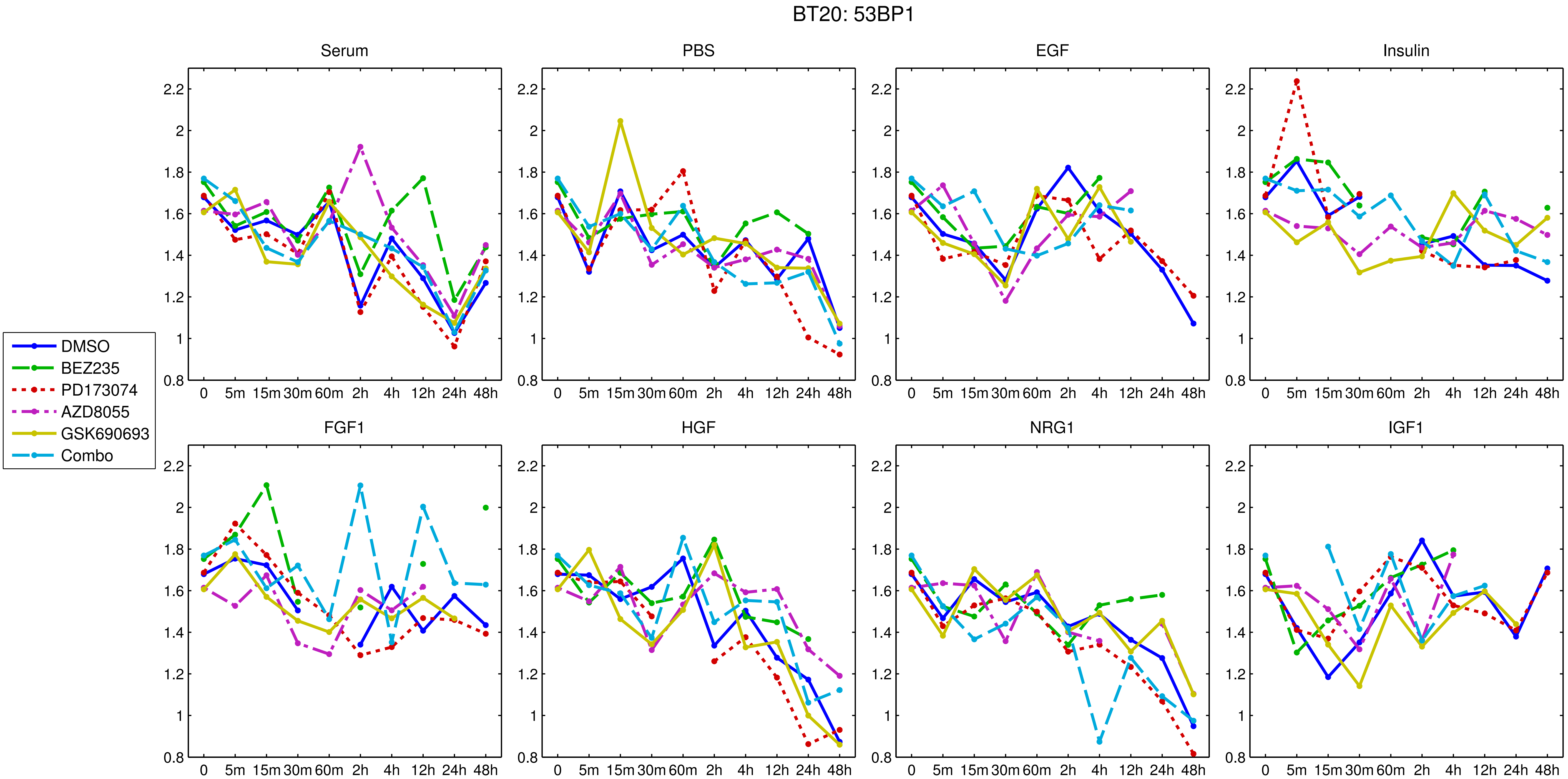

BT20: 53BP1
Serum
PBS
EGF
Insulin
FGF1
HGF
NRG1
IGF1
DMSO
BEZ235
PD173074
AZD8055
GSK690693
Combo
0.8
1
1.2
1.4
1.6
1.8
2
2.2
0
5m
15m
30m
60m
2h
4h
12h
24h
48h

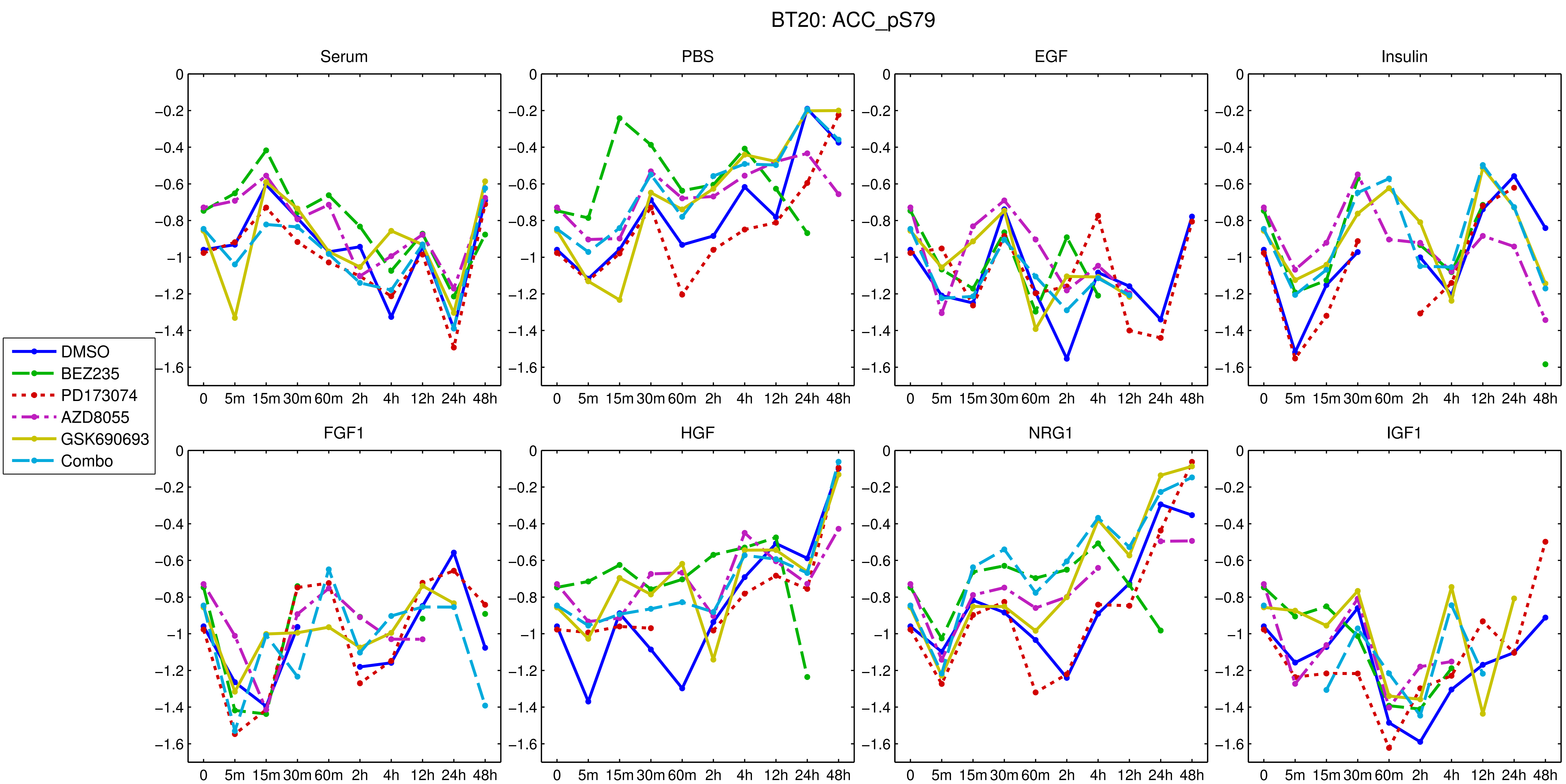
BT20: ACC_pS79
Serum
PBS
EGF
Insulin
FGF1
HGF
NRG1
IGF1
0
−0.2
−0.4
−0.6
−0.8
−1
−1.2
−1.4
−1.6
0 5m 15m 30m 60m 2h 4h 12h 24h 48h
DMSO
BEZ235
PD173074
AZD8055
GSK690693
Combo

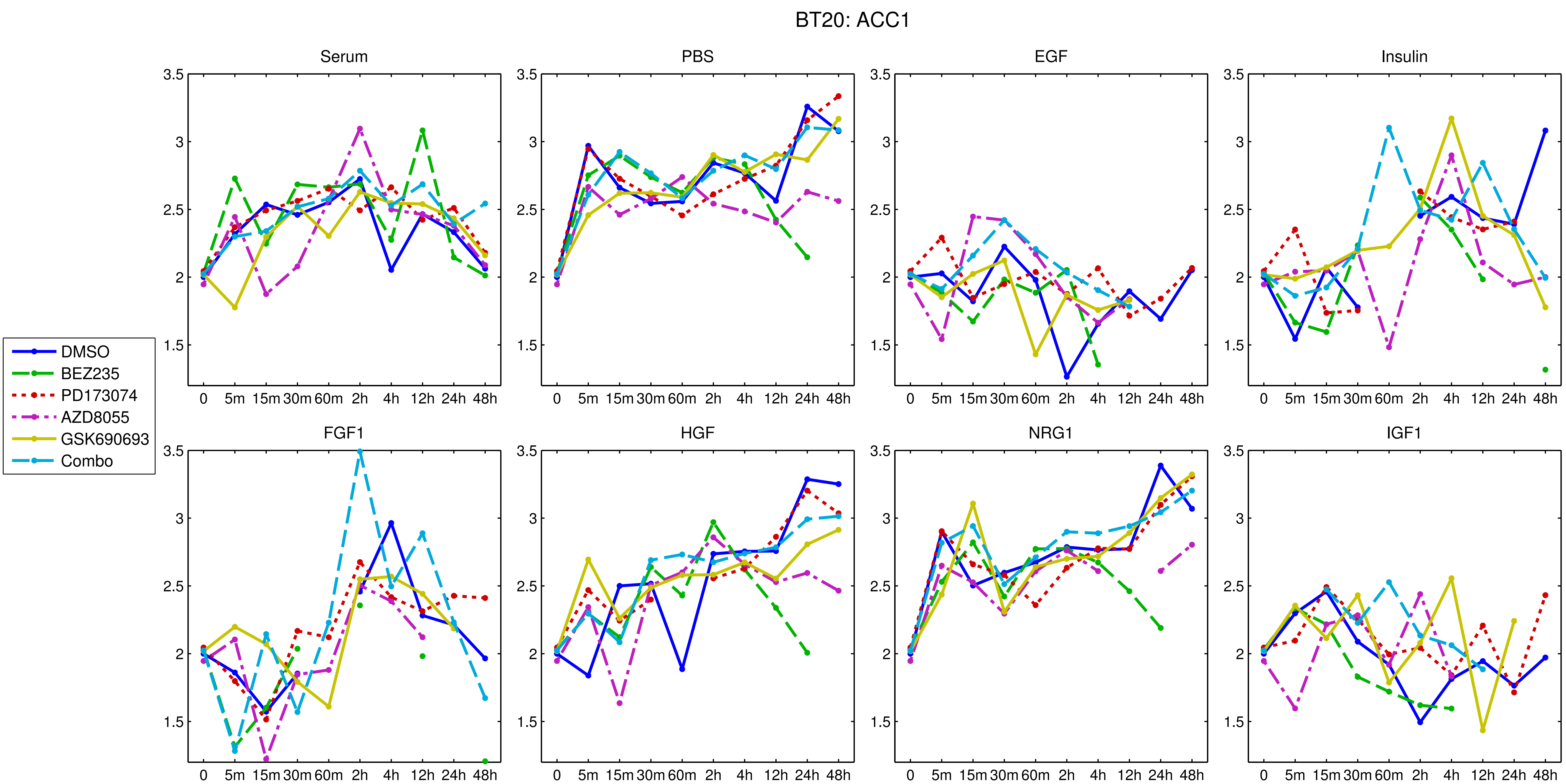
BT20: ACC1
Serum
PBS
EGF
Insulin
FGF1
HGF
NRG1
IGF1
DMSO
BEZ235
PD173074
AZD8055
GSK690693
Combo
3.5
3
2.5
2
1.5
0
5m
15m
30m
60m
2h
4h
12h
24h
48h

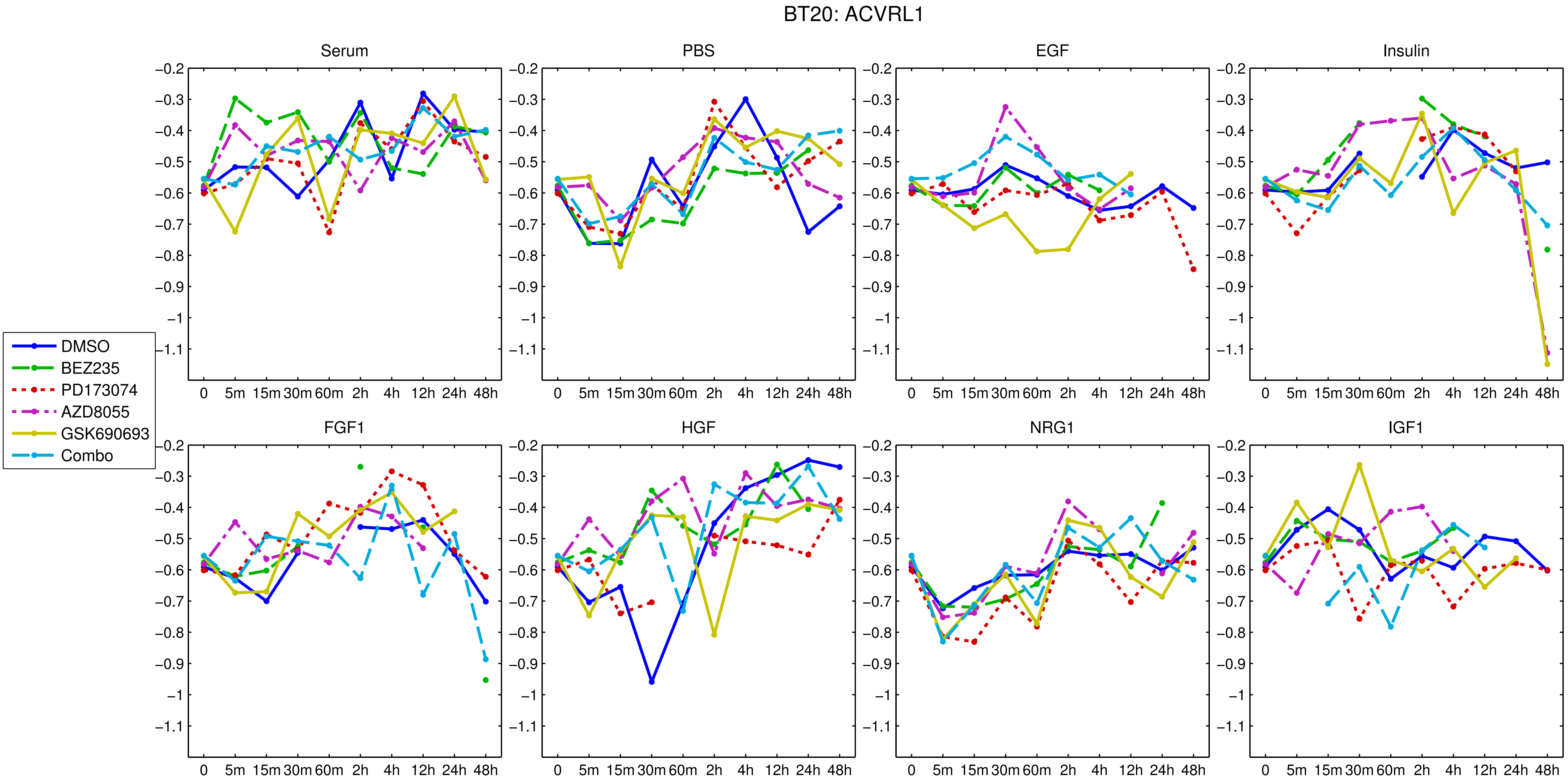

BT20: ACVRL1
Serum
PBS
EGF
Insulin
FGF1
HGF
NRG1
IGF1
DMSO
BEZ235
PD173074
AZD8055
GSK690693
Combo
−0.2
−0.3
−0.4
−0.5
−0.6
−0.7
−0.8
−0.9
−1
−1.1
0
5m
15m
30m
60m
2h
4h
12h
24h
48h

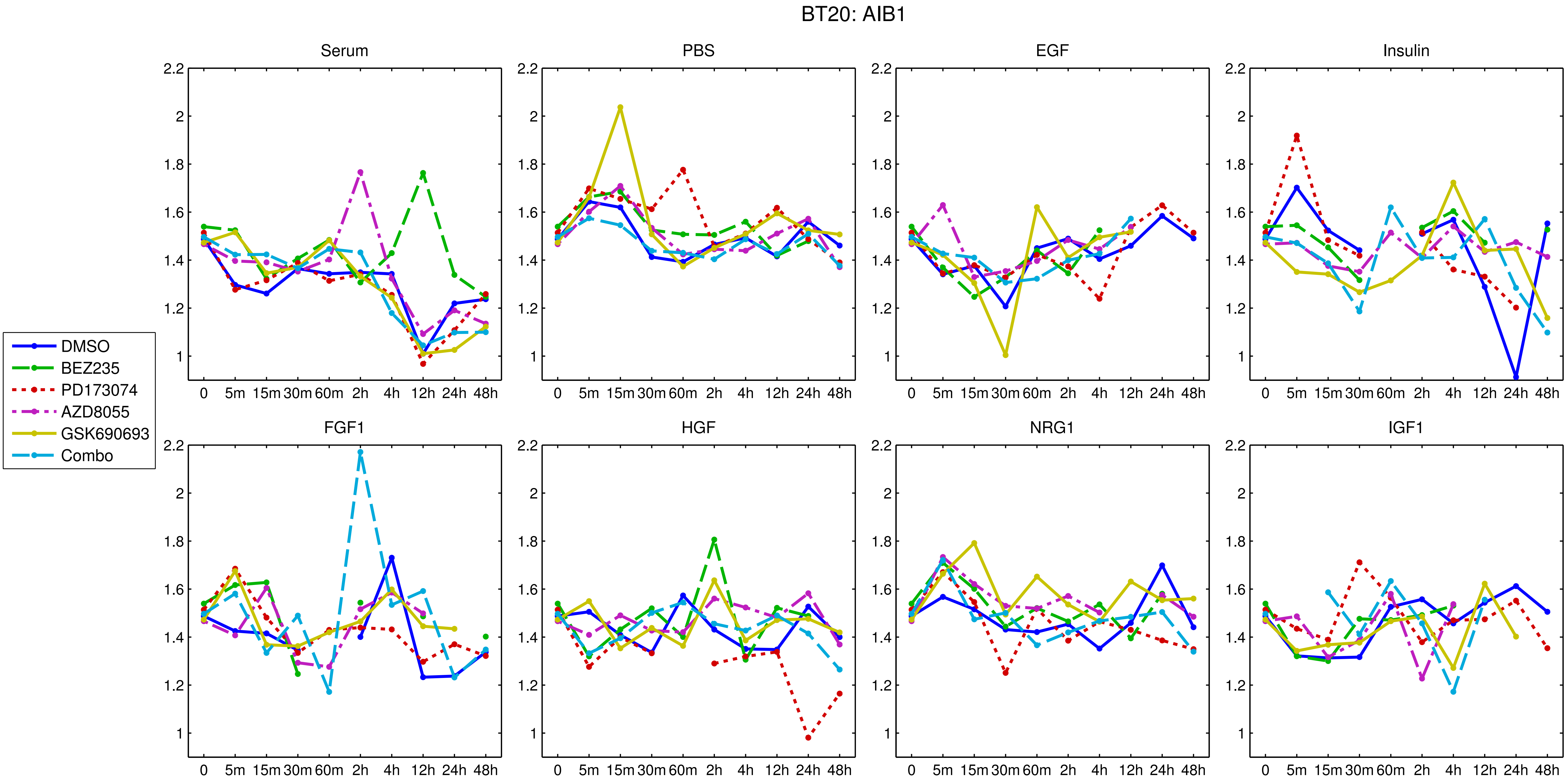
BT20: AIB1
Serum
PBS
EGF
Insulin
FGF1
HGF
NRG1
IGF1
DMSO
BEZ235
PD173074
AZD8055
GSK690693
Combo
2.2
2
1.8
1.6
1.4
1.2
1
0
5m
15m
30m
60m
2h
4h
12h
24h
48h

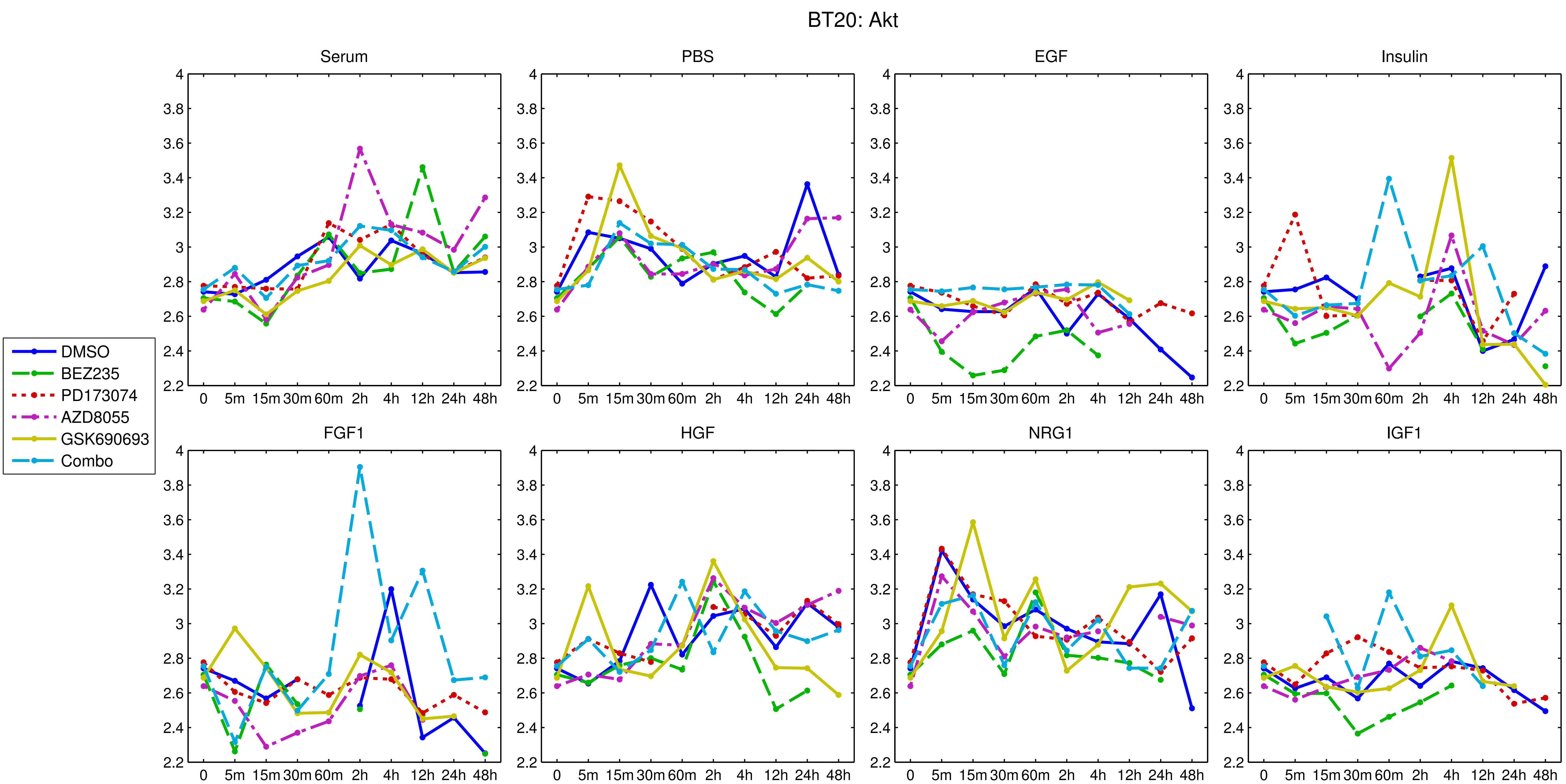

BT20: Akt
Serum
PBS
EGF
Insulin
FGF1
HGF
NRG1
IGF1
DMSO
BEZ235
PD173074
AZD8055
GSK690693
Combo
4
3.8
3.6
3.4
3.2
3
2.8
2.6
2.4
2.2
0
5m
15m
30m
60m
2h
4h
12h
24h
48h

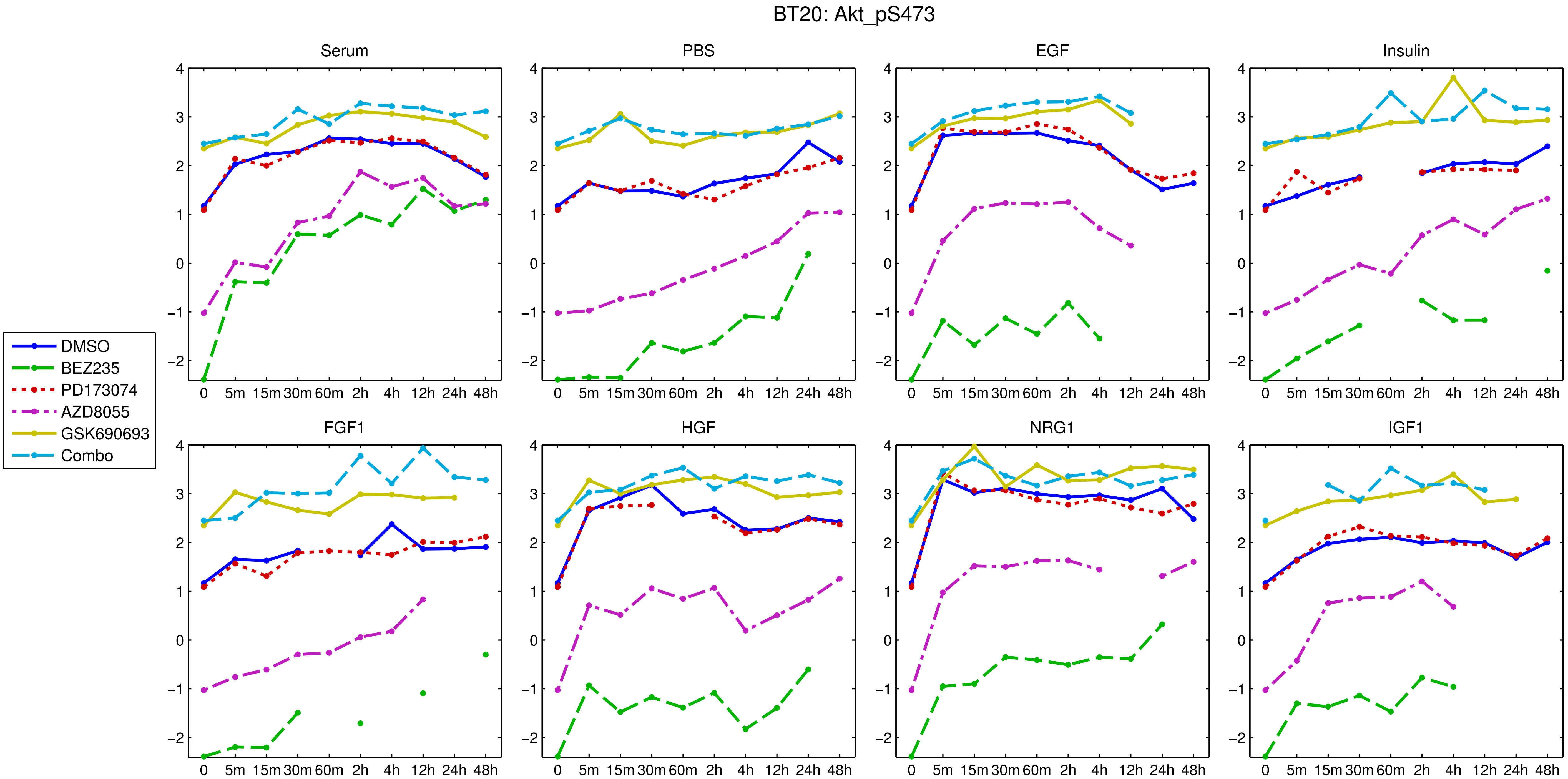

BT20: Akt_pS473
Serum
PBS
EGF
Insulin
FGF1
HGF
NRG1
IGF1
DMSO
BEZ235
PD173074
AZD8055
GSK690693
Combo
0
5m
15m
30m
60m
2h
4h
12h
24h
48h

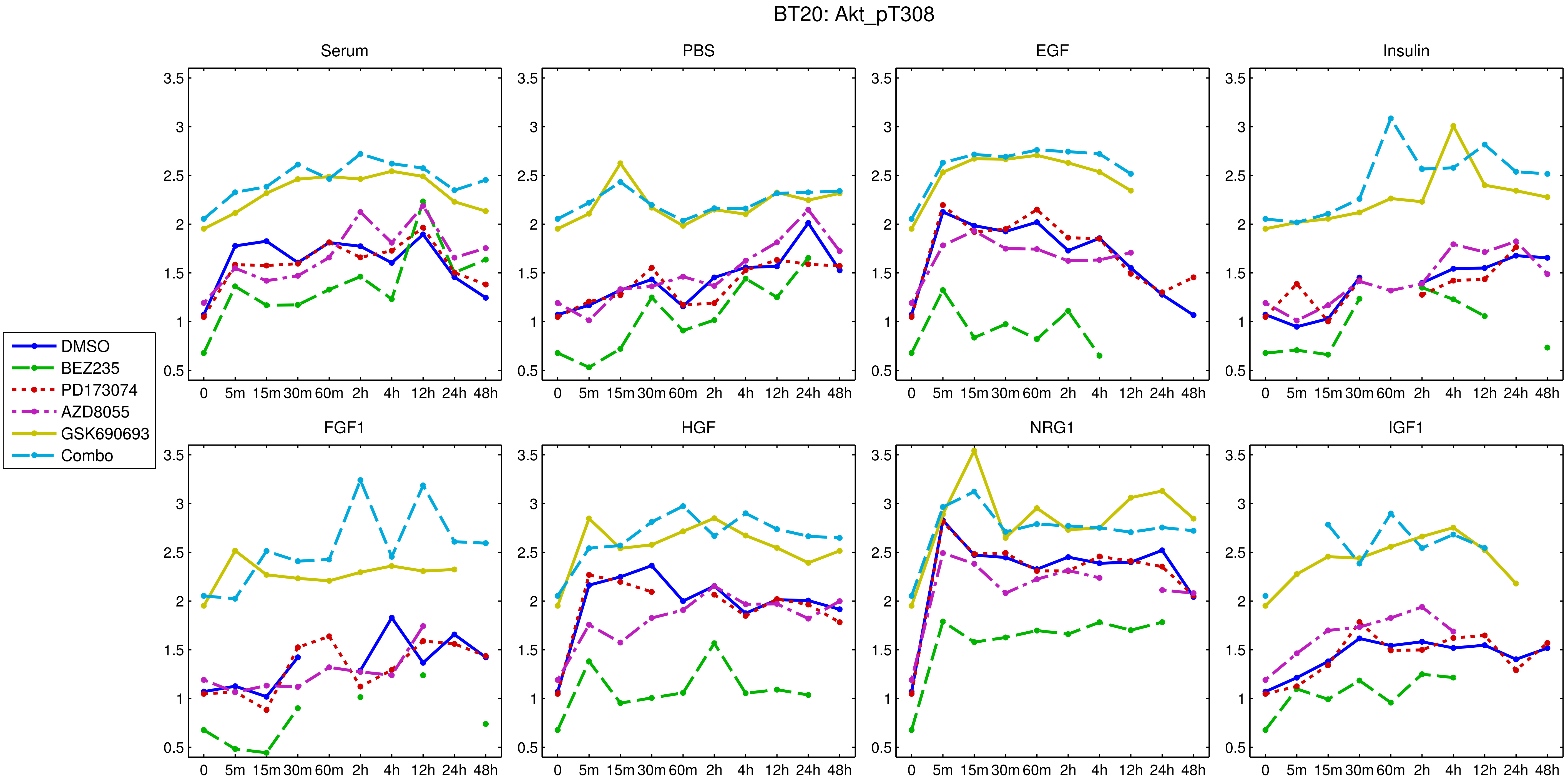
BT20: Akt_pT308
Serum
PBS
EGF
Insulin
FGF1
HGF
NRG1
IGF1
DMSO
BEZ235
PD173074
AZD8055
GSK690693
Combo
0.5
1
1.5
2
2.5
3
3.5
0
5m
15m
30m
60m
2h
4h
12h
24h
48h

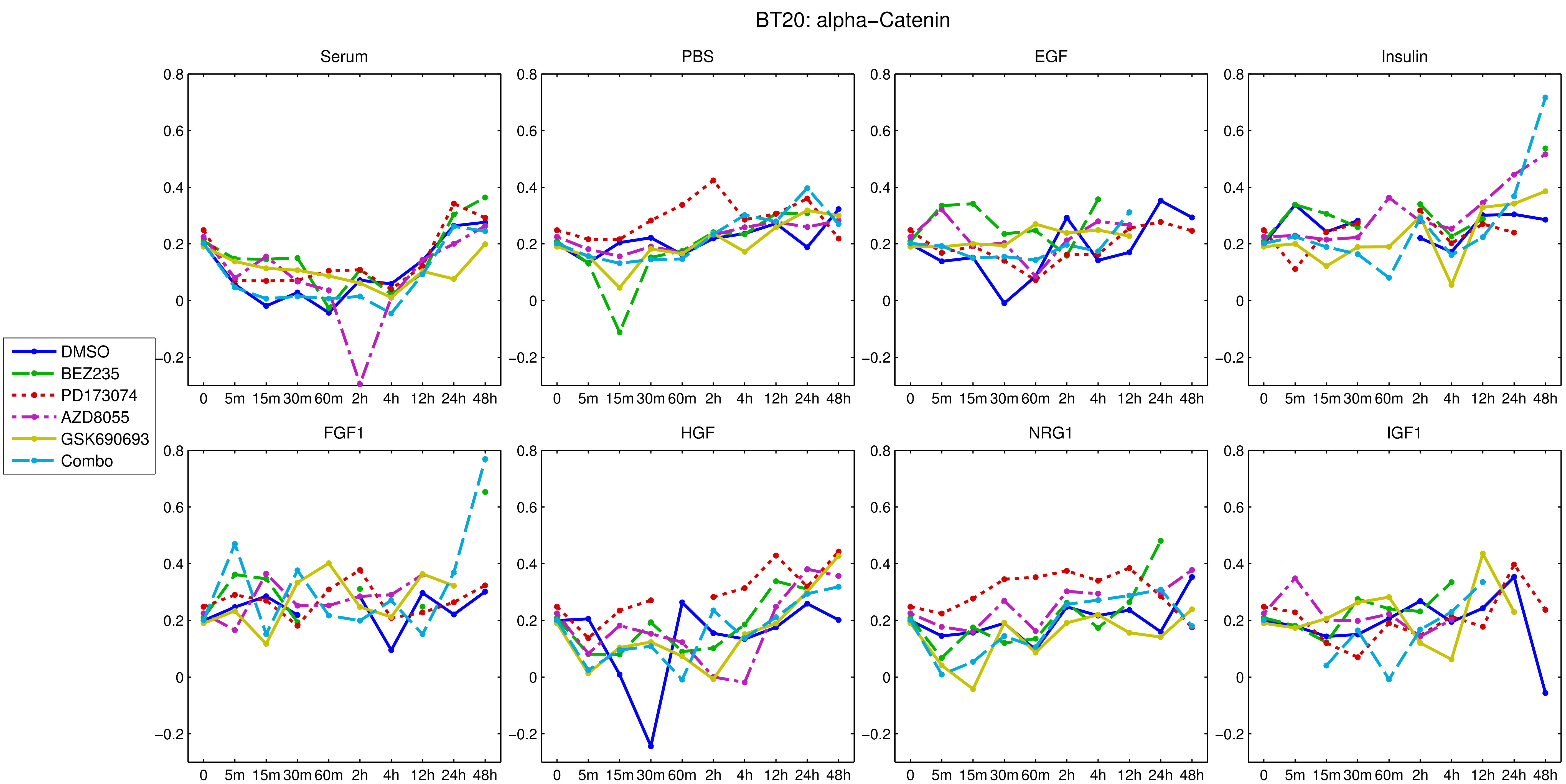

BT20: alpha−Catenin
Serum
PBS
EGF
Insulin
FGF1
HGF
NRG1
IGF1
0.8
0.6
0.4
0.2
0
−0.2
0
5m
15m
30m
60m
2h
4h
12h
24h
48h
DMSO
BEZ235
PD173074
AZD8055
GSK690693
Combo

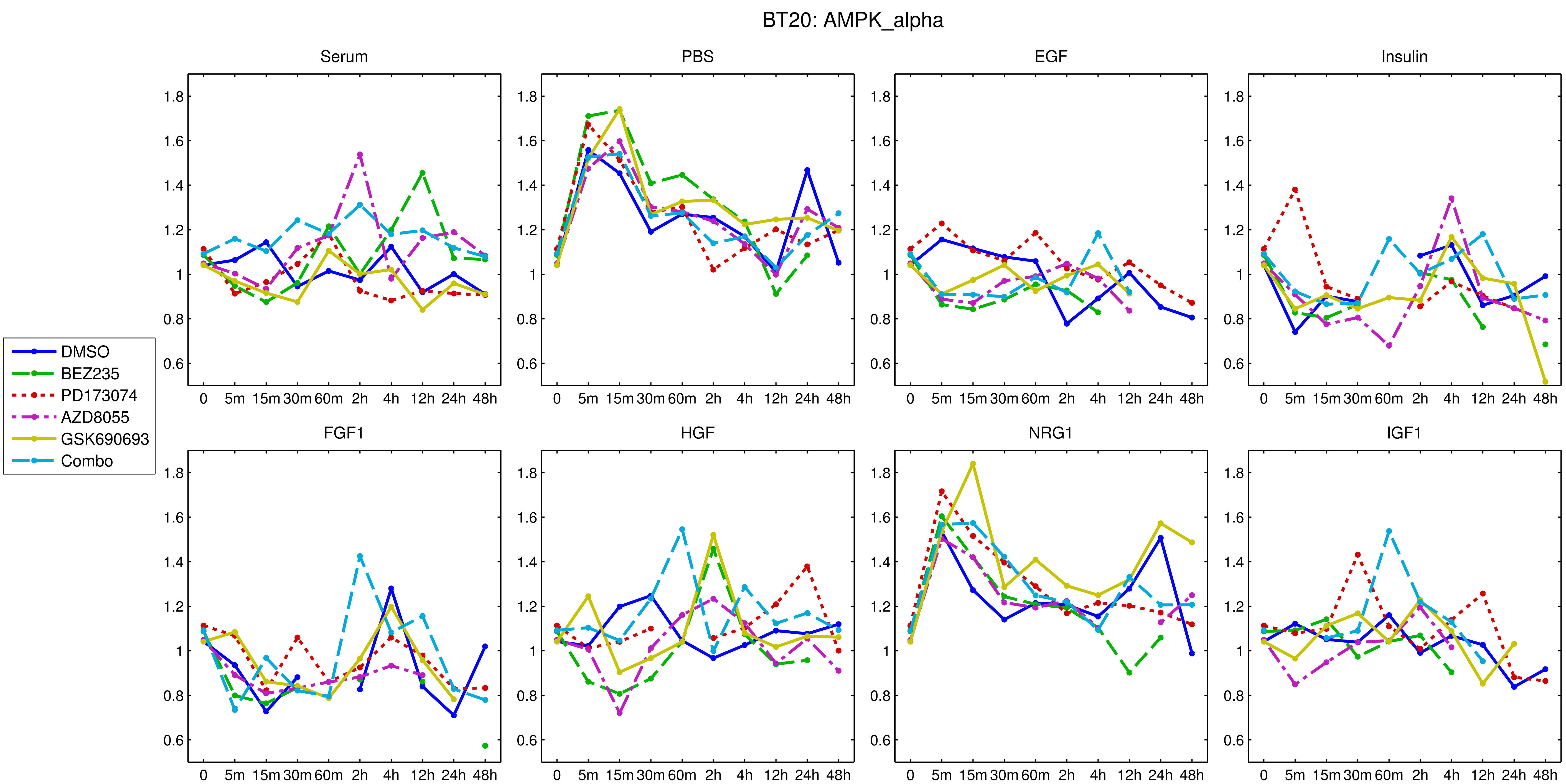
BT20: AMPK_alpha
Serum
PBS
EGF
Insulin
FGF1
HGF
NRG1
IGF1
DMSO
BEZ235
PD173074
AZD8055
GSK690693
Combo
0.6
0.8
1
1.2
1.4
1.6
1.8
0
5m
15m
30m
60m
2h
4h
12h
24h
48h

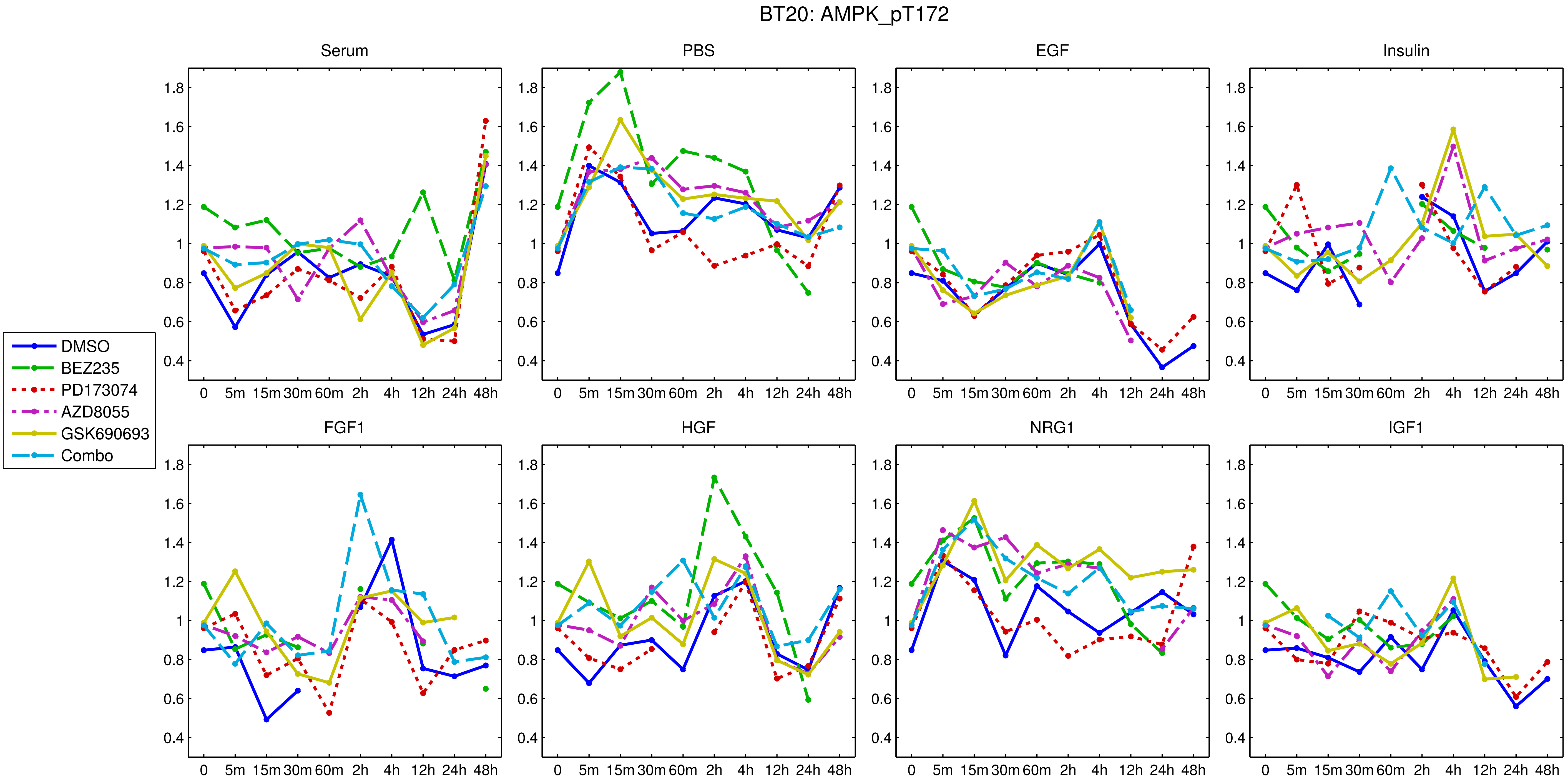

BT20: AMPK_pT172
Serum
PBS
EGF
Insulin
FGF1
HGF
NRG1
IGF1
DMSO
BEZ235
PD173074
AZD8055
GSK690693
Combo
1.8
1.6
1.4
1.2
1
0.8
0.6
0.4
0
5m
15m
30m
60m
2h
4h
12h
24h
48h

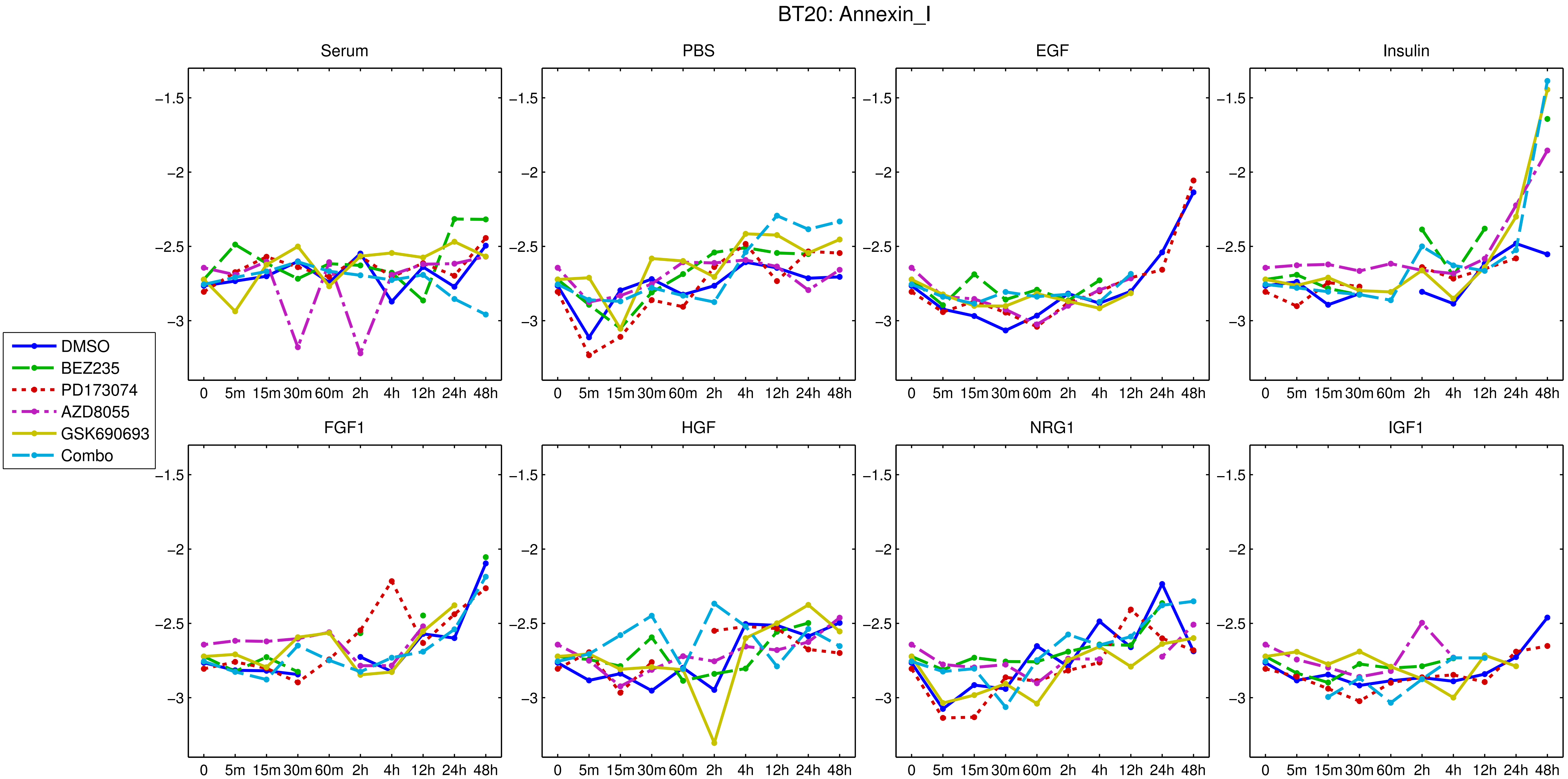
BT20: Annexin_I
Serum
PBS
EGF
Insulin
FGF1
HGF
NRG1
IGF1
−1.5
−2
−2.5
−3
0
5m
15m
30m
60m
2h
4h
12h
24h
48h
DMSO
BEZ235
PD173074
AZD8055
GSK690693
Combo

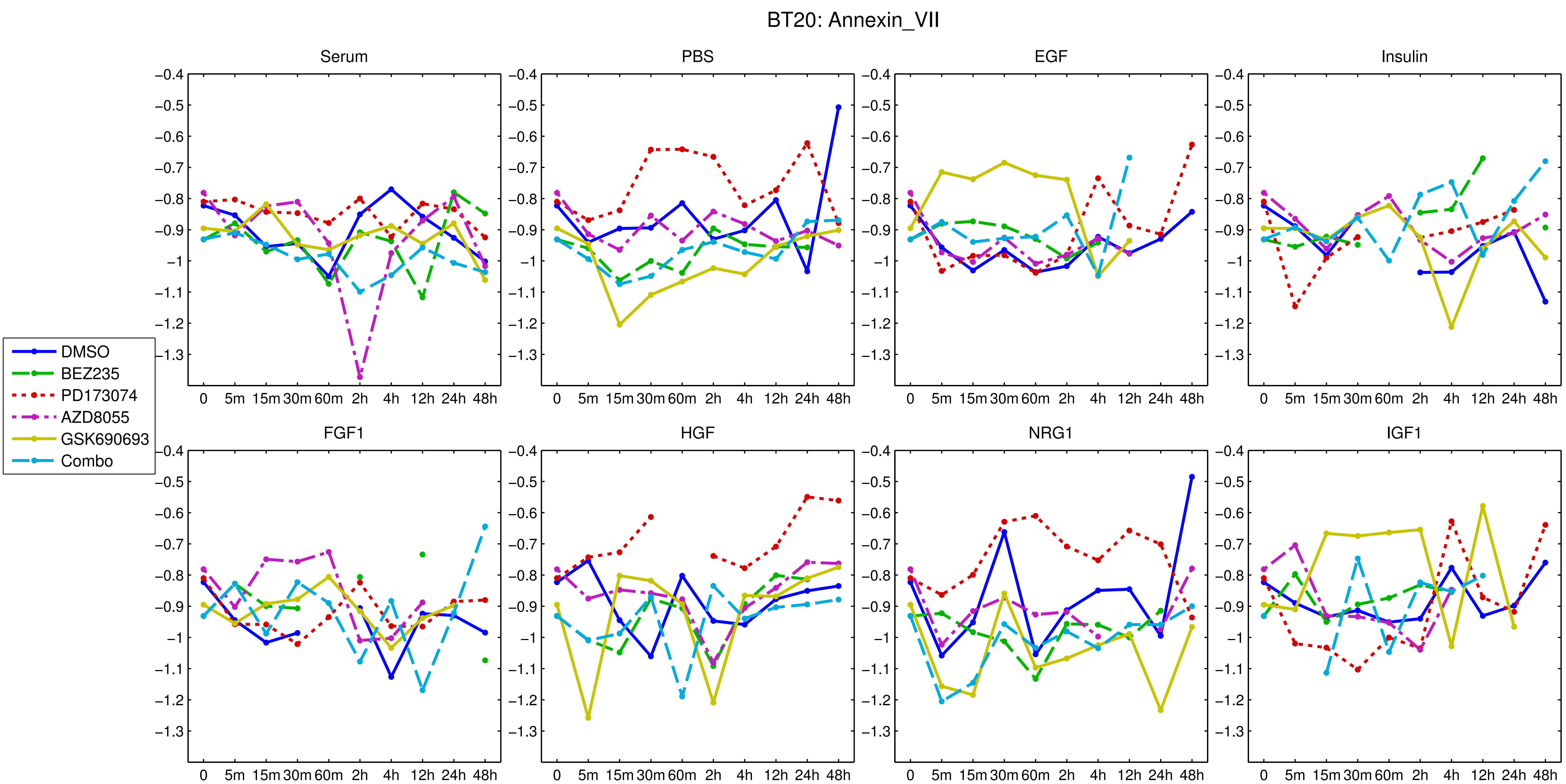

BT20: Annexin_VII
Serum
PBS
EGF
Insulin
FGF1
HGF
NRG1
IGF1
DMSO
BEZ235
PD173074
AZD8055
GSK690693
Combo
−0.4
−0.5
−0.6
−0.7
−0.8
−0.9
−1
−1.1
−1.2
−1.3
0
5m
15m
30m
60m
2h
4h
12h
24h
48h

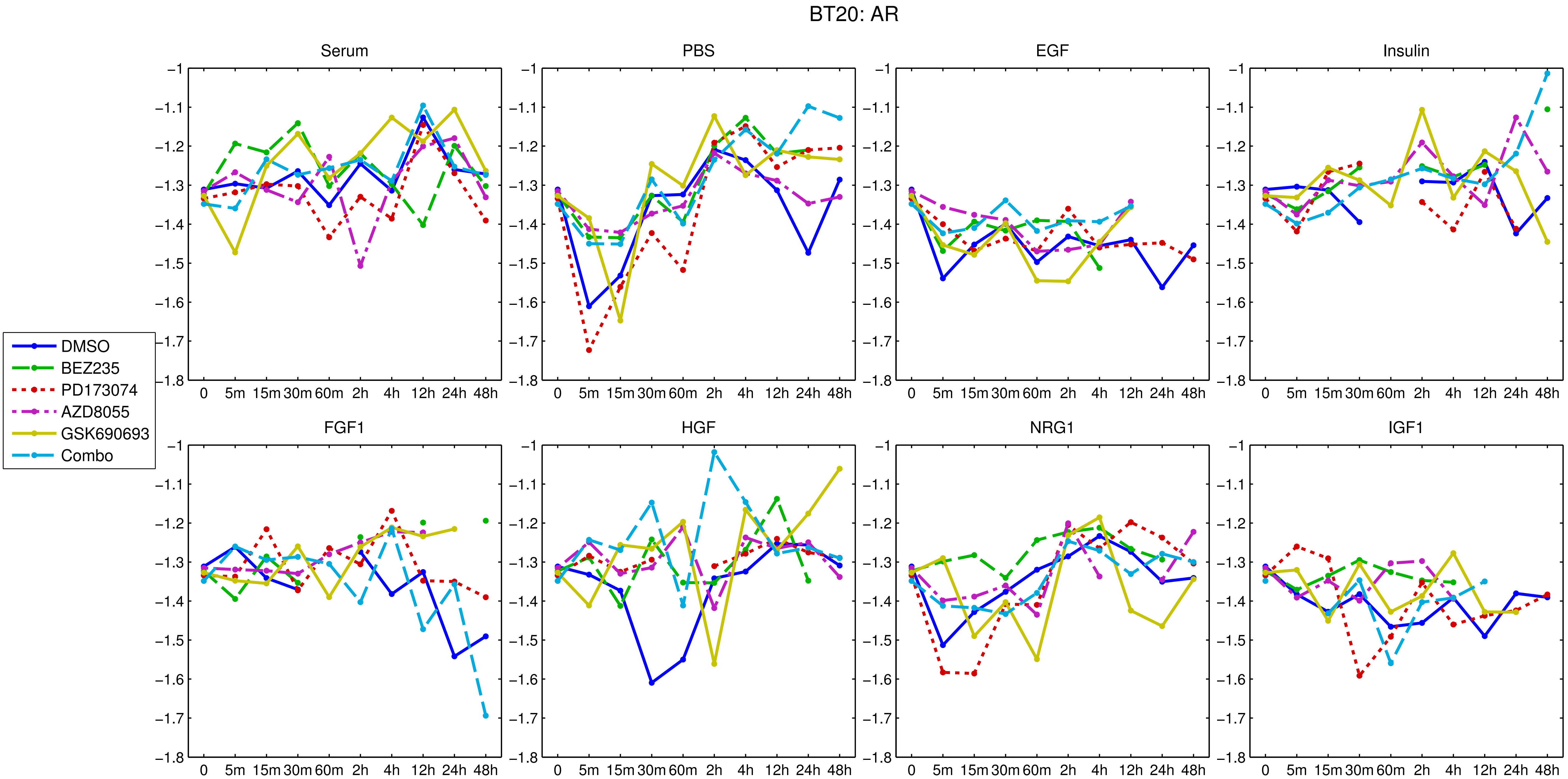

BT20: AR
Serum
PBS
EGF
Insulin
FGF1
HGF
NRG1
IGF1
DMSO
BEZ235
PD173074
AZD8055
GSK690693
Combo
−1
−1.1
−1.2
−1.3
−1.4
−1.5
−1.6
−1.7
−1.8
0
5m
15m
30m
60m
2h
4h
12h
24h
48h

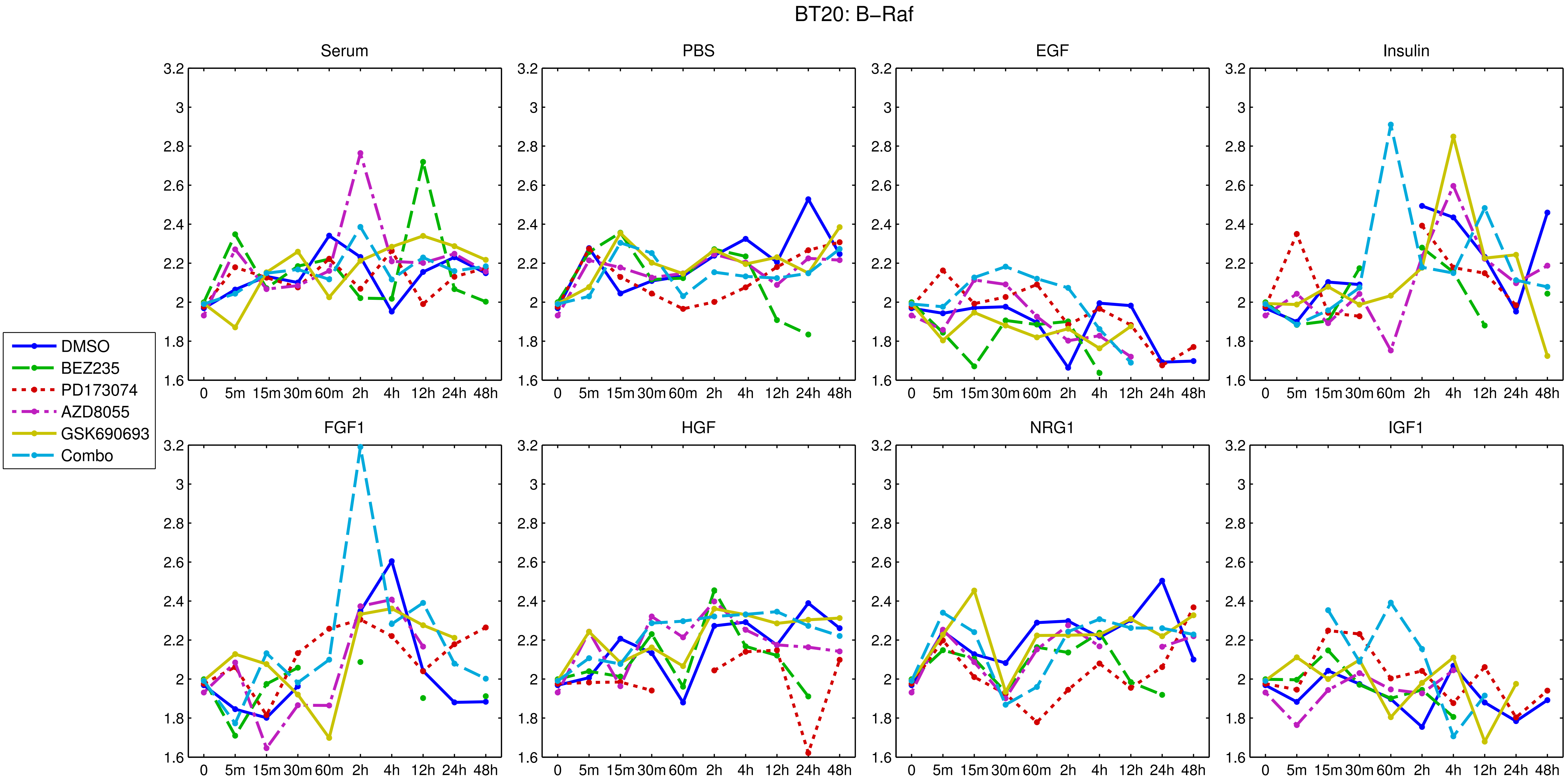

BT20: B−Raf
Serum
PBS
EGF
Insulin
FGF1
HGF
NRG1
IGF1
DMSO
BEZ235
PD173074
AZD8055
GSK690693
Combo
3.2
3
2.8
2.6
2.4
2.2
2
1.8
1.6
0
5m
15m
30m
60m
2h
4h
12h
24h
48h

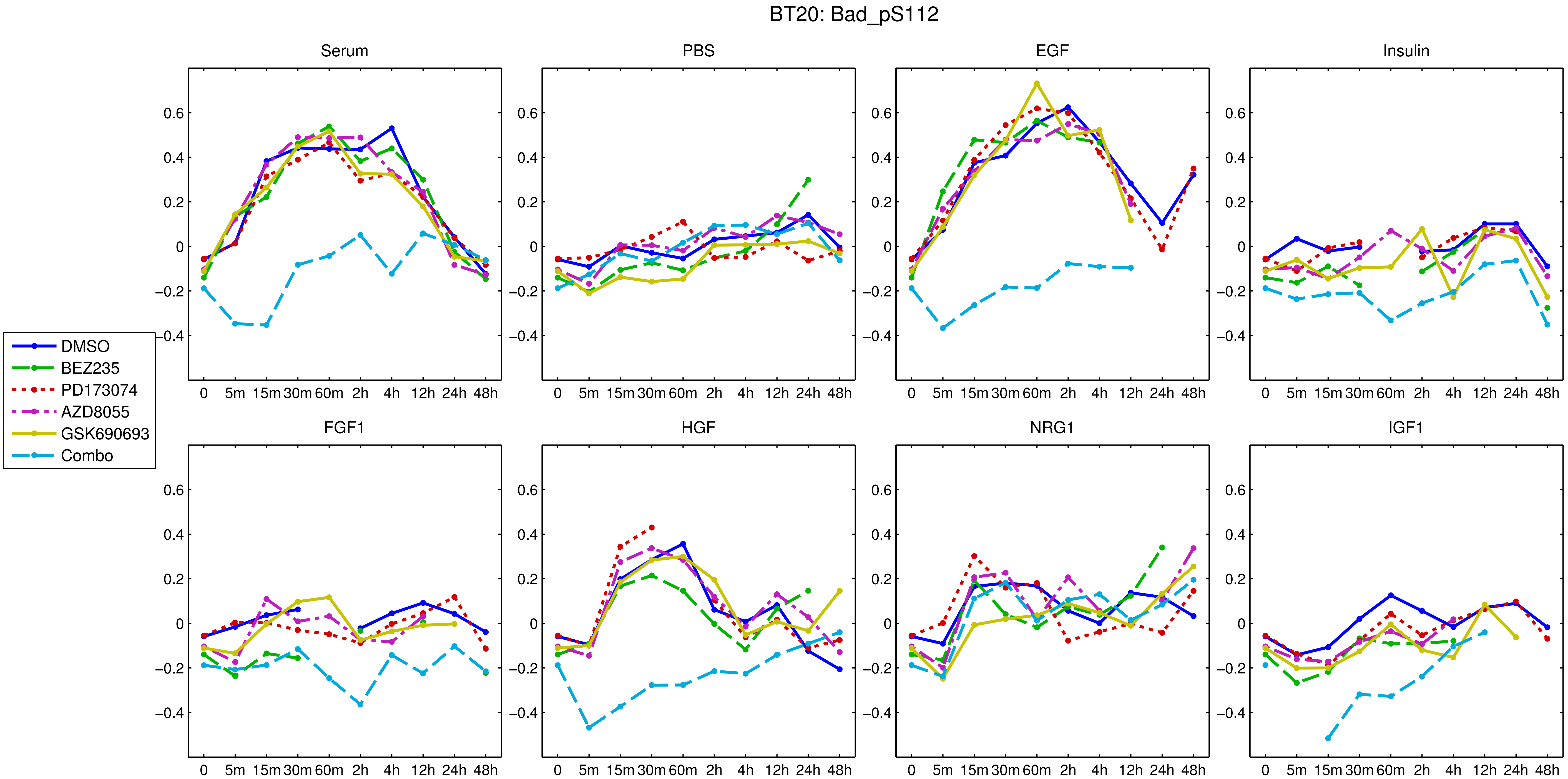

BT20: Bad_pS112
Serum
PBS
EGF
Insulin
FGF1
HGF
NRG1
IGF1
DMSO
BEZ235
PD173074
AZD8055
GSK690693
Combo
0.6
0.4
0.2
0
−0.2
−0.4
0 5m 15m 30m 60m 2h 4h 12h 24h 48h

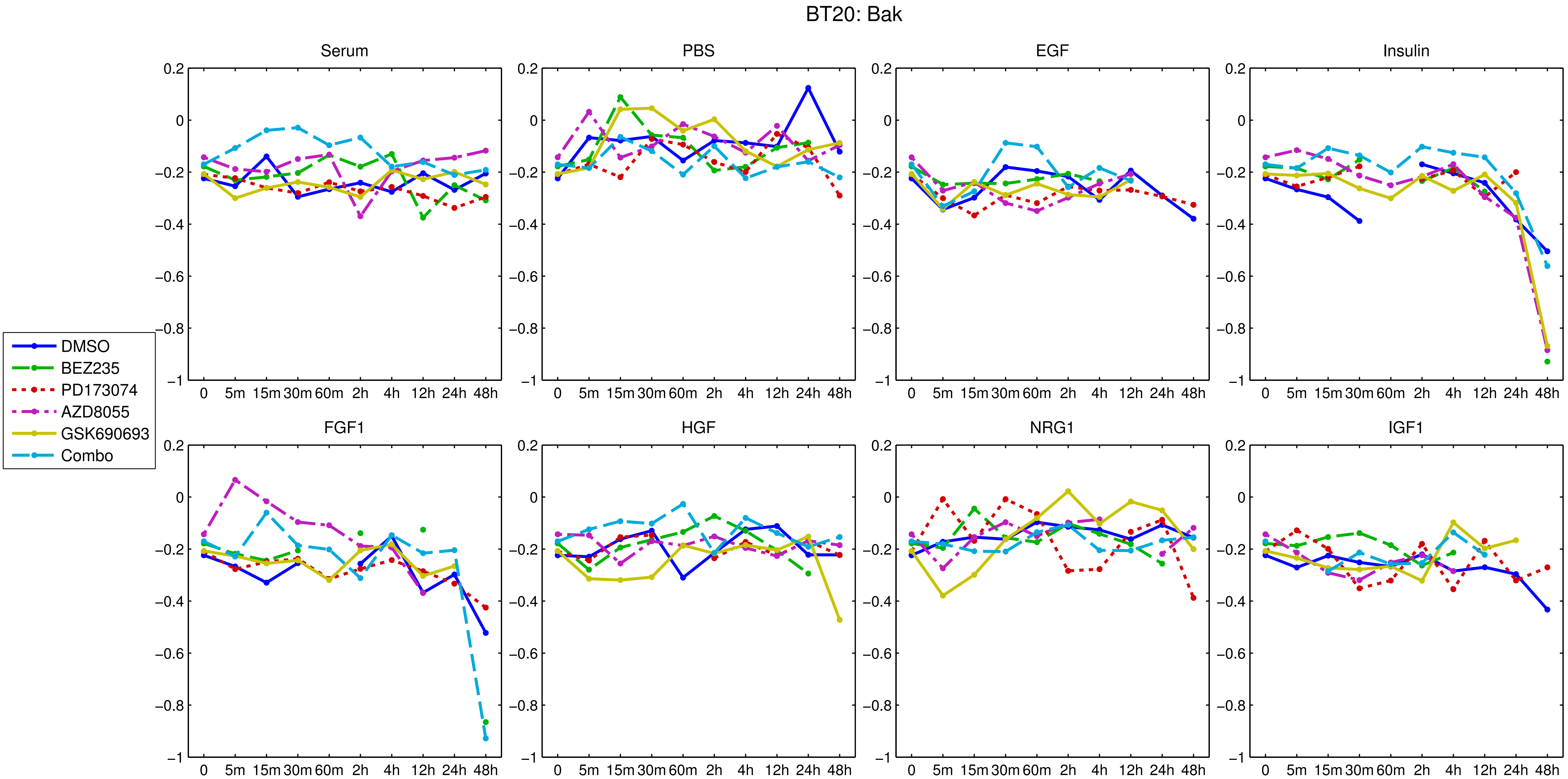
BT20: Bak
Serum
PBS
EGF
Insulin
FGF1
HGF
NRG1
IGF1
0.2
0
−0.2
−0.4
−0.6
−0.8
−1
0 5m 15m 30m 60m 2h 4h 12h 24h 48h
DMSO
BEZ235
PD173074
AZD8055
GSK690693
Combo

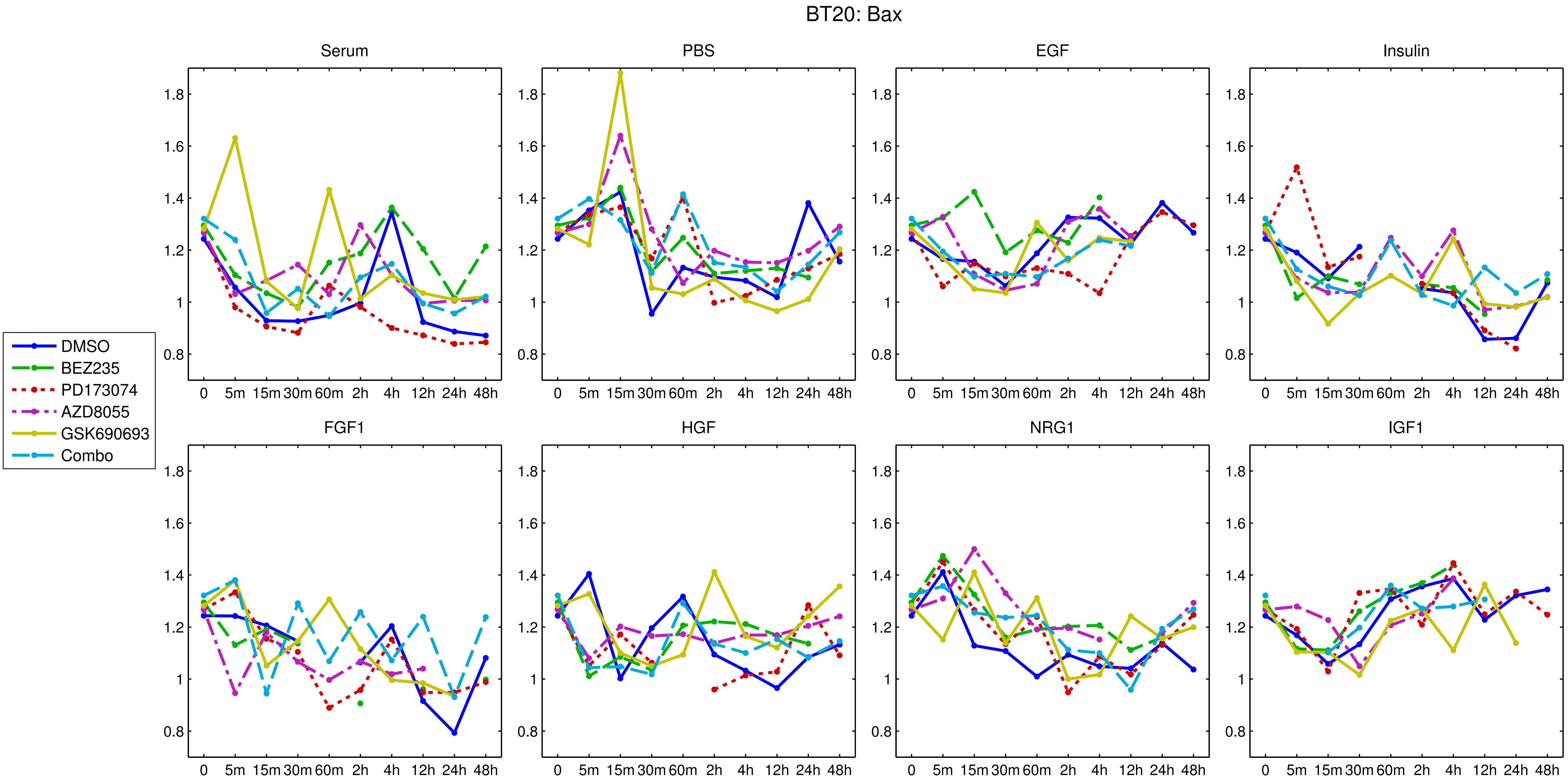
BT20: Bax
Serum
PBS
EGF
Insulin
FGF1
HGF
NRG1
IGF1
DMSO
BEZ235
PD173074
AZD8055
GSK690693
Combo
1.8
1.6
1.4
1.2
1
0.8
0
5m
15m
30m
60m
2h
4h
12h
24h
48h

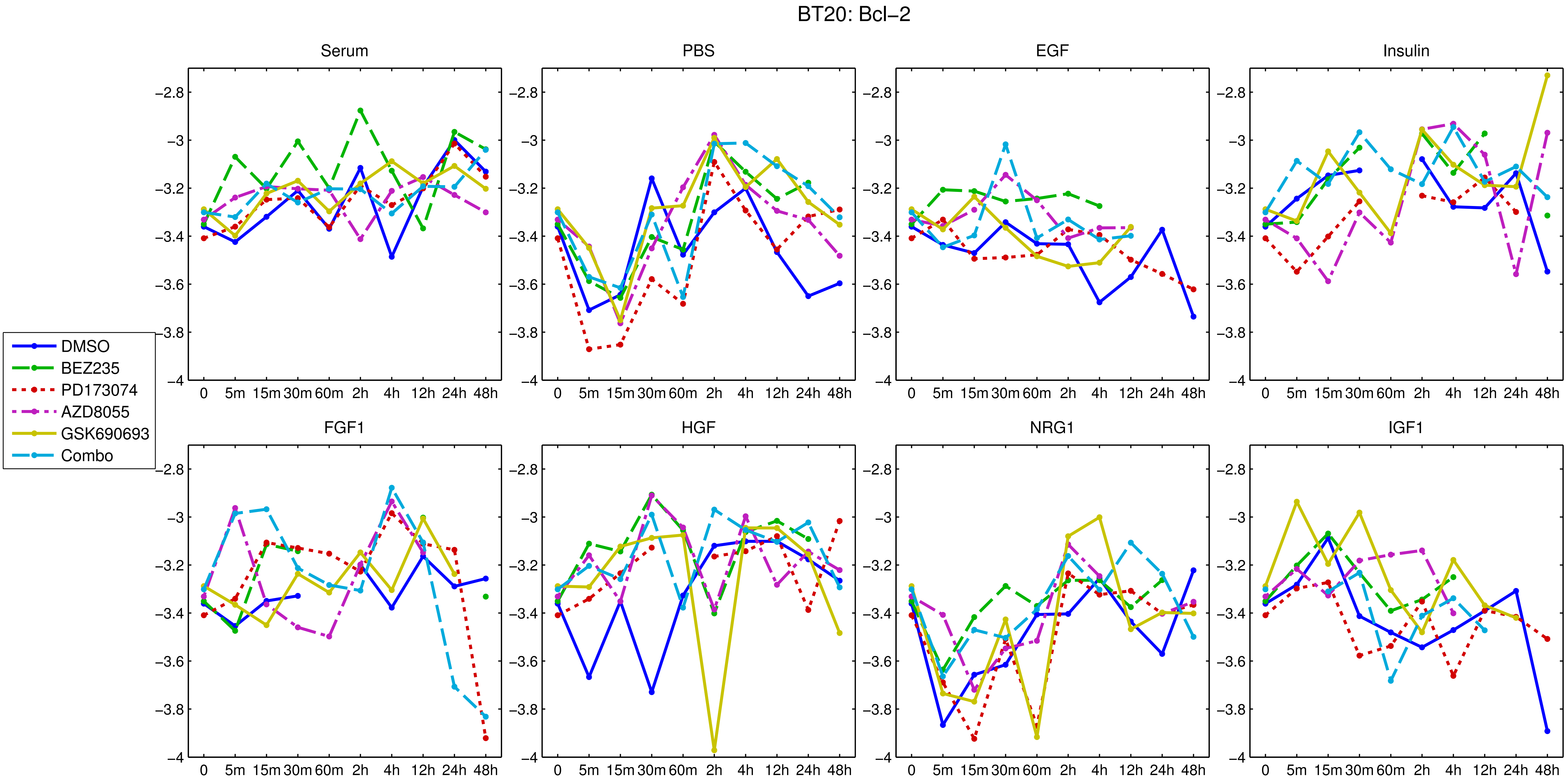

BT20: Bcl–2
Serum
PBS
EGF
Insulin
FGF1
HGF
NRG1
IGF1
DMSO
BEZ235
PD173074
AZD8055
GSK690693
Combo
–2.8
–3
–3.2
–3.4
–3.6
–3.8
–4
0
5m
15m
30m
60m
2h
4h
12h
24h
48h

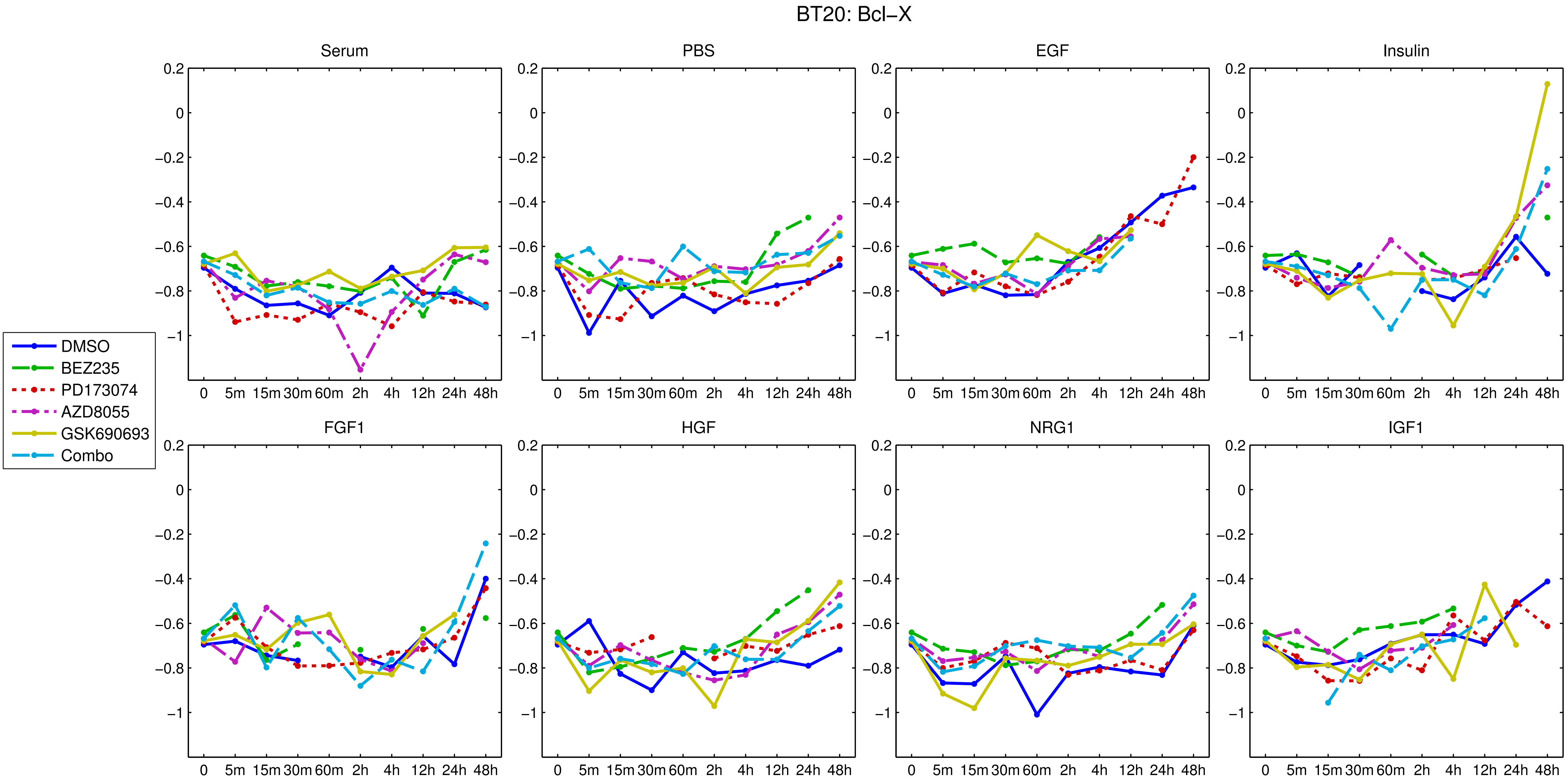
BT20: Bcl–X
Serum
PBS
EGF
Insulin
FGF1
HGF
NRG1
IGF1
DMSO
BEZ235
PD173074
AZD8055
GSK690693
Combo
0.2
0
−0.2
−0.4
−0.6
−0.8
−1
0
5m
15m
30m
60m
2h
4h
12h
24h
48h

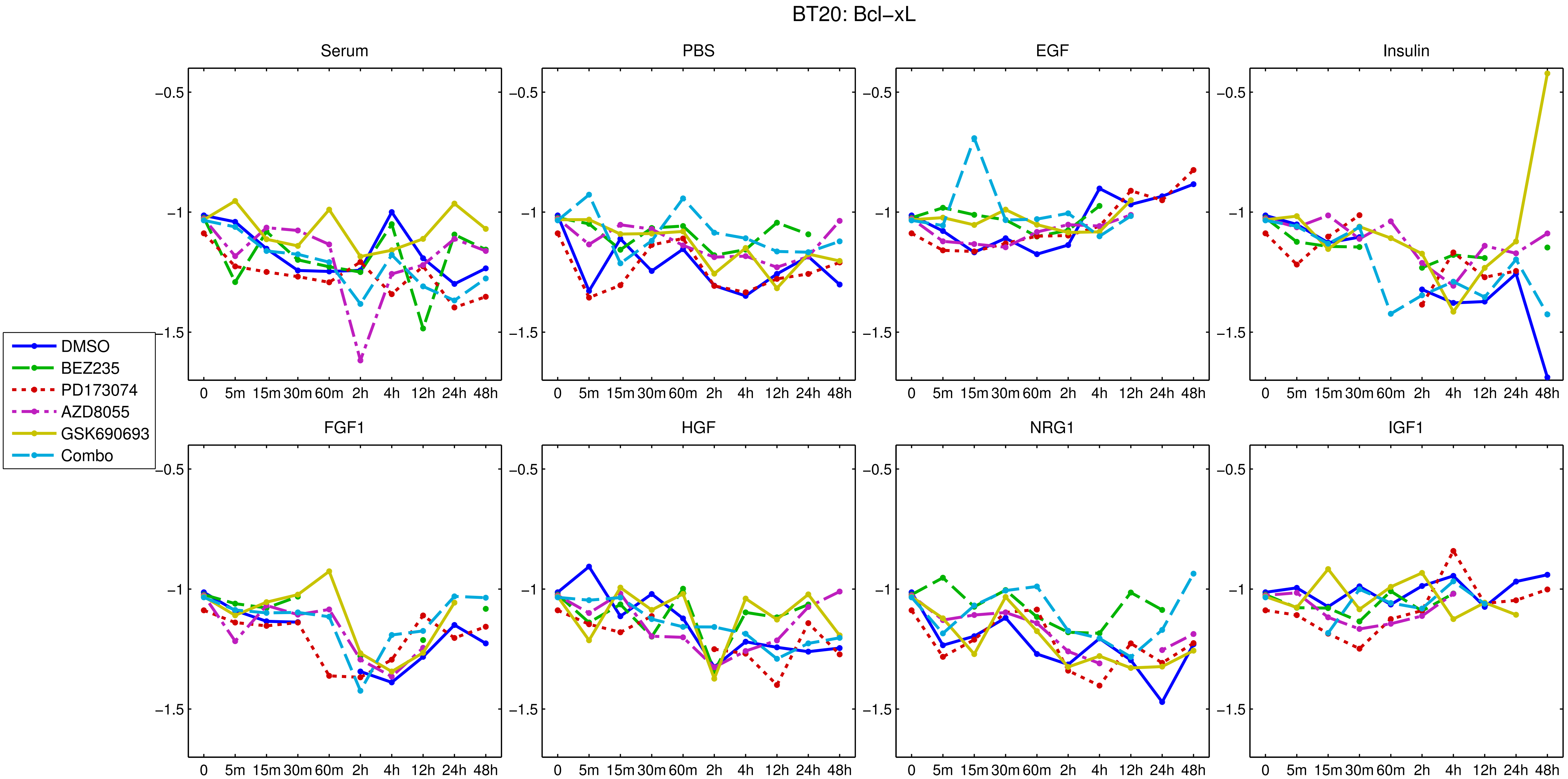

BT20: Bcl−xL
Serum
PBS
EGF
Insulin
FGF1
HGF
NRG1
IGF1
−0.5
−1
−1.5
0
5m
15m
30m
60m
2h
4h
12h
24h
48h
DMSO
BEZ235
PD173074
AZD8055
GSK690693
Combo

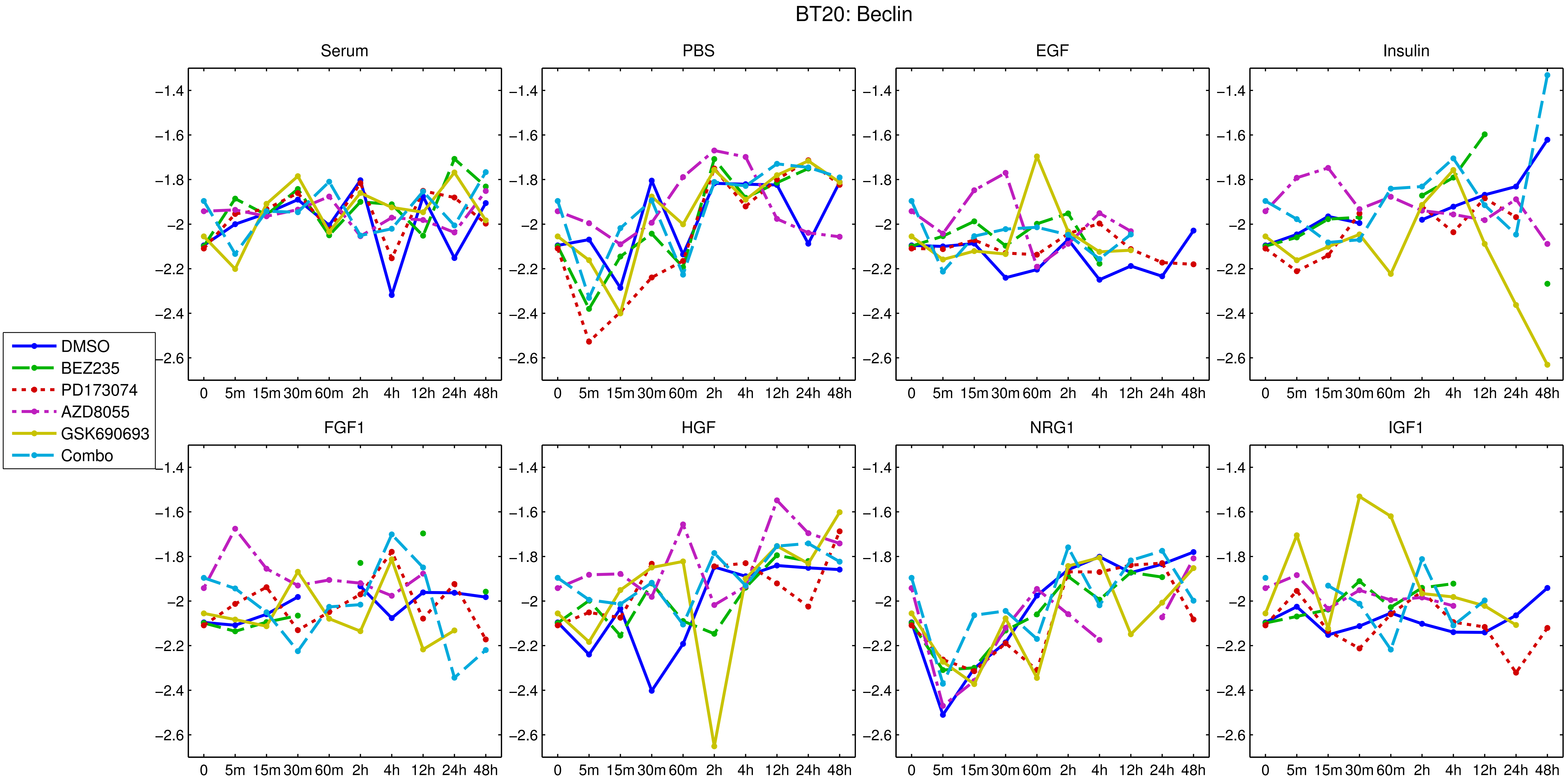

BT20: Beclin
Serum
PBS
EGF
Insulin
FGF1
HGF
NRG1
IGF1
DMSO
BEZ235
PD173074
AZD8055
GSK690693
Combo
0
5m
15m
30m
60m
2h
4h
12h
24h
48h
−1.4
−1.6
−1.8
−2
−2.2
−2.4
−2.6

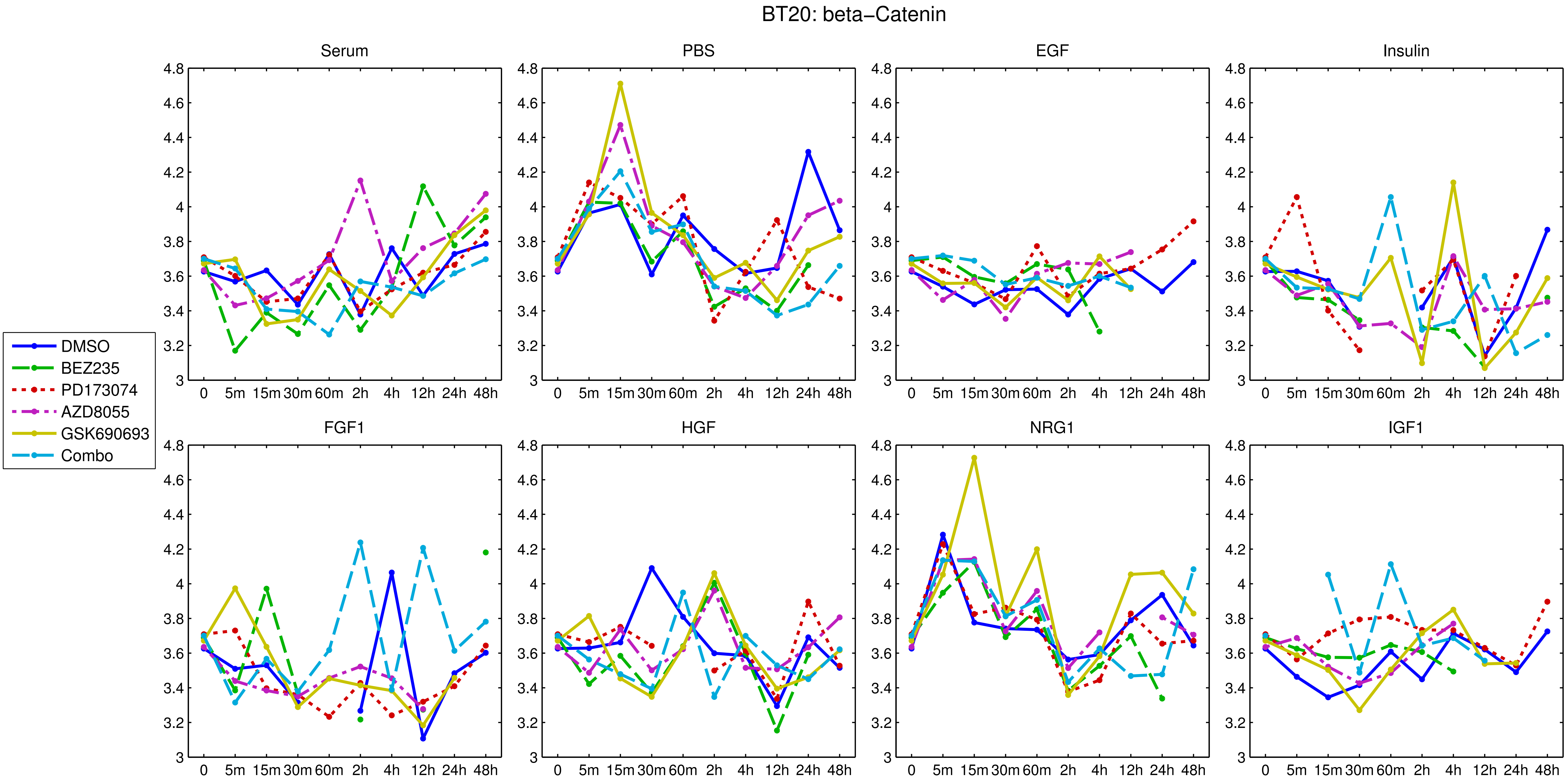
BT20: beta−Catenin
Serum
PBS
EGF
Insulin
FGF1
HGF
NRG1
IGF1
DMSO
BEZ235
PD173074
AZD8055
GSK690693
Combo
0 5m 15m 30m 60m 2h 4h 12h 24h 48h
3 3.2 3.4 3.6 3.8 4 4.2 4.4 4.6 4.8

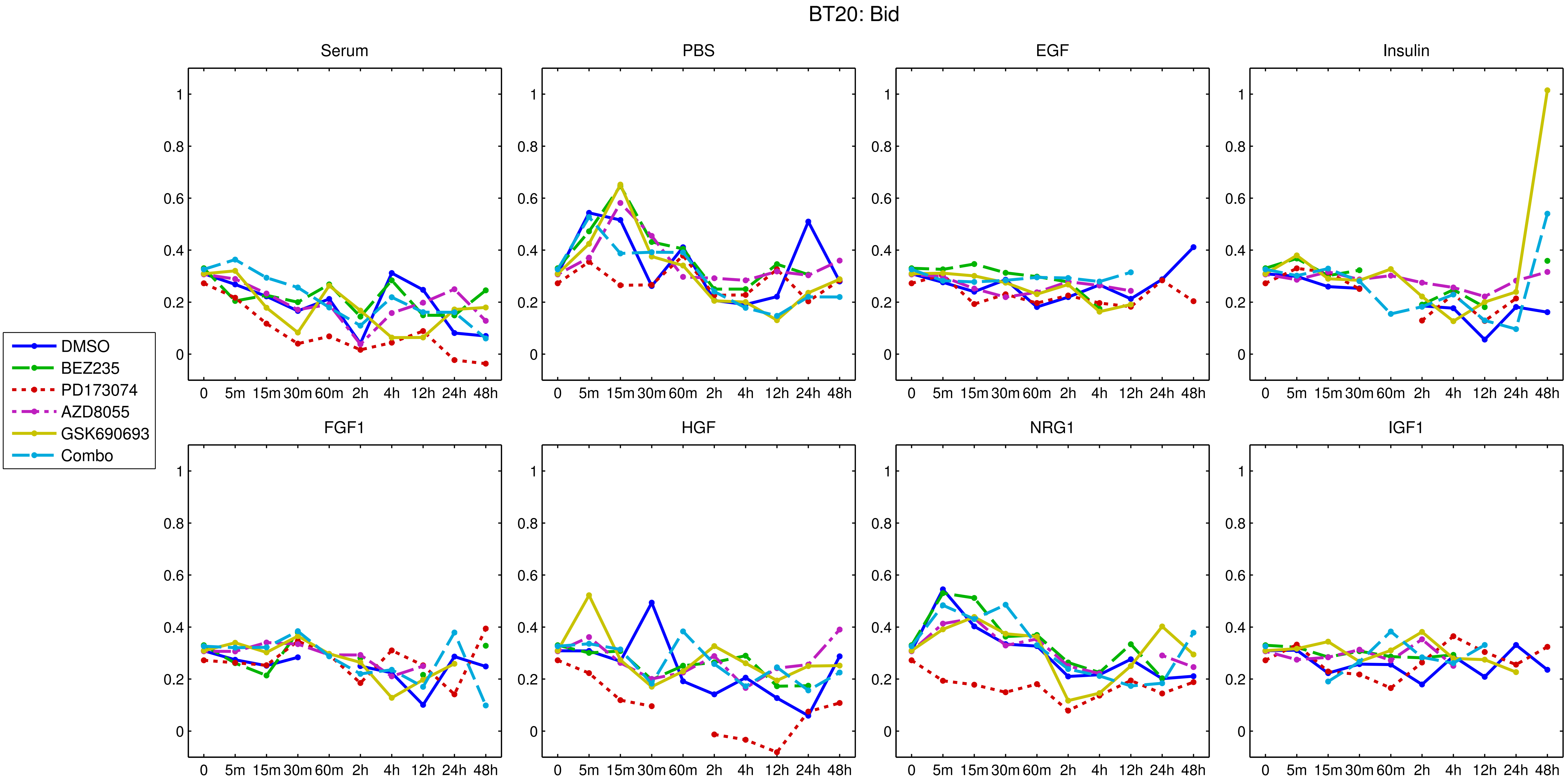

BT20: Bid
Serum
PBS
EGF
Insulin
FGF1
HGF
NRG1
IGF1
DMSO
BEZ235
PD173074
AZD8055
GSK690693
Combo
0
0.2
0.4
0.6
0.8
1
0 5m 15m 30m 60m 2h 4h 12h 24h 48h

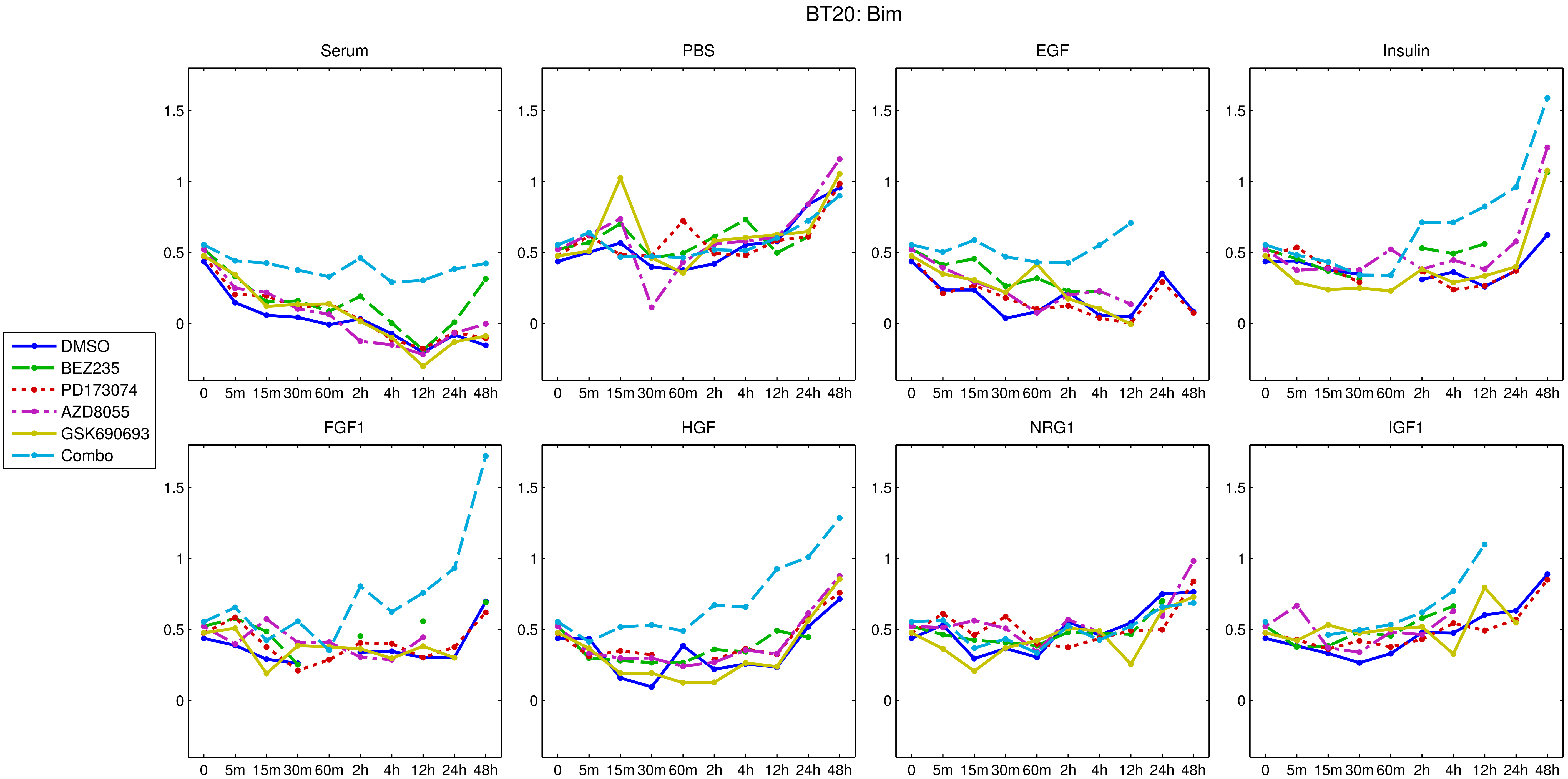
BT20: Bim
Serum
PBS
EGF
Insulin
FGF1
HGF
NRG1
IGF1
DMSO
BEZ235
PD173074
AZD8055
GSK690693
Combo
1.5
1
0.5
0
0
5m
15m
30m
60m
2h
4h
12h
24h
48h

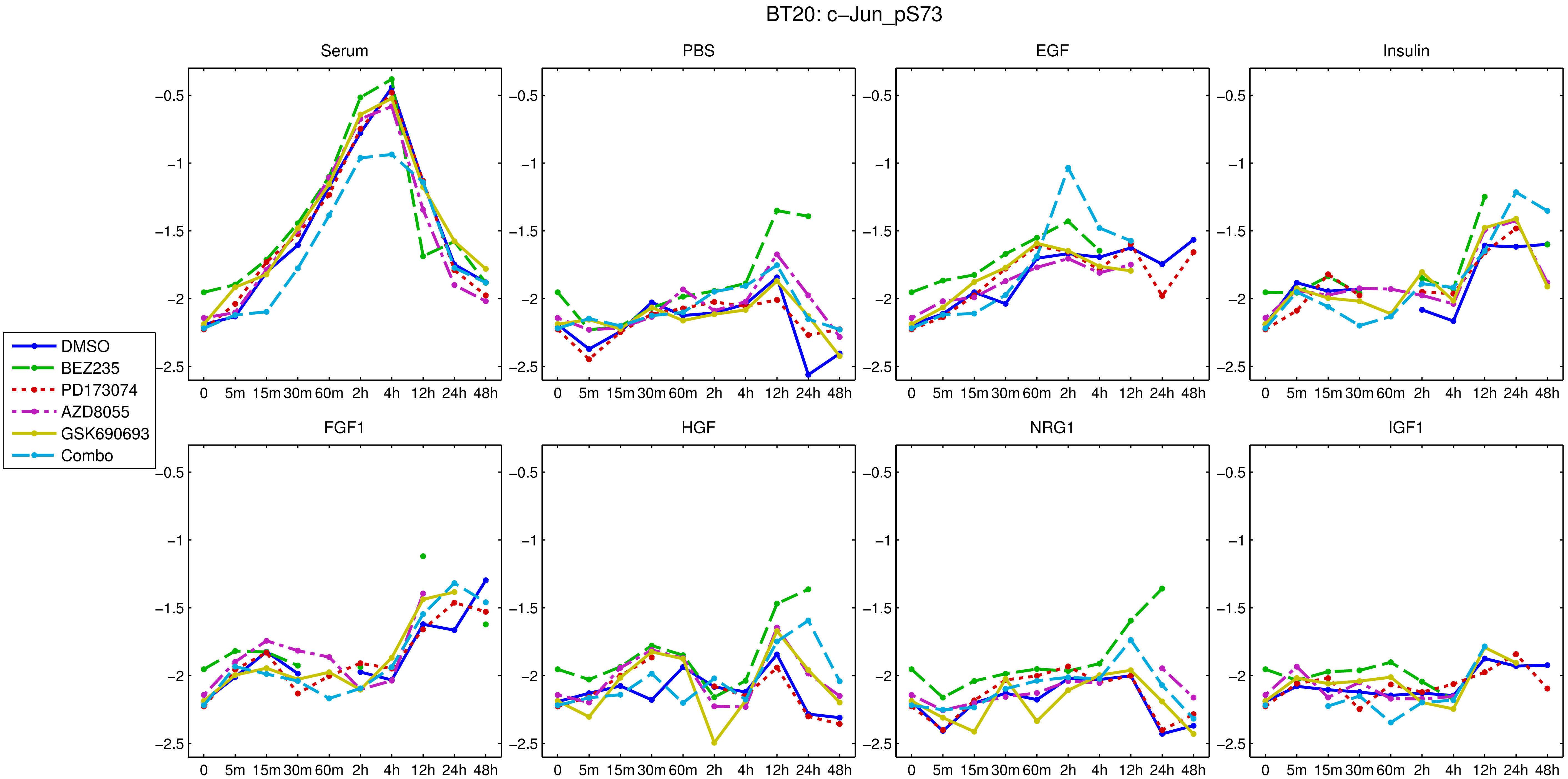
BT20: c−Jun_pS73
Serum
PBS
EGF
Insulin
FGF1
HGF
NRG1
IGF1
DMSO
BEZ235
PD173074
AZD8055
GSK690693
Combo
−0.5
−1
−1.5
−2
−2.5
0
5m
15m
30m
60m
2h
4h
12h
24h
48h

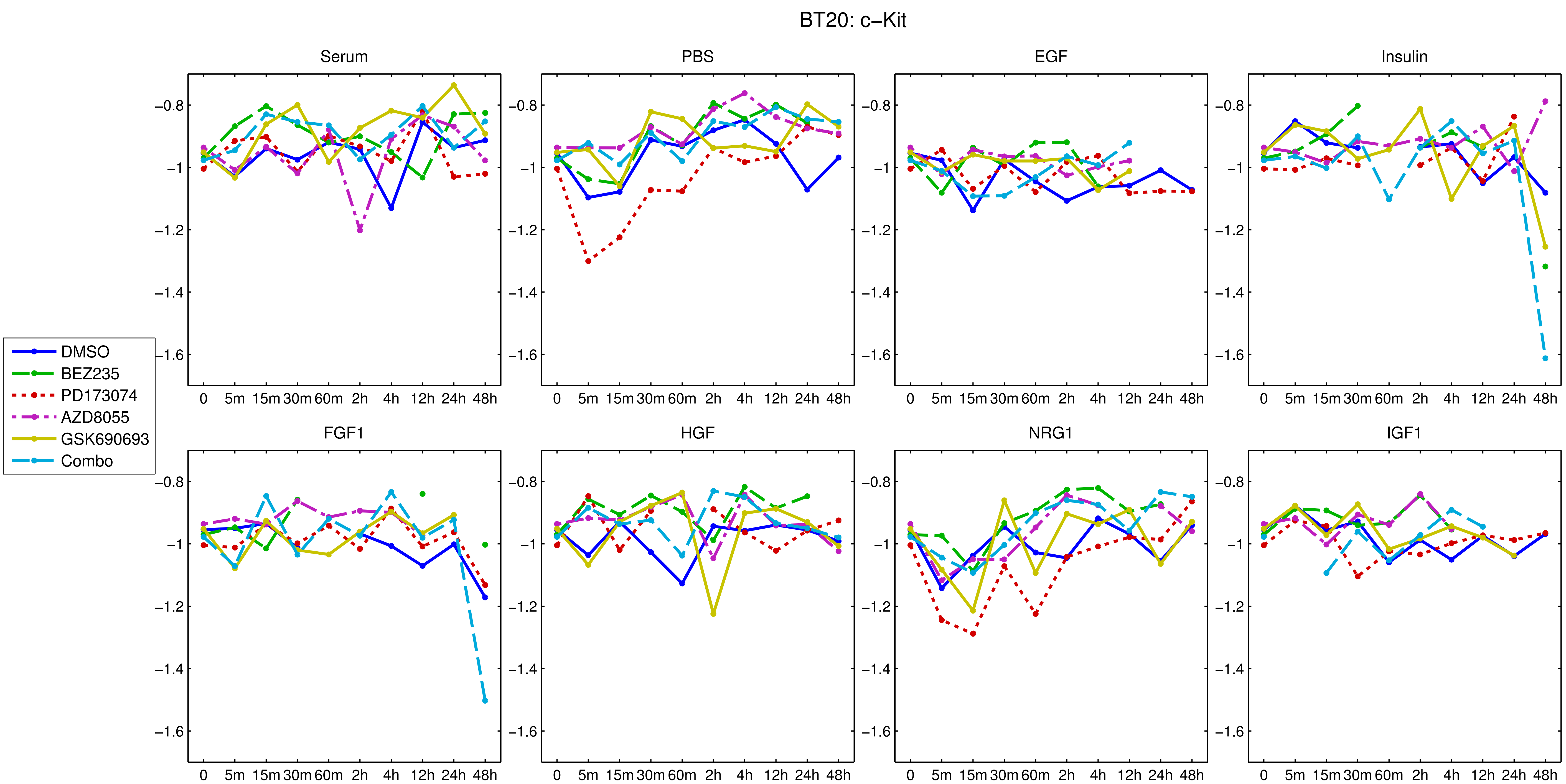

BT20: c−Kit
Serum
PBS
EGF
Insulin
FGF1
HGF
NRG1
IGF1
DMSO
BEZ235
PD173074
AZD8055
GSK690693
Combo
−0.8
−1
−1.2
−1.4
−1.6
0
5m
15m
30m
60m
2h
4h
12h
24h
48h

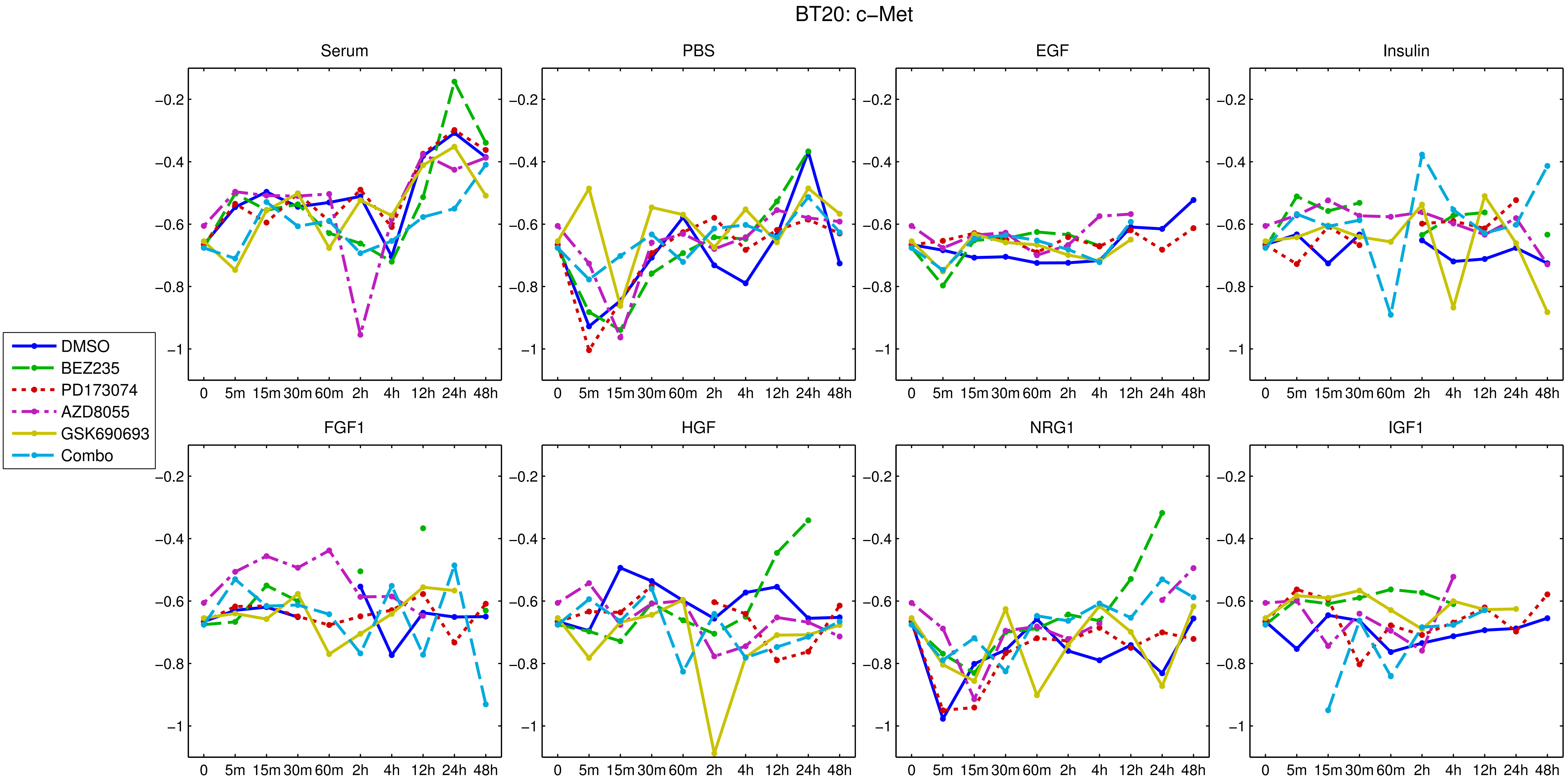
BT20: c−Met
Serum
PBS
EGF
Insulin
FGF1
HGF
NRG1
IGF1
DMSO
BEZ235
PD173074
AZD8055
GSK690693
Combo
−0.2
−0.4
−0.6
−0.8
−1
0
5m
15m
30m
60m
2h
4h
12h
24h
48h

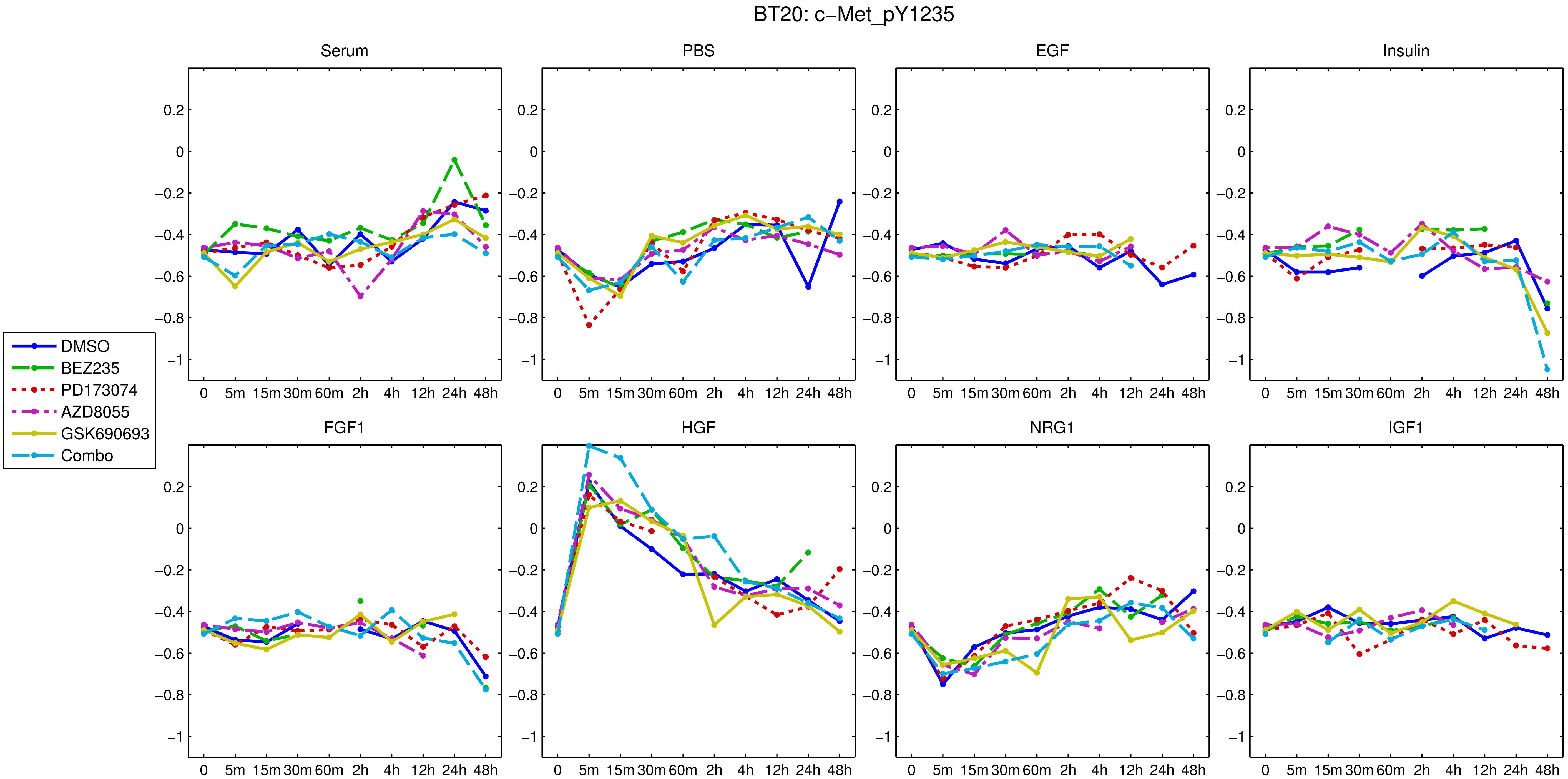

BT20: c–Met_pY1235
Serum
PBS
EGF
Insulin
FGF1
HGF
NRG1
IGF1
0.2
0
–0.2
–0.4
–0.6
–0.8
–1
0
5m
15m
30m
60m
2h
4h
12h
24h
48h
DMSO
BEZ235
PD173074
AZD8055
GSK690693
Combo

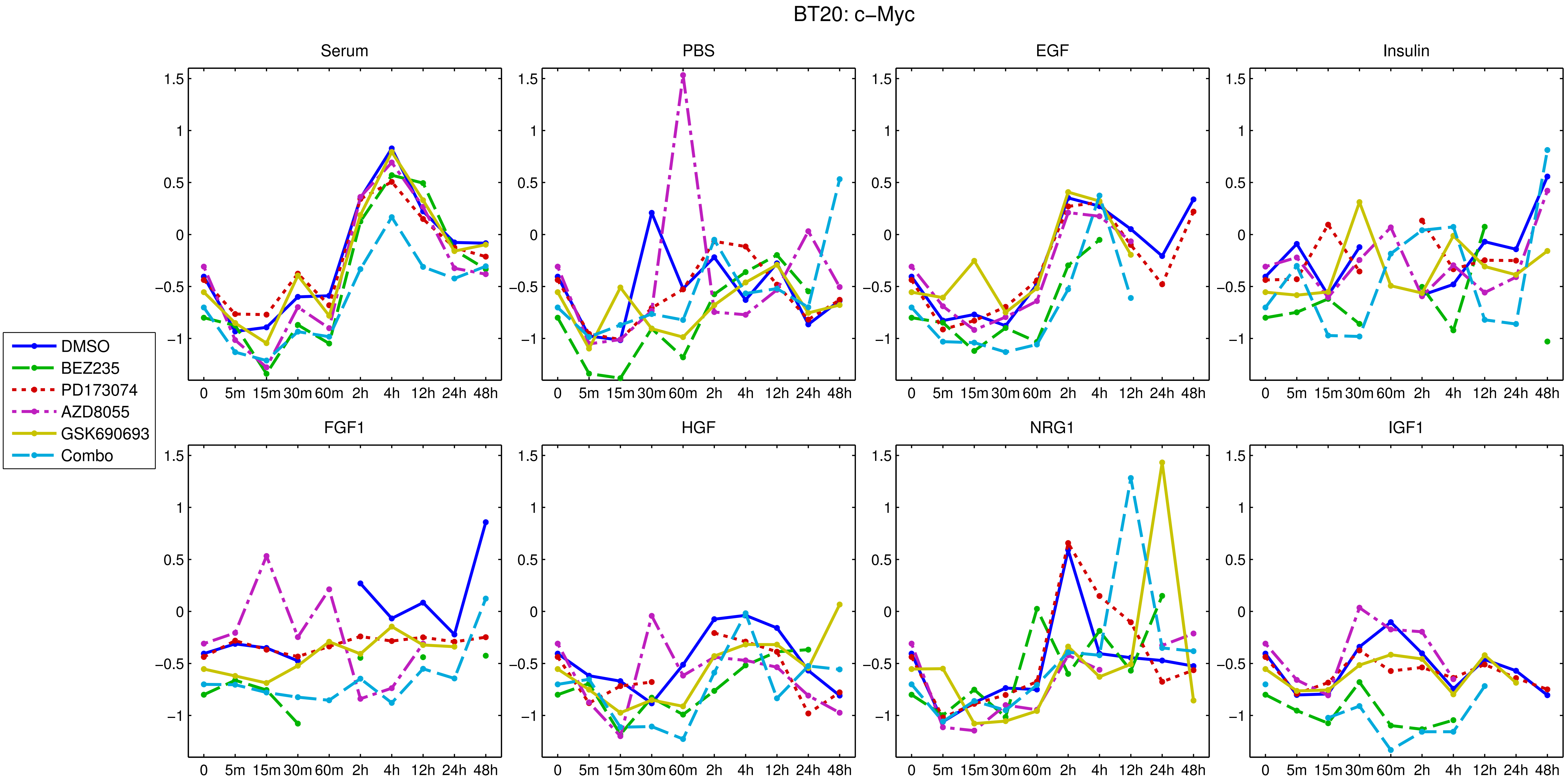

BT20: c–Myc
Serum
PBS
EGF
Insulin
FGF1
HGF
NRG1
IGF1
DMSO
BEZ235
PD173074
AZD8055
GSK690693
Combo
1.5
1
0.5
0
−0.5
−1
0
5m
15m
30m
60m
2h
4h
12h
24h
48h

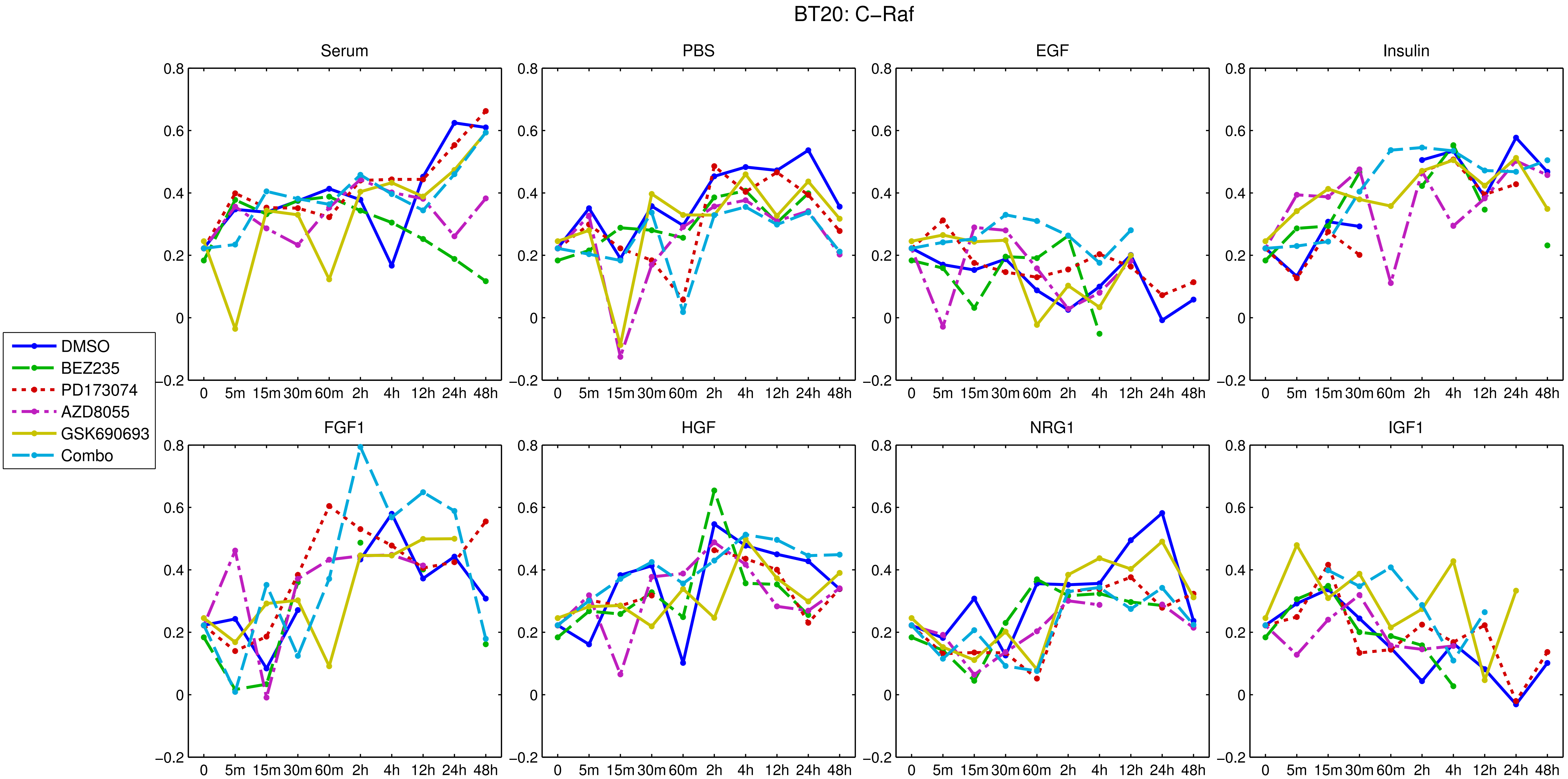

BT20: C−Raf
Serum
PBS
EGF
Insulin
FGF1
HGF
NRG1
IGF1
DMSO
BEZ235
PD173074
AZD8055
GSK690693
Combo
0.8
0.6
0.4
0.2
0
−0.2
0
5m
15m
30m
60m
2h
4h
12h
24h
48h

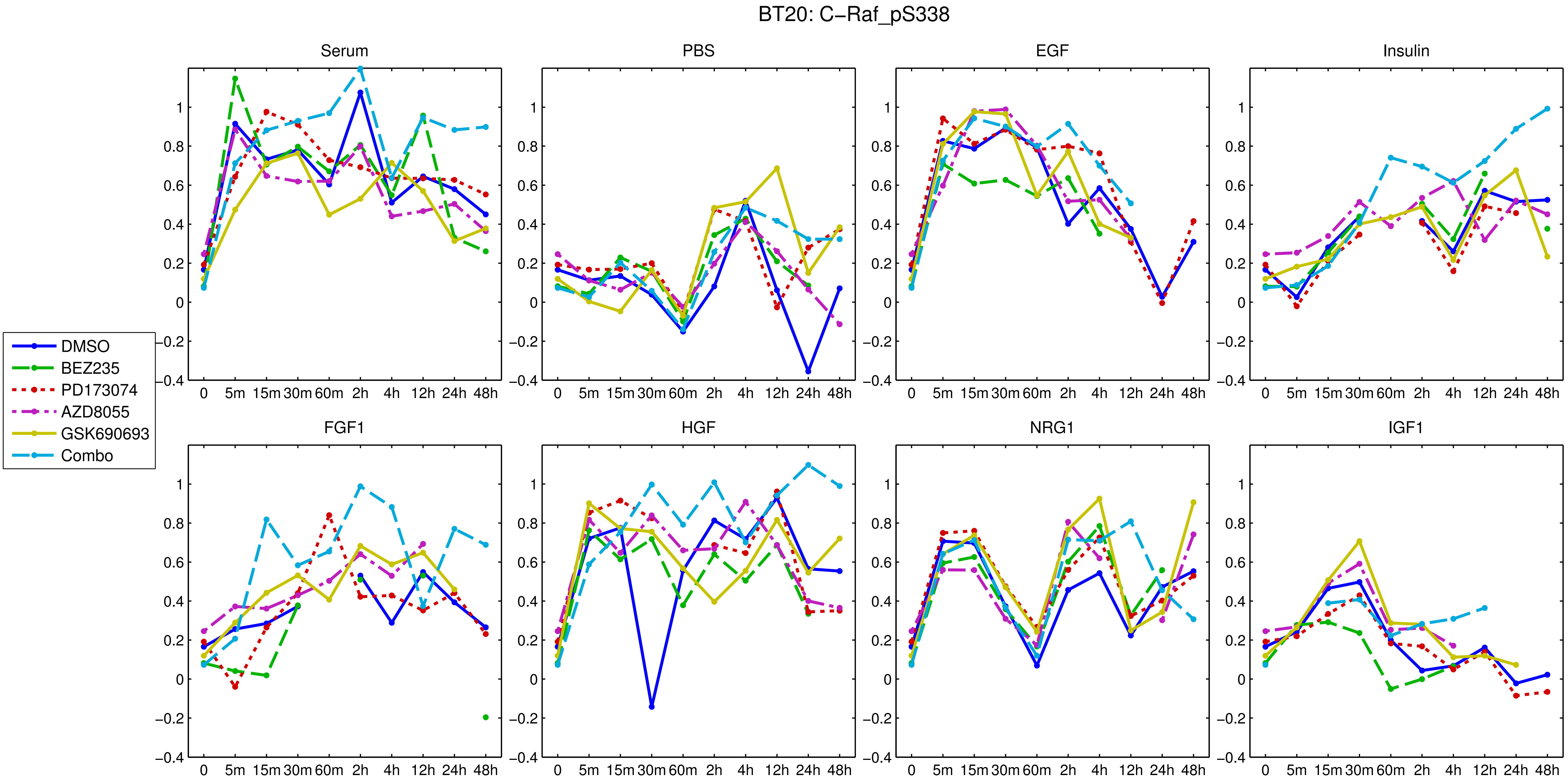
BT20: C−Raf_pS338
Serum
PBS
EGF
Insulin
FGF1
HGF
NRG1
IGF1
DMSO
BEZ235
PD173074
AZD8055
GSK690693
Combo
1
0.8
0.6
0.4
0.2
0
−0.2
−0.4
0 5m 15m 30m 60m 2h 4h 12h 24h 48h

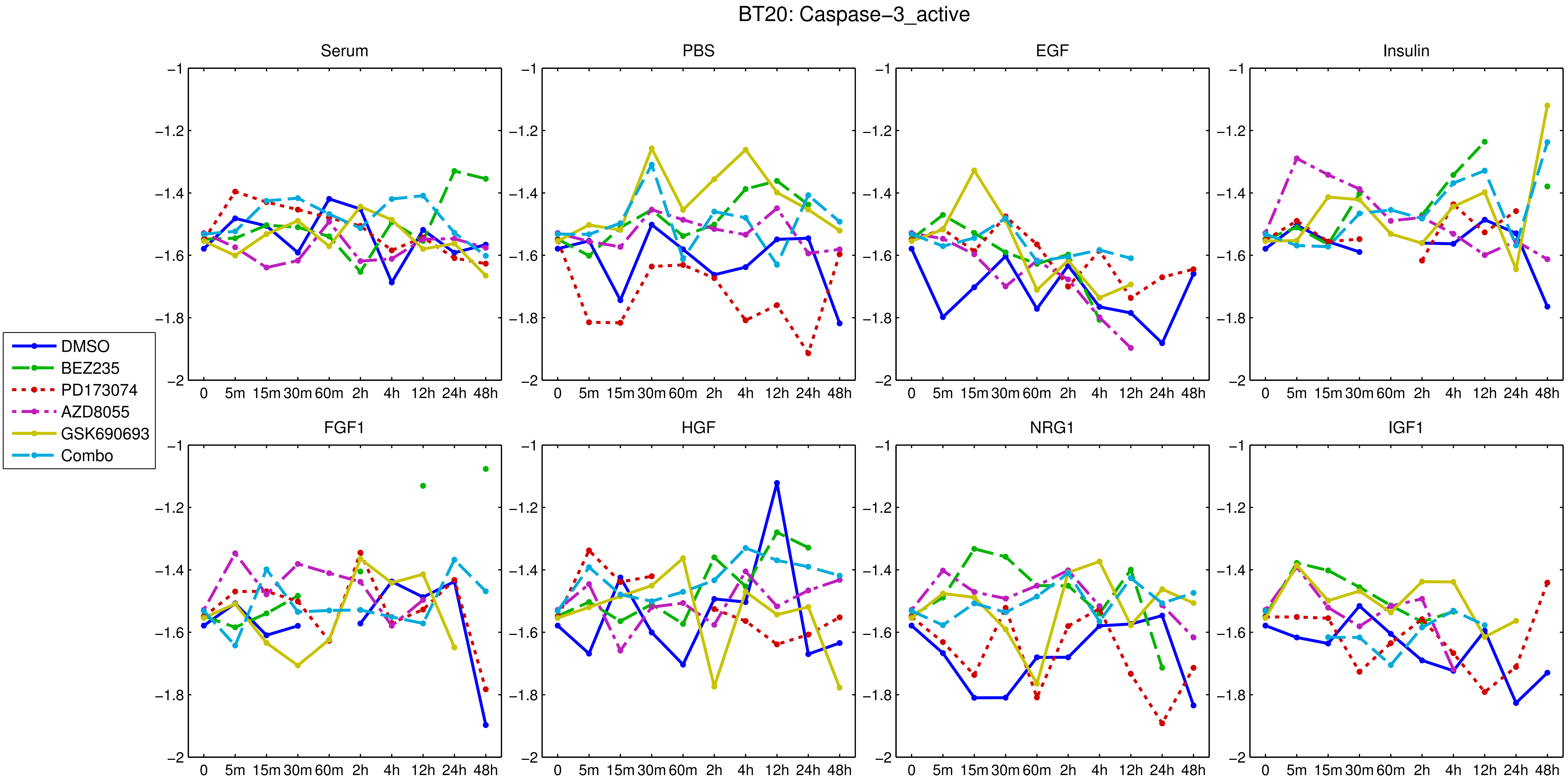
BT20: Caspase−3_active
Serum
PBS
EGF
Insulin
FGF1
HGF
NRG1
IGF1
DMSO
BEZ235
PD173074
AZD8055
GSK690693
Combo
−1
−1.2
−1.4
−1.6
−1.8
−2
0
5m
15m
30m
60m
2h
4h
12h
24h
48h

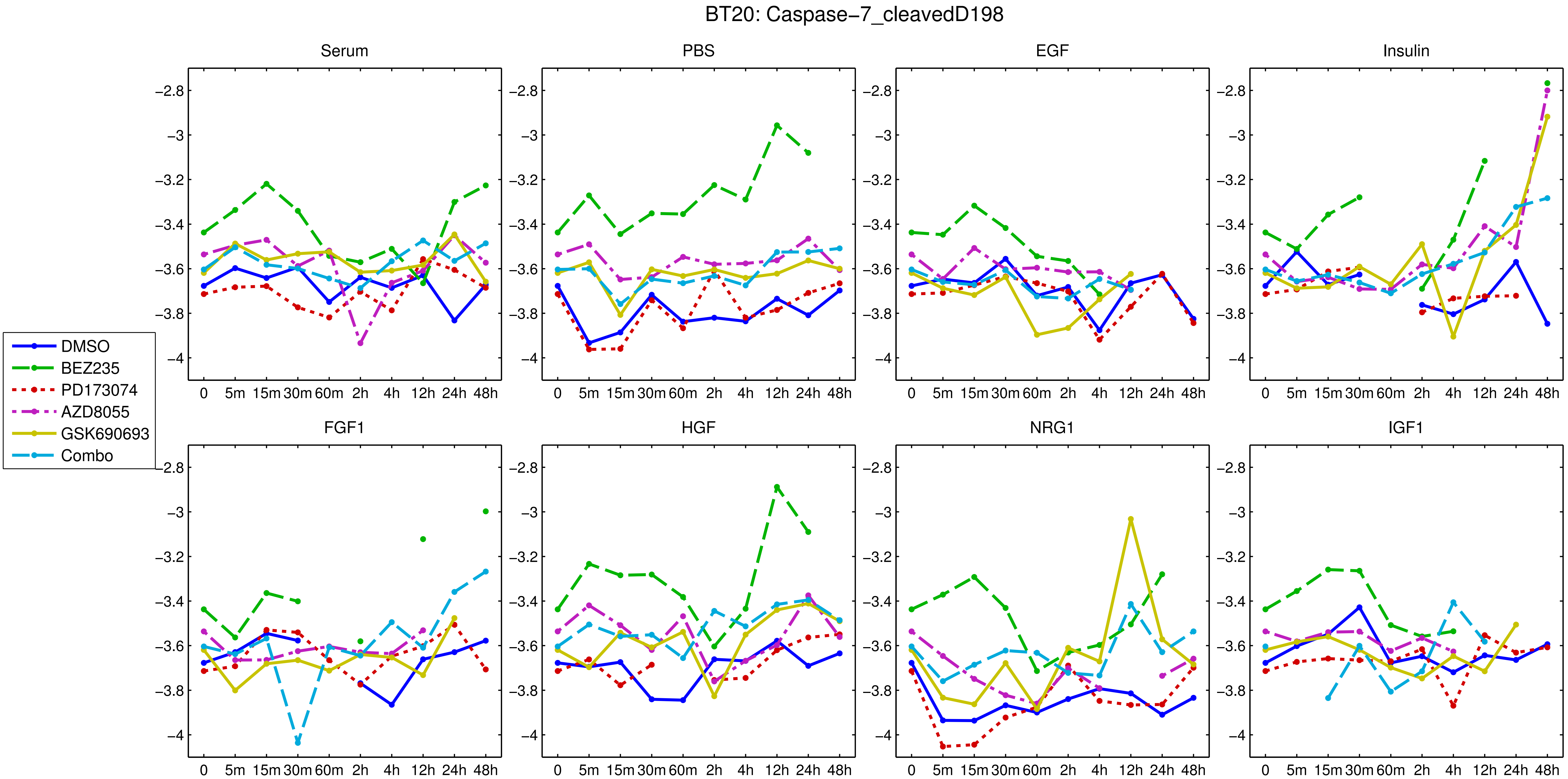
BT20: Caspase−7_cleavedD198
Serum
PBS
EGF
Insulin
FGF1
HGF
NRG1
IGF1
DMSO
BEZ235
PD173074
AZD8055
GSK690693
Combo
−2.8
−3
−3.2
−3.4
−3.6
−3.8
−4
0
5m
15m
30m
60m
2h
4h
12h
24h
48h

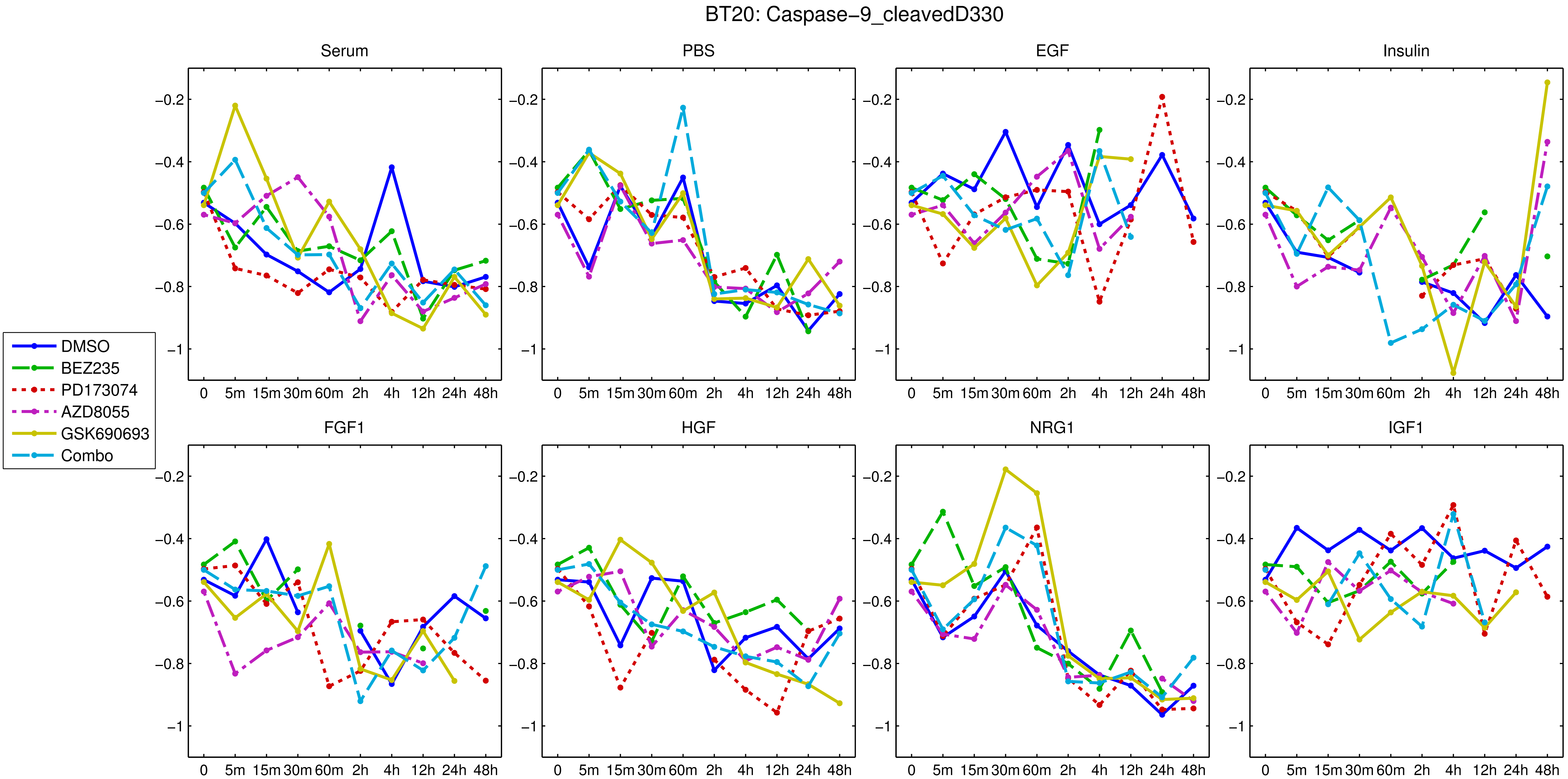
BT20: Caspase−9_cleavedD330
Serum
PBS
EGF
Insulin
FGF1
HGF
NRG1
IGF1
DMSO
BEZ235
PD173074
AZD8055
GSK690693
Combo
−0.2
−0.4
−0.6
−0.8
−1
0
5m
15m
30m
60m
2h
4h
12h
24h
48h

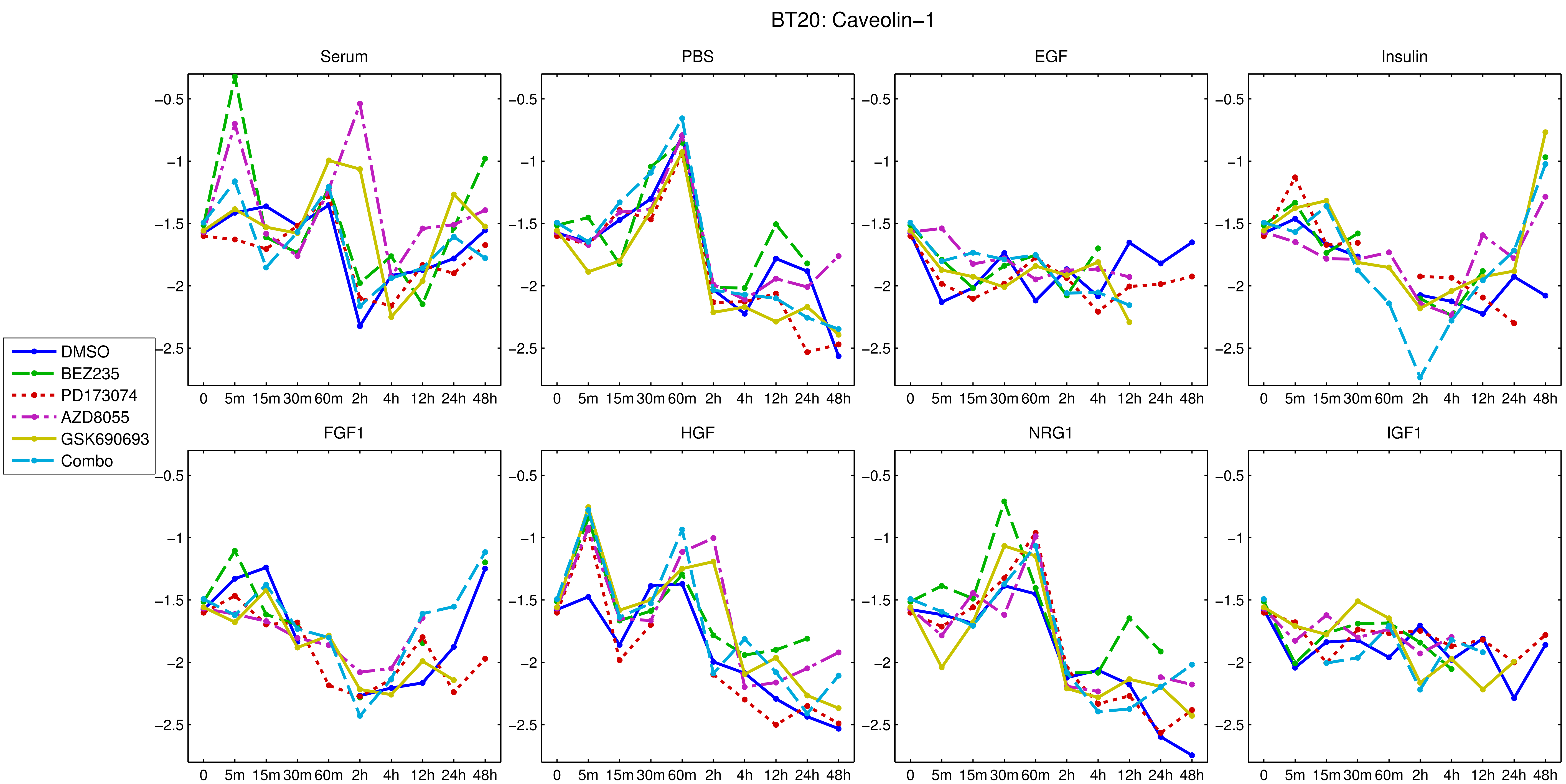

BT20: Caveolin−1
Serum
PBS
EGF
Insulin
FGF1
HGF
NRG1
IGF1
DMSO
BEZ235
PD173074
AZD8055
GSK690693
Combo
−0.5
−1
−1.5
−2
−2.5
0
5m
15m
30m
60m
2h
4h
12h
24h
48h

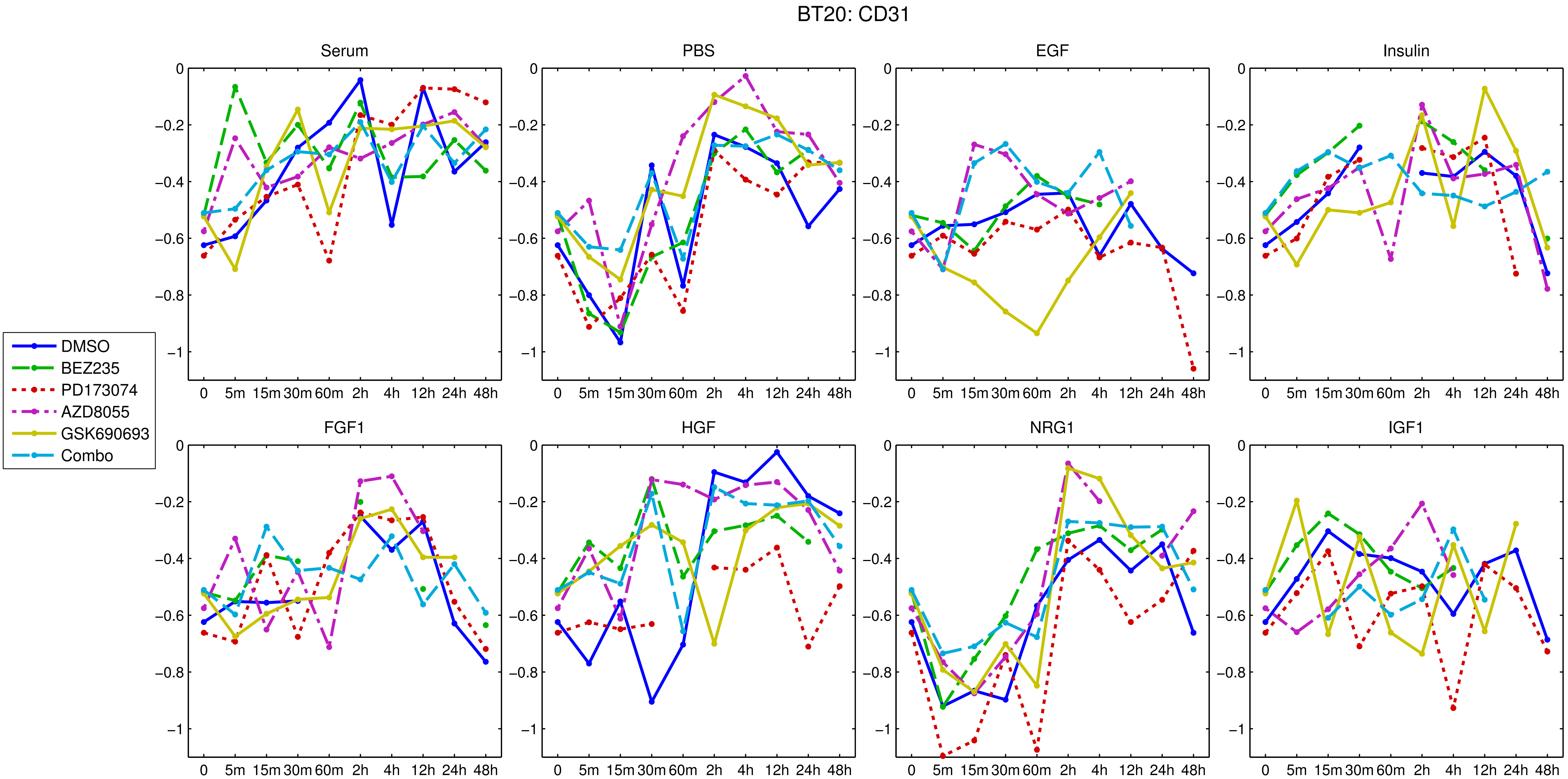
BT20: CD31
Serum
PBS
EGF
Insulin
FGF1
HGF
NRG1
IGF1
DMSO
BEZ235
PD173074
AZD8055
GSK690693
Combo
0
−0.2
−0.4
−0.6
−0.8
−1
0 5m 15m 30m 60m 2h 4h 12h 24h 48h

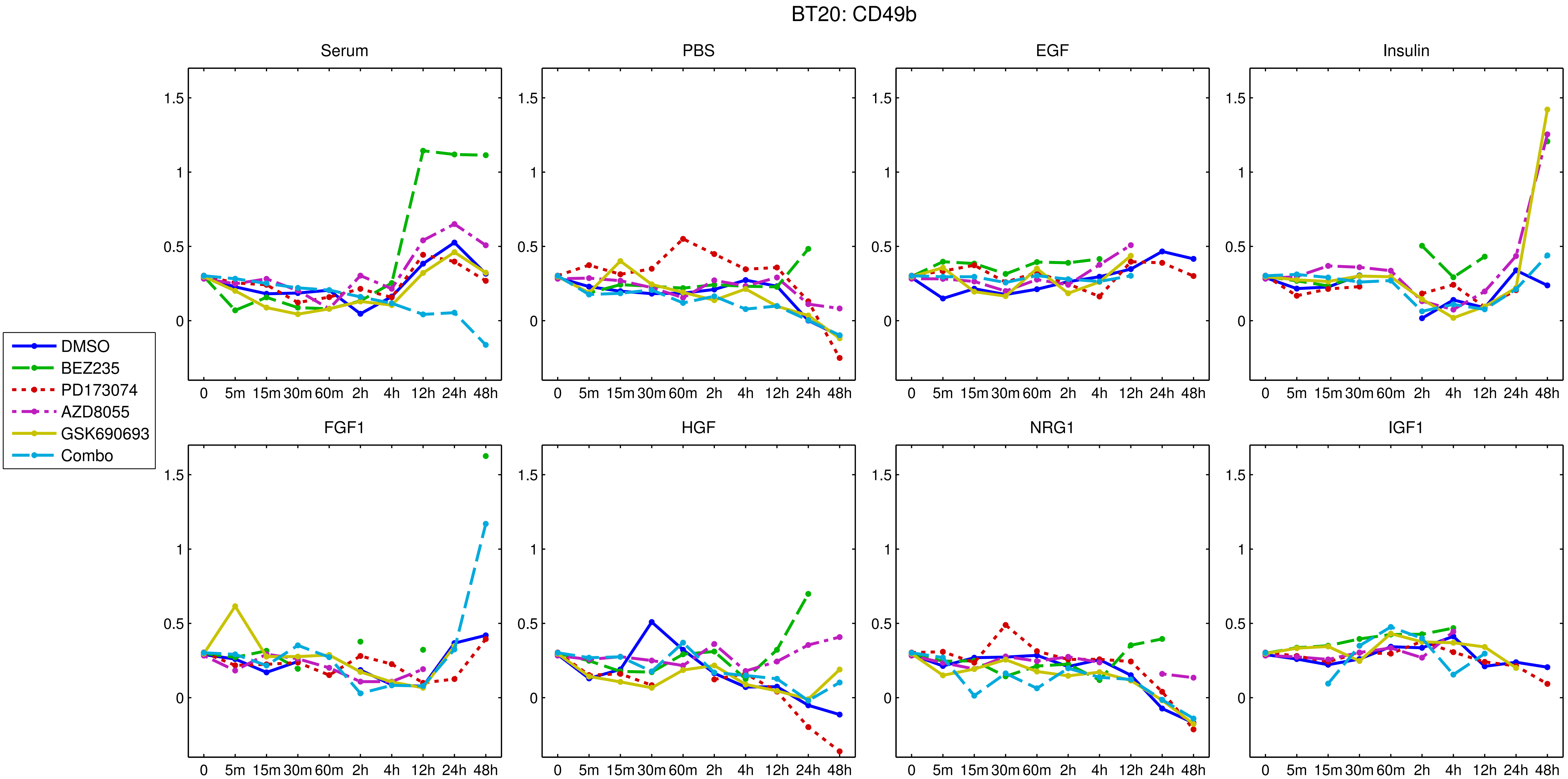
BT20: CD49b
Serum
PBS
EGF
Insulin
FGF1
HGF
NRG1
IGF1
DMSO
BEZ235
PD173074
AZD8055
GSK690693
Combo
0
0.5
1
1.5
0 5m 15m 30m 60m 2h 4h 12h 24h 48h

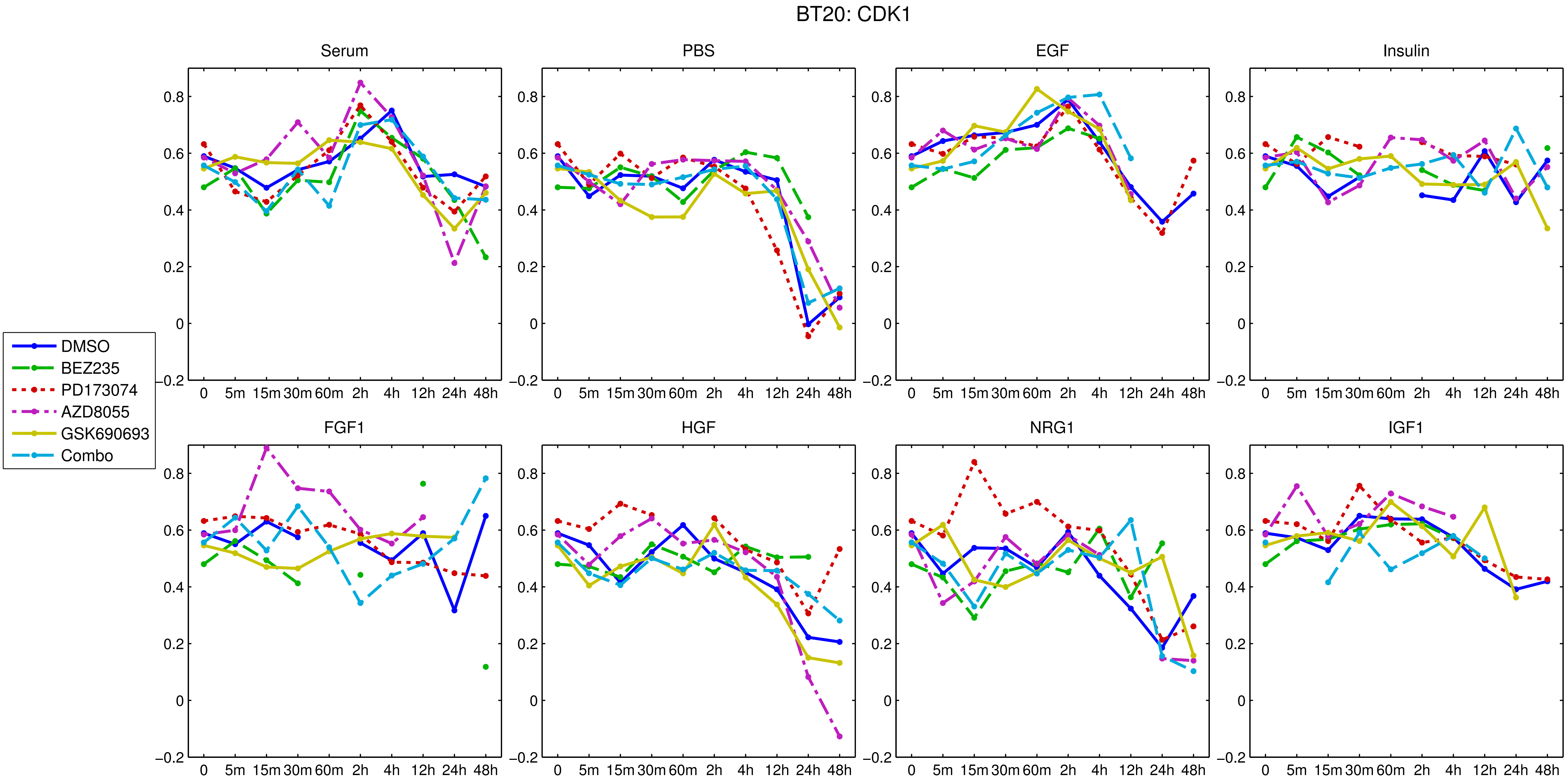

BT20: CDK1
Serum
PBS
EGF
Insulin
FGF1
HGF
NRG1
IGF1
DMSO
BEZ235
PD173074
AZD8055
GSK690693
Combo
0.8
0.6
0.4
0.2
0
−0.2
0
5m
15m
30m
60m
2h
4h
12h
24h
48h

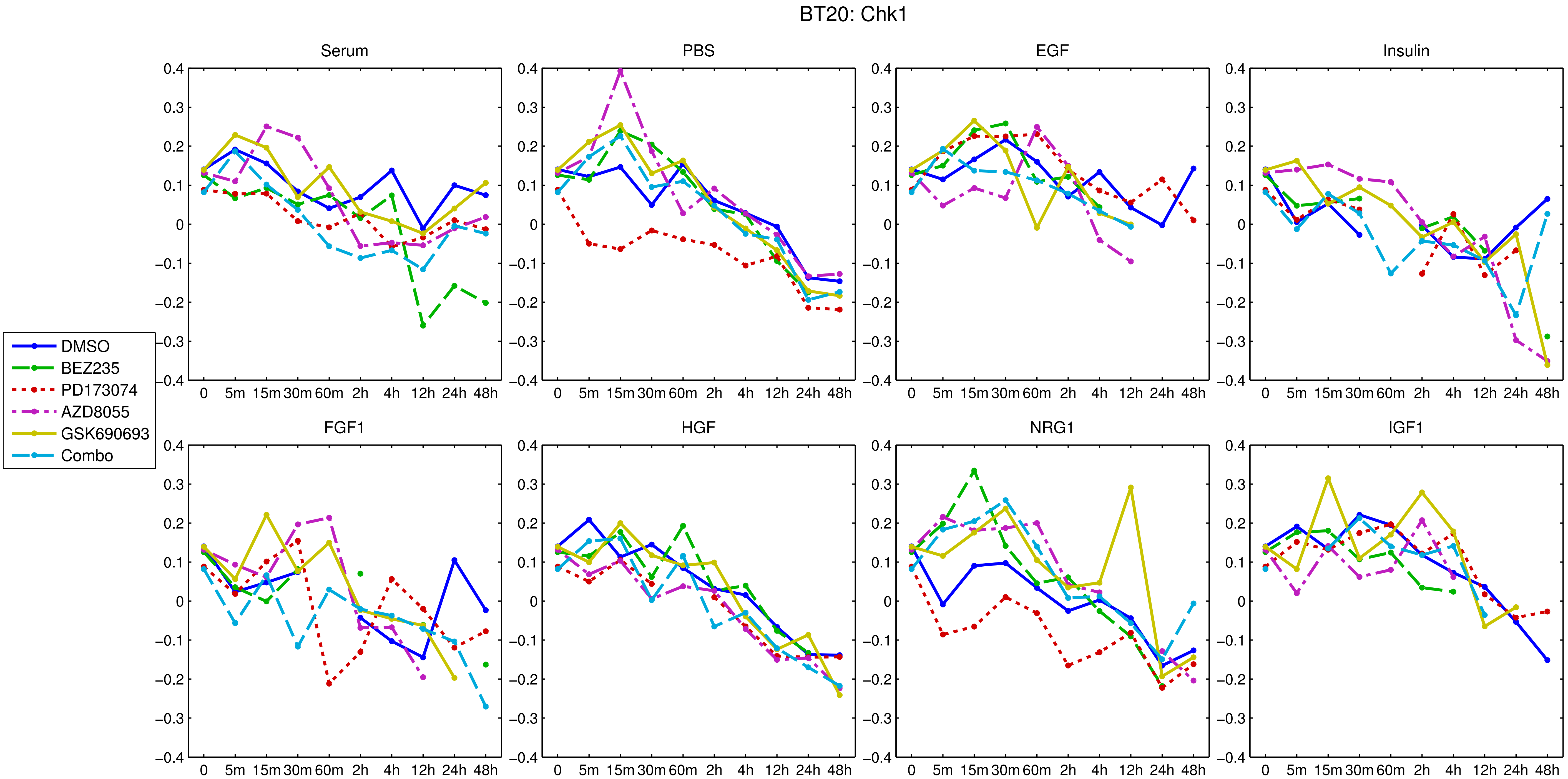
BT20: Chk1
Serum
PBS
EGF
Insulin
FGF1
HGF
NRG1
IGF1
DMSO
BEZ235
PD173074
AZD8055
GSK690693
Combo
0.4
0.3
0.2
0.1
0
−0.1
−0.2
−0.3
−0.4
0
5m
15m
30m
60m
2h
4h
12h
24h
48h

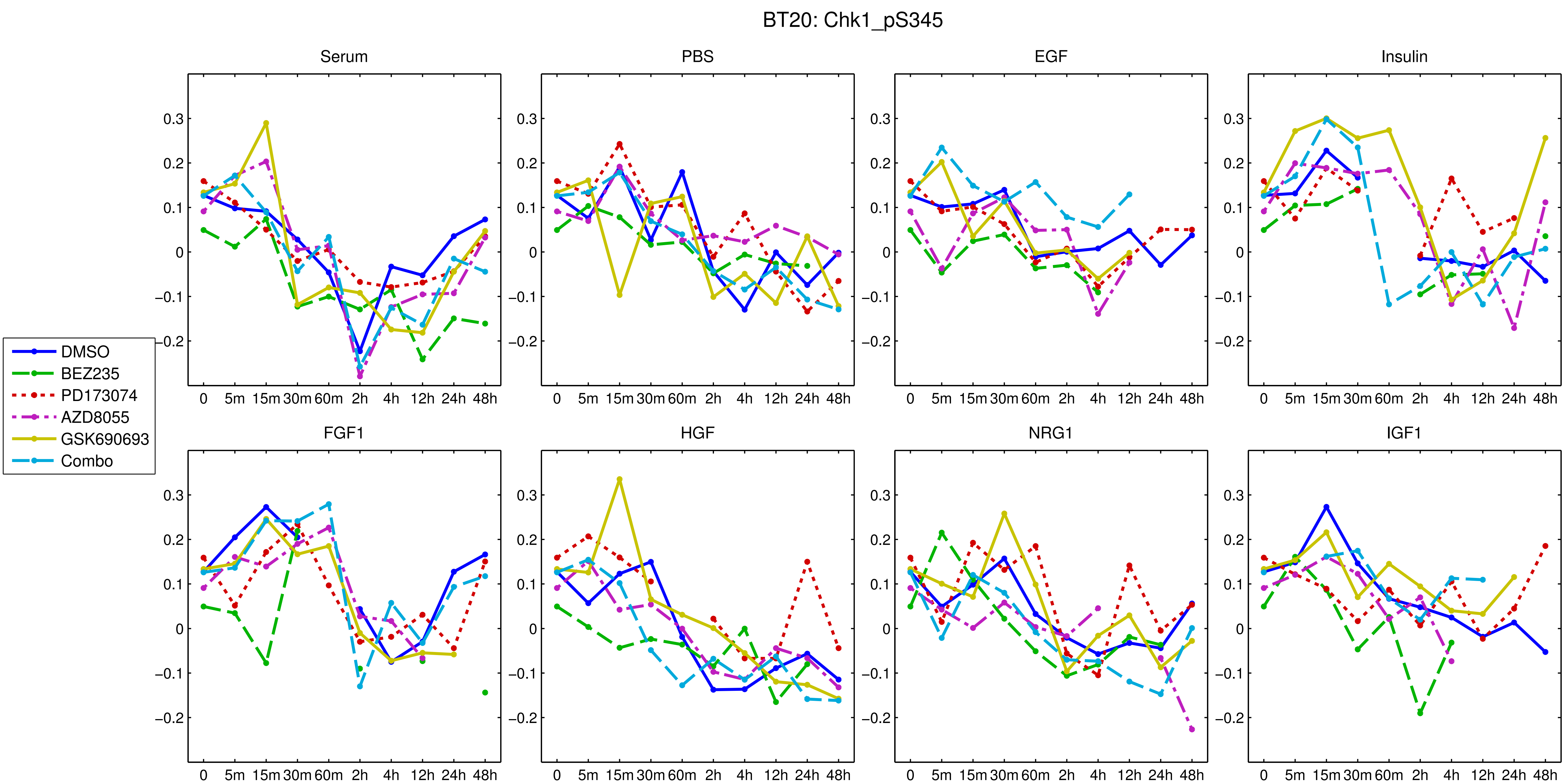
BT20: Chk1_pS345
Serum
PBS
EGF
Insulin
FGF1
HGF
NRG1
IGF1
DMSO
BEZ235
PD173074
AZD8055
GSK690693
Combo
0.3
0.2
0.1
0
−0.1
−0.2
0 5m 15m 30m 60m 2h 4h 12h 24h 48h

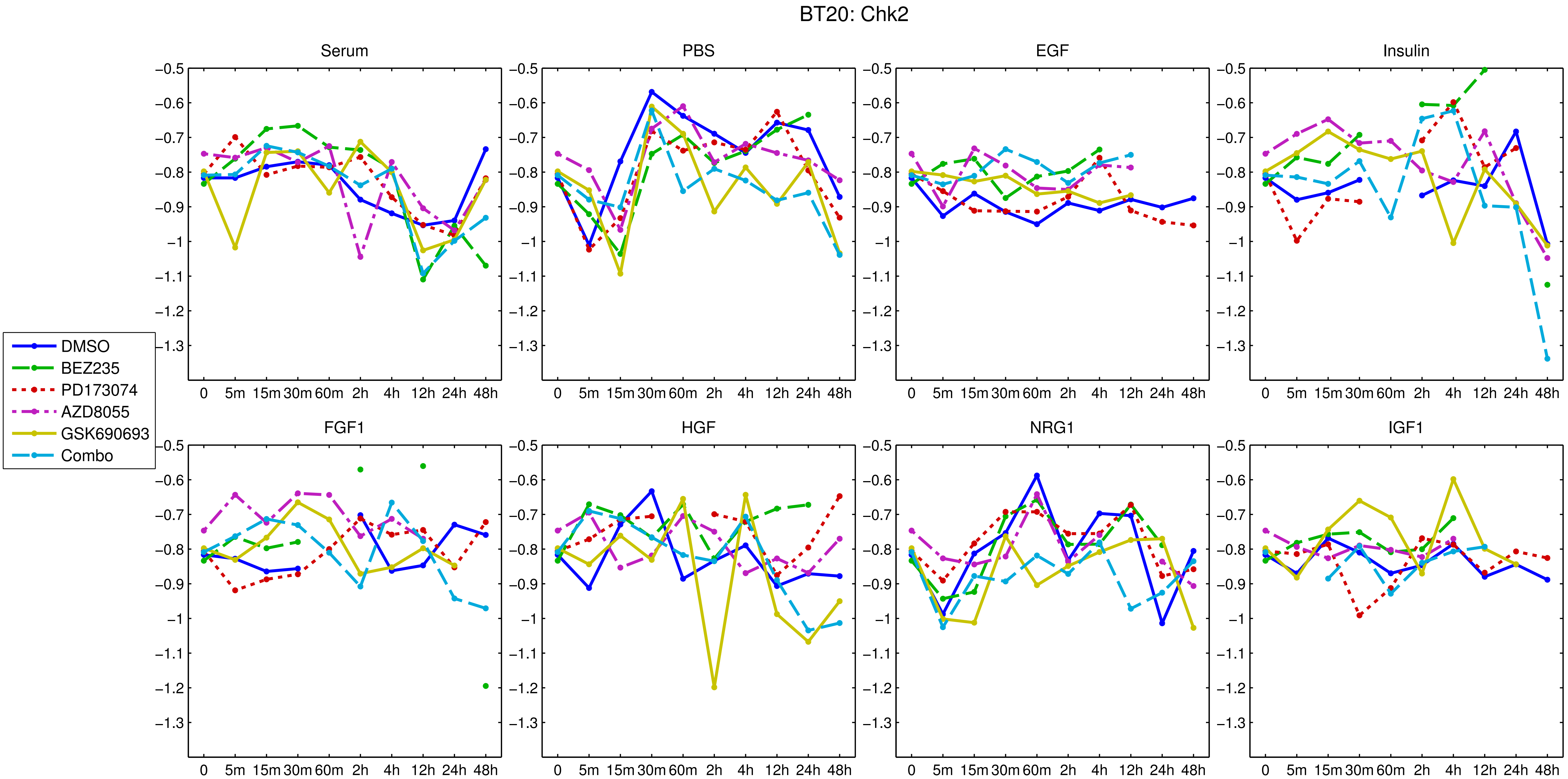
BT20: Chk2
Serum
PBS
EGF
Insulin
FGF1
HGF
NRG1
IGF1
DMSO
BEZ235
PD173074
AZD8055
GSK690693
Combo
−0.5
−0.6
−0.7
−0.8
−0.9
−1
−1.1
−1.2
−1.3
0
5m
15m
30m
60m
2h
4h
12h
24h
48h

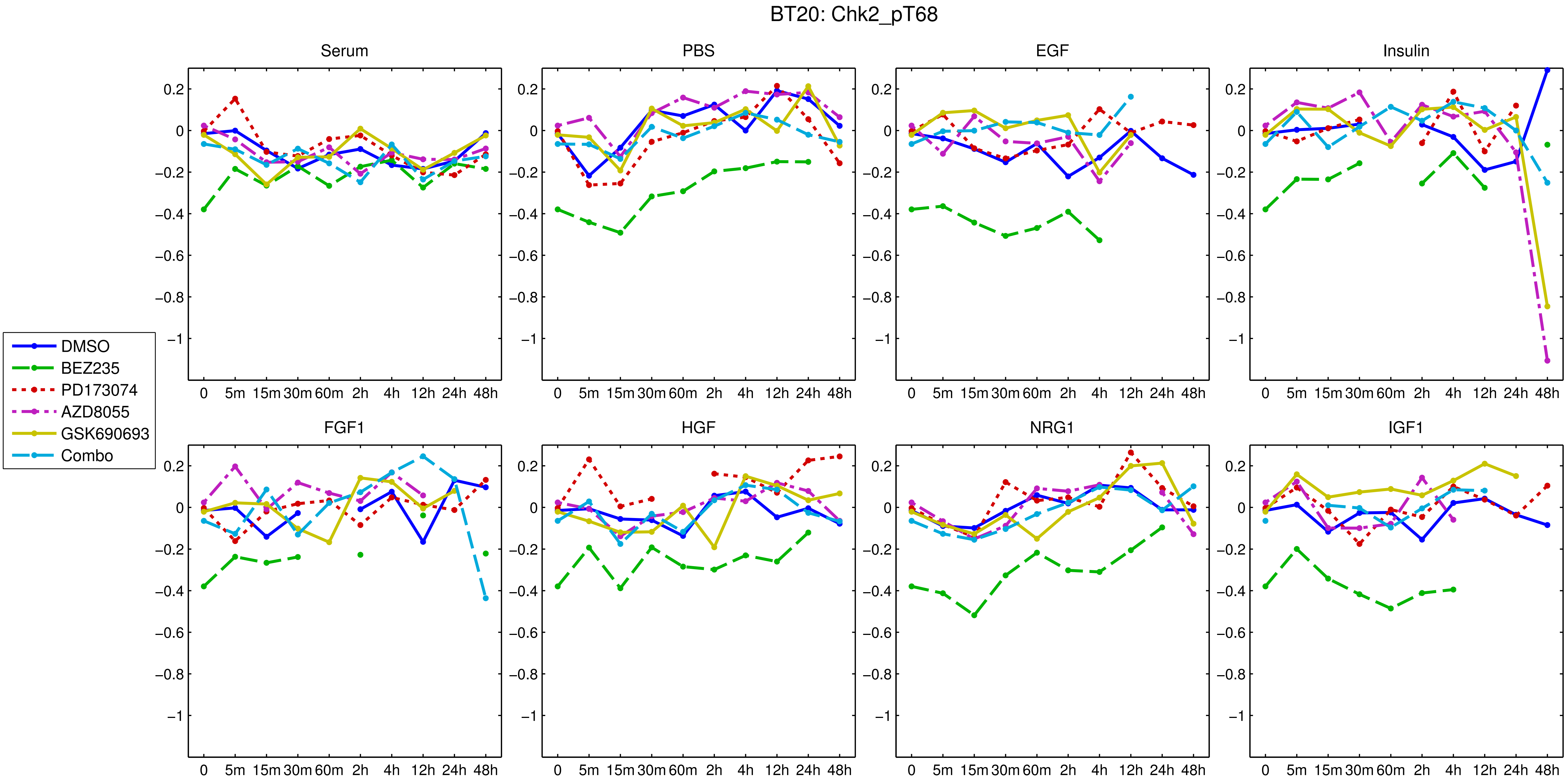
BT20: Chk2_pT68
Serum
PBS
EGF
Insulin
FGF1
HGF
NRG1
IGF1
DMSO
BEZ235
PD173074
AZD8055
GSK690693
Combo
0
5m
15m
30m
60m
2h
4h
12h
24h
48h
0.2
0
−0.2
−0.4
−0.6
−0.8
−1

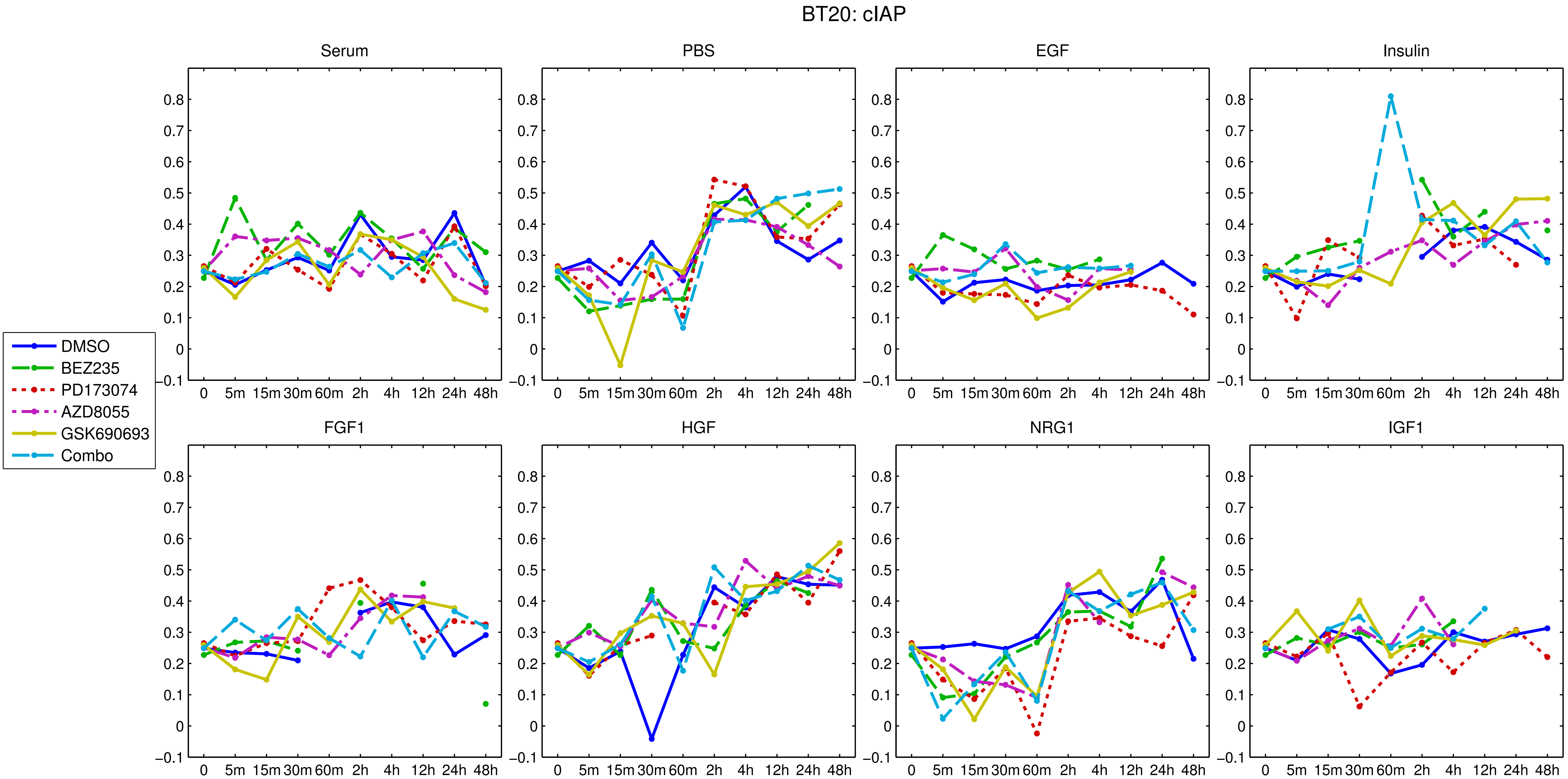
BT20: cIAP
Serum
PBS
EGF
Insulin
FGF1
HGF
NRG1
IGF1
DMSO
BEZ235
PD173074
AZD8055
GSK690693
Combo
0.8
0.7
0.6
0.5
0.4
0.3
0.2
0.1
0
−0.1
0
5m
15m
30m
60m
2h
4h
12h
24h
48h

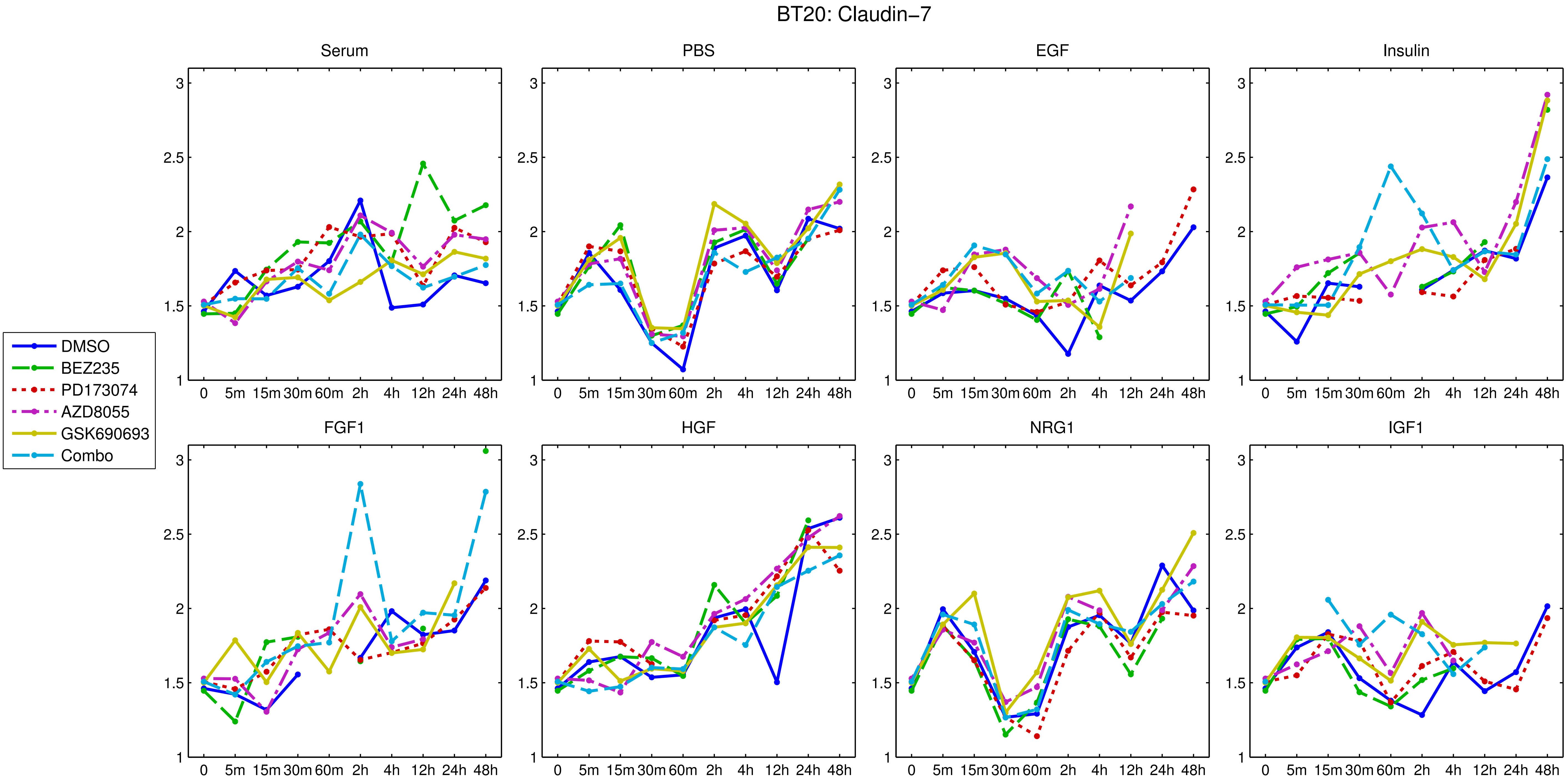

BT20: Claudin−7
Serum
PBS
EGF
Insulin
FGF1
HGF
NRG1
IGF1
DMSO
BEZ235
PD173074
AZD8055
GSK690693
Combo
0
5m
15m
30m
60m
2h
4h
12h
24h
48h
1
1.5
2
2.5
3

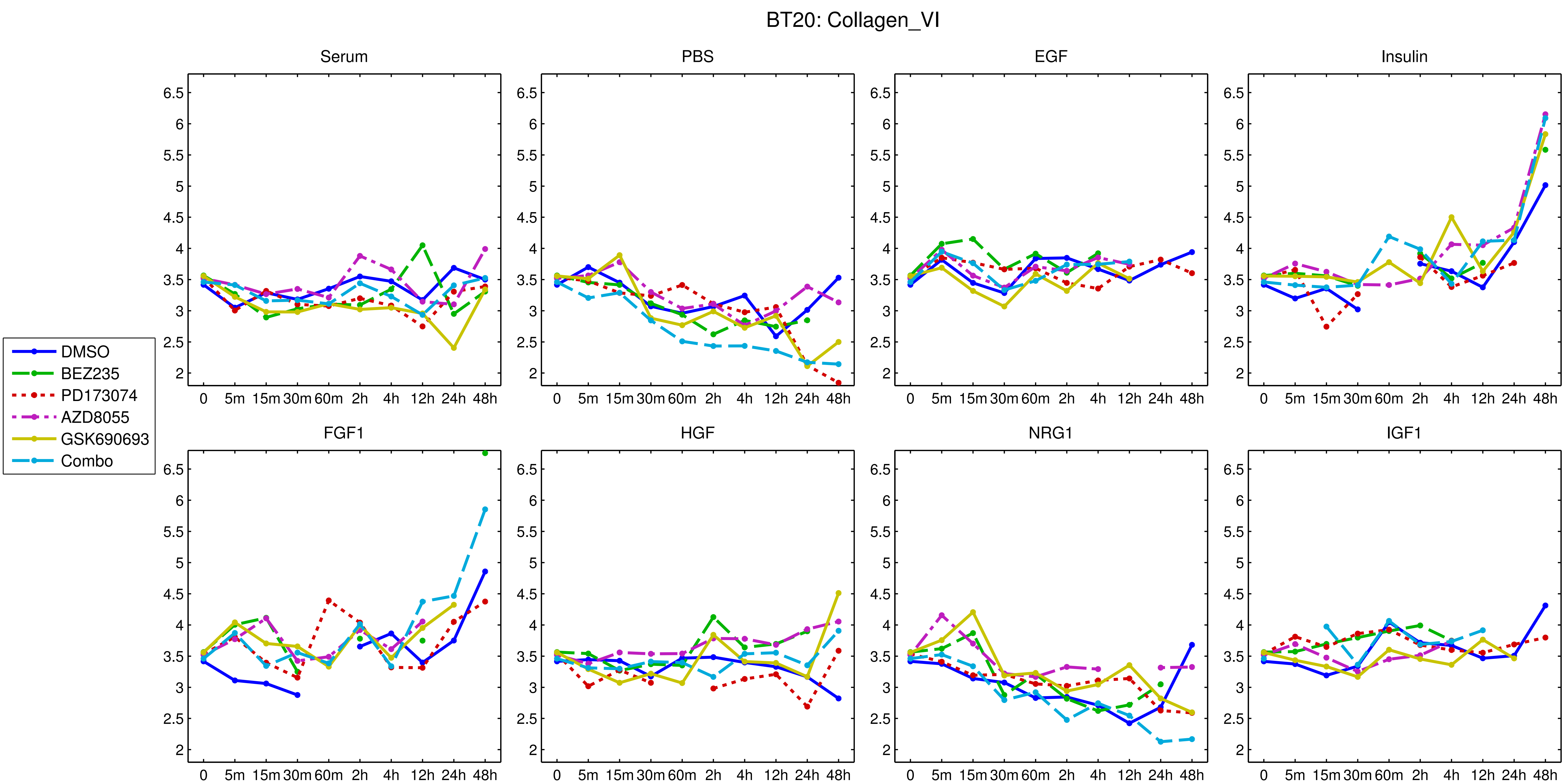
BT20: Collagen_VI
Serum
PBS
EGF
Insulin
FGF1
HGF
NRG1
IGF1
DMSO
BEZ235
PD173074
AZD8055
GSK690693
Combo
2
2.5
3
3.5
4
4.5
5
5.5
6
6.5
0
5m
15m
30m
60m
2h
4h
12h
24h
48h

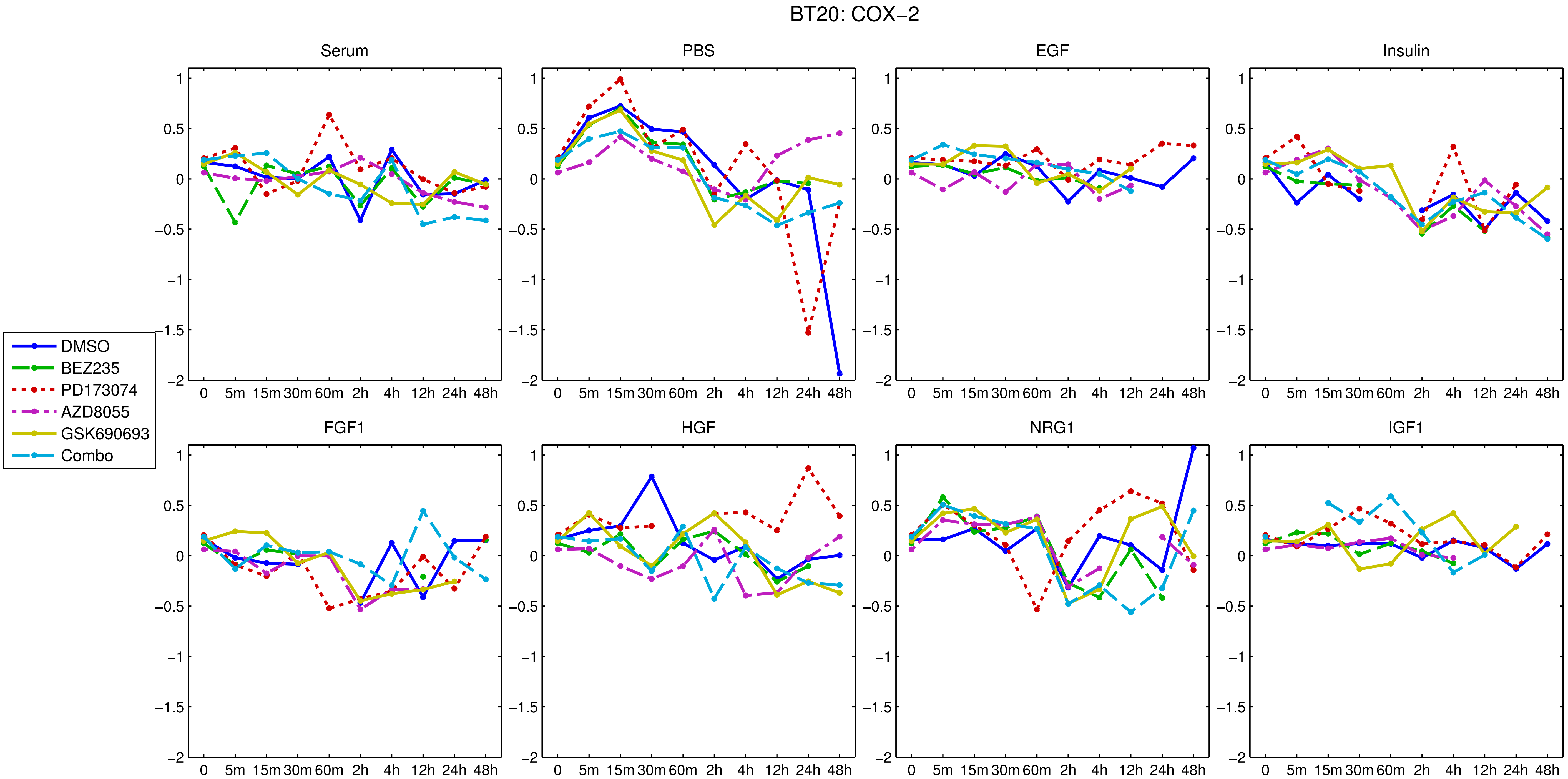
BT20: COX−2
Serum
PBS
EGF
Insulin
FGF1
HGF
NRG1
IGF1
DMSO
BEZ235
PD173074
AZD8055
GSK690693
Combo
1
0.5
0
−0.5
−1
−1.5
−2
0
5m
15m
30m
60m
2h
4h
12h
24h
48h

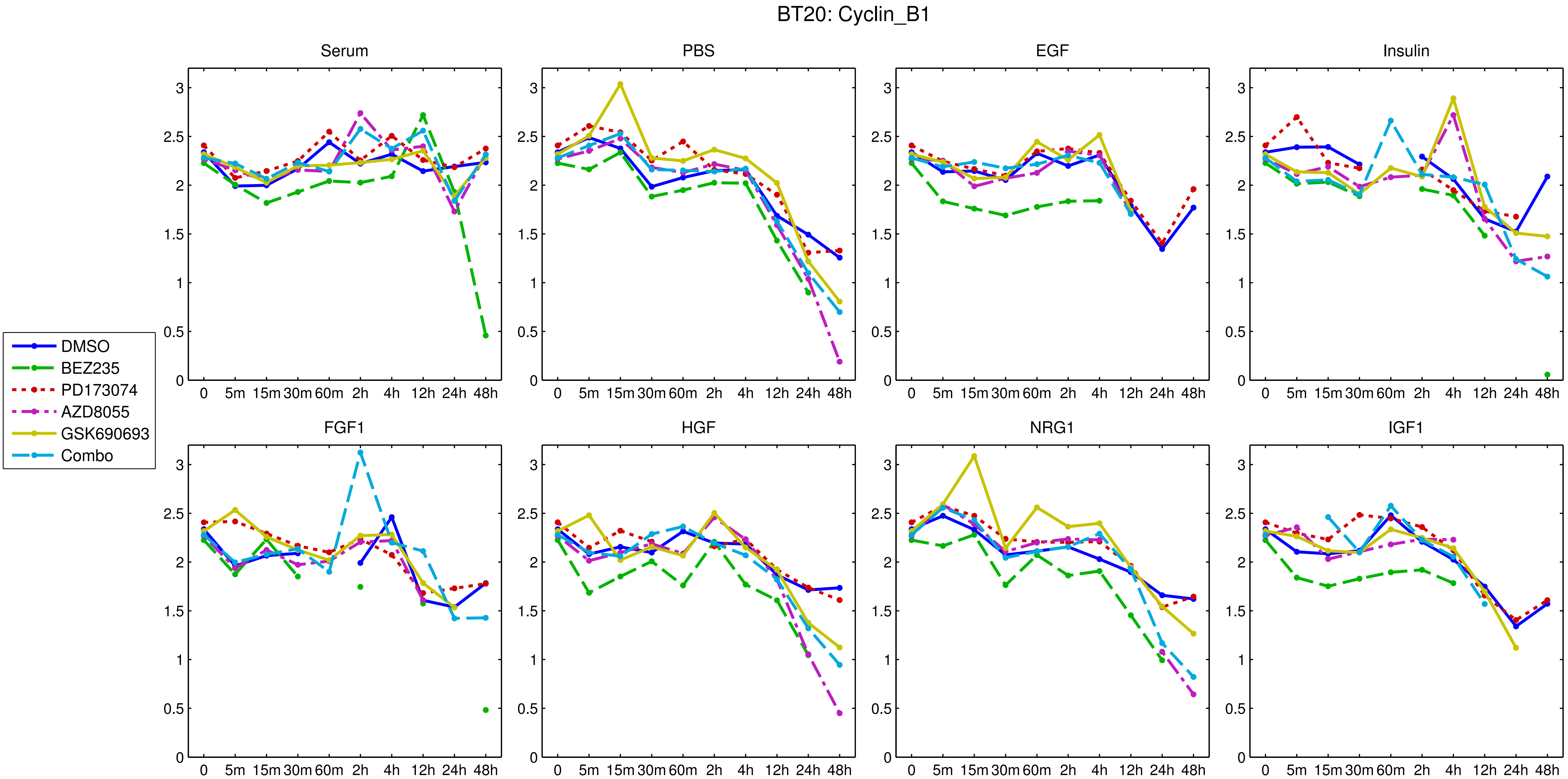
BT20: Cyclin_B1
Serum
PBS
EGF
Insulin
FGF1
HGF
NRG1
IGF1
0
0.5
1
1.5
2
2.5
3
0 5m 15m 30m 60m 2h 4h 12h 24h 48h
DMSO
BEZ235
PD173074
AZD8055
GSK690693
Combo

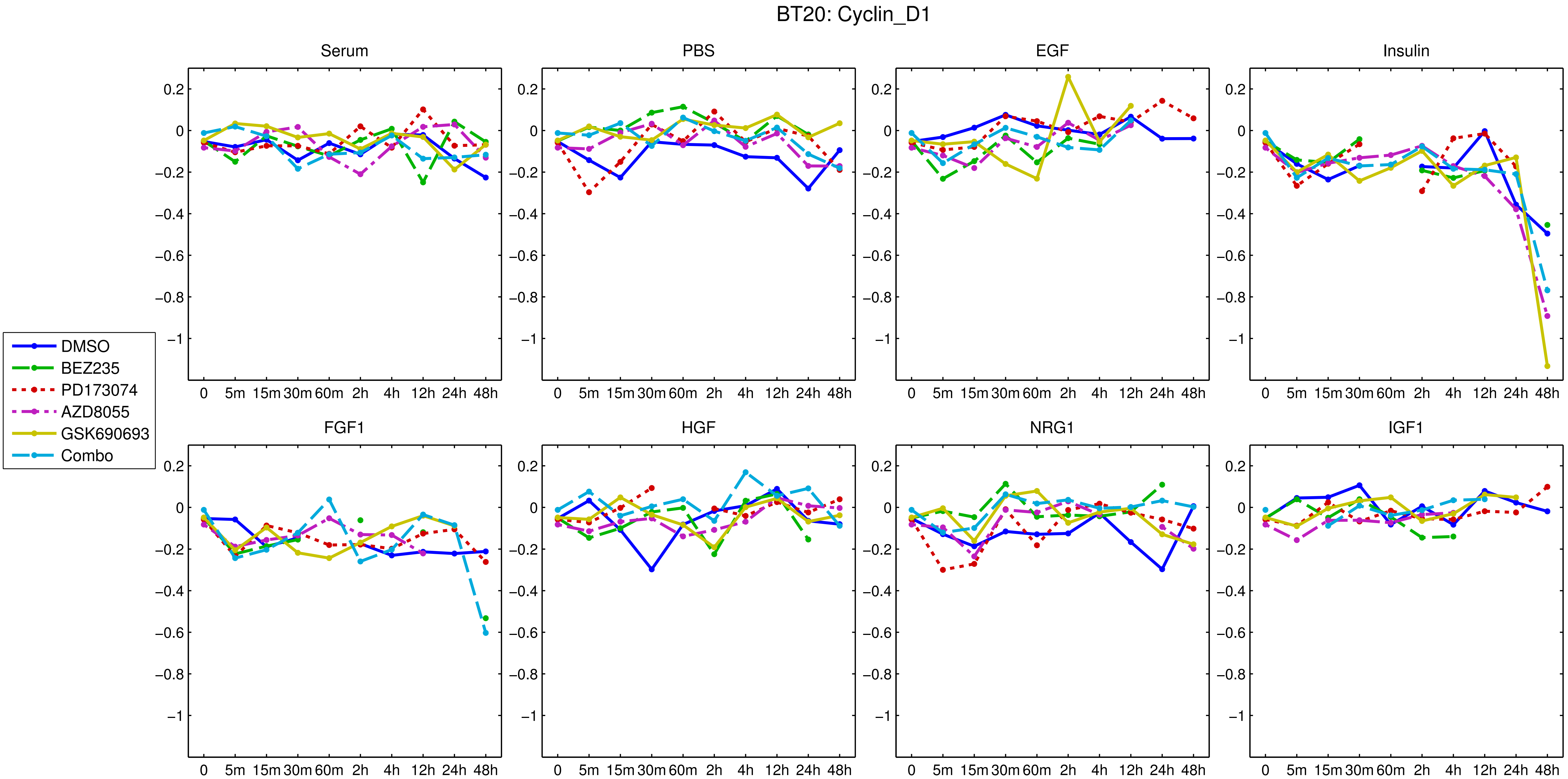
BT20: Cyclin_D1
Serum
PBS
EGF
Insulin
FGF1
HGF
NRG1
IGF1
DMSO
BEZ235
PD173074
AZD8055
GSK690693
Combo
0.2
0
−0.2
−0.4
−0.6
−0.8
−1
0
5m
15m
30m
60m
2h
4h
12h
24h
48h

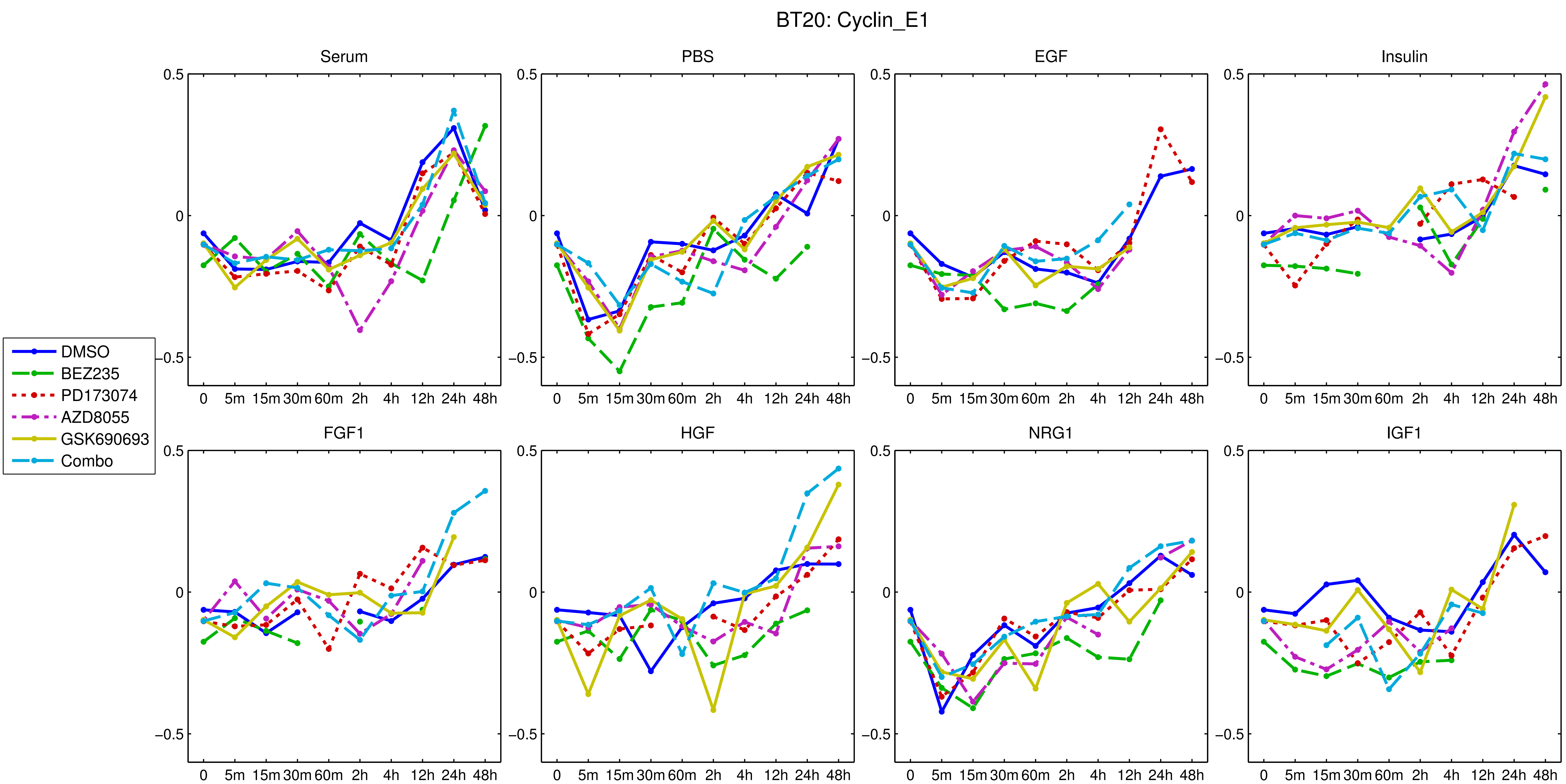
BT20: Cyclin_E1
Serum
PBS
EGF
Insulin
FGF1
HGF
NRG1
IGF1
0.5
0
−0.5
0
5m
15m
30m
60m
2h
4h
12h
24h
48h
DMSO
BEZ235
PD173074
AZD8055
GSK690693
Combo

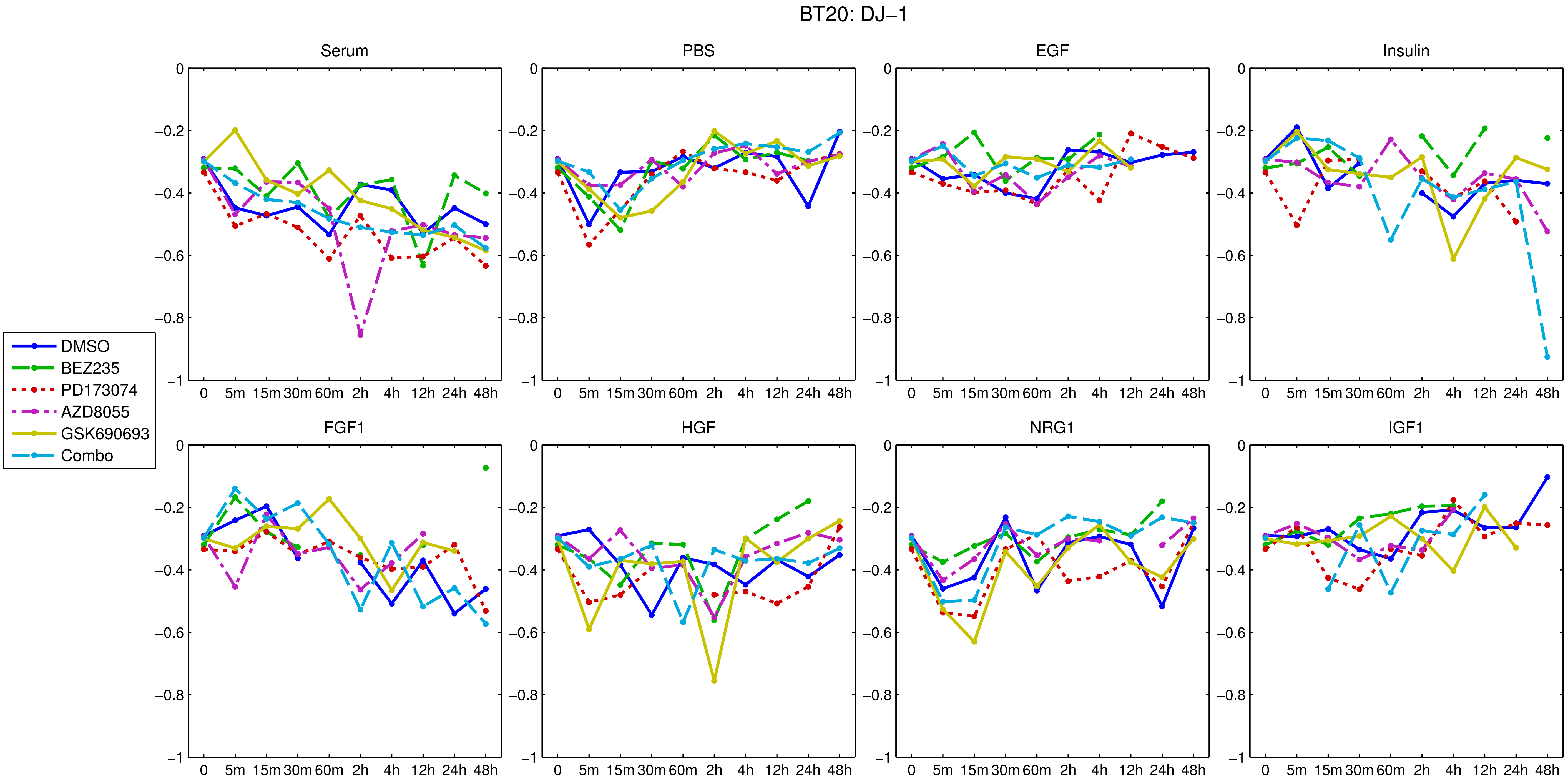
BT20: DJ−1
Serum
PBS
EGF
Insulin
FGF1
HGF
NRG1
IGF1
0
−0.2
−0.4
−0.6
−0.8
−1
0 5m 15m 30m 60m 2h 4h 12h 24h 48h
DMSO
BEZ235
PD173074
AZD8055
GSK690693
Combo

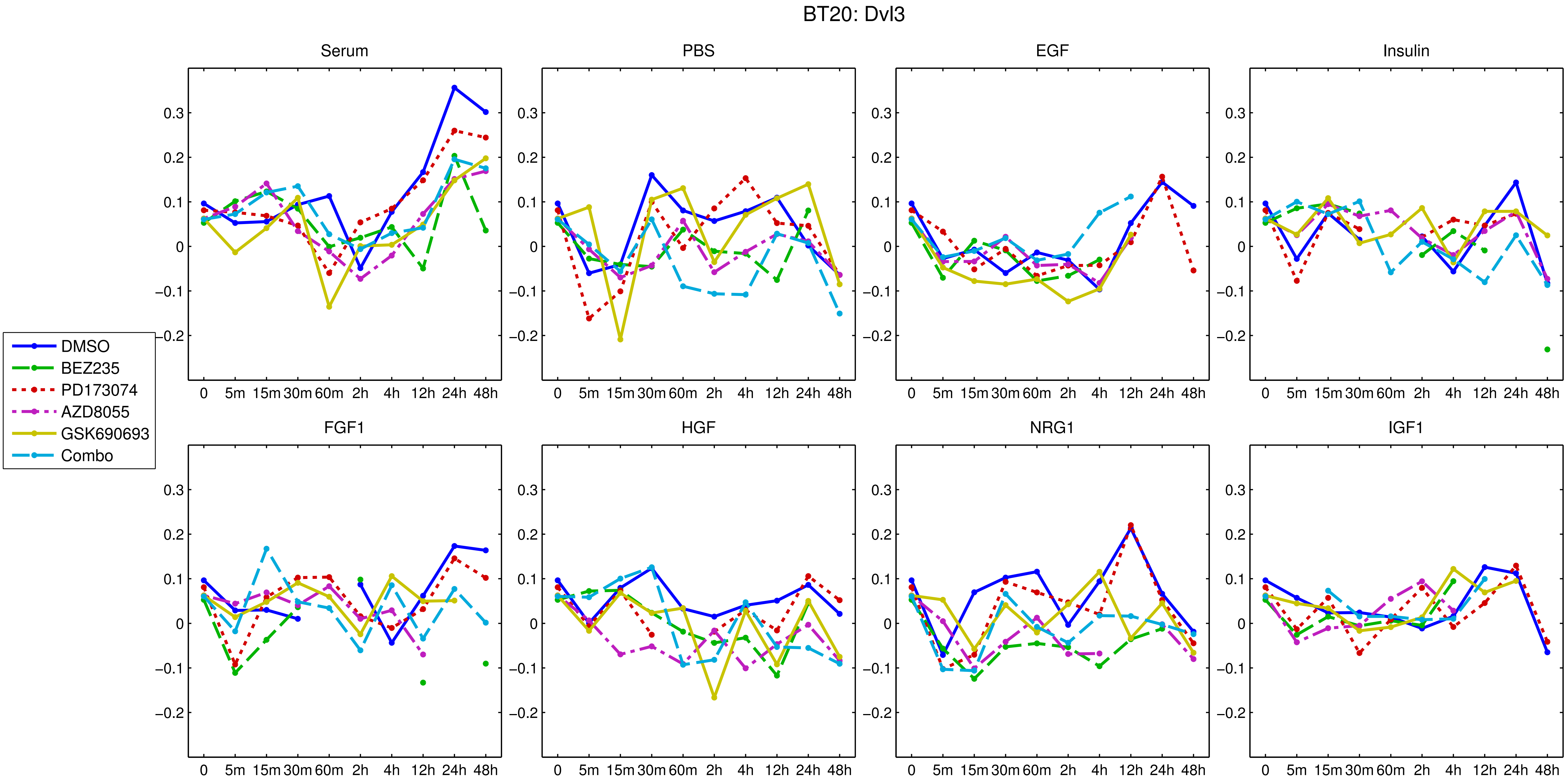

BT20: Dvl3
Serum
PBS
EGF
Insulin
FGF1
HGF
NRG1
IGF1
DMSO
BEZ235
PD173074
AZD8055
GSK690693
Combo
0.3
0.2
0.1
0
−0.1
−0.2
0
5m
15m
30m
60m
2h
4h
12h
24h
48h

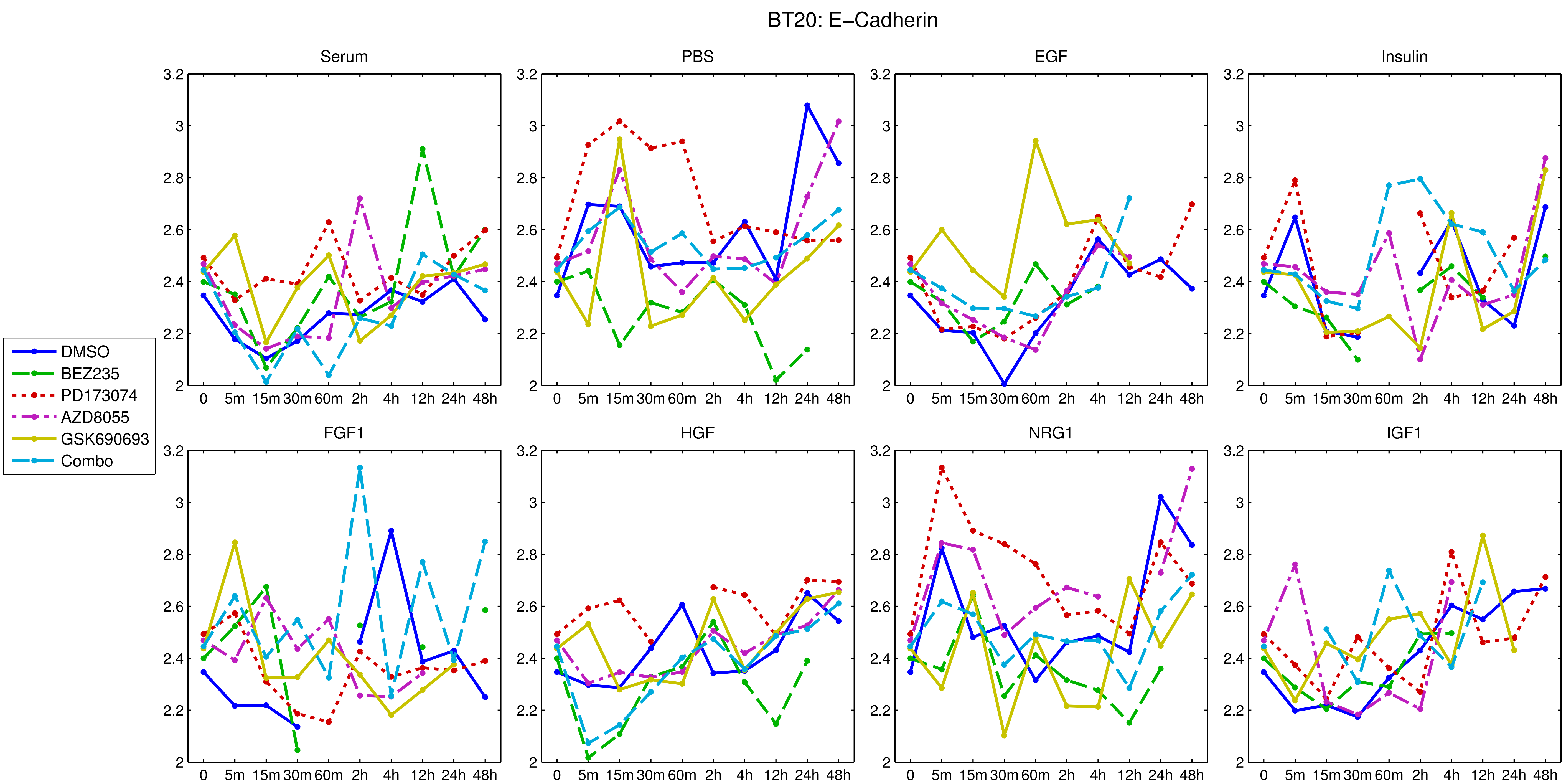
BT20: E−Cadherin
Serum
PBS
EGF
Insulin
FGF1
HGF
NRG1
IGF1
DMSO
BEZ235
PD173074
AZD8055
GSK690693
Combo
0
5m
15m
30m
60m
2h
4h
12h
24h
48h
2
2.2
2.4
2.6
2.8
3
3.2

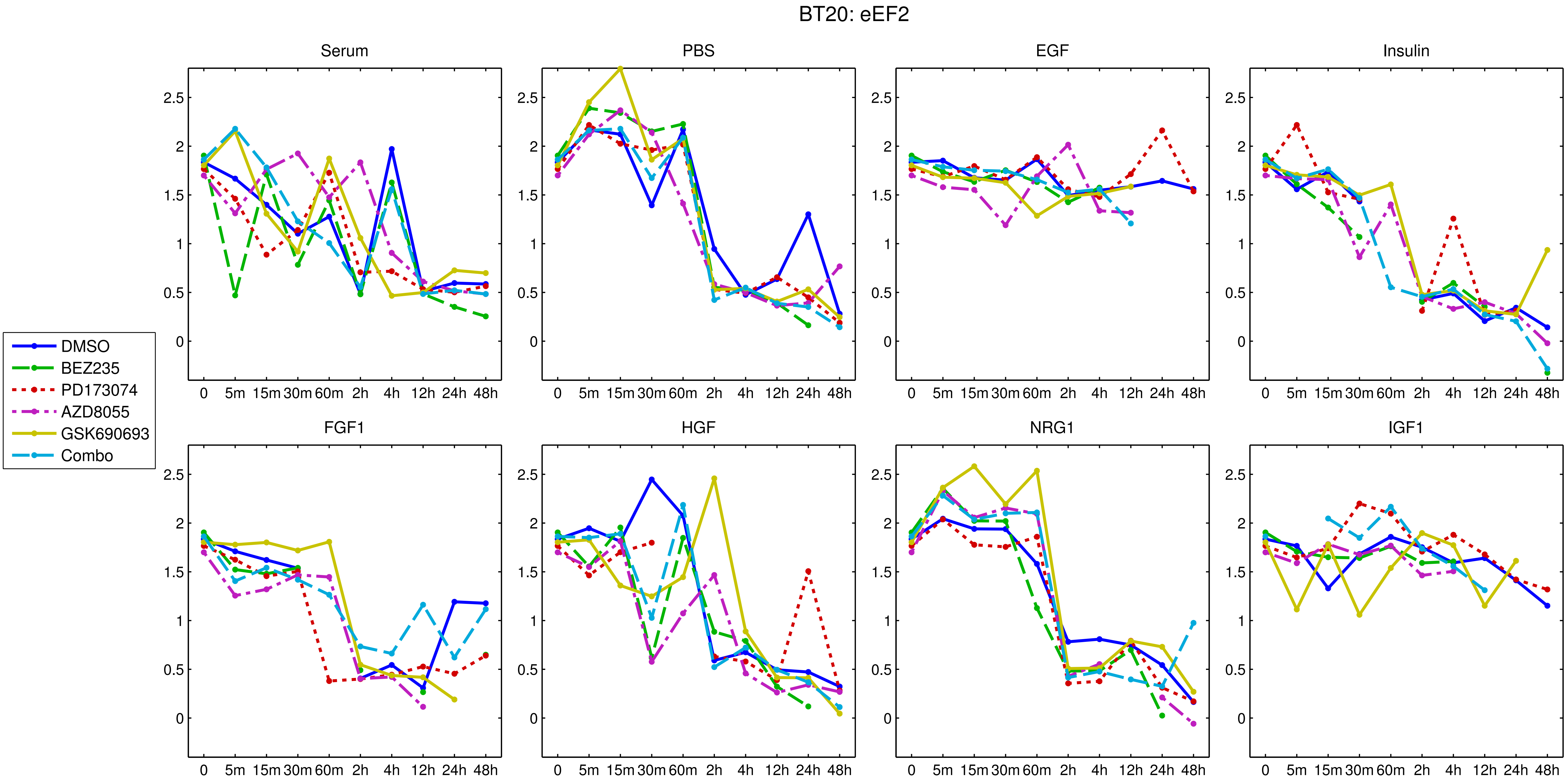
BT20: eEF2
Serum
PBS
EGF
Insulin
FGF1
HGF
NRG1
IGF1
DMSO
BEZ235
PD173074
AZD8055
GSK690693
Combo
0
0.5
1
1.5
2
2.5
0 5m 15m 30m 60m 2h 4h 12h 24h 48h

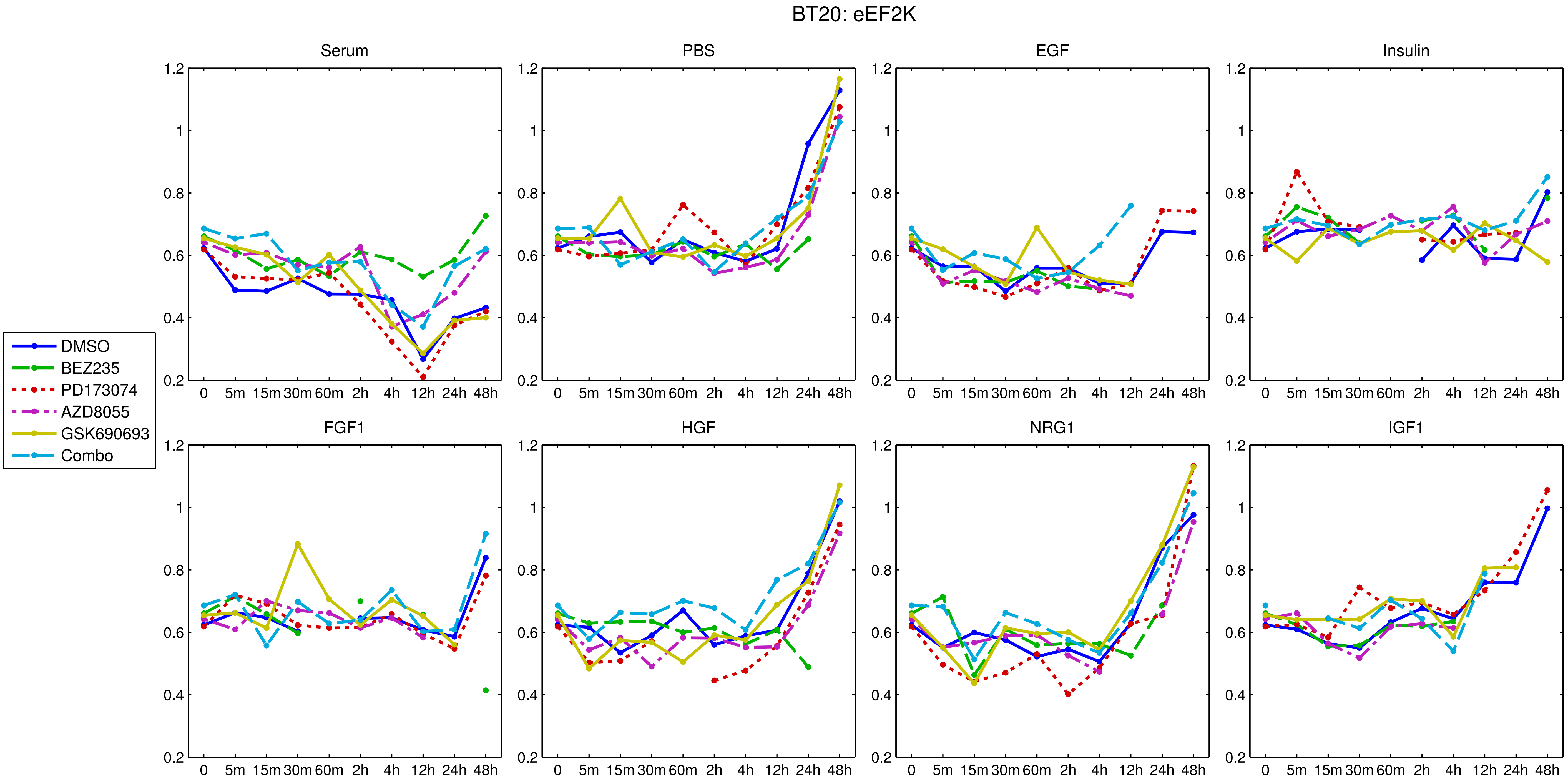

BT20: eEF2K
Serum
PBS
EGF
Insulin
FGF1
HGF
NRG1
IGF1
DMSO
BEZ235
PD173074
AZD8055
GSK690693
Combo
0.2
0.4
0.6
0.8
1
1.2
0
5m
15m
30m
60m
2h
4h
12h
24h
48h

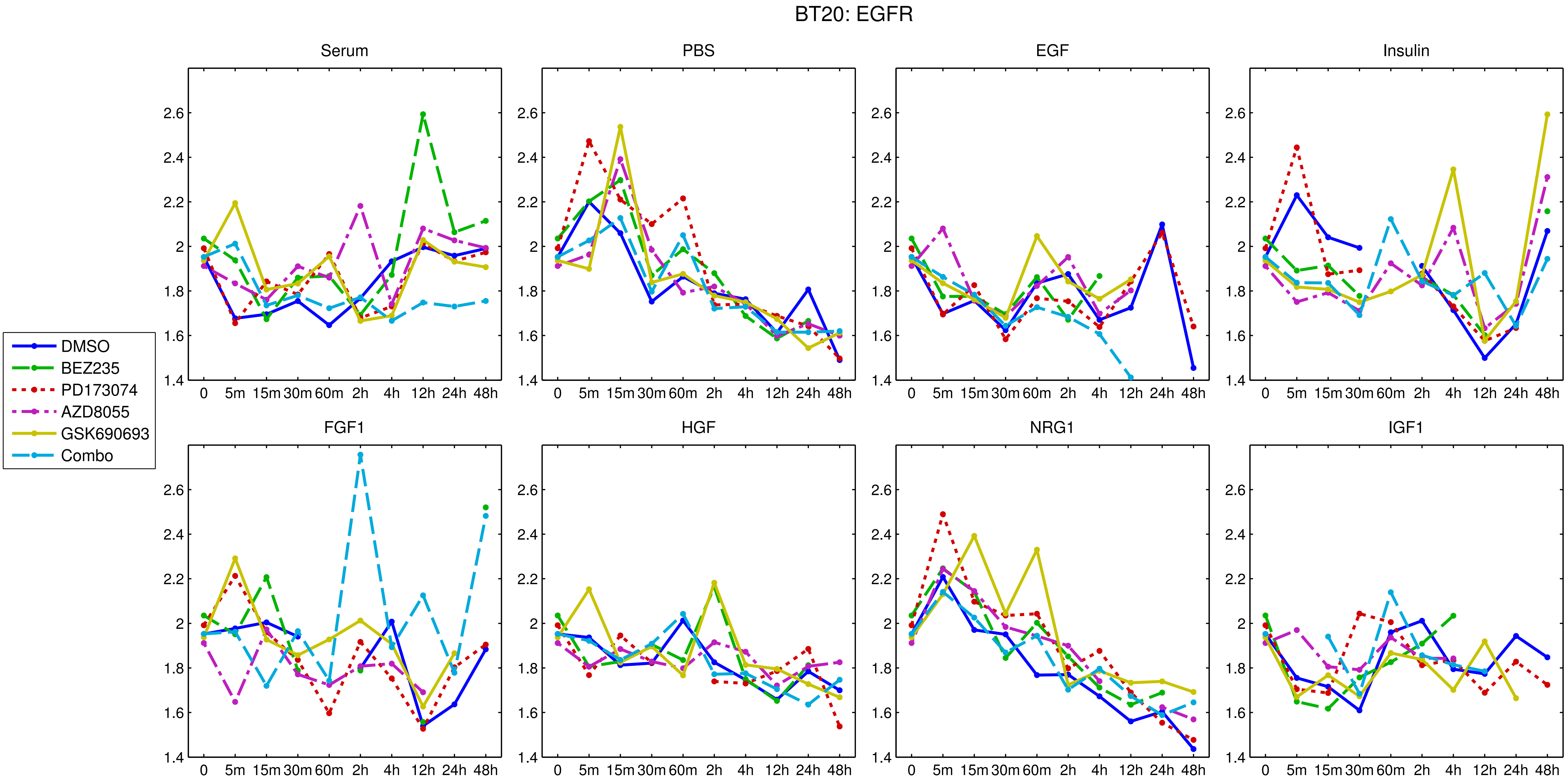

BT20: EGFR
Serum
PBS
EGF
Insulin
FGF1
HGF
NRG1
IGF1
DMSO
BEZ235
PD173074
AZD8055
GSK690693
Combo
2.6
2.4
2.2
2
1.8
1.6
1.4
0
5m
15m
30m
60m
2h
4h
12h
24h
48h

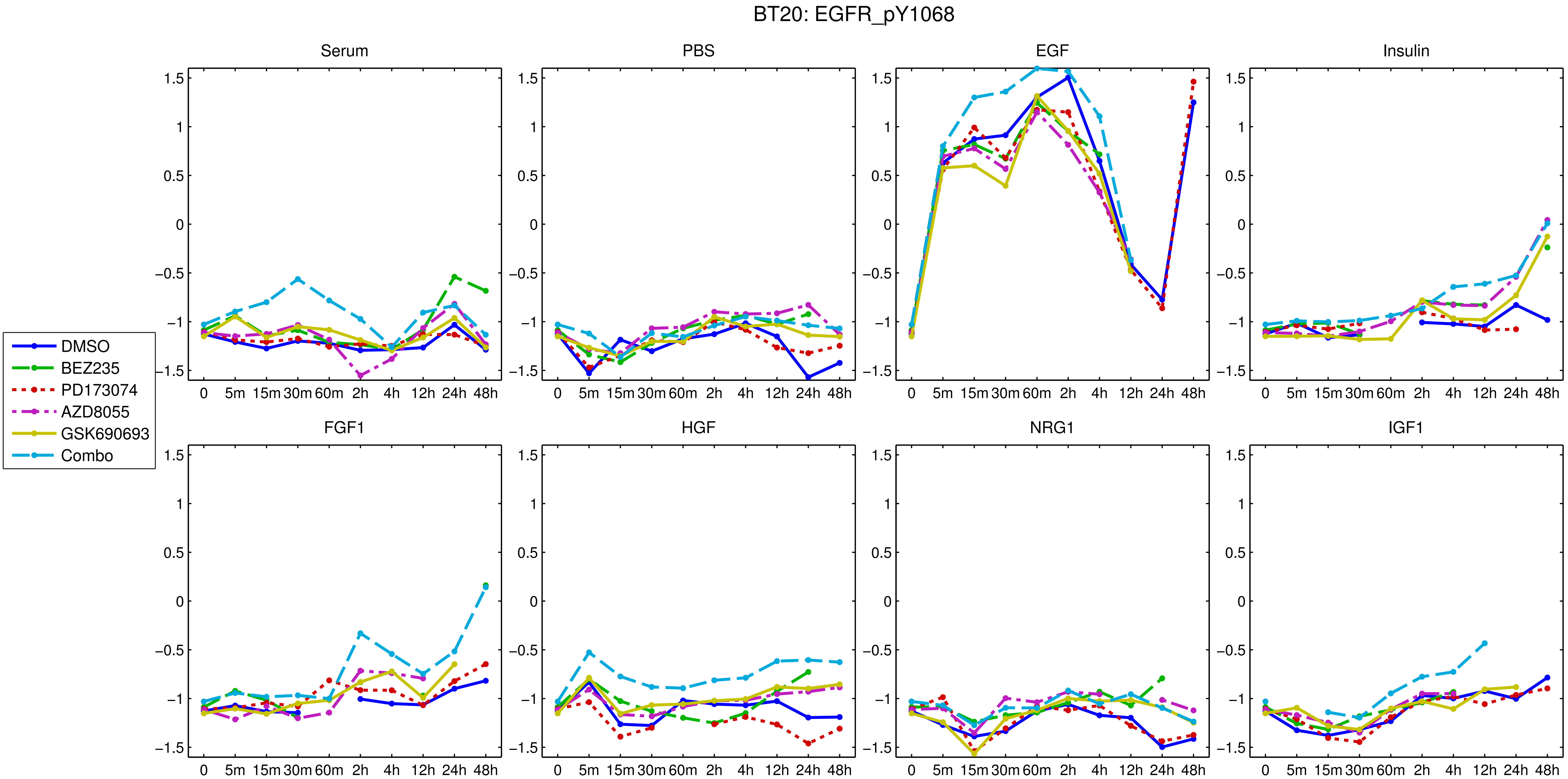

BT20: EGFR_pY1068
Serum
PBS
EGF
Insulin
FGF1
HGF
NRG1
IGF1
DMSO
BEZ235
PD173074
AZD8055
GSK690693
Combo
1.5
1
0.5
0
−0.5
−1
−1.5
0 5m 15m 30m 60m 2h 4h 12h 24h 48h

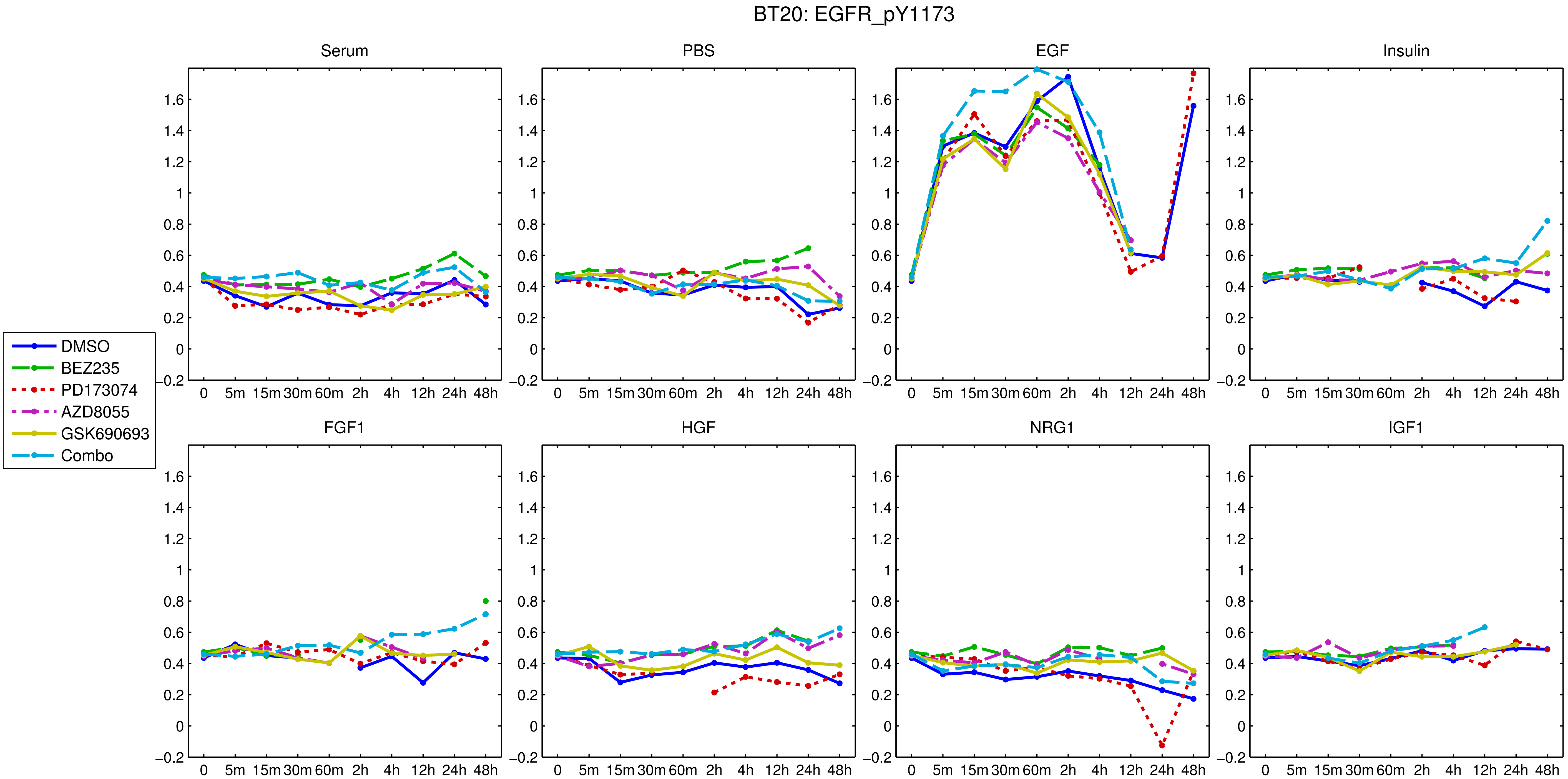
BT20: EGFR_pY1173
Serum
PBS
EGF
Insulin
FGF1
HGF
NRG1
IGF1
DMSO
BEZ235
PD173074
AZD8055
GSK690693
Combo
1.6
1.4
1.2
1
0.8
0.6
0.4
0.2
0
−0.2
0
5m
15m
30m
60m
2h
4h
12h
24h
48h

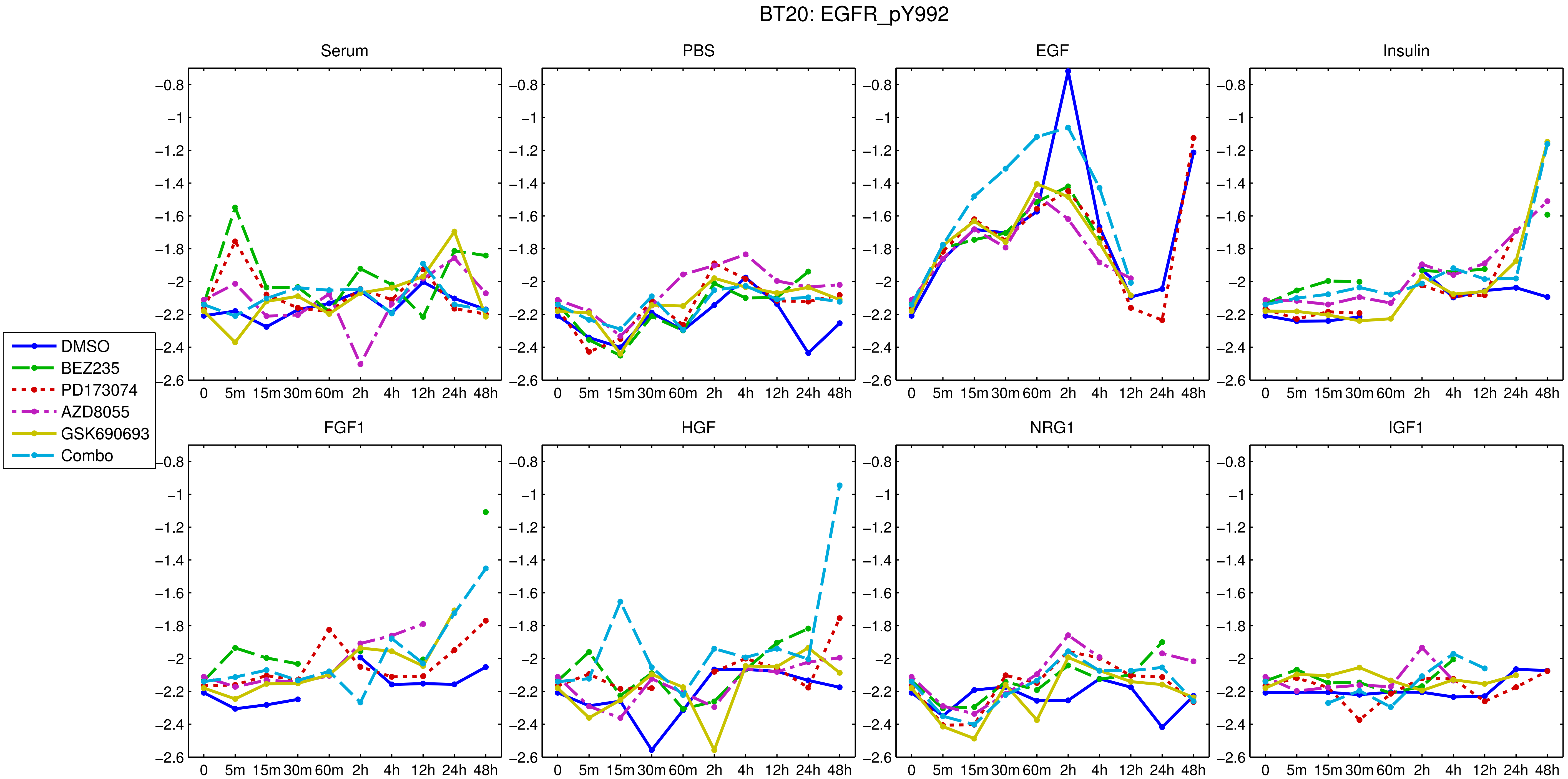
BT20: EGFR_pY992
Serum
PBS
EGF
Insulin
FGF1
HGF
NRG1
IGF1
DMSO
BEZ235
PD173074
AZD8055
GSK690693
Combo
−0.8
−1
−1.2
−1.4
−1.6
−1.8
−2
−2.2
−2.4
−2.6
0
5m
15m
30m
60m
2h
4h
12h
24h
48h

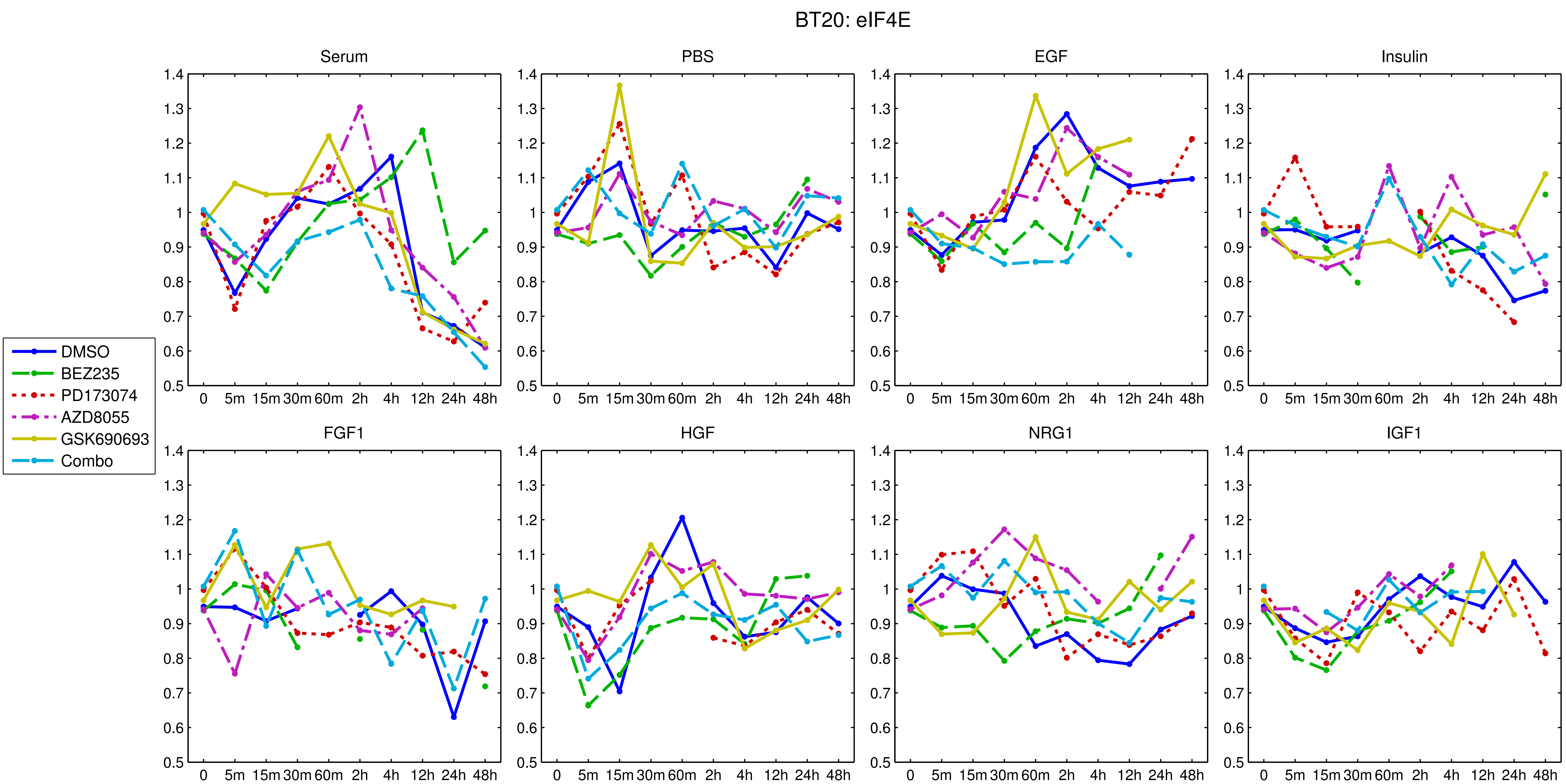

BT20: eIF4E
Serum
PBS
EGF
Insulin
FGF1
HGF
NRG1
IGF1
DMSO
BEZ235
PD173074
AZD8055
GSK690693
Combo
0
5m
15m
30m
60m
2h
4h
12h
24h
48h
0.5
0.6
0.7
0.8
0.9
1
1.1
1.2
1.3
1.4

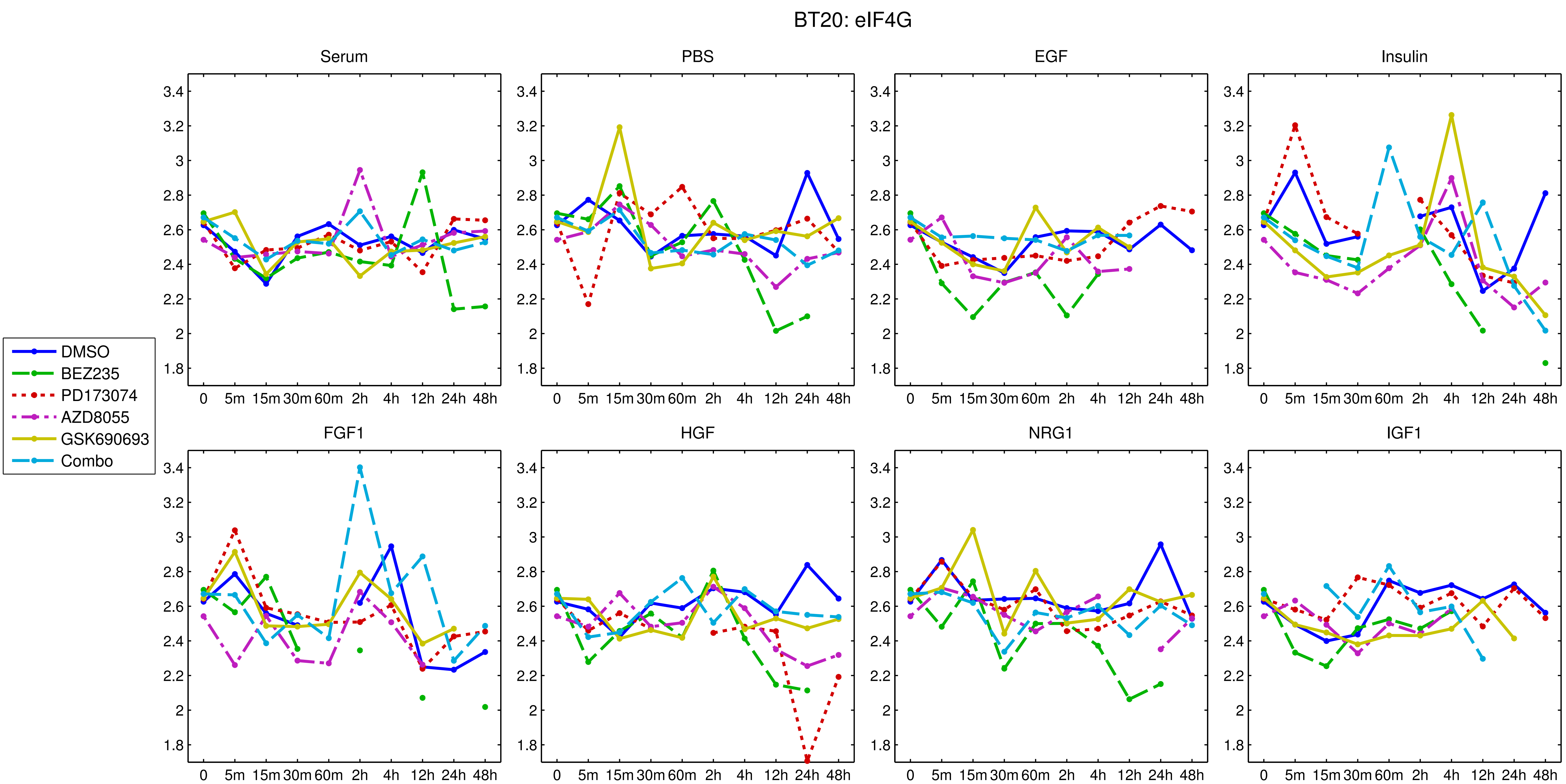

BT20: eIF4G
Serum
PBS
EGF
Insulin
FGF1
HGF
NRG1
IGF1
DMSO
BEZ235
PD173074
AZD8055
GSK690693
Combo
3.4
3.2
3
2.8
2.6
2.4
2.2
2
1.8
0
5m
15m
30m
60m
2h
4h
12h
24h
48h

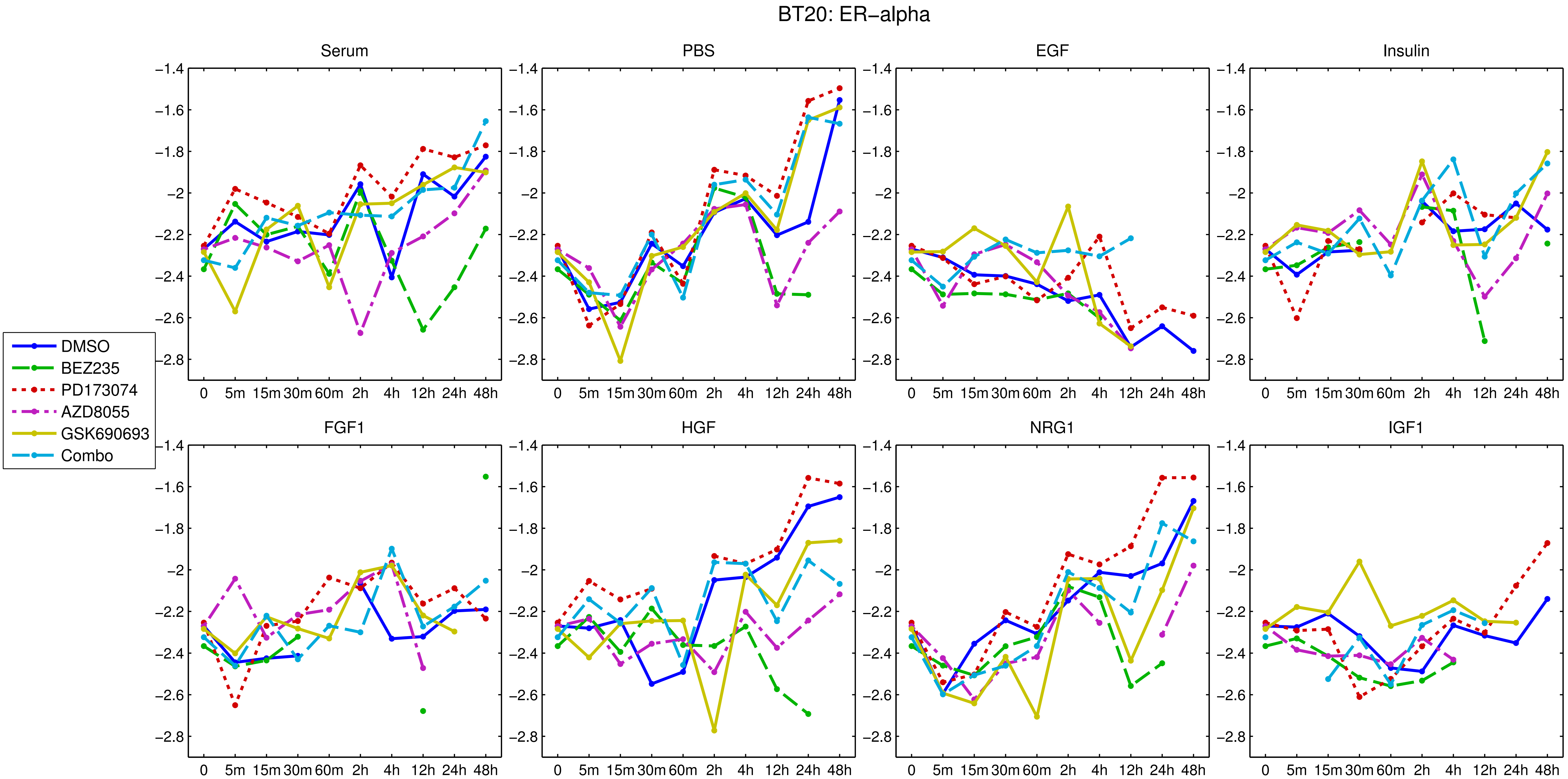
BT20: ER−alpha
Serum
PBS
EGF
Insulin
FGF1
HGF
NRG1
IGF1
DMSO
BEZ235
PD173074
AZD8055
GSK690693
Combo
−1.4
−1.6
−1.8
−2
−2.2
−2.4
−2.6
−2.8
0
5m
15m
30m
60m
2h
4h
12h
24h
48h

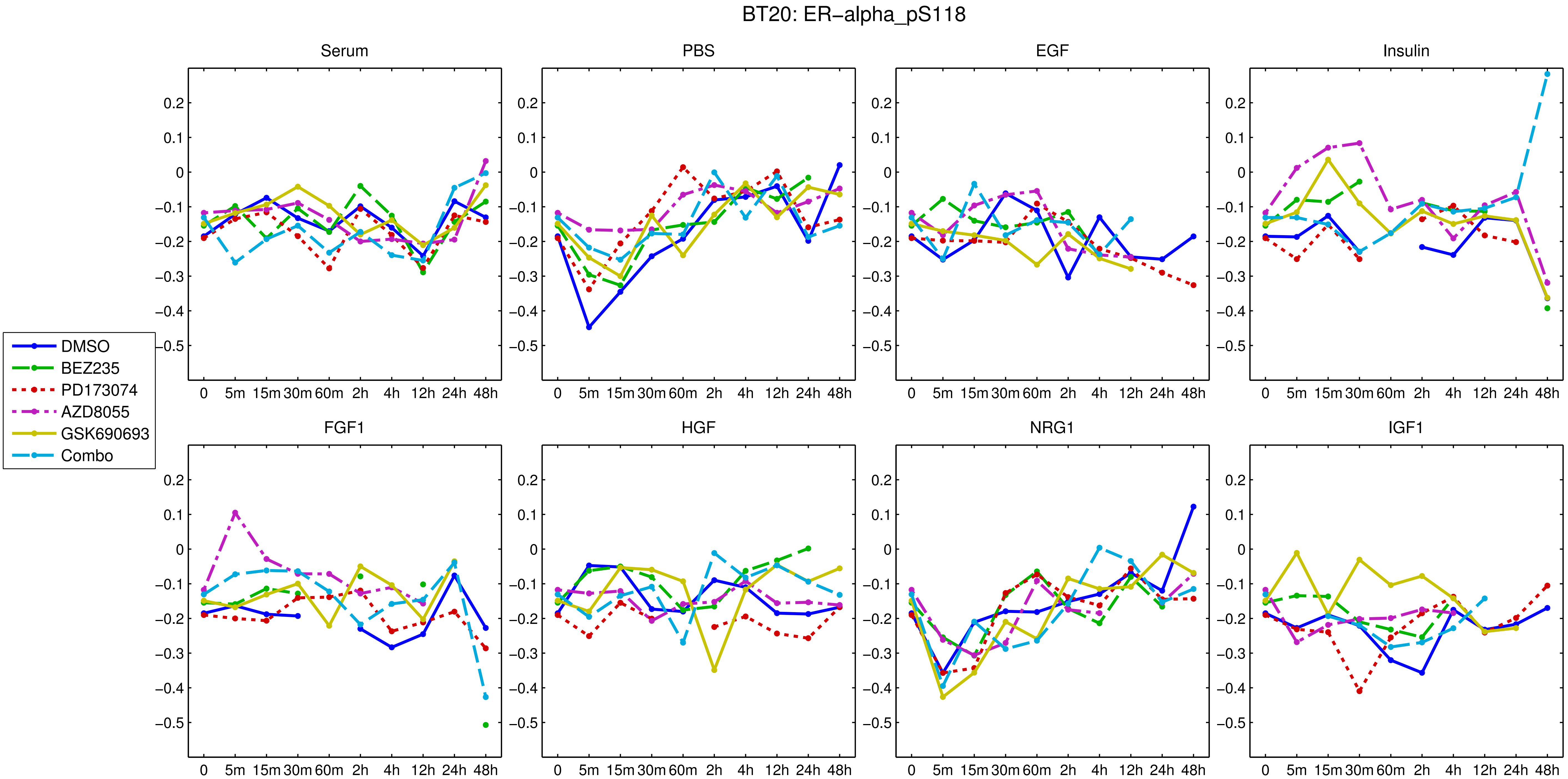

BT20: ER−alpha_pS118
Serum
PBS
EGF
Insulin
FGF1
HGF
NRG1
IGF1
DMSO
BEZ235
PD173074
AZD8055
GSK690693
Combo
0.2
0.1
0
−0.1
−0.2
−0.3
−0.4
−0.5
0 5m 15m 30m 60m 2h 4h 12h 24h 48h

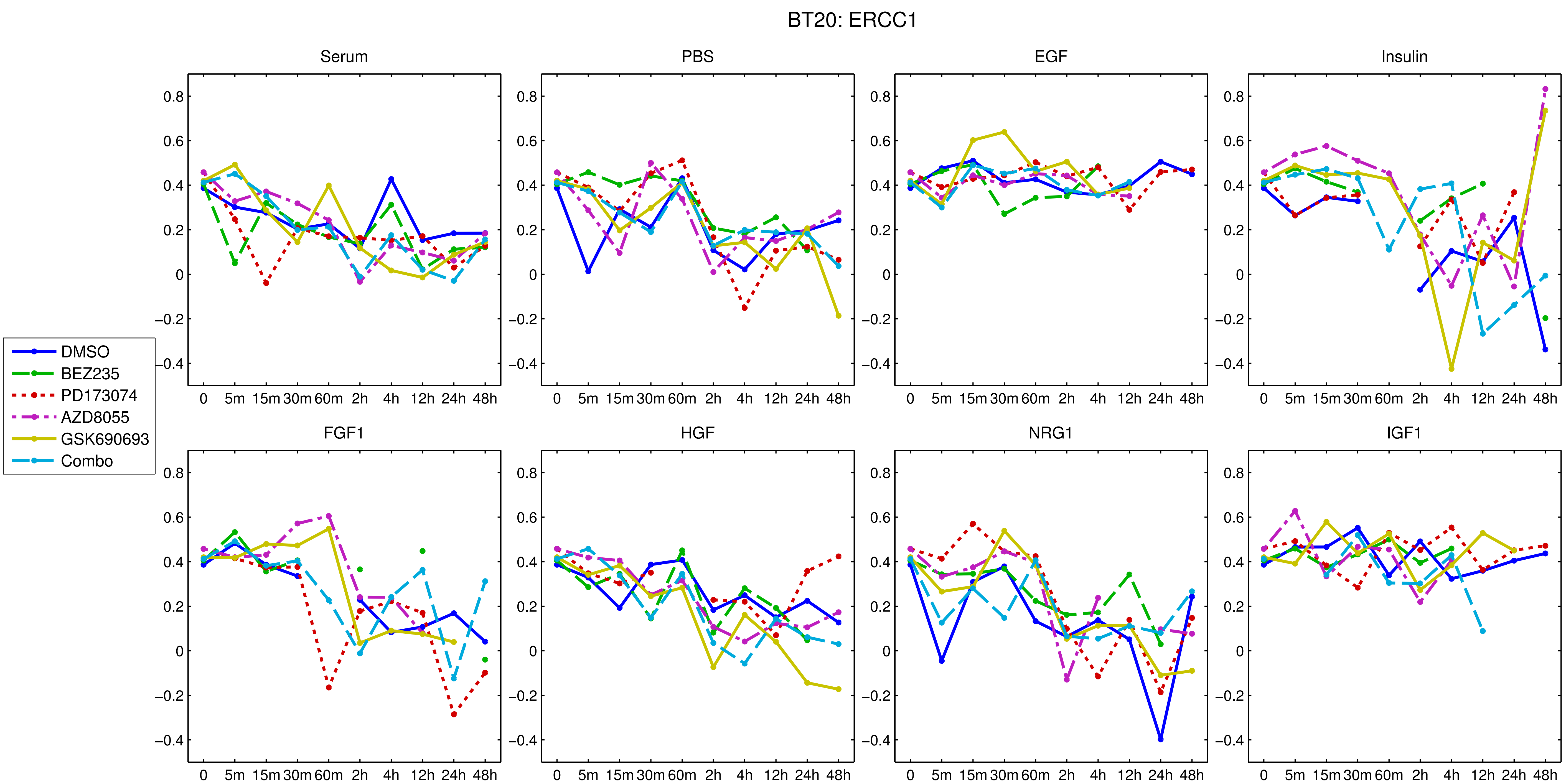

BT20: ERCC1
Serum
PBS
EGF
Insulin
FGF1
HGF
NRG1
IGF1
DMSO
BEZ235
PD173074
AZD8055
GSK690693
Combo
0.8
0.6
0.4
0.2
0
−0.2
−0.4
0
5m
15m
30m
60m
2h
4h
12h
24h
48h

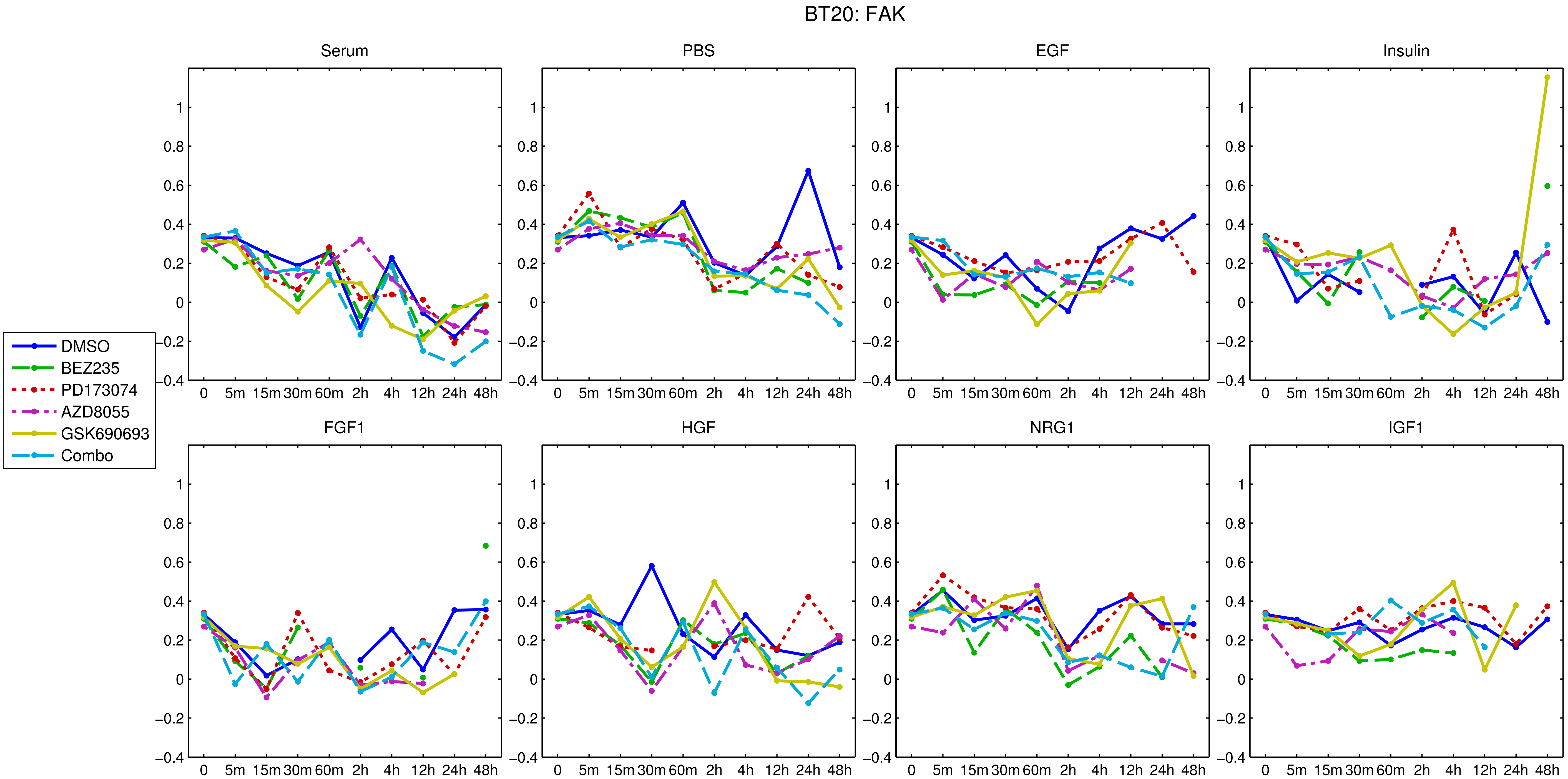
BT20: FAK
Serum
PBS
EGF
Insulin
FGF1
HGF
NRG1
IGF1
1
0.8
0.6
0.4
0.2
0
−0.2
−0.4
0 5m 15m 30m 60m 2h 4h 12h 24h 48h
DMSO
BEZ235
PD173074
AZD8055
GSK690693
Combo

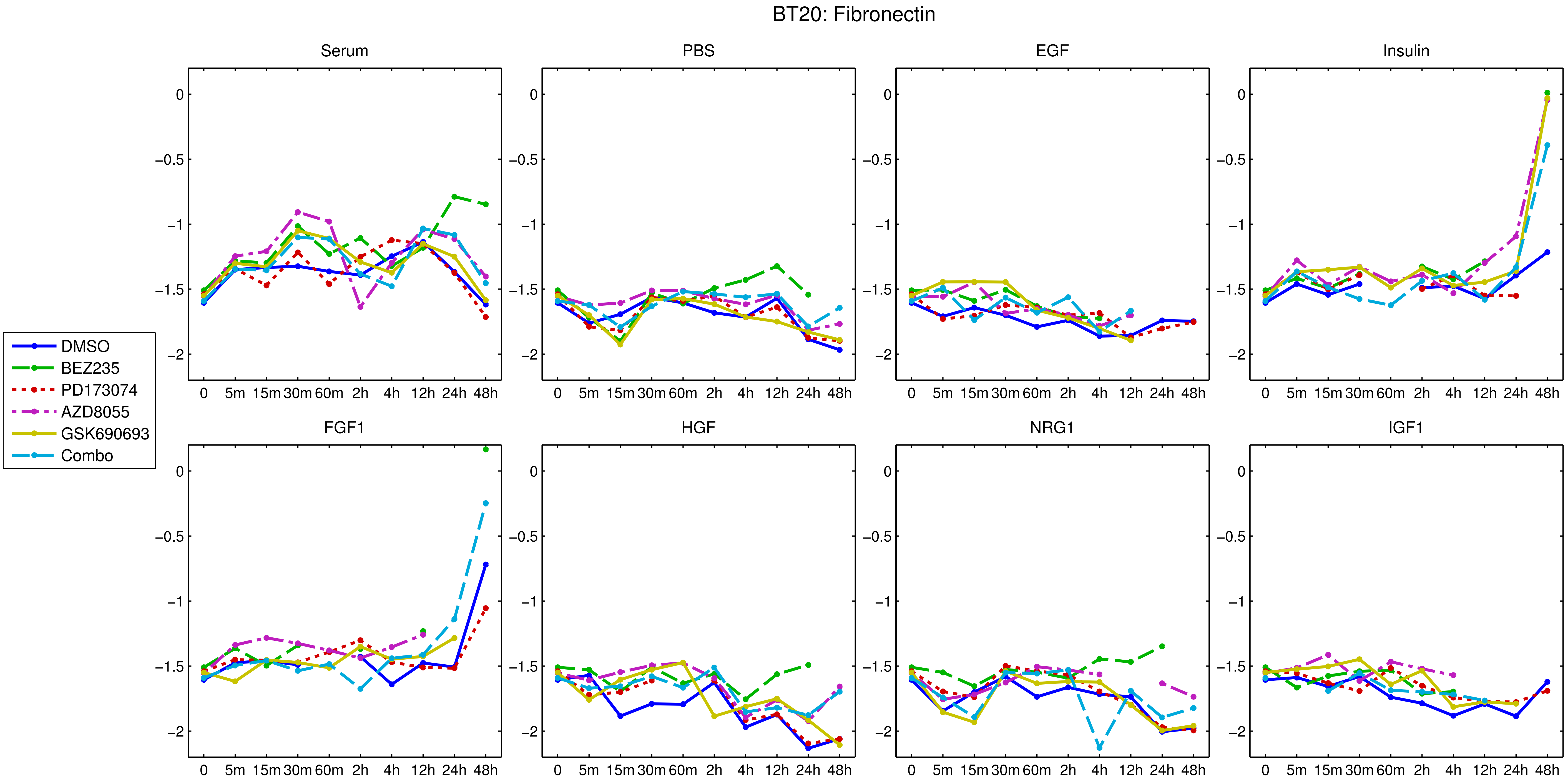
BT20: Fibronectin
Serum
PBS
EGF
Insulin
FGF1
HGF
NRG1
IGF1
DMSO
BEZ235
PD173074
AZD8055
GSK690693
Combo
0
−0.5
−1
−1.5
−2
0 5m 15m 30m 60m 2h 4h 12h 24h 48h

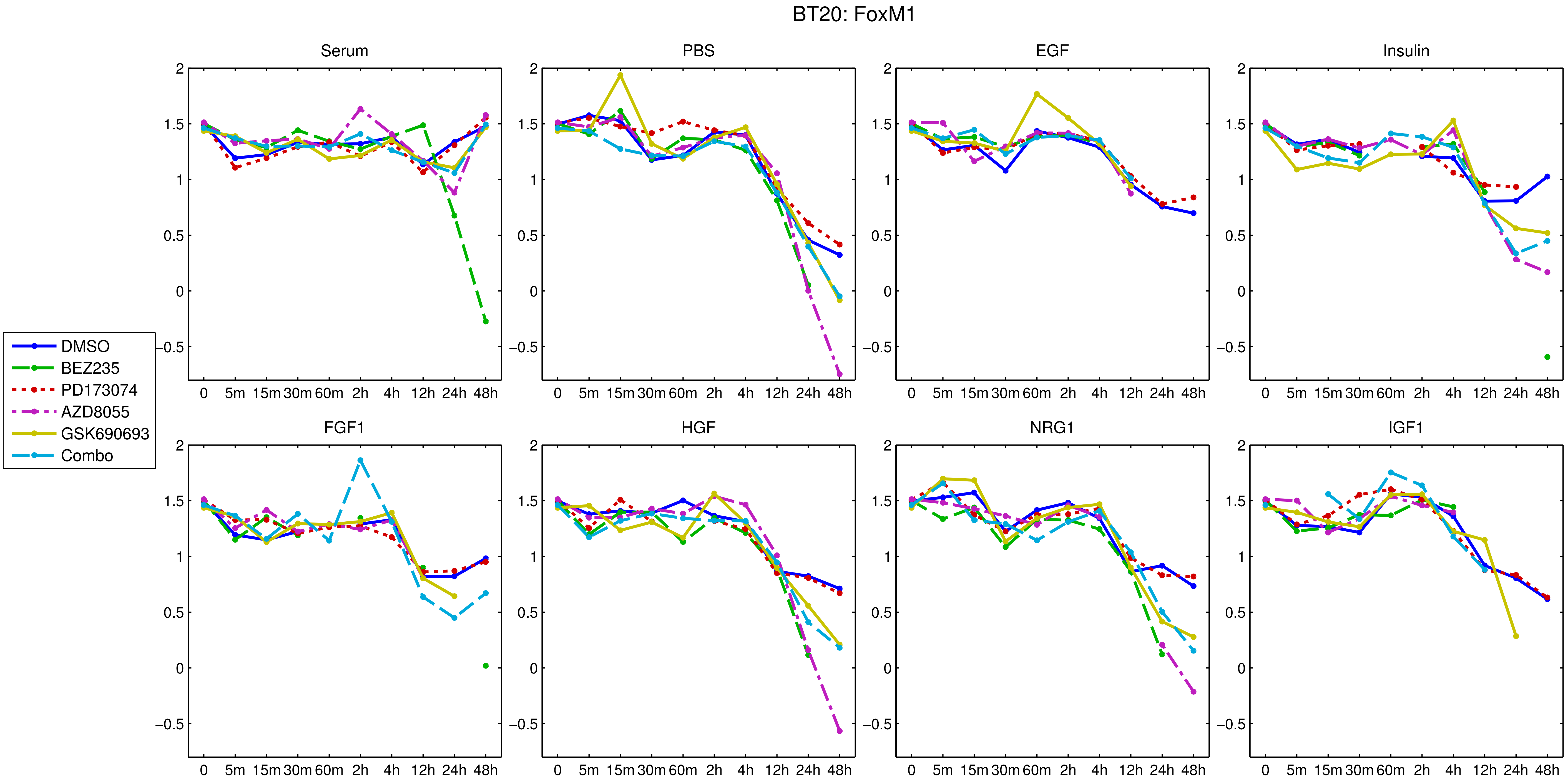
BT20: FoxM1
Serum
PBS
EGF
Insulin
FGF1
HGF
NRG1
IGF1
DMSO
BEZ235
PD173074
AZD8055
GSK690693
Combo
0
5m
15m
30m
60m
2h
4h
12h
24h
48h
2
1.5
1
0.5
0
−0.5

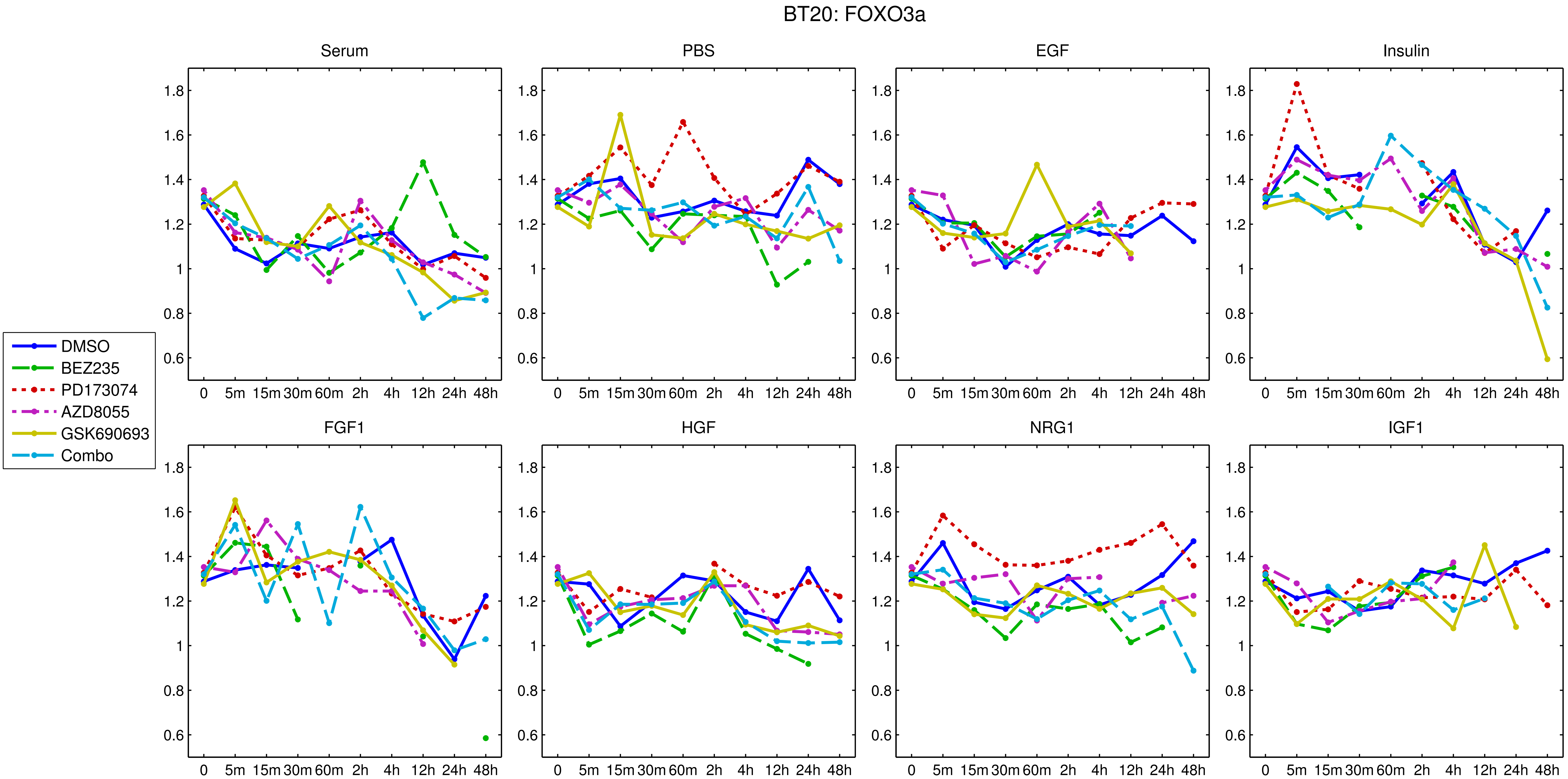

BT20: FOXO3a
Serum
PBS
EGF
Insulin
FGF1
HGF
NRG1
IGF1
DMSO
BEZ235
PD173074
AZD8055
GSK690693
Combo
1.8
1.6
1.4
1.2
1
0.8
0.6
0 5m 15m 30m 60m 2h 4h 12h 24h 48h

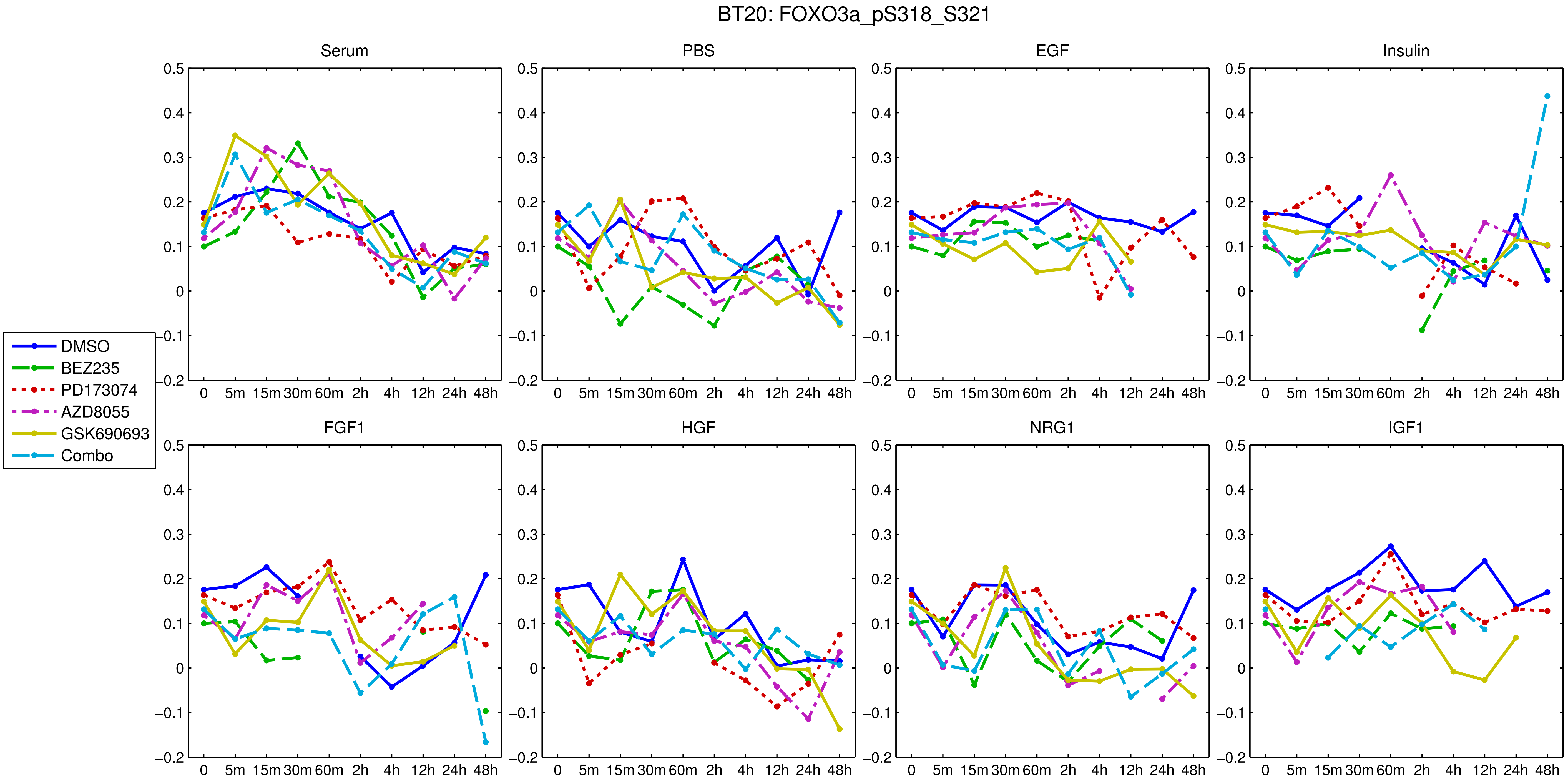
BT20: FOXO3a_pS318_S321
Serum
PBS
EGF
Insulin
FGF1
HGF
NRG1
IGF1
DMSO
BEZ235
PD173074
AZD8055
GSK690693
Combo
0
5m
15m
30m
60m
2h
4h
12h
24h
48h
0.5
0.4
0.3
0.2
0.1
−0.1
−0.2

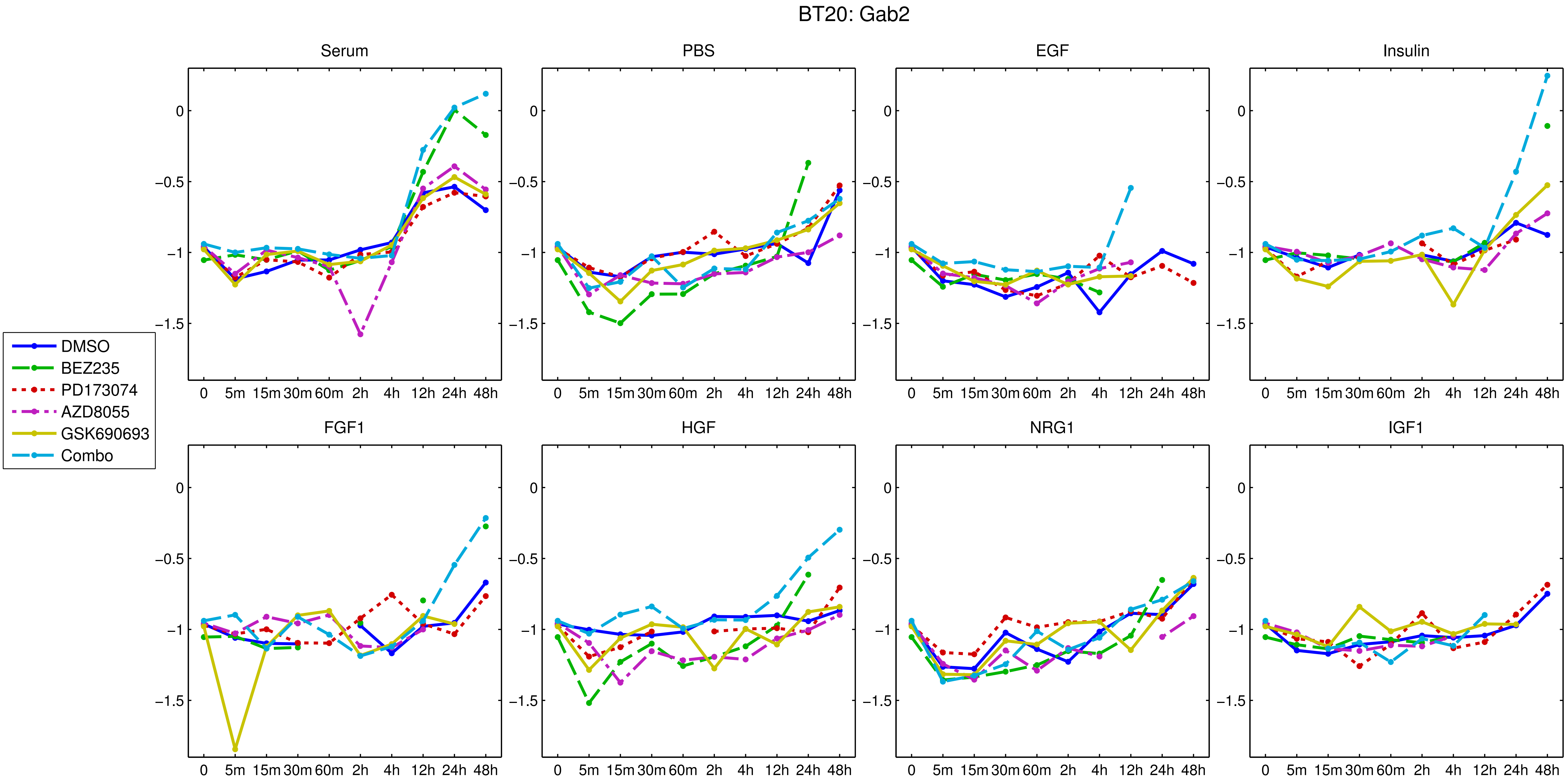
BT20: Gab2
Serum
PBS
EGF
Insulin
FGF1
HGF
NRG1
IGF1
DMSO
BEZ235
PD173074
AZD8055
GSK690693
Combo
0
−0.5
−1
−1.5
0
5m
15m
30m
60m
2h
4h
12h
24h
48h

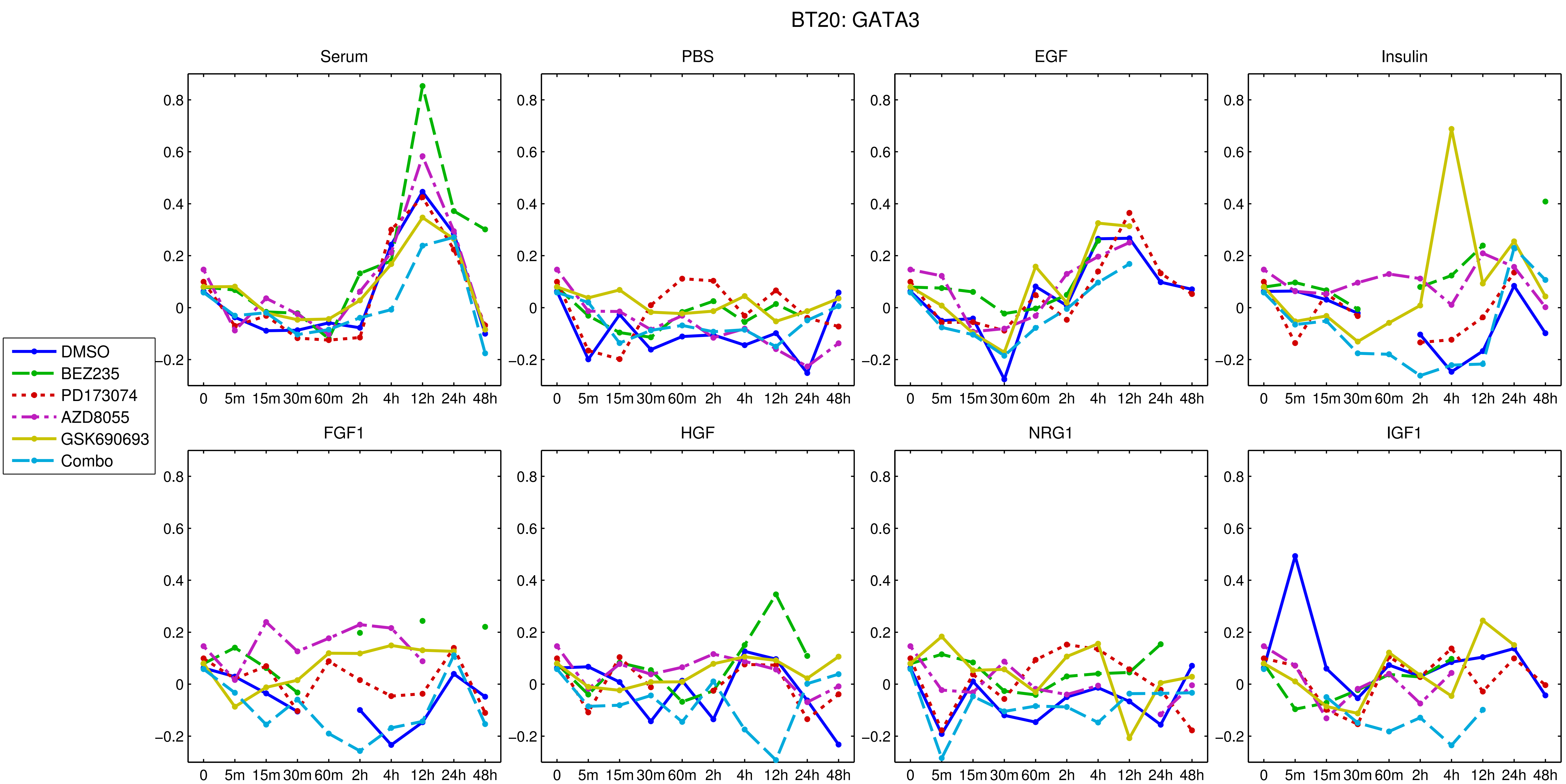

BT20: GATA3
Serum
PBS
EGF
Insulin
FGF1
HGF
NRG1
IGF1
DMSO
BEZ235
PD173074
AZD8055
GSK690693
Combo
0.8
0.6
0.4
0.2
0
−0.2
0
5m
15m
30m
60m
2h
4h
12h
24h
48h

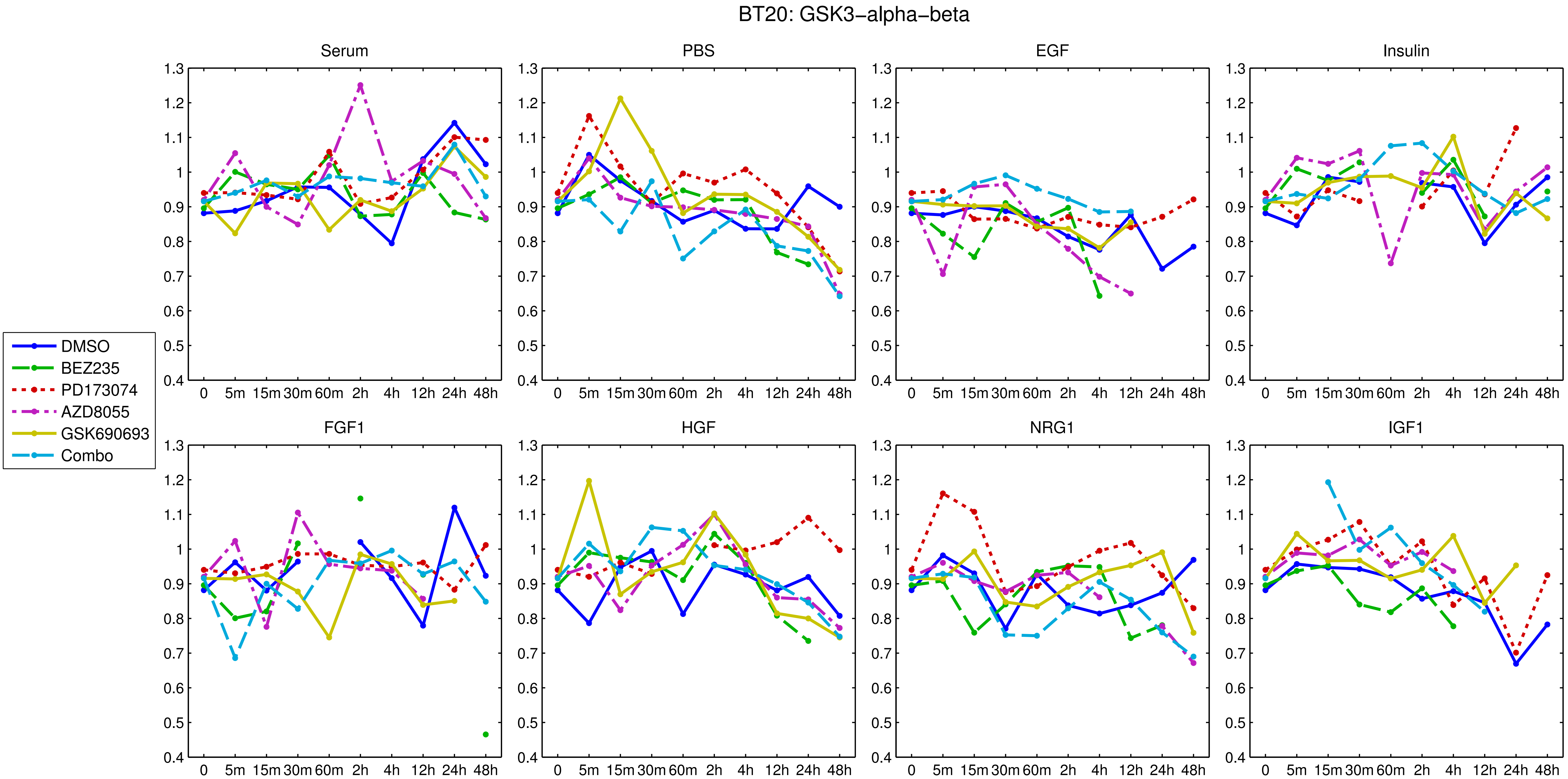
BT20: GSK3-alpha-beta
Serum
PBS
EGF
Insulin
FGF1
HGF
NRG1
IGF1
DMSO
BEZ235
PD173074
AZD8055
GSK690693
Combo
1.3
1.2
1.1
1
0.9
0.8
0.7
0.6
0.5
0.4
0
5m
15m
30m
60m
2h
4h
12h
24h
48h

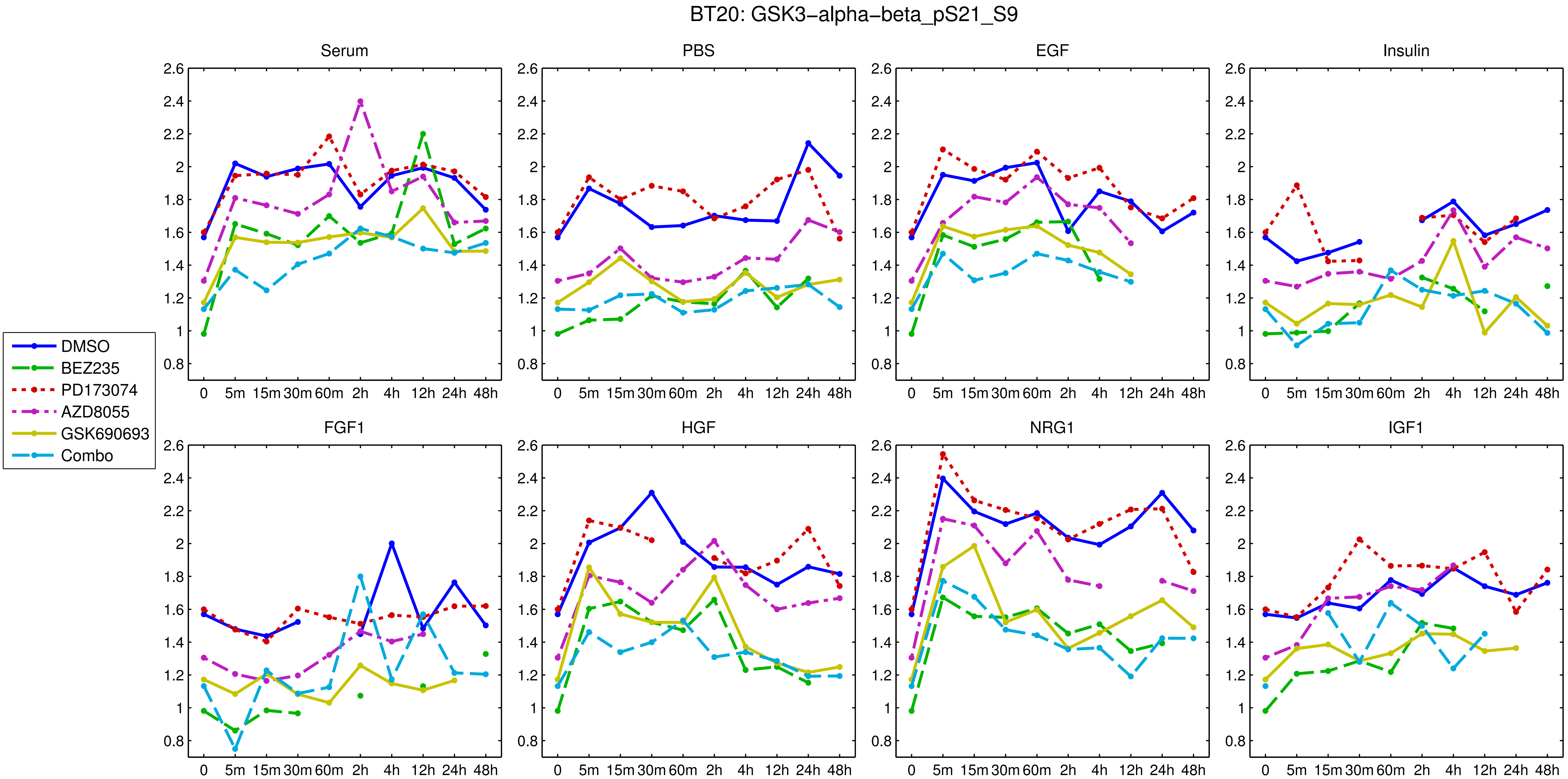
BT20: GSK3−alpha−beta_pS21_S9
Serum
PBS
EGF
Insulin
FGF1
HGF
NRG1
IGF1
2.6
2.4
2.2
2
1.8
1.6
1.4
1.2
1
0.8
0
5m
15m
30m
60m
2h
4h
12h
24h
48h
DMSO
BEZ235
PD173074
AZD8055
GSK690693
Combo

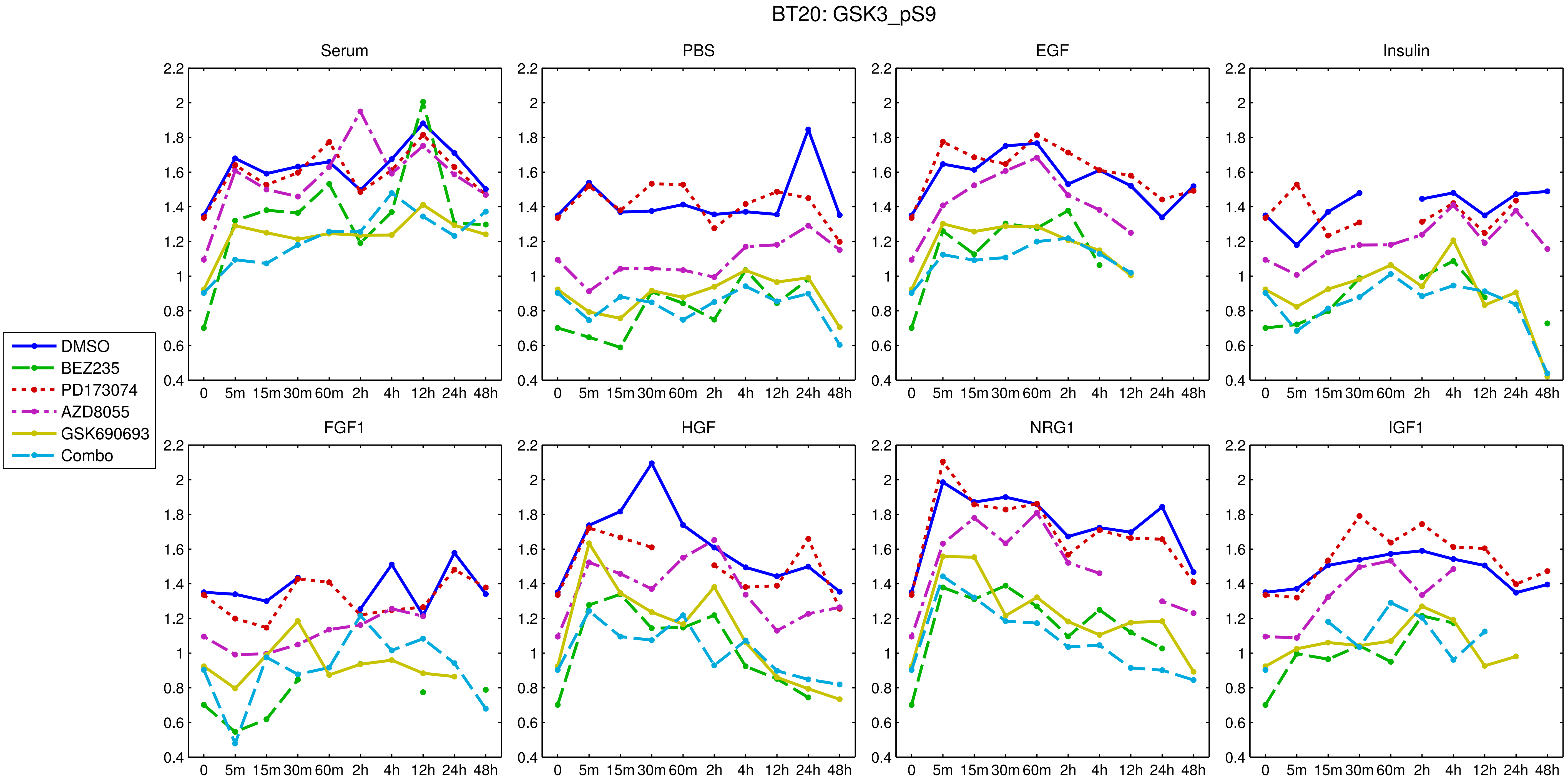

BT20: GSK3_pS9
Serum
PBS
EGF
Insulin
FGF1
HGF
NRG1
IGF1
DMSO
BEZ235
PD173074
AZD8055
GSK690693
Combo
0.4
0.6
0.8
1
1.2
1.4
1.6
1.8
2
2.2
0
5m
15m
30m
60m
2h
4h
12h
24h
48h

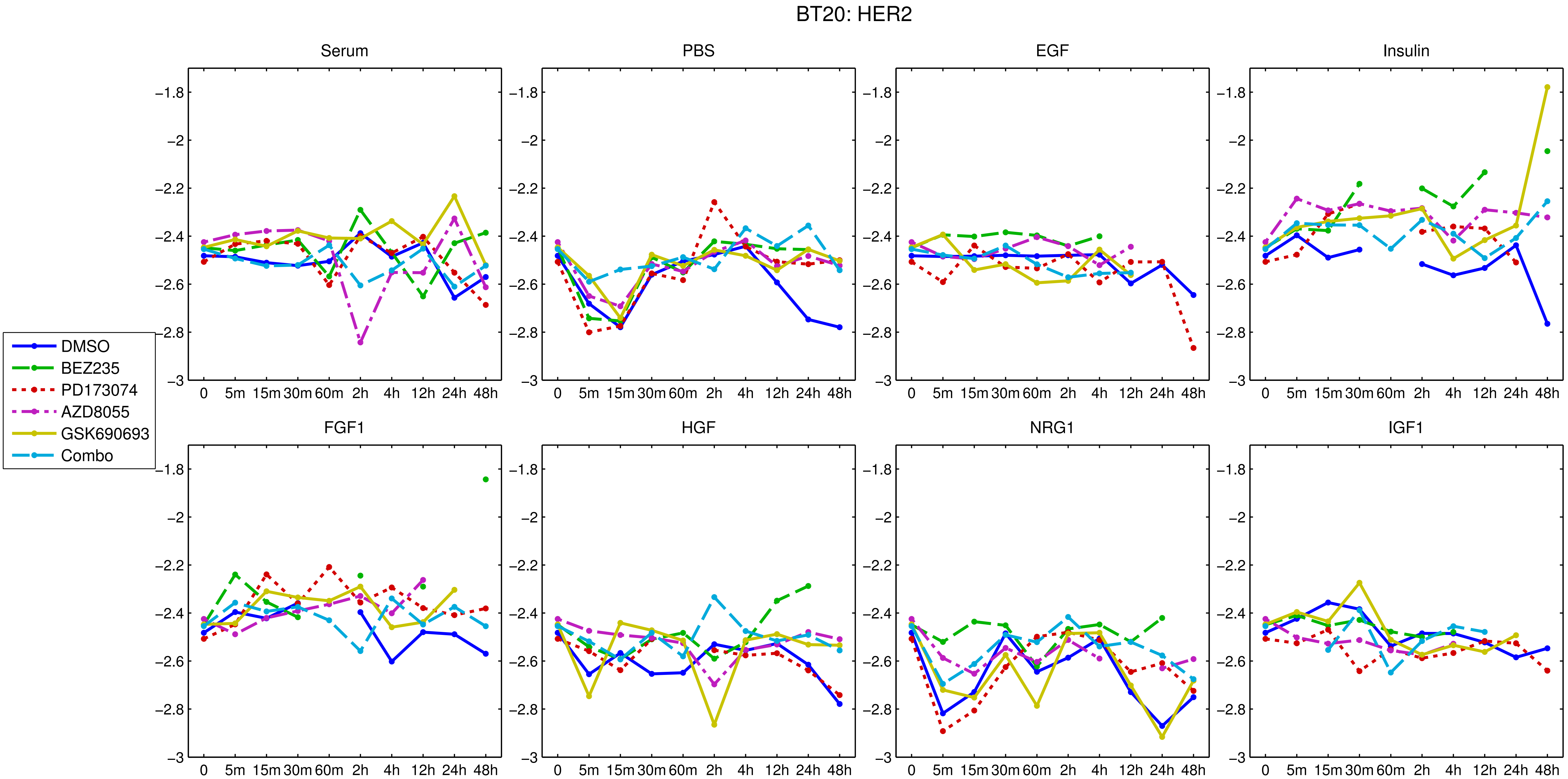
BT20: HER2
Serum
PBS
EGF
Insulin
FGF1
HGF
NRG1
IGF1
DMSO
BEZ235
PD173074
AZD8055
GSK690693
Combo
−1.8
−2
−2.2
−2.4
−2.6
−2.8
−3
0
5m
15m
30m
60m
2h
4h
12h
24h
48h

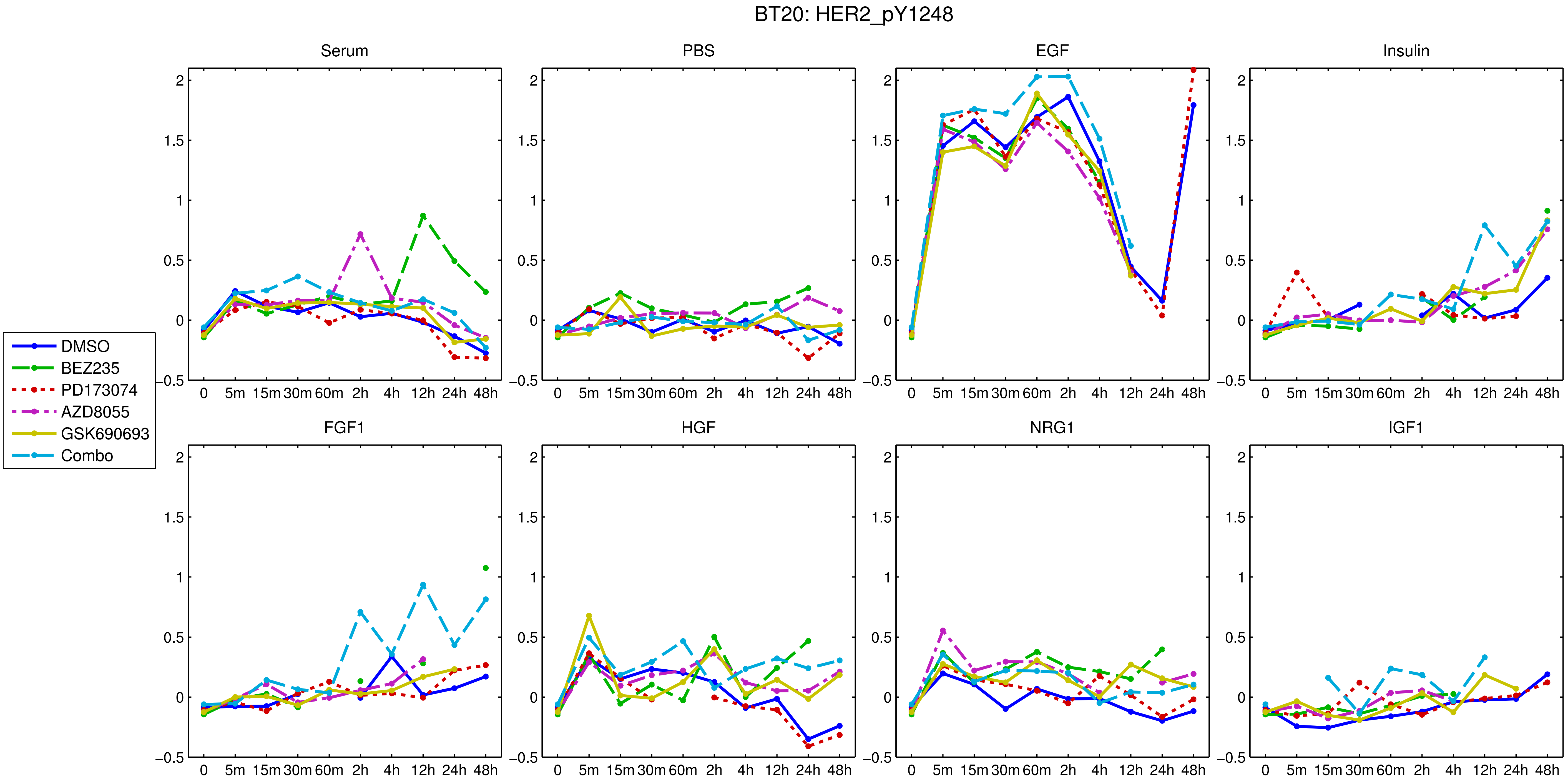

BT20: HER2_pY1248
Serum
PBS
EGF
Insulin
FGF1
HGF
NRG1
IGF1
DMSO
BEZ235
PD173074
AZD8055
GSK690693
Combo
0
5m
15m
30m
60m
2h
4h
12h
24h
48h
2
1.5
1
0.5
0
−0.5

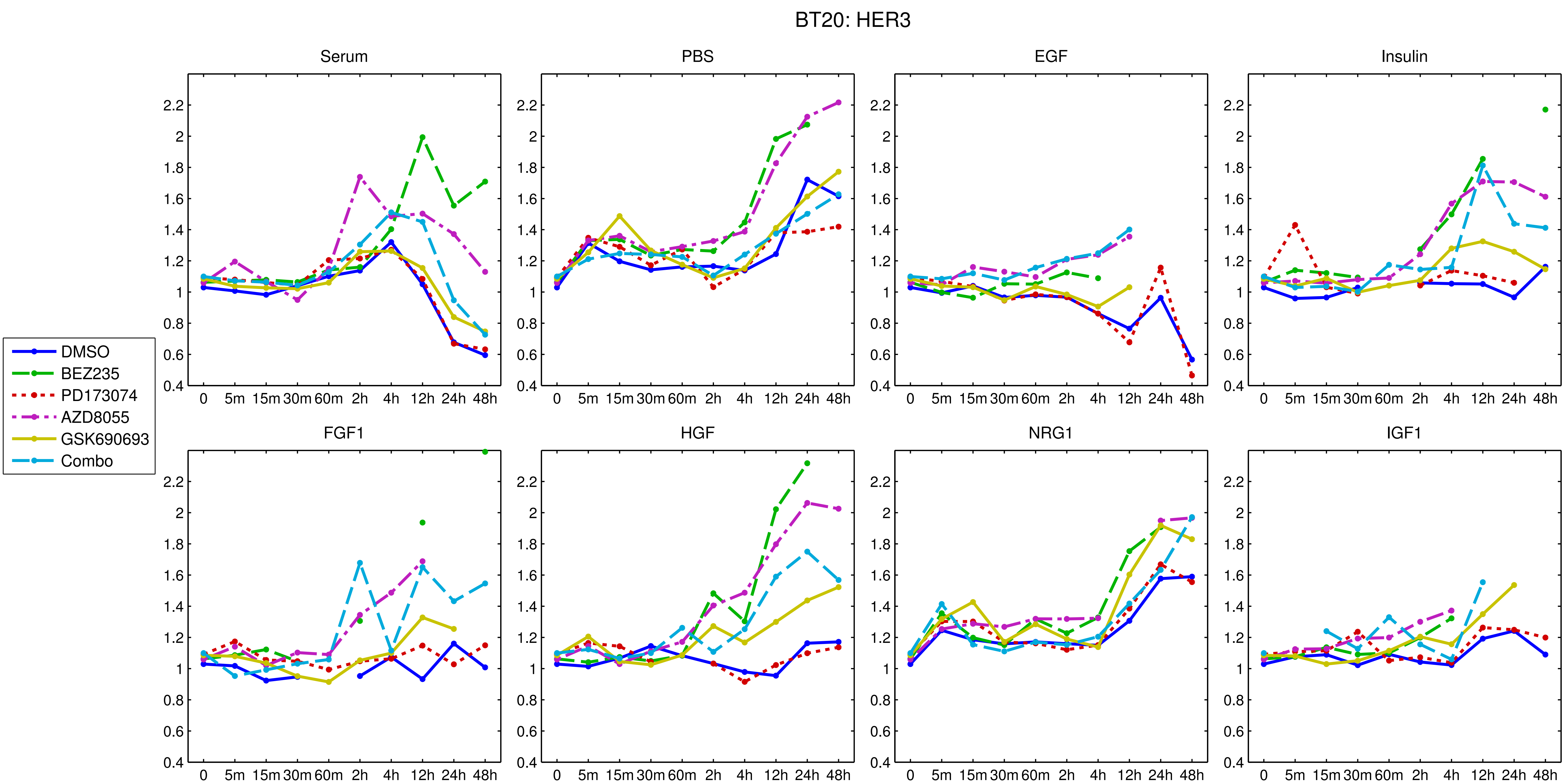

BT20: HER3
Serum
PBS
EGF
Insulin
FGF1
HGF
NRG1
IGF1
DMSO
BEZ235
PD173074
AZD8055
GSK690693
Combo
0.4
0.6
0.8
1
1.2
1.4
1.6
1.8
2
2.2
0 5m 15m 30m 60m 2h 4h 12h 24h 48h

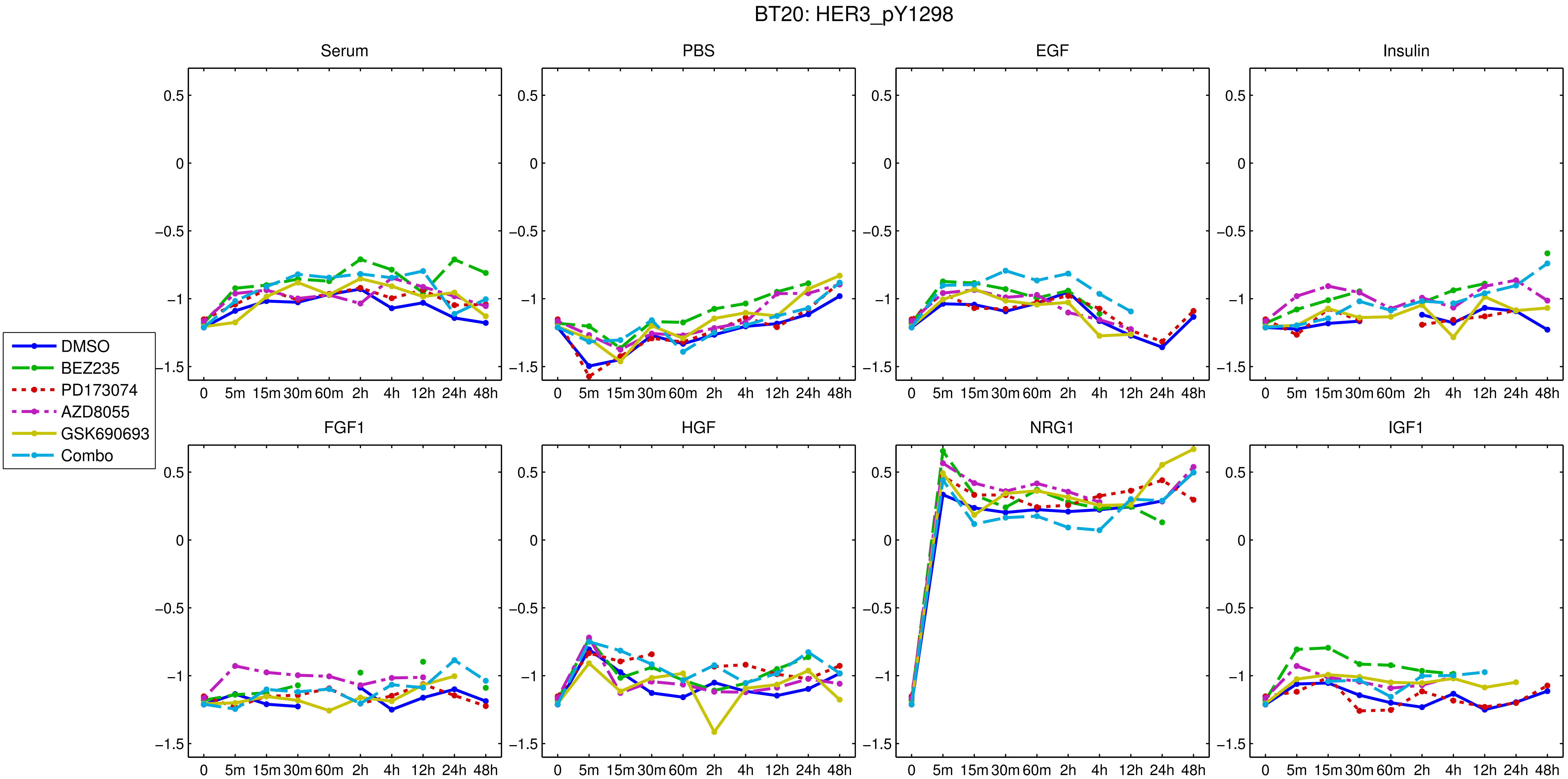
BT20: HER3_pY1298
Serum
PBS
EGF
Insulin
FGF1
HGF
NRG1
IGF1
0.5
0
−0.5
−1
−1.5
0
5m
15m
30m
60m
2h
4h
12h
24h
48h
DMSO
BEZ235
PD173074
AZD8055
GSK690693
Combo

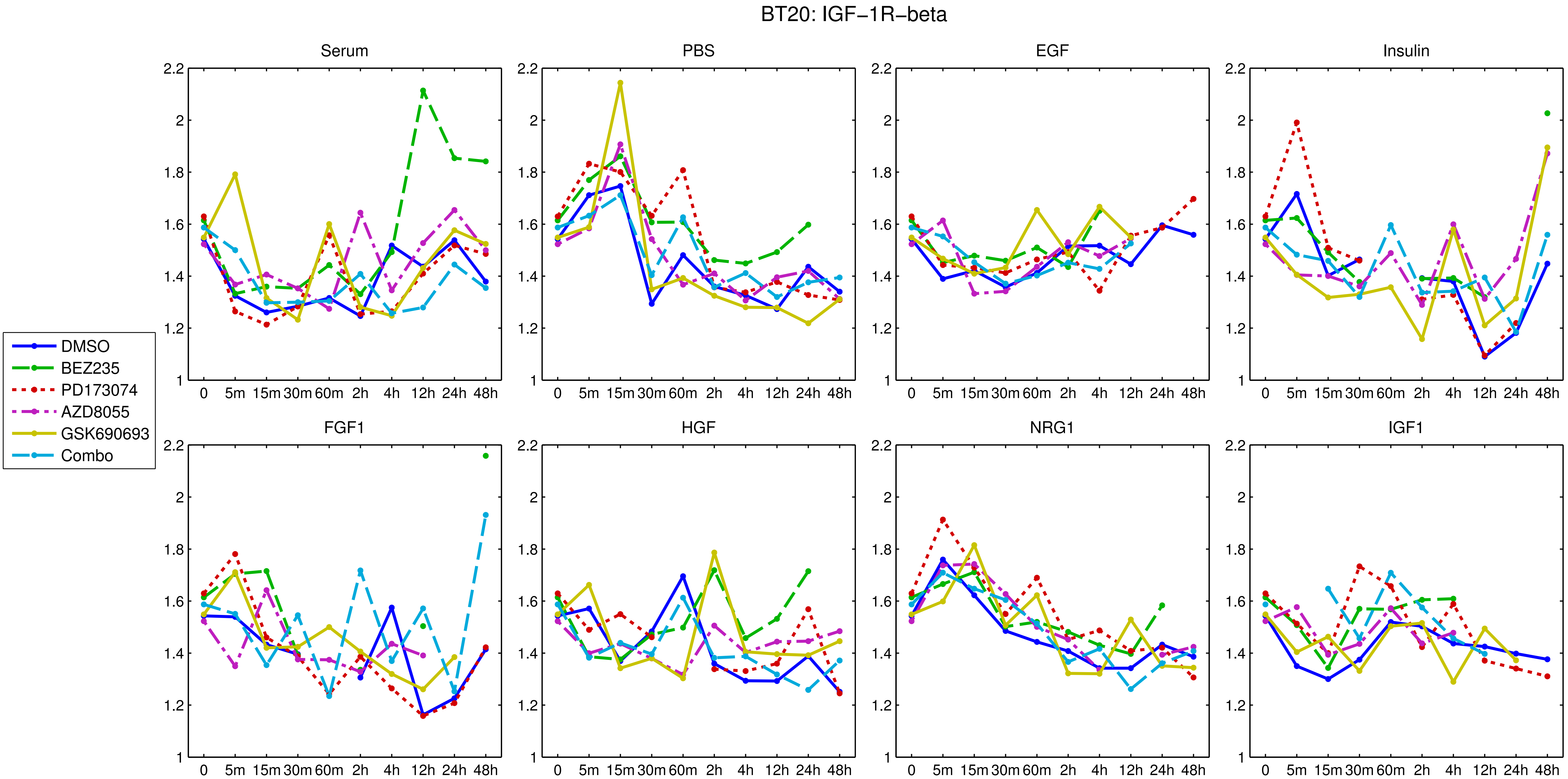
BT20: IGF−1R−beta
Serum
PBS
EGF
Insulin
FGF1
HGF
NRG1
IGF1
DMSO
BEZ235
PD173074
AZD8055
GSK690693
Combo
2.2
2
1.8
1.6
1.4
1.2
1
0
5m
15m
30m
60m
2h
4h
12h
24h
48h

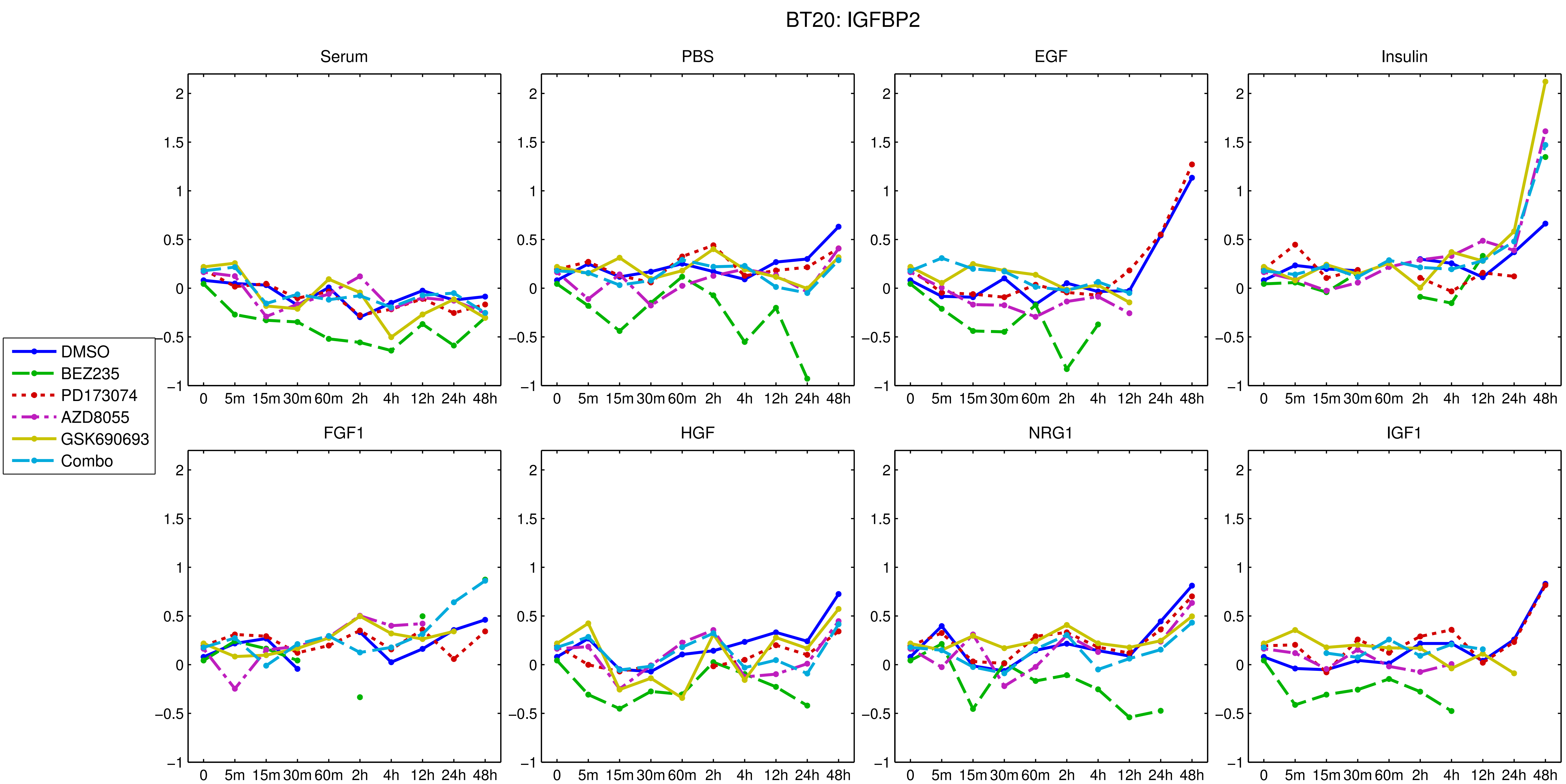

BT20: IGFBP2
Serum
PBS
EGF
Insulin
FGF1
HGF
NRG1
IGF1
DMSO
BEZ235
PD173074
AZD8055
GSK690693
Combo
0
5m
15m
30m
60m
2h
4h
12h
24h
48h
2
1.5
1
0.5
0
−0.5
−1

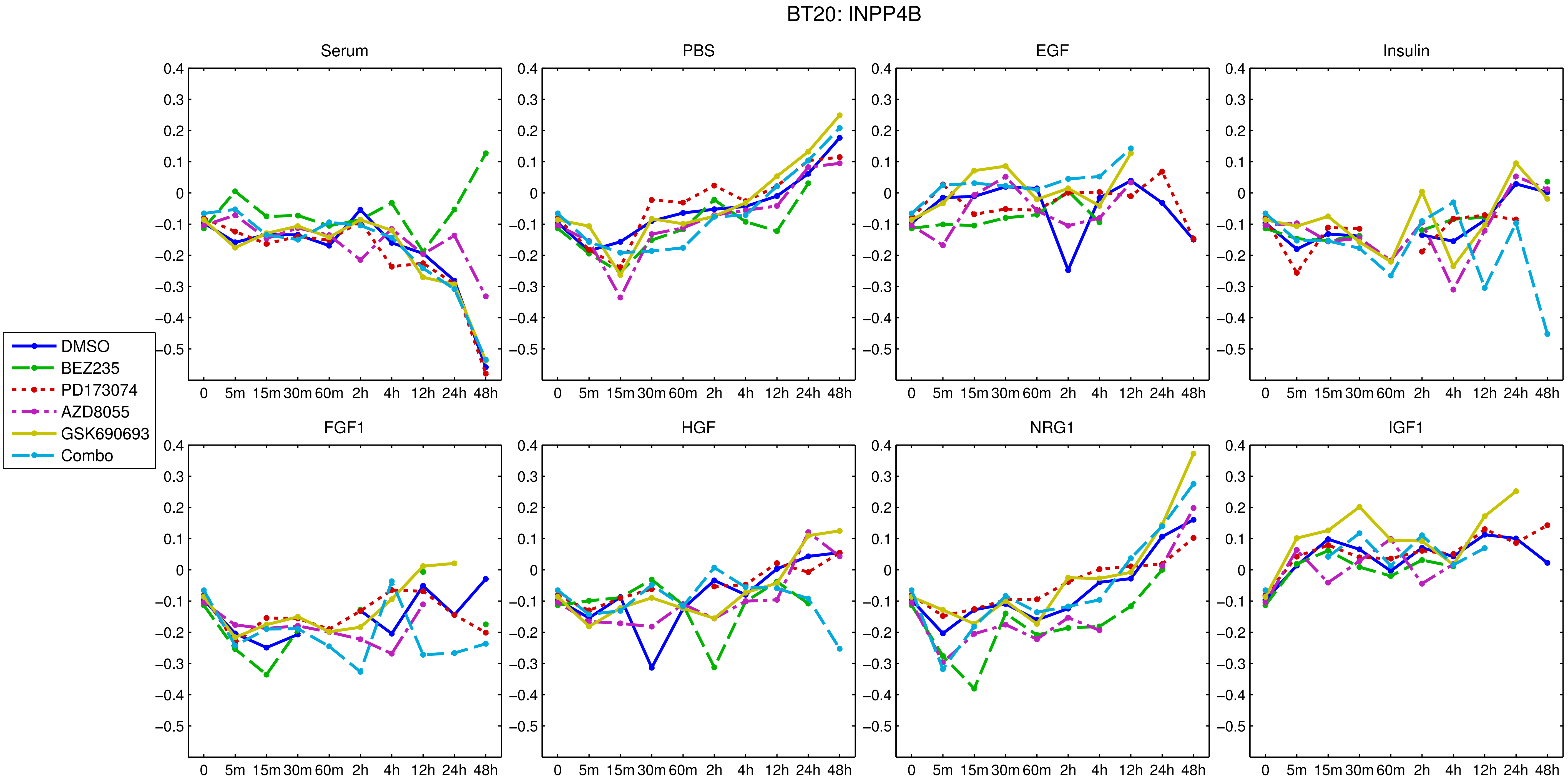

BT20: INPP4B
Serum
PBS
EGF
Insulin
FGF1
HGF
NRG1
IGF1
DMSO
BEZ235
PD173074
AZD8055
GSK690693
Combo
0.4
0.3
0.2
0.1
0
−0.1
−0.2
−0.3
−0.4
−0.5
0
5m
15m
30m
60m
2h
4h
12h
24h
48h

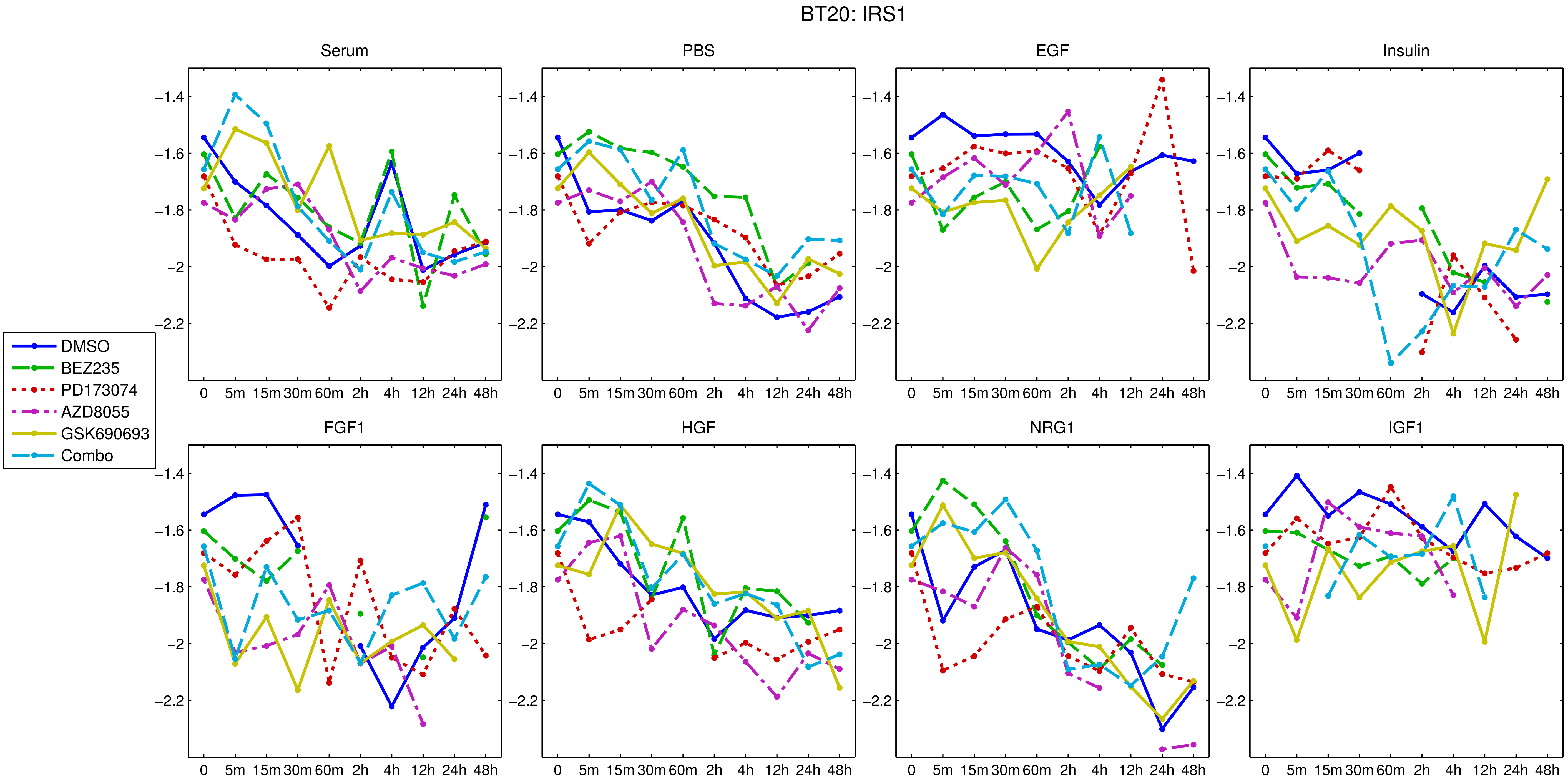

BT20: IRS1
Serum
PBS
EGF
Insulin
FGF1
HGF
NRG1
IGF1
−1.4
−1.6
−1.8
−2
−2.2
0 5m 15m 30m 60m 2h 4h 12h 24h 48h
DMSO
BEZ235
PD173074
AZD8055
GSK690693
Combo

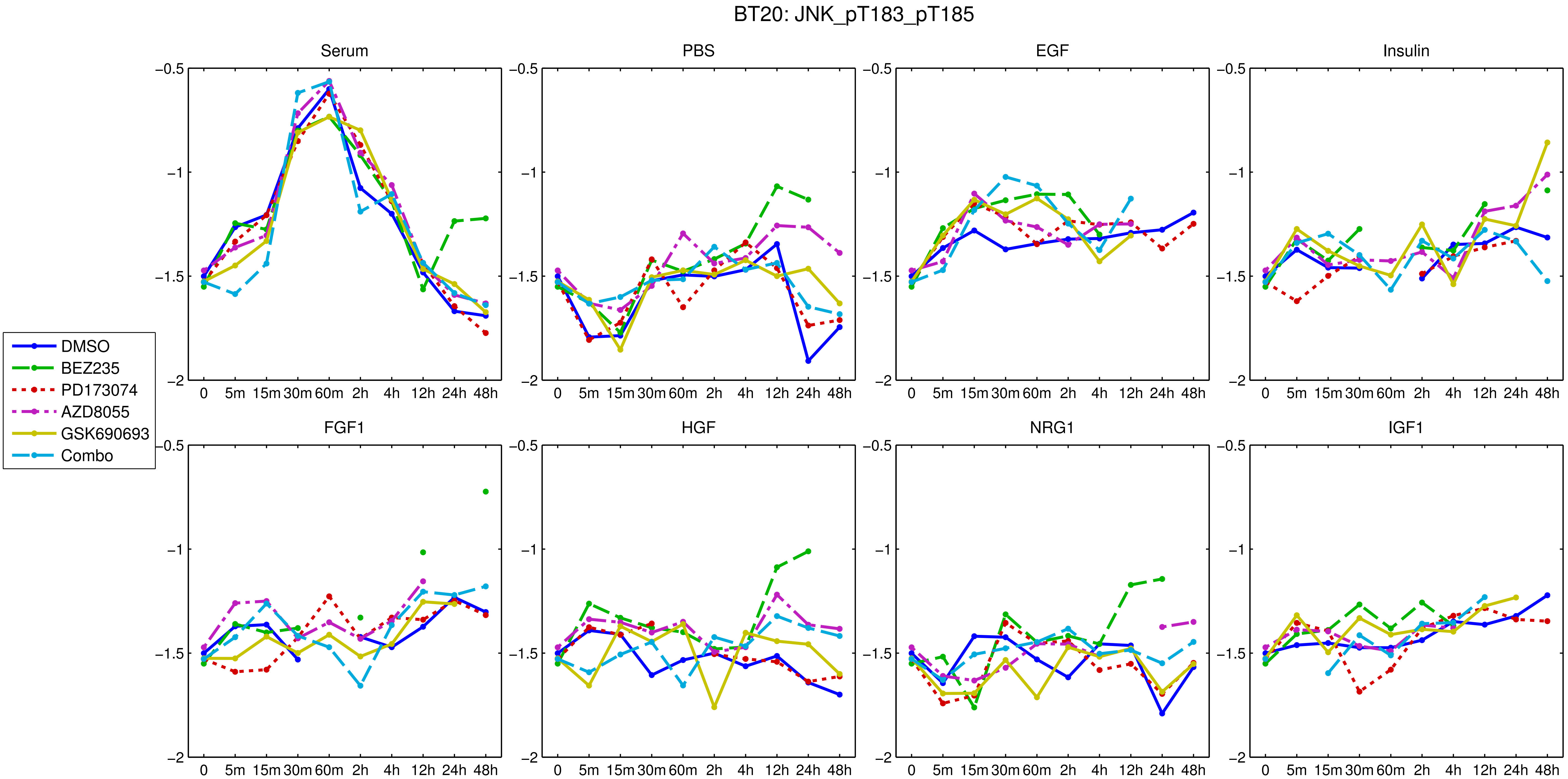

BT20: JNK_pT183_pT185
Serum
PBS
EGF
Insulin
FGF1
HGF
NRG1
IGF1
−0.5
−1
−1.5
−2
0
5m
15m
30m
60m
2h
4h
12h
24h
48h
DMSO
BEZ235
PD173074
AZD8055
GSK690693
Combo

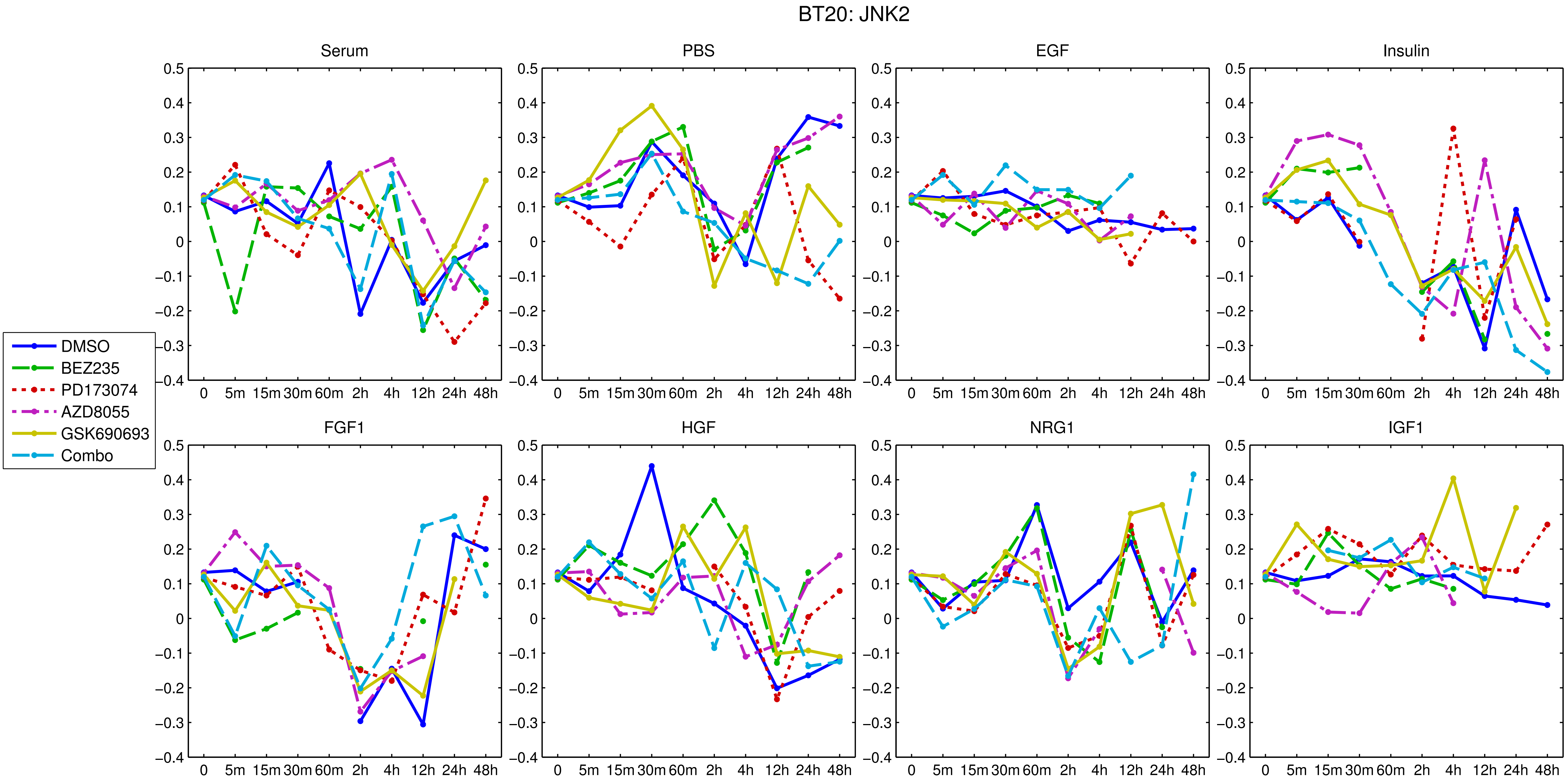
BT20: JNK2
Serum
PBS
EGF
Insulin
FGF1
HGF
NRG1
IGF1
DMSO
BEZ235
PD173074
AZD8055
GSK690693
Combo
0.5
0.4
0.3
0.2
0.1
0
−0.1
−0.2
−0.3
−0.4
0
5m
15m
30m
60m
2h
4h
12h
24h
48h

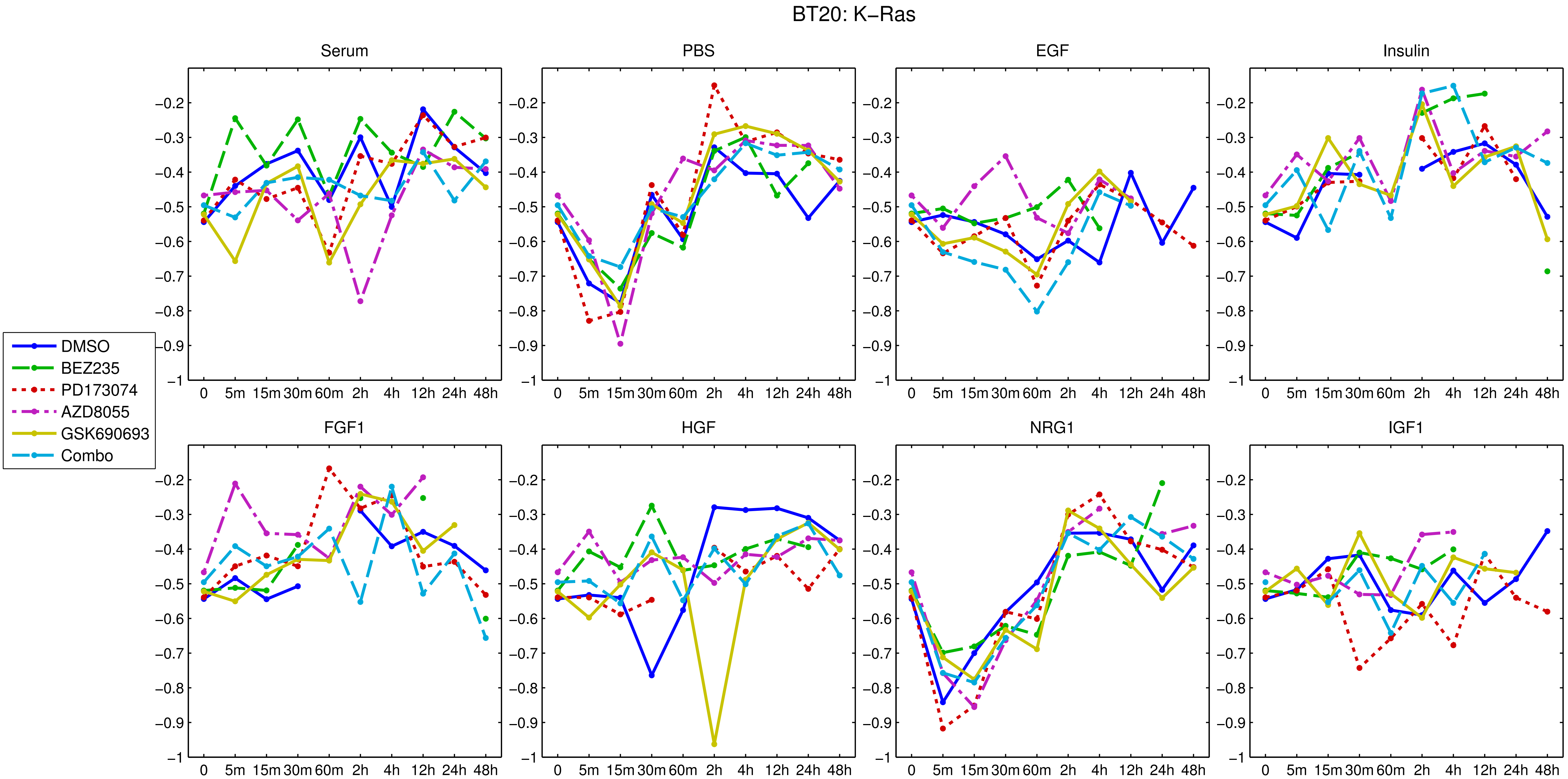

BT20: K−Ras
Serum
PBS
EGF
Insulin
FGF1
HGF
NRG1
IGF1
DMSO
BEZ235
PD173074
AZD8055
GSK690693
Combo
−0.2
−0.3
−0.4
−0.5
−0.6
−0.7
−0.8
−0.9
−1
0
5m
15m
30m
60m
2h
4h
12h
24h
48h

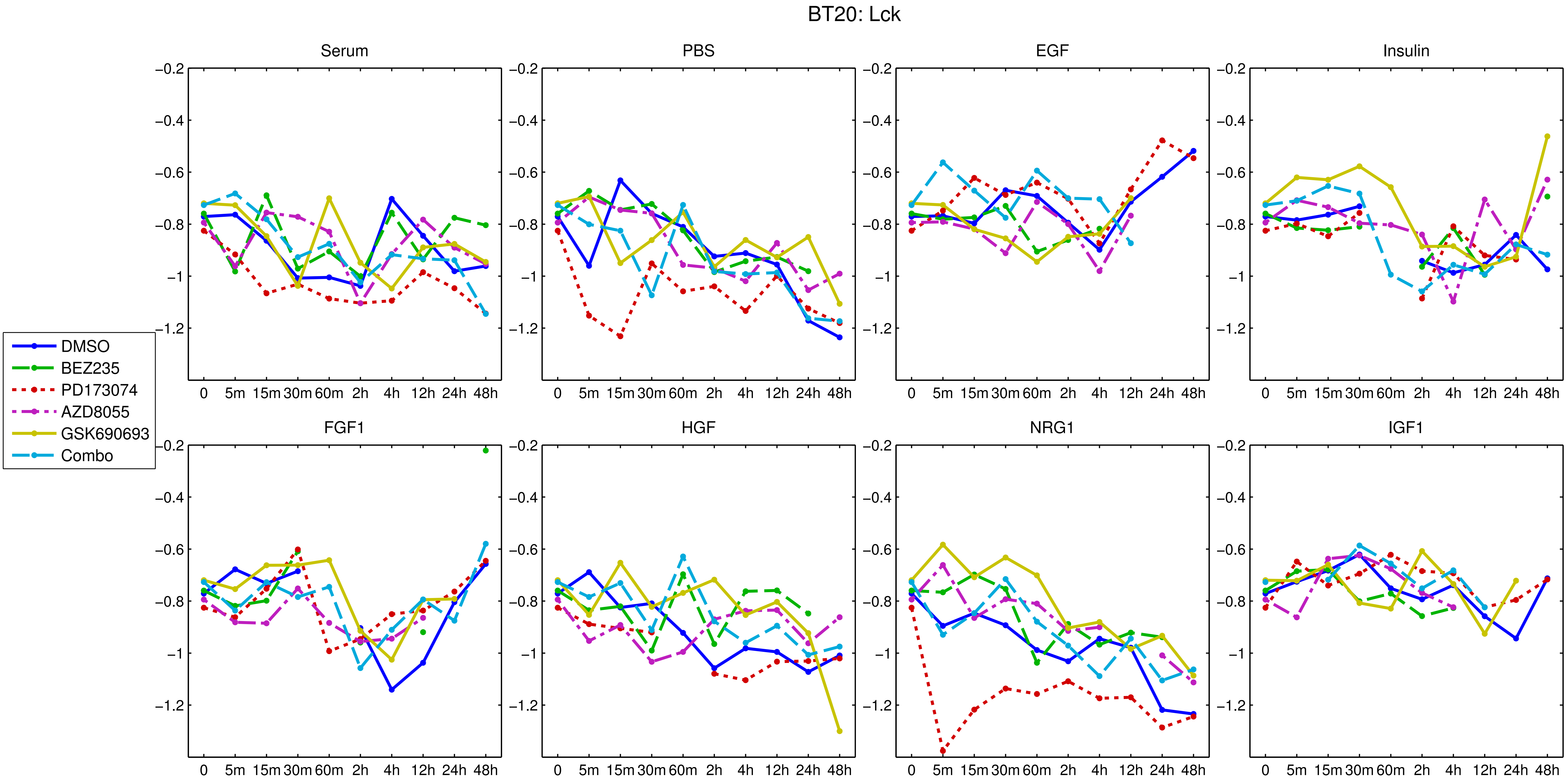

BT20: Lck
Serum
PBS
EGF
Insulin
FGF1
HGF
NRG1
IGF1
DMSO
BEZ235
PD173074
AZD8055
GSK690693
Combo
−0.2
−0.4
−0.6
−0.8
−1
−1.2
0
5m
15m
30m
60m
2h
4h
12h
24h
48h

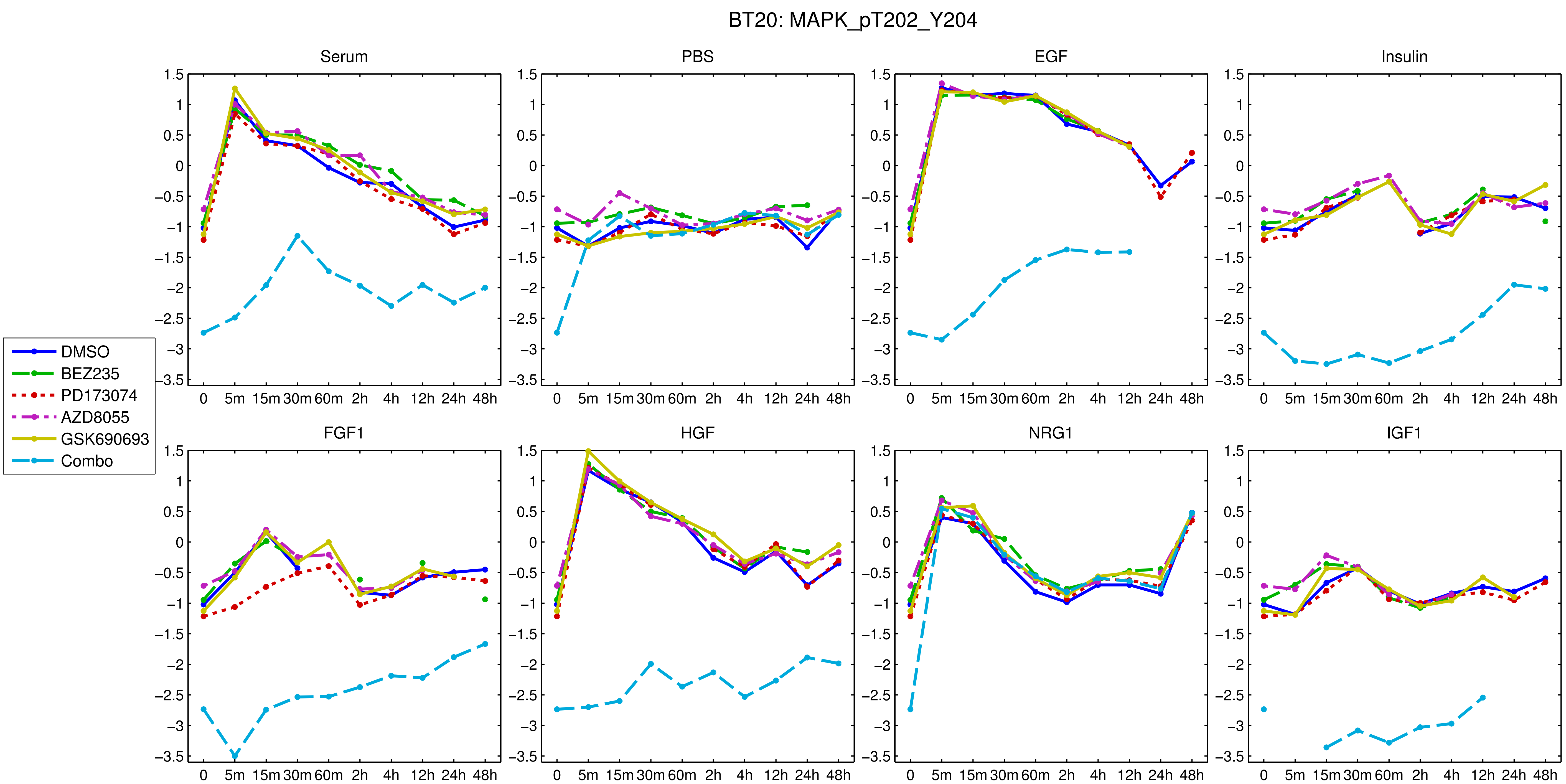

BT20: MAPK_pT202_Y204
Serum
PBS
EGF
Insulin
FGF1
HGF
NRG1
IGF1
DMSO
BEZ235
PD173074
AZD8055
GSK690693
Combo
1.5
1
0.5
0
−0.5
−1
−1.5
−2
−2.5
−3
−3.5
0
5m
15m
30m
60m
2h
4h
12h
24h
48h

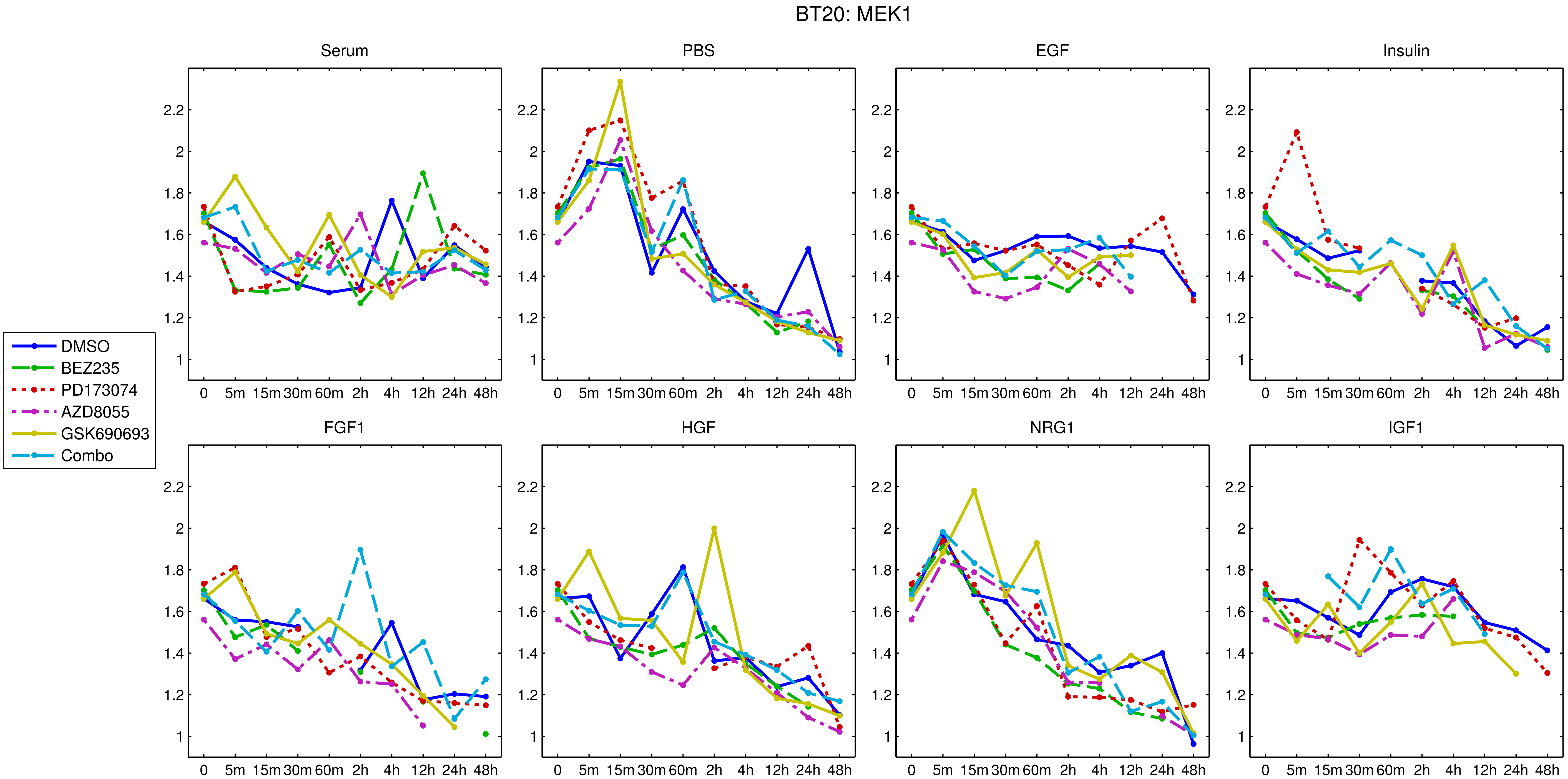
BT20: MEK1
Serum
PBS
EGF
Insulin
FGF1
HGF
NRG1
IGF1
DMSO
BEZ235
PD173074
AZD8055
GSK690693
Combo
2.2
2
1.8
1.6
1.4
1.2
1
0
5m
15m
30m
60m
2h
4h
12h
24h
48h

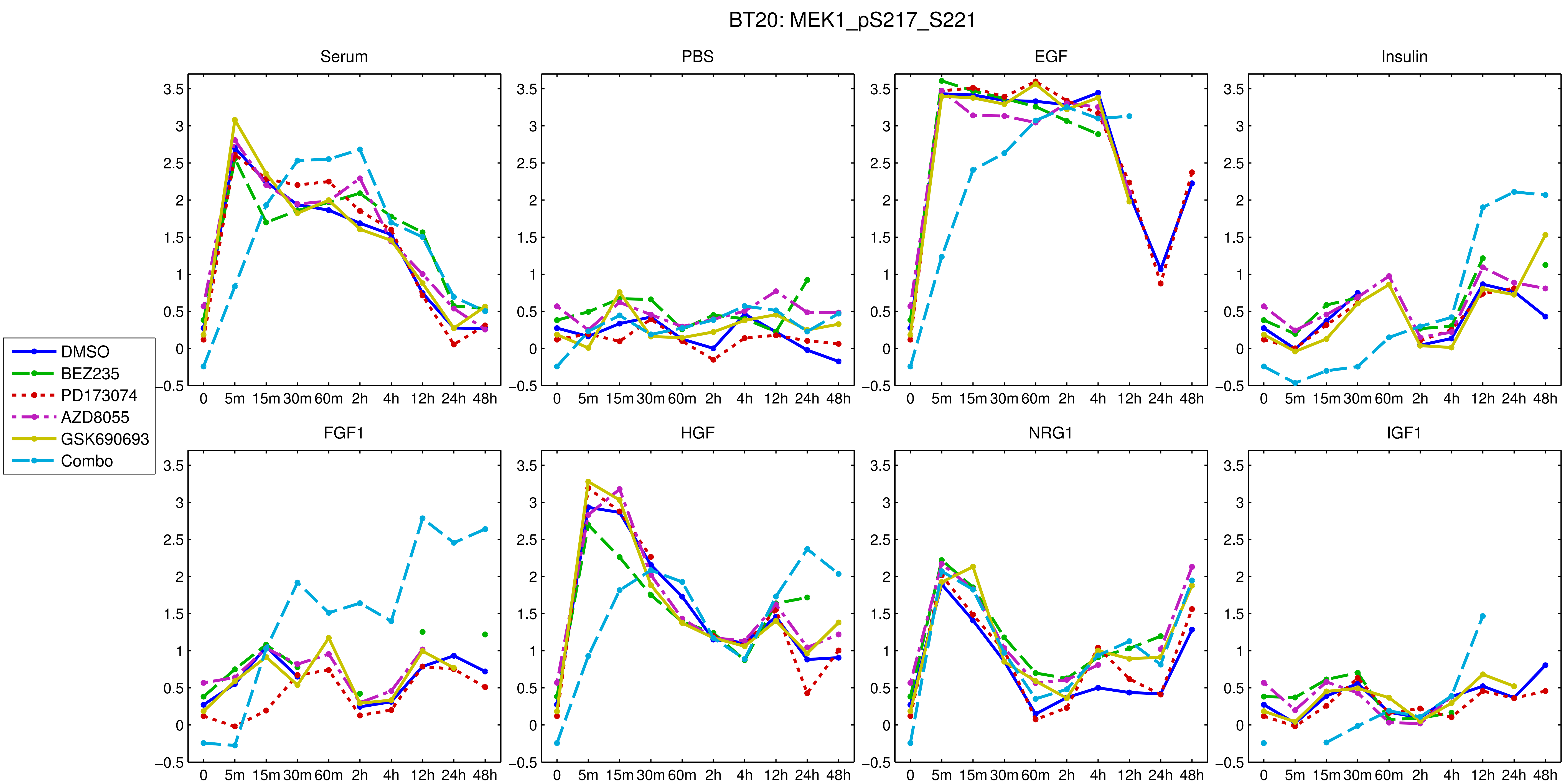

BT20: MEK1_pS217_S221
Serum
PBS
EGF
Insulin
FGF1
HGF
NRG1
IGF1
DMSO
BEZ235
PD173074
AZD8055
GSK690693
Combo
3.5
3
2.5
2
1.5
1
0.5
0
−0.5
0
5m
15m
30m
60m
2h
4h
12h
24h
48h

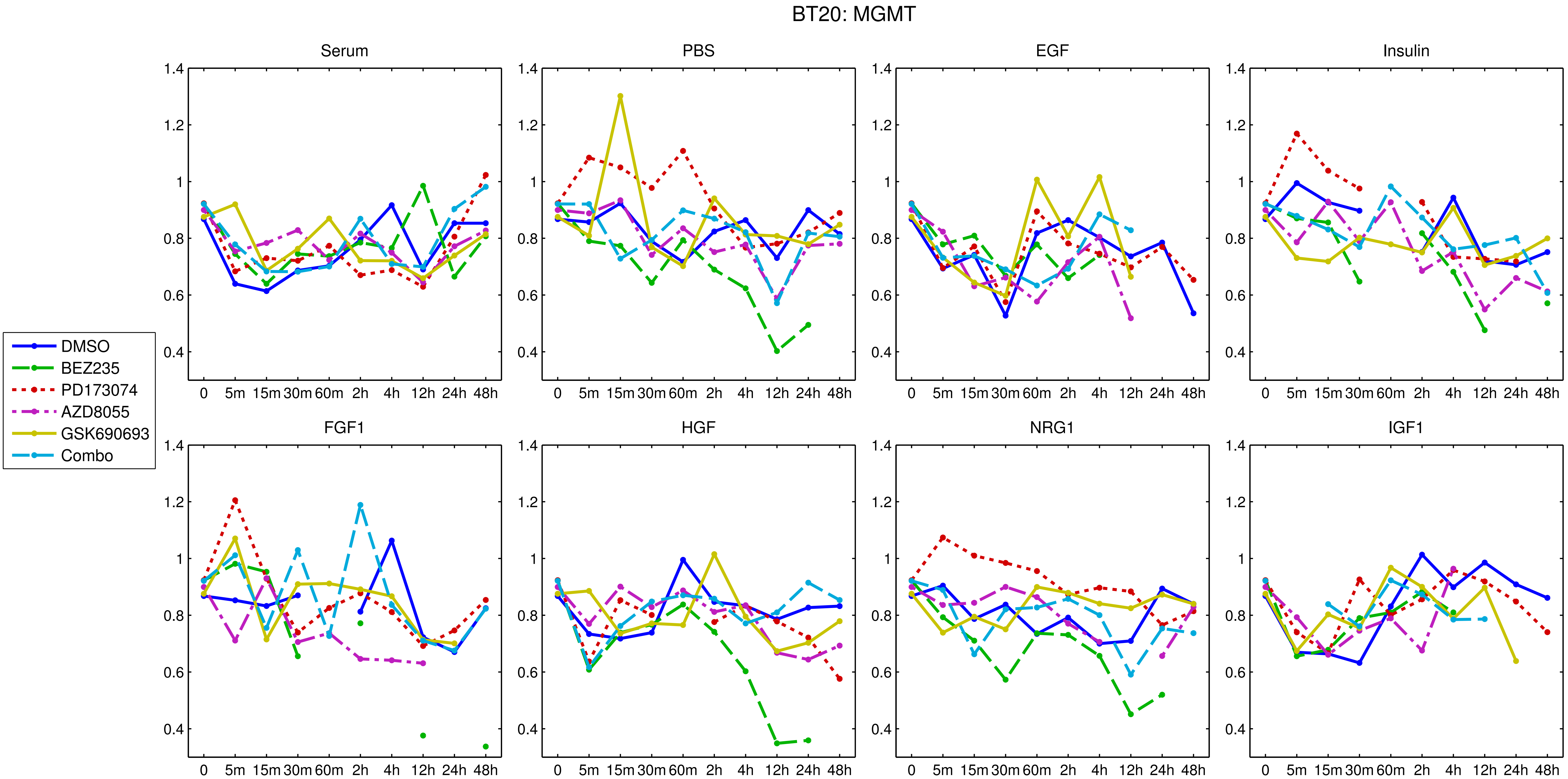

BT20: MGMT
Serum
PBS
EGF
Insulin
FGF1
HGF
NRG1
IGF1
0.4
0.6
0.8
1
1.2
1.4
0
5m
15m
30m
60m
2h
4h
12h
24h
48h
DMSO
BEZ235
PD173074
AZD8055
GSK690693
Combo

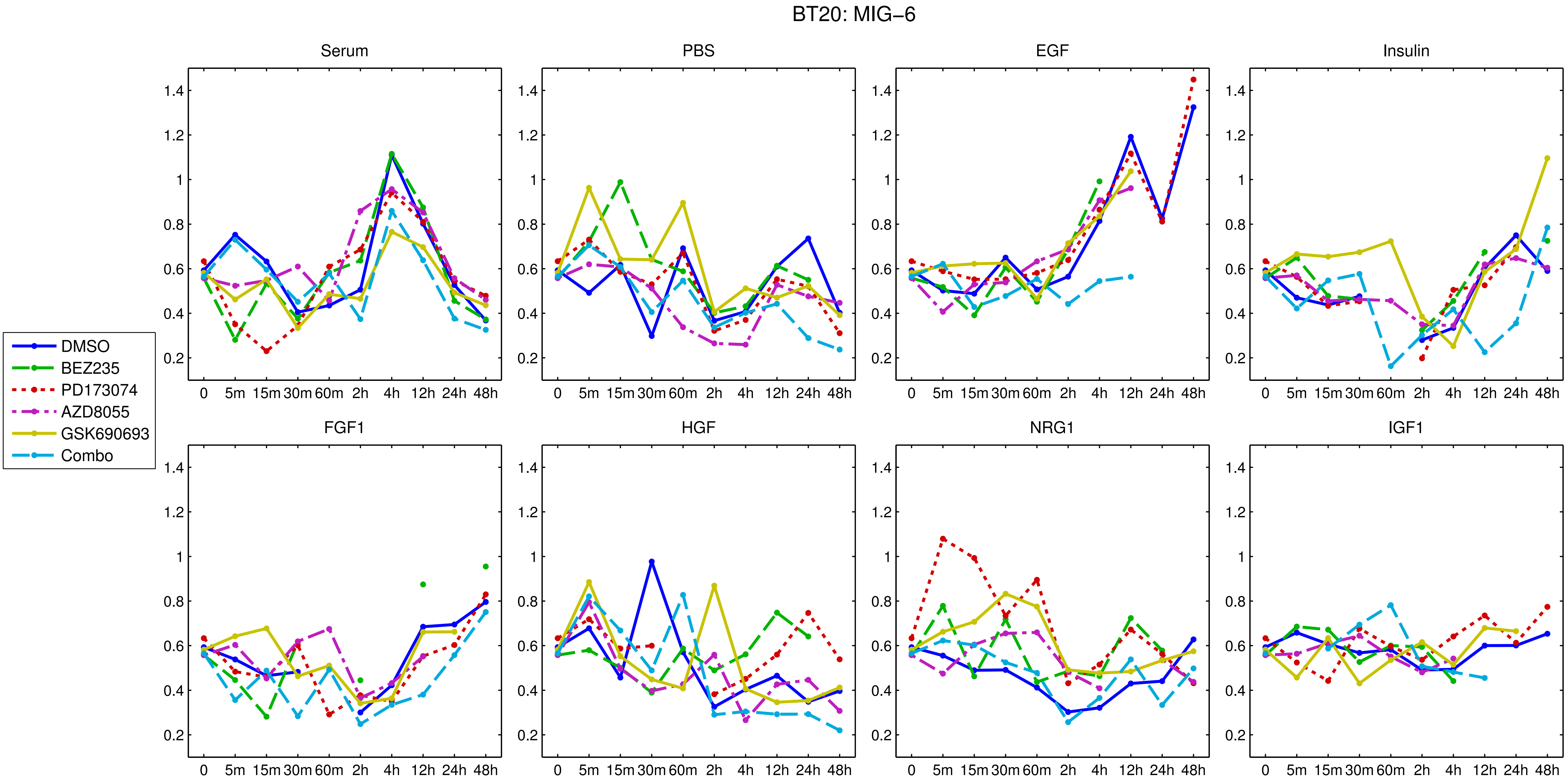

BT20: MIG−6
Serum
PBS
EGF
Insulin
FGF1
HGF
NRG1
IGF1
DMSO
BEZ235
PD173074
AZD8055
GSK690693
Combo
0.2
0.4
0.6
0.8
1
1.2
1.4
0
5m
15m
30m
60m
2h
4h
12h
24h
48h

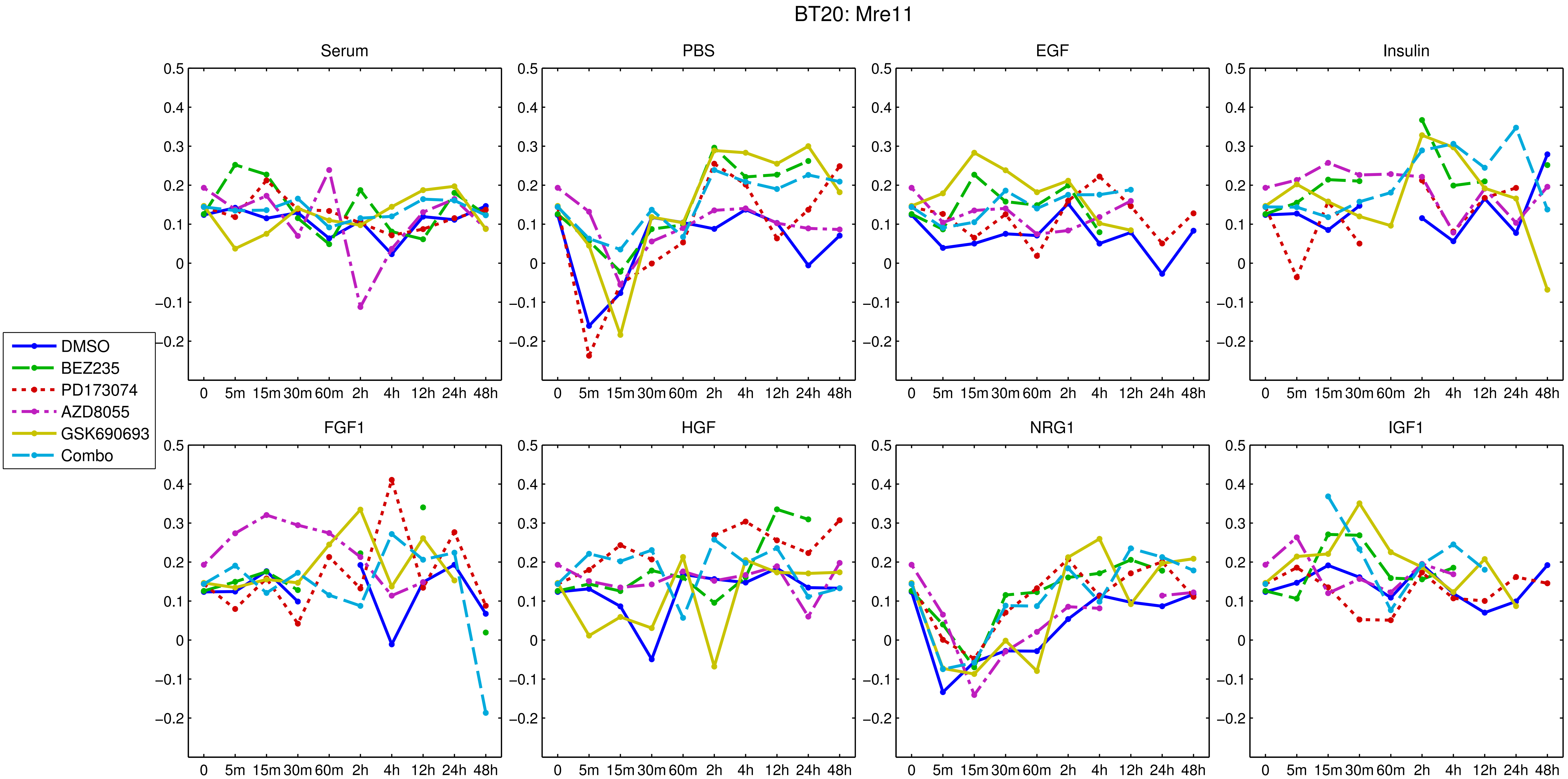
BT20: Mre11
Serum
PBS
EGF
Insulin
FGF1
HGF
NRG1
IGF1
0.5
0.4
0.3
0.2
0.1
0
−0.1
−0.2
0
5m
15m
30m
60m
2h
4h
12h
24h
48h
DMSO
BEZ235
PD173074
AZD8055
GSK690693
Combo

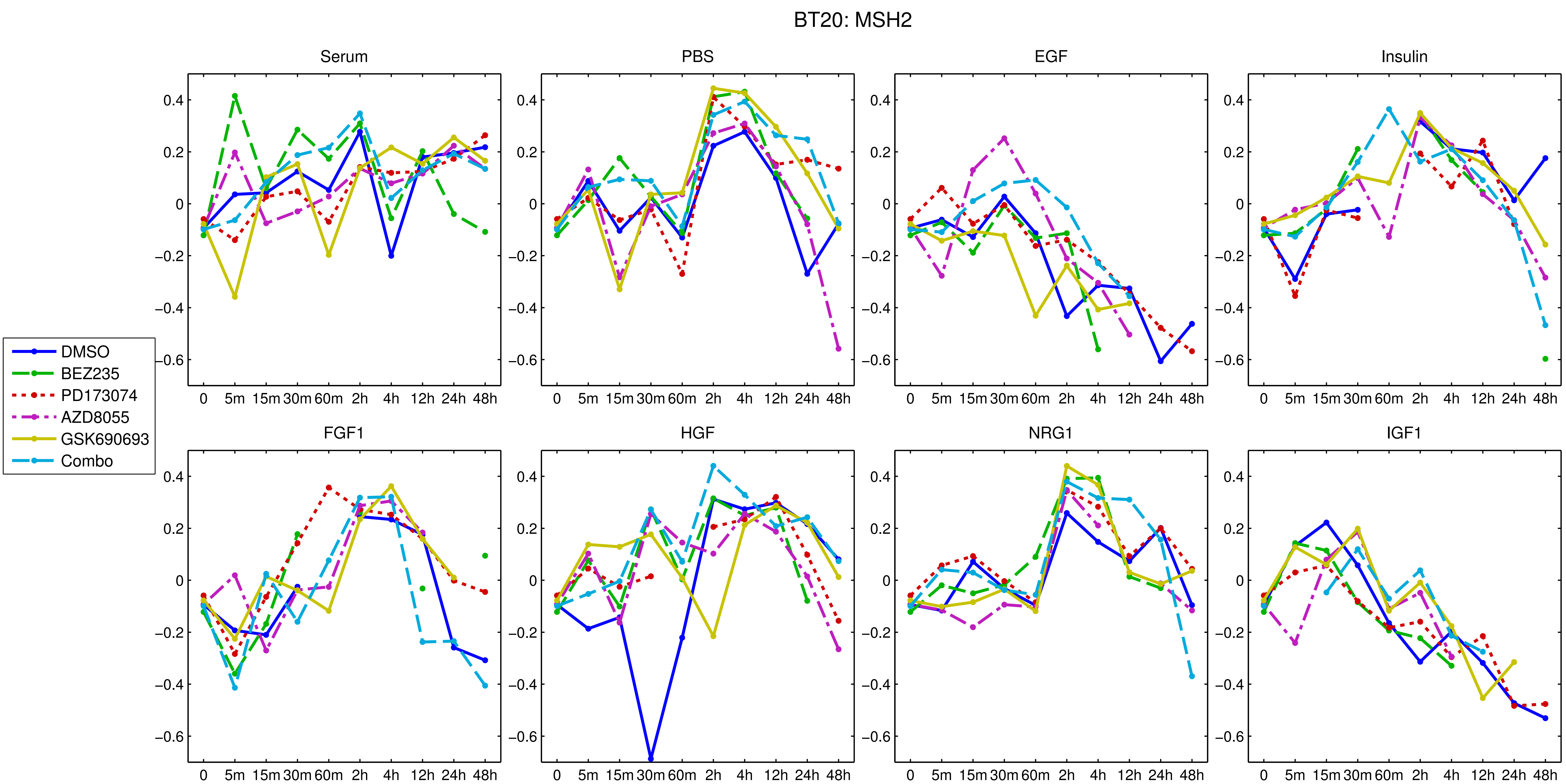
BT20: MSH2
Serum
PBS
EGF
Insulin
FGF1
HGF
NRG1
IGF1
0.4
0.2
0
−0.2
−0.4
−0.6
0
5m
15m
30m
60m
2h
4h
12h
24h
48h
DMSO
BEZ235
PD173074
AZD8055
GSK690693
Combo

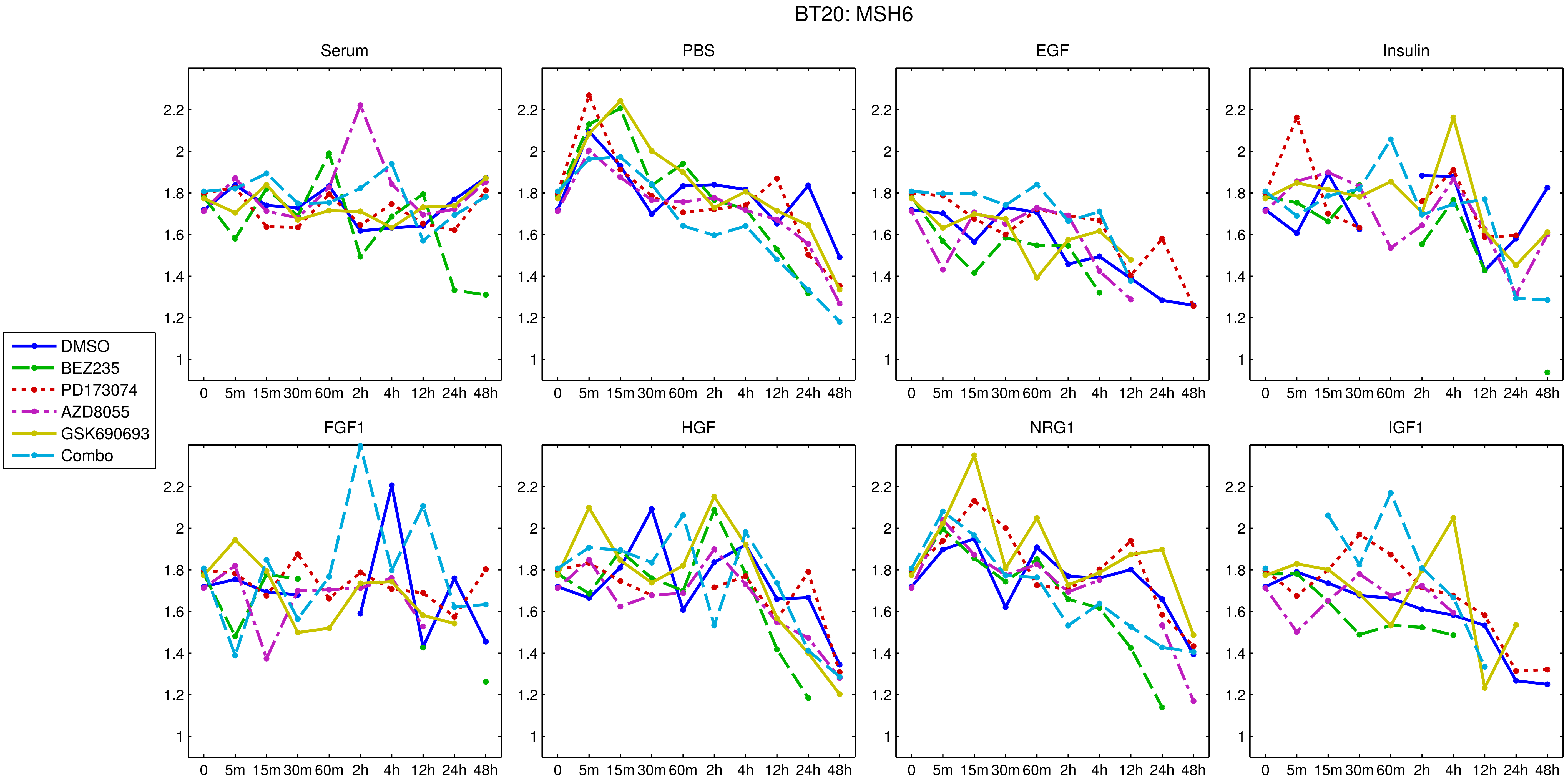
BT20: MSH6
Serum
PBS
EGF
Insulin
FGF1
HGF
NRG1
IGF1
DMSO
BEZ235
PD173074
AZD8055
GSK690693
Combo
2.2
2
1.8
1.6
1.4
1.2
1
0
5m
15m
30m
60m
2h
4h
12h
24h
48h

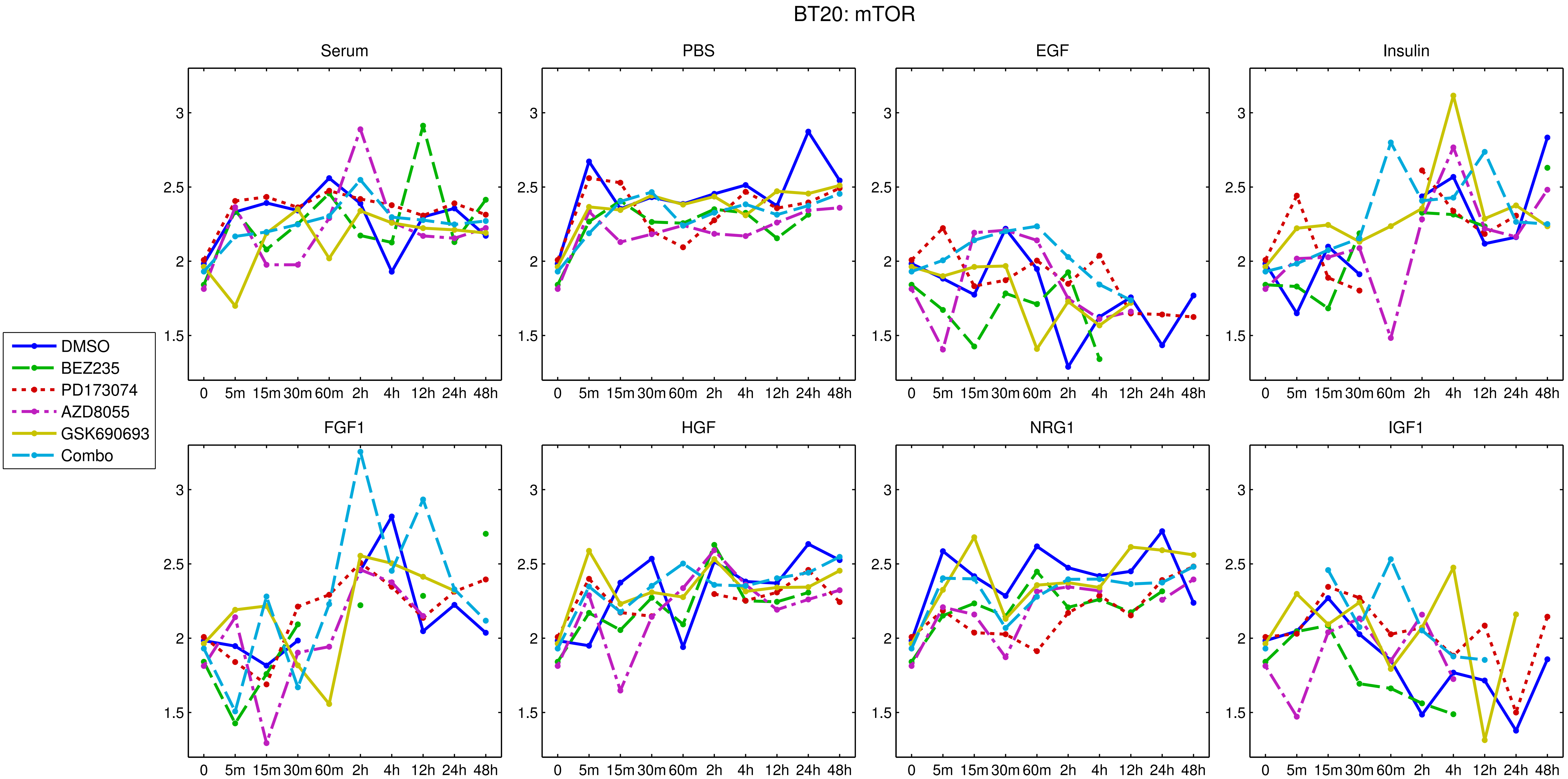
BT20: mTOR
Serum
PBS
EGF
Insulin
FGF1
HGF
NRG1
IGF1
DMSO
BEZ235
PD173074
AZD8055
GSK690693
Combo
0 5m 15m 30m 60m 2h 4h 12h 24h 48h
1.5
2
2.5
3

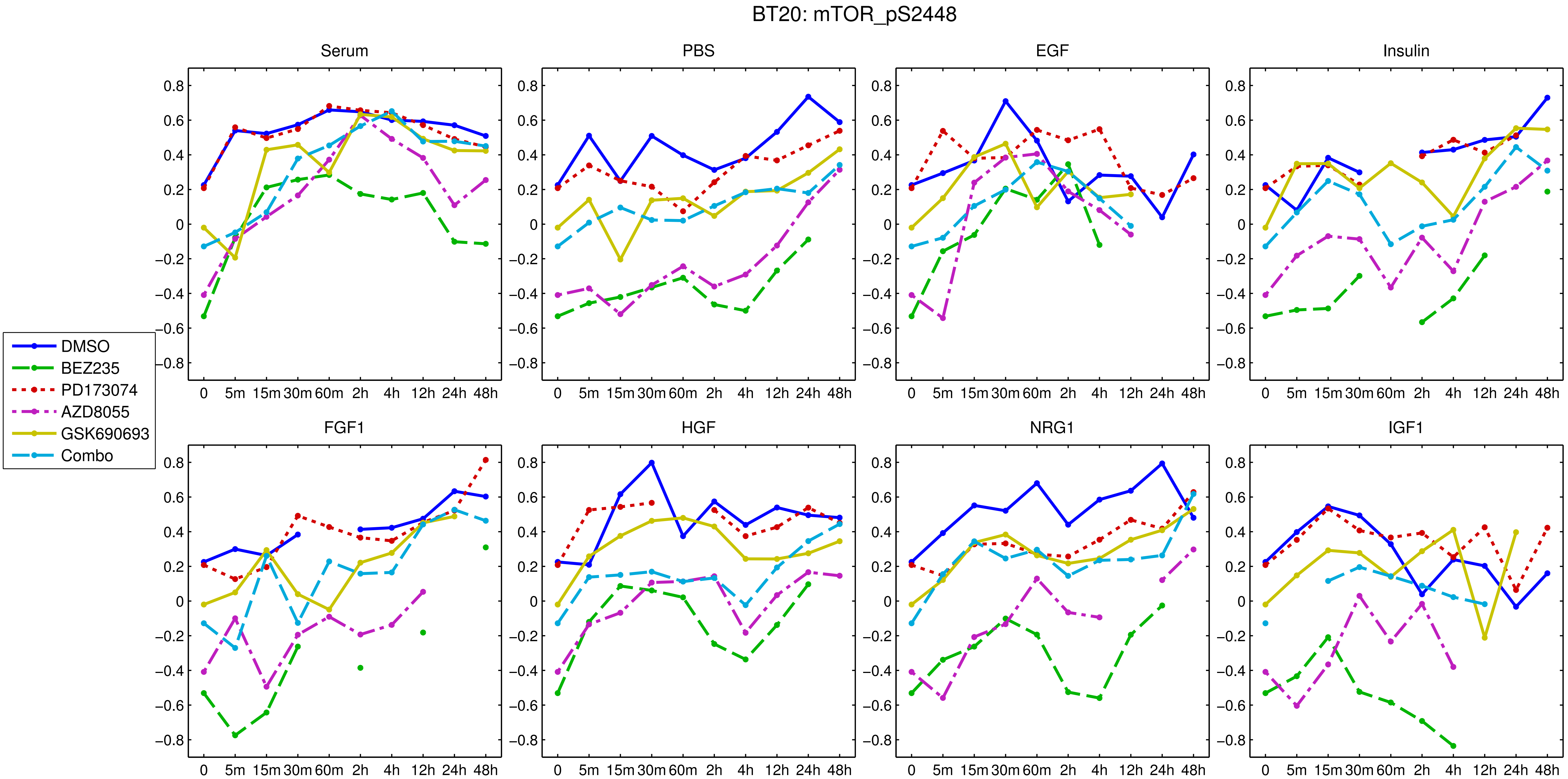

BT20: mTOR_pS2448
Serum
PBS
EGF
Insulin
FGF1
HGF
NRG1
IGF1
DMSO
BEZ235
PD173074
AZD8055
GSK690693
Combo
0
5m
15m
30m
60m
2h
4h
12h
24h
48h
0.8
0.6
0.4
0.2
−0.2
−0.4
−0.6
−0.8

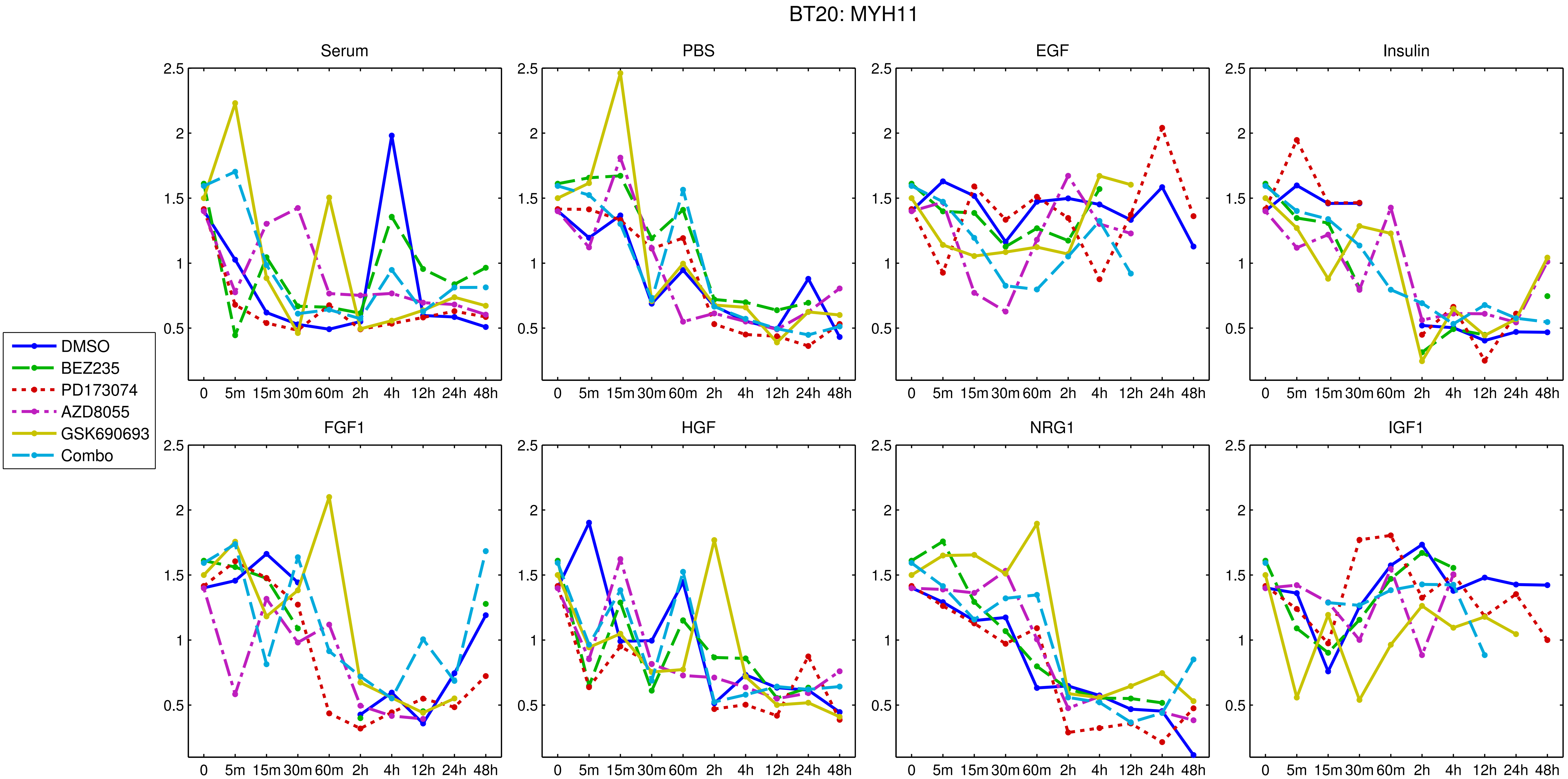

BT20: MYH11
Serum
PBS
EGF
Insulin
FGF1
HGF
NRG1
IGF1
DMSO
BEZ235
PD173074
AZD8055
GSK690693
Combo
0
5m
15m
30m
60m
2h
4h
12h
24h
48h
0.5
1
1.5
2
2.5

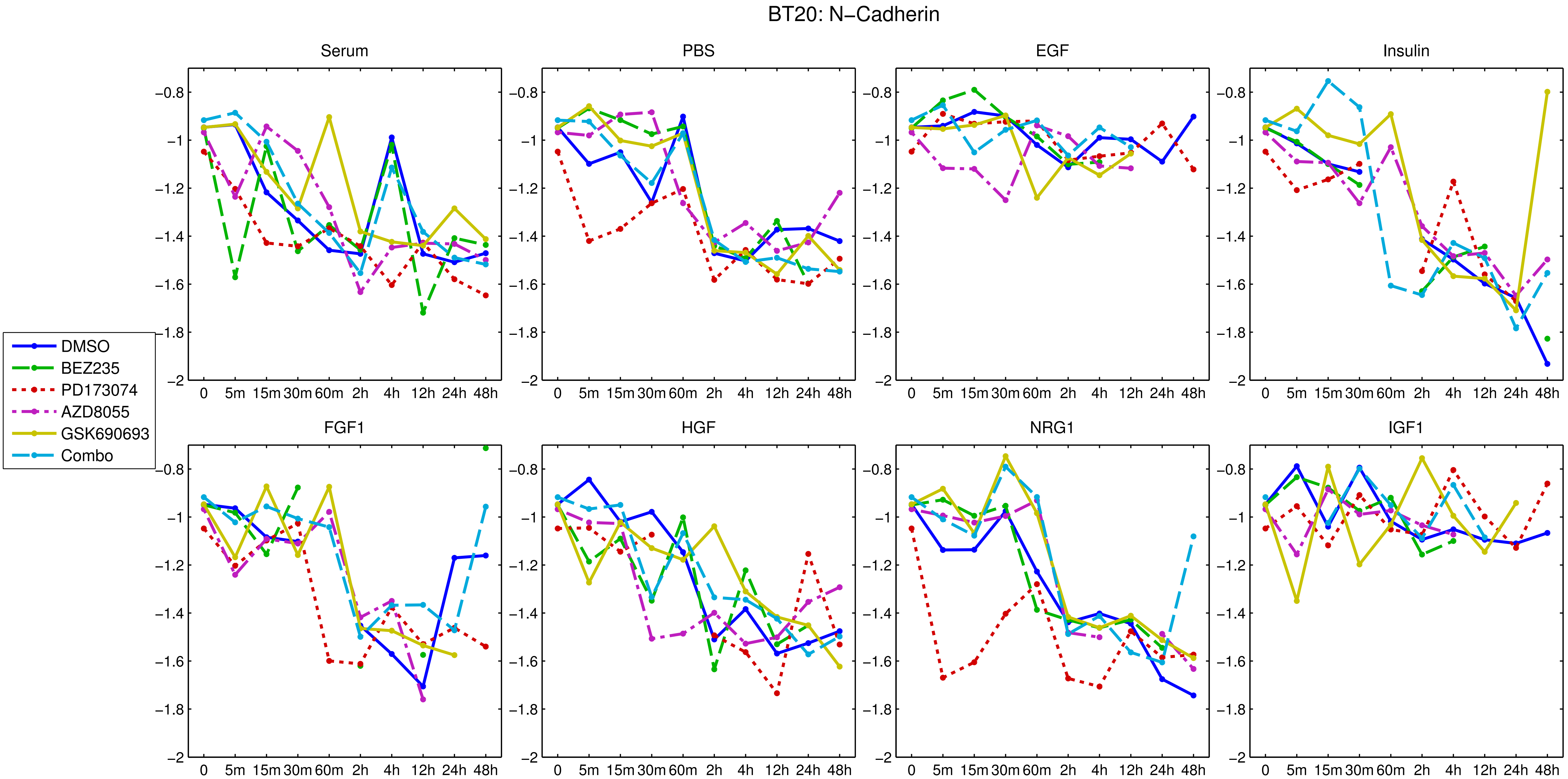
BT20: N−Cadherin
Serum
PBS
EGF
Insulin
FGF1
HGF
NRG1
IGF1
DMSO
BEZ235
PD173074
AZD8055
GSK690693
Combo
−0.8
−1
−1.2
−1.4
−1.6
−1.8
−2
0
5m
15m
30m
60m
2h
4h
12h
24h
48h

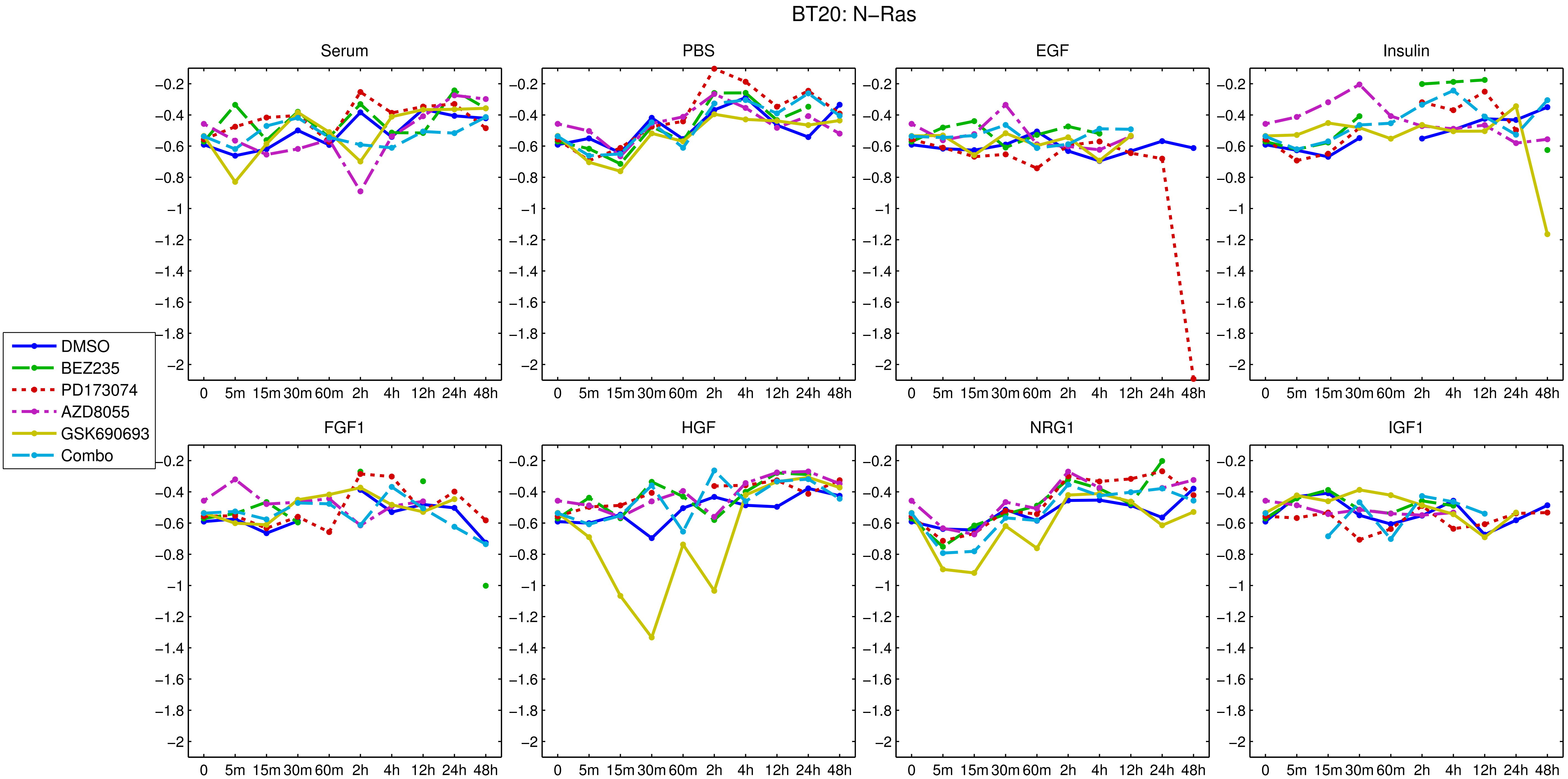

BT20: N−Ras
Serum
PBS
EGF
Insulin
FGF1
HGF
NRG1
IGF1
DMSO
BEZ235
PD173074
AZD8055
GSK690693
Combo
−0.2
−0.4
−0.6
−0.8
−1
−1.2
−1.4
−1.6
−1.8
−2
0
5m
15m
30m
60m
2h
4h
12h
24h
48h

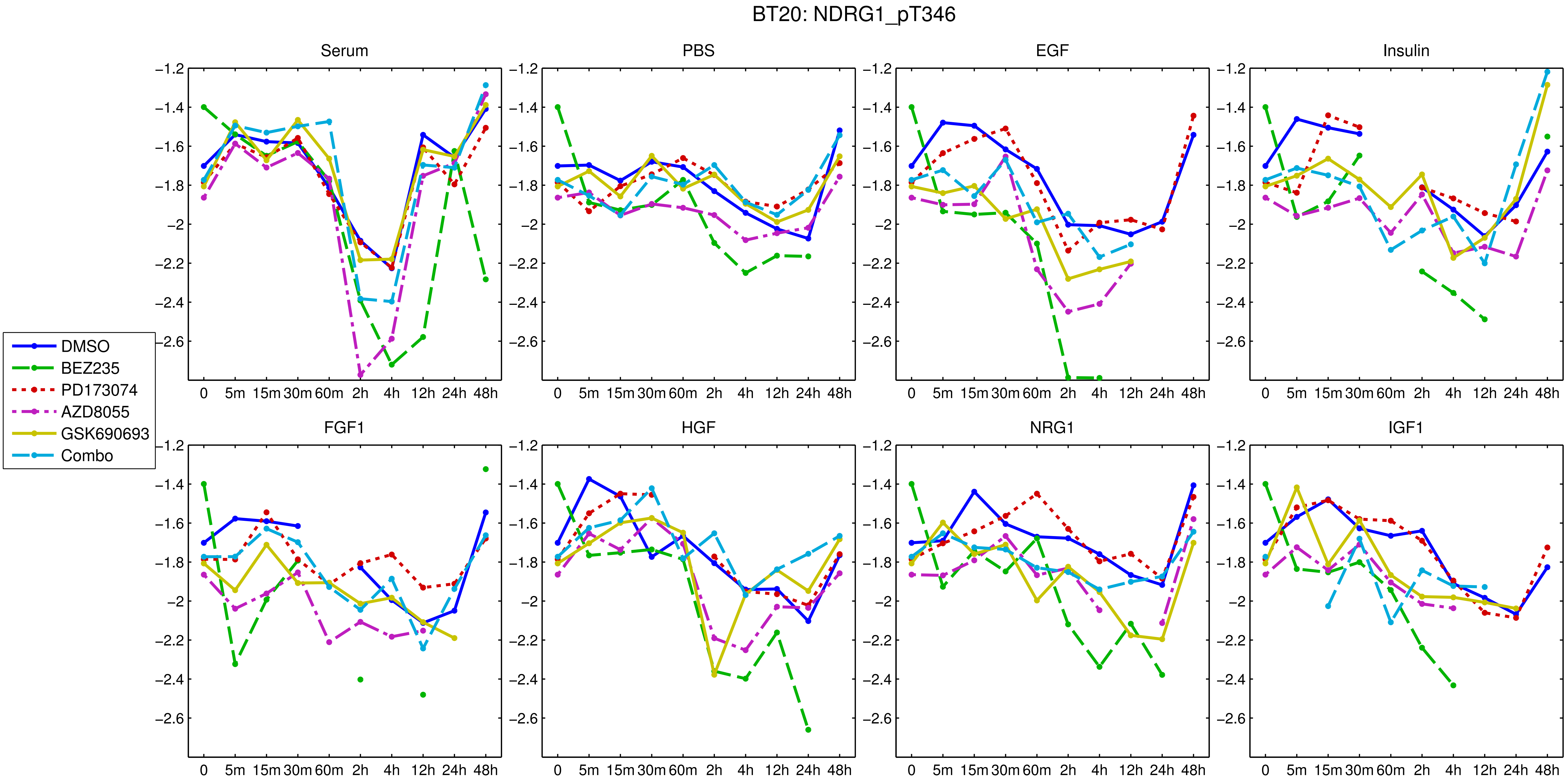

BT20: NDRG1_pT346
Serum
PBS
EGF
Insulin
FGF1
HGF
NRG1
IGF1
DMSO
BEZ235
PD173074
AZD8055
GSK690693
Combo
−1.2
−1.4
−1.6
−1.8
−2
−2.2
−2.4
−2.6
0
5m
15m
30m
60m
2h
4h
12h
24h
48h

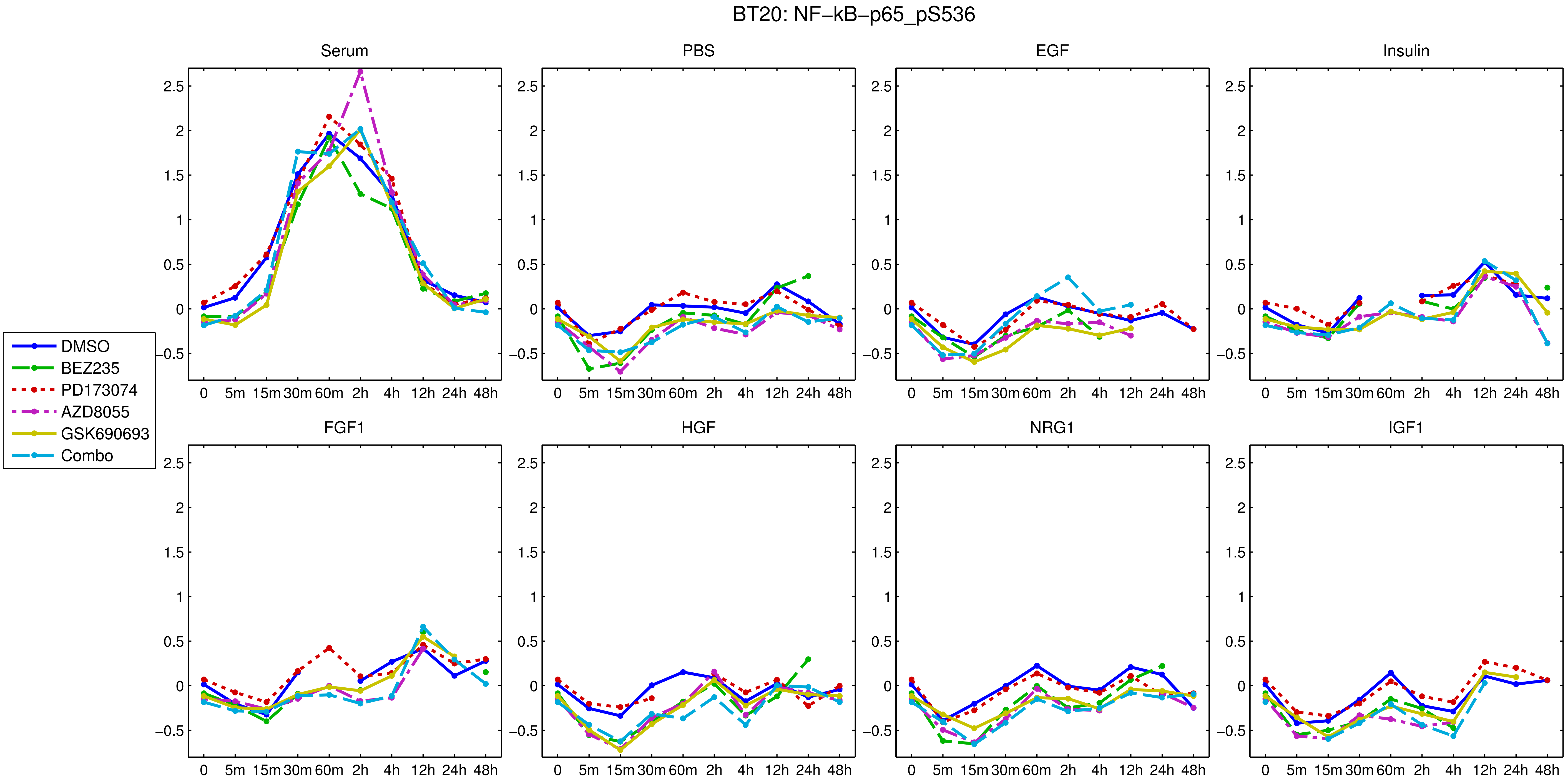
BT20: NF−kB−p65_pS536
Serum
PBS
EGF
Insulin
FGF1
HGF
NRG1
IGF1
DMSO
BEZ235
PD173074
AZD8055
GSK690693
Combo
0
5m
15m
30m
60m
2h
4h
12h
24h
48h
2.5
2
1.5
1
0.5
0
−0.5

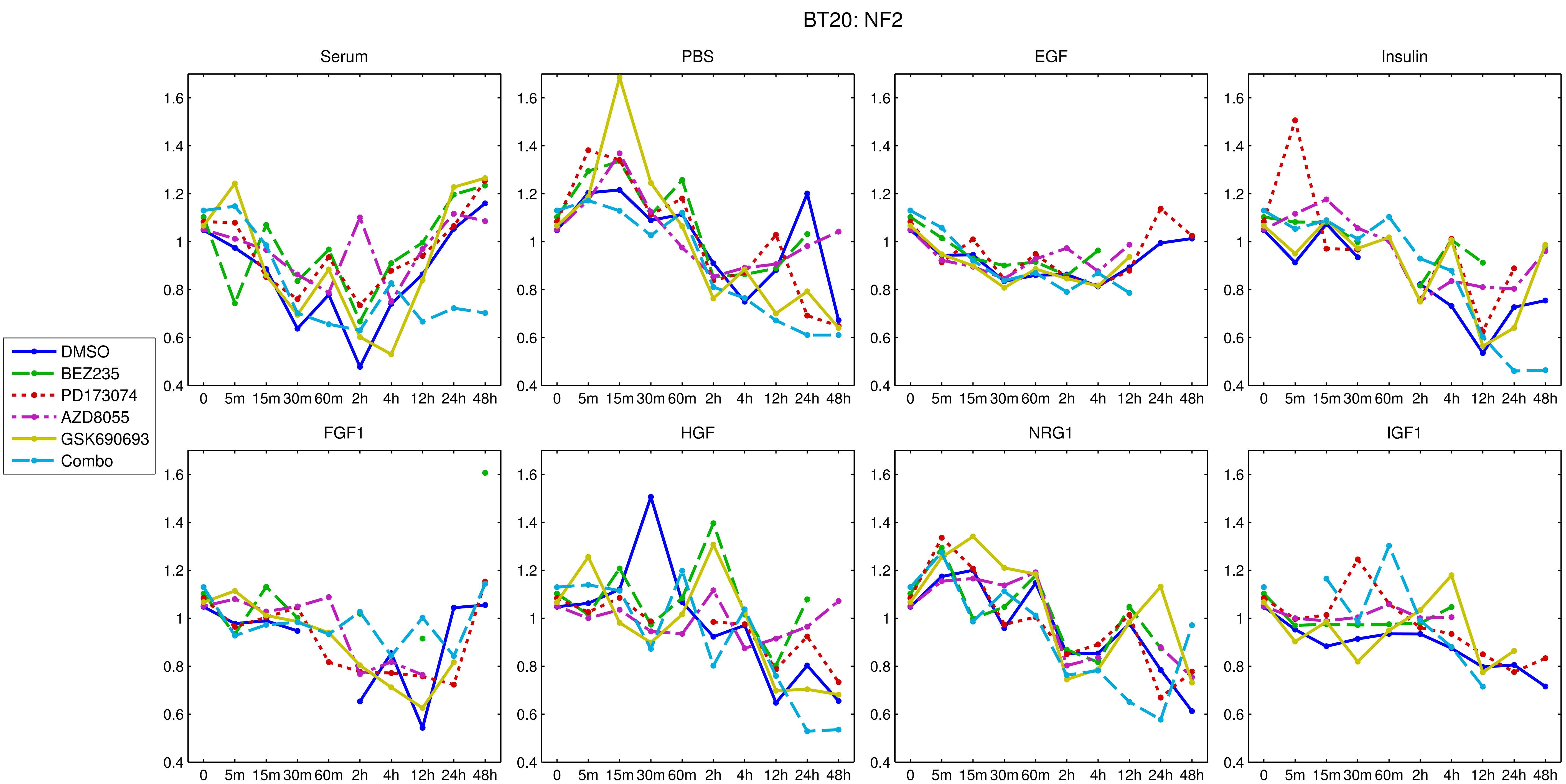
BT20: NF2
Serum
PBS
EGF
Insulin
FGF1
HGF
NRG1
IGF1
DMSO
BEZ235
PD173074
AZD8055
GSK690693
Combo
0.4
0.6
0.8
1
1.2
1.4
1.6
0
5m
15m
30m
60m
2h
4h
12h
24h
48h

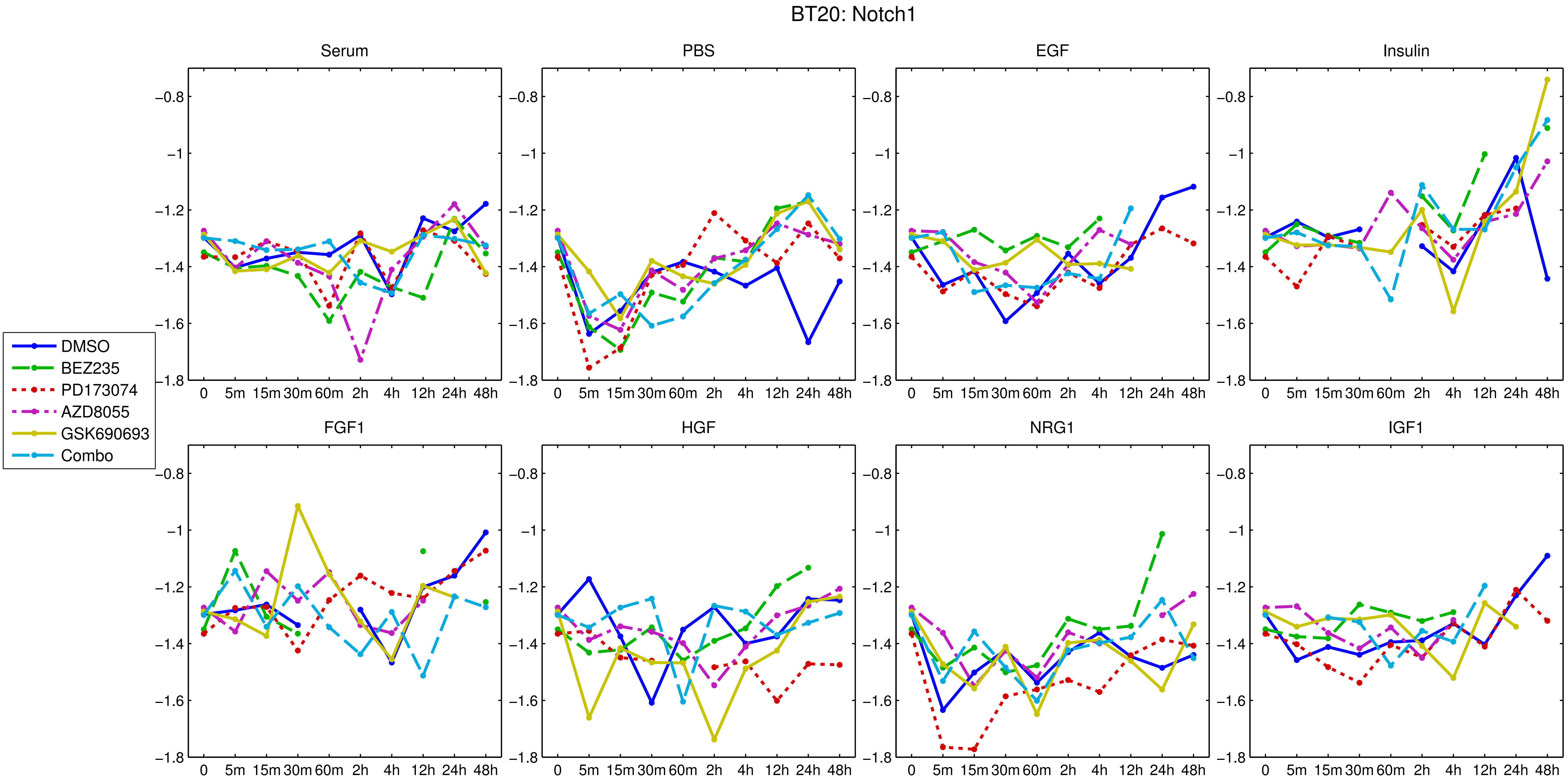

BT20: Notch1
Serum
PBS
EGF
Insulin
FGF1
HGF
NRG1
IGF1
DMSO
BEZ235
PD173074
AZD8055
GSK690693
Combo
−0.8
−1
−1.2
−1.4
−1.6
−1.8
0
5m
15m
30m
60m
2h
4h
12h
24h
48h

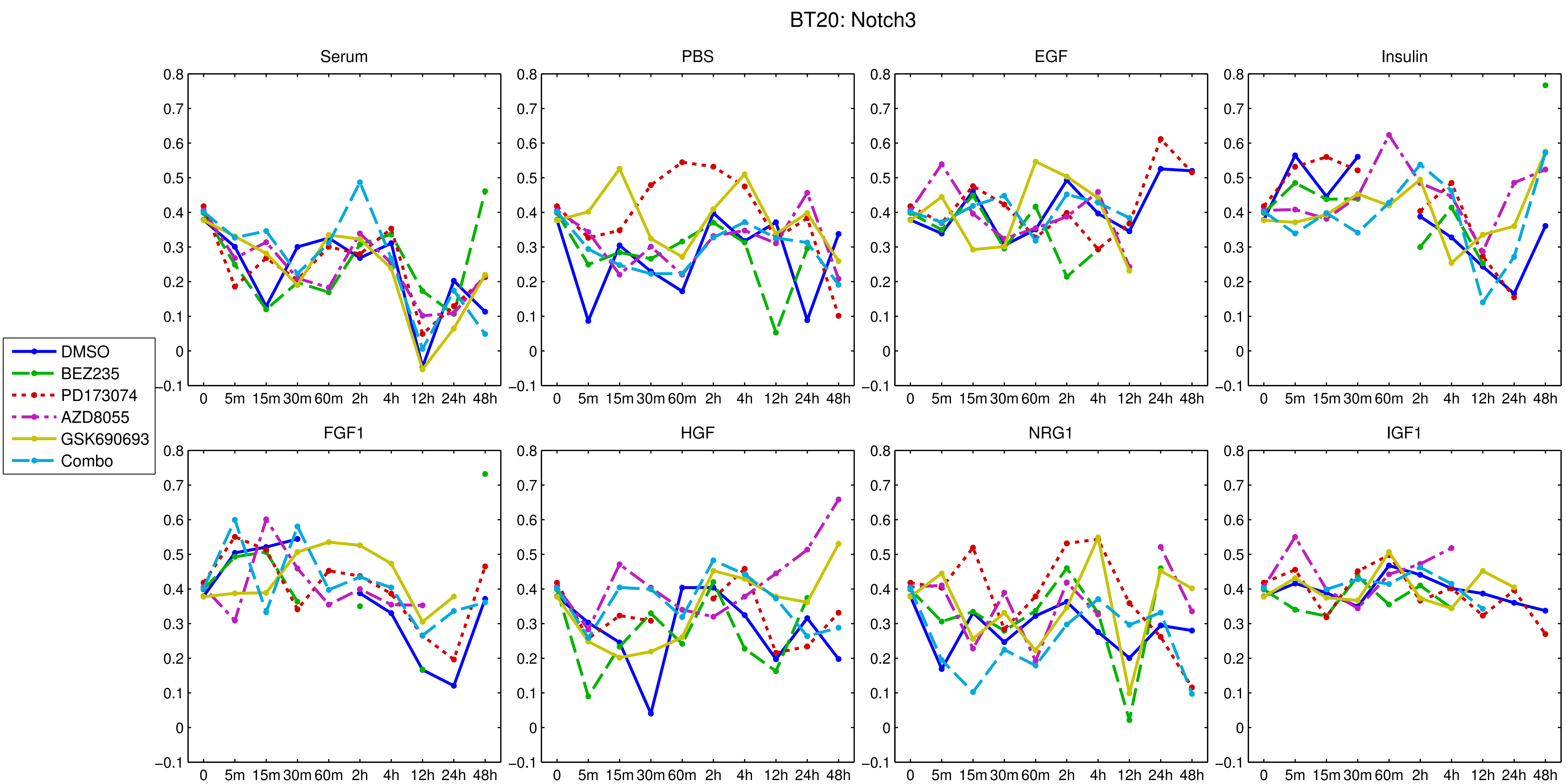

BT20: Notch3
Serum
PBS
EGF
Insulin
FGF1
HGF
NRG1
IGF1
DMSO
BEZ235
PD173074
AZD8055
GSK690693
Combo
0.8
0.7
0.6
0.5
0.4
0.3
0.2
0.1
0
−0.1
0
5m
15m
30m
60m
2h
4h
12h
24h
48h

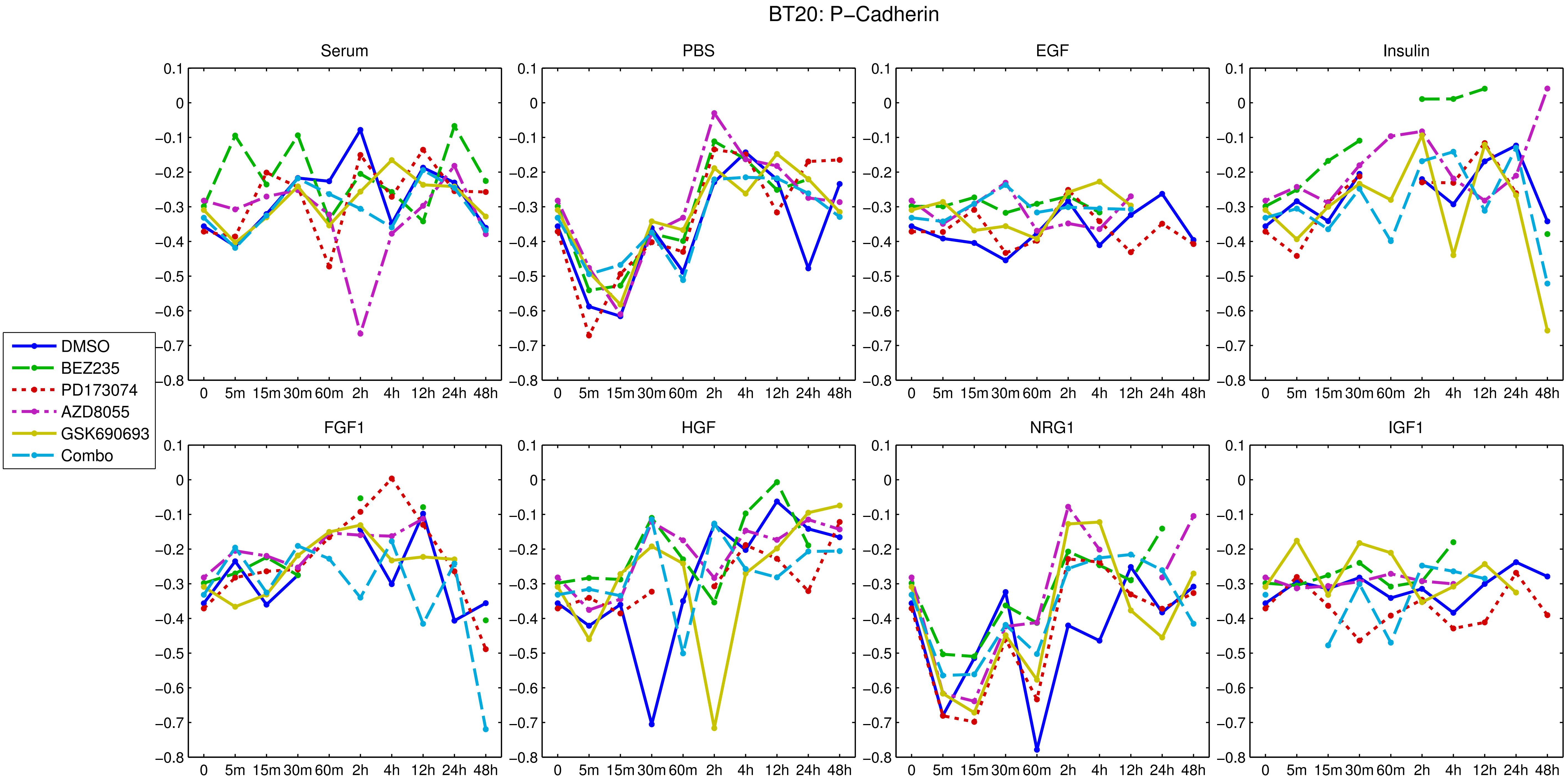

BT20: P−Cadherin
Serum
PBS
EGF
Insulin
FGF1
HGF
NRG1
IGF1
DMSO
BEZ235
PD173074
AZD8055
GSK690693
Combo
0.1
0
−0.1
−0.2
−0.3
−0.4
−0.5
−0.6
−0.7
−0.8
0
5m
15m
30m
60m
2h
4h
12h
24h
48h

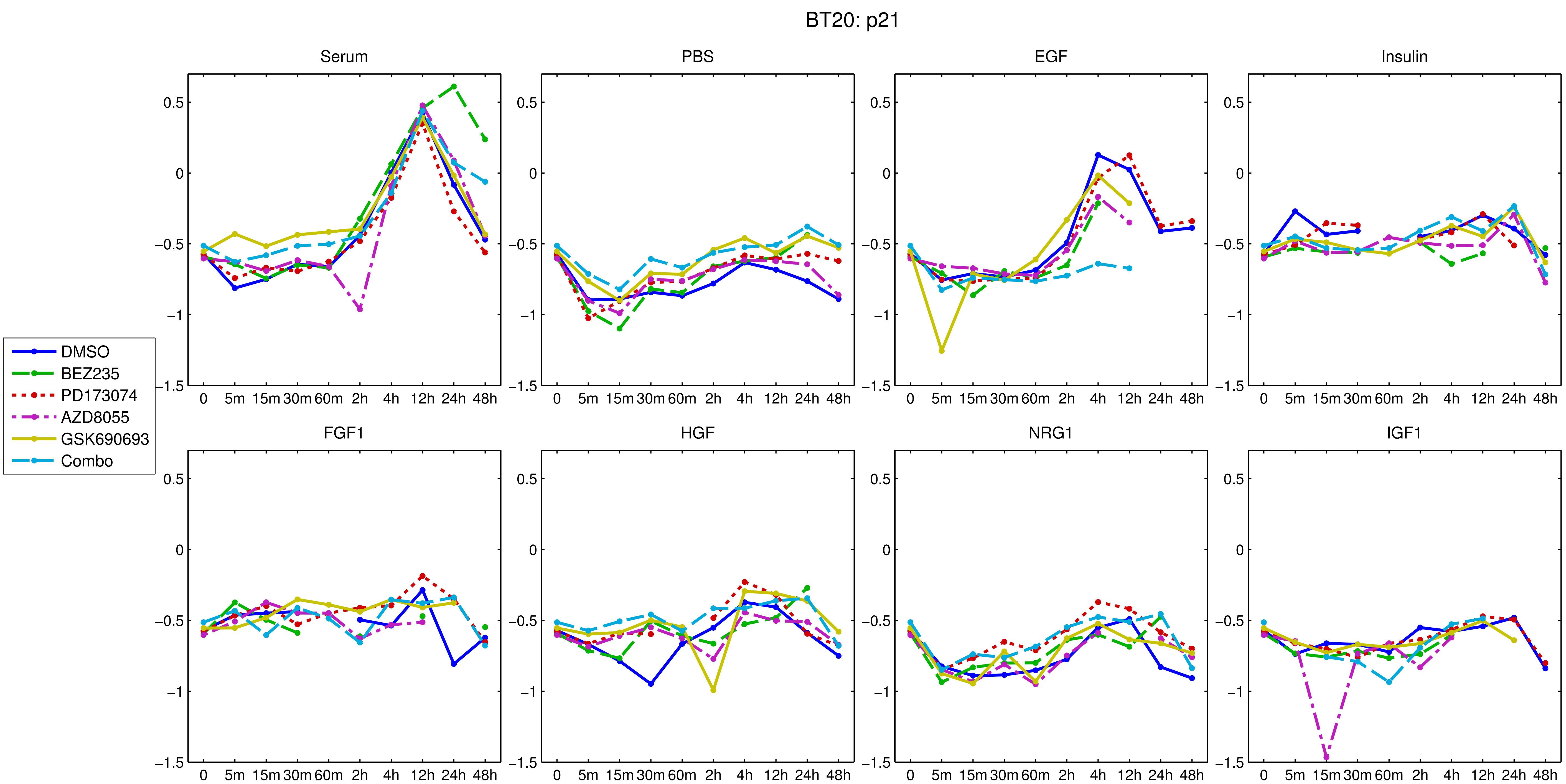
BT20: p21
Serum
PBS
EGF
Insulin
FGF1
HGF
NRG1
IGF1
DMSO
BEZ235
PD173074
AZD8055
GSK690693
Combo
0.5
0
−0.5
−1
−1.5
0 5m 15m 30m 60m 2h 4h 12h 24h 48h

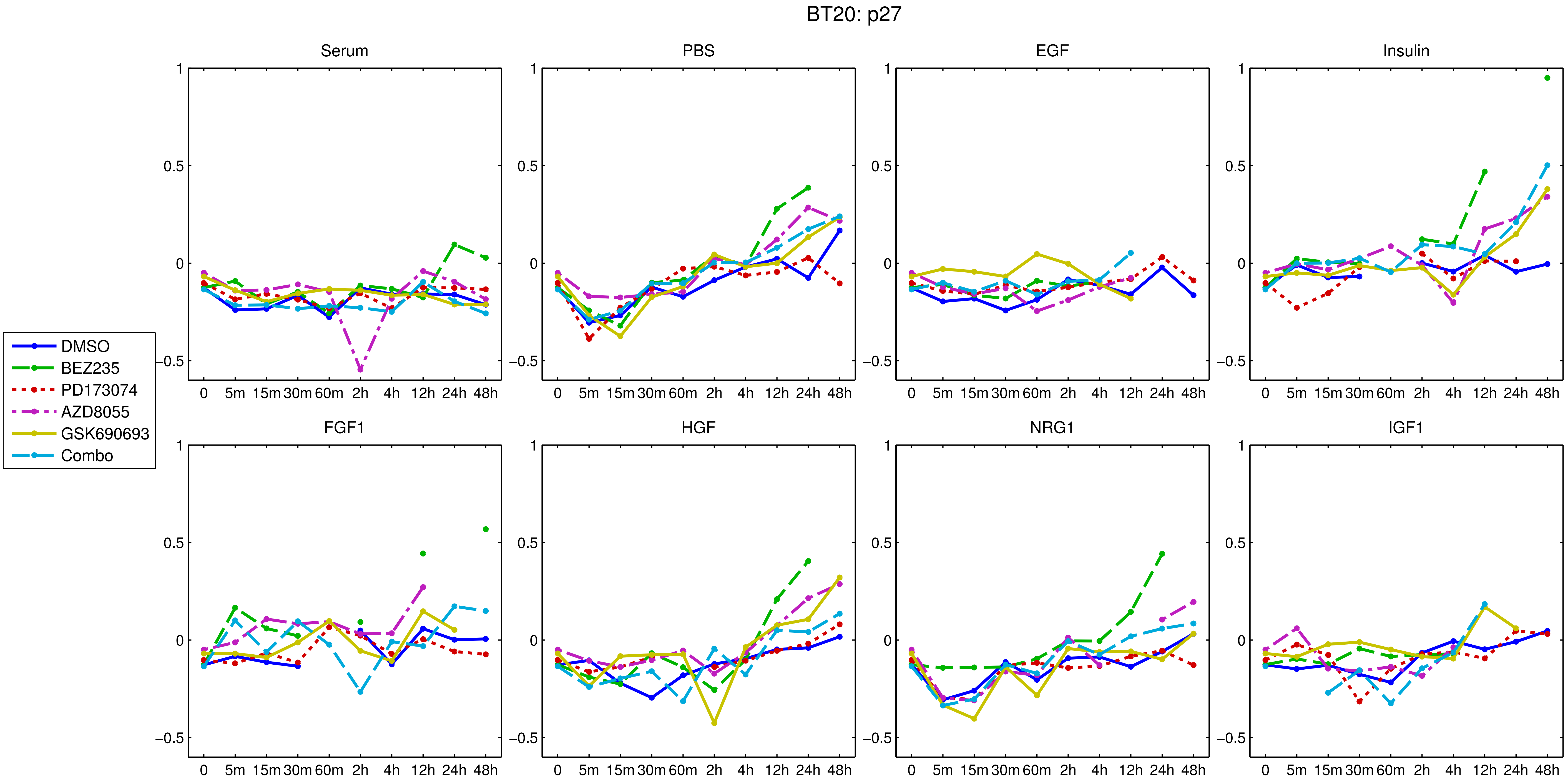
BT20: p27
Serum
PBS
EGF
Insulin
FGF1
HGF
NRG1
IGF1
DMSO
BEZ235
PD173074
AZD8055
GSK690693
Combo
1
0.5
0
−0.5
0
5m
15m
30m
60m
2h
4h
12h
24h
48h

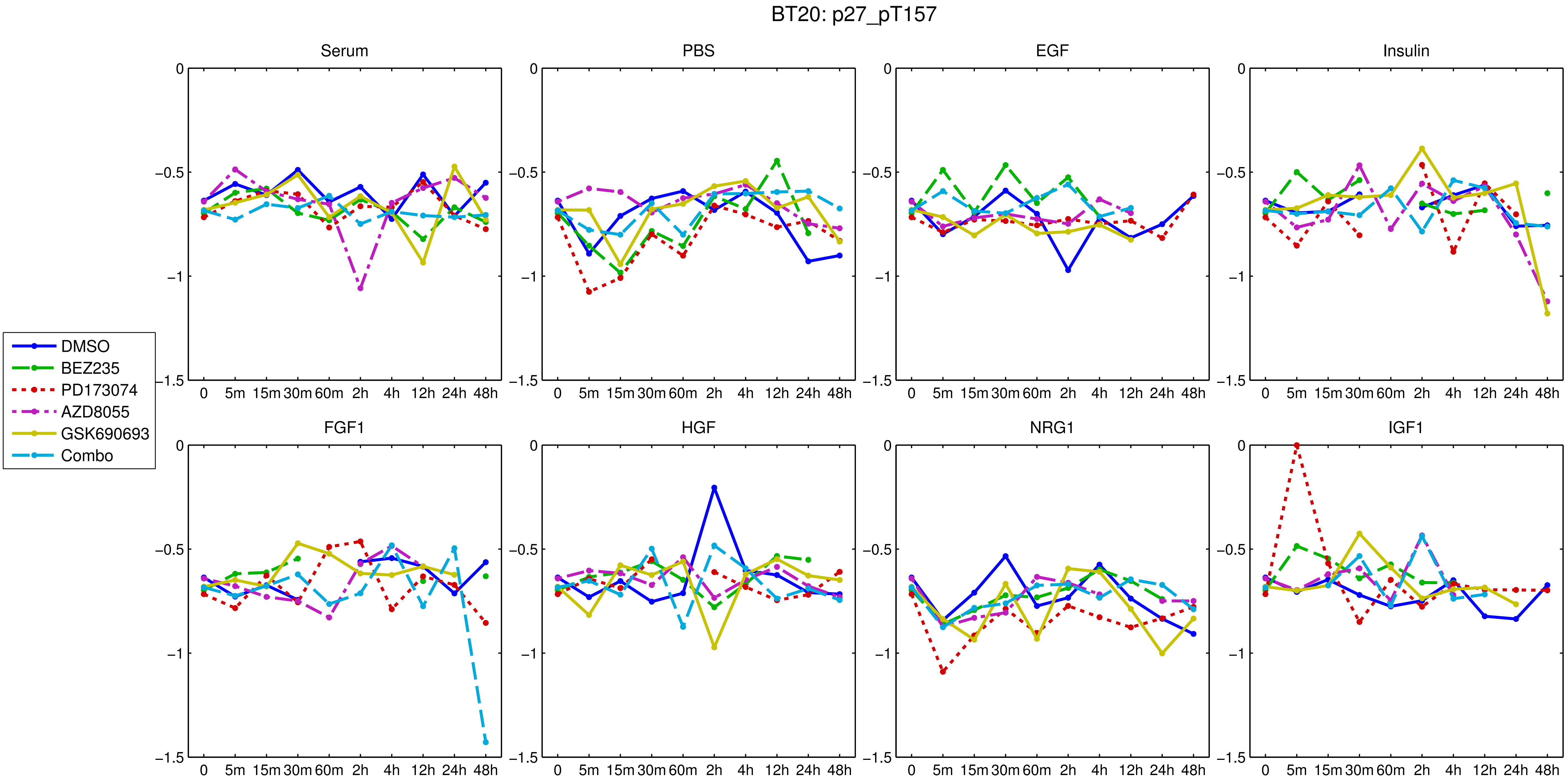

BT20: p27_pT157
Serum
PBS
EGF
Insulin
FGF1
HGF
NRG1
IGF1
0
−0.5
−1
−1.5
0 5m 15m 30m 60m 2h 4h 12h 24h 48h
DMSO
BEZ235
PD173074
AZD8055
GSK690693
Combo

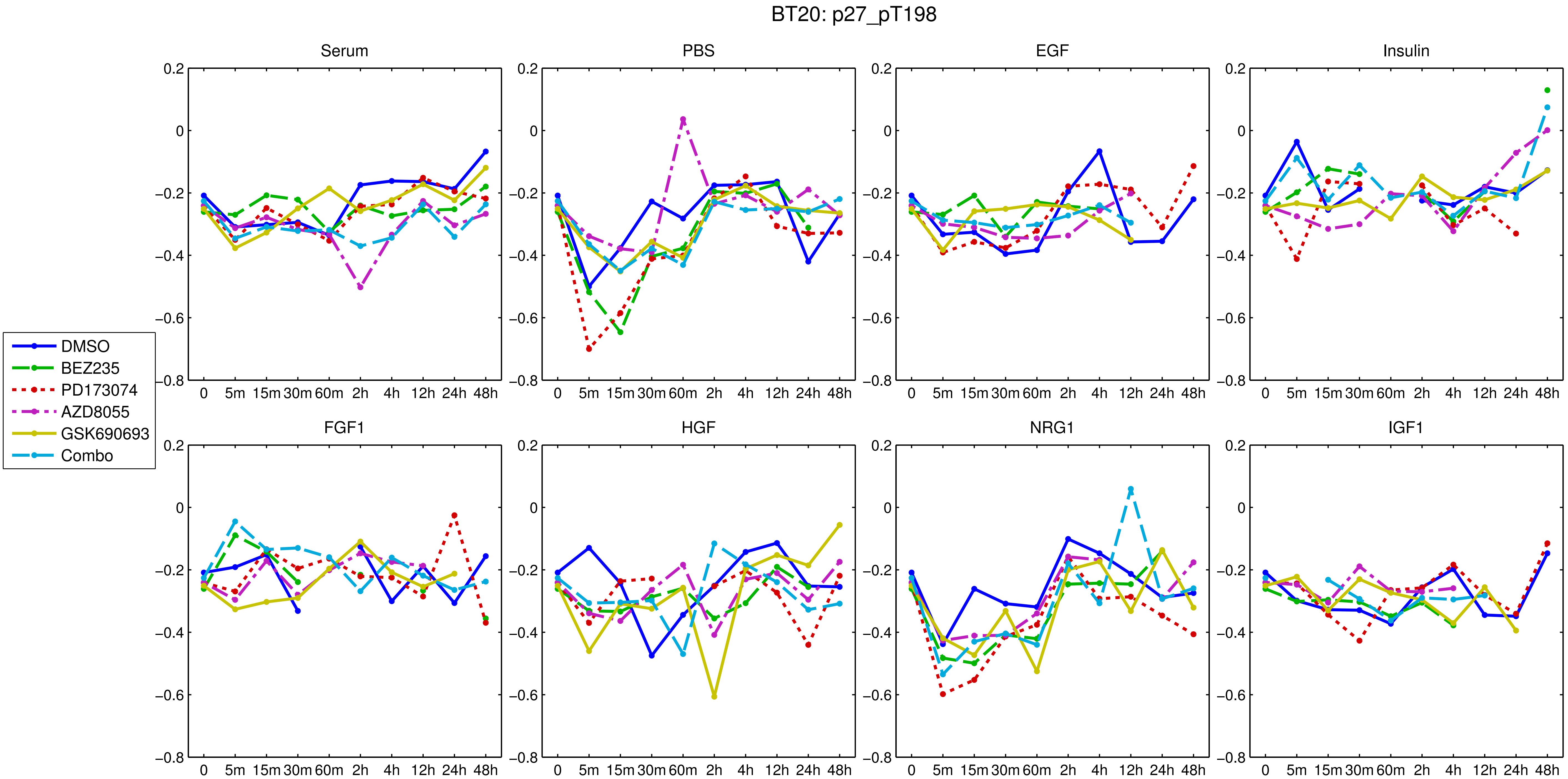
BT20: p27_pT198
Serum
PBS
EGF
Insulin
FGF1
HGF
NRG1
IGF1
0.2
0
−0.2
−0.4
−0.6
−0.8
0 5m 15m 30m 60m 2h 4h 12h 24h 48h
DMSO
BEZ235
PD173074
AZD8055
GSK690693
Combo

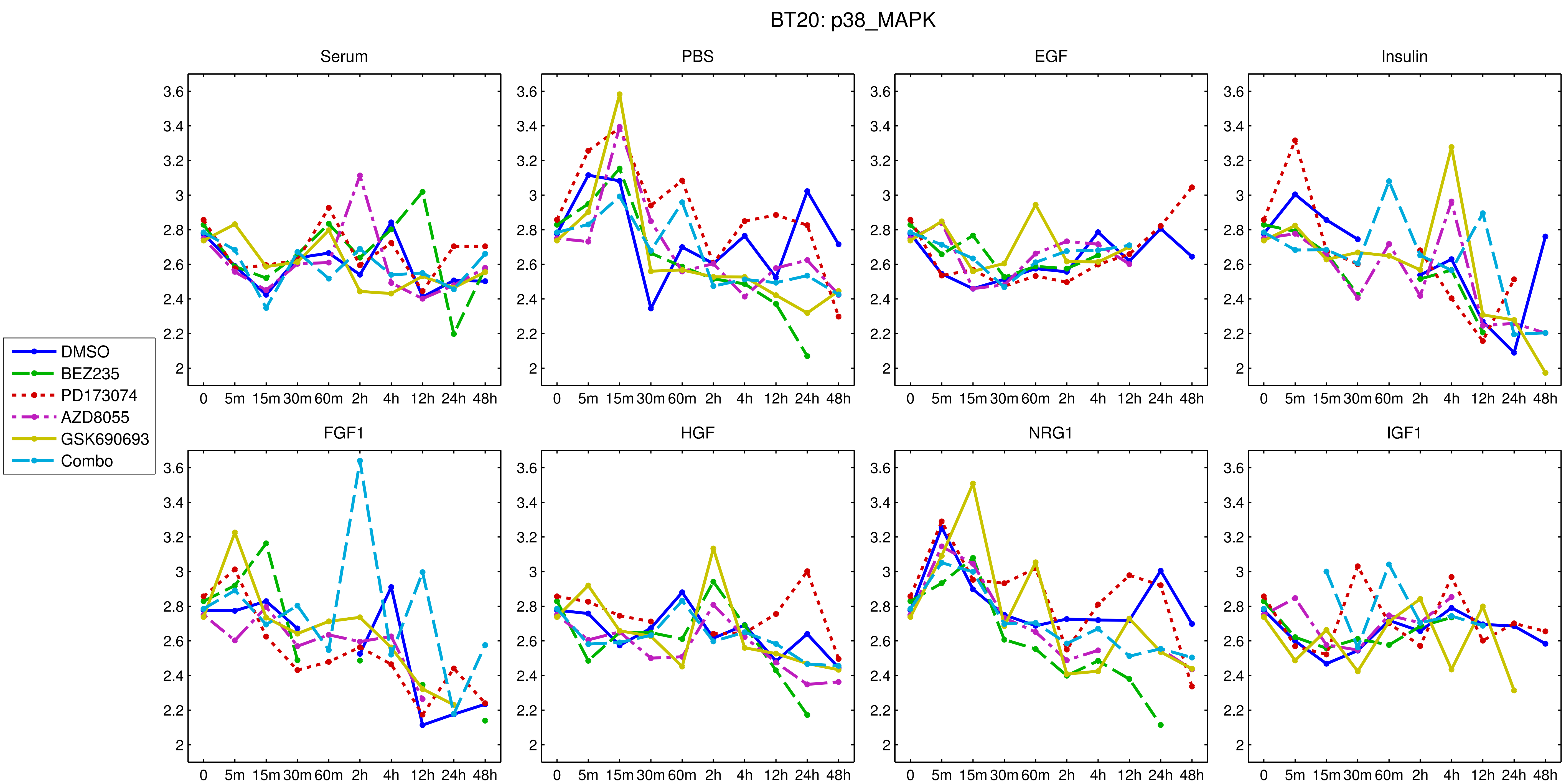

BT20: p38_MAPK
Serum
PBS
EGF
Insulin
FGF1
HGF
NRG1
IGF1
DMSO
BEZ235
PD173074
AZD8055
GSK690693
Combo
3.6
3.4
3.2
3
2.8
2.6
2.4
2.2
2
0
5m
15m
30m
60m
2h
4h
12h
24h
48h

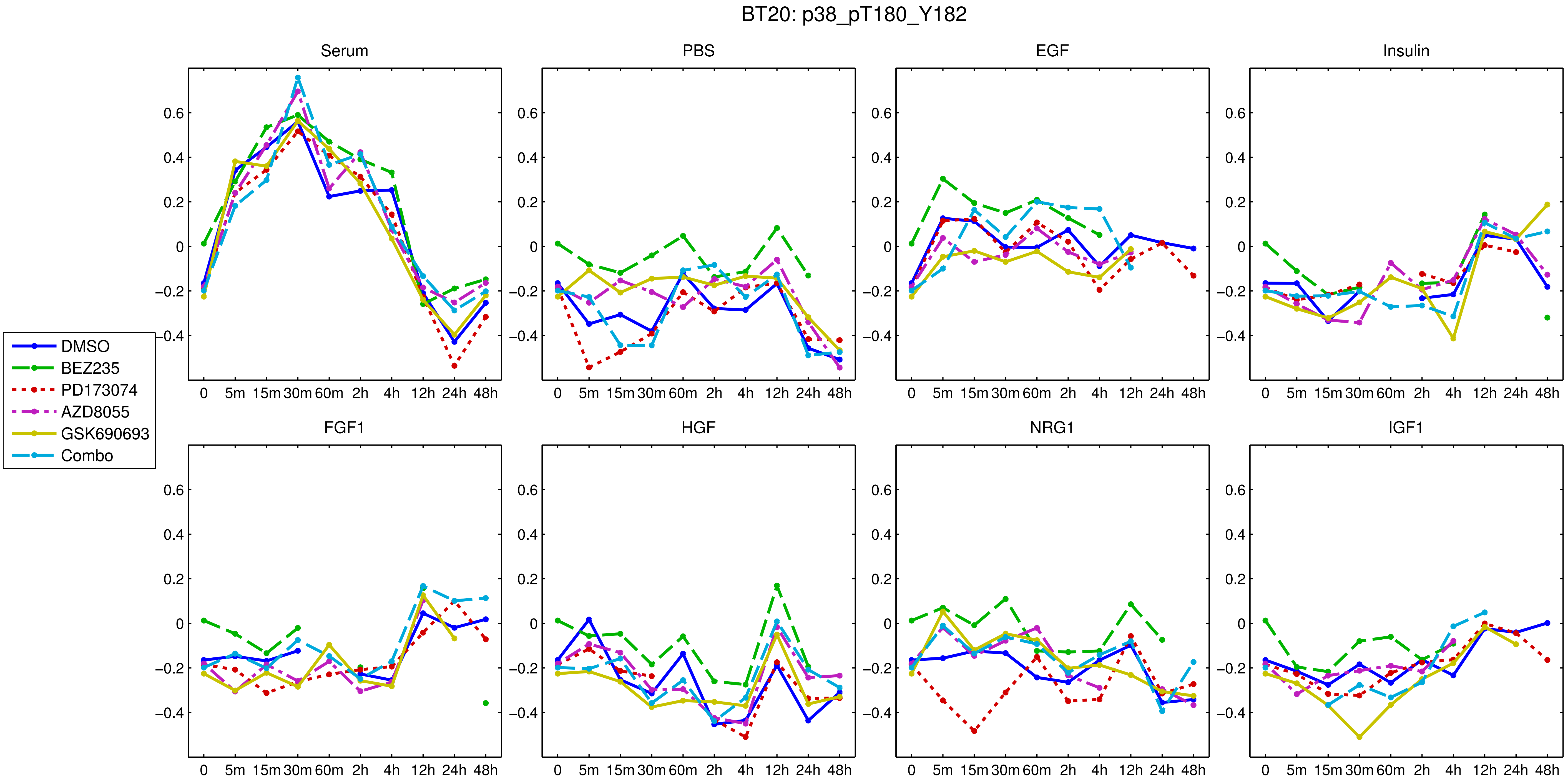

BT20: p38_pT180_Y182
Serum
PBS
EGF
Insulin
FGF1
HGF
NRG1
IGF1
DMSO
BEZ235
PD173074
AZD8055
GSK690693
Combo
0.6
0.4
0.2
0
−0.2
−0.4
0 5m 15m 30m 60m 2h 4h 12h 24h 48h

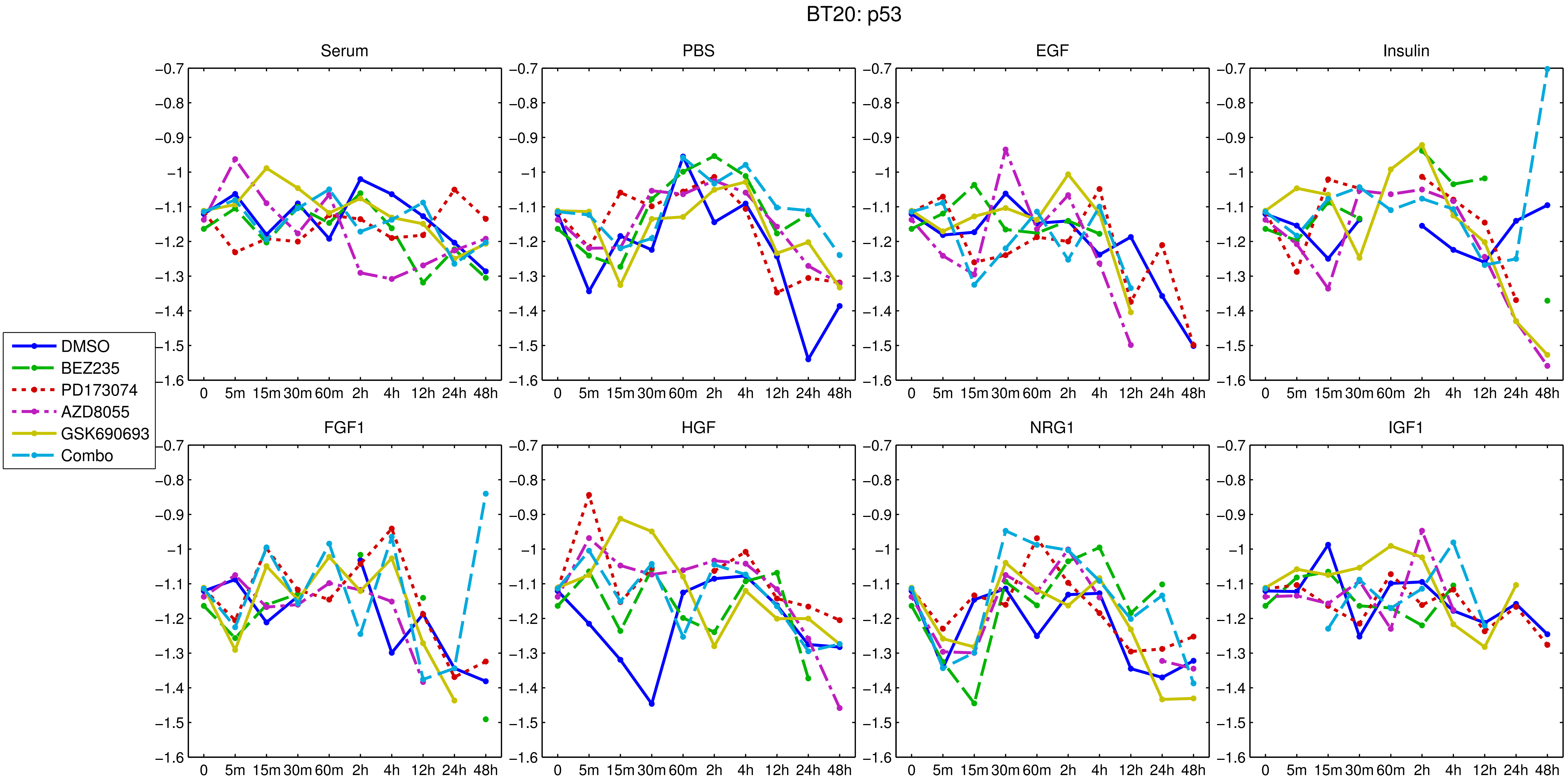

BT20: p53
Serum
PBS
EGF
Insulin
FGF1
HGF
NRG1
IGF1
−0.7
−0.8
−0.9
−1
−1.1
−1.2
−1.3
−1.4
−1.5
−1.6
0 5m 15m 30m 60m 2h 4h 12h 24h 48h
DMSO
BEZ235
PD173074
AZD8055
GSK690693
Combo

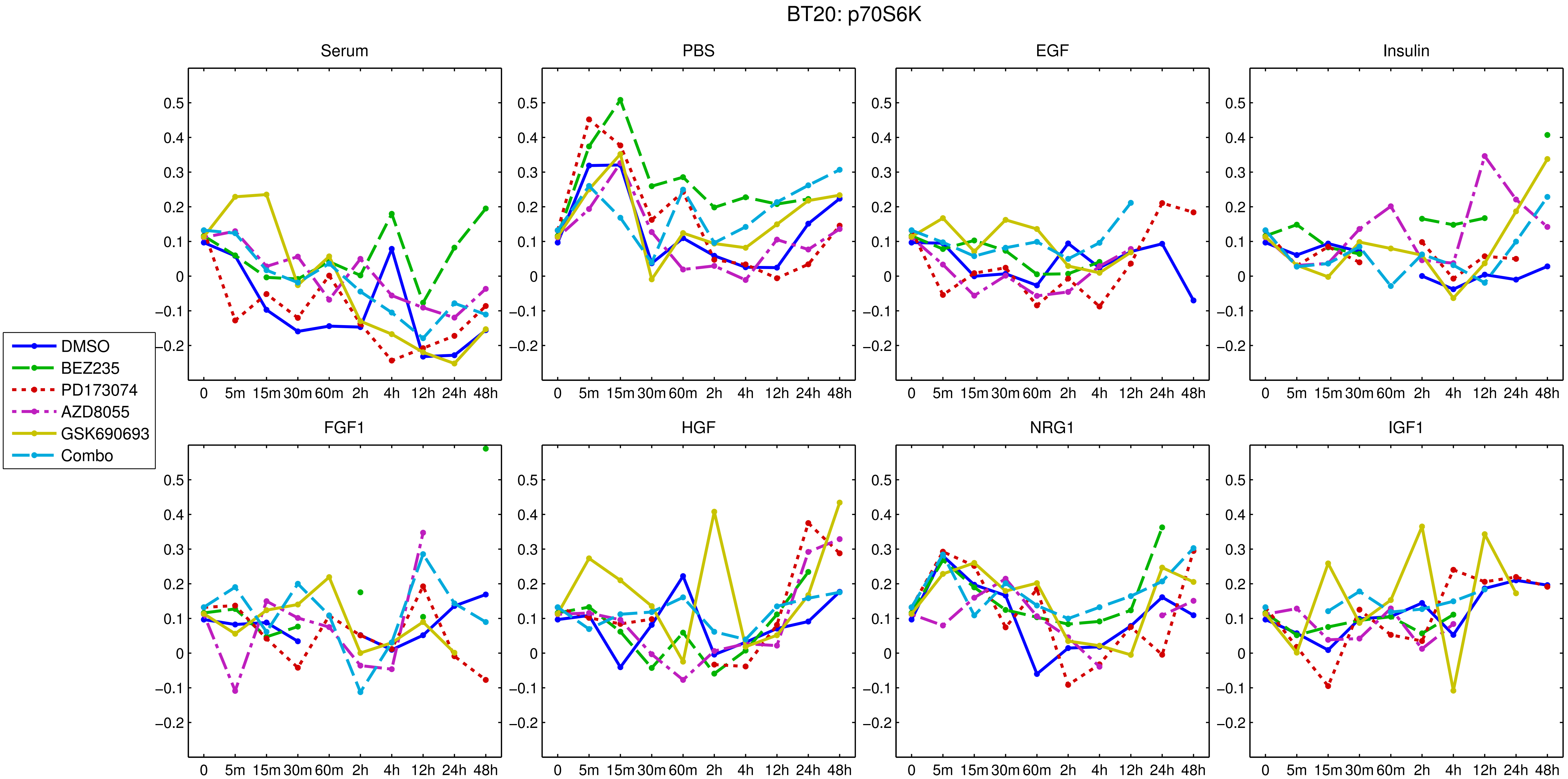
BT20: p70S6K
Serum
PBS
EGF
Insulin
FGF1
HGF
NRG1
IGF1
DMSO
BEZ235
PD173074
AZD8055
GSK690693
Combo
0.5
0.4
0.3
0.2
0.1
0
−0.1
−0.2
0
5m
15m
30m
60m
2h
4h
12h
24h
48h

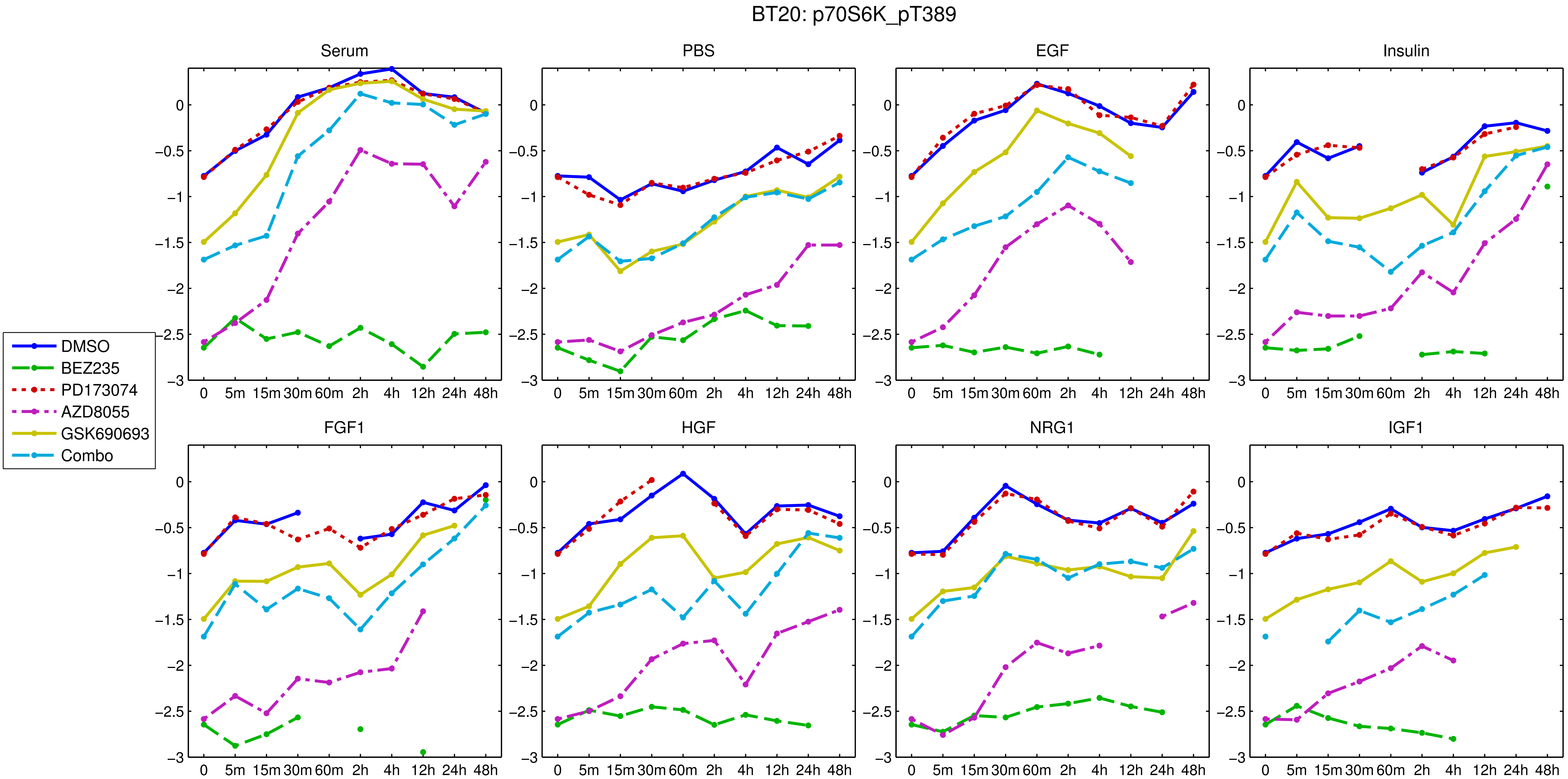

BT20: p70S6K_pT389
Serum
PBS
EGF
Insulin
FGF1
HGF
NRG1
IGF1
DMSO
BEZ235
PD173074
AZD8055
GSK690693
Combo
0
−0.5
−1
−1.5
−2
−2.5
−3
0 5m 15m 30m 60m 2h 4h 12h 24h 48h

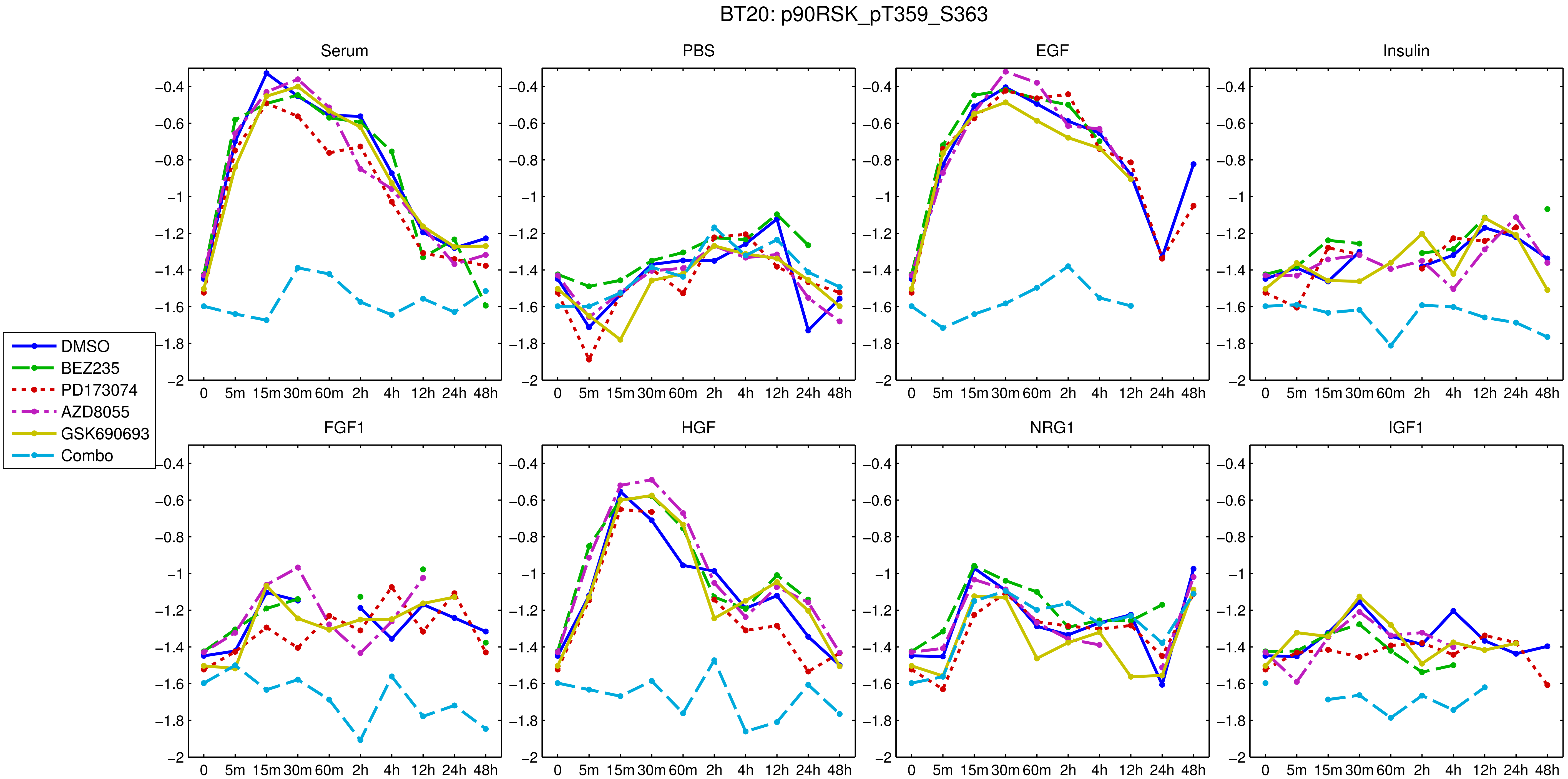
BT20: p90RSK_pT359_S363
Serum
PBS
EGF
Insulin
FGF1
HGF
NRG1
IGF1
DMSO
BEZ235
PD173074
AZD8055
GSK690693
Combo
−0.4
−0.6
−0.8
−1
−1.2
−1.4
−1.6
−1.8
−2
0
5m
15m
30m
60m
2h
4h
12h
24h
48h

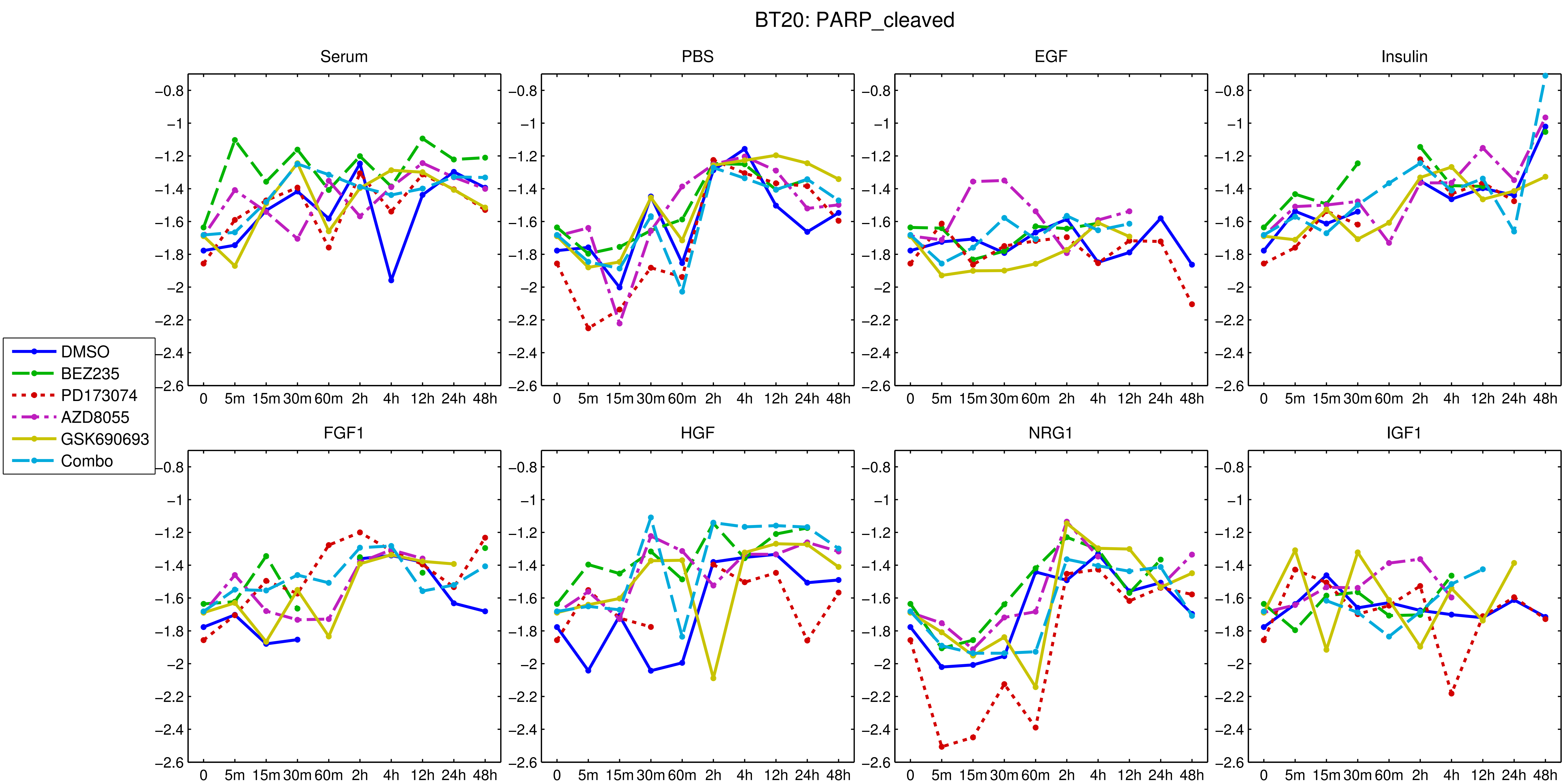

BT20: PARP_cleaved
Serum
PBS
EGF
Insulin
FGF1
HGF
NRG1
IGF1
DMSO
BEZ235
PD173074
AZD8055
GSK690693
Combo
−0.8
−1
−1.2
−1.4
−1.6
−1.8
−2
−2.2
−2.4
−2.6
0
5m
15m
30m
60m
2h
4h
12h
24h
48h

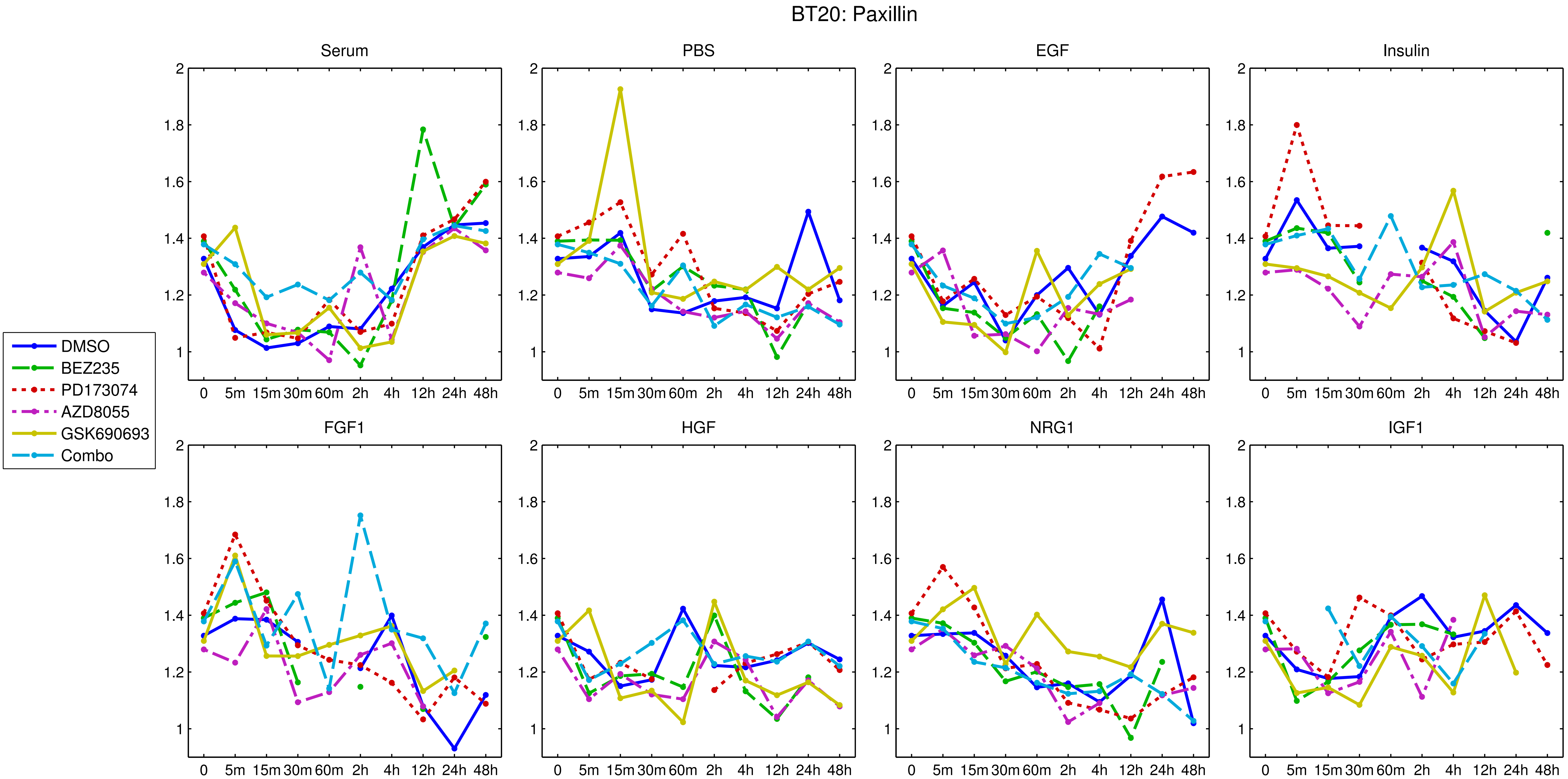
BT20: Paxillin
Serum
PBS
EGF
Insulin
FGF1
HGF
NRG1
IGF1
DMSO
BEZ235
PD173074
AZD8055
GSK690693
Combo
2
1.8
1.6
1.4
1.2
1
0
5m
15m
30m
60m
2h
4h
12h
24h
48h

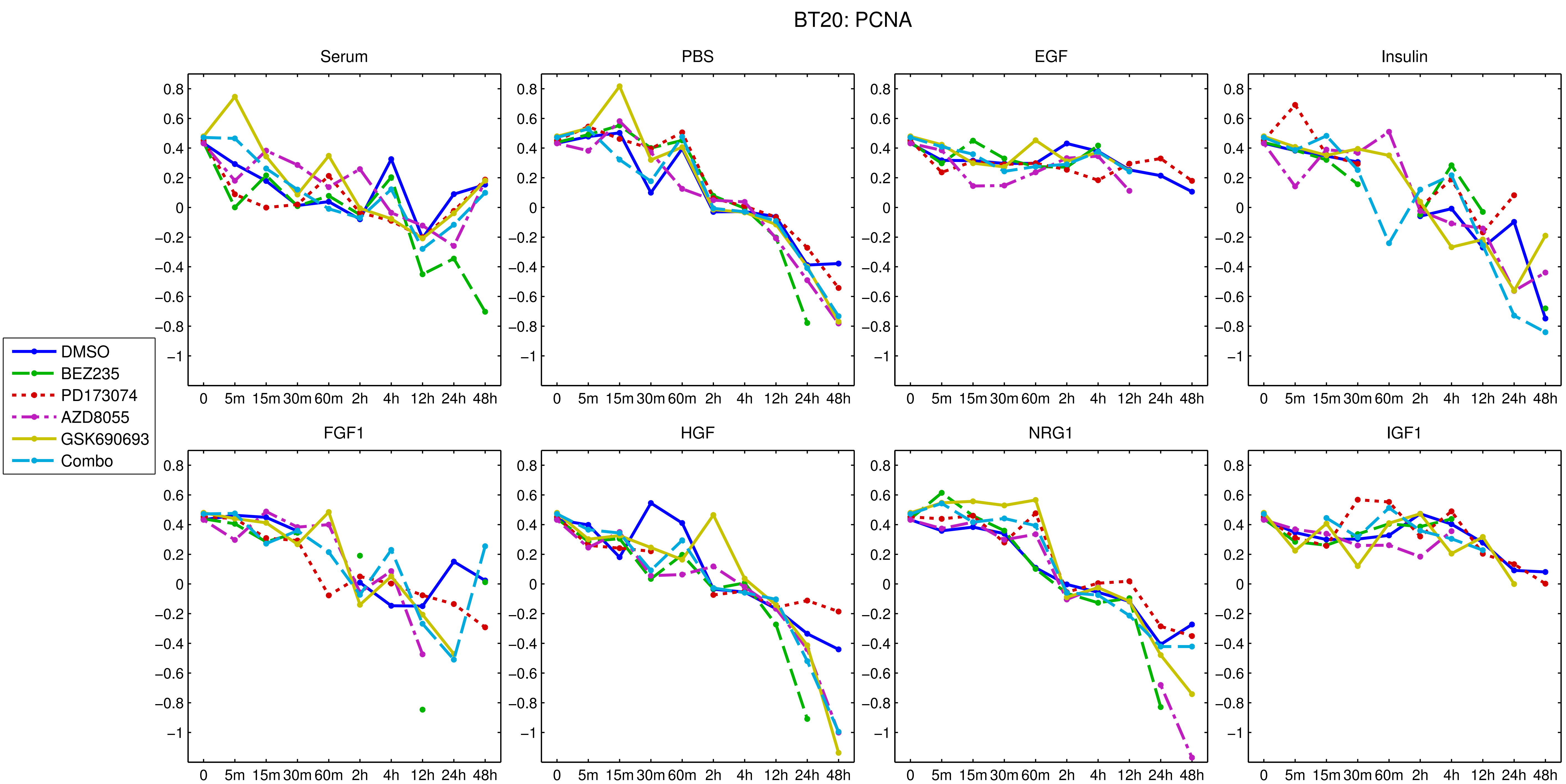
BT20: PCNA
Serum
PBS
EGF
Insulin
FGF1
HGF
NRG1
IGF1
DMSO
BEZ235
PD173074
AZD8055
GSK690693
Combo
0.8
0.6
0.4
0.2
0
−0.2
−0.4
−0.6
−0.8
−1
0
5m
15m
30m
60m
2h
4h
12h
24h
48h

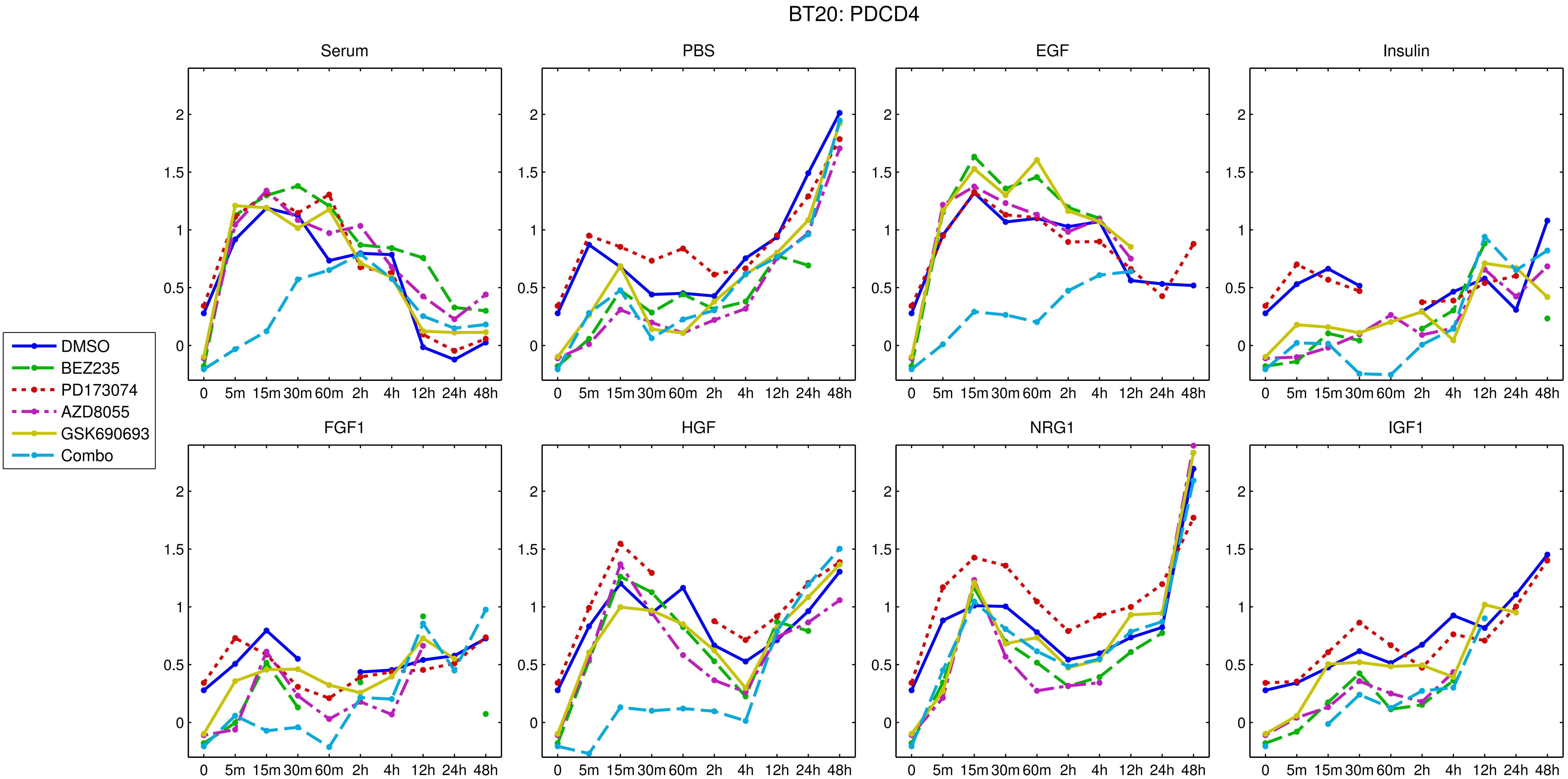
BT20: PDCD4
Serum
PBS
EGF
Insulin
FGF1
HGF
NRG1
IGF1
DMSO
BEZ235
PD173074
AZD8055
GSK690693
Combo
0
5m
15m
30m
60m
2h
4h
12h
24h
48h
0
0.5
1
1.5
2

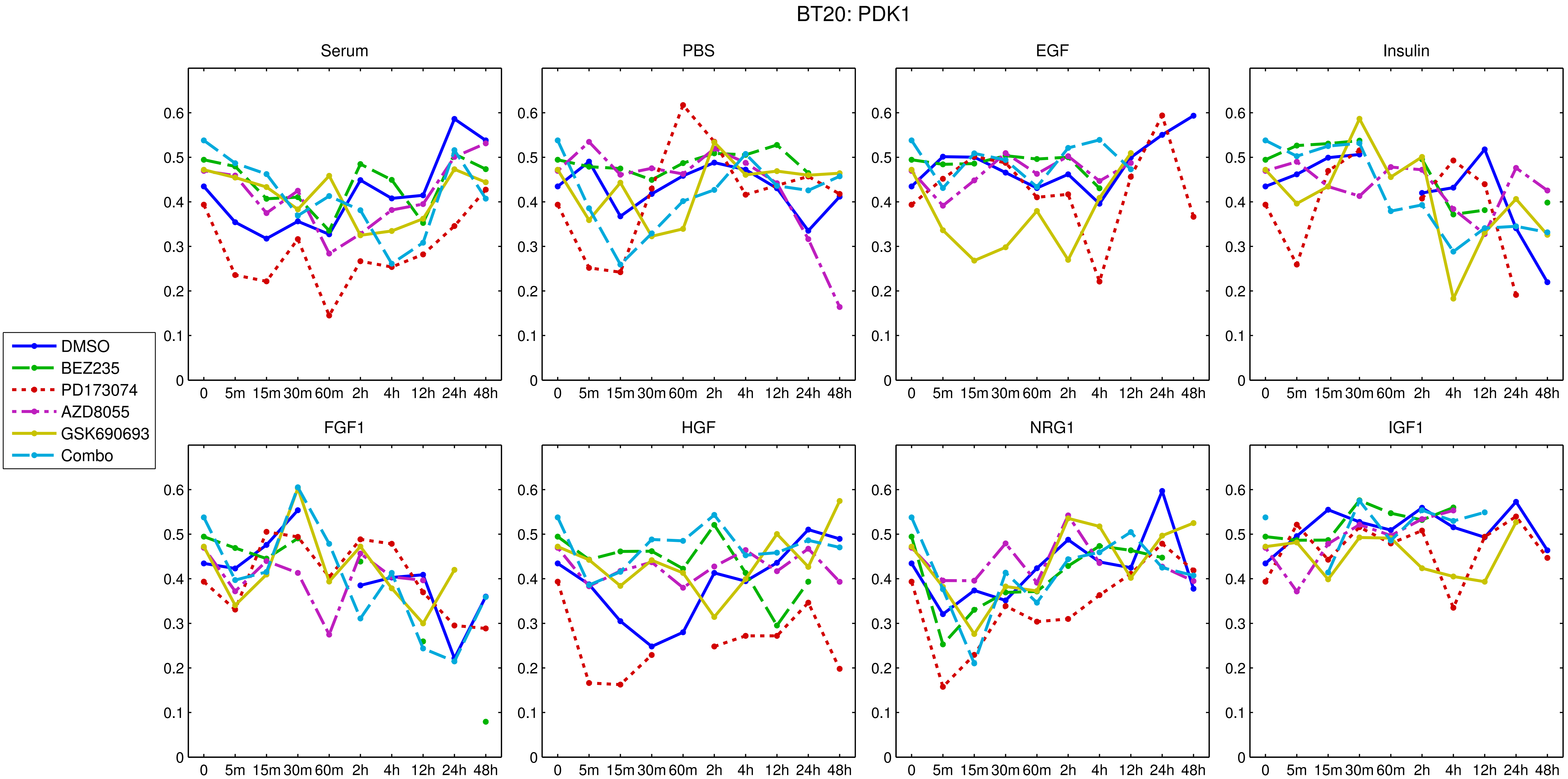
BT20: PDK1
Serum
PBS
EGF
Insulin
FGF1
HGF
NRG1
IGF1
DMSO
BEZ235
PD173074
AZD8055
GSK690693
Combo
0
0.1
0.2
0.3
0.4
0.5
0.6
0
5m
15m
30m
60m
2h
4h
12h
24h
48h

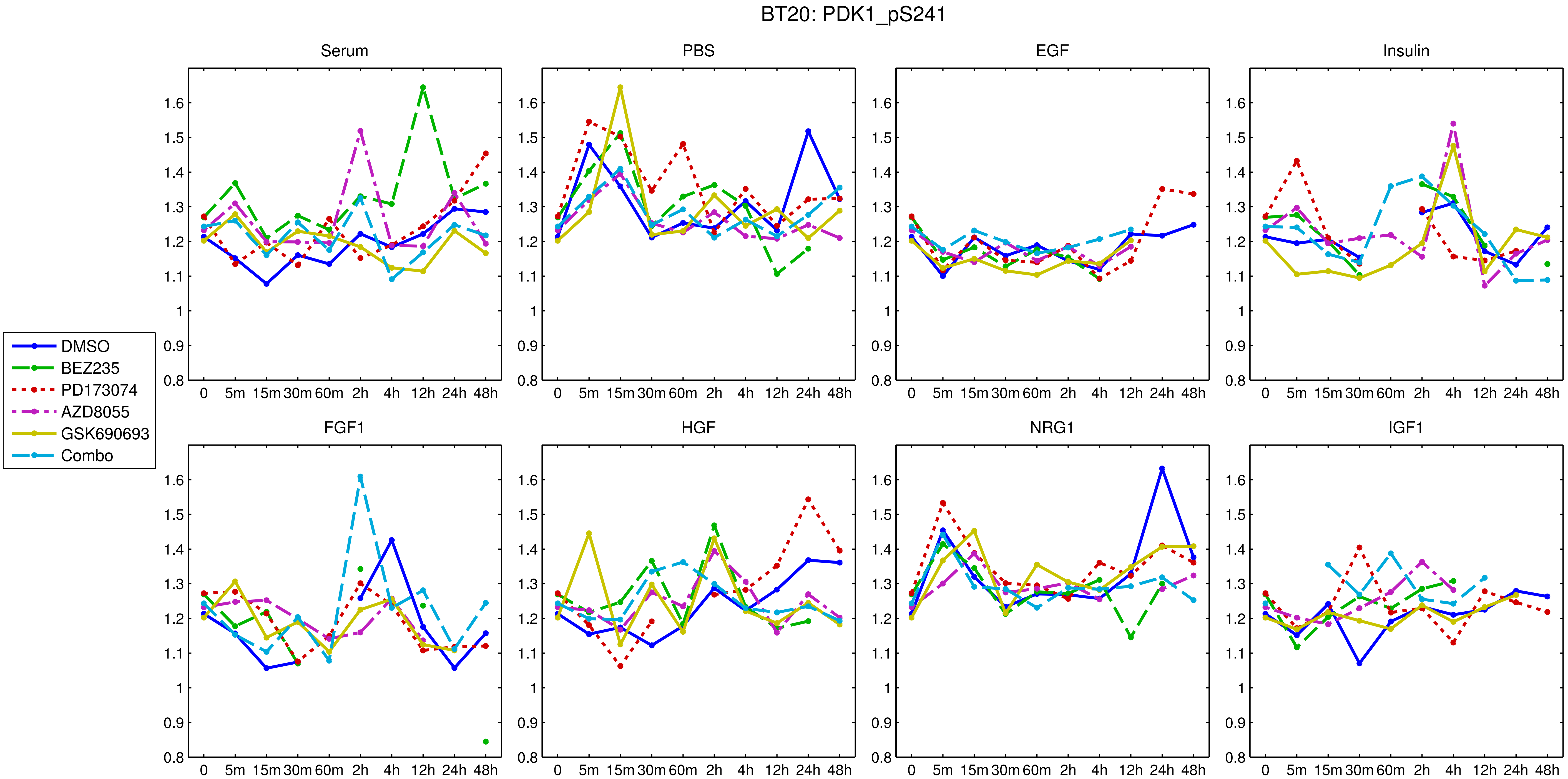
BT20: PDK1_pS241
Serum
PBS
EGF
Insulin
FGF1
HGF
NRG1
IGF1
DMSO
BEZ235
PD173074
AZD8055
GSK690693
Combo
0.8
0.9
1
1.1
1.2
1.3
1.4
1.5
1.6
0
5m
15m
30m
60m
2h
4h
12h
24h
48h

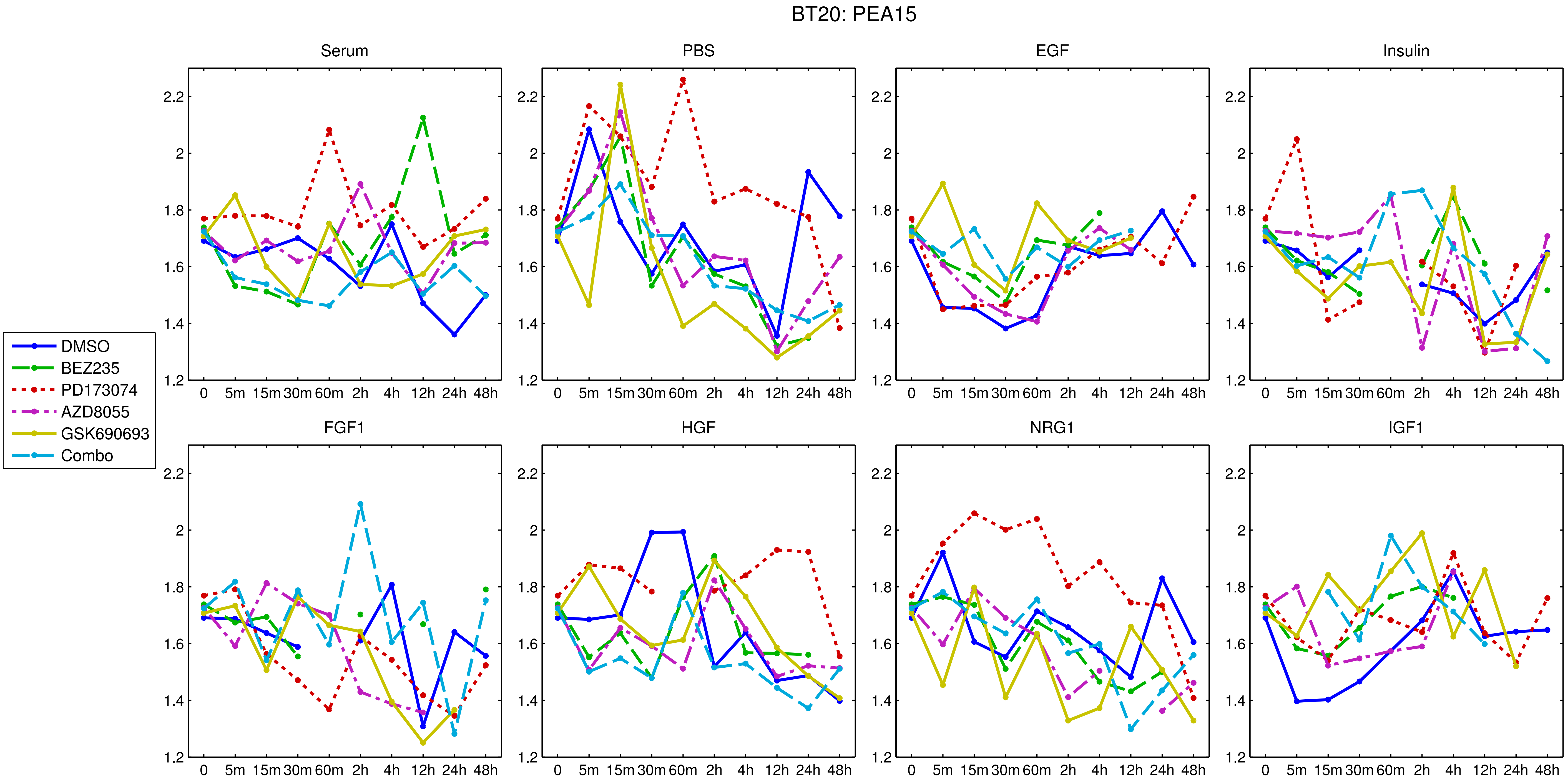

BT20: PEA15
Serum
PBS
EGF
Insulin
FGF1
HGF
NRG1
IGF1
DMSO
BEZ235
PD173074
AZD8055
GSK690693
Combo
2.2
2
1.8
1.6
1.4
1.2
0
5m
15m
30m
60m
2h
4h
12h
24h
48h

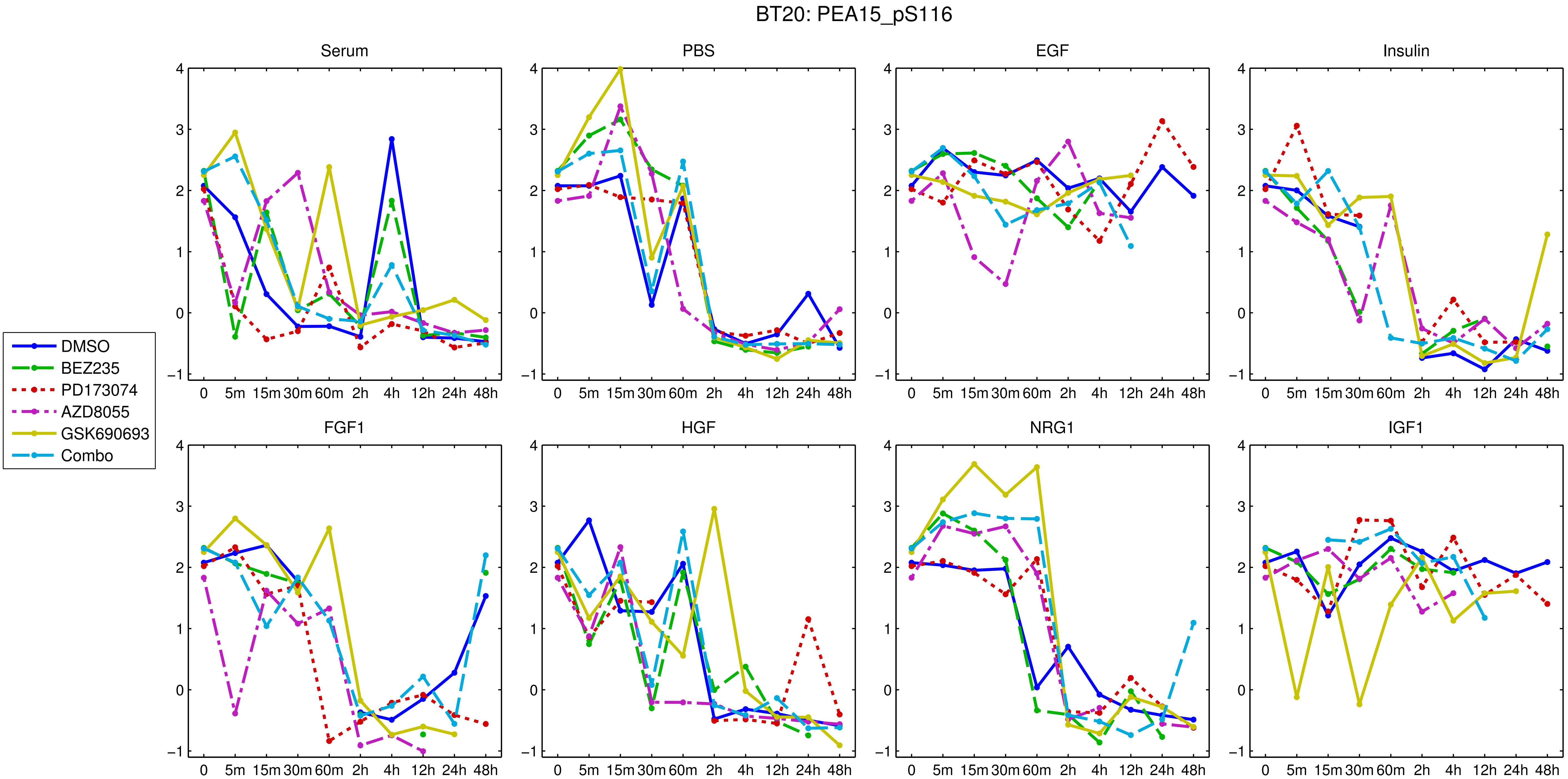
BT20: PEA15_pS116
Serum
PBS
EGF
Insulin
FGF1
HGF
NRG1
IGF1
DMSO
BEZ235
PD173074
AZD8055
GSK690693
Combo
0
5m
15m
30m
60m
2h
4h
12h
24h
48h
4
3
2
1
0
−1

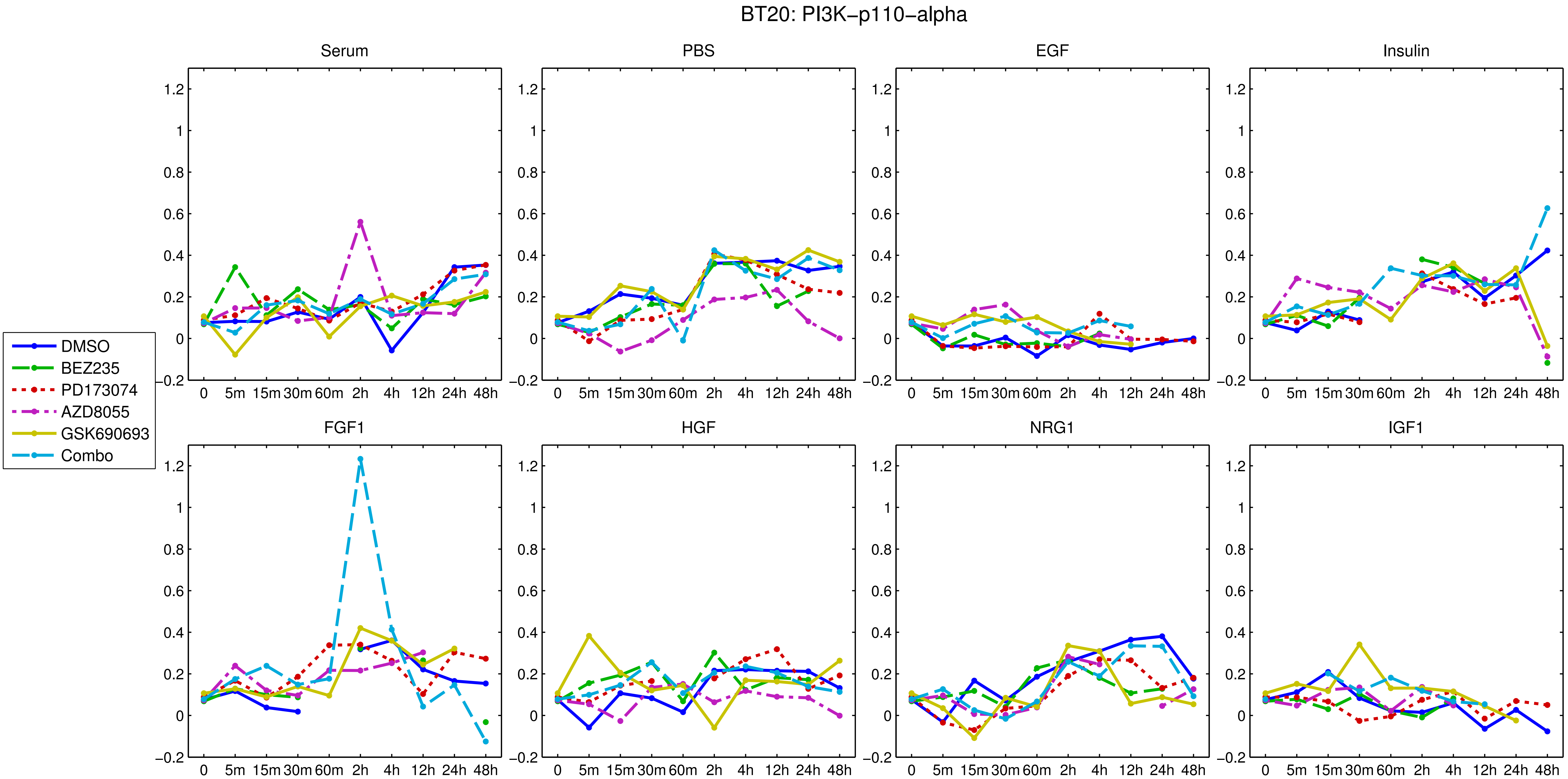

BT20: PI3K−p110−alpha
Serum
PBS
EGF
Insulin
FGF1
HGF
NRG1
IGF1
DMSO
BEZ235
PD173074
AZD8055
GSK690693
Combo
1.2
1
0.8
0.6
0.4
0.2
0
−0.2
0 5m 15m 30m 60m 2h 4h 12h 24h 48h

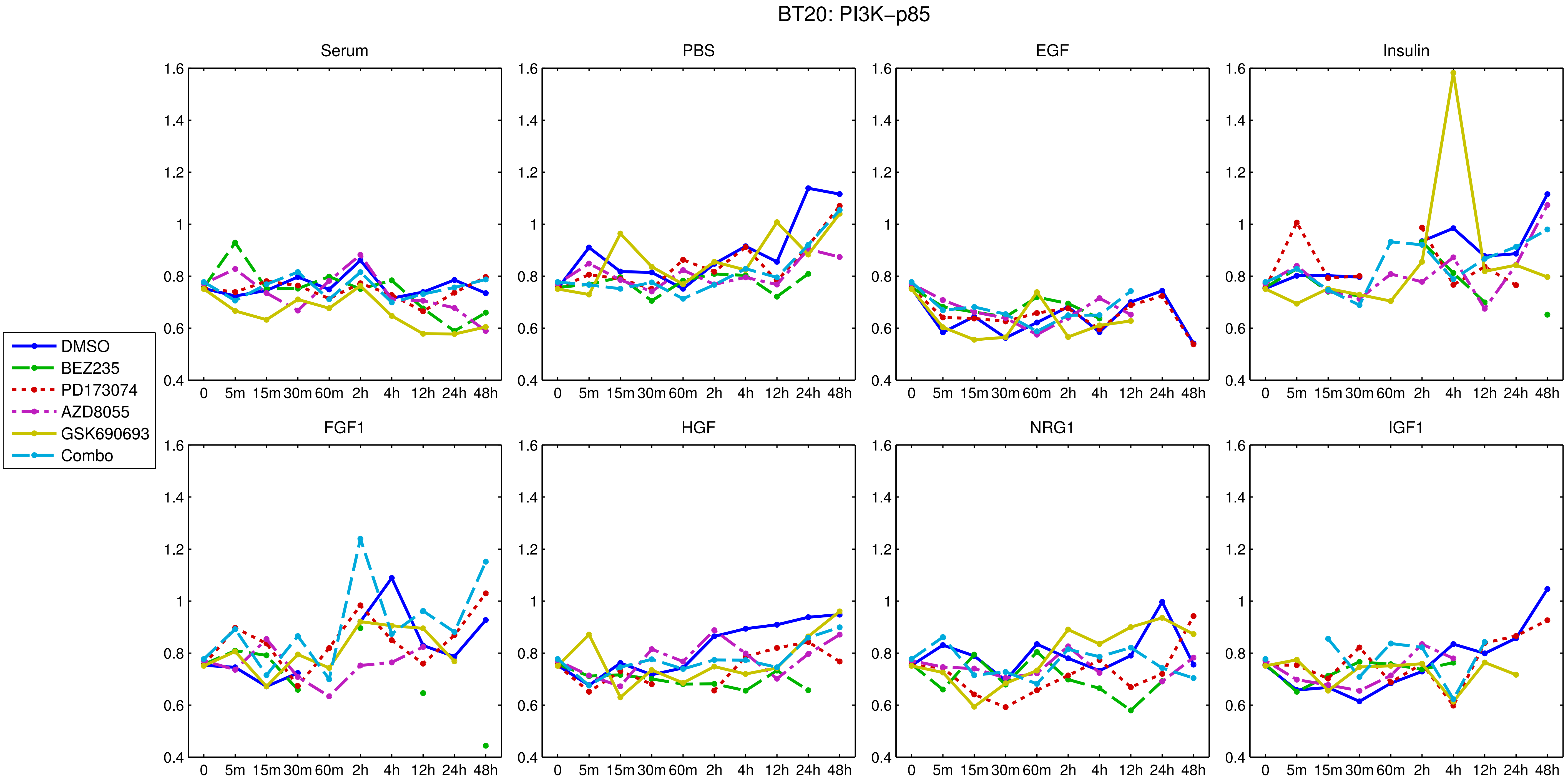
BT20: PI3K−p85
Serum
PBS
EGF
Insulin
FGF1
HGF
NRG1
IGF1
1.6
1.4
1.2
1
0.8
0.6
0.4
0 5m 15m 30m 60m 2h 4h 12h 24h 48h
DMSO
BEZ235
PD173074
AZD8055
GSK690693
Combo

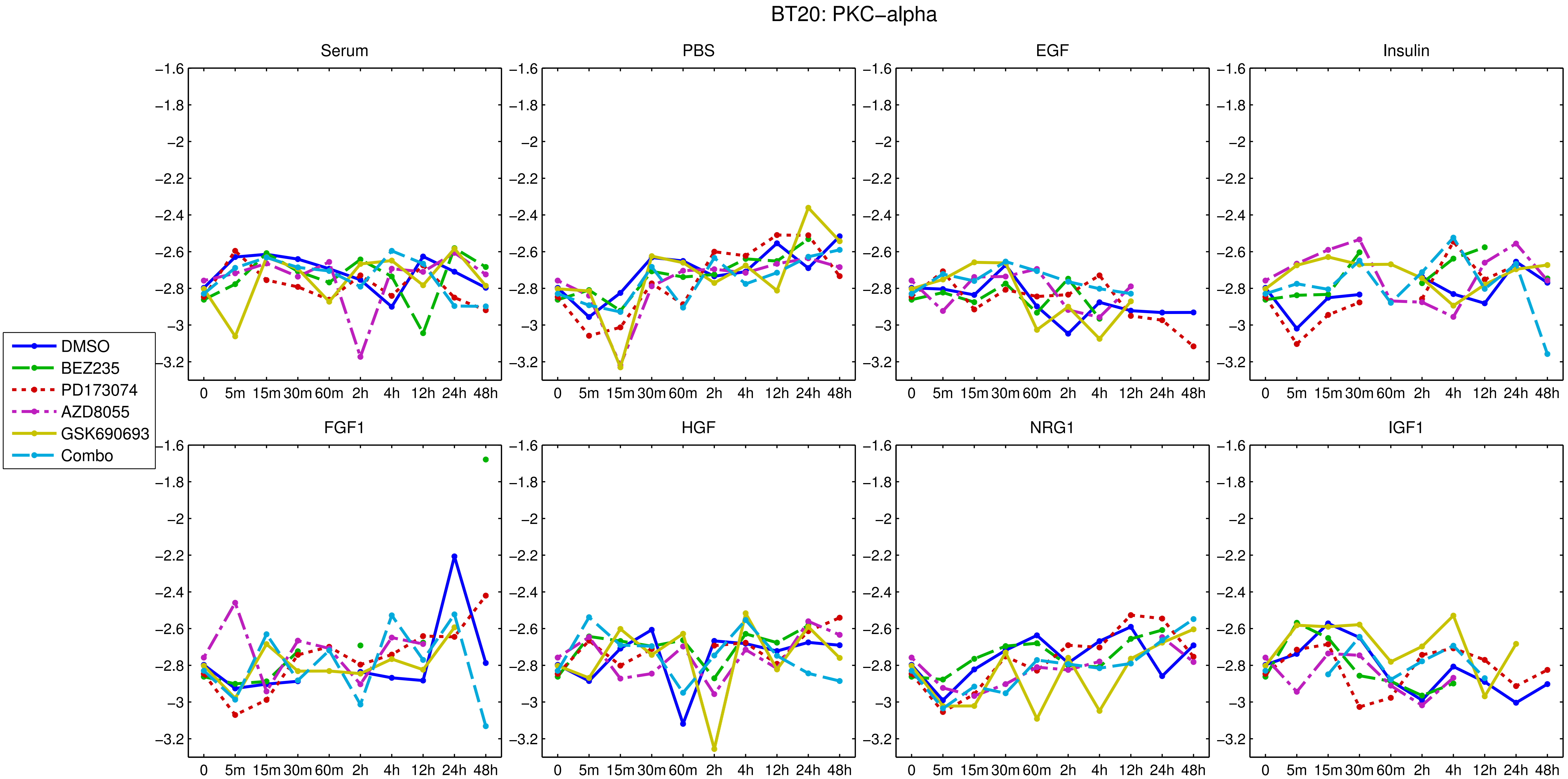

BT20: PKC−alpha
Serum
PBS
EGF
Insulin
FGF1
HGF
NRG1
IGF1
DMSO
BEZ235
PD173074
AZD8055
GSK690693
Combo
−1.6
−1.8
−2
−2.2
−2.4
−2.6
−2.8
−3
−3.2
0
5m
15m
30m
60m
2h
4h
12h
24h
48h

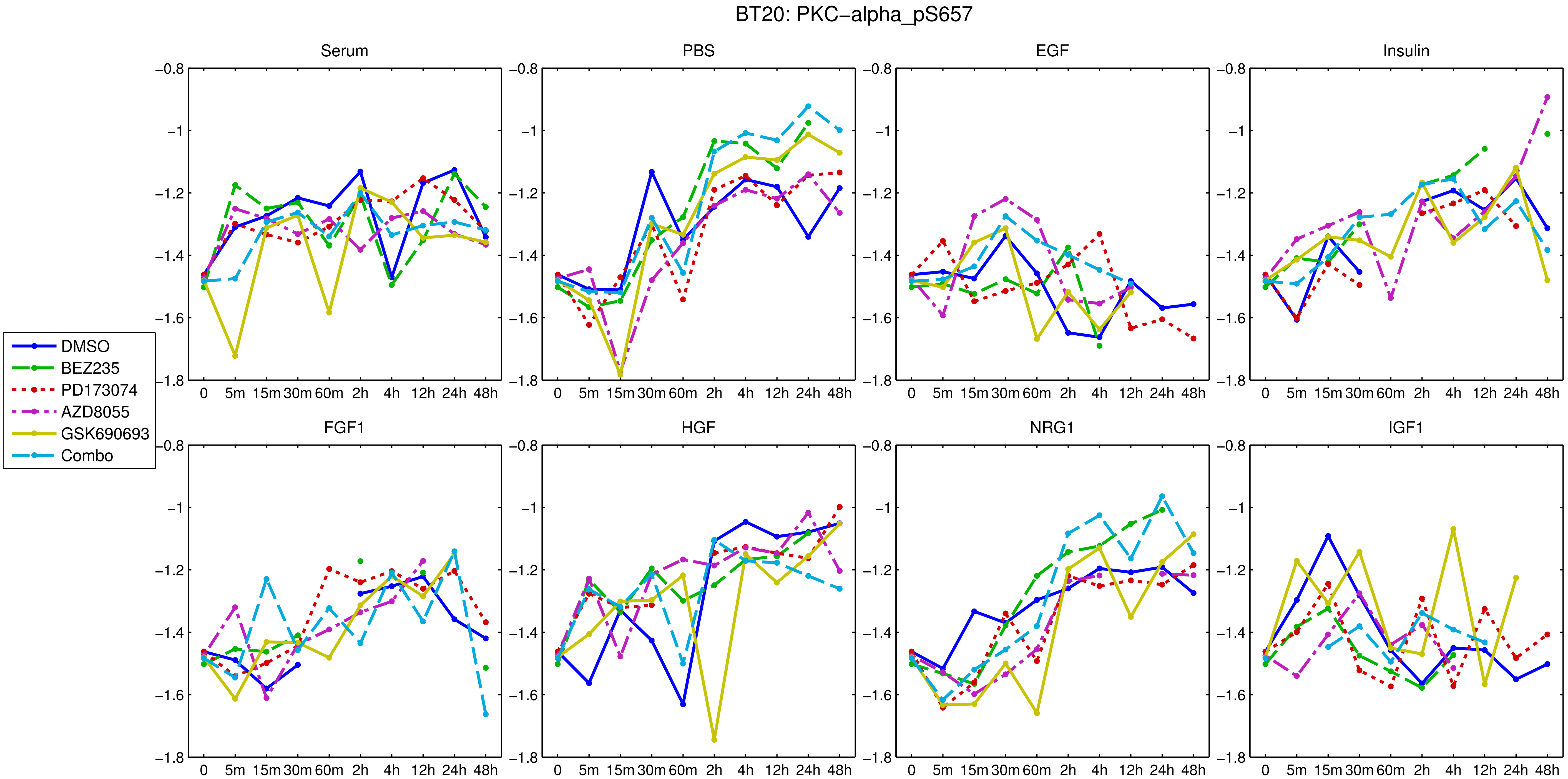
BT20: PKC−alpha_pS657
Serum
PBS
EGF
Insulin
FGF1
HGF
NRG1
IGF1
DMSO
BEZ235
PD173074
AZD8055
GSK690693
Combo
−0.8
−1
−1.2
−1.4
−1.6
−1.8
0
5m
15m
30m
60m
2h
4h
12h
24h
48h

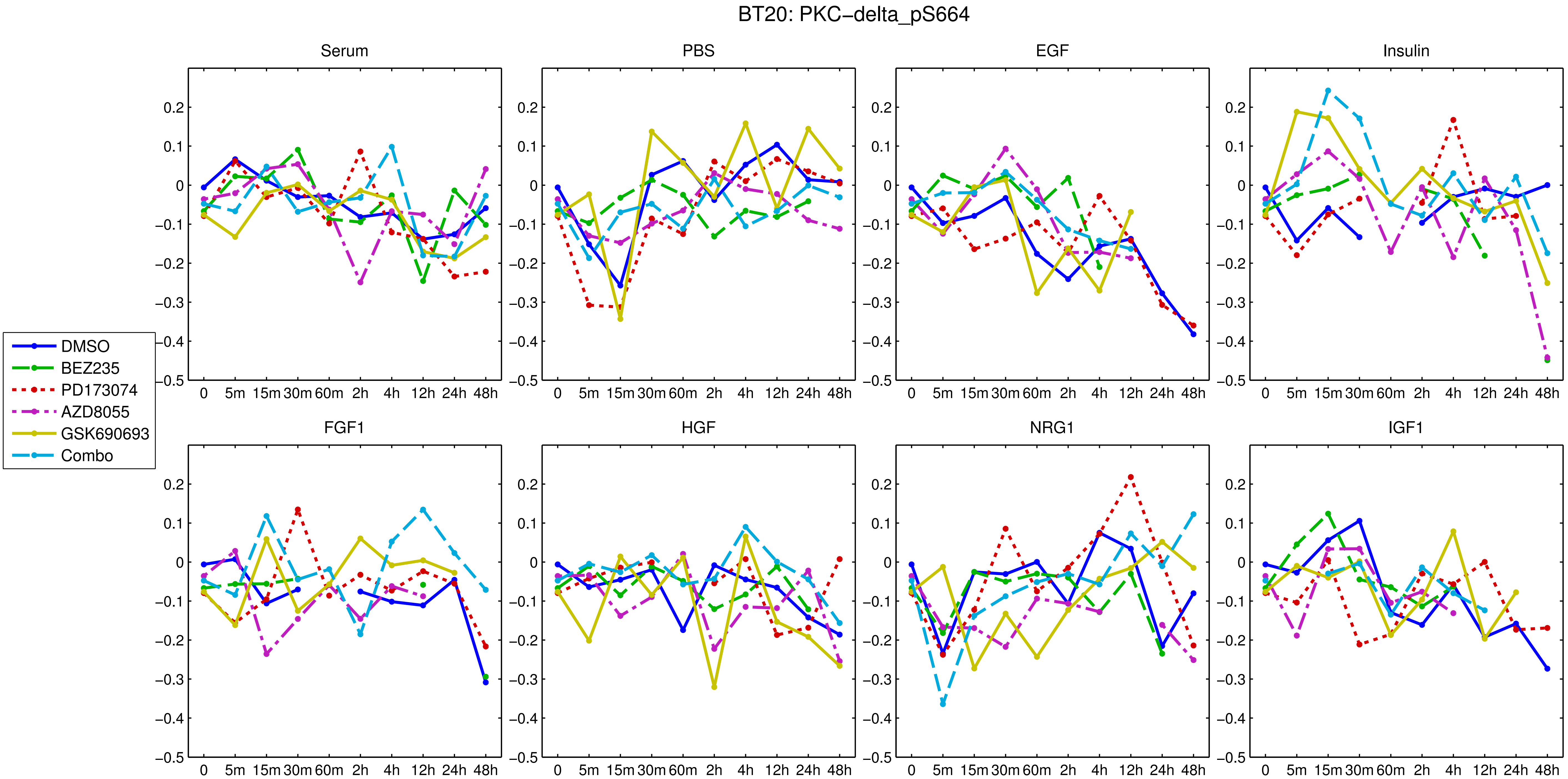

BT20: PKC−delta_pS664
Serum
PBS
EGF
Insulin
FGF1
HGF
NRG1
IGF1
DMSO
BEZ235
PD173074
AZD8055
GSK690693
Combo
0.2
0.1
0
−0.1
−0.2
−0.3
−0.4
−0.5
0 5m 15m 30m 60m 2h 4h 12h 24h 48h

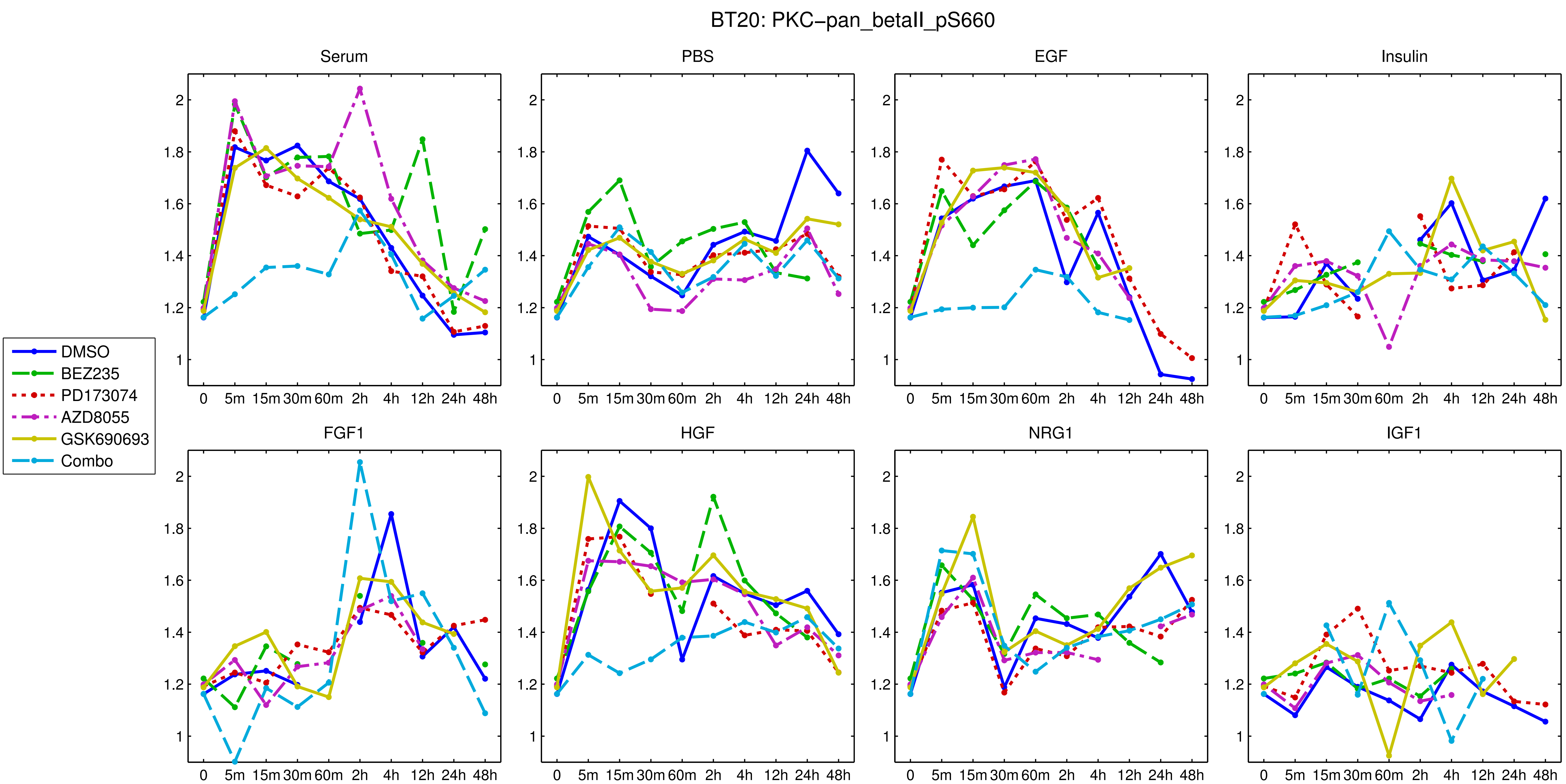

BT20: PKC−pan_betaII_pS660
Serum
PBS
EGF
Insulin
FGF1
HGF
NRG1
IGF1
DMSO
BEZ235
PD173074
AZD8055
GSK690693
Combo
0
5m
15m
30m
60m
2h
4h
12h
24h
48h
1
1.2
1.4
1.6
1.8
2

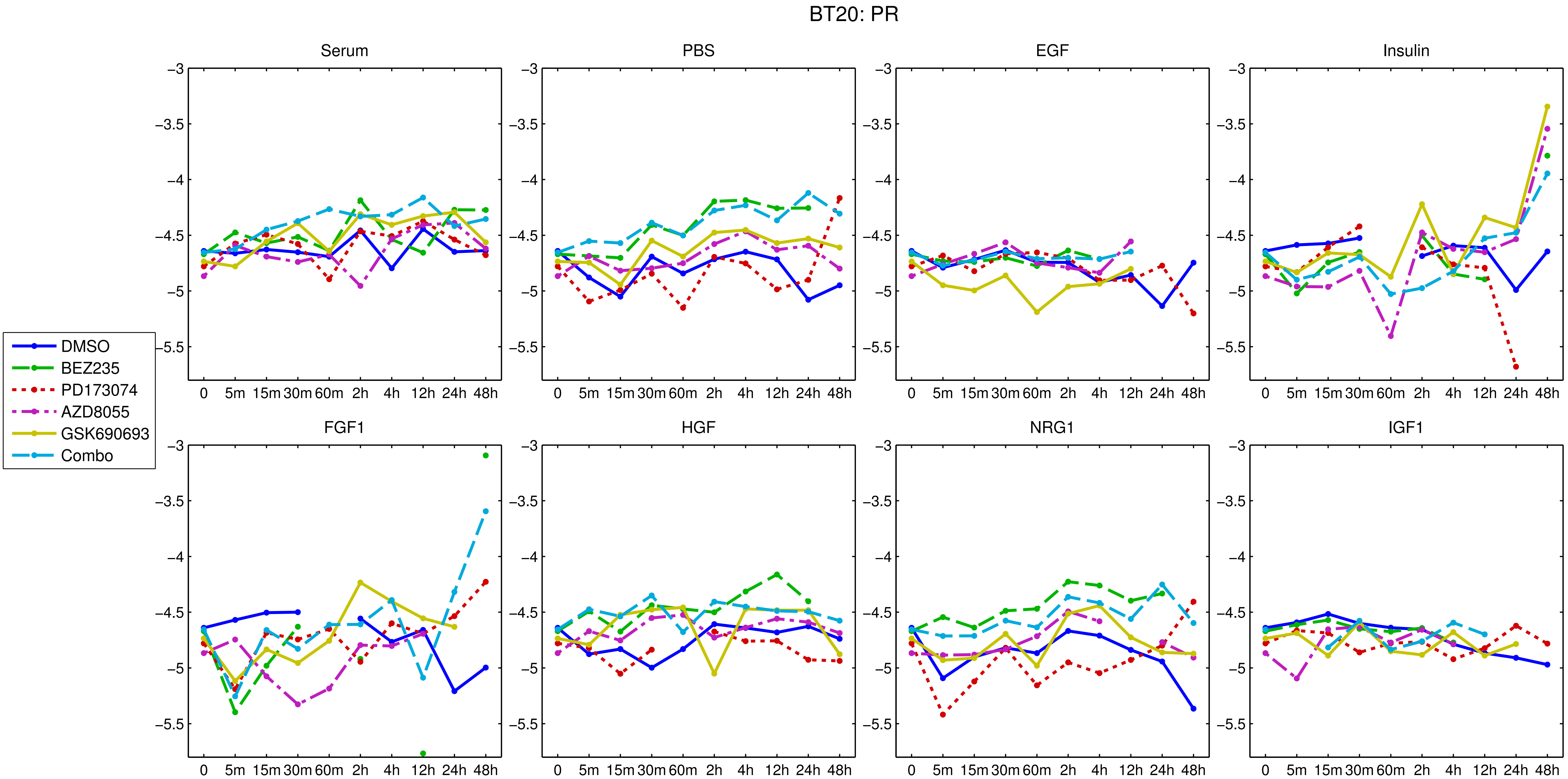

BT20: PR
Serum
PBS
EGF
Insulin
FGF1
HGF
NRG1
IGF1
−3
−3.5
−4
−4.5
−5
−5.5
0
5m
15m
30m
60m
2h
4h
12h
24h
48h
DMSO
BEZ235
PD173074
AZD8055
GSK690693
Combo

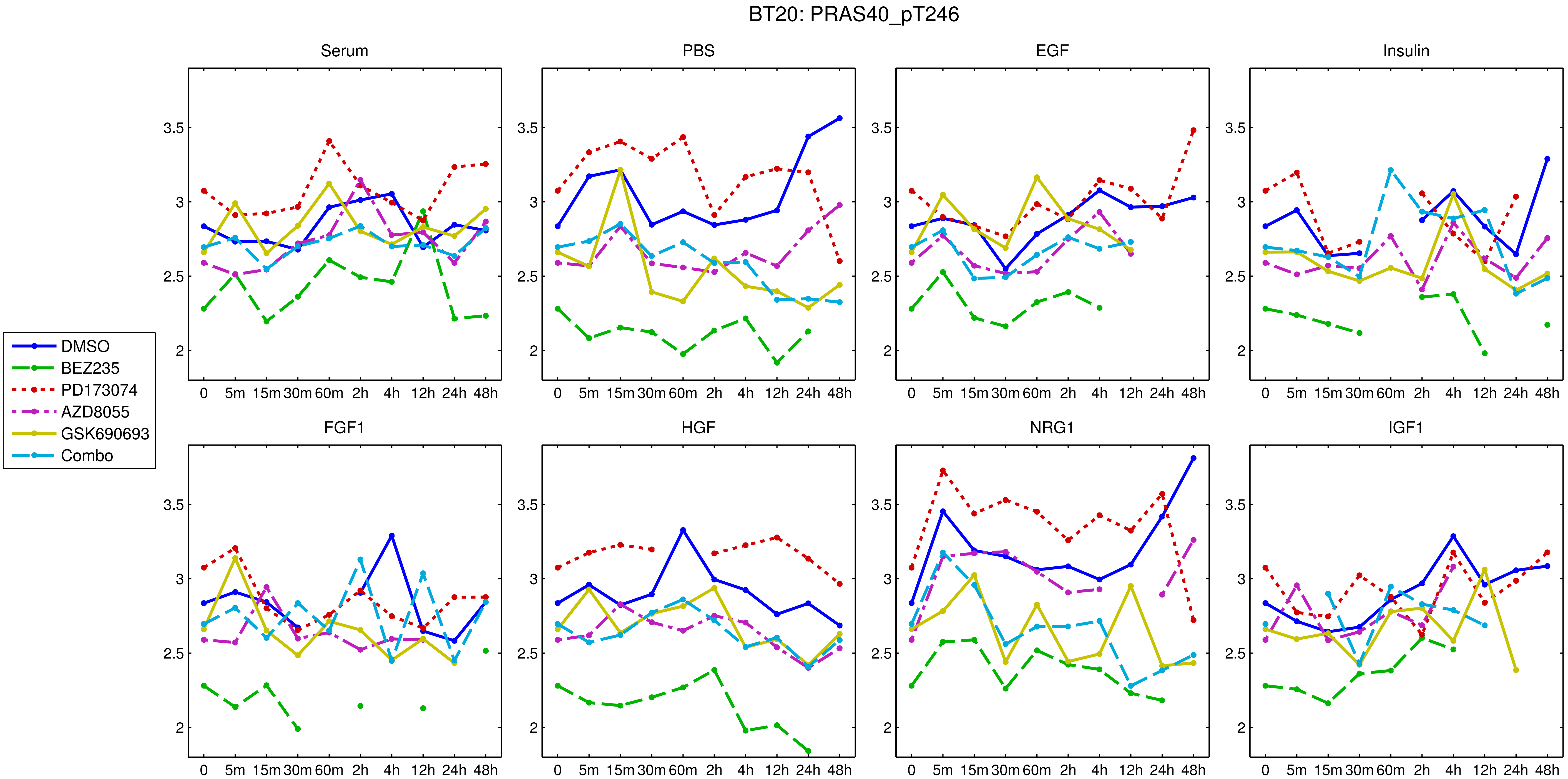
BT20: PRAS40_pT246
Serum
PBS
EGF
Insulin
FGF1
HGF
NRG1
IGF1
DMSO
BEZ235
PD173074
AZD8055
GSK690693
Combo
3.5
3
2.5
2
0
5m
15m
30m
60m
2h
4h
12h
24h
48h

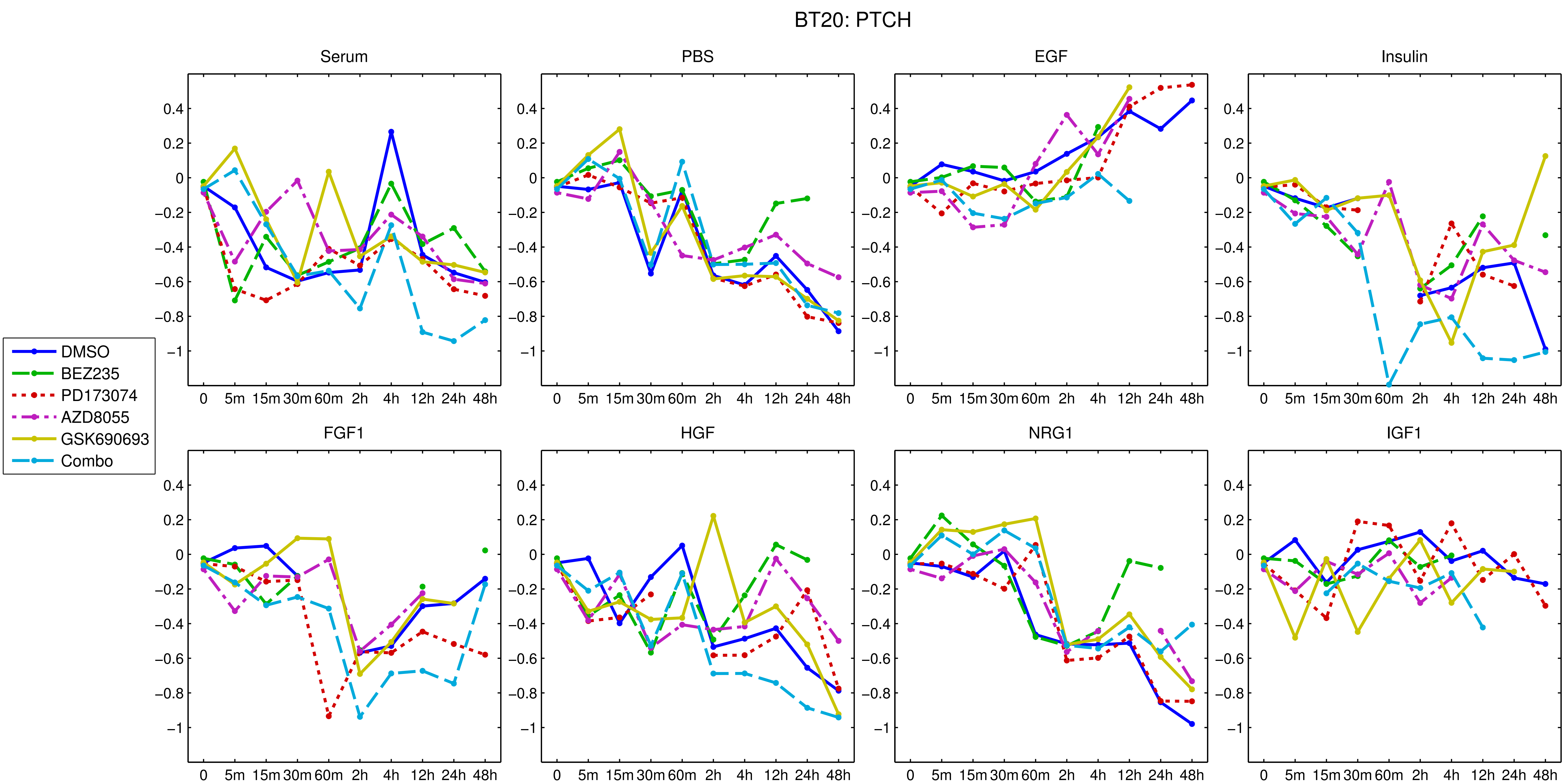
BT20: PTCH
Serum
PBS
EGF
Insulin
FGF1
HGF
NRG1
IGF1
0.4
0.2
0
−0.2
−0.4
−0.6
−0.8
−1
0 5m 15m 30m 60m 2h 4h 12h 24h 48h
DMSO
BEZ235
PD173074
AZD8055
GSK690693
Combo

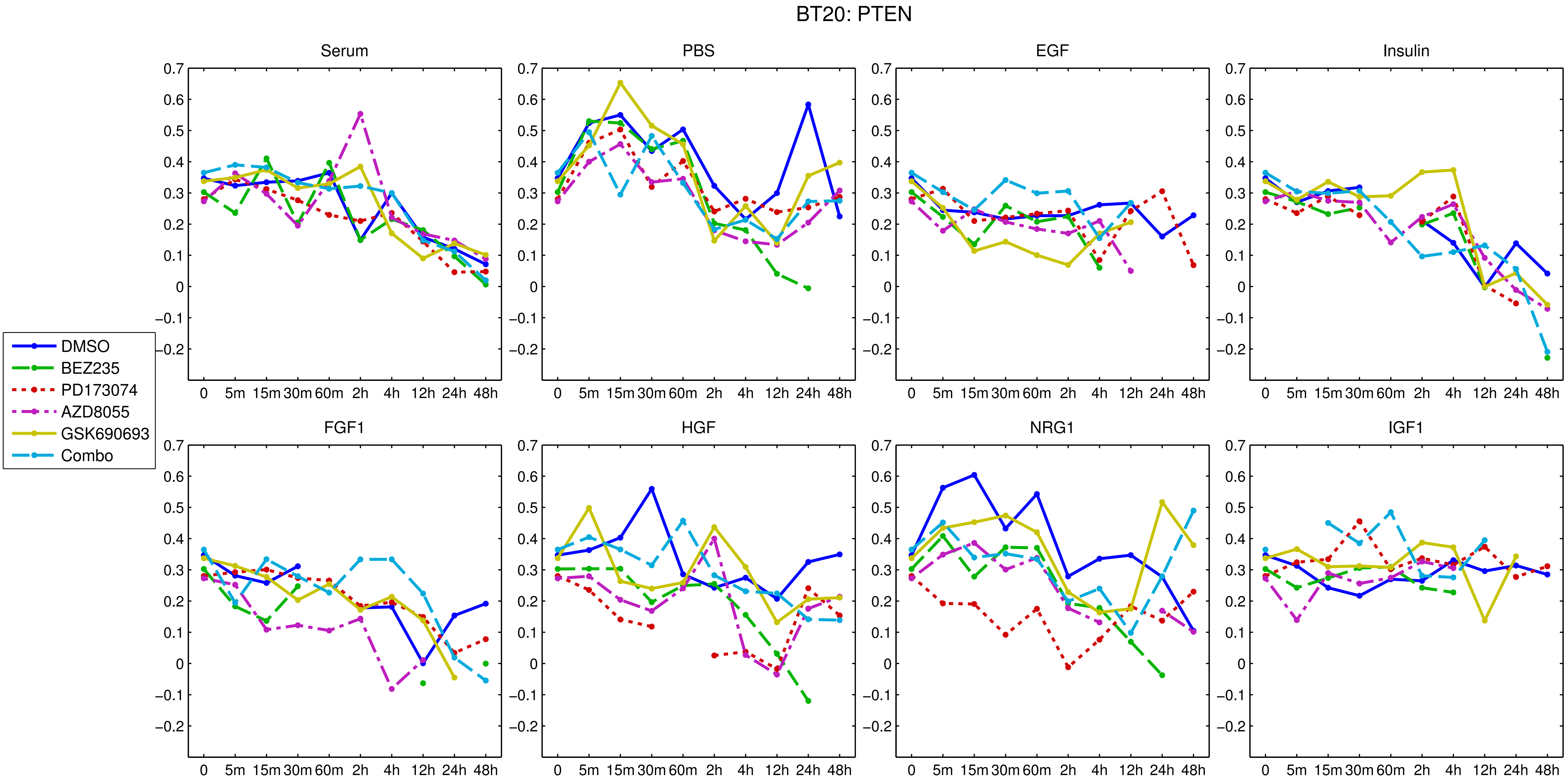
BT20: PTEN
Serum
PBS
EGF
Insulin
FGF1
HGF
NRG1
IGF1
DMSO
BEZ235
PD173074
AZD8055
GSK690693
Combo
0.7
0.6
0.5
0.4
0.3
0.2
0.1
0
−0.1
−0.2
0
5m
15m
30m
60m
2h
4h
12h
24h
48h

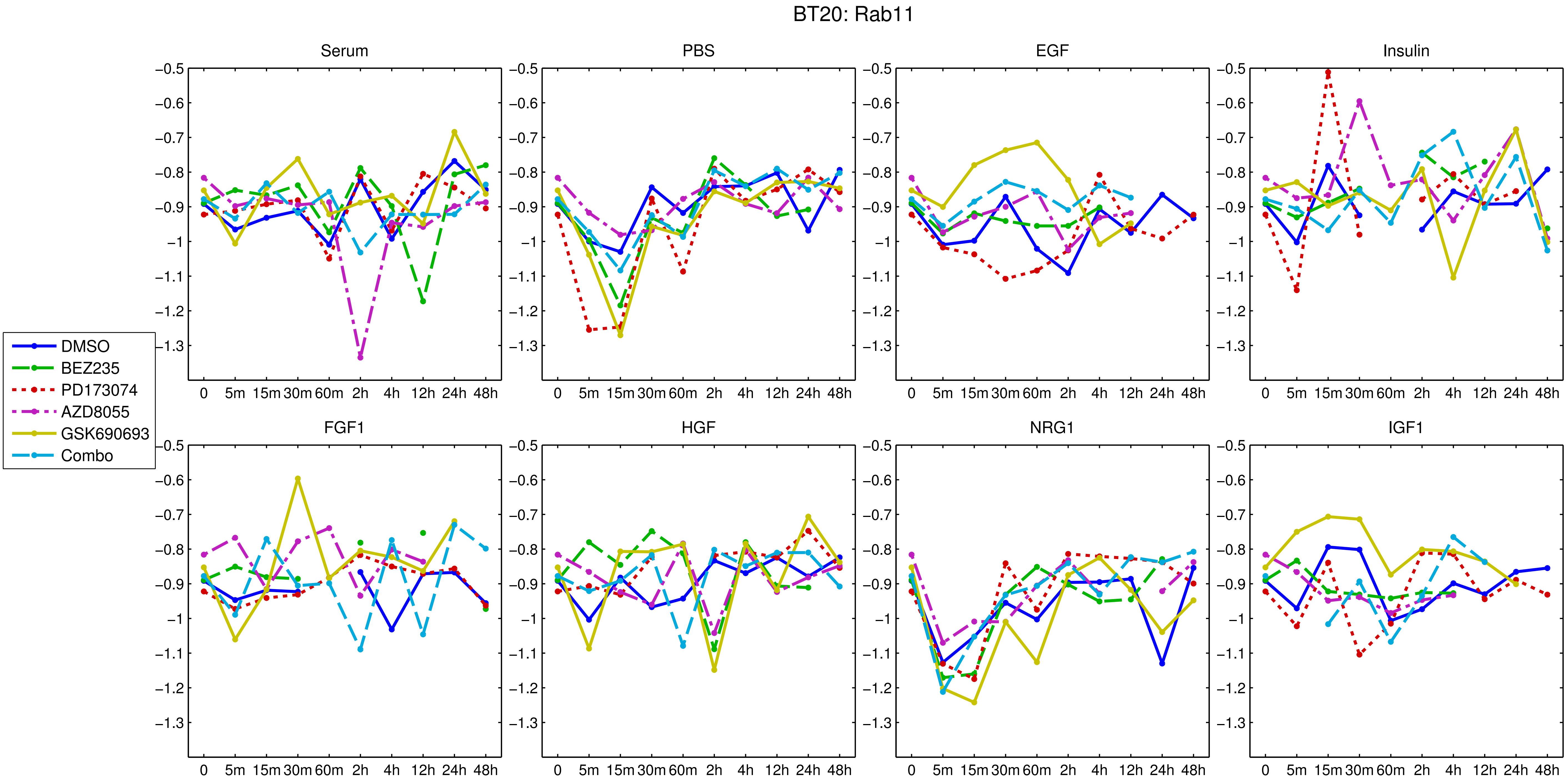
BT20: Rab11
Serum
PBS
EGF
Insulin
FGF1
HGF
NRG1
IGF1
DMSO
BEZ235
PD173074
AZD8055
GSK690693
Combo
−0.5
−0.6
−0.7
−0.8
−0.9
−1
−1.1
−1.2
−1.3
0 5m 15m 30m 60m 2h 4h 12h 24h 48h

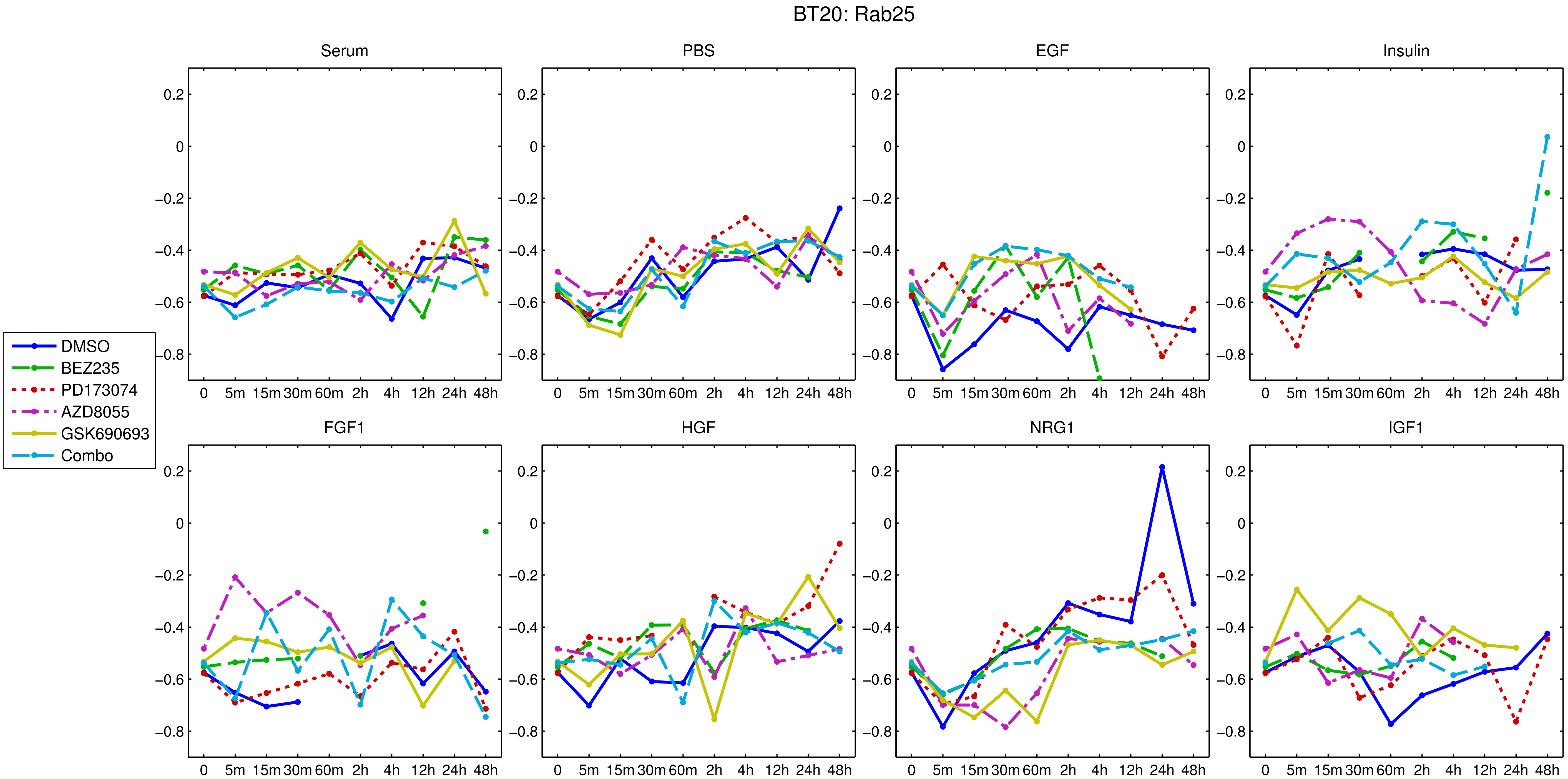
BT20: Rab25
Serum
PBS
EGF
Insulin
FGF1
HGF
NRG1
IGF1
DMSO
BEZ235
PD173074
AZD8055
GSK690693
Combo
0.2
0
−0.2
−0.4
−0.6
−0.8
0 5m 15m 30m 60m 2h 4h 12h 24h 48h

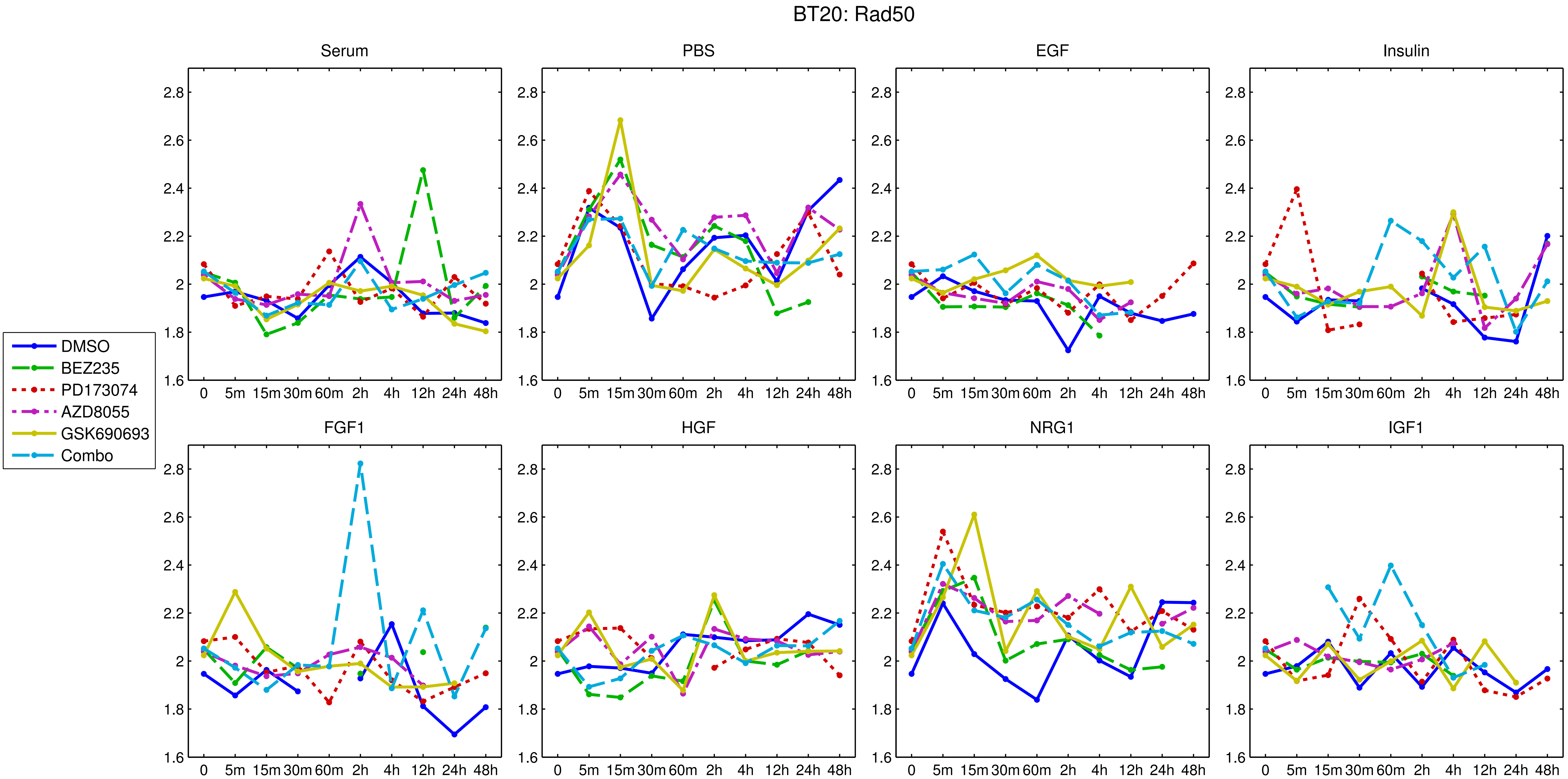
BT20: Rad50
Serum
PBS
EGF
Insulin
FGF1
HGF
NRG1
IGF1
DMSO
BEZ235
PD173074
AZD8055
GSK690693
Combo
2.8
2.6
2.4
2.2
2
1.8
1.6
0
5m
15m
30m
60m
2h
4h
12h
24h
48h

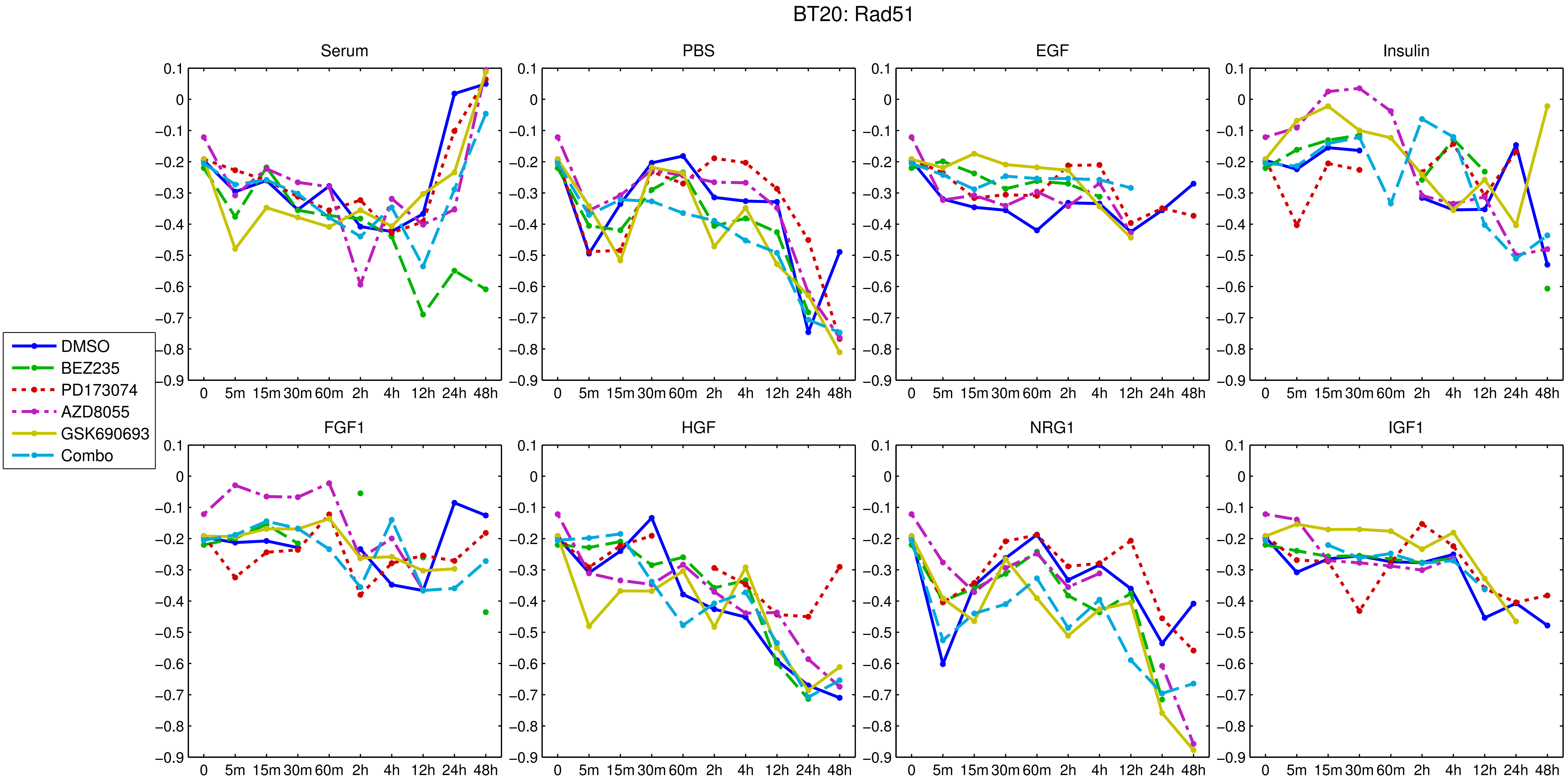

BT20: Rad51
Serum
PBS
EGF
Insulin
FGF1
HGF
NRG1
IGF1
DMSO
BEZ235
PD173074
AZD8055
GSK690693
Combo
0.1
0
−0.1
−0.2
−0.3
−0.4
−0.5
−0.6
−0.7
−0.8
−0.9
0
5m
15m
30m
60m
2h
4h
12h
24h
48h

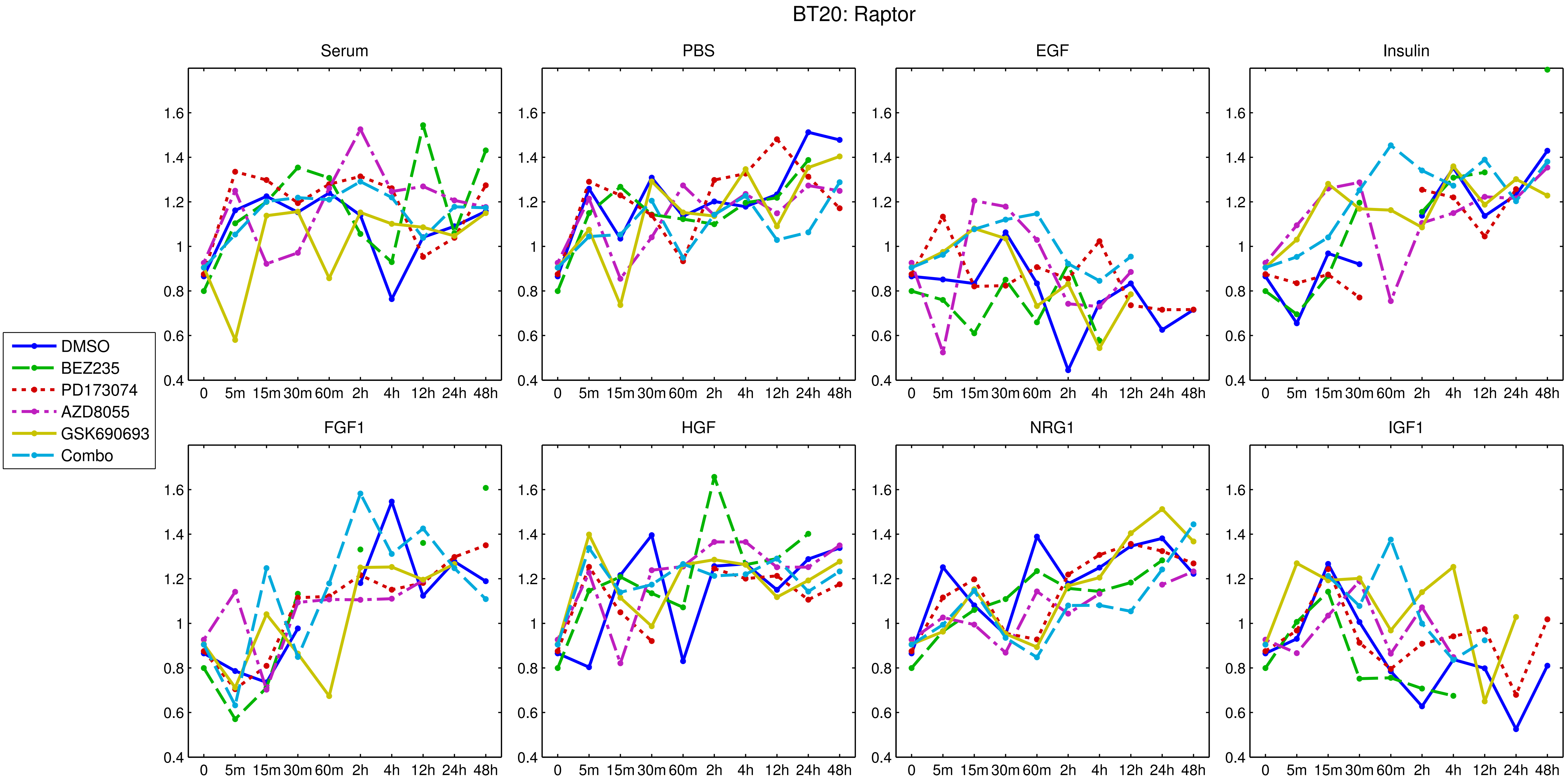

BT20: Raptor
Serum
PBS
EGF
Insulin
FGF1
HGF
NRG1
IGF1
DMSO
BEZ235
PD173074
AZD8055
GSK690693
Combo
0.4
0.6
0.8
1
1.2
1.4
1.6
0
5m
15m
30m
60m
2h
4h
12h
24h
48h

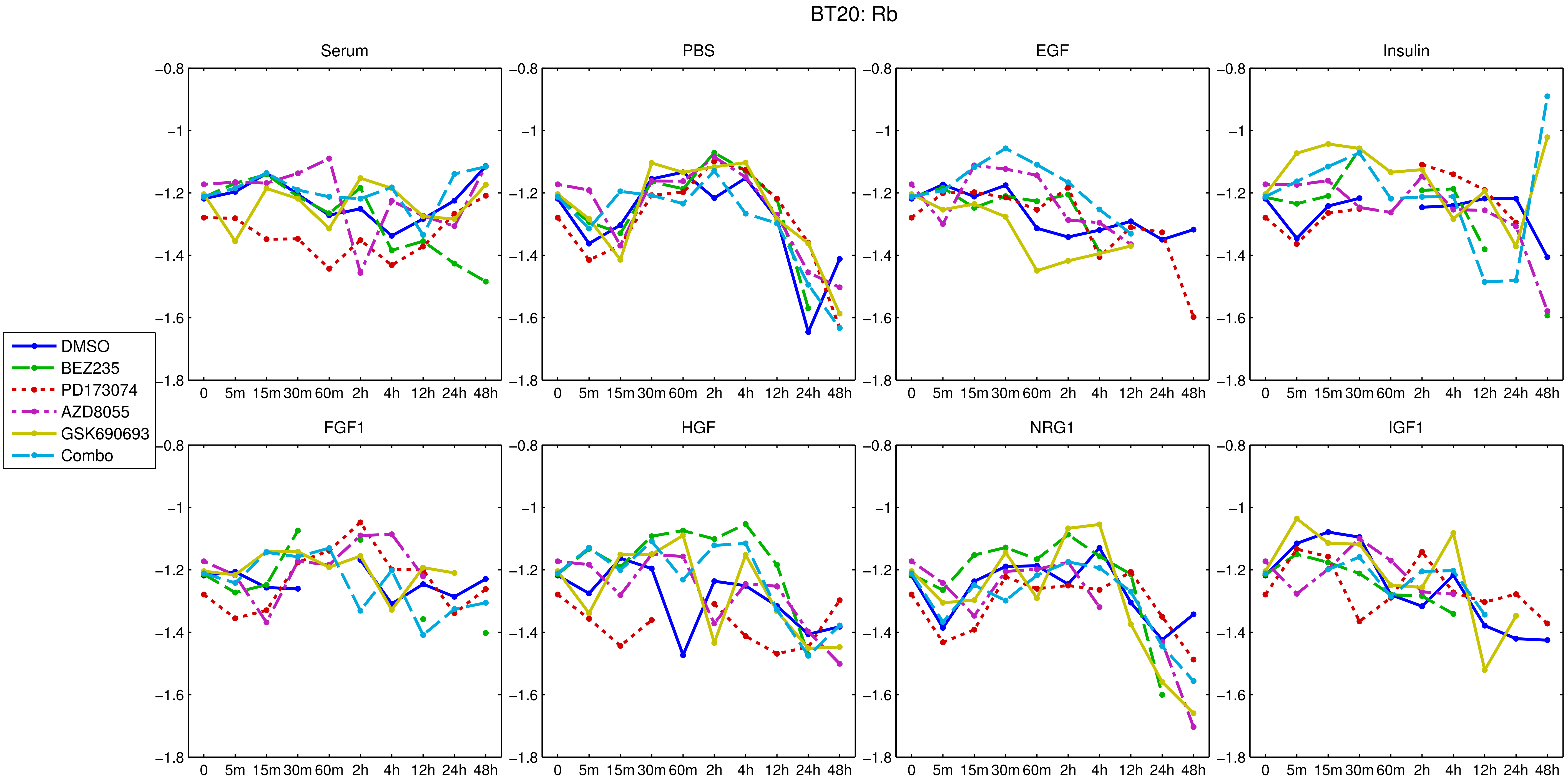
BT20: Rb
Serum
PBS
EGF
Insulin
FGF1
HGF
NRG1
IGF1
DMSO
BEZ235
PD173074
AZD8055
GSK690693
Combo
−0.8
−1
−1.2
−1.4
−1.6
−1.8
0
5m
15m
30m
60m
2h
4h
12h
24h
48h

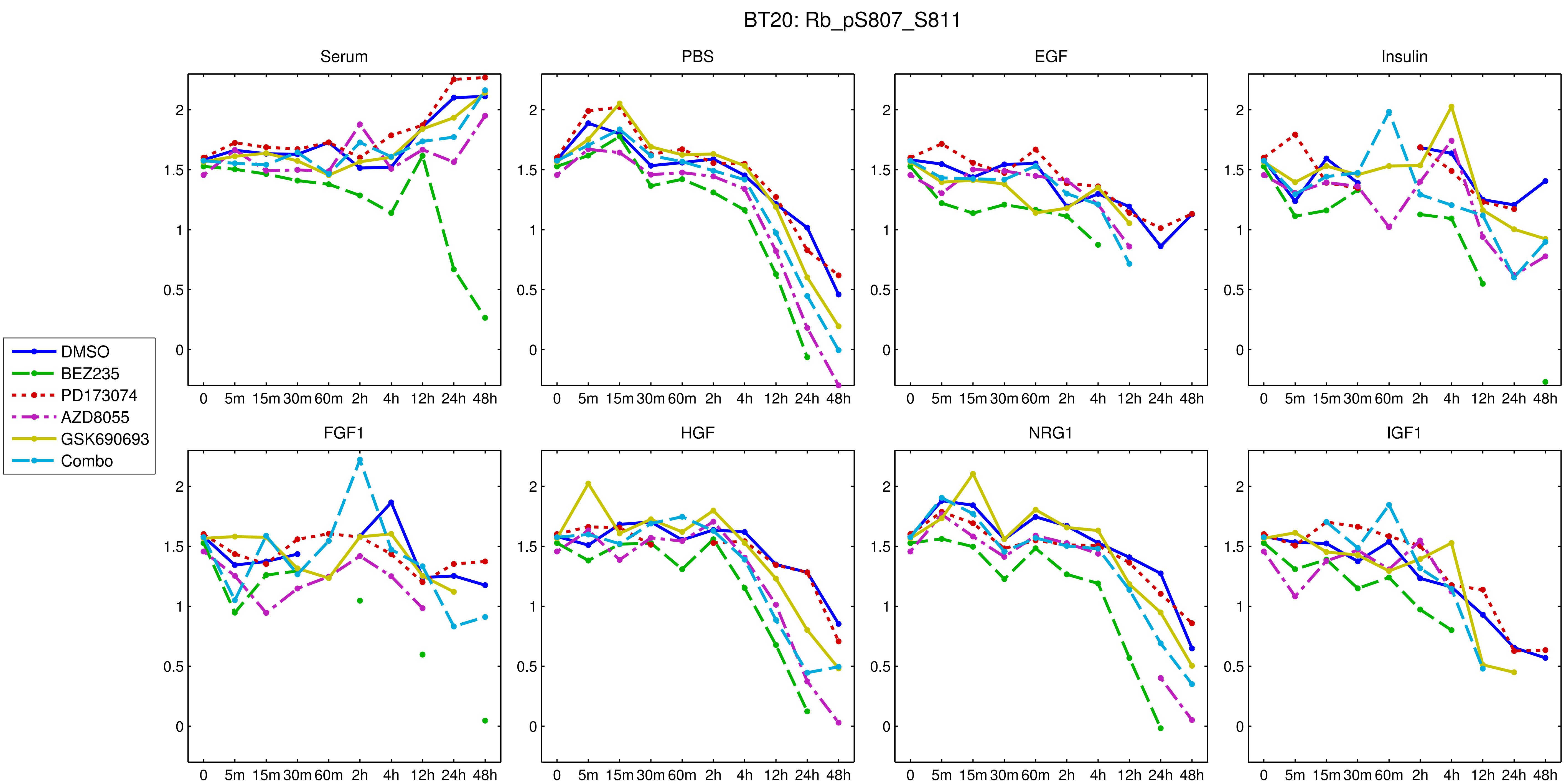
BT20: Rb_pS807_S811
Serum
PBS
EGF
Insulin
FGF1
HGF
NRG1
IGF1
DMSO
BEZ235
PD173074
AZD8055
GSK690693
Combo
0
5m
15m
30m
60m
2h
4h
12h
24h
48h

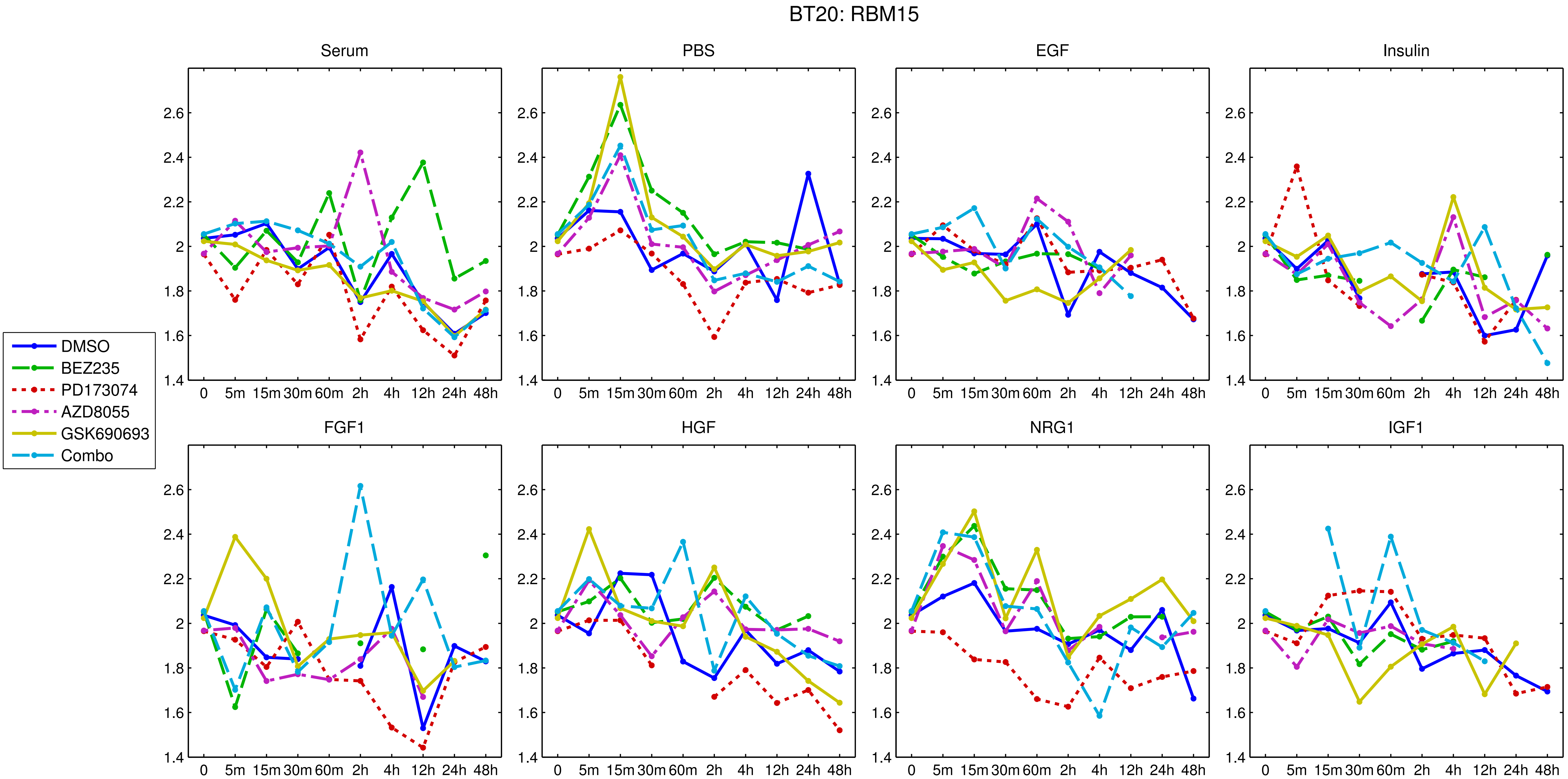
BT20: RBM15
Serum
PBS
EGF
Insulin
FGF1
HGF
NRG1
IGF1
DMSO
BEZ235
PD173074
AZD8055
GSK690693
Combo
0 5m 15m 30m 60m 2h 4h 12h 24h 48h
1.4 1.6 1.8 2 2.2 2.4 2.6

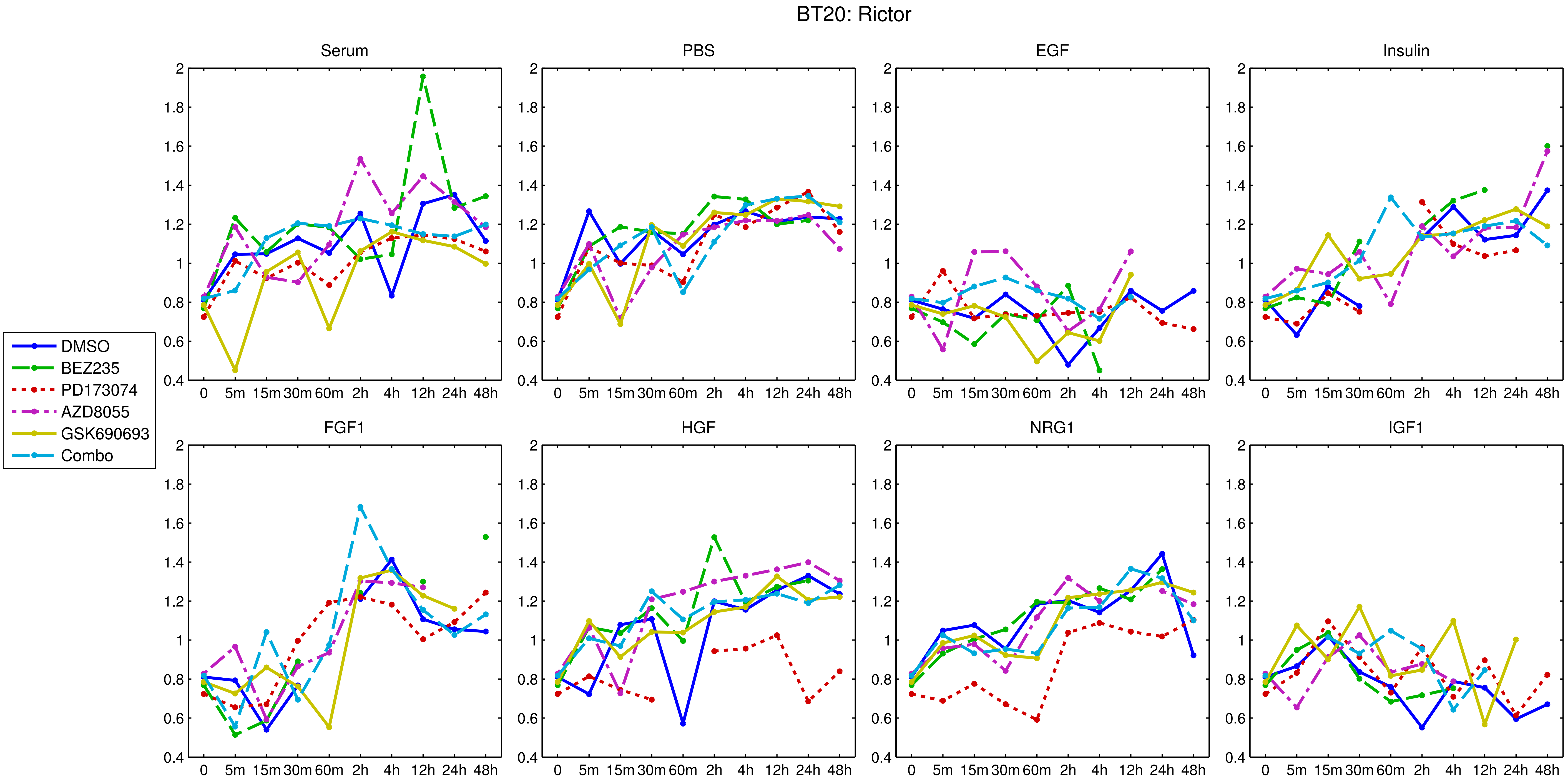
BT20: Rictor
Serum
PBS
EGF
Insulin
FGF1
HGF
NRG1
IGF1
DMSO
BEZ235
PD173074
AZD8055
GSK690693
Combo
0
5m
15m
30m
60m
2h
4h
12h
24h
48h
0.4
0.6
0.8
1
1.2
1.4
1.6
1.8
2

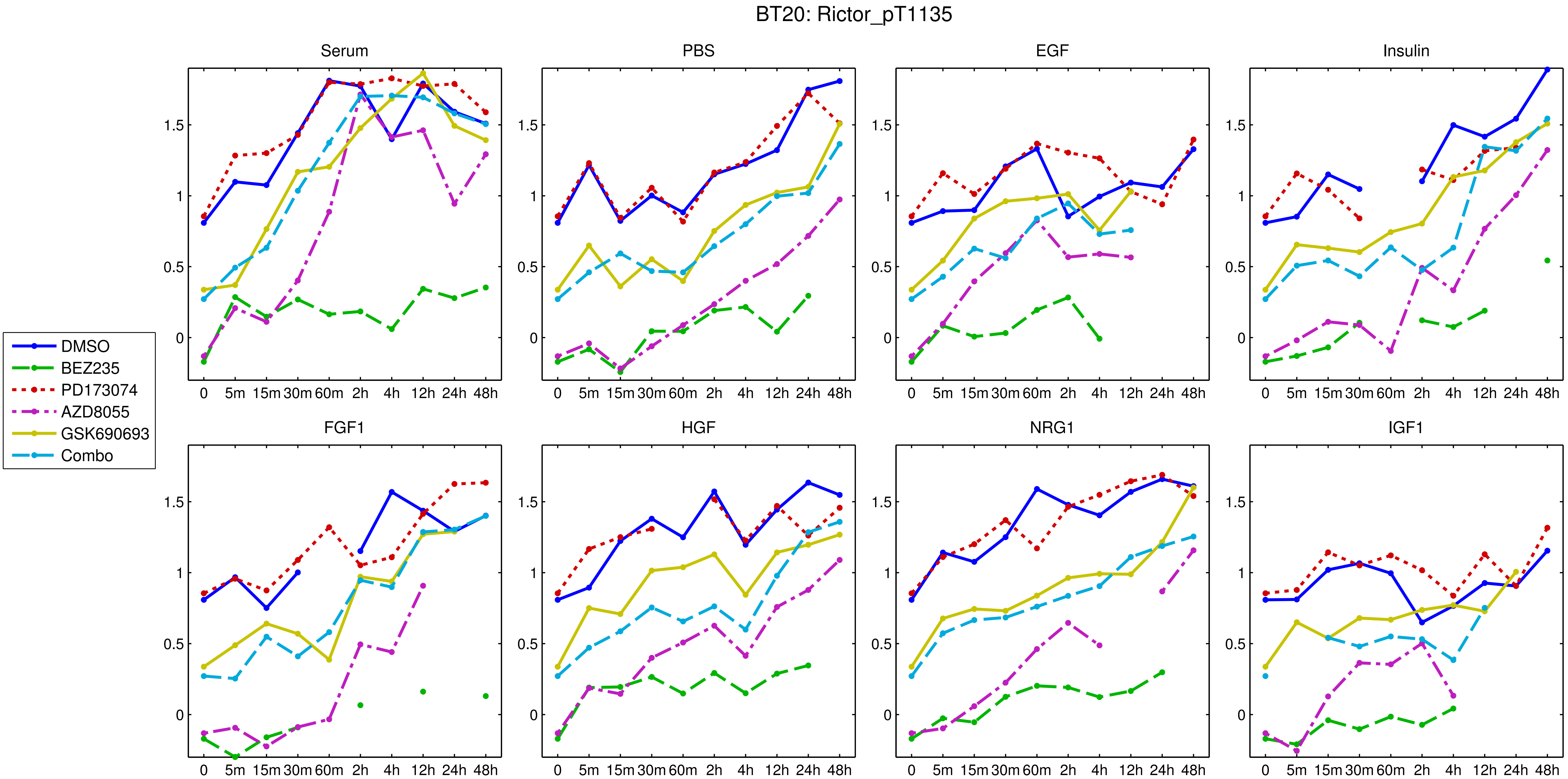
BT20: Rictor_pT1135
Serum
PBS
EGF
Insulin
FGF1
HGF
NRG1
IGF1
DMSO
BEZ235
PD173074
AZD8055
GSK690693
Combo
1.5
1
0.5
0
0
5m
15m
30m
60m
2h
4h
12h
24h
48h

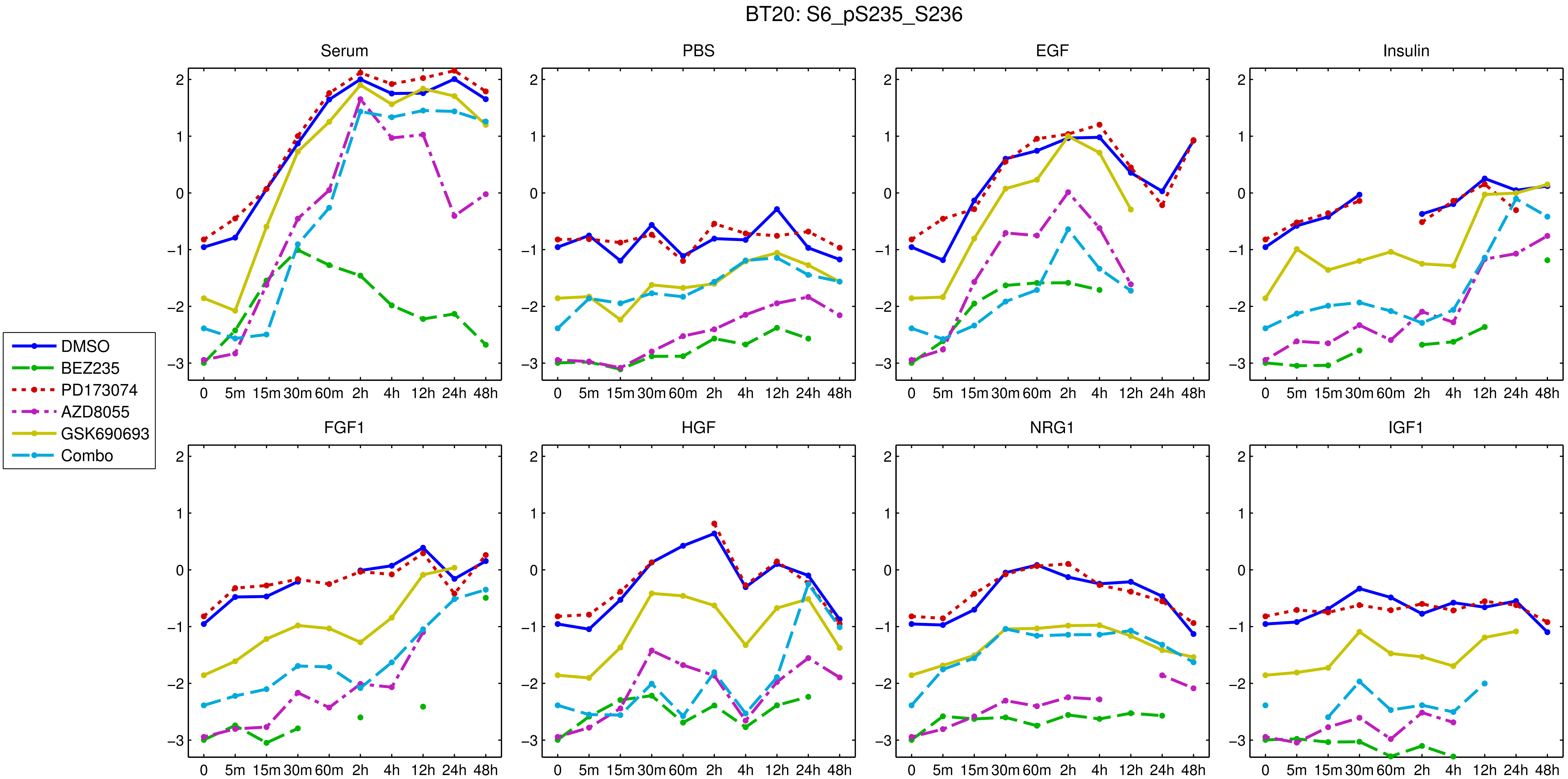
BT20: S6_pS235_S236
Serum
PBS
EGF
Insulin
FGF1
HGF
NRG1
IGF1
DMSO
BEZ235
PD173074
AZD8055
GSK690693
Combo
0
5m
15m
30m
60m
2h
4h
12h
24h
48h
2
1
0
−1
−2
−3

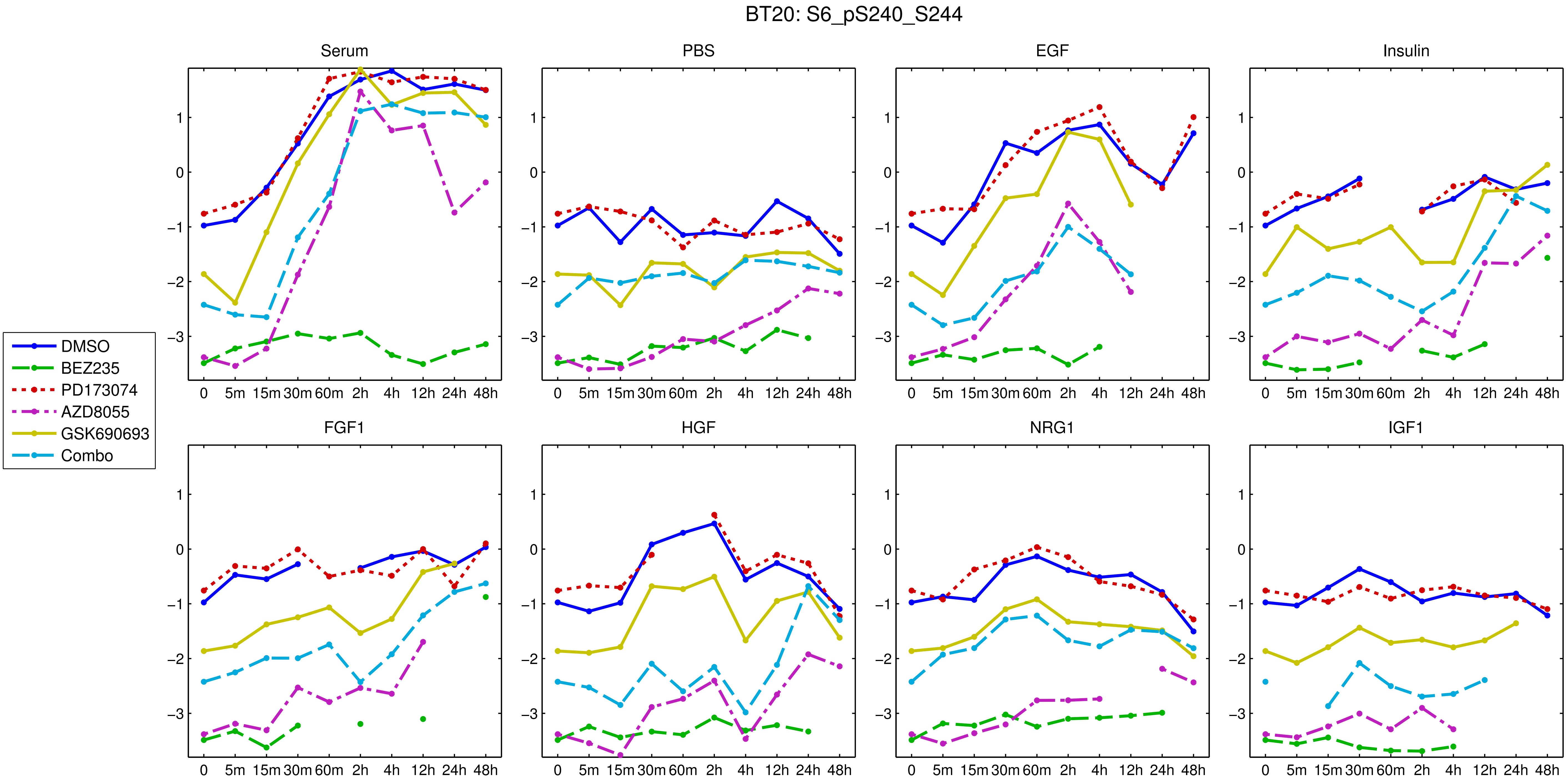
BT20: S6_pS240_S244
Serum
PBS
EGF
Insulin
FGF1
HGF
NRG1
IGF1
DMSO
BEZ235
PD173074
AZD8055
GSK690693
Combo
0
5m
15m
30m
60m
2h
4h
12h
24h
48h
1
0
−1
−2
−3

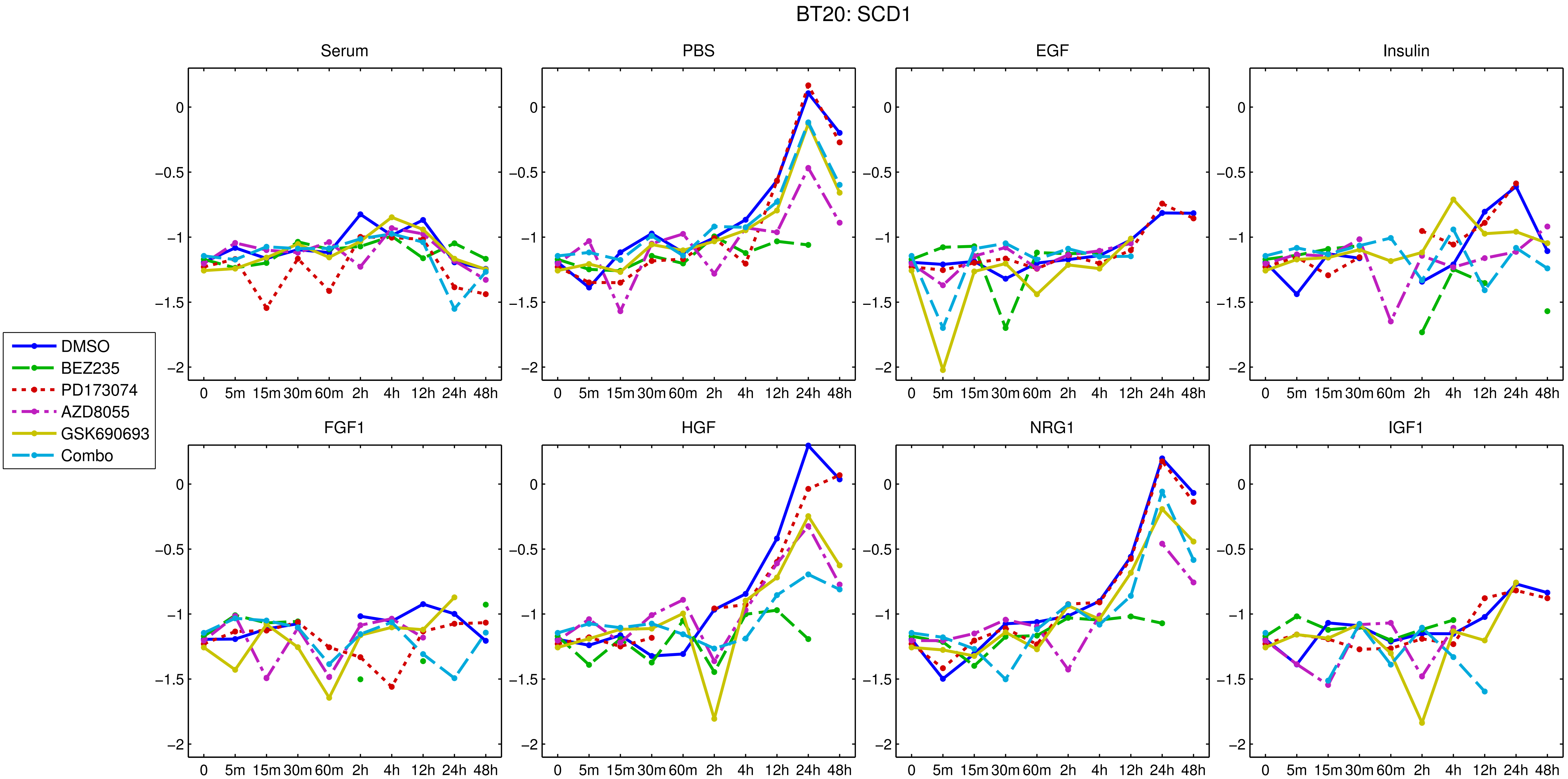
BT20: SCD1
Serum
PBS
EGF
Insulin
FGF1
HGF
NRG1
IGF1
DMSO
BEZ235
PD173074
AZD8055
GSK690693
Combo
0
−0.5
−1
−1.5
−2
0 5m 15m 30m 60m 2h 4h 12h 24h 48h

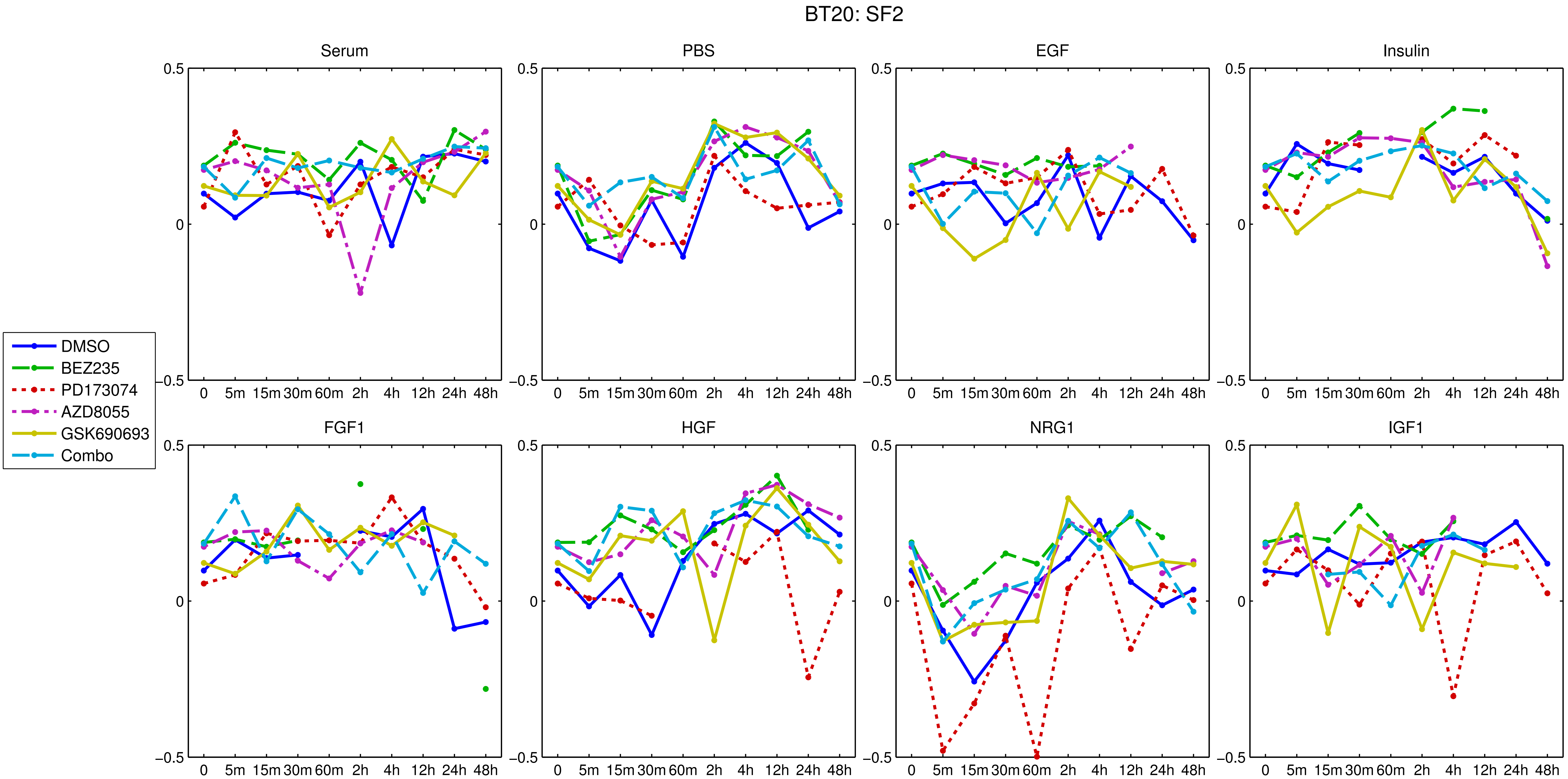

BT20: SF2
Serum
PBS
EGF
Insulin
FGF1
HGF
NRG1
IGF1
0.5
0
−0.5
0
5m
15m
30m
60m
2h
4h
12h
24h
48h
DMSO
BEZ235
PD173074
AZD8055
GSK690693
Combo

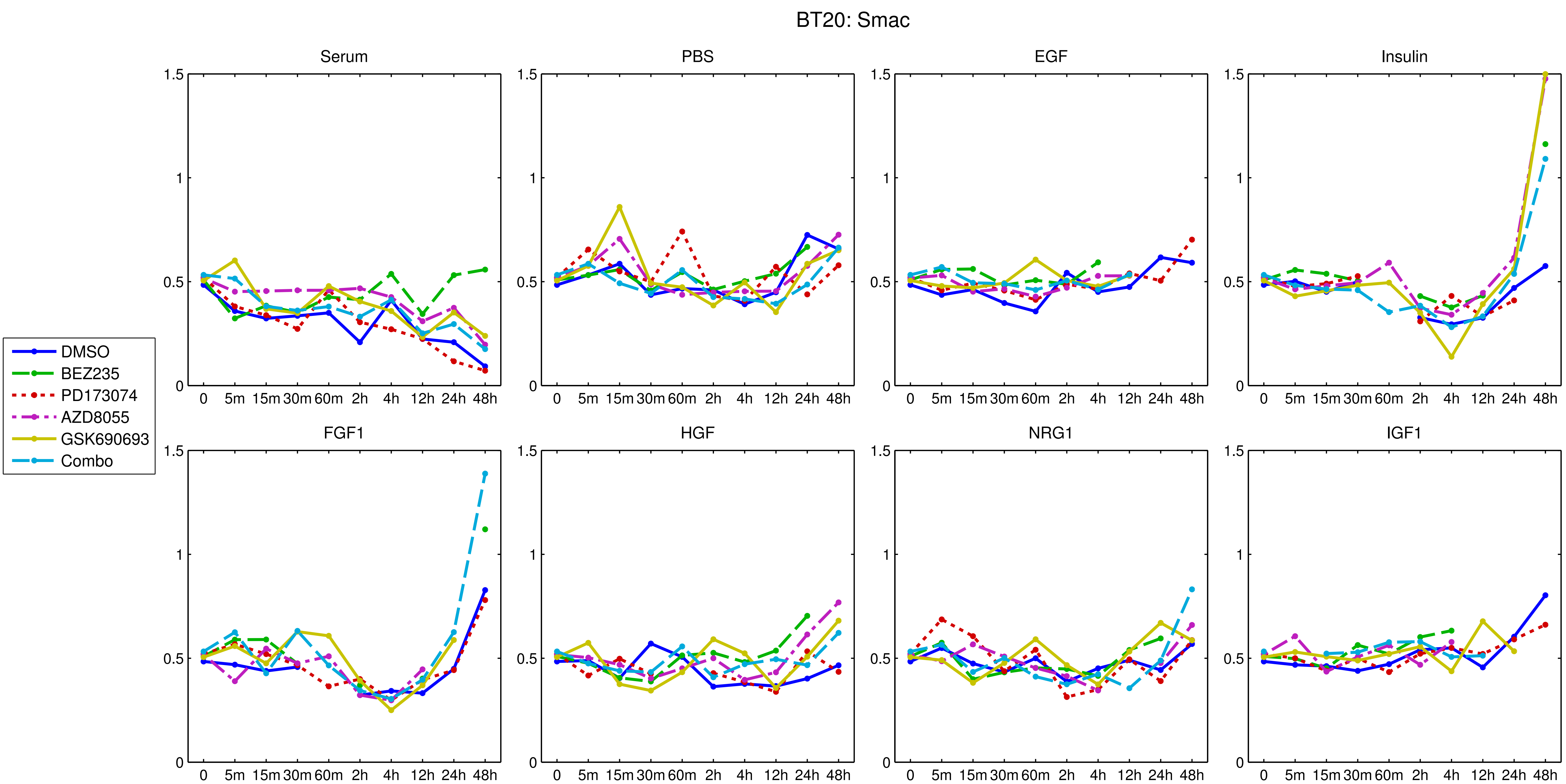
BT20: Smac
Serum
PBS
EGF
Insulin
FGF1
HGF
NRG1
IGF1
DMSO
BEZ235
PD173074
AZD8055
GSK690693
Combo
0
0.5
1
1.5
0 5m 15m 30m 60m 2h 4h 12h 24h 48h

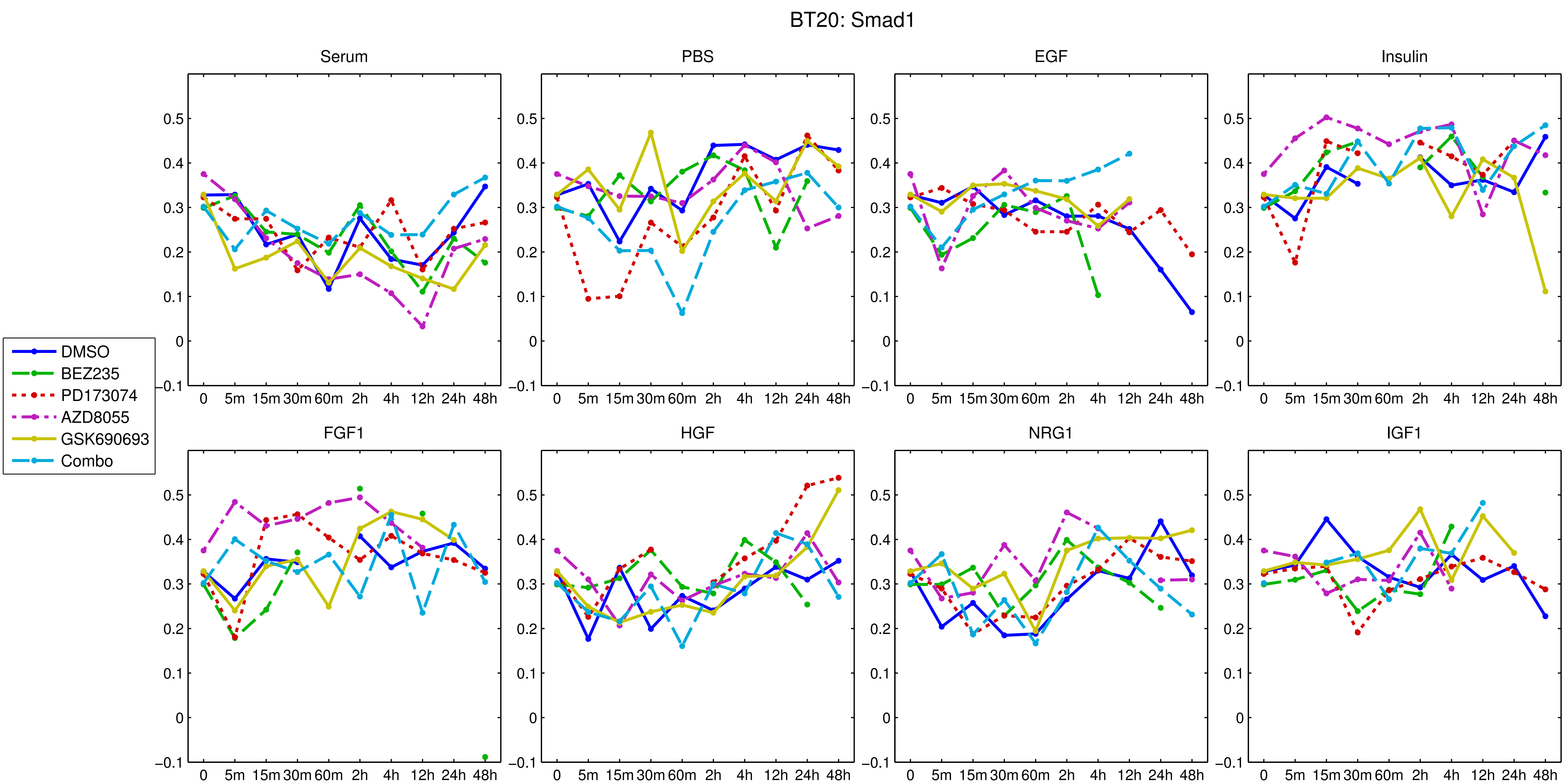
BT20: Smad1
Serum
PBS
EGF
Insulin
FGF1
HGF
NRG1
IGF1
0.5
0.4
0.3
0.2
0.1
0
−0.1
0
5m
15m
30m
60m
2h
4h
12h
24h
48h
DMSO
BEZ235
PD173074
AZD8055
GSK690693
Combo

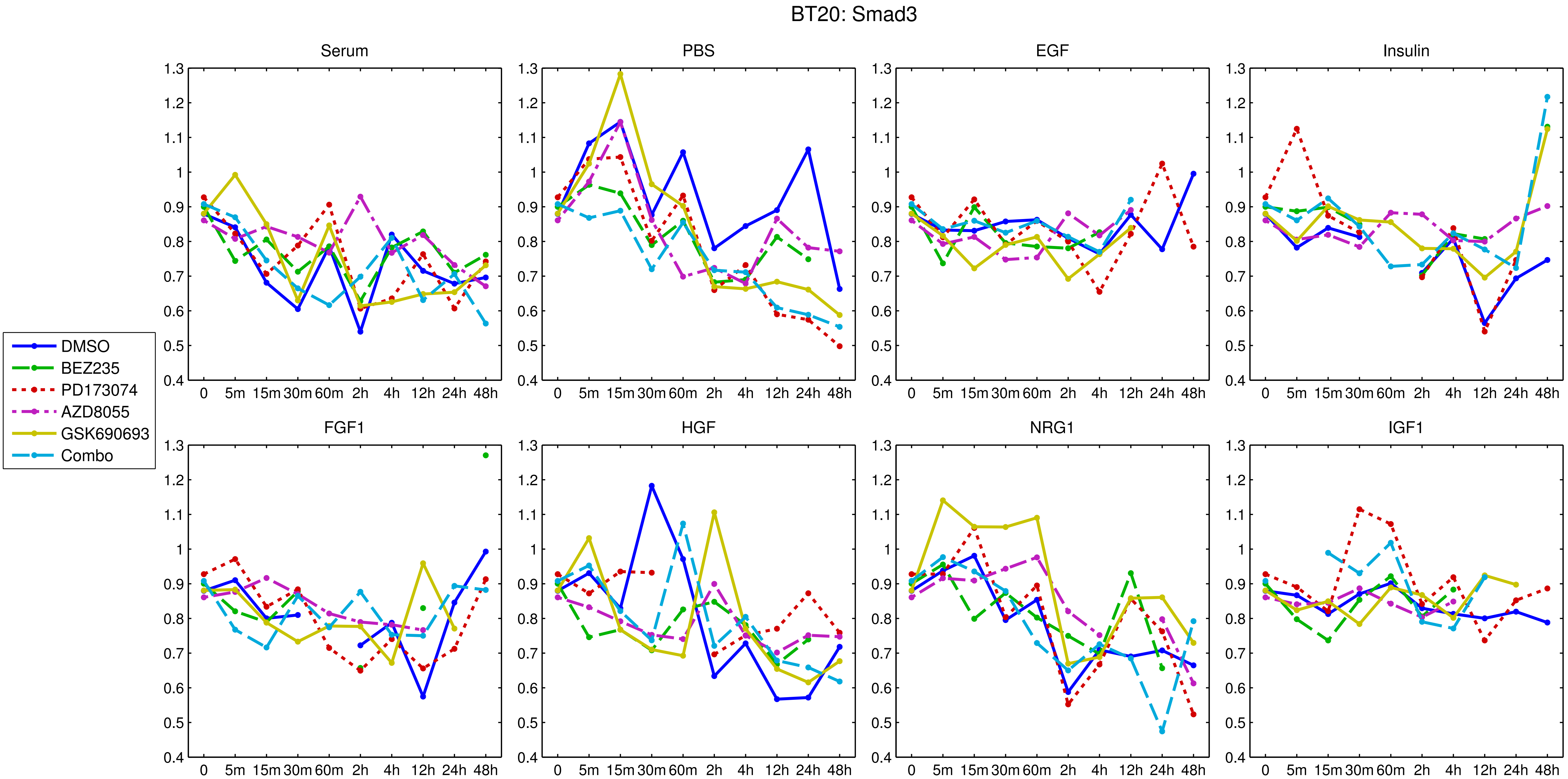
BT20: Smad3
Serum
PBS
EGF
Insulin
FGF1
HGF
NRG1
IGF1
DMSO
BEZ235
PD173074
AZD8055
GSK690693
Combo
0
5m
15m
30m
60m
2h
4h
12h
24h
48h
0.4
0.5
0.6
0.7
0.8
0.9
1
1.1
1.2
1.3

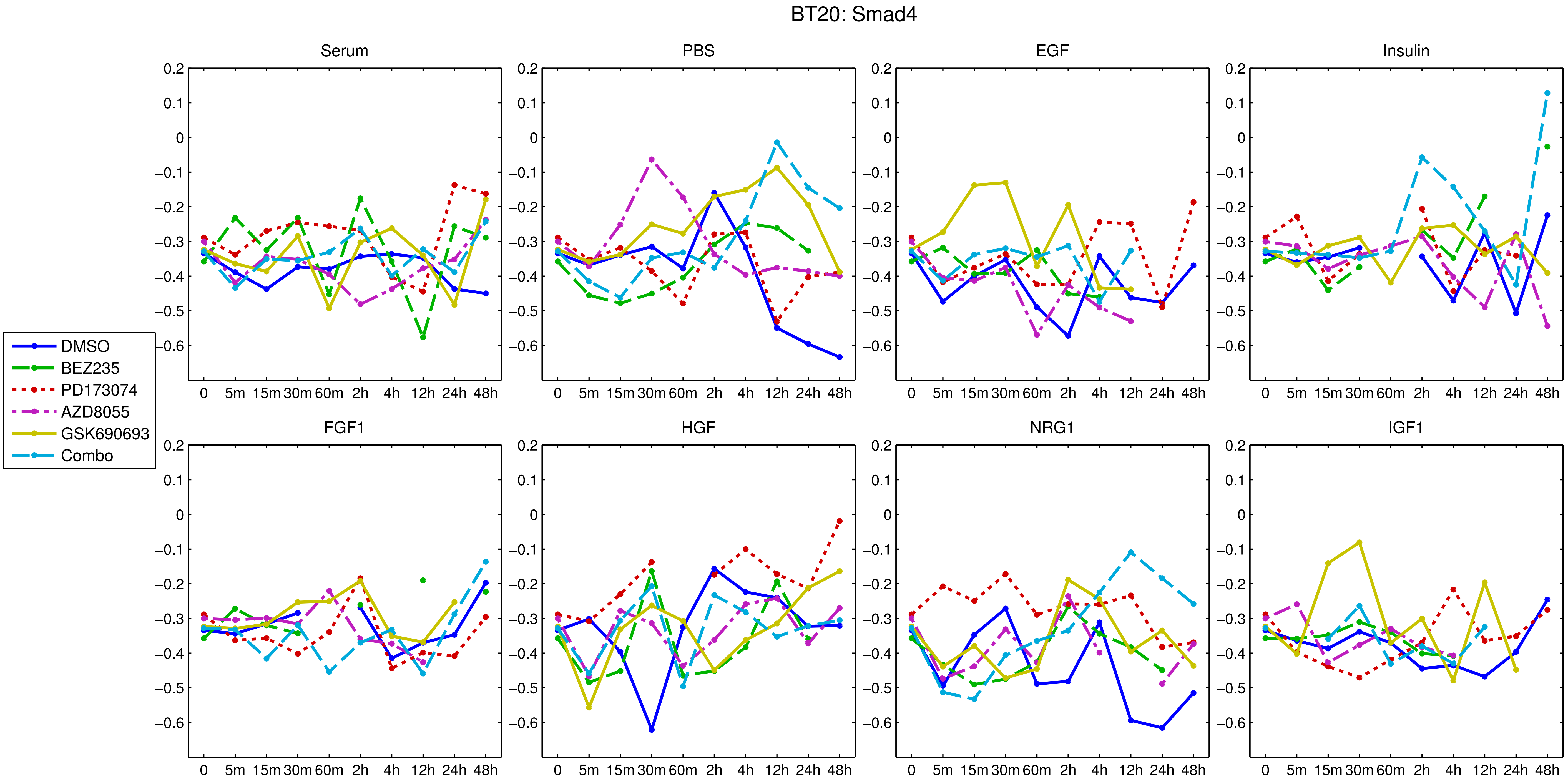
BT20: Smad4
Serum
PBS
EGF
Insulin
FGF1
HGF
NRG1
IGF1
DMSO
BEZ235
PD173074
AZD8055
GSK690693
Combo
0.2
0.1
0
−0.1
−0.2
−0.3
−0.4
−0.5
−0.6
0
5m
15m
30m
60m
2h
4h
12h
24h
48h

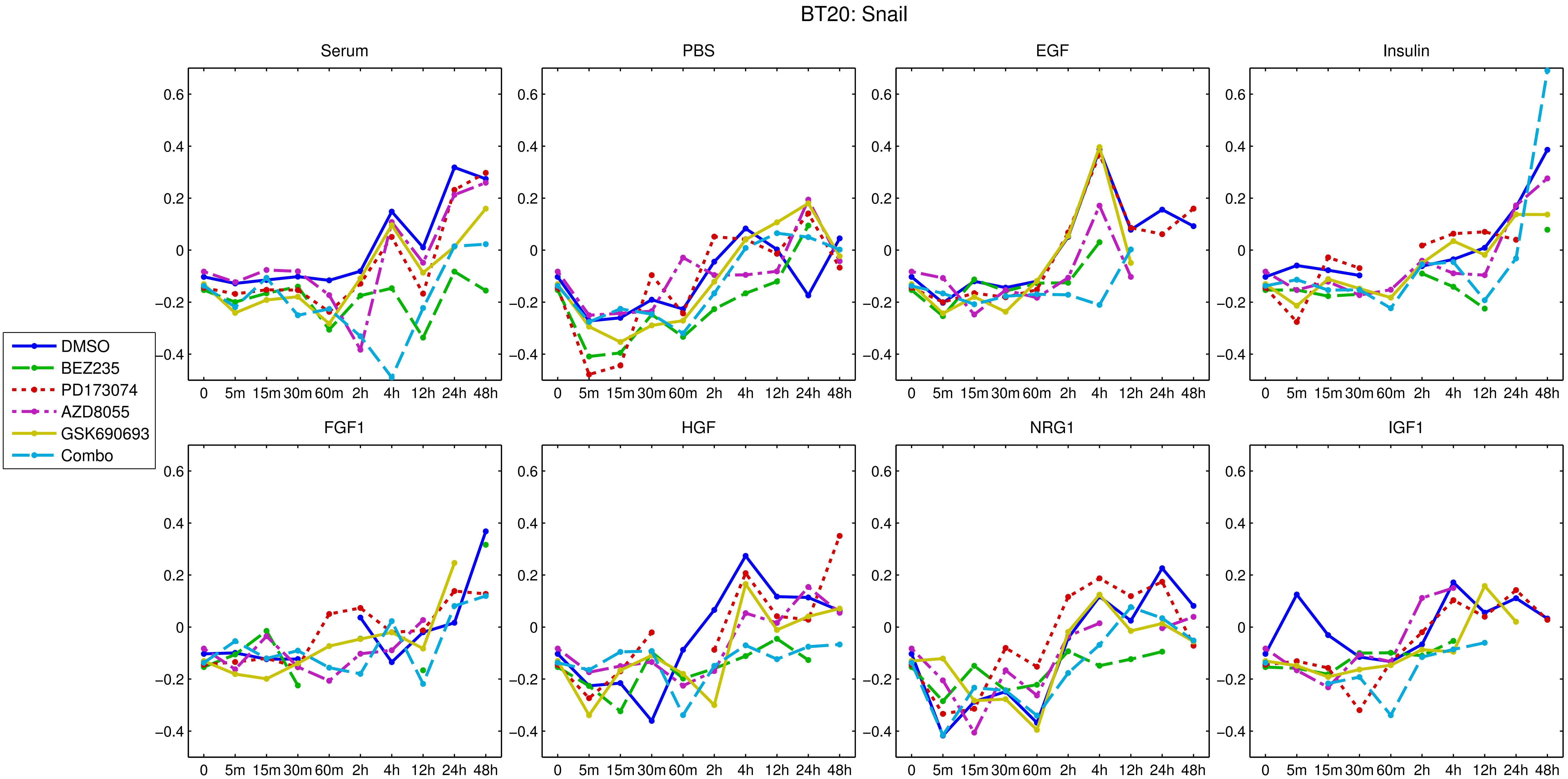

BT20: Snail
Serum
PBS
EGF
Insulin
FGF1
HGF
NRG1
IGF1
DMSO
BEZ235
PD173074
AZD8055
GSK690693
Combo
0.6
0.4
0.2
0
−0.2
−0.4
0
5m
15m
30m
60m
2h
4h
12h
24h
48h

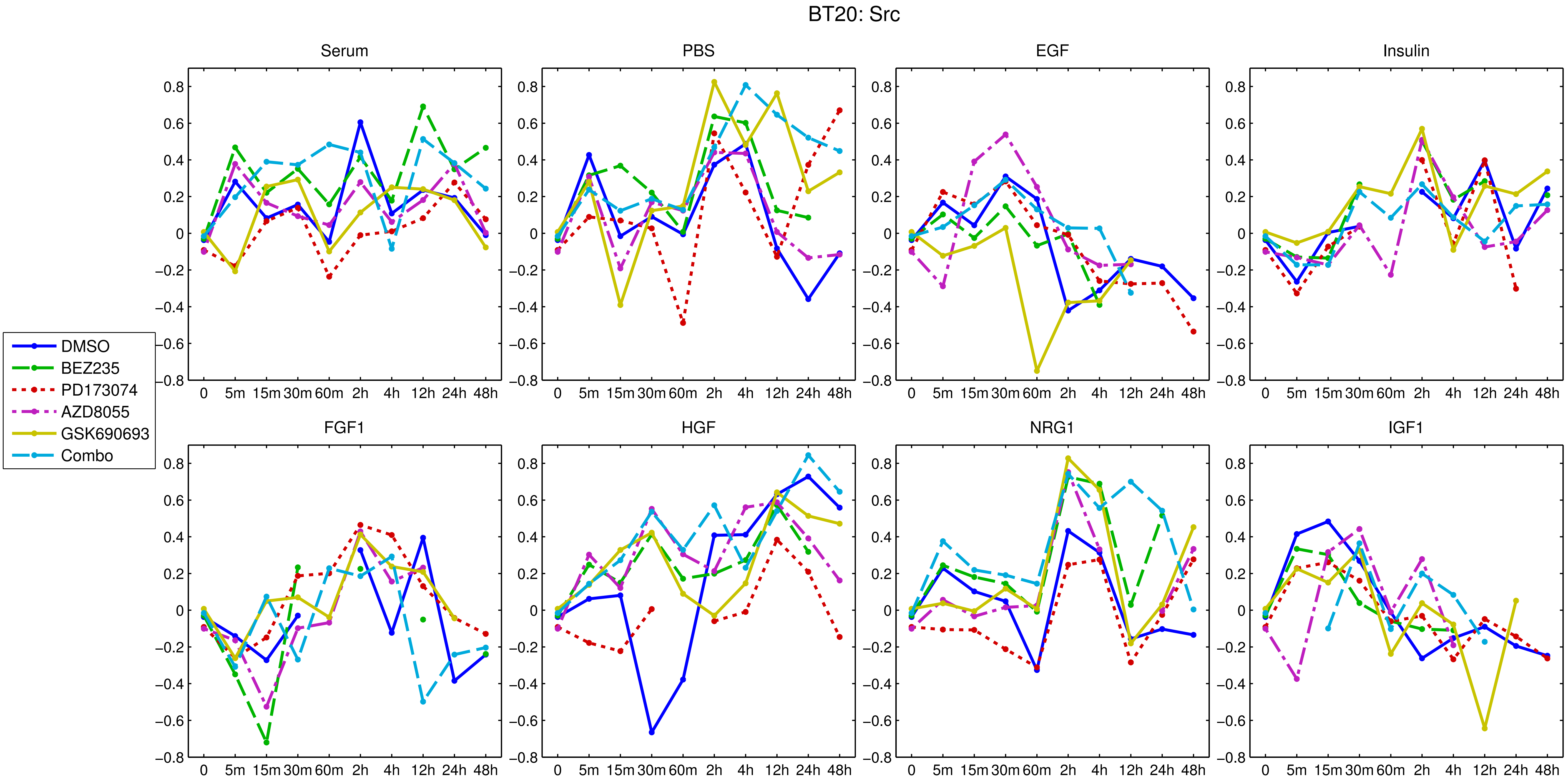
BT20: Src
Serum
PBS
EGF
Insulin
FGF1
HGF
NRG1
IGF1
DMSO
BEZ235
PD173074
AZD8055
GSK690693
Combo
0.8
0.6
0.4
0.2
0
−0.2
−0.4
−0.6
−0.8
0
5m
15m
30m
60m
2h
4h
12h
24h
48h

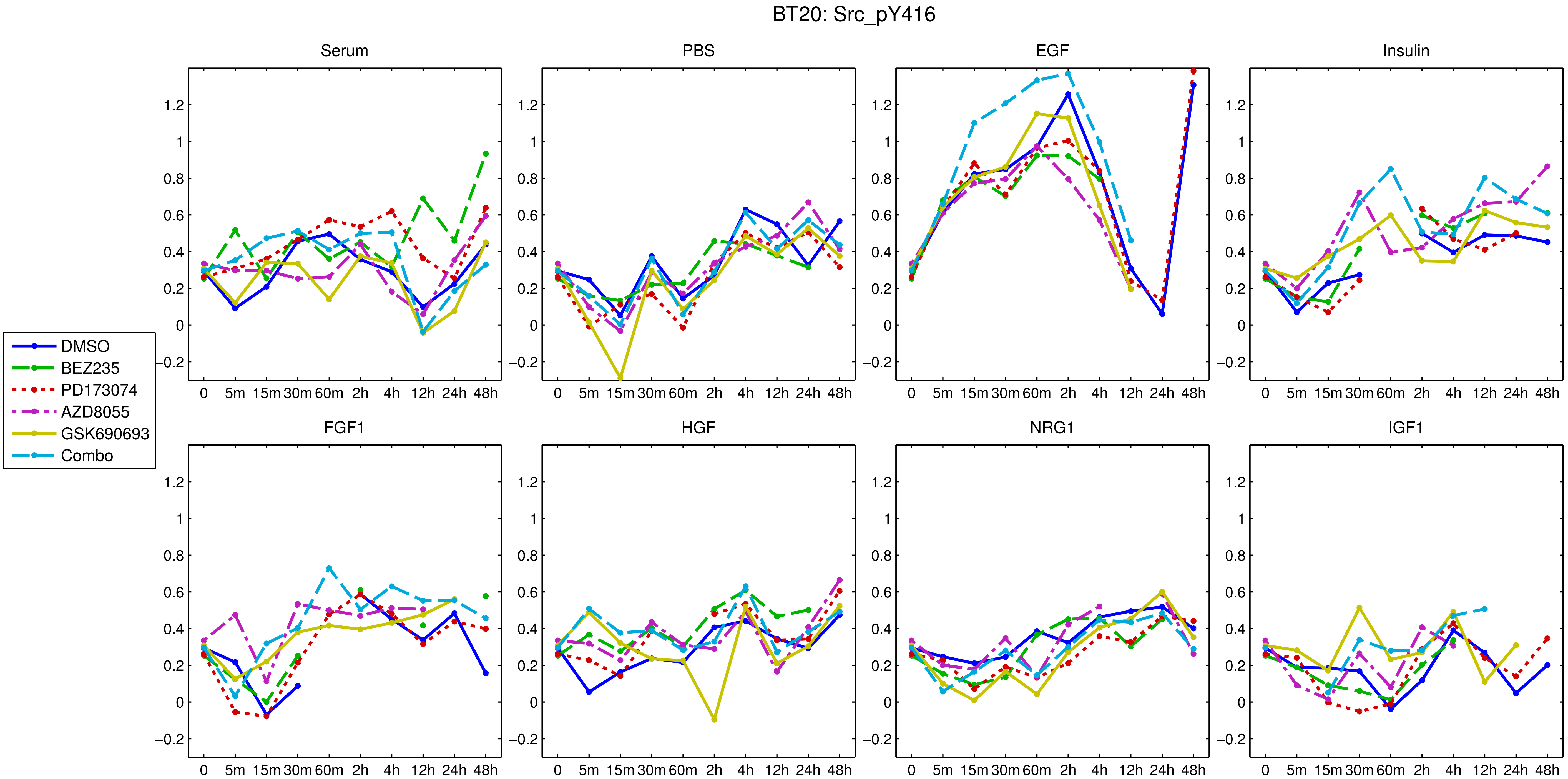

BT20: Src_pY416
Serum
PBS
EGF
Insulin
FGF1
HGF
NRG1
IGF1
DMSO
BEZ235
PD173074
AZD8055
GSK690693
Combo
0 5m 15m 30m 60m 2h 4h 12h 24h 48h

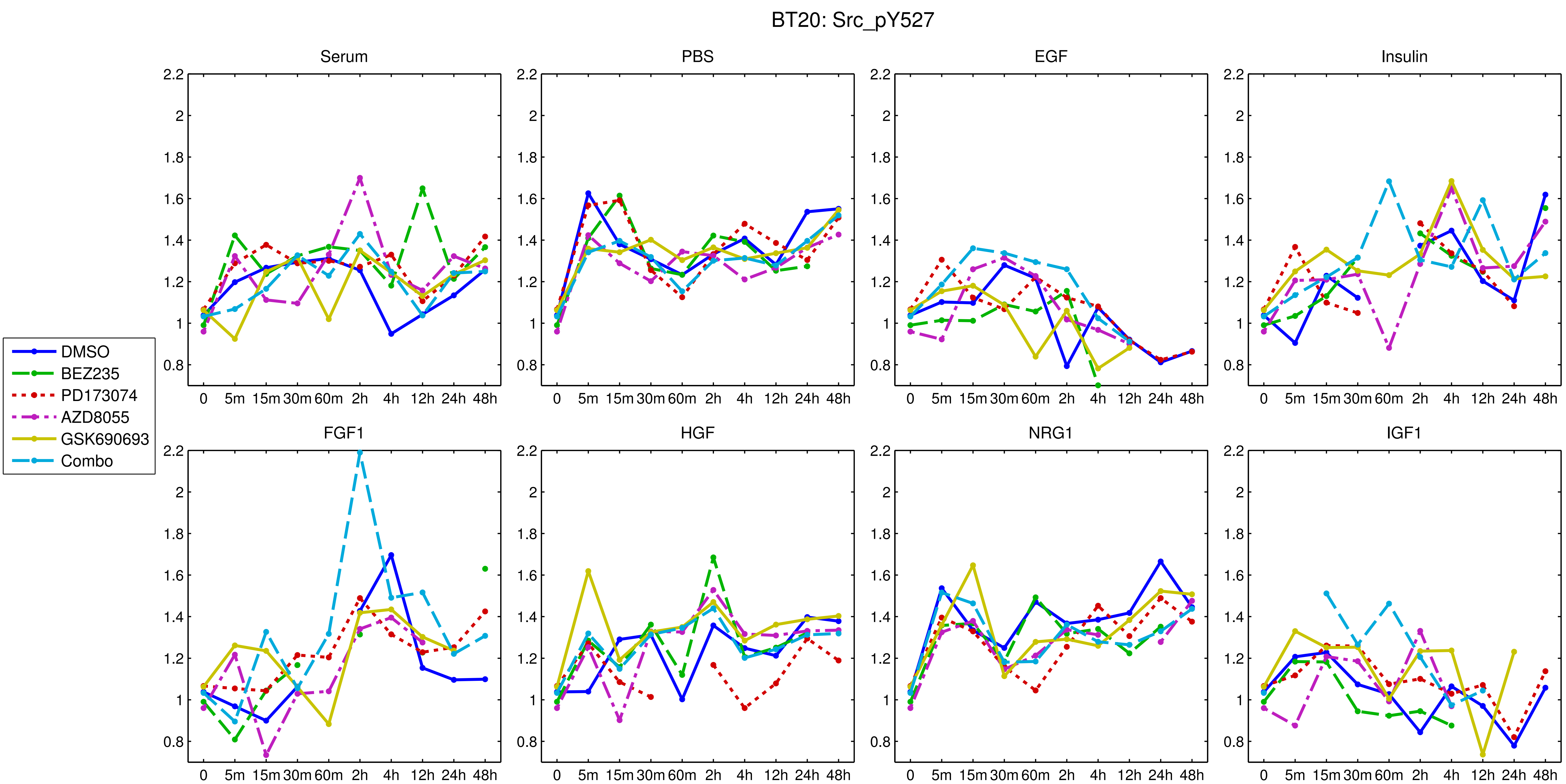
BT20: Src_pY527
Serum
PBS
EGF
Insulin
FGF1
HGF
NRG1
IGF1
DMSO
BEZ235
PD173074
AZD8055
GSK690693
Combo
2.2
2
1.8
1.6
1.4
1.2
1
0.8
0
5m
15m
30m
60m
2h
4h
12h
24h
48h

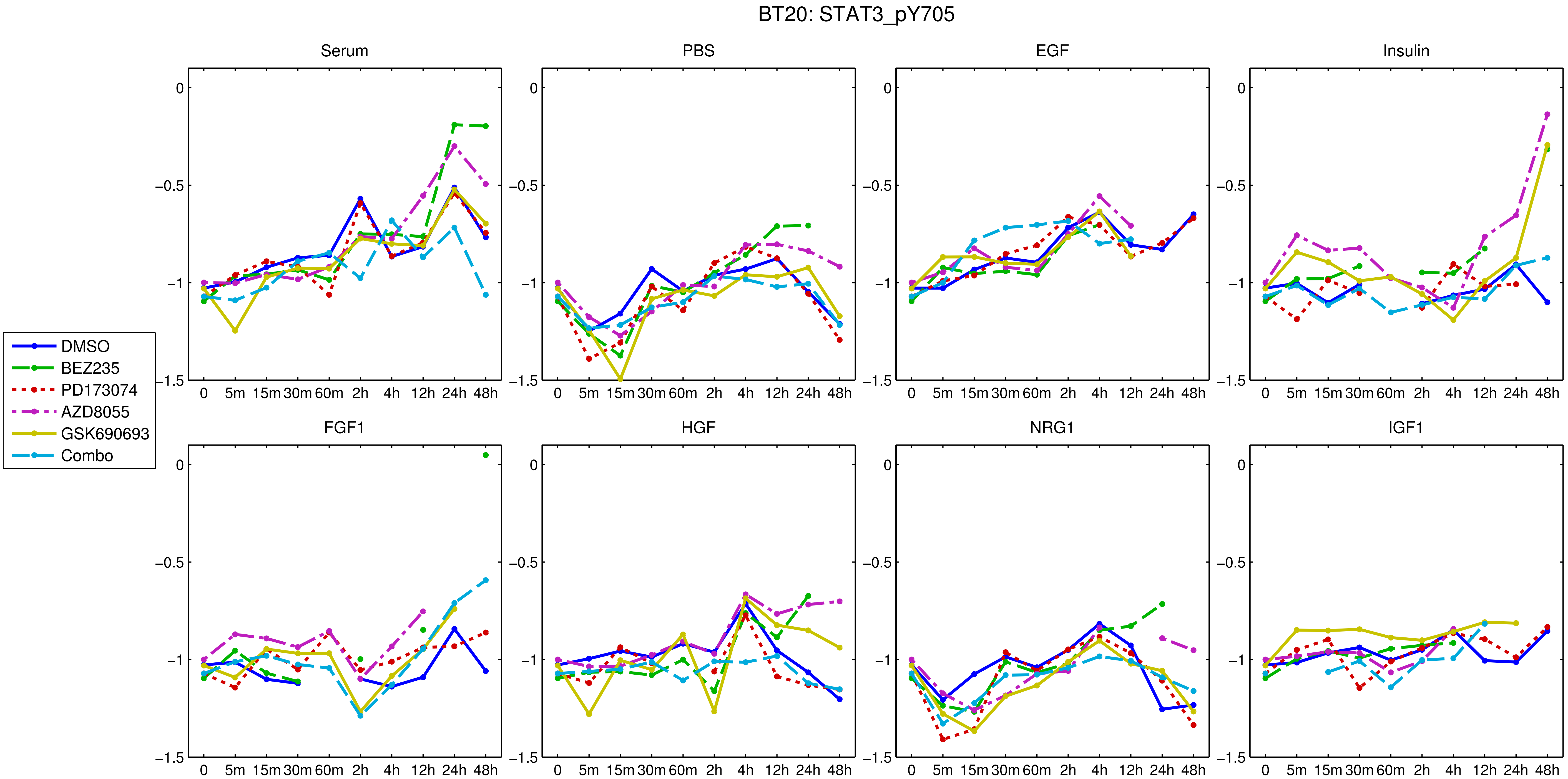
BT20: STAT3_pY705
Serum
PBS
EGF
Insulin
FGF1
HGF
NRG1
IGF1
DMSO
BEZ235
PD173074
AZD8055
GSK690693
Combo
0
−0.5
−1
−1.5
0 5m 15m 30m 60m 2h 4h 12h 24h 48h

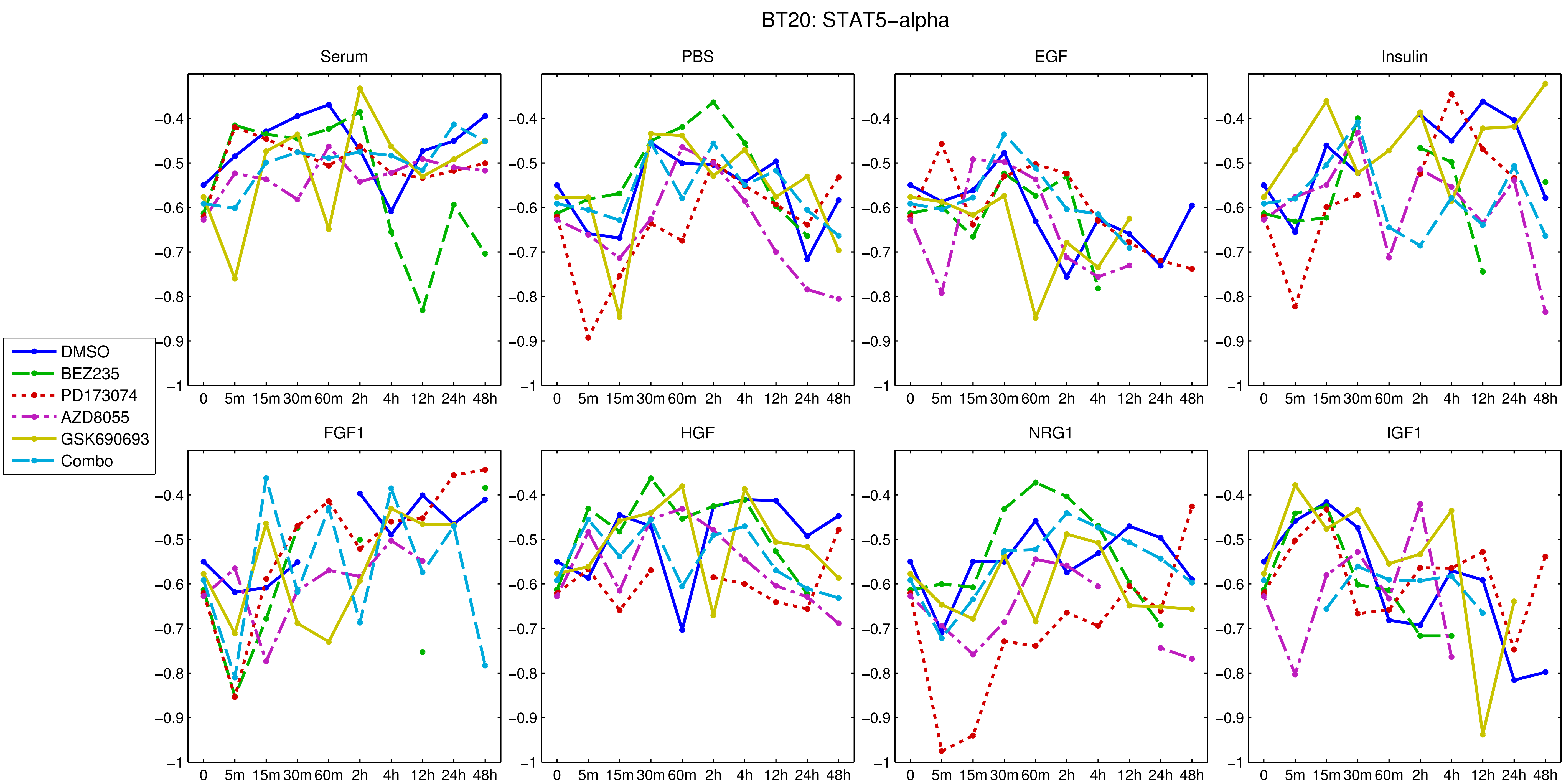

BT20: STAT5−alpha
Serum
PBS
EGF
Insulin
FGF1
HGF
NRG1
IGF1
DMSO
BEZ235
PD173074
AZD8055
GSK690693
Combo
−0.4
−0.5
−0.6
−0.7
−0.8
−0.9
−1
0
5m
15m
30m
60m
2h
4h
12h
24h
48h

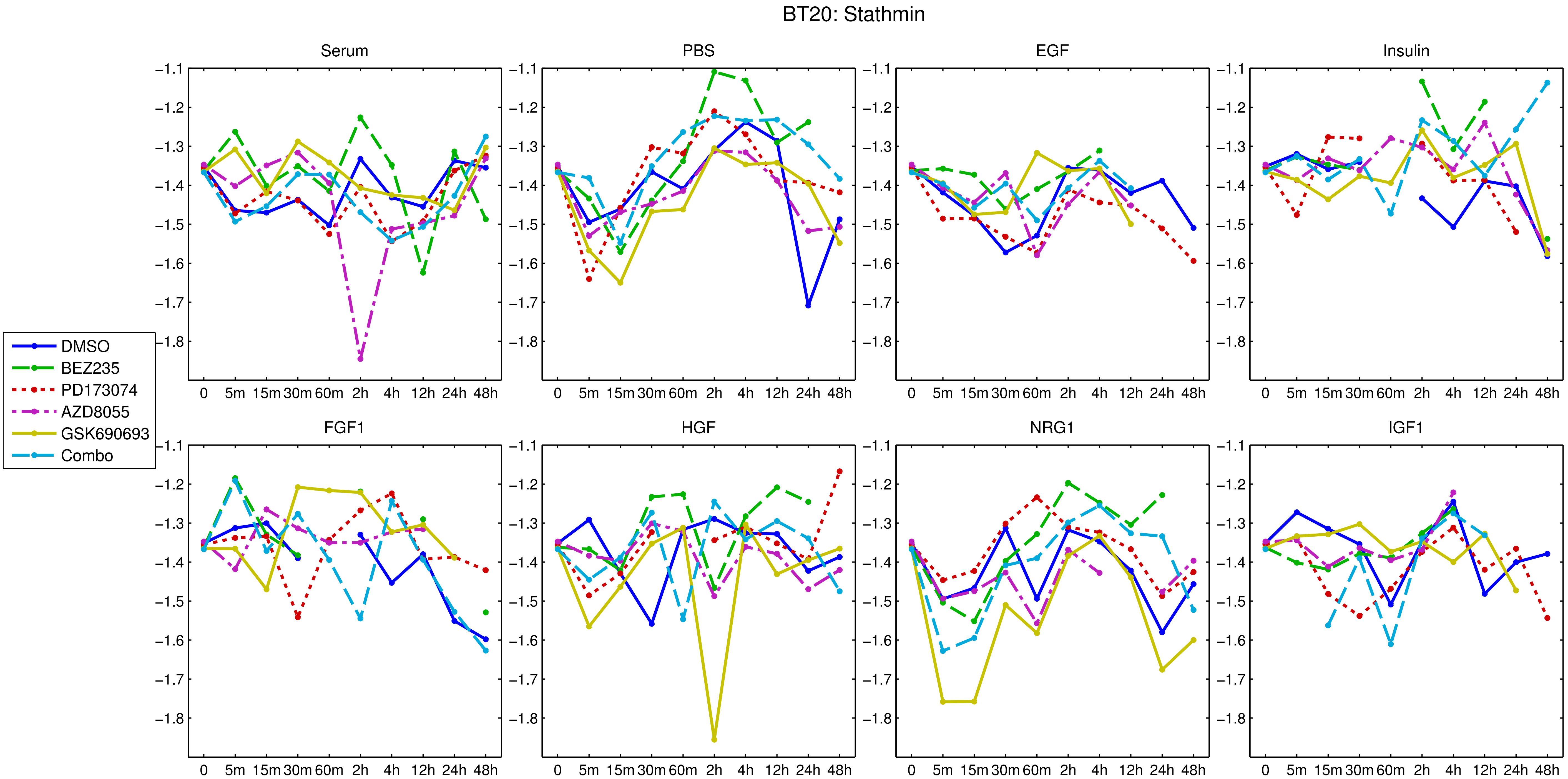

BT20: Stathmin
Serum
PBS
EGF
Insulin
FGF1
HGF
NRG1
IGF1
DMSO
BEZ235
PD173074
AZD8055
GSK690693
Combo
−1.1
−1.2
−1.3
−1.4
−1.5
−1.6
−1.7
−1.8
0
5m
15m
30m
60m
2h
4h
12h
24h
48h

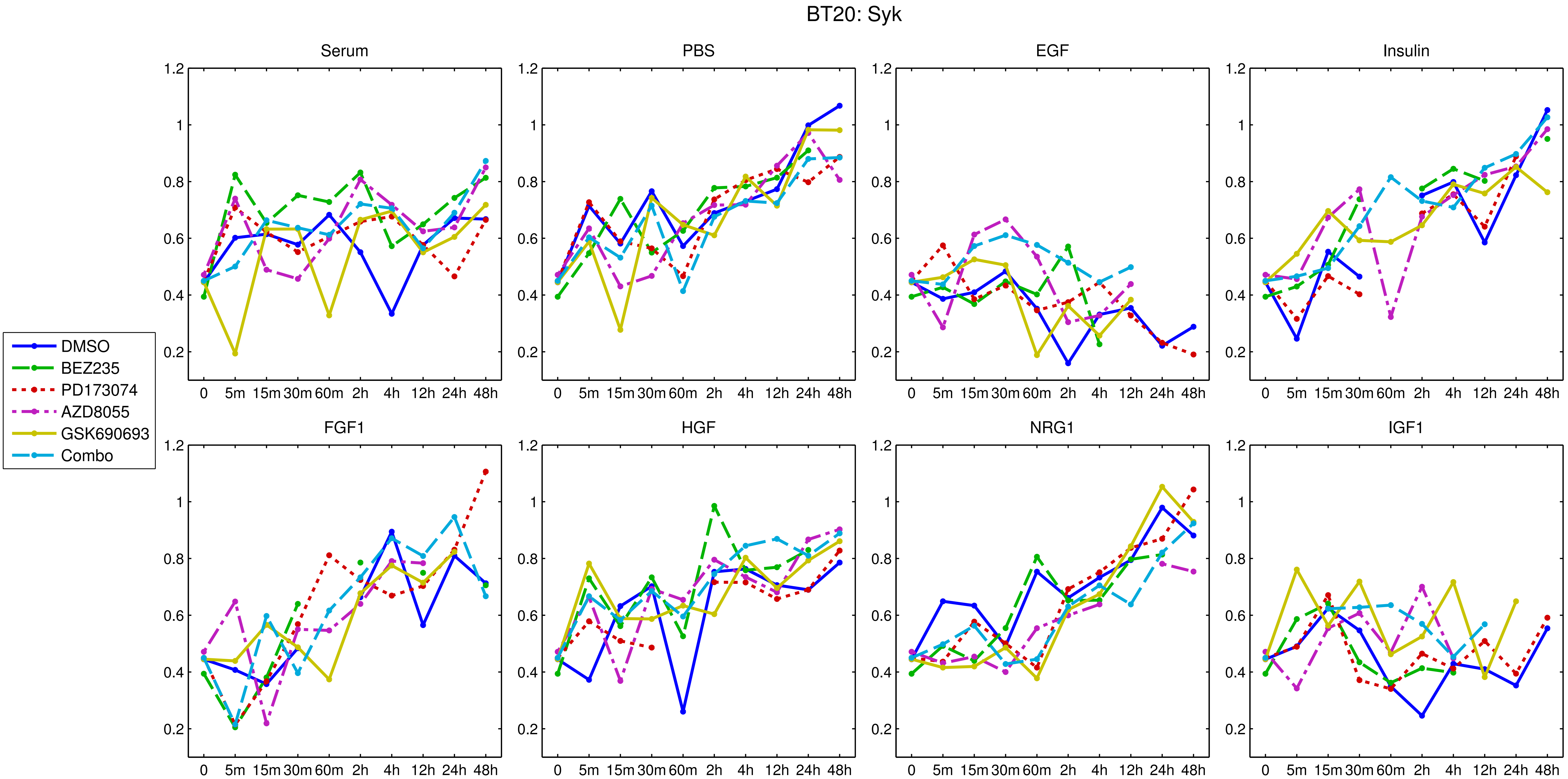
BT20: Syk
Serum
PBS
EGF
Insulin
FGF1
HGF
NRG1
IGF1
1.2
1
0.8
0.6
0.4
0.2
0
5m
15m
30m
60m
2h
4h
12h
24h
48h
DMSO
BEZ235
PD173074
AZD8055
GSK690693
Combo

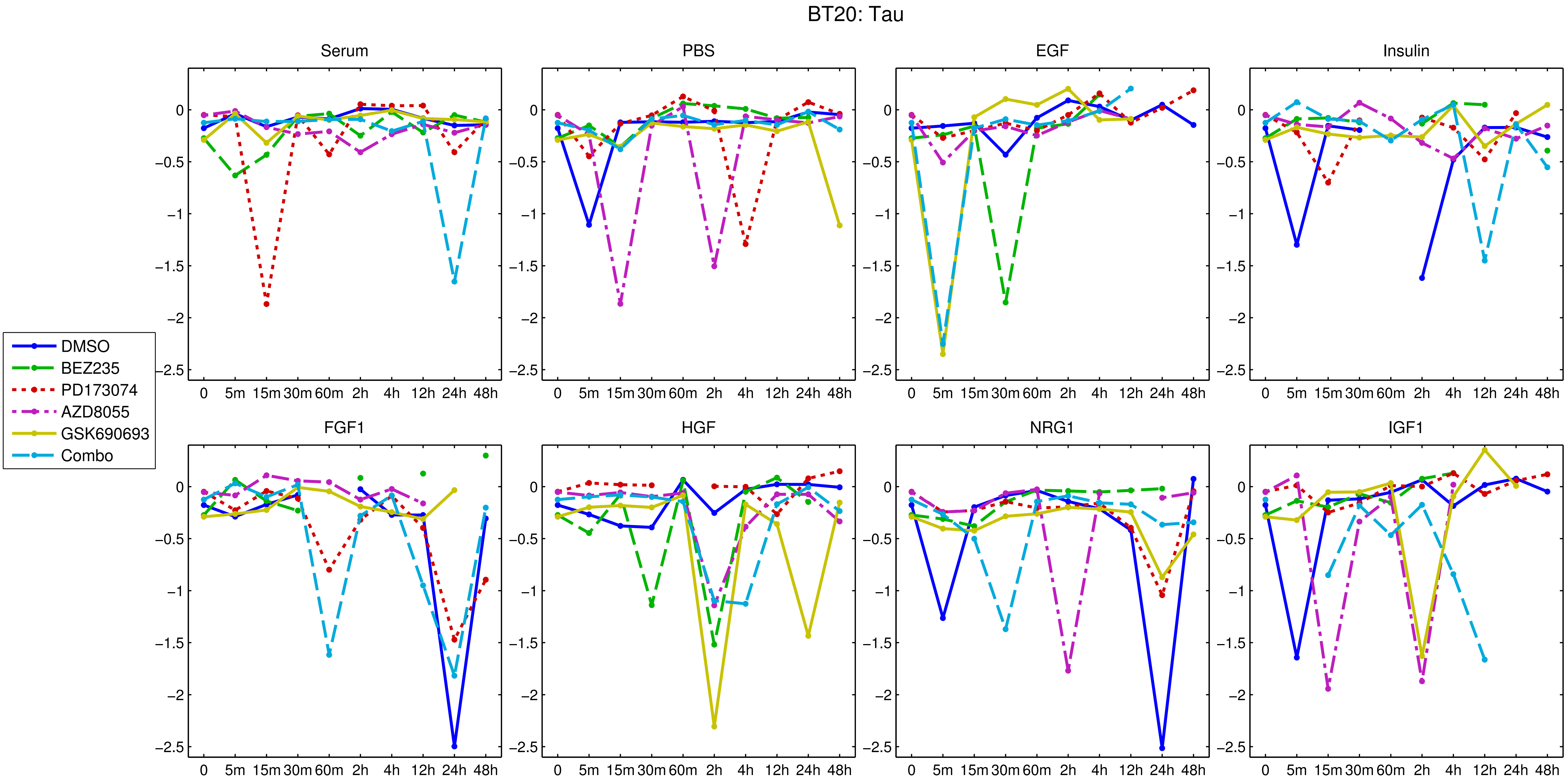
BT20: Tau
Serum
PBS
EGF
Insulin
FGF1
HGF
NRG1
IGF1
0
−0.5
−1
−1.5
−2
−2.5
0 5m 15m 30m 60m 2h 4h 12h 24h 48h
DMSO
BEZ235
PD173074
AZD8055
GSK690693
Combo

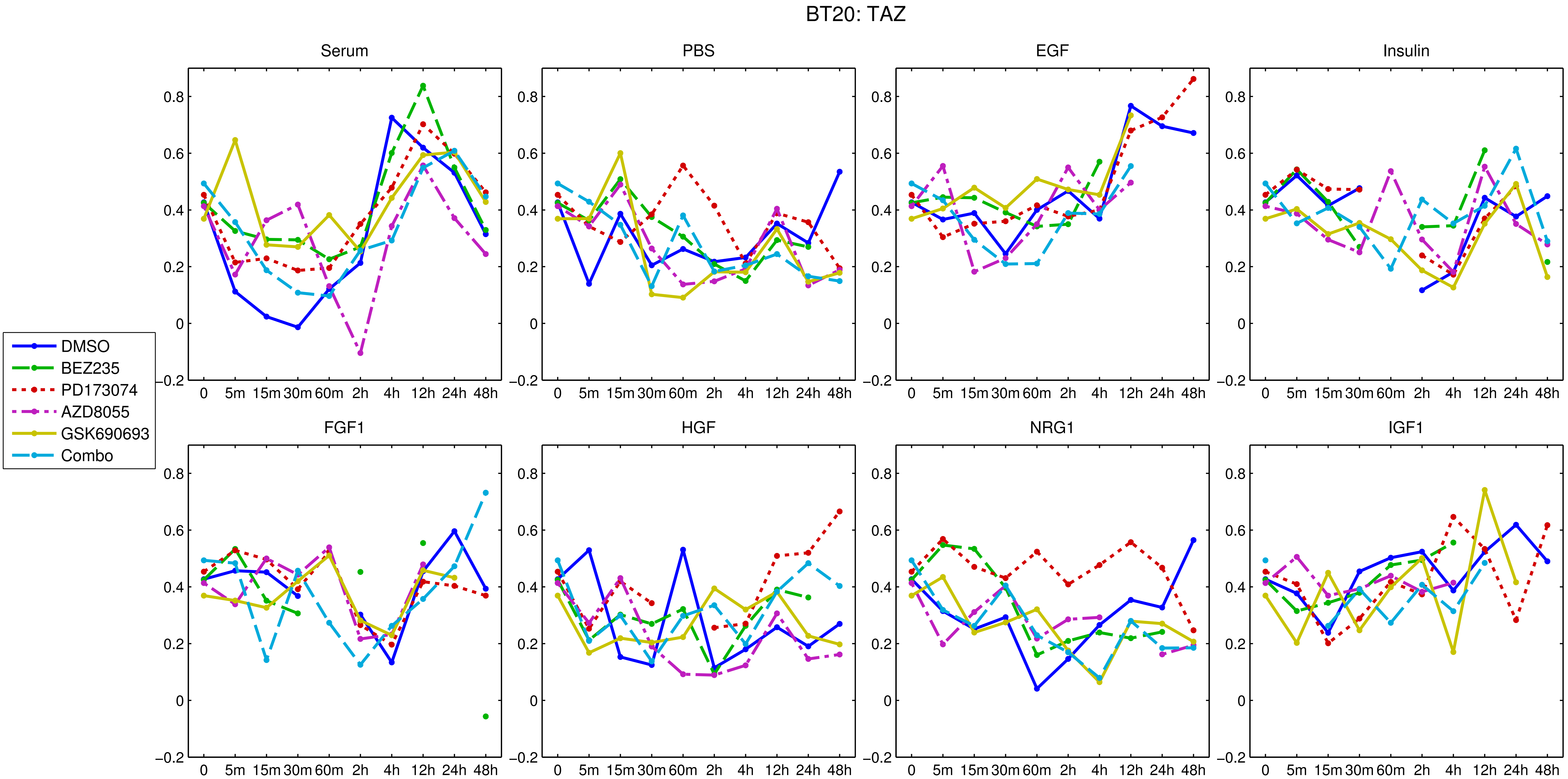

BT20: TAZ
Serum
PBS
EGF
Insulin
FGF1
HGF
NRG1
IGF1
DMSO
BEZ235
PD173074
AZD8055
GSK690693
Combo
0.8
0.6
0.4
0.2
0
−0.2
0
5m
15m
30m
60m
2h
4h
12h
24h
48h

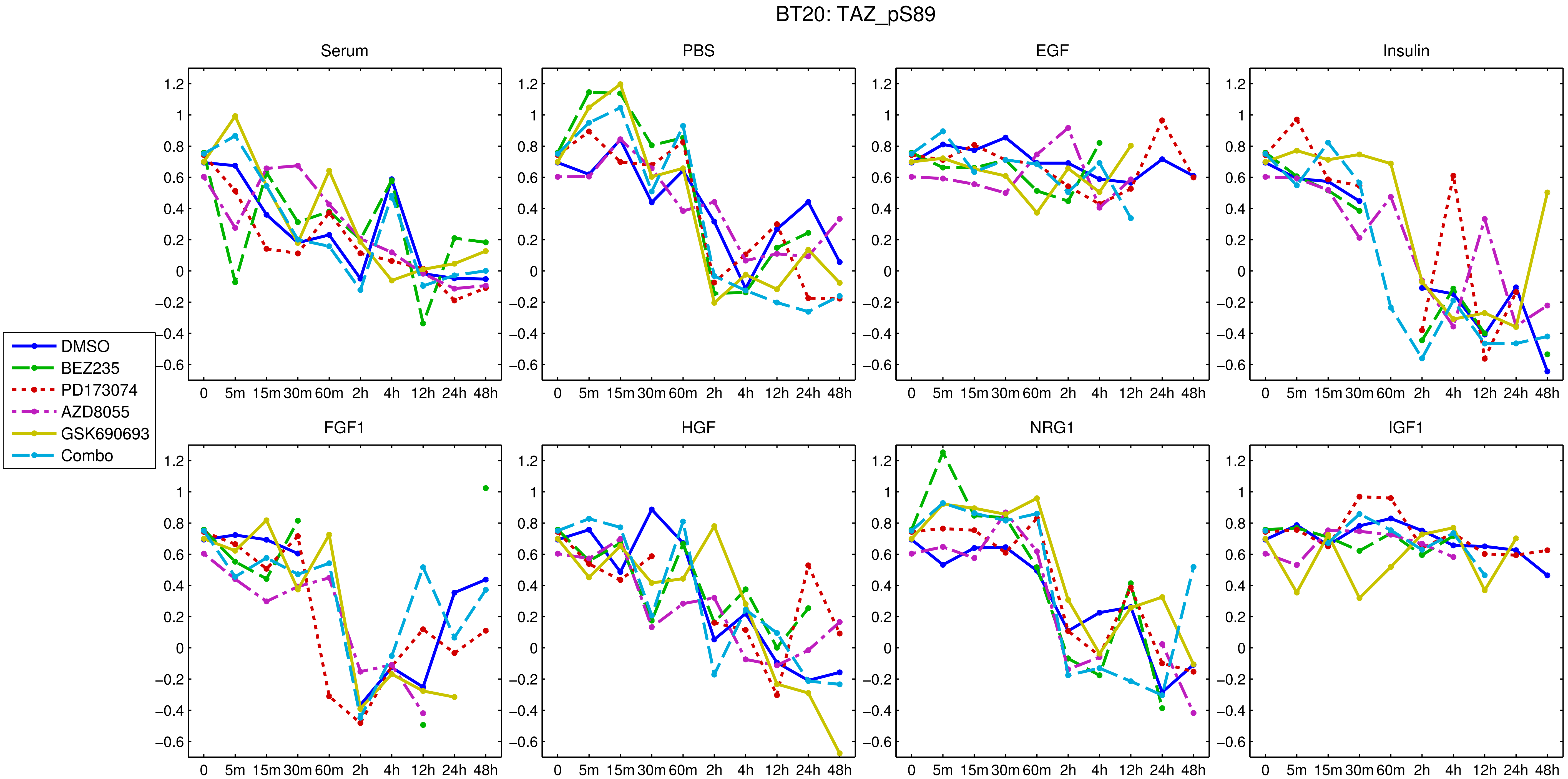
BT20: TAZ_pS89
Serum
PBS
EGF
Insulin
FGF1
HGF
NRG1
IGF1
DMSO
BEZ235
PD173074
AZD8055
GSK690693
Combo
1.2
1
0.8
0.6
0.4
0.2
0
−0.2
−0.4
−0.6
0
5m
15m
30m
60m
2h
4h
12h
24h
48h

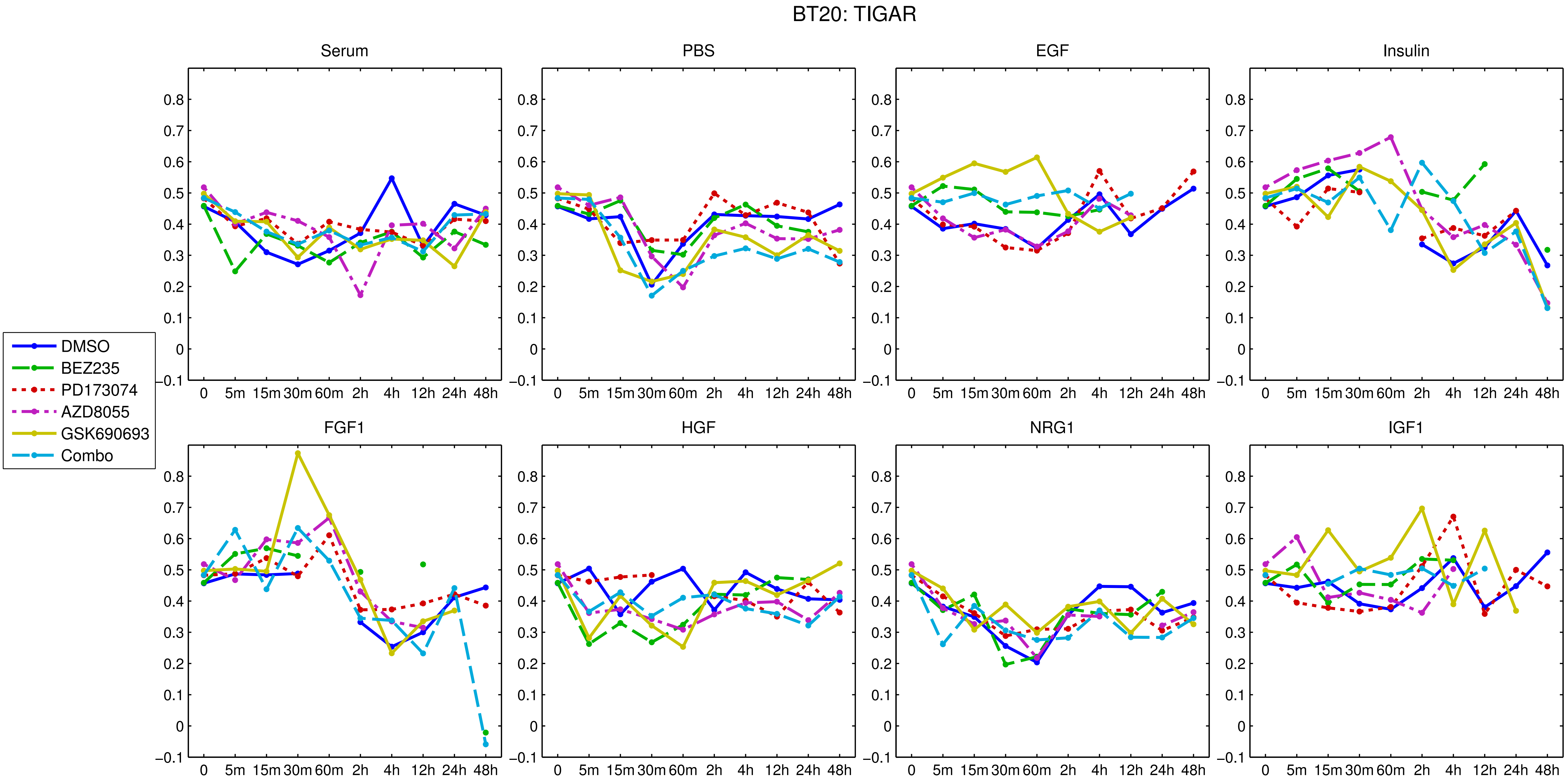
BT20: TIGAR
Serum
PBS
EGF
Insulin
FGF1
HGF
NRG1
IGF1
DMSO
BEZ235
PD173074
AZD8055
GSK690693
Combo
0.8
0.7
0.6
0.5
0.4
0.3
0.2
0.1
0
−0.1
0
5m
15m
30m
60m
2h
4h
12h
24h
48h

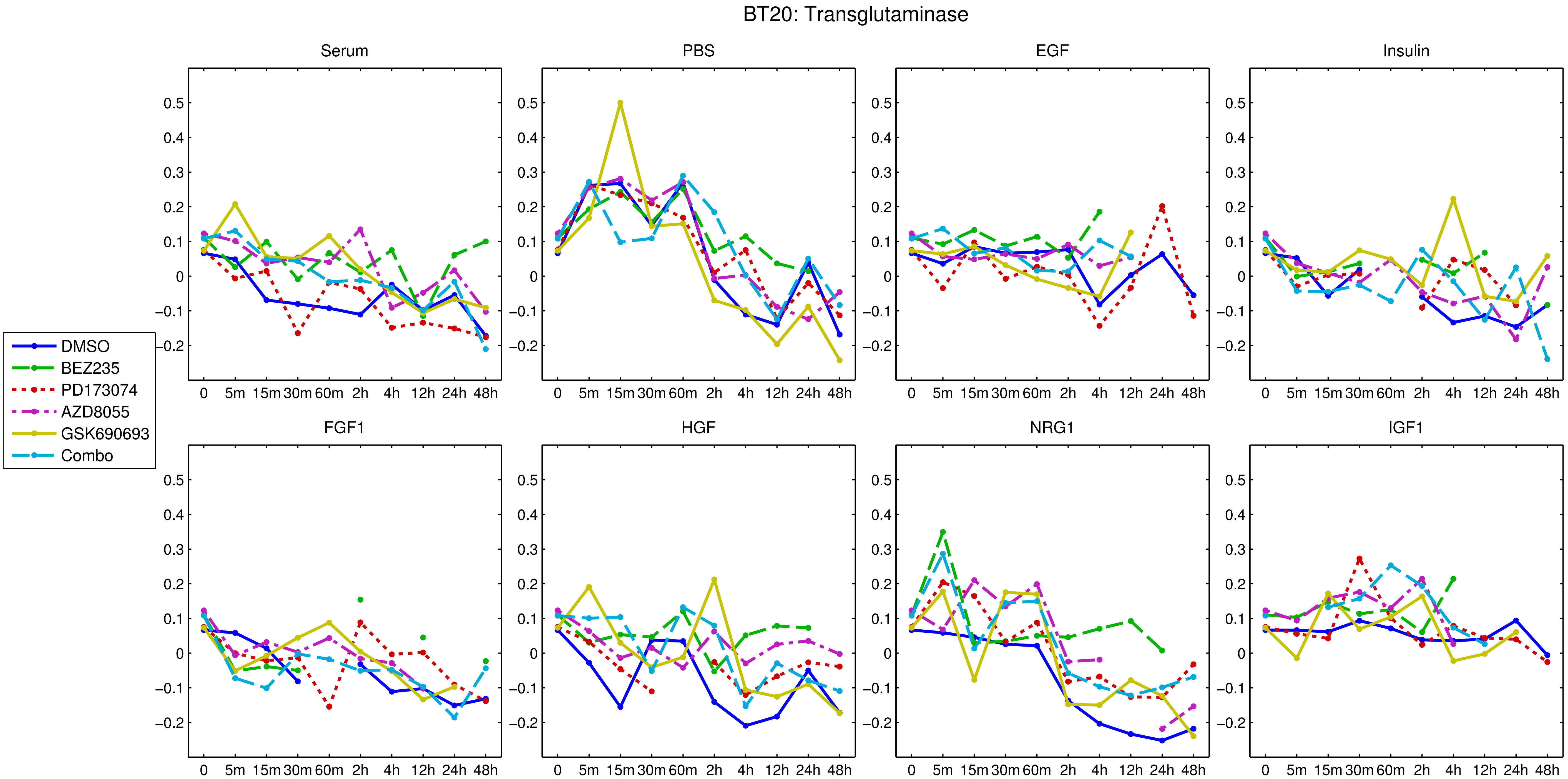

BT20: Transglutaminase
Serum
PBS
EGF
Insulin
FGF1
HGF
NRG1
IGF1
0.5
0.4
0.3
0.2
0.1
0
−0.1
−0.2
0 5m 15m 30m 60m 2h 4h 12h 24h 48h
DMSO
BEZ235
PD173074
AZD8055
GSK690693
Combo

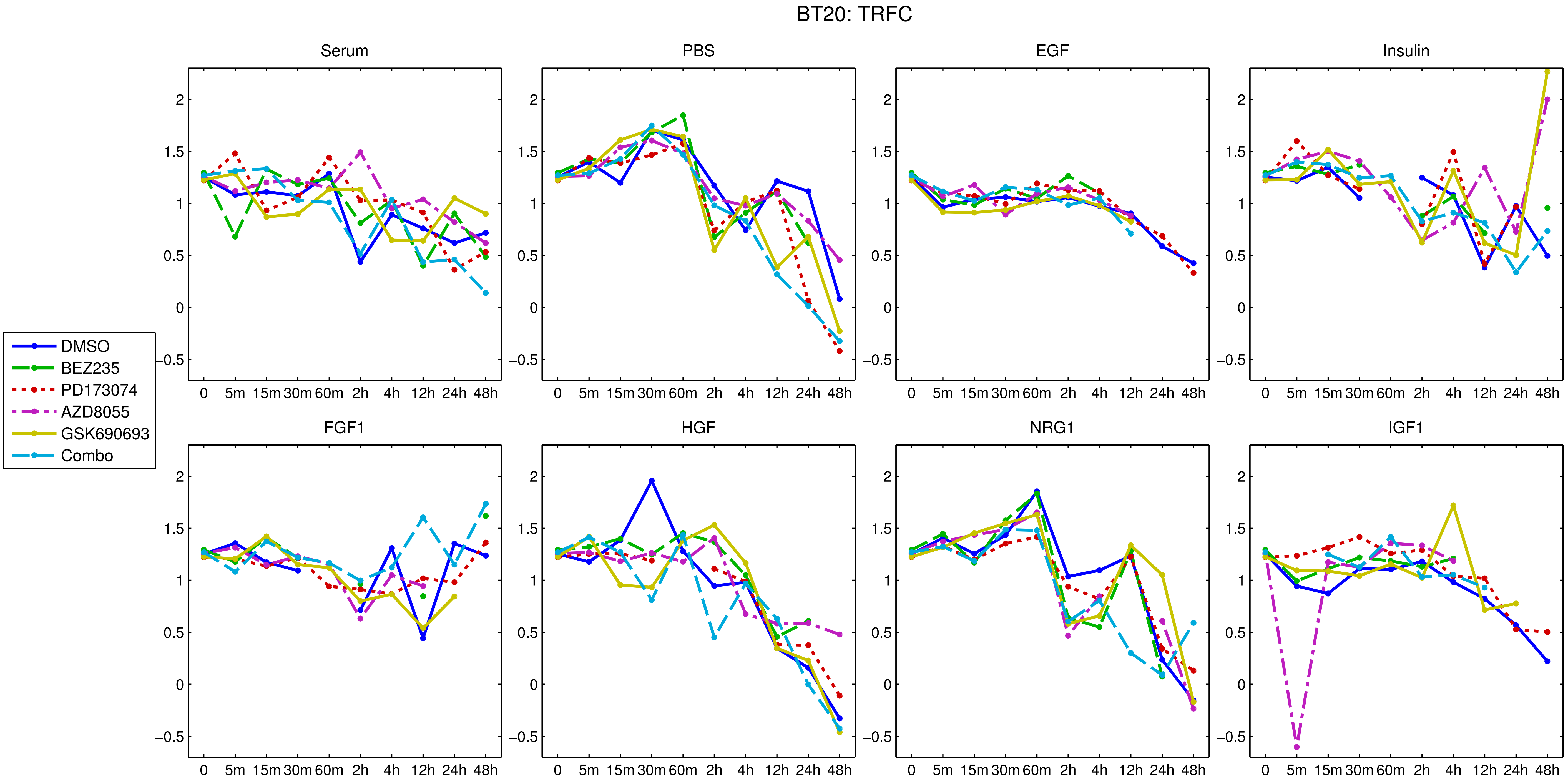

BT20: TRFC
Serum
PBS
EGF
Insulin
FGF1
HGF
NRG1
IGF1
DMSO
BEZ235
PD173074
AZD8055
GSK690693
Combo
0
5m
15m
30m
60m
2h
4h
12h
24h
48h
2
1.5
1
0.5
0
−0.5

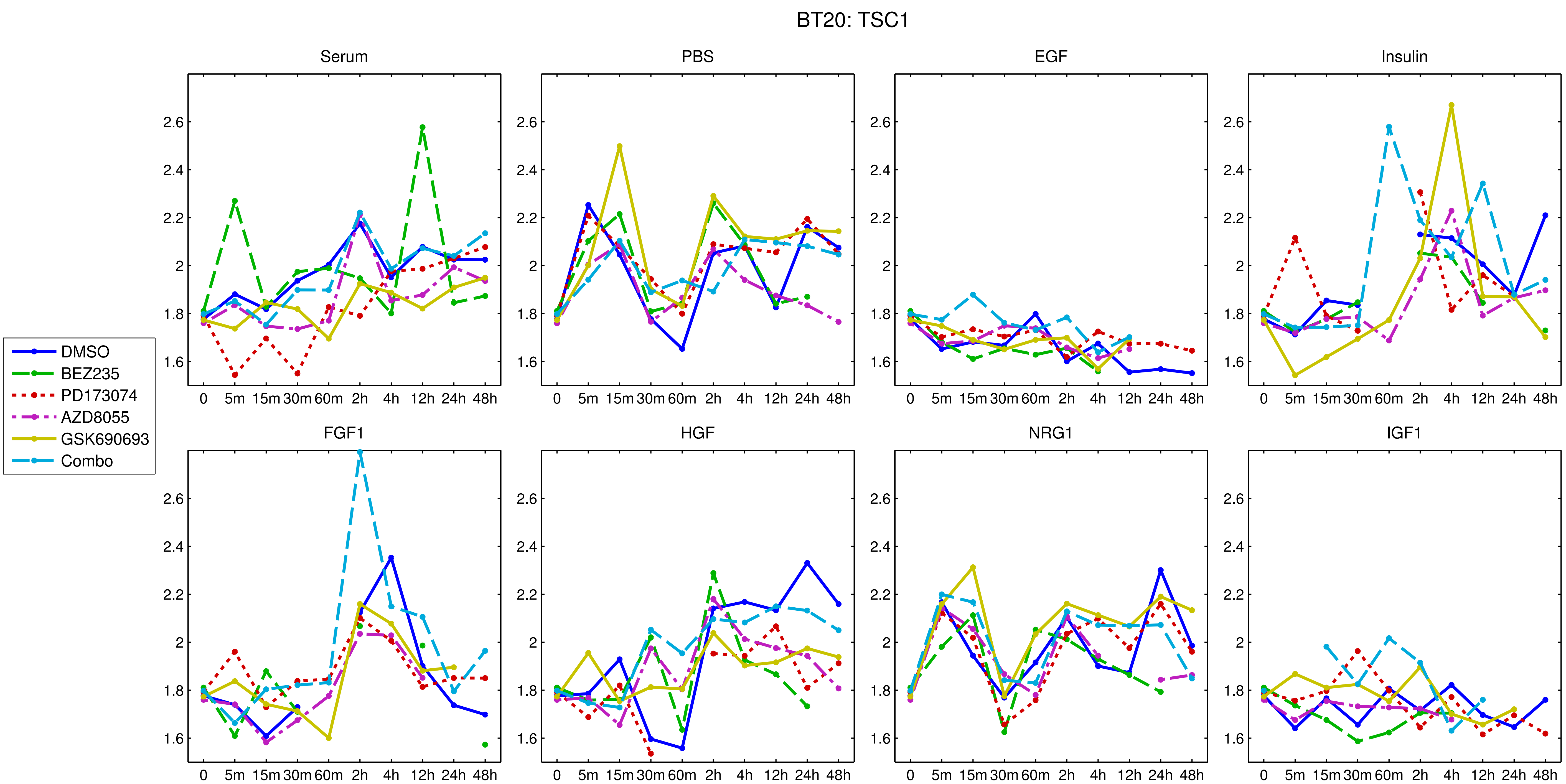
BT20: TSC1
Serum
PBS
EGF
Insulin
FGF1
HGF
NRG1
IGF1
DMSO
BEZ235
PD173074
AZD8055
GSK690693
Combo
2.6
2.4
2.2
2
1.8
1.6
0
5m
15m
30m
60m
2h
4h
12h
24h
48h

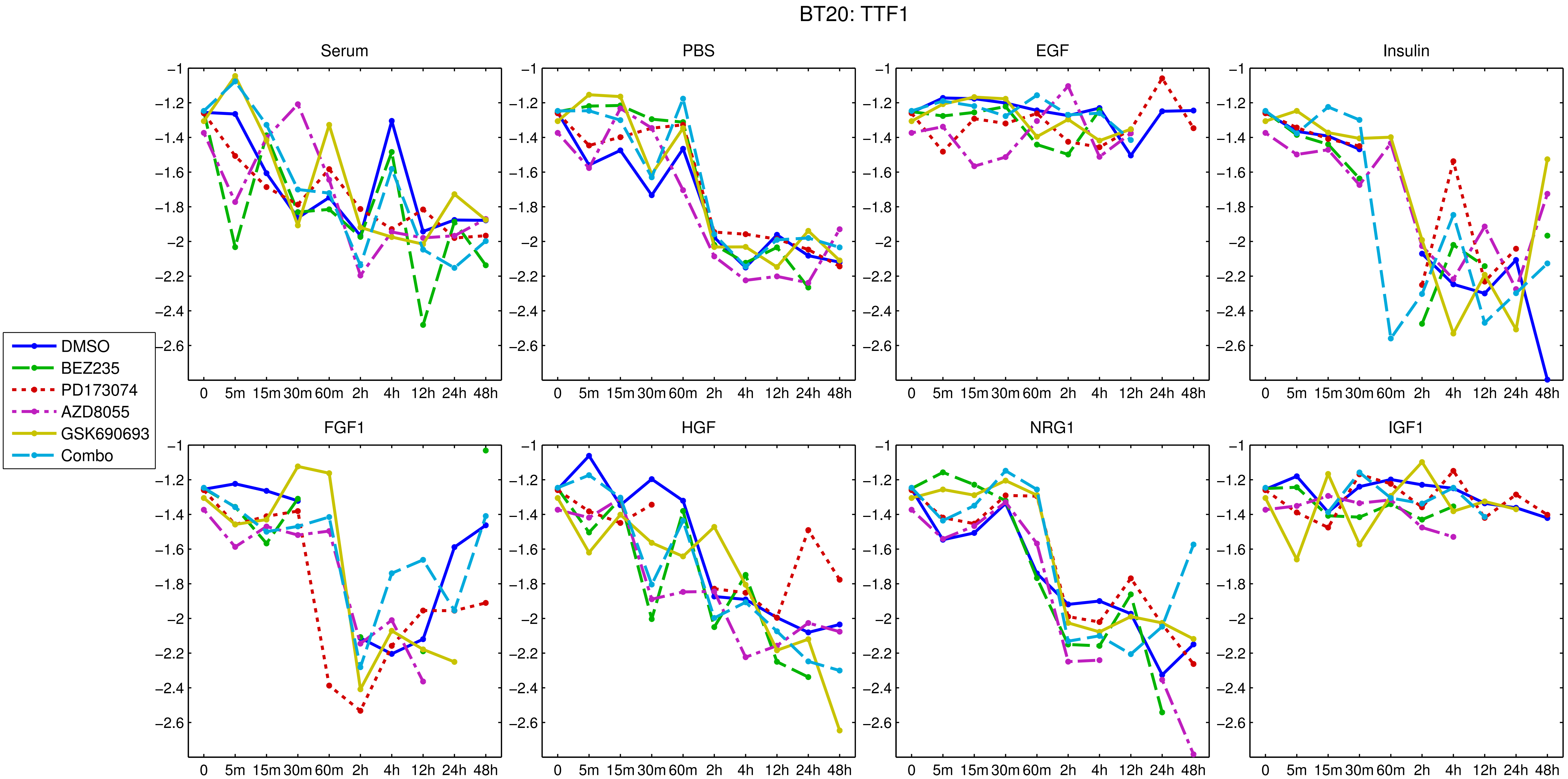
BT20: TTF1
Serum
PBS
EGF
Insulin
FGF1
HGF
NRG1
IGF1
DMSO
BEZ235
PD173074
AZD8055
GSK690693
Combo
−1
−1.2
−1.4
−1.6
−1.8
−2
−2.2
−2.4
−2.6
0
5m
15m
30m
60m
2h
4h
12h
24h
48h

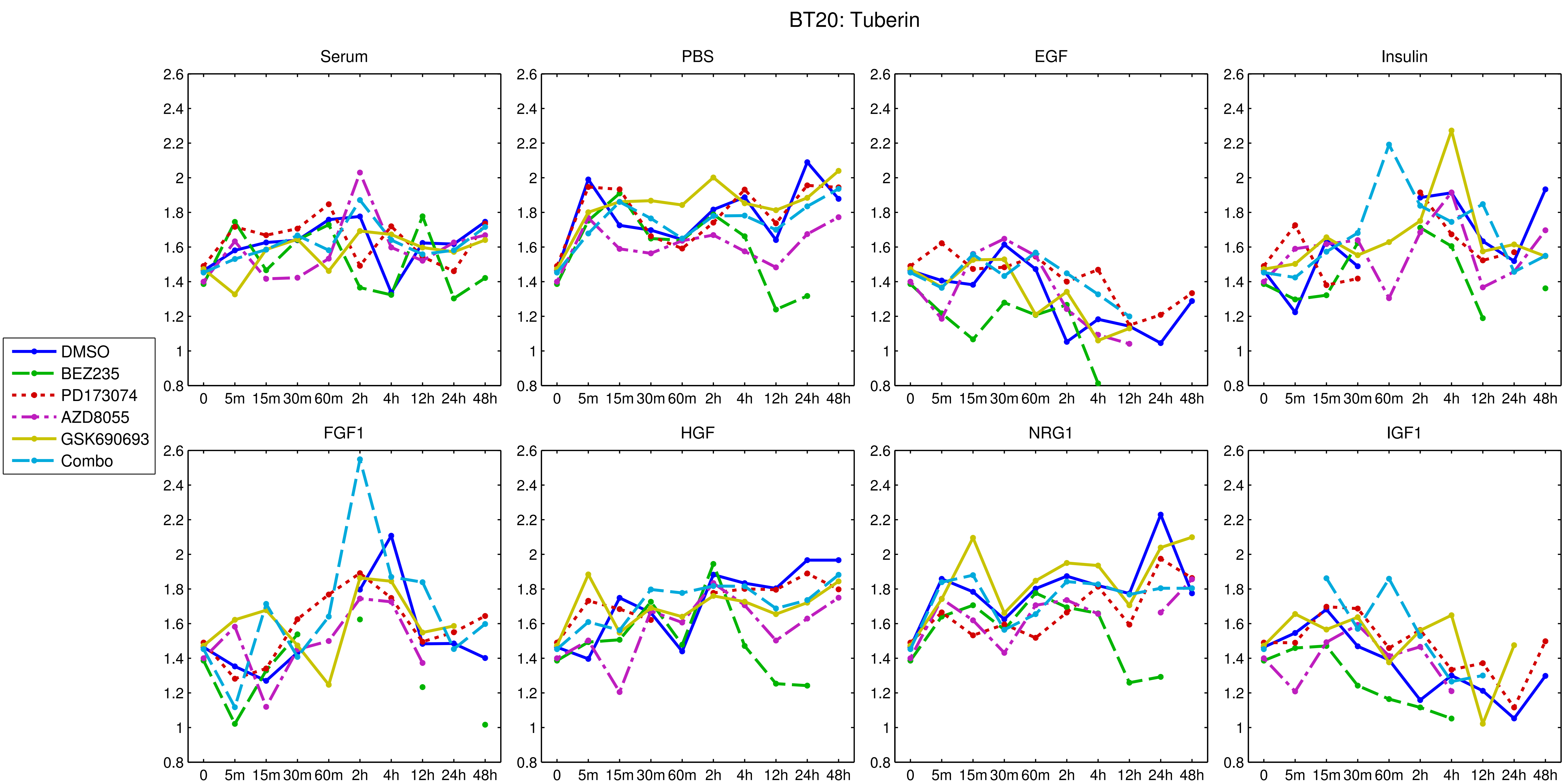

BT20: Tuberin
Serum
PBS
EGF
Insulin
FGF1
HGF
NRG1
IGF1
DMSO
BEZ235
PD173074
AZD8055
GSK690693
Combo
2.6
2.4
2.2
2
1.8
1.6
1.4
1.2
1
0.8
0 5m 15m 30m 60m 2h 4h 12h 24h 48h

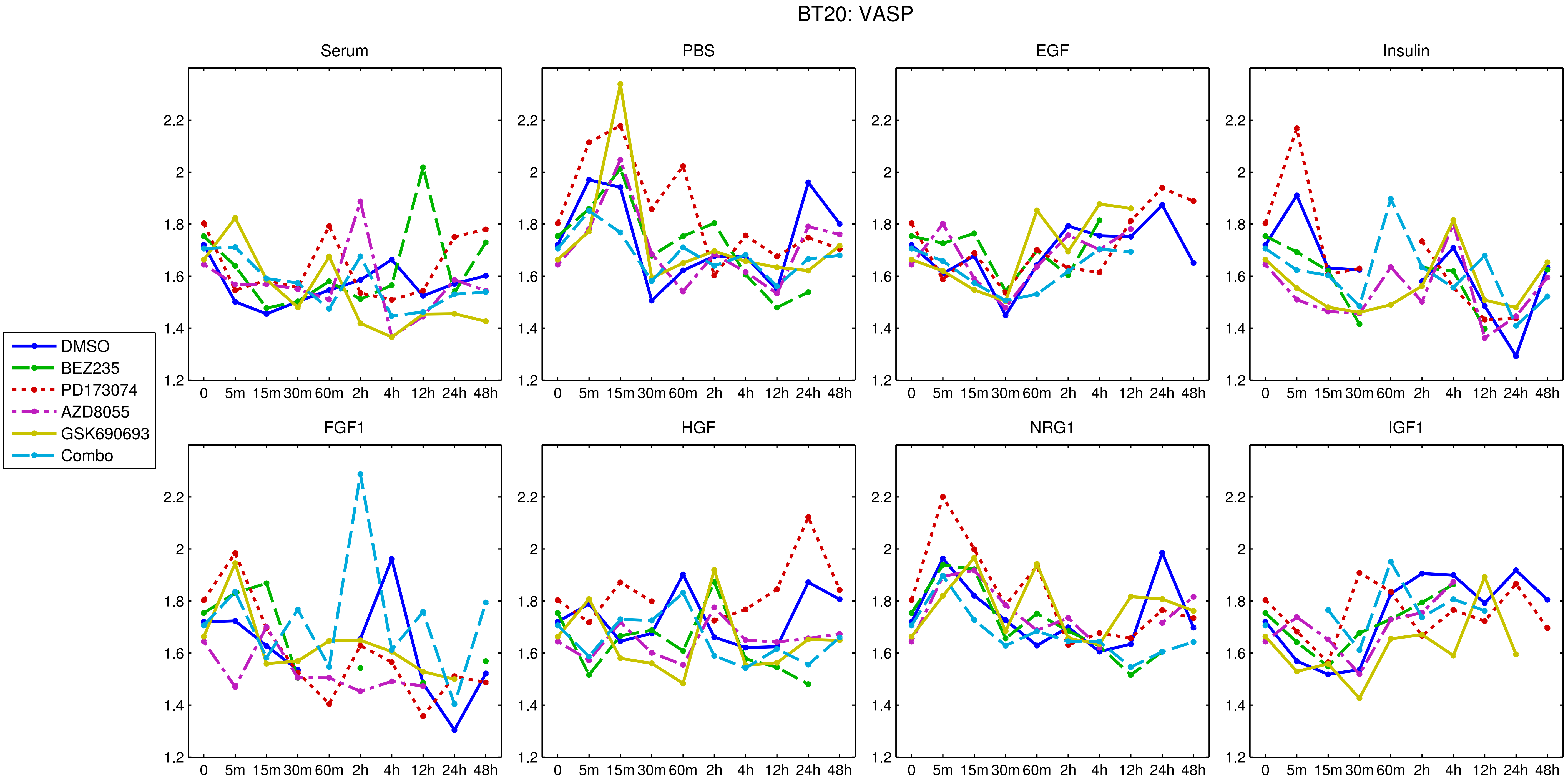
BT20: VASP
Serum
PBS
EGF
Insulin
FGF1
HGF
NRG1
IGF1
DMSO
BEZ235
PD173074
AZD8055
GSK690693
Combo
2.2
2
1.8
1.6
1.4
1.2
0
5m
15m
30m
60m
2h
4h
12h
24h
48h

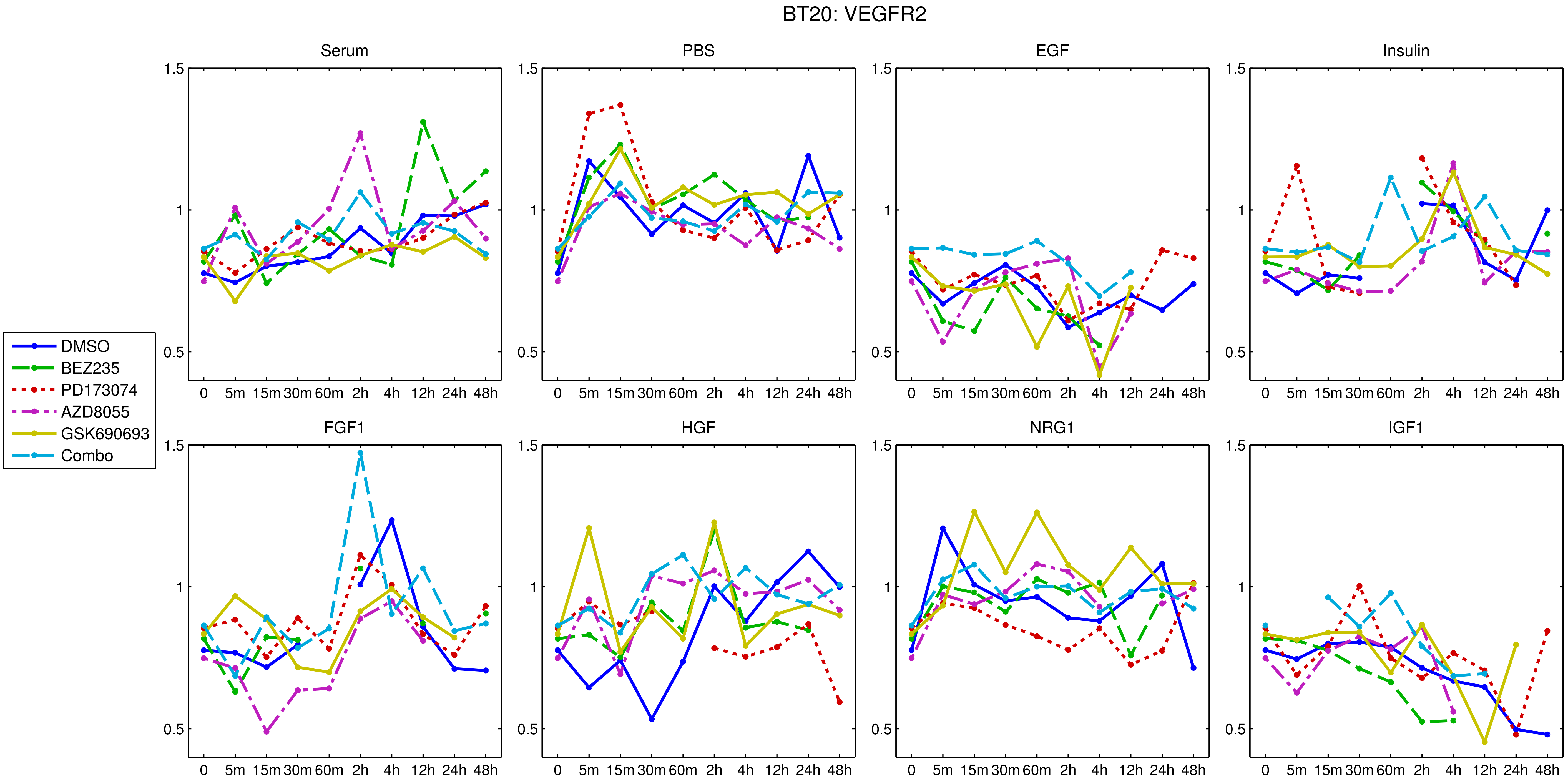

BT20: VEGFR2
Serum
PBS
EGF
Insulin
FGF1
HGF
NRG1
IGF1
1.5
1
0.5
0
5m
15m
30m
60m
2h
4h
12h
24h
48h
DMSO
BEZ235
PD173074
AZD8055
GSK690693
Combo

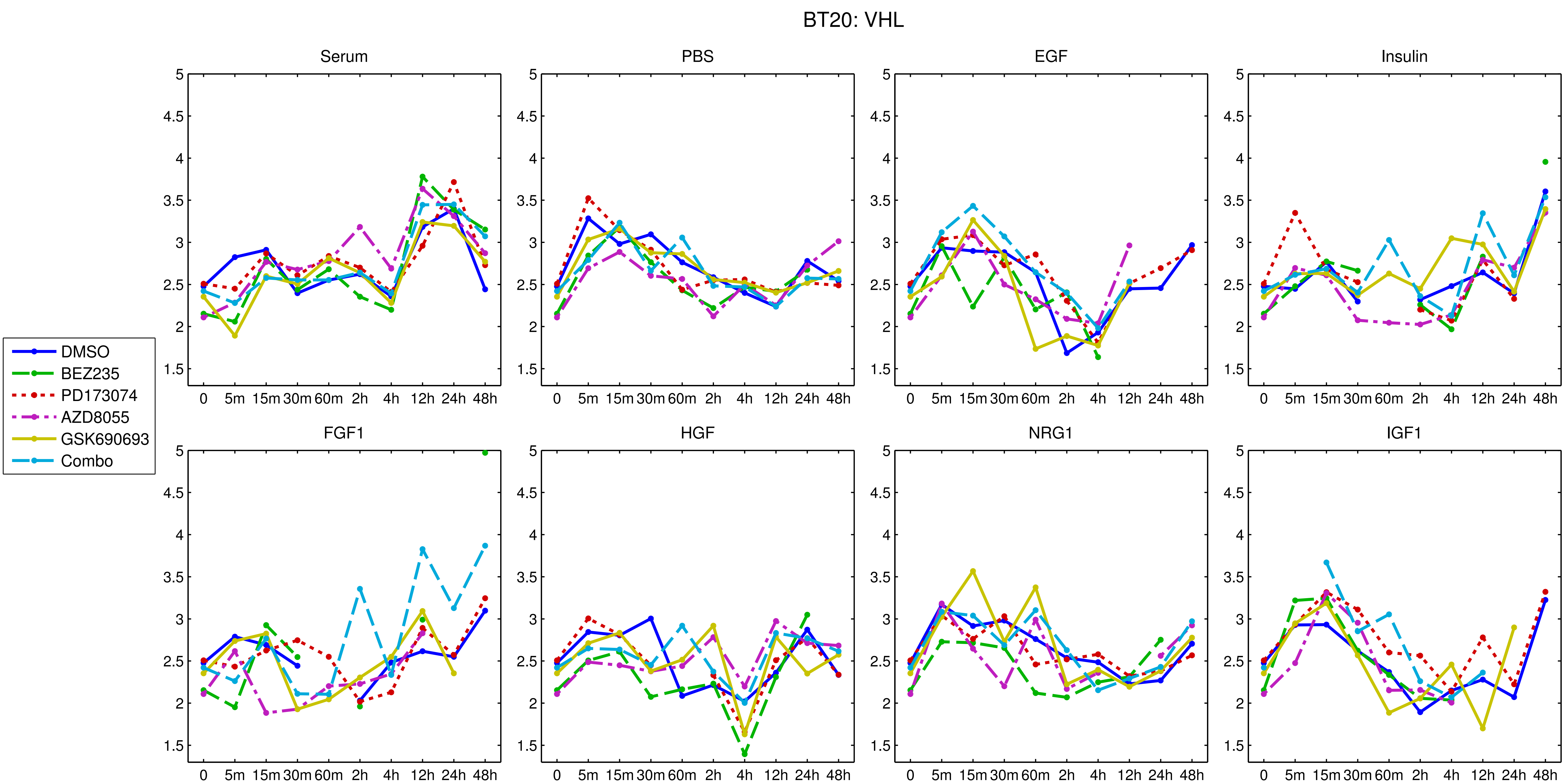
BT20: VHL
Serum
PBS
EGF
Insulin
FGF1
HGF
NRG1
IGF1
DMSO
BEZ235
PD173074
AZD8055
GSK690693
Combo
0 5m 15m 30m 60m 2h 4h 12h 24h 48h
1.5 2 2.5 3 3.5 4 4.5 5

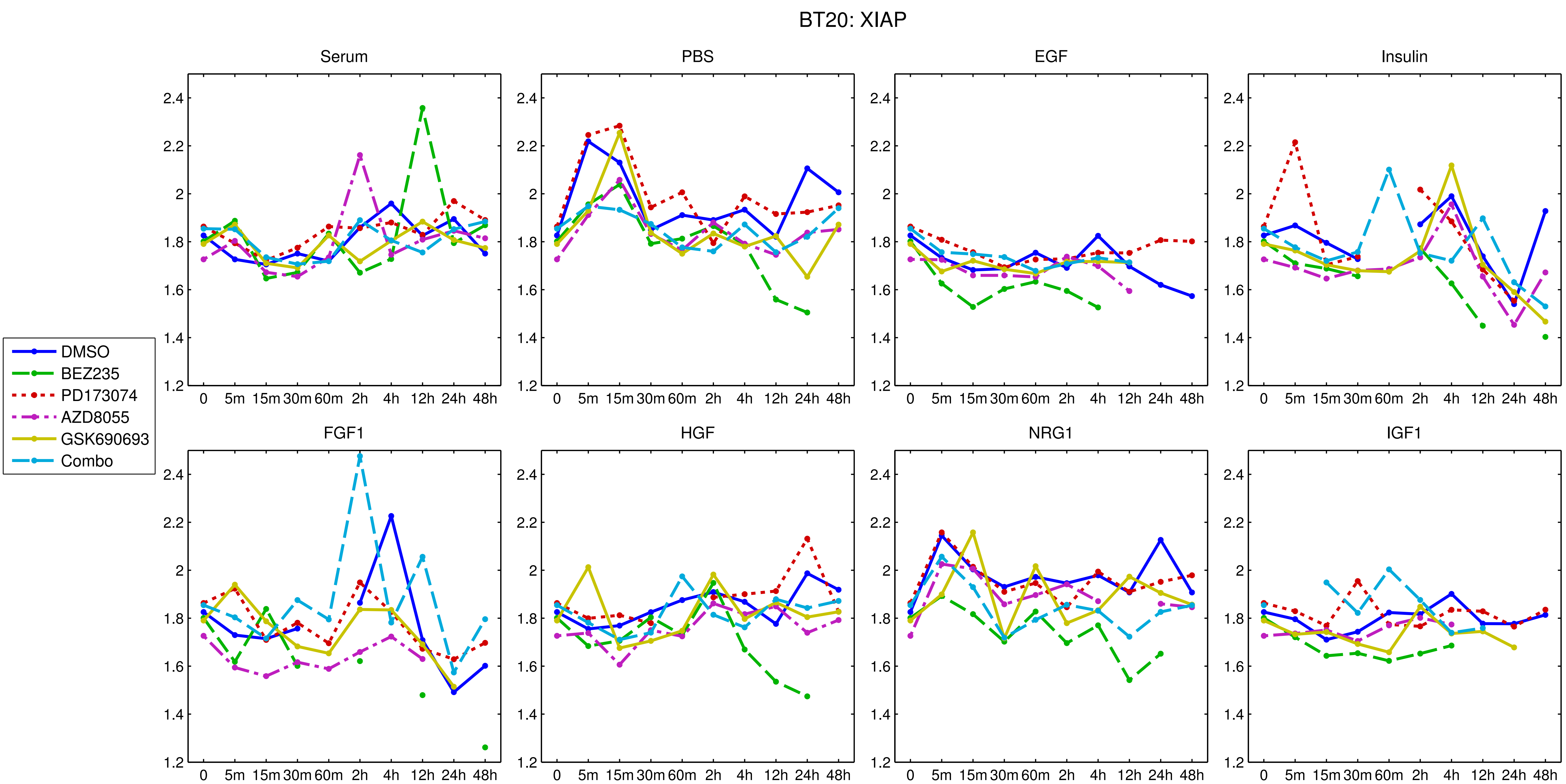
BT20: XIAP
Serum
PBS
EGF
Insulin
FGF1
HGF
NRG1
IGF1
DMSO
BEZ235
PD173074
AZD8055
GSK690693
Combo
2.4
2.2
2
1.8
1.6
1.4
1.2
0
5m
15m
30m
60m
2h
4h
12h
24h
48h

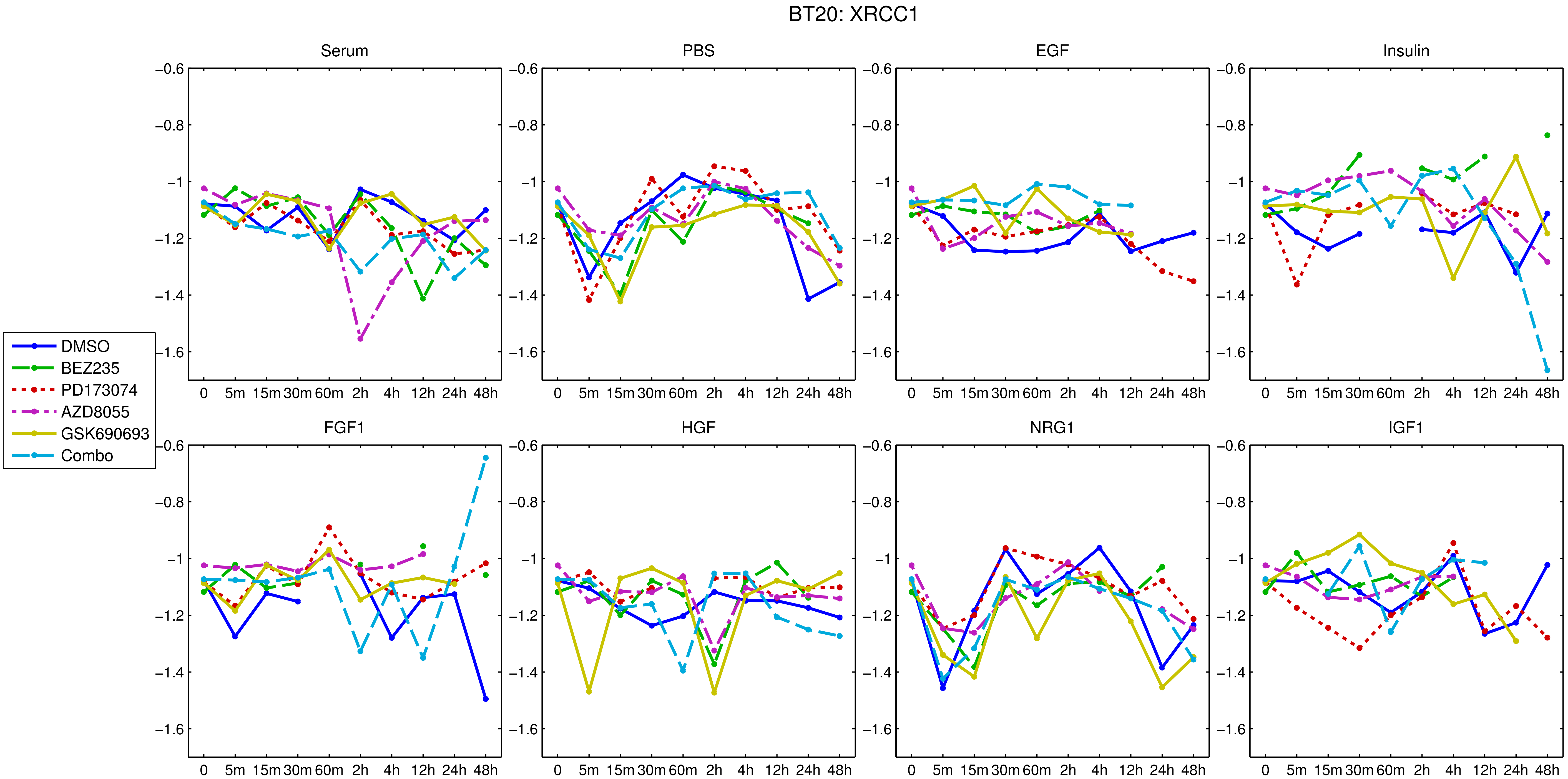
BT20: XRCC1
Serum
PBS
EGF
Insulin
FGF1
HGF
NRG1
IGF1
−0.6
−0.8
−1
−1.2
−1.4
−1.6
0
5m
15m
30m
60m
2h
4h
12h
24h
48h
DMSO
BEZ235
PD173074
AZD8055
GSK690693
Combo

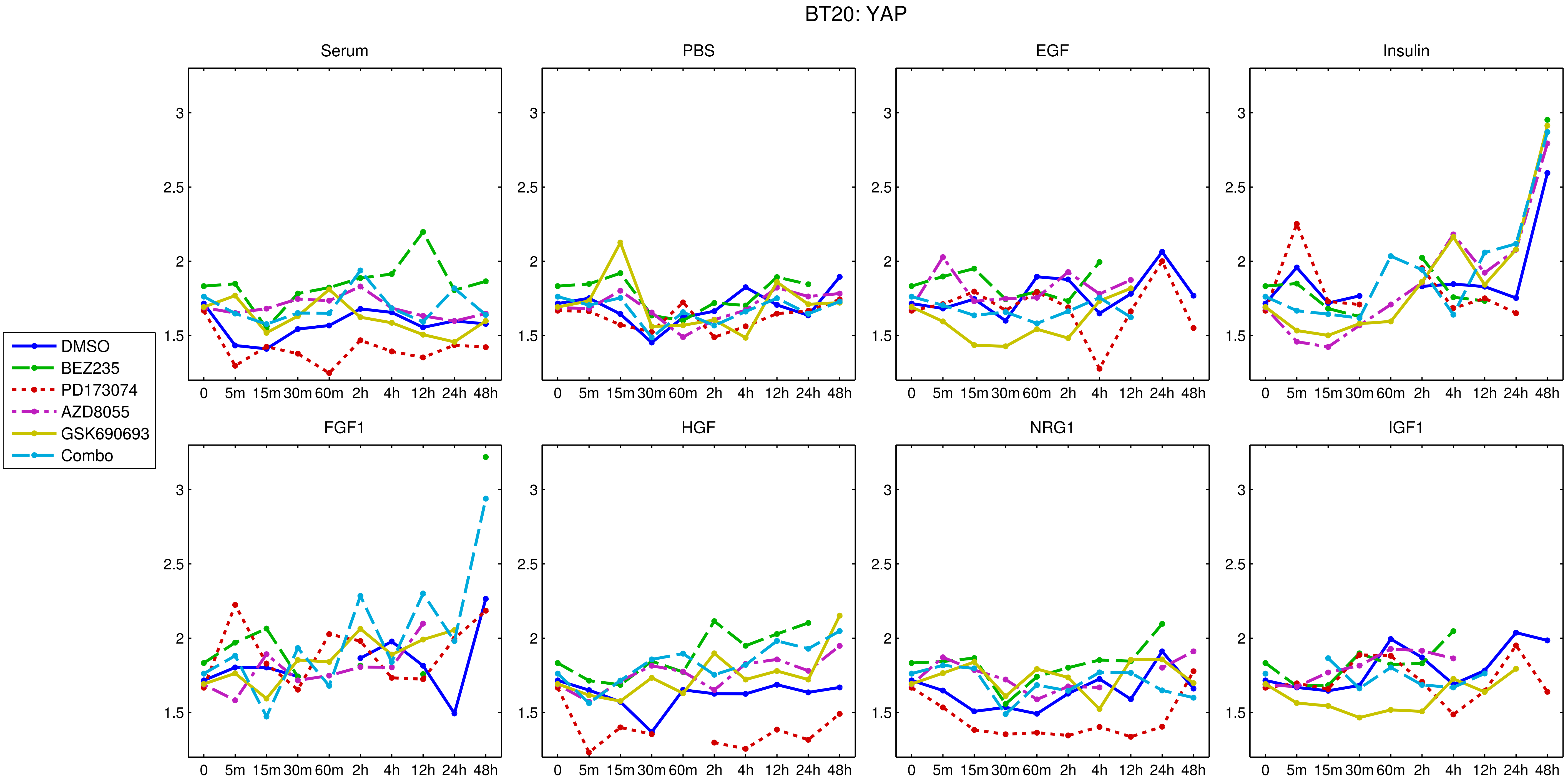

BT20: YAP
Serum
PBS
EGF
Insulin
FGF1
HGF
NRG1
IGF1
DMSO
BEZ235
PD173074
AZD8055
GSK690693
Combo
3
2.5
2
1.5
0
5m
15m
30m
60m
2h
4h
12h
24h
48h

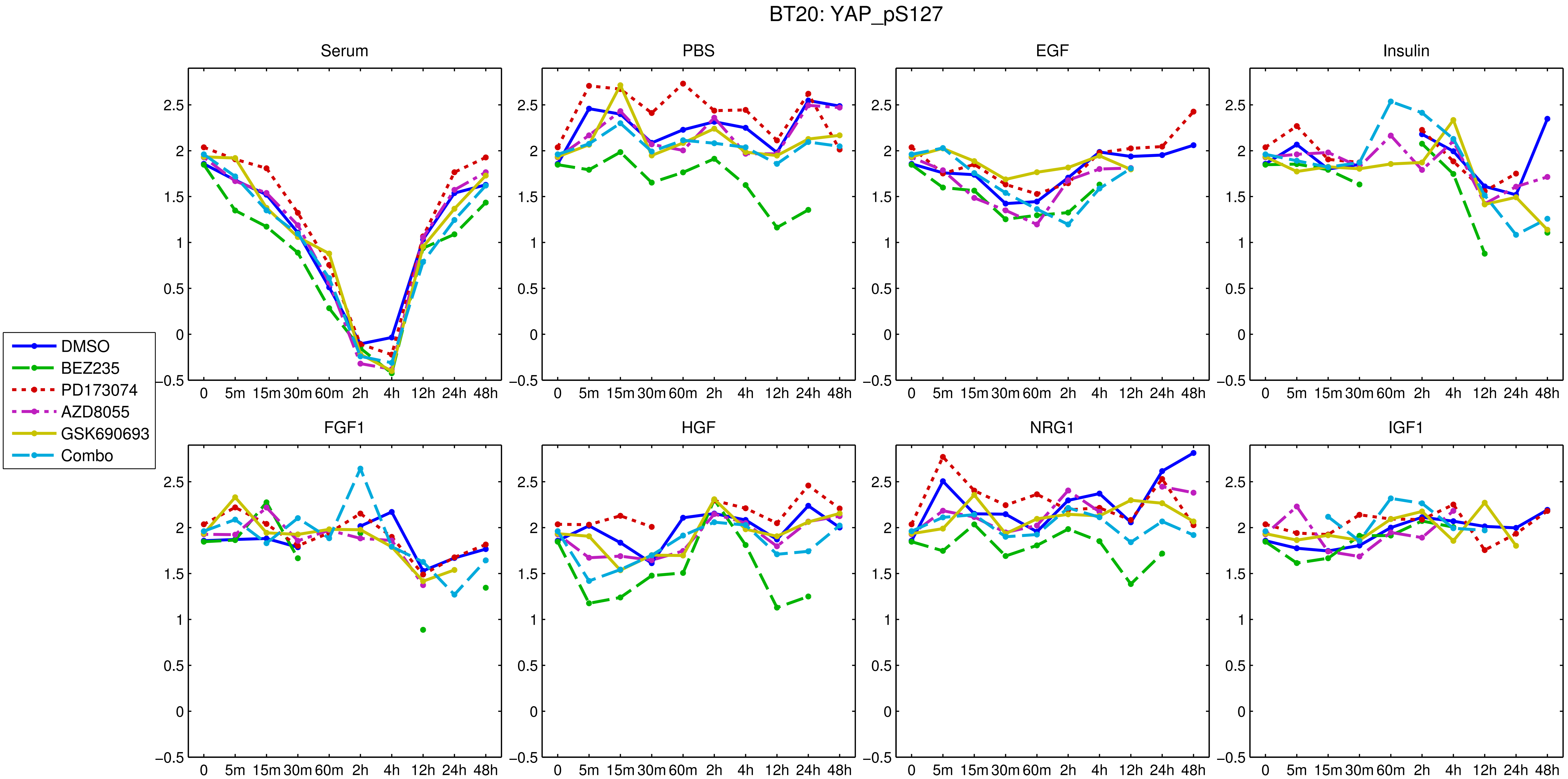
BT20: YAP_pS127
Serum
PBS
EGF
Insulin
FGF1
HGF
NRG1
IGF1
−0.5
0
0.5
1
1.5
2
2.5
0
5m
15m
30m
60m
2h
4h
12h
24h
48h
DMSO
BEZ235
PD173074
AZD8055
GSK690693
Combo

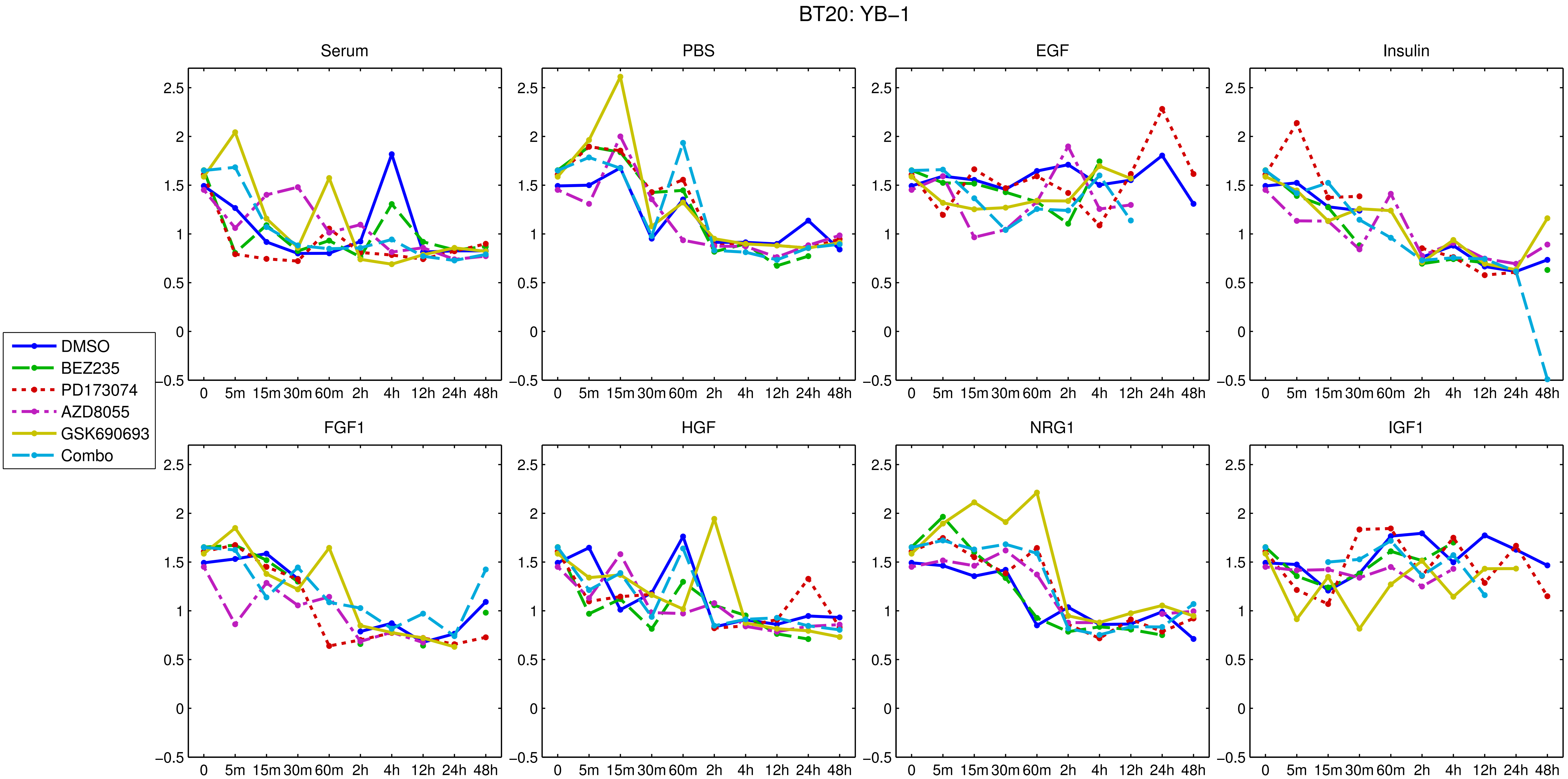
BT20: YB−1
Serum
PBS
EGF
Insulin
FGF1
HGF
NRG1
IGF1
DMSO
BEZ235
PD173074
AZD8055
GSK690693
Combo
2.5
2
1.5
1
0.5
0
−0.5
0
5m
15m
30m
60m
2h
4h
12h
24h
48h

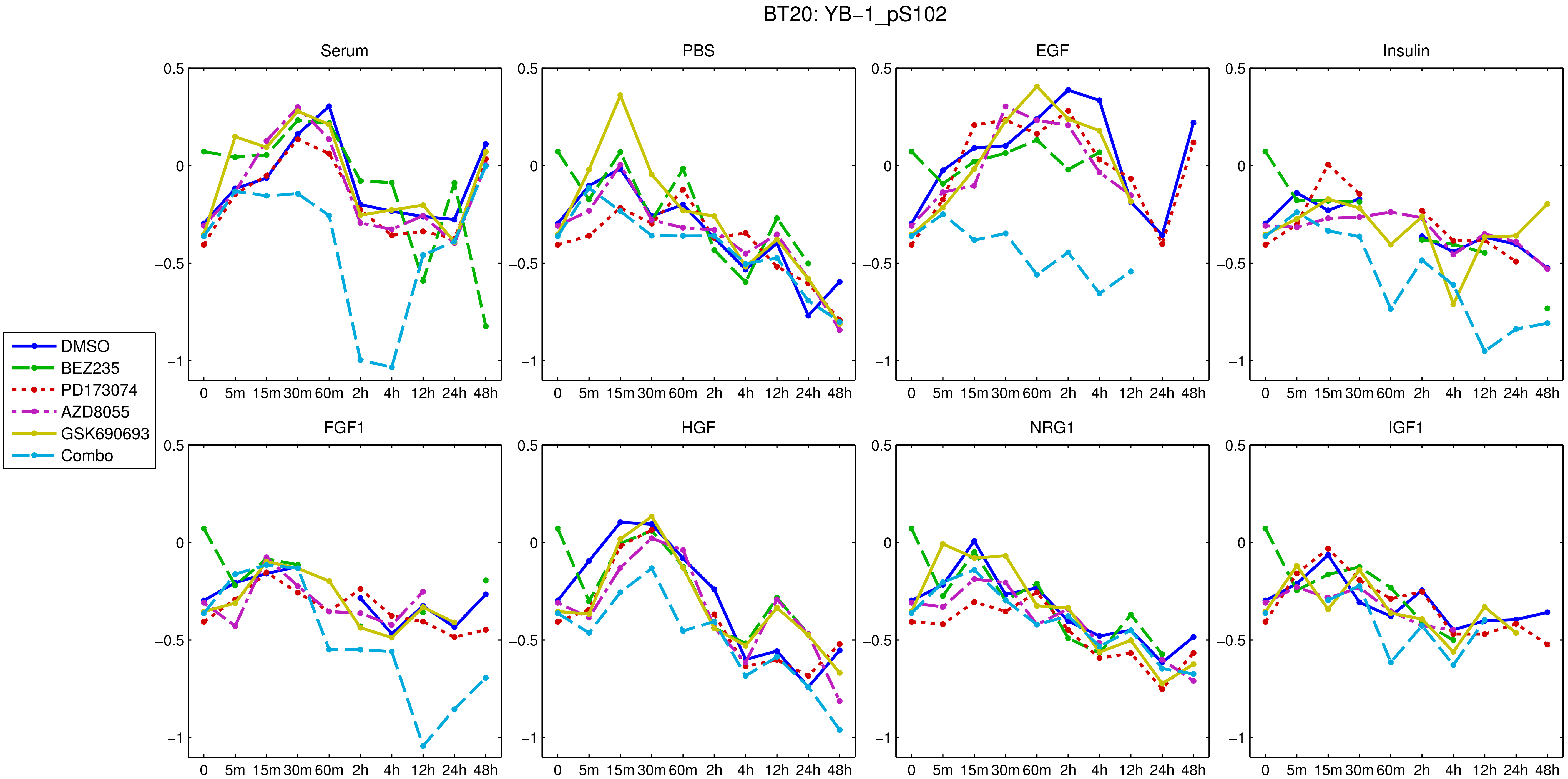

BT20: YB−1_pS102
Serum
PBS
EGF
Insulin
FGF1
HGF
NRG1
IGF1
DMSO
BEZ235
PD173074
AZD8055
GSK690693
Combo
0.5
0
−0.5
−1
0 5m 15m 30m 60m 2h 4h 12h 24h 48h
