# Supplementary material for: Context Specificity in Causal Signaling Networks Revealed by Phosphoprotein Profiling
Source: Cell Syst. 2017 Jan 25;4(1):73–83.e10. doi: 10.1016/j.cels.2016.11.013 (PMC5279869; doi:10.1016/j.cels.2016.11.013)
Supplement: Data S2. RPPA Data Time-Course Plots, Related to STAR Methods — A zip archive containing time-course plots of the reverse-phase protein array data generated in this study. See the README file included in the zip archive for further details. [file mmc6.zip › DataS2/BT549.pdf]

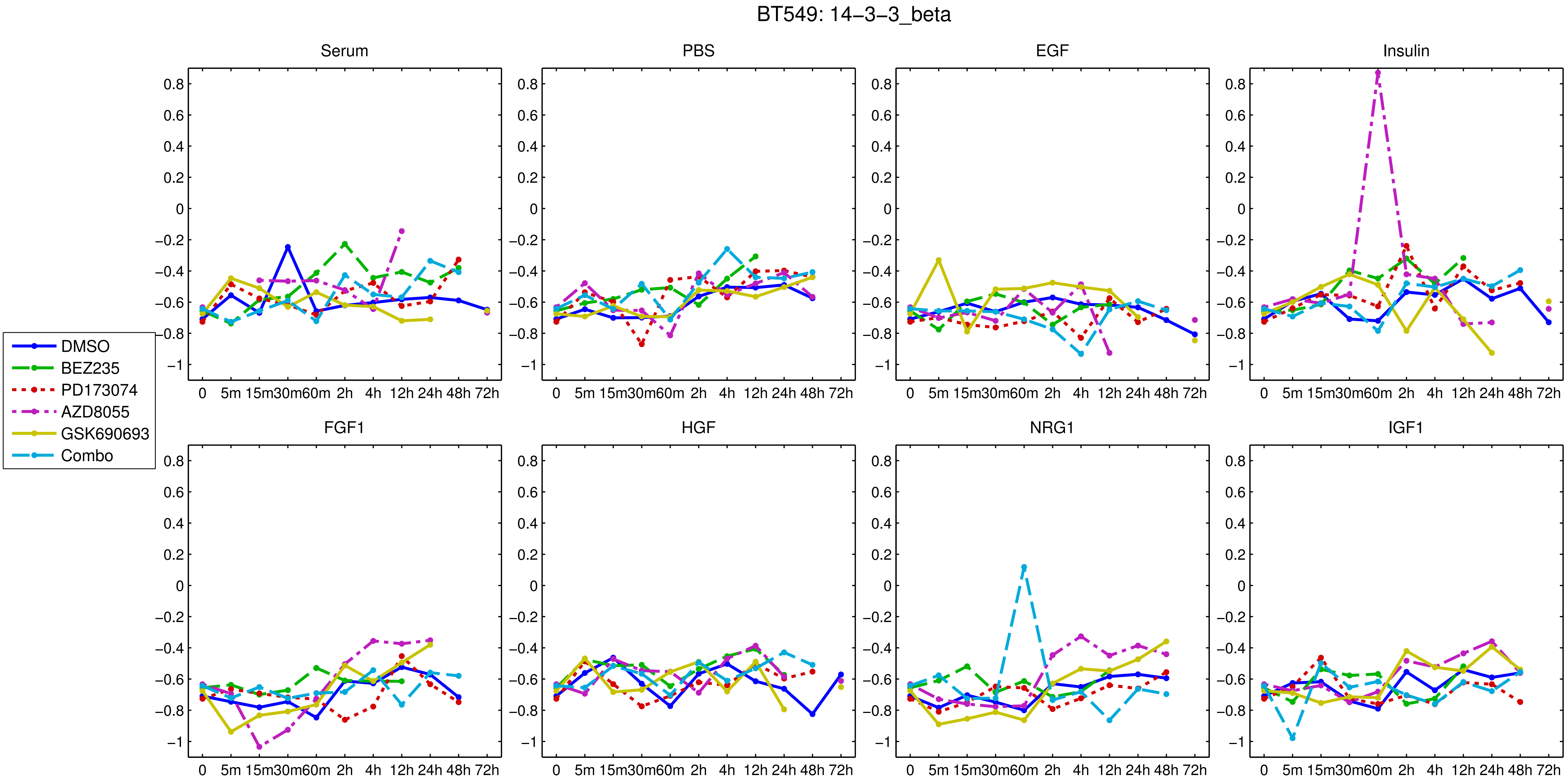
BT549: 14−3−3_beta
Serum
PBS
EGF
Insulin
FGF1
HGF
NRG1
IGF1
DMSO
BEZ235
PD173074
AZD8055
GSK690693
Combo
0.8
0.6
0.4
0.2
0
−0.2
−0.4
−0.6
−0.8
−1
0 5m 15m30m60m 2h 4h 12h 24h 48h 72h

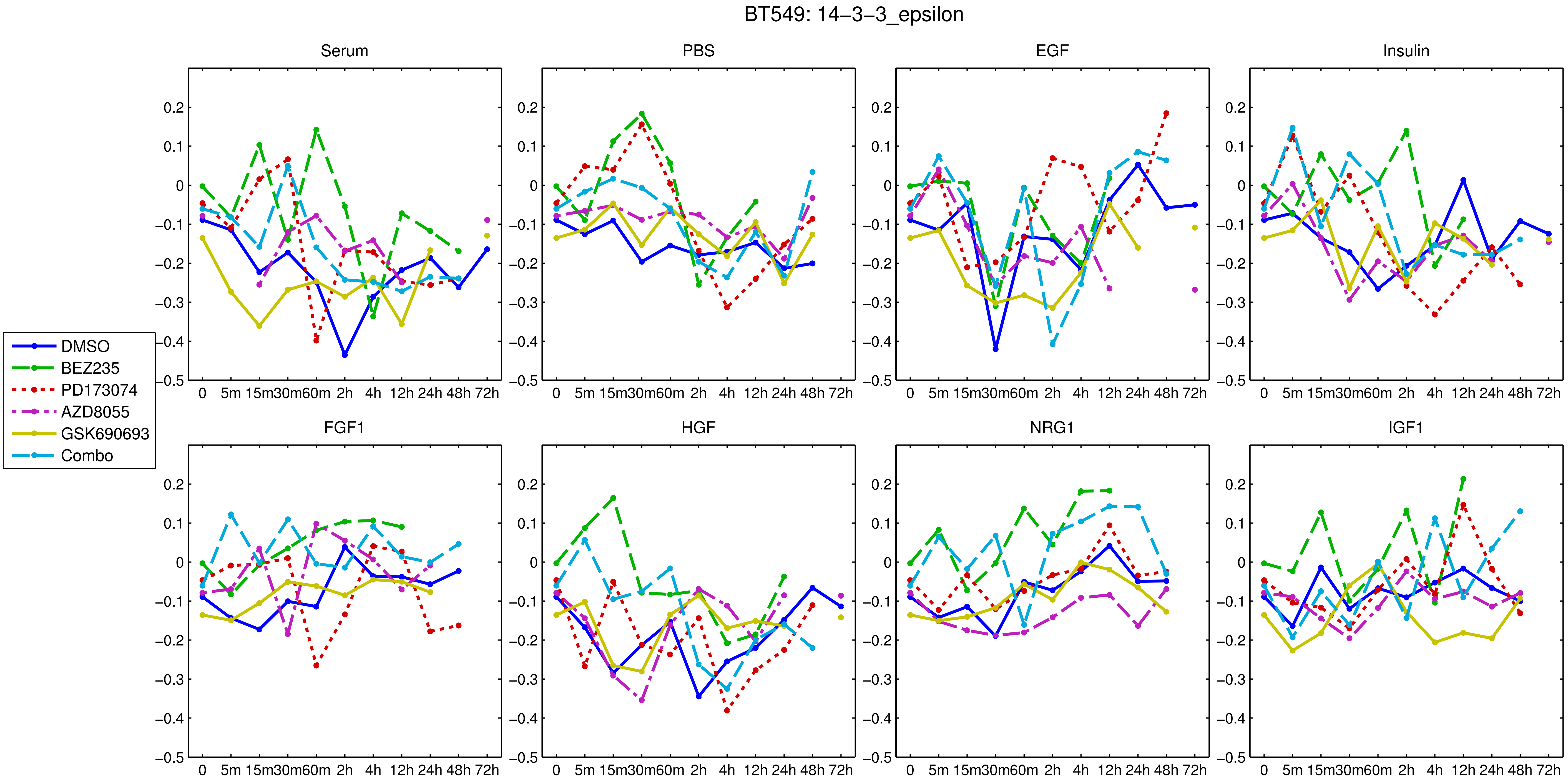
BT549: 14−3−3_epsilon
Serum
PBS
EGF
Insulin
FGF1
HGF
NRG1
IGF1
DMSO
BEZ235
PD173074
AZD8055
GSK690693
Combo
0.2
0.1
0
−0.1
−0.2
−0.3
−0.4
−0.5
0
5m
15m
30m
60m
2h
4h
12h
24h
48h
72h

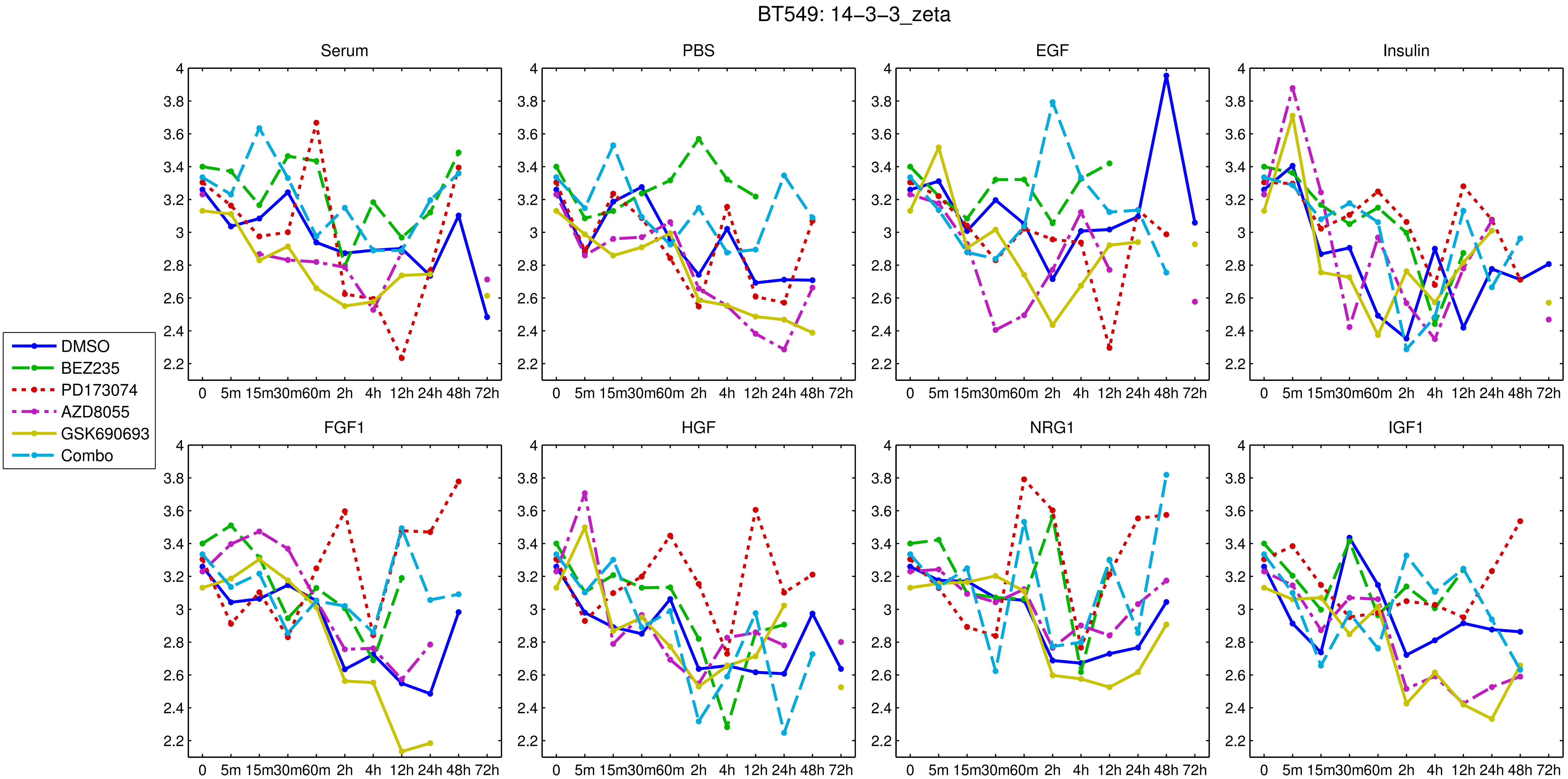

BT549: 14−3−3_zeta
Serum
PBS
EGF
Insulin
FGF1
HGF
NRG1
IGF1
DMSO
BEZ235
PD173074
AZD8055
GSK690693
Combo
4
3.8
3.6
3.4
3.2
3
2.8
2.6
2.4
2.2
0 5m 15m 30m 60m 2h 4h 12h 24h 48h 72h

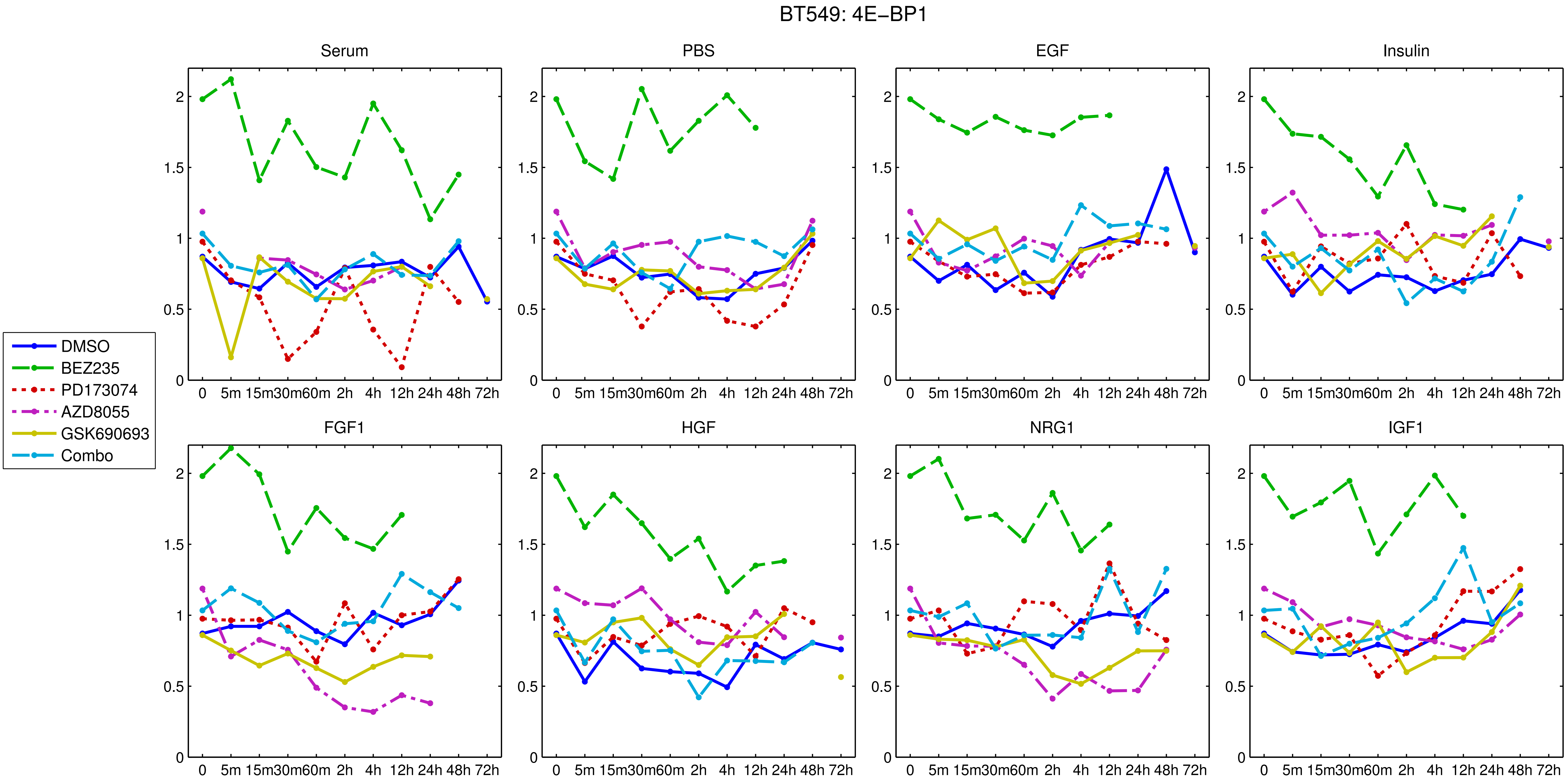
BT549: 4E−BP1
Serum
PBS
EGF
Insulin
FGF1
HGF
NRG1
IGF1
DMSO
BEZ235
PD173074
AZD8055
GSK690693
Combo
0 5m 15m 30m 60m 2h 4h 12h 24h 48h 72h
0 0.5 1 1.5 2

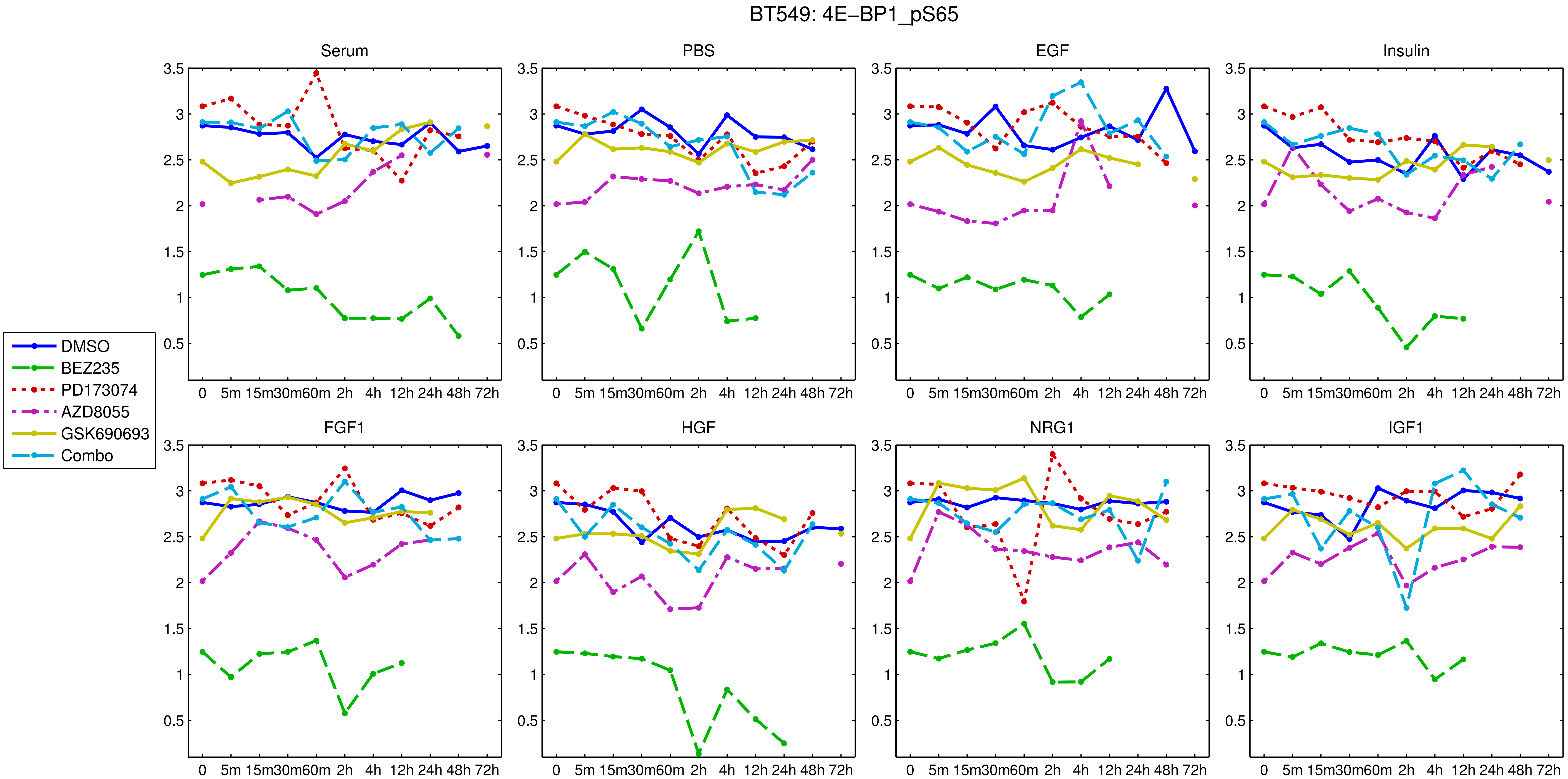

BT549: 4E−BP1_pS65
Serum
PBS
EGF
Insulin
FGF1
HGF
NRG1
IGF1
DMSO
BEZ235
PD173074
AZD8055
GSK690693
Combo
0 5m 15m 30m 60m 2h 4h 12h 24h 48h 72h
3.5
3
2.5
2
1.5
1
0.5

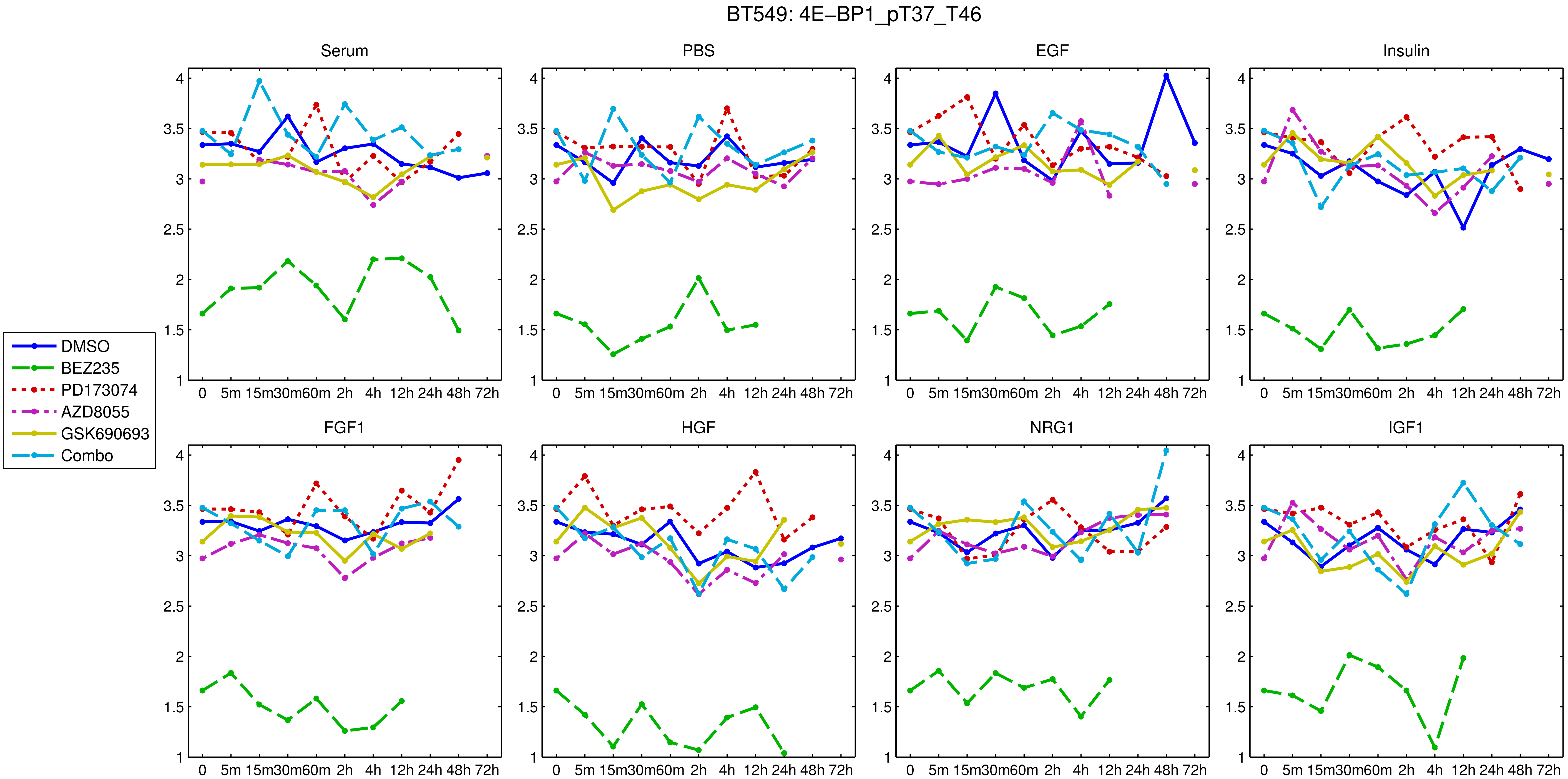
BT549: 4E−BP1_pT37_T46
Serum
PBS
EGF
Insulin
FGF1
HGF
NRG1
IGF1
DMSO
BEZ235
PD173074
AZD8055
GSK690693
Combo
0 5m 15m 30m 60m 2h 4h 12h 24h 48h 72h
1 1.5 2 2.5 3 3.5 4

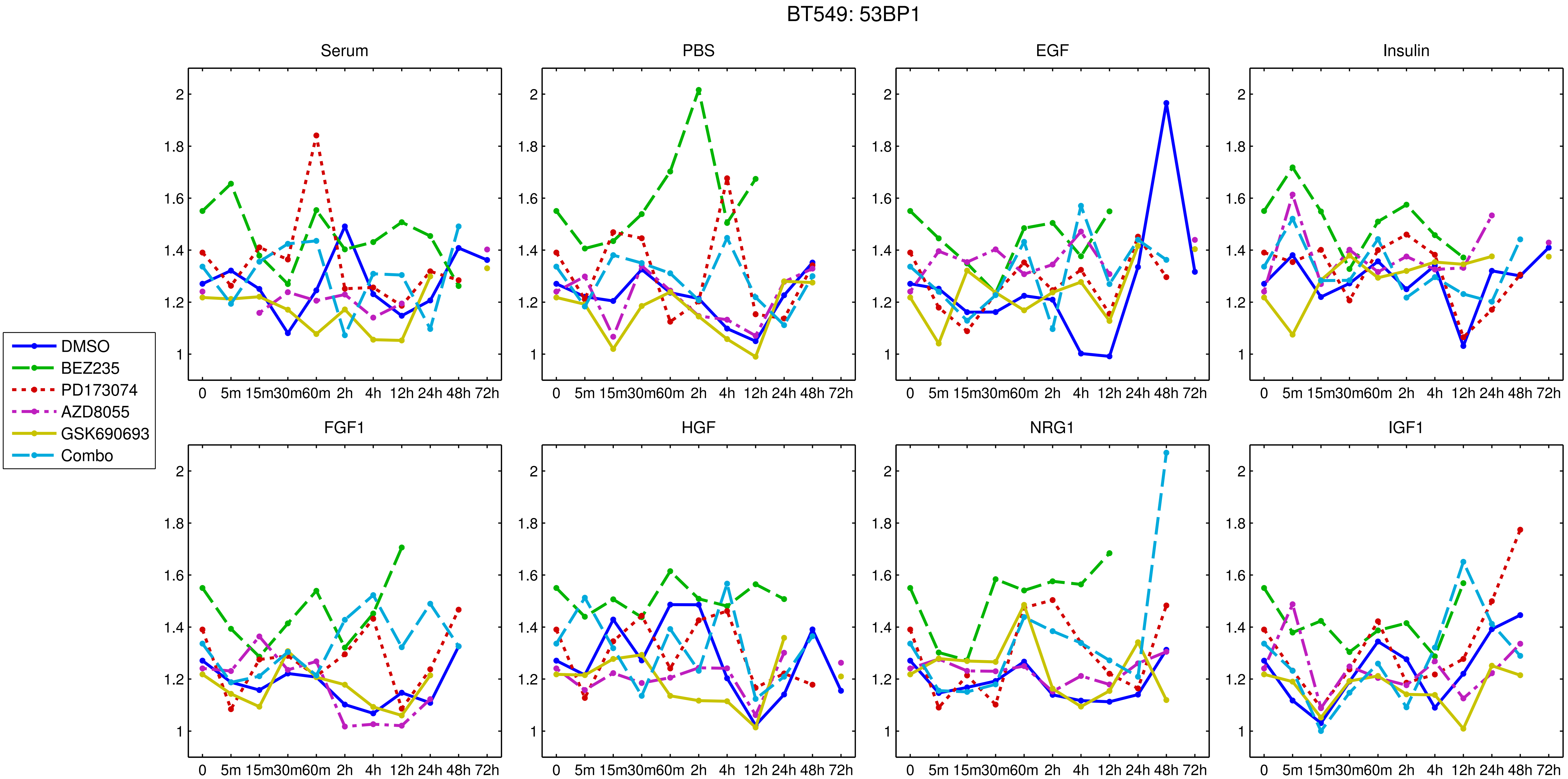
BT549: 53BP1
Serum
PBS
EGF
Insulin
FGF1
HGF
NRG1
IGF1
DMSO
BEZ235
PD173074
AZD8055
GSK690693
Combo
0
5m
15m
30m
60m
2h
4h
12h
24h
48h
72h
1
1.2
1.4
1.6
1.8
2

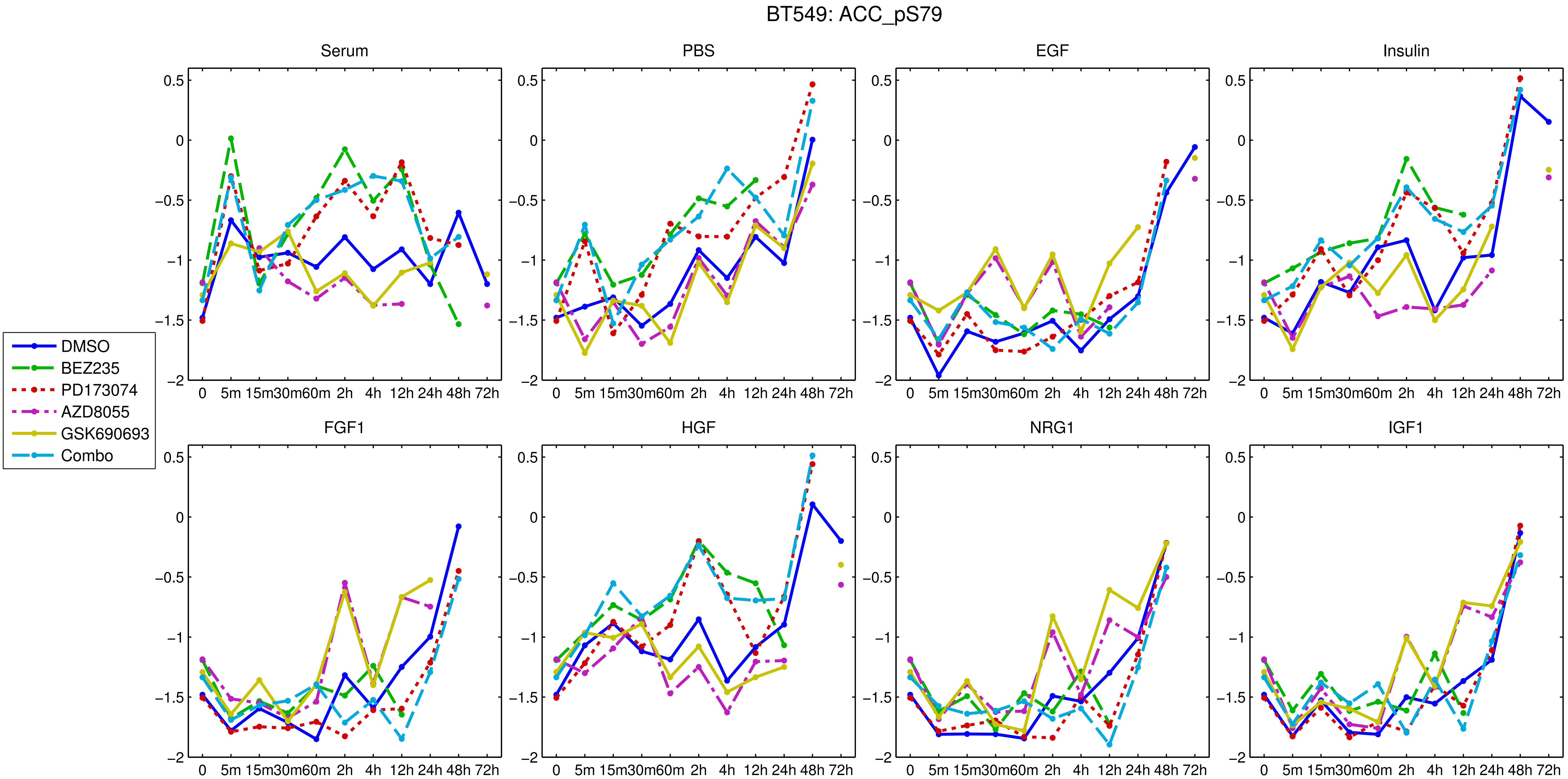

BT549: ACC_pS79
Serum
PBS
EGF
Insulin
FGF1
HGF
NRG1
IGF1
0.5
0
−0.5
−1
−1.5
−2
0
5m
15m
30m
60m
2h
4h
12h
24h
48h
72h
DMSO
BEZ235
PD173074
AZD8055
GSK690693
Combo

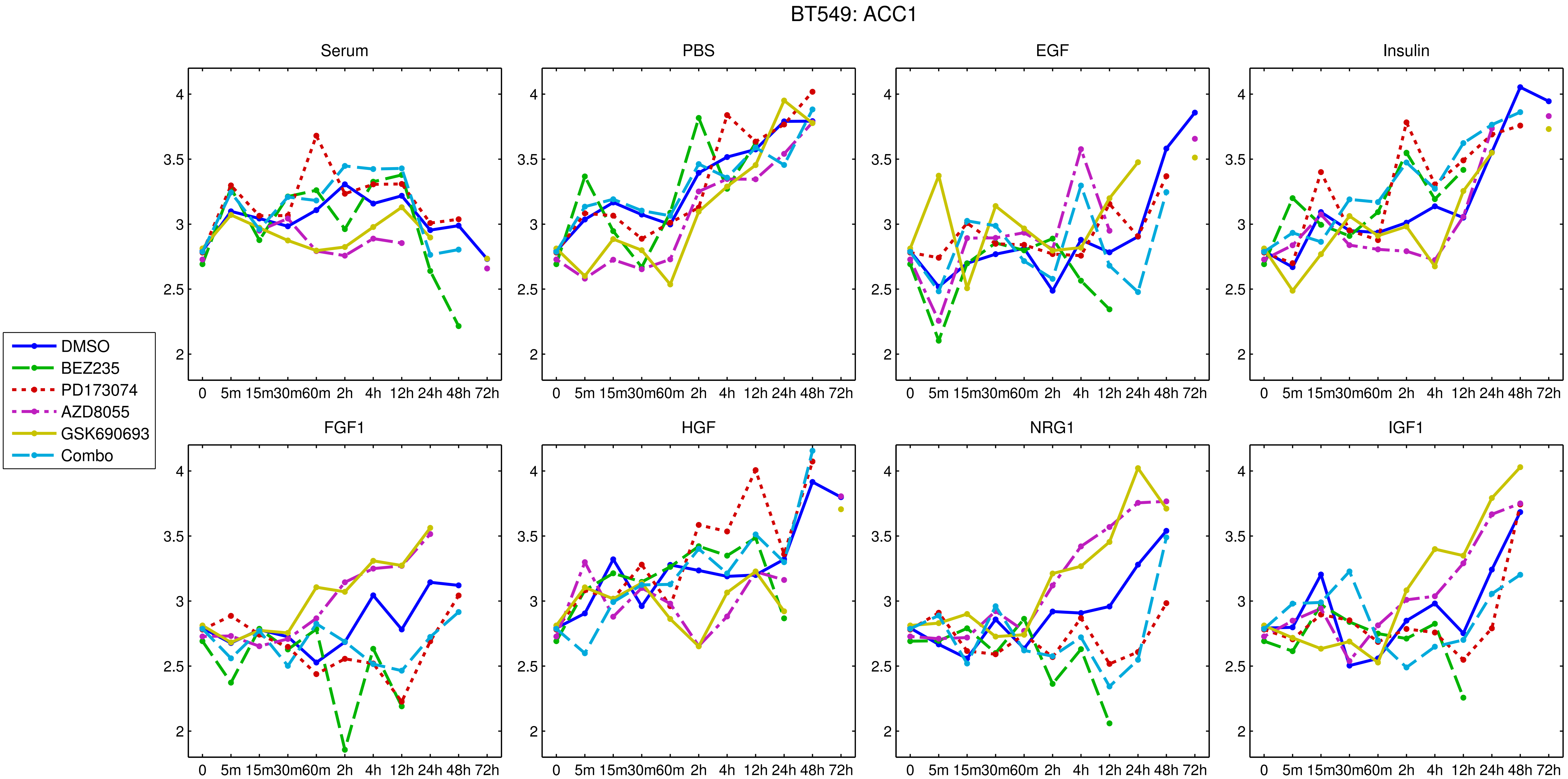
BT549: ACC1
Serum
PBS
EGF
Insulin
FGF1
HGF
NRG1
IGF1
DMSO
BEZ235
PD173074
AZD8055
GSK690693
Combo
0 5m 15m 30m 60m 2h 4h 12h 24h 48h 72h
2
2.5
3
3.5
4

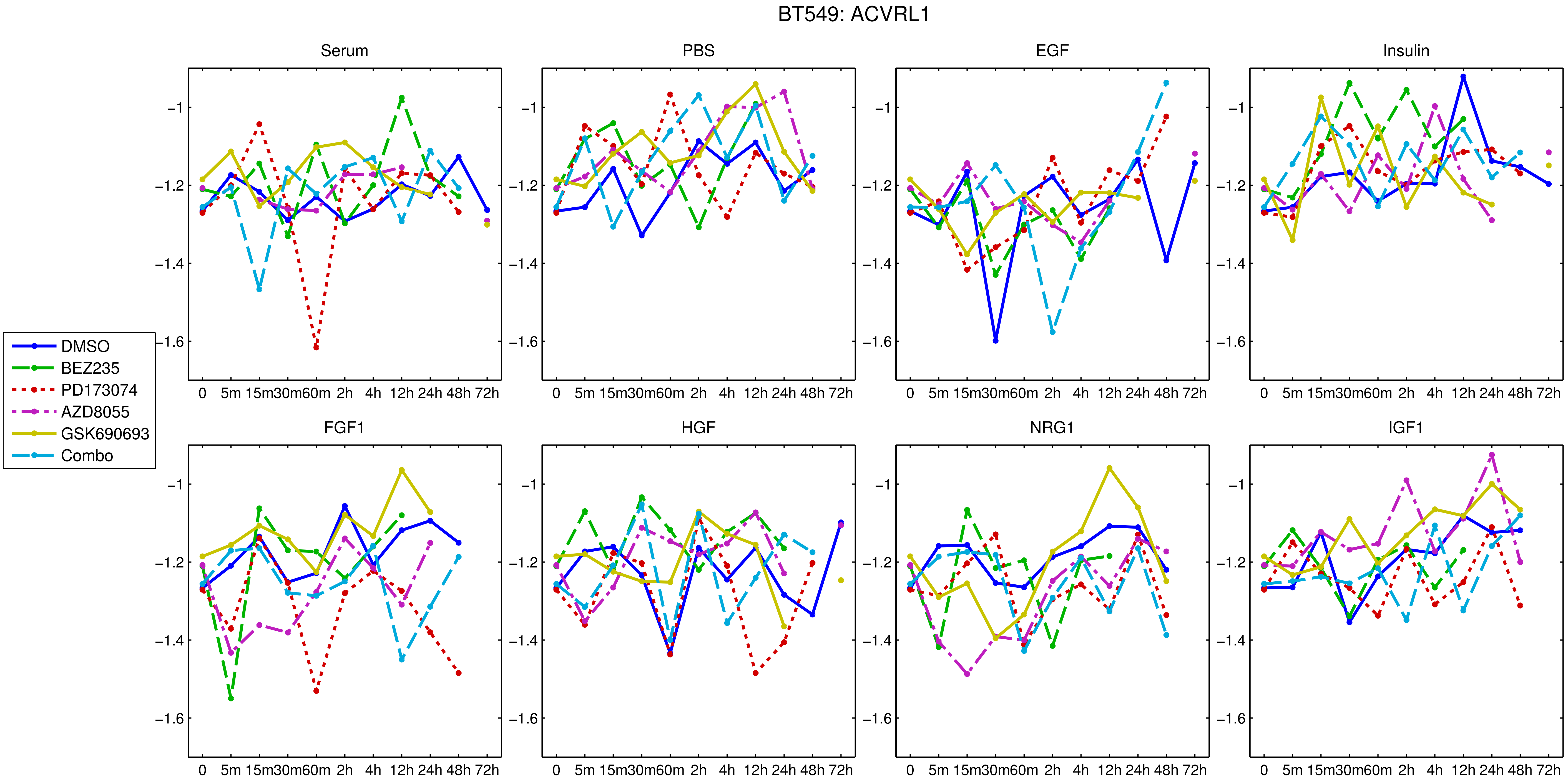
BT549: ACVRL1
Serum
PBS
EGF
Insulin
FGF1
HGF
NRG1
IGF1
DMSO
BEZ235
PD173074
AZD8055
GSK690693
Combo
−1
−1.2
−1.4
−1.6
0
5m
15m
30m
60m
2h
4h
12h
24h
48h
72h

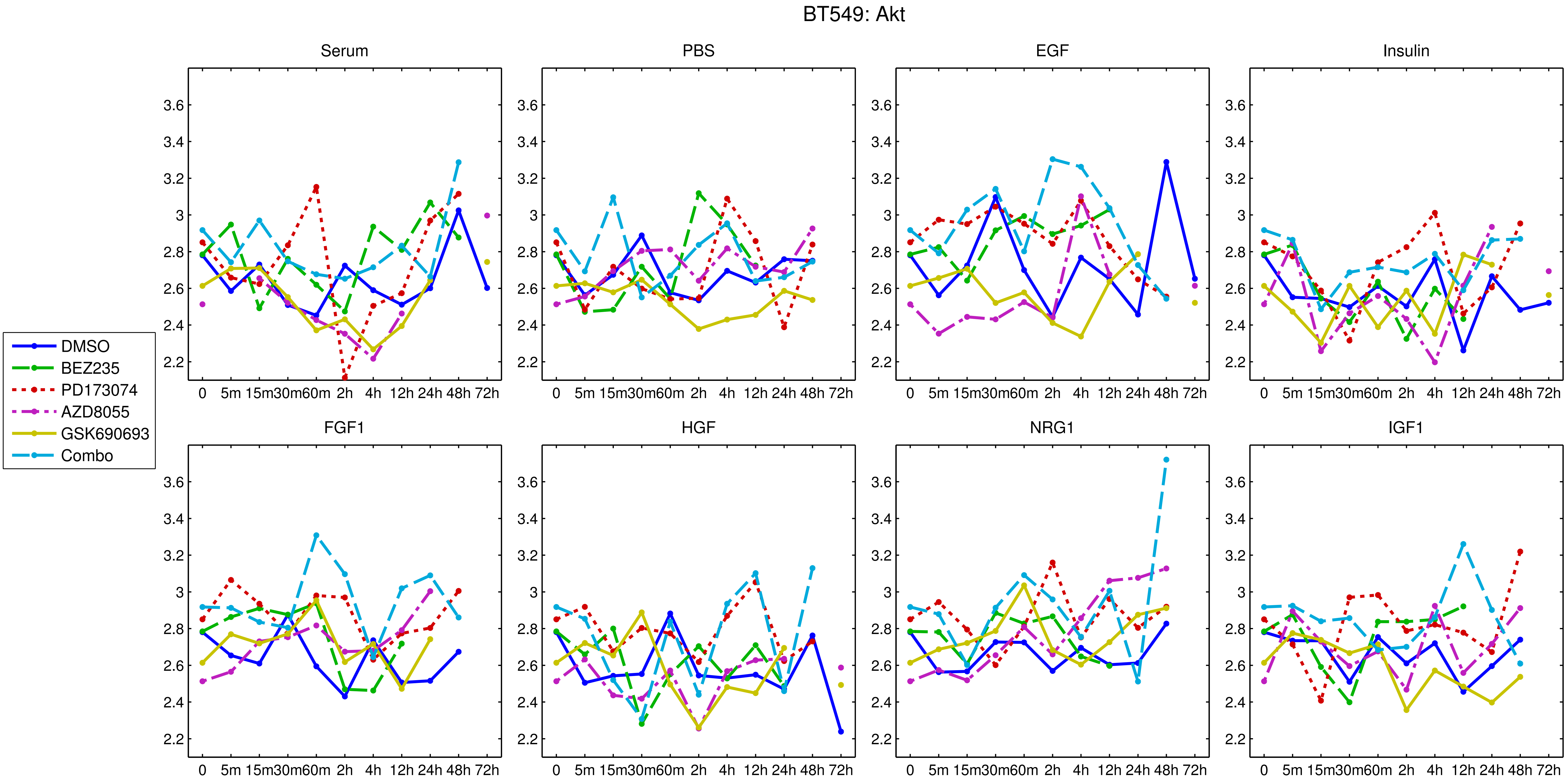
BT549: Akt
Serum
PBS
EGF
Insulin
FGF1
HGF
NRG1
IGF1
3.6
3.4
3.2
3
2.8
2.6
2.4
2.2
0 5m 15m 30m 60m 2h 4h 12h 24h 48h 72h
DMSO
BEZ235
PD173074
AZD8055
GSK690693
Combo

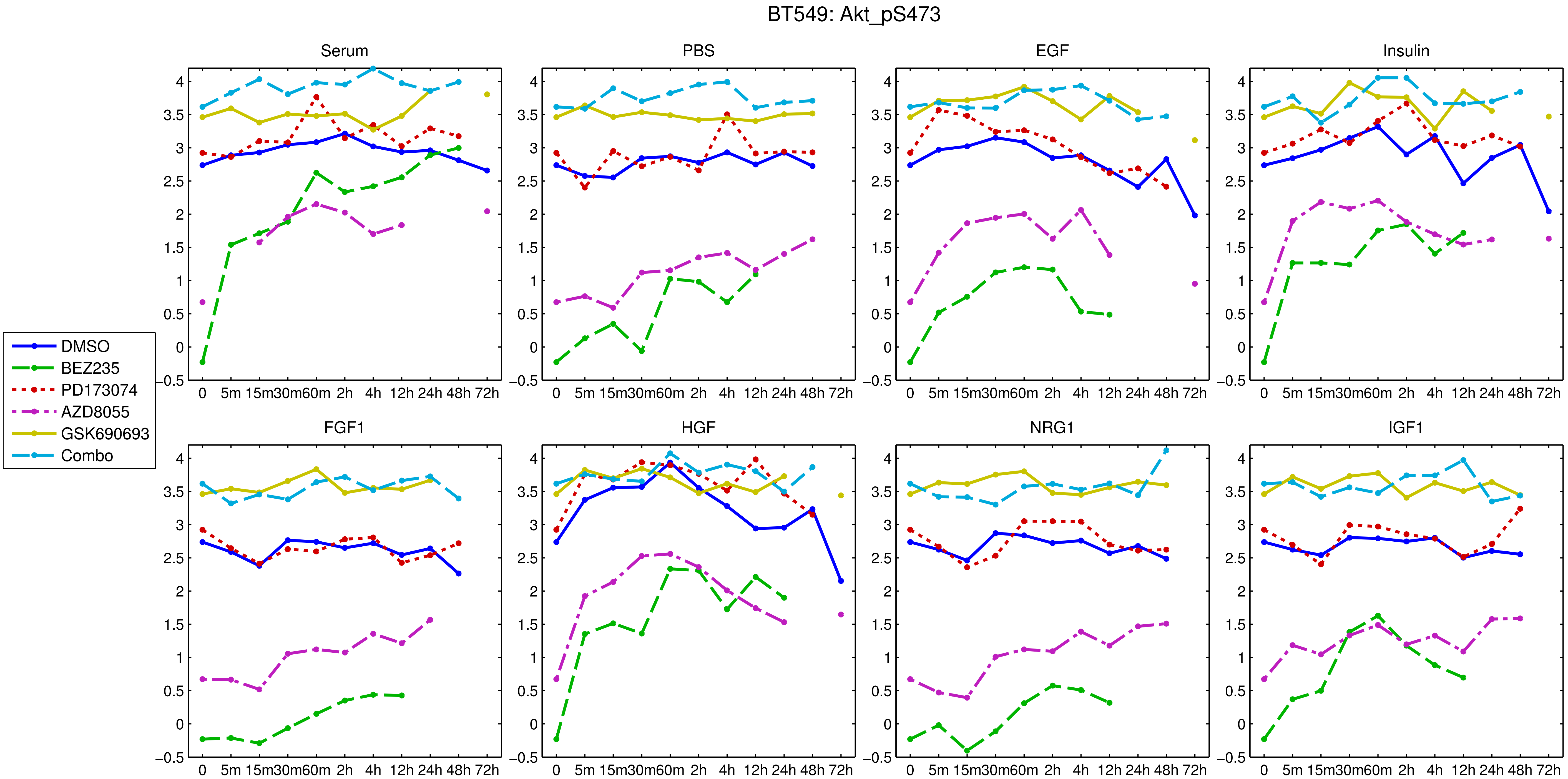
BT549: Akt_pS473
Serum
PBS
EGF
Insulin
FGF1
HGF
NRG1
IGF1
DMSO
BEZ235
PD173074
AZD8055
GSK690693
Combo
0
5m
15m
30m
60m
2h
4h
12h
24h
48h
72h
−0.5
0
0.5
1
1.5
2
2.5
3
3.5
4

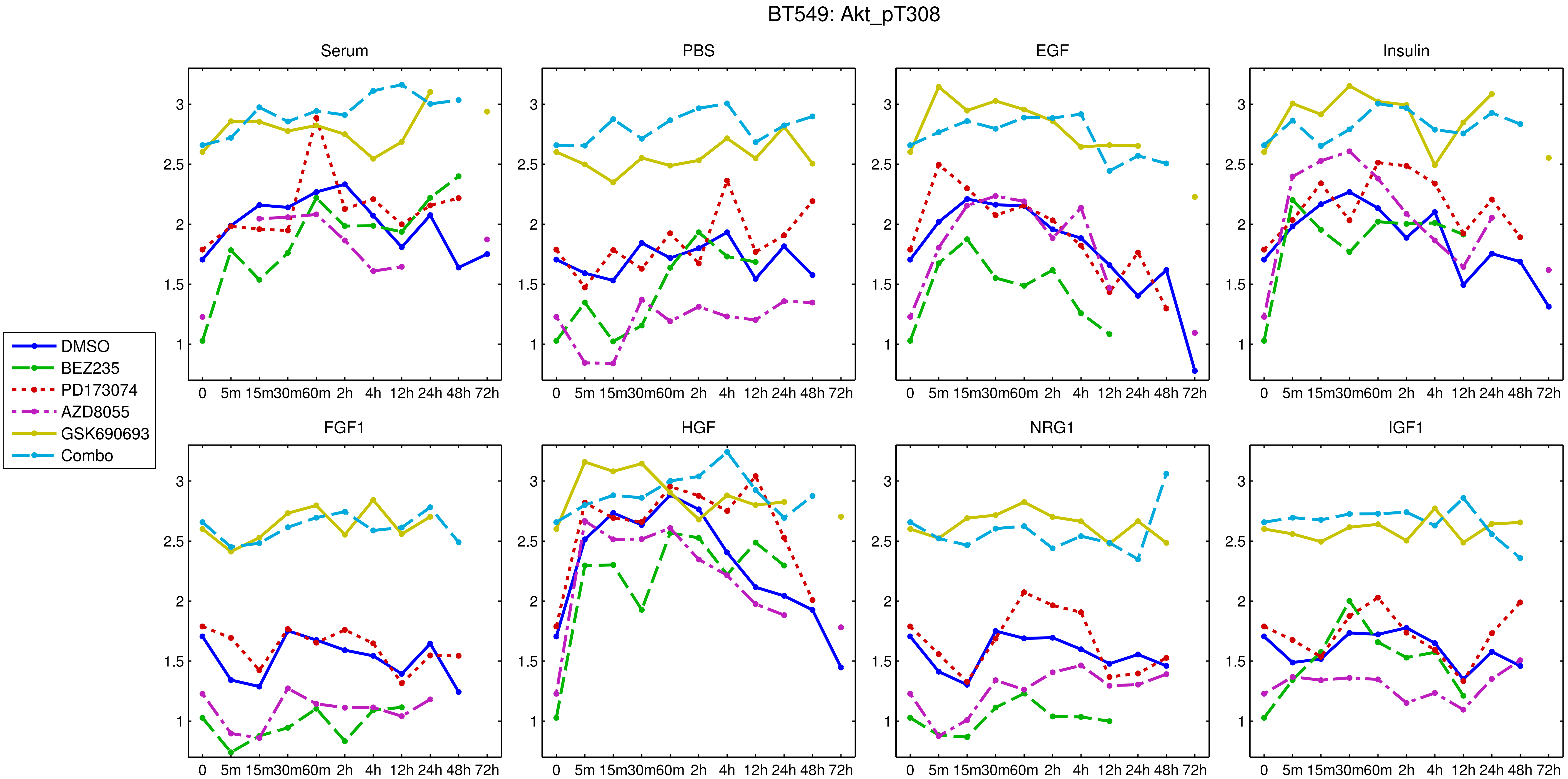
BT549: Akt_pT308
Serum
PBS
EGF
Insulin
FGF1
HGF
NRG1
IGF1
0 5m 15m 30m 60m 2h 4h 12h 24h 48h 72h
1
1.5
2
2.5
3
DMSO
BEZ235
PD173074
AZD8055
GSK690693
Combo

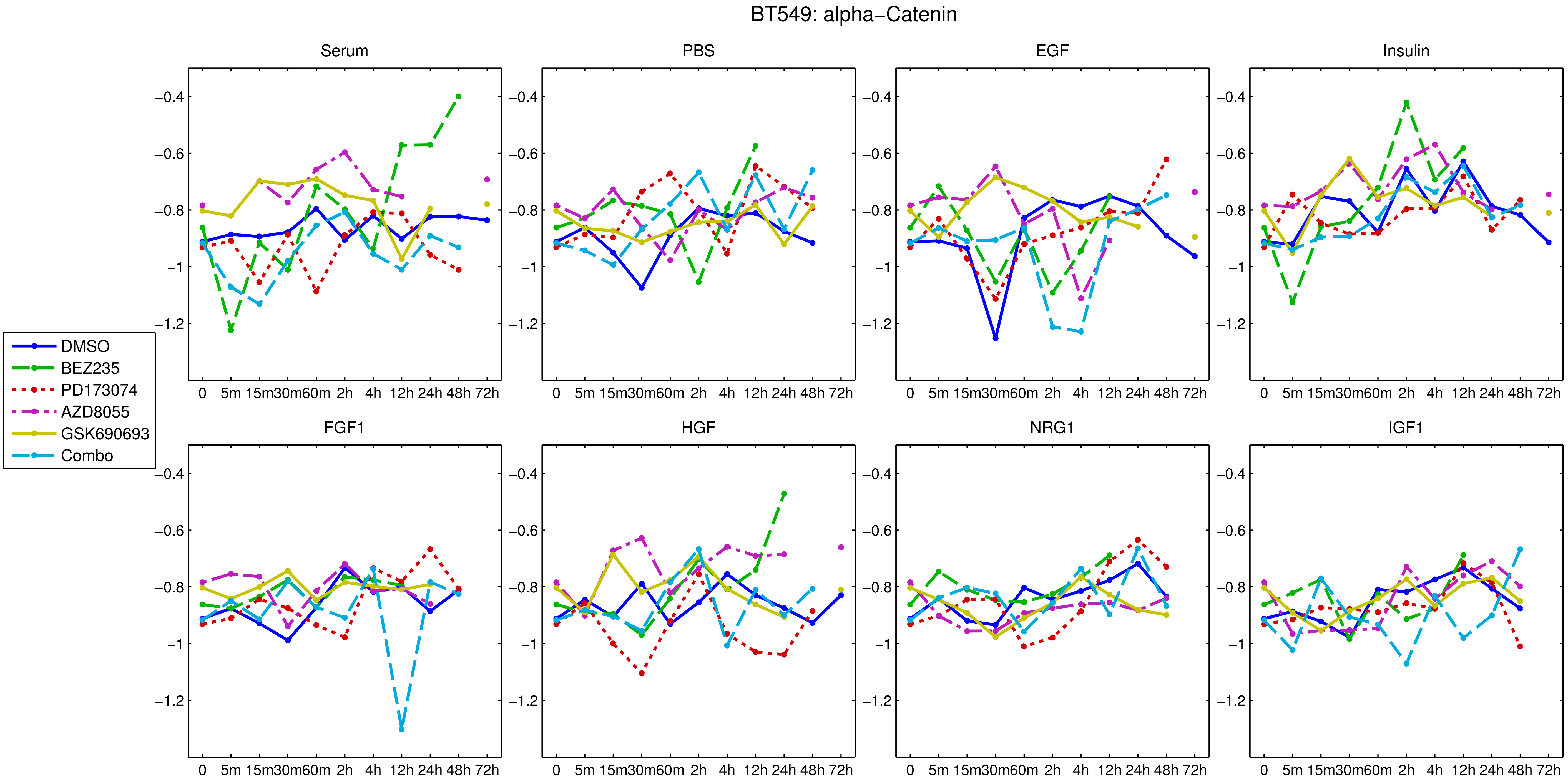
BT549: alpha−Catenin
Serum
PBS
EGF
Insulin
FGF1
HGF
NRG1
IGF1
DMSO
BEZ235
PD173074
AZD8055
GSK690693
Combo
−0.4
−0.6
−0.8
−1
−1.2
0
5m
15m
30m
60m
2h
4h
12h
24h
48h
72h

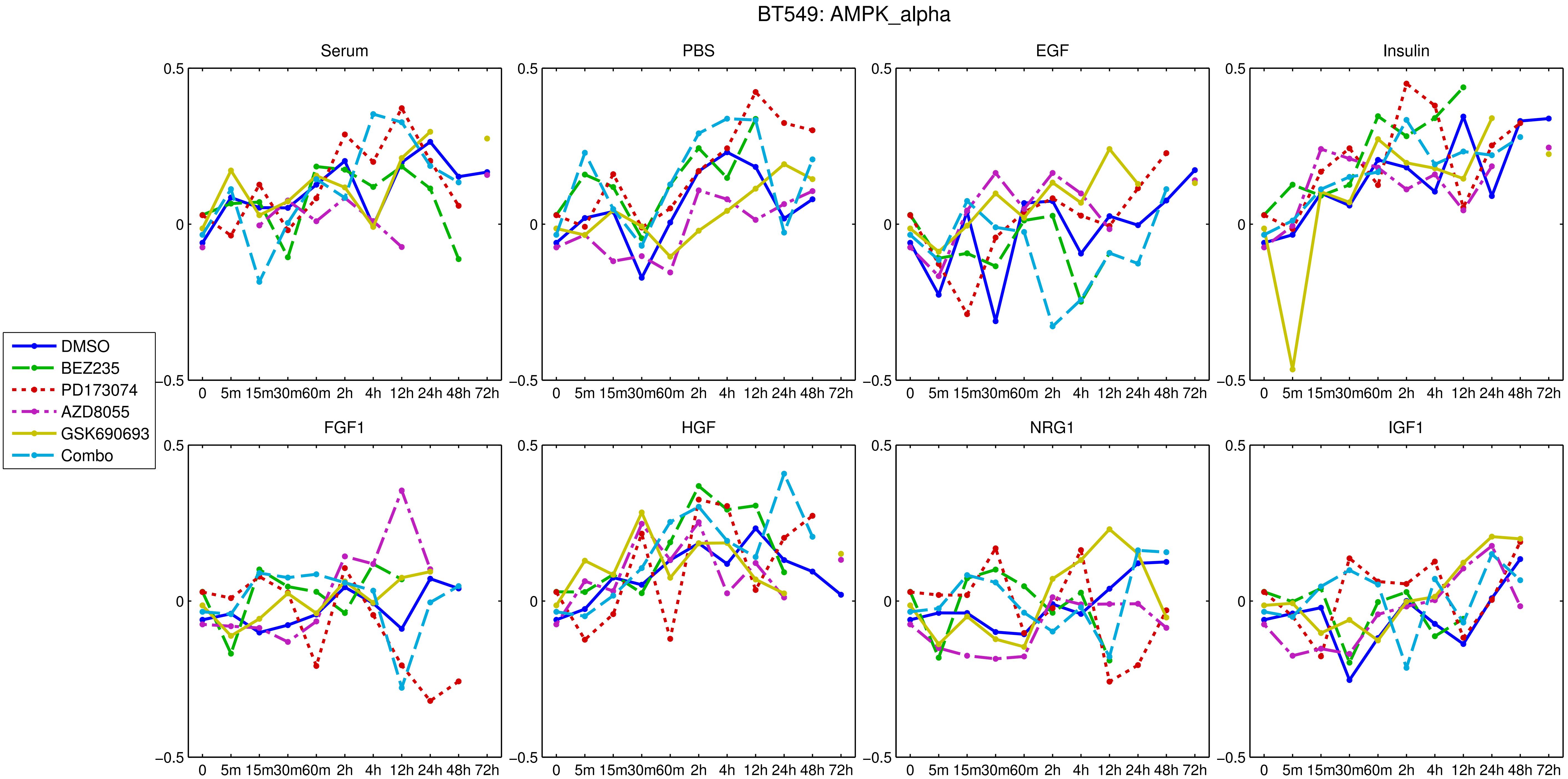
BT549: AMPK_alpha
Serum
PBS
EGF
Insulin
FGF1
HGF
NRG1
IGF1
0.5
0
−0.5
0
5m
15m
30m
60m
2h
4h
12h
24h
48h
72h
DMSO
BEZ235
PD173074
AZD8055
GSK690693
Combo

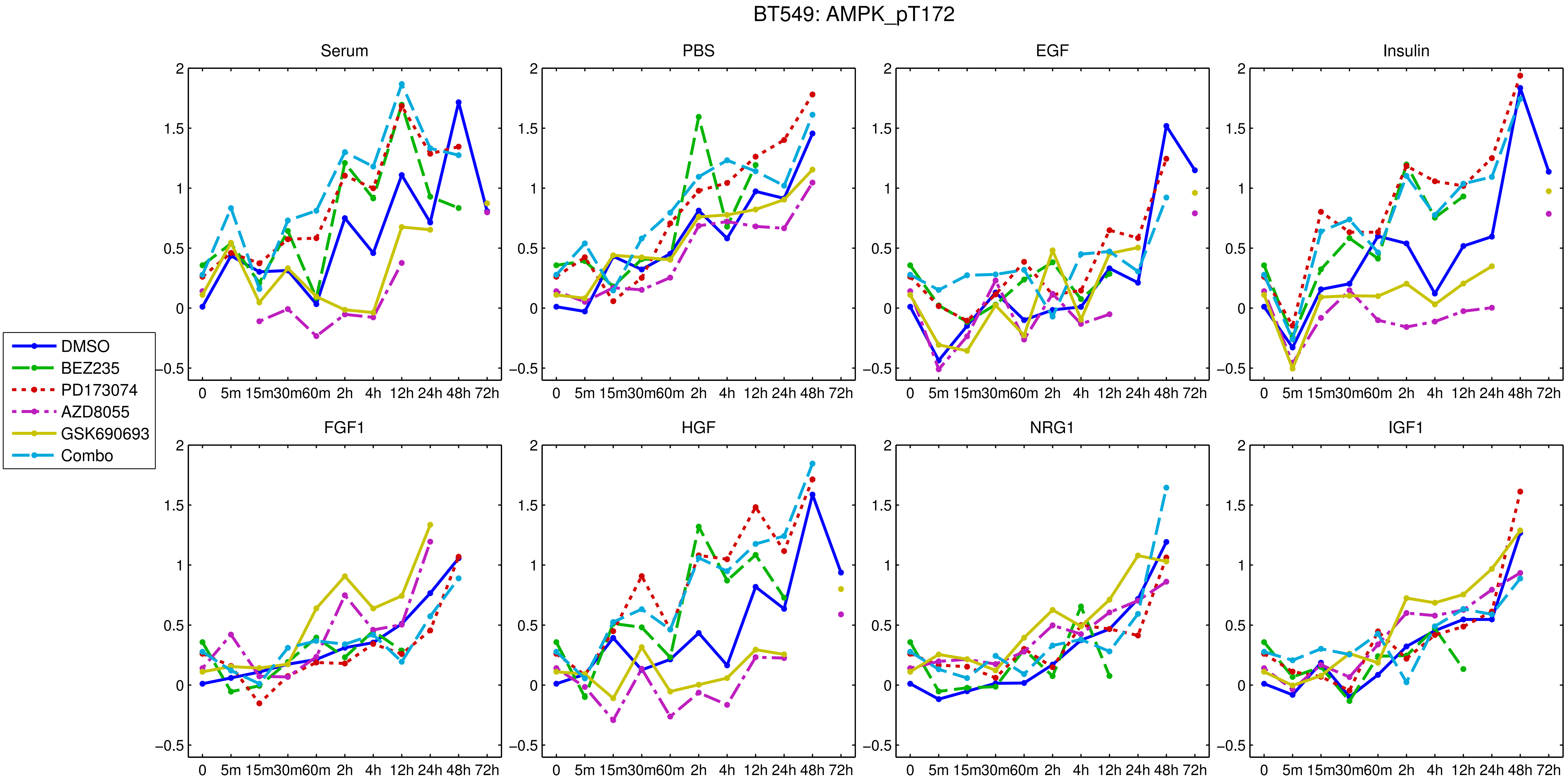
BT549: AMPK_pT172
Serum
PBS
EGF
Insulin
FGF1
HGF
NRG1
IGF1
DMSO
BEZ235
PD173074
AZD8055
GSK690693
Combo
0 5m 15m 30m 60m 2h 4h 12h 24h 48h 72h

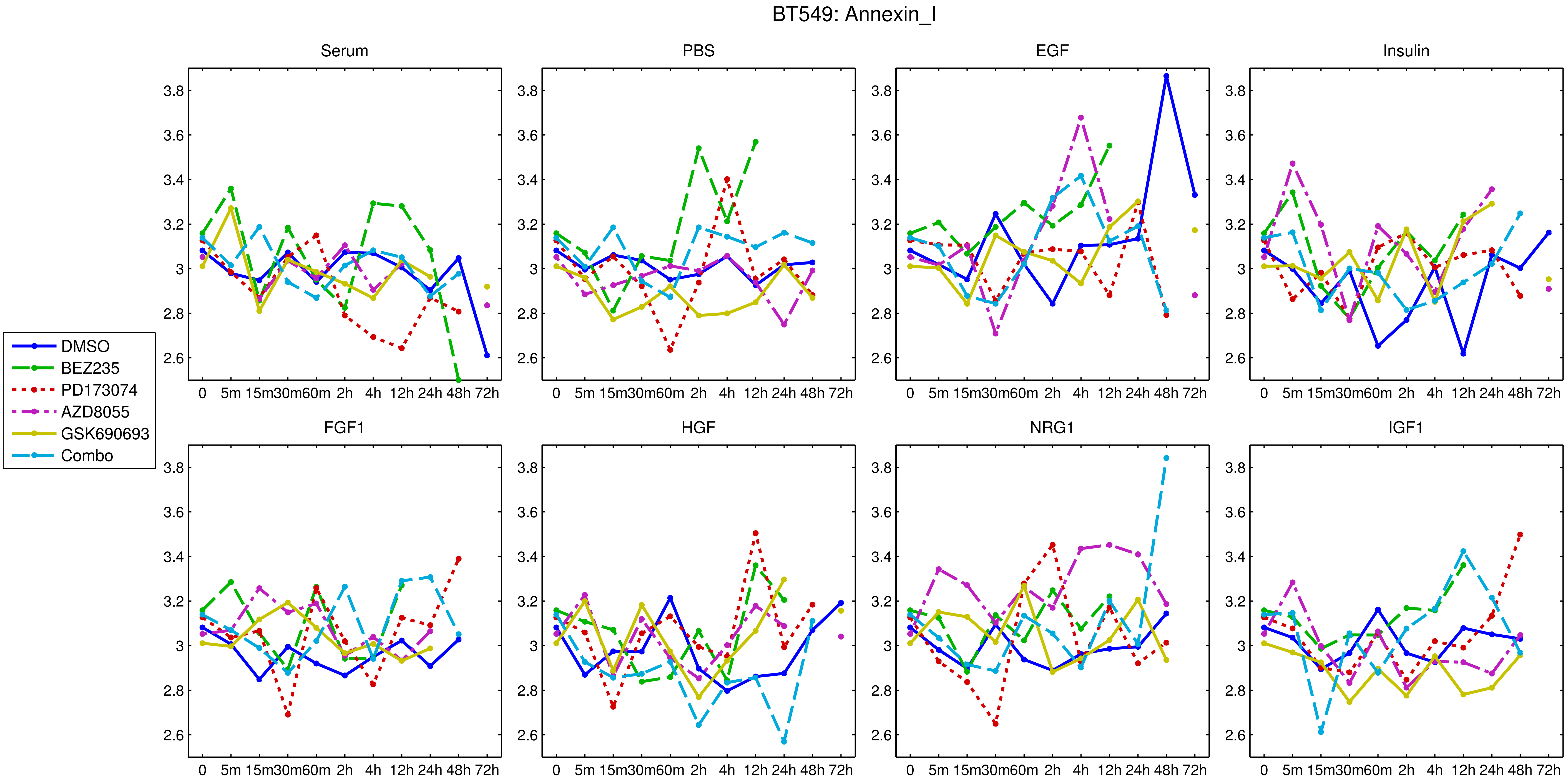
BT549: Annexin_I
Serum
PBS
EGF
Insulin
FGF1
HGF
NRG1
IGF1
3.8
3.6
3.4
3.2
3
2.8
2.6
0
5m
15m
30m
60m
2h
4h
12h
24h
48h
72h
DMSO
BEZ235
PD173074
AZD8055
GSK690693
Combo

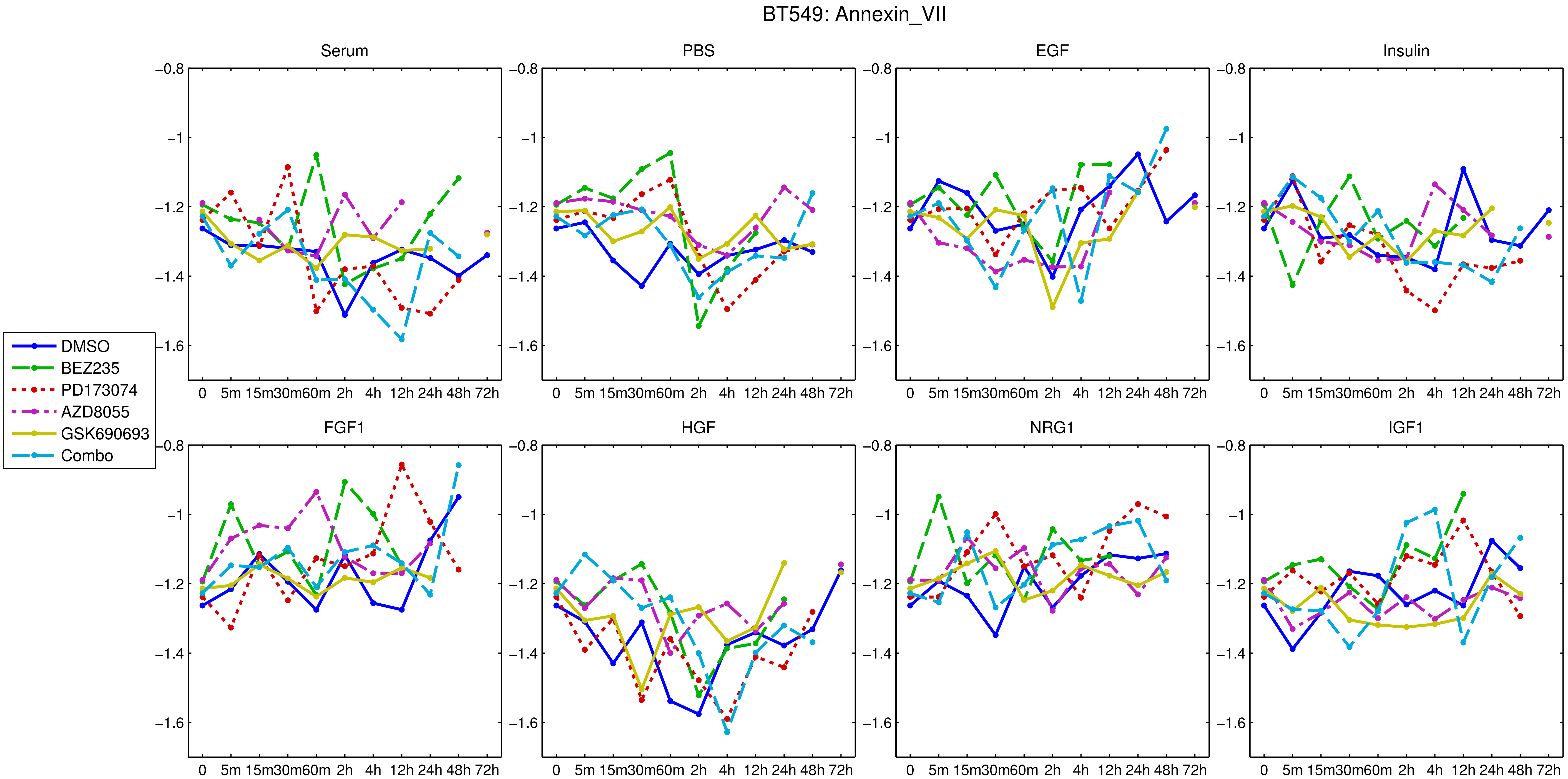
BT549: Annexin_VII
Serum
PBS
EGF
Insulin
FGF1
HGF
NRG1
IGF1
DMSO
BEZ235
PD173074
AZD8055
GSK690693
Combo
−0.8
−1
−1.2
−1.4
−1.6
0
5m
15m
30m
60m
2h
4h
12h
24h
48h
72h

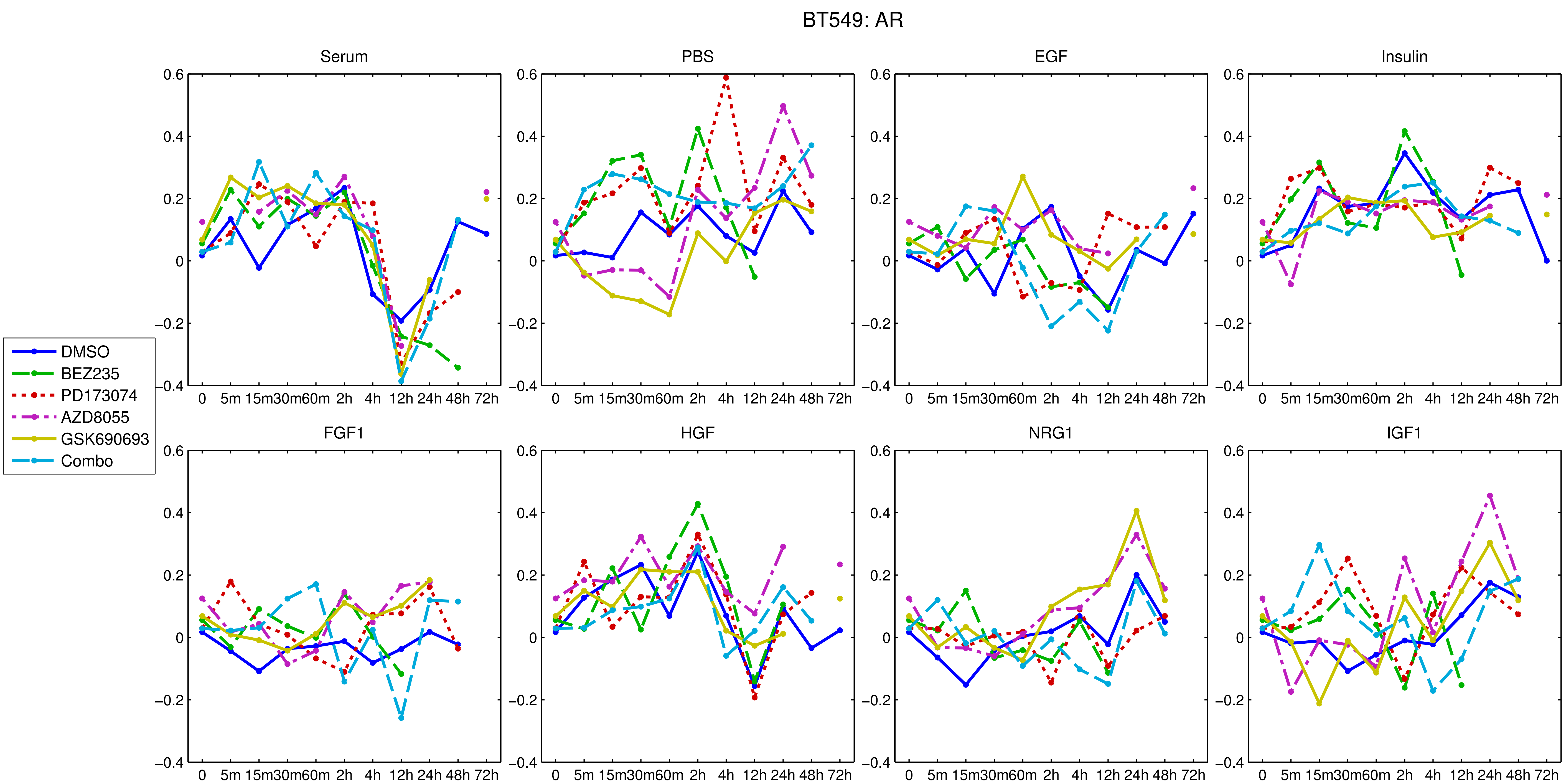
BT549: AR
Serum
PBS
EGF
Insulin
FGF1
HGF
NRG1
IGF1
DMSO
BEZ235
PD173074
AZD8055
GSK690693
Combo
0.6
0.4
0.2
0
−0.2
−0.4
0 5m 15m 30m 60m 2h 4h 12h 24h 48h 72h

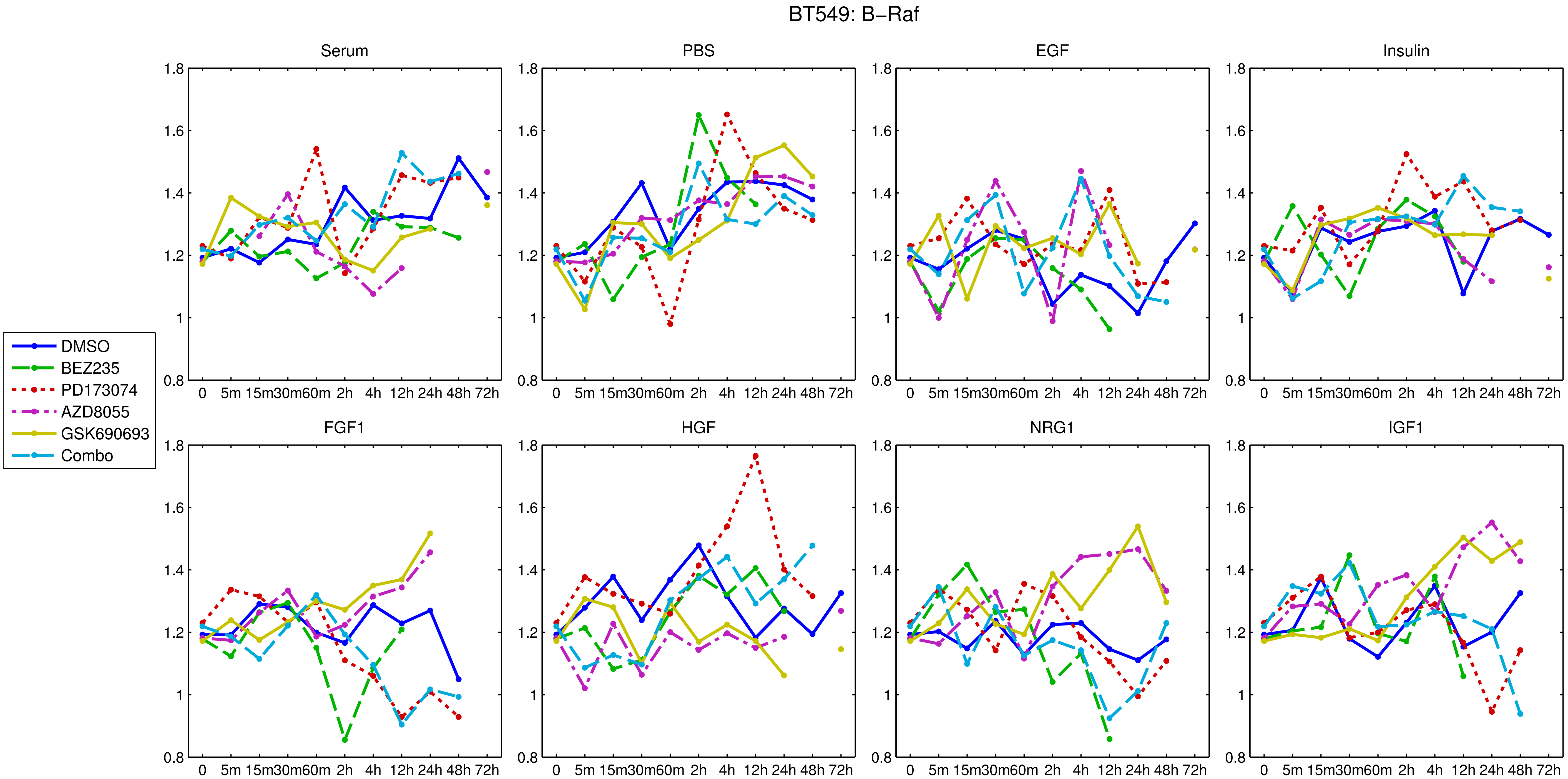
BT549: B−Raf
Serum
PBS
EGF
Insulin
FGF1
HGF
NRG1
IGF1
1.8
1.6
1.4
1.2
1
0.8
0 5m 15m 30m 60m 2h 4h 12h 24h 48h 72h
DMSO
BEZ235
PD173074
AZD8055
GSK690693
Combo

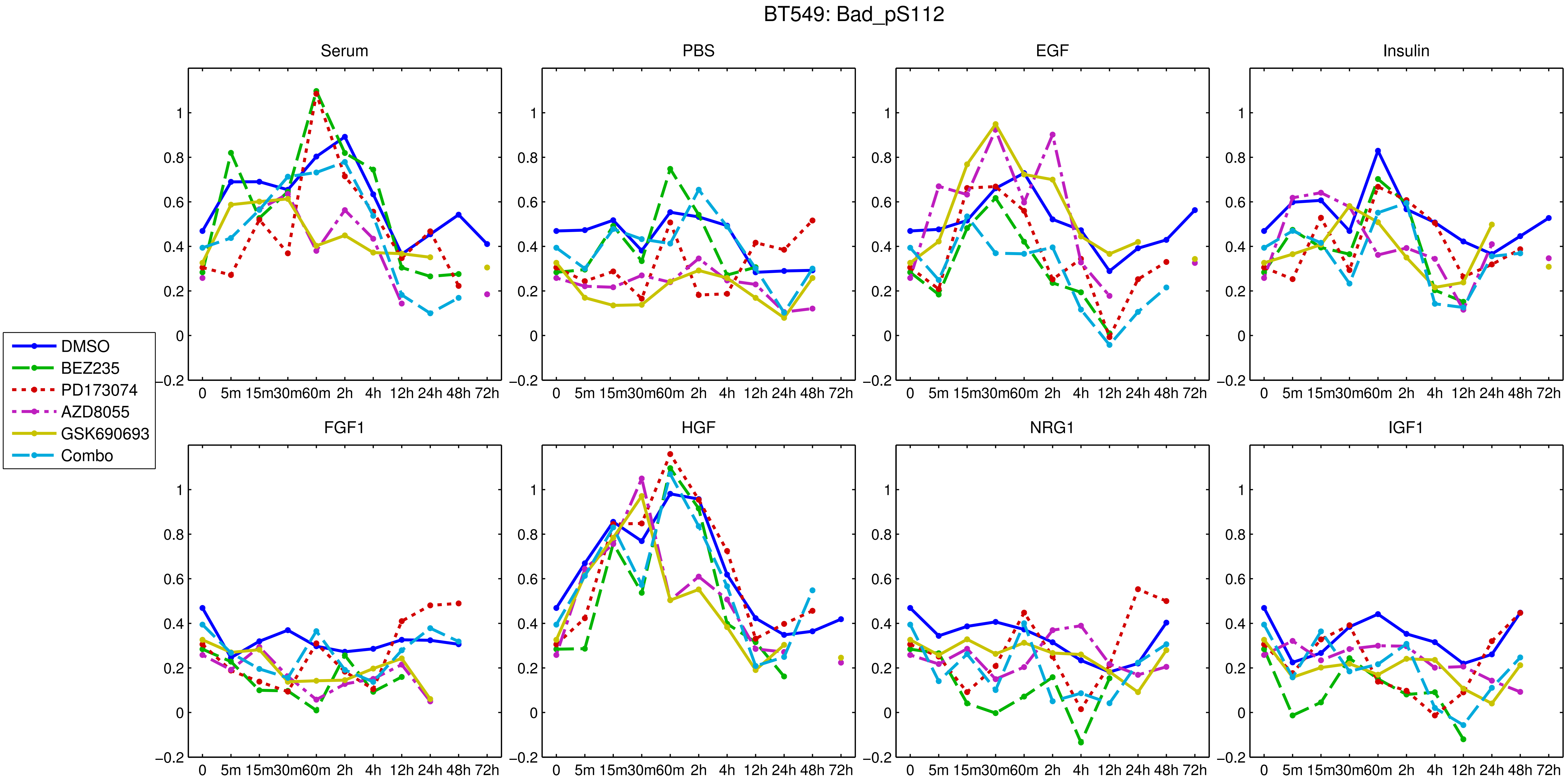
BT549: Bad_pS112
Serum
PBS
EGF
Insulin
FGF1
HGF
NRG1
IGF1
DMSO
BEZ235
PD173074
AZD8055
GSK690693
Combo
0 5m 15m 30m 60m 2h 4h 12h 24h 48h 72h

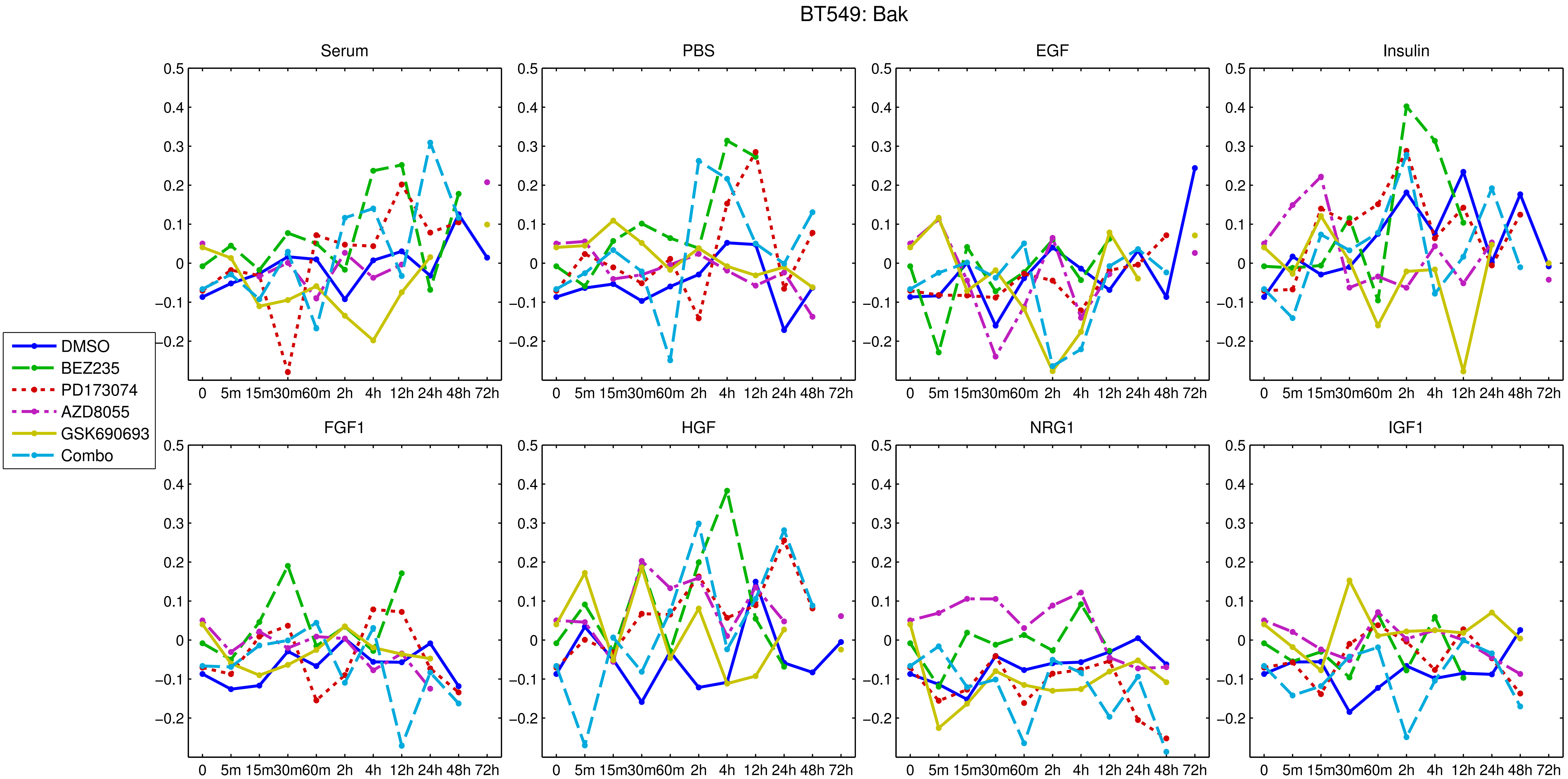
BT549: Bak
Serum
PBS
EGF
Insulin
FGF1
HGF
NRG1
IGF1
DMSO
BEZ235
PD173074
AZD8055
GSK690693
Combo
0.5
0.4
0.3
0.2
0.1
0
−0.1
−0.2
0 5m 15m 30m 60m 2h 4h 12h 24h 48h 72h

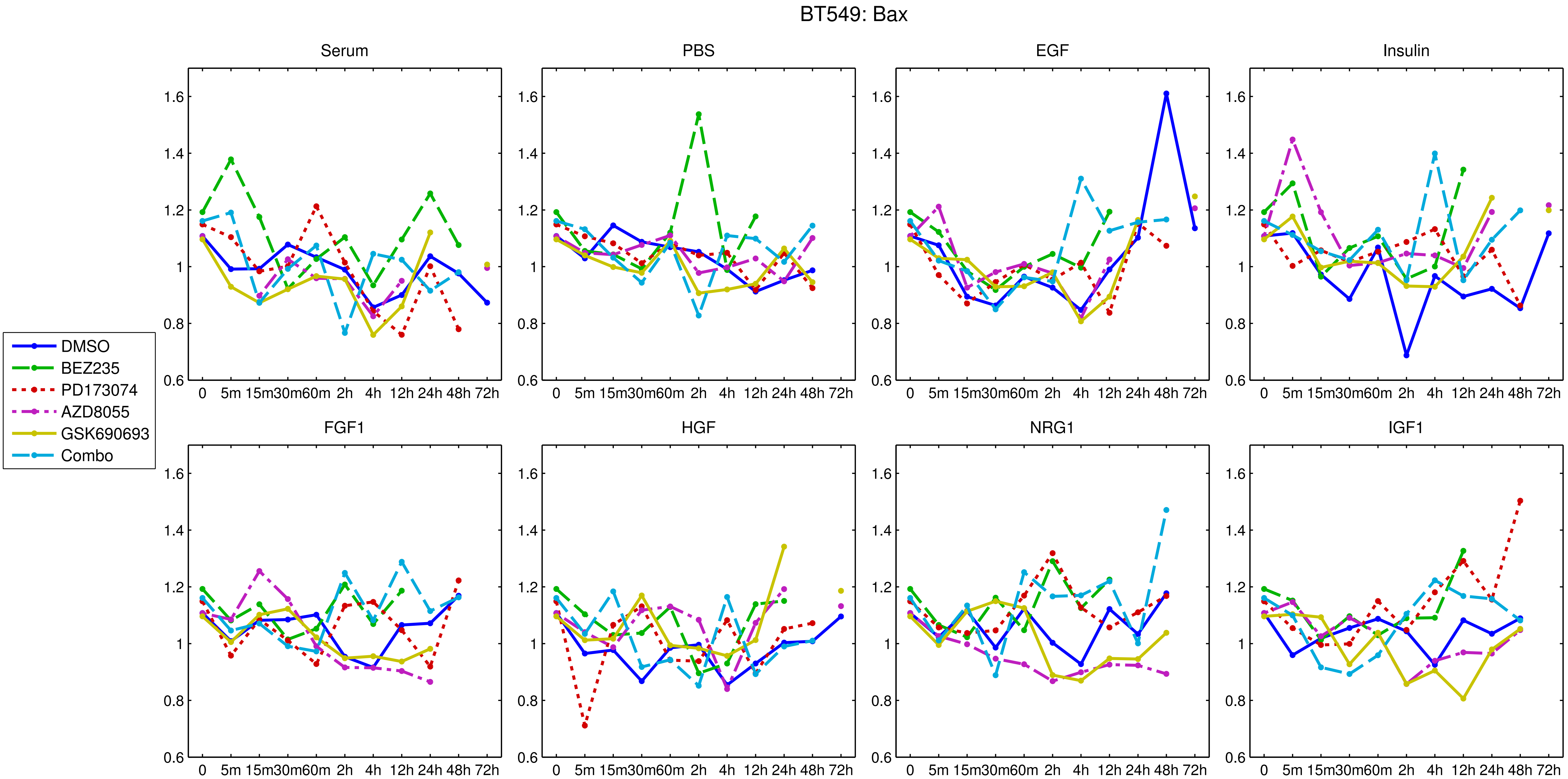
BT549: Bax
Serum
PBS
EGF
Insulin
FGF1
HGF
NRG1
IGF1
1.6
1.4
1.2
1
0.8
0.6
0 5m 15m 30m 60m 2h 4h 12h 24h 48h 72h
DMSO
BEZ235
PD173074
AZD8055
GSK690693
Combo

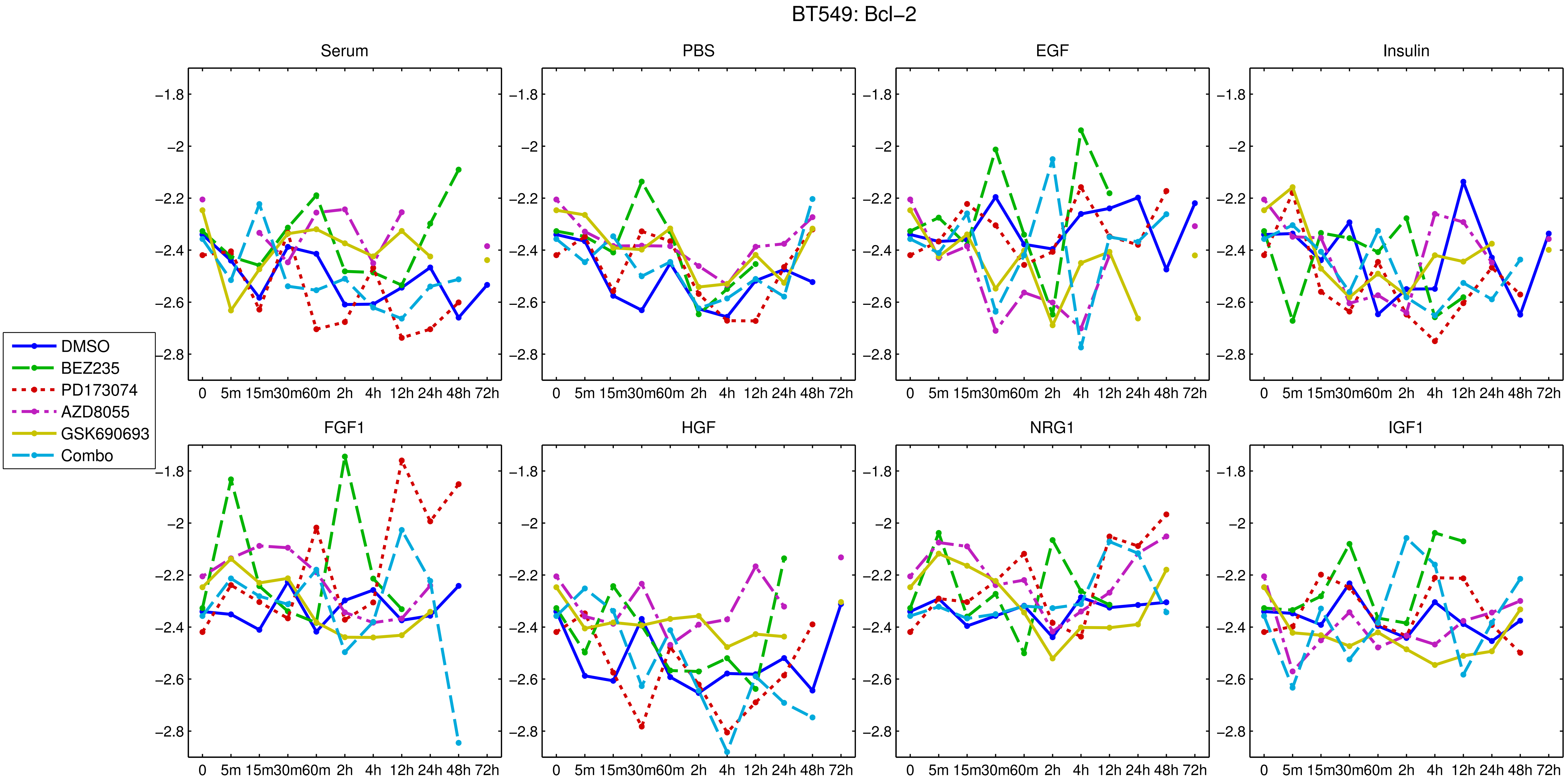

BT549: Bcl–2
Serum
PBS
EGF
Insulin
FGF1
HGF
NRG1
IGF1
DMSO
BEZ235
PD173074
AZD8055
GSK690693
Combo
−1.8
−2
−2.2
−2.4
−2.6
−2.8
0 5m 15m 30m 60m 2h 4h 12h 24h 48h 72h

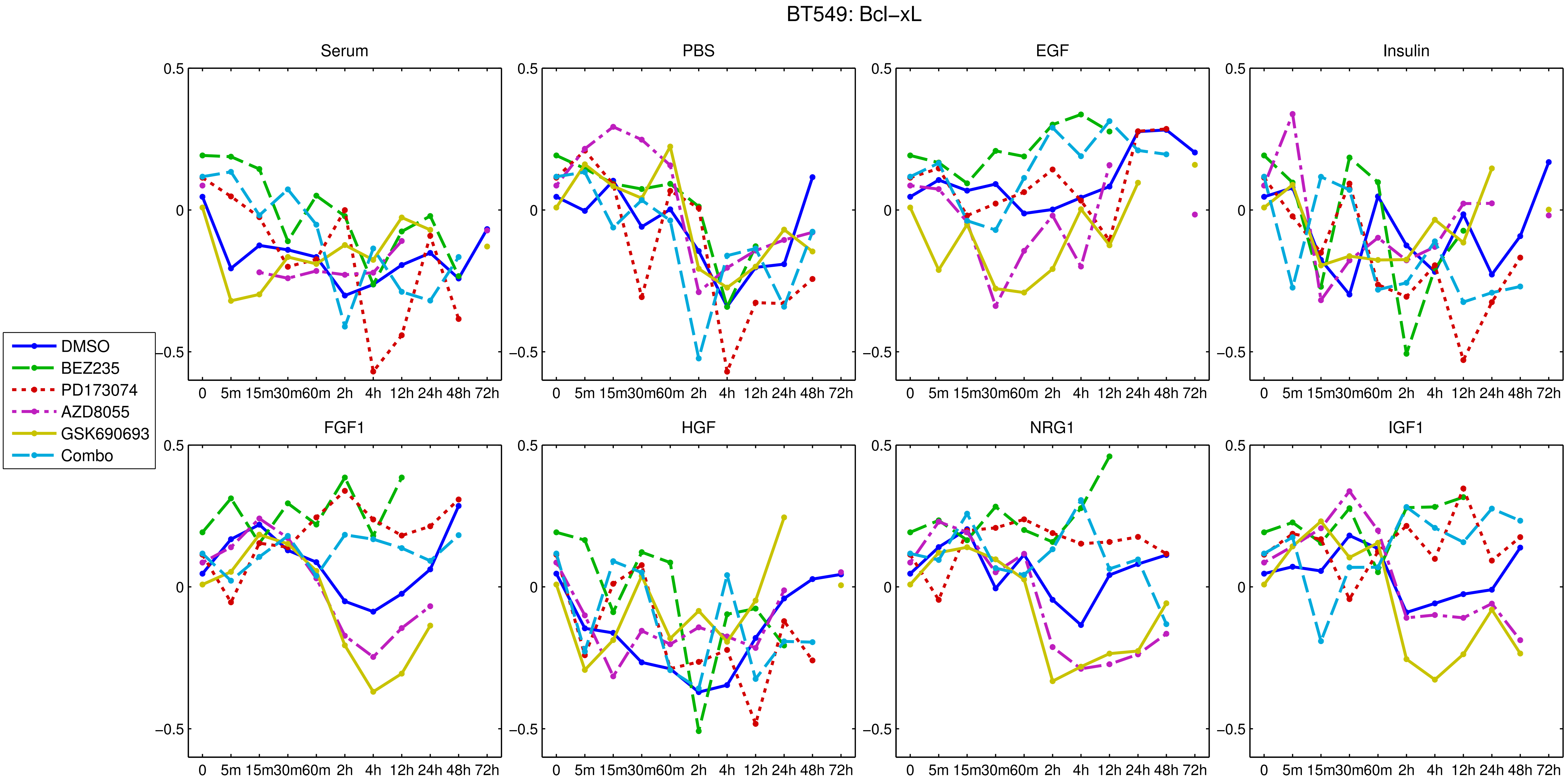
BT549: Bcl−xL
Serum
PBS
EGF
Insulin
FGF1
HGF
NRG1
IGF1
DMSO
BEZ235
PD173074
AZD8055
GSK690693
Combo
0.5
0
−0.5
0
5m
15m
30m
60m
2h
4h
12h
24h
48h
72h

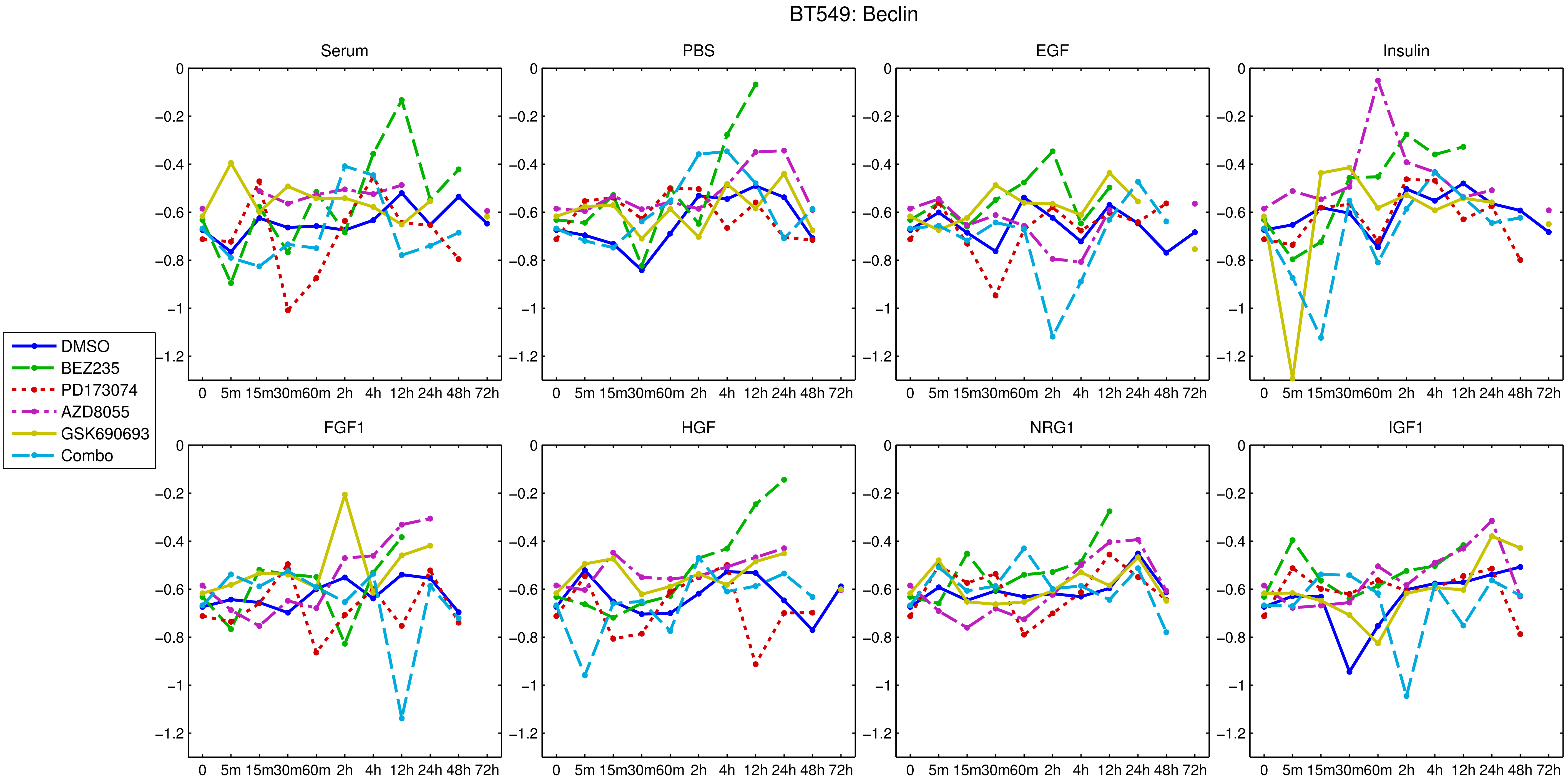

BT549: Beclin
Serum
PBS
EGF
Insulin
FGF1
HGF
NRG1
IGF1
DMSO
BEZ235
PD173074
AZD8055
GSK690693
Combo
0
−0.2
−0.4
−0.6
−0.8
−1
−1.2
0
5m
15m
30m
60m
2h
4h
12h
24h
48h
72h

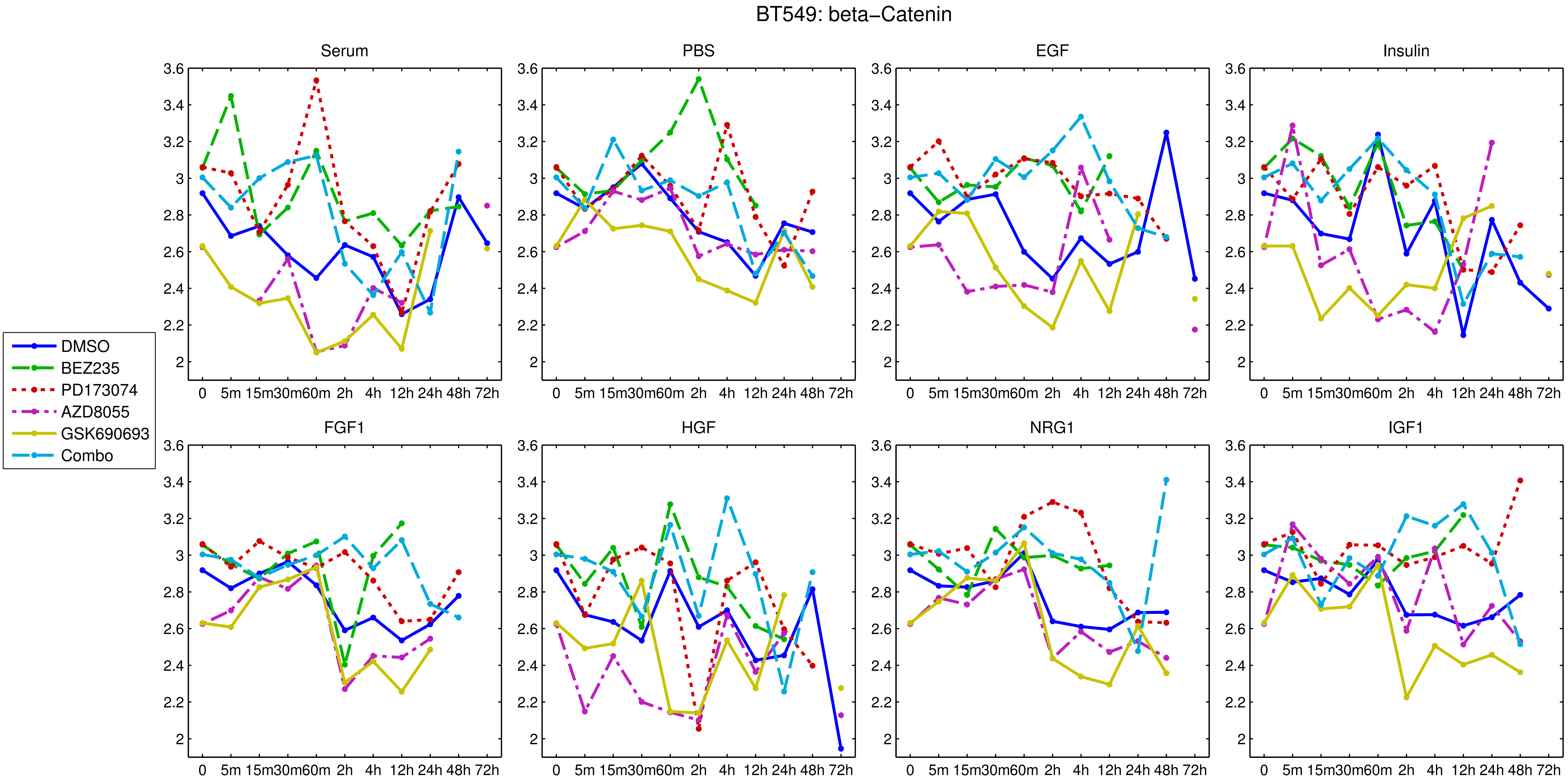
BT549: beta−Catenin
Serum
PBS
EGF
Insulin
FGF1
HGF
NRG1
IGF1
DMSO
BEZ235
PD173074
AZD8055
GSK690693
Combo
3.6
3.4
3.2
3
2.8
2.6
2.4
2.2
2
0
5m
15m
30m
60m
2h
4h
12h
24h
48h
72h

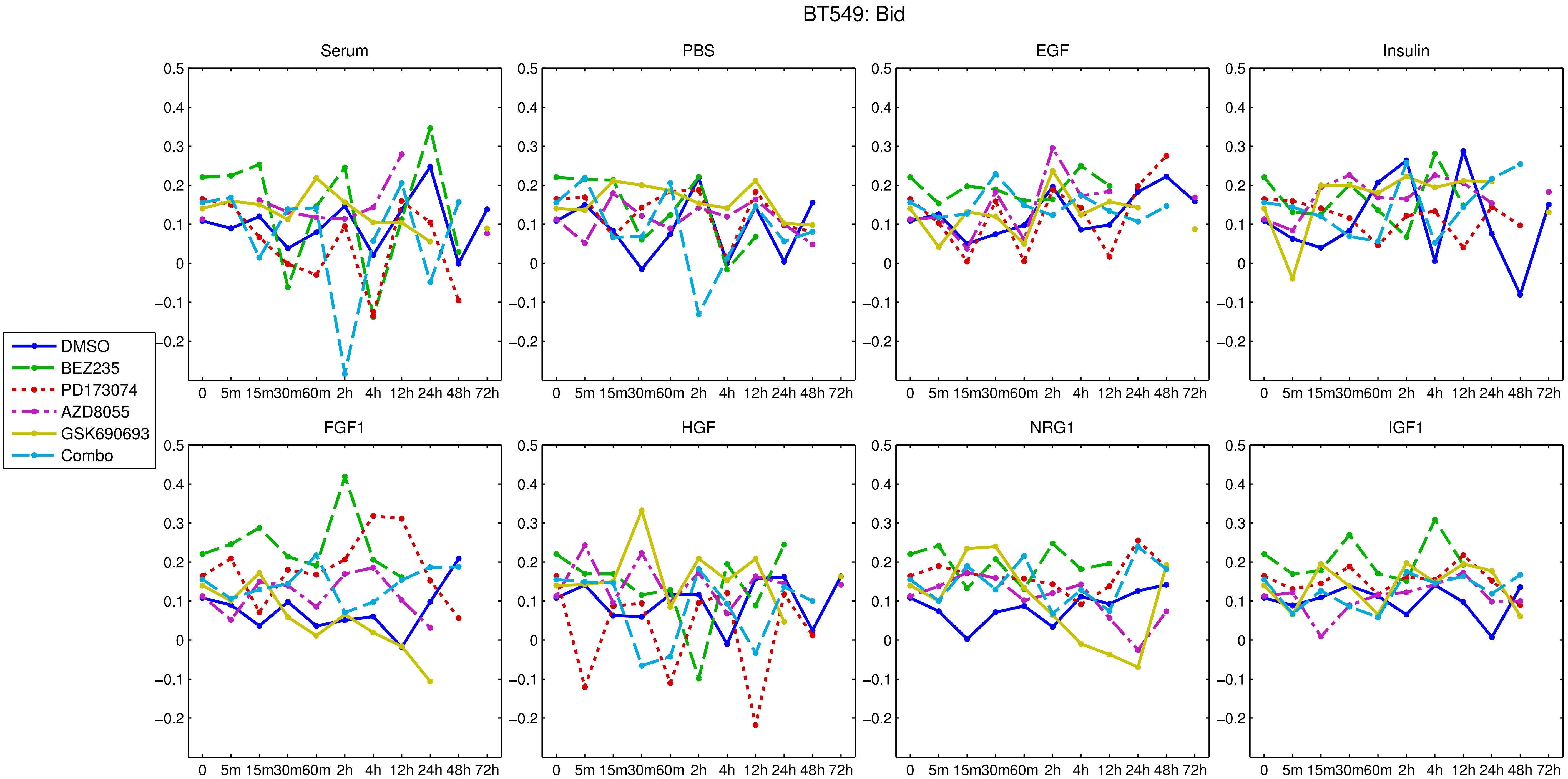
BT549: Bid
Serum
PBS
EGF
Insulin
FGF1
HGF
NRG1
IGF1
DMSO
BEZ235
PD173074
AZD8055
GSK690693
Combo
0.5
0.4
0.3
0.2
0.1
0
−0.1
−0.2
0 5m 15m 30m 60m 2h 4h 12h 24h 48h 72h

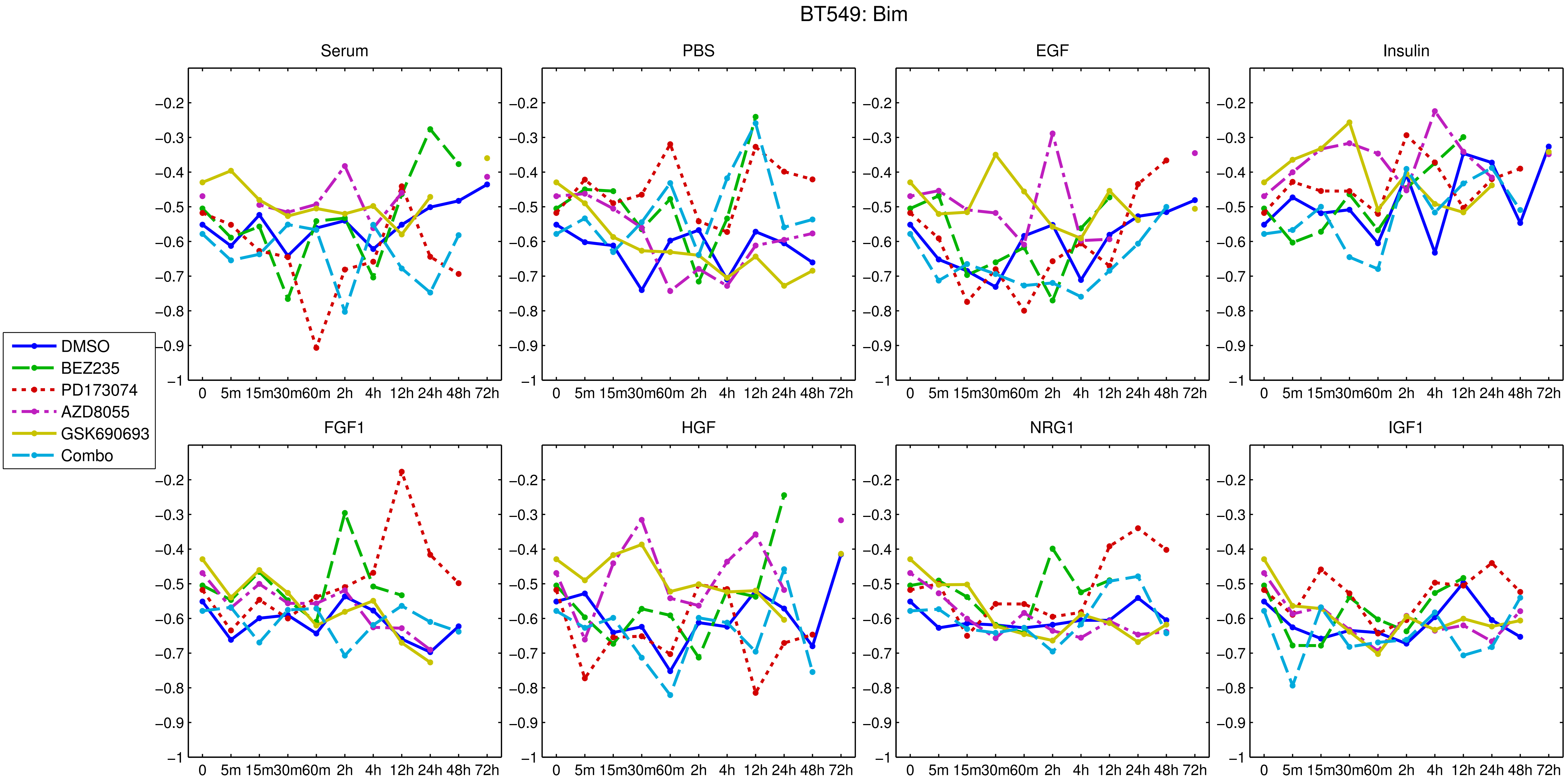

BT549: Bim
Serum
PBS
EGF
Insulin
FGF1
HGF
NRG1
IGF1
DMSO
BEZ235
PD173074
AZD8055
GSK690693
Combo
−0.2
−0.3
−0.4
−0.5
−0.6
−0.7
−0.8
−0.9
−1
0
5m
15m
30m
60m
2h
4h
12h
24h
48h
72h

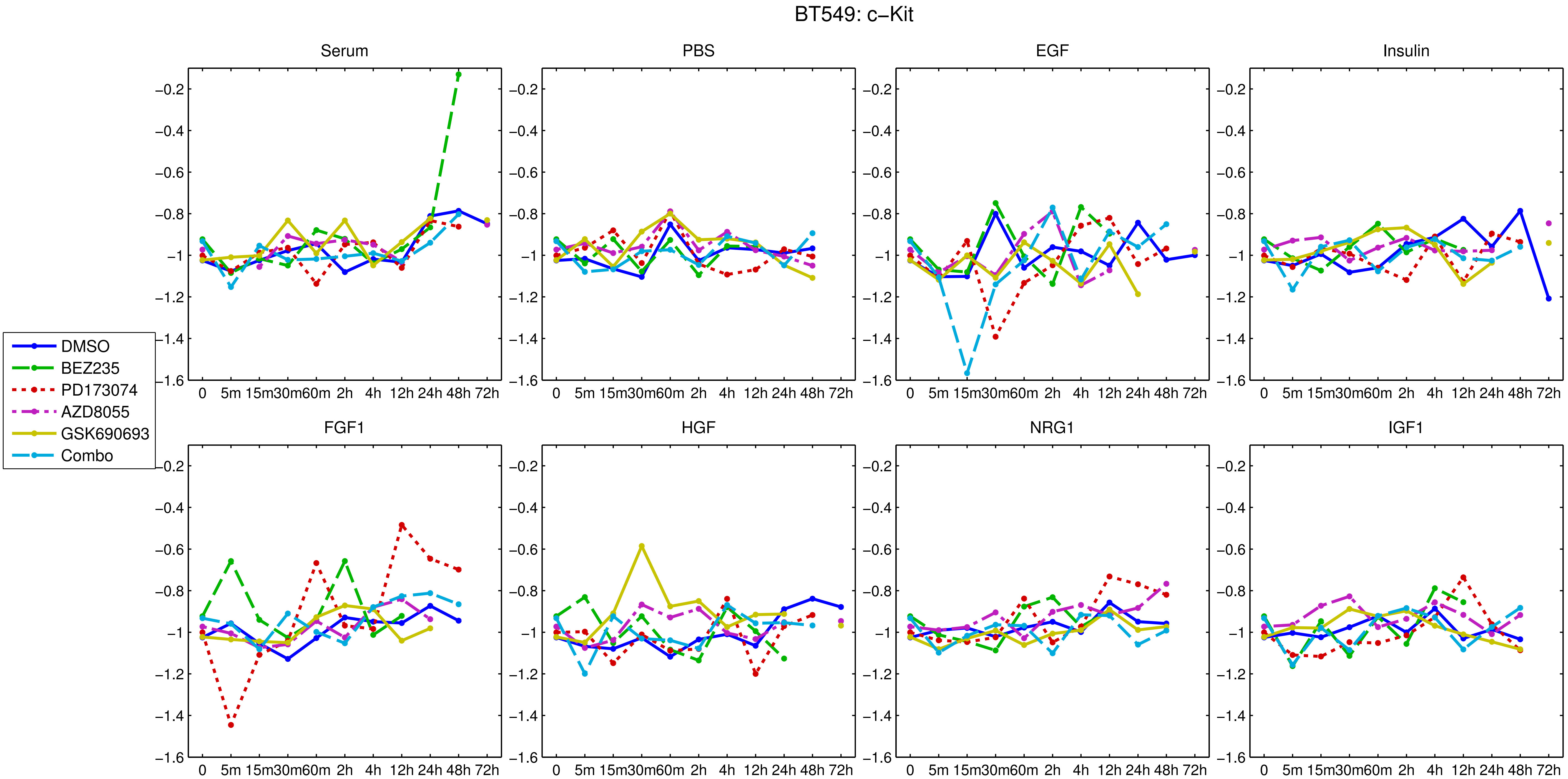

BT549: c−Kit
Serum
PBS
EGF
Insulin
FGF1
HGF
NRG1
IGF1
DMSO
BEZ235
PD173074
AZD8055
GSK690693
Combo
−0.2
−0.4
−0.6
−0.8
−1
−1.2
−1.4
−1.6
0 5m 15m 30m 60m 2h 4h 12h 24h 48h 72h

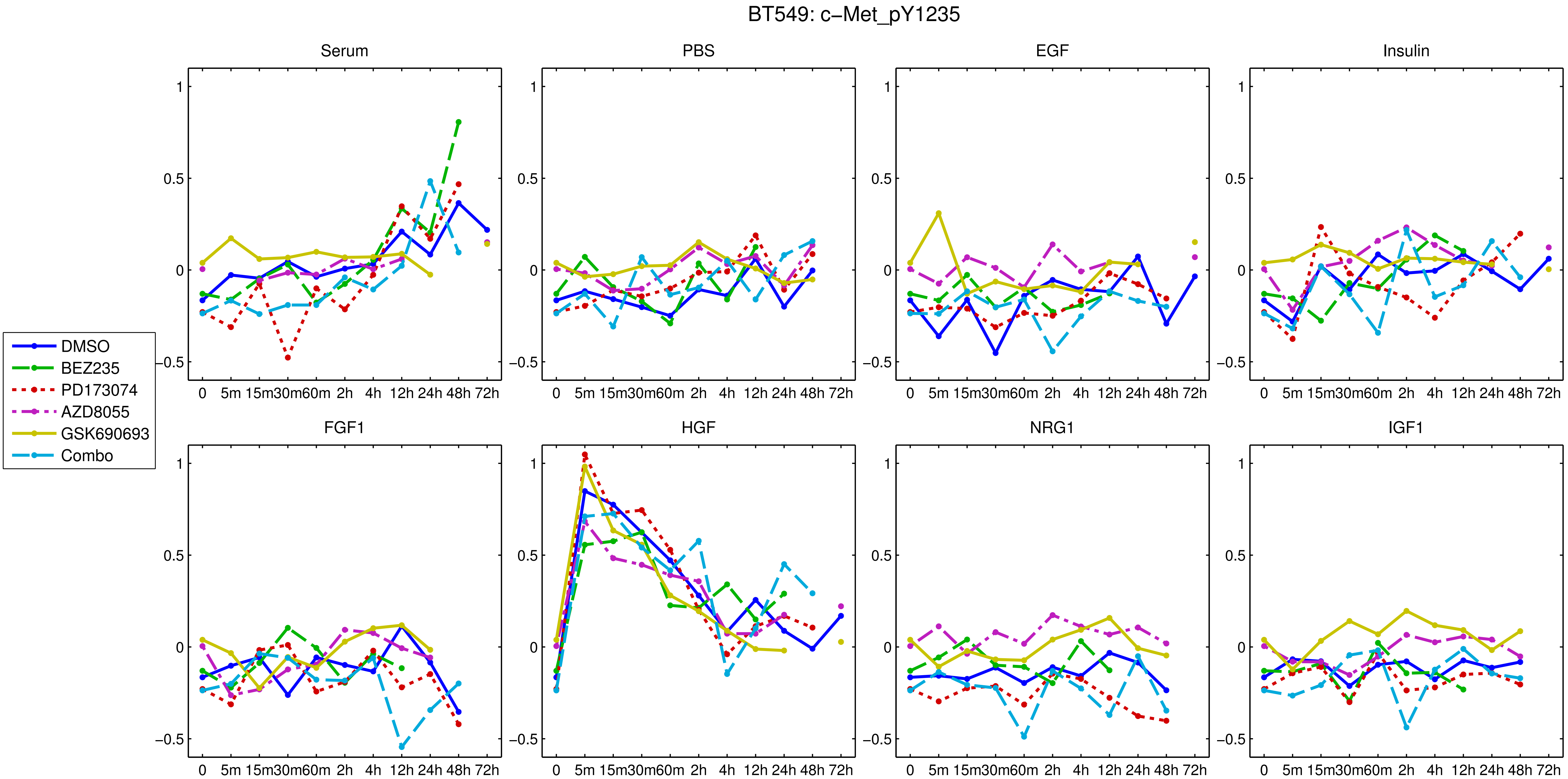
BT549: c–Met_pY1235
Serum
PBS
EGF
Insulin
FGF1
HGF
NRG1
IGF1
DMSO
BEZ235
PD173074
AZD8055
GSK690693
Combo
0 5m 15m 30m 60m 2h 4h 12h 24h 48h 72h

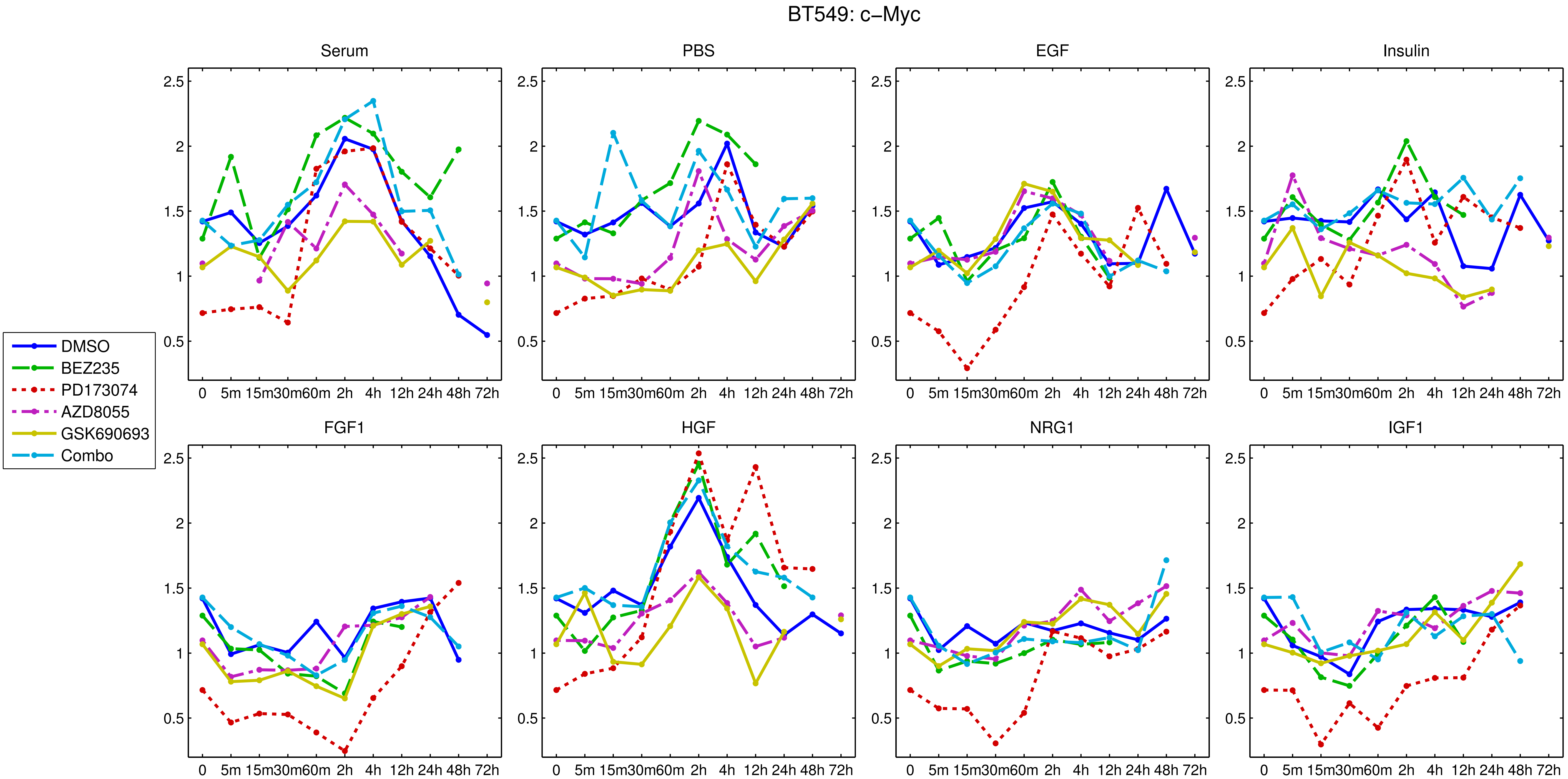

BT549: c–Myc
Serum
PBS
EGF
Insulin
FGF1
HGF
NRG1
IGF1
DMSO
BEZ235
PD173074
AZD8055
GSK690693
Combo
2.5
2
1.5
1
0.5
0 5m 15m30m60m 2h 4h 12h 24h 48h 72h

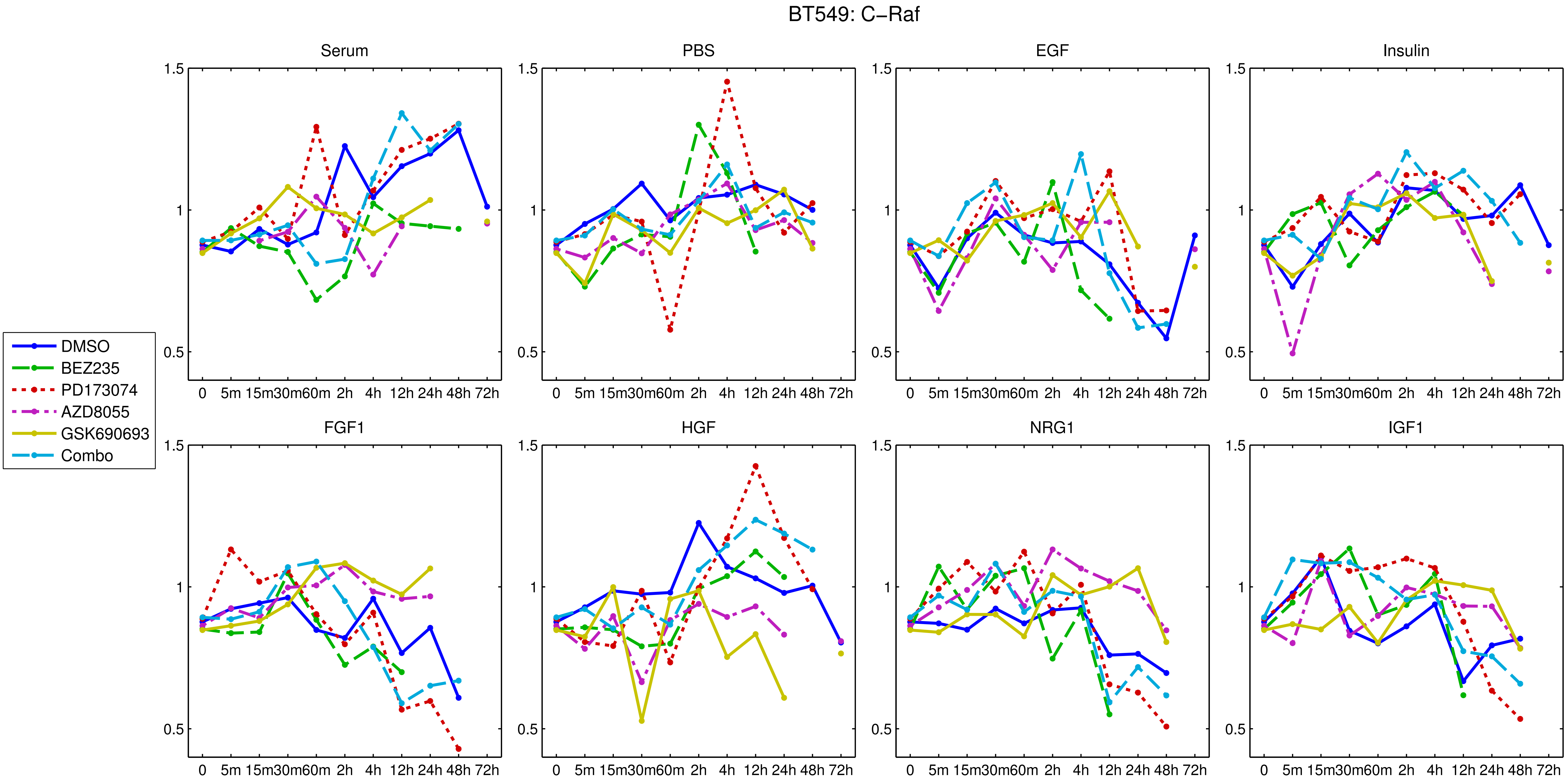
BT549: C−Raf
Serum
PBS
EGF
Insulin
FGF1
HGF
NRG1
IGF1
1.5
1
0.5
0
5m
15m
30m
60m
2h
4h
12h
24h
48h
72h
DMSO
BEZ235
PD173074
AZD8055
GSK690693
Combo

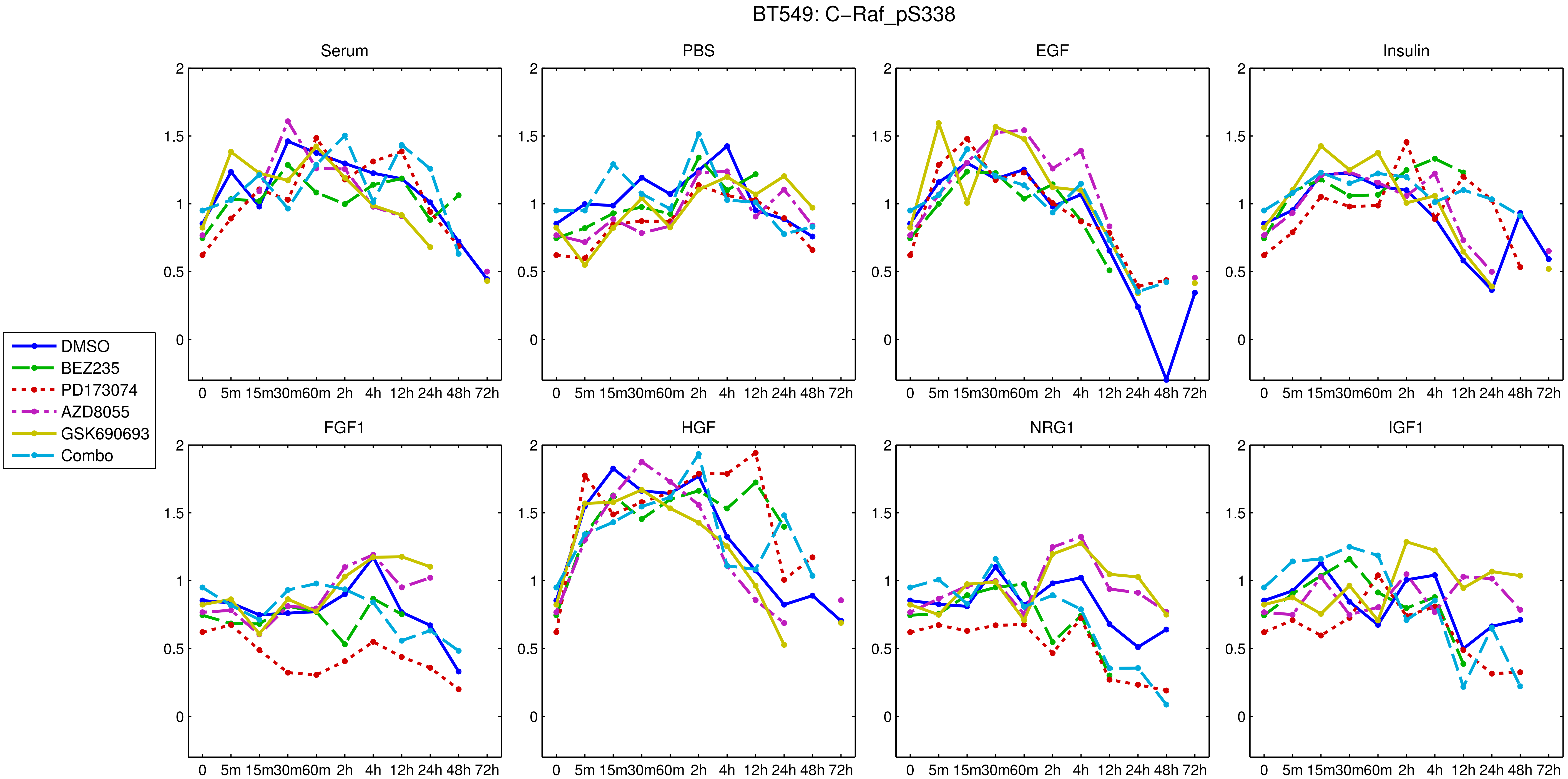
BT549: C−Raf_pS338
Serum
PBS
EGF
Insulin
FGF1
HGF
NRG1
IGF1
0 5m 15m30m60m 2h 4h 12h 24h 48h 72h
DMSO
BEZ235
PD173074
AZD8055
GSK690693
Combo

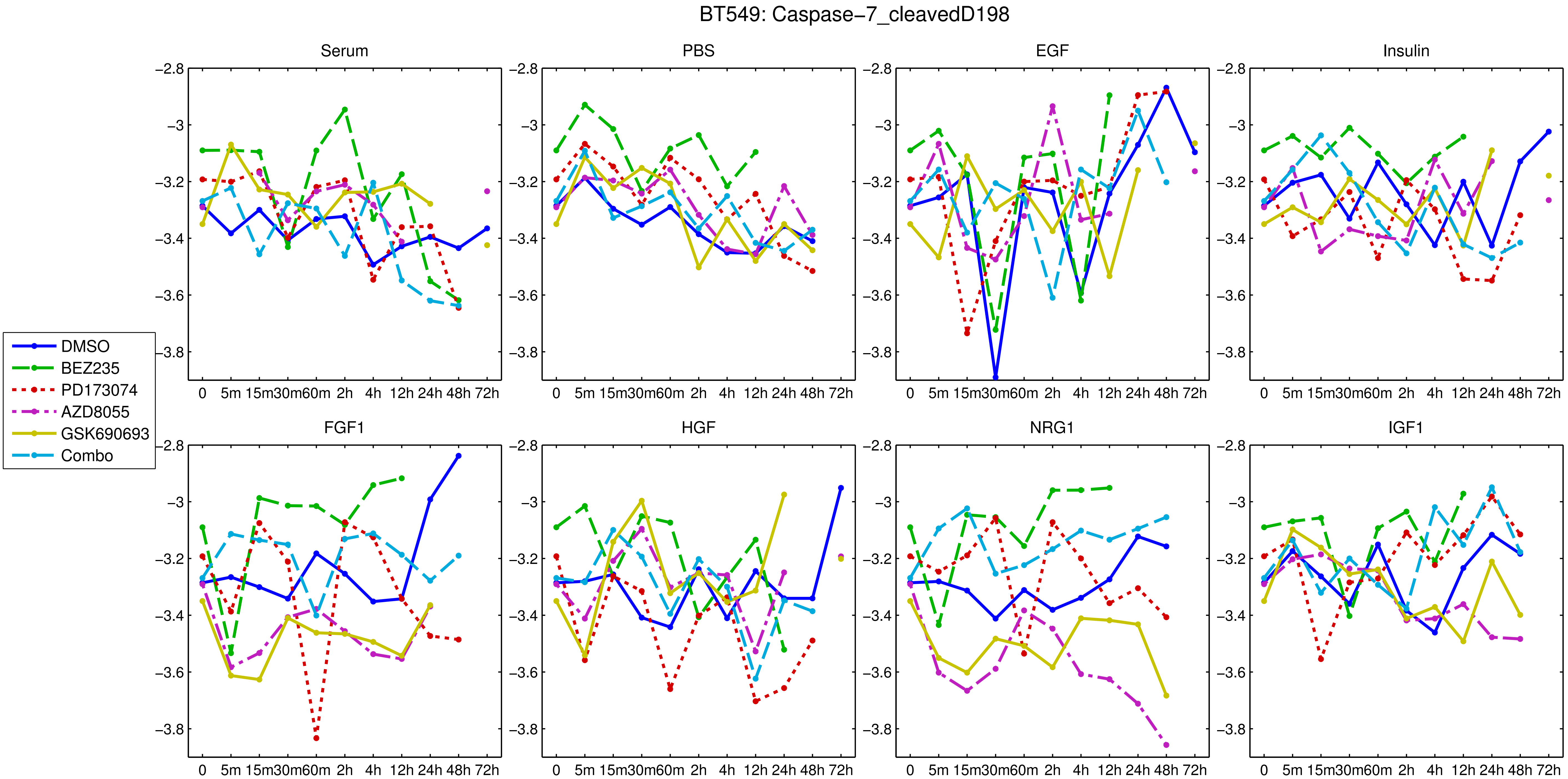
BT549: Caspase−7_cleavedD198
Serum
PBS
EGF
Insulin
FGF1
HGF
NRG1
IGF1
DMSO
BEZ235
PD173074
AZD8055
GSK690693
Combo
0 5m 15m 30m 60m 2h 4h 12h 24h 48h 72h
−2.8
−3
−3.2
−3.4
−3.6
−3.8

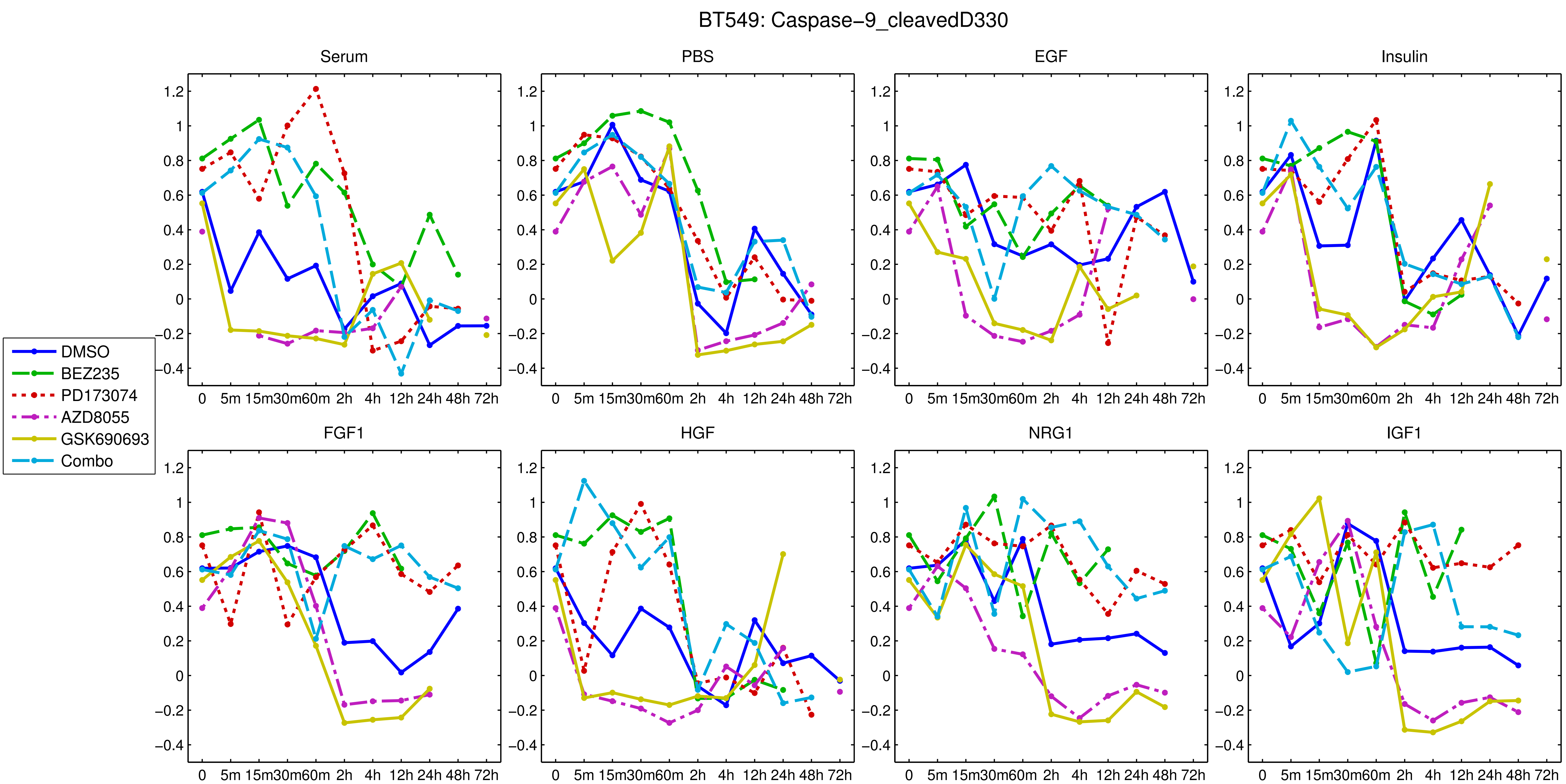
BT549: Caspase−9_cleavedD330
Serum
PBS
EGF
Insulin
FGF1
HGF
NRG1
IGF1
DMSO
BEZ235
PD173074
AZD8055
GSK690693
Combo
1.2
1
0.8
0.6
0.4
0.2
0
−0.2
−0.4
0
5m
15m
30m
60m
2h
4h
12h
24h
48h
72h

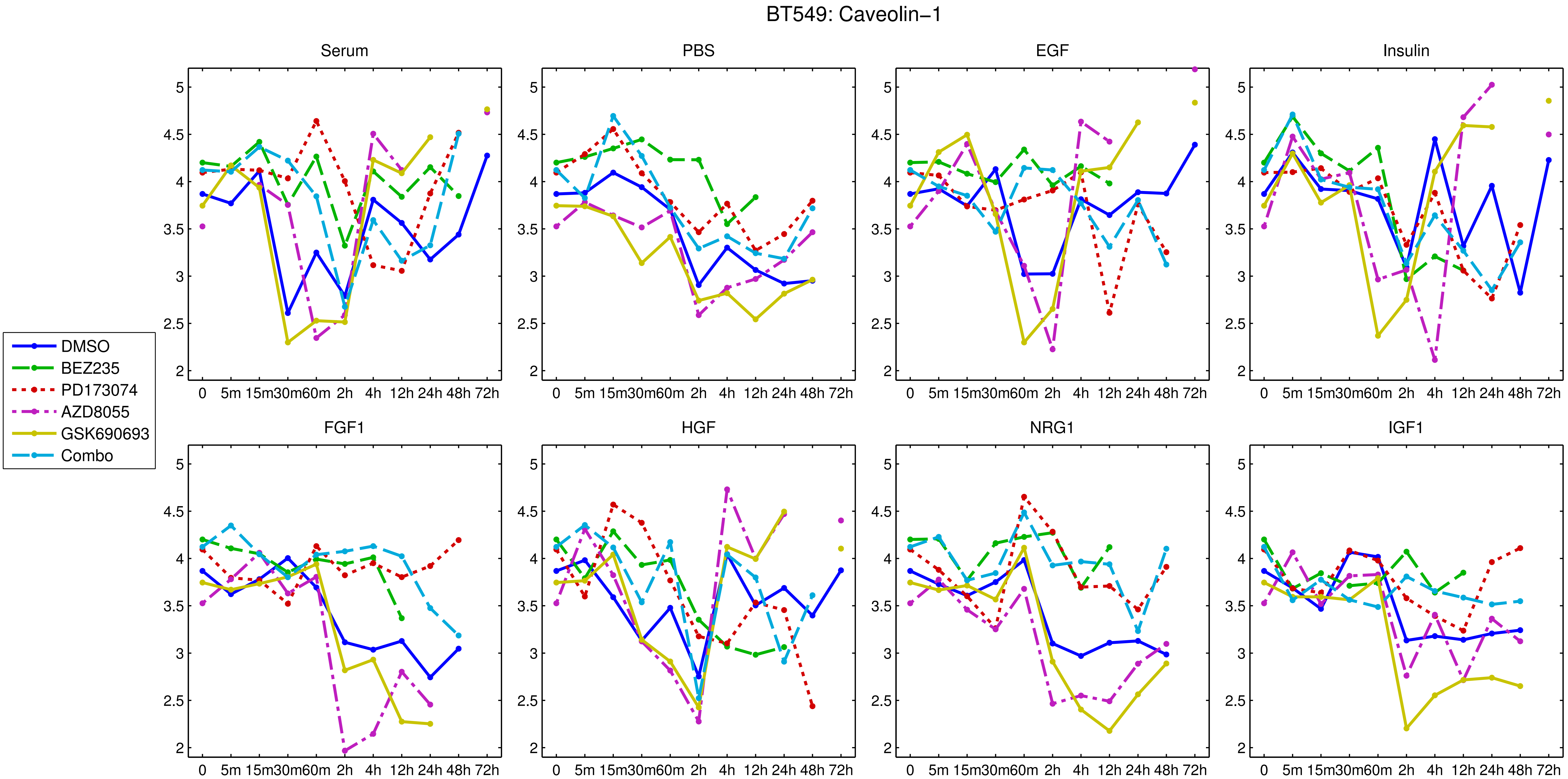
BT549: Caveolin−1
Serum
PBS
EGF
Insulin
FGF1
HGF
NRG1
IGF1
DMSO
BEZ235
PD173074
AZD8055
GSK690693
Combo
0 5m 15m 30m 60m 2h 4h 12h 24h 48h 72h
2 2.5 3 3.5 4 4.5 5

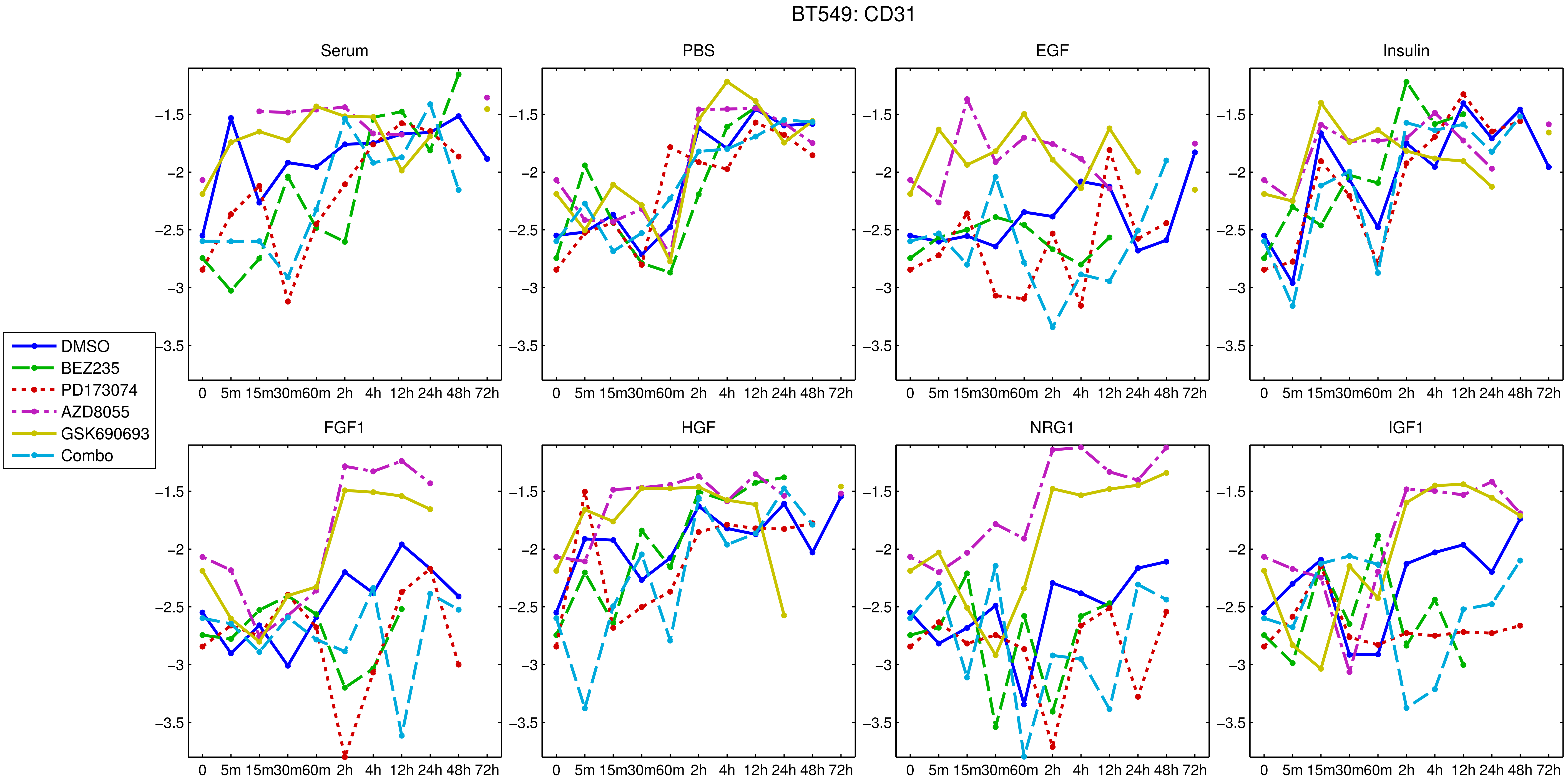

BT549: CD31
Serum
PBS
EGF
Insulin
FGF1
HGF
NRG1
IGF1
−1.5
−2
−2.5
−3
−3.5
0
5m
15m
30m
60m
2h
4h
12h
24h
48h
72h
DMSO
BEZ235
PD173074
AZD8055
GSK690693
Combo

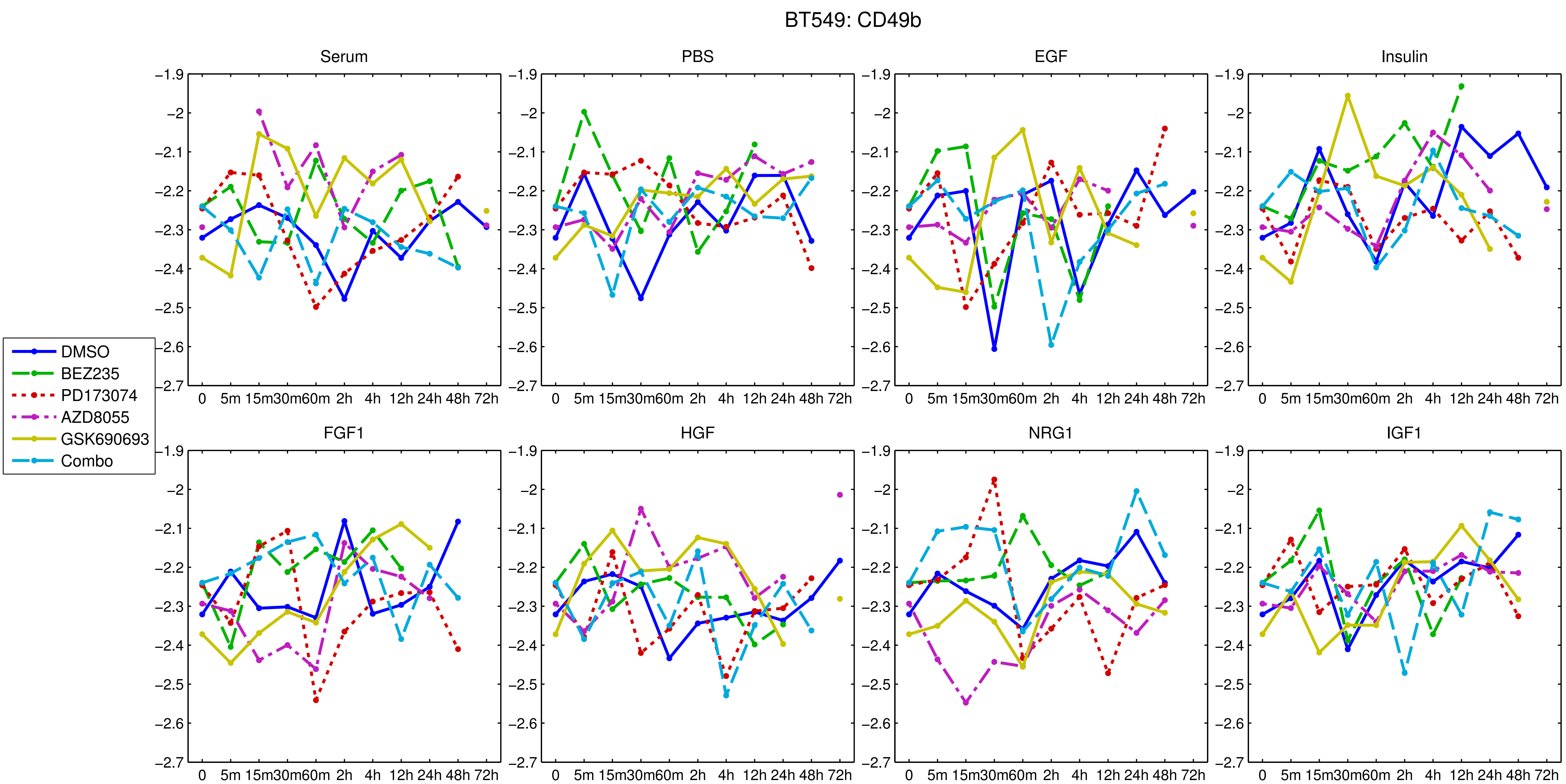
BT549: CD49b
Serum
PBS
EGF
Insulin
FGF1
HGF
NRG1
IGF1
DMSO
BEZ235
PD173074
AZD8055
GSK690693
Combo
−1.9
−2
−2.1
−2.2
−2.3
−2.4
−2.5
−2.6
−2.7
0
5m
15m
30m
60m
2h
4h
12h
24h
48h
72h

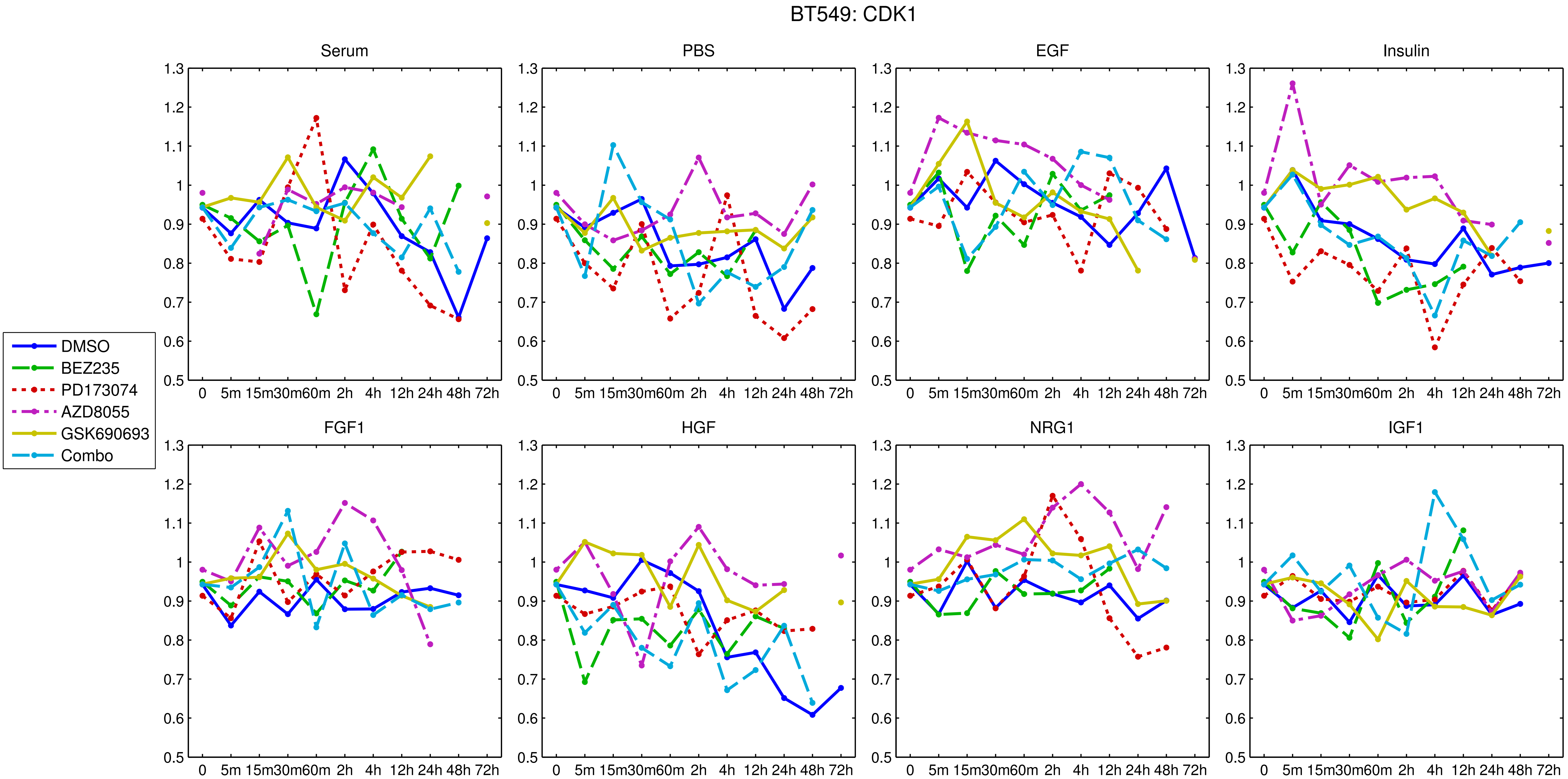
BT549: CDK1
Serum
PBS
EGF
Insulin
FGF1
HGF
NRG1
IGF1
DMSO
BEZ235
PD173074
AZD8055
GSK690693
Combo
0
5m
15m
30m
60m
2h
4h
12h
24h
48h
72h
0.5
0.6
0.7
0.8
0.9
1
1.1
1.2
1.3

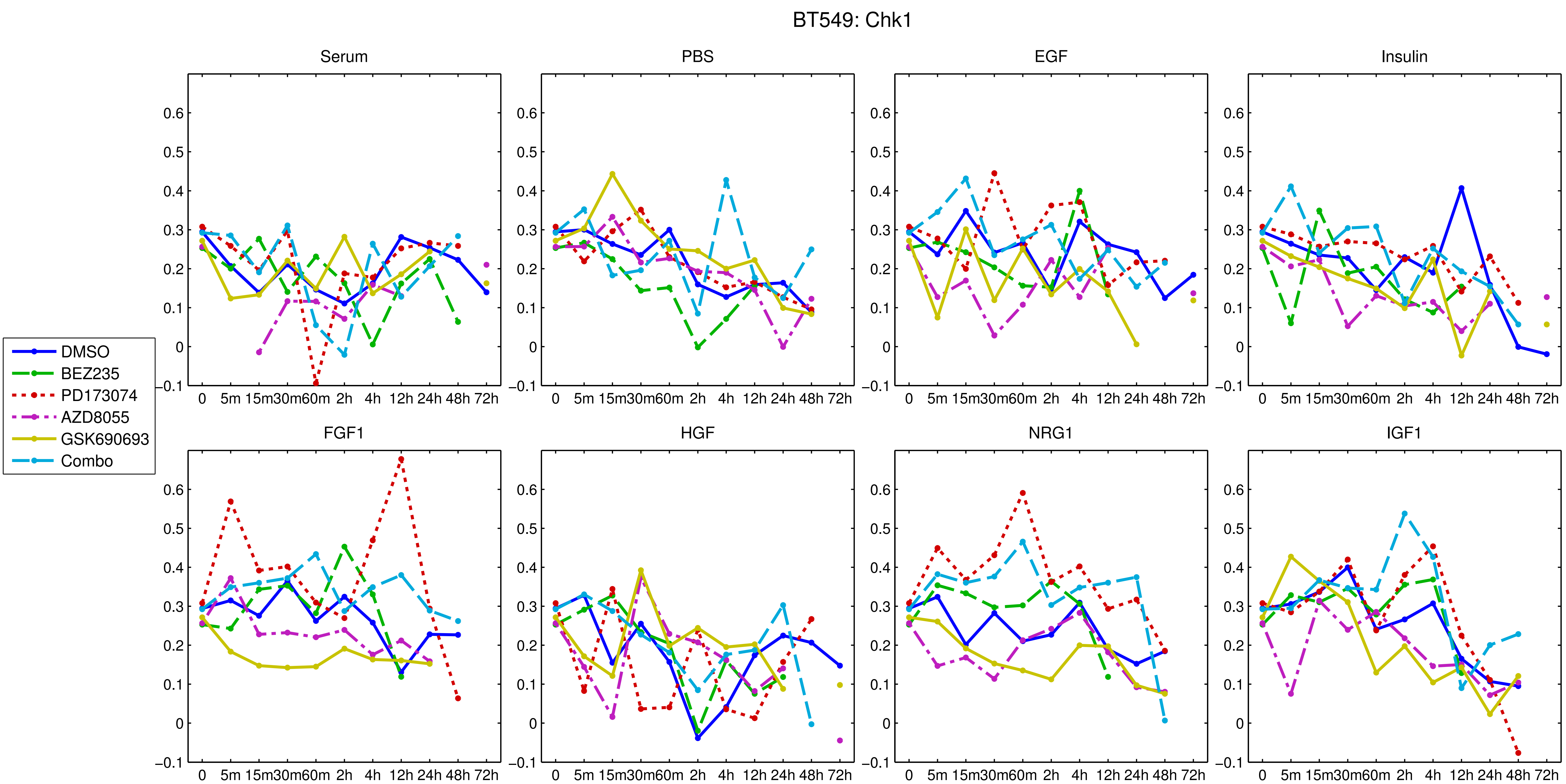
BT549: Chk1
Serum
PBS
EGF
Insulin
FGF1
HGF
NRG1
IGF1
DMSO
BEZ235
PD173074
AZD8055
GSK690693
Combo
0.6
0.5
0.4
0.3
0.2
0.1
0
−0.1
0 5m 15m30m60m 2h 4h 12h 24h 48h 72h

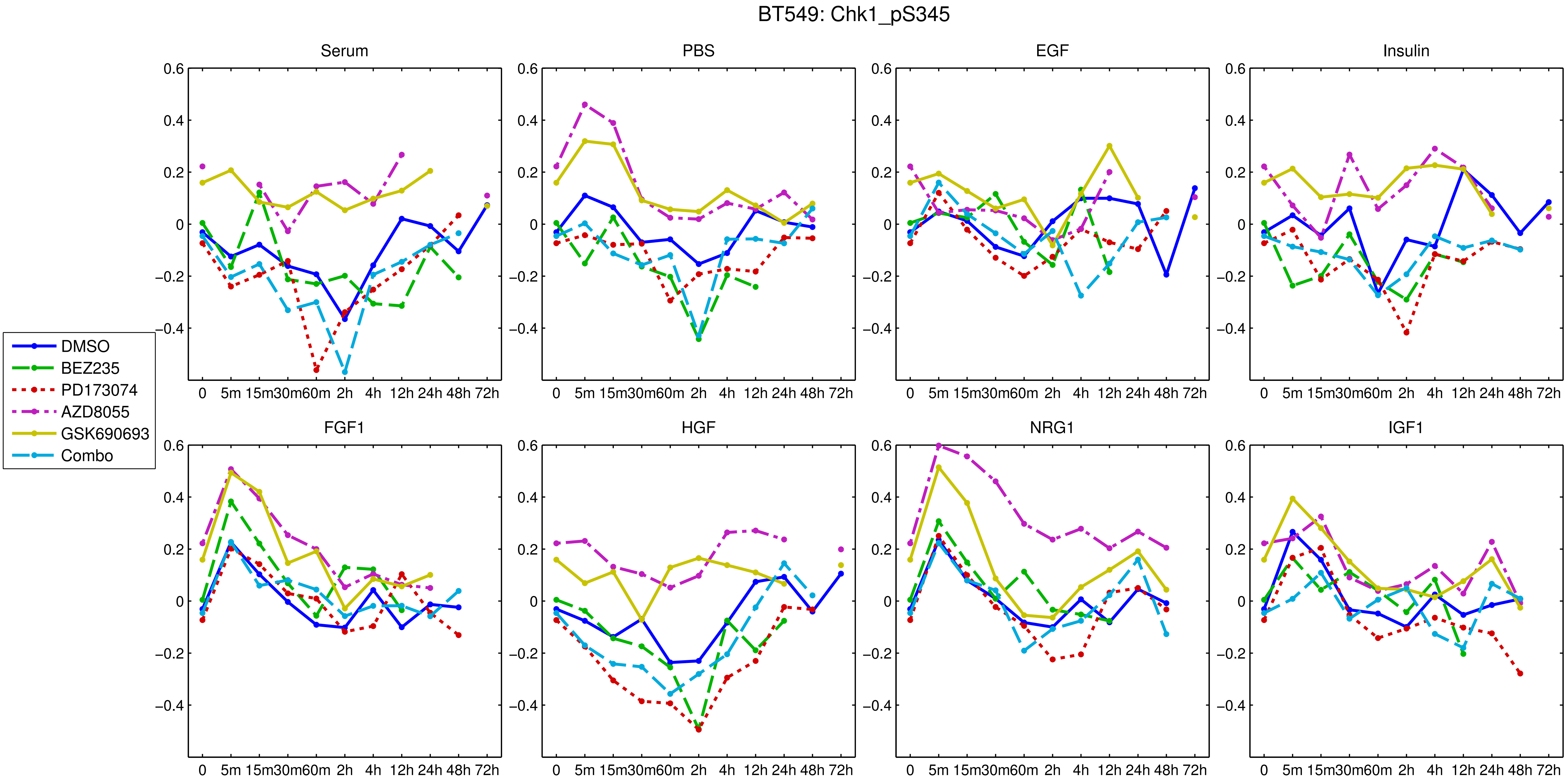

BT549: Chk1_pS345
Serum
PBS
EGF
Insulin
FGF1
HGF
NRG1
IGF1
DMSO
BEZ235
PD173074
AZD8055
GSK690693
Combo
0 5m 15m30m60m 2h 4h 12h 24h 48h 72h

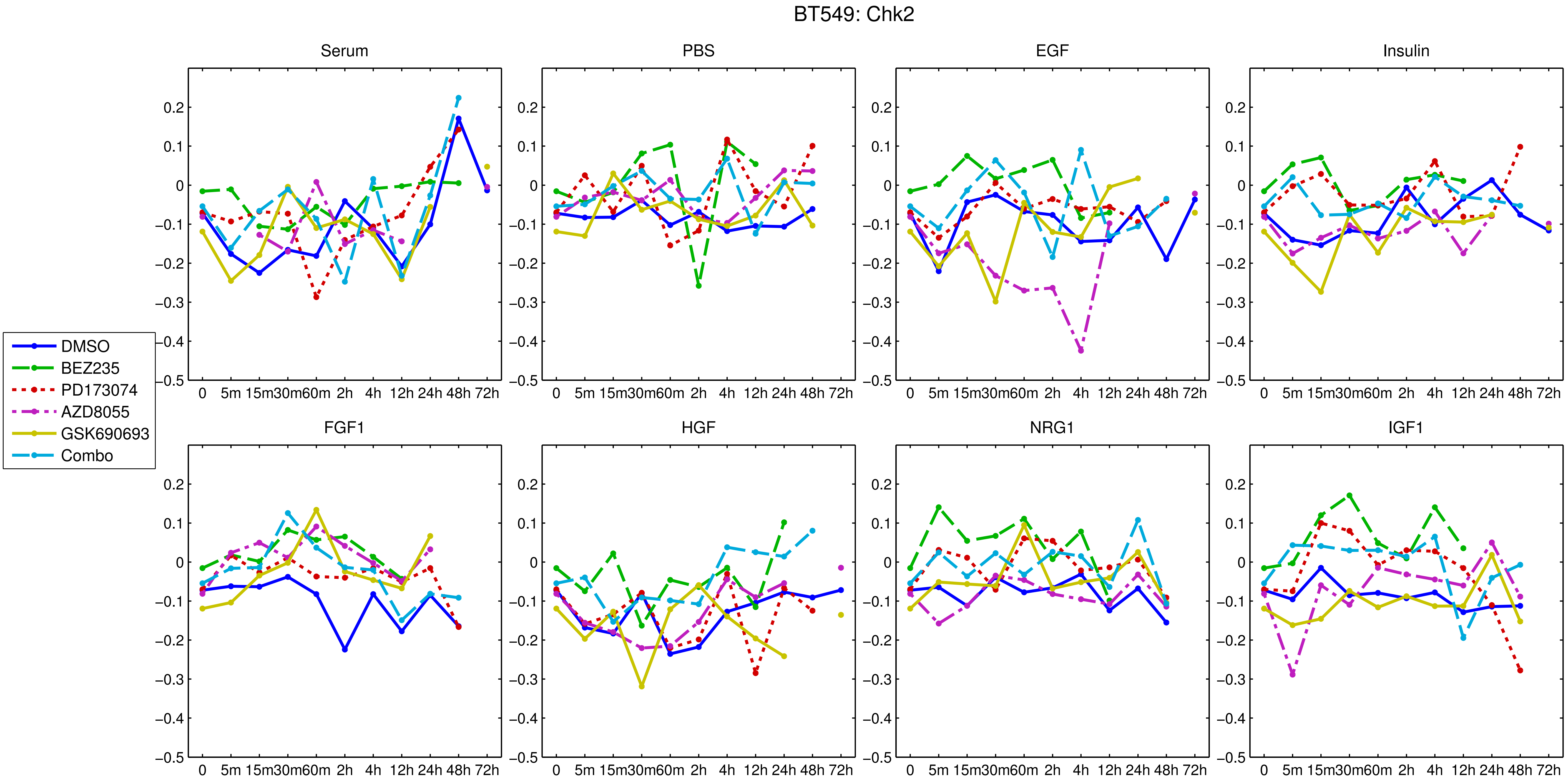
BT549: Chk2
Serum
PBS
EGF
Insulin
FGF1
HGF
NRG1
IGF1
0.2
0.1
0
−0.1
−0.2
−0.3
−0.4
−0.5
0
5m
15m
30m
60m
2h
4h
12h
24h
48h
72h
DMSO
BEZ235
PD173074
AZD8055
GSK690693
Combo

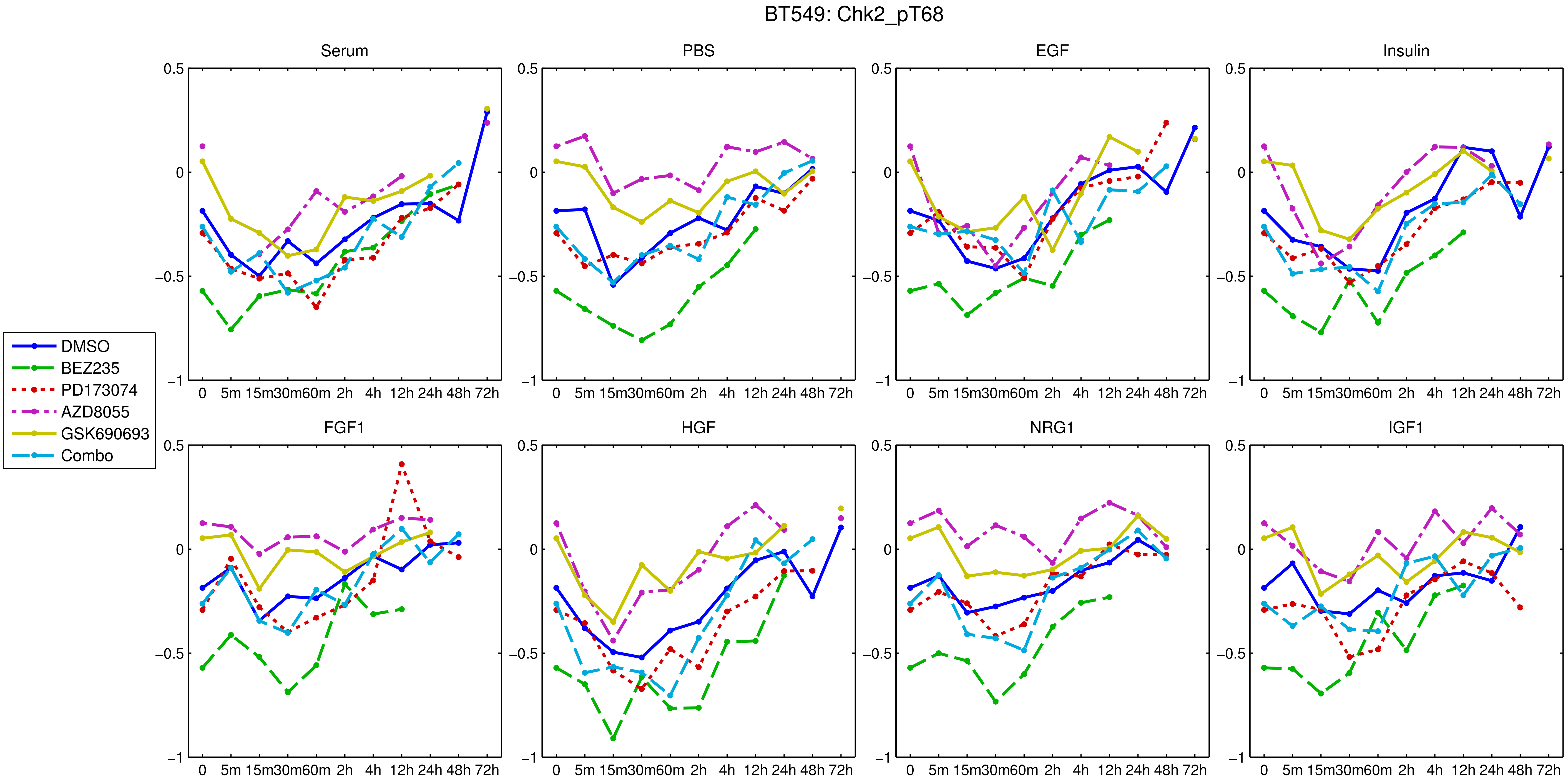

BT549: Chk2_pT68
Serum
PBS
EGF
Insulin
FGF1
HGF
NRG1
IGF1
0.5
0
−0.5
−1
0
5m
15m
30m
60m
2h
4h
12h
24h
48h
72h
DMSO
BEZ235
PD173074
AZD8055
GSK690693
Combo

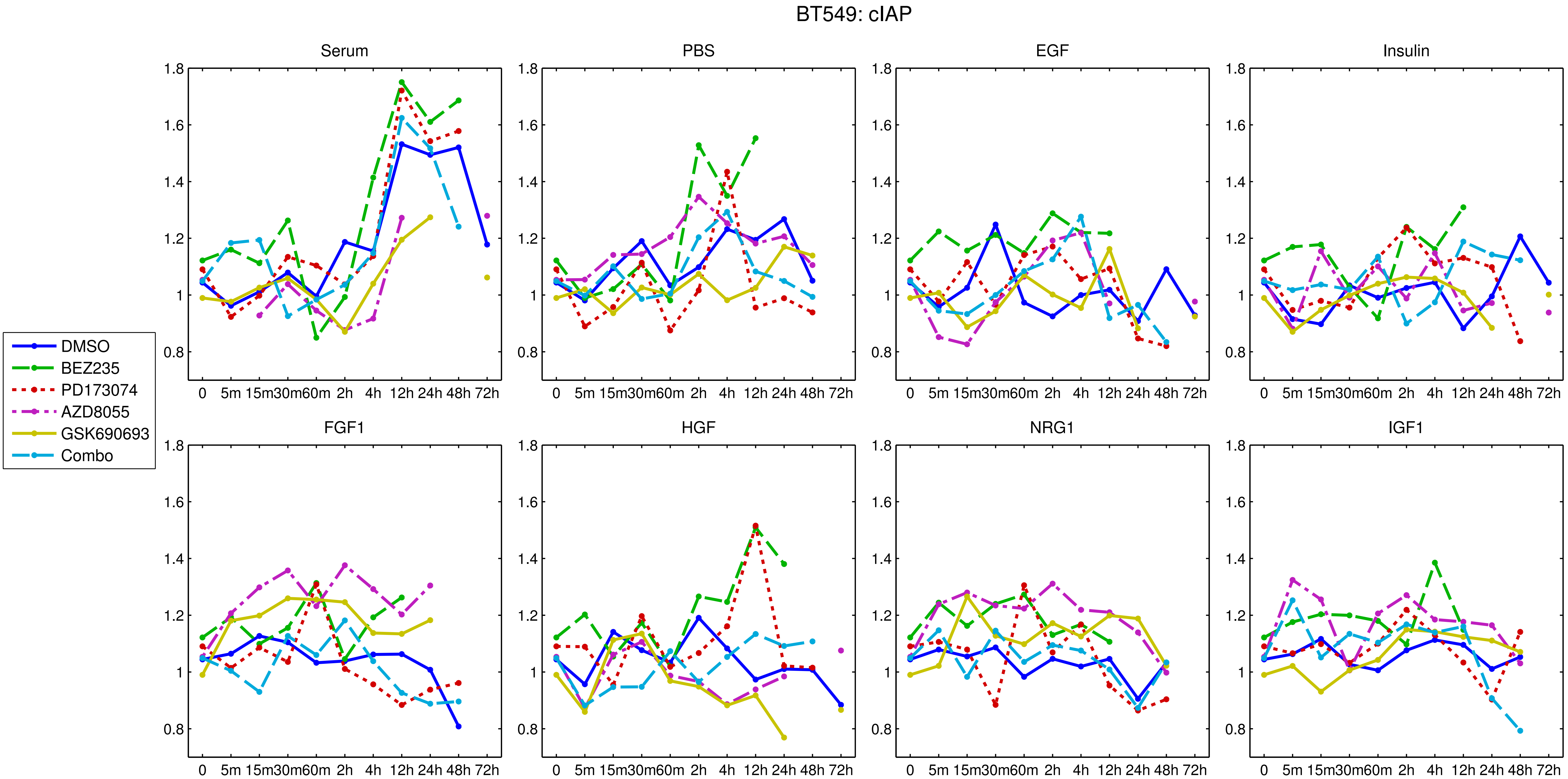
BT549: cIAP
Serum
PBS
EGF
Insulin
FGF1
HGF
NRG1
IGF1
DMSO
BEZ235
PD173074
AZD8055
GSK690693
Combo
1.8
1.6
1.4
1.2
1
0.8
0
5m
15m
30m
60m
2h
4h
12h
24h
48h
72h

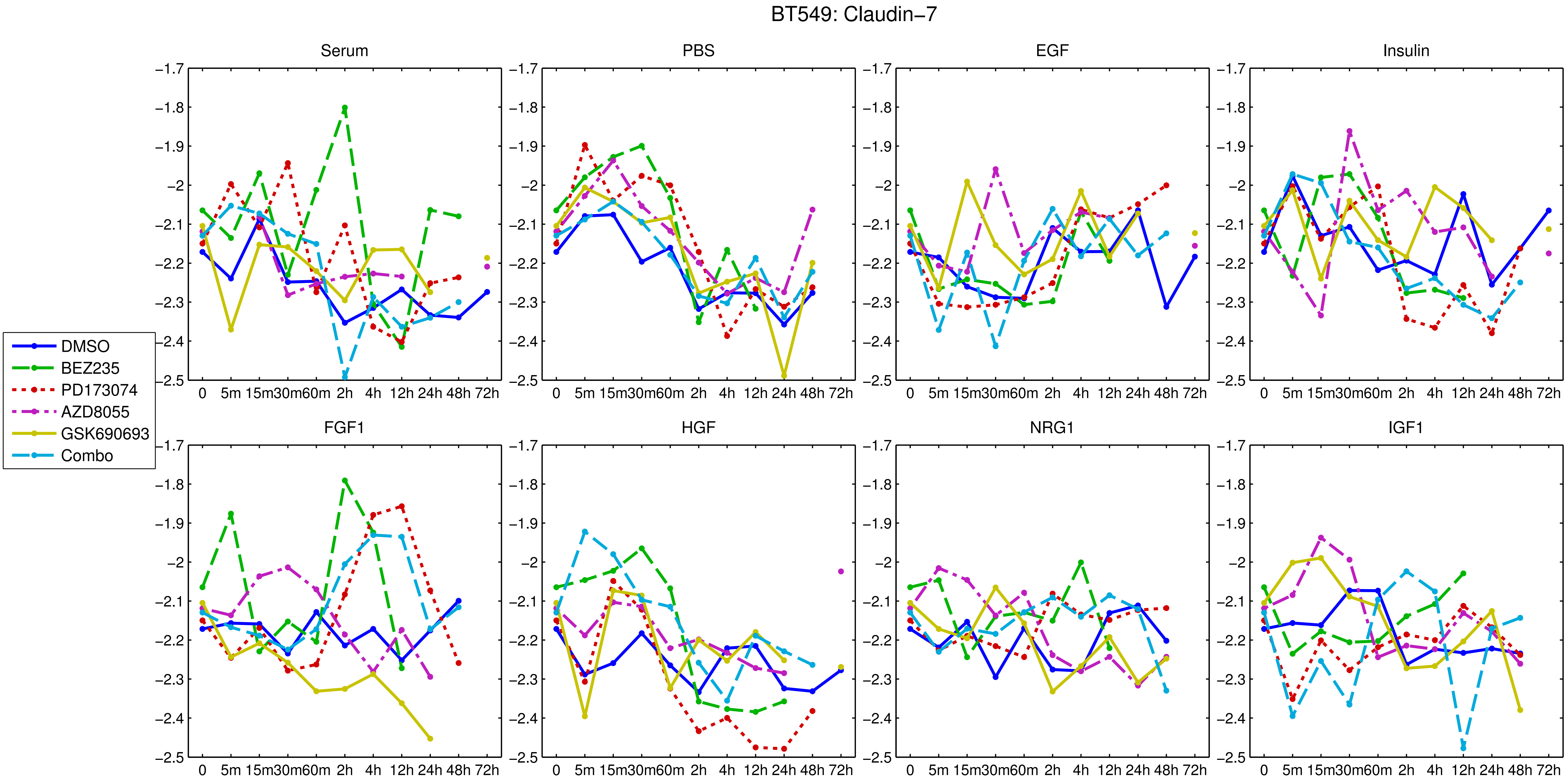

BT549: Claudin−7
Serum
PBS
EGF
Insulin
FGF1
HGF
NRG1
IGF1
−1.7
−1.8
−1.9
−2
−2.1
−2.2
−2.3
−2.4
−2.5
0 5m 15m 30m 60m 2h 4h 12h 24h 48h 72h
DMSO
BEZ235
PD173074
AZD8055
GSK690693
Combo

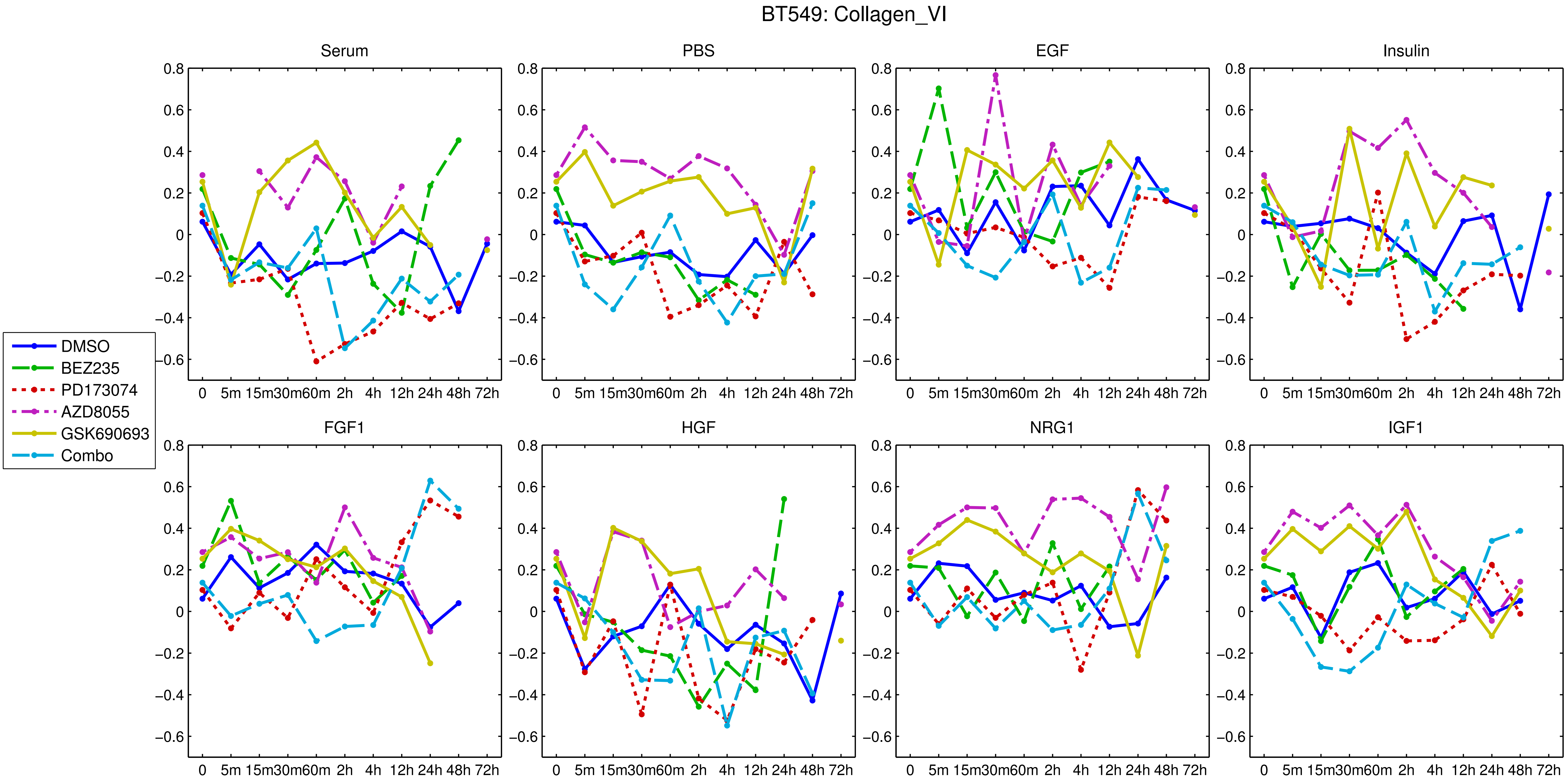

BT549: Collagen_VI
Serum
PBS
EGF
Insulin
FGF1
HGF
NRG1
IGF1
DMSO
BEZ235
PD173074
AZD8055
GSK690693
Combo
0.8
0.6
0.4
0.2
0
−0.2
−0.4
−0.6
0 5m 15m 30m 60m 2h 4h 12h 24h 48h 72h

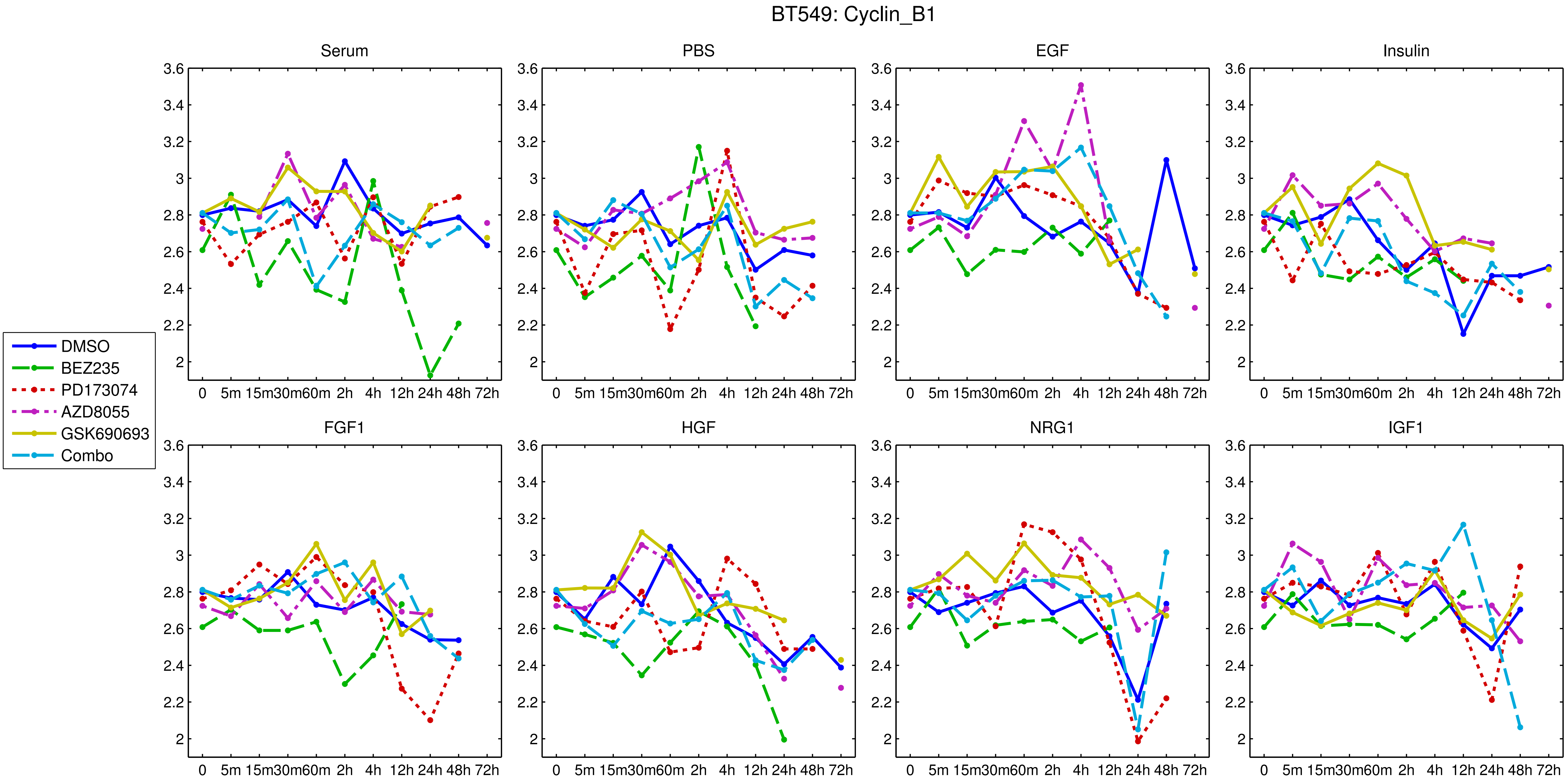
BT549: Cyclin_B1
Serum
PBS
EGF
Insulin
FGF1
HGF
NRG1
IGF1
3.6
3.4
3.2
3
2.8
2.6
2.4
2.2
2
0
5m
15m
30m
60m
2h
4h
12h
24h
48h
72h
DMSO
BEZ235
PD173074
AZD8055
GSK690693
Combo

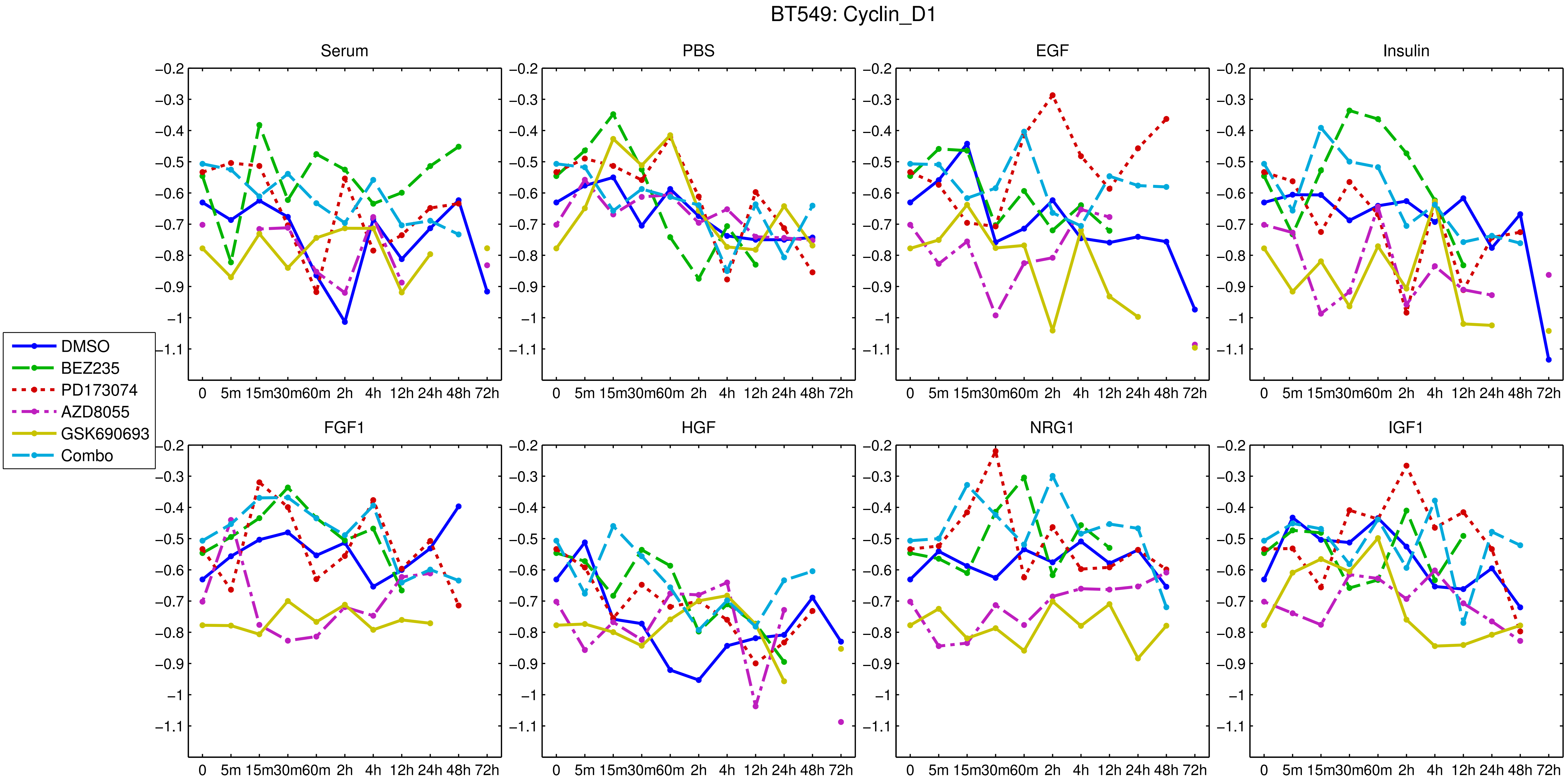
BT549: Cyclin_D1
Serum
PBS
EGF
Insulin
FGF1
HGF
NRG1
IGF1
DMSO
BEZ235
PD173074
AZD8055
GSK690693
Combo
−0.2
−0.3
−0.4
−0.5
−0.6
−0.7
−0.8
−0.9
−1
−1.1
0
5m
15m
30m
60m
2h
4h
12h
24h
48h
72h

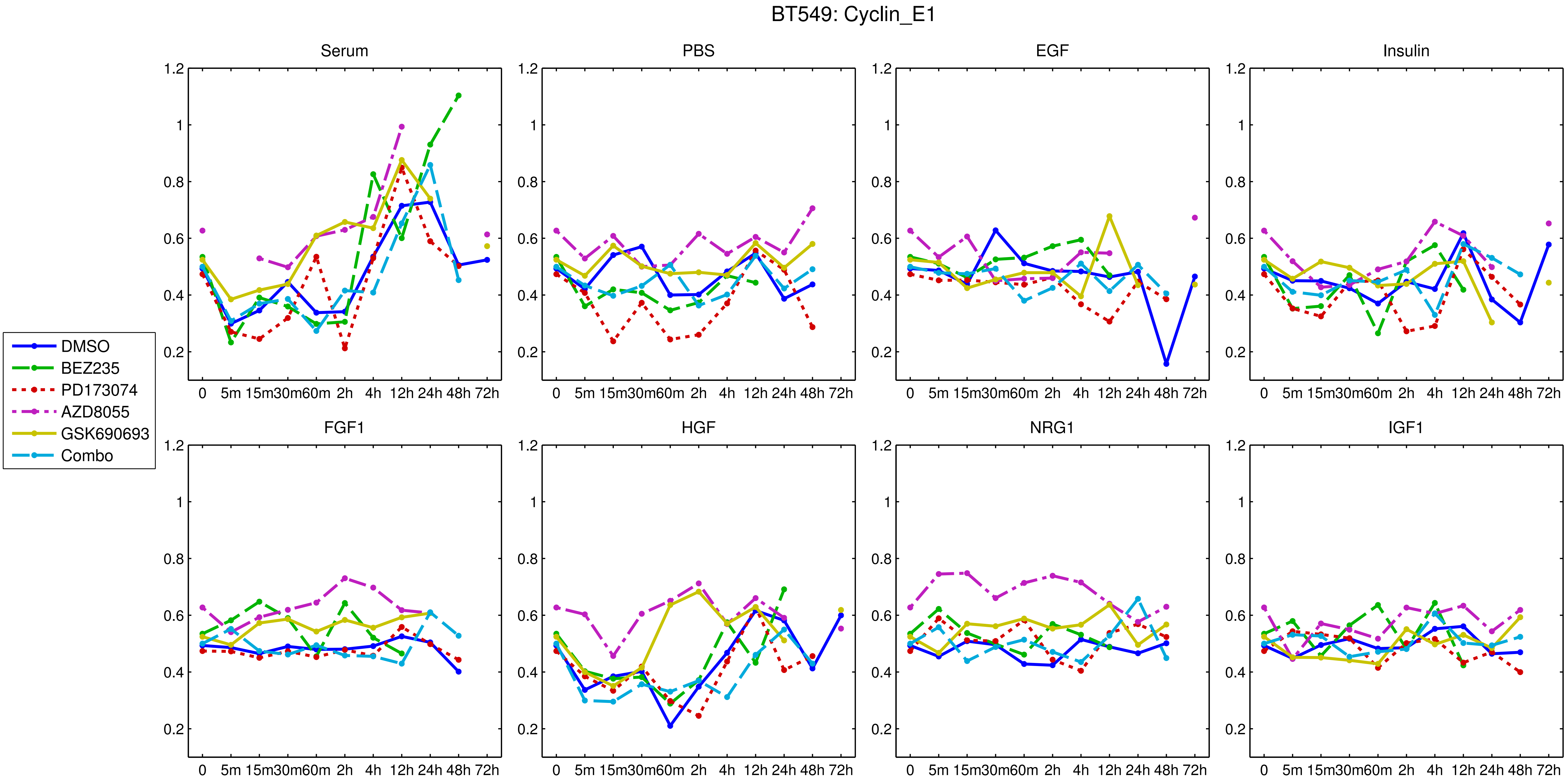
BT549: Cyclin_E1
Serum
PBS
EGF
Insulin
FGF1
HGF
NRG1
IGF1
DMSO
BEZ235
PD173074
AZD8055
GSK690693
Combo
0 5m 15m30m60m 2h 4h 12h 24h 48h 72h
1.2
1
0.8
0.6
0.4
0.2

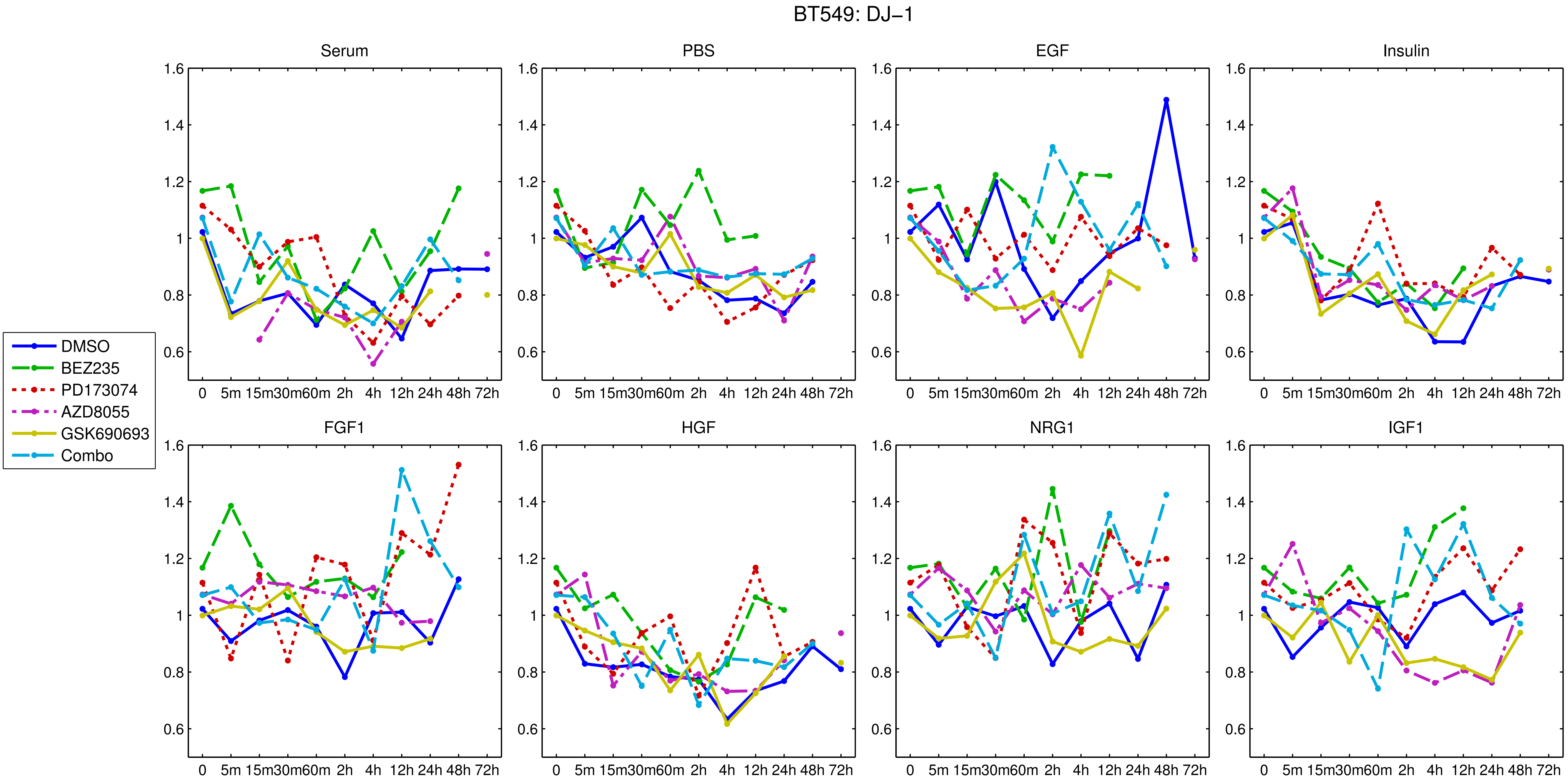
BT549: DJ−1
Serum
PBS
EGF
Insulin
FGF1
HGF
NRG1
IGF1
DMSO
BEZ235
PD173074
AZD8055
GSK690693
Combo
1.6
1.4
1.2
1
0.8
0.6
0
5m
15m
30m
60m
2h
4h
12h
24h
48h
72h

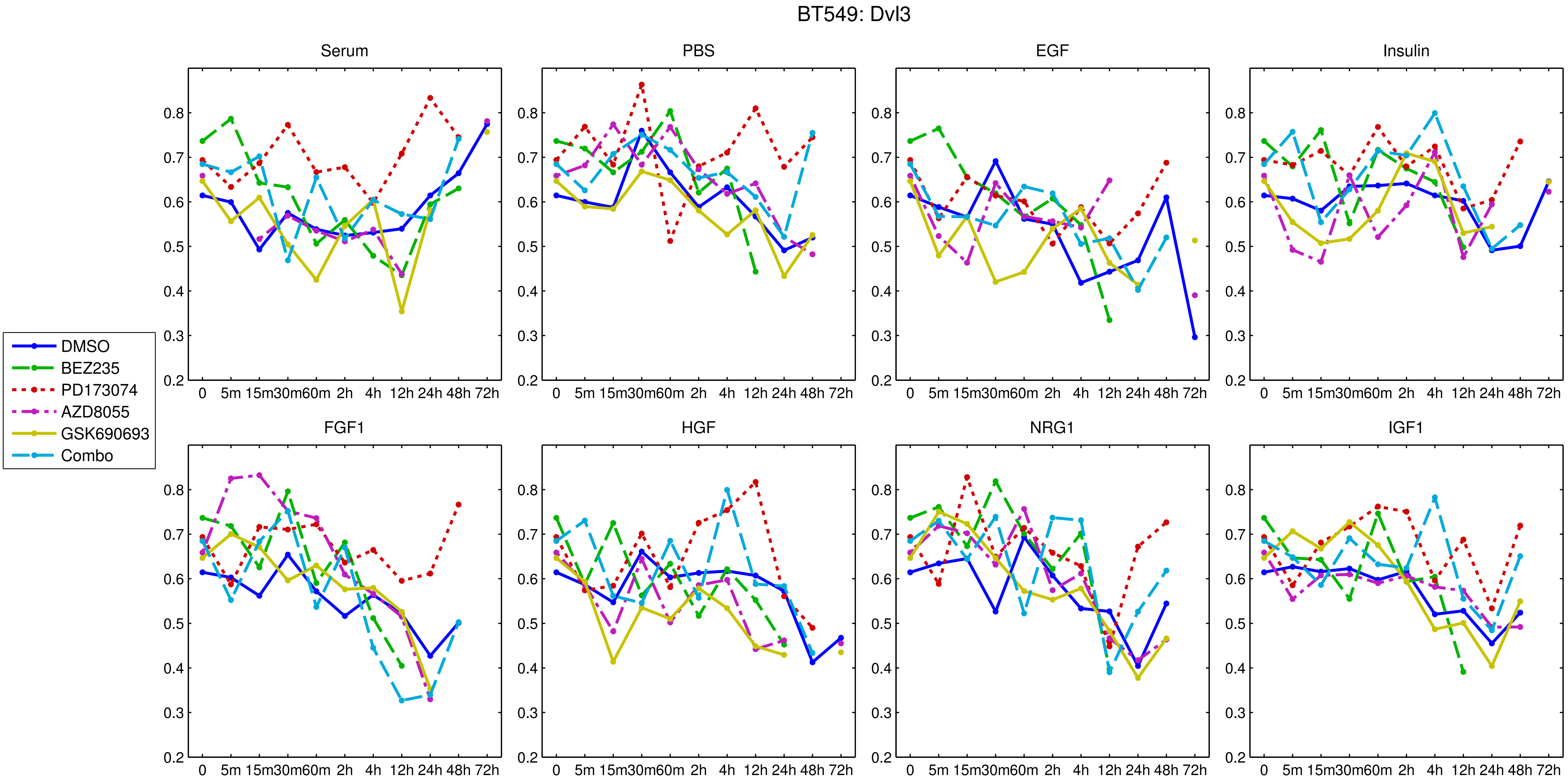
BT549: Dvl3
Serum
PBS
EGF
Insulin
FGF1
HGF
NRG1
IGF1
DMSO
BEZ235
PD173074
AZD8055
GSK690693
Combo
0.2
0.3
0.4
0.5
0.6
0.7
0.8
0 5m 15m 30m 60m 2h 4h 12h 24h 48h 72h

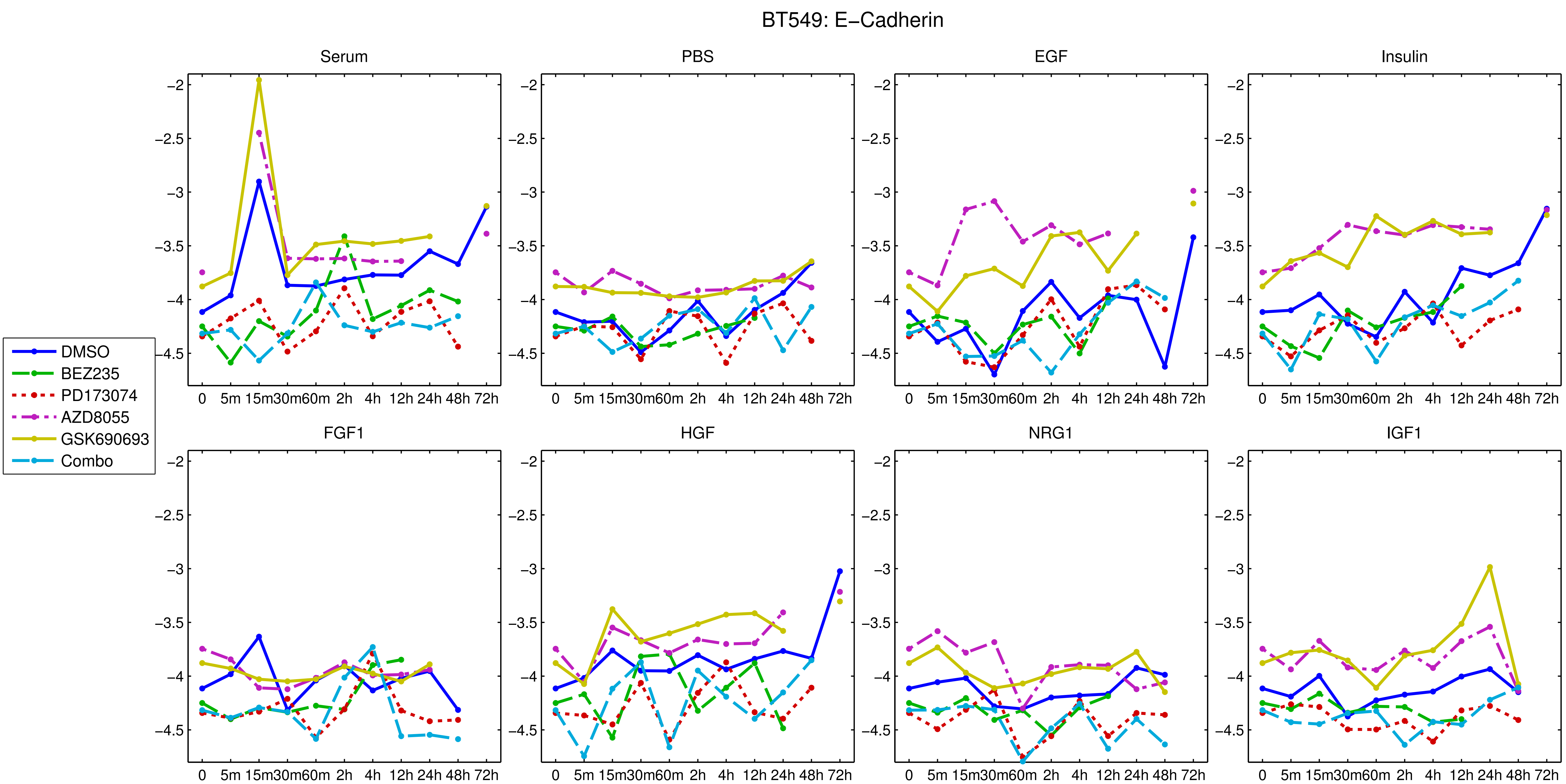
BT549: E−Cadherin
Serum
PBS
EGF
Insulin
FGF1
HGF
NRG1
IGF1
DMSO
BEZ235
PD173074
AZD8055
GSK690693
Combo
0 5m 15m 30m 60m 2h 4h 12h 24h 48h 72h

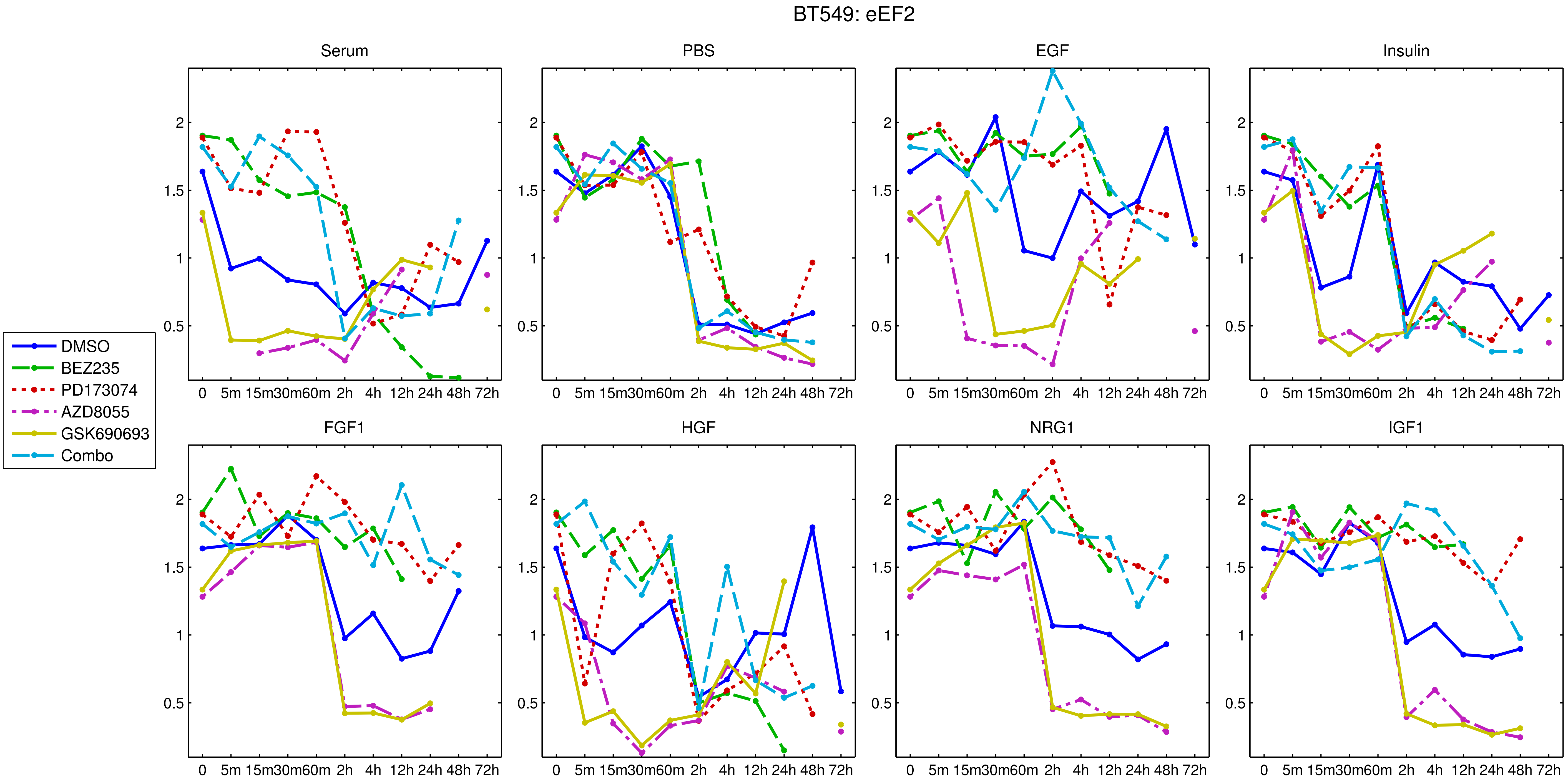

BT549: eEF2
Serum
PBS
EGF
Insulin
FGF1
HGF
NRG1
IGF1
DMSO
BEZ235
PD173074
AZD8055
GSK690693
Combo
0
0.5
1
1.5
2
0 5m 15m30m60m 2h 4h 12h 24h 48h 72h

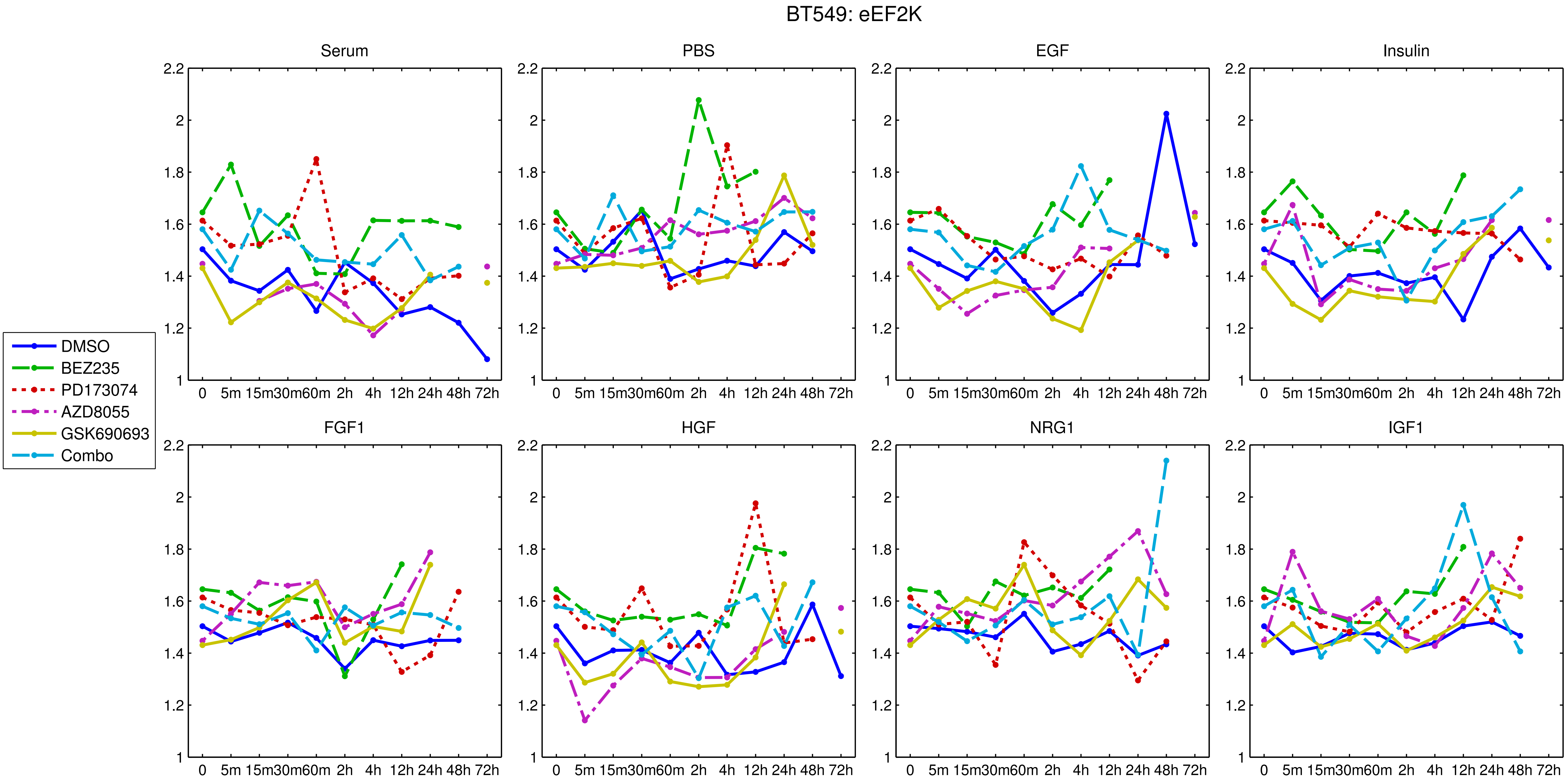

BT549: eEF2K
Serum
PBS
EGF
Insulin
FGF1
HGF
NRG1
IGF1
DMSO
BEZ235
PD173074
AZD8055
GSK690693
Combo
0
5m
15m
30m
60m
2h
4h
12h
24h
48h
72h
1
1.2
1.4
1.6
1.8
2
2.2

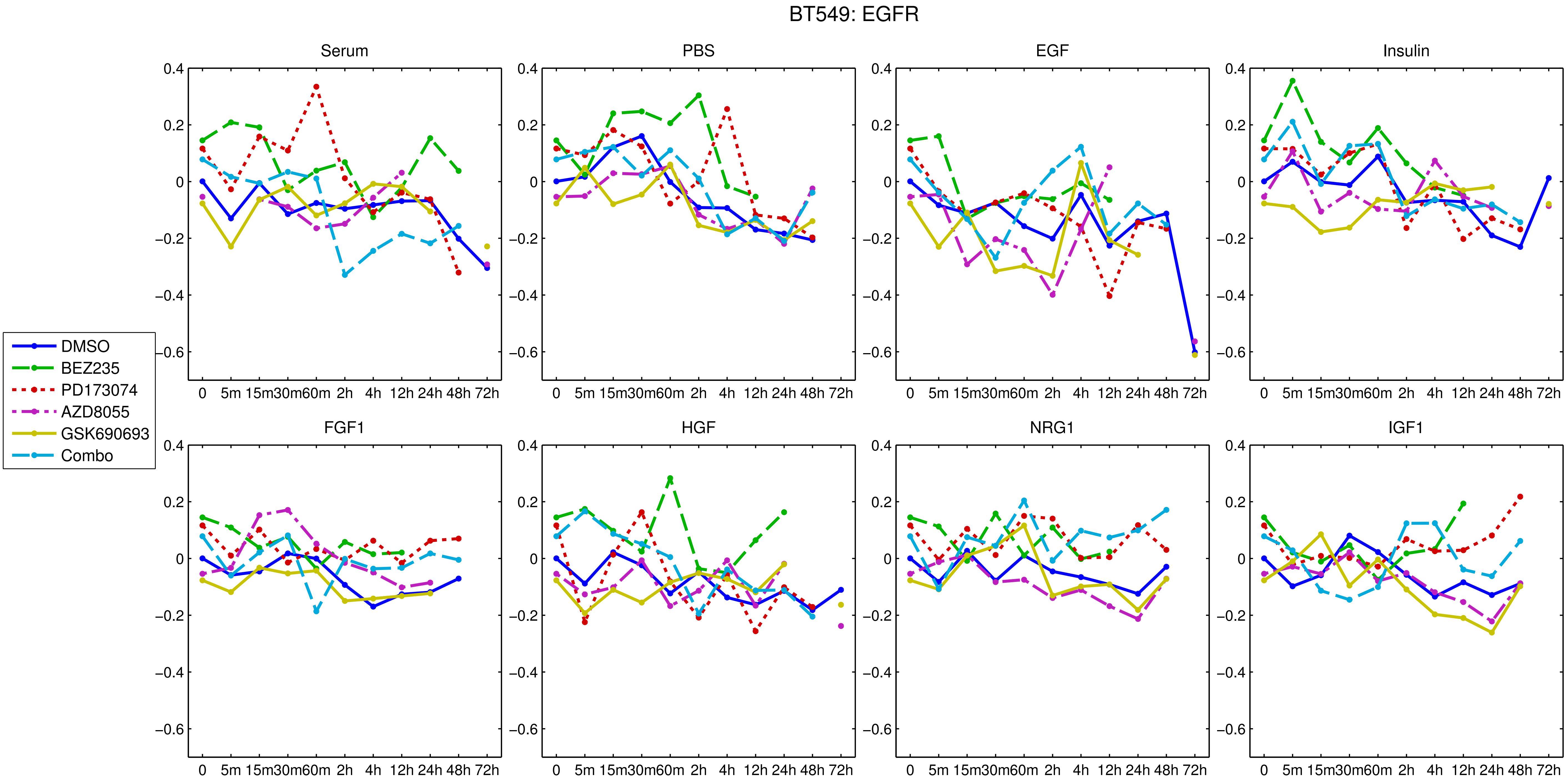
BT549: EGFR
Serum
PBS
EGF
Insulin
FGF1
HGF
NRG1
IGF1
0.4
0.2
0
−0.2
−0.4
−0.6
0
5m
15m
30m
60m
2h
4h
12h
24h
48h
72h
DMSO
BEZ235
PD173074
AZD8055
GSK690693
Combo

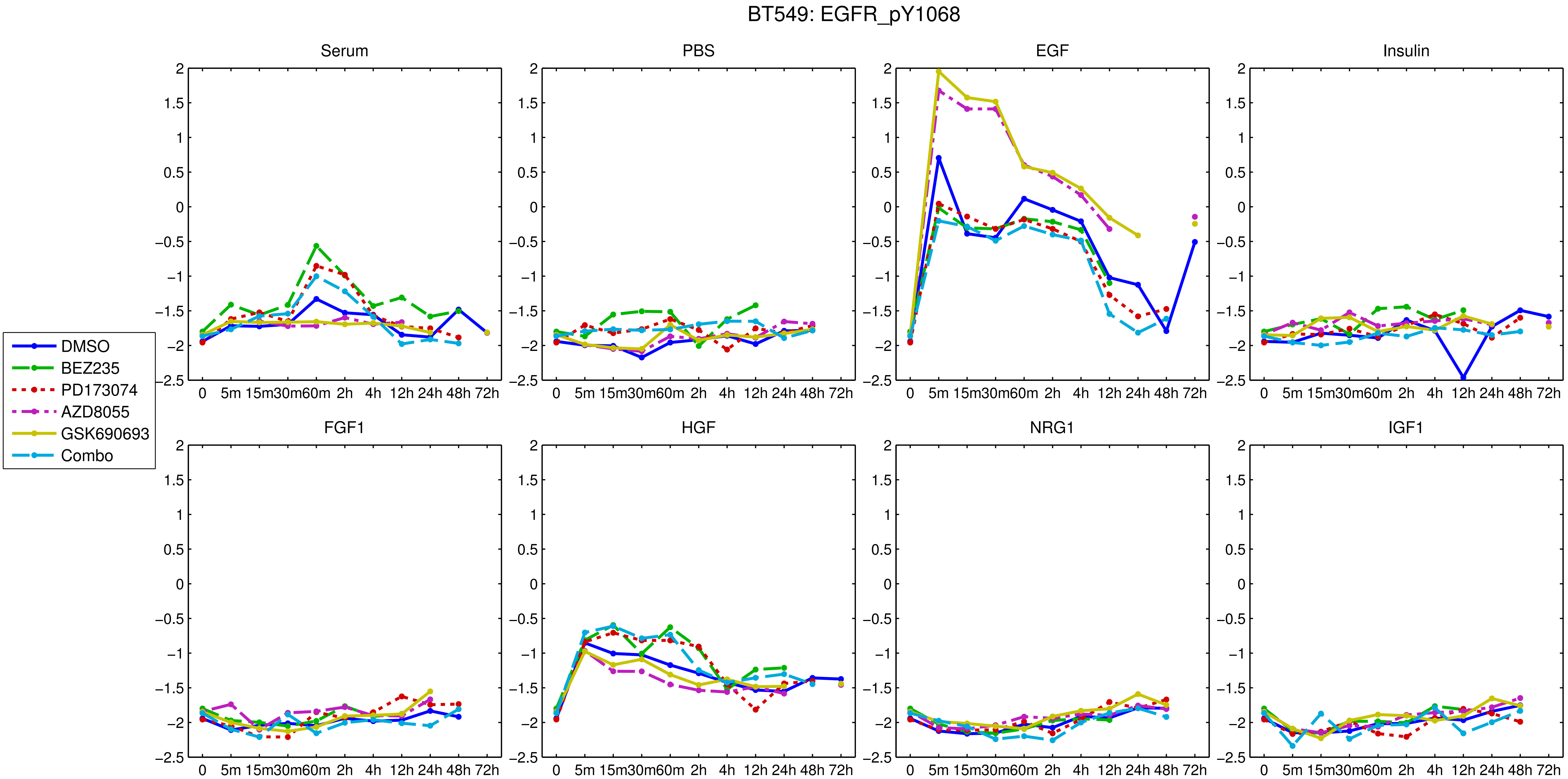
BT549: EGFR_pY1068
Serum
PBS
EGF
Insulin
FGF1
HGF
NRG1
IGF1
DMSO
BEZ235
PD173074
AZD8055
GSK690693
Combo
0
5m
15m
30m
60m
2h
4h
12h
24h
48h
72h
2
1.5
1
0.5
0
−0.5
−1
−1.5
−2
−2.5

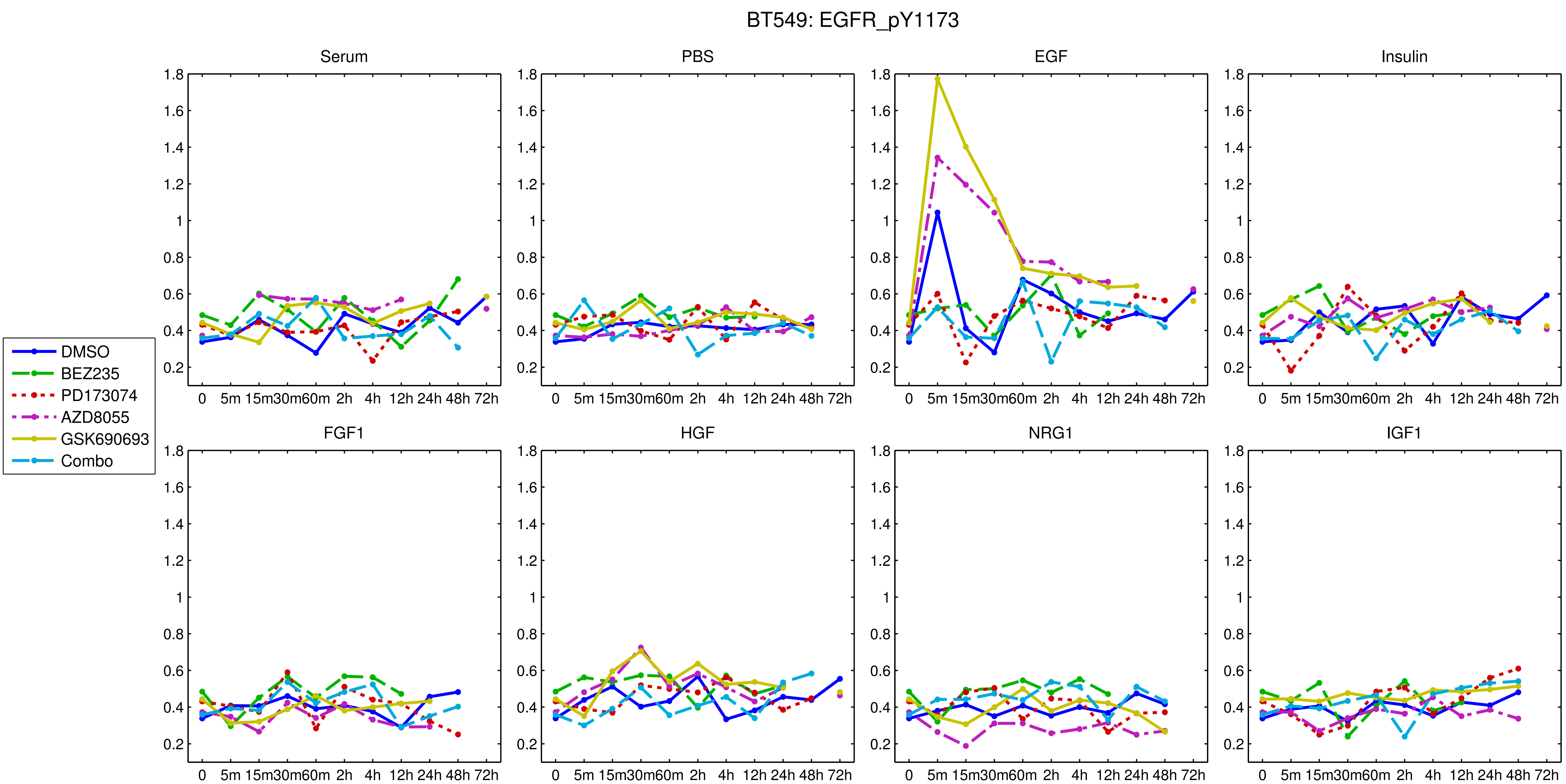

BT549: EGFR_pY1173
Serum
PBS
EGF
Insulin
FGF1
HGF
NRG1
IGF1
DMSO
BEZ235
PD173074
AZD8055
GSK690693
Combo
0.2 0.4 0.6 0.8 1 1.2 1.4 1.6 1.8
0 5m 15m 30m 60m 2h 4h 12h 24h 48h 72h

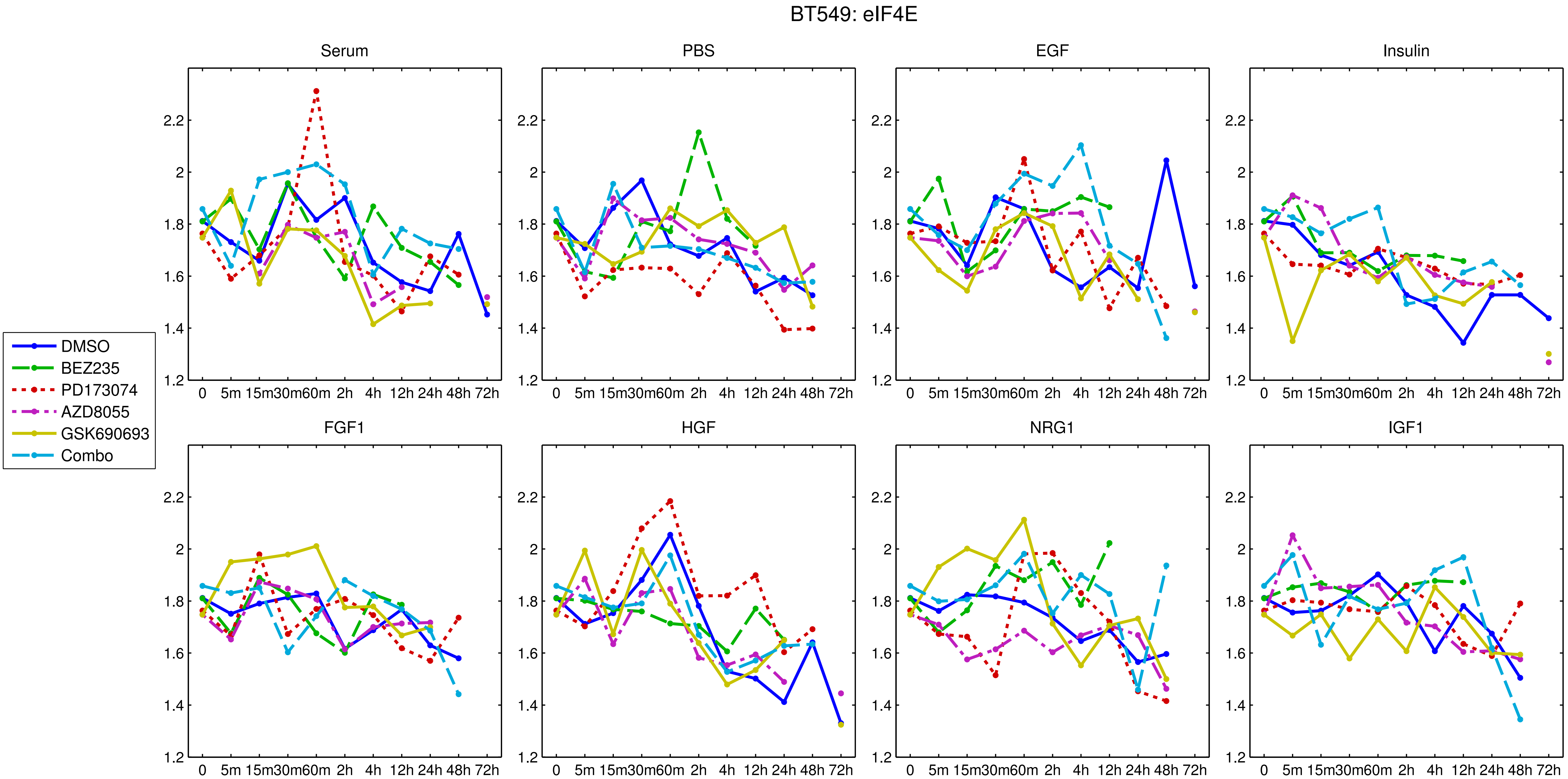
BT549: eIF4E
Serum
PBS
EGF
Insulin
FGF1
HGF
NRG1
IGF1
DMSO
BEZ235
PD173074
AZD8055
GSK690693
Combo
1.2
1.4
1.6
1.8
2
2.2
0
5m
15m
30m
60m
2h
4h
12h
24h
48h
72h

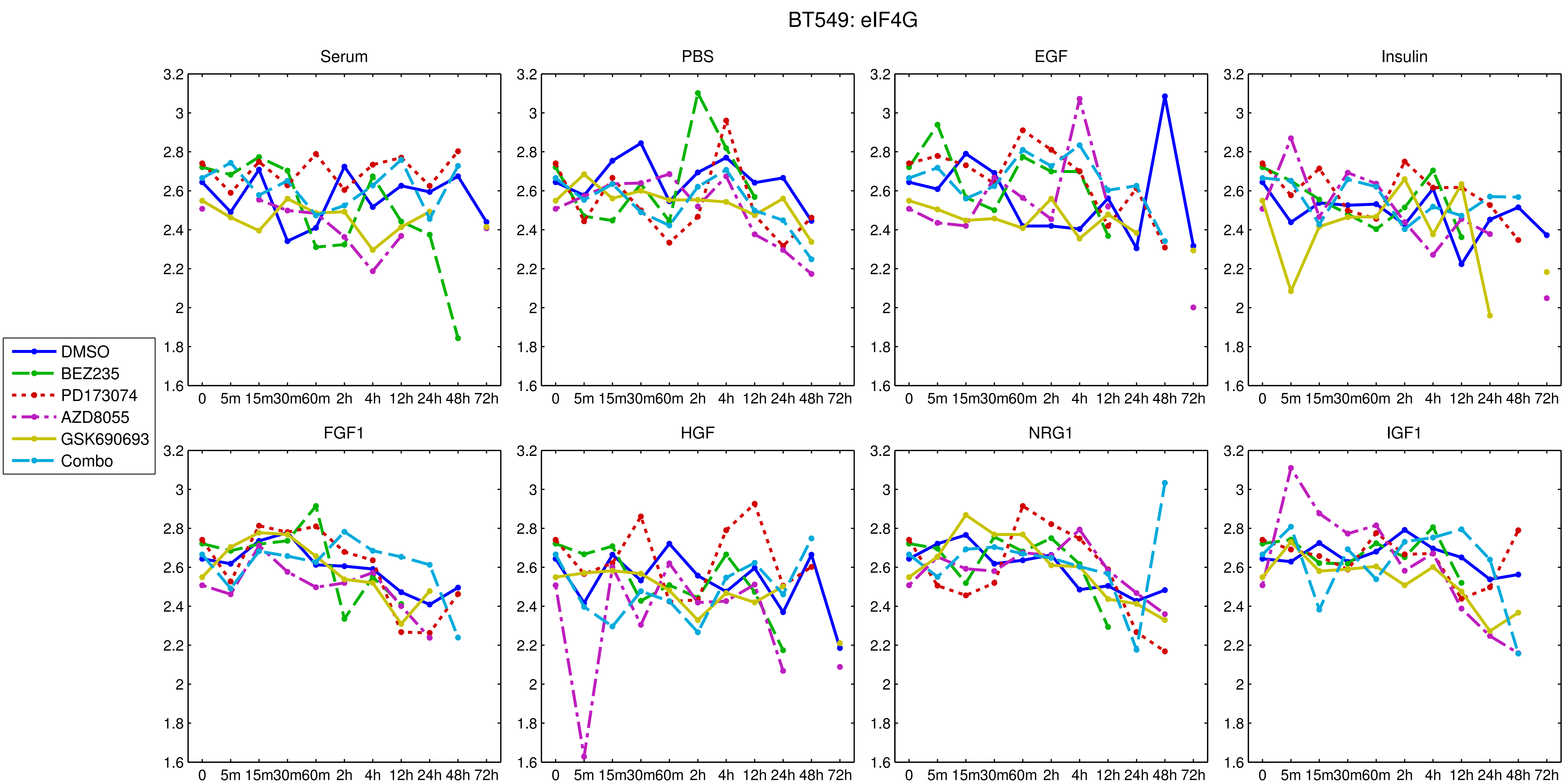
BT549: eIF4G
Serum
PBS
EGF
Insulin
FGF1
HGF
NRG1
IGF1
3.2
3
2.8
2.6
2.4
2.2
2
1.8
1.6
0
5m
15m
30m
60m
2h
4h
12h
24h
48h
72h
DMSO
BEZ235
PD173074
AZD8055
GSK690693
Combo

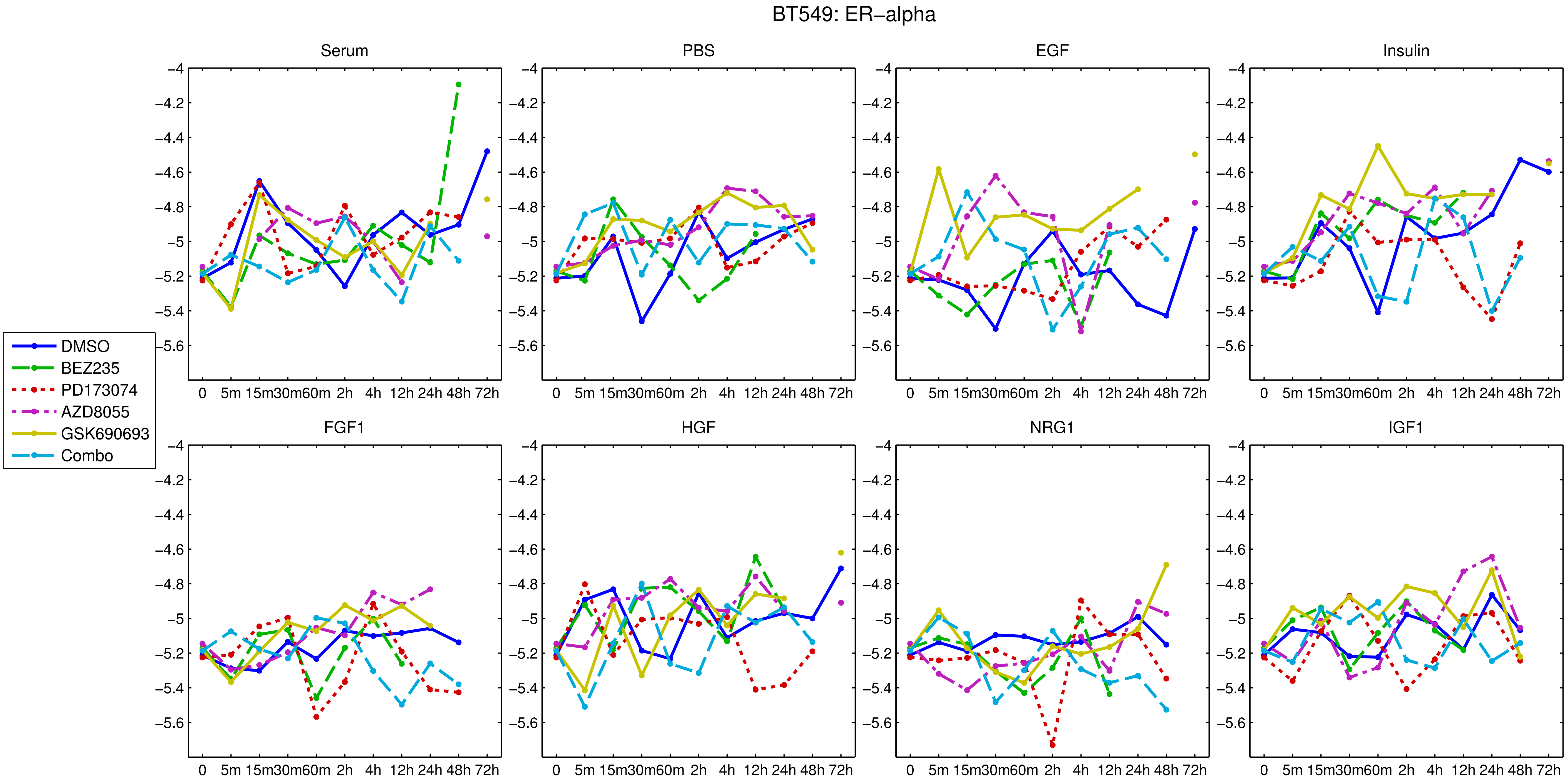

BT549: ER−alpha
Serum
PBS
EGF
Insulin
FGF1
HGF
NRG1
IGF1
DMSO
BEZ235
PD173074
AZD8055
GSK690693
Combo
0 5m 15m 30m 60m 2h 4h 12h 24h 48h 72h
−4
−4.2
−4.4
−4.6
−4.8
−5
−5.2
−5.4
−5.6

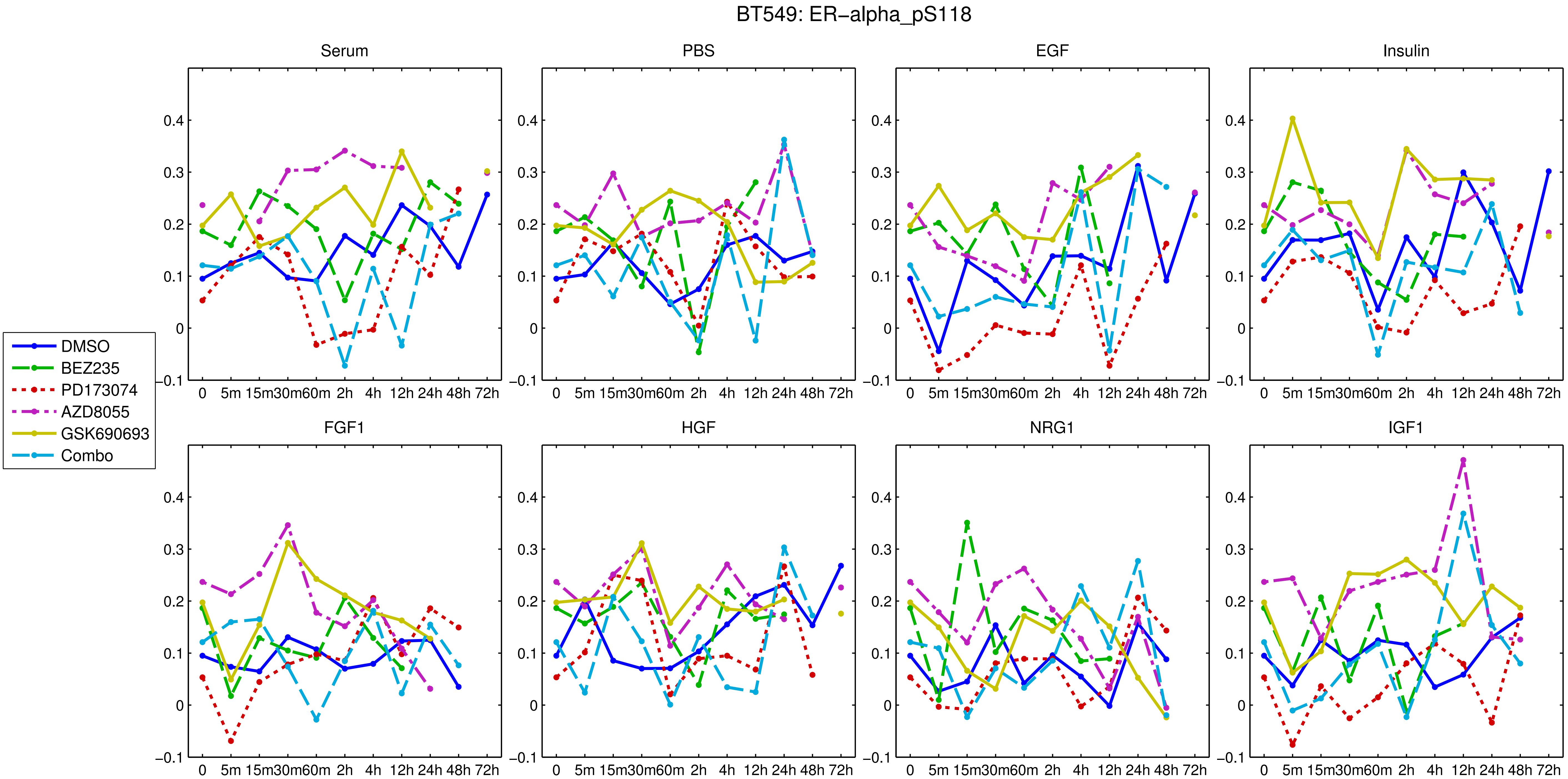
BT549: ER−alpha_pS118
Serum
PBS
EGF
Insulin
FGF1
HGF
NRG1
IGF1
DMSO
BEZ235
PD173074
AZD8055
GSK690693
Combo
0.4
0.3
0.2
0.1
0
−0.1
0 5m 15m 30m 60m 2h 4h 12h 24h 48h 72h

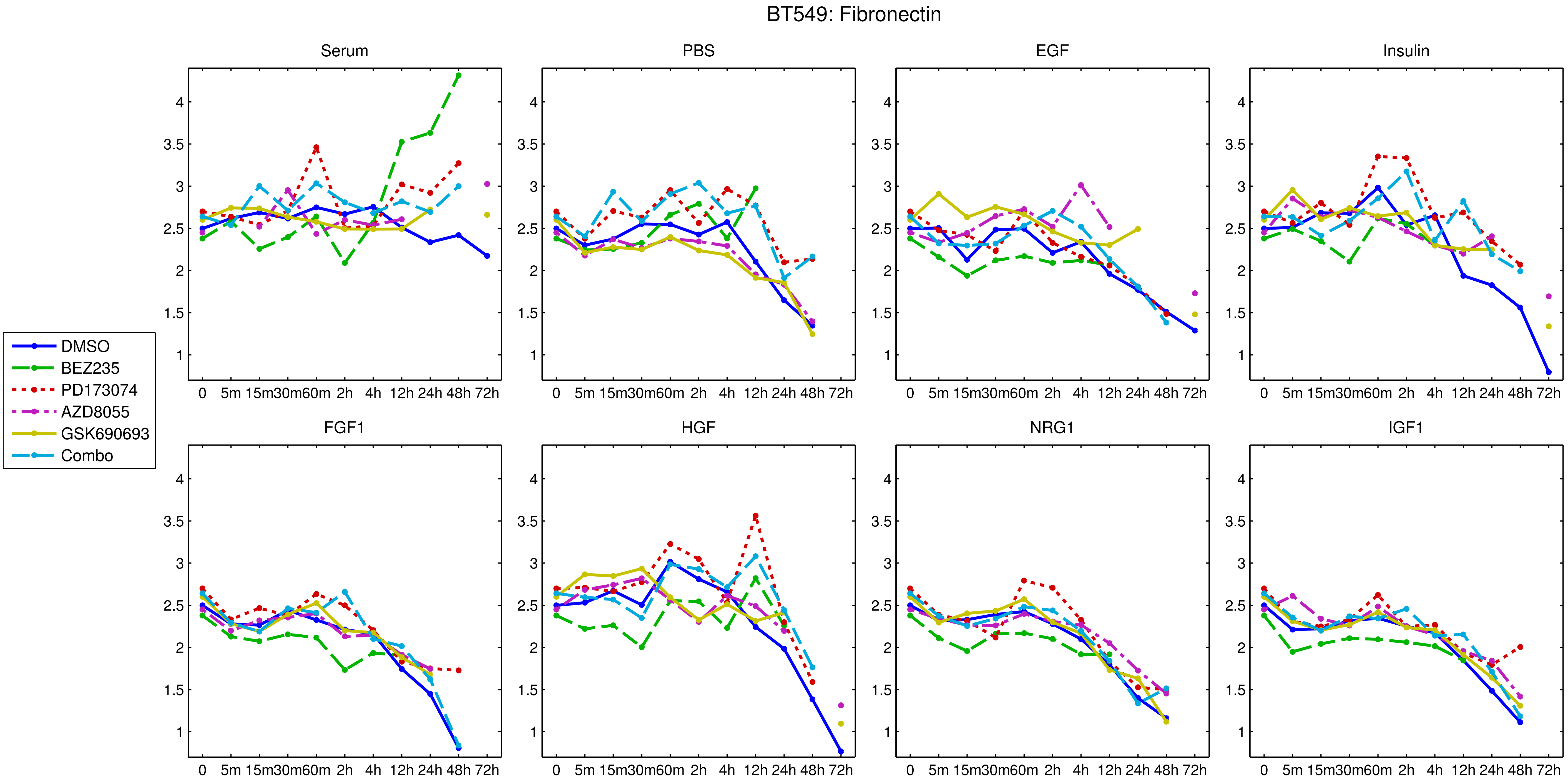
BT549: Fibronectin
Serum
PBS
EGF
Insulin
FGF1
HGF
NRG1
IGF1
DMSO
BEZ235
PD173074
AZD8055
GSK690693
Combo
0
5m
15m
30m
60m
2h
4h
12h
24h
48h
72h
1
1.5
2
2.5
3
3.5
4

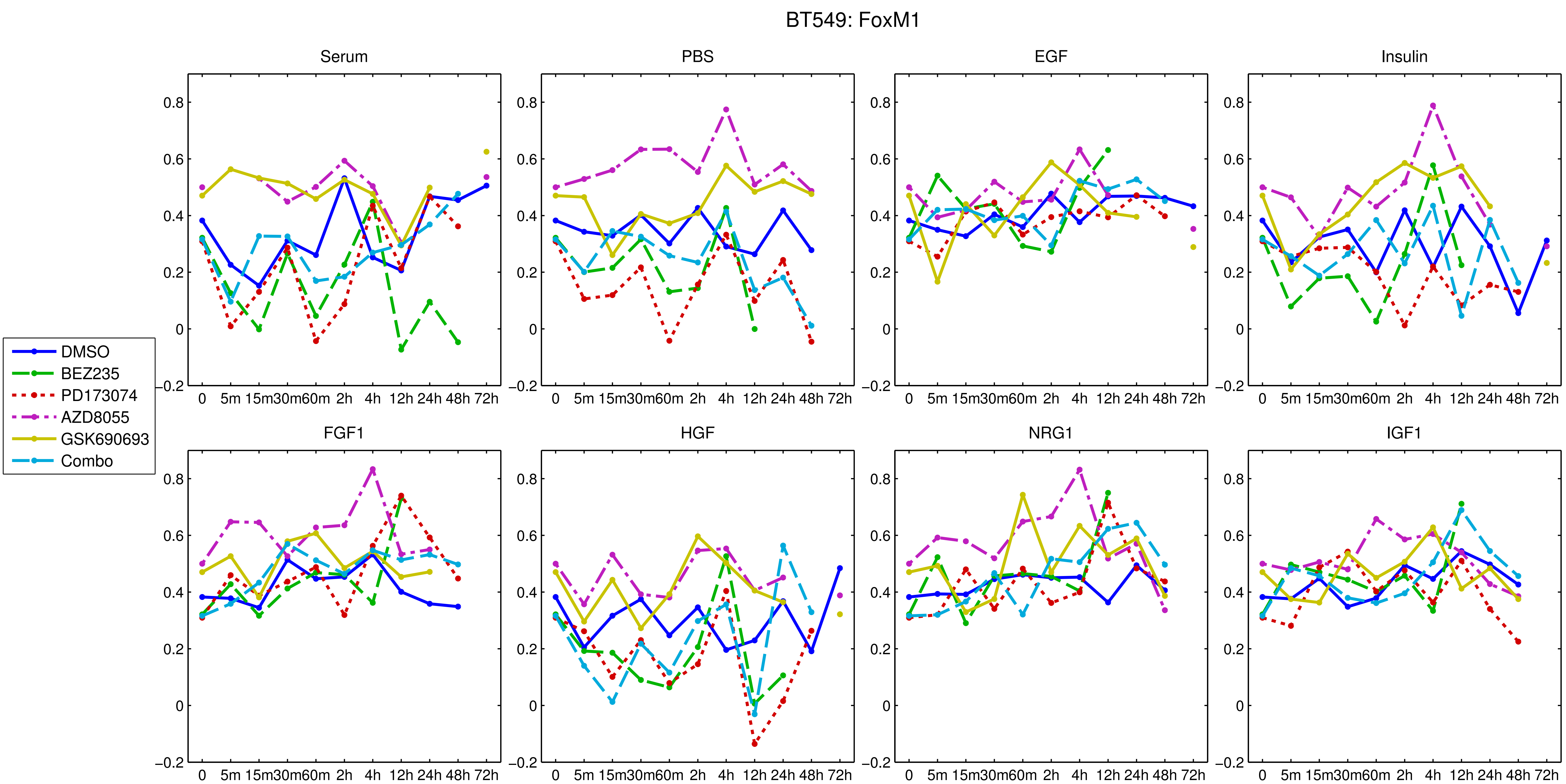
BT549: FoxM1
Serum
PBS
EGF
Insulin
FGF1
HGF
NRG1
IGF1
DMSO
BEZ235
PD173074
AZD8055
GSK690693
Combo
0.8
0.6
0.4
0.2
0
−0.2
0
5m
15m
30m
60m
2h
4h
12h
24h
48h
72h

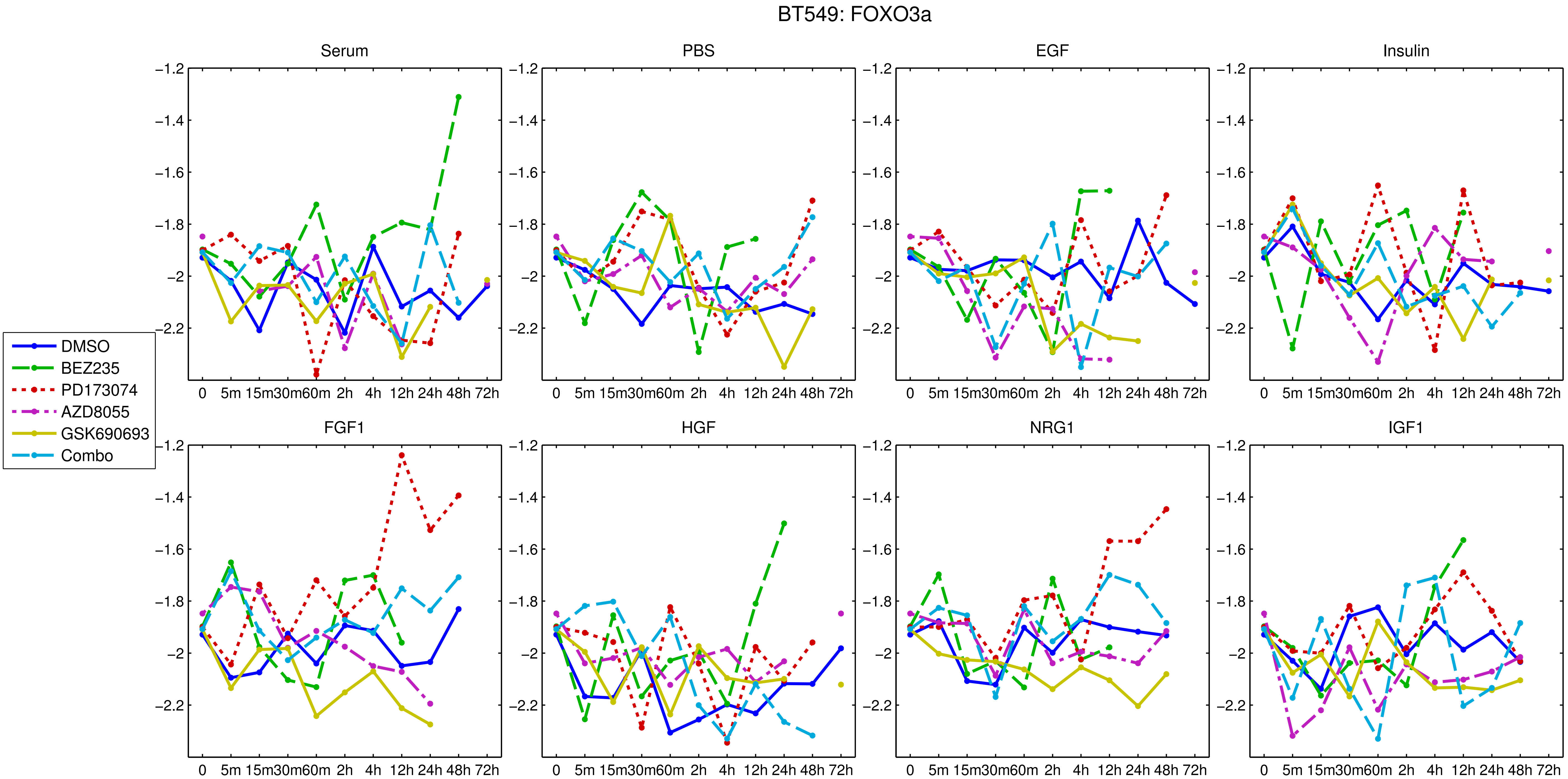
BT549: FOXO3a
Serum
PBS
EGF
Insulin
FGF1
HGF
NRG1
IGF1
DMSO
BEZ235
PD173074
AZD8055
GSK690693
Combo
−1.2
−1.4
−1.6
−1.8
−2
−2.2
0
5m
15m
30m
60m
2h
4h
12h
24h
48h
72h

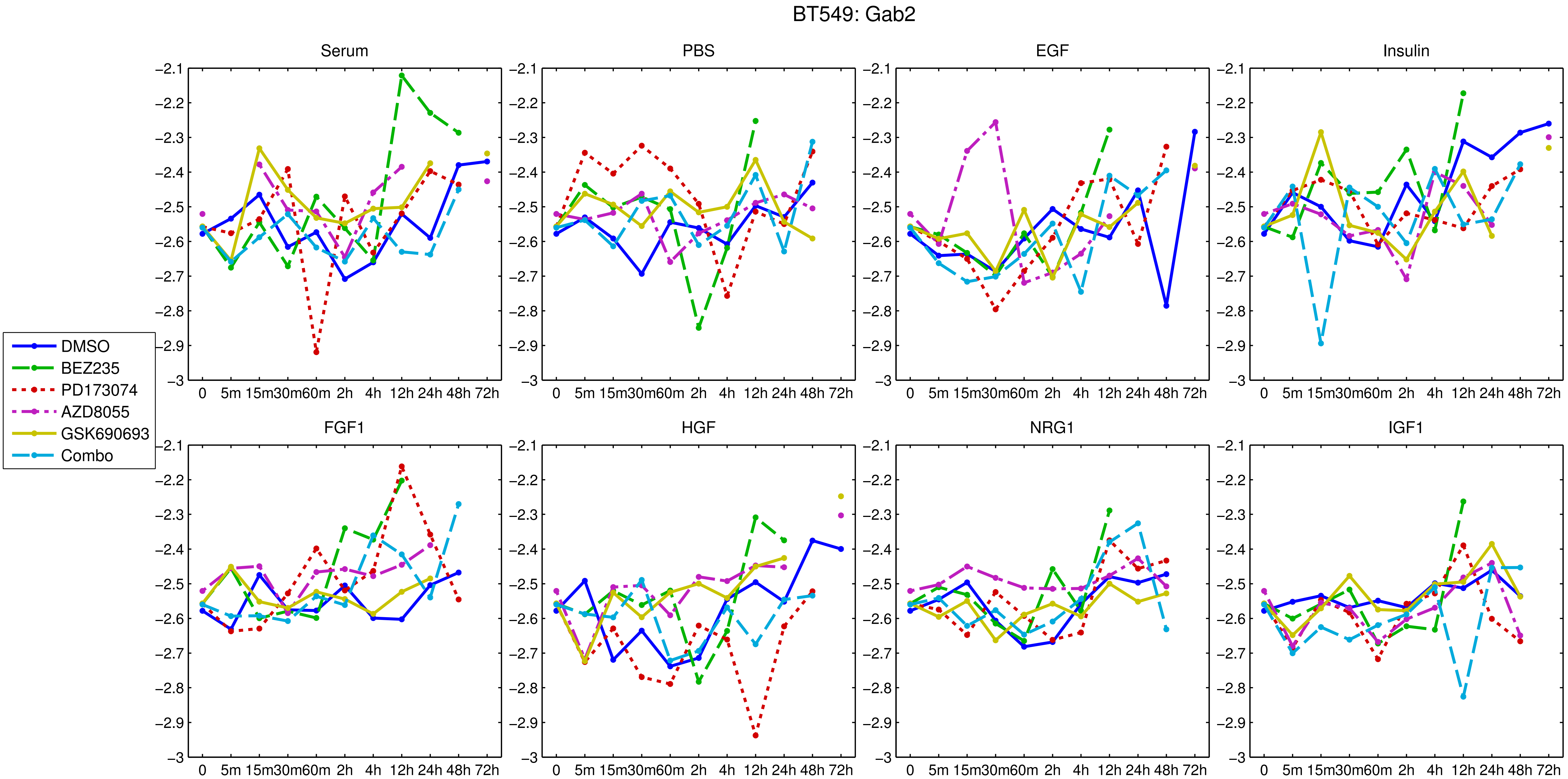

BT549: Gab2
Serum
PBS
EGF
Insulin
FGF1
HGF
NRG1
IGF1
DMSO
BEZ235
PD173074
AZD8055
GSK690693
Combo
−2.1
−2.2
−2.3
−2.4
−2.5
−2.6
−2.7
−2.8
−2.9
−3
0
5m
15m
30m
60m
2h
4h
12h
24h
48h
72h

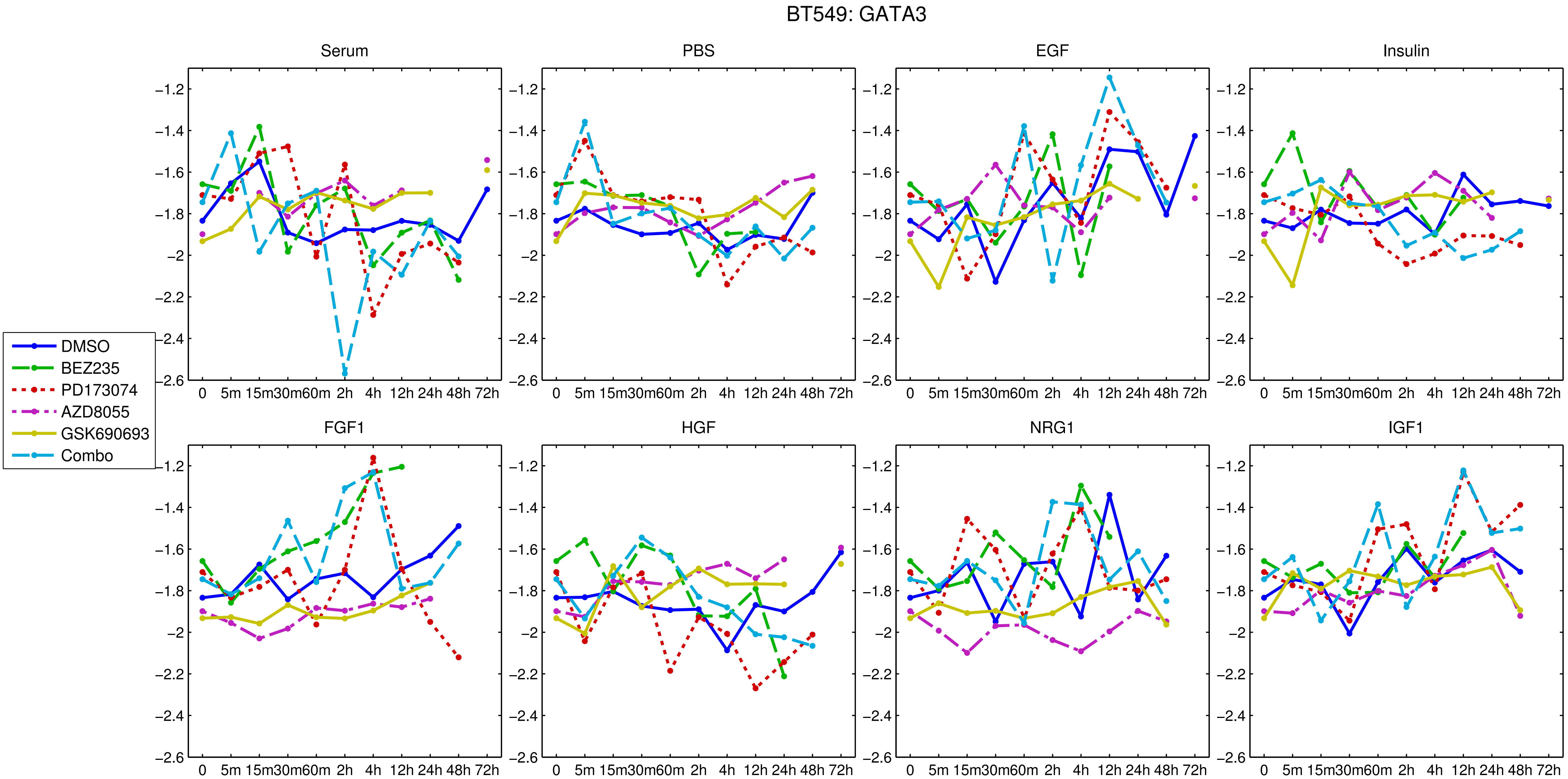

BT549: GATA3
Serum
PBS
EGF
Insulin
FGF1
HGF
NRG1
IGF1
DMSO
BEZ235
PD173074
AZD8055
GSK690693
Combo
−1.2
−1.4
−1.6
−1.8
−2
−2.2
−2.4
−2.6
0 5m 15m 30m 60m 2h 4h 12h 24h 48h 72h

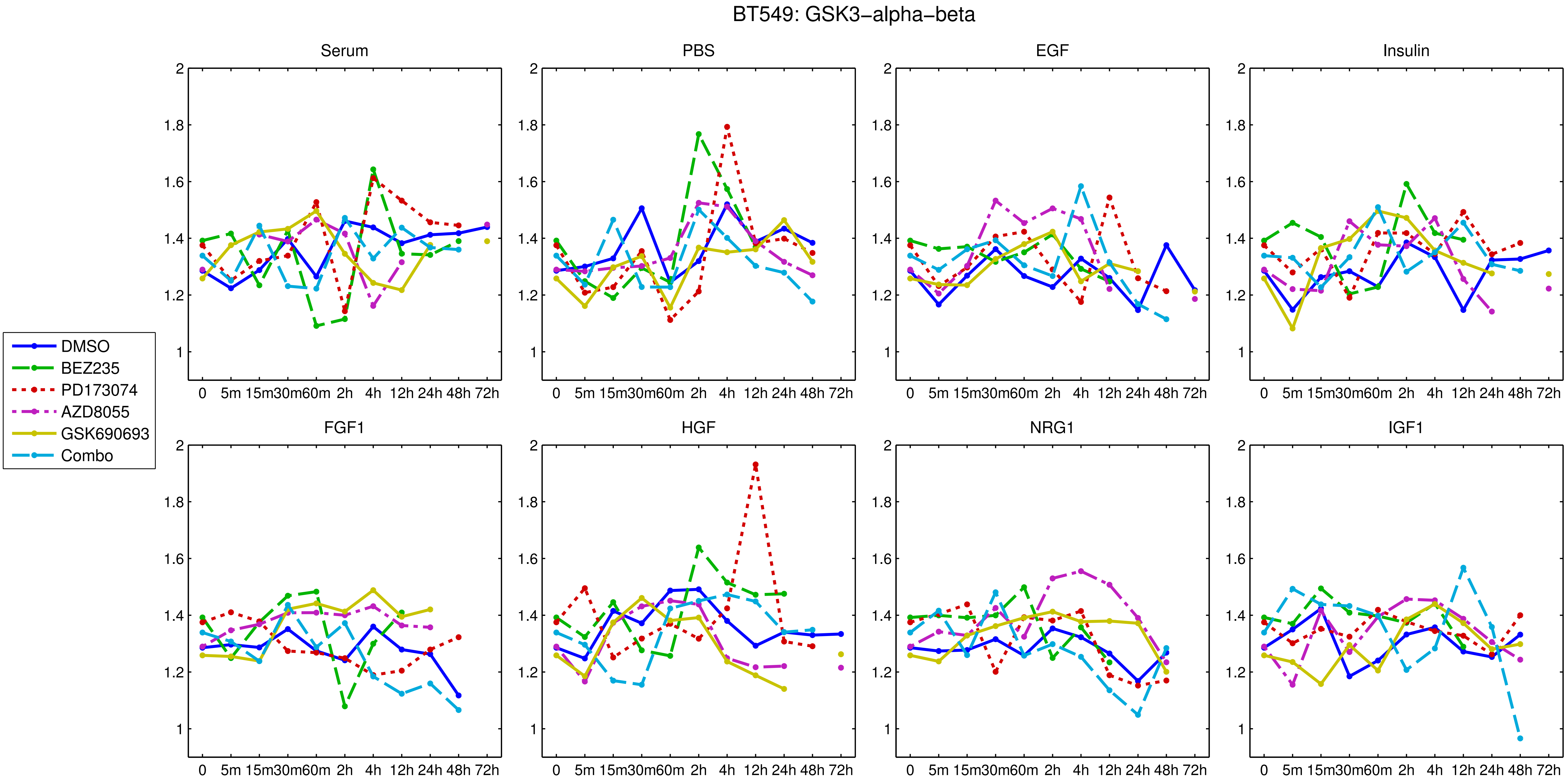
BT549: GSK3−alpha−beta
Serum
PBS
EGF
Insulin
FGF1
HGF
NRG1
IGF1
DMSO
BEZ235
PD173074
AZD8055
GSK690693
Combo
2
1.8
1.6
1.4
1.2
1
0
5m
15m
30m
60m
2h
4h
12h
24h
48h
72h

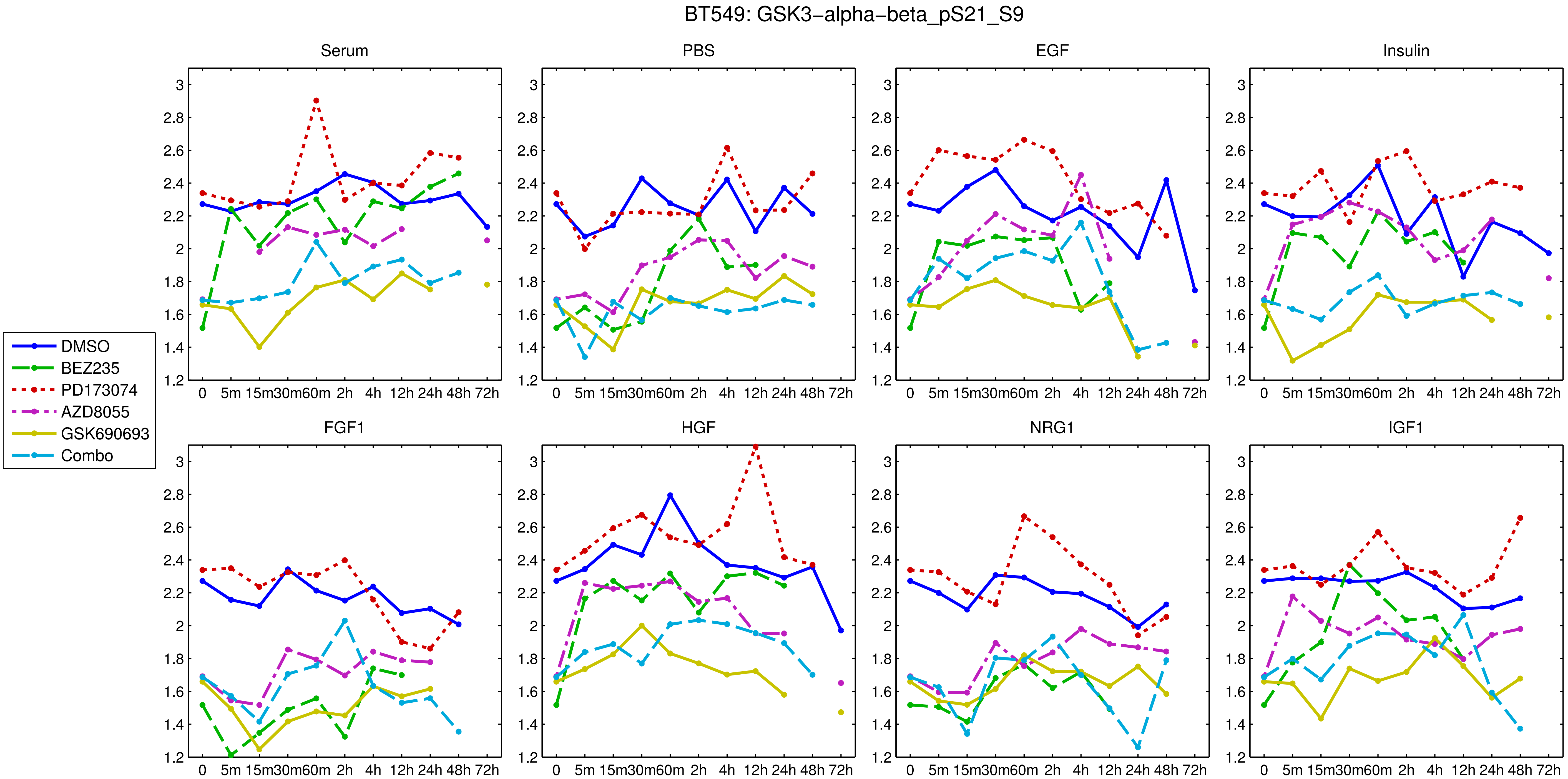

BT549: GSK3−alpha−beta_pS21_S9
Serum
PBS
EGF
Insulin
FGF1
HGF
NRG1
IGF1
DMSO
BEZ235
PD173074
AZD8055
GSK690693
Combo
0 5m 15m 30m 60m 2h 4h 12h 24h 48h 72h
1.2 1.4 1.6 1.8 2 2.2 2.4 2.6 2.8 3

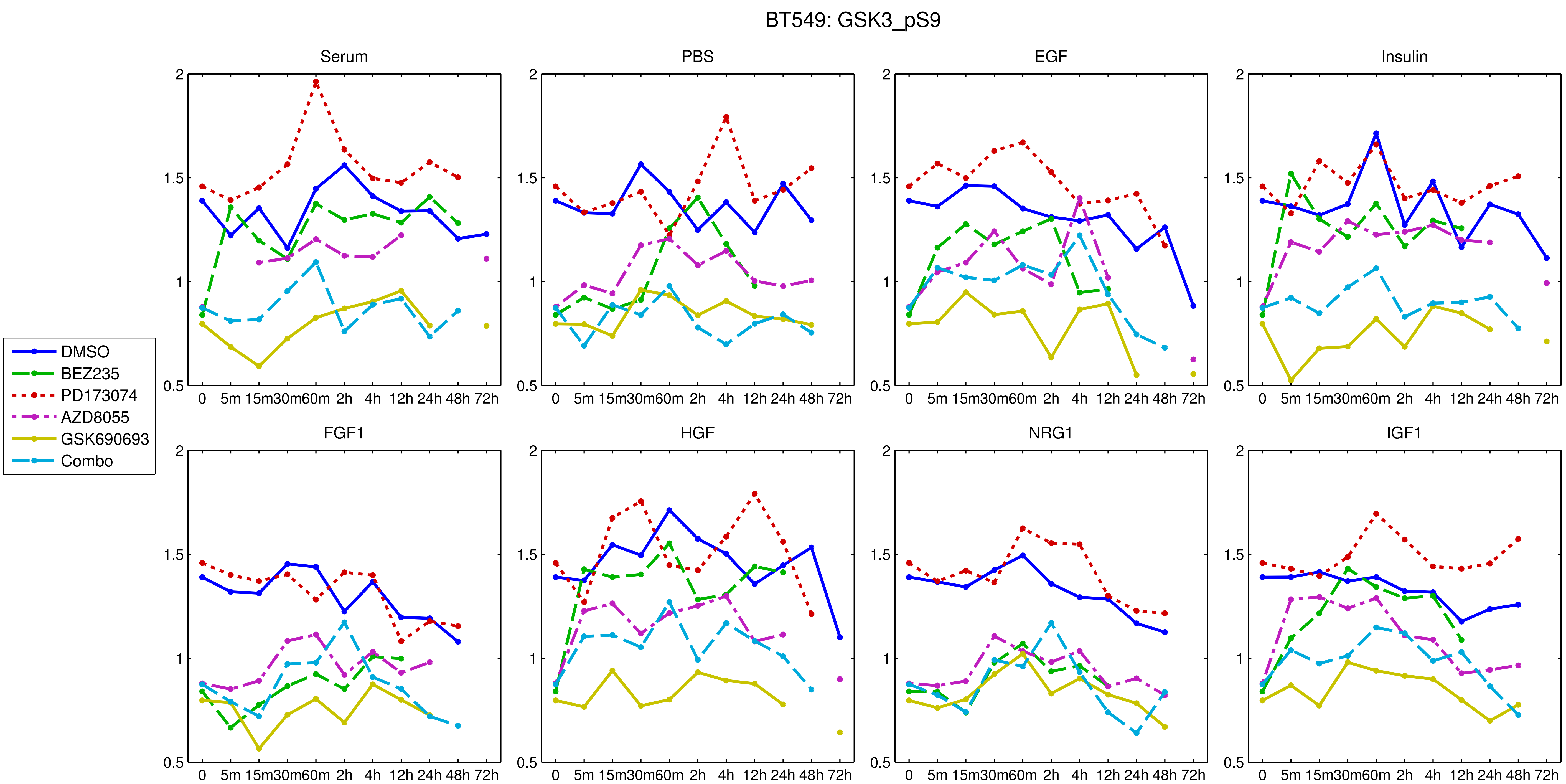

BT549: GSK3_pS9
Serum
PBS
EGF
Insulin
FGF1
HGF
NRG1
IGF1
DMSO
BEZ235
PD173074
AZD8055
GSK690693
Combo
0
0.5
1
1.5
2
0 5m 15m 30m 60m 2h 4h 12h 24h 48h 72h

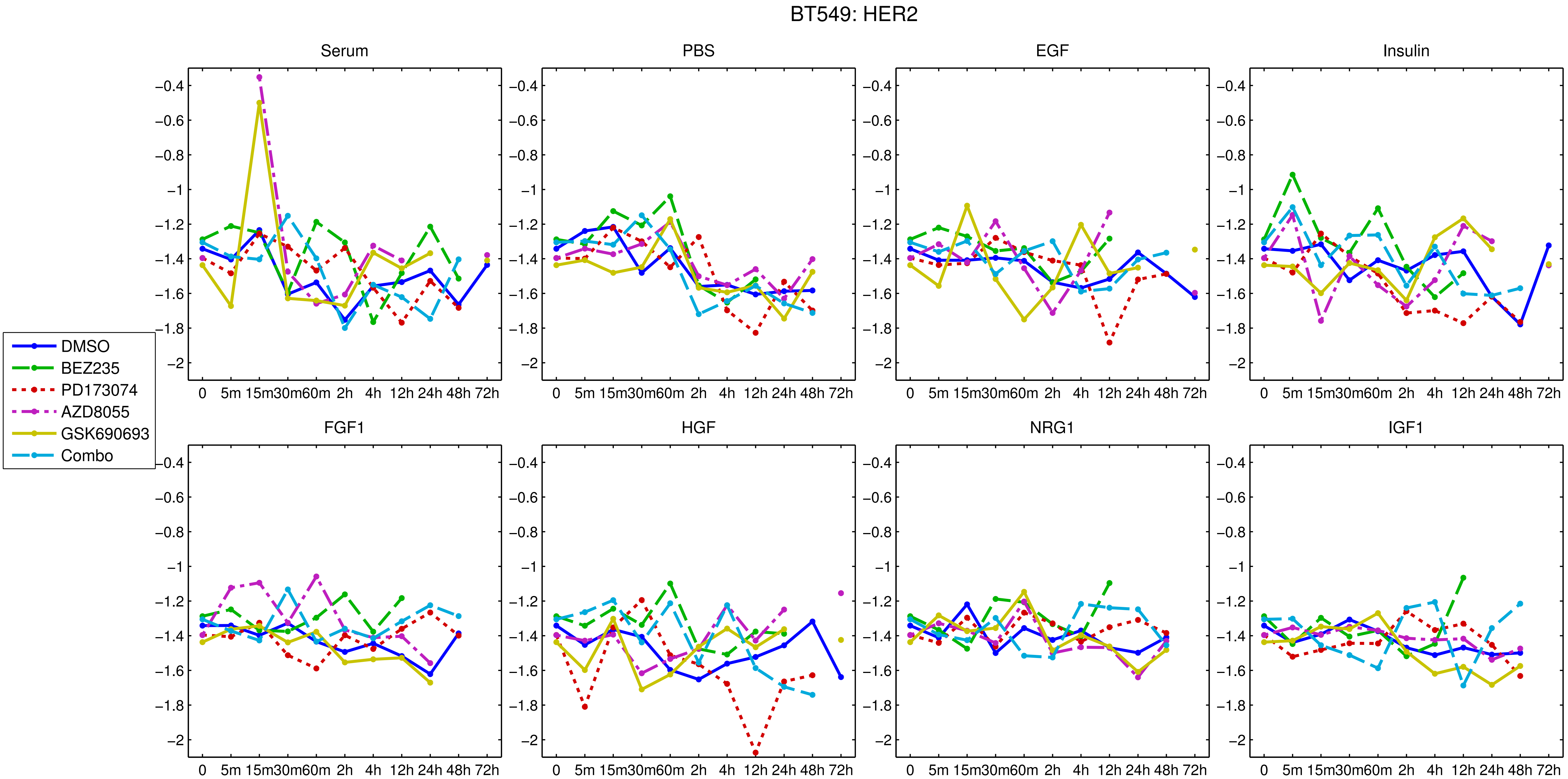
BT549: HER2
Serum
PBS
EGF
Insulin
FGF1
HGF
NRG1
IGF1
DMSO
BEZ235
PD173074
AZD8055
GSK690693
Combo
−0.4
−0.6
−0.8
−1
−1.2
−1.4
−1.6
−1.8
−2
0 5m 15m 30m 60m 2h 4h 12h 24h 48h 72h

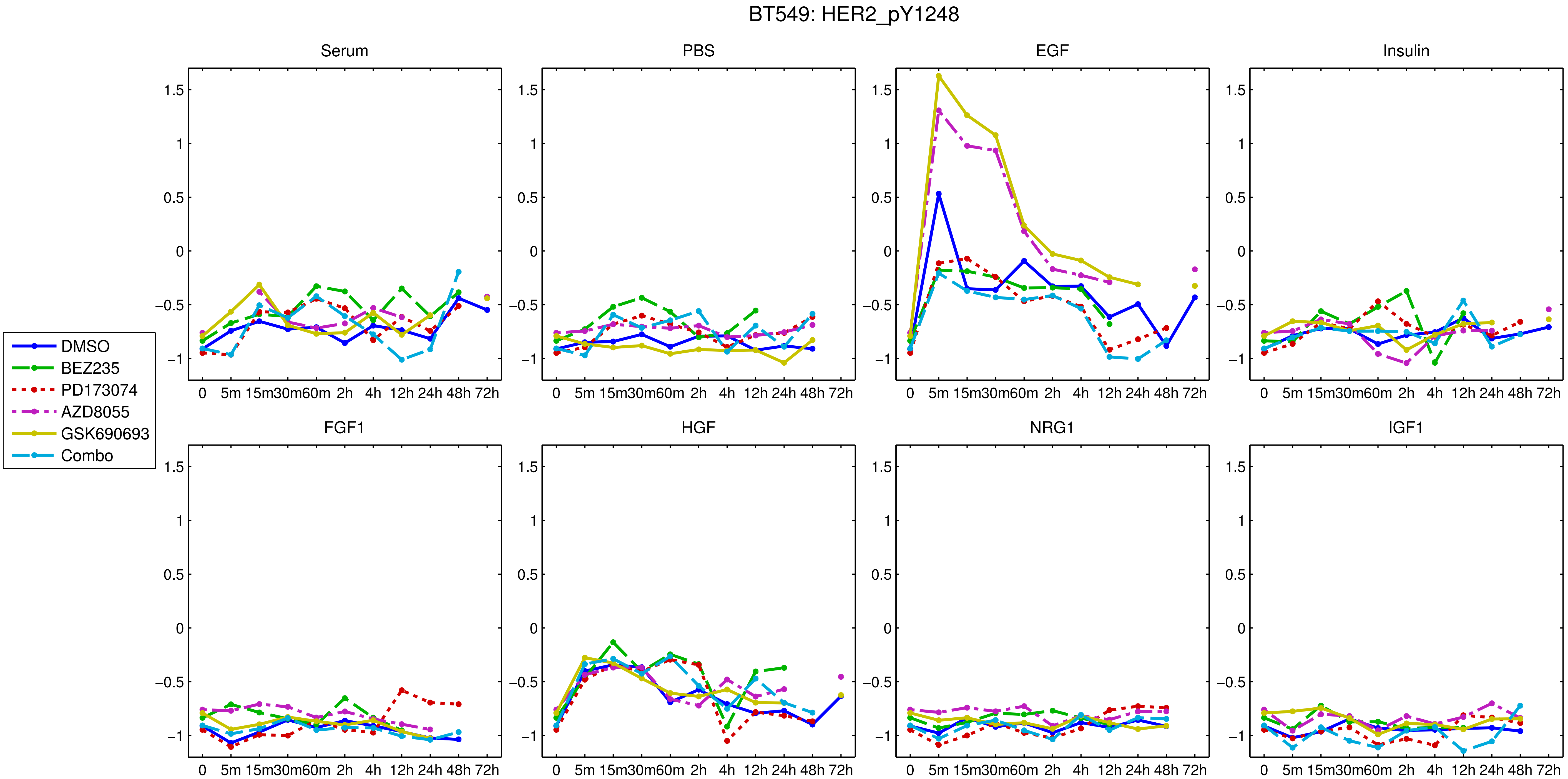

BT549: HER2_pY1248
Serum
PBS
EGF
Insulin
FGF1
HGF
NRG1
IGF1
1.5
1
0.5
0
−0.5
−1
0
5m
15m
30m
60m
2h
4h
12h
24h
48h
72h
DMSO
BEZ235
PD173074
AZD8055
GSK690693
Combo

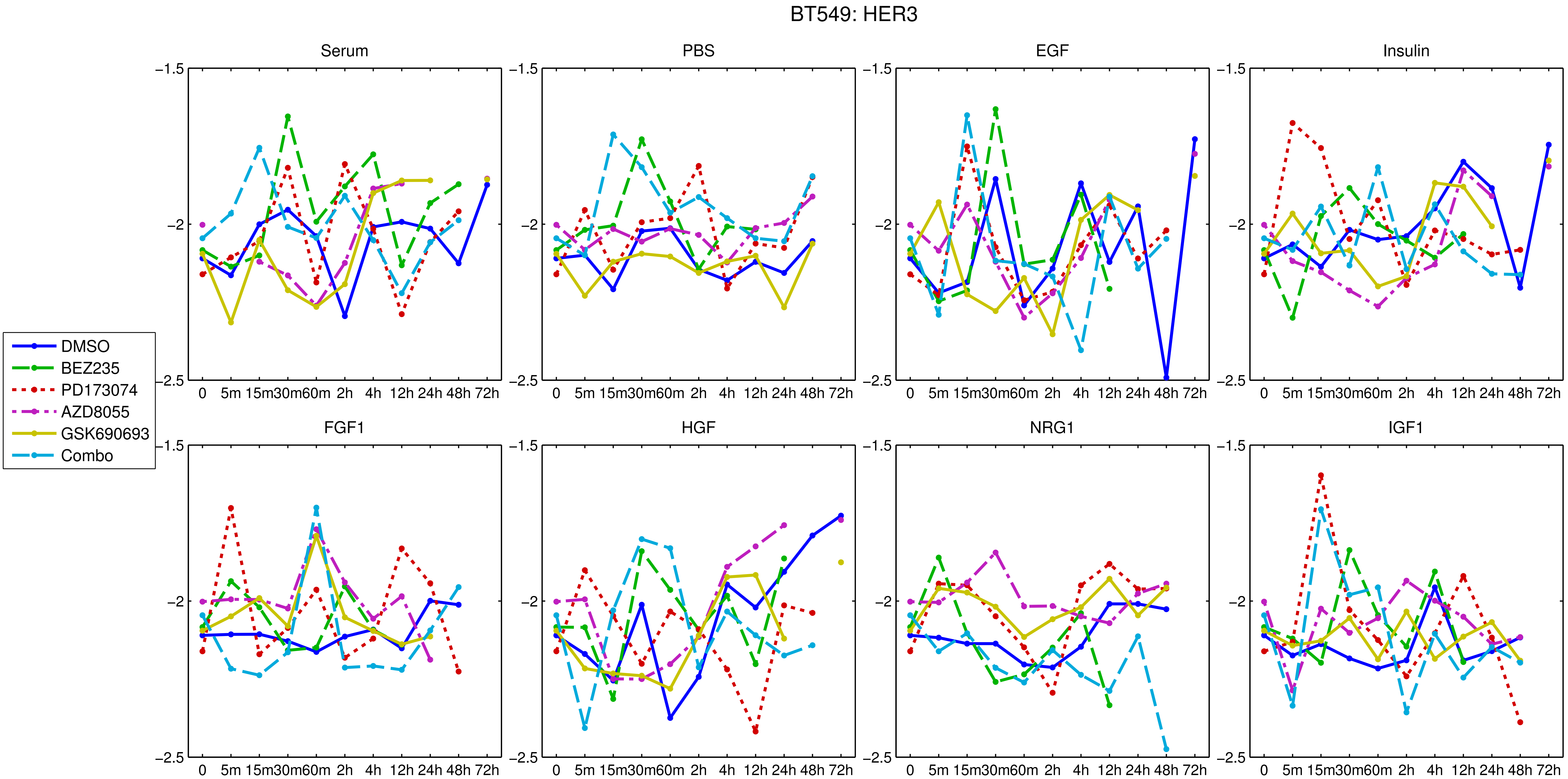

BT549: HER3
Serum
PBS
EGF
Insulin
FGF1
HGF
NRG1
IGF1
−1.5
−2
−2.5
0 5m 15m 30m 60m 2h 4h 12h 24h 48h 72h
DMSO
BEZ235
PD173074
AZD8055
GSK690693
Combo

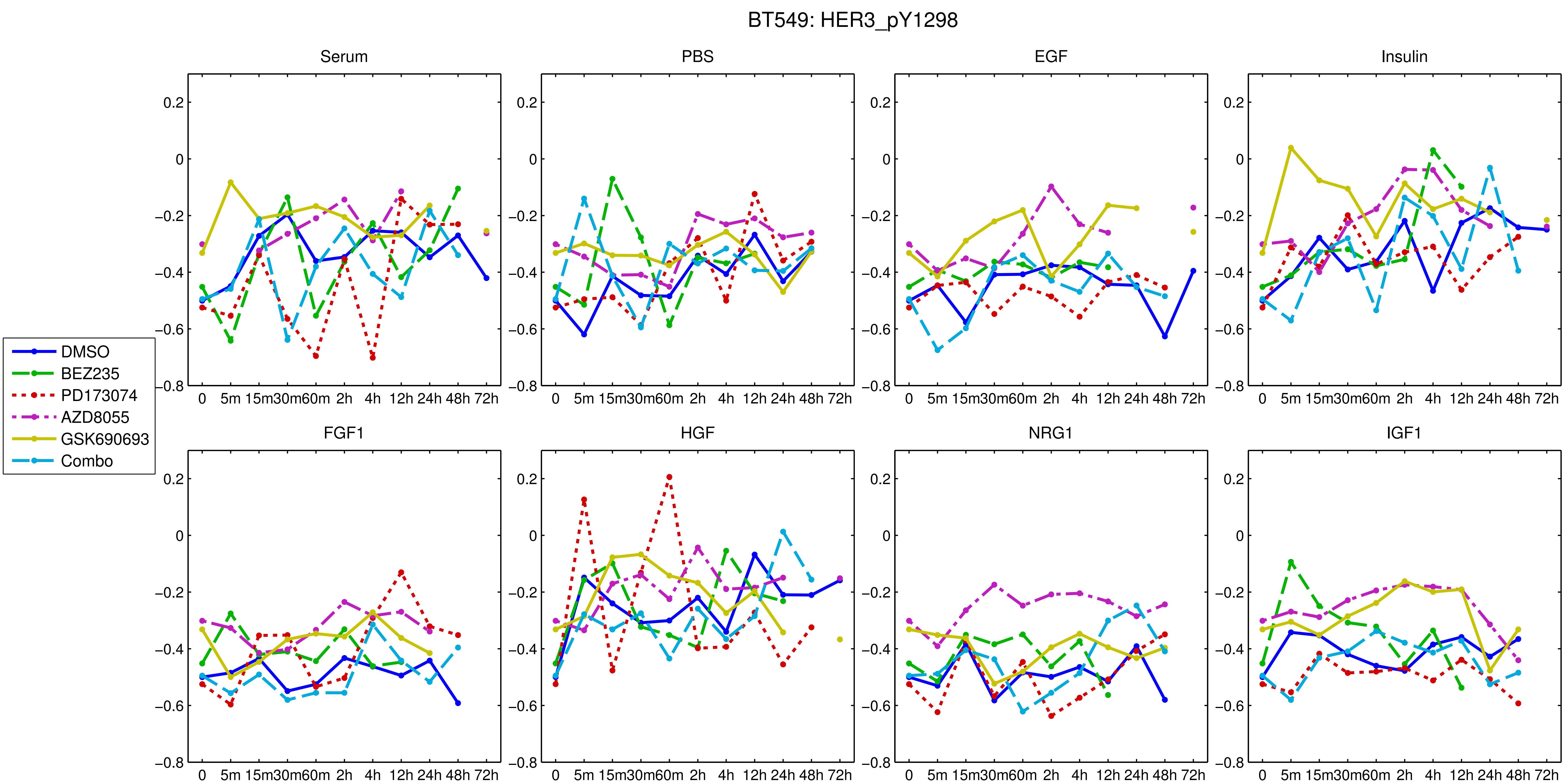
BT549: HER3_pY1298
Serum
PBS
EGF
Insulin
FGF1
HGF
NRG1
IGF1
DMSO
BEZ235
PD173074
AZD8055
GSK690693
Combo
0.2
0
−0.2
−0.4
−0.6
−0.8
0
5m
15m
30m
60m
2h
4h
12h
24h
48h
72h

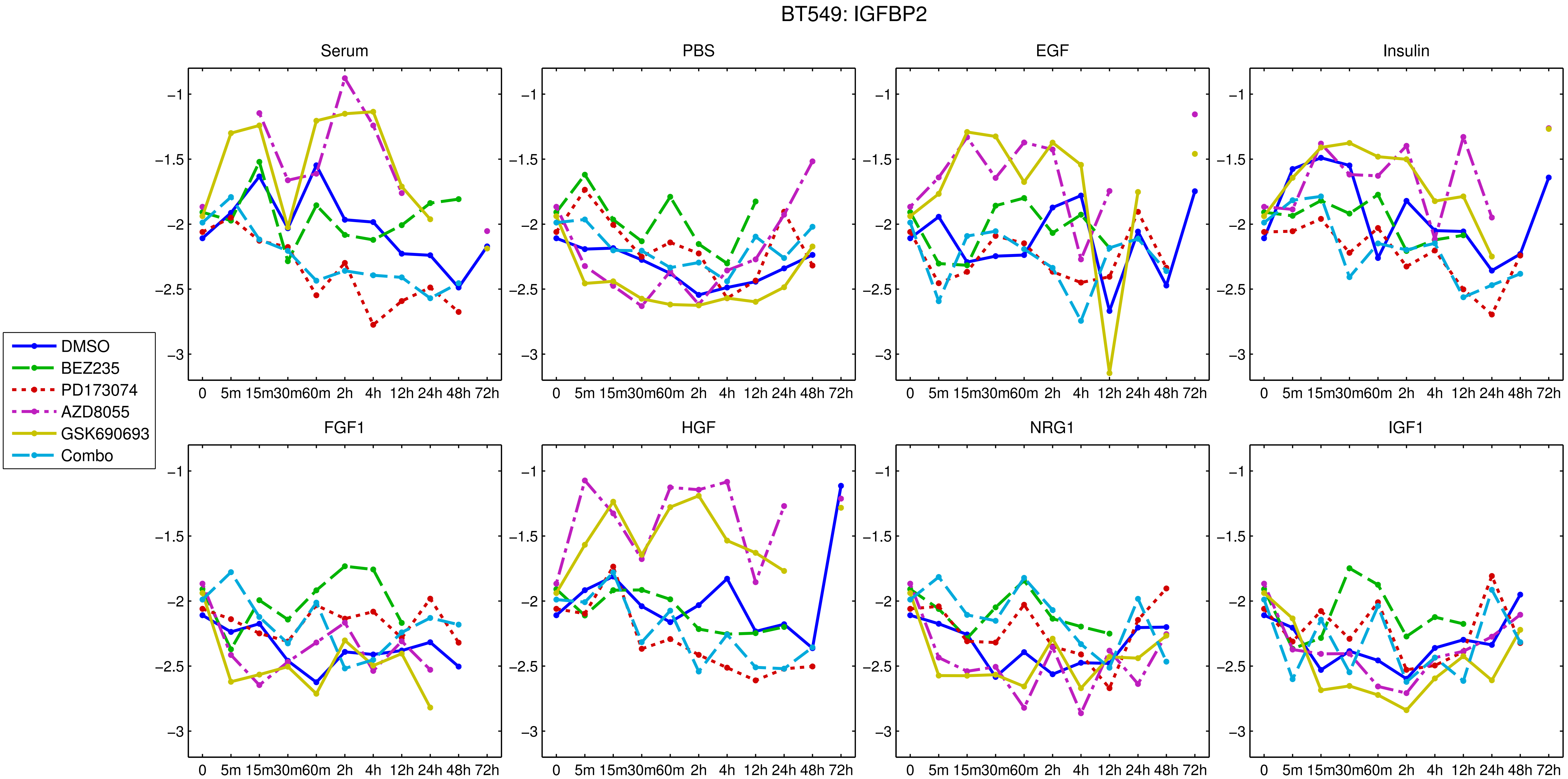
BT549: IGFBP2
Serum
PBS
EGF
Insulin
FGF1
HGF
NRG1
IGF1
DMSO
BEZ235
PD173074
AZD8055
GSK690693
Combo
−1
−1.5
−2
−2.5
−3
0
5m
15m
30m
60m
2h
4h
12h
24h
48h
72h

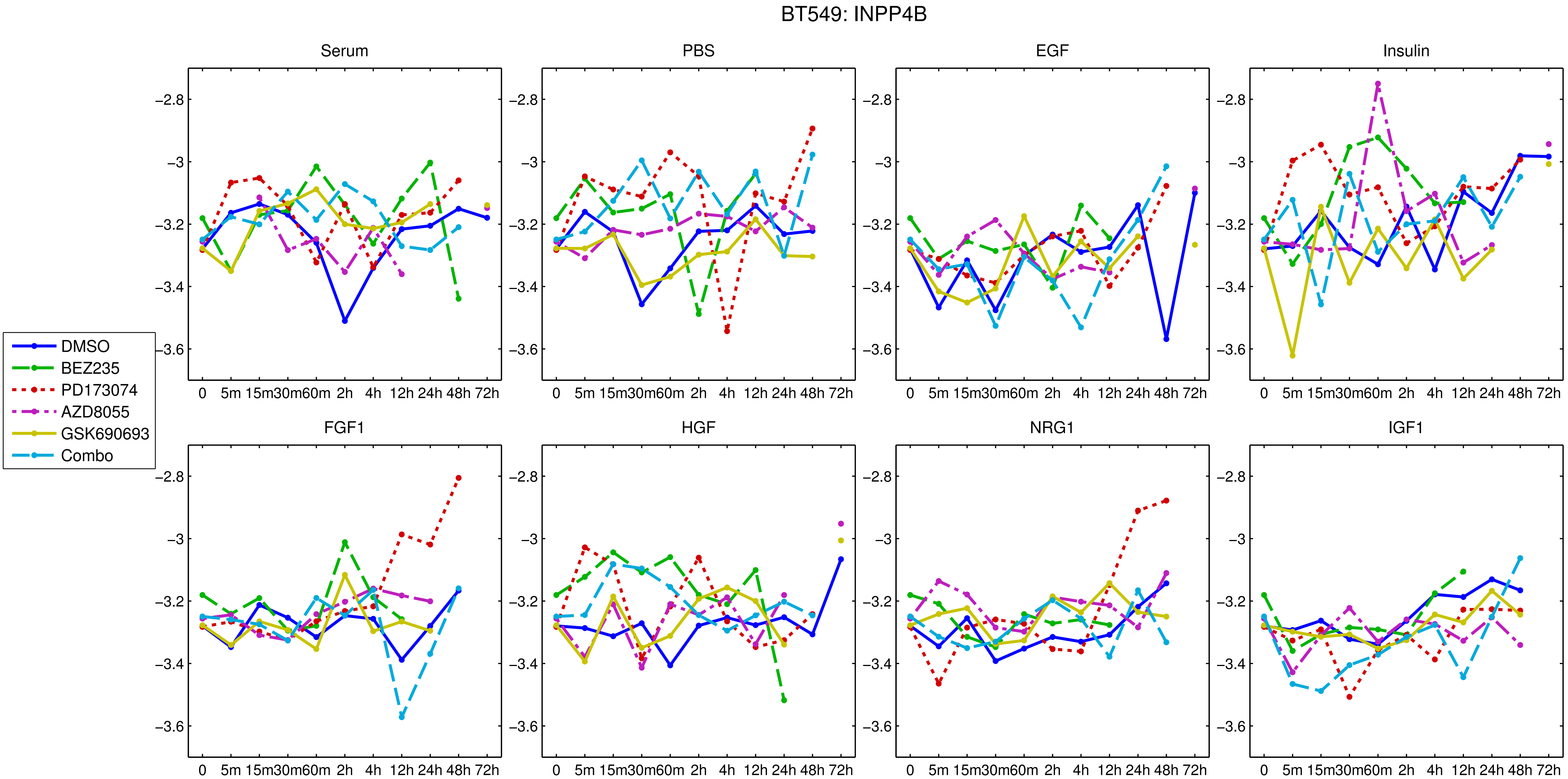
BT549: INPP4B
Serum
PBS
EGF
Insulin
FGF1
HGF
NRG1
IGF1
DMSO
BEZ235
PD173074
AZD8055
GSK690693
Combo
−2.8
−3
−3.2
−3.4
−3.6
0 5m 15m 30m 60m 2h 4h 12h 24h 48h 72h

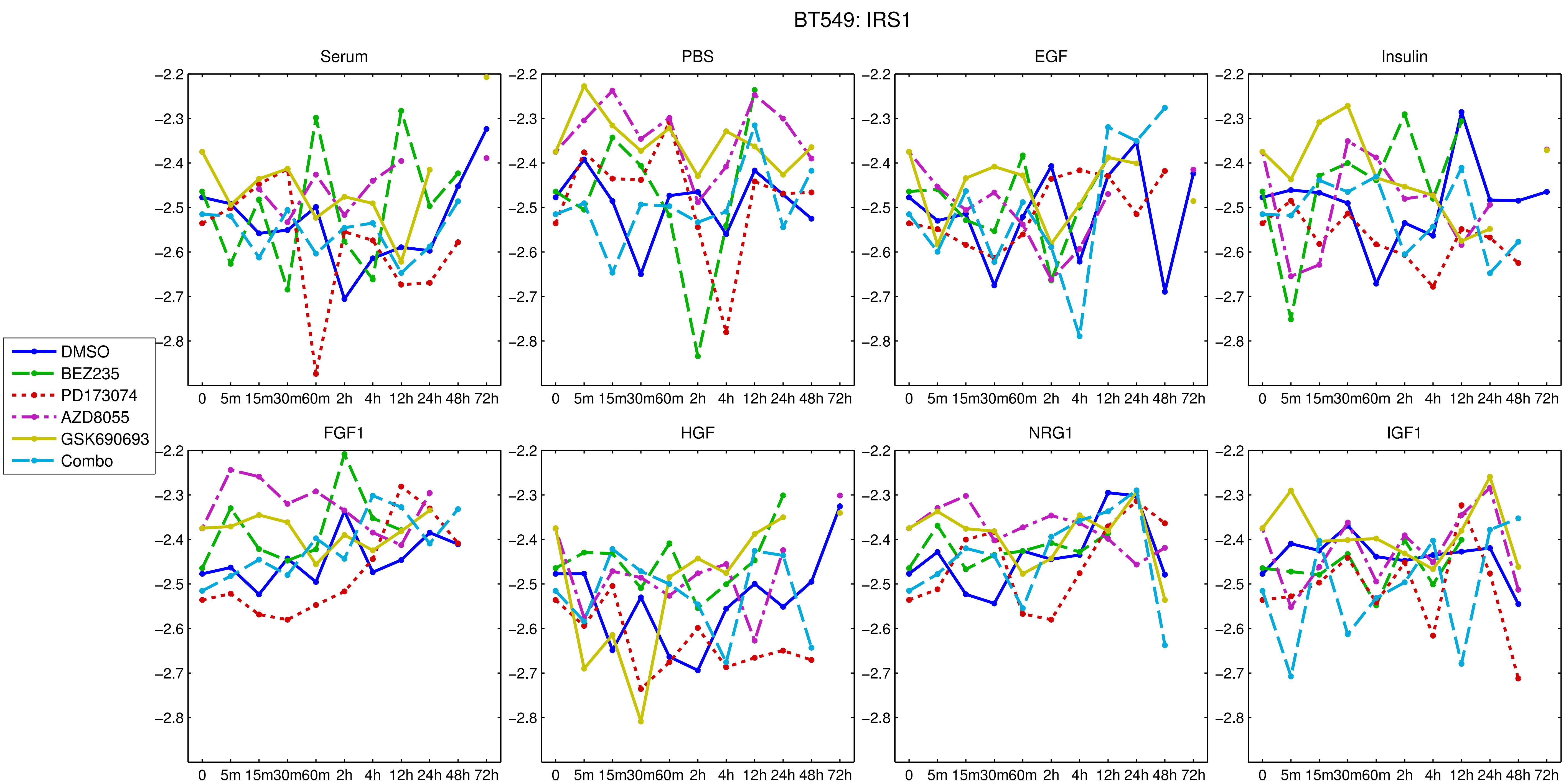
BT549: IRS1
Serum
PBS
EGF
Insulin
FGF1
HGF
NRG1
IGF1
−2.2
−2.3
−2.4
−2.5
−2.6
−2.7
−2.8
0
5m
15m
30m
60m
2h
4h
12h
24h
48h
72h
DMSO
BEZ235
PD173074
AZD8055
GSK690693
Combo

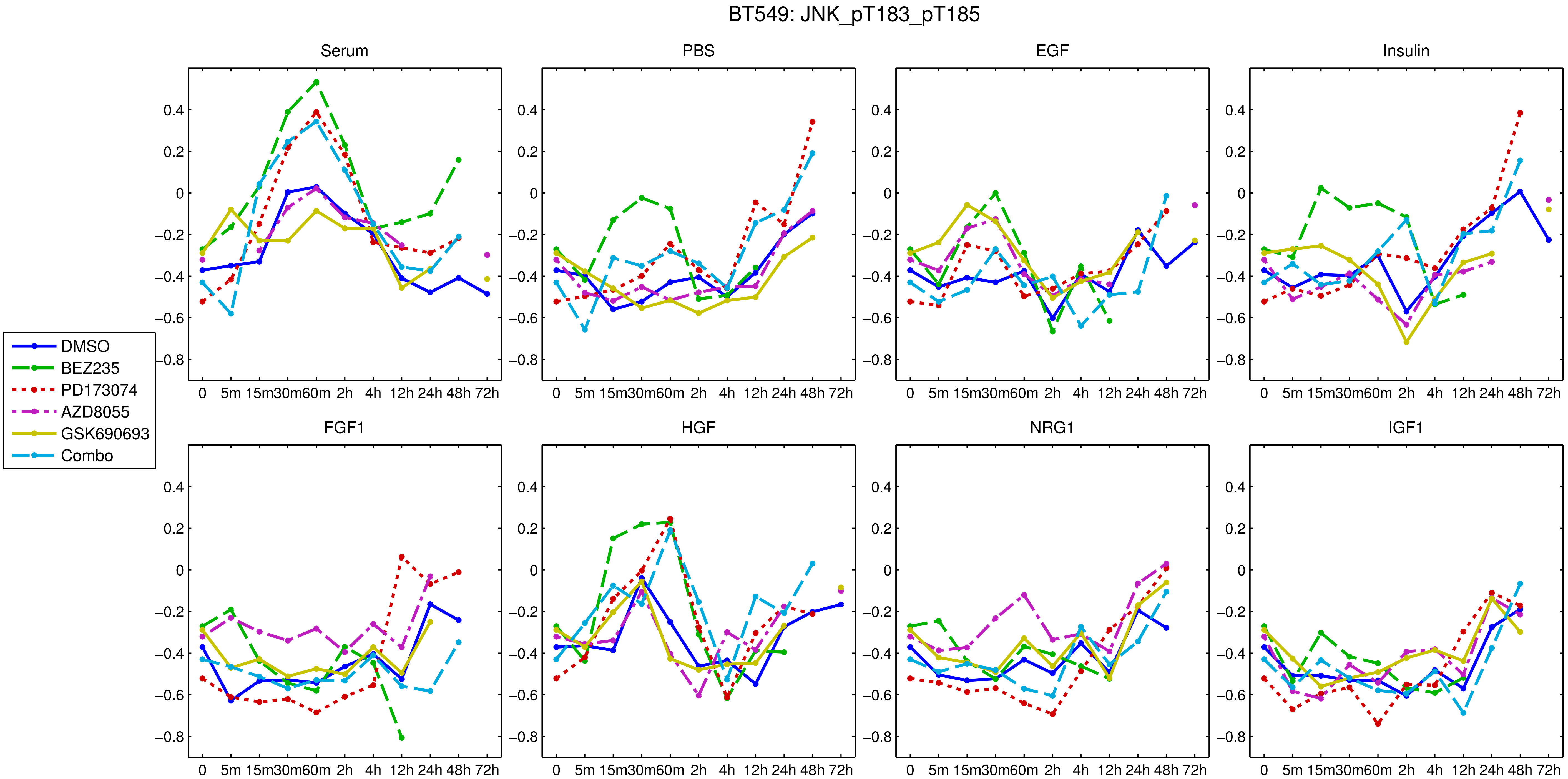
BT549: JNK_pT183_pT185
Serum
PBS
EGF
Insulin
FGF1
HGF
NRG1
IGF1
DMSO
BEZ235
PD173074
AZD8055
GSK690693
Combo
0.4
0.2
0
−0.2
−0.4
−0.6
−0.8
0
5m
15m
30m
60m
2h
4h
12h
24h
48h
72h

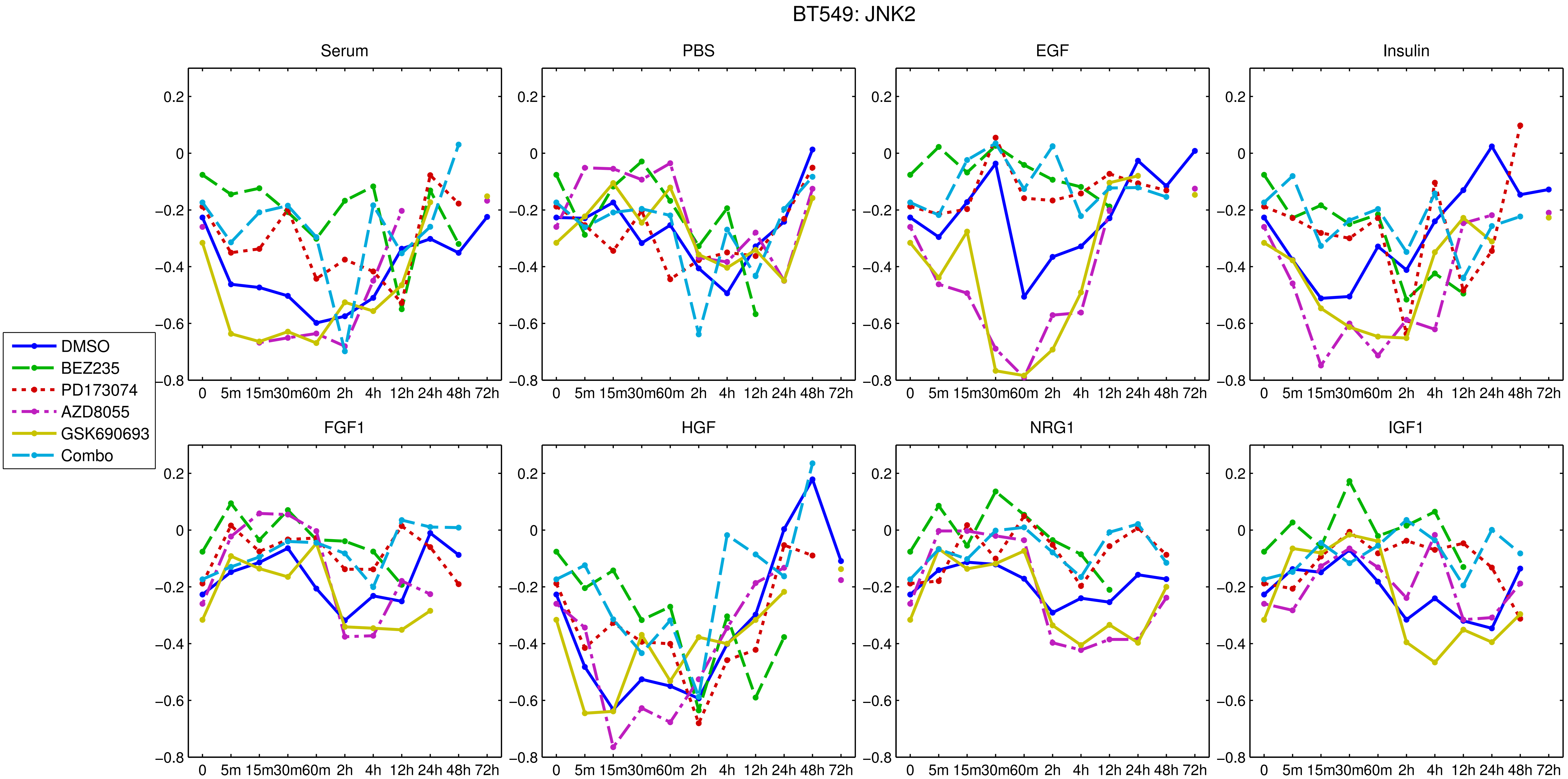
BT549: JNK2
Serum
PBS
EGF
Insulin
FGF1
HGF
NRG1
IGF1
DMSO
BEZ235
PD173074
AZD8055
GSK690693
Combo
0.2
0
−0.2
−0.4
−0.6
−0.8
0 5m 15m 30m 60m 2h 4h 12h 24h 48h 72h

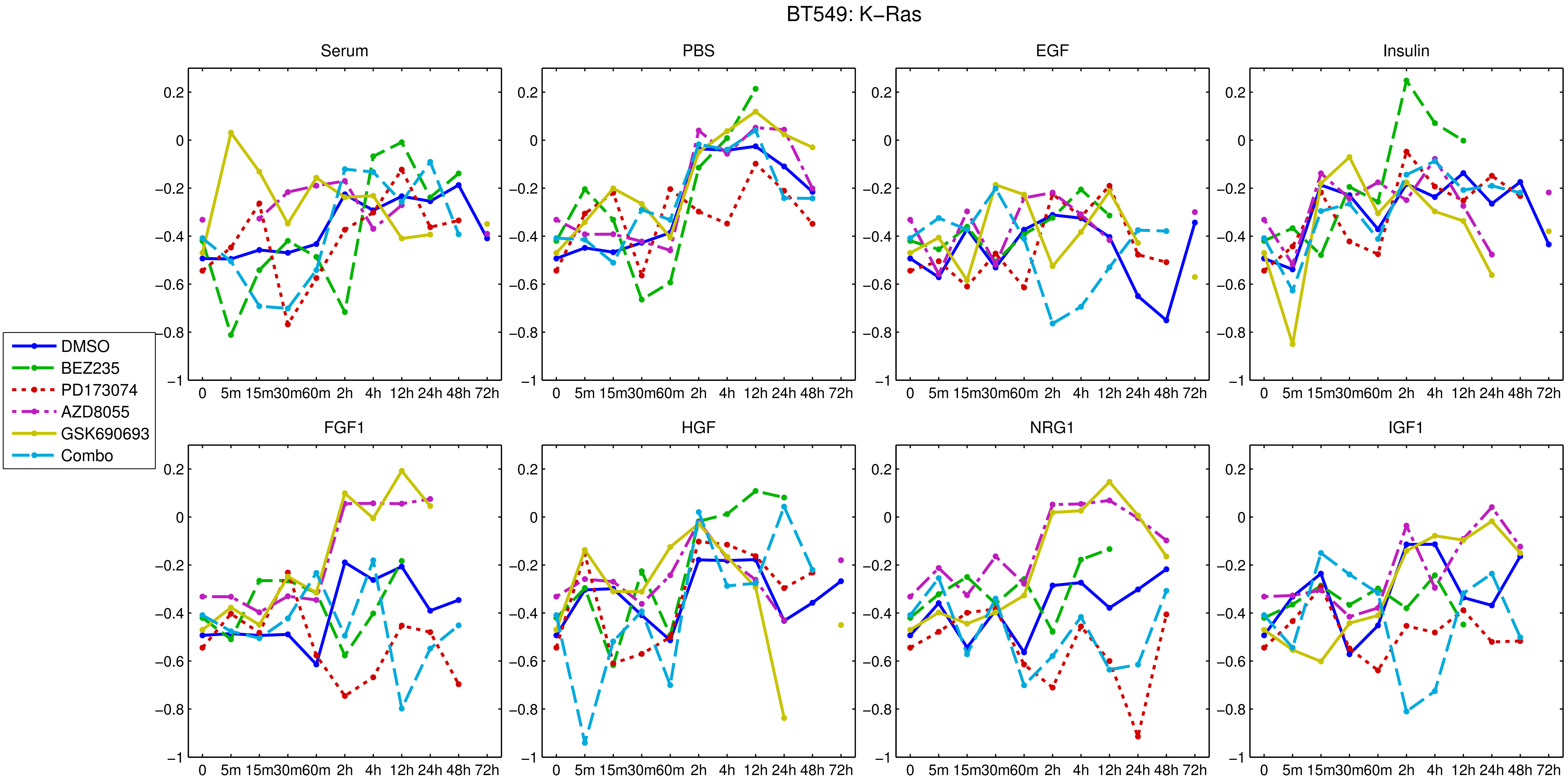
BT549: K−Ras
Serum
PBS
EGF
Insulin
FGF1
HGF
NRG1
IGF1
DMSO
BEZ235
PD173074
AZD8055
GSK690693
Combo
0.2
0
−0.2
−0.4
−0.6
−0.8
−1
0 5m 15m 30m 60m 2h 4h 12h 24h 48h 72h

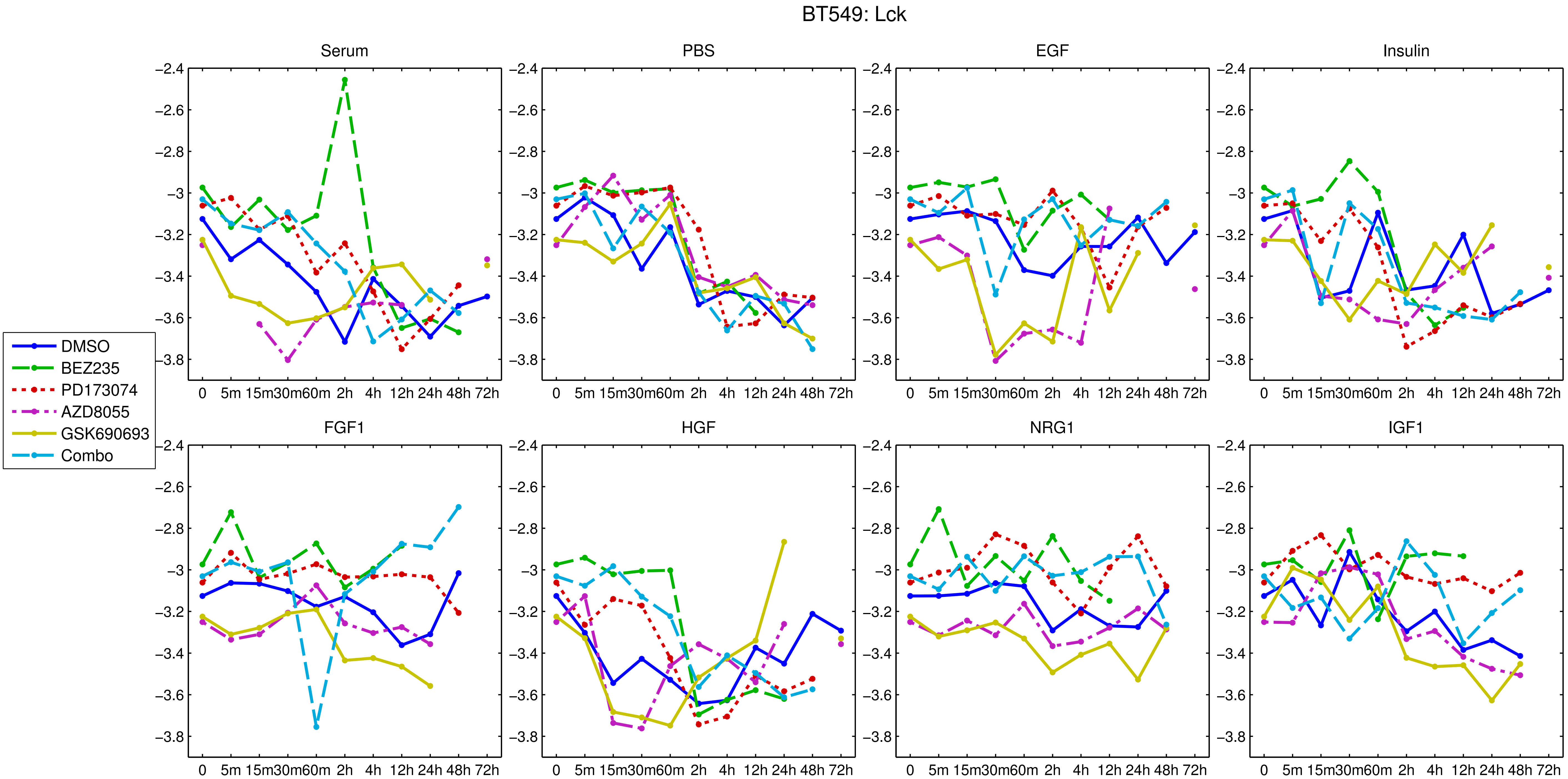
BT549: Lck
Serum
PBS
EGF
Insulin
FGF1
HGF
NRG1
IGF1
DMSO
BEZ235
PD173074
AZD8055
GSK690693
Combo
−2.4
−2.6
−2.8
−3
−3.2
−3.4
−3.6
−3.8
0 5m 15m 30m 60m 2h 4h 12h 24h 48h 72h

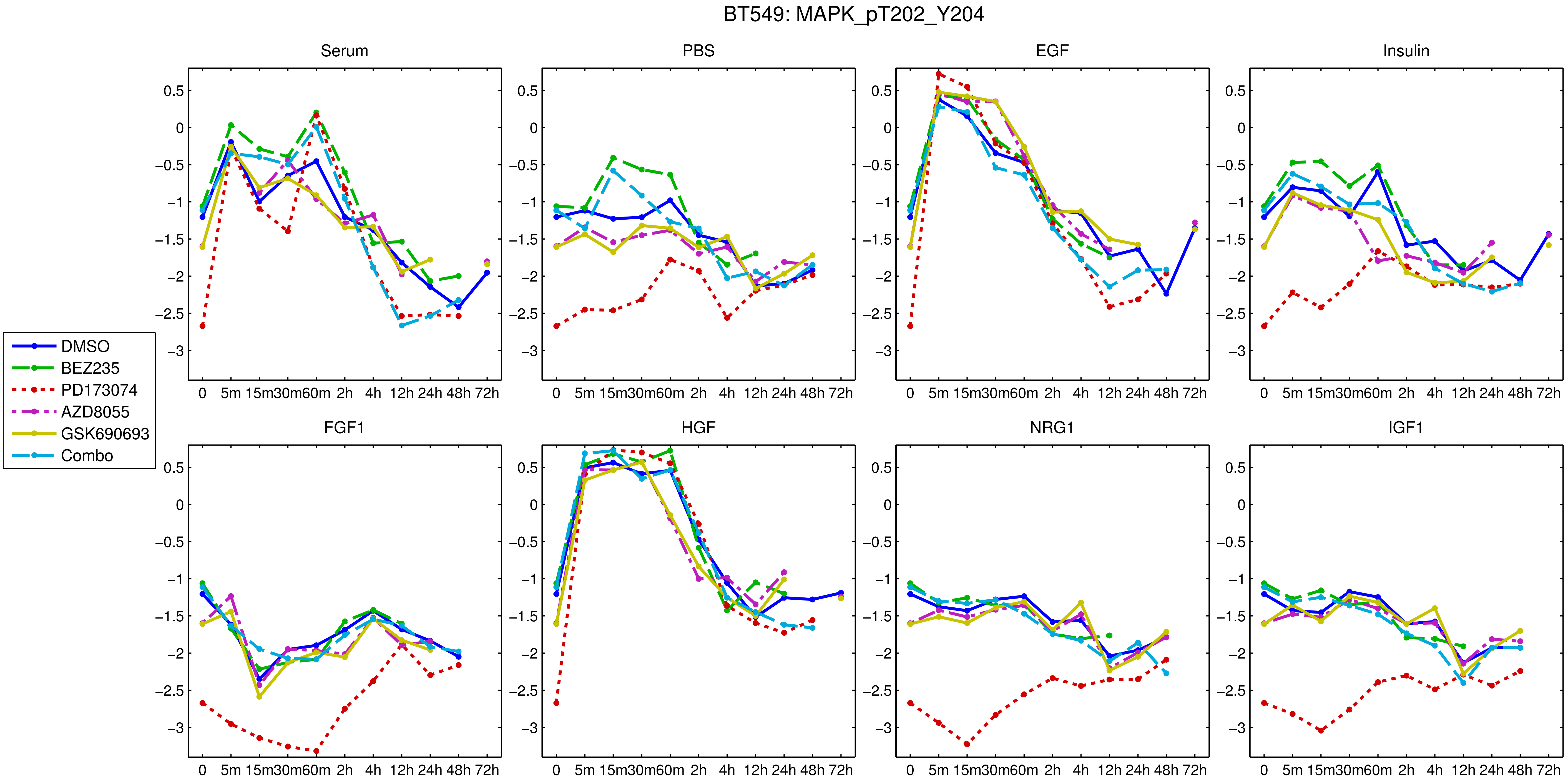

BT549: MAPK_pT202_Y204
Serum
PBS
EGF
Insulin
FGF1
HGF
NRG1
IGF1
0.5
0
−0.5
−1
−1.5
−2
−2.5
−3
0
5m
15m
30m
60m
2h
4h
12h
24h
48h
72h
DMSO
BEZ235
PD173074
AZD8055
GSK690693
Combo

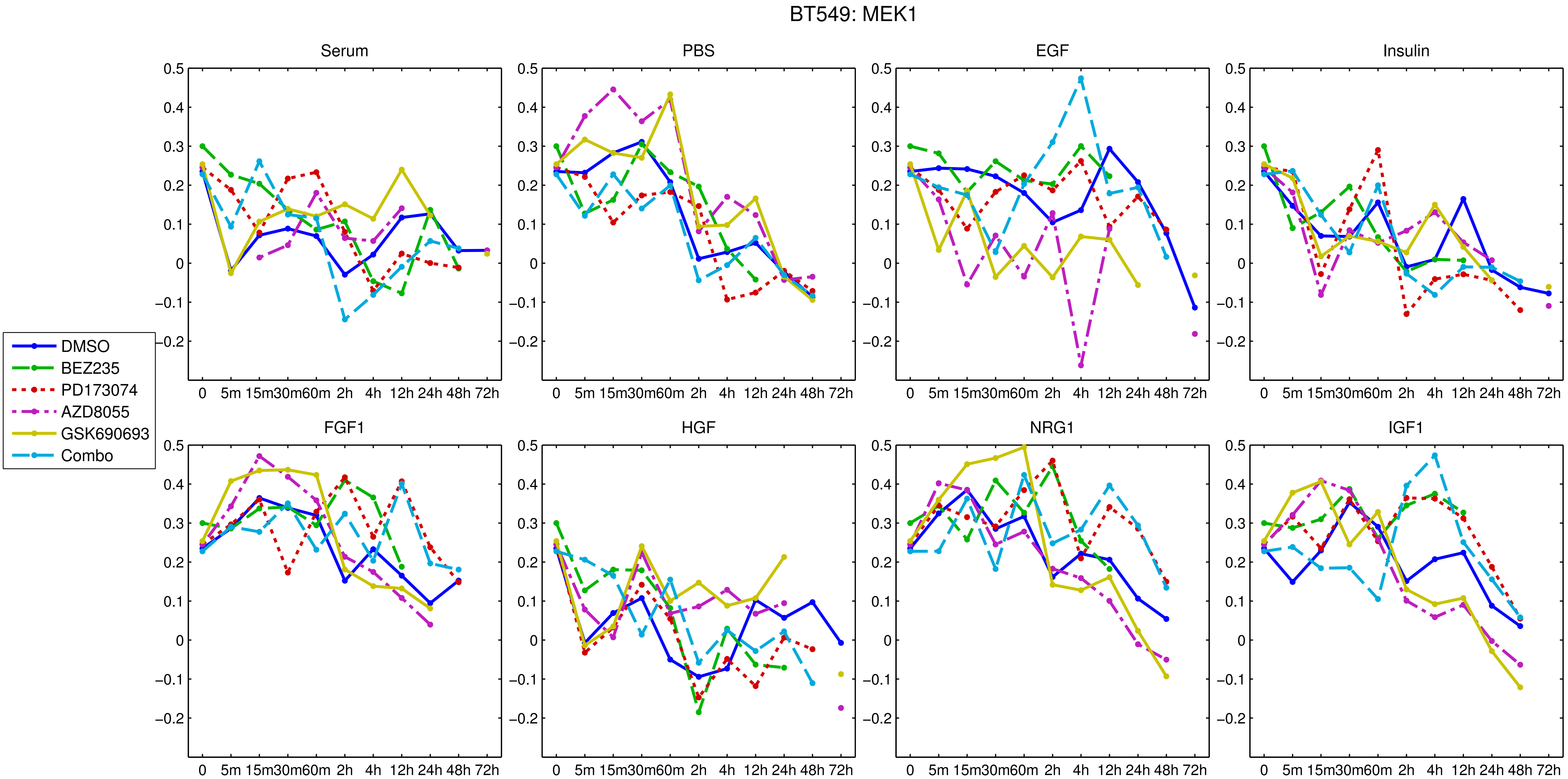
BT549: MEK1
Serum
PBS
EGF
Insulin
FGF1
HGF
NRG1
IGF1
DMSO
BEZ235
PD173074
AZD8055
GSK690693
Combo
0.5
0.4
0.3
0.2
0.1
0
−0.1
−0.2
0
5m
15m
30m
60m
2h
4h
12h
24h
48h
72h

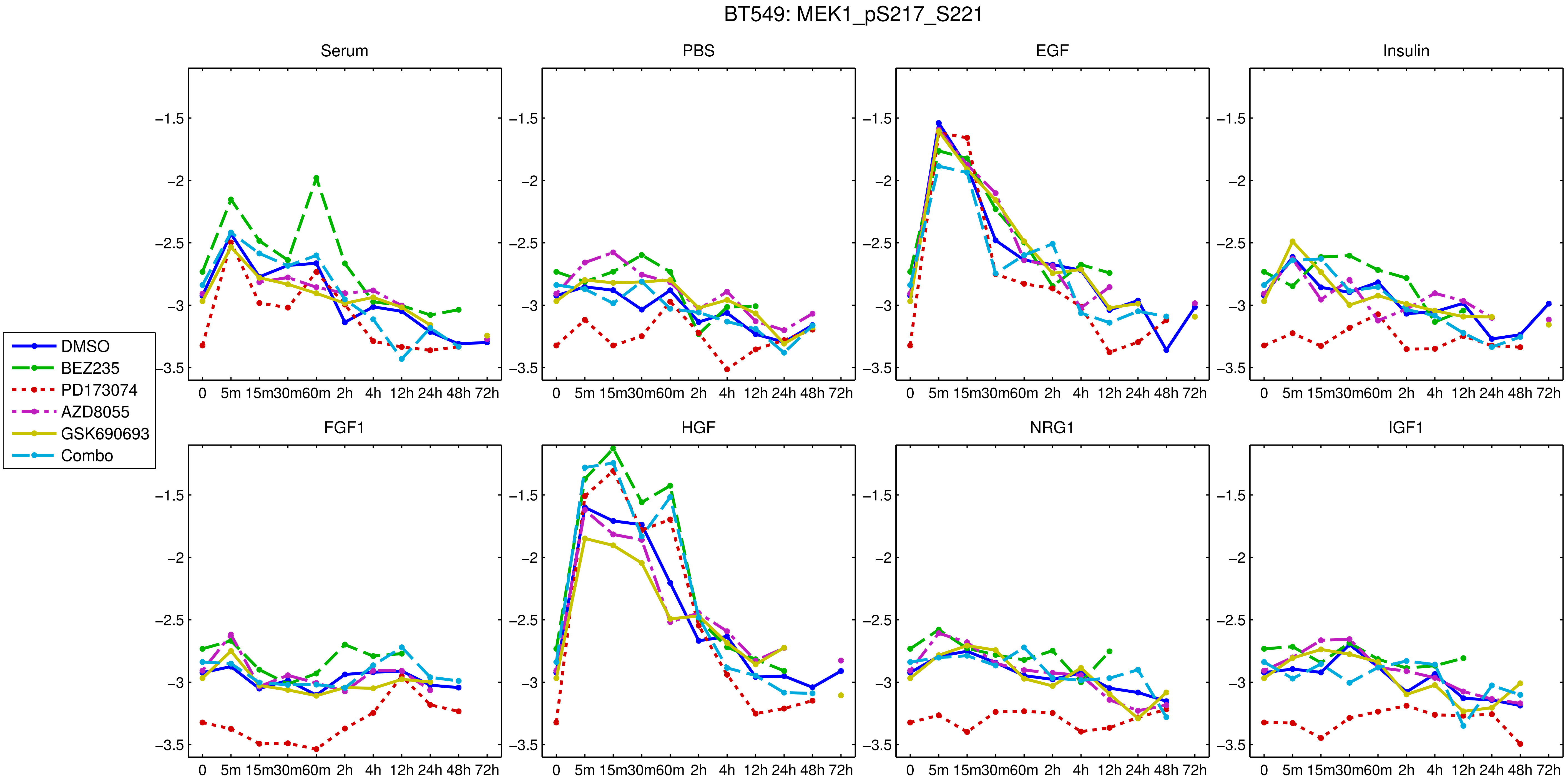
BT549: MEK1_pS217_S221
Serum
PBS
EGF
Insulin
FGF1
HGF
NRG1
IGF1
−1.5
−2
−2.5
−3
−3.5
0
5m
15m
30m
60m
2h
4h
12h
24h
48h
72h
DMSO
BEZ235
PD173074
AZD8055
GSK690693
Combo

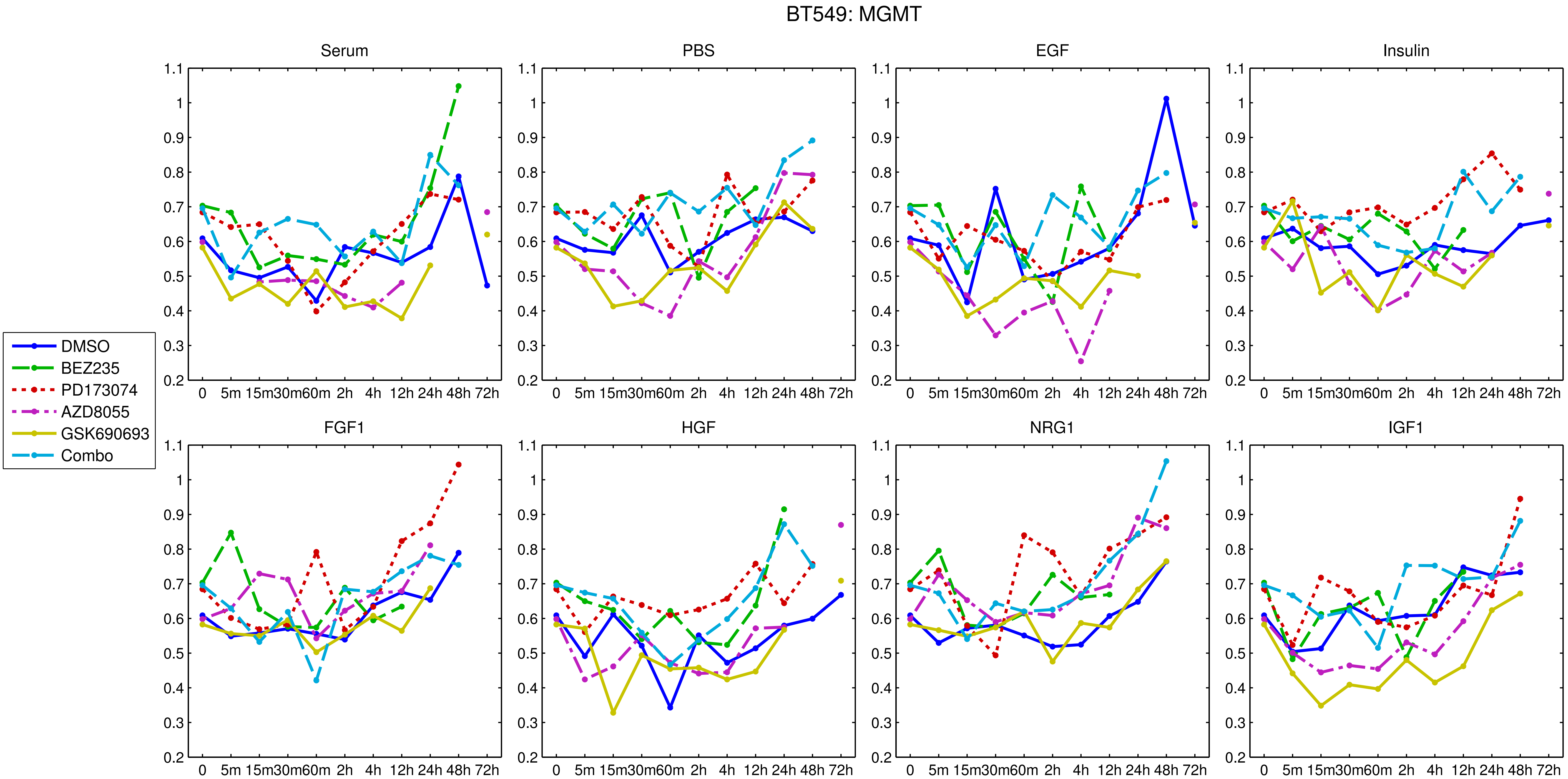
BT549: MGMT
Serum
PBS
EGF
Insulin
FGF1
HGF
NRG1
IGF1
DMSO
BEZ235
PD173074
AZD8055
GSK690693
Combo
0
5m
15m
30m
60m
2h
4h
12h
24h
48h
72h
0.2
0.3
0.4
0.5
0.6
0.7
0.8
0.9
1
1.1

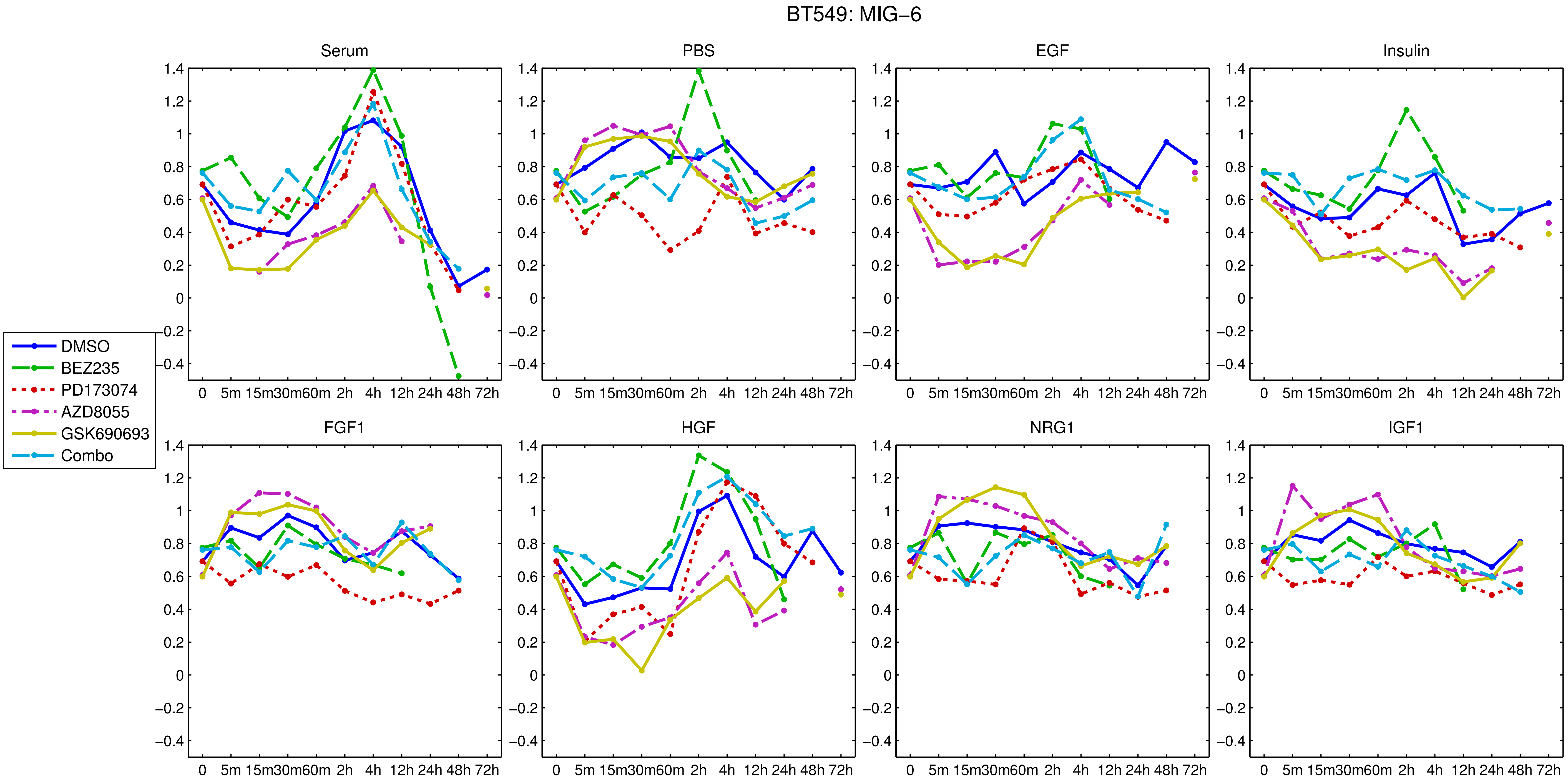

BT549: MIG-6
Serum
PBS
EGF
Insulin
FGF1
HGF
NRG1
IGF1
DMSO
BEZ235
PD173074
AZD8055
GSK690693
Combo
1.4
1.2
1
0.8
0.6
0.4
0.2
0
−0.2
−0.4
0
5m
15m
30m
60m
2h
4h
12h
24h
48h
72h

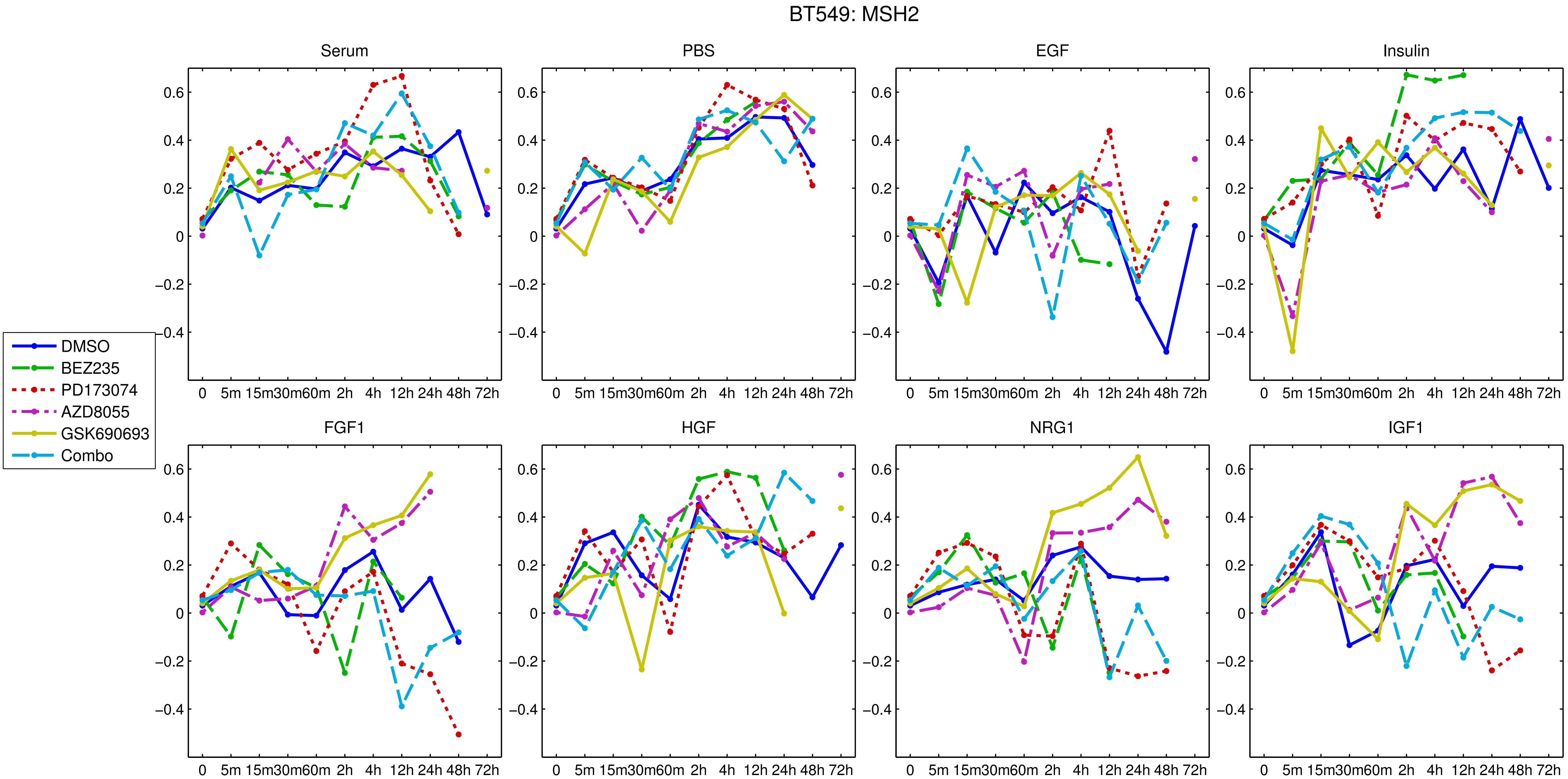
BT549: MSH2
Serum
PBS
EGF
Insulin
FGF1
HGF
NRG1
IGF1
0.6
0.4
0.2
0
−0.2
−0.4
0 5m 15m 30m 60m 2h 4h 12h 24h 48h 72h
DMSO
BEZ235
PD173074
AZD8055
GSK690693
Combo

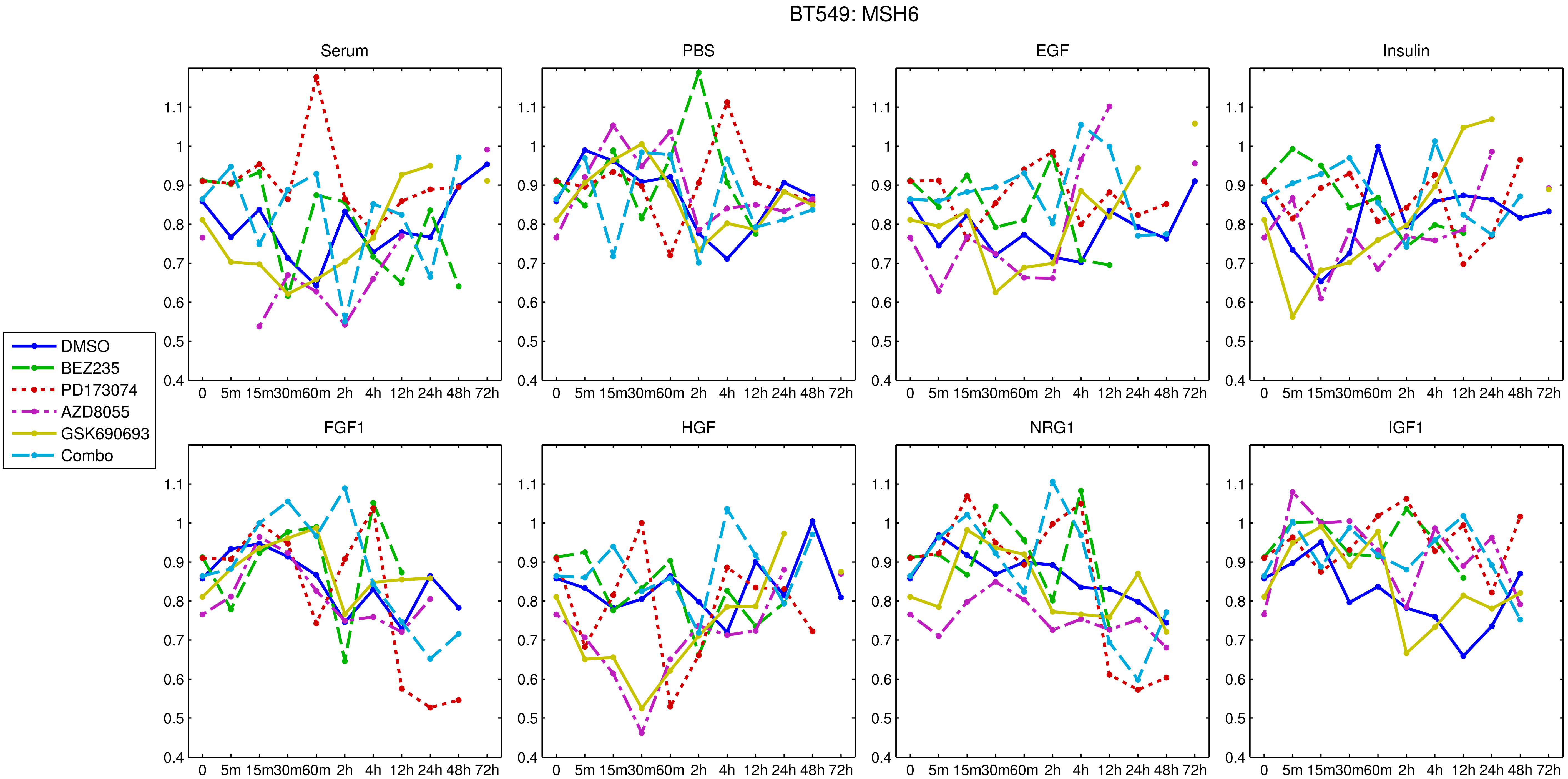
BT549: MSH6
Serum
PBS
EGF
Insulin
FGF1
HGF
NRG1
IGF1
DMSO
BEZ235
PD173074
AZD8055
GSK690693
Combo
0.4
0.5
0.6
0.7
0.8
0.9
1
1.1
0 5m 15m 30m 60m 2h 4h 12h 24h 48h 72h

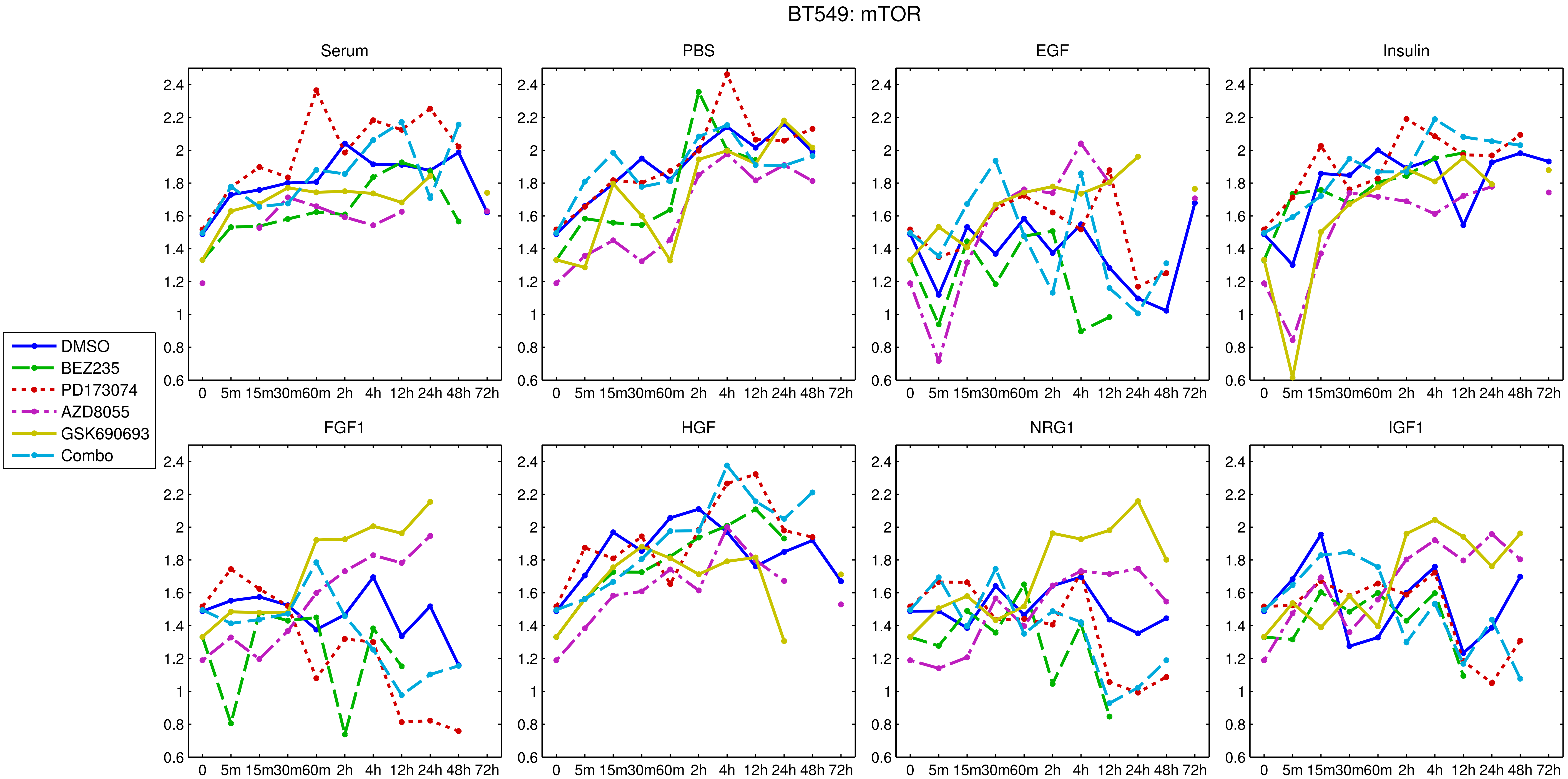

BT549: mTOR
Serum
PBS
EGF
Insulin
FGF1
HGF
NRG1
IGF1
DMSO
BEZ235
PD173074
AZD8055
GSK690693
Combo
0.6
0.8
1
1.2
1.4
1.6
1.8
2
2.2
2.4
0
5m
15m
30m
60m
2h
4h
12h
24h
48h
72h

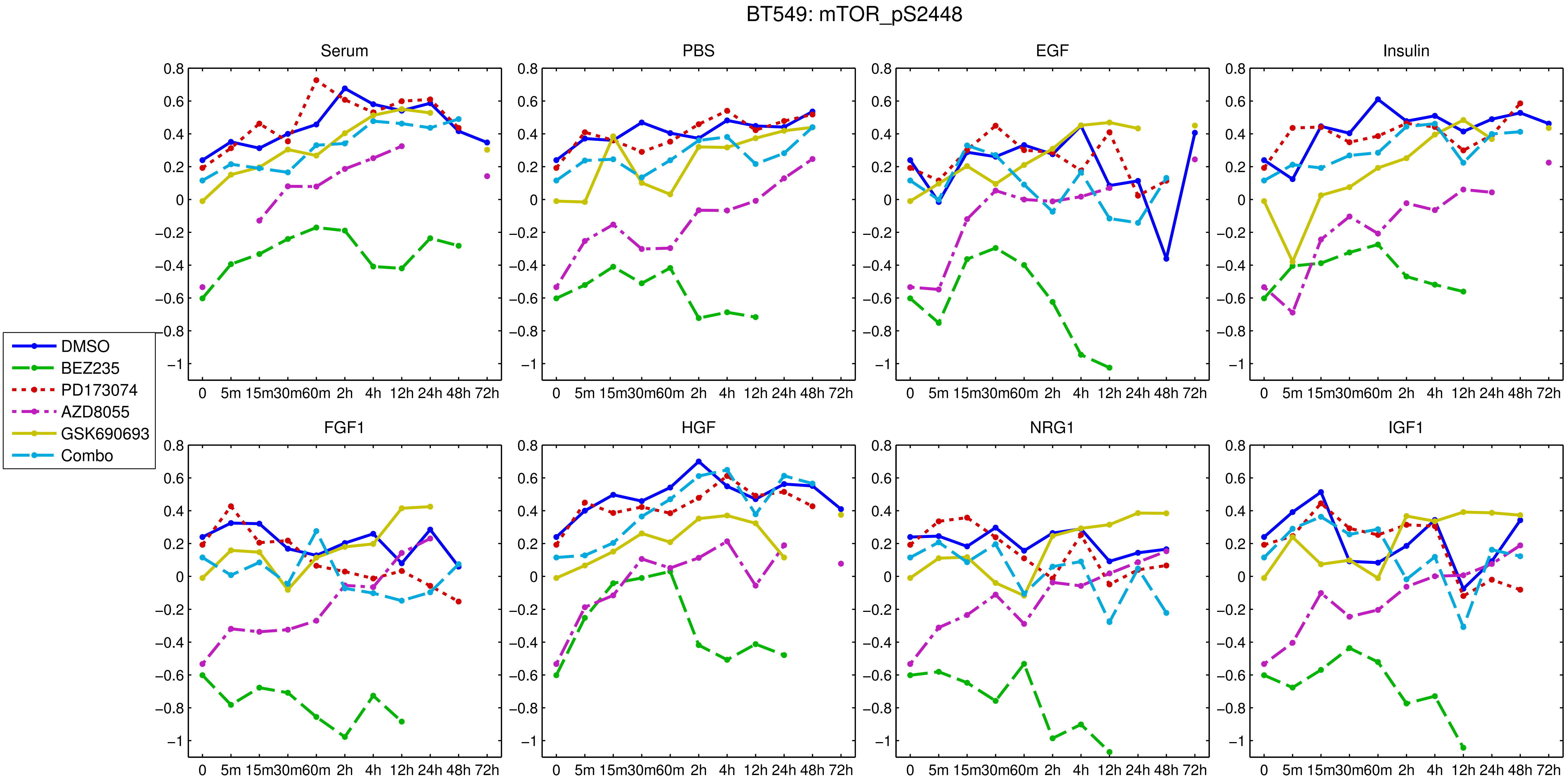

BT549: mTOR_pS2448
Serum
PBS
EGF
Insulin
FGF1
HGF
NRG1
IGF1
DMSO
BEZ235
PD173074
AZD8055
GSK690693
Combo
0 5m 15m 30m 60m 2h 4h 12h 24h 48h 72h

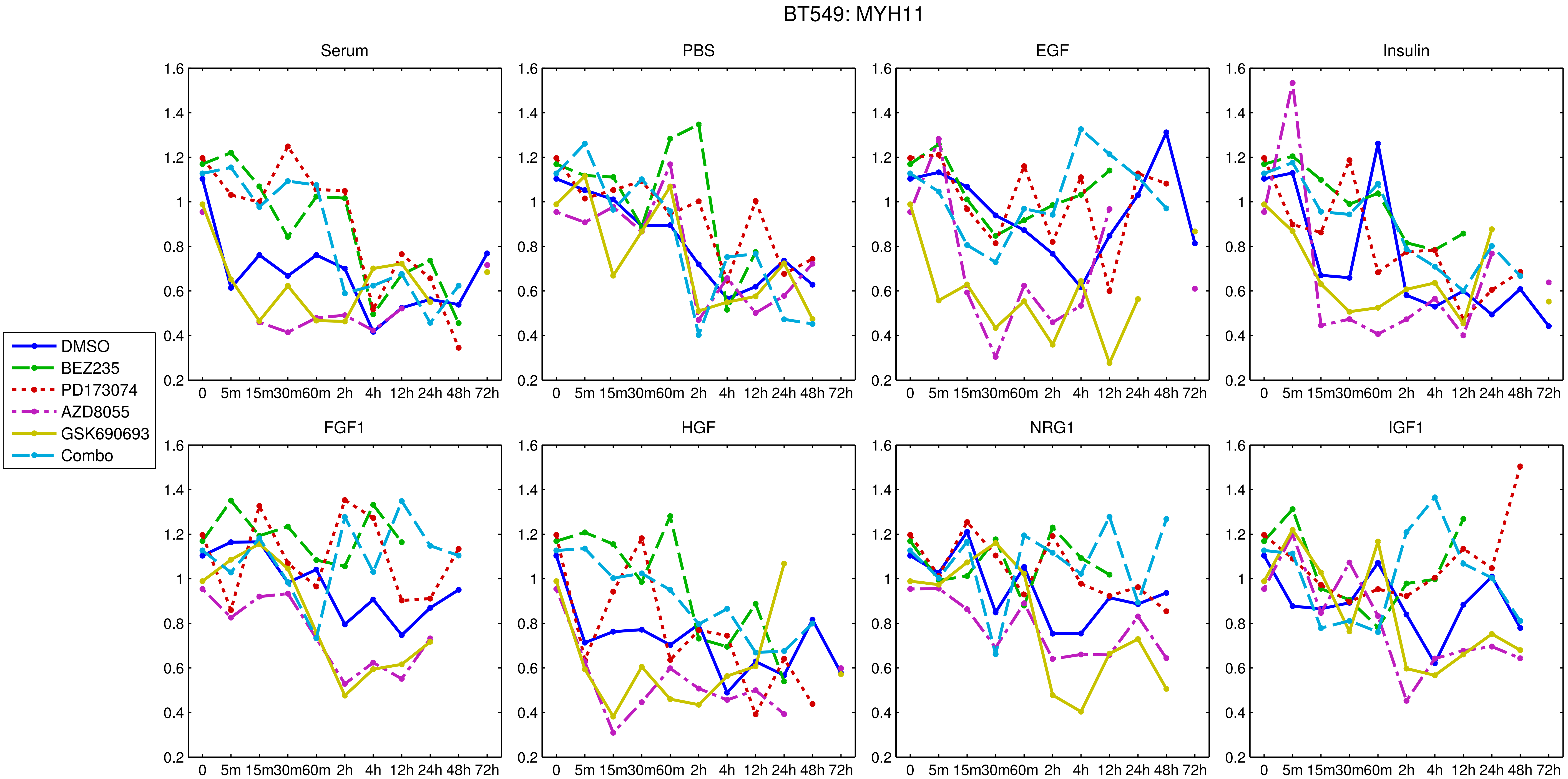
BT549: MYH11
Serum
PBS
EGF
Insulin
FGF1
HGF
NRG1
IGF1
DMSO
BEZ235
PD173074
AZD8055
GSK690693
Combo
0 5m 15m 30m 60m 2h 4h 12h 24h 48h 72h
0.2 0.4 0.6 0.8 1 1.2 1.4 1.6

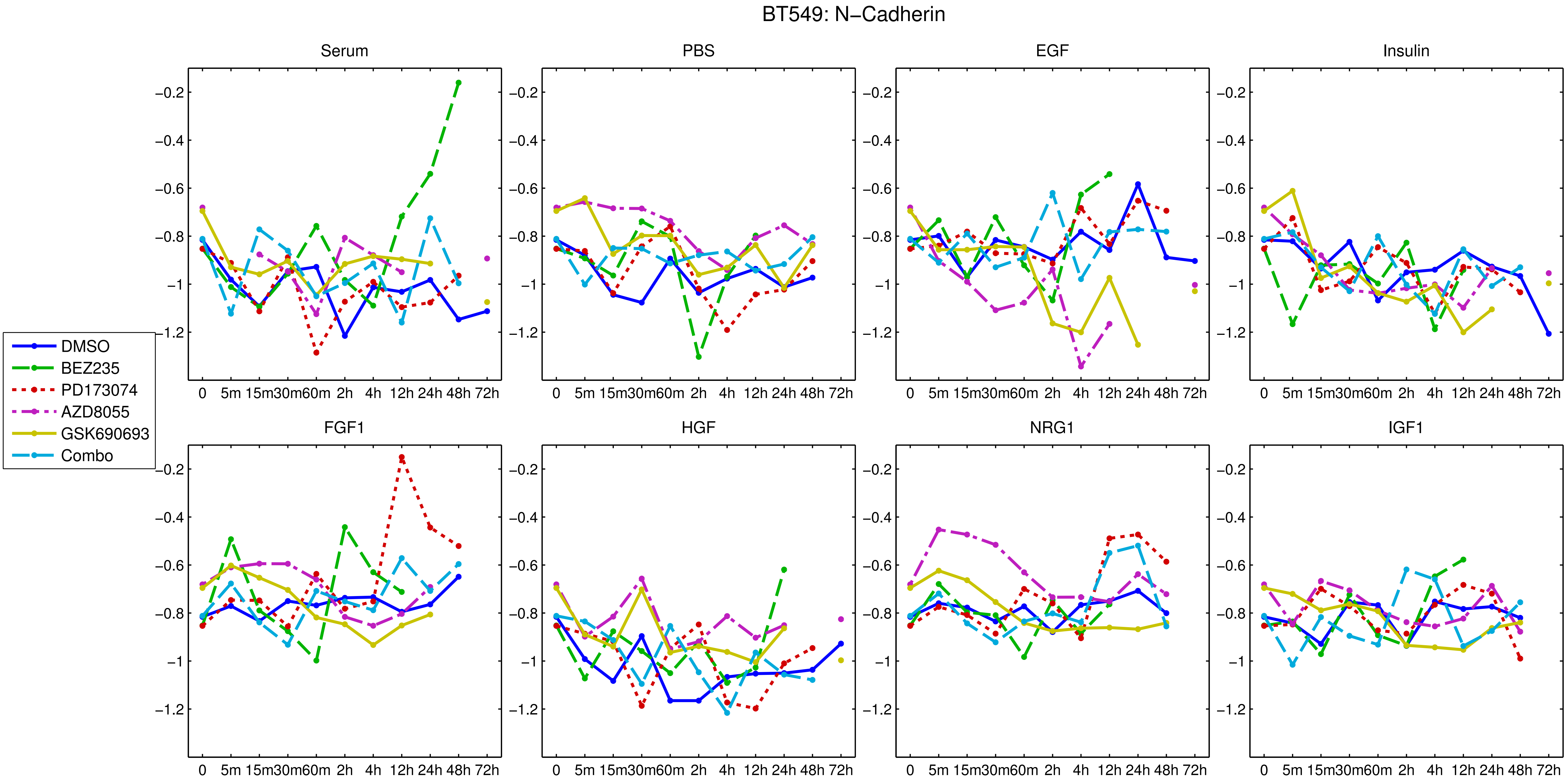
BT549: N−Cadherin
Serum
PBS
EGF
Insulin
FGF1
HGF
NRG1
IGF1
DMSO
BEZ235
PD173074
AZD8055
GSK690693
Combo
−0.2
−0.4
−0.6
−0.8
−1
−1.2
0
5m
15m
30m
60m
2h
4h
12h
24h
48h
72h

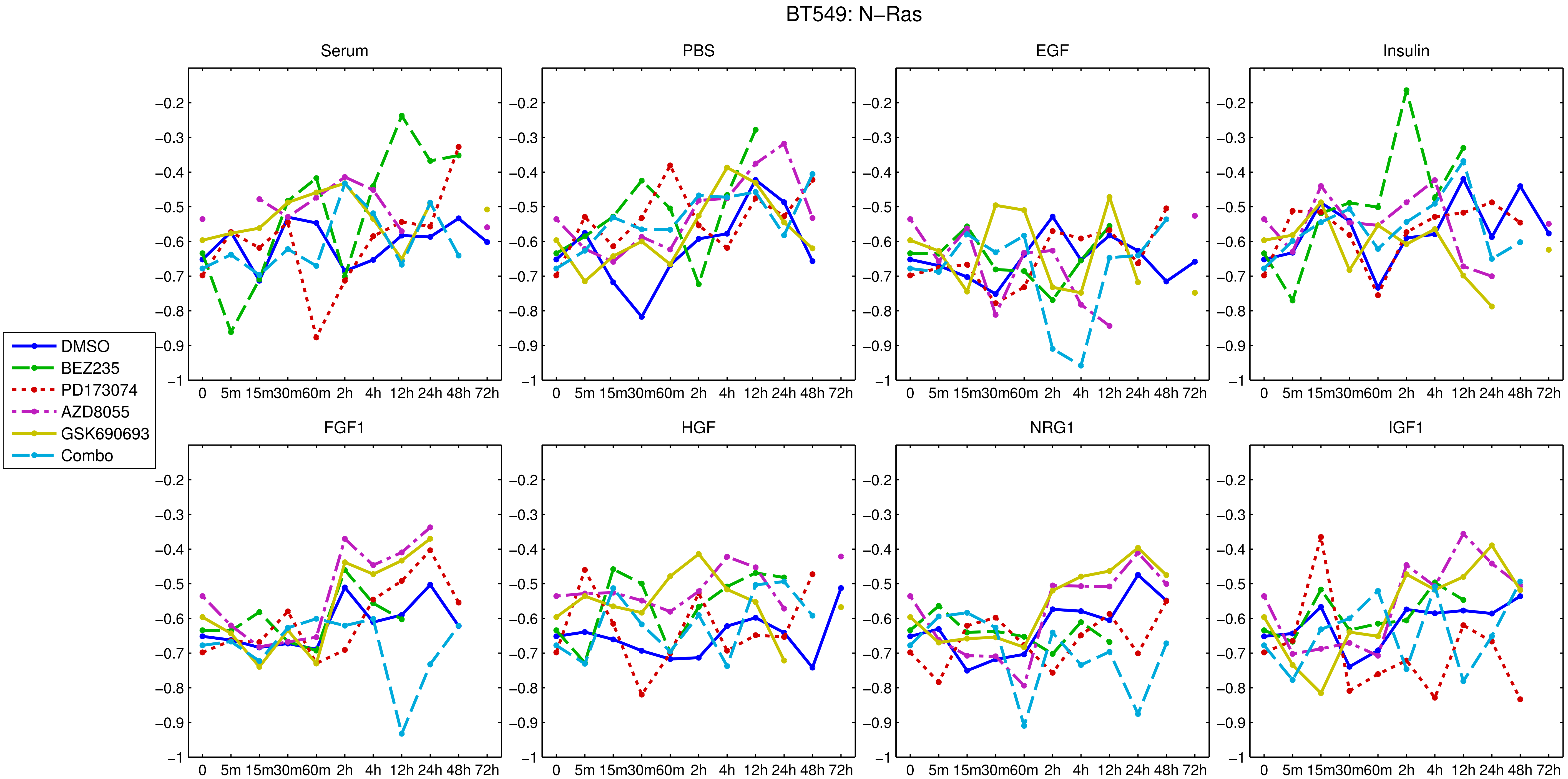
BT549: N−Ras
Serum
PBS
EGF
Insulin
FGF1
HGF
NRG1
IGF1
DMSO
BEZ235
PD173074
AZD8055
GSK690693
Combo
−0.2
−0.3
−0.4
−0.5
−0.6
−0.7
−0.8
−0.9
−1
0
5m
15m
30m
60m
2h
4h
12h
24h
48h
72h

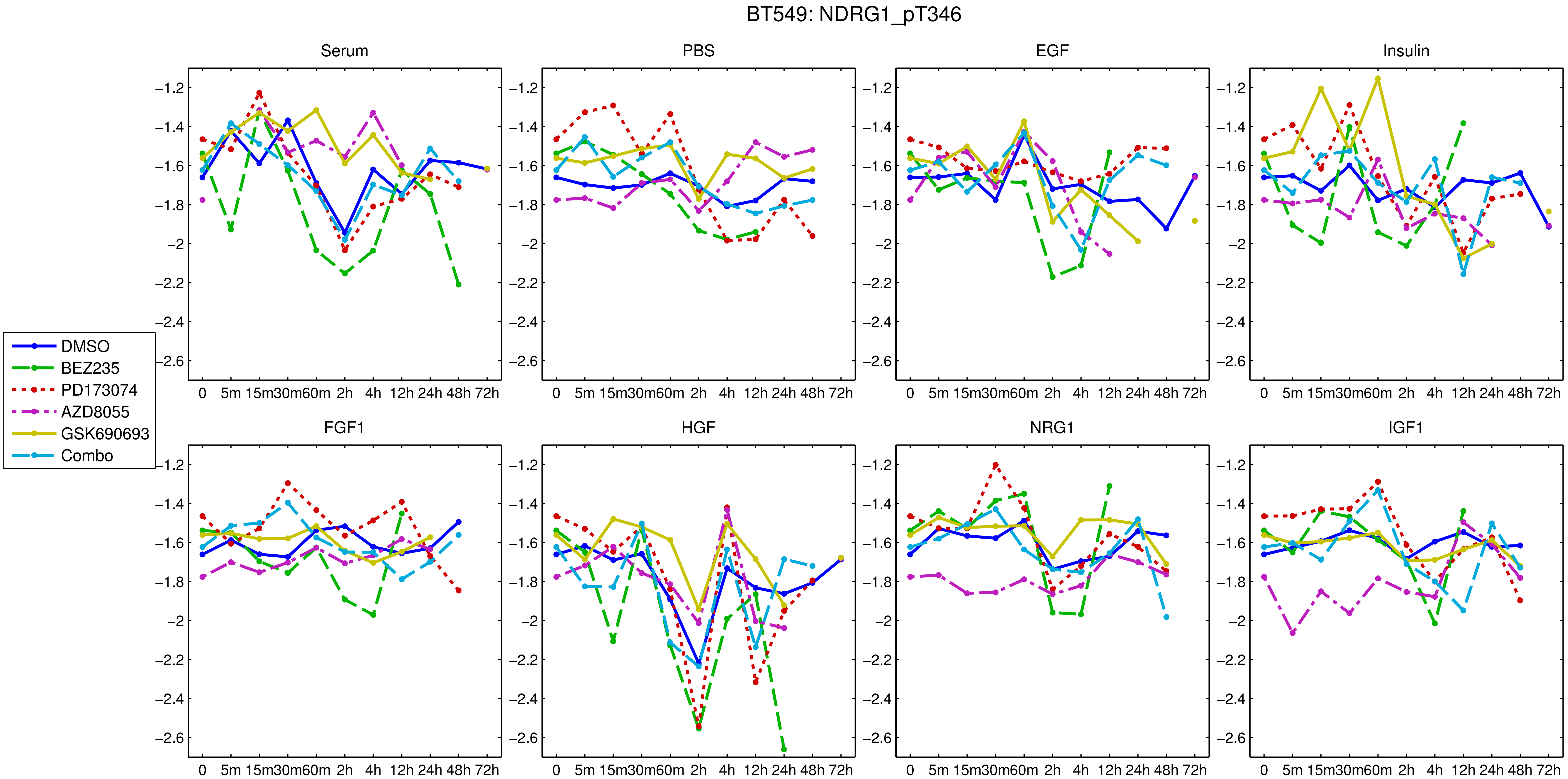
BT549: NDRG1_pT346
Serum
PBS
EGF
Insulin
FGF1
HGF
NRG1
IGF1
DMSO
BEZ235
PD173074
AZD8055
GSK690693
Combo
−1.2
−1.4
−1.6
−1.8
−2
−2.2
−2.4
−2.6
0
5m
15m
30m
60m
2h
4h
12h
24h
48h
72h

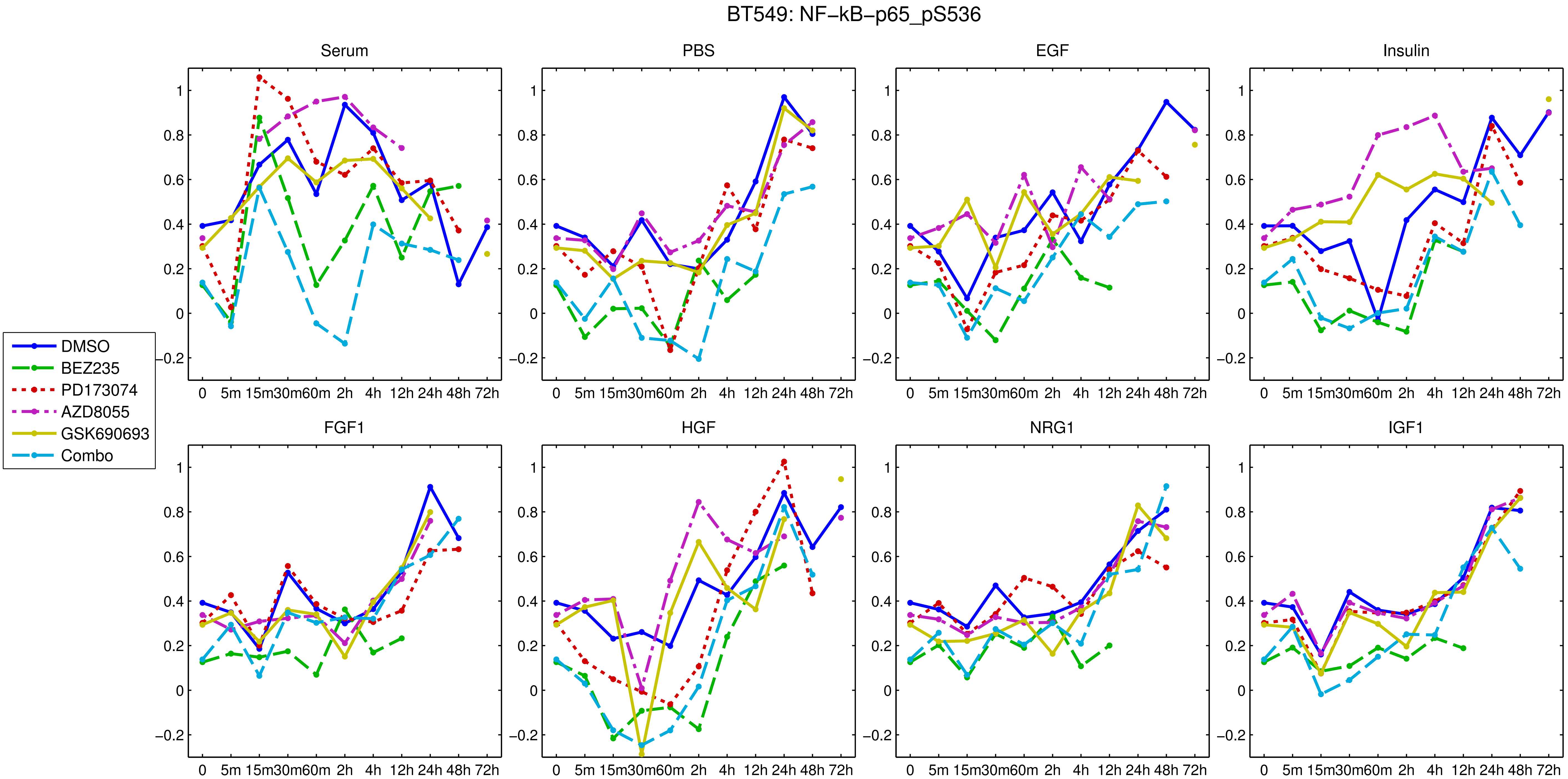
BT549: NF−kB−p65_pS536
Serum
PBS
EGF
Insulin
FGF1
HGF
NRG1
IGF1
DMSO
BEZ235
PD173074
AZD8055
GSK690693
Combo
0
5m
15m
30m
60m
2h
4h
12h
24h
48h
72h
1
0.8
0.6
0.4
0.2
−0.2

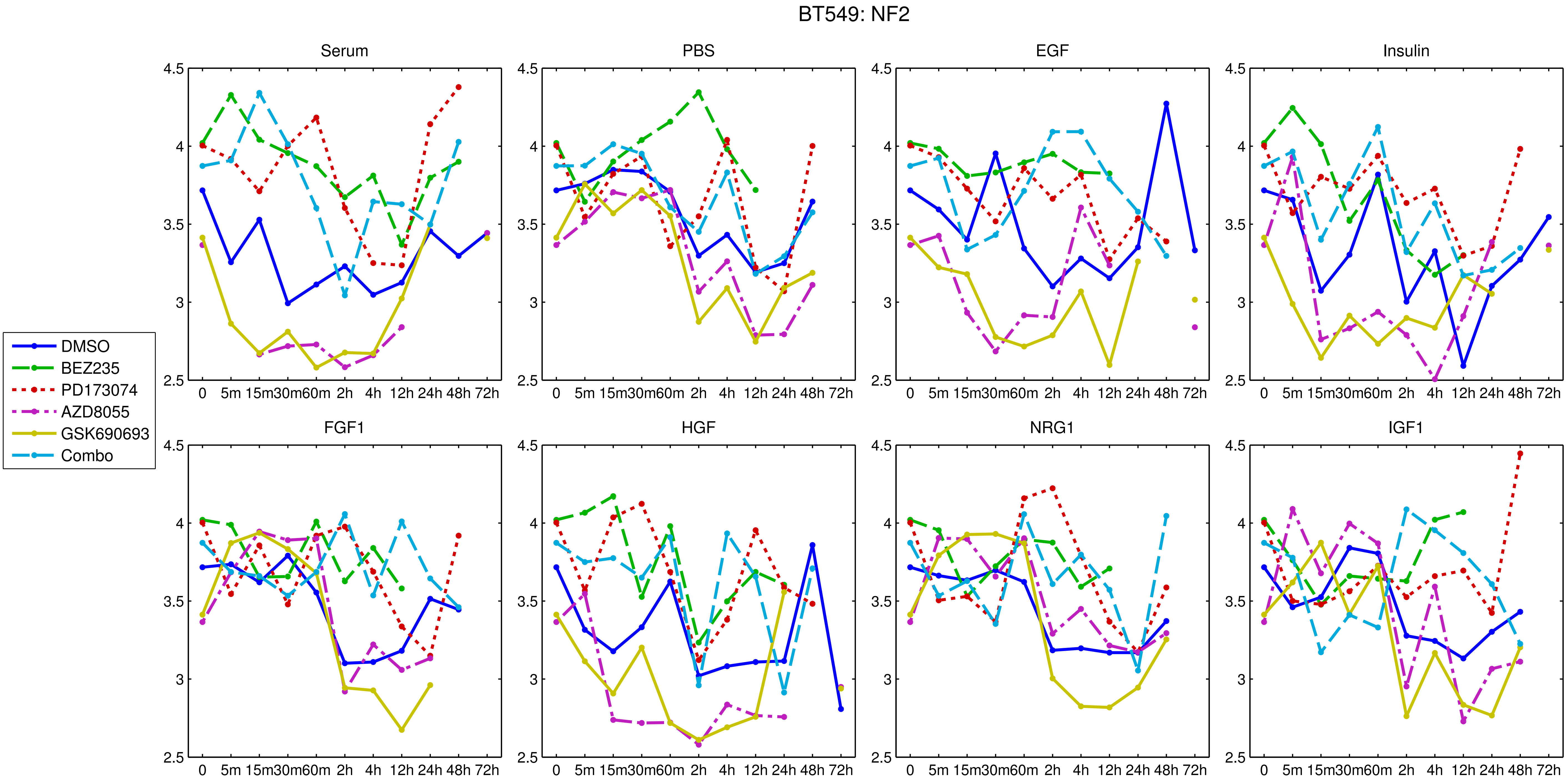
BT549: NF2
Serum
PBS
EGF
Insulin
FGF1
HGF
NRG1
IGF1
DMSO
BEZ235
PD173074
AZD8055
GSK690693
Combo
4.5
4
3.5
3
2.5
0
5m
15m
30m
60m
2h
4h
12h
24h
48h
72h

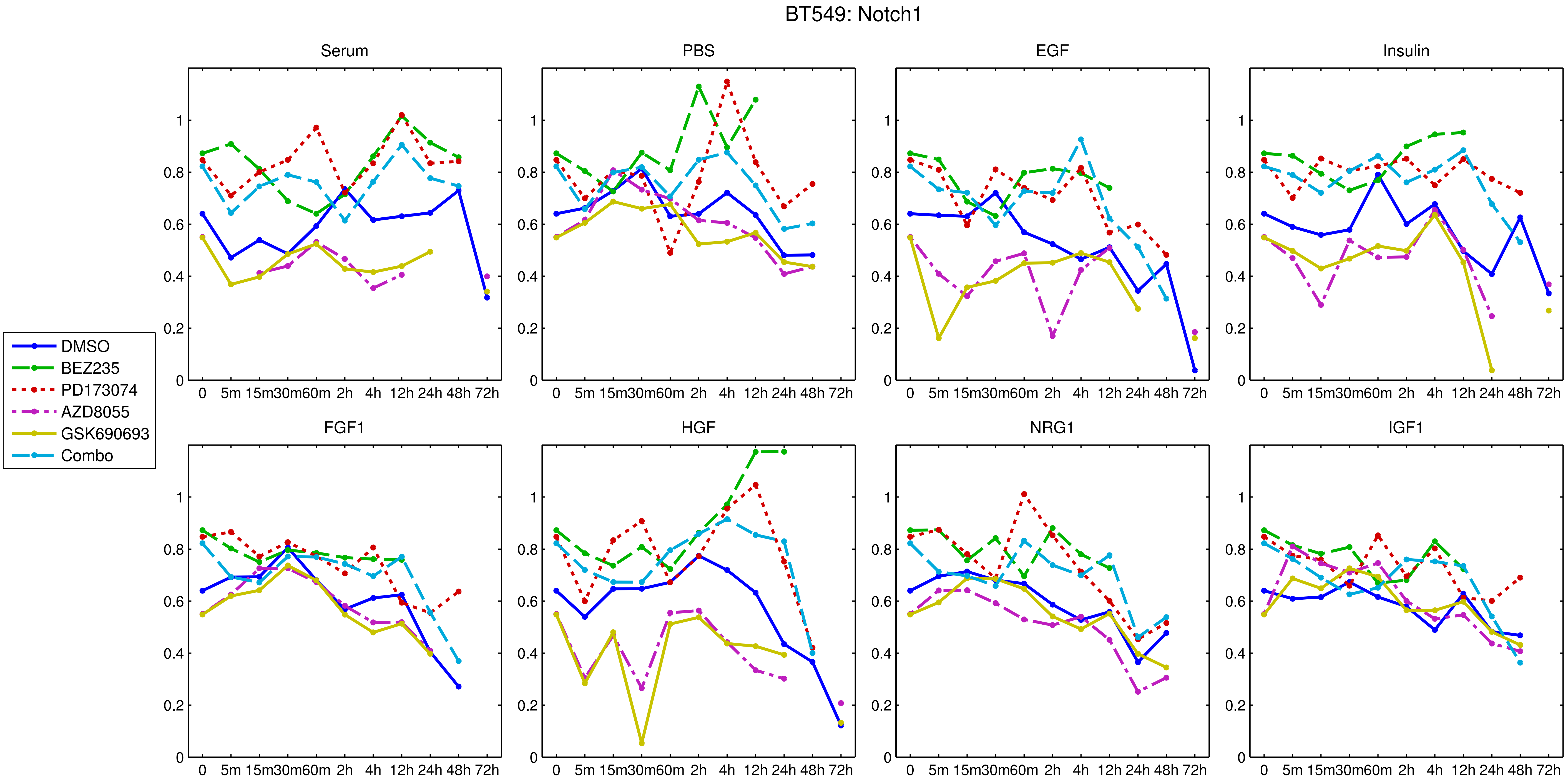
BT549: Notch1
Serum
PBS
EGF
Insulin
FGF1
HGF
NRG1
IGF1
DMSO
BEZ235
PD173074
AZD8055
GSK690693
Combo
0
0.2
0.4
0.6
0.8
1
0 5m 15m30m60m 2h 4h 12h 24h 48h 72h

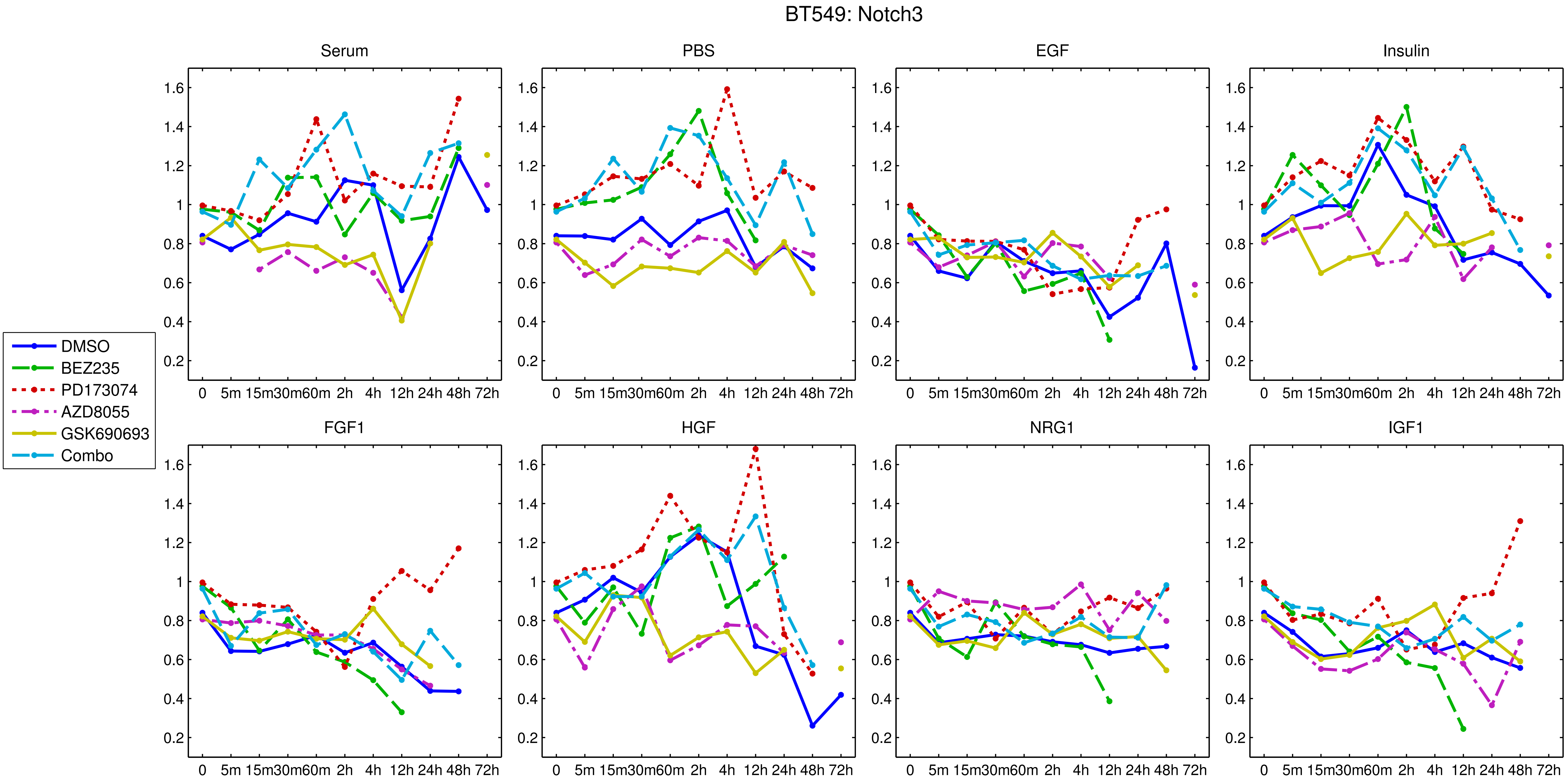
BT549: Notch3
Serum
PBS
EGF
Insulin
FGF1
HGF
NRG1
IGF1
DMSO
BEZ235
PD173074
AZD8055
GSK690693
Combo
0 5m 15m 30m 60m 2h 4h 12h 24h 48h 72h
0.2 0.4 0.6 0.8 1 1.2 1.4 1.6

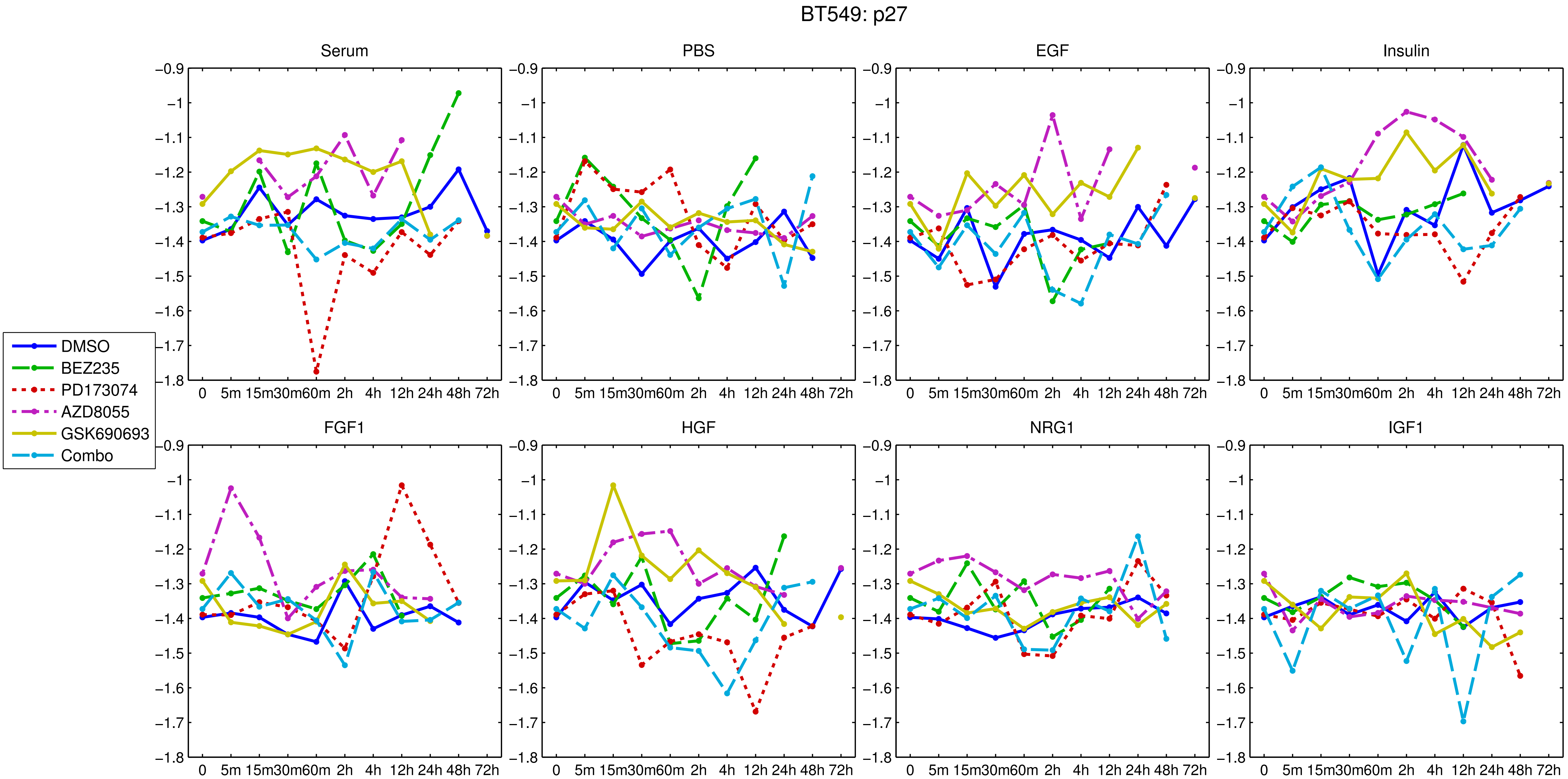
BT549: p27
Serum
PBS
EGF
Insulin
FGF1
HGF
NRG1
IGF1
DMSO
BEZ235
PD173074
AZD8055
GSK690693
Combo
−0.9
−1
−1.1
−1.2
−1.3
−1.4
−1.5
−1.6
−1.7
−1.8
0
5m
15m
30m
60m
2h
4h
12h
24h
48h
72h

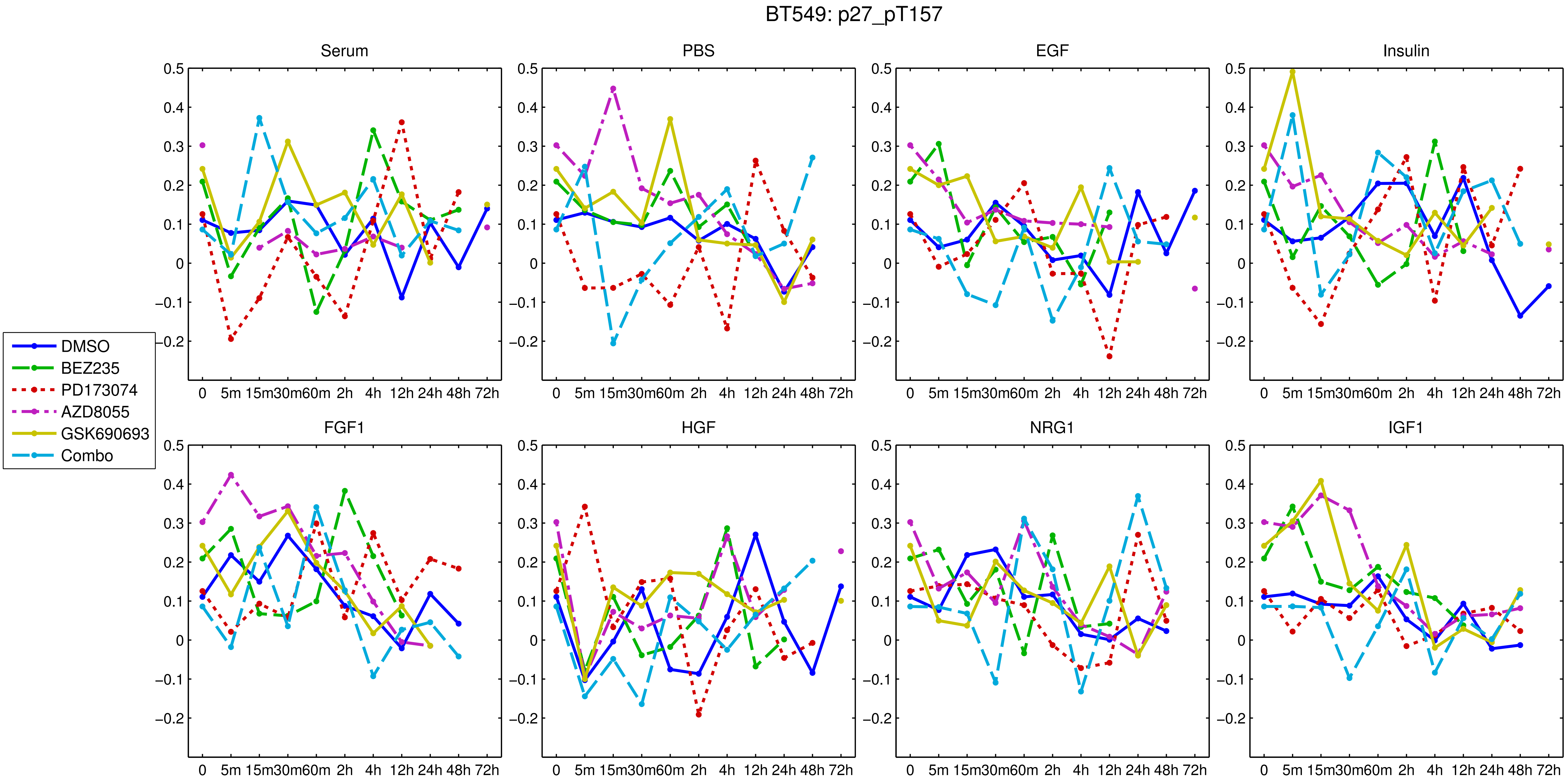
BT549: p27_pT157
Serum
PBS
EGF
Insulin
FGF1
HGF
NRG1
IGF1
DMSO
BEZ235
PD173074
AZD8055
GSK690693
Combo
0.5
0.4
0.3
0.2
0.1
0
−0.1
−0.2
0 5m 15m 30m 60m 2h 4h 12h 24h 48h 72h

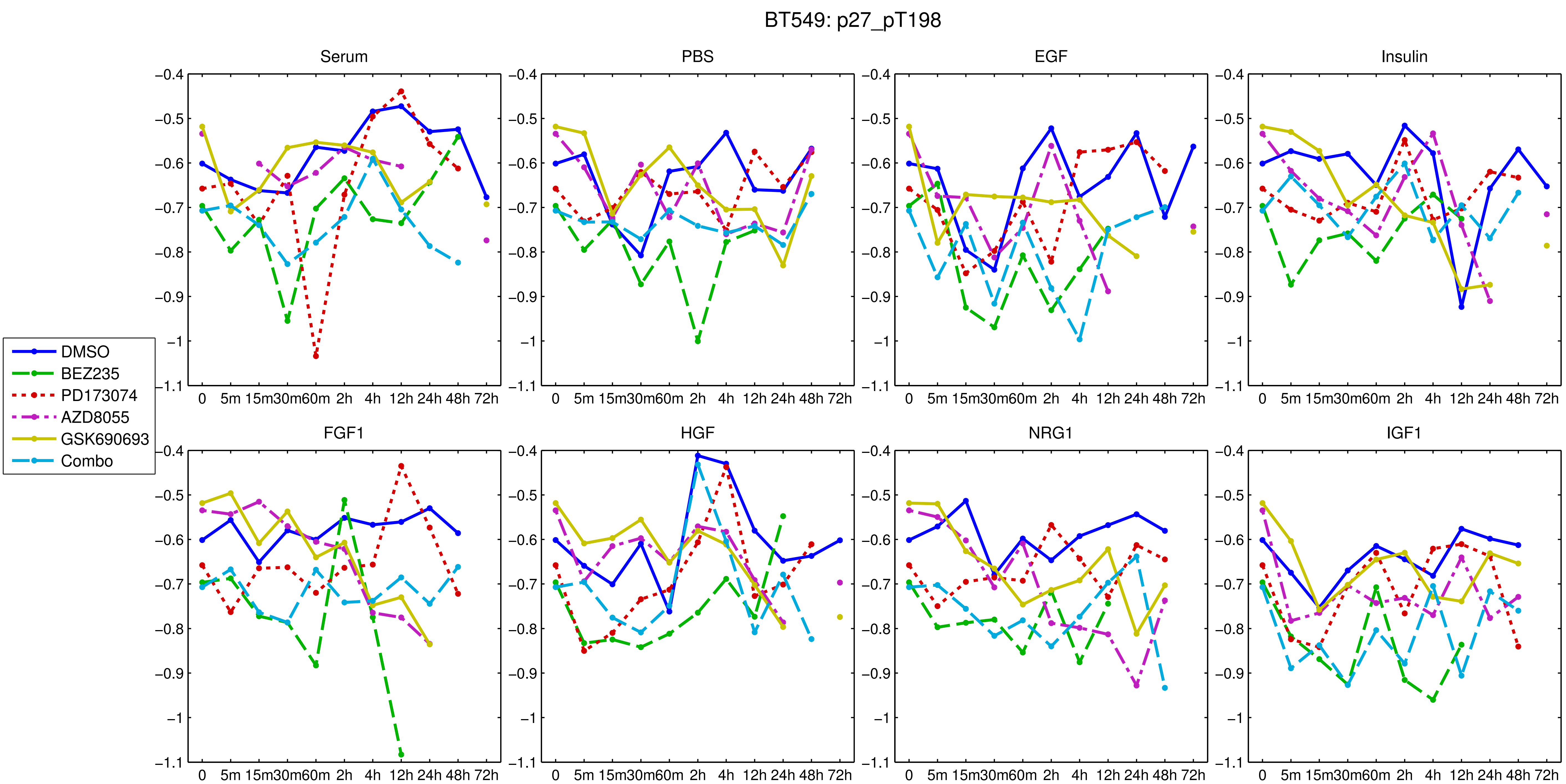
BT549: p27_pT198
Serum
PBS
EGF
Insulin
FGF1
HGF
NRG1
IGF1
DMSO
BEZ235
PD173074
AZD8055
GSK690693
Combo
0 5m 15m 30m 60m 2h 4h 12h 24h 48h 72h
−0.4
−0.5
−0.6
−0.7
−0.8
−0.9
−1
−1.1

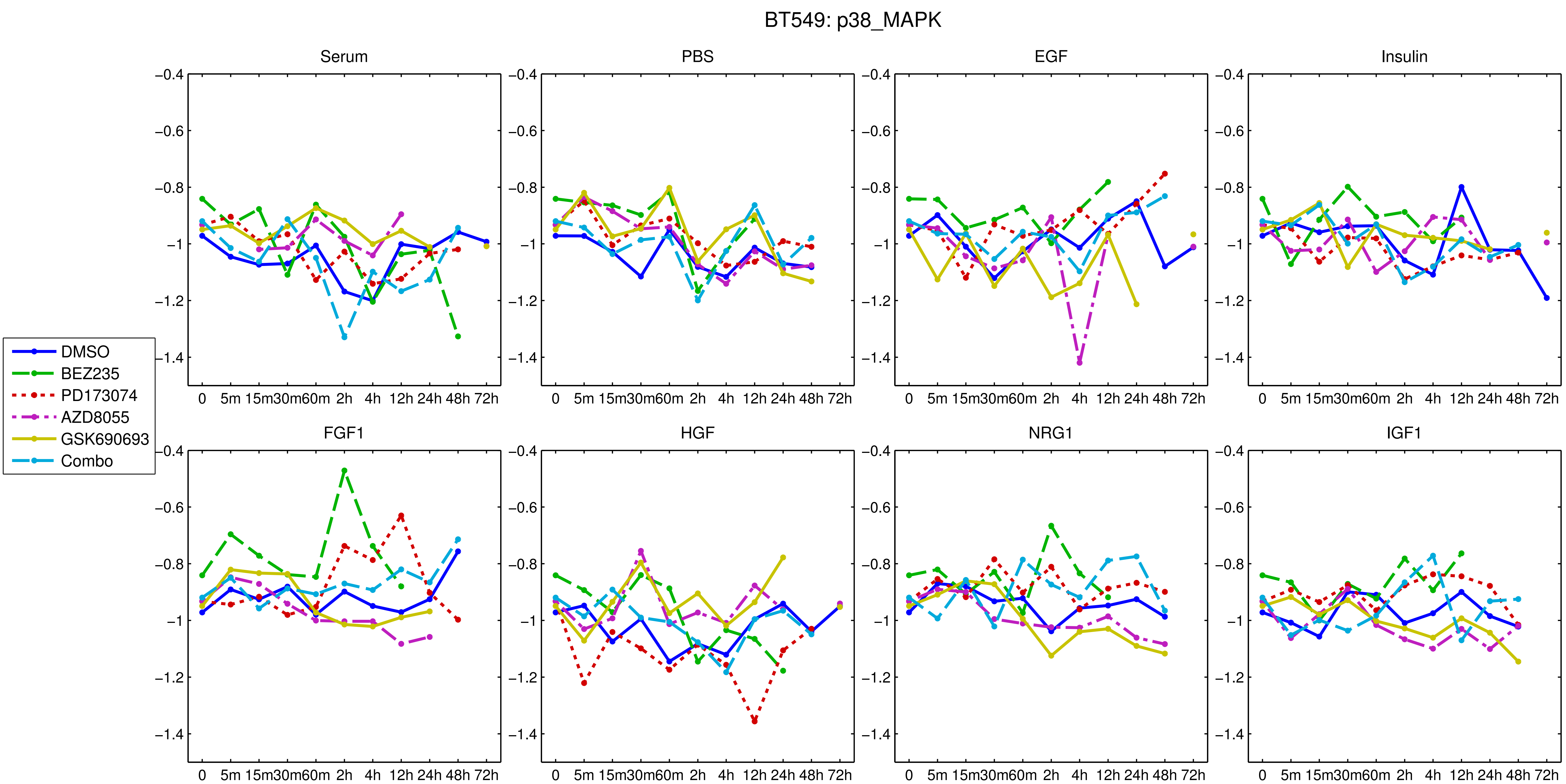
BT549: p38_MAPK
Serum
PBS
EGF
Insulin
FGF1
HGF
NRG1
IGF1
−0.4
−0.6
−0.8
−1
−1.2
−1.4
0
5m
15m
30m
60m
2h
4h
12h
24h
48h
72h
DMSO
BEZ235
PD173074
AZD8055
GSK690693
Combo

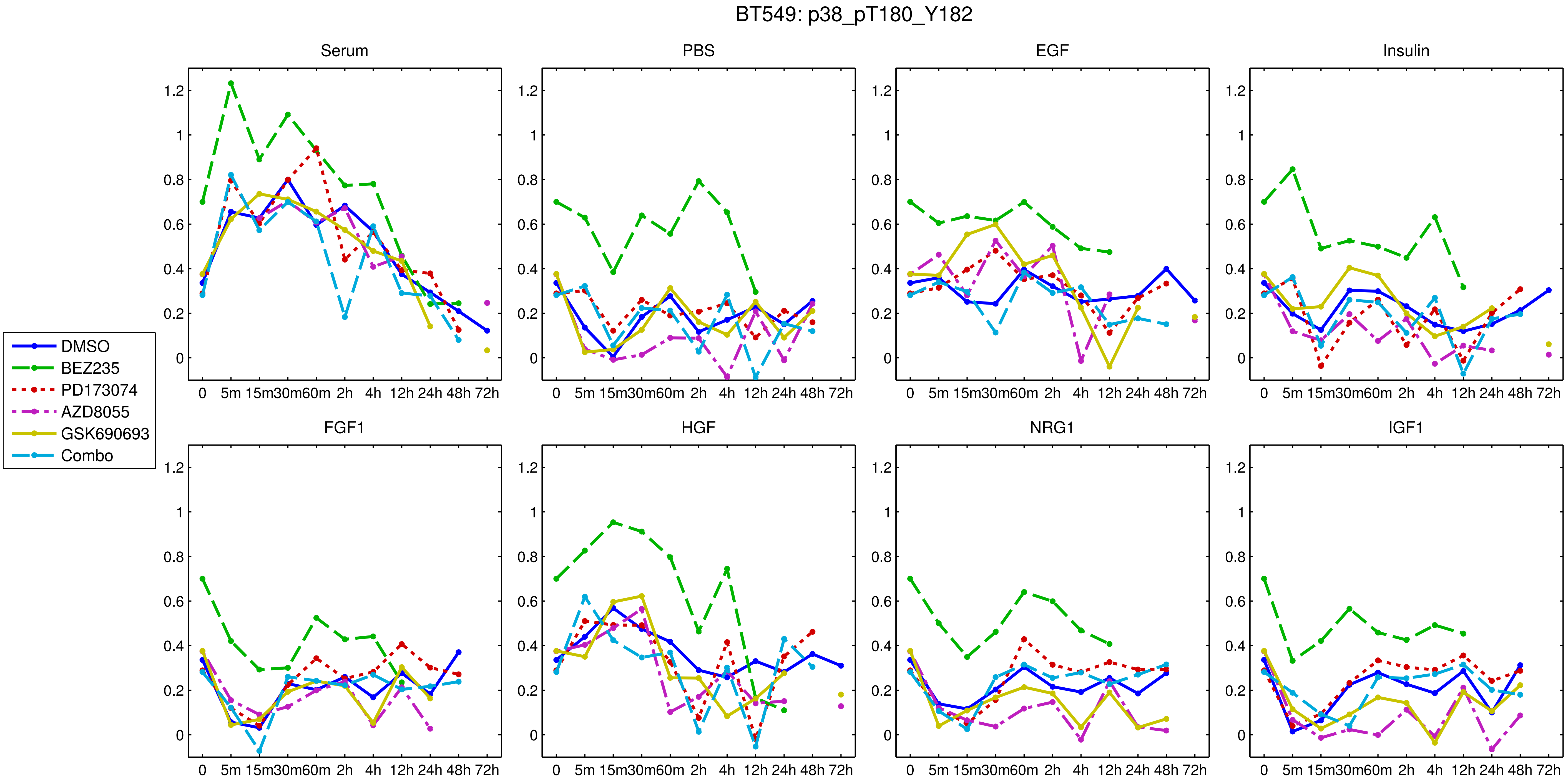

BT549: p38_pT180_Y182
Serum
PBS
EGF
Insulin
FGF1
HGF
NRG1
IGF1
DMSO
BEZ235
PD173074
AZD8055
GSK690693
Combo
0
5m
15m
30m
60m
2h
4h
12h
24h
48h
72h
1.2
1
0.8
0.6
0.4
0.2

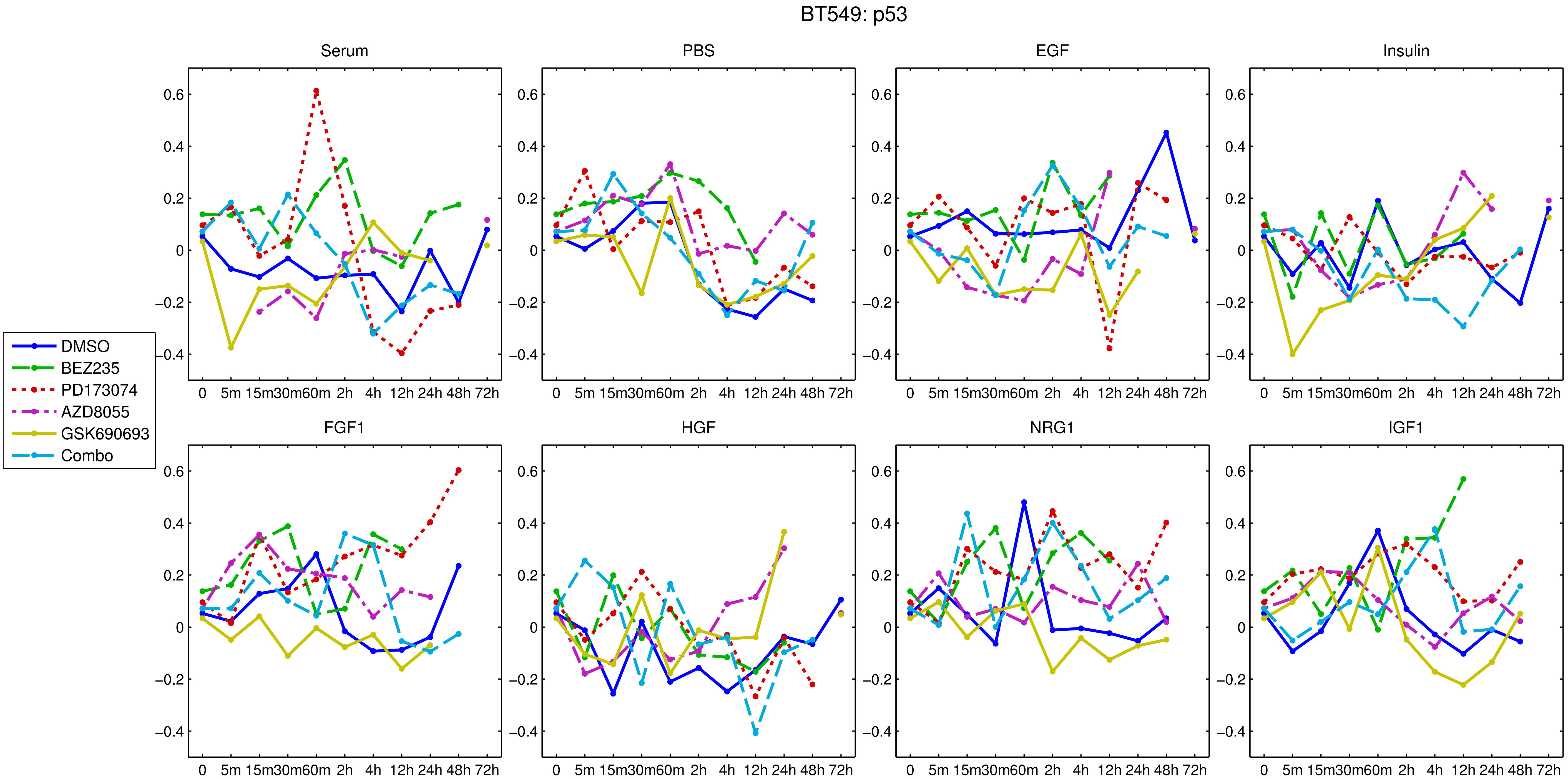
BT549: p53
Serum
PBS
EGF
Insulin
FGF1
HGF
NRG1
IGF1
DMSO
BEZ235
PD173074
AZD8055
GSK690693
Combo
0.6
0.4
0.2
0
−0.2
−0.4
0
5m
15m
30m
60m
2h
4h
12h
24h
48h
72h

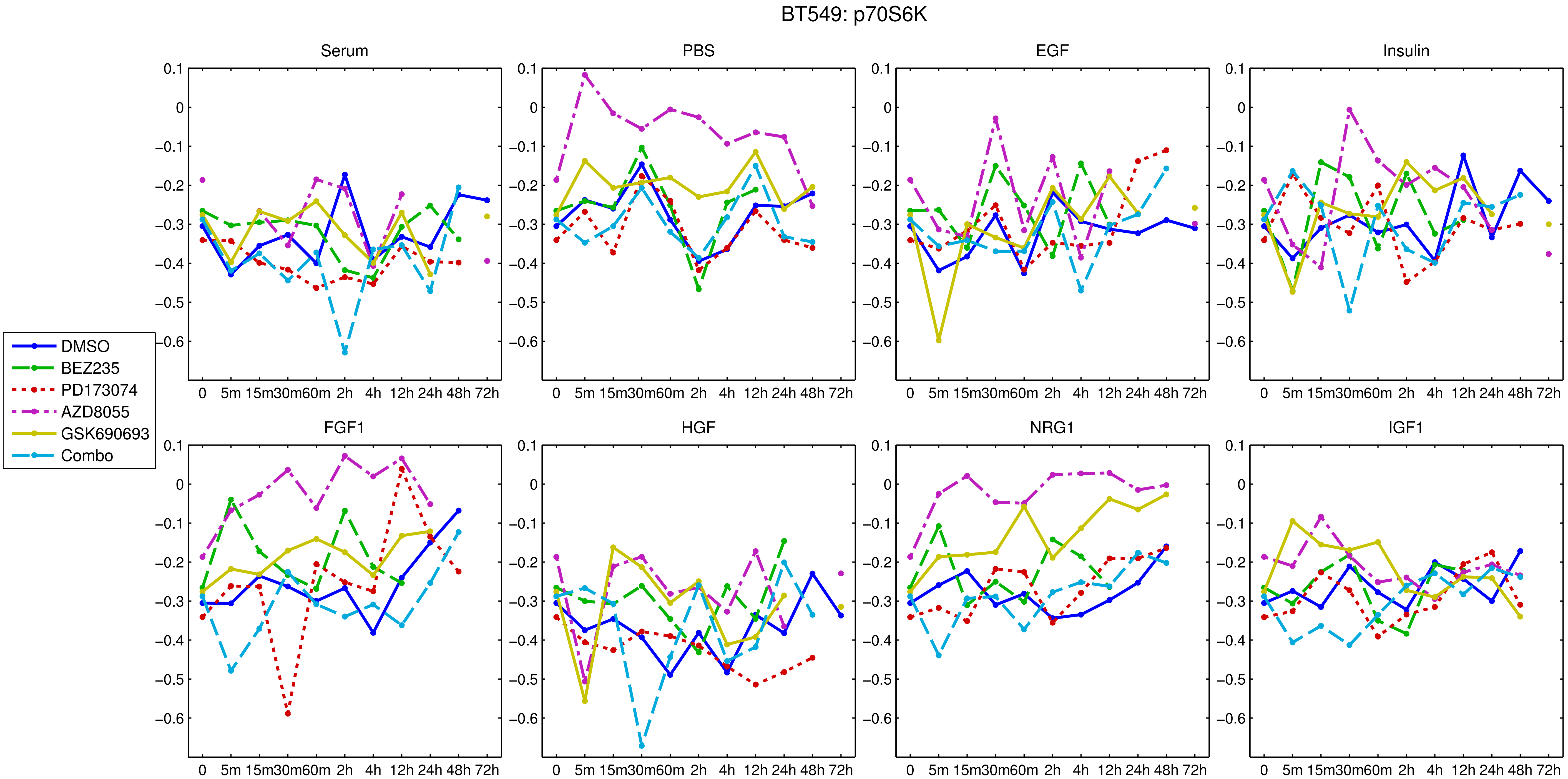
BT549: p70S6K
Serum
PBS
EGF
Insulin
FGF1
HGF
NRG1
IGF1
DMSO
BEZ235
PD173074
AZD8055
GSK690693
Combo
0.1
0
−0.1
−0.2
−0.3
−0.4
−0.5
−0.6
0
5m
15m
30m
60m
2h
4h
12h
24h
48h
72h

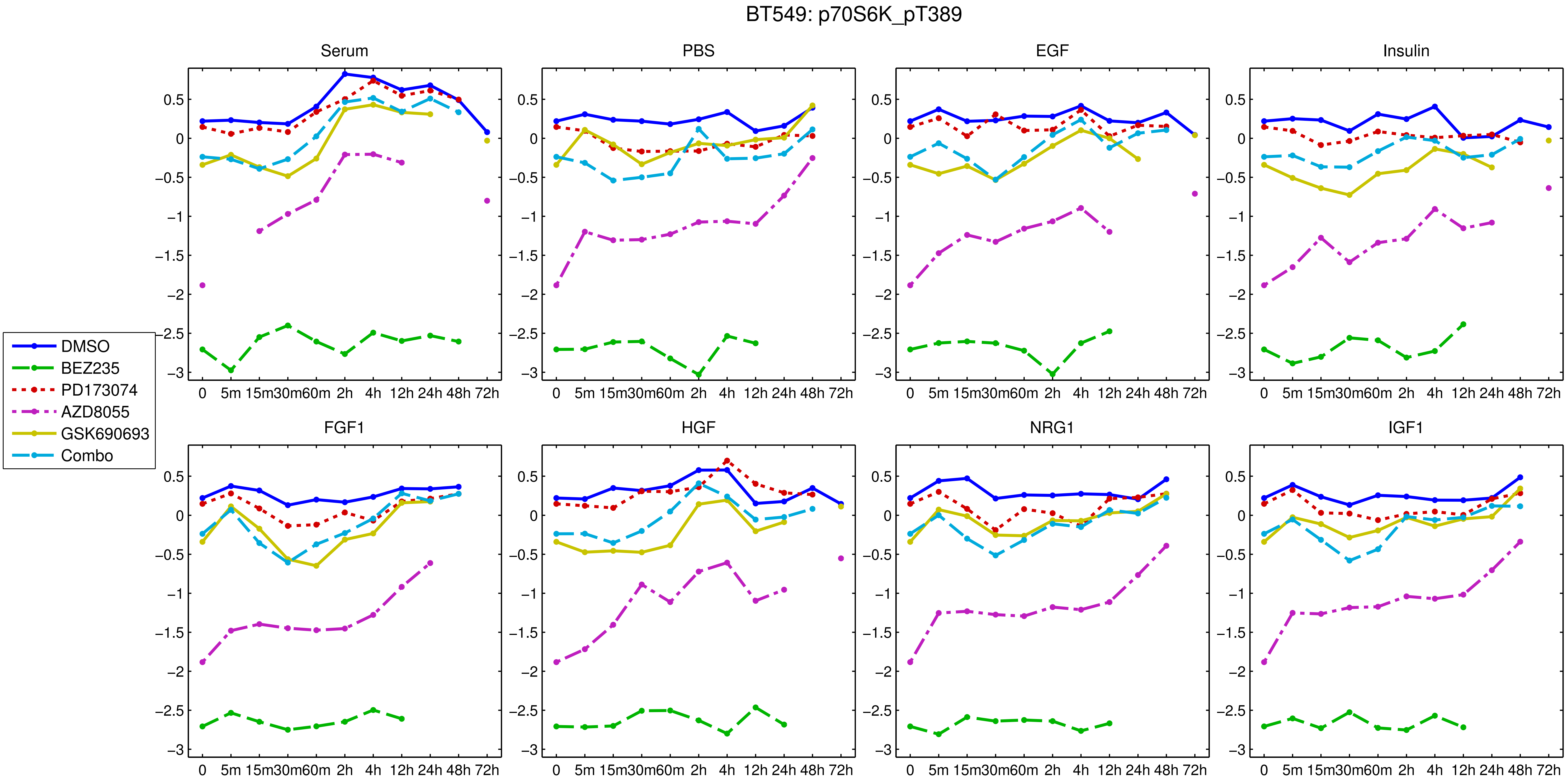

BT549: p70S6K_pT389
Serum
PBS
EGF
Insulin
FGF1
HGF
NRG1
IGF1
0.5
0
−0.5
−1
−1.5
−2
−2.5
−3
0
5m
15m
30m
60m
2h
4h
12h
24h
48h
72h
DMSO
BEZ235
PD173074
AZD8055
GSK690693
Combo

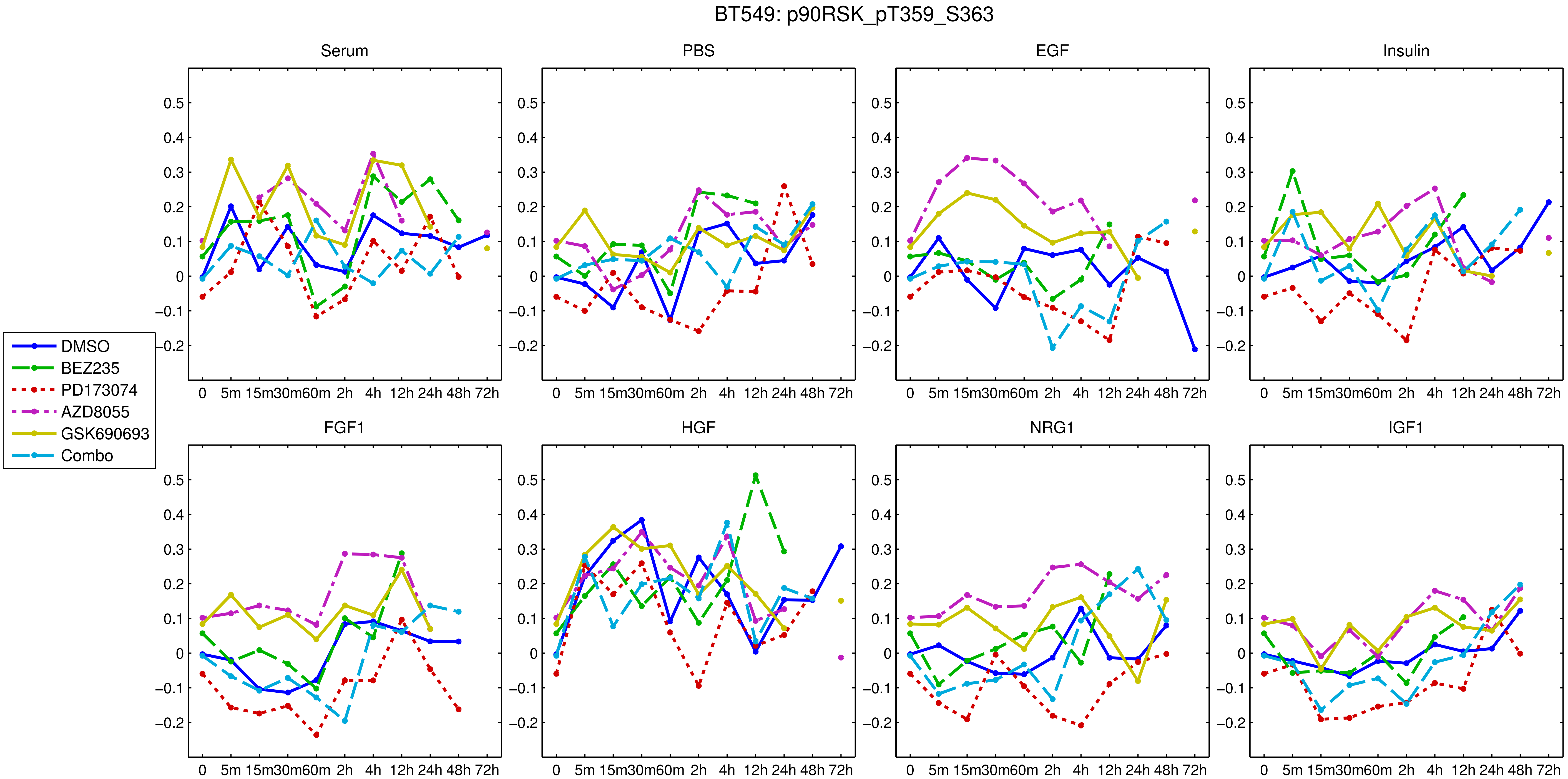
BT549: p90RSK_pT359_S363
Serum
PBS
EGF
Insulin
FGF1
HGF
NRG1
IGF1
0.5
0.4
0.3
0.2
0.1
0
−0.1
−0.2
0 5m 15m 30m 60m 2h 4h 12h 24h 48h 72h
DMSO
BEZ235
PD173074
AZD8055
GSK690693
Combo

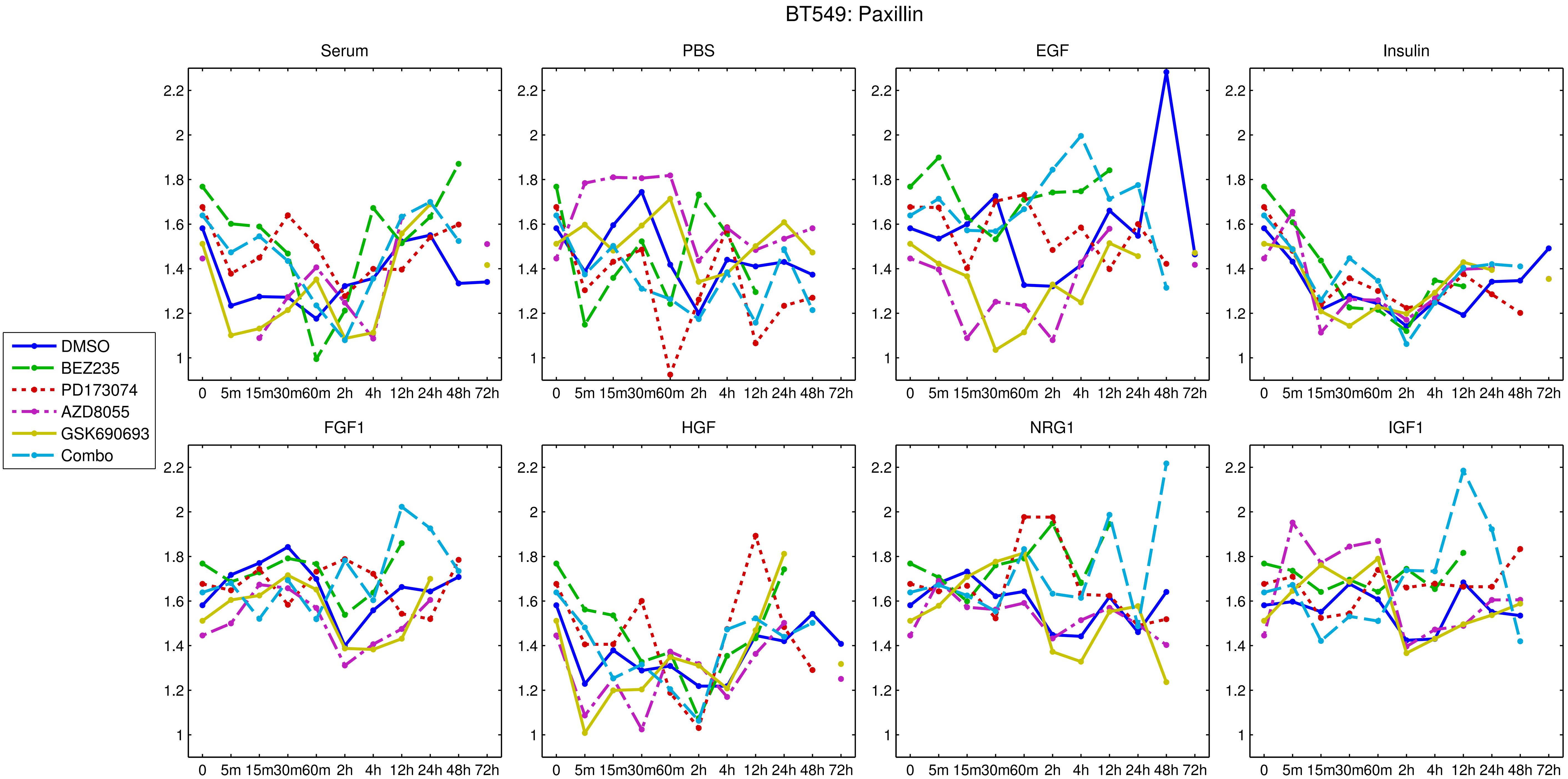

BT549: Paxillin
Serum
PBS
EGF
Insulin
FGF1
HGF
NRG1
IGF1
DMSO
BEZ235
PD173074
AZD8055
GSK690693
Combo
2.2
2
1.8
1.6
1.4
1.2
1
0
5m
15m
30m
60m
2h
4h
12h
24h
48h
72h

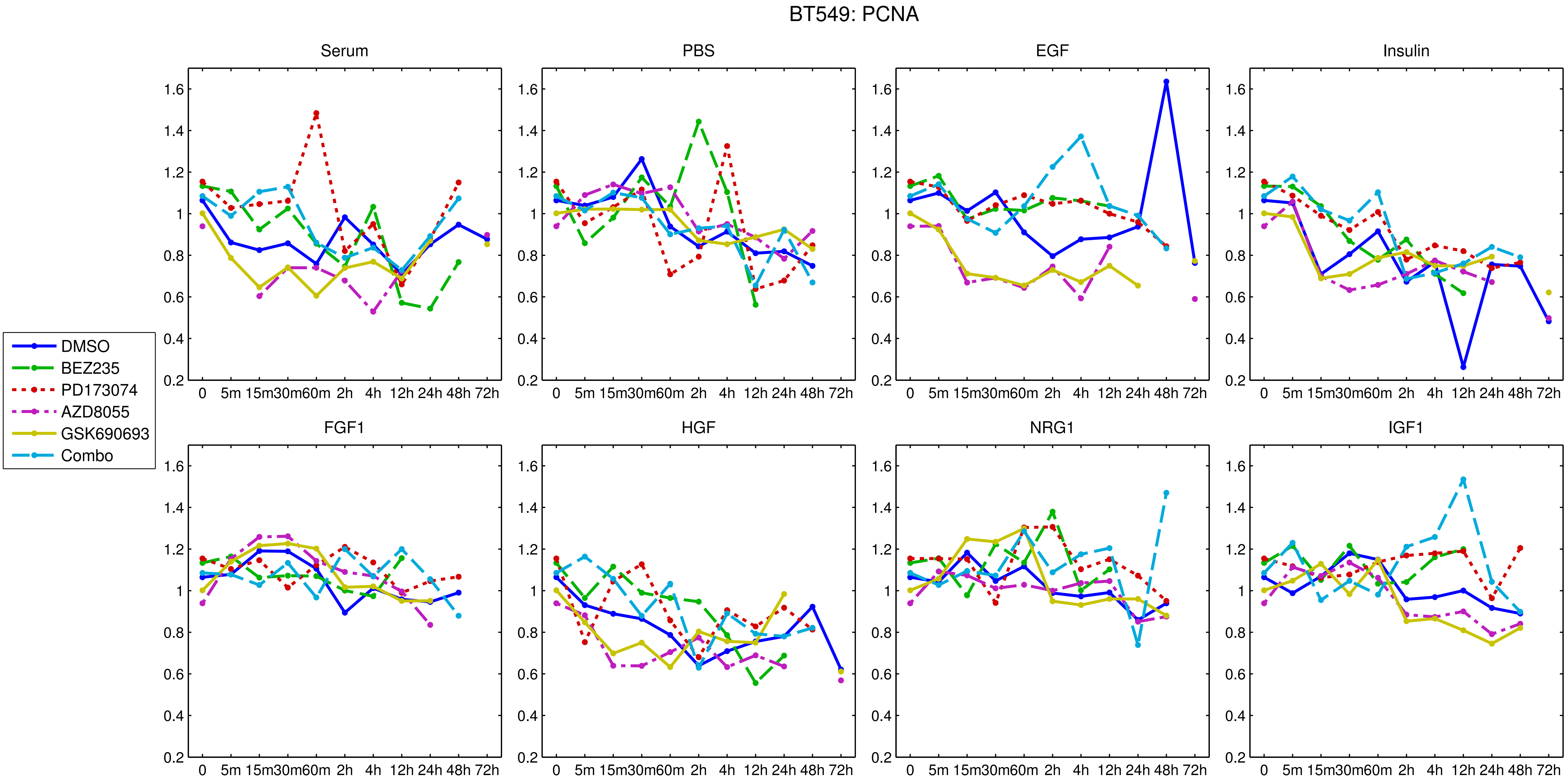
BT549: PCNA
Serum
PBS
EGF
Insulin
FGF1
HGF
NRG1
IGF1
DMSO
BEZ235
PD173074
AZD8055
GSK690693
Combo
0.2
0.4
0.6
0.8
1
1.2
1.4
1.6
0
5m
15m
30m
60m
2h
4h
12h
24h
48h
72h

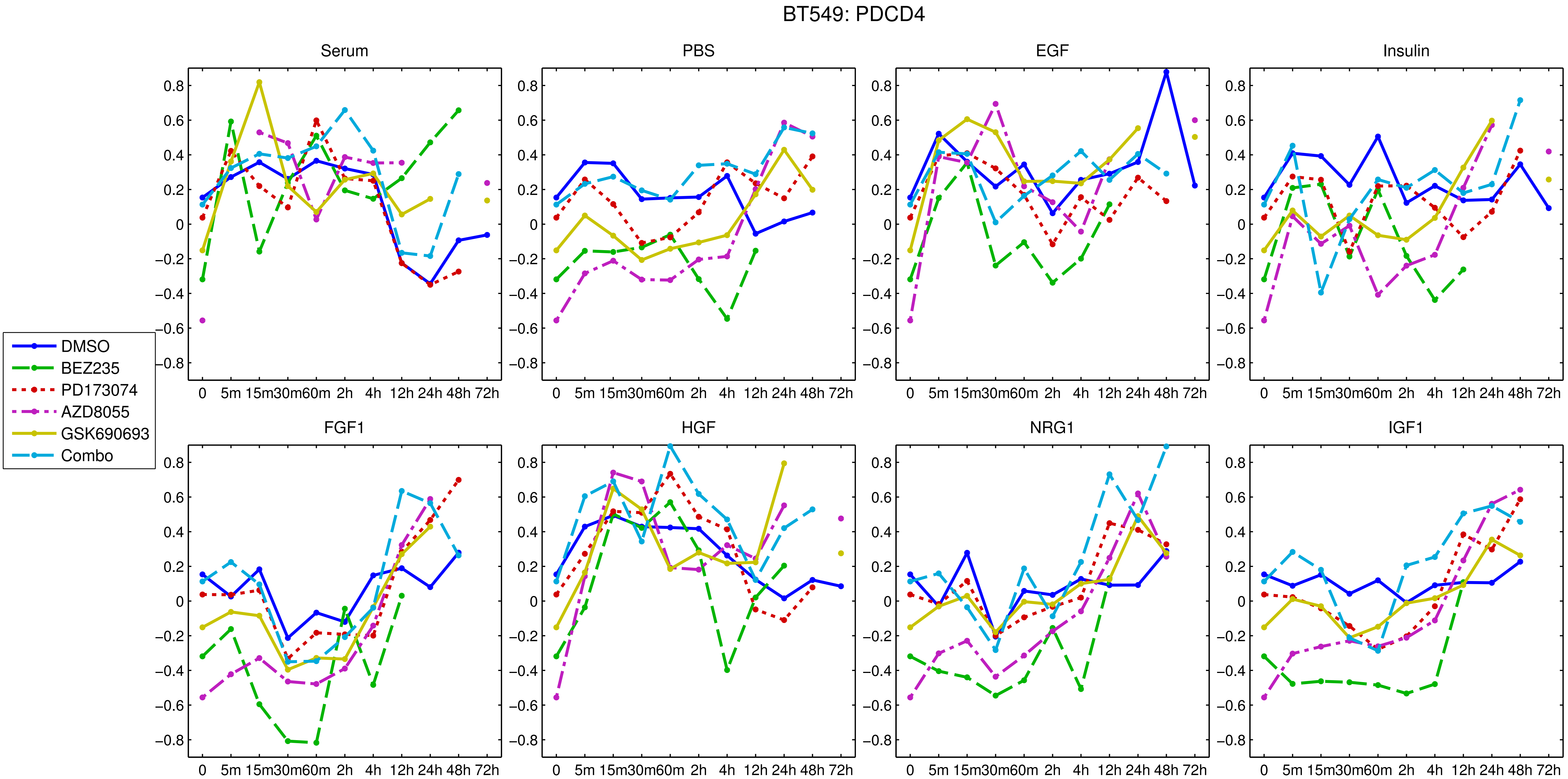
BT549: PDCD4
Serum
PBS
EGF
Insulin
FGF1
HGF
NRG1
IGF1
0.8
0.6
0.4
0.2
0
−0.2
−0.4
−0.6
−0.8
0
5m
15m
30m
60m
2h
4h
12h
24h
48h
72h
DMSO
BEZ235
PD173074
AZD8055
GSK690693
Combo

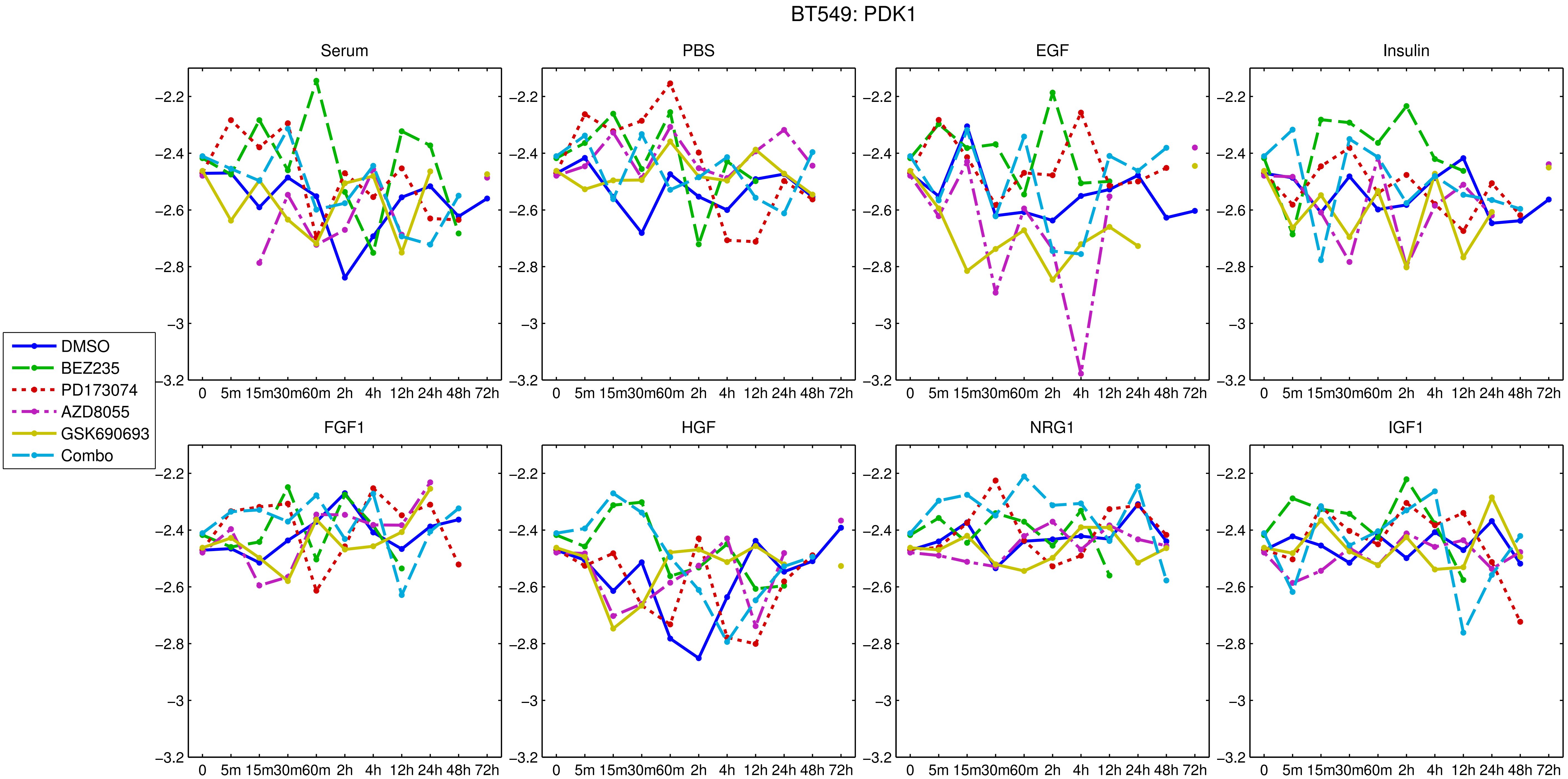

BT549: PDK1
Serum
PBS
EGF
Insulin
FGF1
HGF
NRG1
IGF1
−2.2
−2.4
−2.6
−2.8
−3
−3.2
0
5m
15m
30m
60m
2h
4h
12h
24h
48h
72h
DMSO
BEZ235
PD173074
AZD8055
GSK690693
Combo

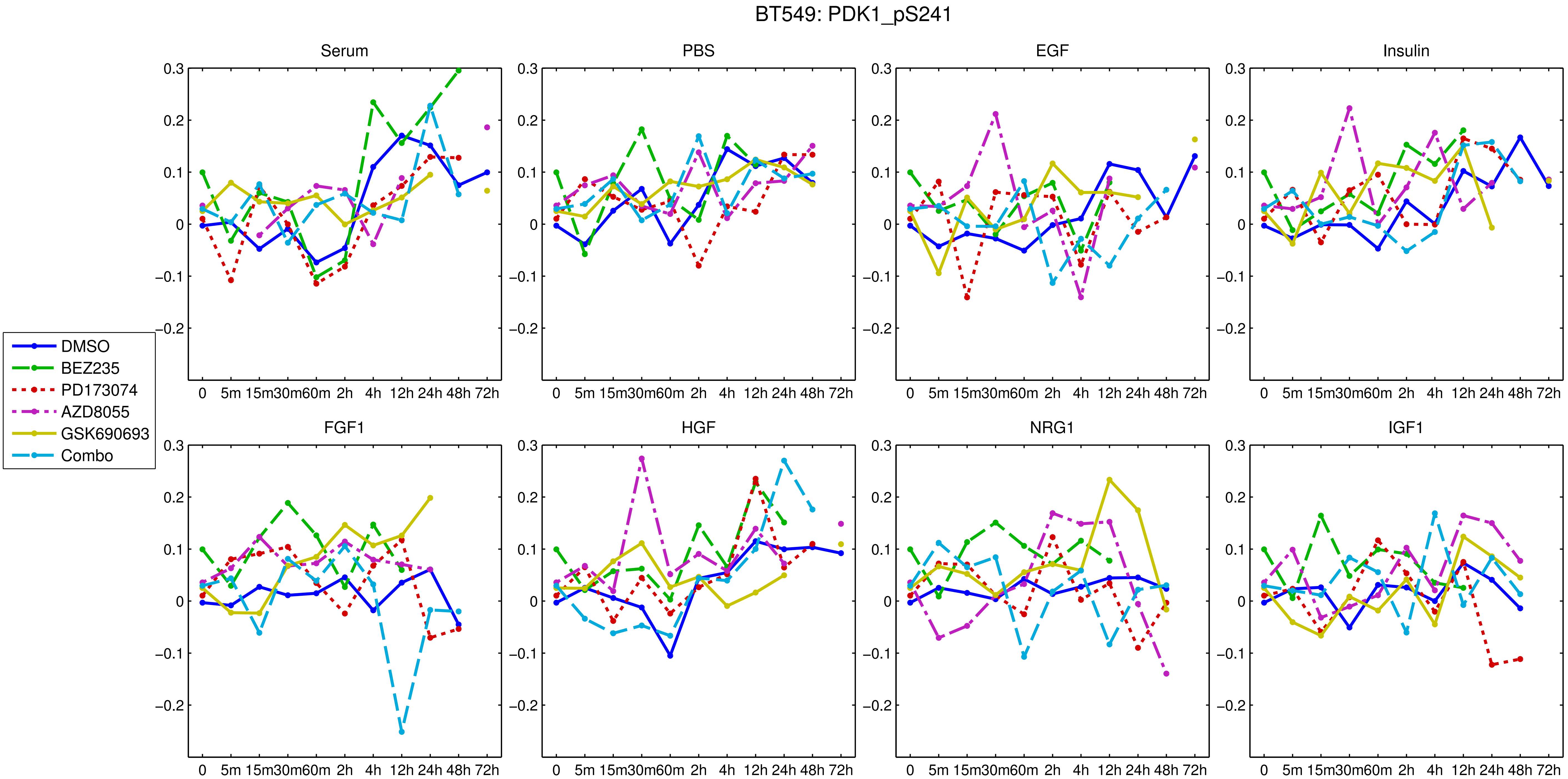
BT549: PDK1_pS241
Serum
PBS
EGF
Insulin
FGF1
HGF
NRG1
IGF1
0.3
0.2
0.1
0
−0.1
−0.2
0 5m 15m 30m 60m 2h 4h 12h 24h 48h 72h
DMSO
BEZ235
PD173074
AZD8055
GSK690693
Combo

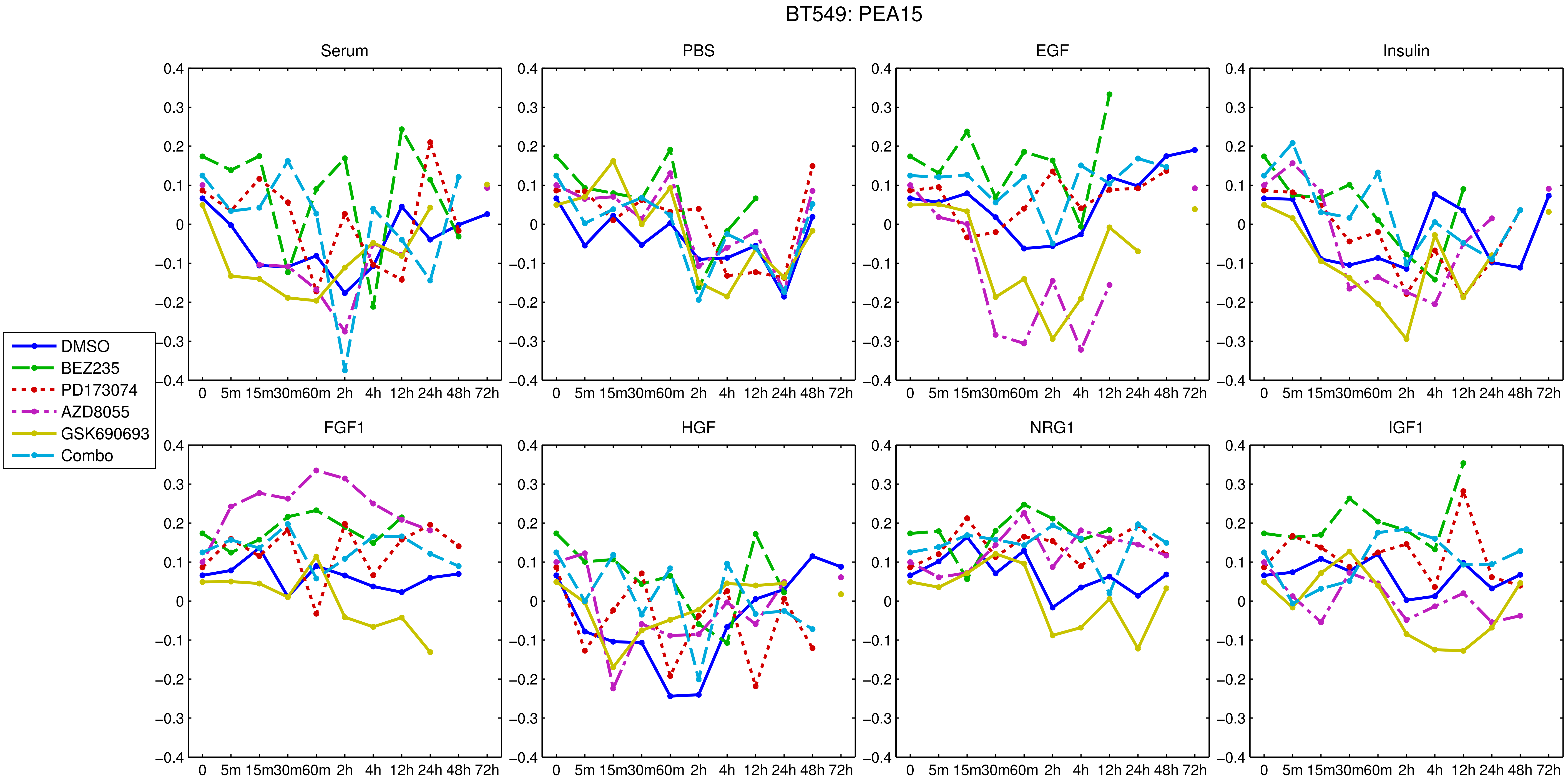

BT549: PEA15
Serum
PBS
EGF
Insulin
FGF1
HGF
NRG1
IGF1
DMSO
BEZ235
PD173074
AZD8055
GSK690693
Combo
0.4
0.3
0.2
0.1
0
−0.1
−0.2
−0.3
−0.4
0 5m 15m 30m 60m 2h 4h 12h 24h 48h 72h

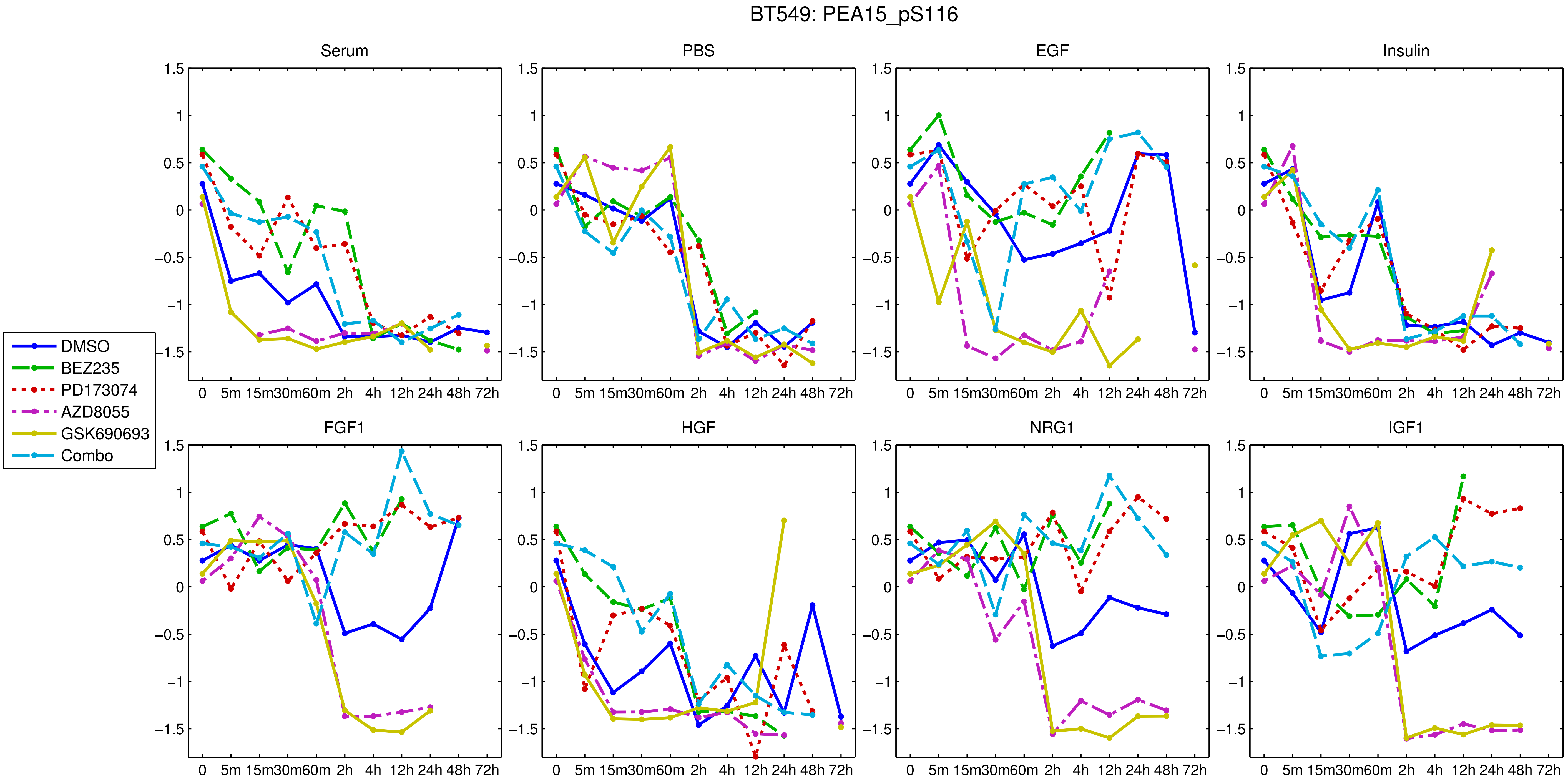
BT549: PEA15_pS116
Serum
PBS
EGF
Insulin
FGF1
HGF
NRG1
IGF1
DMSO
BEZ235
PD173074
AZD8055
GSK690693
Combo
1.5
1
0.5
0
−0.5
−1
−1.5
0
5m
15m
30m
60m
2h
4h
12h
24h
48h
72h

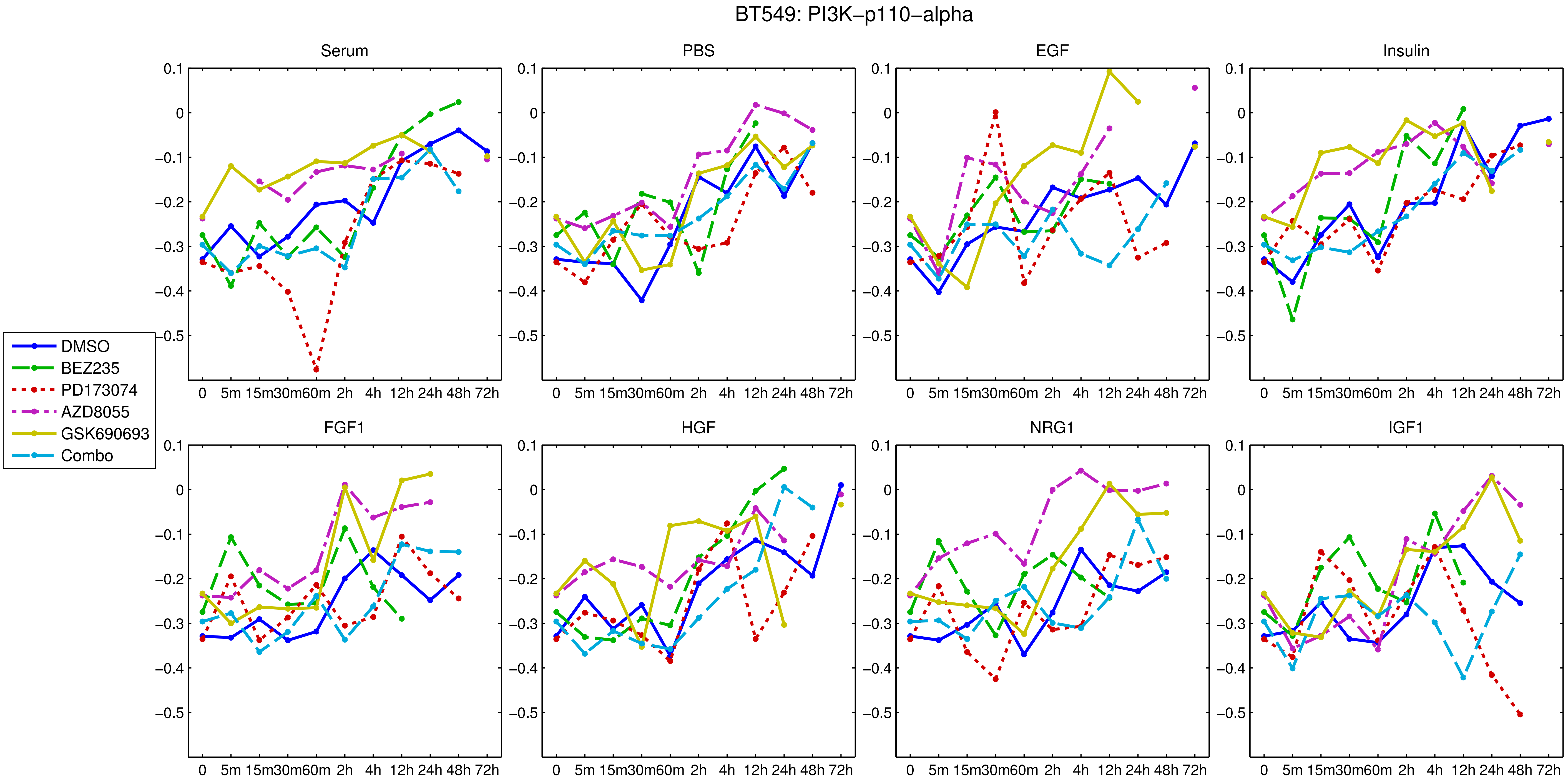
BT549: PI3K−p110−alpha
Serum
PBS
EGF
Insulin
FGF1
HGF
NRG1
IGF1
0.1
0
−0.1
−0.2
−0.3
−0.4
−0.5
0
5m
15m
30m
60m
2h
4h
12h
24h
48h
72h
DMSO
BEZ235
PD173074
AZD8055
GSK690693
Combo

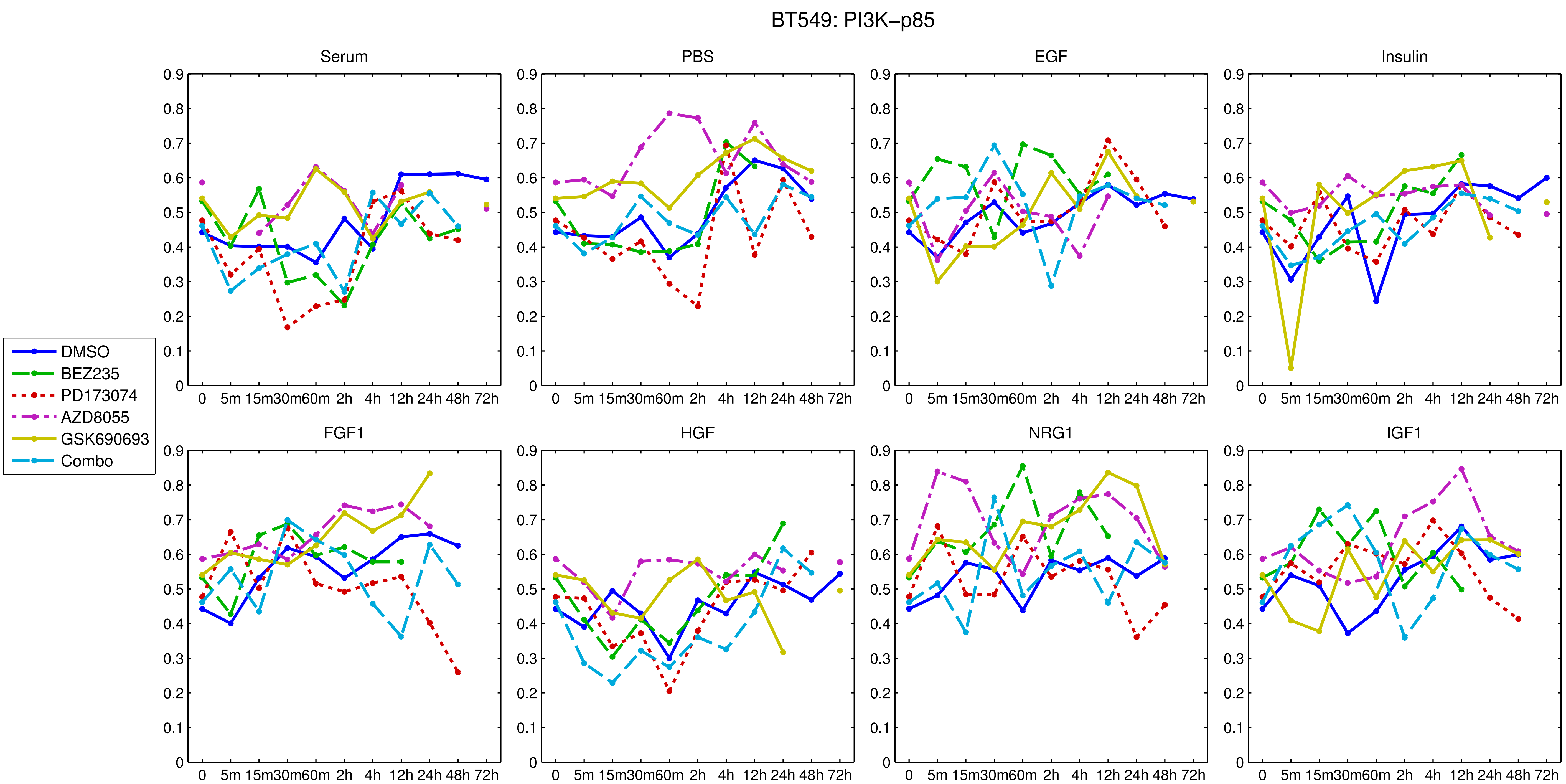
BT549: PI3K−p85
Serum
PBS
EGF
Insulin
FGF1
HGF
NRG1
IGF1
0 5m 15m30m60m 2h 4h 12h 24h 48h 72h
DMSO
BEZ235
PD173074
AZD8055
GSK690693
Combo

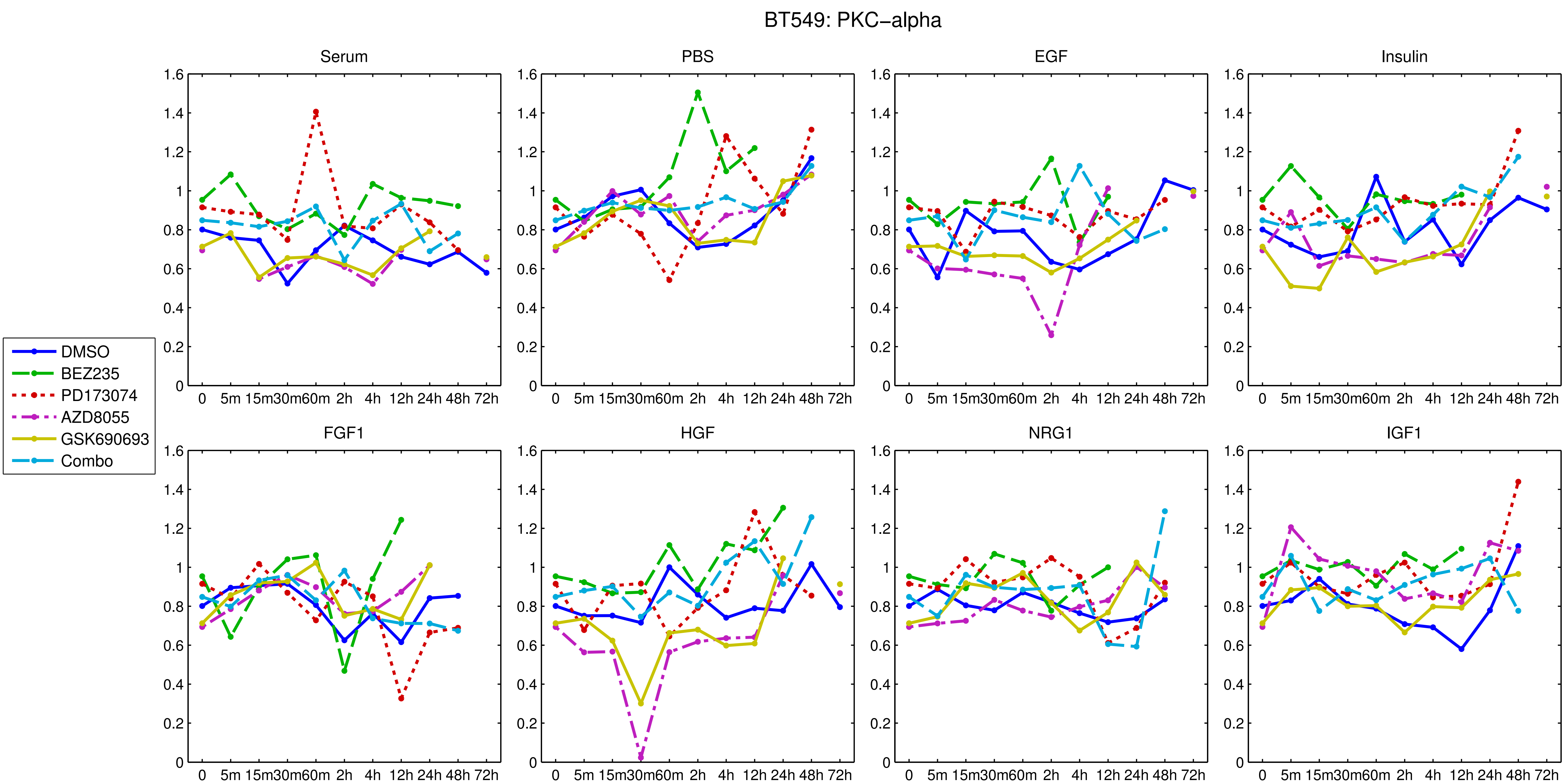
BT549: PKC−alpha
Serum
PBS
EGF
Insulin
FGF1
HGF
NRG1
IGF1
DMSO
BEZ235
PD173074
AZD8055
GSK690693
Combo
0
0.2
0.4
0.6
0.8
1
1.2
1.4
1.6
0
5m
15m
30m
60m
2h
4h
12h
24h
48h
72h

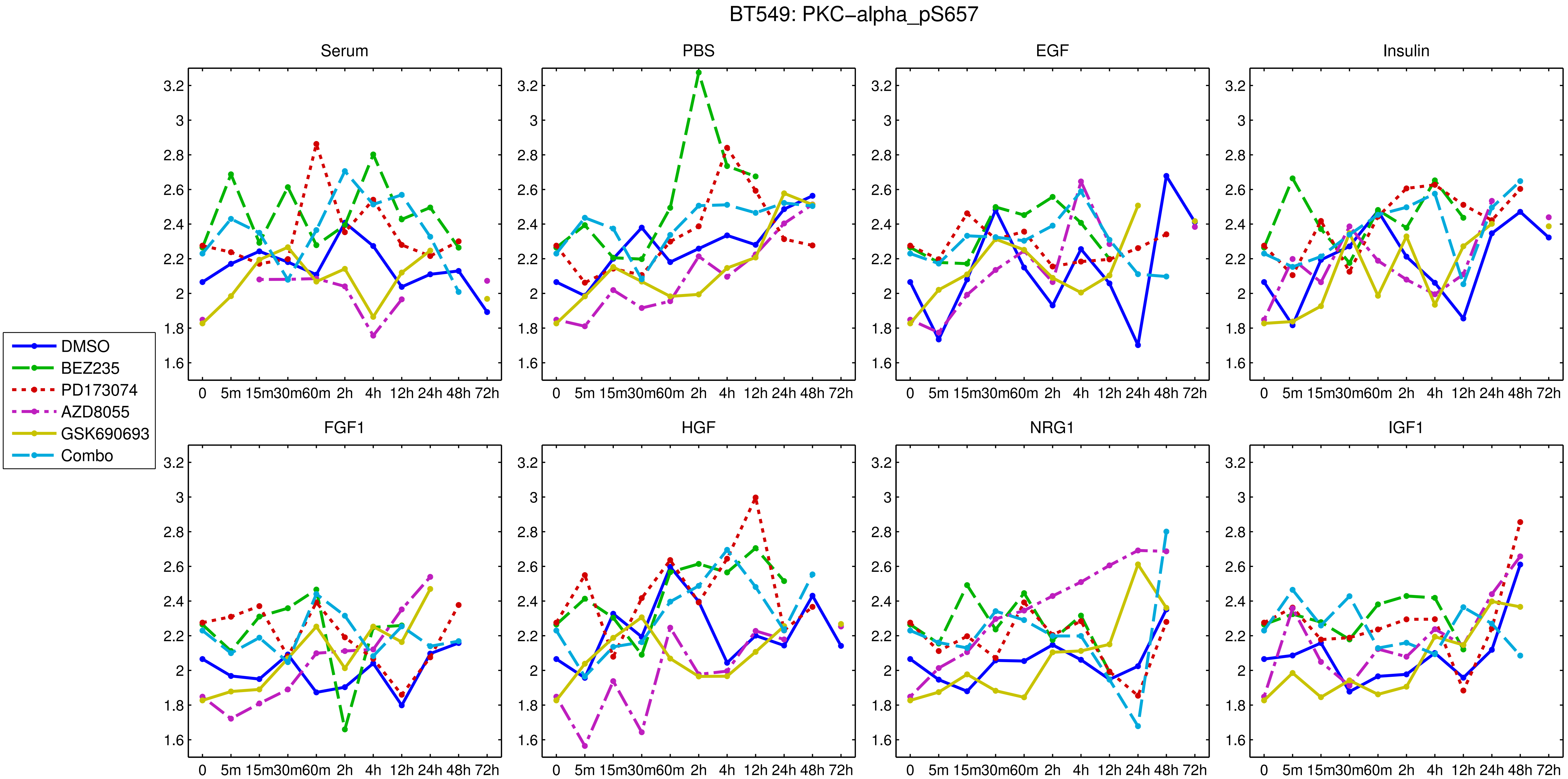

BT549: PKC−alpha_pS657
Serum
PBS
EGF
Insulin
FGF1
HGF
NRG1
IGF1
DMSO
BEZ235
PD173074
AZD8055
GSK690693
Combo
3.2
3
2.8
2.6
2.4
2.2
2
1.8
1.6
0
5m
15m
30m
60m
2h
4h
12h
24h
48h
72h

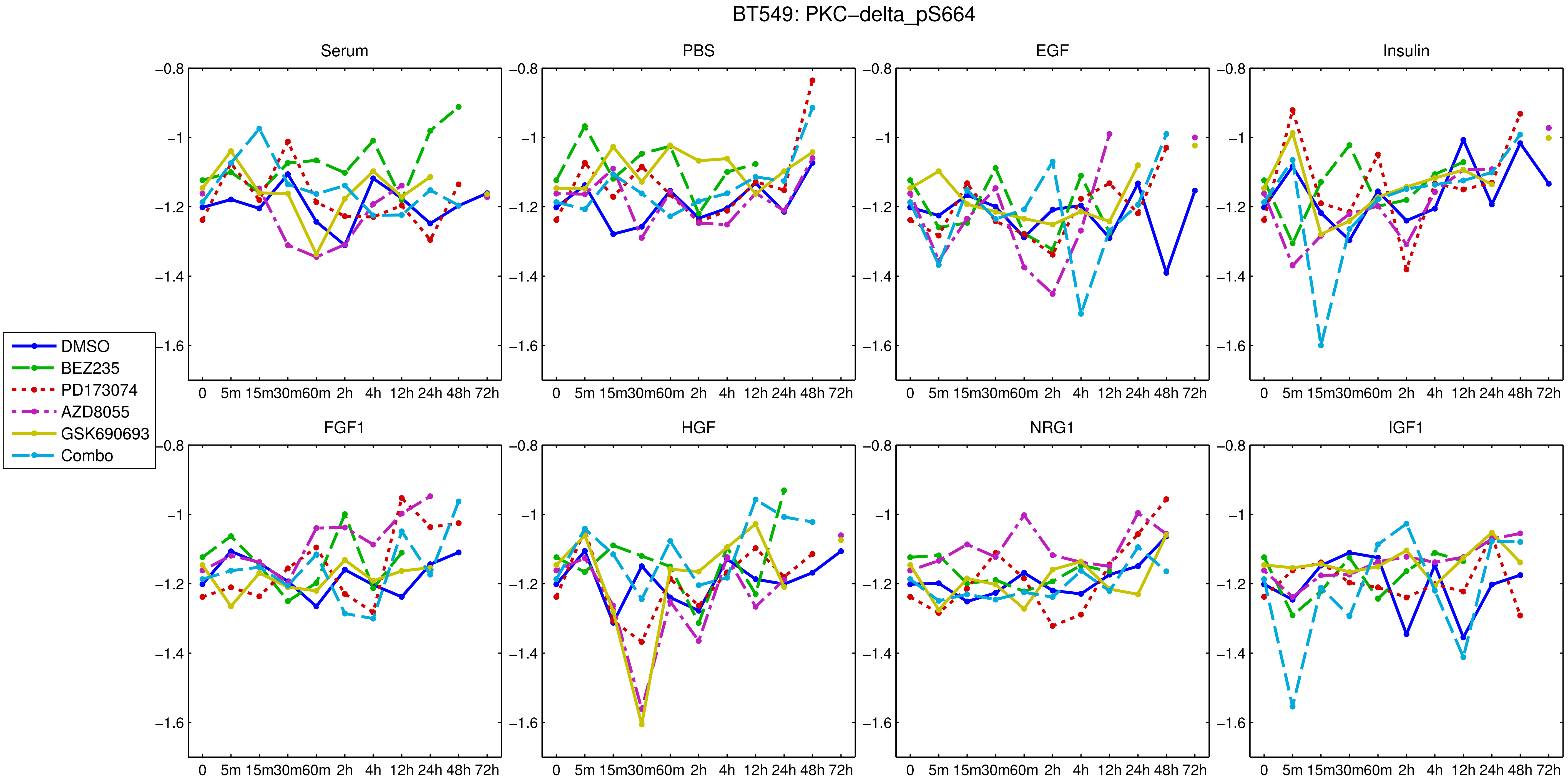

BT549: PKC−delta_pS664
Serum
PBS
EGF
Insulin
FGF1
HGF
NRG1
IGF1
−0.8
−1
−1.2
−1.4
−1.6
0
5m
15m
30m
60m
2h
4h
12h
24h
48h
72h
DMSO
BEZ235
PD173074
AZD8055
GSK690693
Combo

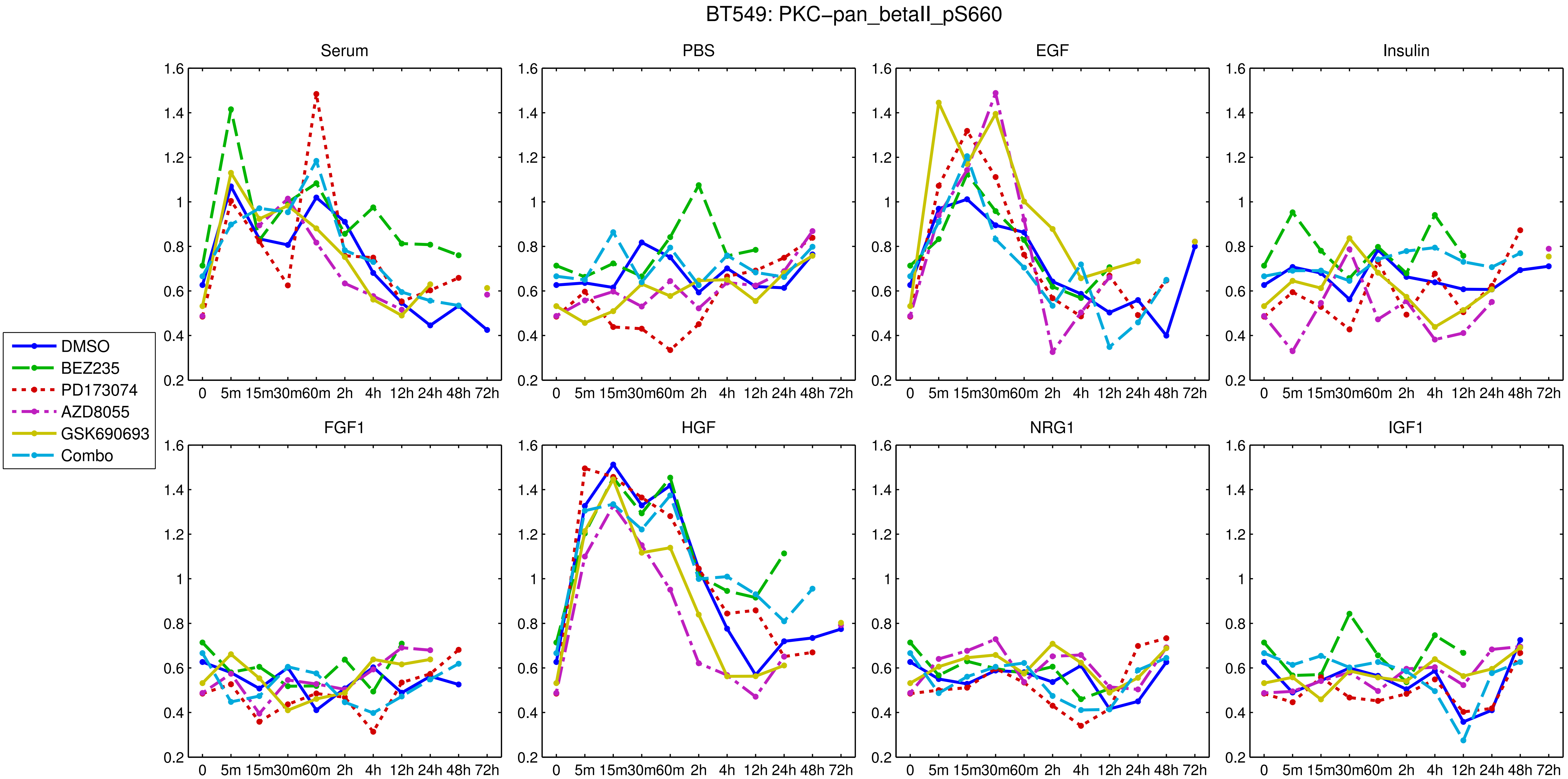
BT549: PKC−pan_betaII_pS660
Serum
PBS
EGF
Insulin
FGF1
HGF
NRG1
IGF1
DMSO
BEZ235
PD173074
AZD8055
GSK690693
Combo
0.2
0.4
0.6
0.8
1
1.2
1.4
1.6
0
5m
15m
30m
60m
2h
4h
12h
24h
48h
72h

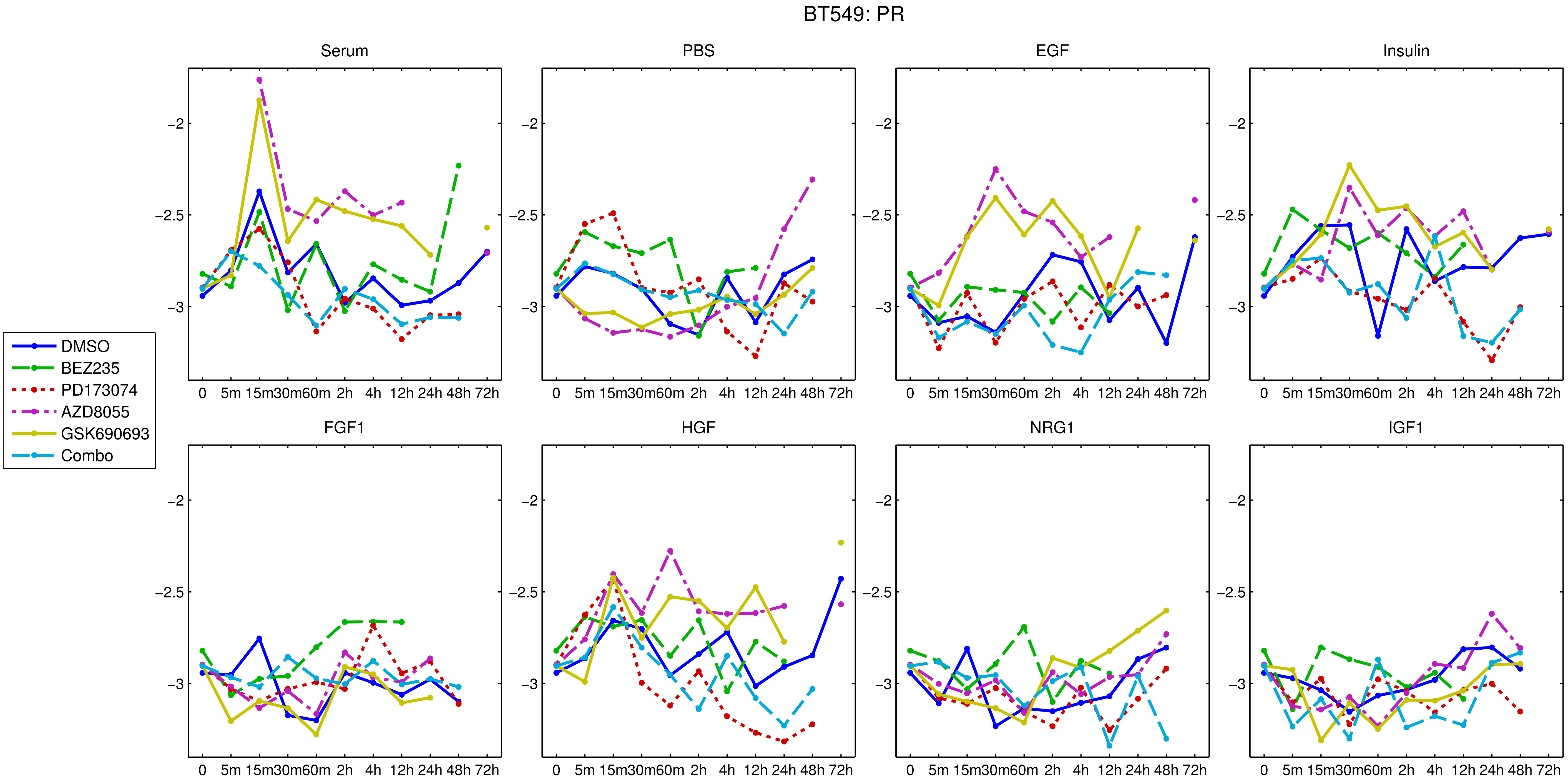
BT549: PR
Serum
PBS
EGF
Insulin
FGF1
HGF
NRG1
IGF1
−2
−2.5
−3
0 5m 15m 30m 60m 2h 4h 12h 24h 48h 72h
DMSO
BEZ235
PD173074
AZD8055
GSK690693
Combo

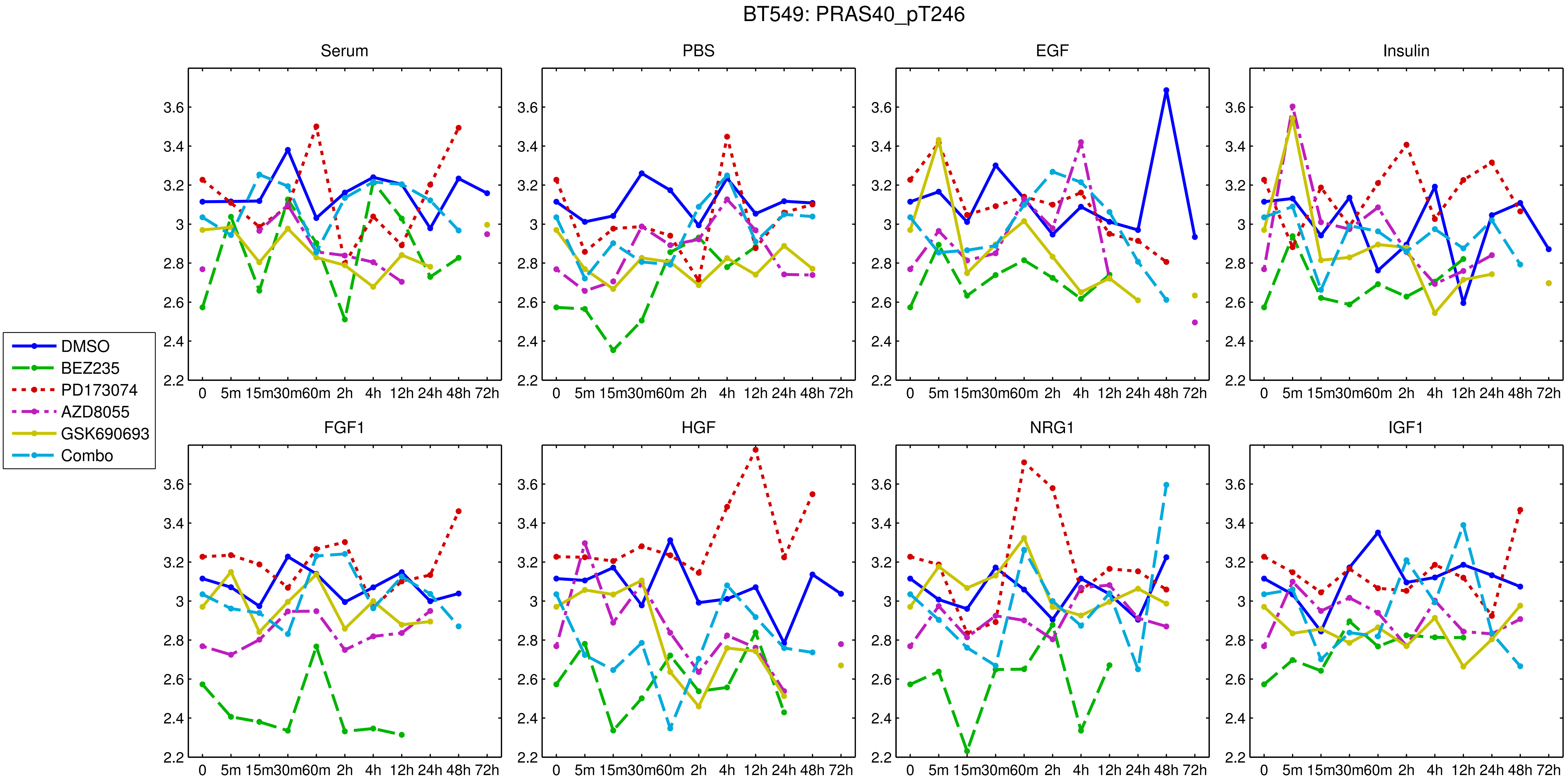

BT549: PRAS40_pT246
Serum
PBS
EGF
Insulin
FGF1
HGF
NRG1
IGF1
DMSO
BEZ235
PD173074
AZD8055
GSK690693
Combo
3.6
3.4
3.2
3
2.8
2.6
2.4
2.2
0 5m 15m 30m 60m 2h 4h 12h 24h 48h 72h

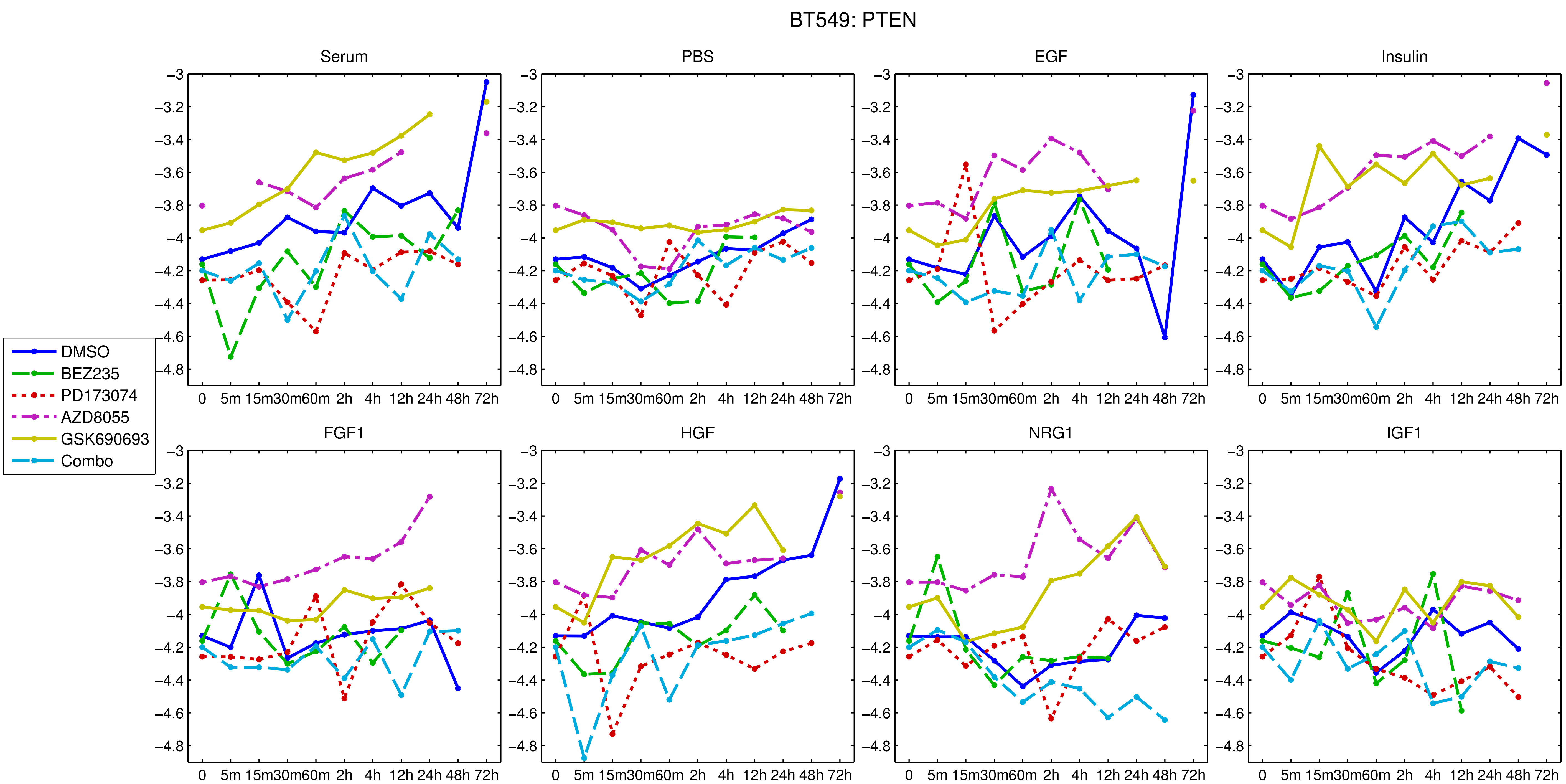

BT549: PTEN
Serum
PBS
EGF
Insulin
FGF1
HGF
NRG1
IGF1
−3
−3.2
−3.4
−3.6
−3.8
−4
−4.2
−4.4
−4.6
−4.8
0
5m
15m
30m
60m
2h
4h
12h
24h
48h
72h
DMSO
BEZ235
PD173074
AZD8055
GSK690693
Combo

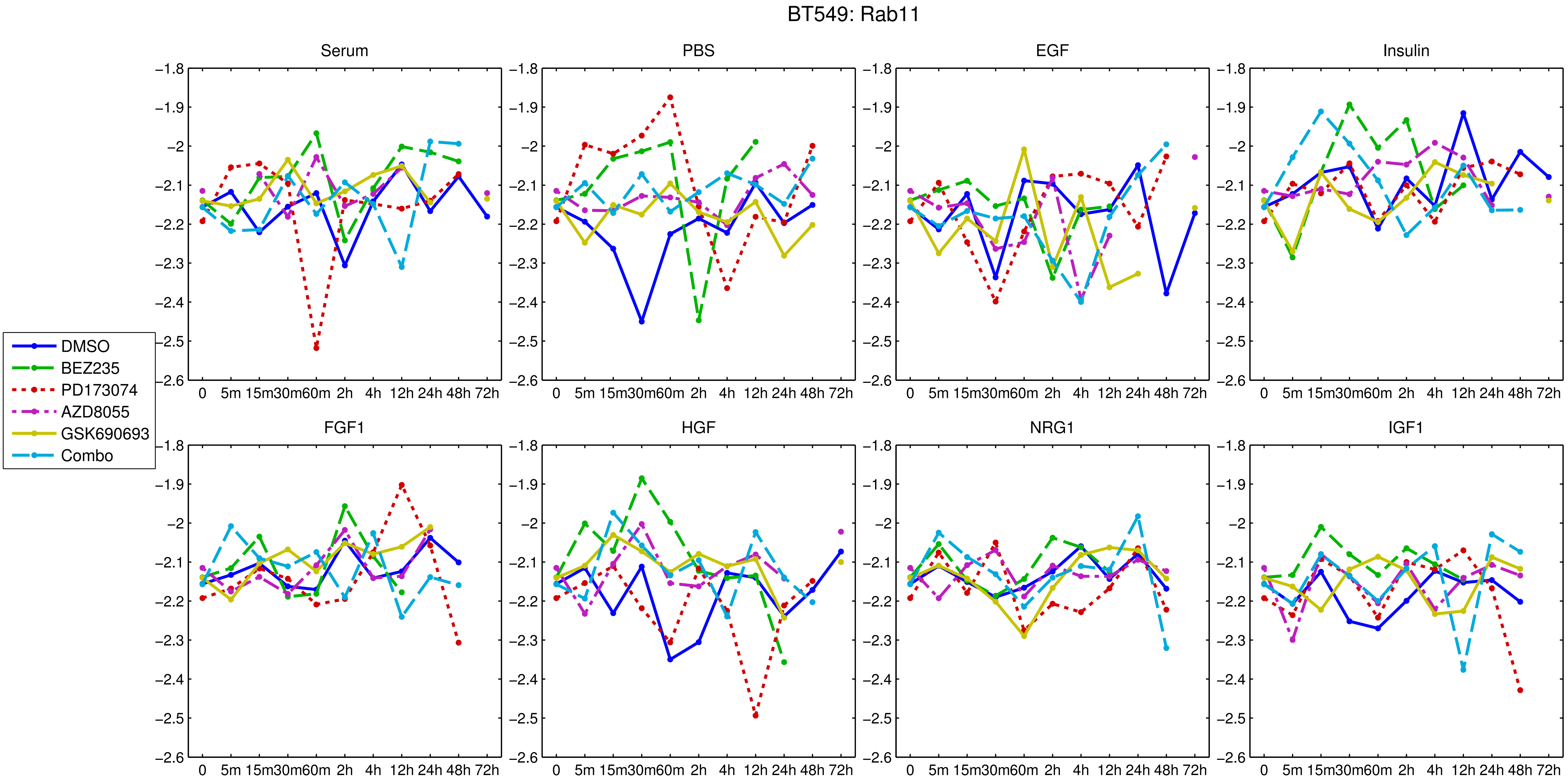
BT549: Rab11
Serum
PBS
EGF
Insulin
FGF1
HGF
NRG1
IGF1
DMSO
BEZ235
PD173074
AZD8055
GSK690693
Combo
−1.8
−1.9
−2
−2.1
−2.2
−2.3
−2.4
−2.5
−2.6
0
5m
15m
30m
60m
2h
4h
12h
24h
48h
72h

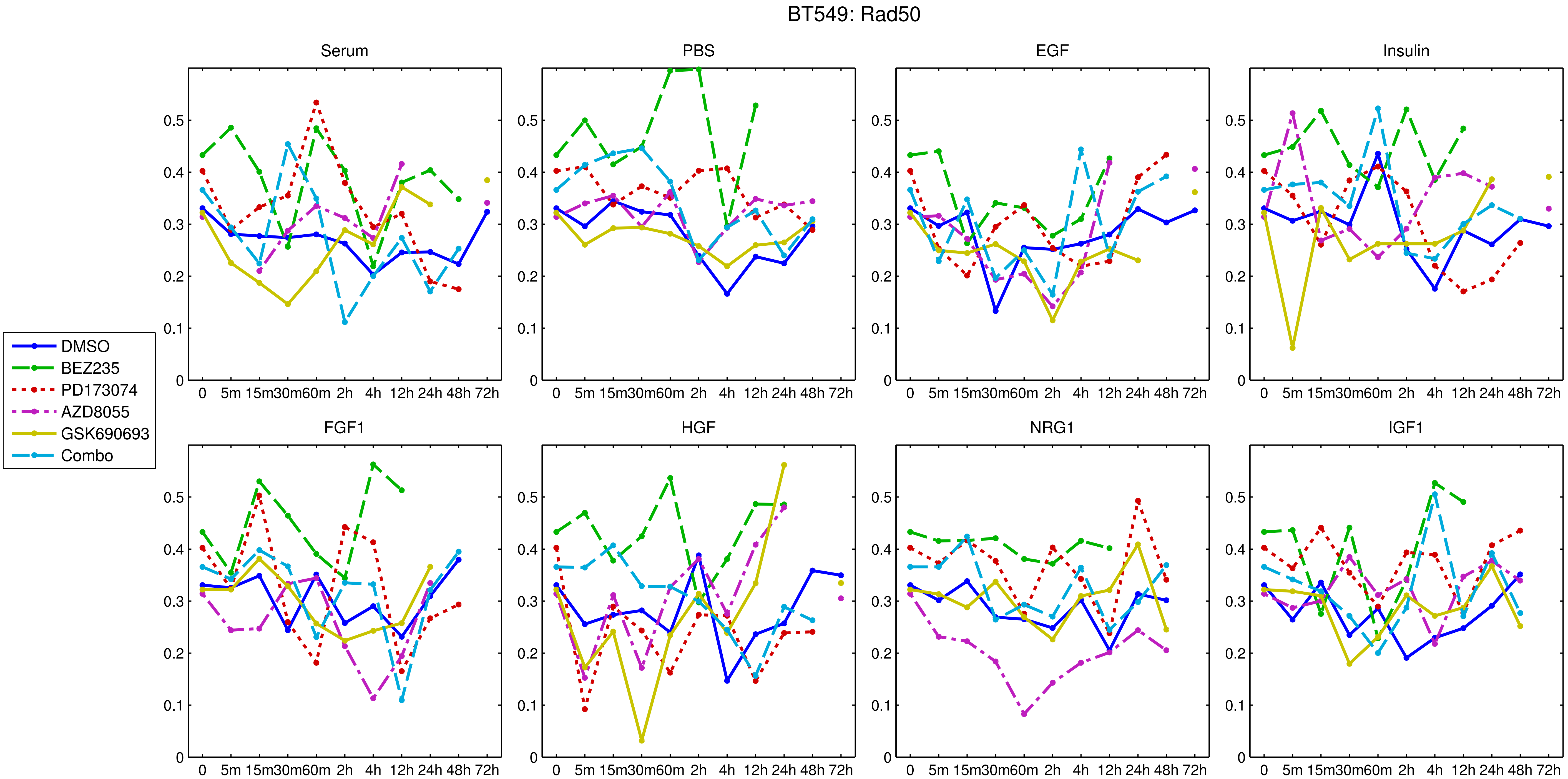
BT549: Rad50
Serum
PBS
EGF
Insulin
FGF1
HGF
NRG1
IGF1
DMSO
BEZ235
PD173074
AZD8055
GSK690693
Combo
0 5m 15m 30m 60m 2h 4h 12h 24h 48h 72h
0 0.1 0.2 0.3 0.4 0.5

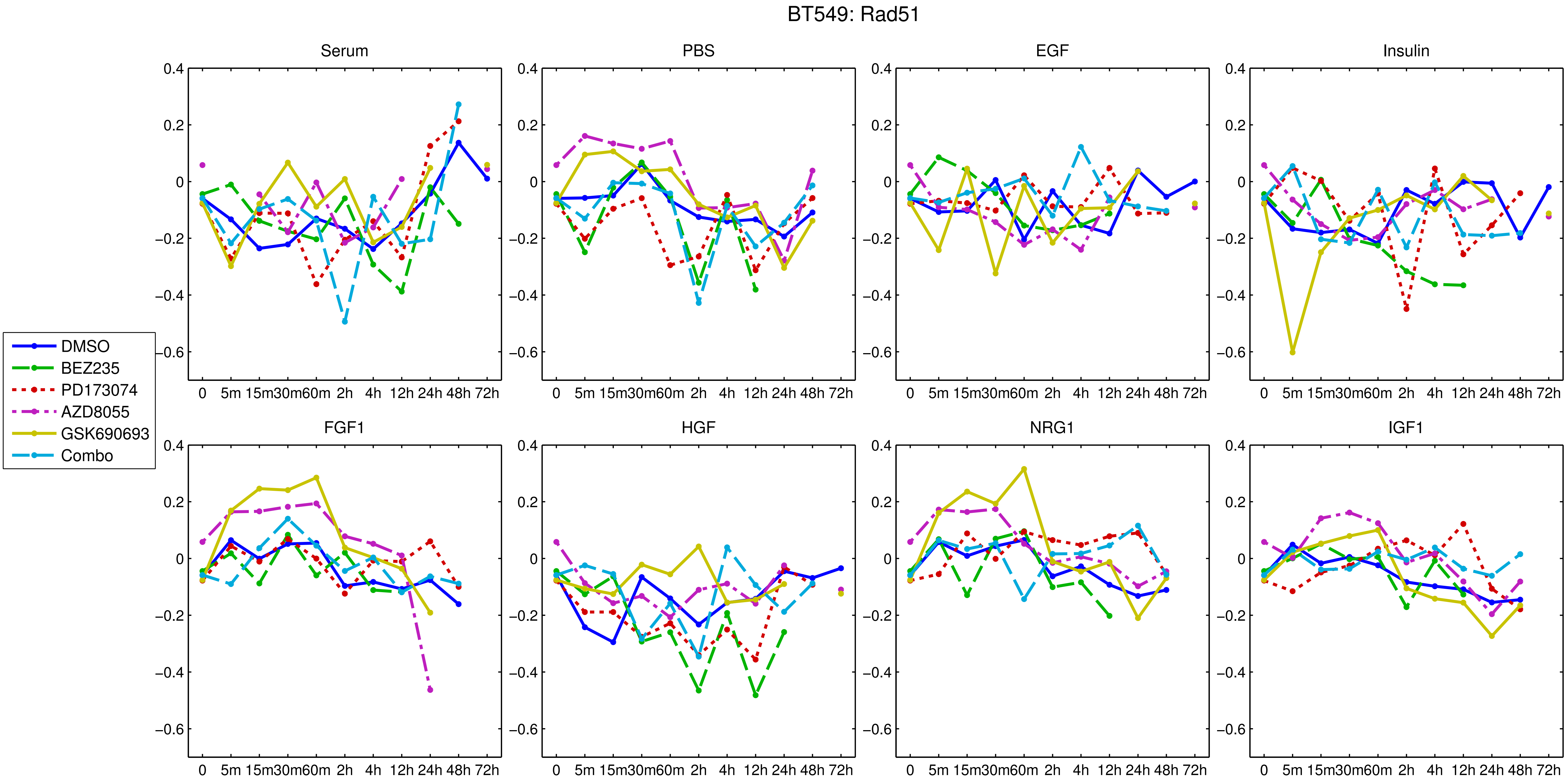

BT549: Rad51
Serum
PBS
EGF
Insulin
FGF1
HGF
NRG1
IGF1
0.4
0.2
0
−0.2
−0.4
−0.6
0
5m
15m
30m
60m
2h
4h
12h
24h
48h
72h
DMSO
BEZ235
PD173074
AZD8055
GSK690693
Combo

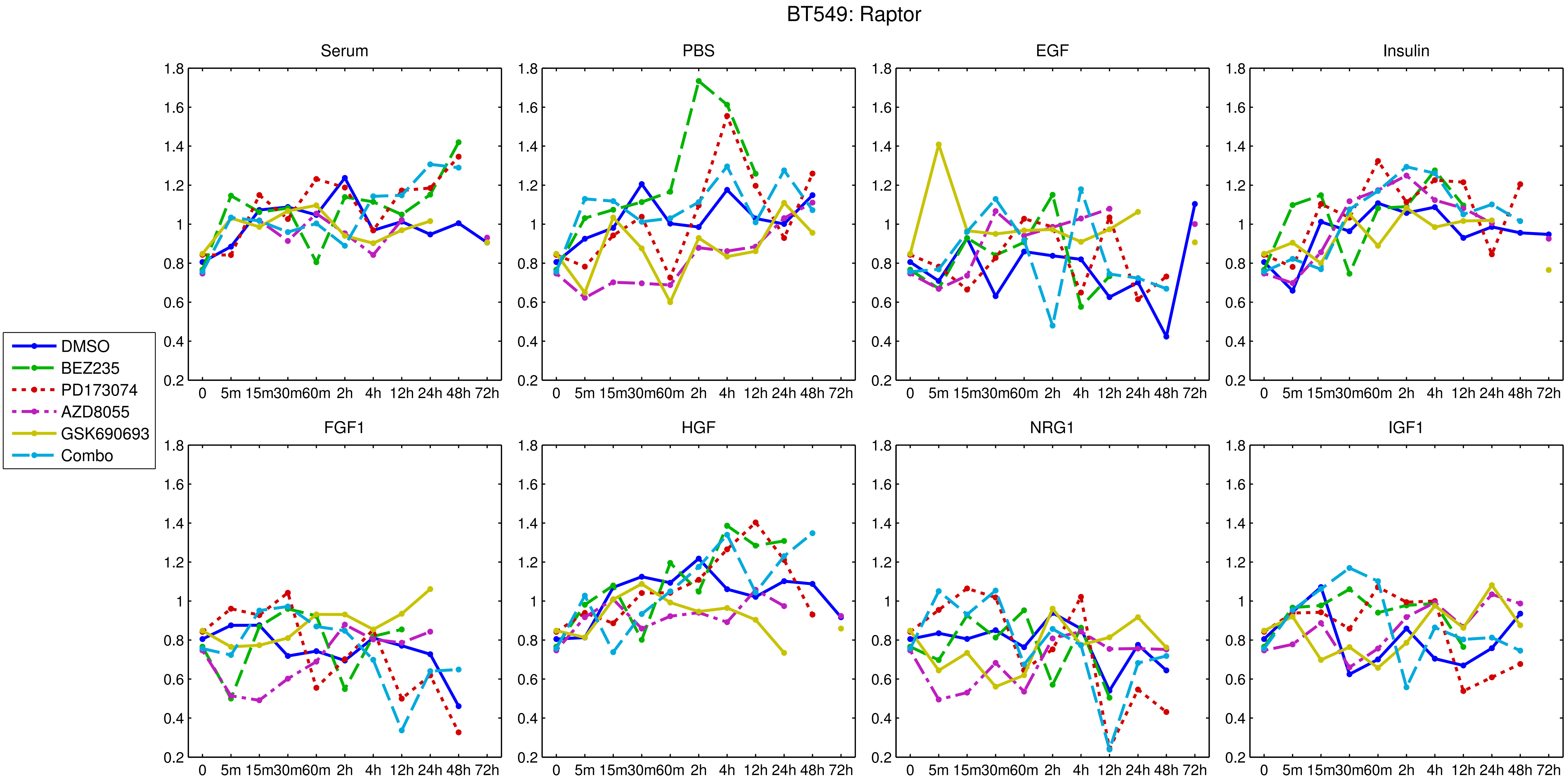

BT549: Raptor
Serum
PBS
EGF
Insulin
FGF1
HGF
NRG1
IGF1
DMSO
BEZ235
PD173074
AZD8055
GSK690693
Combo
1.8
1.6
1.4
1.2
1
0.8
0.6
0.4
0.2
0
5m
15m
30m
60m
2h
4h
12h
24h
48h
72h

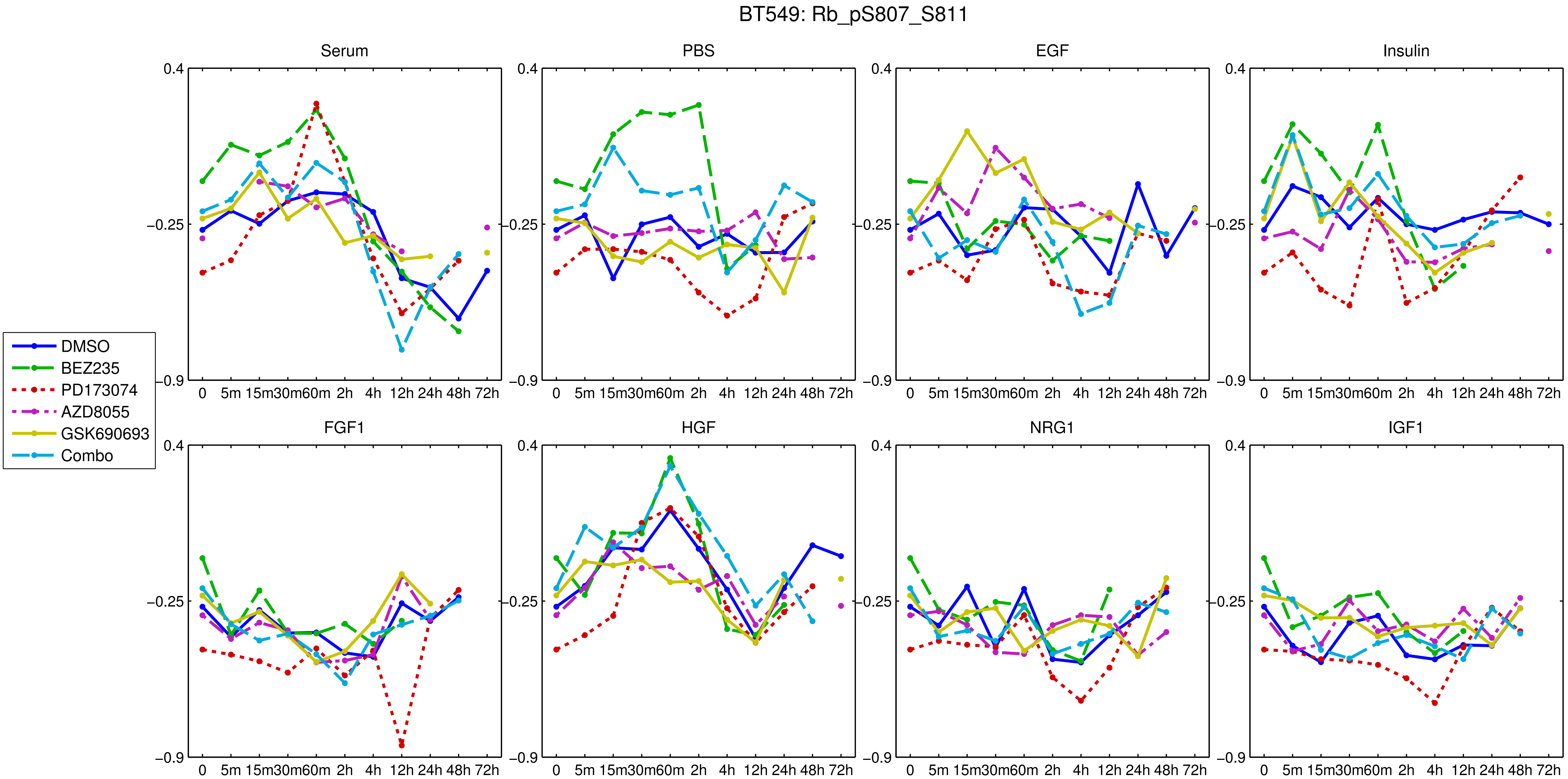

BT549: Rb_pS807_S811
Serum
PBS
EGF
Insulin
FGF1
HGF
NRG1
IGF1
0.4
−0.25
−0.9
0
5m
15m
30m
60m
2h
4h
12h
24h
48h
72h
DMSO
BEZ235
PD173074
AZD8055
GSK690693
Combo

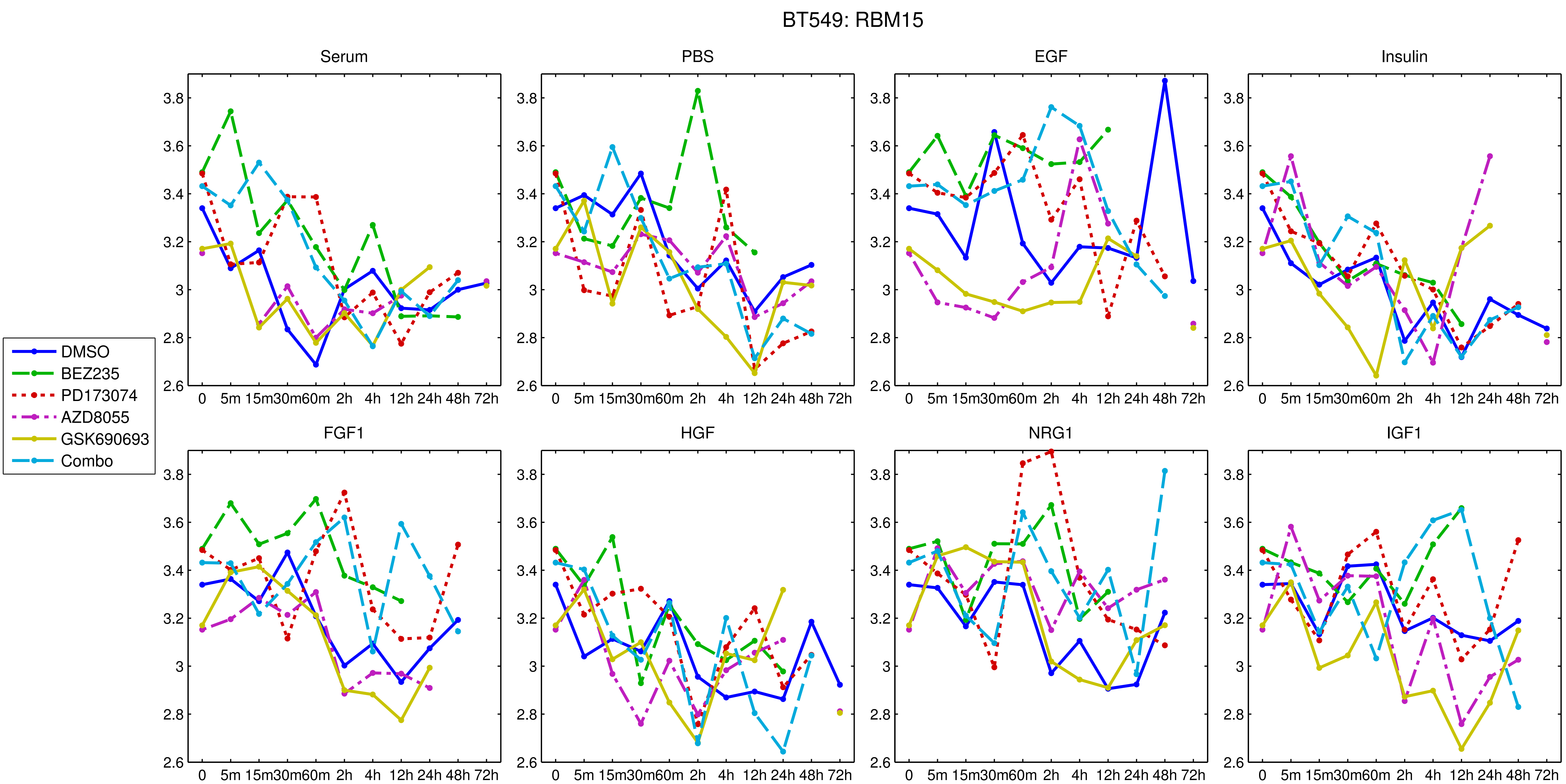

BT549: RBM15
Serum
PBS
EGF
Insulin
FGF1
HGF
NRG1
IGF1
DMSO
BEZ235
PD173074
AZD8055
GSK690693
Combo
3.8
3.6
3.4
3.2
3
2.8
2.6
0
5m
15m
30m
60m
2h
4h
12h
24h
48h
72h

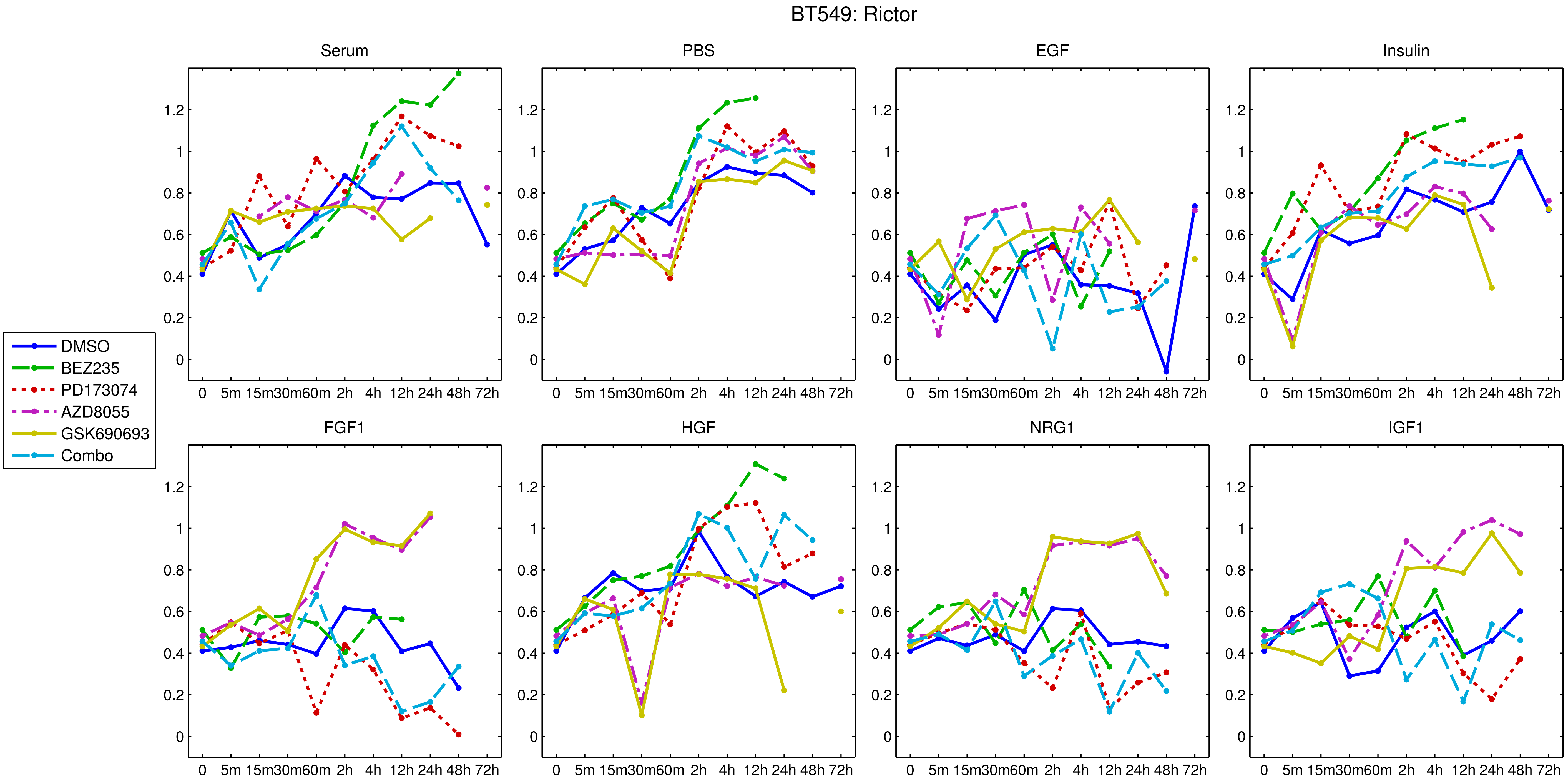
BT549: Rictor
Serum
PBS
EGF
Insulin
FGF1
HGF
NRG1
IGF1
DMSO
BEZ235
PD173074
AZD8055
GSK690693
Combo
0
0.2
0.4
0.6
0.8
1
1.2
0 5m 15m30m60m 2h 4h 12h 24h 48h 72h

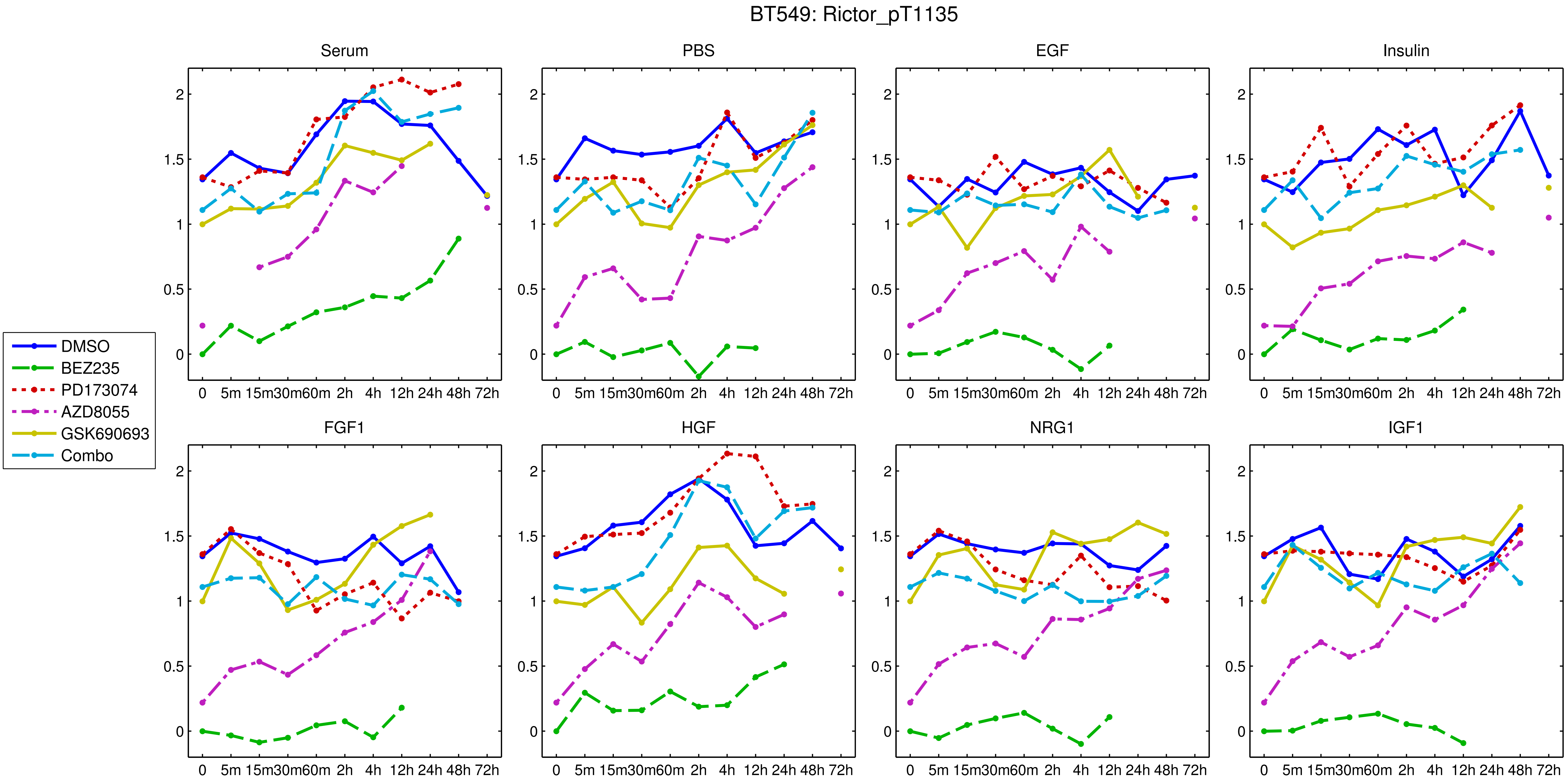

BT549: Rictor_pT1135
Serum
PBS
EGF
Insulin
FGF1
HGF
NRG1
IGF1
DMSO
BEZ235
PD173074
AZD8055
GSK690693
Combo
0
0.5
1
1.5
2
0 5m 15m30m60m 2h 4h 12h 24h 48h 72h

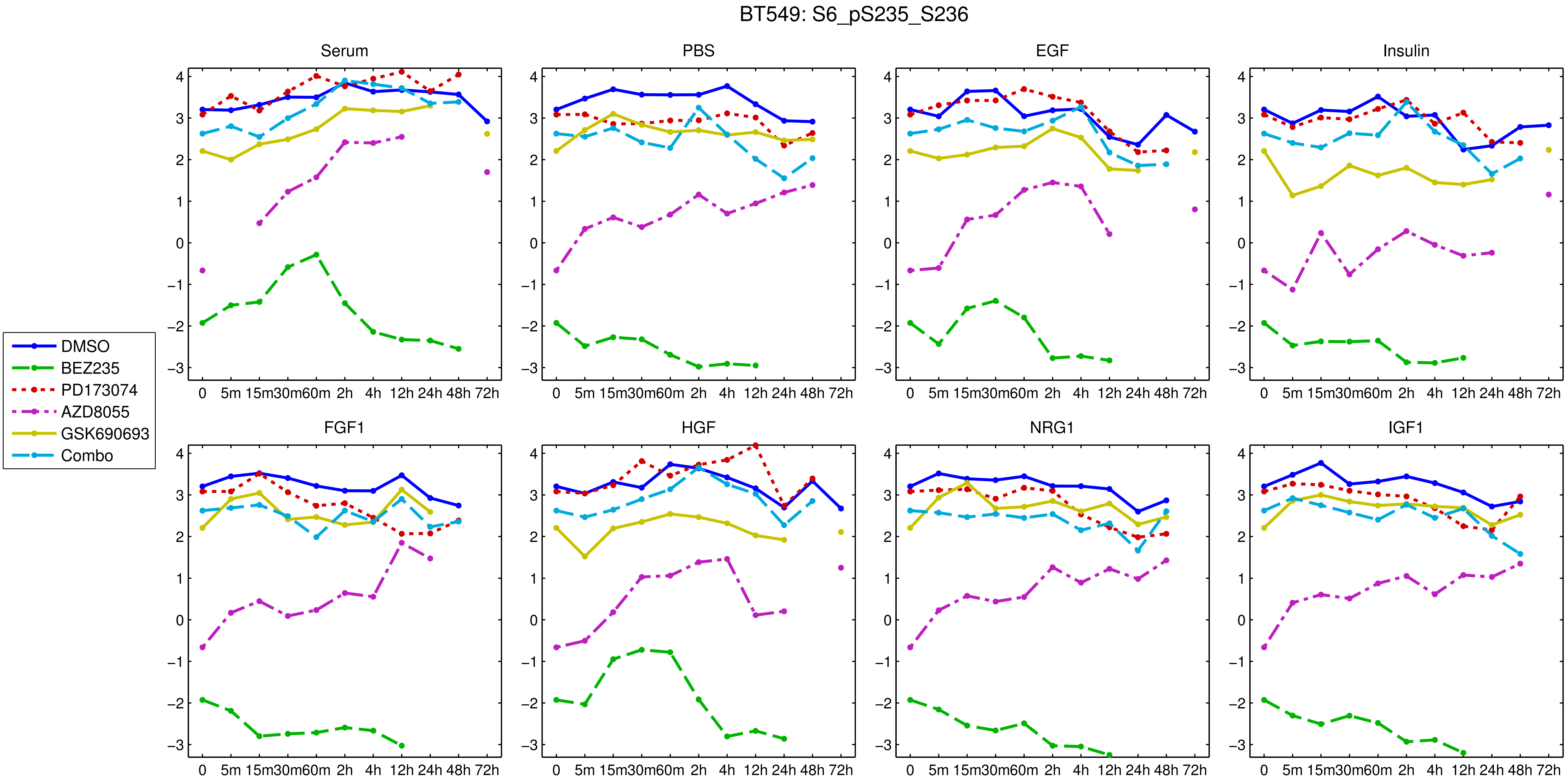
BT549: S6_pS235_S236
Serum
PBS
EGF
Insulin
FGF1
HGF
NRG1
IGF1
DMSO
BEZ235
PD173074
AZD8055
GSK690693
Combo
0 5m 15m 30m 60m 2h 4h 12h 24h 48h 72h

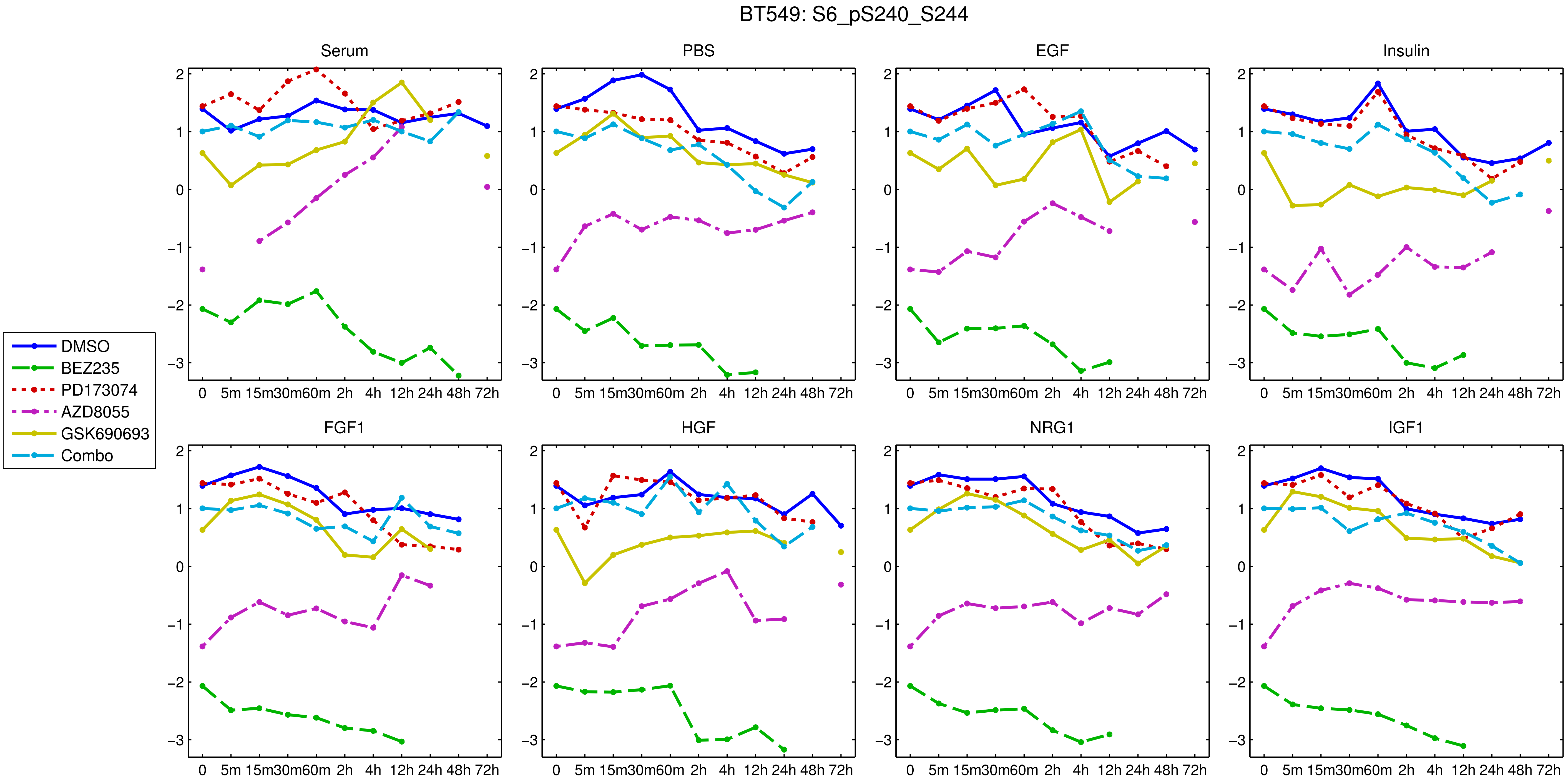
BT549: S6_pS240_S244
Serum
PBS
EGF
Insulin
FGF1
HGF
NRG1
IGF1
DMSO
BEZ235
PD173074
AZD8055
GSK690693
Combo
0 5m 15m 30m 60m 2h 4h 12h 24h 48h 72h

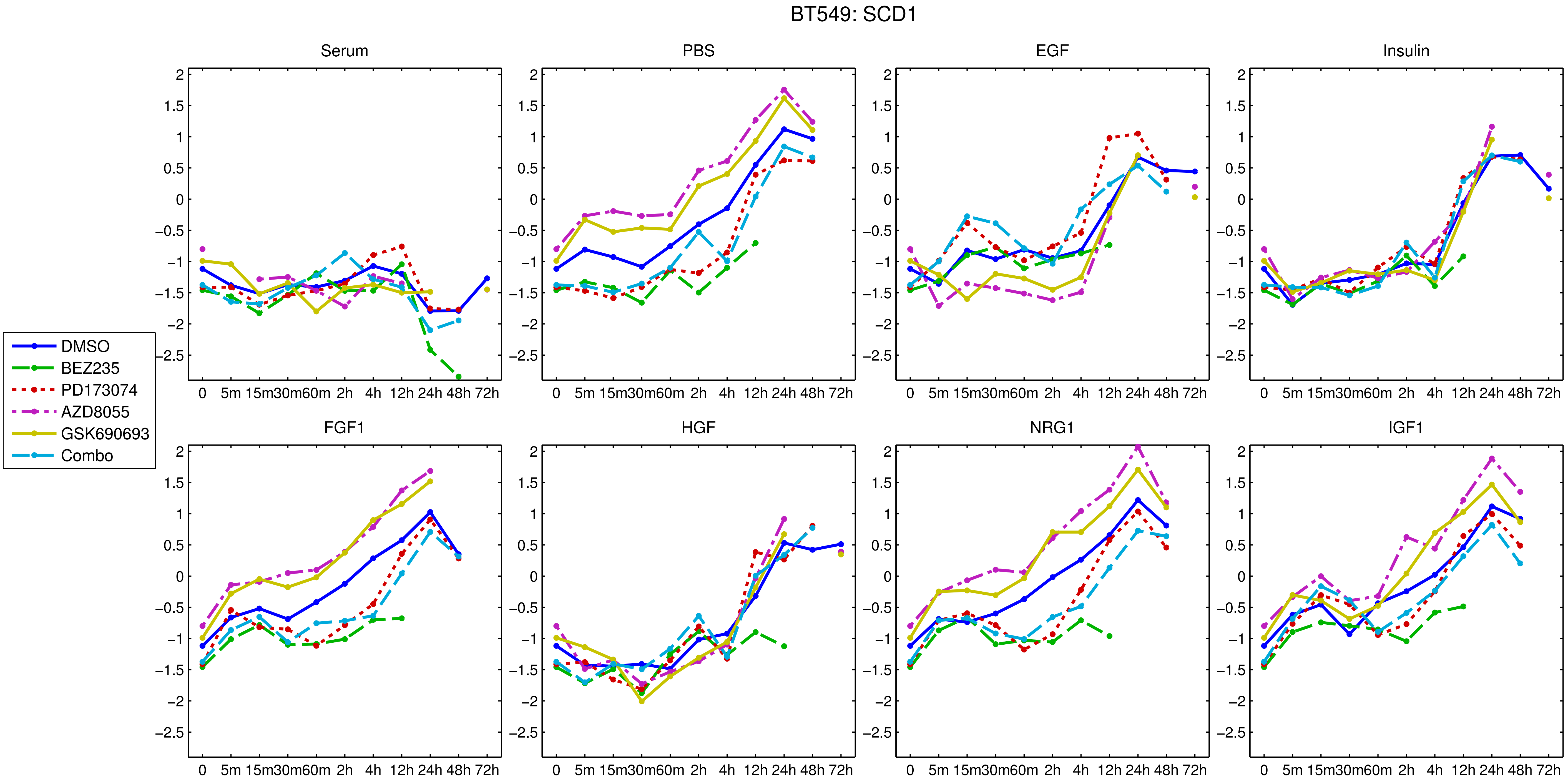
BT549: SCD1
Serum
PBS
EGF
Insulin
FGF1
HGF
NRG1
IGF1
DMSO
BEZ235
PD173074
AZD8055
GSK690693
Combo
0
5m
15m
30m
60m
2h
4h
12h
24h
48h
72h
2
1.5
1
0.5
0
−0.5
−1
−1.5
−2
−2.5

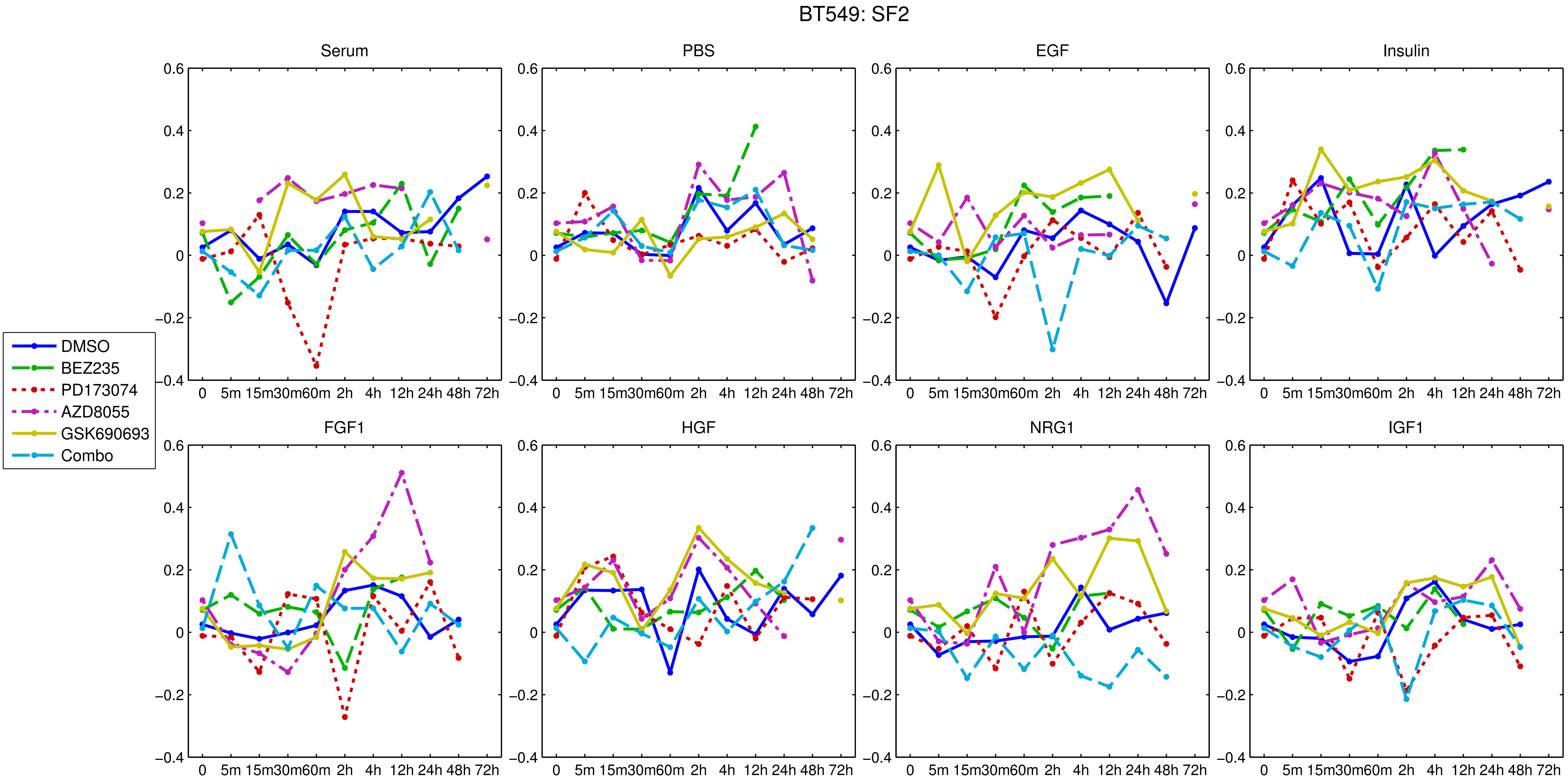
BT549: SF2
Serum
PBS
EGF
Insulin
FGF1
HGF
NRG1
IGF1
0.6
0.4
0.2
0
−0.2
−0.4
0 5m 15m30m60m 2h 4h 12h 24h 48h 72h
DMSO
BEZ235
PD173074
AZD8055
GSK690693
Combo

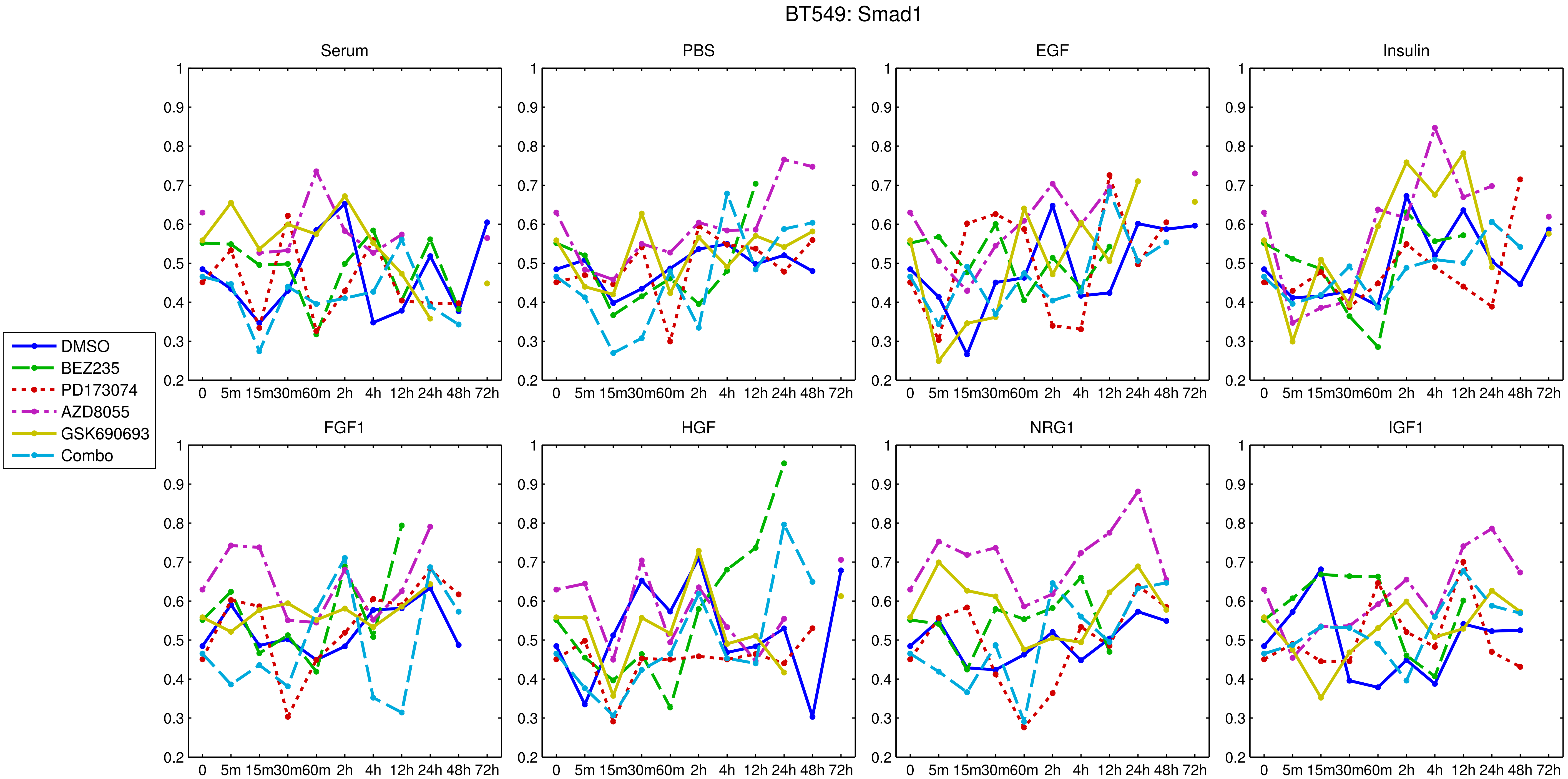
BT549: Smad1
Serum
PBS
EGF
Insulin
FGF1
HGF
NRG1
IGF1
DMSO
BEZ235
PD173074
AZD8055
GSK690693
Combo
0
5m
15m
30m
60m
2h
4h
12h
24h
48h
72h
0.2
0.3
0.4
0.5
0.6
0.7
0.8
0.9
1

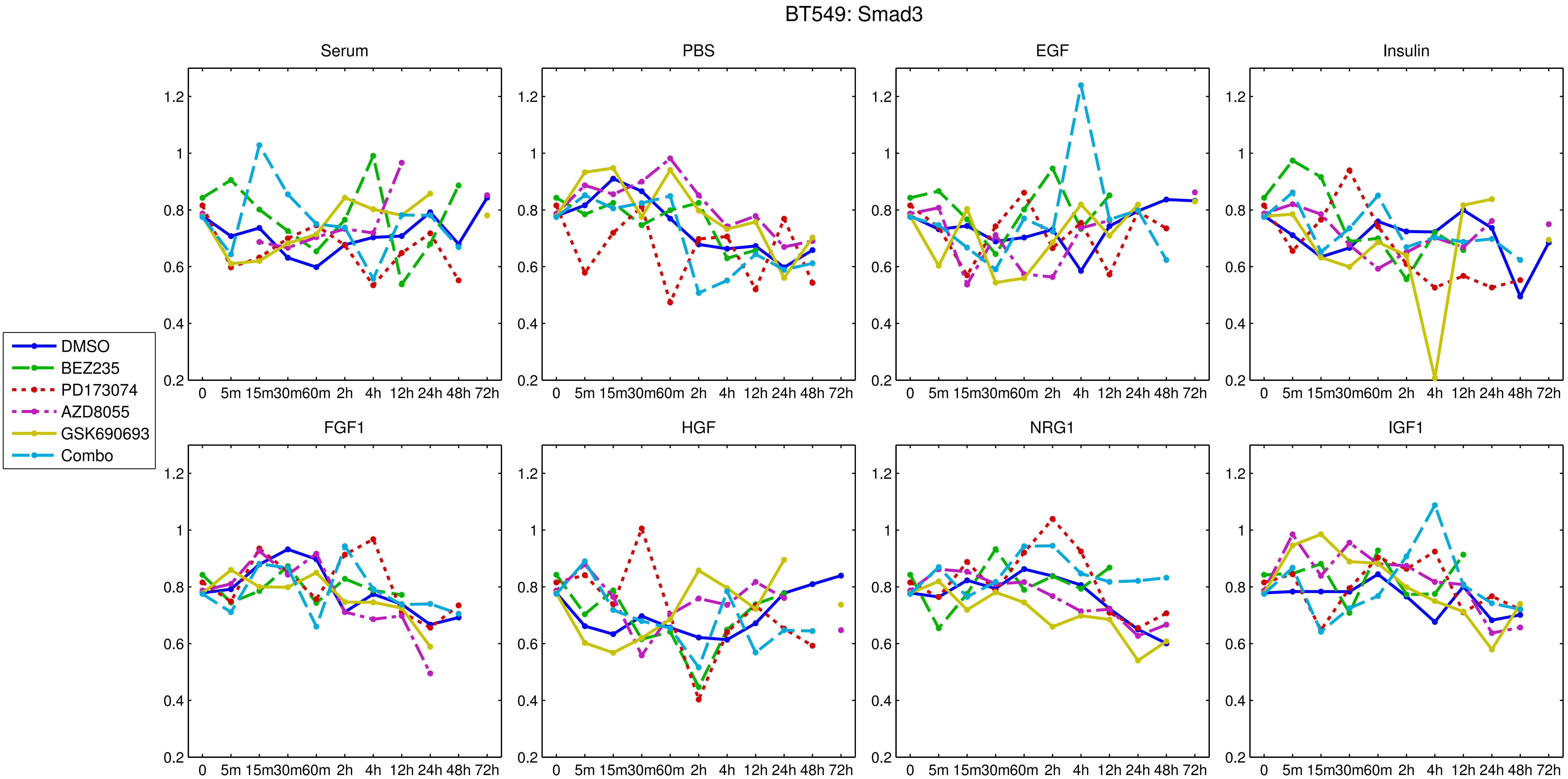

BT549: Smad3
Serum
PBS
EGF
Insulin
FGF1
HGF
NRG1
IGF1
DMSO
BEZ235
PD173074
AZD8055
GSK690693
Combo
0.2
0.4
0.6
0.8
1
1.2
0
5m
15m
30m
60m
2h
4h
12h
24h
48h
72h

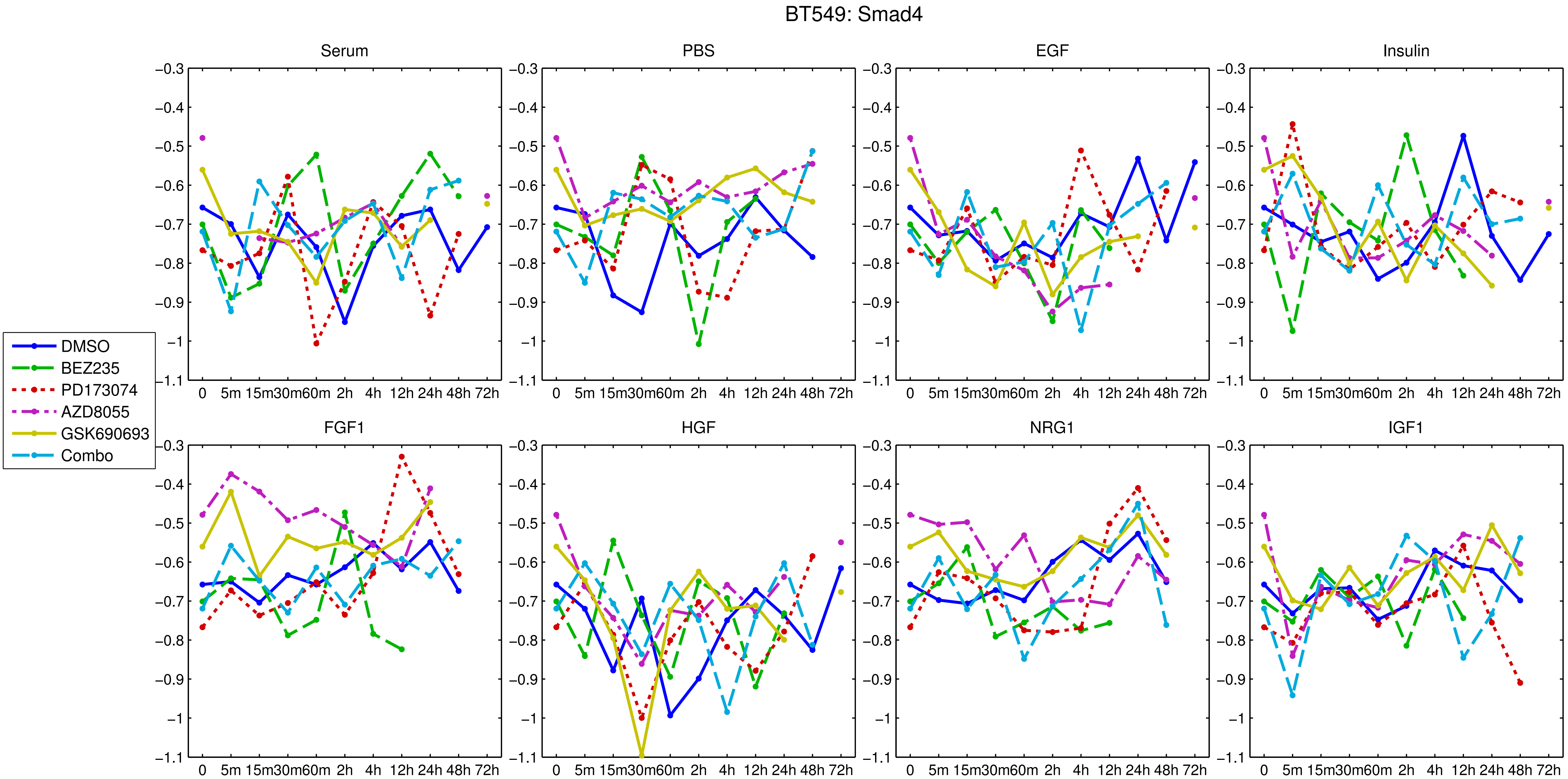
BT549: Smad4
Serum
PBS
EGF
Insulin
FGF1
HGF
NRG1
IGF1
DMSO
BEZ235
PD173074
AZD8055
GSK690693
Combo
0
5m
15m
30m
60m
2h
4h
12h
24h
48h
72h
−0.3
−0.4
−0.5
−0.6
−0.7
−0.8
−0.9
−1
−1.1

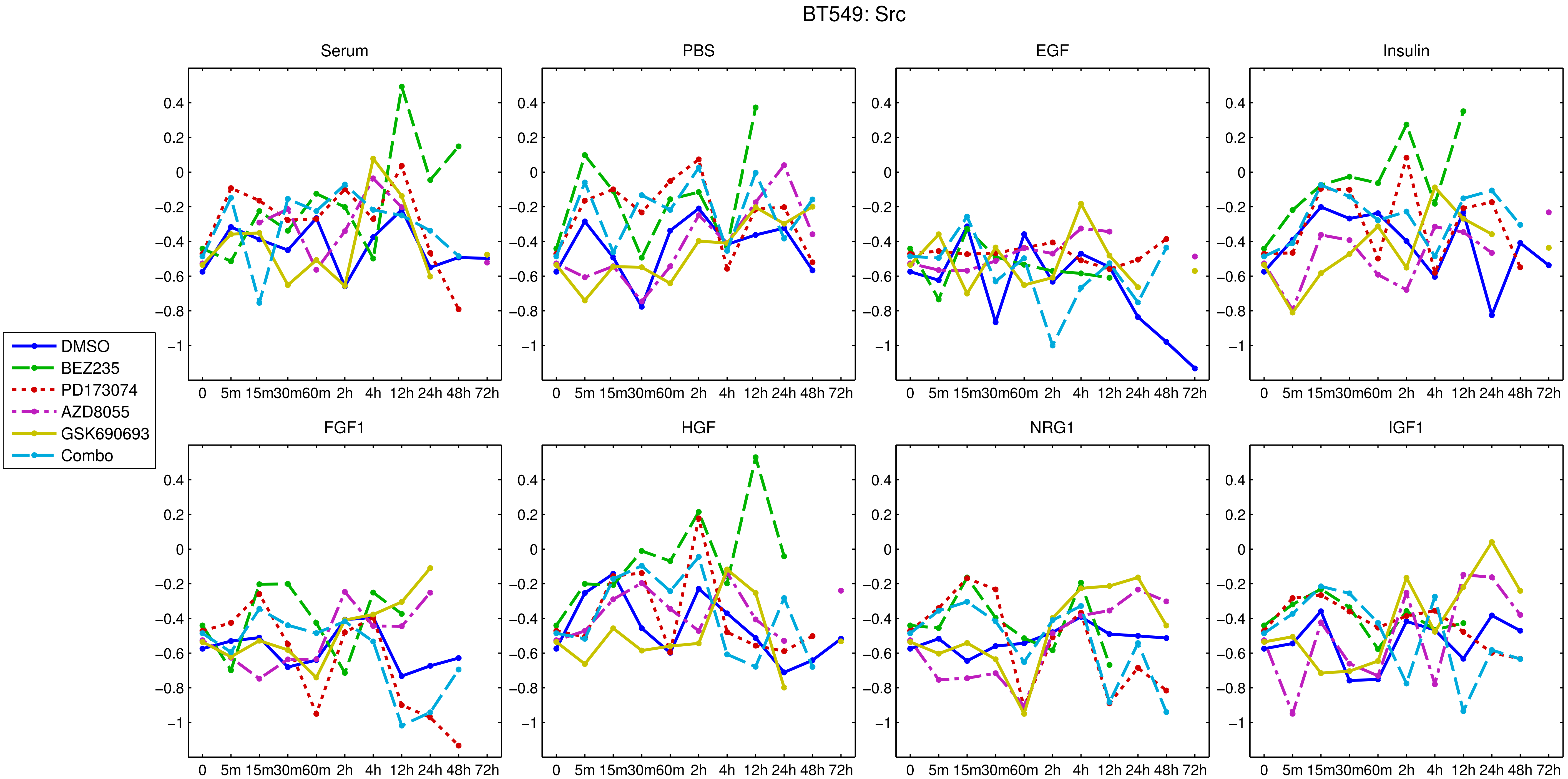
BT549: Src
Serum
PBS
EGF
Insulin
FGF1
HGF
NRG1
IGF1
DMSO
BEZ235
PD173074
AZD8055
GSK690693
Combo
0
5m
15m
30m
60m
2h
4h
12h
24h
48h
72h
0.4
0.2
−0.2
−0.4
−0.6
−0.8
−1

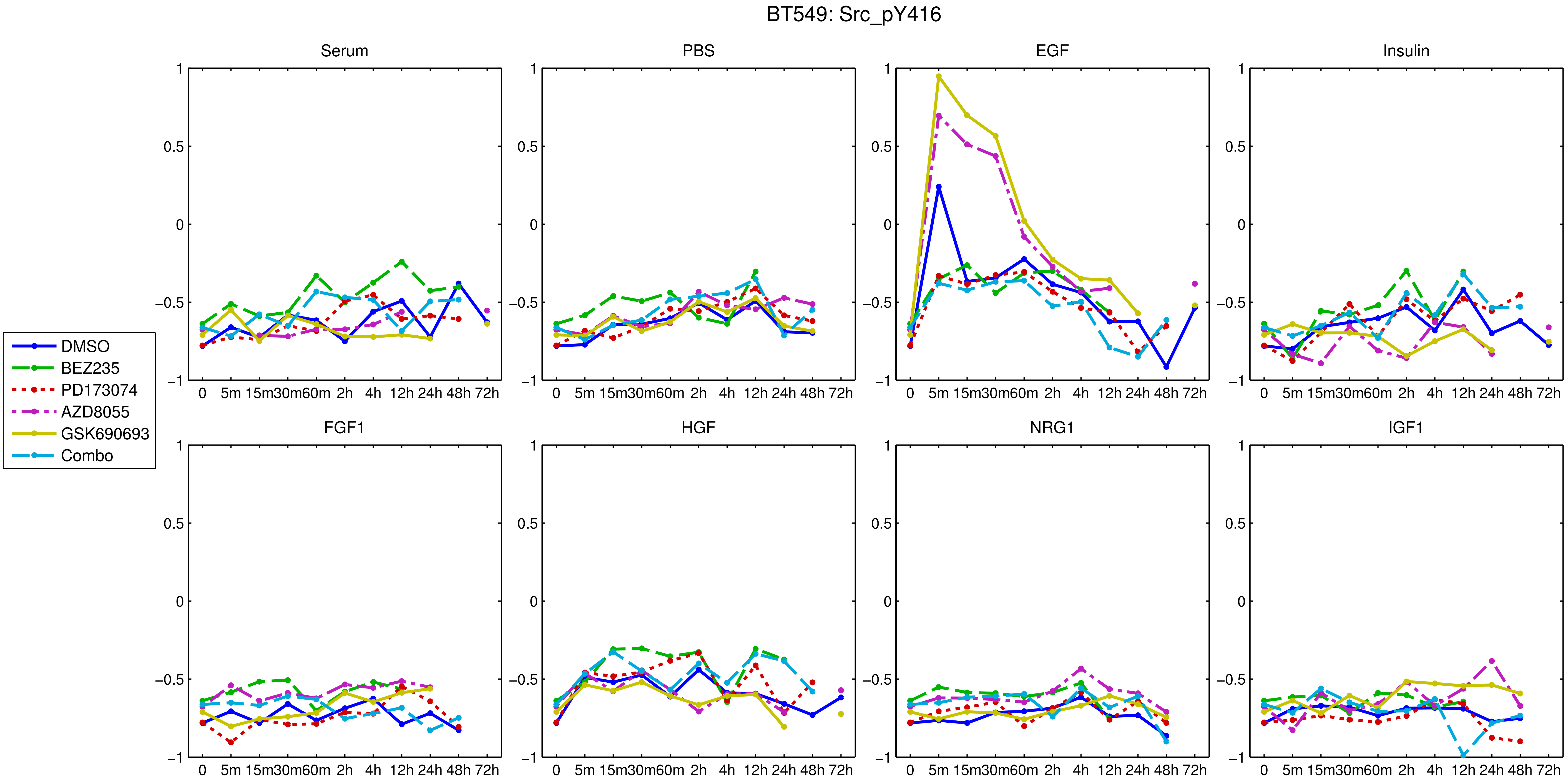

BT549: Src_pY416
Serum
PBS
EGF
Insulin
FGF1
HGF
NRG1
IGF1
DMSO
BEZ235
PD173074
AZD8055
GSK690693
Combo
1
0.5
0
−0.5
−1
0
5m
15m
30m
60m
2h
4h
12h
24h
48h
72h

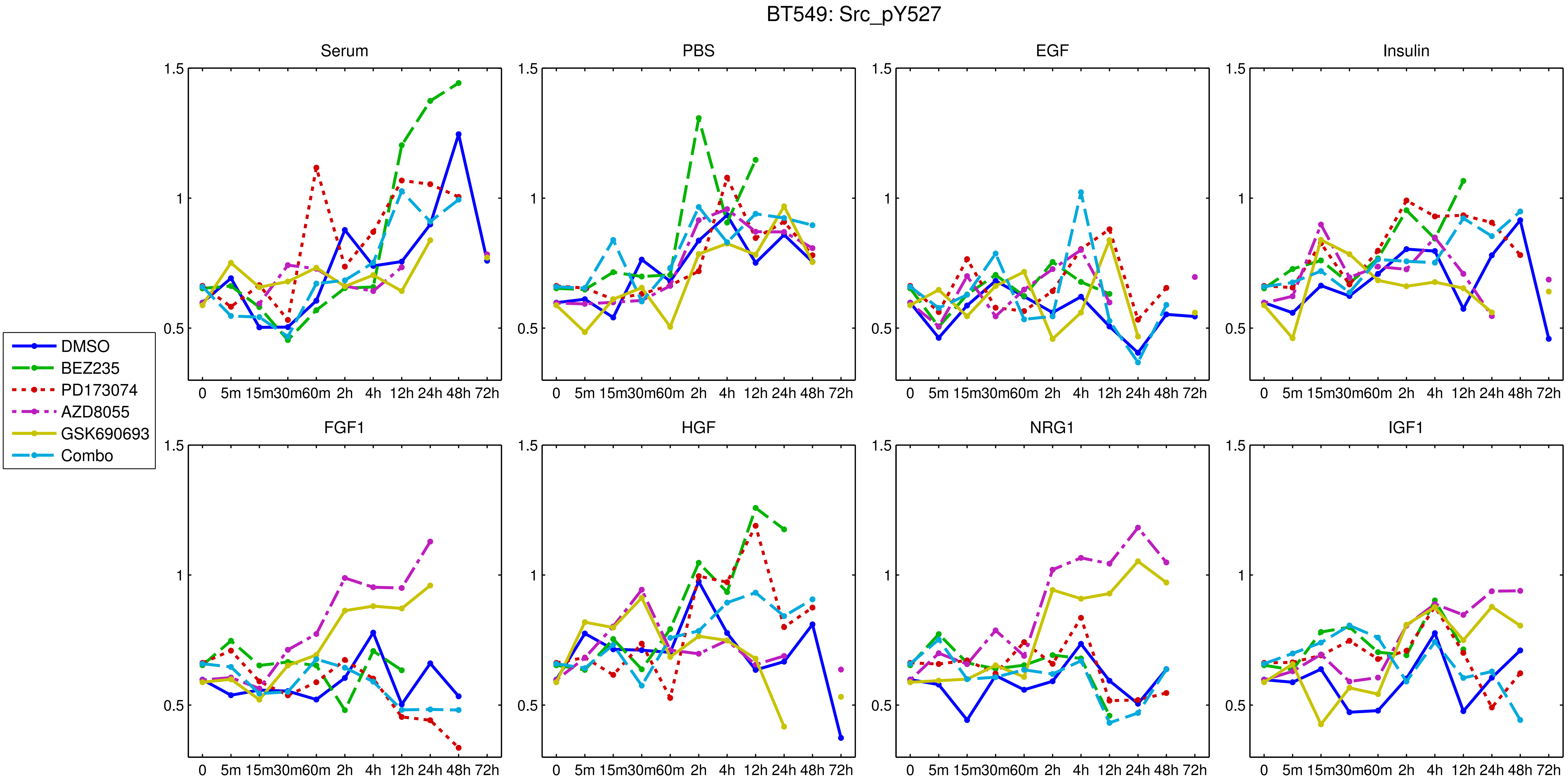

BT549: Src_pY527
Serum
PBS
EGF
Insulin
FGF1
HGF
NRG1
IGF1
1.5
1
0.5
0
5m
15m
30m
60m
2h
4h
12h
24h
48h
72h
DMSO
BEZ235
PD173074
AZD8055
GSK690693
Combo

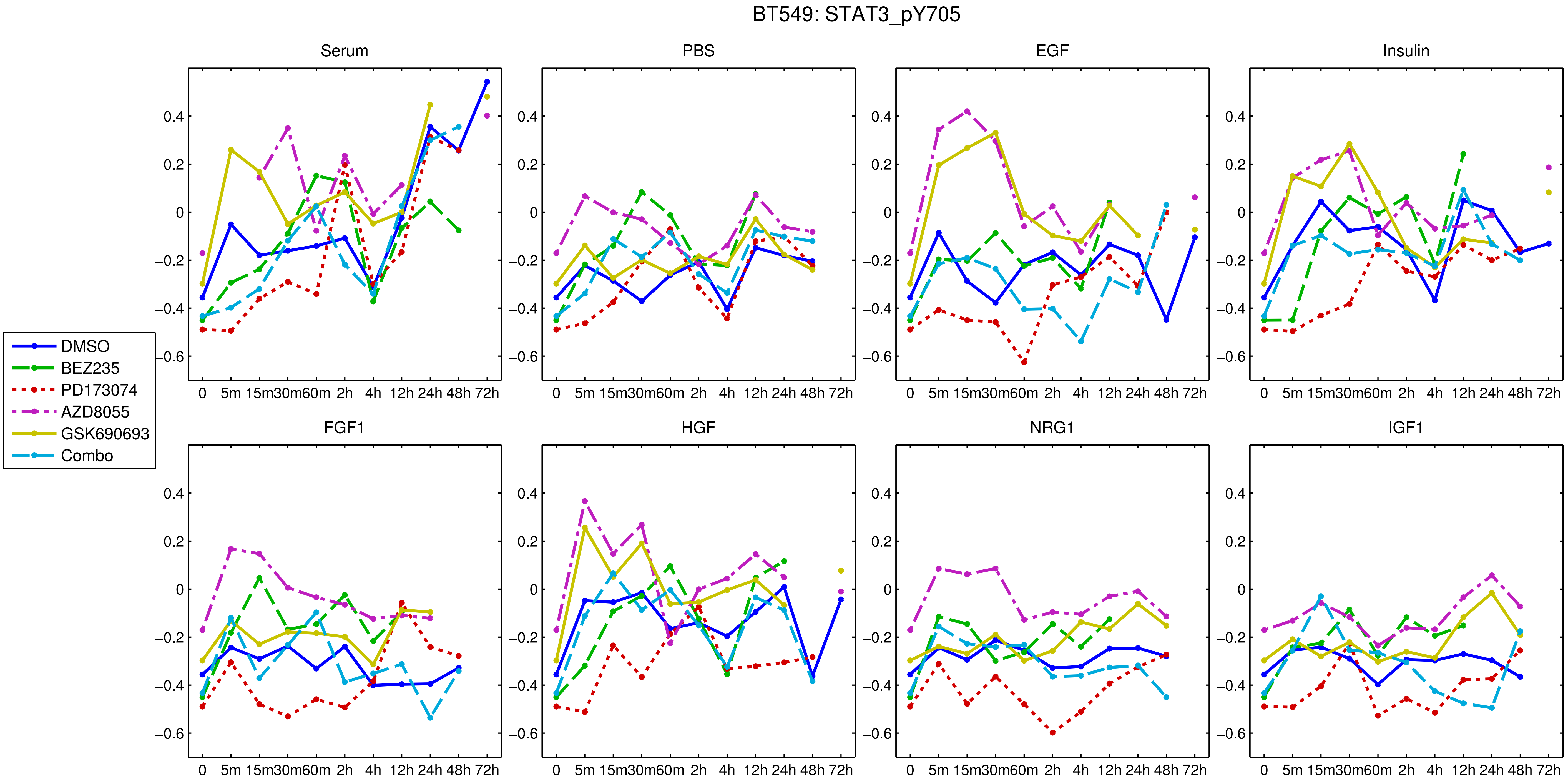
BT549: STAT3_pY705
Serum
PBS
EGF
Insulin
FGF1
HGF
NRG1
IGF1
DMSO
BEZ235
PD173074
AZD8055
GSK690693
Combo
0.4
0.2
0
−0.2
−0.4
−0.6
0 5m 15m 30m 60m 2h 4h 12h 24h 48h 72h

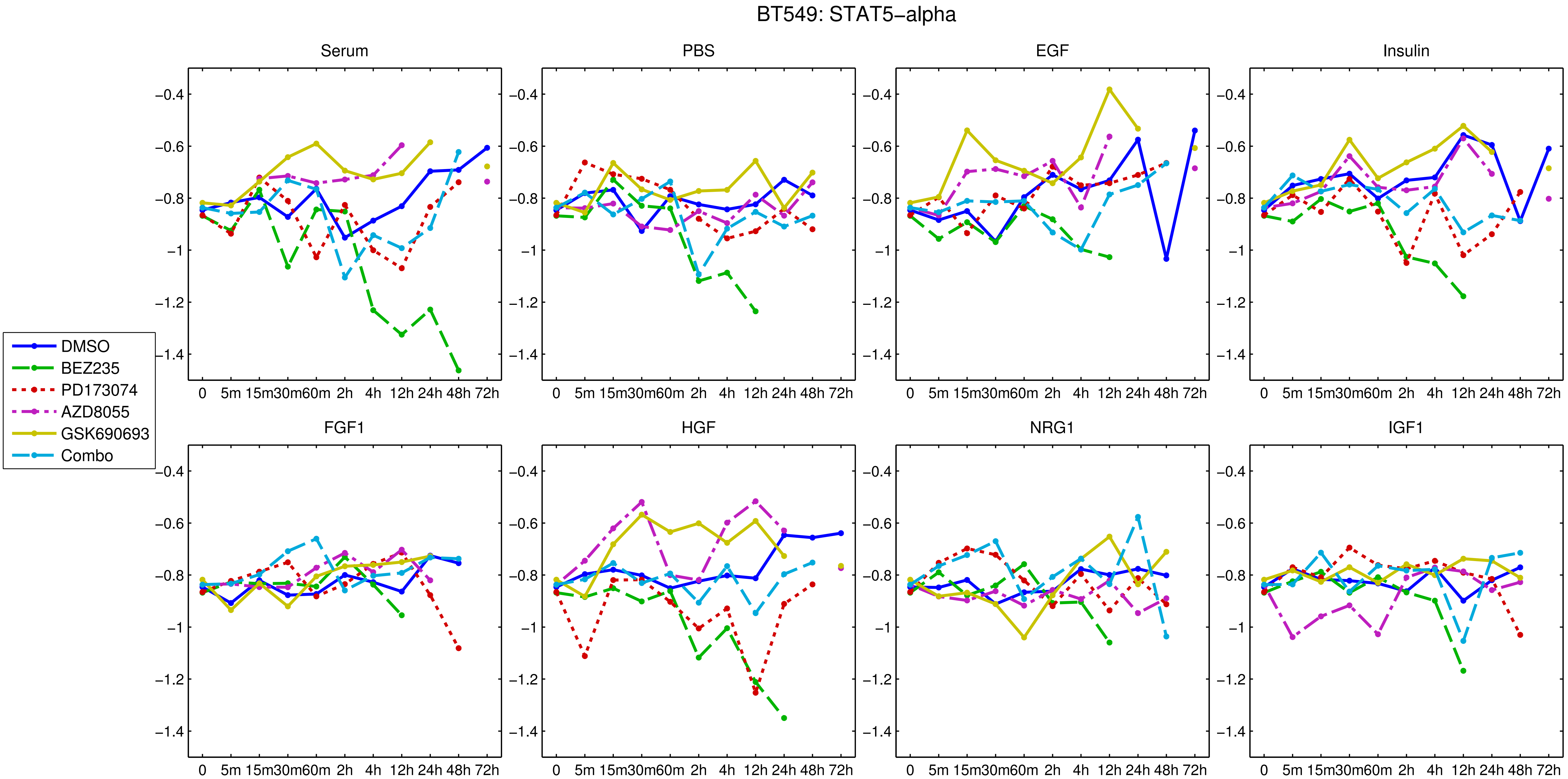
BT549: STAT5−alpha
Serum
PBS
EGF
Insulin
FGF1
HGF
NRG1
IGF1
DMSO
BEZ235
PD173074
AZD8055
GSK690693
Combo
−0.4
−0.6
−0.8
−1
−1.2
−1.4
0
5m
15m
30m
60m
2h
4h
12h
24h
48h
72h

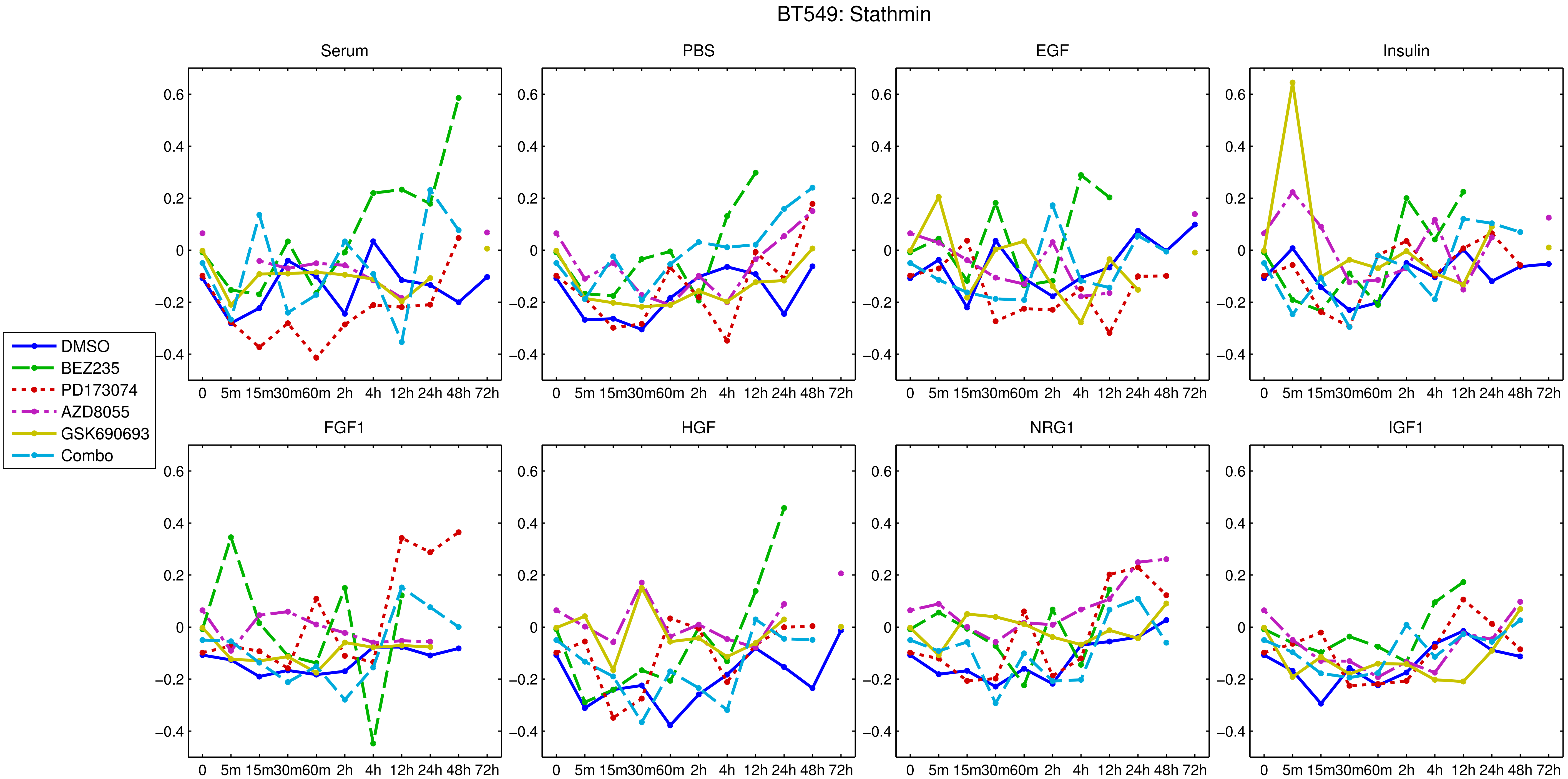
BT549: Stathmin
Serum
PBS
EGF
Insulin
FGF1
HGF
NRG1
IGF1
DMSO
BEZ235
PD173074
AZD8055
GSK690693
Combo
0.6
0.4
0.2
0
−0.2
−0.4
0
5m
15m
30m
60m
2h
4h
12h
24h
48h
72h

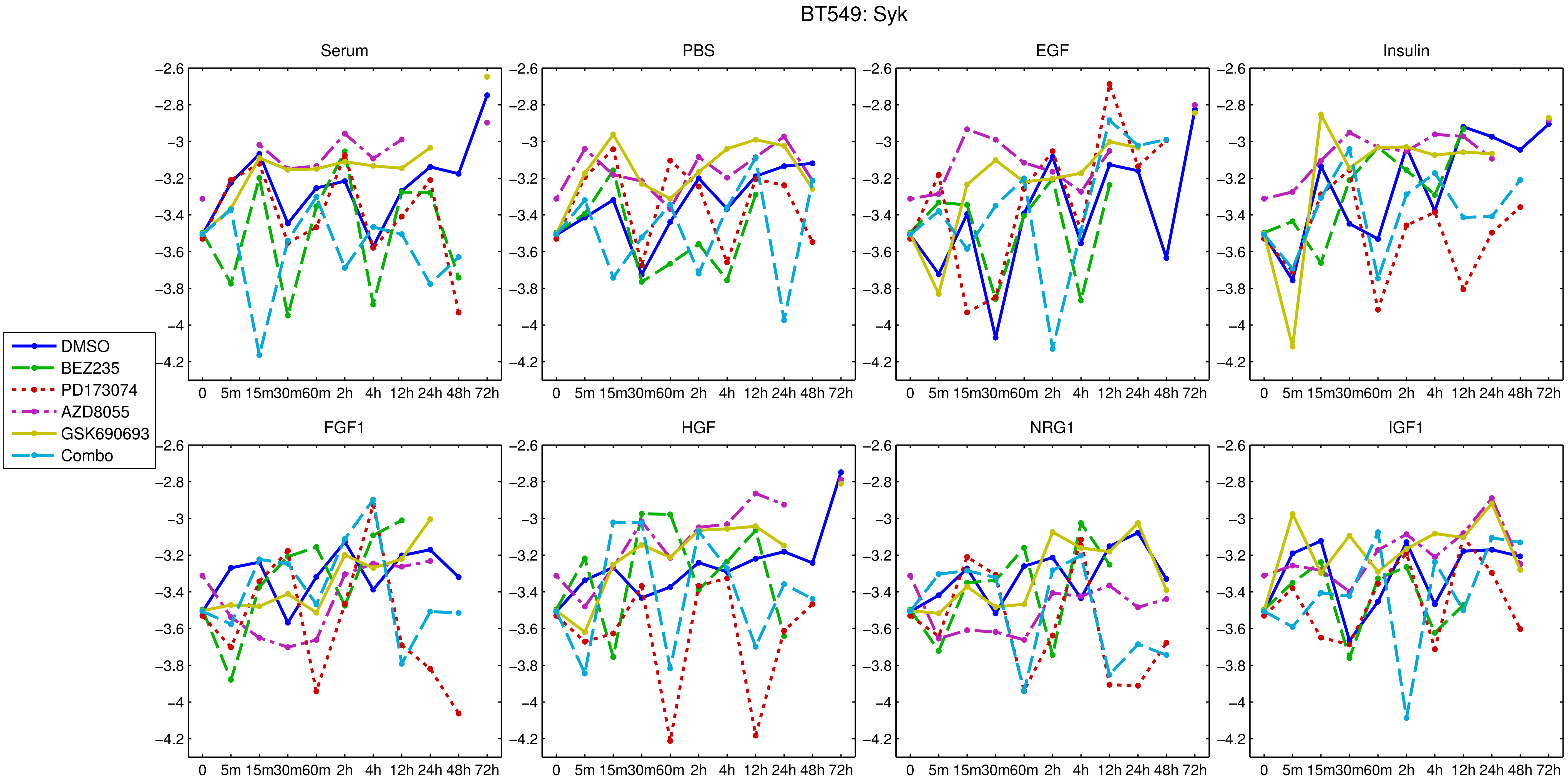
BT549: Syk
Serum
PBS
EGF
Insulin
FGF1
HGF
NRG1
IGF1
−2.6
−2.8
−3
−3.2
−3.4
−3.6
−3.8
−4
−4.2
0
5m
15m
30m
60m
2h
4h
12h
24h
48h
72h
DMSO
BEZ235
PD173074
AZD8055
GSK690693
Combo

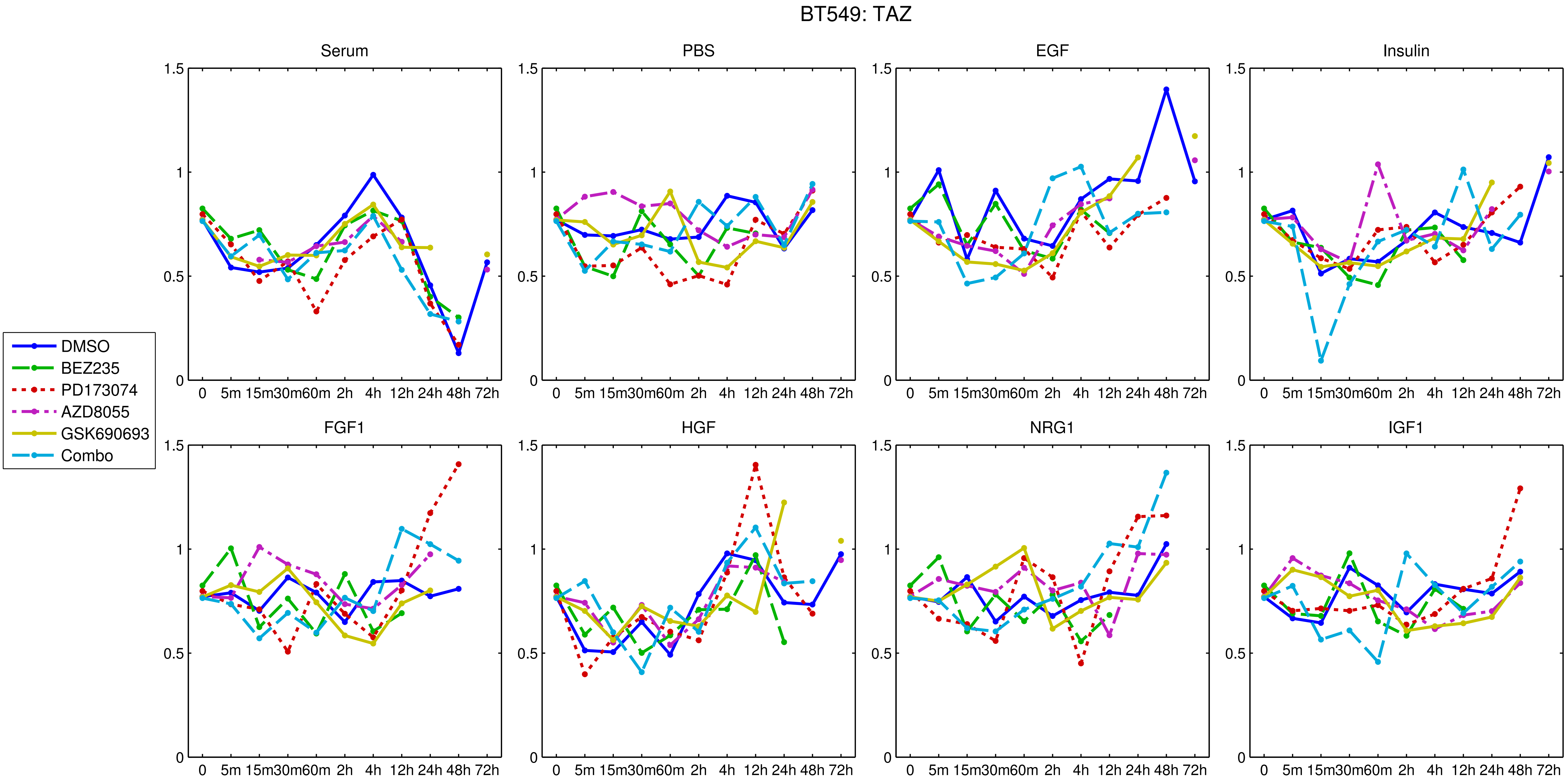
BT549: TAZ
Serum
PBS
EGF
Insulin
FGF1
HGF
NRG1
IGF1
DMSO
BEZ235
PD173074
AZD8055
GSK690693
Combo
0
5m
15m
30m
60m
2h
4h
12h
24h
48h
72h
0
0.5
1
1.5

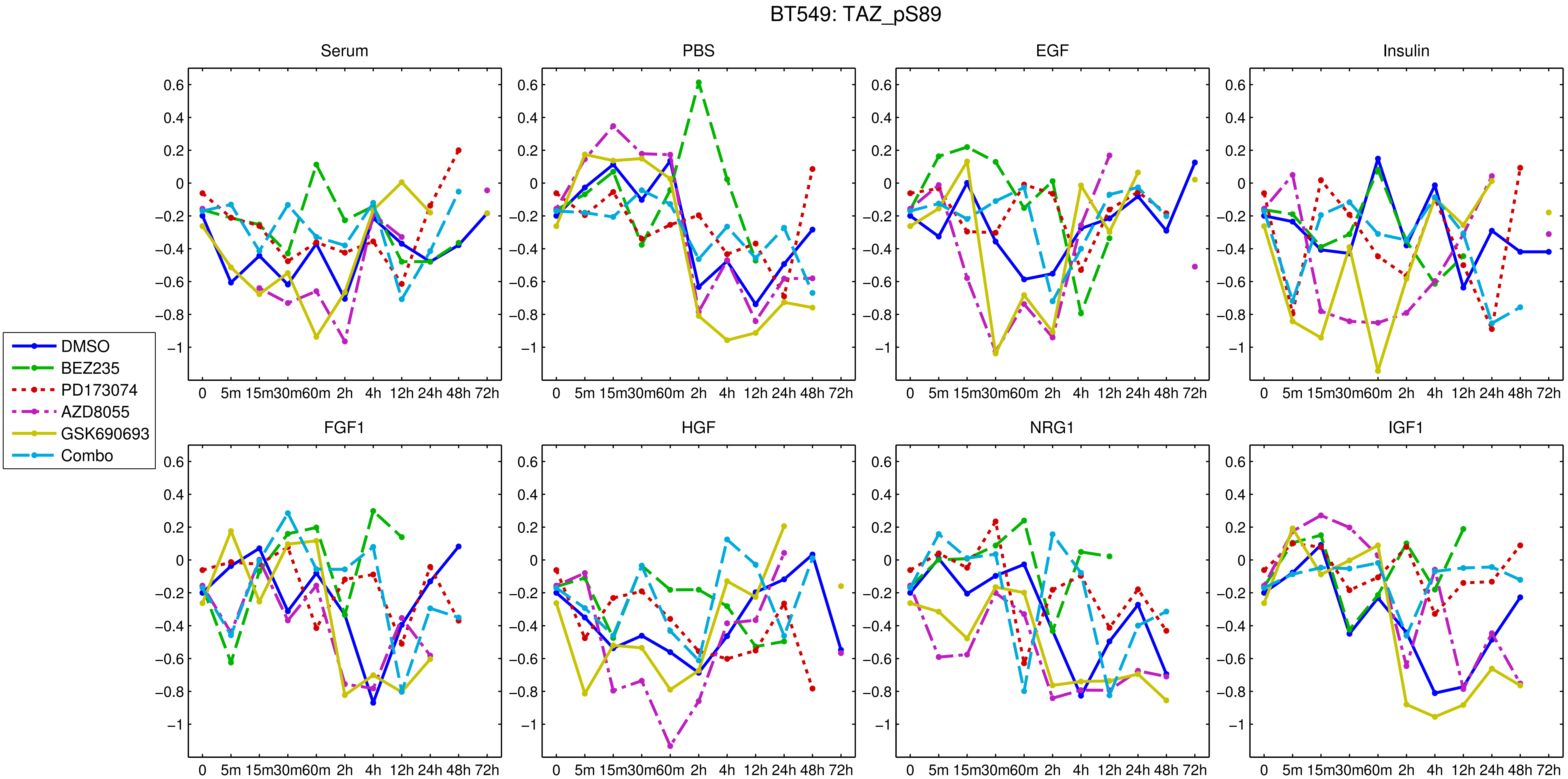
BT549: TAZ_pS89
Serum
PBS
EGF
Insulin
FGF1
HGF
NRG1
IGF1
DMSO
BEZ235
PD173074
AZD8055
GSK690693
Combo
0.6
0.4
0.2
0
−0.2
−0.4
−0.6
−0.8
−1
0
5m
15m
30m
60m
2h
4h
12h
24h
48h
72h

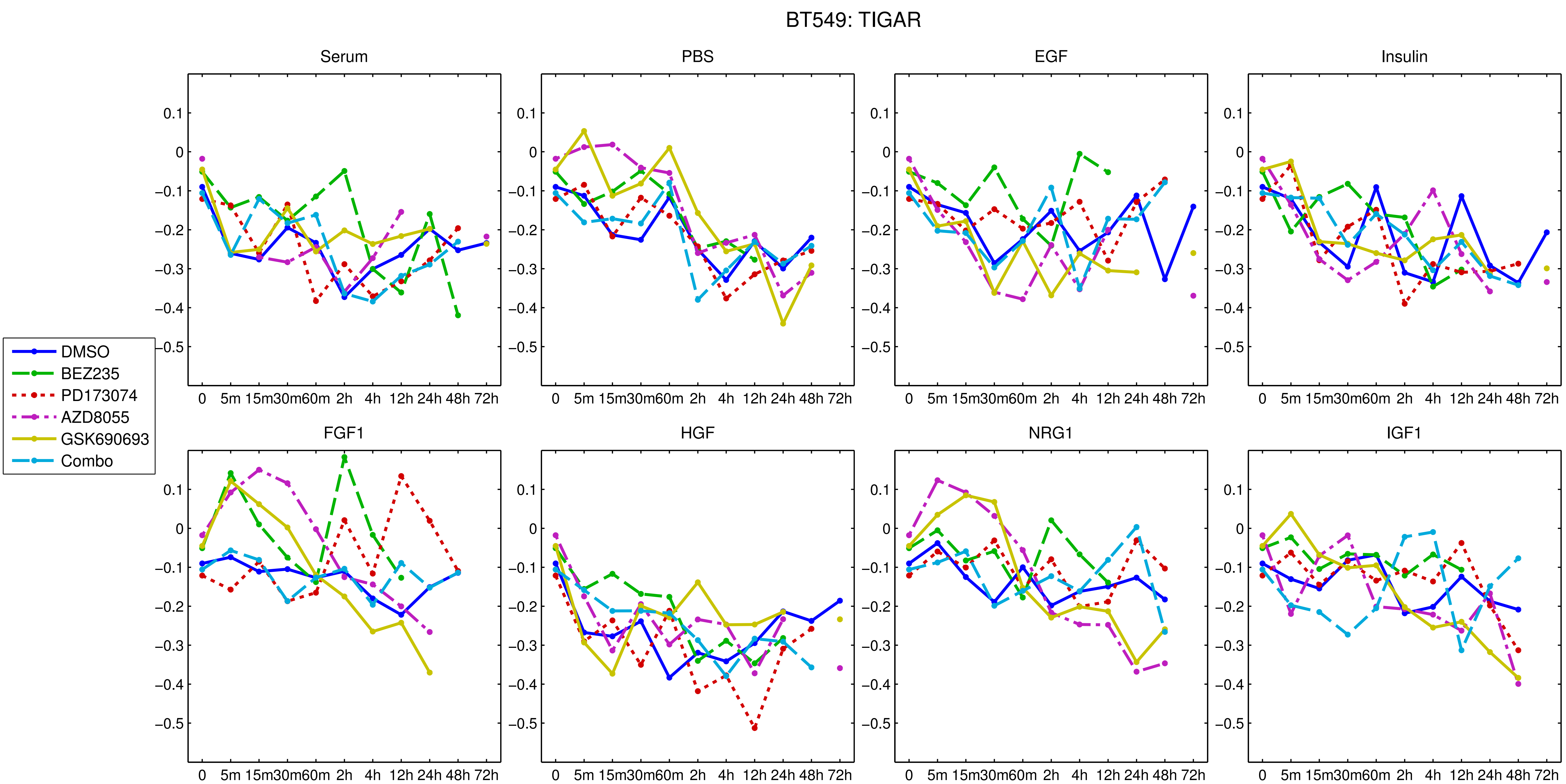
BT549: TIGAR
Serum
PBS
EGF
Insulin
FGF1
HGF
NRG1
IGF1
0.1
0
−0.1
−0.2
−0.3
−0.4
−0.5
0 5m 15m 30m 60m 2h 4h 12h 24h 48h 72h
DMSO
BEZ235
PD173074
AZD8055
GSK690693
Combo

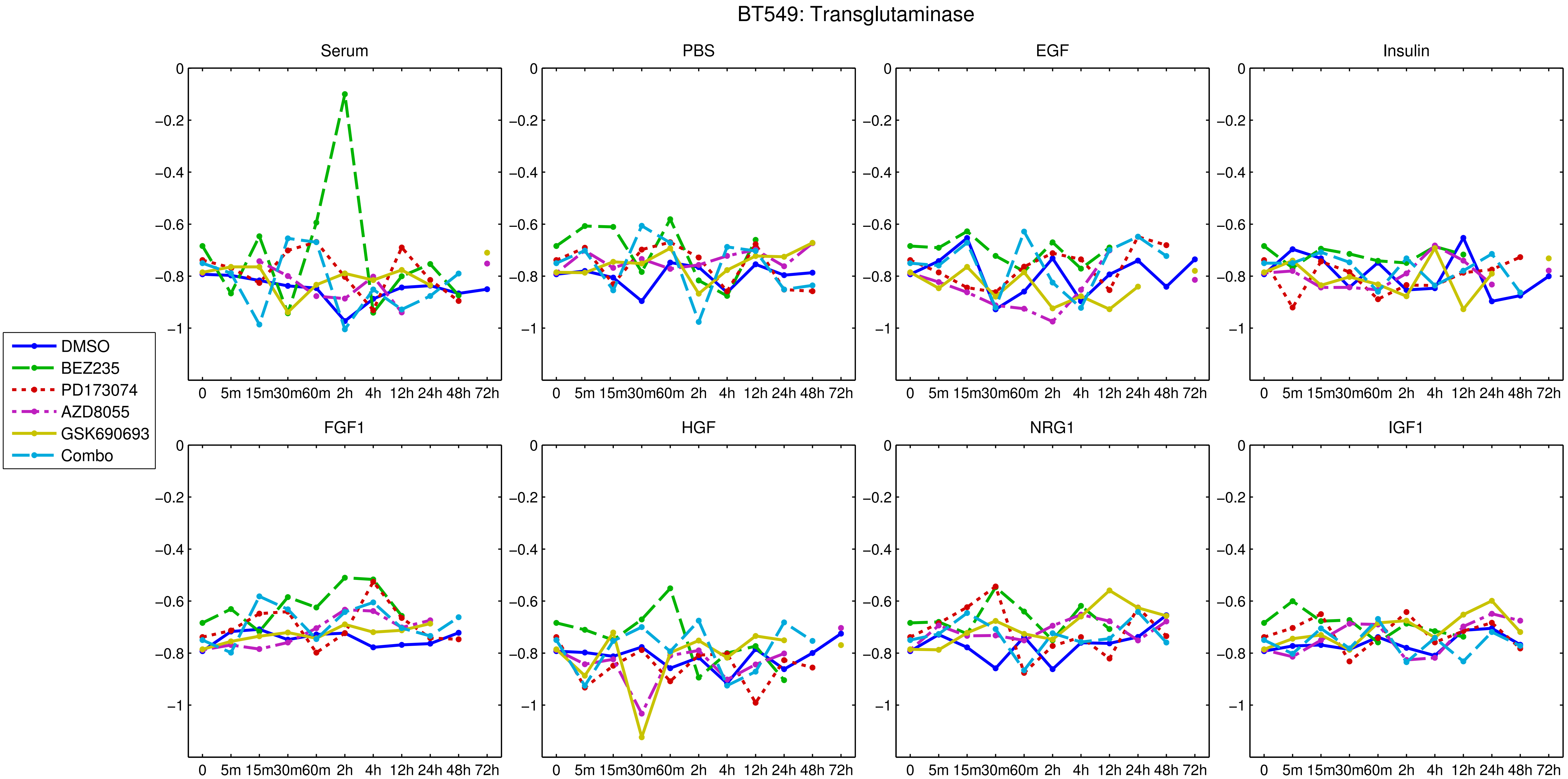
BT549: Transglutaminase
Serum
PBS
EGF
Insulin
FGF1
HGF
NRG1
IGF1
0
−0.2
−0.4
−0.6
−0.8
−1
0
5m
15m
30m
60m
2h
4h
12h
24h
48h
72h
DMSO
BEZ235
PD173074
AZD8055
GSK690693
Combo

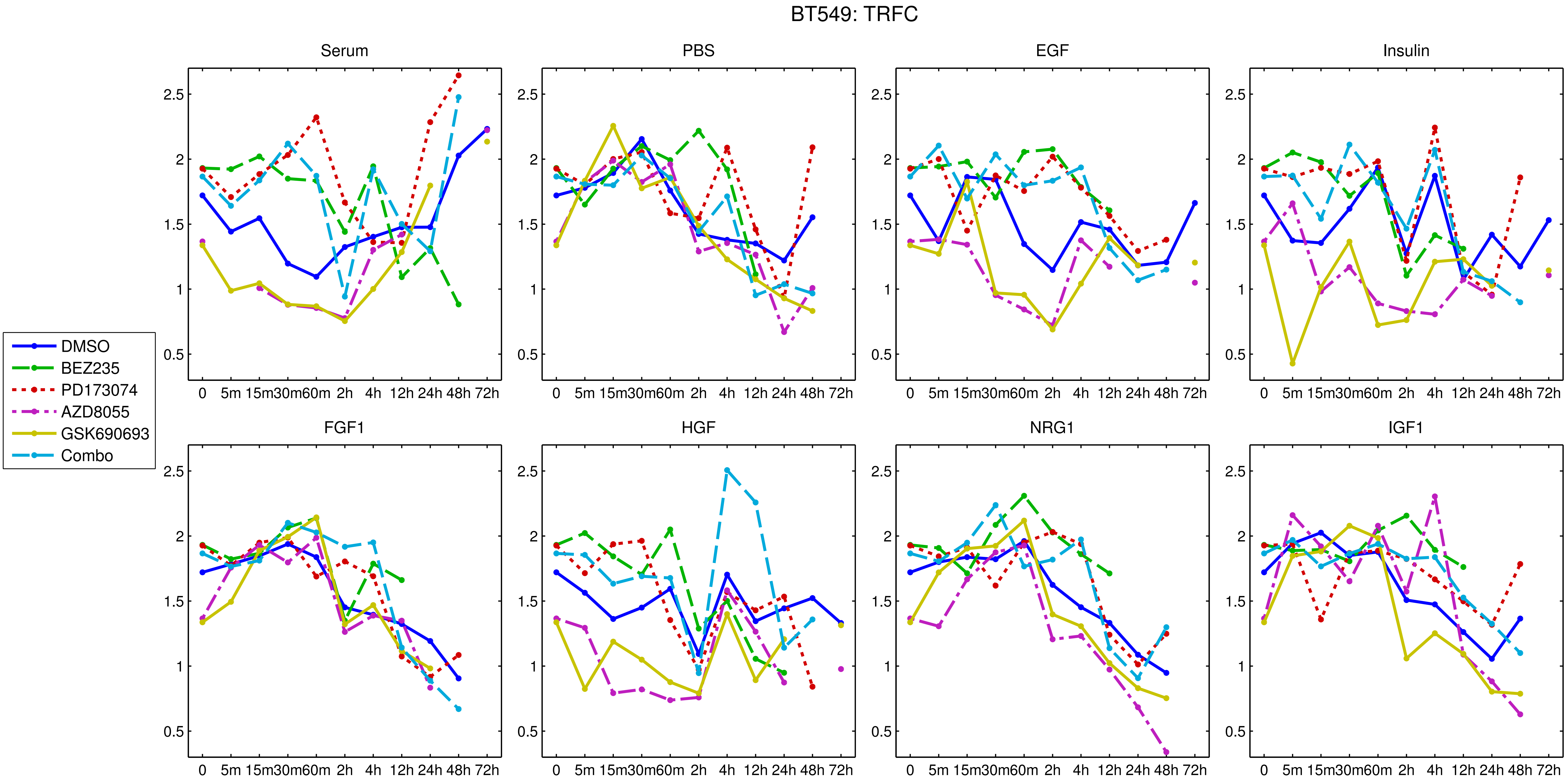
BT549: TRFC
Serum
PBS
EGF
Insulin
FGF1
HGF
NRG1
IGF1
DMSO
BEZ235
PD173074
AZD8055
GSK690693
Combo
2.5
2
1.5
1
0.5
0
5m
15m
30m
60m
2h
4h
12h
24h
48h
72h

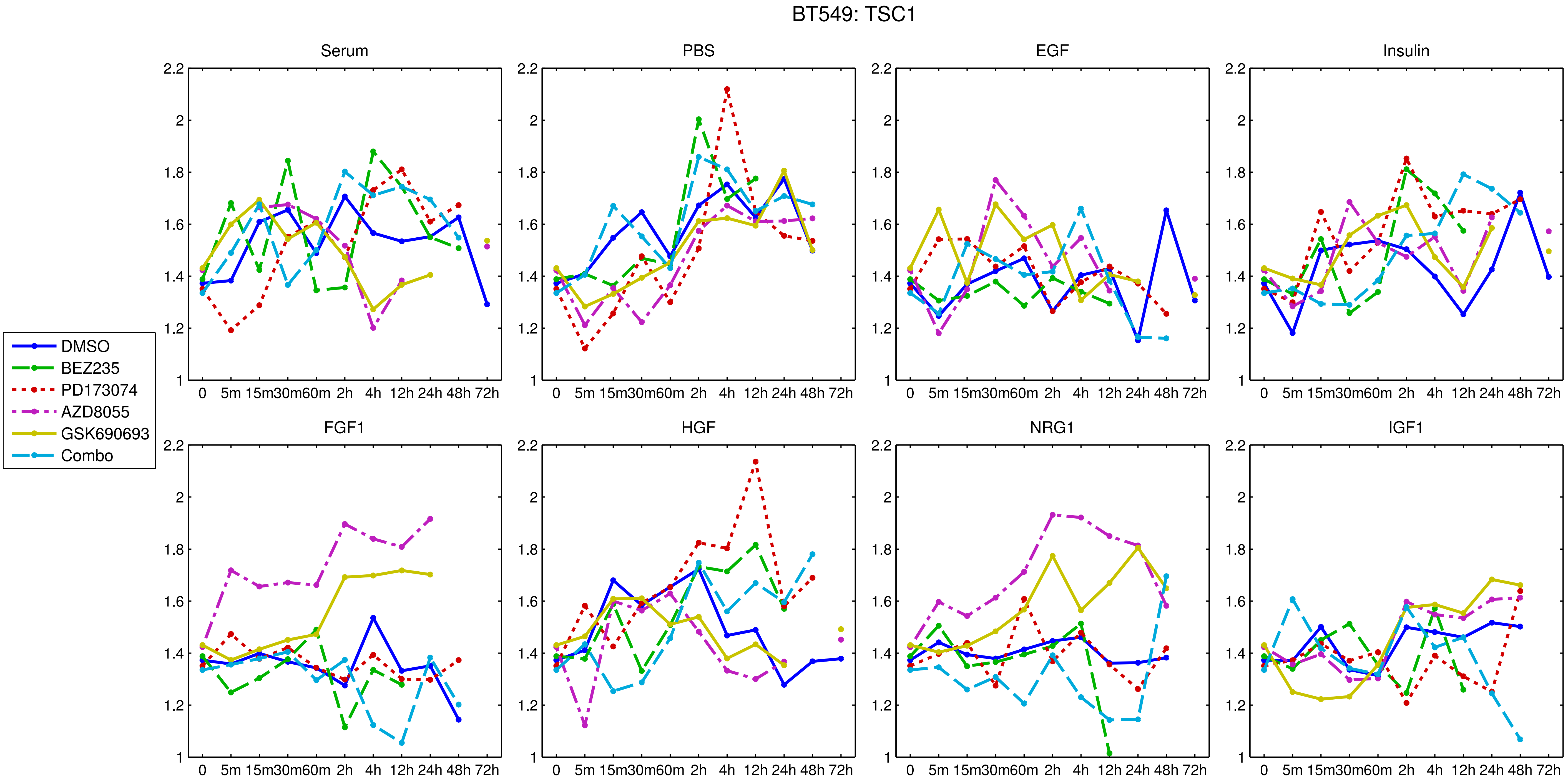
BT549: TSC1
Serum
PBS
EGF
Insulin
FGF1
HGF
NRG1
IGF1
DMSO
BEZ235
PD173074
AZD8055
GSK690693
Combo
0
5m
15m
30m
60m
2h
4h
12h
24h
48h
72h
1
1.2
1.4
1.6
1.8
2
2.2

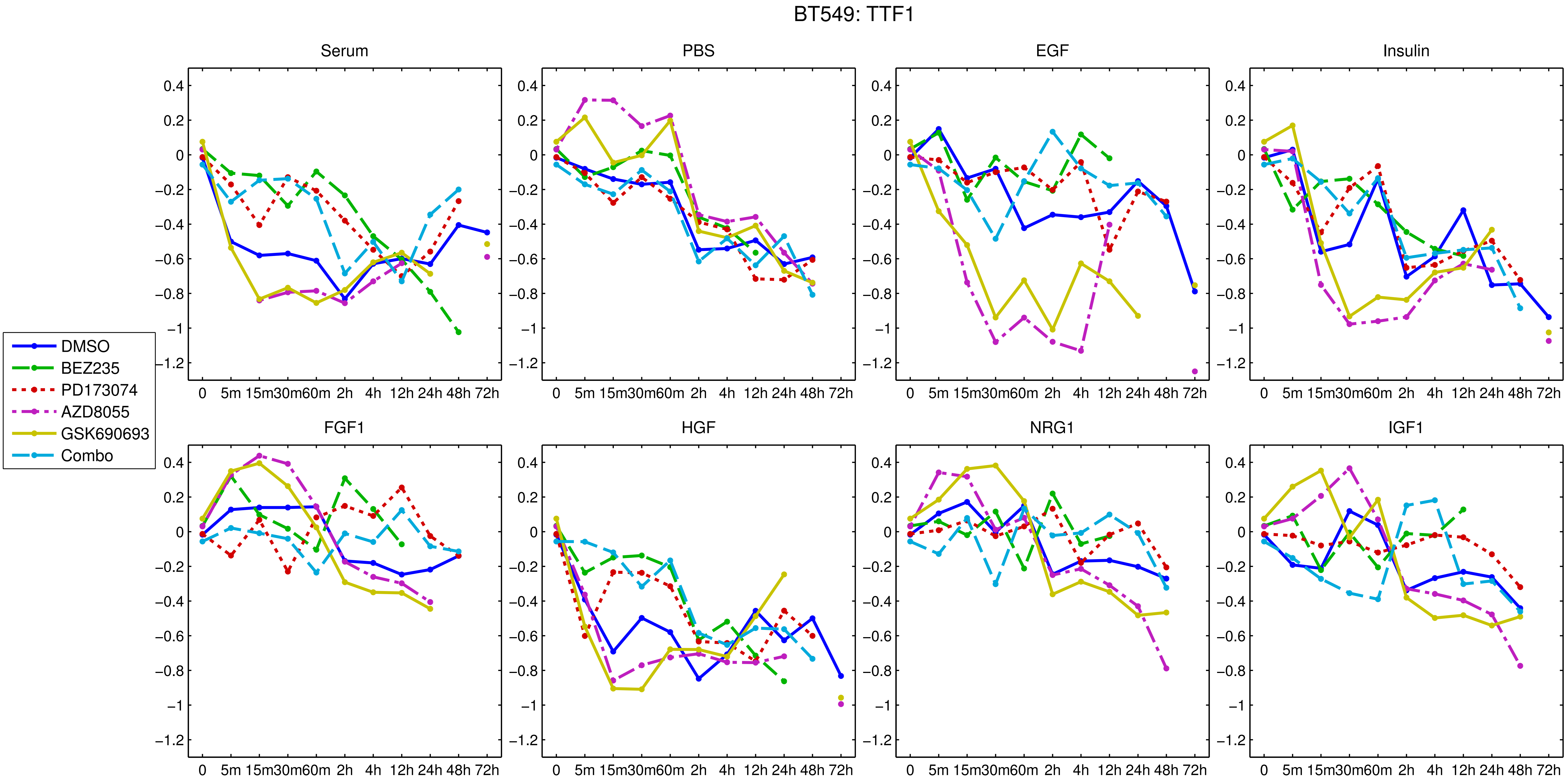
BT549: TTF1
Serum
PBS
EGF
Insulin
FGF1
HGF
NRG1
IGF1
0.4
0.2
0
−0.2
−0.4
−0.6
−0.8
−1
−1.2
0 5m 15m 30m 60m 2h 4h 12h 24h 48h 72h
DMSO
BEZ235
PD173074
AZD8055
GSK690693
Combo

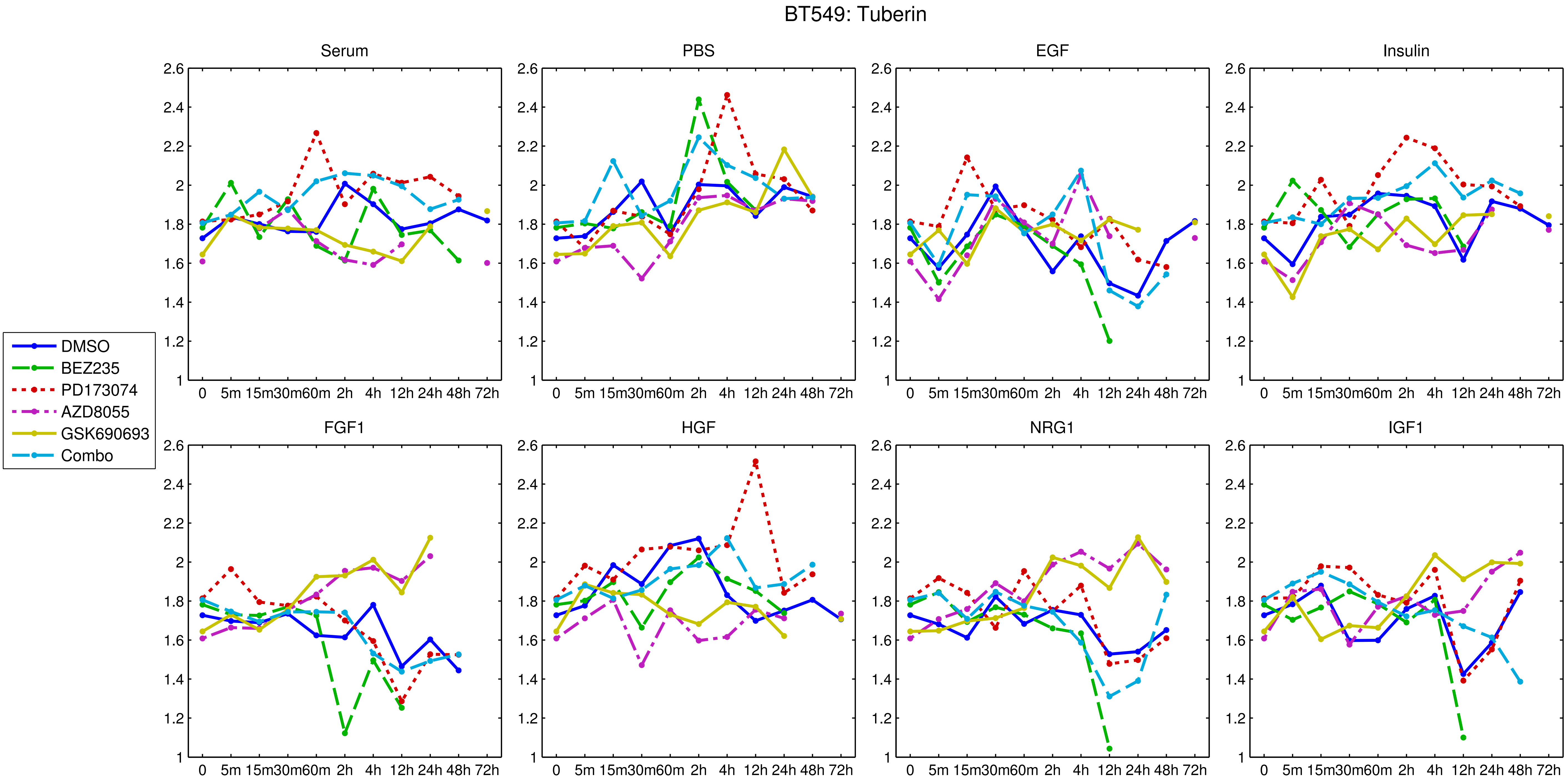
BT549: Tuberin
Serum
PBS
EGF
Insulin
FGF1
HGF
NRG1
IGF1
DMSO
BEZ235
PD173074
AZD8055
GSK690693
Combo
2.6
2.4
2.2
2
1.8
1.6
1.4
1.2
1
0 5m 15m30m60m 2h 4h 12h 24h 48h 72h

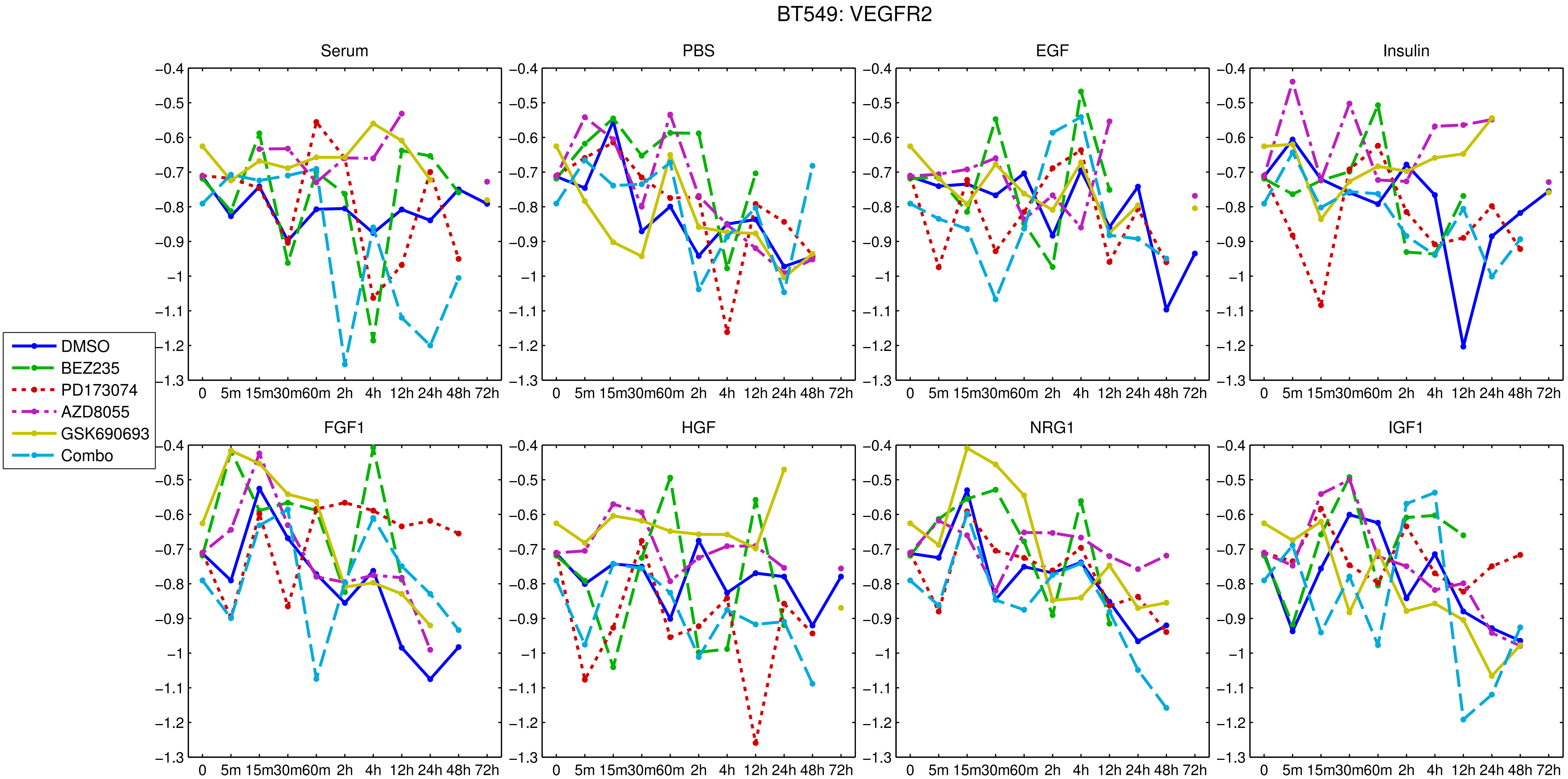
BT549: VEGFR2
Serum
PBS
EGF
Insulin
FGF1
HGF
NRG1
IGF1
DMSO
BEZ235
PD173074
AZD8055
GSK690693
Combo
−0.4
−0.5
−0.6
−0.7
−0.8
−0.9
−1
−1.1
−1.2
−1.3
0
5m
15m
30m
60m
2h
4h
12h
24h
48h
72h

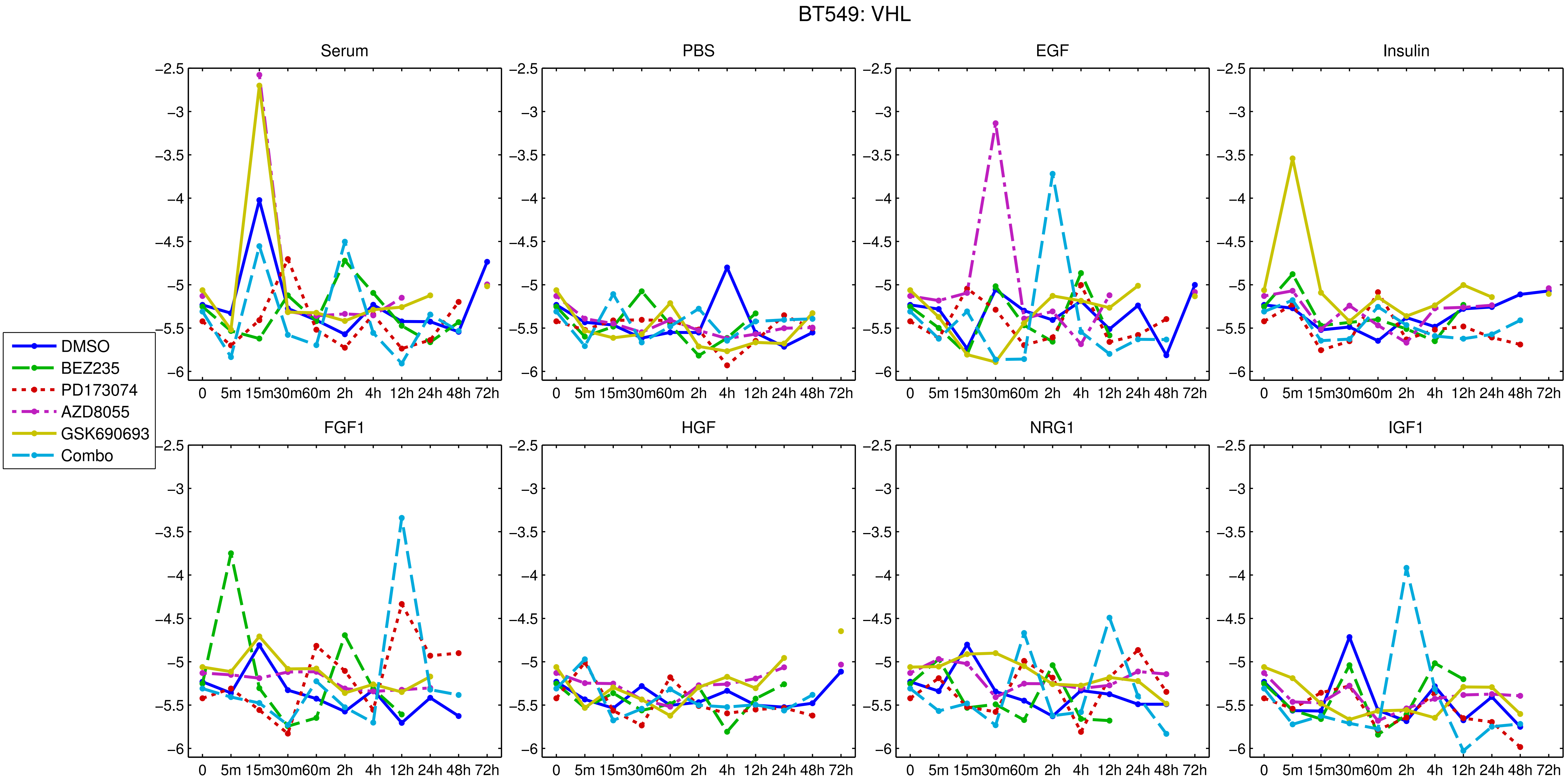
BT549: VHL
Serum
PBS
EGF
Insulin
FGF1
HGF
NRG1
IGF1
DMSO
BEZ235
PD173074
AZD8055
GSK690693
Combo
−2.5
−3
−3.5
−4
−4.5
−5
−5.5
−6
0 5m 15m 30m 60m 2h 4h 12h 24h 48h 72h

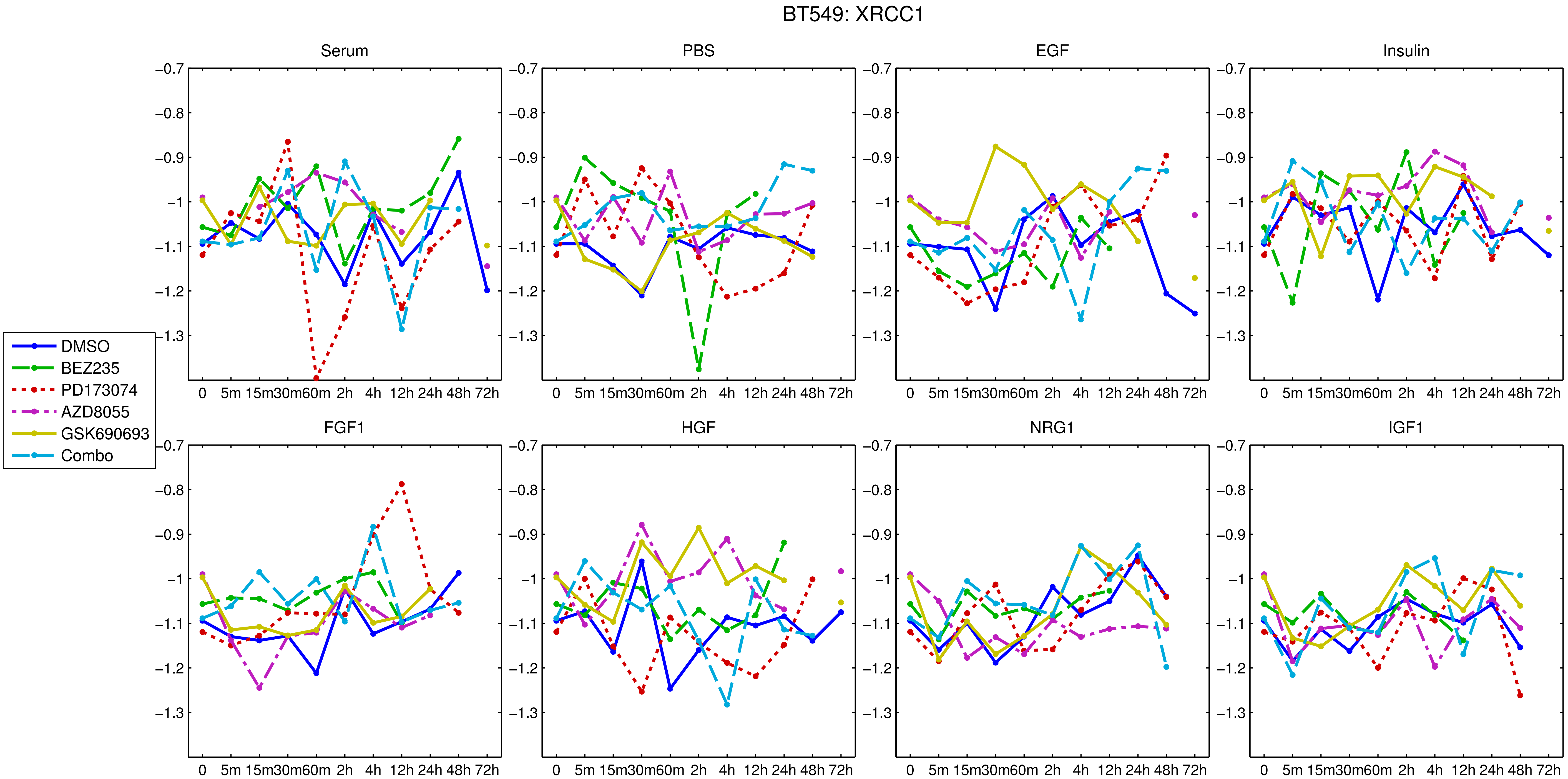
BT549: XRCC1
Serum
PBS
EGF
Insulin
FGF1
HGF
NRG1
IGF1
DMSO
BEZ235
PD173074
AZD8055
GSK690693
Combo
0 5m 15m 30m 60m 2h 4h 12h 24h 48h 72h
−0.7
−0.8
−0.9
−1
−1.1
−1.2
−1.3

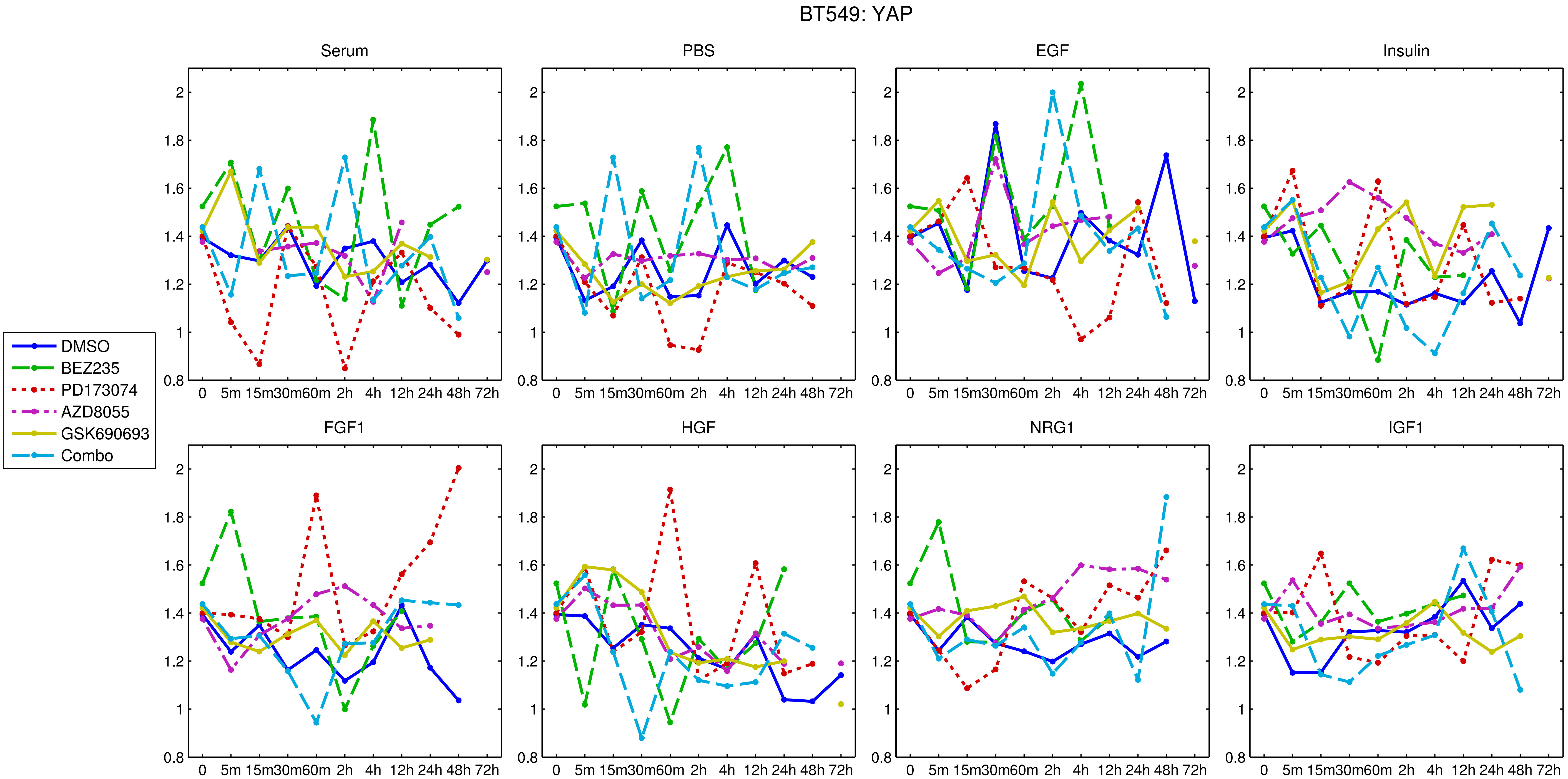
BT549: YAP
Serum
PBS
EGF
Insulin
FGF1
HGF
NRG1
IGF1
DMSO
BEZ235
PD173074
AZD8055
GSK690693
Combo
0.8
1
1.2
1.4
1.6
1.8
2
0 5m 15m30m60m 2h 4h 12h 24h 48h 72h

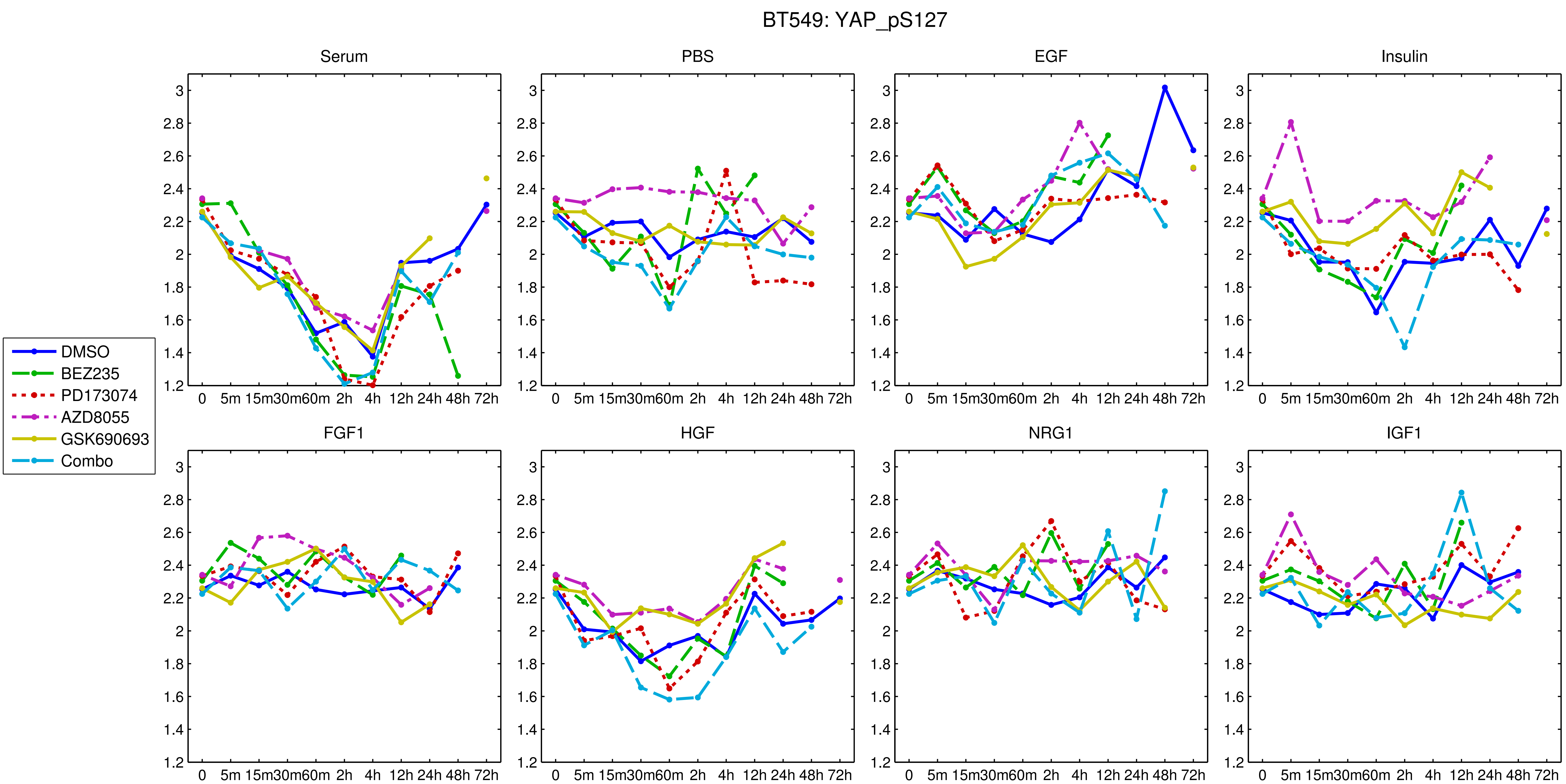

BT549: YAP_pS127
Serum
PBS
EGF
Insulin
FGF1
HGF
NRG1
IGF1
DMSO
BEZ235
PD173074
AZD8055
GSK690693
Combo
0 5m 15m 30m 60m 2h 4h 12h 24h 48h 72h

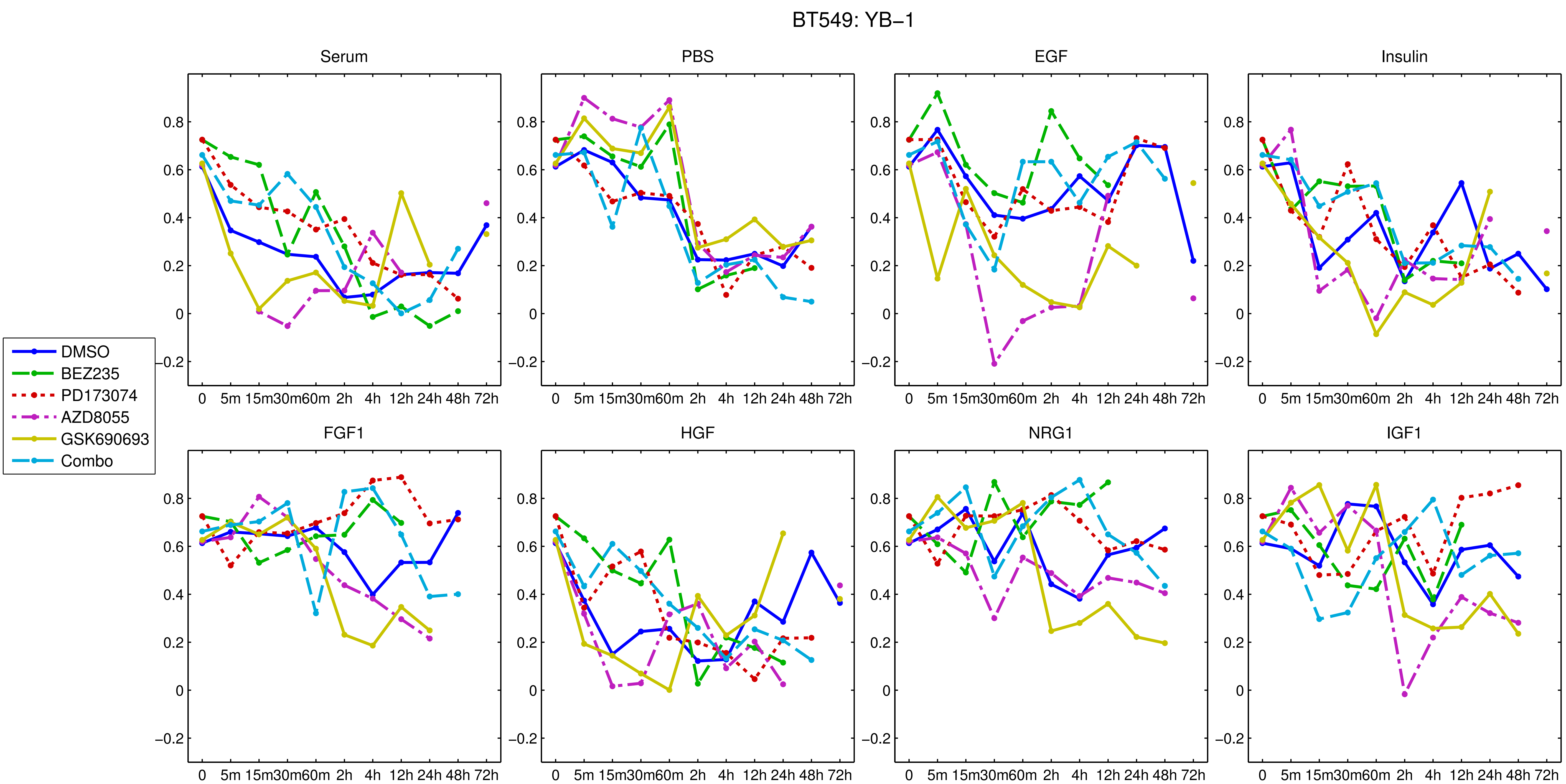

BT549: YB−1
Serum
PBS
EGF
Insulin
FGF1
HGF
NRG1
IGF1
DMSO
BEZ235
PD173074
AZD8055
GSK690693
Combo
0.8
0.6
0.4
0.2
0
−0.2
0 5m 15m 30m 60m 2h 4h 12h 24h 48h 72h

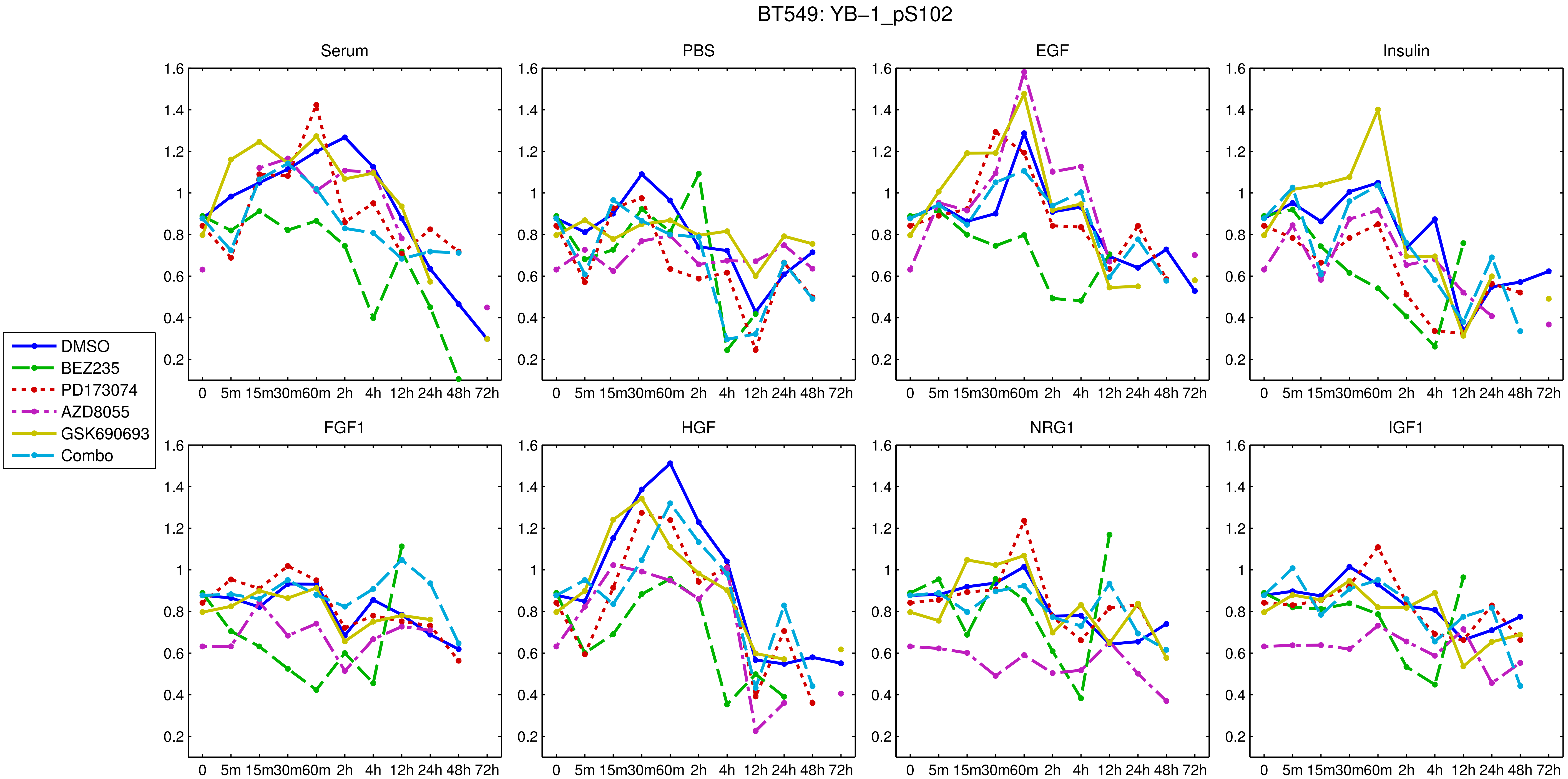
BT549: YB−1_pS102
Serum
PBS
EGF
Insulin
FGF1
HGF
NRG1
IGF1
DMSO
BEZ235
PD173074
AZD8055
GSK690693
Combo
1.6
1.4
1.2
1
0.8
0.6
0.4
0.2
0 5m 15m 30m 60m 2h 4h 12h 24h 48h 72h
