# Supplementary material for: Context Specificity in Causal Signaling Networks Revealed by Phosphoprotein Profiling
Source: Cell Syst. 2017 Jan 25;4(1):73–83.e10. doi: 10.1016/j.cels.2016.11.013 (PMC5279869; doi:10.1016/j.cels.2016.11.013)
Supplement: Data S2. RPPA Data Time-Course Plots, Related to STAR Methods — A zip archive containing time-course plots of the reverse-phase protein array data generated in this study. See the README file included in the zip archive for further details. [file mmc6.zip › DataS2/MCF7.pdf]

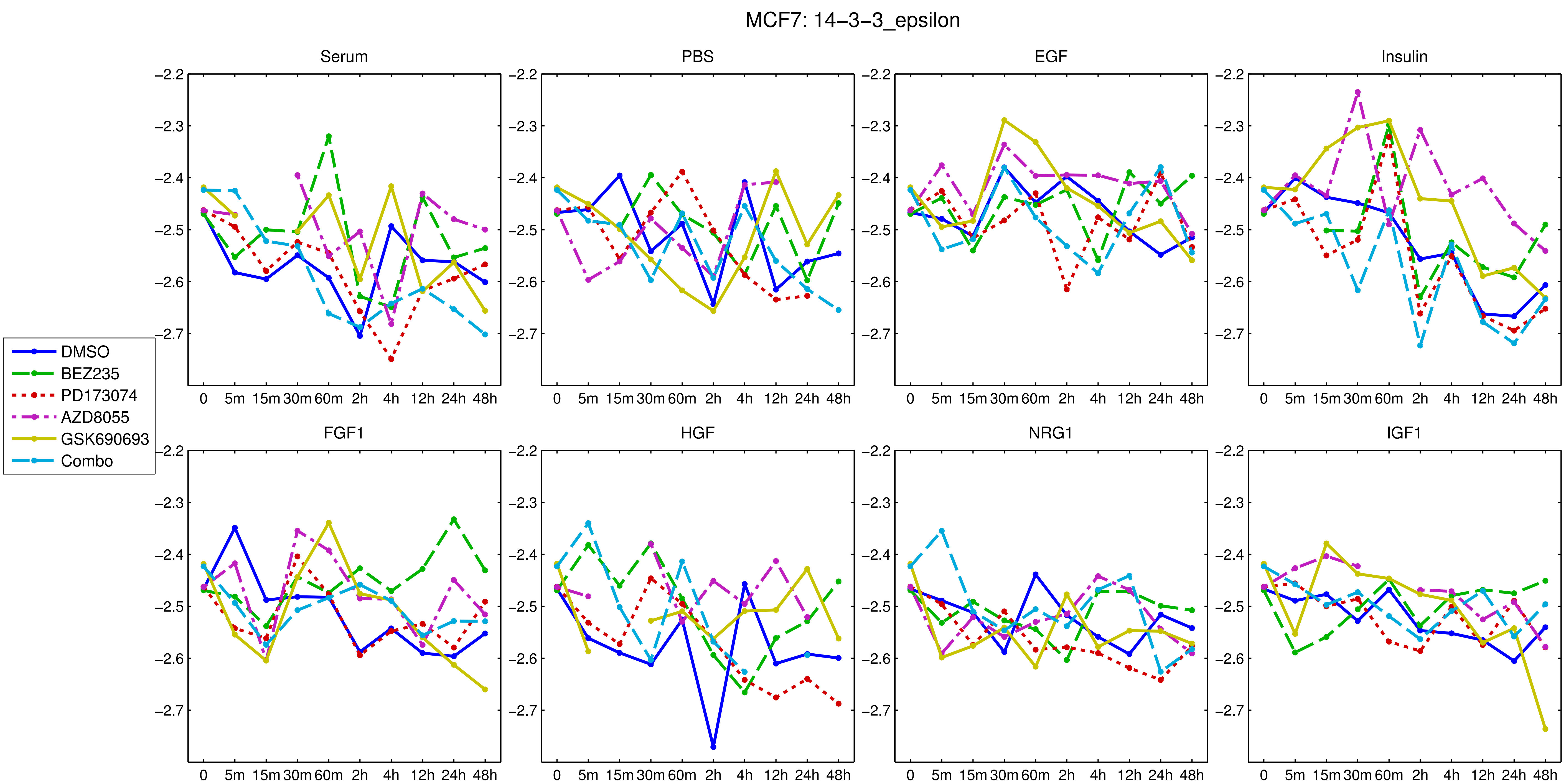
MCF7: 14−3−3_epsilon
Serum
PBS
EGF
Insulin
FGF1
HGF
NRG1
IGF1
DMSO
BEZ235
PD173074
AZD8055
GSK690693
Combo
−2.2
−2.3
−2.4
−2.5
−2.6
−2.7
0
5m
15m
30m
60m
2h
4h
12h
24h
48h

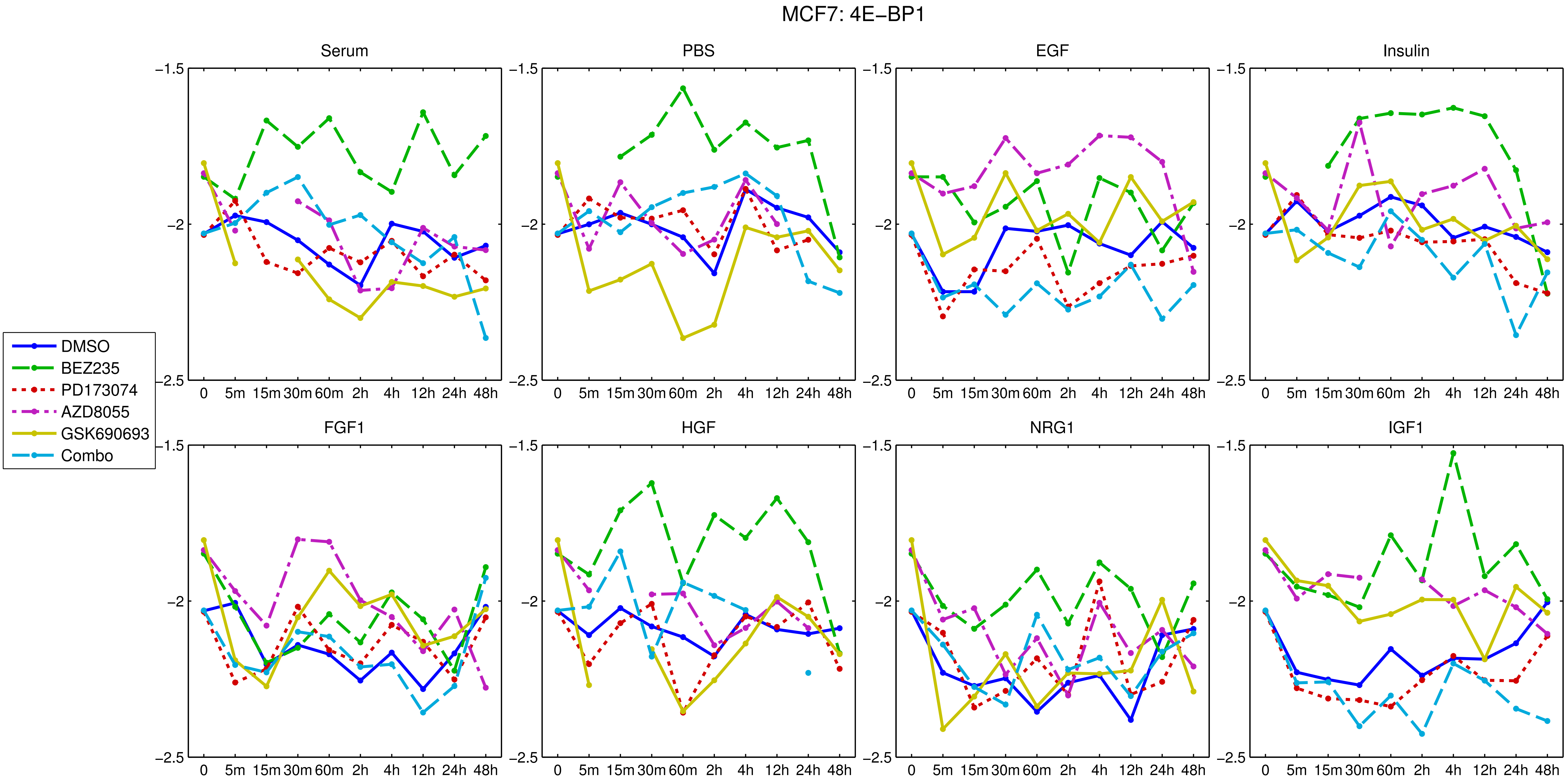
MCF7: 4E−BP1
Serum
PBS
EGF
Insulin
FGF1
HGF
NRG1
IGF1
−1.5
−2
−2.5
0
5m
15m
30m
60m
2h
4h
12h
24h
48h
DMSO
BEZ235
PD173074
AZD8055
GSK690693
Combo

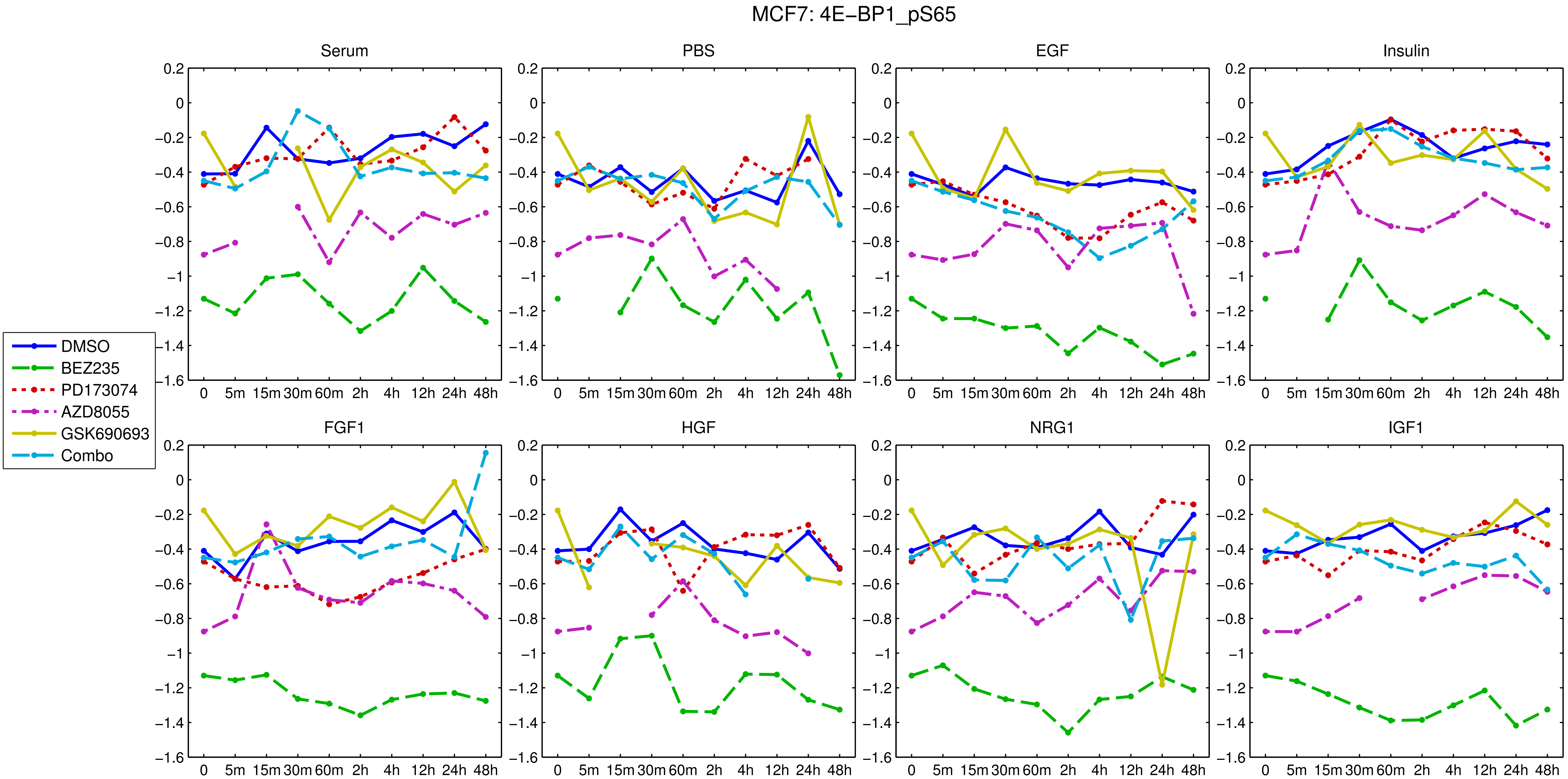

MCF7: 4E−BP1_pS65
Serum
PBS
EGF
Insulin
FGF1
HGF
NRG1
IGF1
DMSO
BEZ235
PD173074
AZD8055
GSK690693
Combo
0
5m
15m
30m
60m
2h
4h
12h
24h
48h

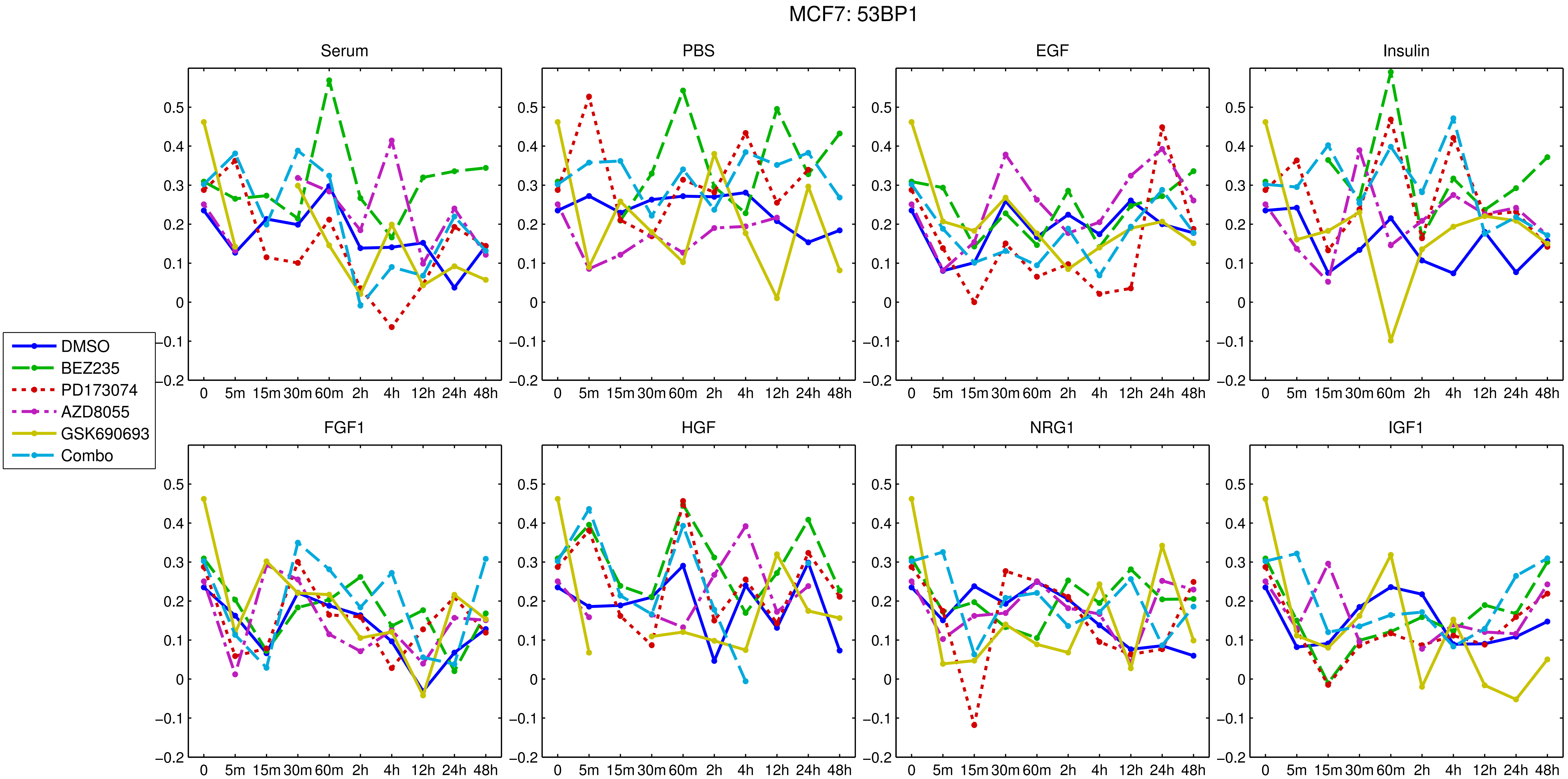
MCF7: 53BP1
Serum
PBS
EGF
Insulin
FGF1
HGF
NRG1
IGF1
DMSO
BEZ235
PD173074
AZD8055
GSK690693
Combo
0.5
0.4
0.3
0.2
0.1
0
−0.1
−0.2
0 5m 15m 30m 60m 2h 4h 12h 24h 48h

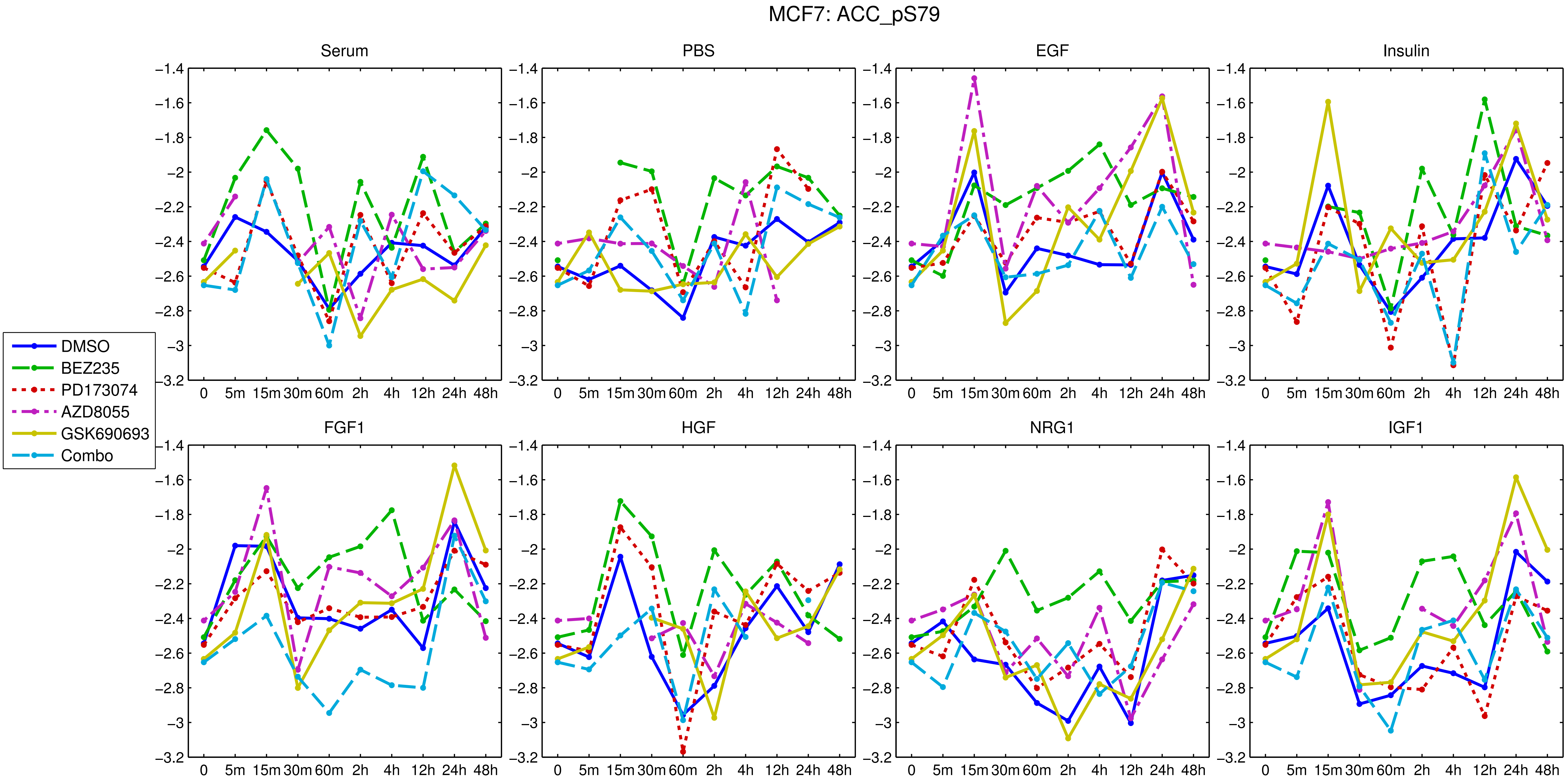
MCF7: ACC_pS79
Serum
PBS
EGF
Insulin
FGF1
HGF
NRG1
IGF1
−1.4
−1.6
−1.8
−2
−2.2
−2.4
−2.6
−2.8
−3
−3.2
0
5m
15m
30m
60m
2h
4h
12h
24h
48h
DMSO
BEZ235
PD173074
AZD8055
GSK690693
Combo

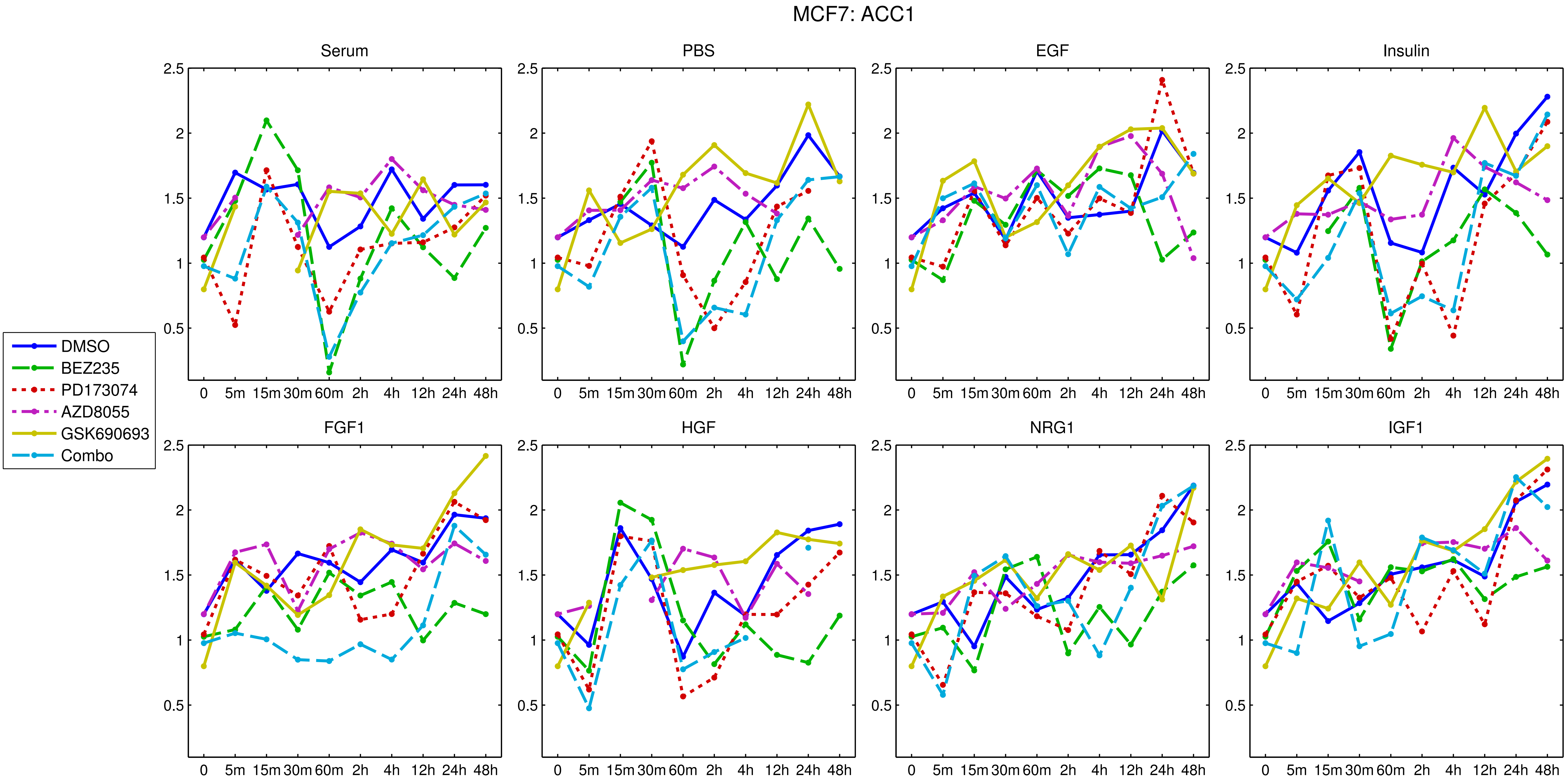

MCF7: ACC1
Serum
PBS
EGF
Insulin
FGF1
HGF
NRG1
IGF1
DMSO
BEZ235
PD173074
AZD8055
GSK690693
Combo
0.5
1
1.5
2
2.5
0
5m
15m
30m
60m
2h
4h
12h
24h
48h

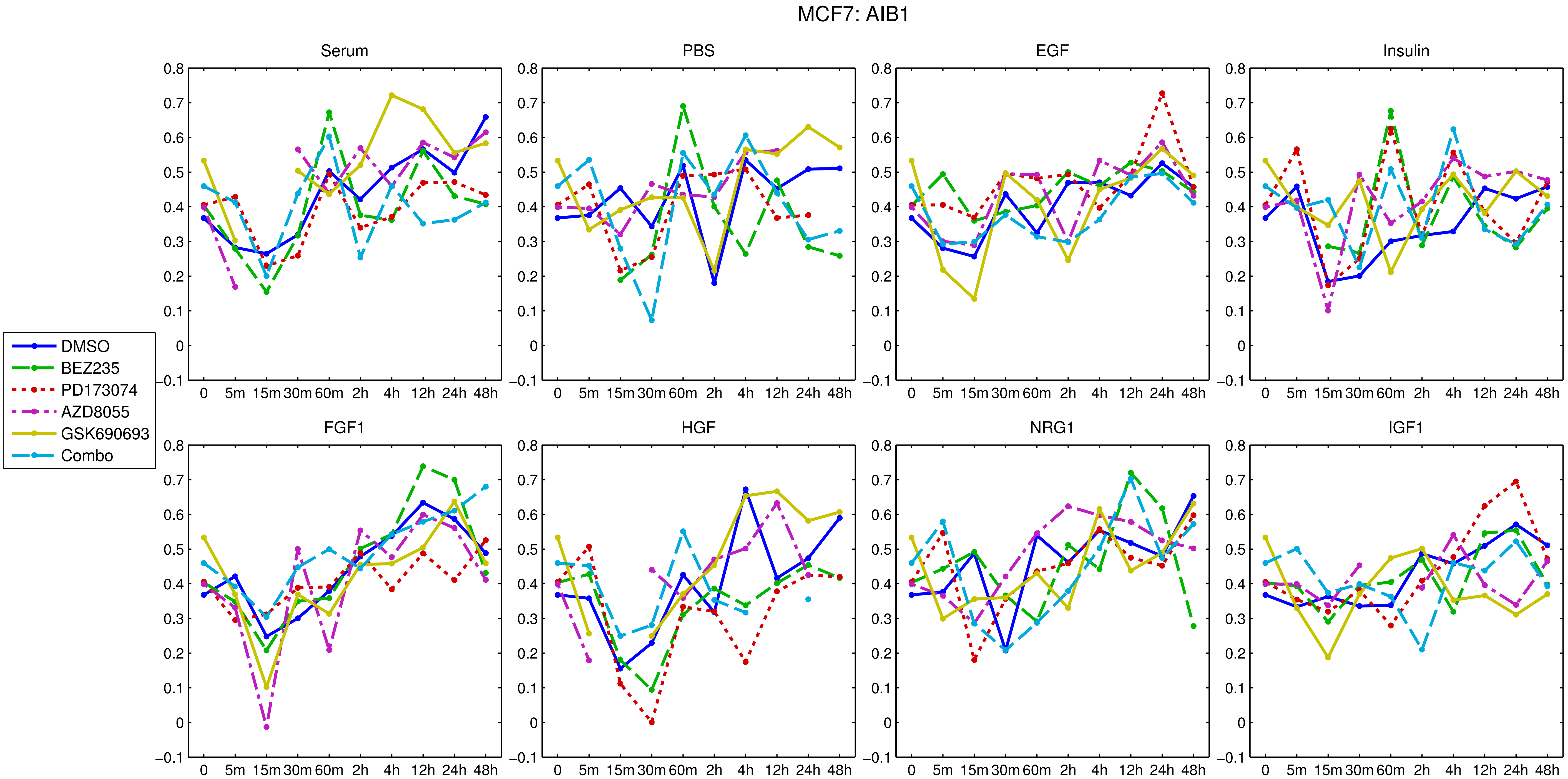
MCF7: AIB1
Serum
PBS
EGF
Insulin
FGF1
HGF
NRG1
IGF1
DMSO
BEZ235
PD173074
AZD8055
GSK690693
Combo
0.8
0.7
0.6
0.5
0.4
0.3
0.2
0.1
0
−0.1
0
5m
15m
30m
60m
2h
4h
12h
24h
48h

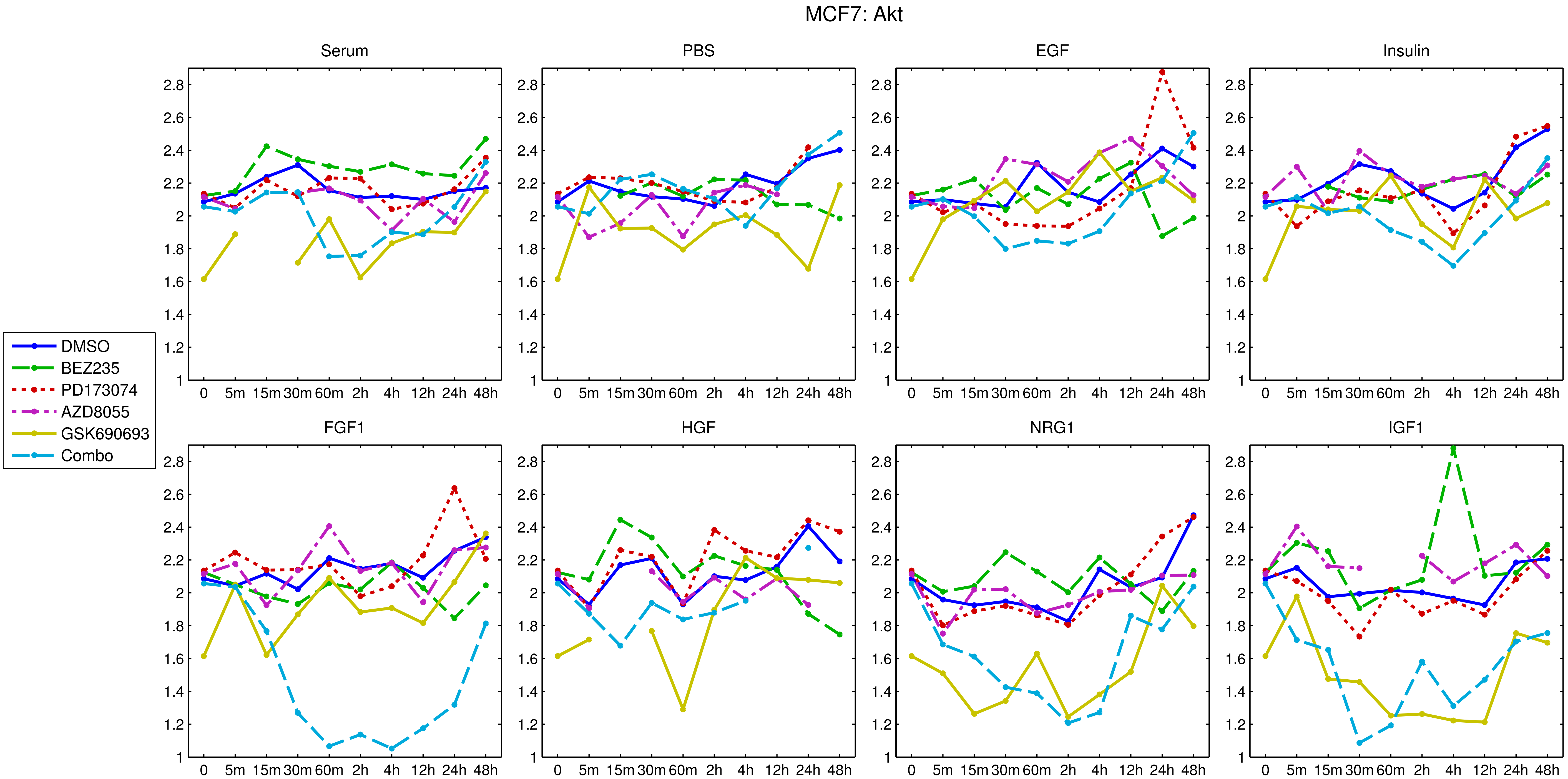
MCF7: Akt
Serum
PBS
EGF
Insulin
FGF1
HGF
NRG1
IGF1
DMSO
BEZ235
PD173074
AZD8055
GSK690693
Combo
1
1.2
1.4
1.6
1.8
2
2.2
2.4
2.6
2.8
0
5m
15m
30m
60m
2h
4h
12h
24h
48h

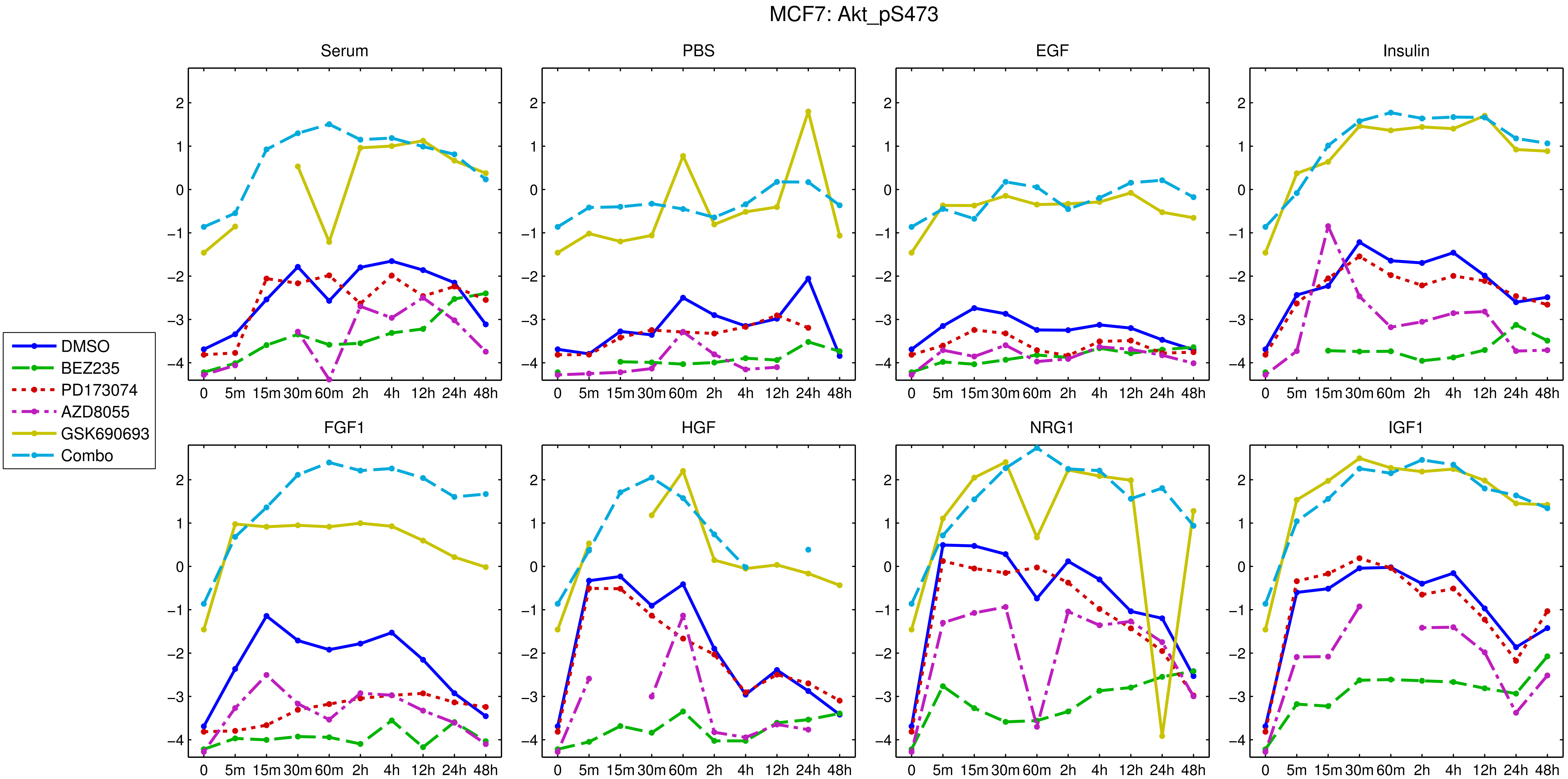

MCF7: Akt_pS473
Serum
PBS
EGF
Insulin
FGF1
HGF
NRG1
IGF1
DMSO
BEZ235
PD173074
AZD8055
GSK690693
Combo
0
5m
15m
30m
60m
2h
4h
12h
24h
48h

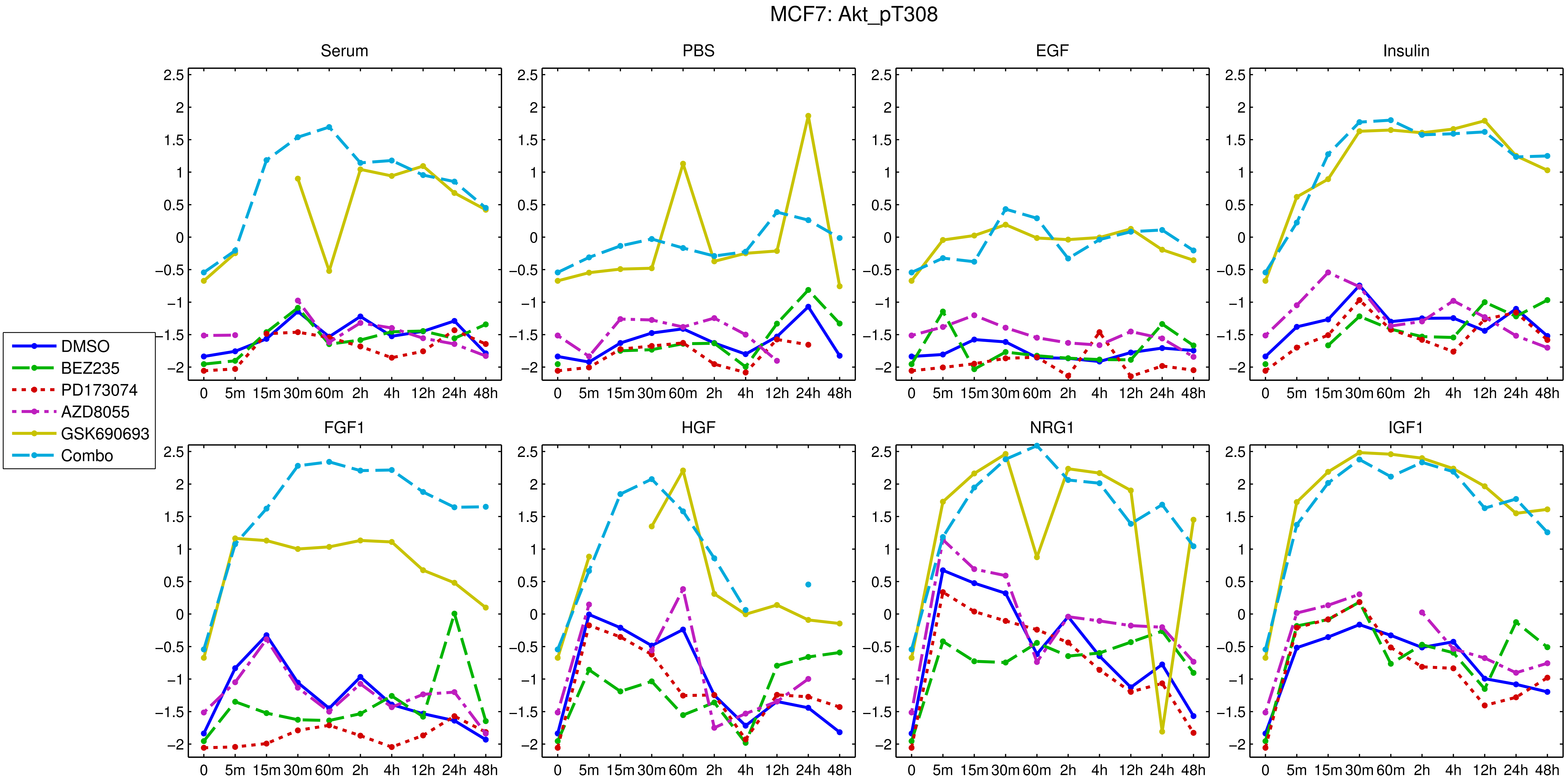

MCF7: Akt_pT308
Serum
PBS
EGF
Insulin
FGF1
HGF
NRG1
IGF1
DMSO
BEZ235
PD173074
AZD8055
GSK690693
Combo
0
5m
15m
30m
60m
2h
4h
12h
24h
48h

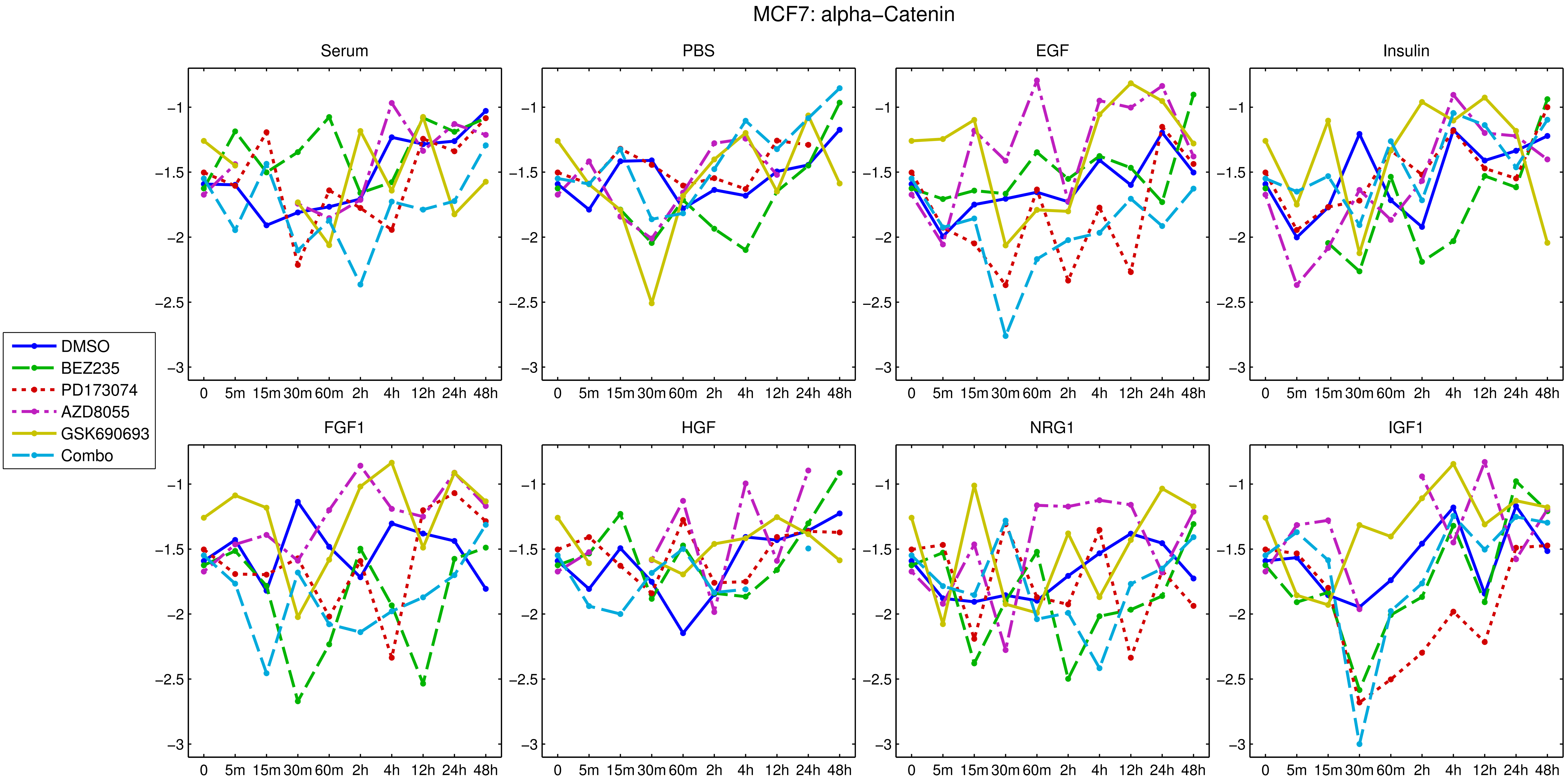

MCF7: alpha−Catenin
Serum
PBS
EGF
Insulin
FGF1
HGF
NRG1
IGF1
−1
−1.5
−2
−2.5
−3
0
5m
15m
30m
60m
2h
4h
12h
24h
48h
DMSO
BEZ235
PD173074
AZD8055
GSK690693
Combo

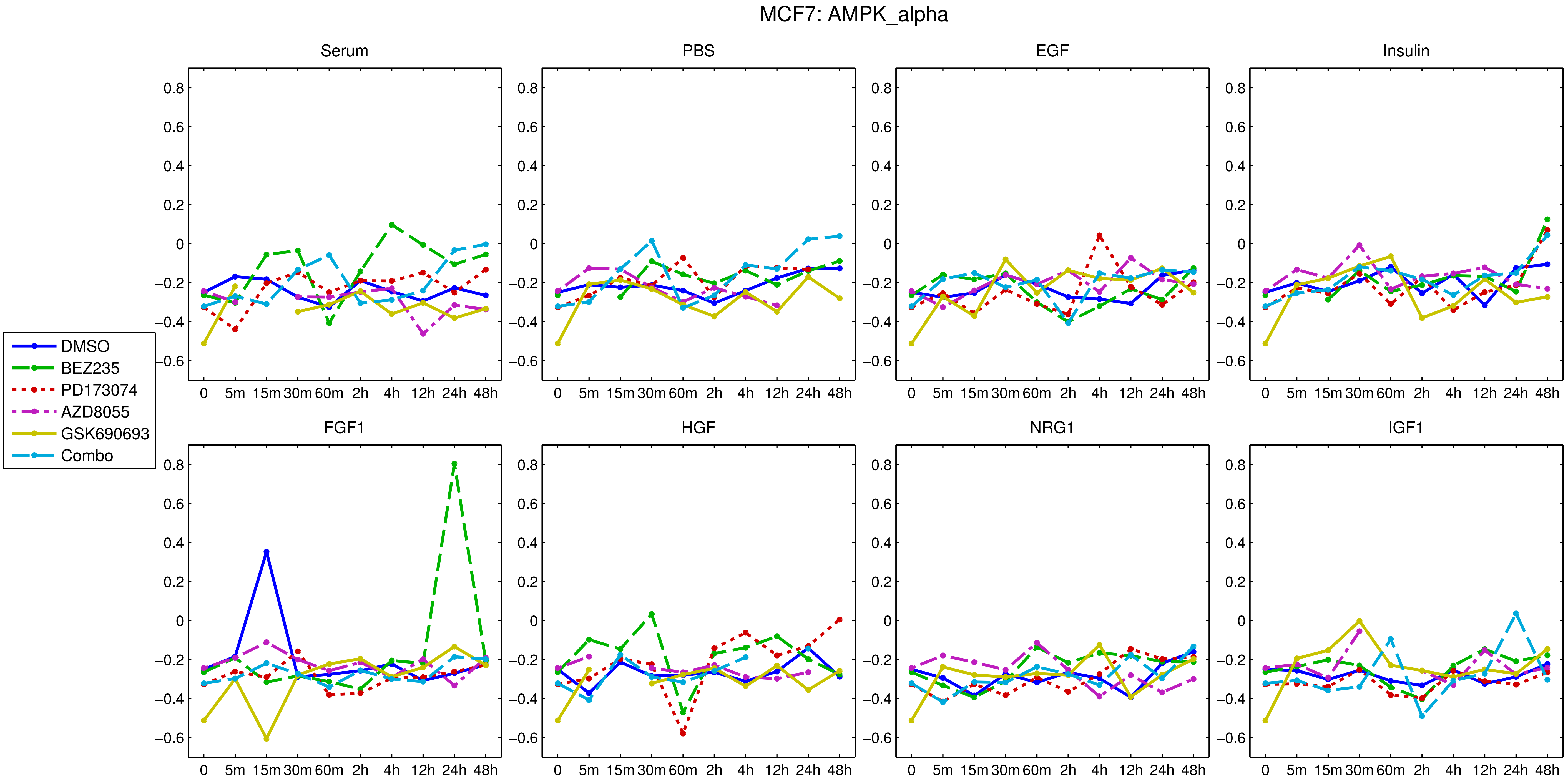
MCF7: AMPK_alpha
Serum
PBS
EGF
Insulin
FGF1
HGF
NRG1
IGF1
0.8
0.6
0.4
0.2
0
−0.2
−0.4
−0.6
0
5m
15m
30m
60m
2h
4h
12h
24h
48h
DMSO
BEZ235
PD173074
AZD8055
GSK690693
Combo

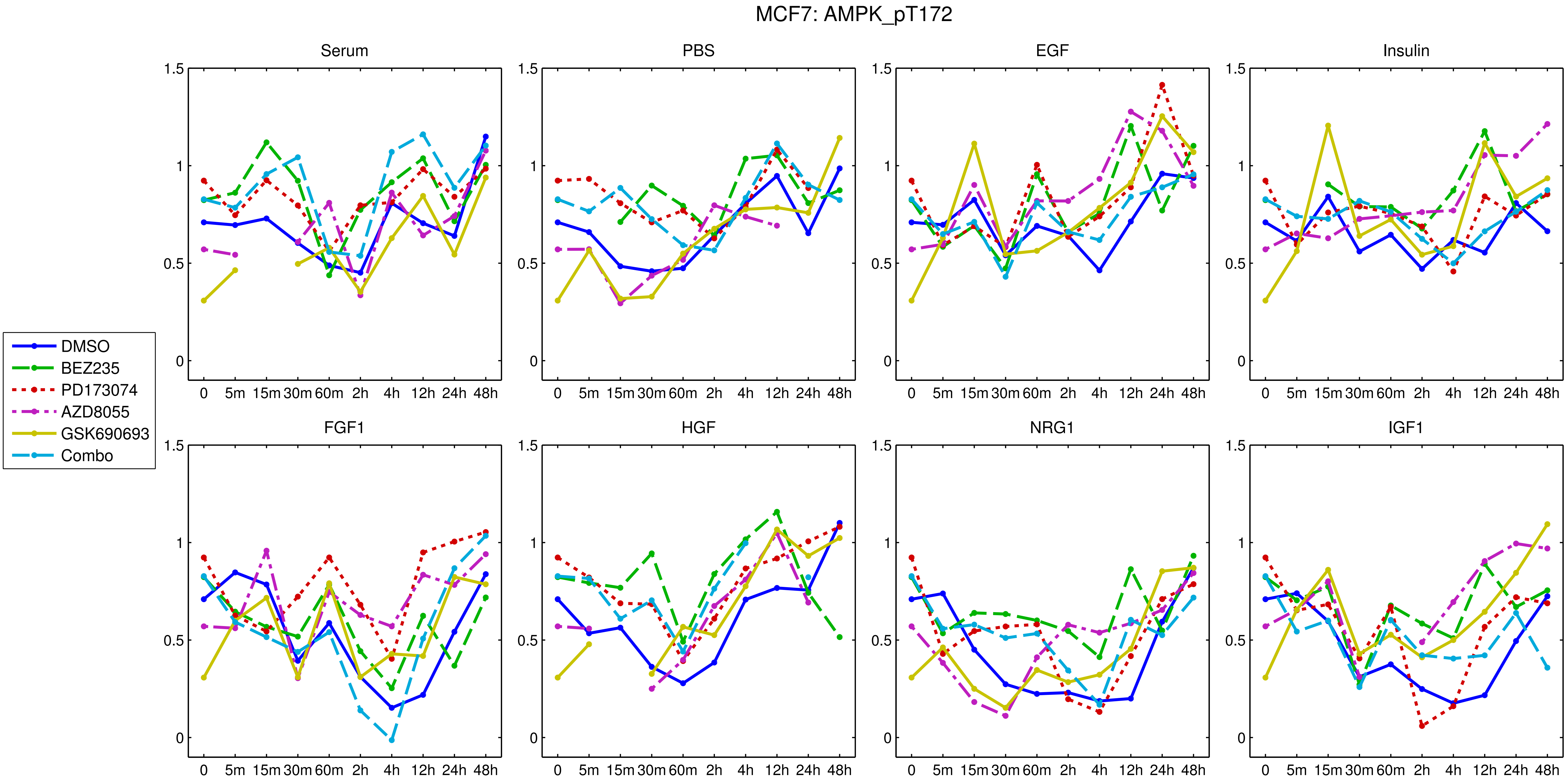
MCF7: AMPK_pT172
Serum
PBS
EGF
Insulin
FGF1
HGF
NRG1
IGF1
DMSO
BEZ235
PD173074
AZD8055
GSK690693
Combo
0
0.5
1
1.5
0
5m
15m
30m
60m
2h
4h
12h
24h
48h

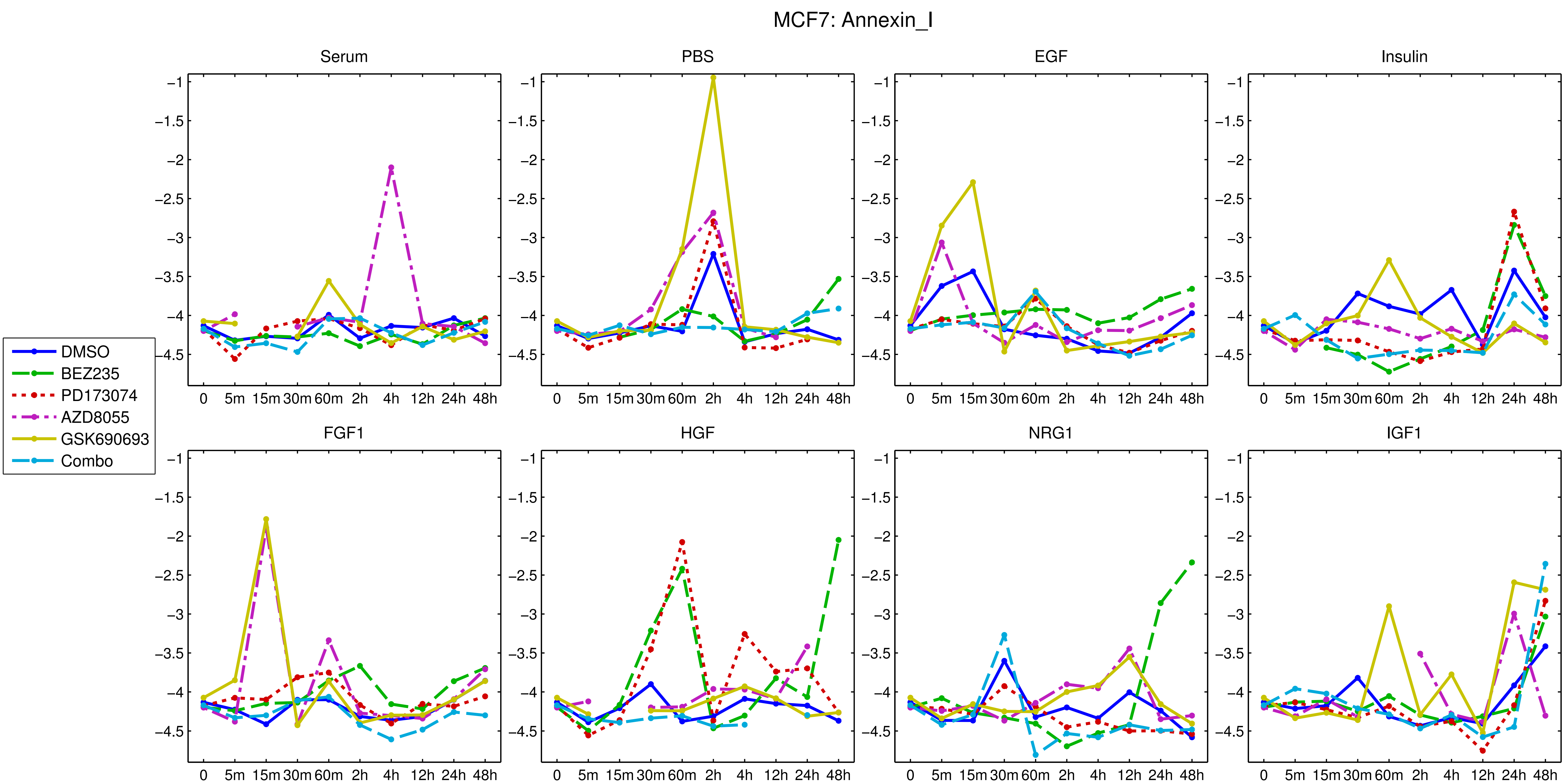

MCF7: Annexin_I
Serum
PBS
EGF
Insulin
FGF1
HGF
NRG1
IGF1
DMSO
BEZ235
PD173074
AZD8055
GSK690693
Combo
0
5m
15m
30m
60m
2h
4h
12h
24h
48h
−1
−1.5
−2
−2.5
−3
−3.5
−4
−4.5

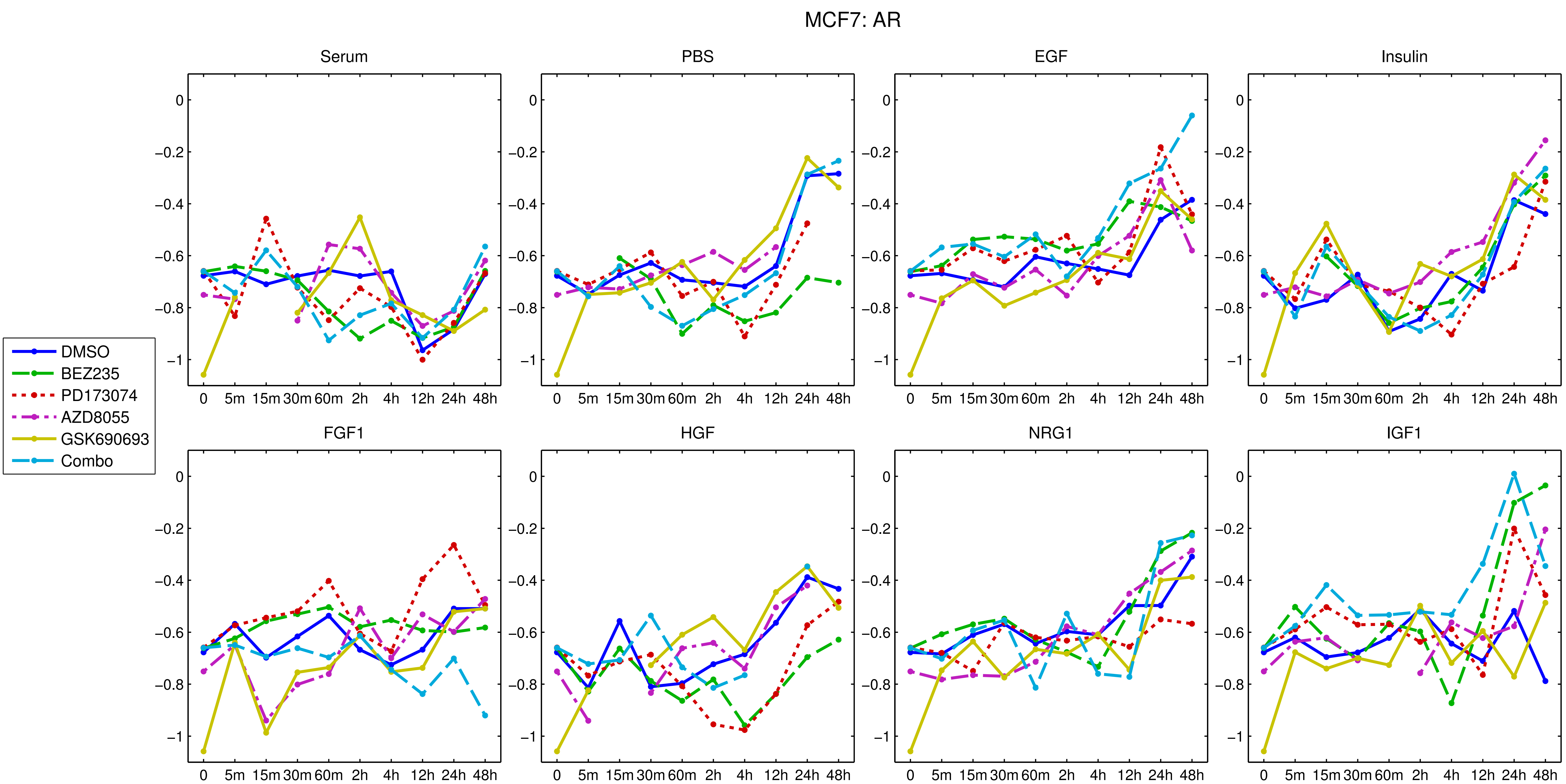
MCF7: AR
Serum
PBS
EGF
Insulin
FGF1
HGF
NRG1
IGF1
0
−0.2
−0.4
−0.6
−0.8
−1
0 5m 15m 30m 60m 2h 4h 12h 24h 48h
DMSO
BEZ235
PD173074
AZD8055
GSK690693
Combo

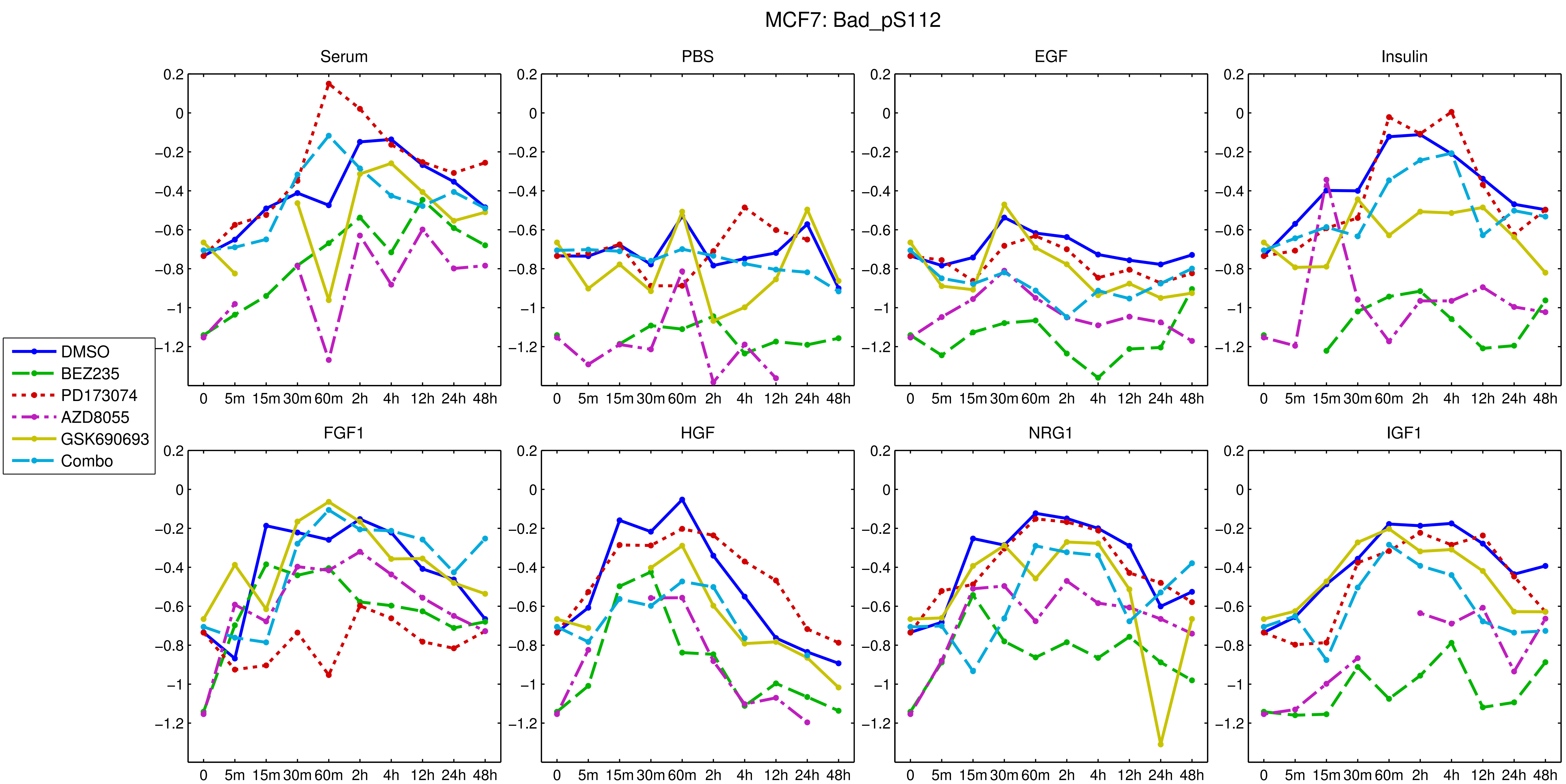
MCF7: Bad_pS112
Serum
PBS
EGF
Insulin
FGF1
HGF
NRG1
IGF1
DMSO
BEZ235
PD173074
AZD8055
GSK690693
Combo
0.2
0
−0.2
−0.4
−0.6
−0.8
−1
−1.2
0
5m
15m
30m
60m
2h
4h
12h
24h
48h

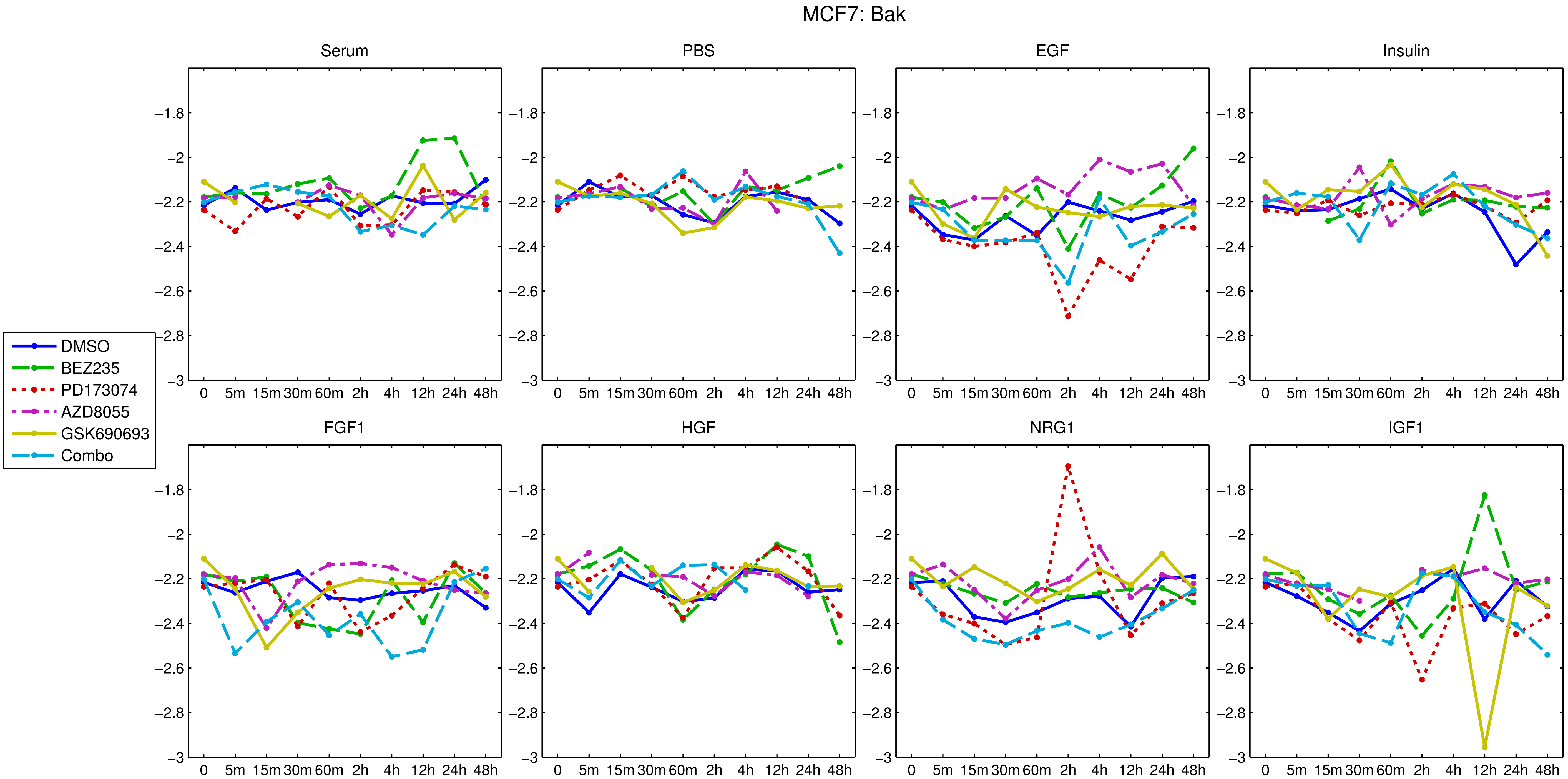

MCF7: Bak
Serum
PBS
EGF
Insulin
FGF1
HGF
NRG1
IGF1
−1.8
−2
−2.2
−2.4
−2.6
−2.8
−3
0
5m
15m
30m
60m
2h
4h
12h
24h
48h
DMSO
BEZ235
PD173074
AZD8055
GSK690693
Combo

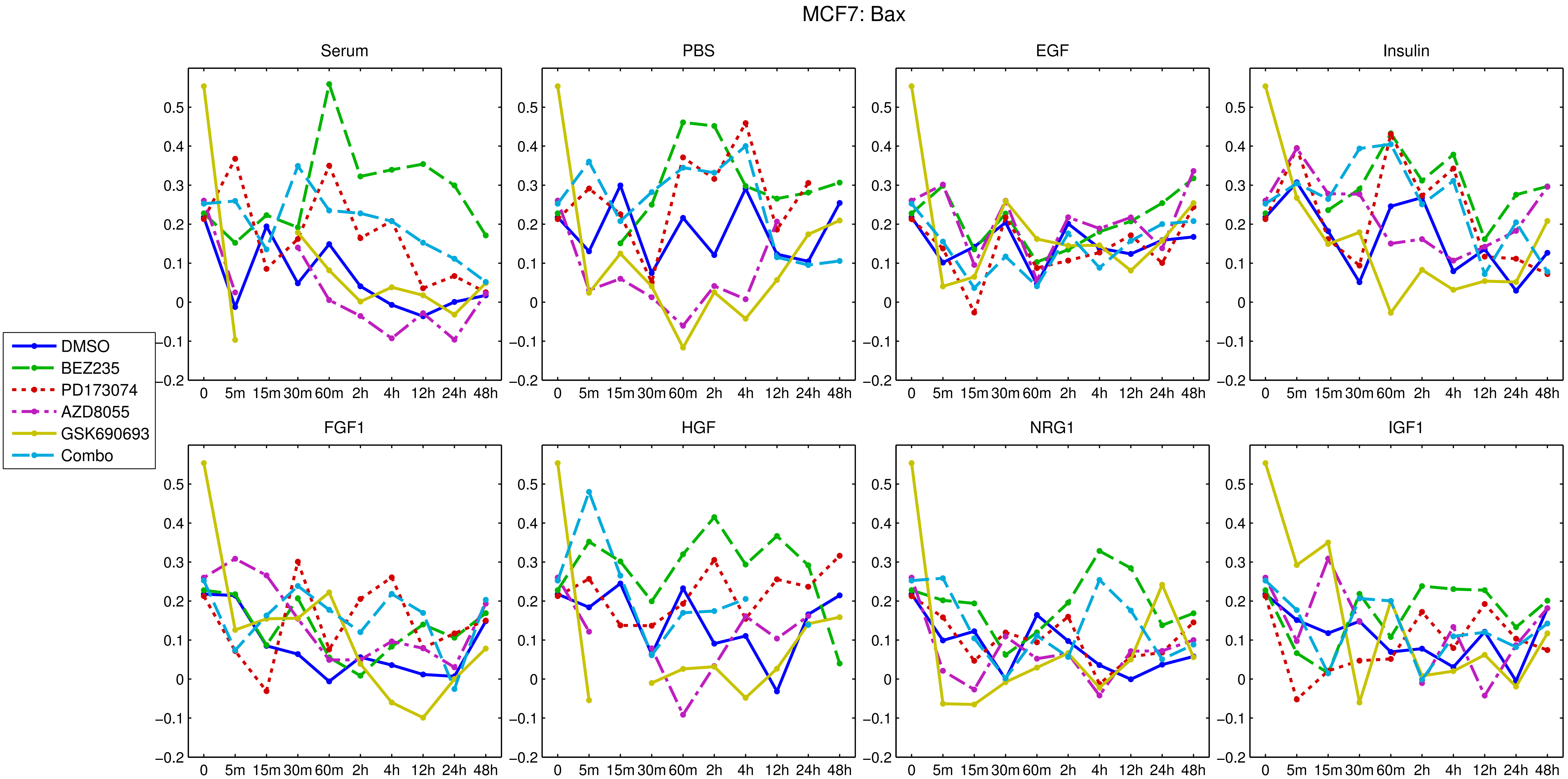
MCF7: Bax
Serum
PBS
EGF
Insulin
FGF1
HGF
NRG1
IGF1
DMSO
BEZ235
PD173074
AZD8055
GSK690693
Combo
0.5
0.4
0.3
0.2
0.1
0
−0.1
−0.2
0 5m 15m 30m 60m 2h 4h 12h 24h 48h

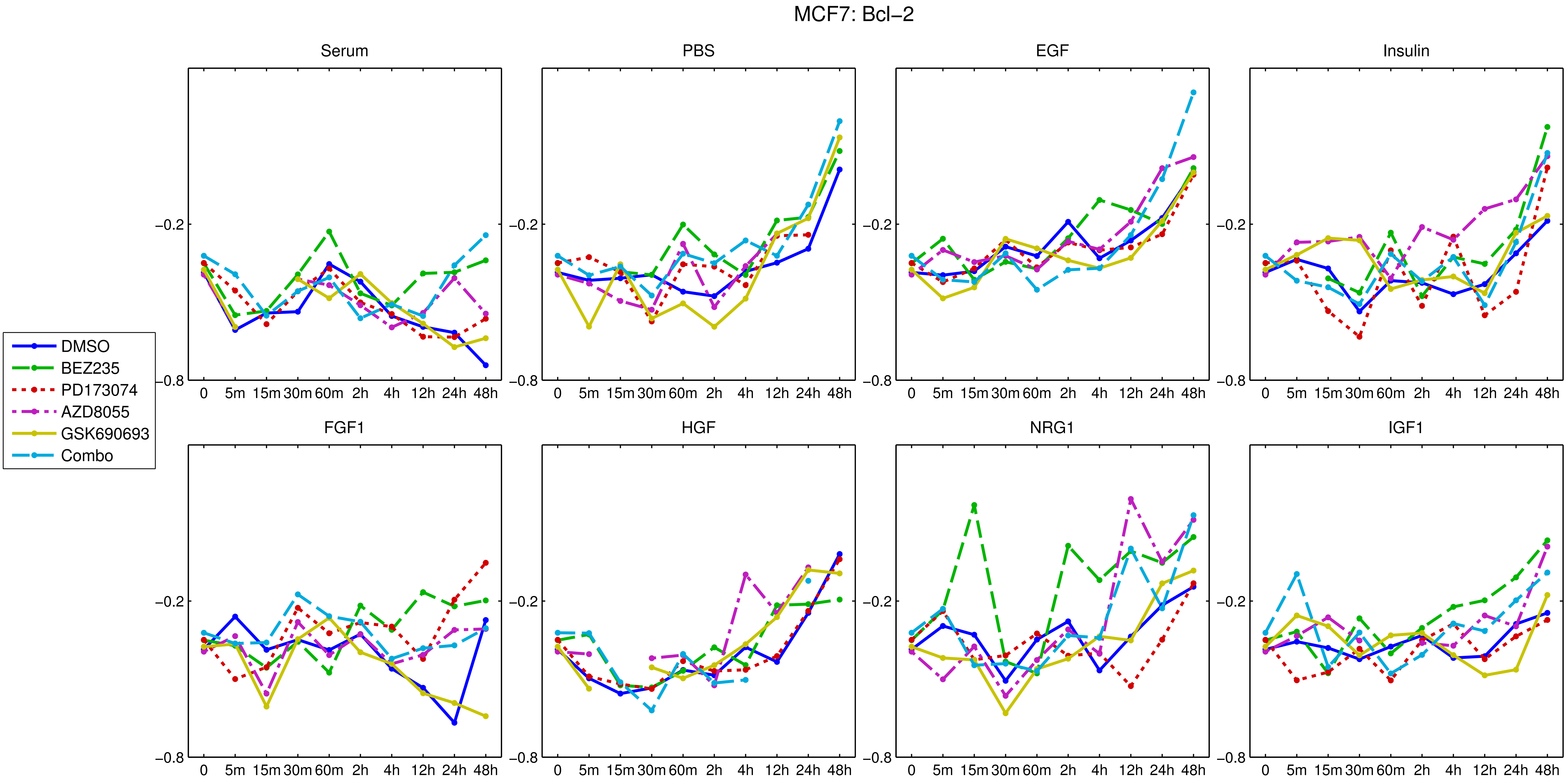

MCF7: Bcl–2
Serum
PBS
EGF
Insulin
FGF1
HGF
NRG1
IGF1
−0.2
−0.8
0
5m
15m
30m
60m
2h
4h
12h
24h
48h
DMSO
BEZ235
PD173074
AZD8055
GSK690693
Combo

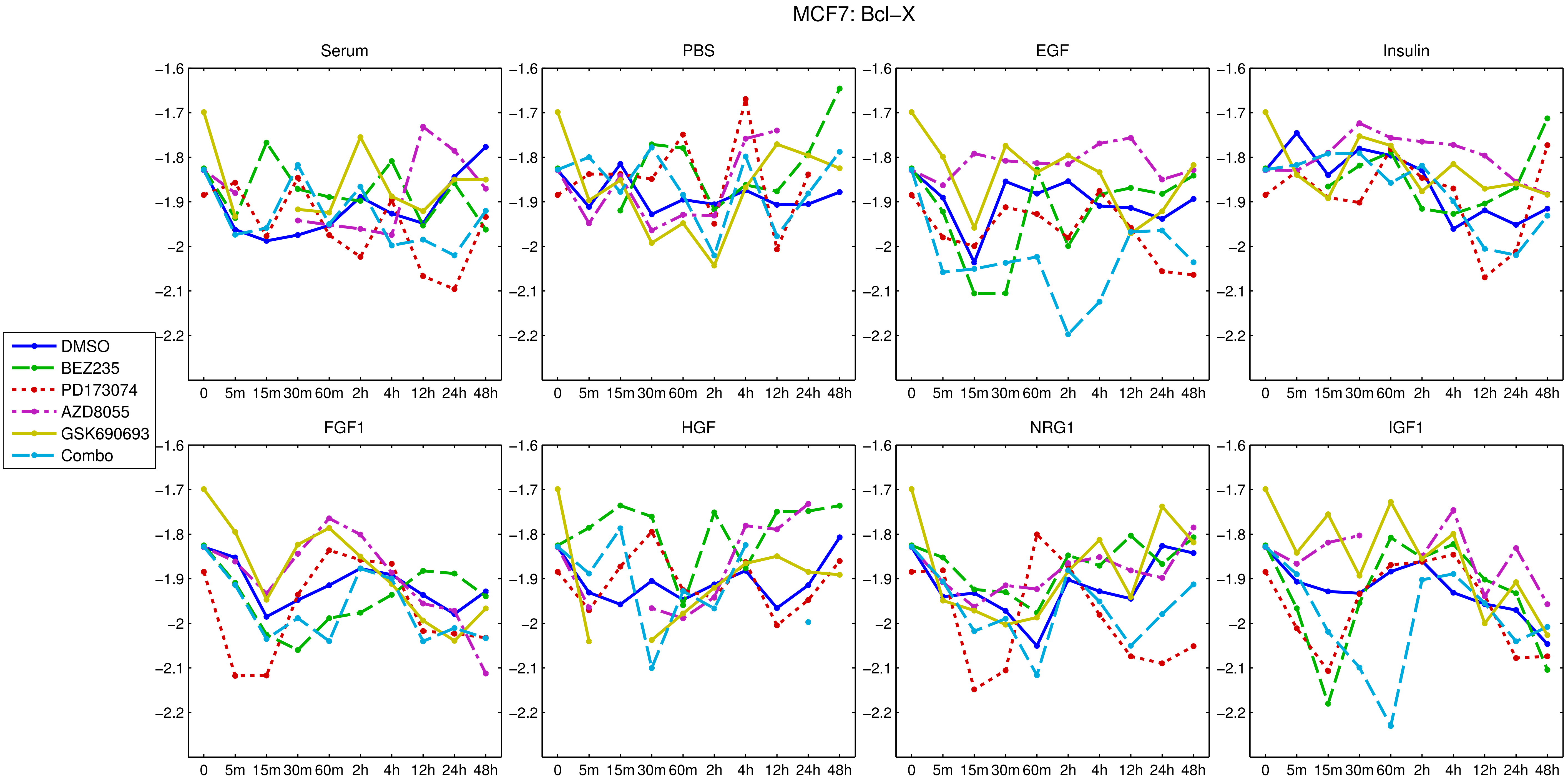
MCF7: Bcl–X
Serum
PBS
EGF
Insulin
FGF1
HGF
NRG1
IGF1
−1.6
−1.7
−1.8
−1.9
−2
−2.1
−2.2
0
5m
15m
30m
60m
2h
4h
12h
24h
48h
DMSO
BEZ235
PD173074
AZD8055
GSK690693
Combo

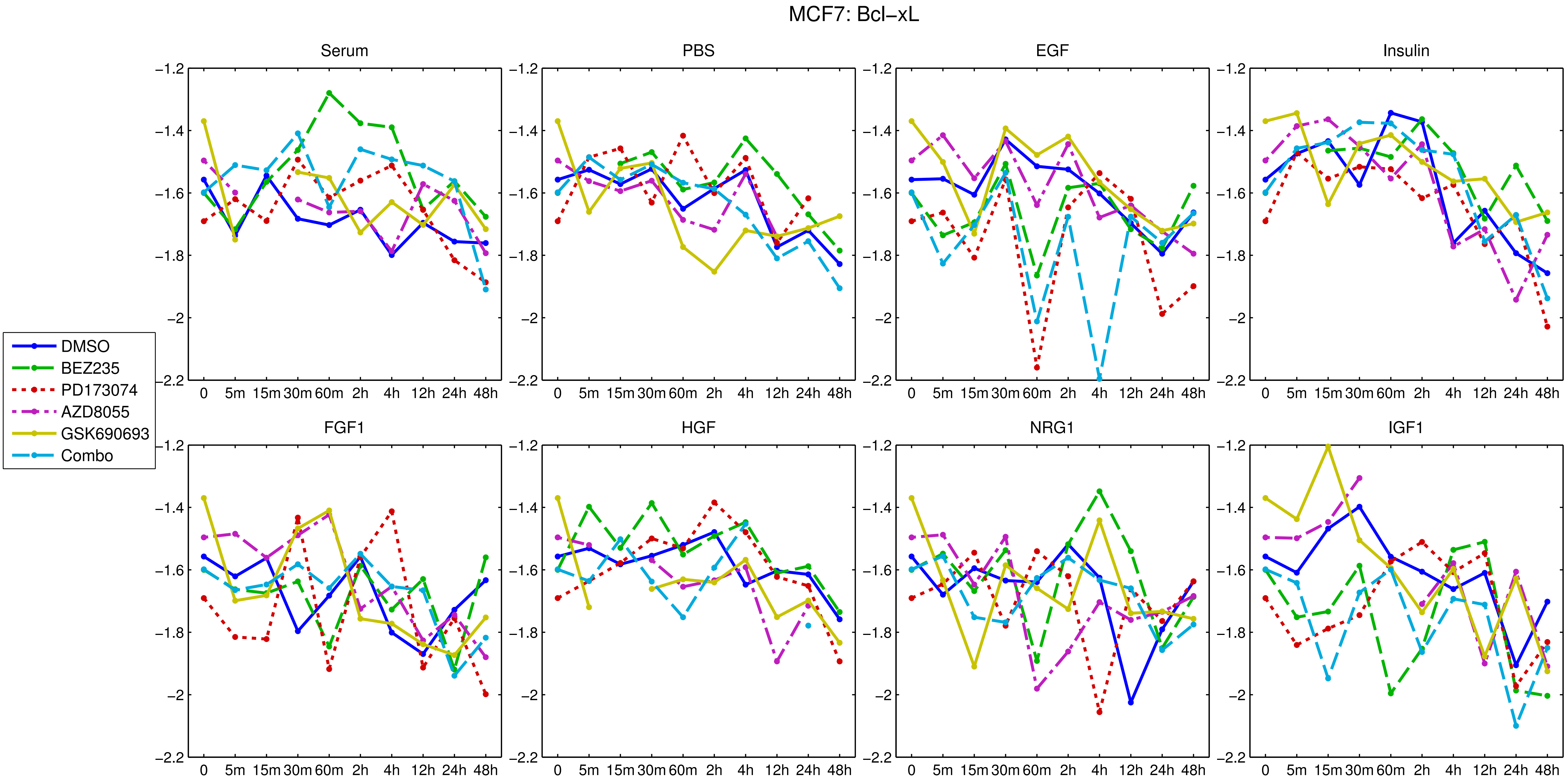

MCF7: Bcl−xL
Serum
PBS
EGF
Insulin
FGF1
HGF
NRG1
IGF1
DMSO
BEZ235
PD173074
AZD8055
GSK690693
Combo
−1.2
−1.4
−1.6
−1.8
−2
−2.2
0
5m
15m
30m
60m
2h
4h
12h
24h
48h

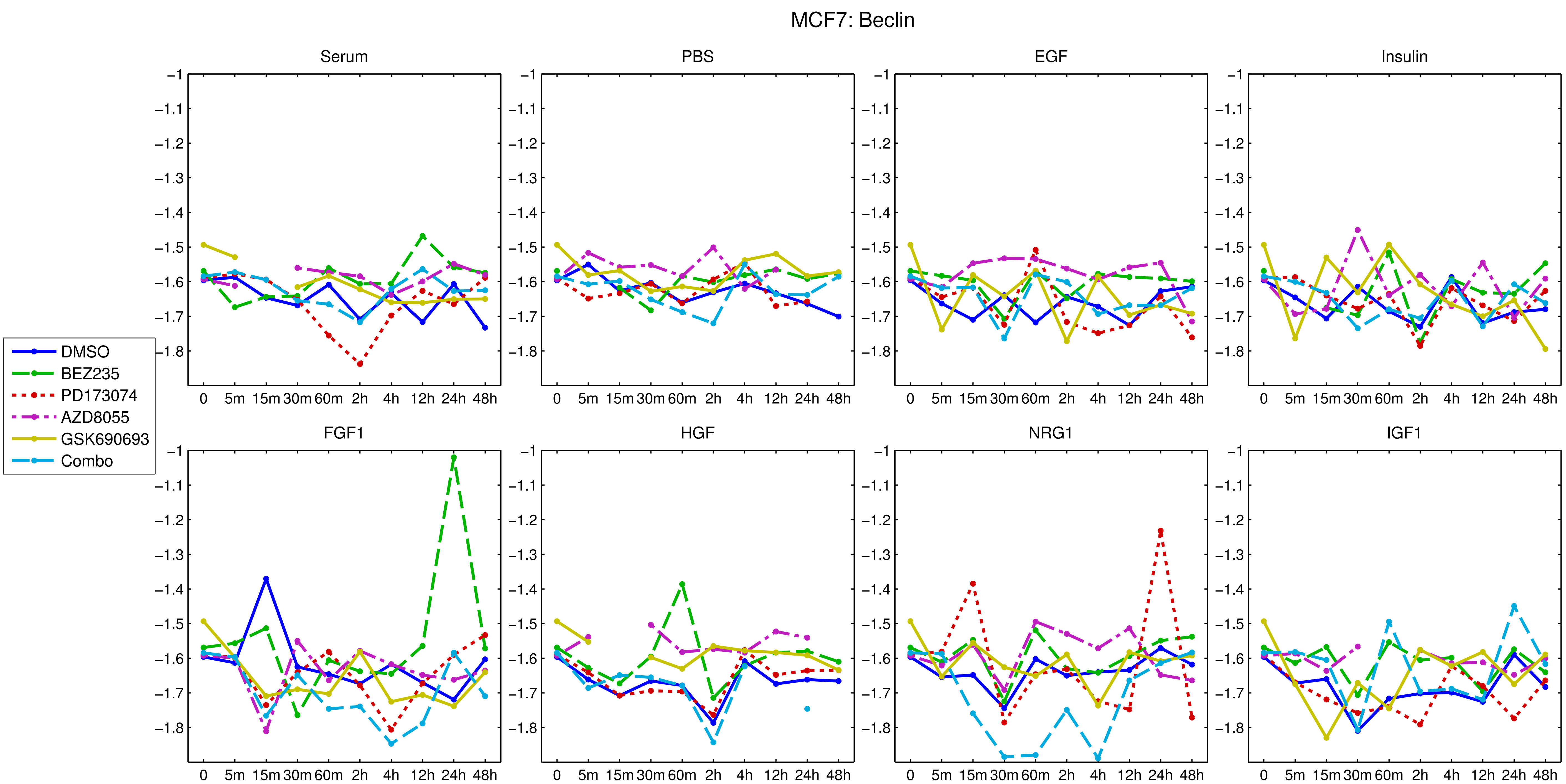

MCF7: Beclin
Serum
PBS
EGF
Insulin
FGF1
HGF
NRG1
IGF1
DMSO
BEZ235
PD173074
AZD8055
GSK690693
Combo
−1
−1.1
−1.2
−1.3
−1.4
−1.5
−1.6
−1.7
−1.8
0
5m
15m
30m
60m
2h
4h
12h
24h
48h

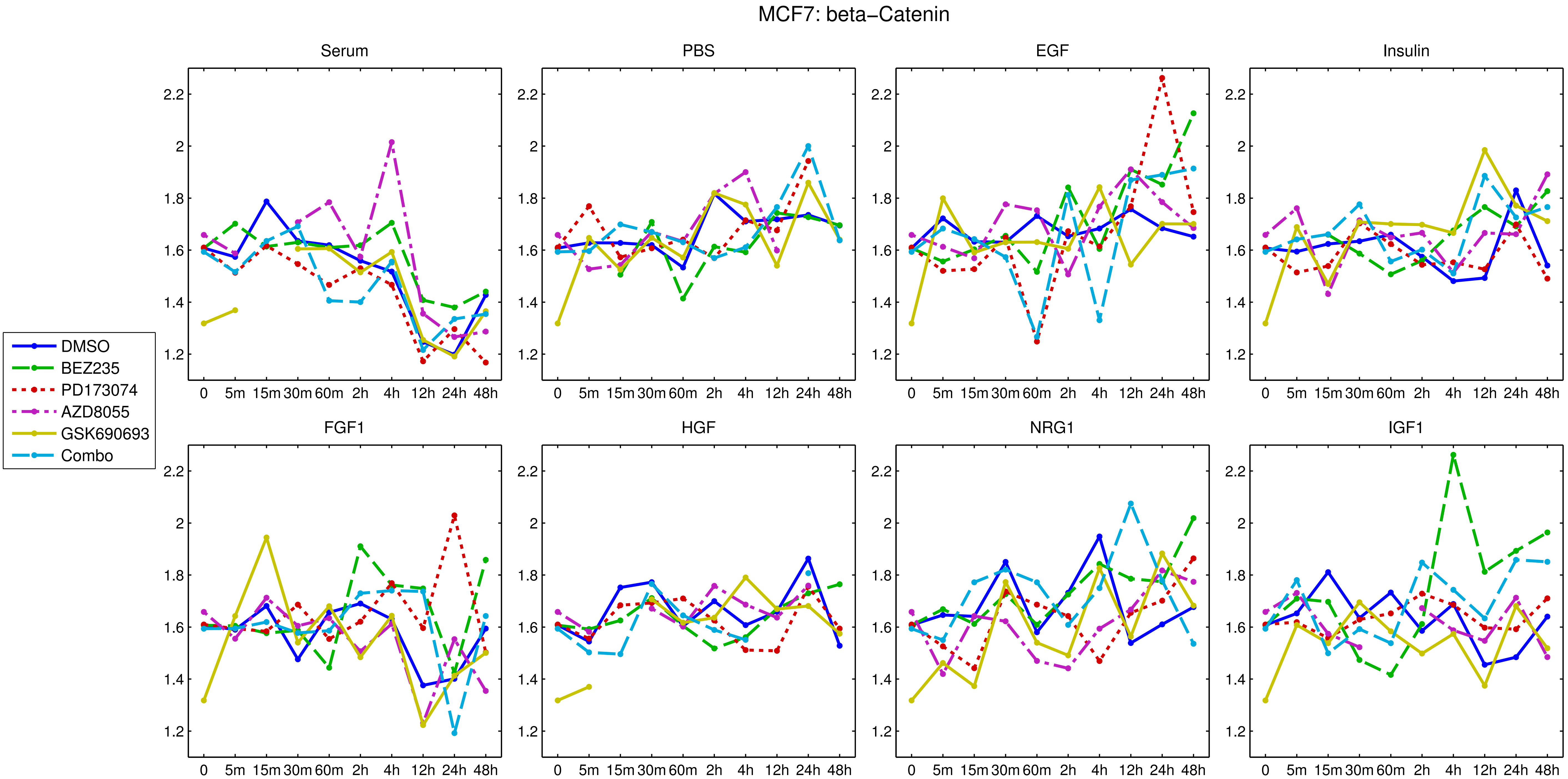
MCF7: beta−Catenin
Serum
PBS
EGF
Insulin
FGF1
HGF
NRG1
IGF1
DMSO
BEZ235
PD173074
AZD8055
GSK690693
Combo
2.2
2
1.8
1.6
1.4
1.2
0
5m
15m
30m
60m
2h
4h
12h
24h
48h

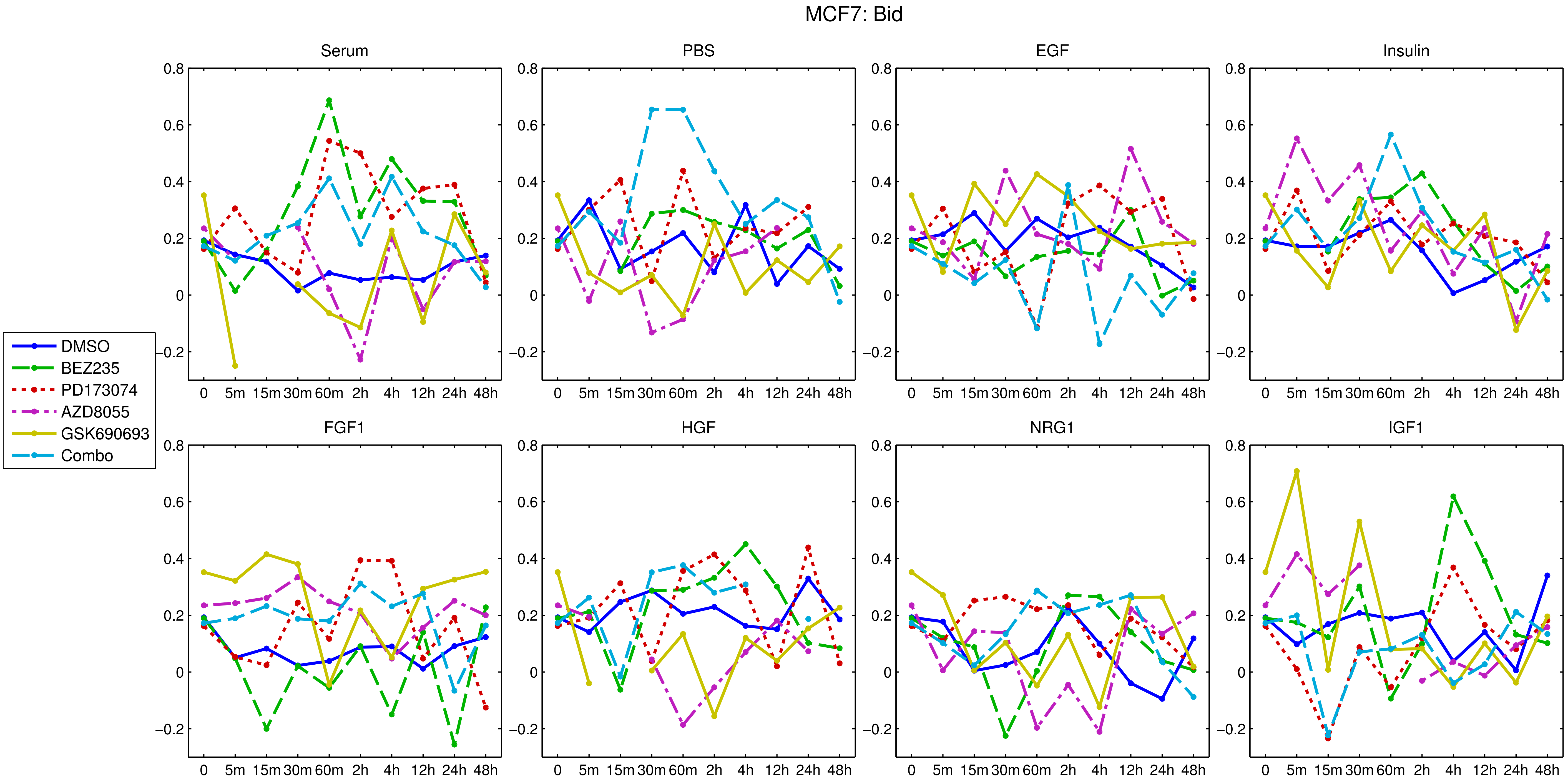
MCF7: Bid
Serum
PBS
EGF
Insulin
FGF1
HGF
NRG1
IGF1
DMSO
BEZ235
PD173074
AZD8055
GSK690693
Combo
0.8
0.6
0.4
0.2
0
−0.2
0
5m
15m
30m
60m
2h
4h
12h
24h
48h

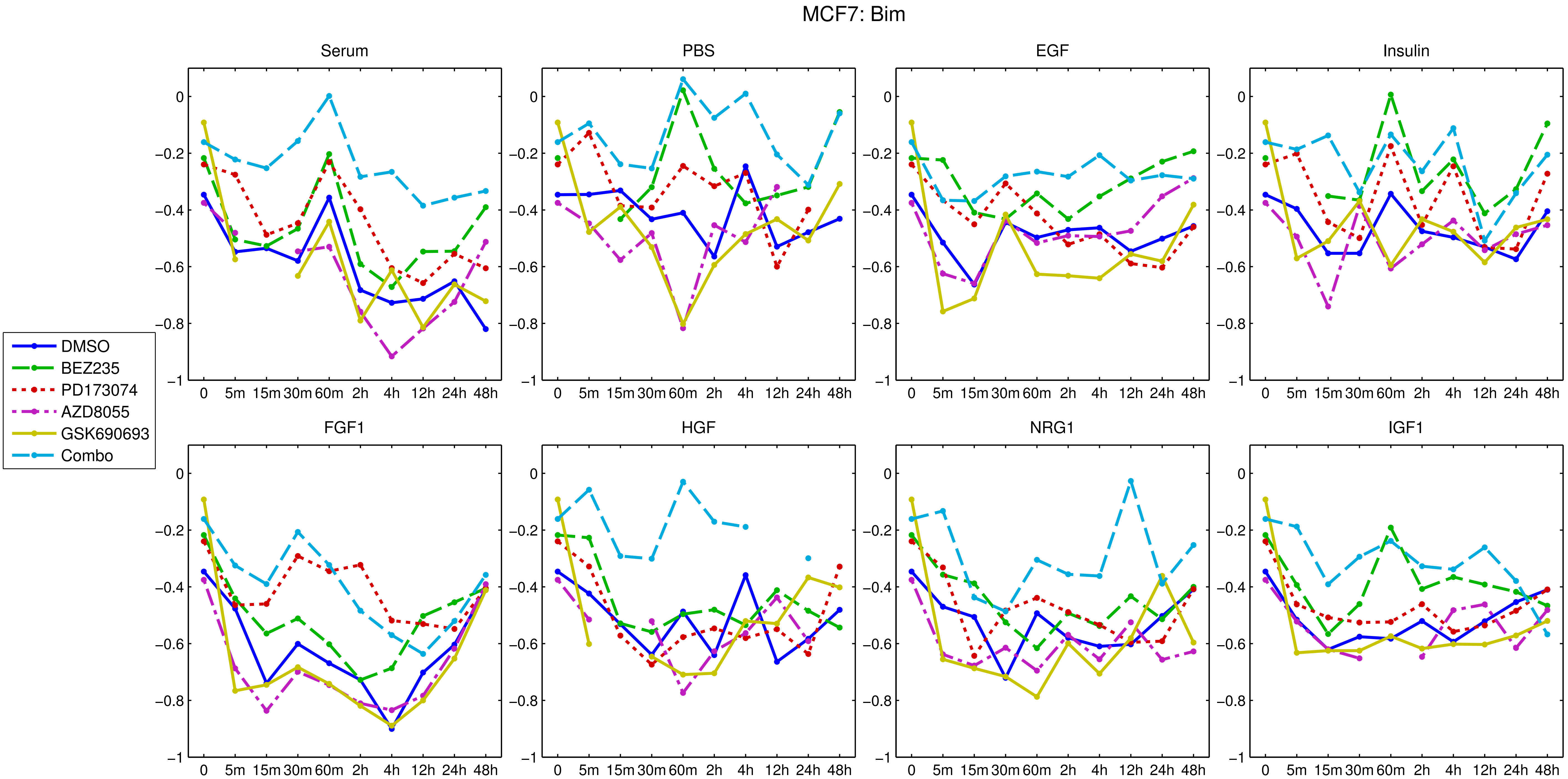
MCF7: Bim
Serum
PBS
EGF
Insulin
FGF1
HGF
NRG1
IGF1
DMSO
BEZ235
PD173074
AZD8055
GSK690693
Combo
0
−0.2
−0.4
−0.6
−0.8
−1
0 5m 15m 30m 60m 2h 4h 12h 24h 48h

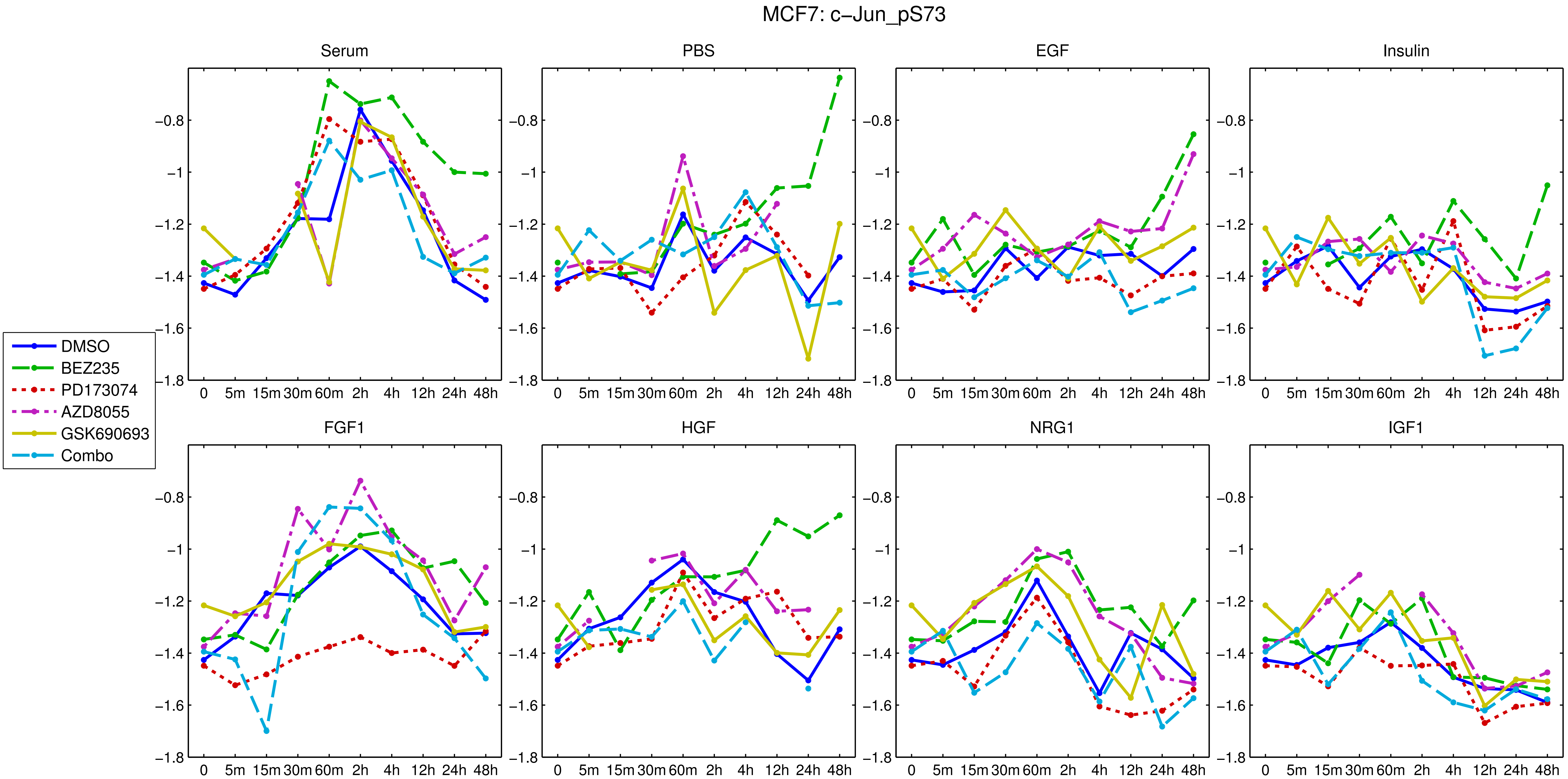
MCF7: c–Jun_pS73
Serum
PBS
EGF
Insulin
FGF1
HGF
NRG1
IGF1
DMSO
BEZ235
PD173074
AZD8055
GSK690693
Combo
−0.8
−1
−1.2
−1.4
−1.6
−1.8
0 5m 15m 30m 60m 2h 4h 12h 24h 48h

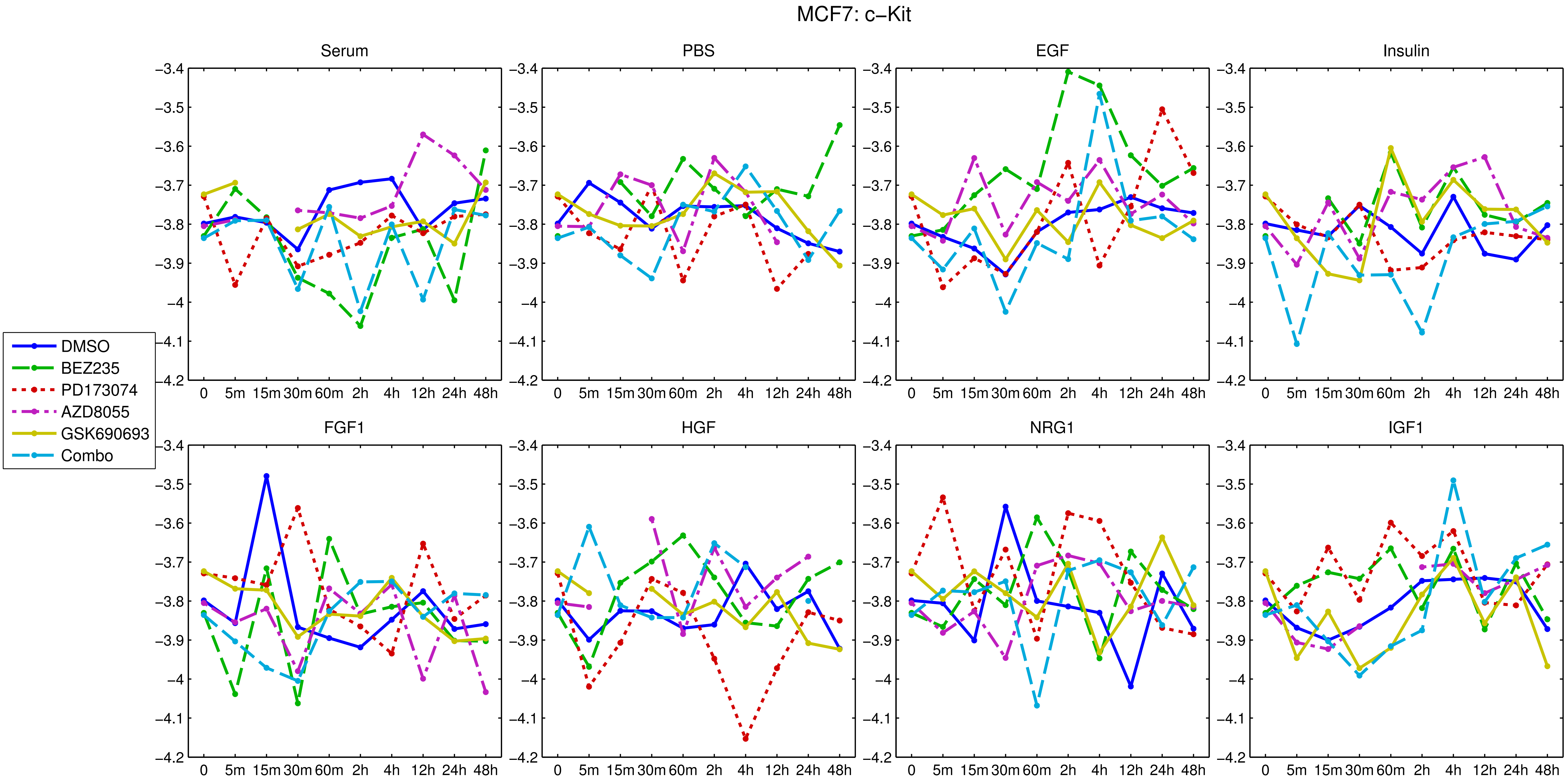
MCF7: c−Kit
Serum
PBS
EGF
Insulin
FGF1
HGF
NRG1
IGF1
DMSO
BEZ235
PD173074
AZD8055
GSK690693
Combo
−3.4
−3.5
−3.6
−3.7
−3.8
−3.9
−4
−4.1
−4.2
0
5m
15m
30m
60m
2h
4h
12h
24h
48h

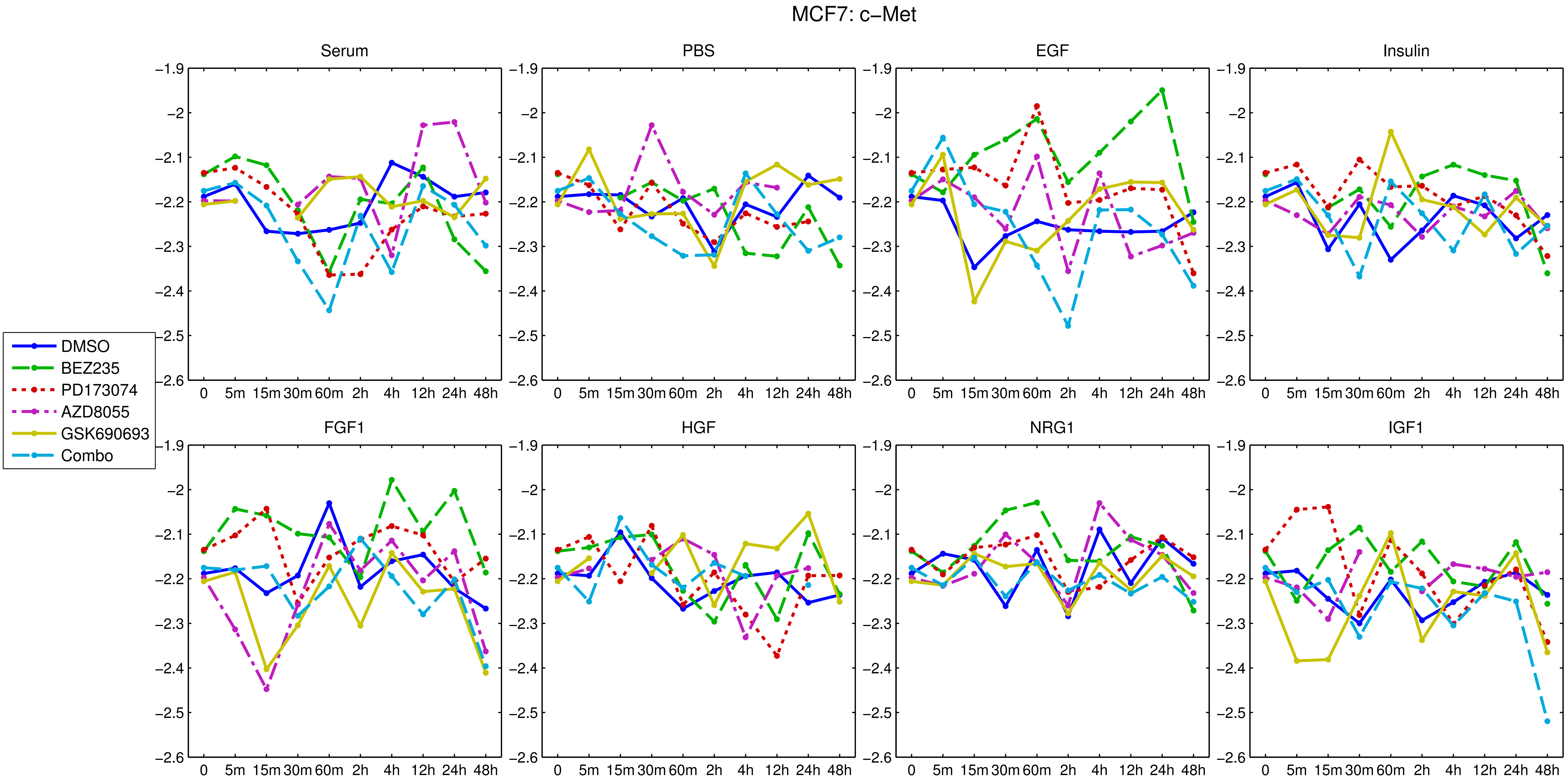
MCF7: c–Met
Serum
PBS
EGF
Insulin
FGF1
HGF
NRG1
IGF1
DMSO
BEZ235
PD173074
AZD8055
GSK690693
Combo
−1.9
−2
−2.1
−2.2
−2.3
−2.4
−2.5
−2.6
0
5m
15m
30m
60m
2h
4h
12h
24h
48h

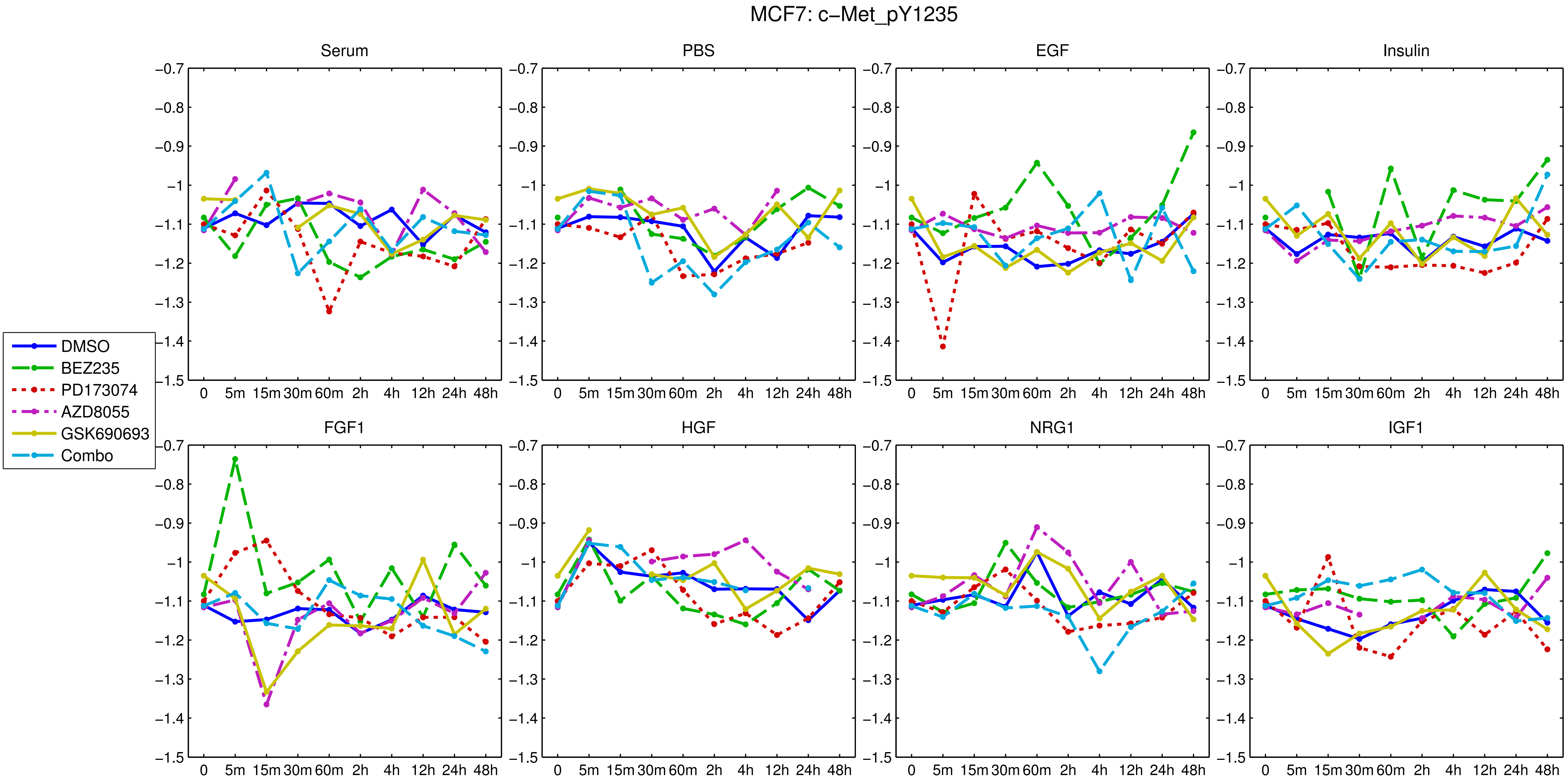

MCF7: c–Met_pY1235
Serum
PBS
EGF
Insulin
FGF1
HGF
NRG1
IGF1
−0.7
−0.8
−0.9
−1
−1.1
−1.2
−1.3
−1.4
−1.5
0
5m
15m
30m
60m
2h
4h
12h
24h
48h
DMSO
BEZ235
PD173074
AZD8055
GSK690693
Combo

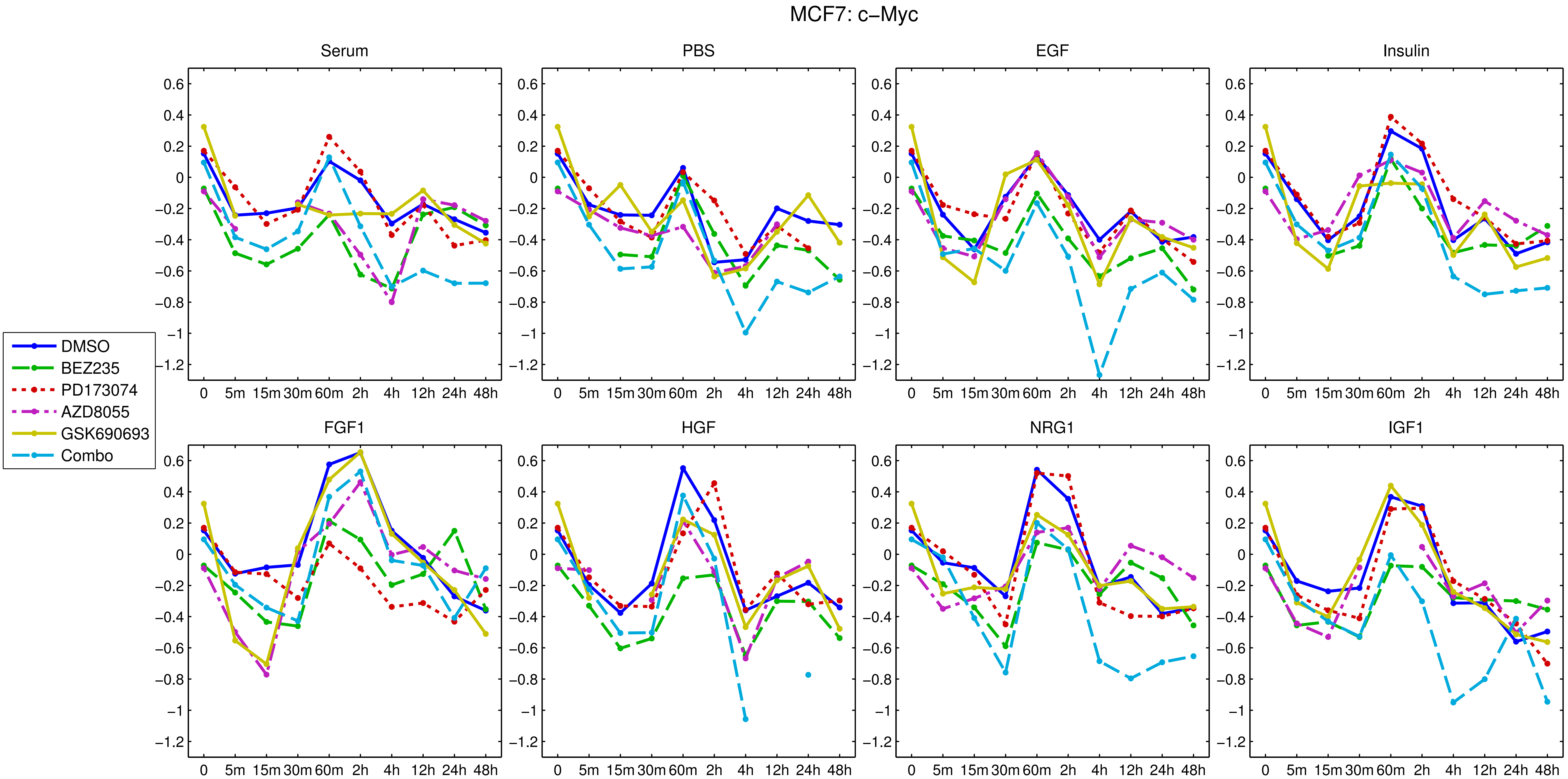
MCF7: c–Myc
Serum
PBS
EGF
Insulin
FGF1
HGF
NRG1
IGF1
DMSO
BEZ235
PD173074
AZD8055
GSK690693
Combo
0.6
0.4
0.2
0
−0.2
−0.4
−0.6
−0.8
−1
−1.2
0 5m 15m 30m 60m 2h 4h 12h 24h 48h

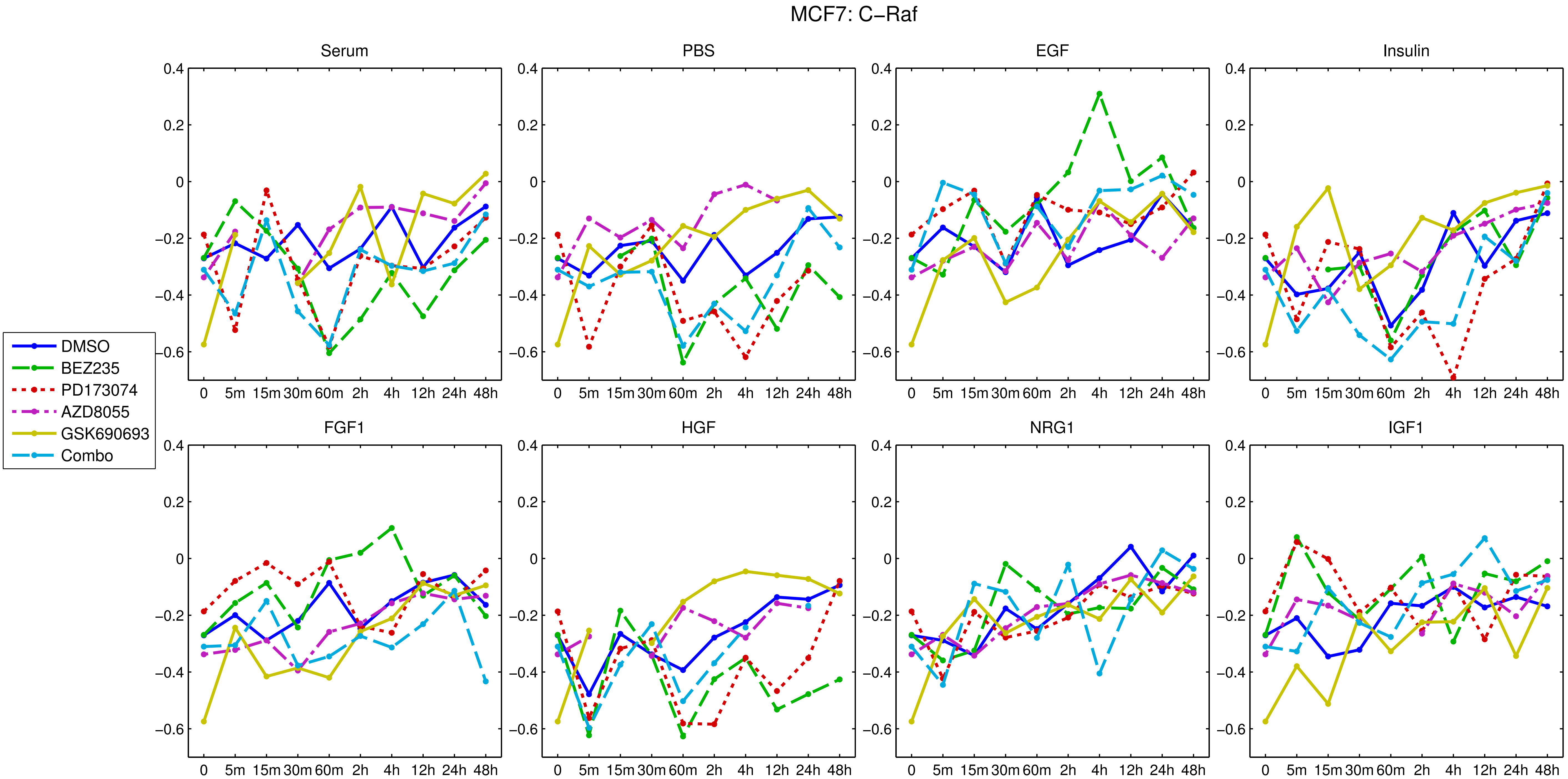
MCF7: C−Raf
Serum
PBS
EGF
Insulin
FGF1
HGF
NRG1
IGF1
DMSO
BEZ235
PD173074
AZD8055
GSK690693
Combo
0.4
0.2
0
−0.2
−0.4
−0.6
0 5m 15m 30m 60m 2h 4h 12h 24h 48h

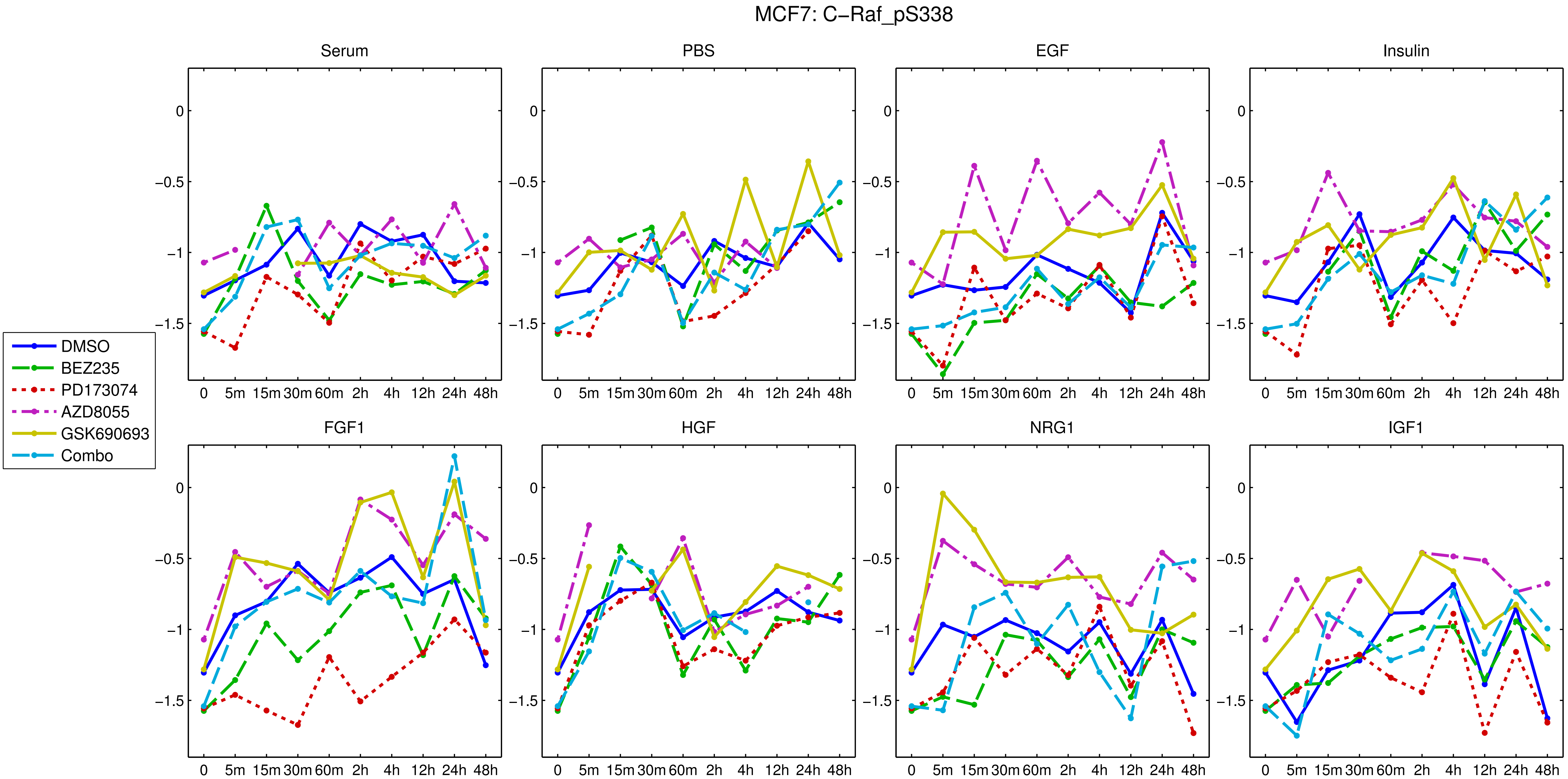
MCF7: C−Raf_pS338
Serum
PBS
EGF
Insulin
FGF1
HGF
NRG1
IGF1
0
−0.5
−1
−1.5
0
5m
15m
30m
60m
2h
4h
12h
24h
48h
DMSO
BEZ235
PD173074
AZD8055
GSK690693
Combo

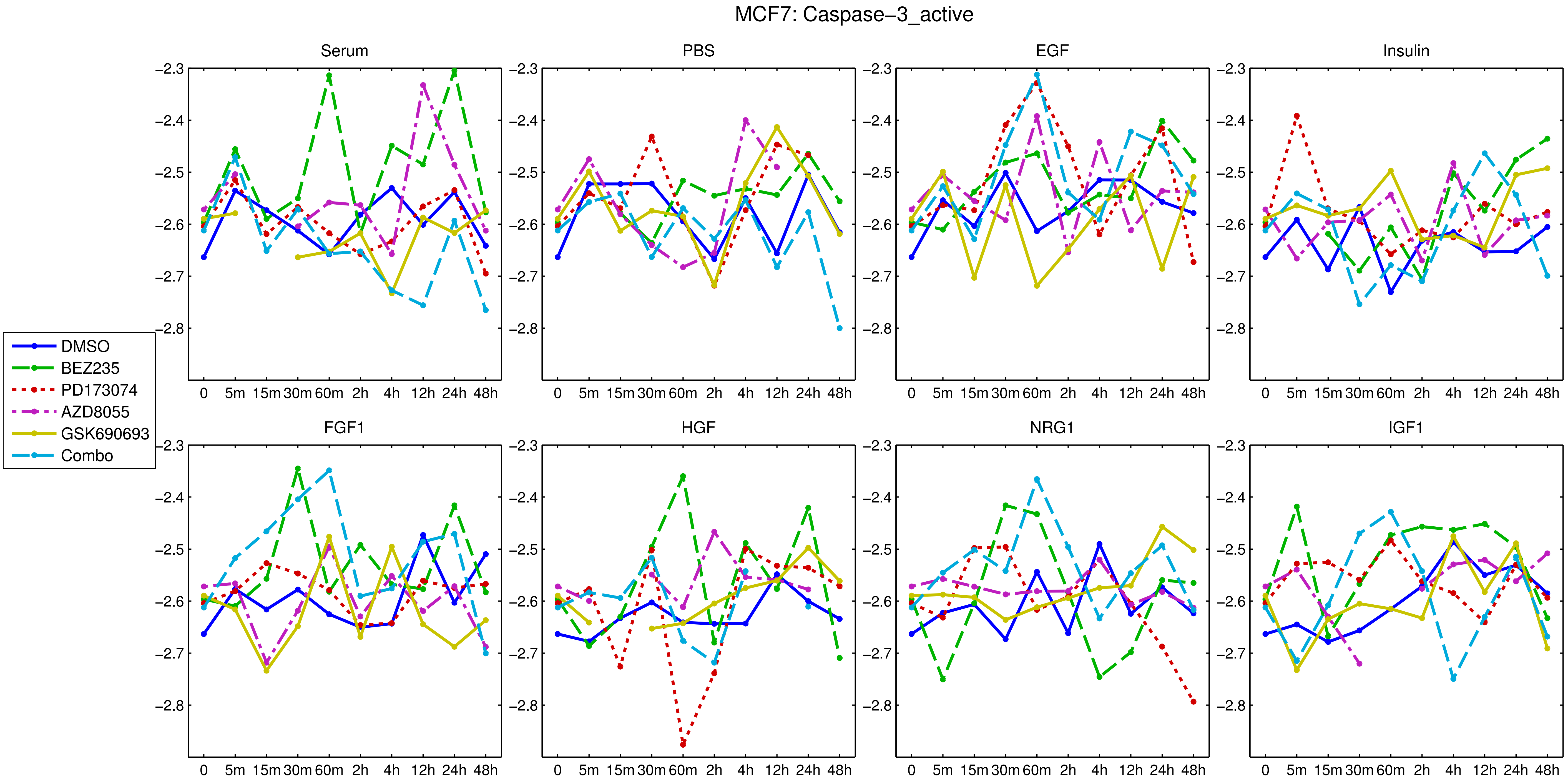

MCF7: Caspase−3_active
Serum
PBS
EGF
Insulin
FGF1
HGF
NRG1
IGF1
−2.3
−2.4
−2.5
−2.6
−2.7
−2.8
0
5m
15m
30m
60m
2h
4h
12h
24h
48h
DMSO
BEZ235
PD173074
AZD8055
GSK690693
Combo

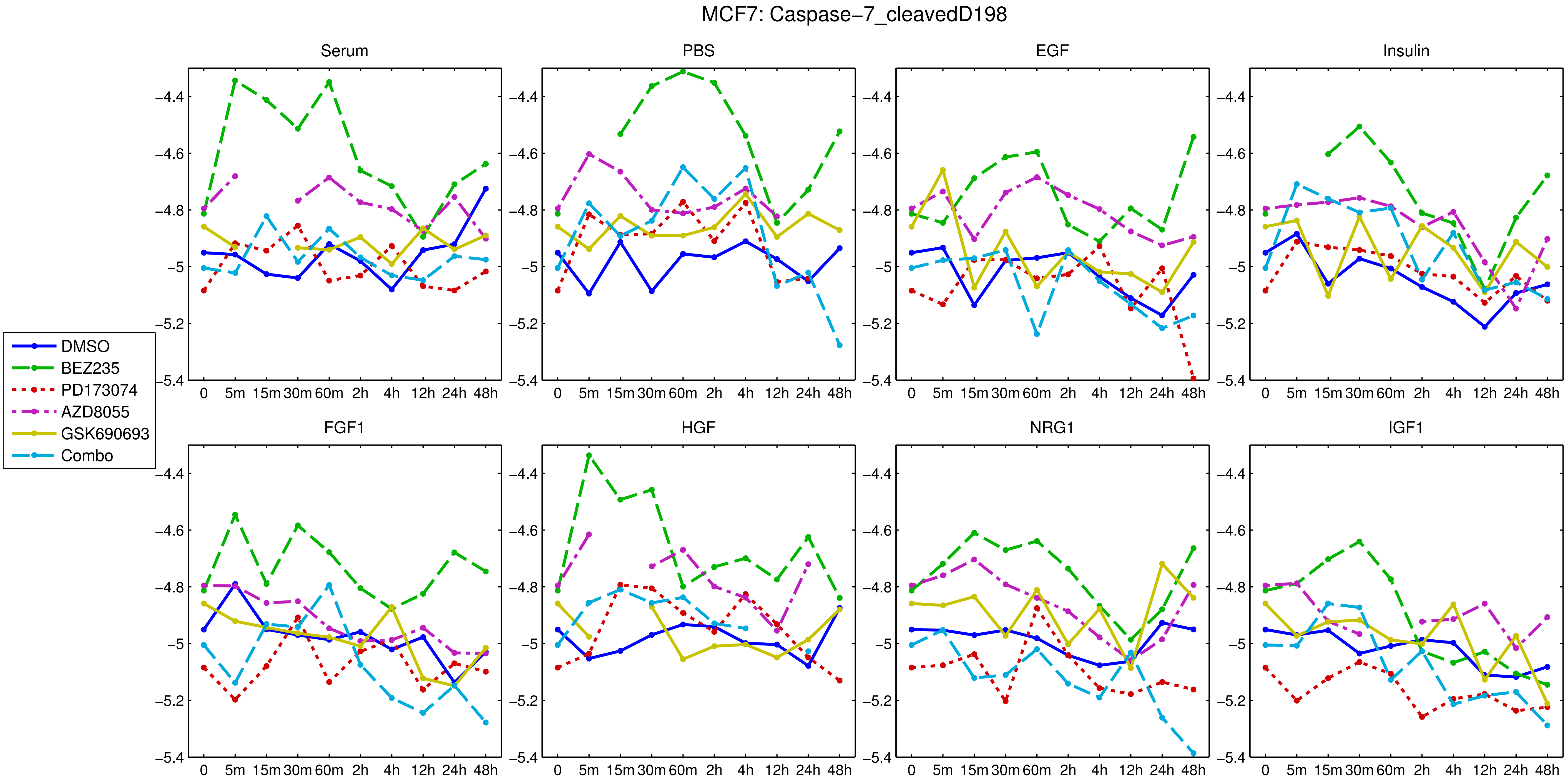
MCF7: Caspase−7_cleavedD198
Serum
PBS
EGF
Insulin
FGF1
HGF
NRG1
IGF1
−4.4
−4.6
−4.8
−5
−5.2
−5.4
0
5m
15m
30m
60m
2h
4h
12h
24h
48h
DMSO
BEZ235
PD173074
AZD8055
GSK690693
Combo

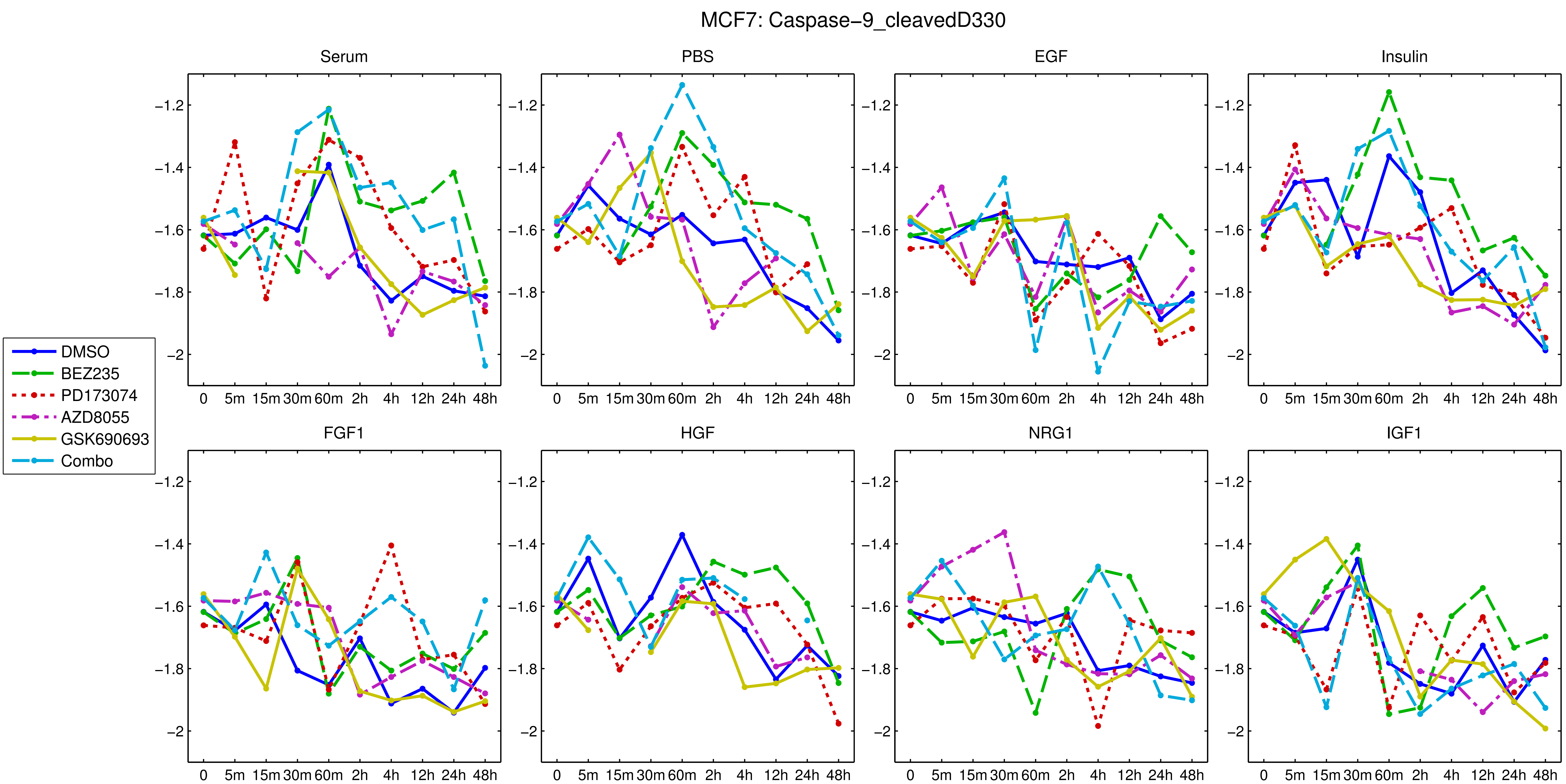

MCF7: Caspase−9_cleavedD330
Serum
PBS
EGF
Insulin
FGF1
HGF
NRG1
IGF1
DMSO
BEZ235
PD173074
AZD8055
GSK690693
Combo
−1.2
−1.4
−1.6
−1.8
−2
0
5m
15m
30m
60m
2h
4h
12h
24h
48h

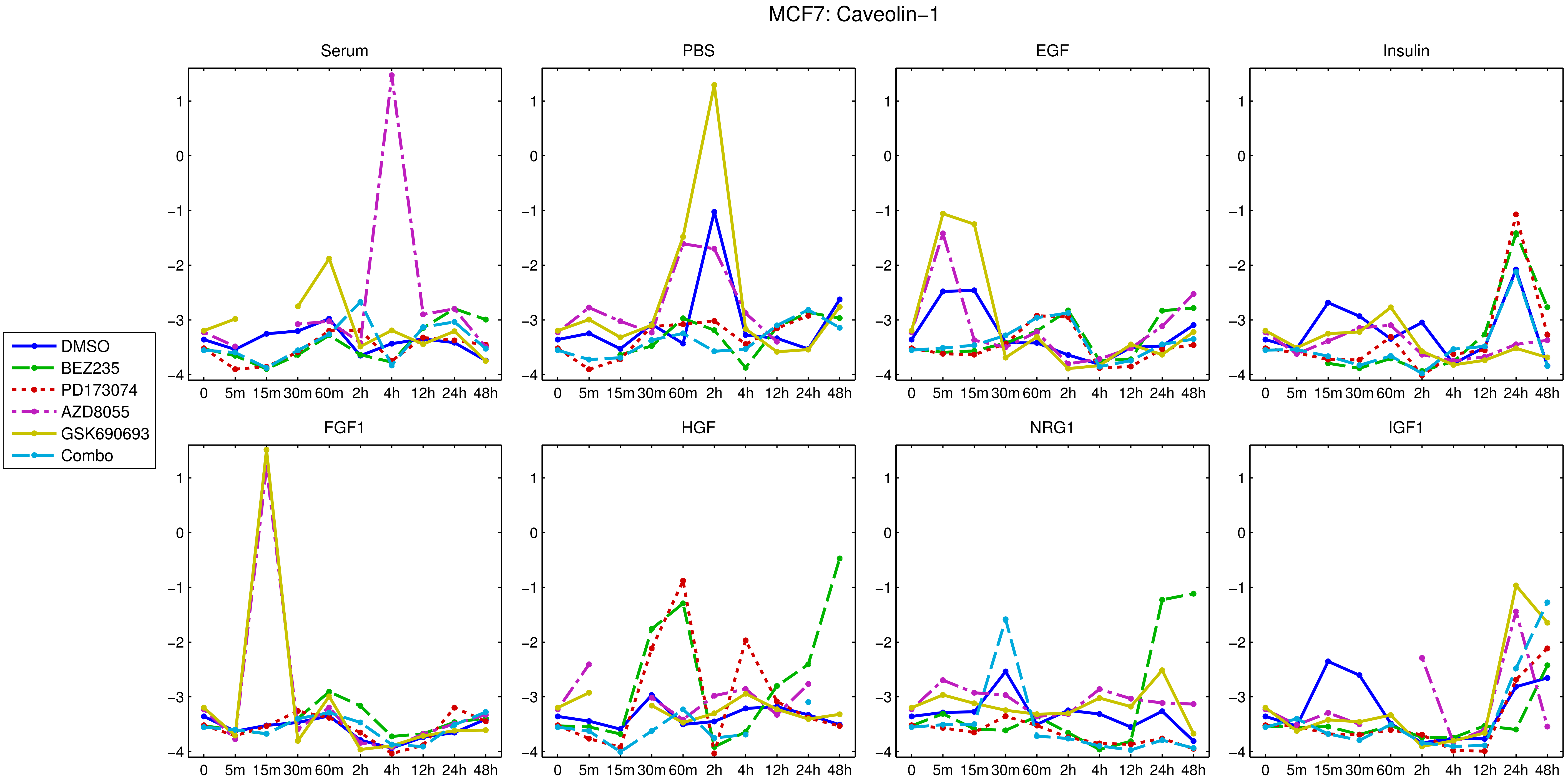
MCF7: Caveolin−1
Serum
PBS
EGF
Insulin
FGF1
HGF
NRG1
IGF1
DMSO
BEZ235
PD173074
AZD8055
GSK690693
Combo
1
0
−1
−2
−3
−4
0
5m
15m
30m
60m
2h
4h
12h
24h
48h

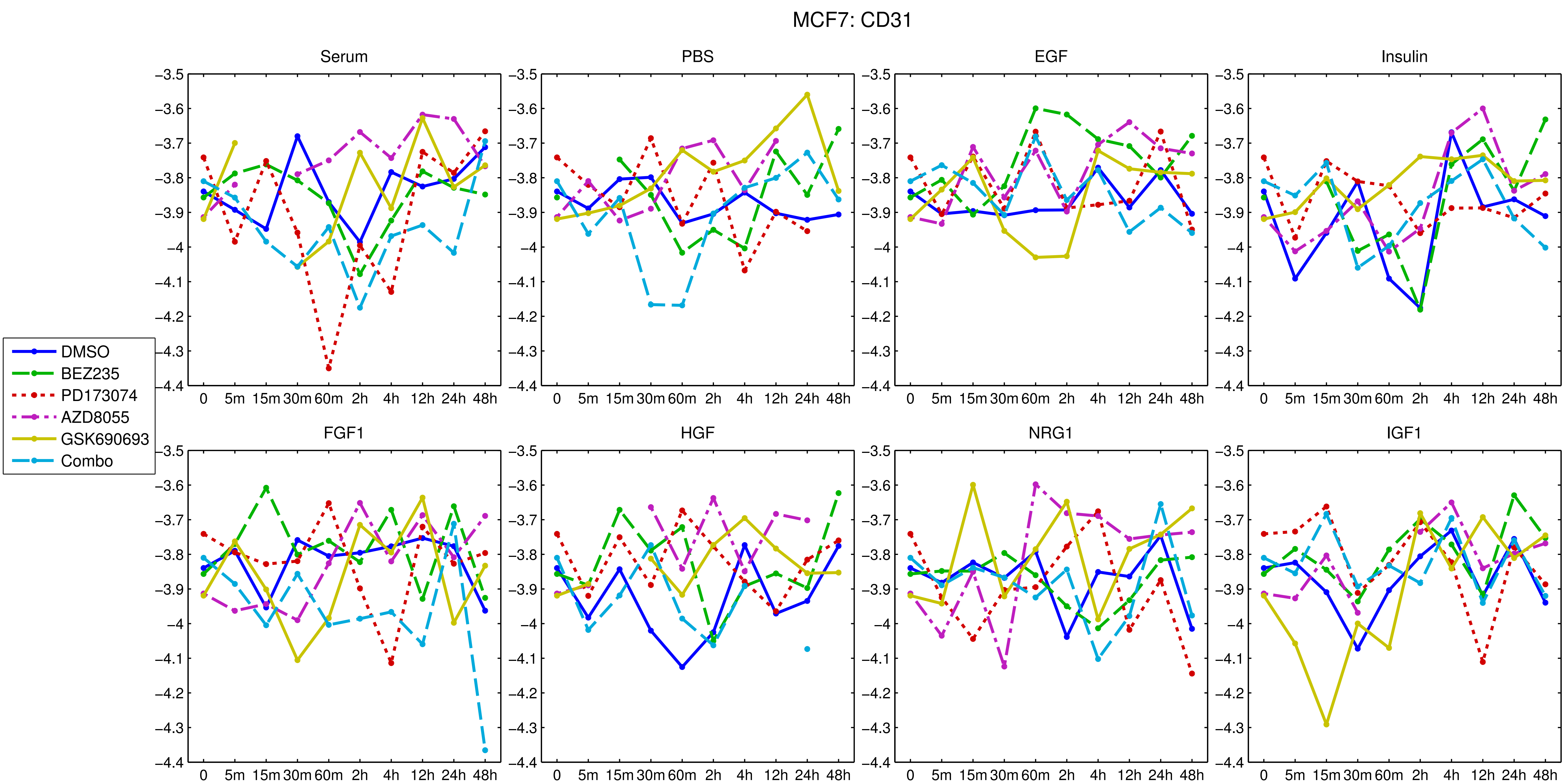

MCF7: CD31
Serum
PBS
EGF
Insulin
FGF1
HGF
NRG1
IGF1
DMSO
BEZ235
PD173074
AZD8055
GSK690693
Combo
−3.5
−3.6
−3.7
−3.8
−3.9
−4
−4.1
−4.2
−4.3
−4.4
0 5m 15m 30m 60m 2h 4h 12h 24h 48h

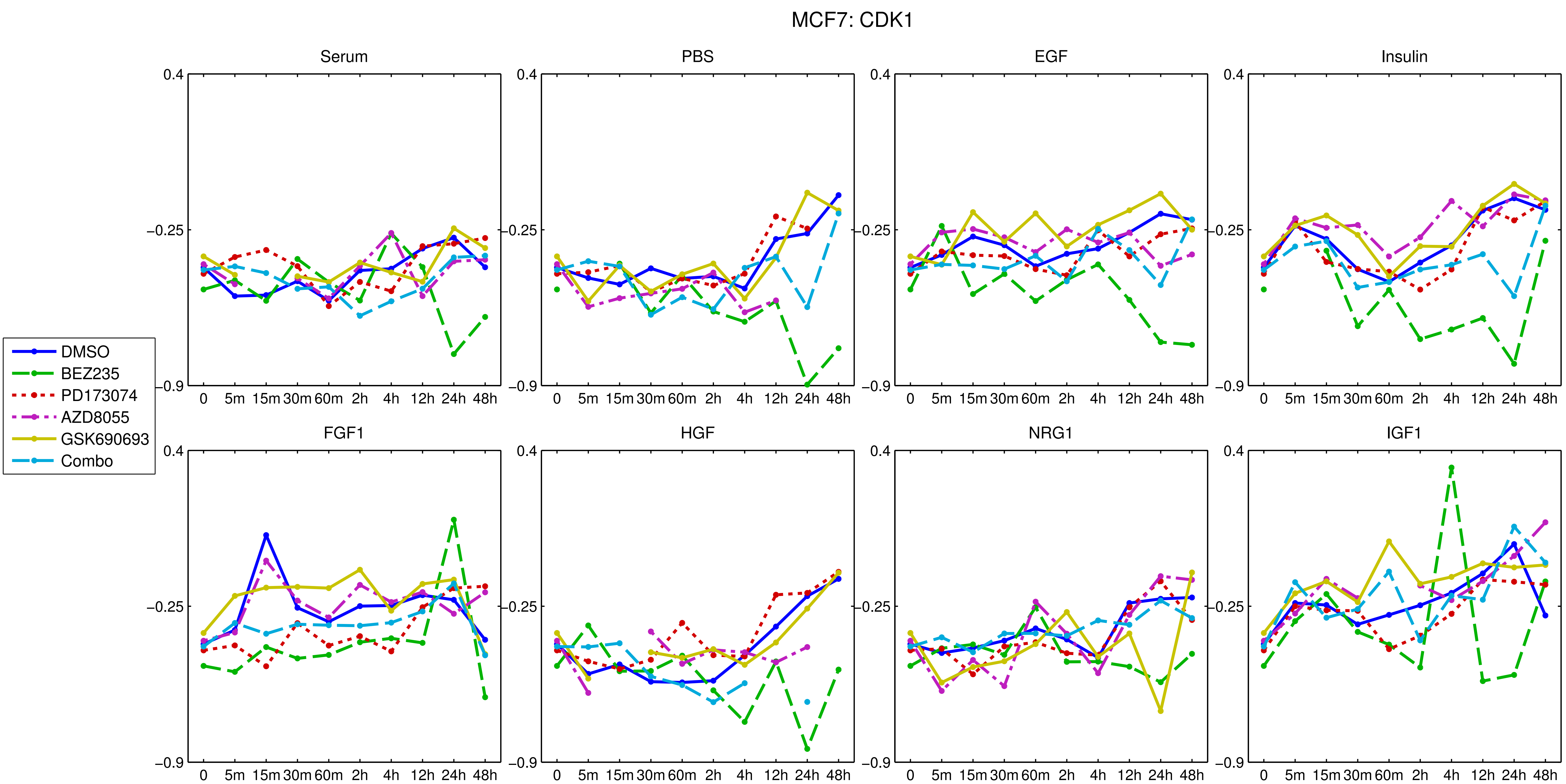

MCF7: CDK1
Serum
PBS
EGF
Insulin
FGF1
HGF
NRG1
IGF1
0.4
−0.25
−0.9
0
5m
15m
30m
60m
2h
4h
12h
24h
48h
DMSO
BEZ235
PD173074
AZD8055
GSK690693
Combo

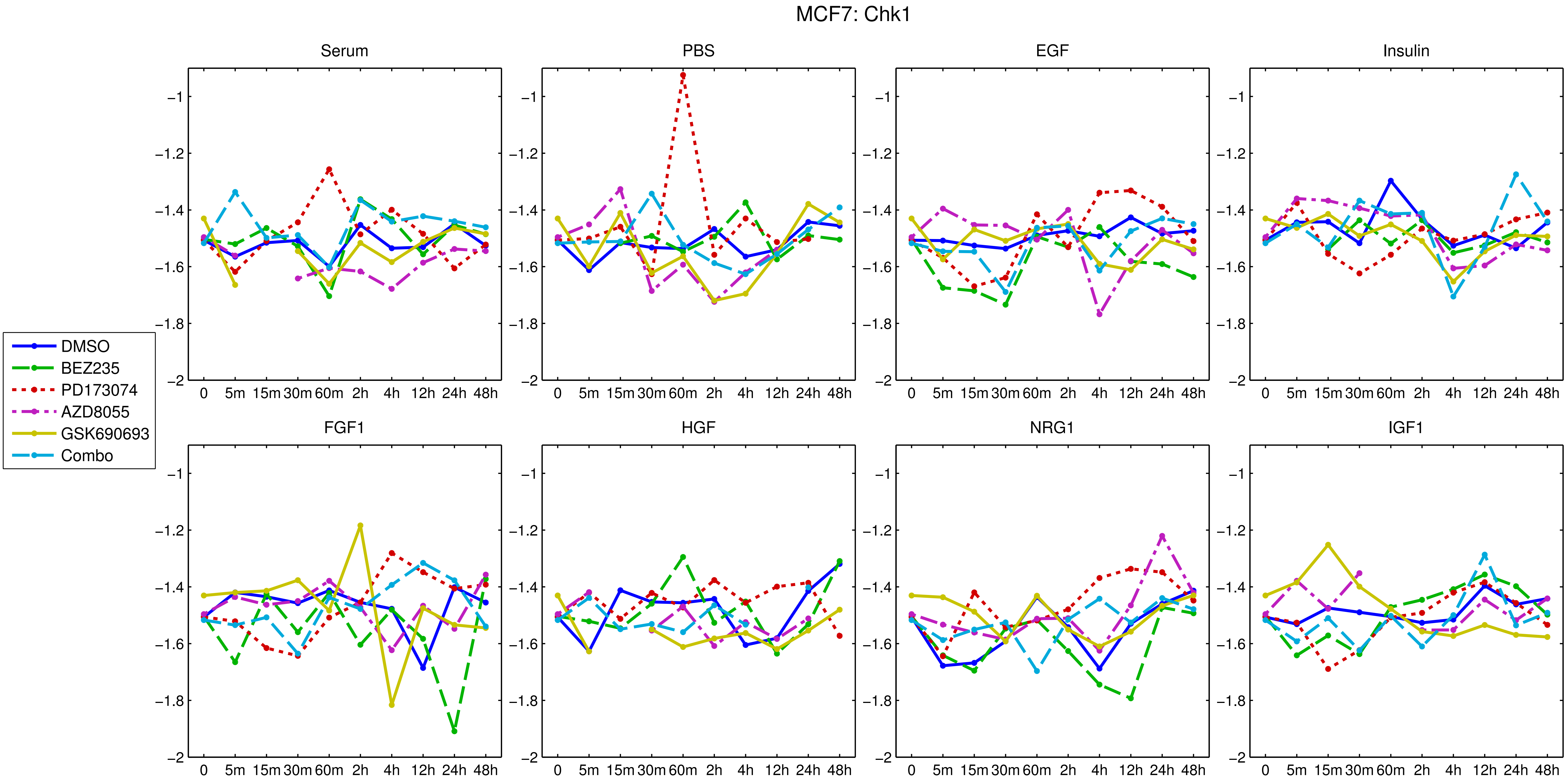
MCF7: Chk1
Serum
PBS
EGF
Insulin
FGF1
HGF
NRG1
IGF1
DMSO
BEZ235
PD173074
AZD8055
GSK690693
Combo
−1
−1.2
−1.4
−1.6
−1.8
−2
0 5m 15m 30m 60m 2h 4h 12h 24h 48h

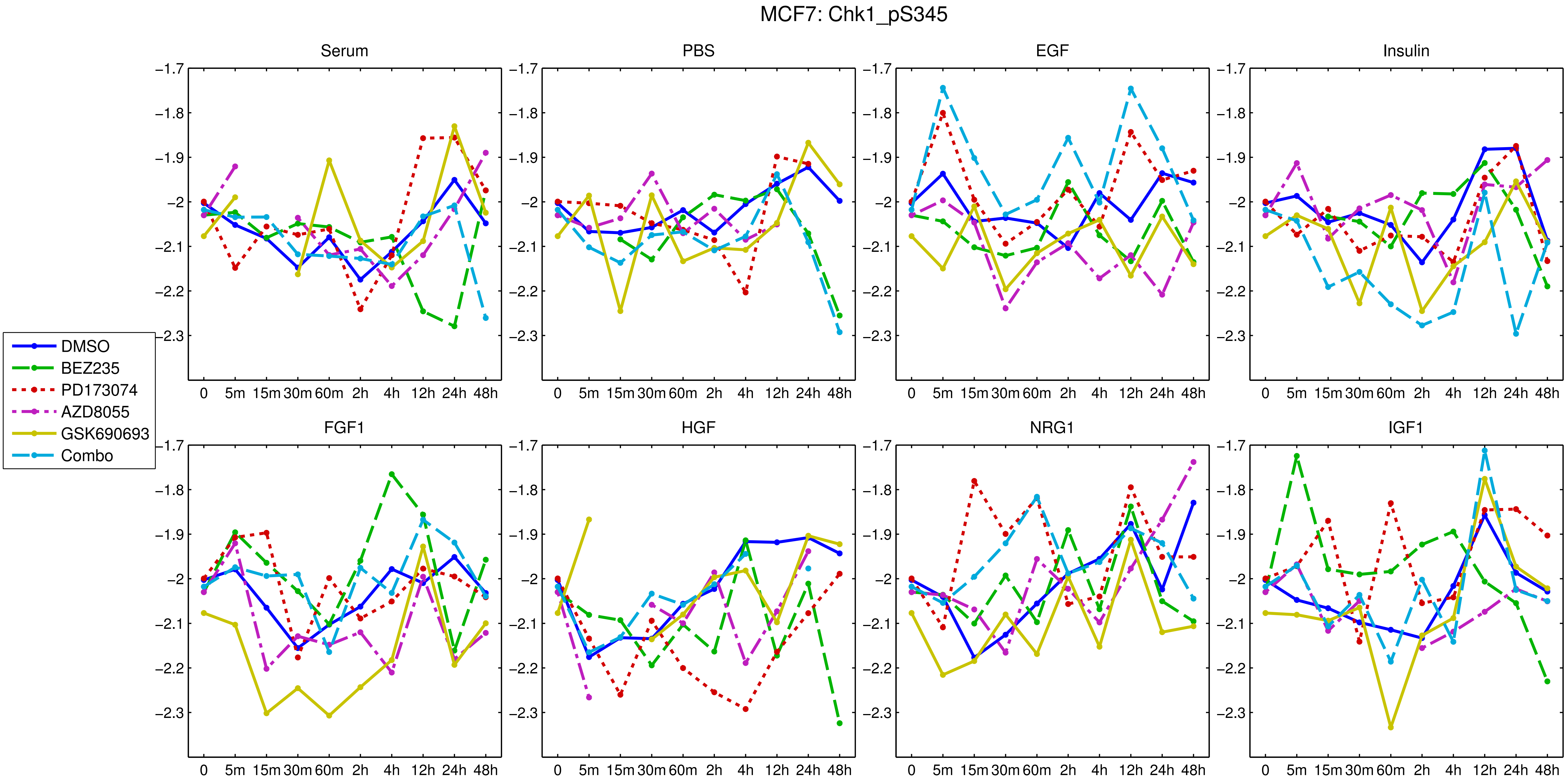

MCF7: Chk1_pS345
Serum
PBS
EGF
Insulin
FGF1
HGF
NRG1
IGF1
−1.7
−1.8
−1.9
−2
−2.1
−2.2
−2.3
0
5m
15m
30m
60m
2h
4h
12h
24h
48h
DMSO
BEZ235
PD173074
AZD8055
GSK690693
Combo

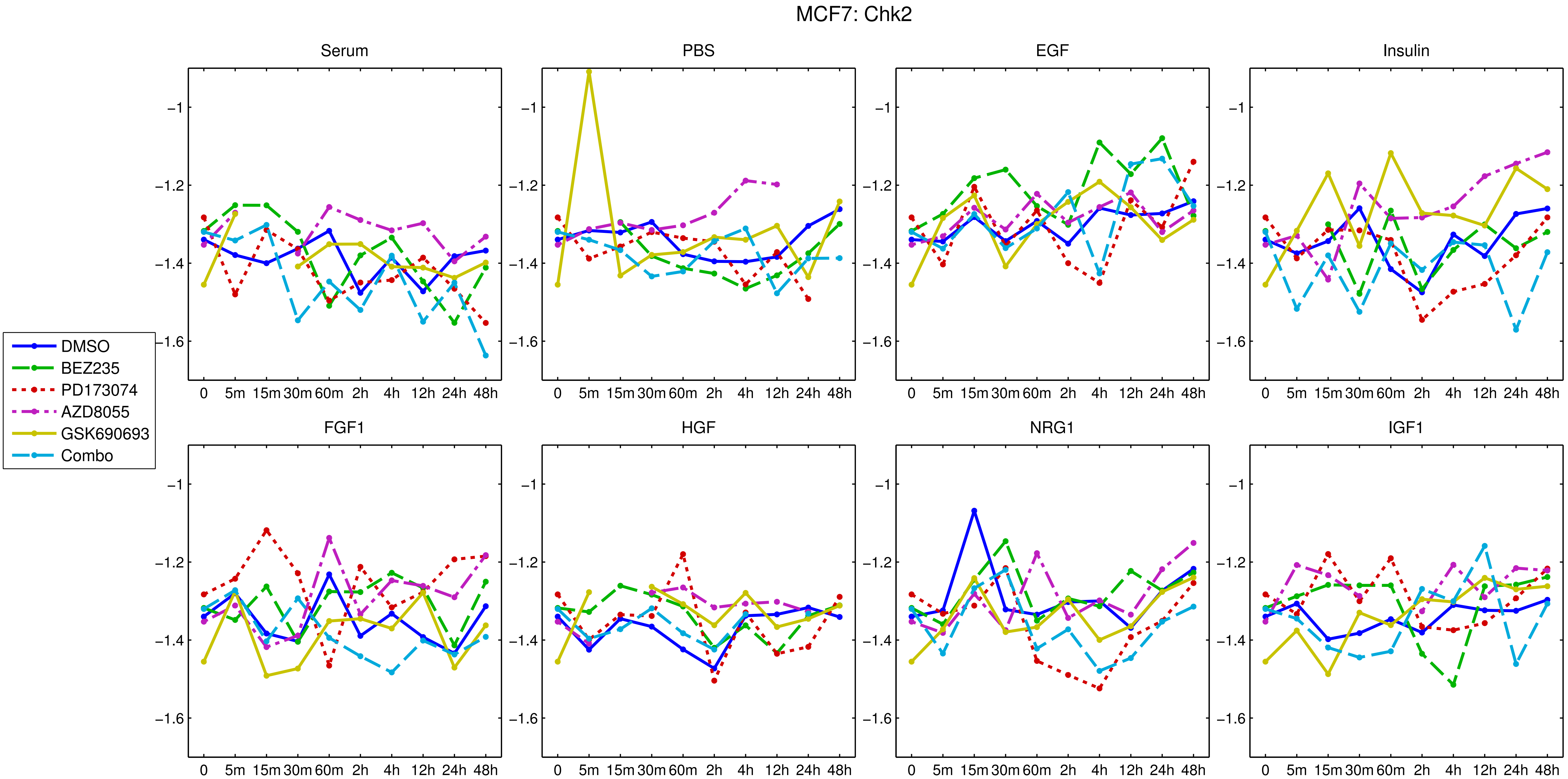

MCF7: Chk2
Serum
PBS
EGF
Insulin
FGF1
HGF
NRG1
IGF1
DMSO
BEZ235
PD173074
AZD8055
GSK690693
Combo
−1
−1.2
−1.4
−1.6
0
5m
15m
30m
60m
2h
4h
12h
24h
48h

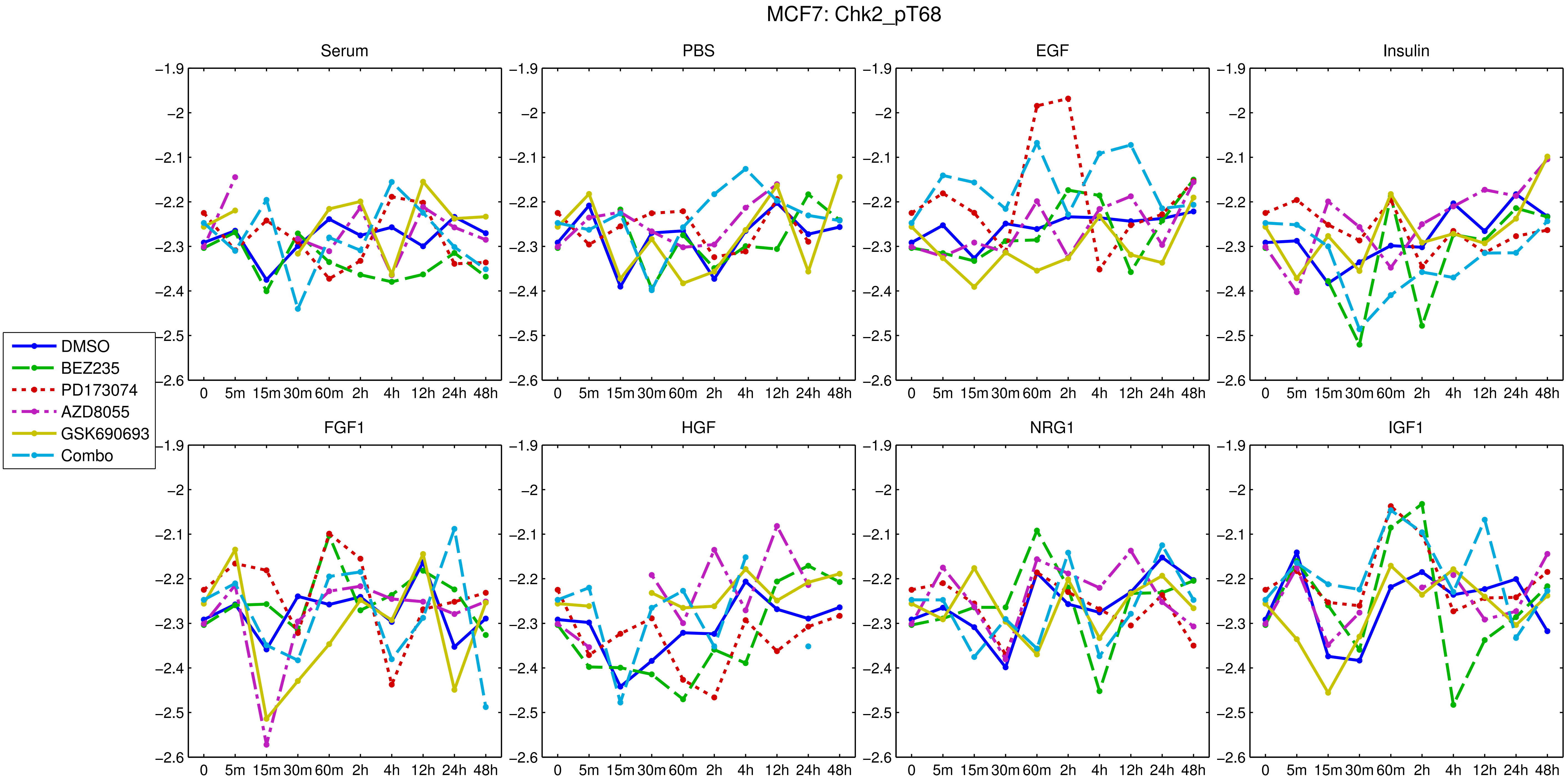

MCF7: Chk2_pT68
Serum
PBS
EGF
Insulin
FGF1
HGF
NRG1
IGF1
−1.9
−2
−2.1
−2.2
−2.3
−2.4
−2.5
−2.6
0
5m
15m
30m
60m
2h
4h
12h
24h
48h
DMSO
BEZ235
PD173074
AZD8055
GSK690693
Combo

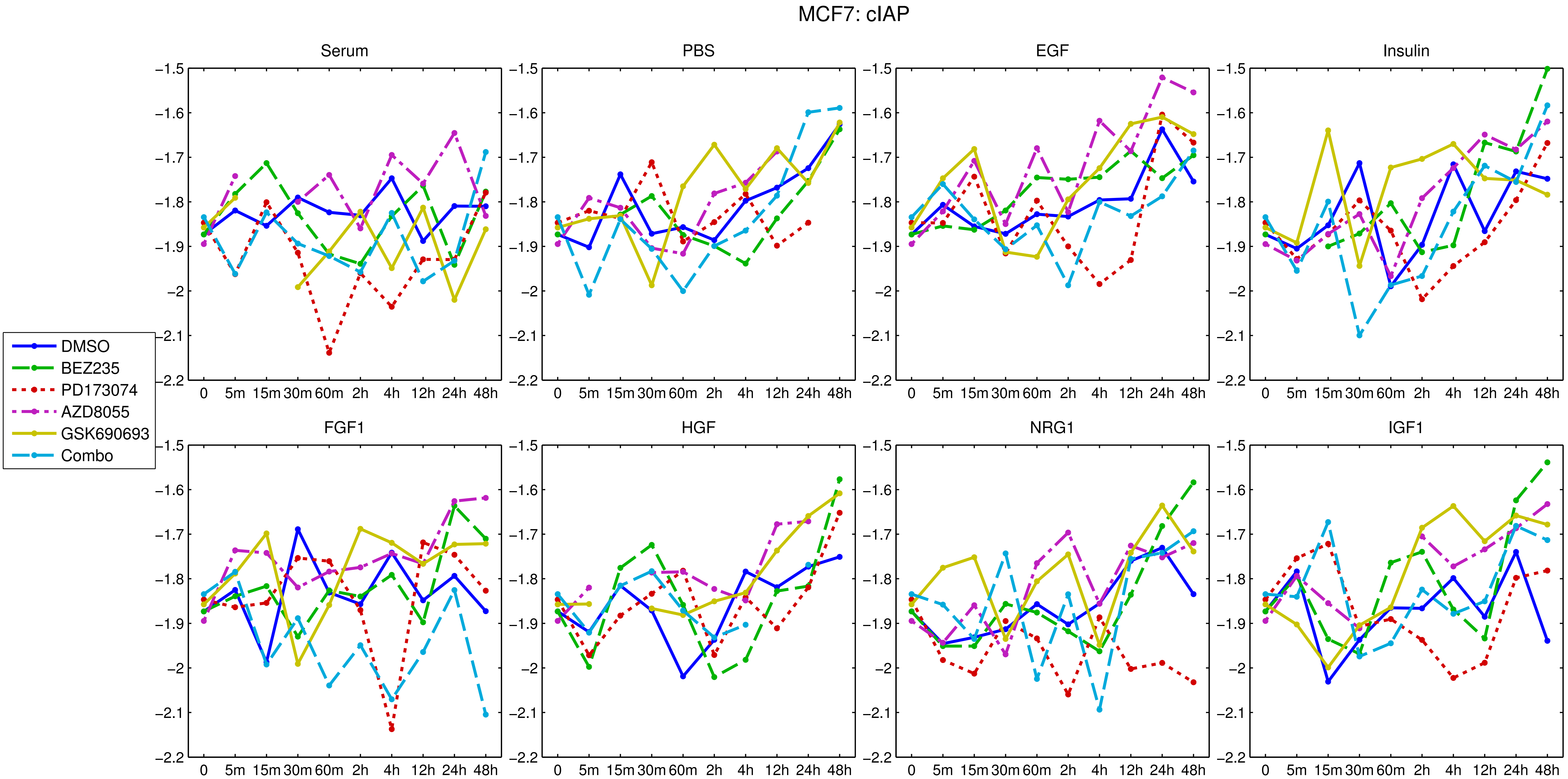
MCF7: cIAP
Serum
PBS
EGF
Insulin
FGF1
HGF
NRG1
IGF1
DMSO
BEZ235
PD173074
AZD8055
GSK690693
Combo
−1.5
−1.6
−1.7
−1.8
−1.9
−2
−2.1
−2.2
0
5m
15m
30m
60m
2h
4h
12h
24h
48h

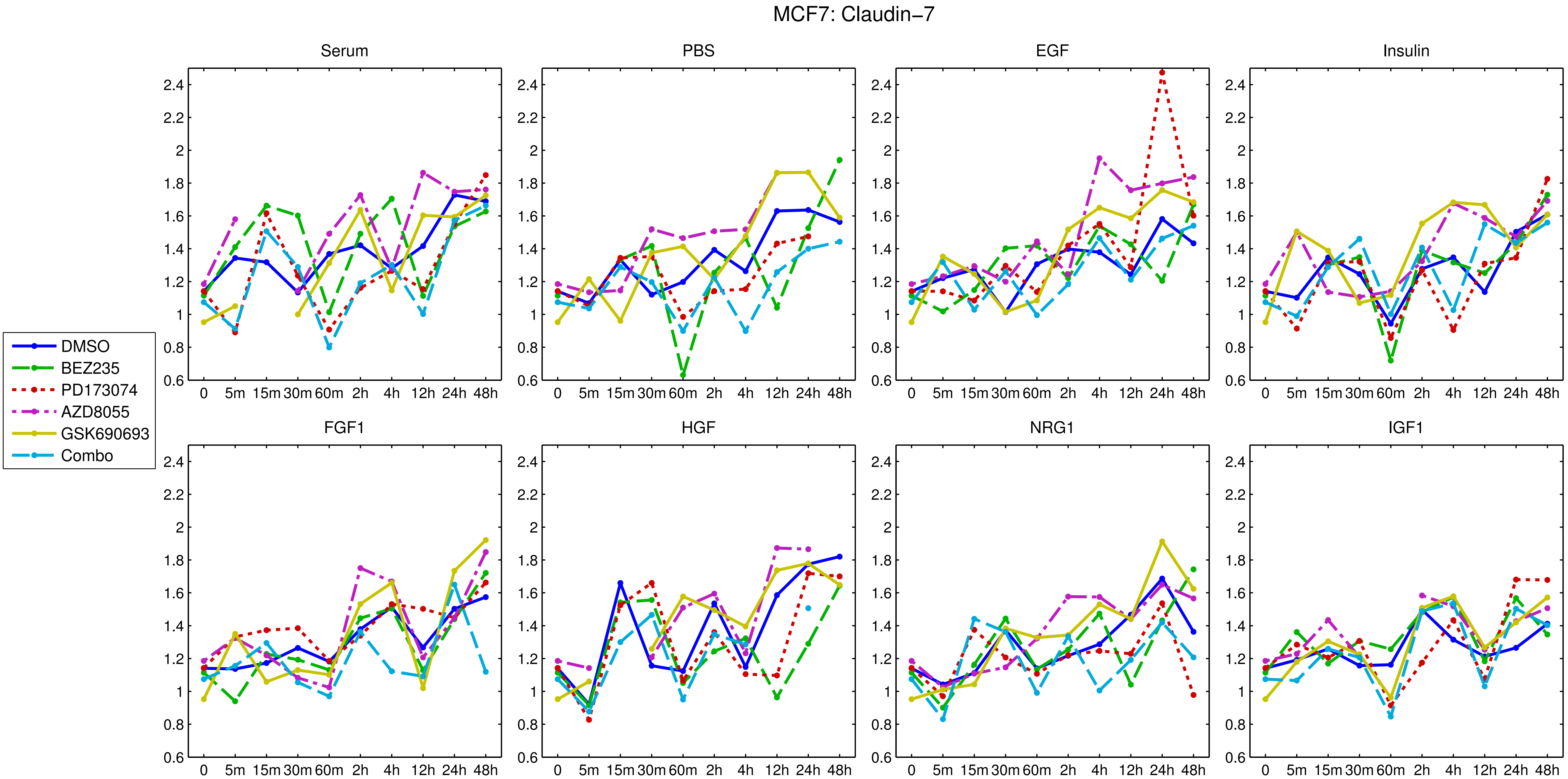

MCF7: Claudin−7
Serum
PBS
EGF
Insulin
FGF1
HGF
NRG1
IGF1
DMSO
BEZ235
PD173074
AZD8055
GSK690693
Combo
0.6
0.8
1
1.2
1.4
1.6
1.8
2
2.2
2.4
0
5m
15m
30m
60m
2h
4h
12h
24h
48h

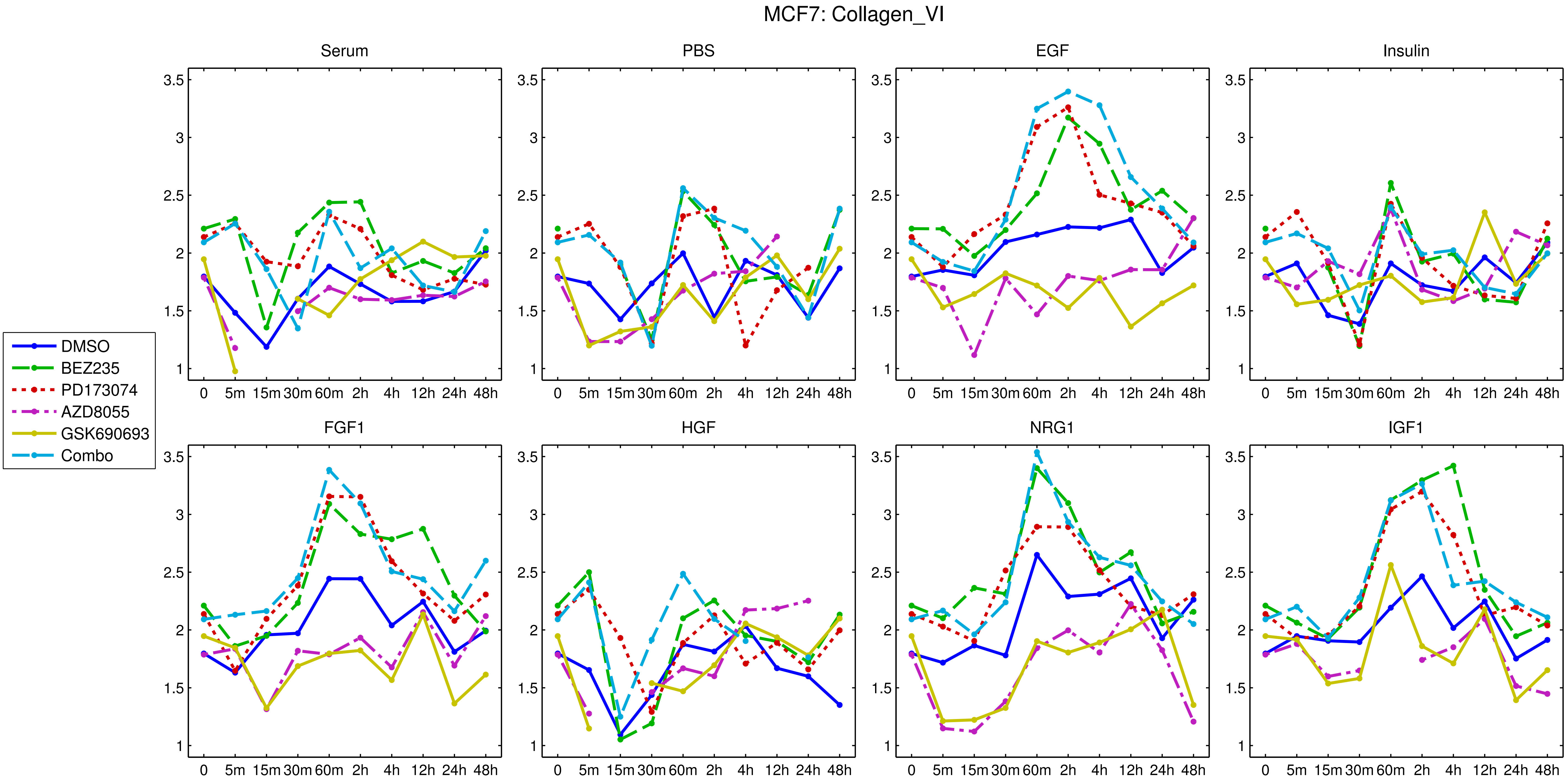
MCF7: Collagen_VI
Serum
PBS
EGF
Insulin
FGF1
HGF
NRG1
IGF1
3.5
3
2.5
2
1.5
1
0
5m
15m
30m
60m
2h
4h
12h
24h
48h
DMSO
BEZ235
PD173074
AZD8055
GSK690693
Combo

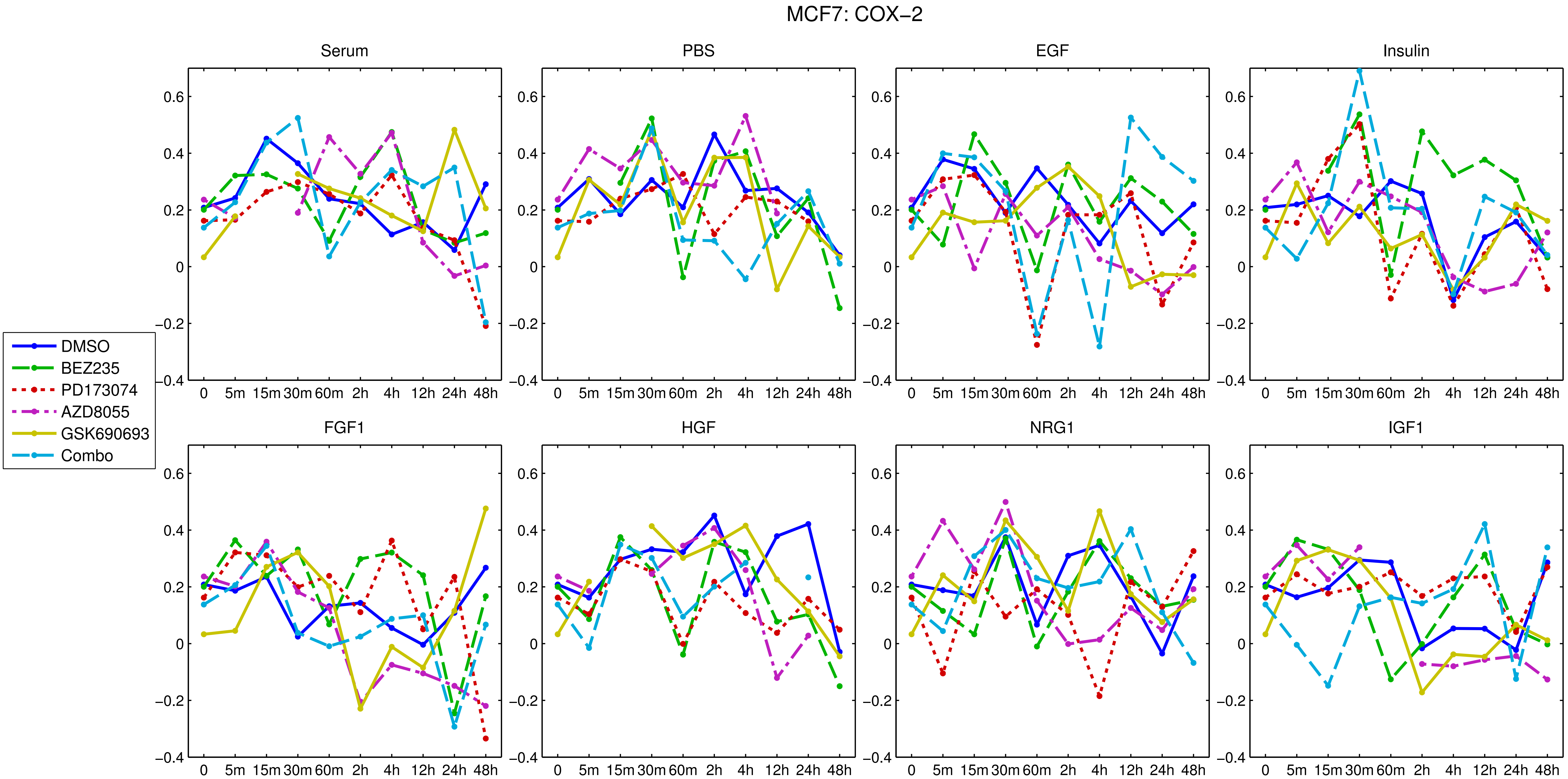

MCF7: COX−2
Serum
PBS
EGF
Insulin
FGF1
HGF
NRG1
IGF1
DMSO
BEZ235
PD173074
AZD8055
GSK690693
Combo
0.6
0.4
0.2
0
−0.2
−0.4
0
5m
15m
30m
60m
2h
4h
12h
24h
48h

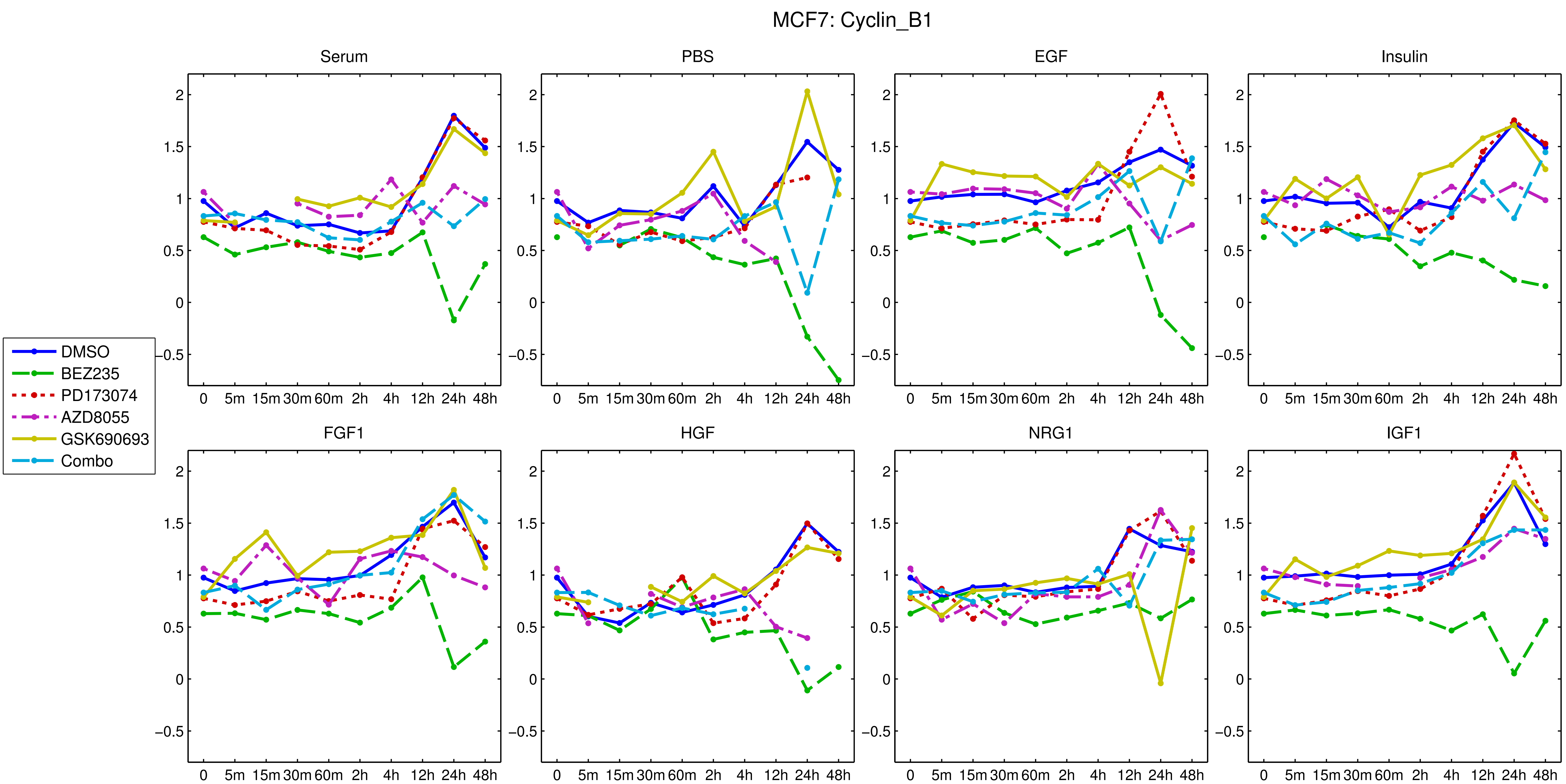
MCF7: Cyclin_B1
Serum
PBS
EGF
Insulin
FGF1
HGF
NRG1
IGF1
DMSO
BEZ235
PD173074
AZD8055
GSK690693
Combo
0
5m
15m
30m
60m
2h
4h
12h
24h
48h
−0.5
0
0.5
1
1.5
2

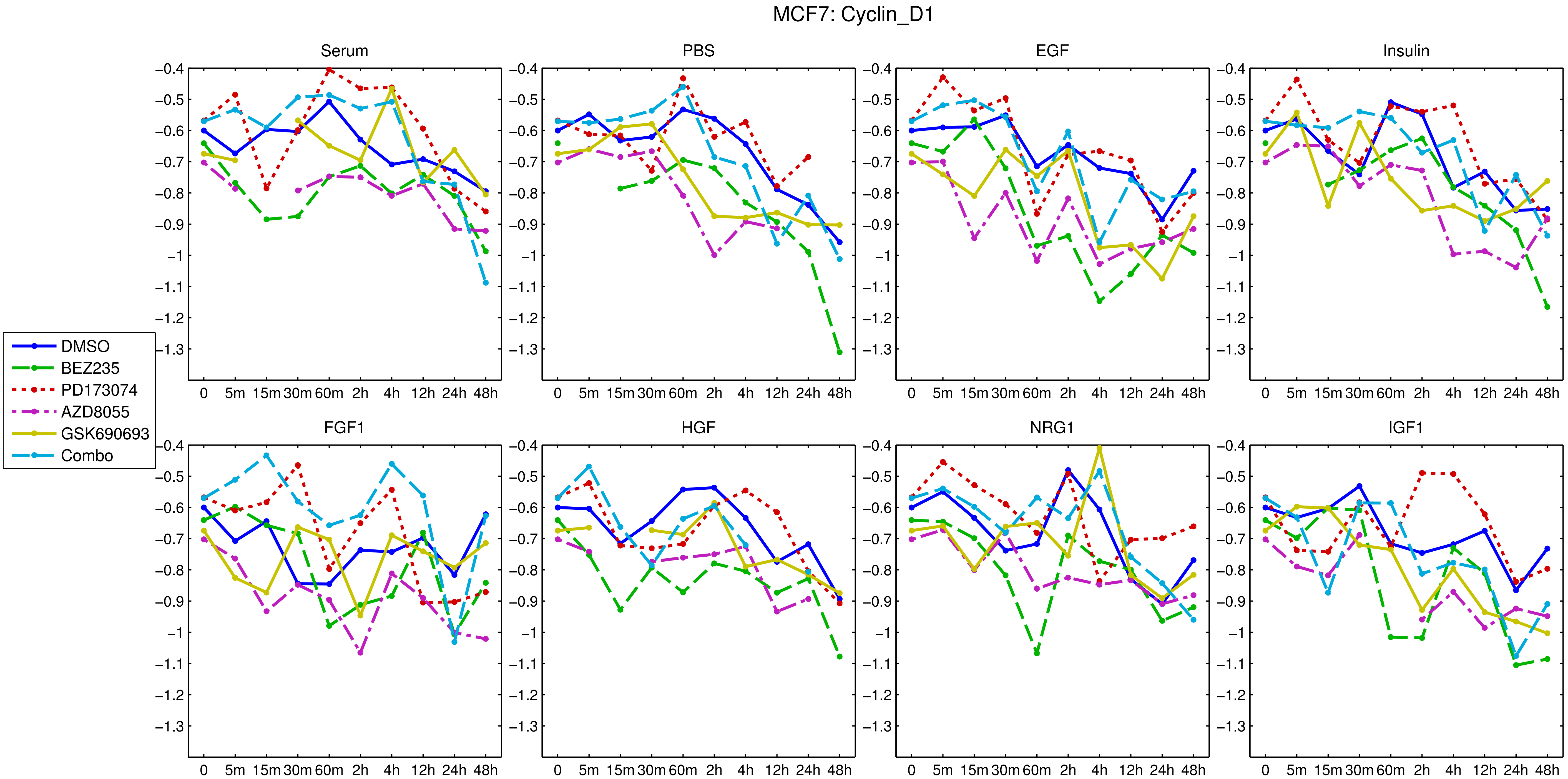

MCF7: Cyclin_D1
Serum
PBS
EGF
Insulin
FGF1
HGF
NRG1
IGF1
DMSO
BEZ235
PD173074
AZD8055
GSK690693
Combo
−0.4
−0.5
−0.6
−0.7
−0.8
−0.9
−1
−1.1
−1.2
−1.3
0
5m
15m
30m
60m
2h
4h
12h
24h
48h

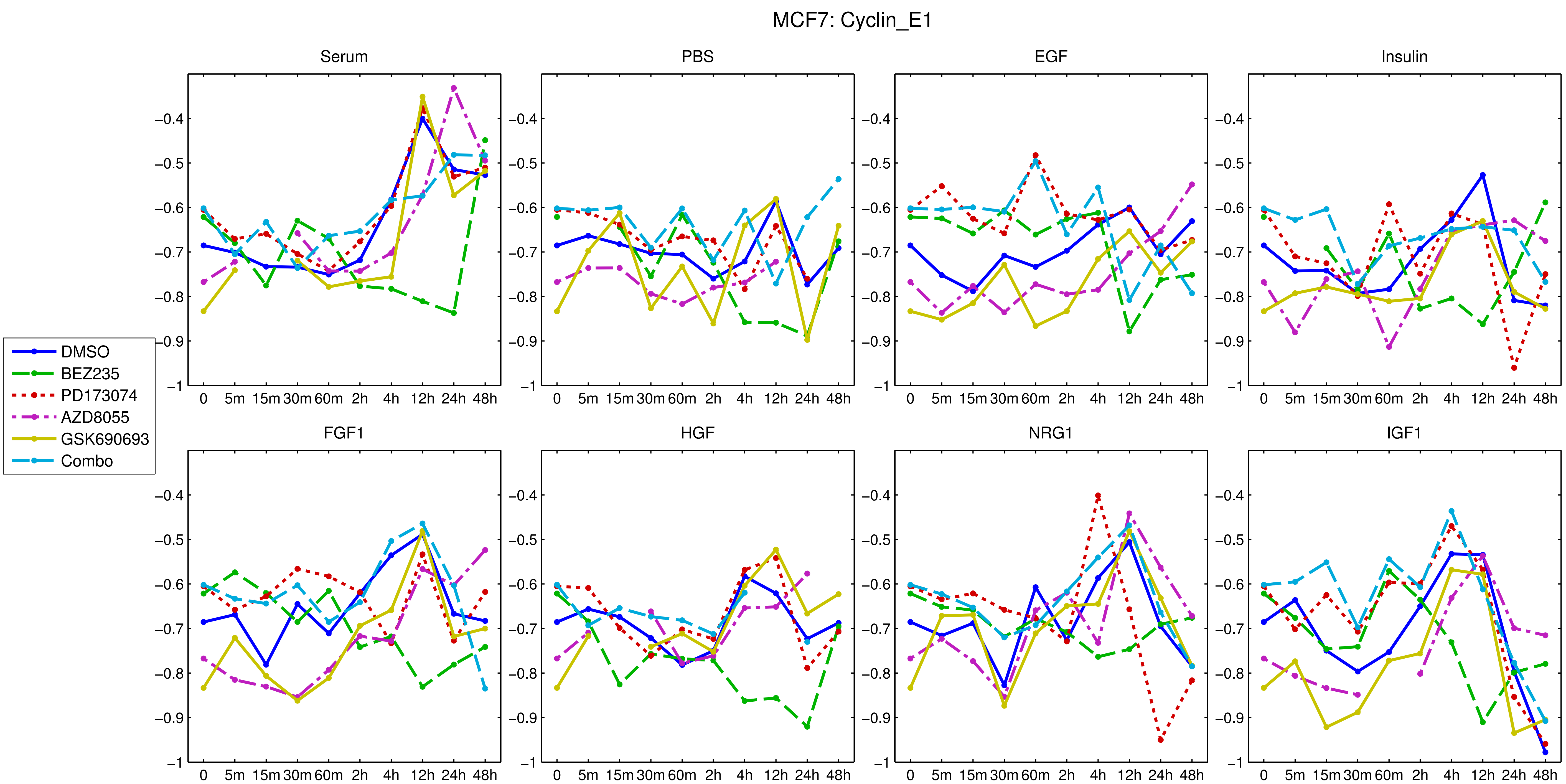
MCF7: Cyclin_E1
Serum
PBS
EGF
Insulin
FGF1
HGF
NRG1
IGF1
DMSO
BEZ235
PD173074
AZD8055
GSK690693
Combo
−0.4
−0.5
−0.6
−0.7
−0.8
−0.9
−1
0
5m
15m
30m
60m
2h
4h
12h
24h
48h

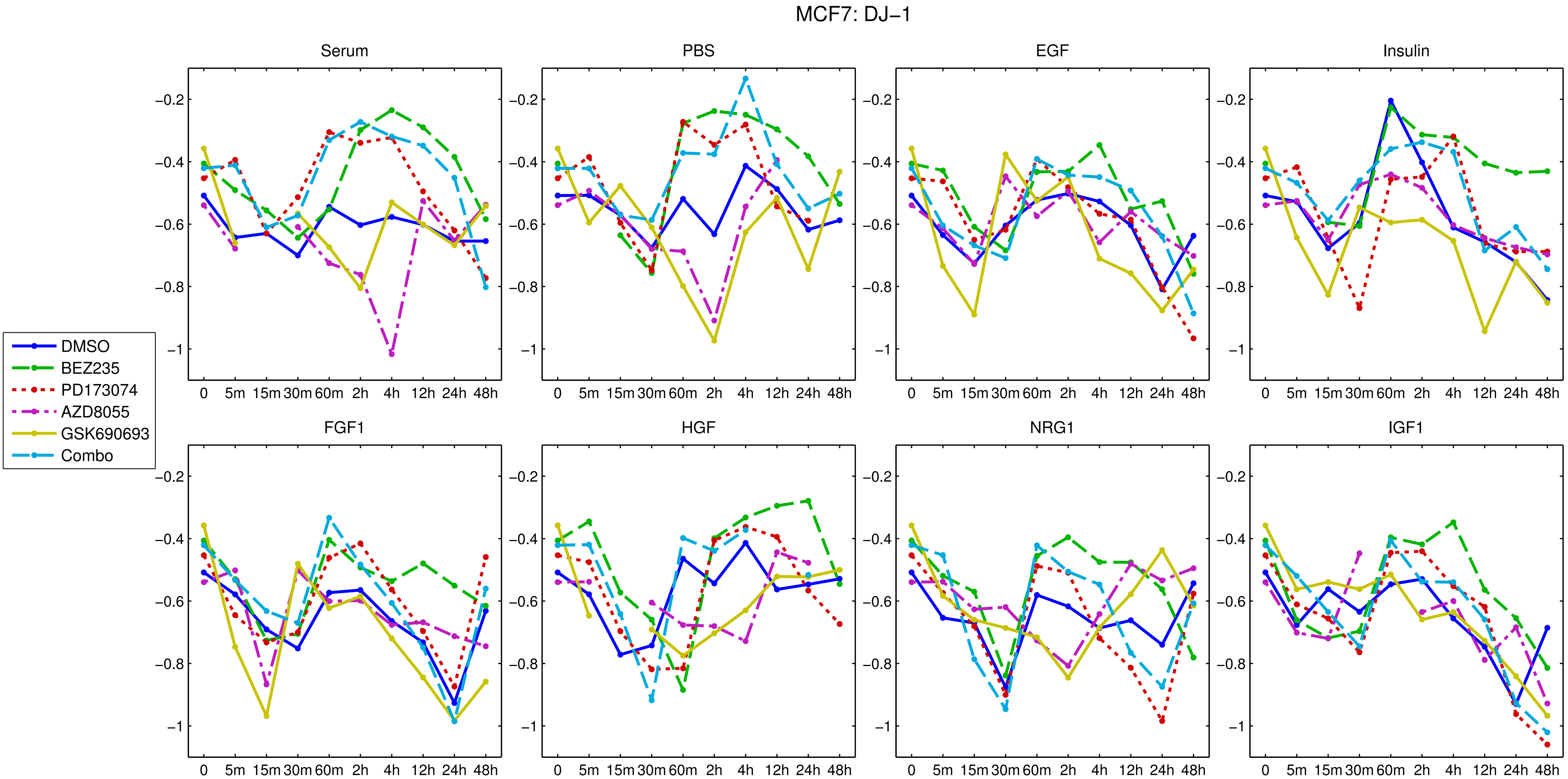
MCF7: DJ–1
Serum
PBS
EGF
Insulin
FGF1
HGF
NRG1
IGF1
DMSO
BEZ235
PD173074
AZD8055
GSK690693
Combo
−0.2
−0.4
−0.6
−0.8
−1
0
5m
15m
30m
60m
2h
4h
12h
24h
48h

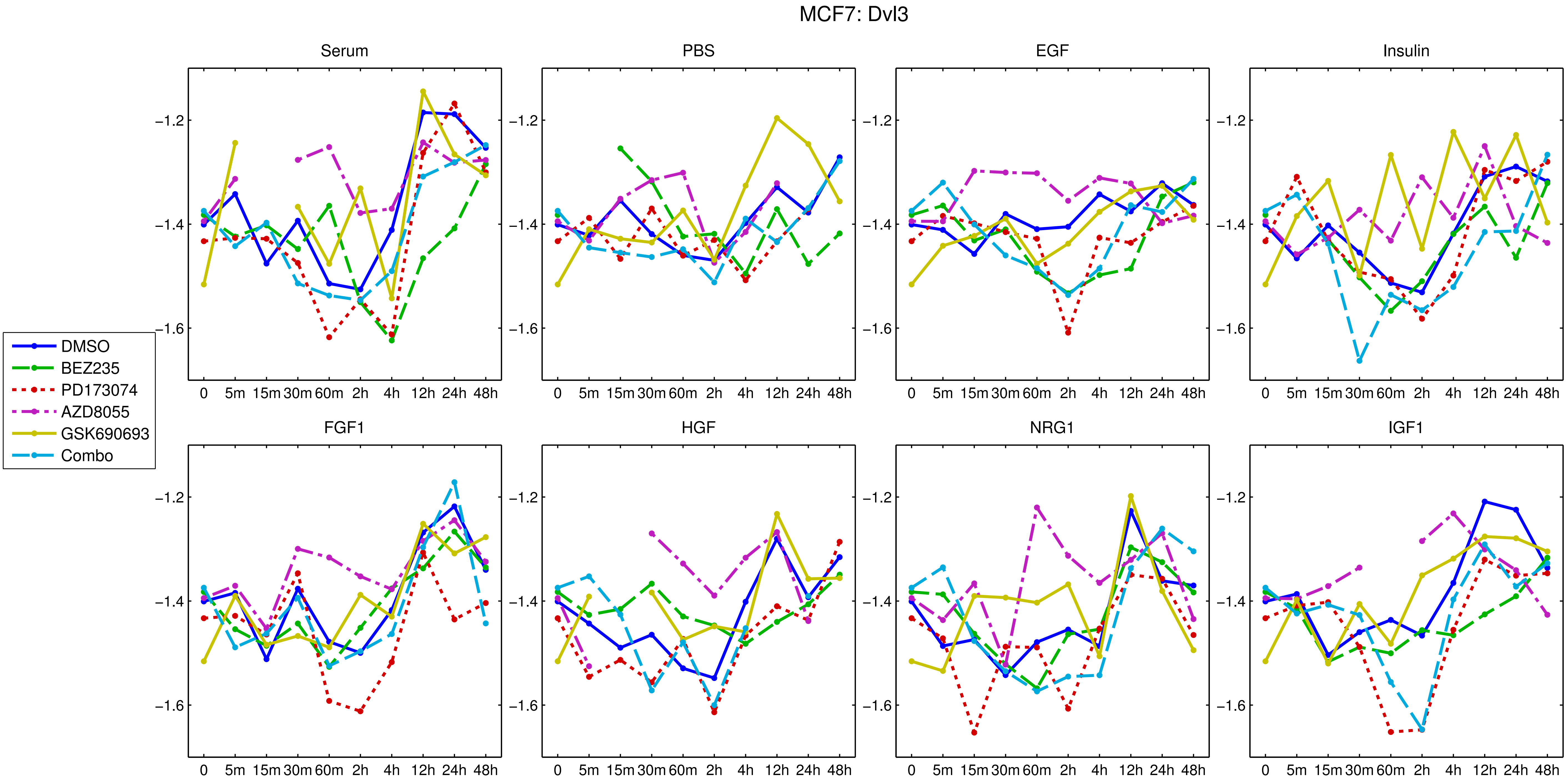
MCF7: Dvl3
Serum
PBS
EGF
Insulin
FGF1
HGF
NRG1
IGF1
−1.2
−1.4
−1.6
0
5m
15m
30m
60m
2h
4h
12h
24h
48h
DMSO
BEZ235
PD173074
AZD8055
GSK690693
Combo

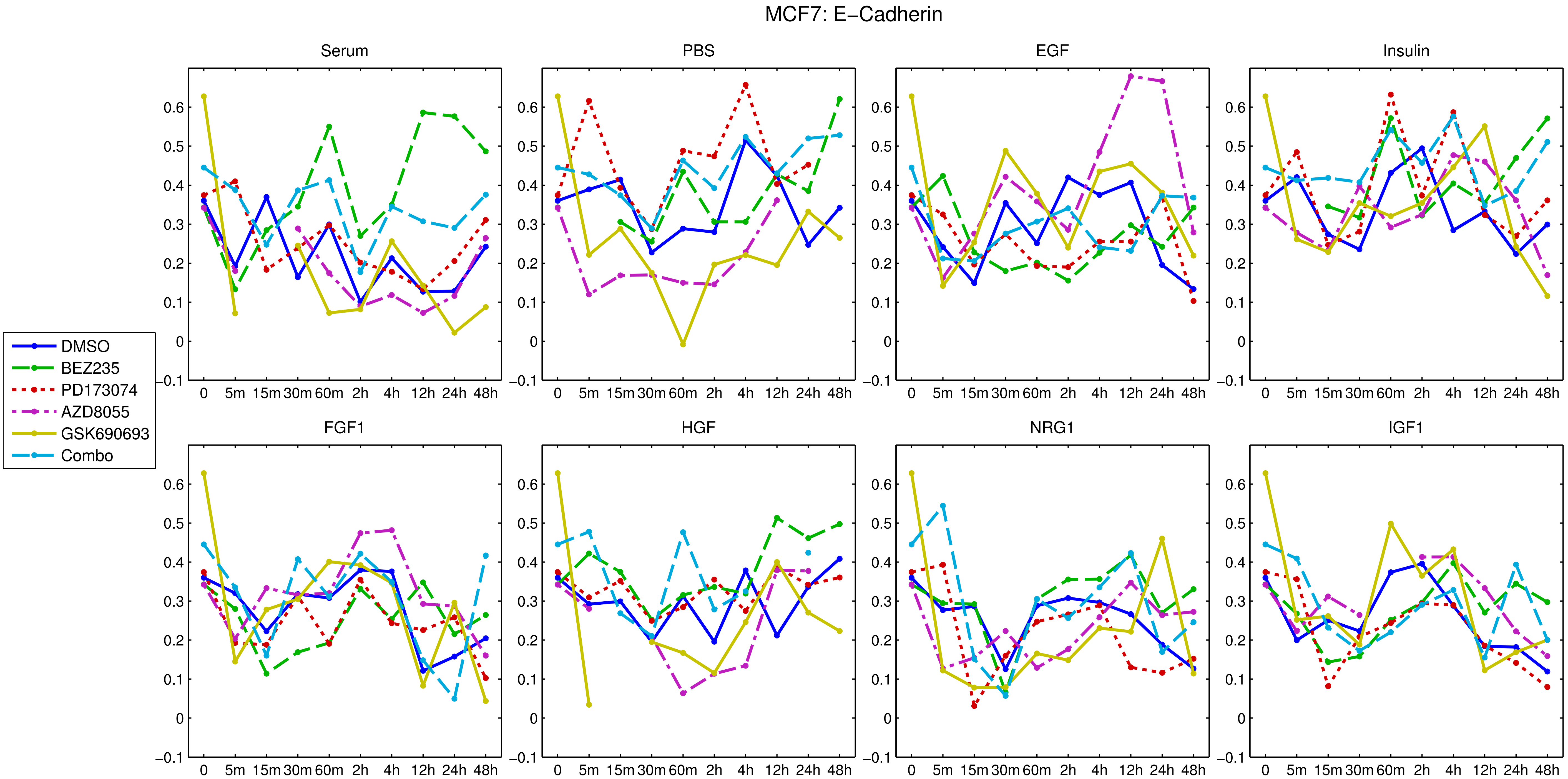
MCF7: E−Cadherin
Serum
PBS
EGF
Insulin
FGF1
HGF
NRG1
IGF1
DMSO
BEZ235
PD173074
AZD8055
GSK690693
Combo
0.6
0.5
0.4
0.3
0.2
0.1
0
−0.1
0
5m
15m
30m
60m
2h
4h
12h
24h
48h

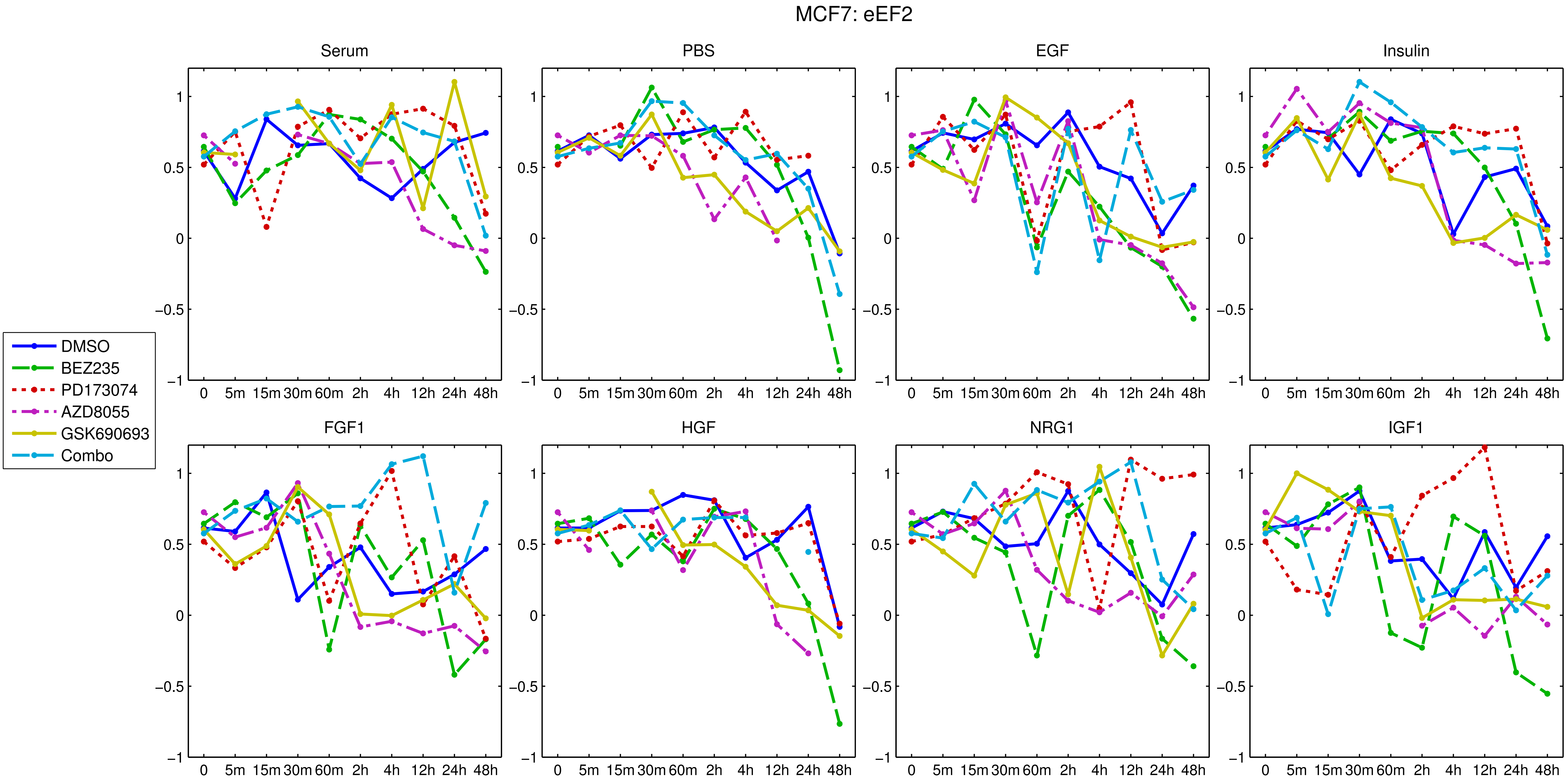

MCF7: eEF2
Serum
PBS
EGF
Insulin
FGF1
HGF
NRG1
IGF1
DMSO
BEZ235
PD173074
AZD8055
GSK690693
Combo
1
0.5
0
−0.5
−1
0
5m
15m
30m
60m
2h
4h
12h
24h
48h

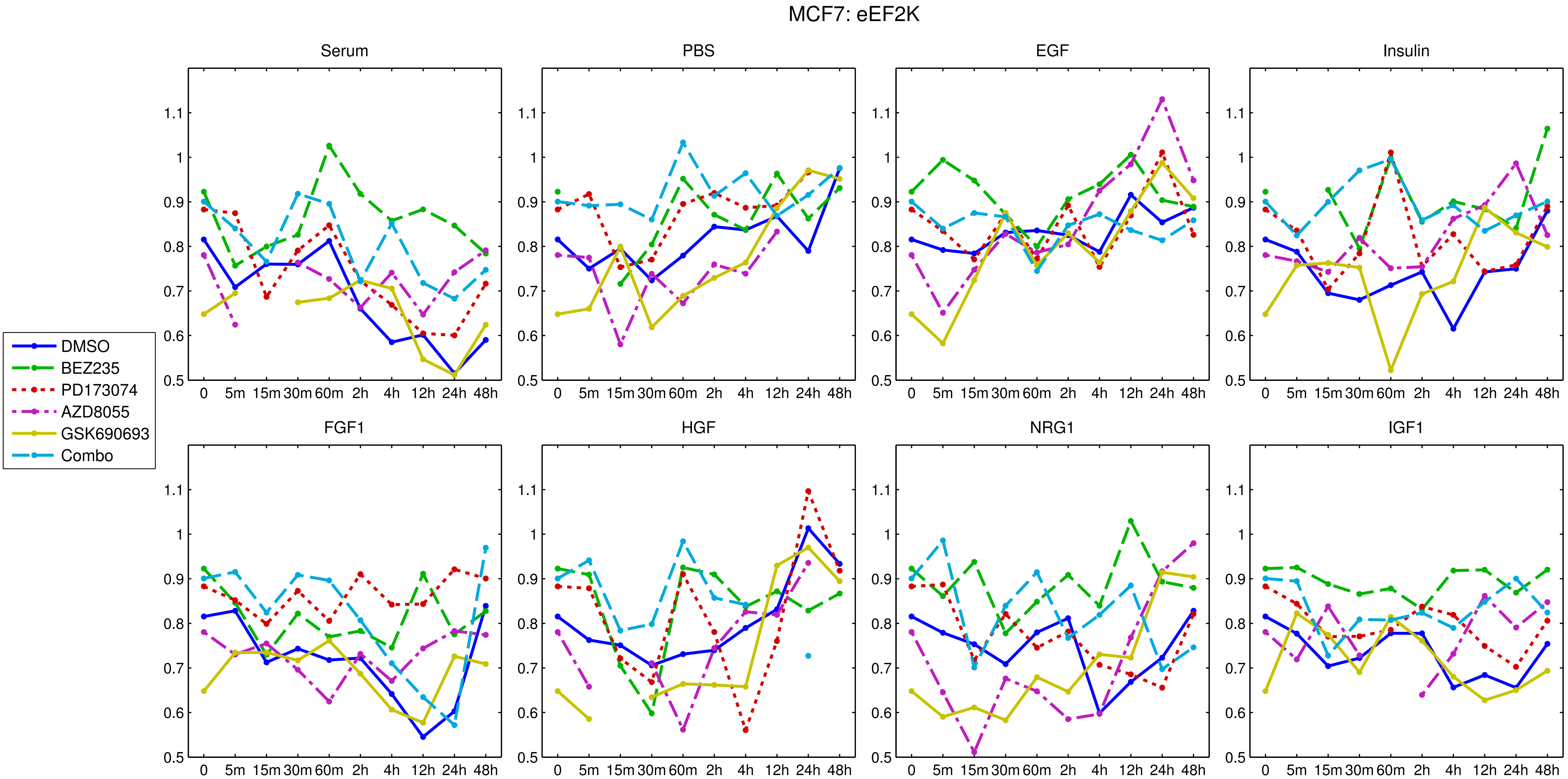
MCF7: eEF2K
Serum
PBS
EGF
Insulin
FGF1
HGF
NRG1
IGF1
DMSO
BEZ235
PD173074
AZD8055
GSK690693
Combo
0.5
0.6
0.7
0.8
0.9
1
1.1
0
5m
15m
30m
60m
2h
4h
12h
24h
48h

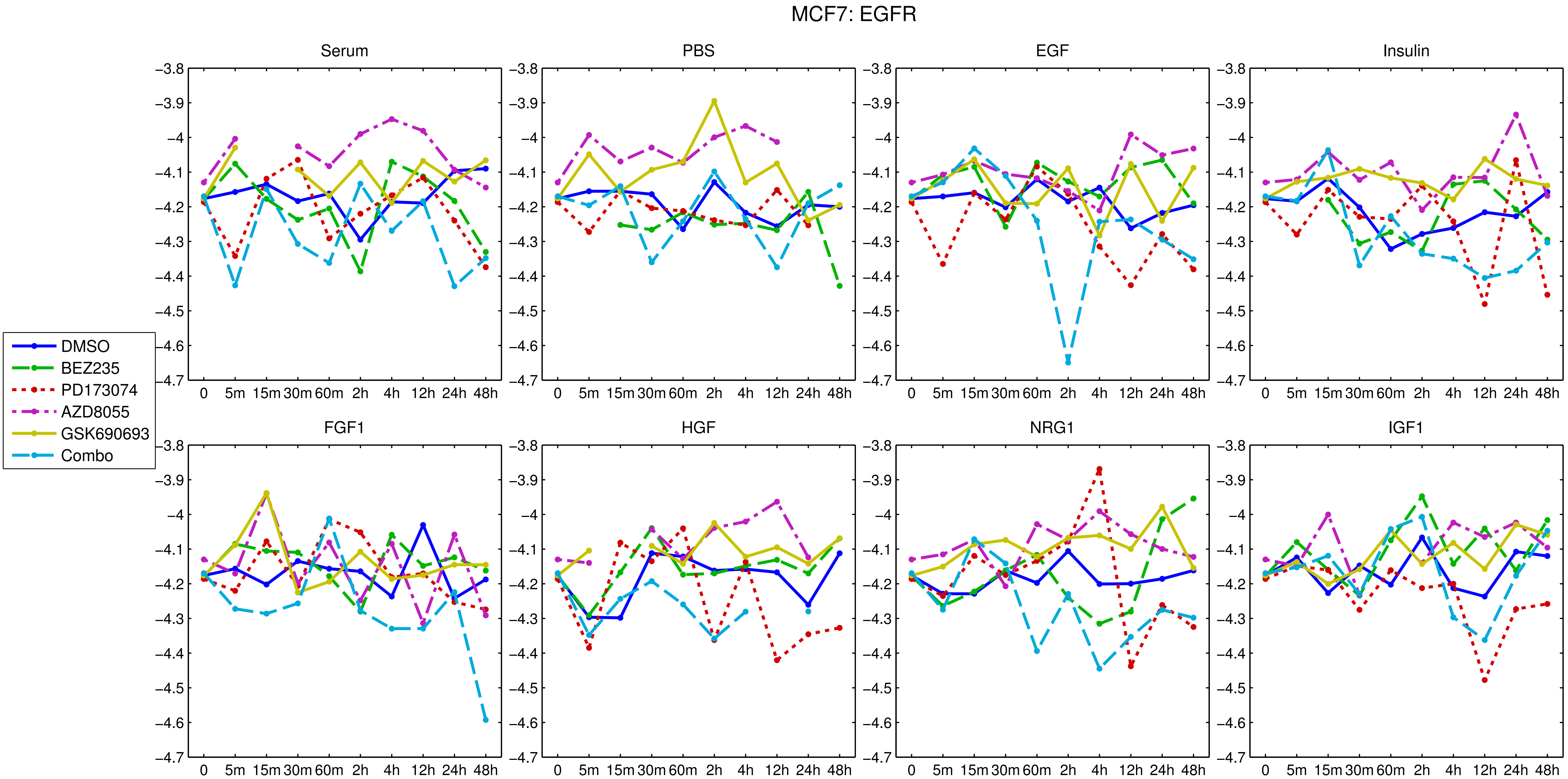

MCF7: EGFR
Serum
PBS
EGF
Insulin
FGF1
HGF
NRG1
IGF1
DMSO
BEZ235
PD173074
AZD8055
GSK690693
Combo
−3.8
−3.9
−4
−4.1
−4.2
−4.3
−4.4
−4.5
−4.6
−4.7
0
5m
15m
30m
60m
2h
4h
12h
24h
48h

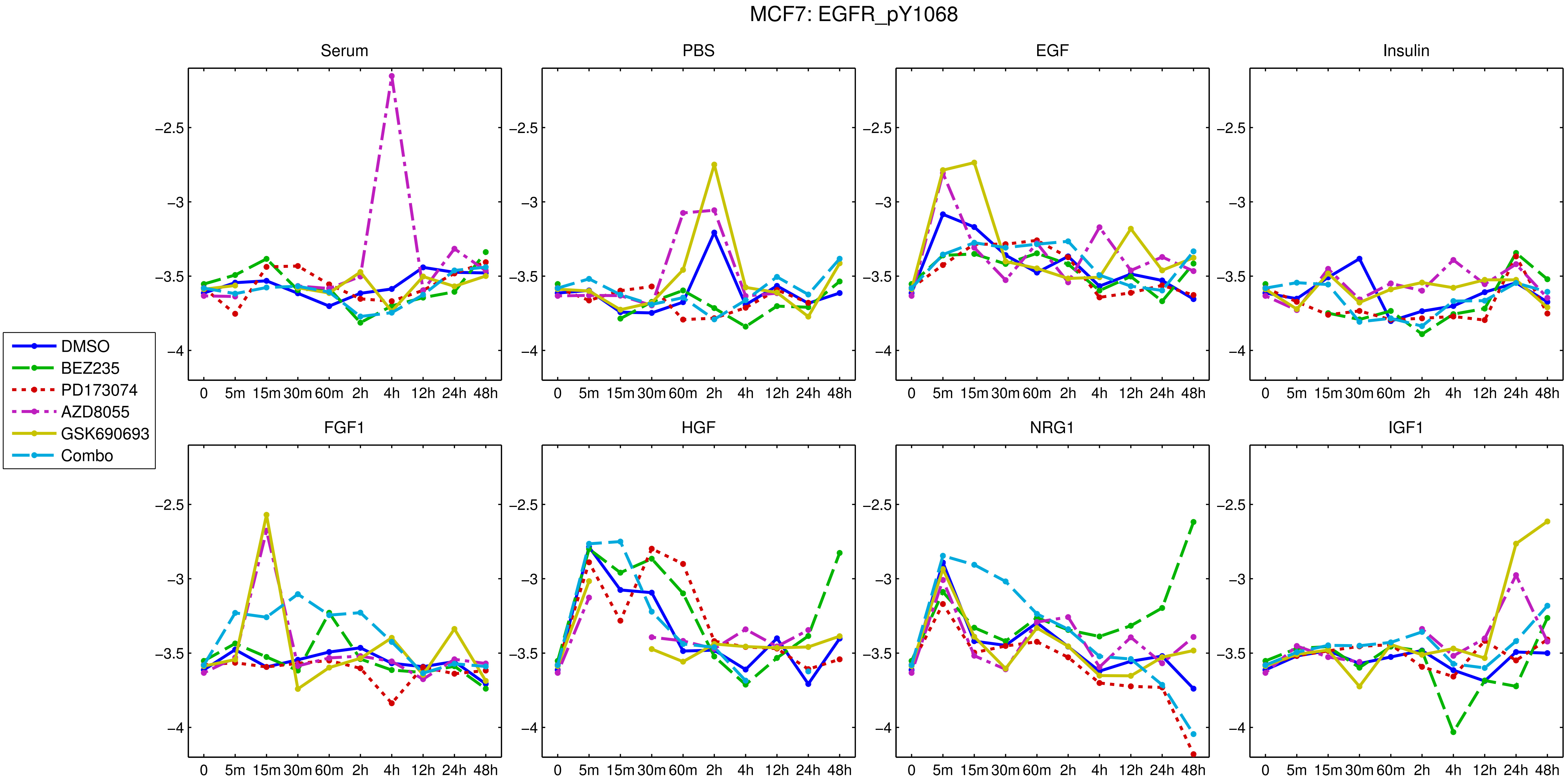

MCF7: EGFR_pY1068
Serum
PBS
EGF
Insulin
FGF1
HGF
NRG1
IGF1
−2.5
−3
−3.5
−4
0
5m
15m
30m
60m
2h
4h
12h
24h
48h
DMSO
BEZ235
PD173074
AZD8055
GSK690693
Combo

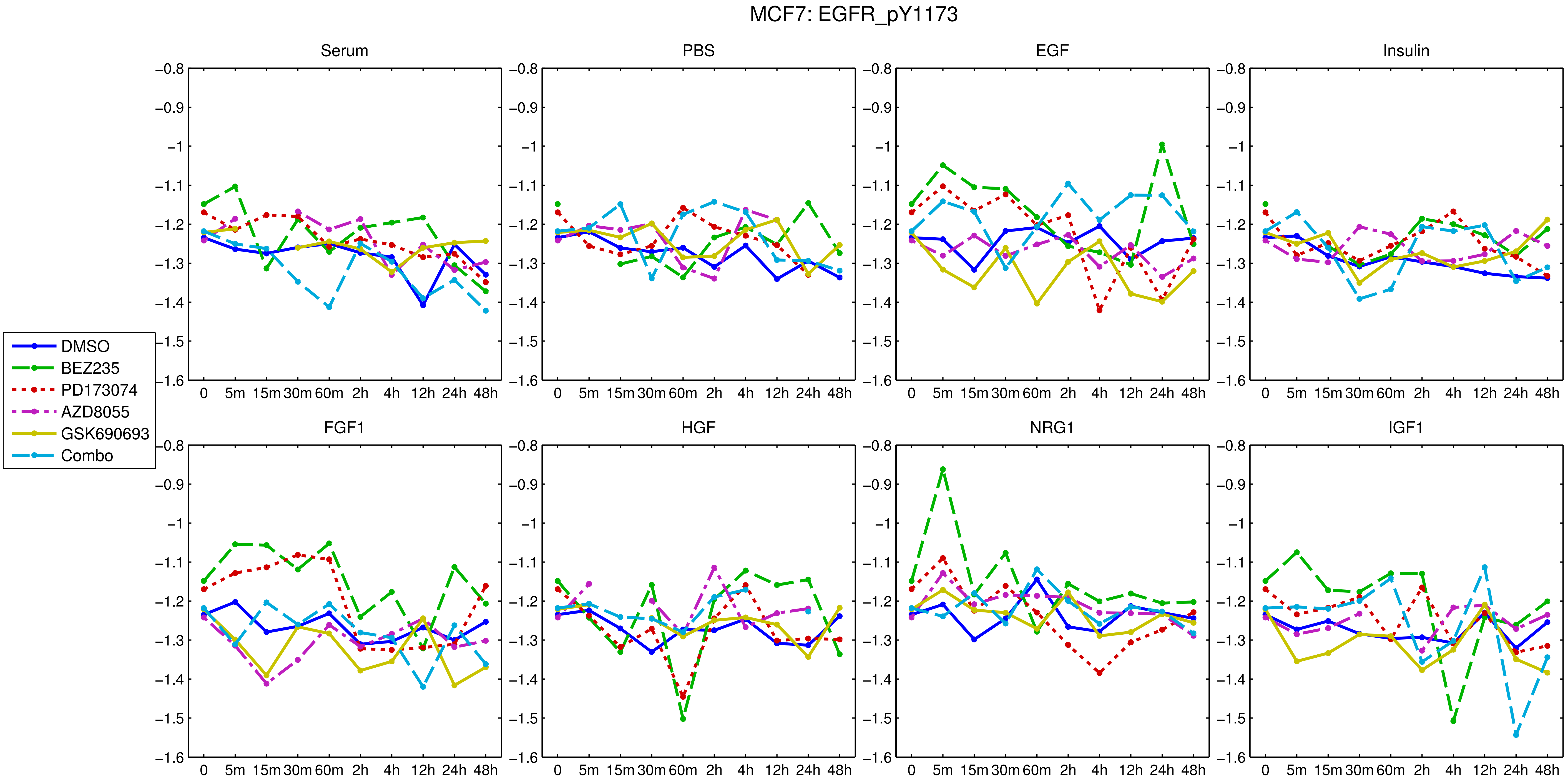

MCF7: EGFR_pY1173
Serum
PBS
EGF
Insulin
FGF1
HGF
NRG1
IGF1
DMSO
BEZ235
PD173074
AZD8055
GSK690693
Combo
−0.8
−0.9
−1
−1.1
−1.2
−1.3
−1.4
−1.5
−1.6
0
5m
15m
30m
60m
2h
4h
12h
24h
48h

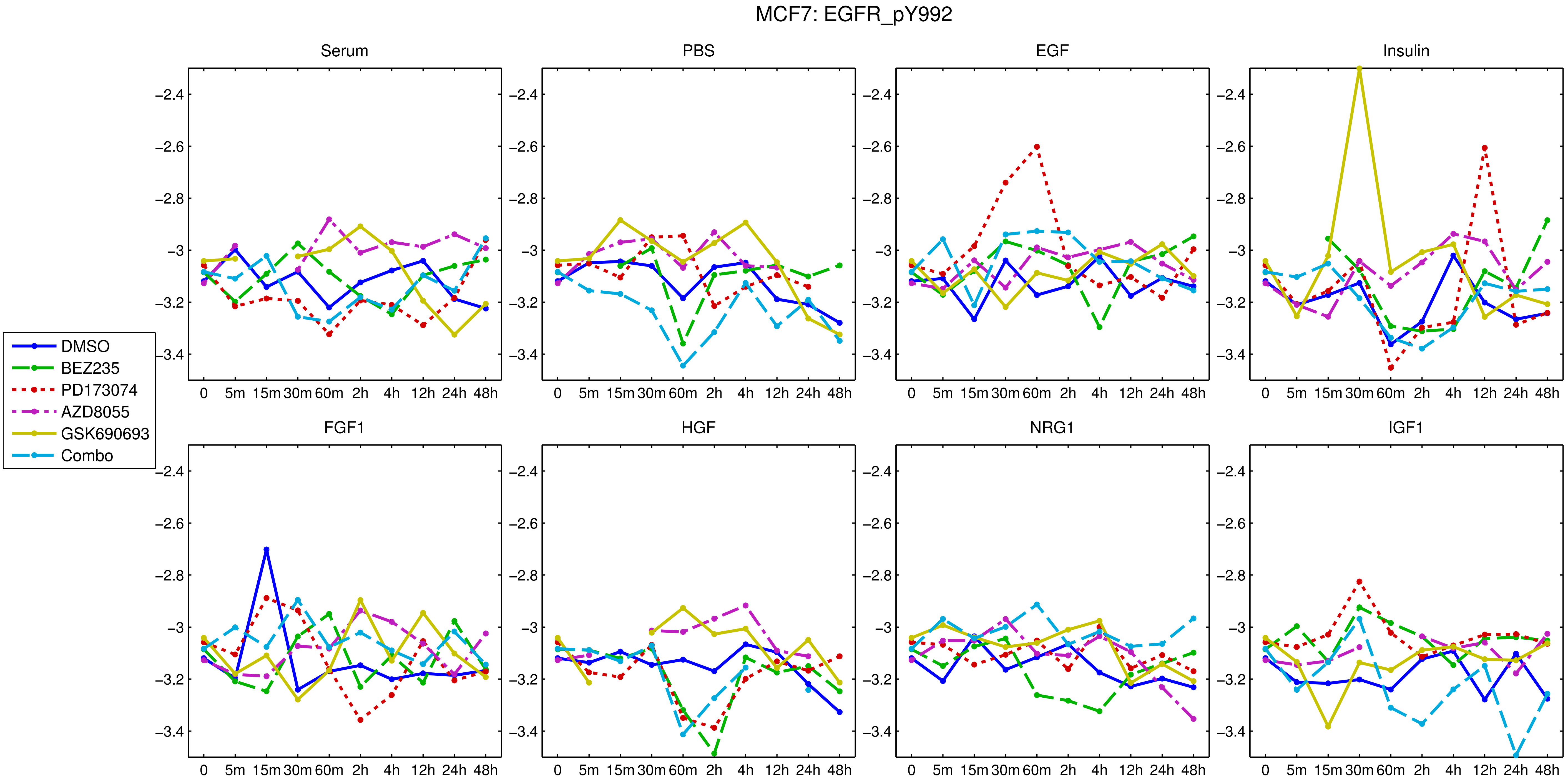

MCF7: EGFR_pY992
Serum
PBS
EGF
Insulin
FGF1
HGF
NRG1
IGF1
−2.4
−2.6
−2.8
−3
−3.2
−3.4
0
5m
15m
30m
60m
2h
4h
12h
24h
48h
DMSO
BEZ235
PD173074
AZD8055
GSK690693
Combo

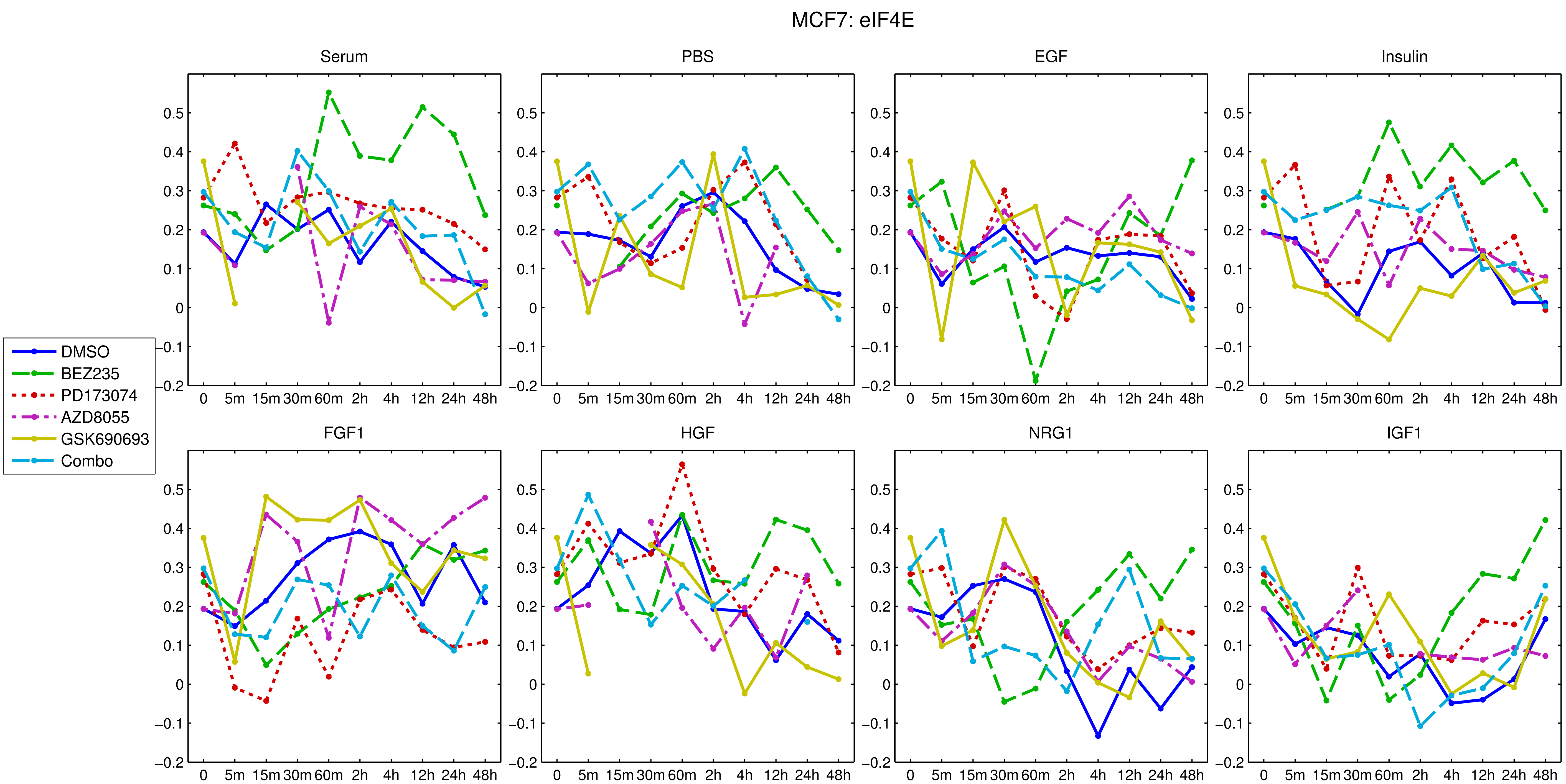
MCF7: eIF4E
Serum
PBS
EGF
Insulin
FGF1
HGF
NRG1
IGF1
DMSO
BEZ235
PD173074
AZD8055
GSK690693
Combo
0.5
0.4
0.3
0.2
0.1
0
−0.1
−0.2
0 5m 15m 30m 60m 2h 4h 12h 24h 48h

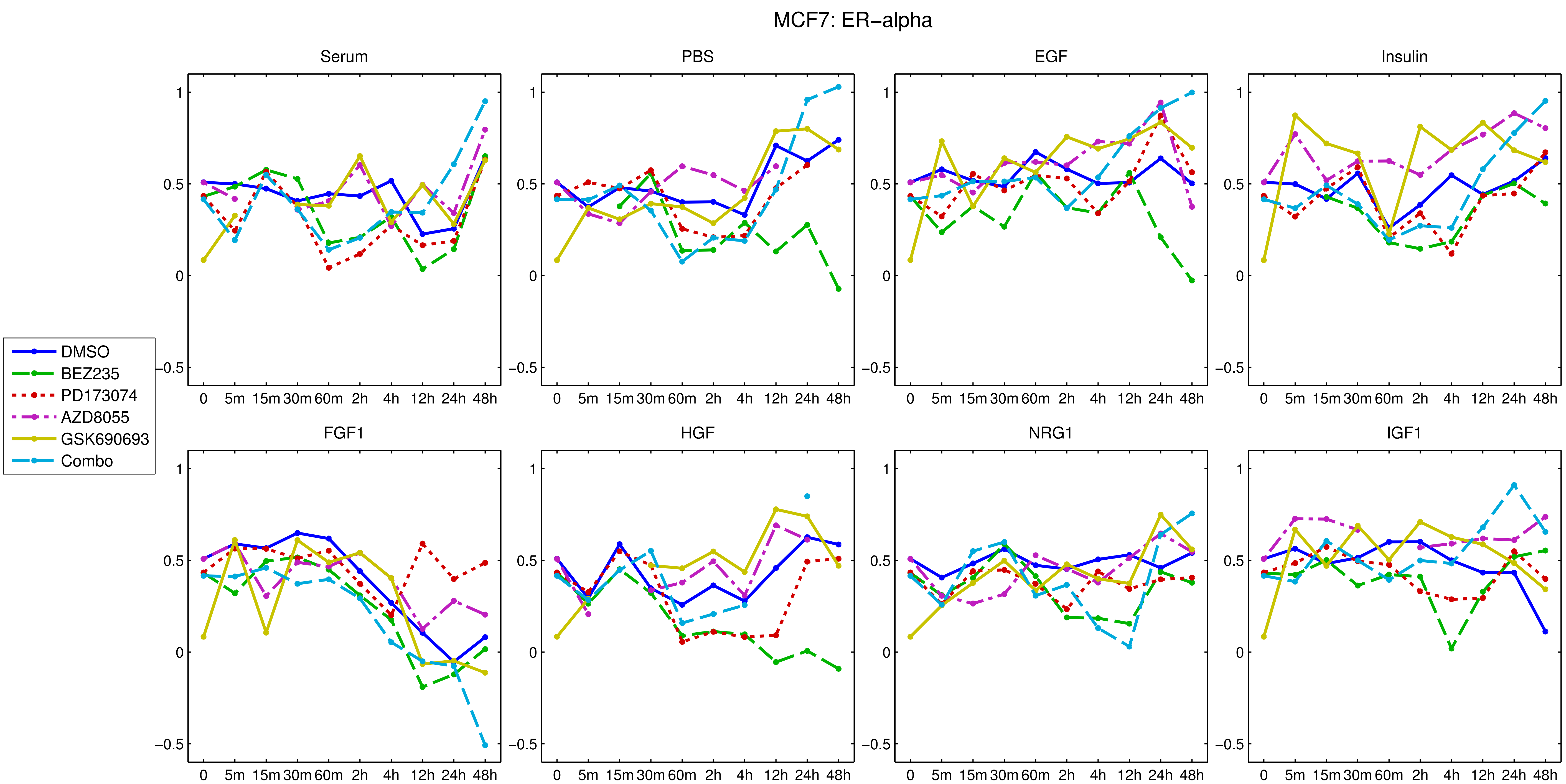
MCF7: ER−alpha
Serum
PBS
EGF
Insulin
FGF1
HGF
NRG1
IGF1
DMSO
BEZ235
PD173074
AZD8055
GSK690693
Combo
1
0.5
0
−0.5
0
5m
15m
30m
60m
2h
4h
12h
24h
48h

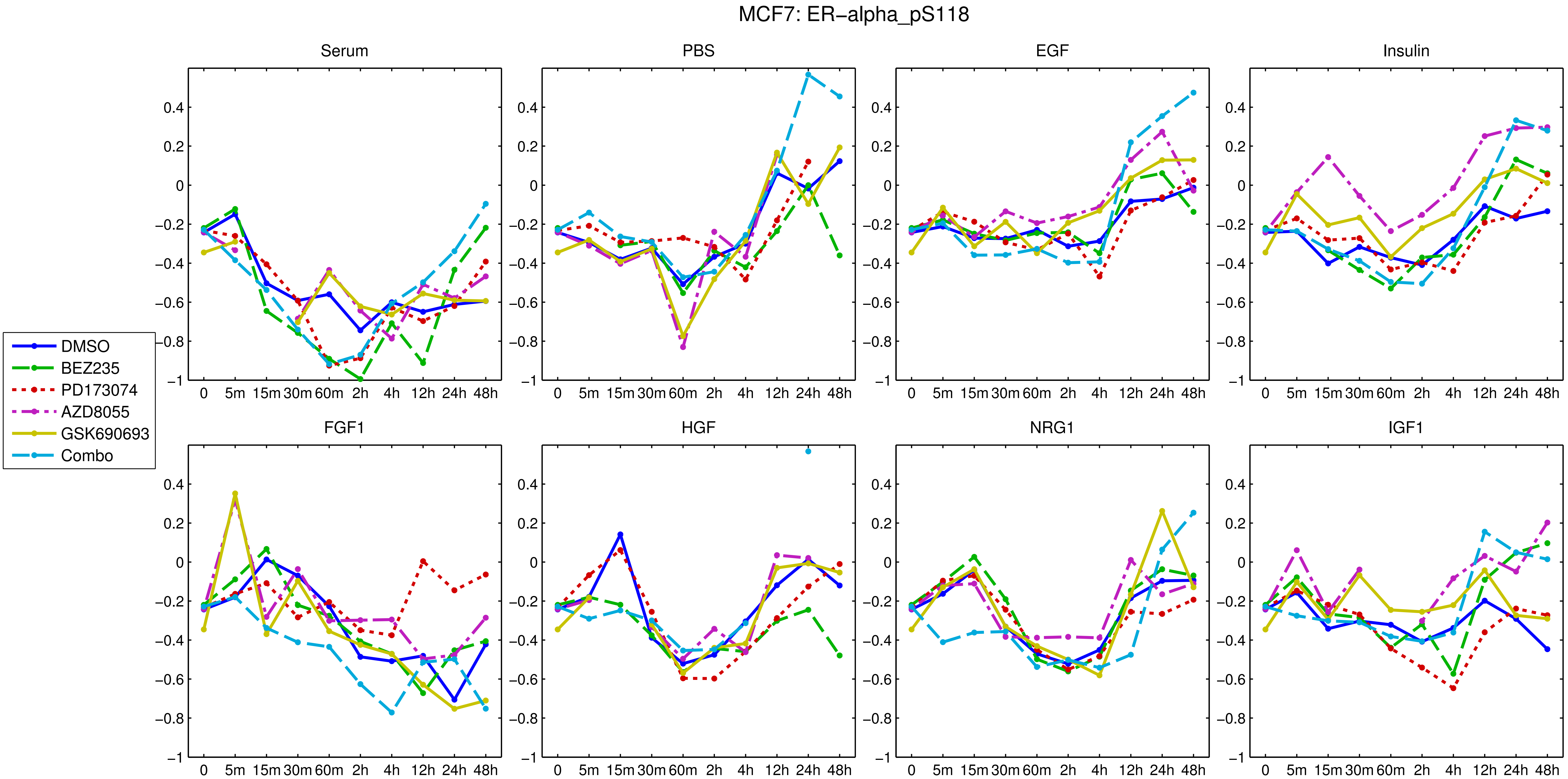
MCF7: ER−alpha_pS118
Serum
PBS
EGF
Insulin
FGF1
HGF
NRG1
IGF1
DMSO
BEZ235
PD173074
AZD8055
GSK690693
Combo
0.4
0.2
0
−0.2
−0.4
−0.6
−0.8
−1
0 5m 15m 30m 60m 2h 4h 12h 24h 48h

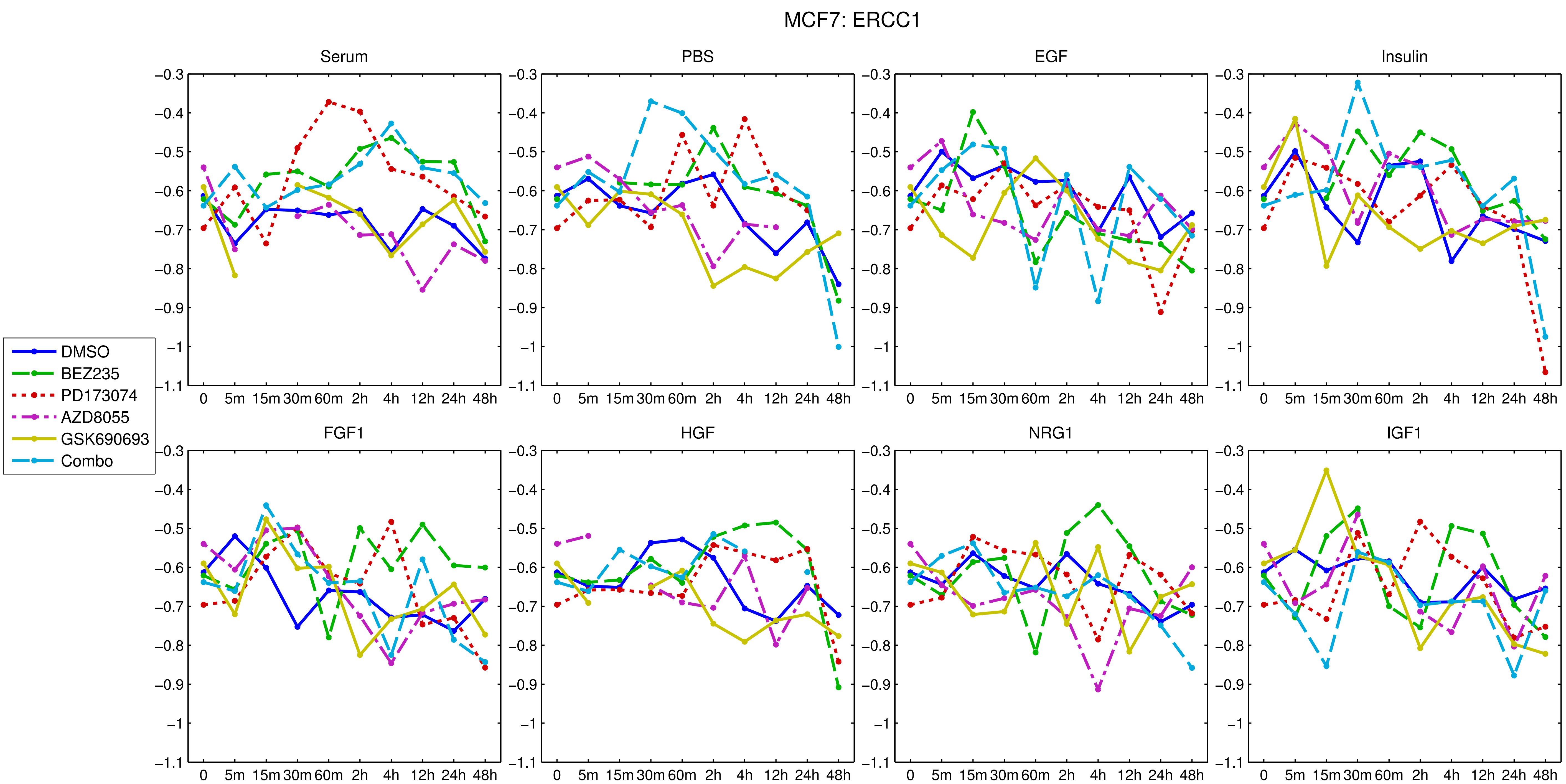
MCF7: ERCC1
Serum
PBS
EGF
Insulin
FGF1
HGF
NRG1
IGF1
DMSO
BEZ235
PD173074
AZD8055
GSK690693
Combo
−0.3
−0.4
−0.5
−0.6
−0.7
−0.8
−0.9
−1
−1.1
0
5m
15m
30m
60m
2h
4h
12h
24h
48h

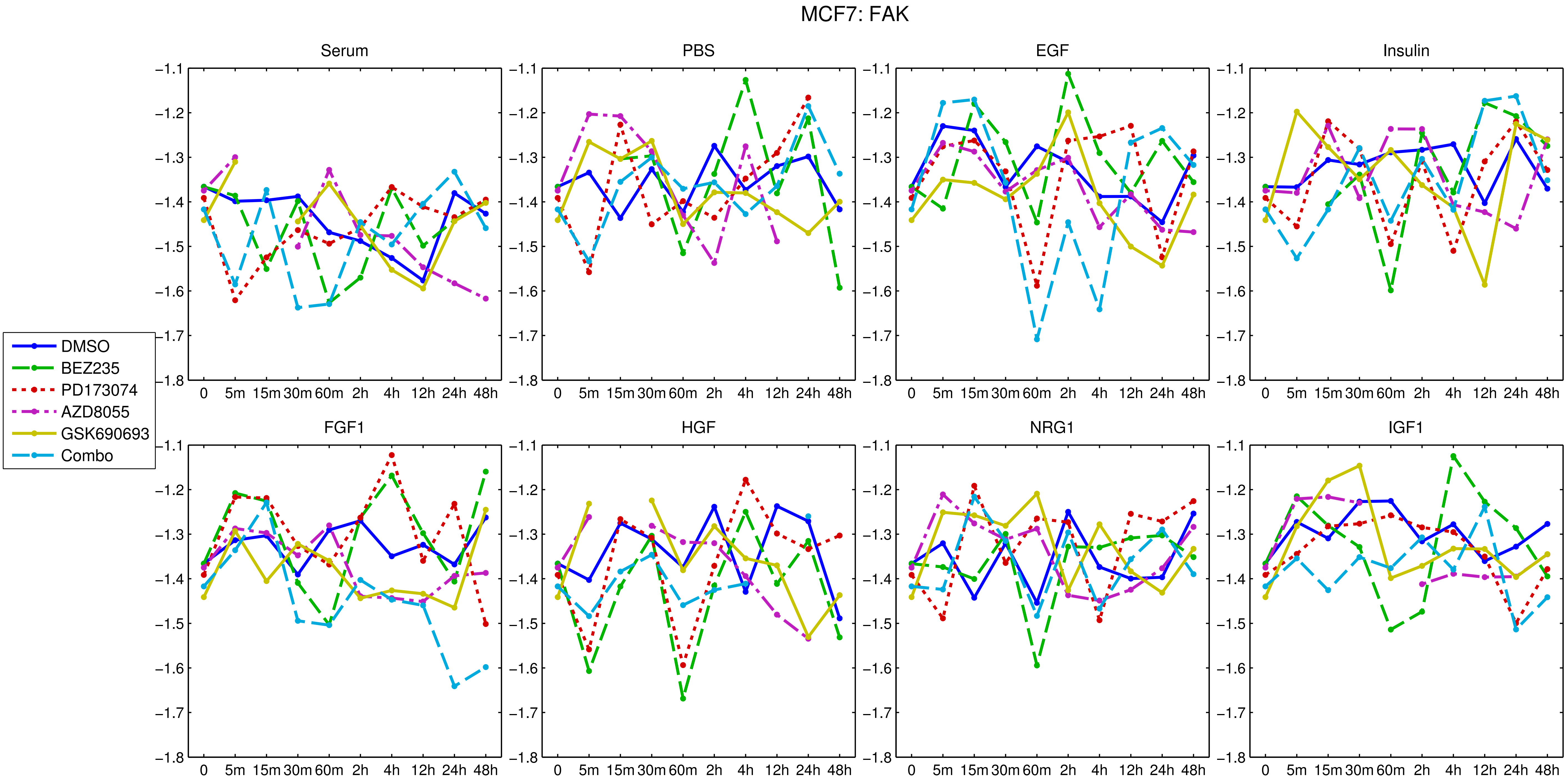
MCF7: FAK
Serum
PBS
EGF
Insulin
FGF1
HGF
NRG1
IGF1
DMSO
BEZ235
PD173074
AZD8055
GSK690693
Combo
−1.1
−1.2
−1.3
−1.4
−1.5
−1.6
−1.7
−1.8
0
5m
15m
30m
60m
2h
4h
12h
24h
48h

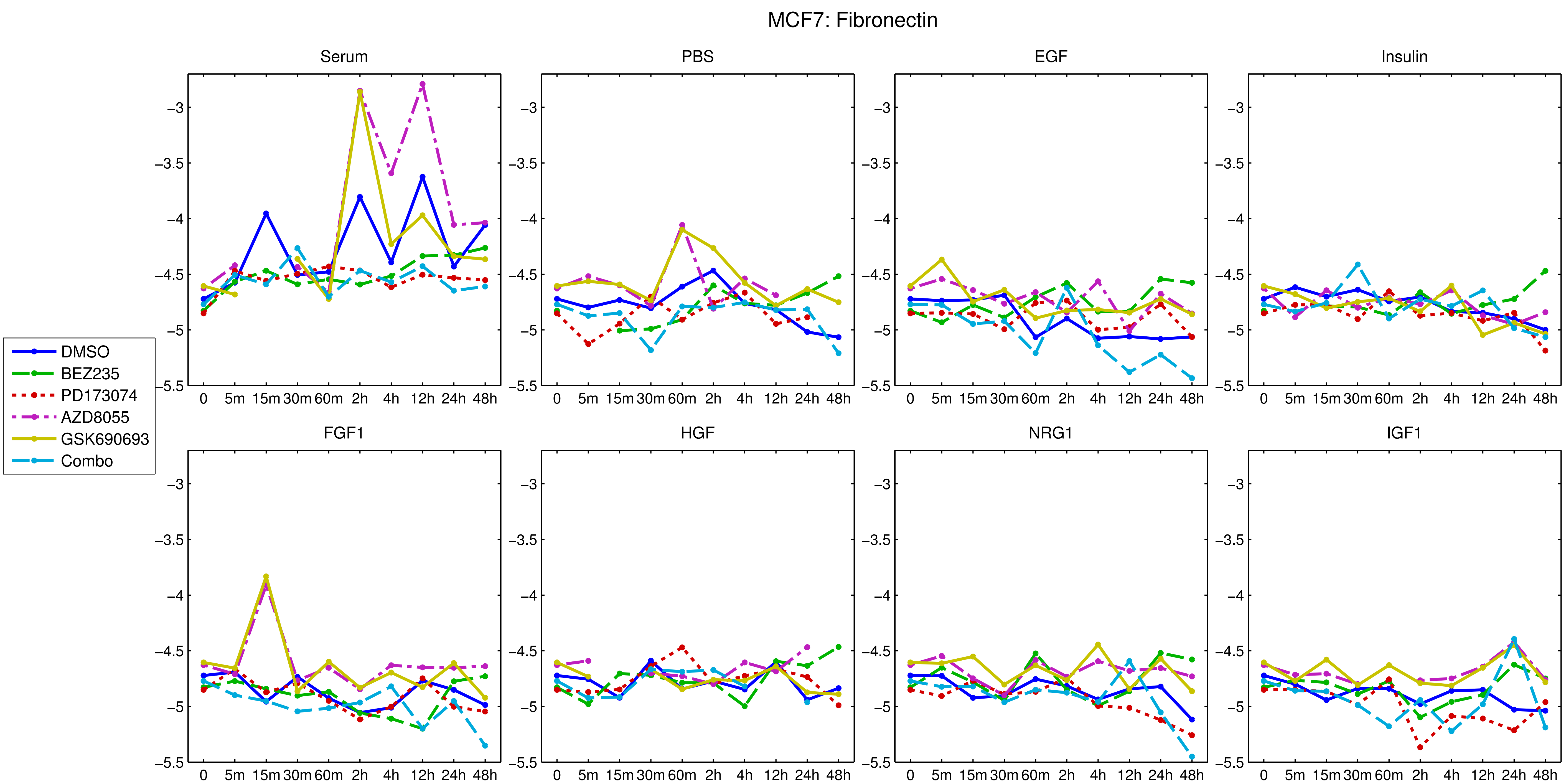

MCF7: Fibronectin
Serum
PBS
EGF
Insulin
FGF1
HGF
NRG1
IGF1
−3
−3.5
−4
−4.5
−5
−5.5
0
5m
15m
30m
60m
2h
4h
12h
24h
48h
DMSO
BEZ235
PD173074
AZD8055
GSK690693
Combo

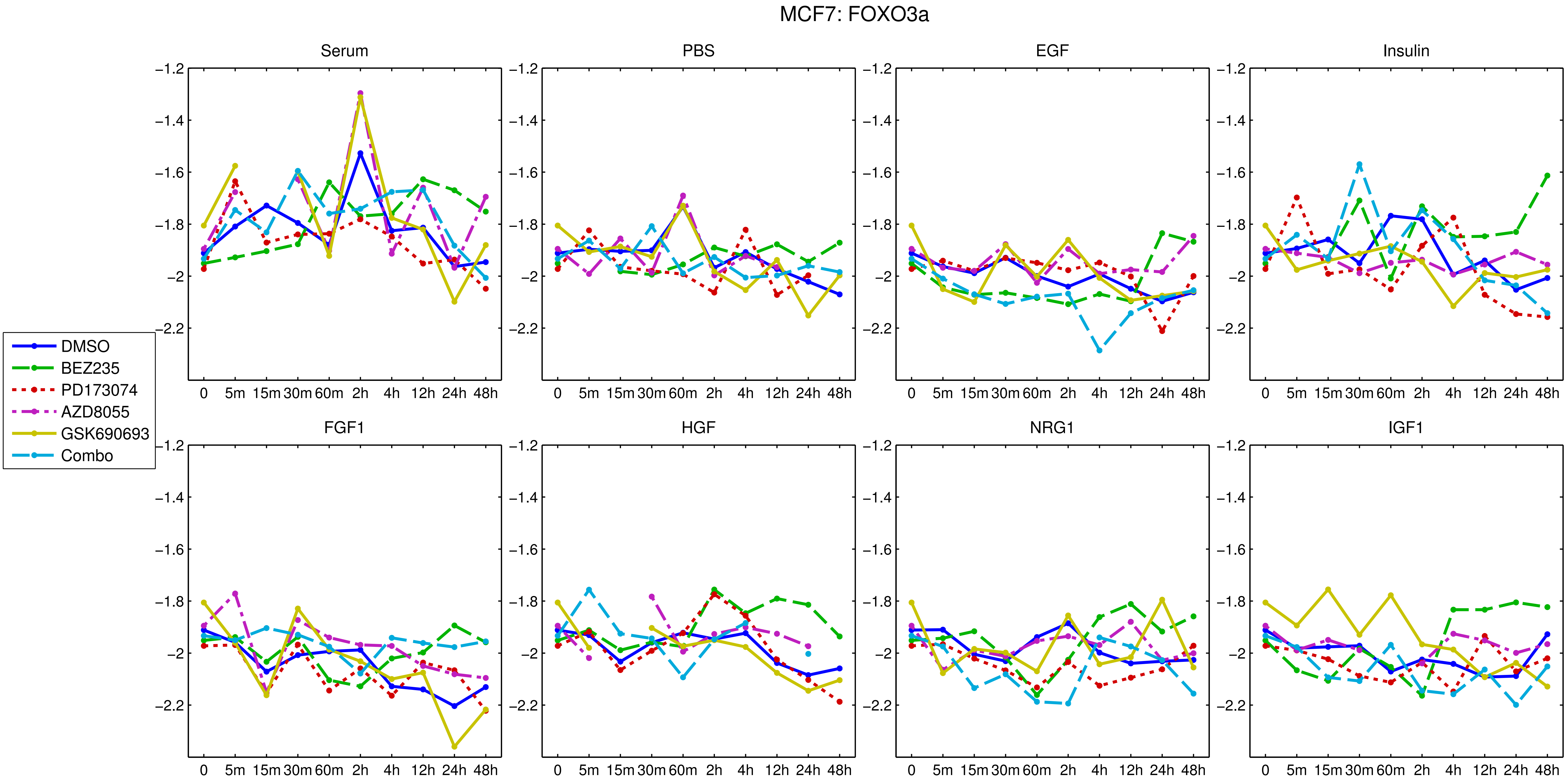
MCF7: FOXO3a
Serum
PBS
EGF
Insulin
FGF1
HGF
NRG1
IGF1
DMSO
BEZ235
PD173074
AZD8055
GSK690693
Combo
−1.2
−1.4
−1.6
−1.8
−2
−2.2
0
5m
15m
30m
60m
2h
4h
12h
24h
48h

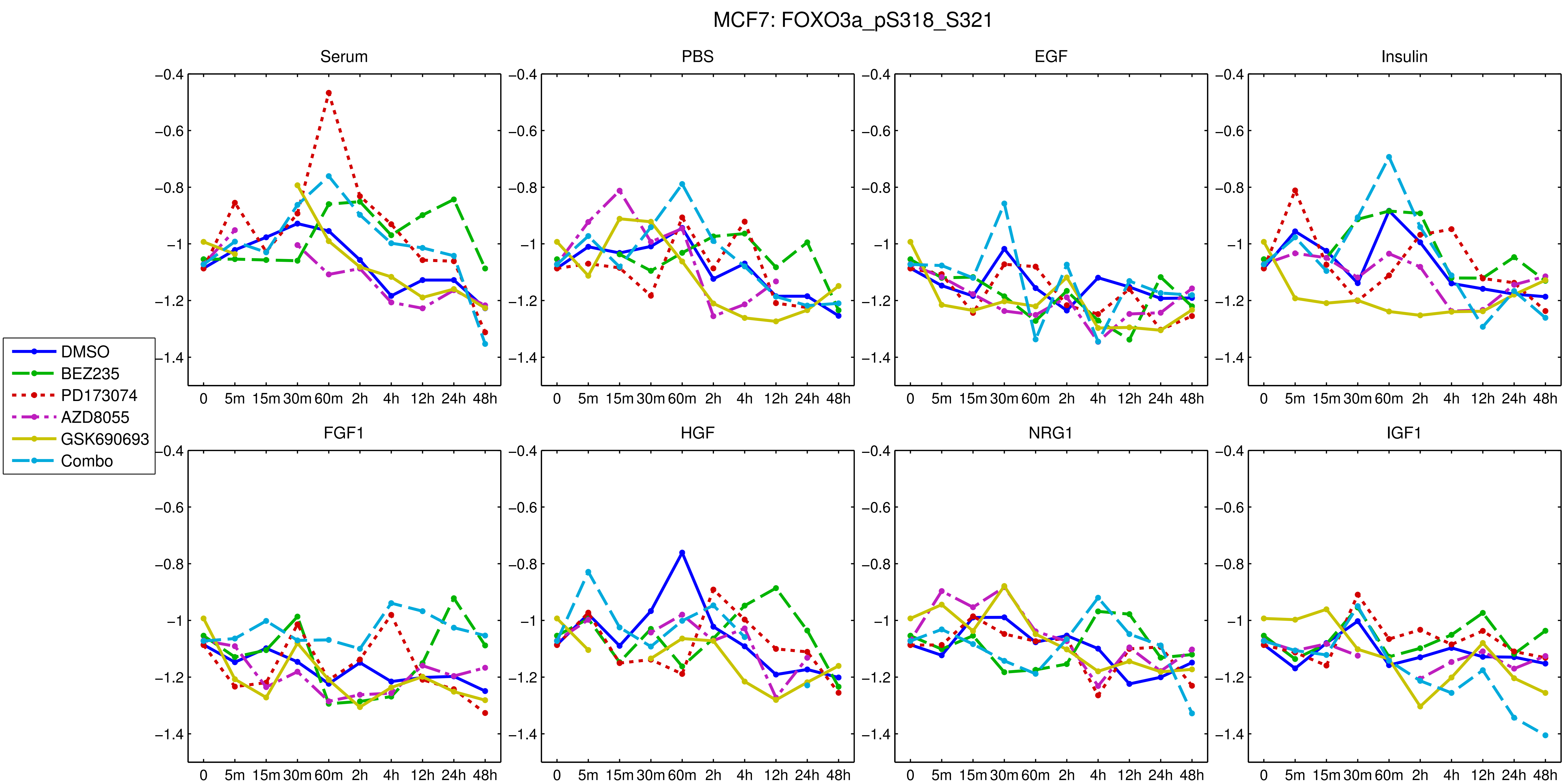
MCF7: FOXO3a_pS318_S321
Serum
PBS
EGF
Insulin
FGF1
HGF
NRG1
IGF1
DMSO
BEZ235
PD173074
AZD8055
GSK690693
Combo
−0.4
−0.6
−0.8
−1
−1.2
−1.4
0
5m
15m
30m
60m
2h
4h
12h
24h
48h

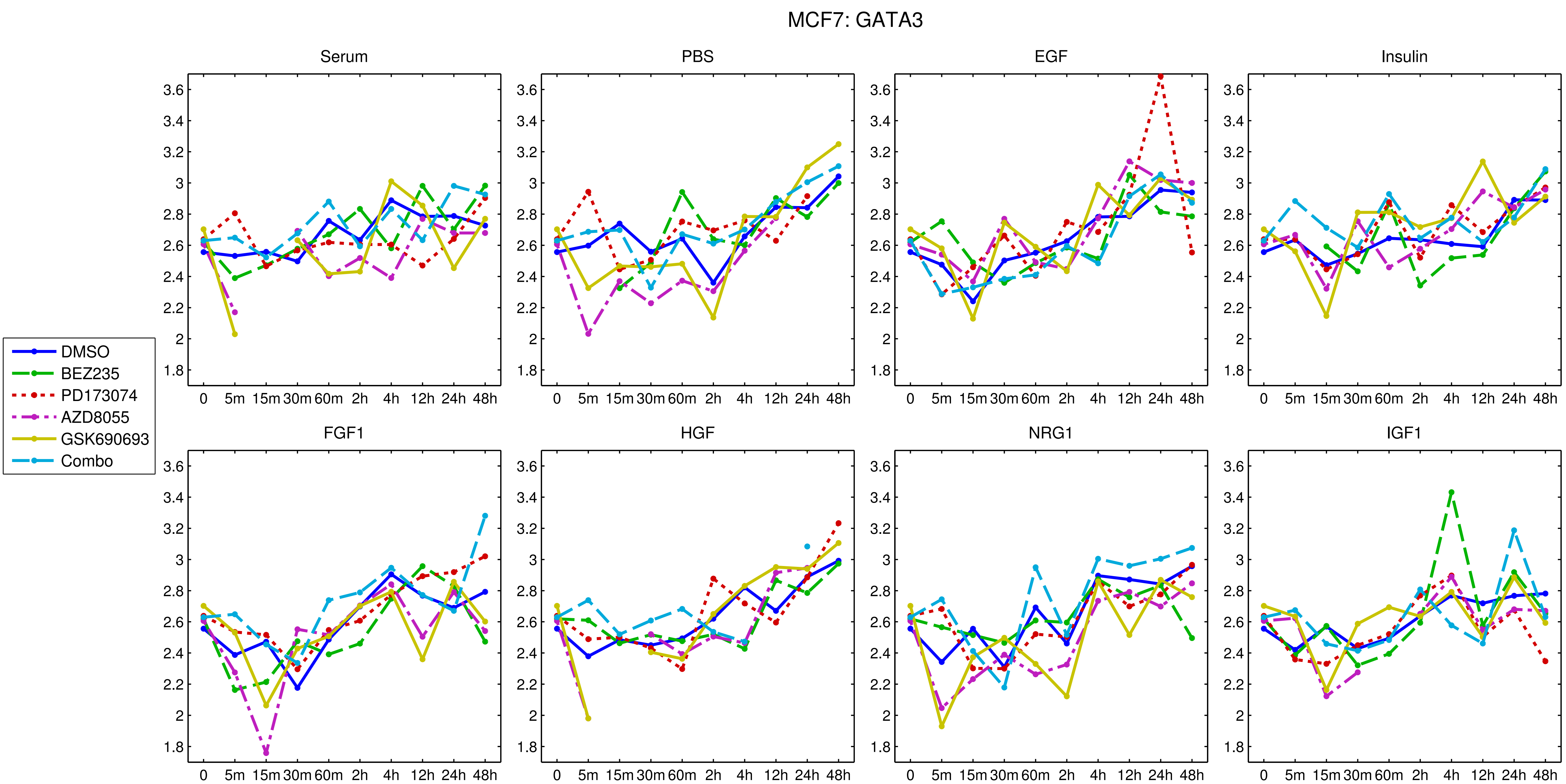
MCF7: GATA3
Serum
PBS
EGF
Insulin
FGF1
HGF
NRG1
IGF1
DMSO
BEZ235
PD173074
AZD8055
GSK690693
Combo
0
5m
15m
30m
60m
2h
4h
12h
24h
48h
1.8
2
2.2
2.4
2.6
2.8
3
3.2
3.4
3.6

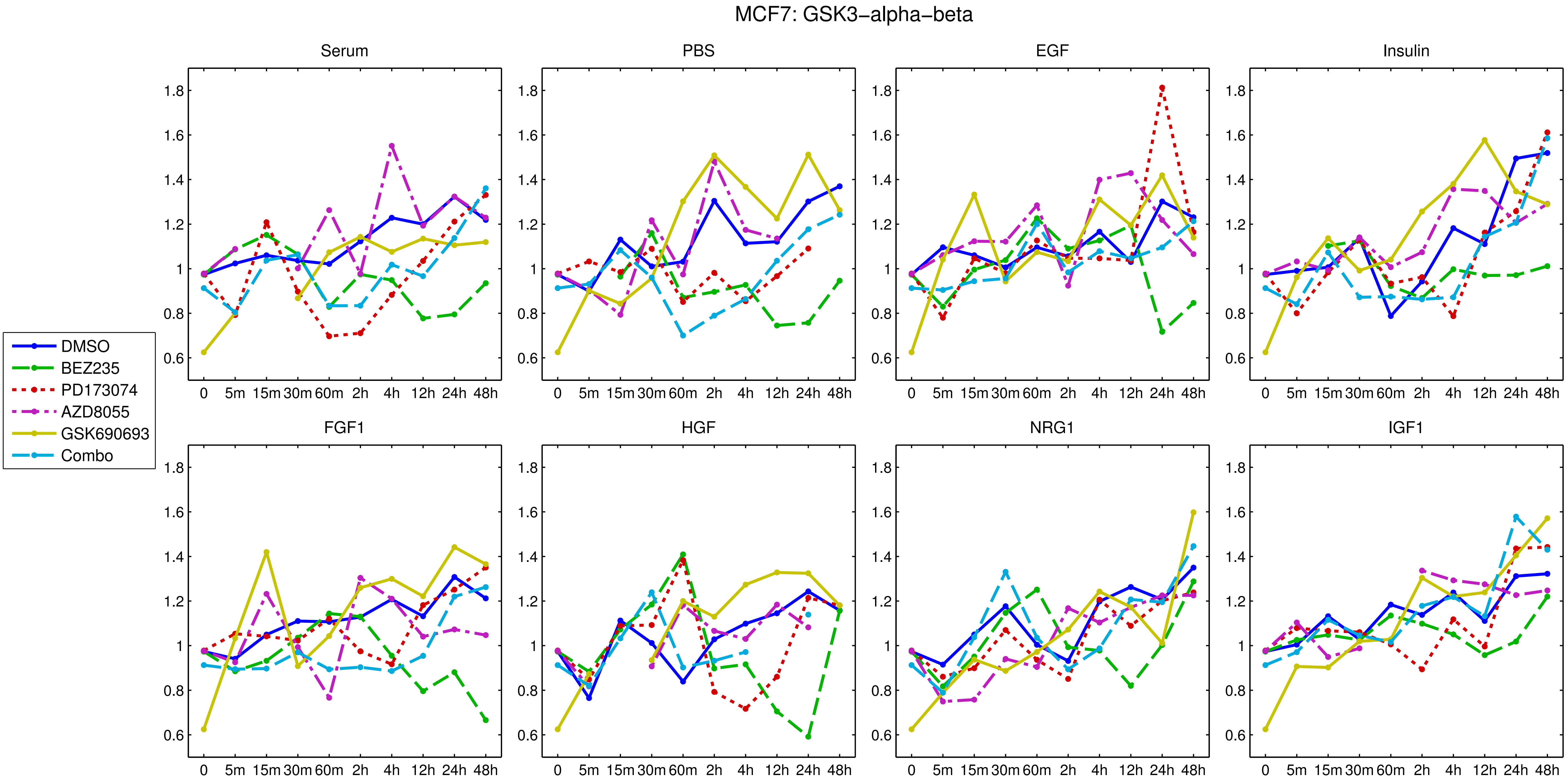
MCF7: GSK3−alpha−beta
Serum
PBS
EGF
Insulin
FGF1
HGF
NRG1
IGF1
DMSO
BEZ235
PD173074
AZD8055
GSK690693
Combo
0.6
0.8
1
1.2
1.4
1.6
1.8
0
5m
15m
30m
60m
2h
4h
12h
24h
48h

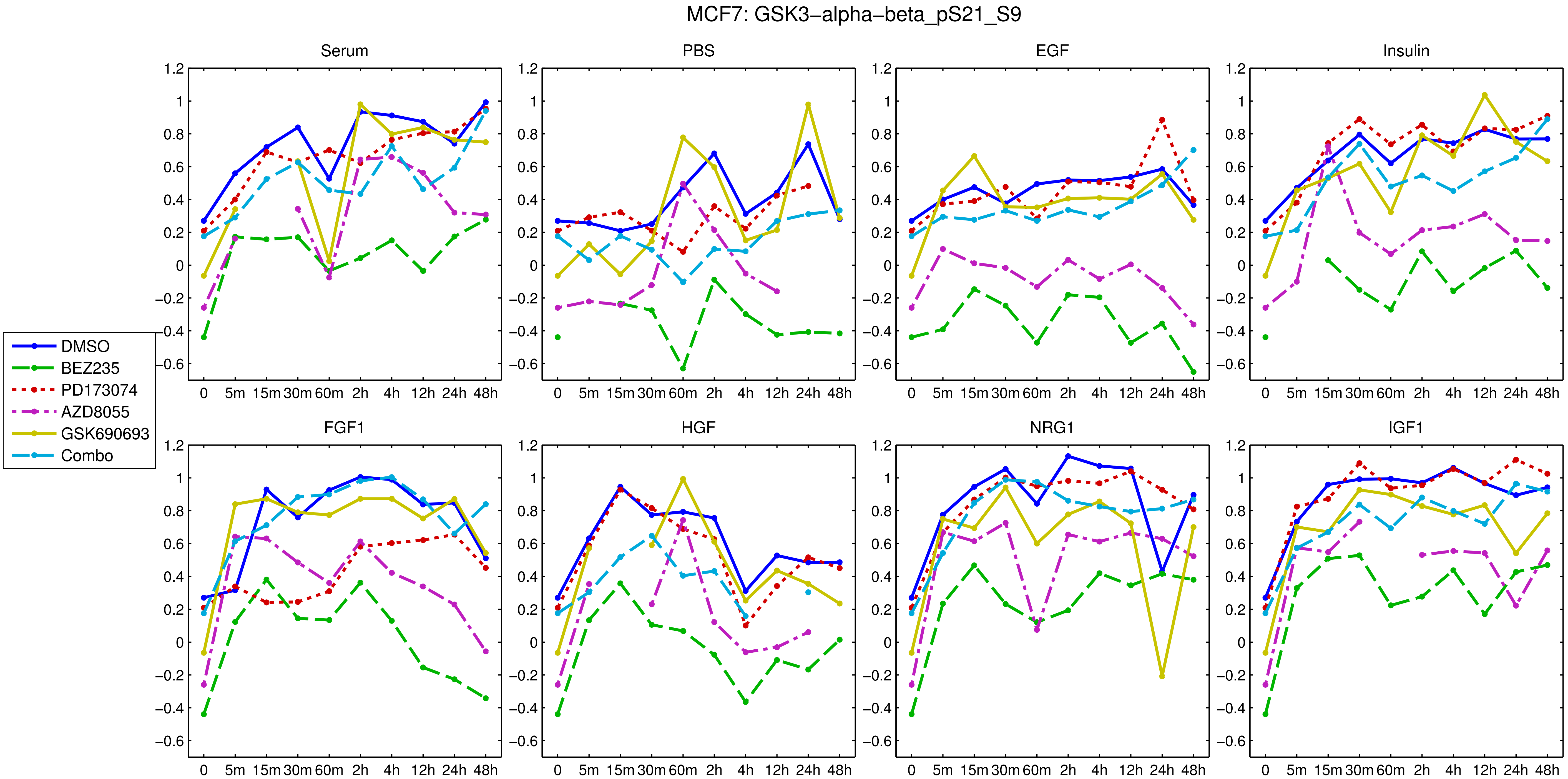
MCF7: GSK3−alpha−beta_pS21_S9
Serum
PBS
EGF
Insulin
FGF1
HGF
NRG1
IGF1
DMSO
BEZ235
PD173074
AZD8055
GSK690693
Combo
0
5m
15m
30m
60m
2h
4h
12h
24h
48h
1.2
1
0.8
0.6
0.4
0.2
0
−0.2
−0.4
−0.6

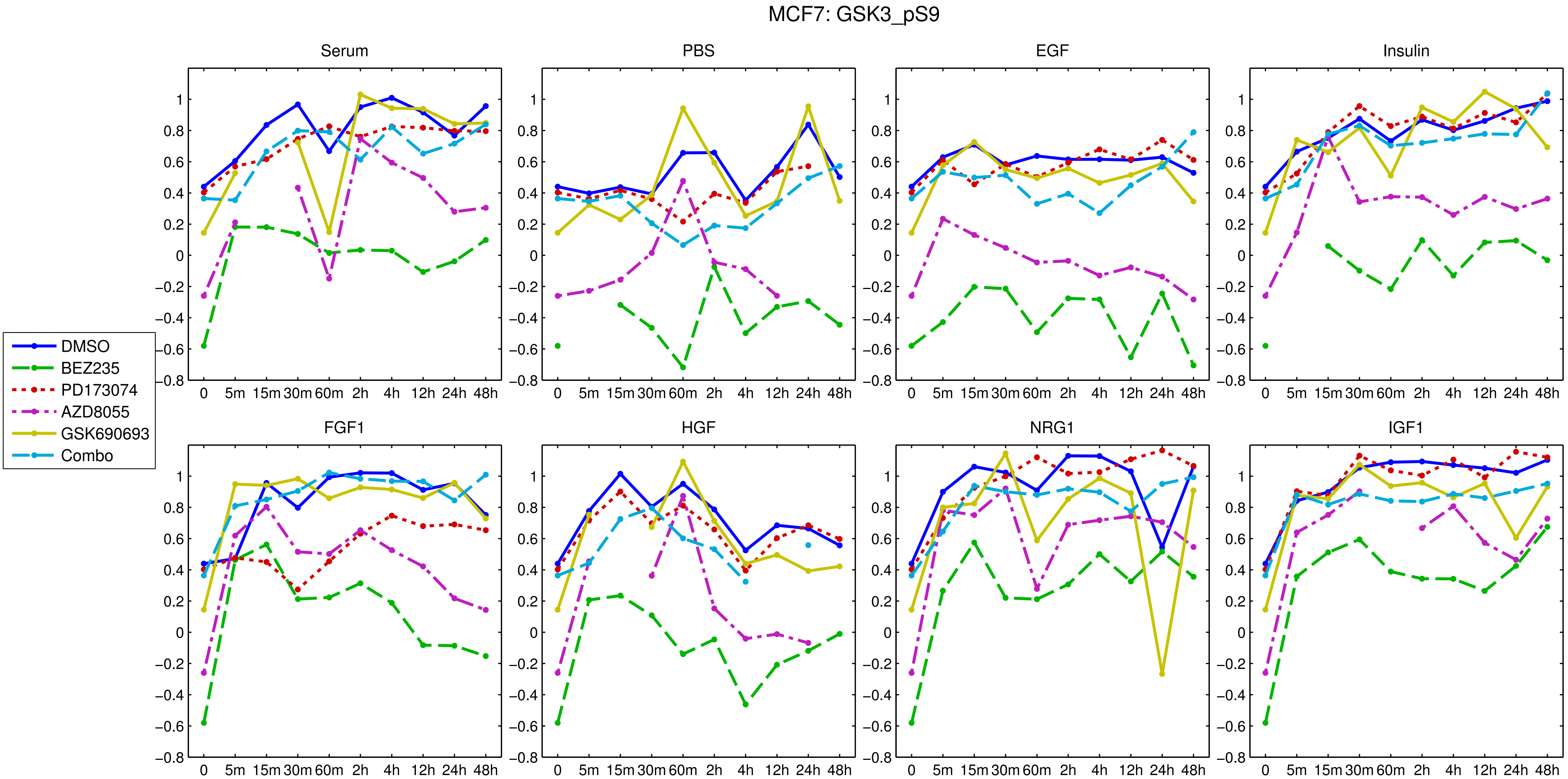
MCF7: GSK3_pS9
Serum
PBS
EGF
Insulin
FGF1
HGF
NRG1
IGF1
DMSO
BEZ235
PD173074
AZD8055
GSK690693
Combo
0
5m
15m
30m
60m
2h
4h
12h
24h
48h
1
0.8
0.6
0.4
0.2
0
−0.2
−0.4
−0.6
−0.8

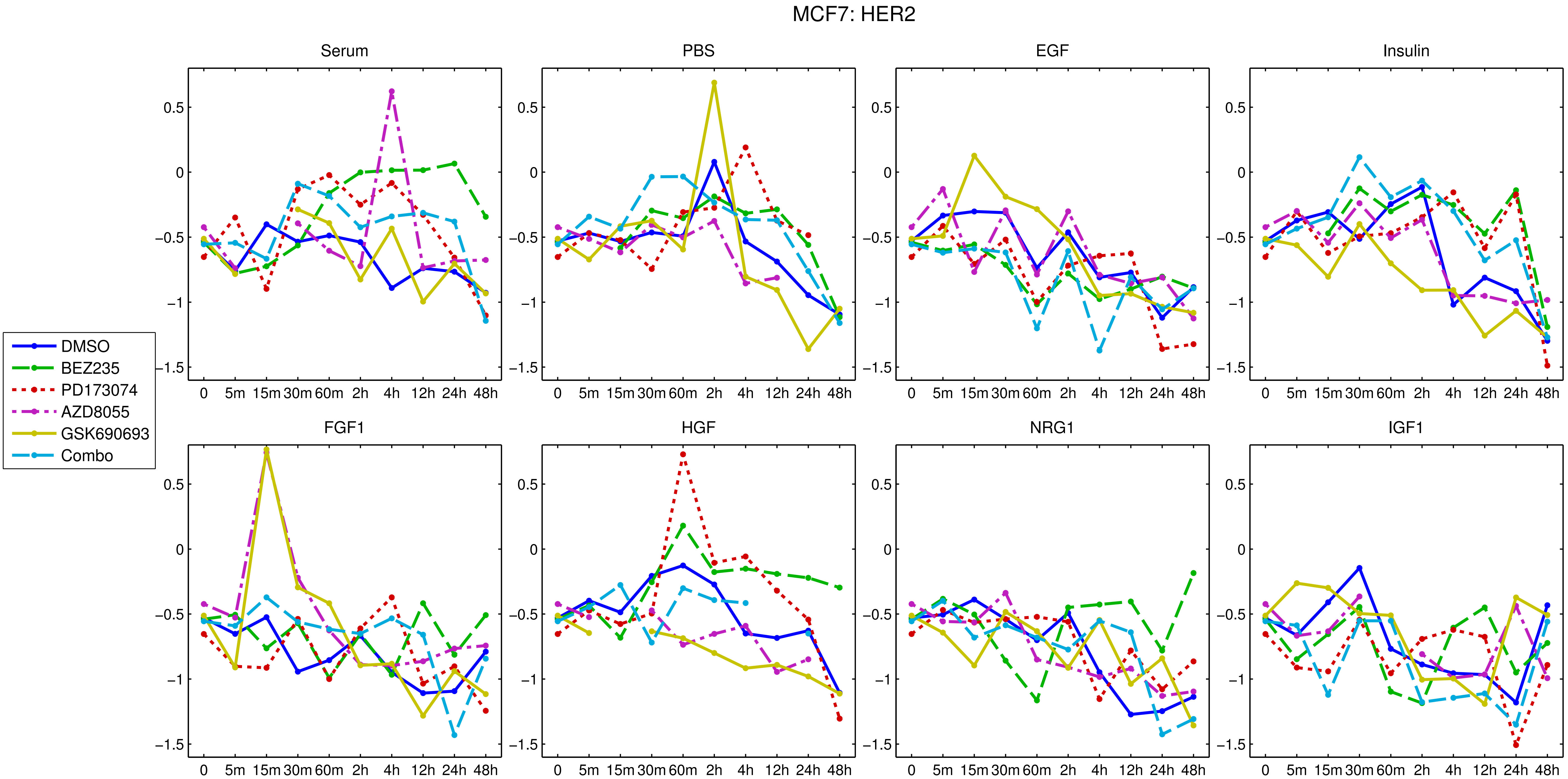
MCF7: HER2
Serum
PBS
EGF
Insulin
FGF1
HGF
NRG1
IGF1
0.5
0
−0.5
−1
−1.5
0
5m
15m
30m
60m
2h
4h
12h
24h
48h
DMSO
BEZ235
PD173074
AZD8055
GSK690693
Combo

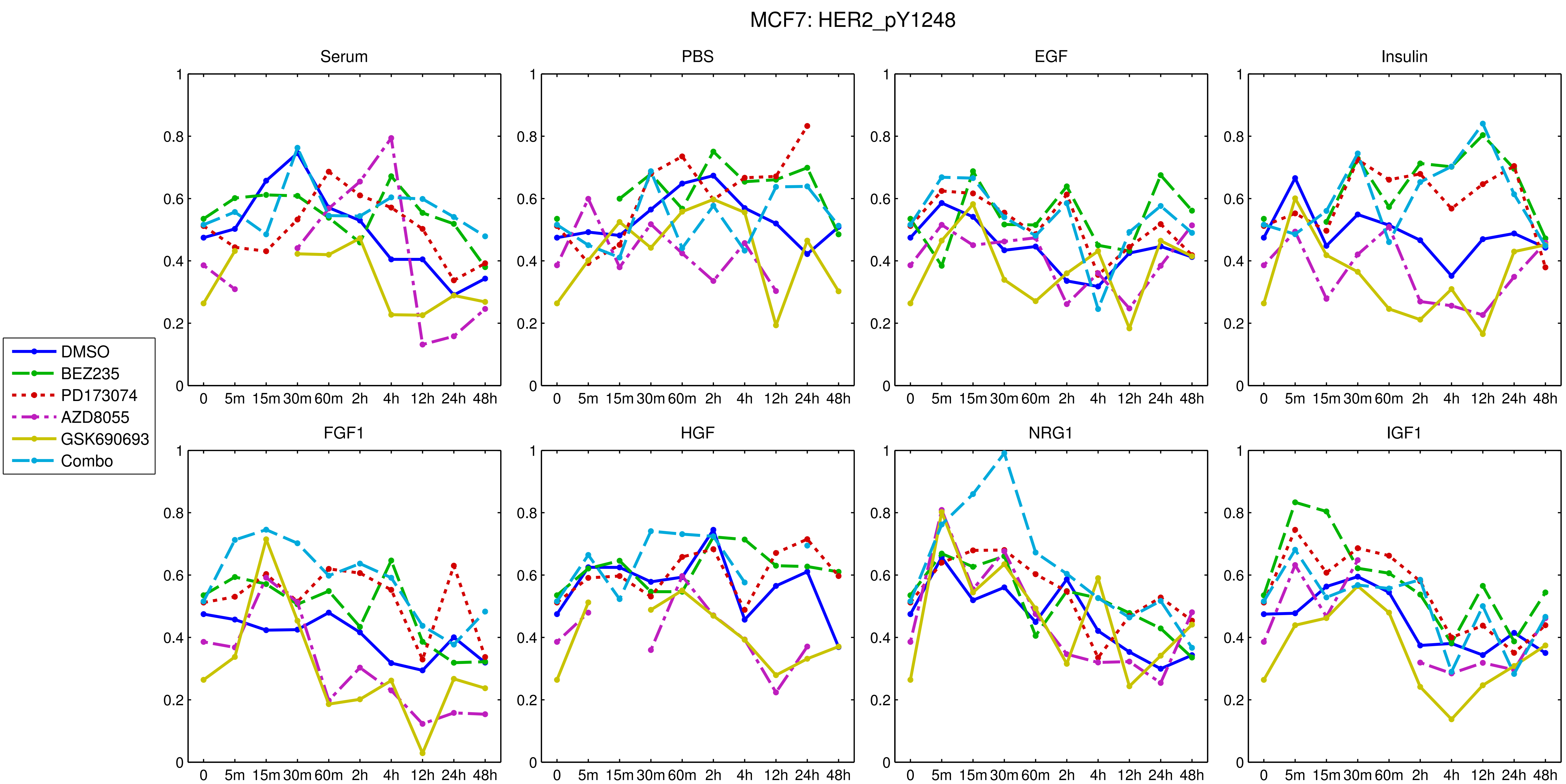
MCF7: HER2_pY1248
Serum
PBS
EGF
Insulin
FGF1
HGF
NRG1
IGF1
DMSO
BEZ235
PD173074
AZD8055
GSK690693
Combo
0
0.2
0.4
0.6
0.8
1
0 5m 15m 30m 60m 2h 4h 12h 24h 48h

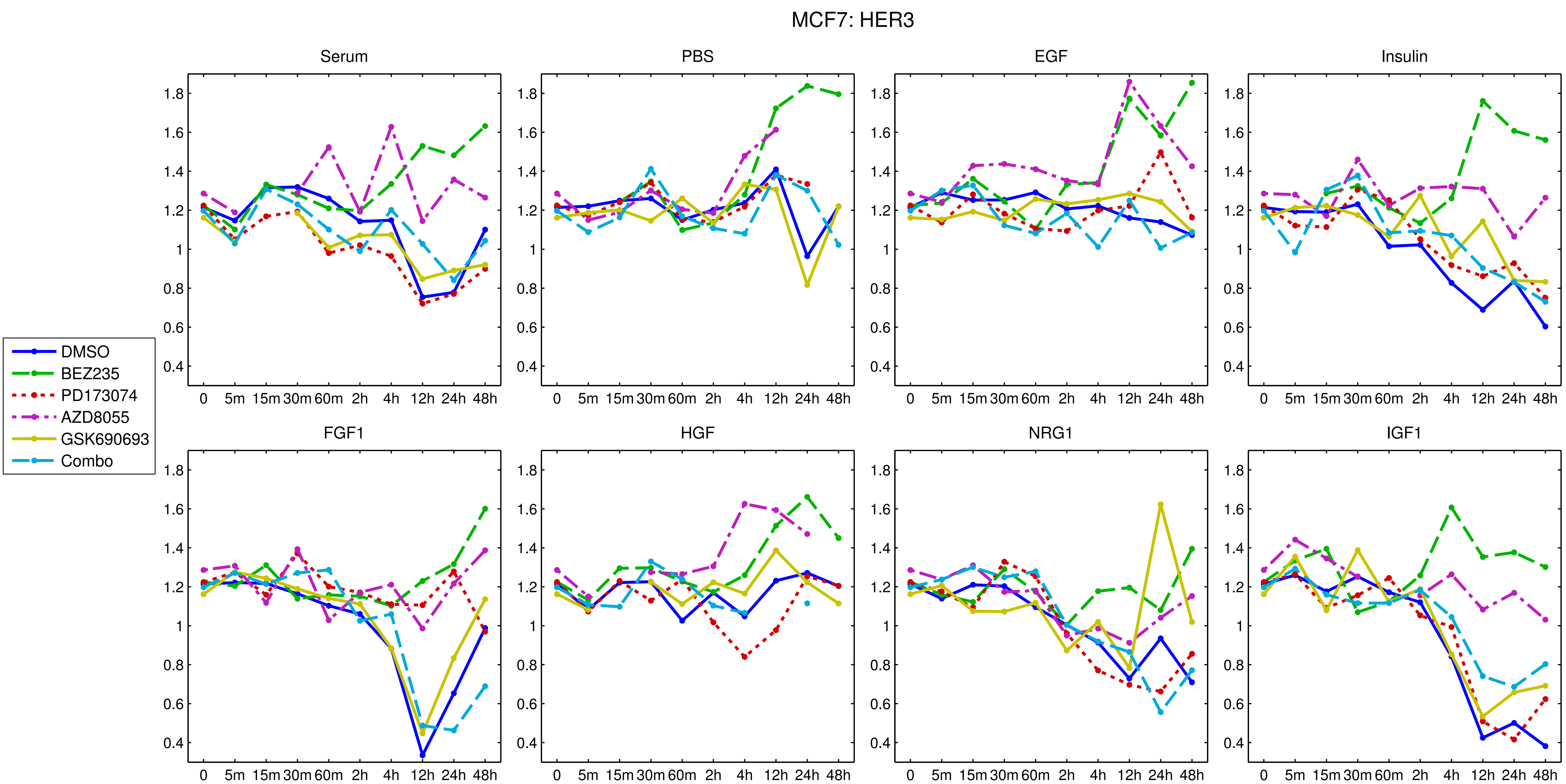
MCF7: HER3
Serum
PBS
EGF
Insulin
FGF1
HGF
NRG1
IGF1
1.8
1.6
1.4
1.2
1
0.8
0.6
0.4
0
5m
15m
30m
60m
2h
4h
12h
24h
48h
DMSO
BEZ235
PD173074
AZD8055
GSK690693
Combo

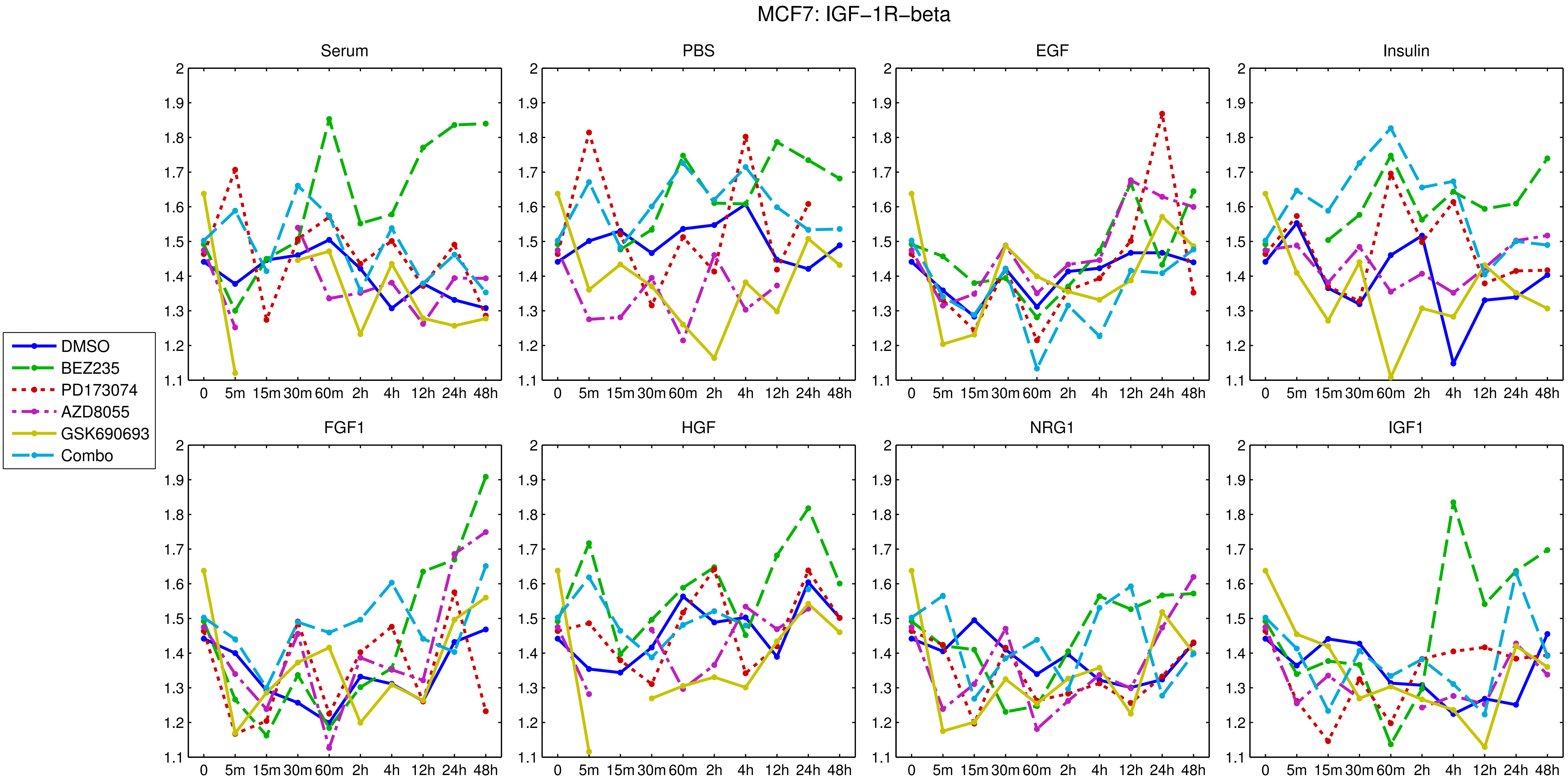

MCF7: IGF−1R−beta
Serum
PBS
EGF
Insulin
FGF1
HGF
NRG1
IGF1
DMSO
BEZ235
PD173074
AZD8055
GSK690693
Combo
0
5m
15m
30m
60m
2h
4h
12h
24h
48h
1.1
1.2
1.3
1.4
1.5
1.6
1.7
1.8
1.9
2

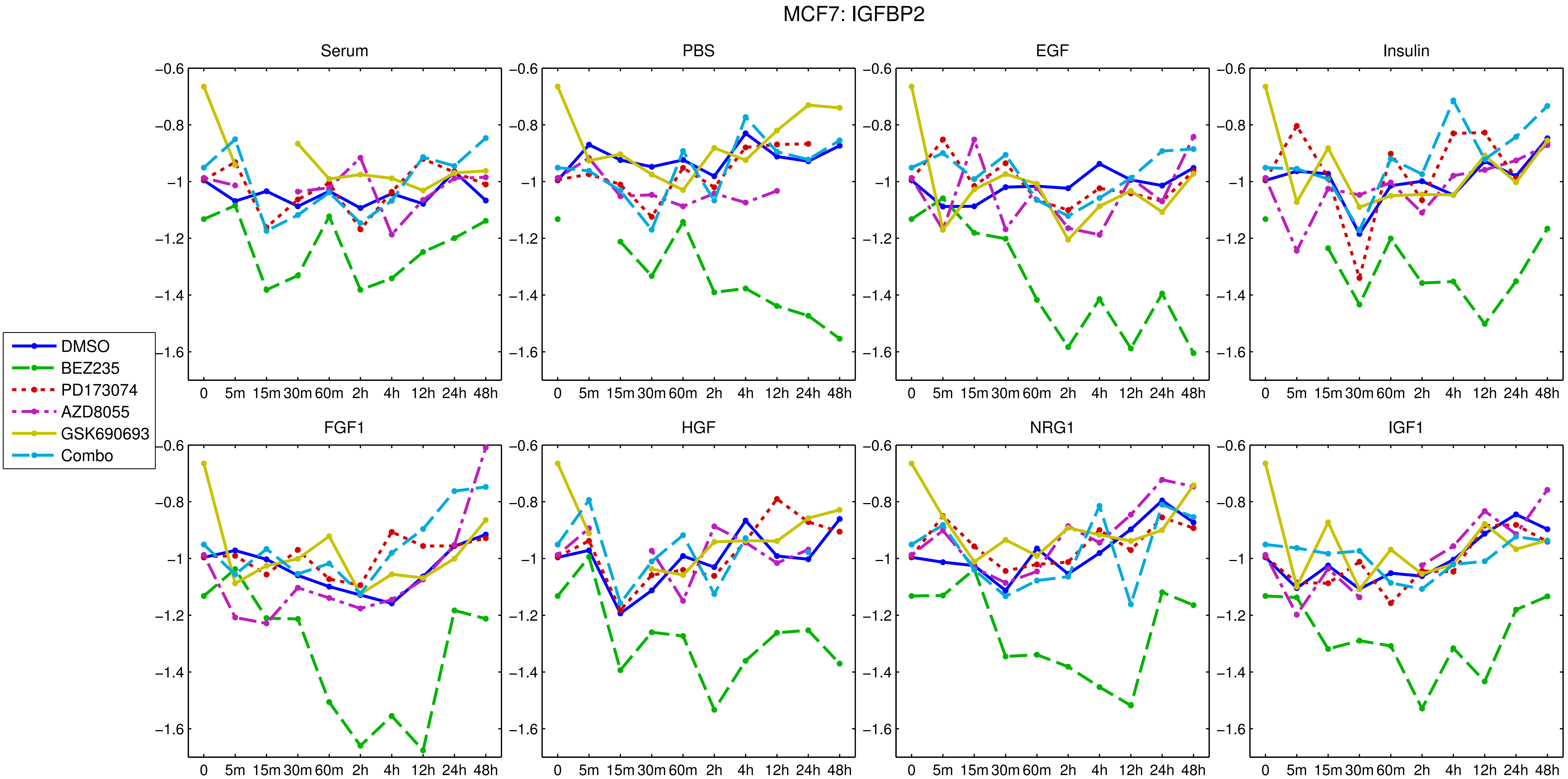
MCF7: IGFBP2
Serum
PBS
EGF
Insulin
FGF1
HGF
NRG1
IGF1
DMSO
BEZ235
PD173074
AZD8055
GSK690693
Combo
−0.6
−0.8
−1
−1.2
−1.4
−1.6
0
5m
15m
30m
60m
2h
4h
12h
24h
48h

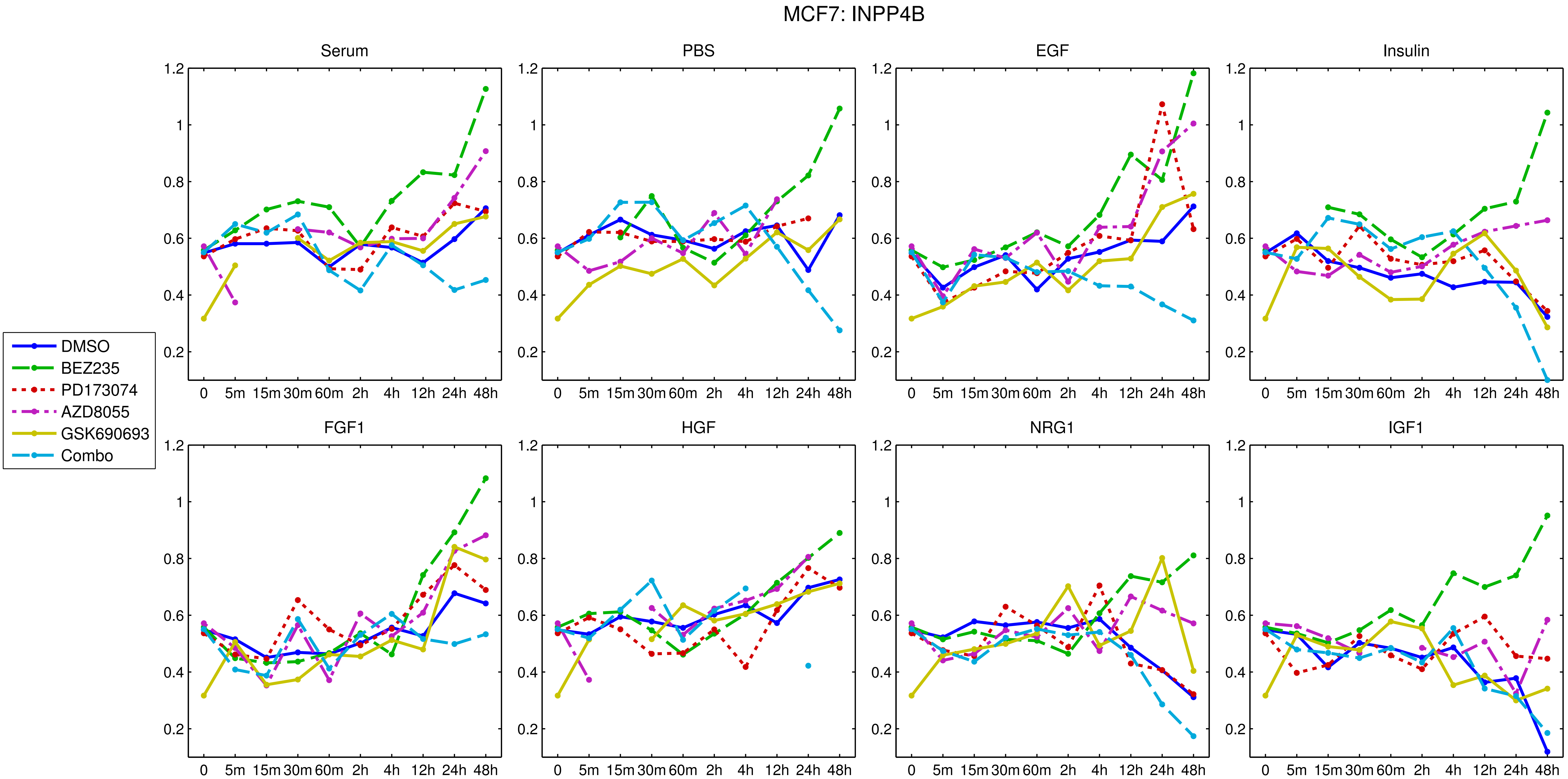
MCF7: INPP4B
Serum
PBS
EGF
Insulin
FGF1
HGF
NRG1
IGF1
DMSO
BEZ235
PD173074
AZD8055
GSK690693
Combo
1.2
1
0.8
0.6
0.4
0.2
0
5m
15m
30m
60m
2h
4h
12h
24h
48h

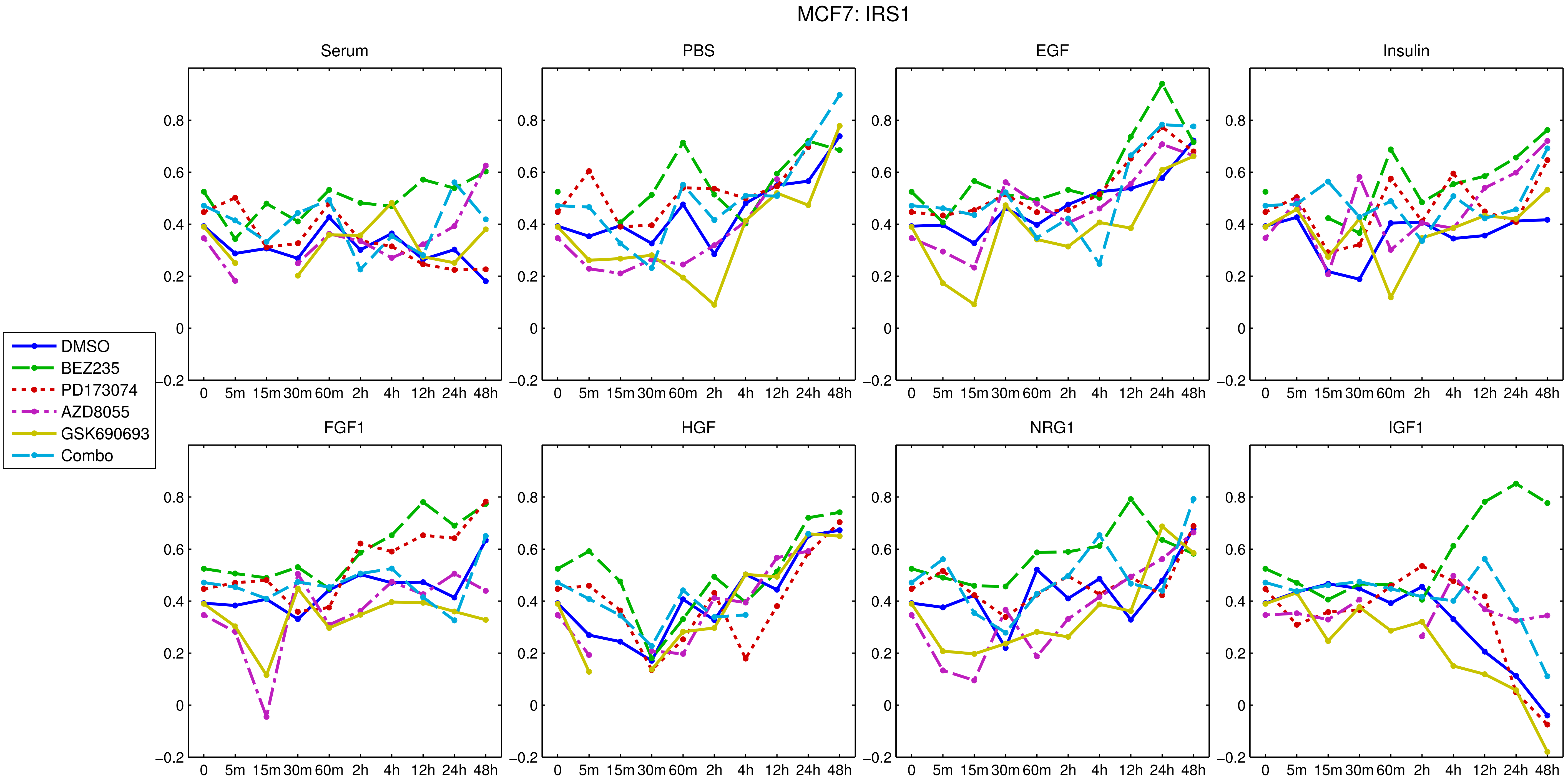
MCF7: IRS1
Serum
PBS
EGF
Insulin
FGF1
HGF
NRG1
IGF1
DMSO
BEZ235
PD173074
AZD8055
GSK690693
Combo
0.8
0.6
0.4
0.2
0
−0.2
0
5m
15m
30m
60m
2h
4h
12h
24h
48h

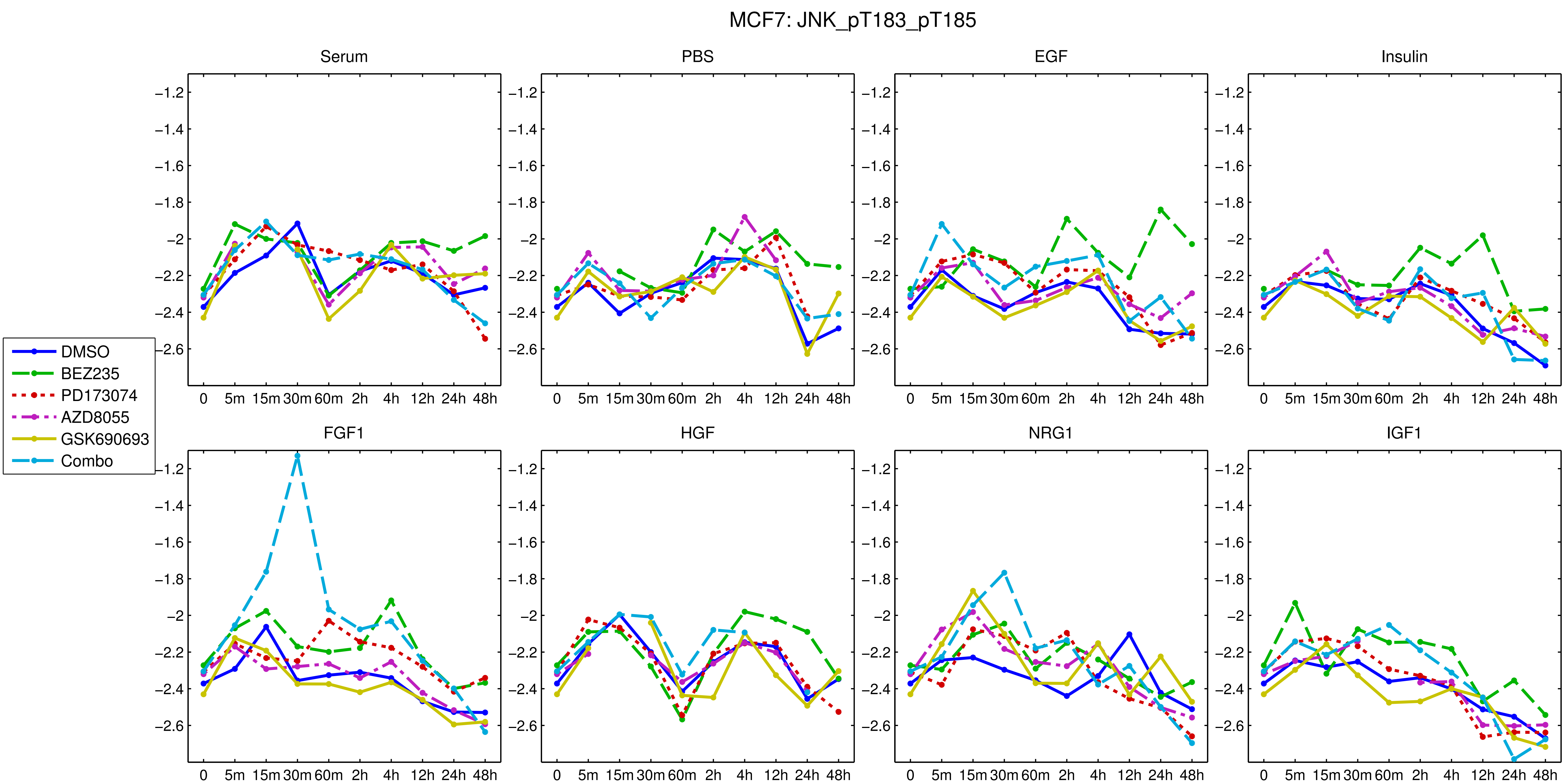

MCF7: JNK_pT183_pT185
Serum
PBS
EGF
Insulin
FGF1
HGF
NRG1
IGF1
DMSO
BEZ235
PD173074
AZD8055
GSK690693
Combo
−1.2
−1.4
−1.6
−1.8
−2
−2.2
−2.4
−2.6
0
5m
15m
30m
60m
2h
4h
12h
24h
48h

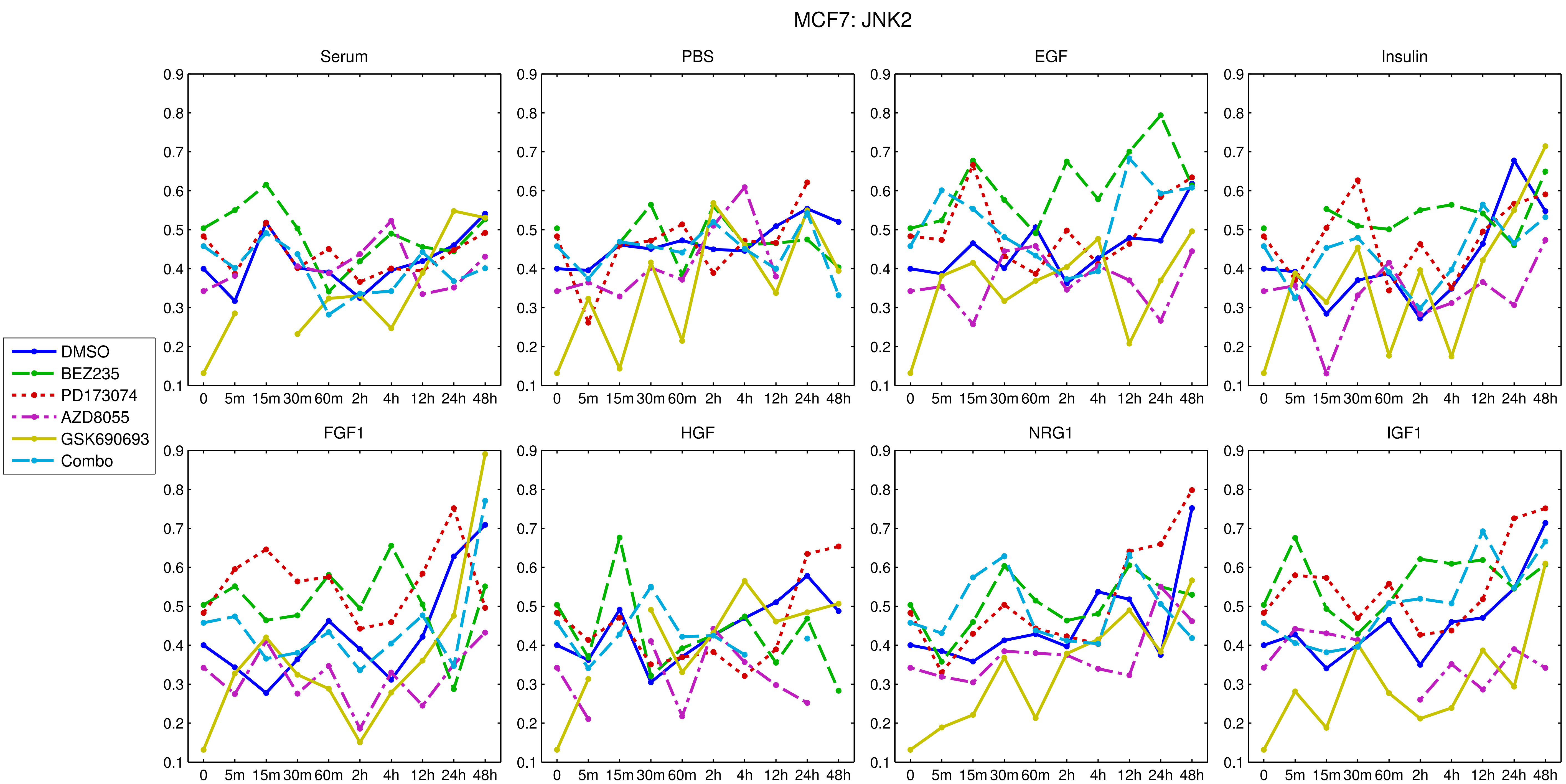
MCF7: JNK2
Serum
PBS
EGF
Insulin
FGF1
HGF
NRG1
IGF1
DMSO
BEZ235
PD173074
AZD8055
GSK690693
Combo
0.9
0.8
0.7
0.6
0.5
0.4
0.3
0.2
0.1
0 5m 15m 30m 60m 2h 4h 12h 24h 48h

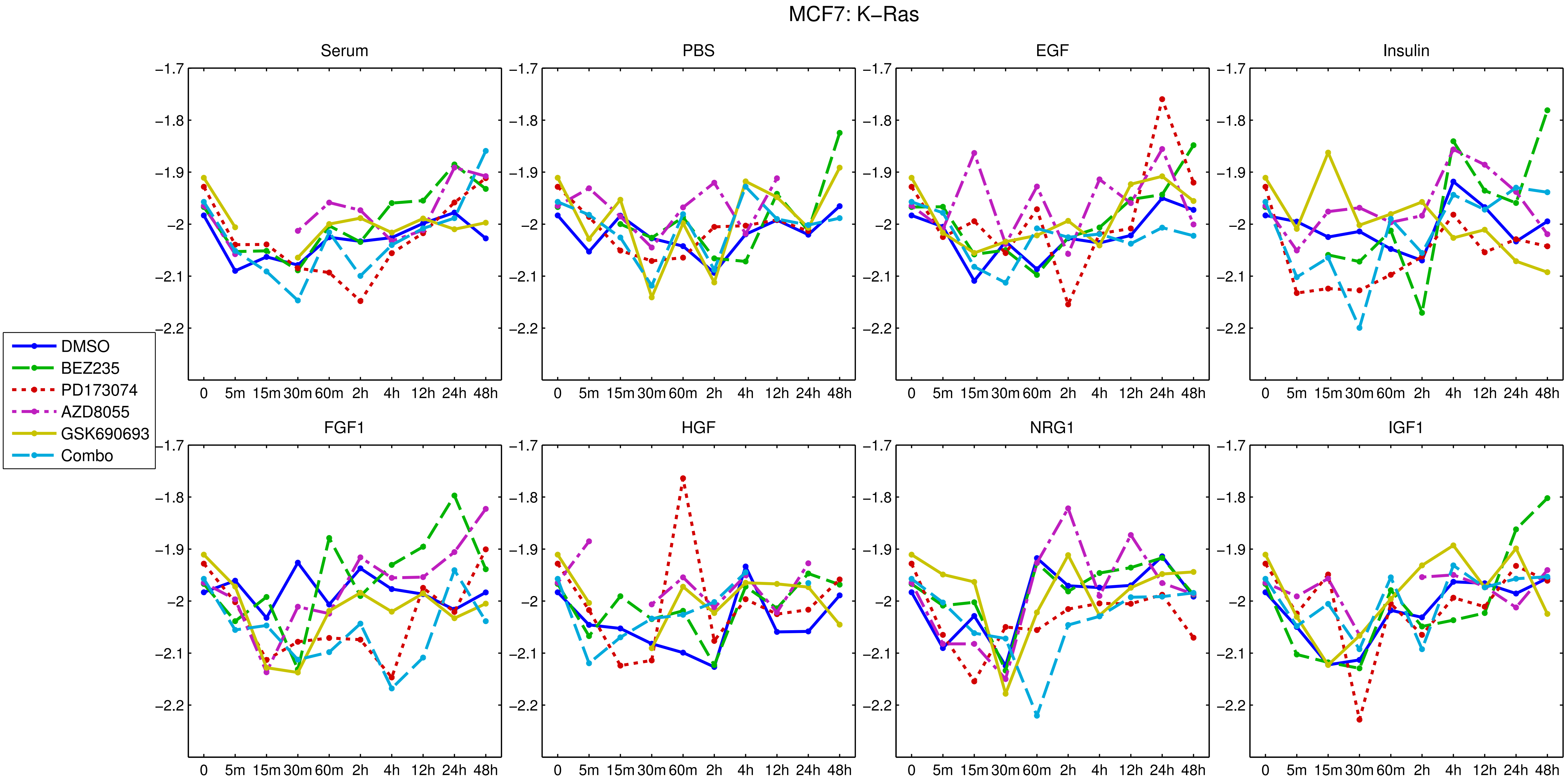
MCF7: K–Ras
Serum
PBS
EGF
Insulin
FGF1
HGF
NRG1
IGF1
−1.7
−1.8
−1.9
−2
−2.1
−2.2
0 5m 15m 30m 60m 2h 4h 12h 24h 48h
DMSO
BEZ235
PD173074
AZD8055
GSK690693
Combo

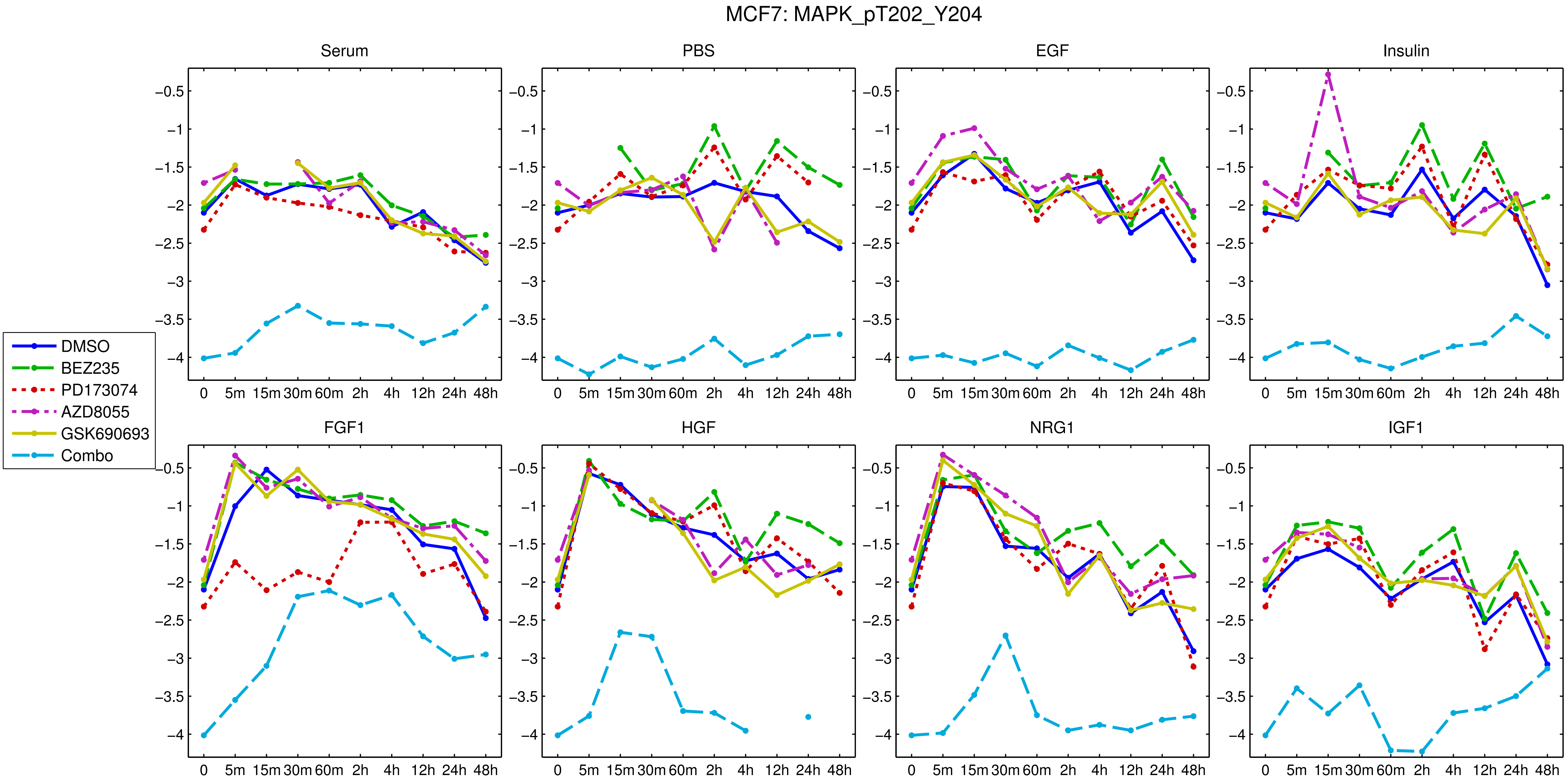
MCF7: MAPK_pT202_Y204
Serum
PBS
EGF
Insulin
FGF1
HGF
NRG1
IGF1
DMSO
BEZ235
PD173074
AZD8055
GSK690693
Combo
0
5m
15m
30m
60m
2h
4h
12h
24h
48h
−0.5
−1
−1.5
−2
−2.5
−3
−3.5
−4

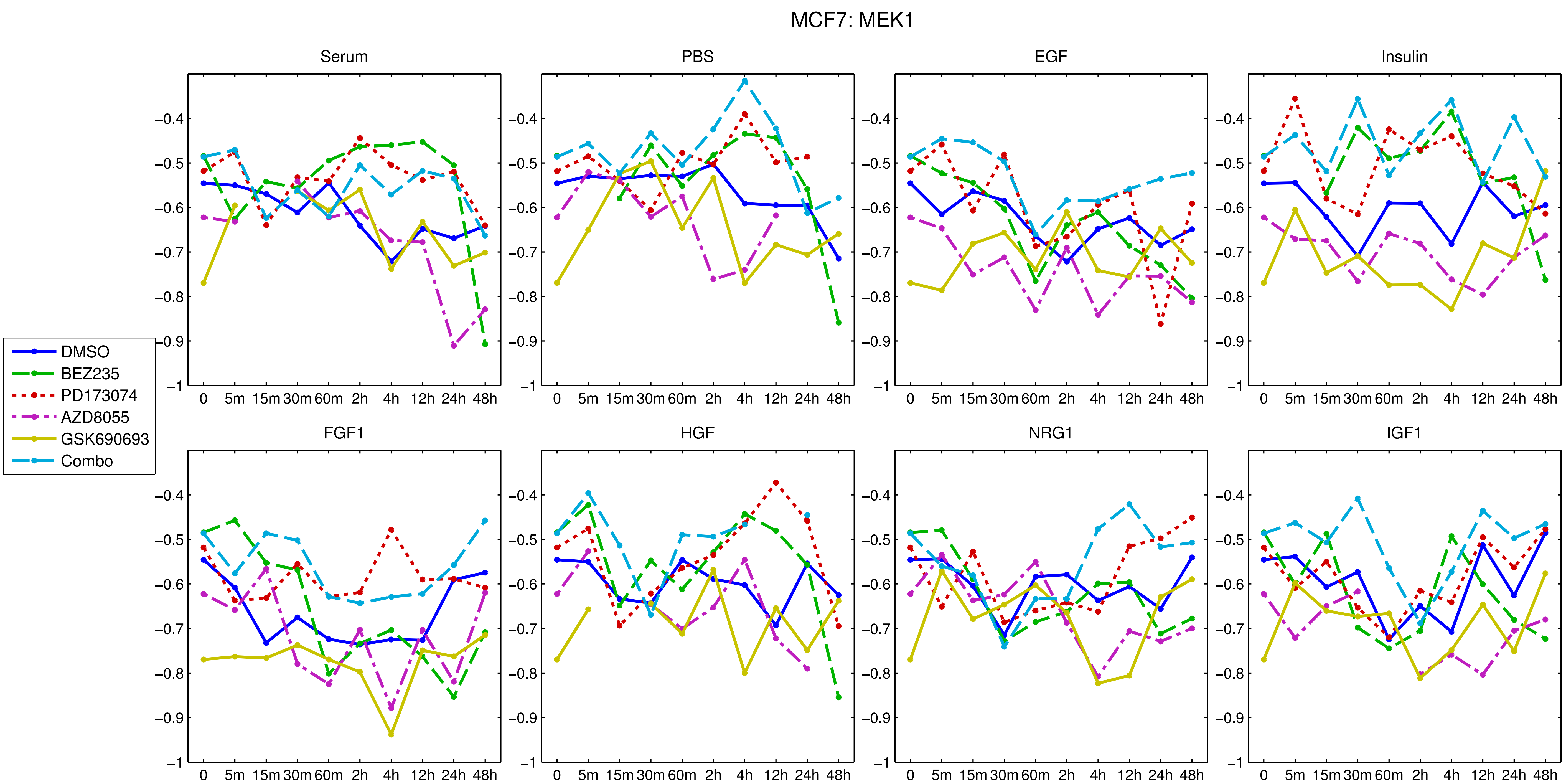
MCF7: MEK1
Serum
PBS
EGF
Insulin
FGF1
HGF
NRG1
IGF1
DMSO
BEZ235
PD173074
AZD8055
GSK690693
Combo
−0.4
−0.5
−0.6
−0.7
−0.8
−0.9
−1
0
5m
15m
30m
60m
2h
4h
12h
24h
48h

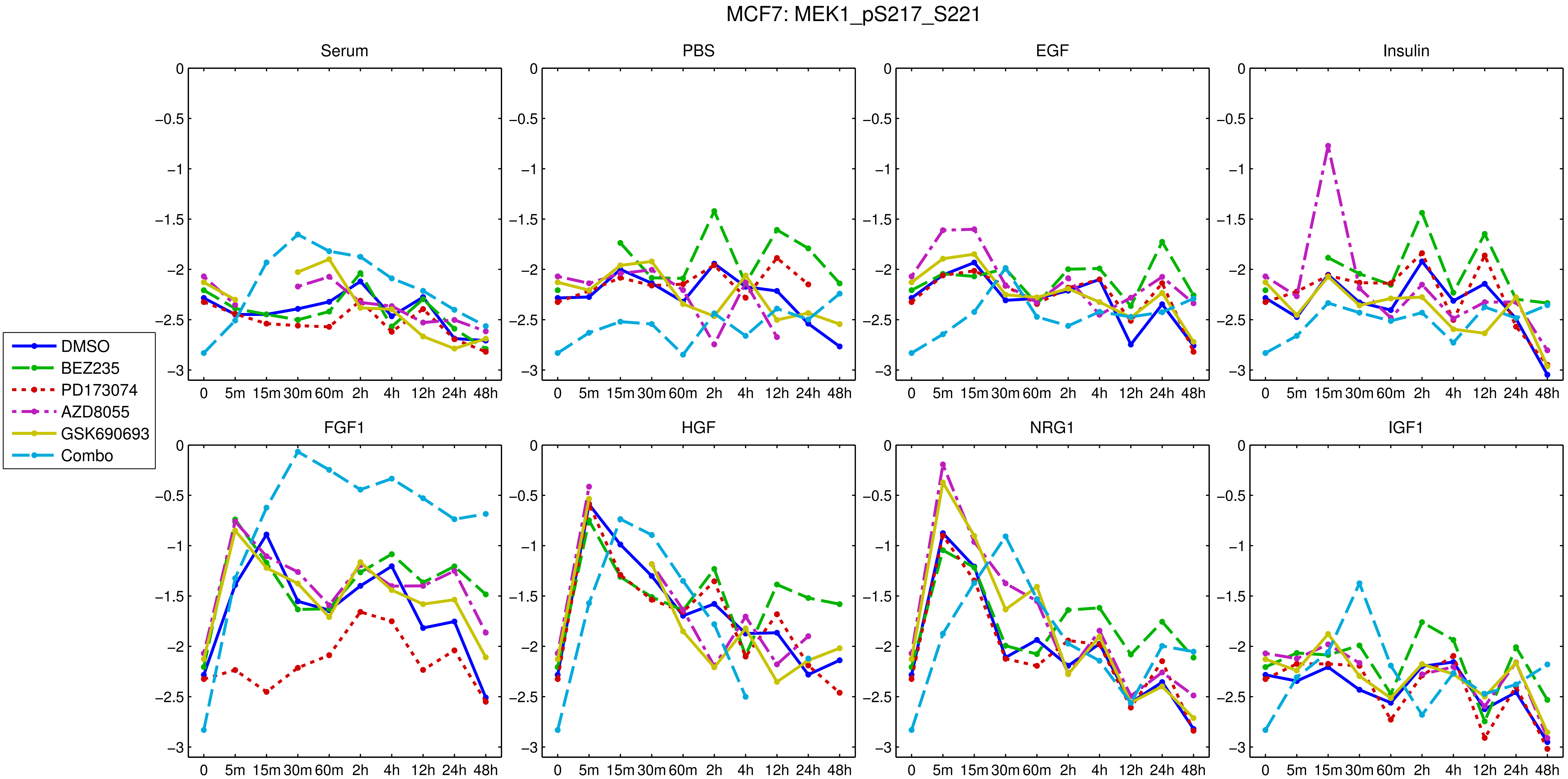

MCF7: MEK1_pS217_S221
Serum
PBS
EGF
Insulin
FGF1
HGF
NRG1
IGF1
DMSO
BEZ235
PD173074
AZD8055
GSK690693
Combo
0
−0.5
−1
−1.5
−2
−2.5
−3
0
5m
15m
30m
60m
2h
4h
12h
24h
48h

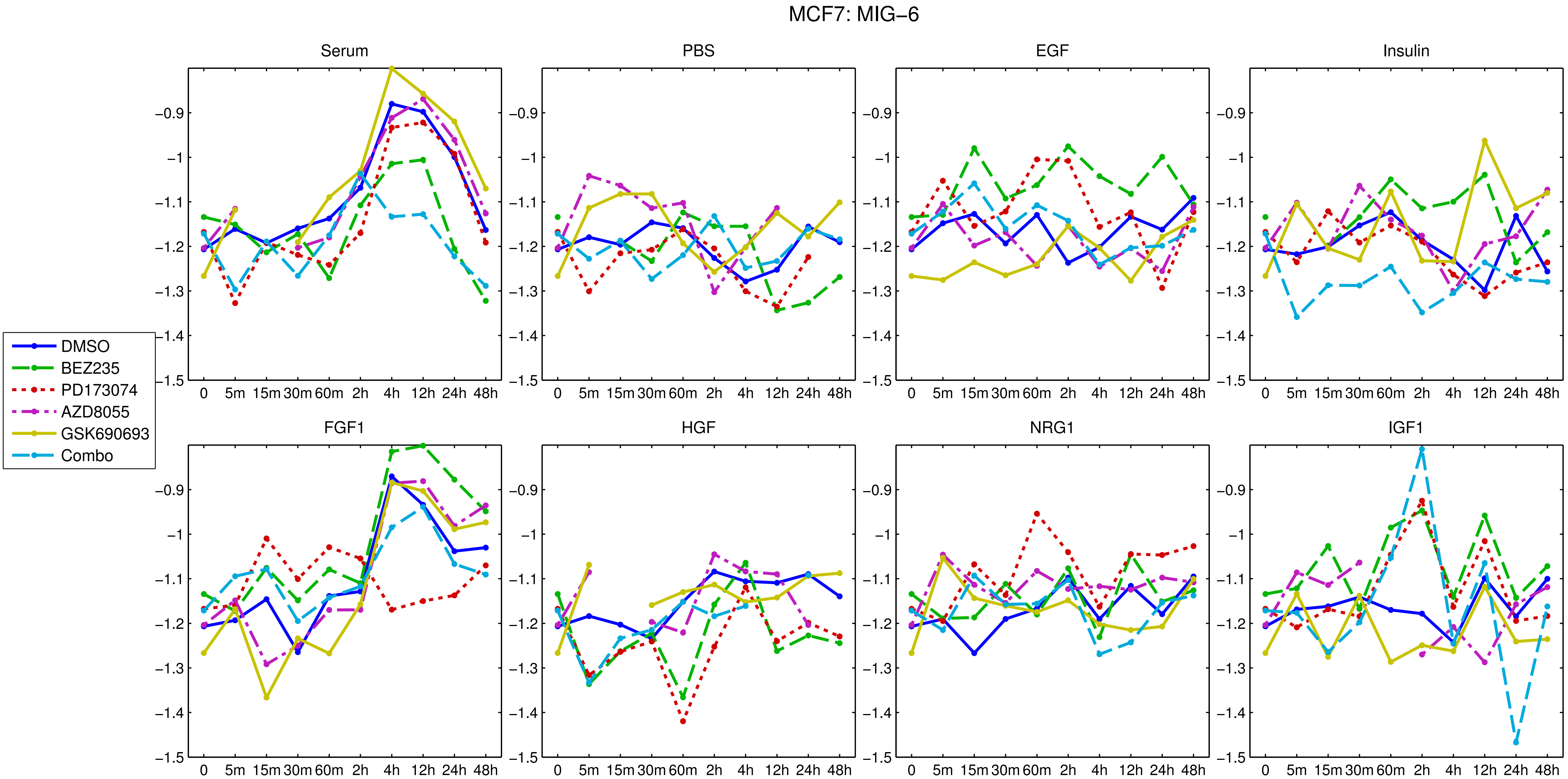

MCF7: MIG–6
Serum
PBS
EGF
Insulin
FGF1
HGF
NRG1
IGF1
DMSO
BEZ235
PD173074
AZD8055
GSK690693
Combo
–0.9
–1
–1.1
–1.2
–1.3
–1.4
–1.5
0
5m
15m
30m
60m
2h
4h
12h
24h
48h

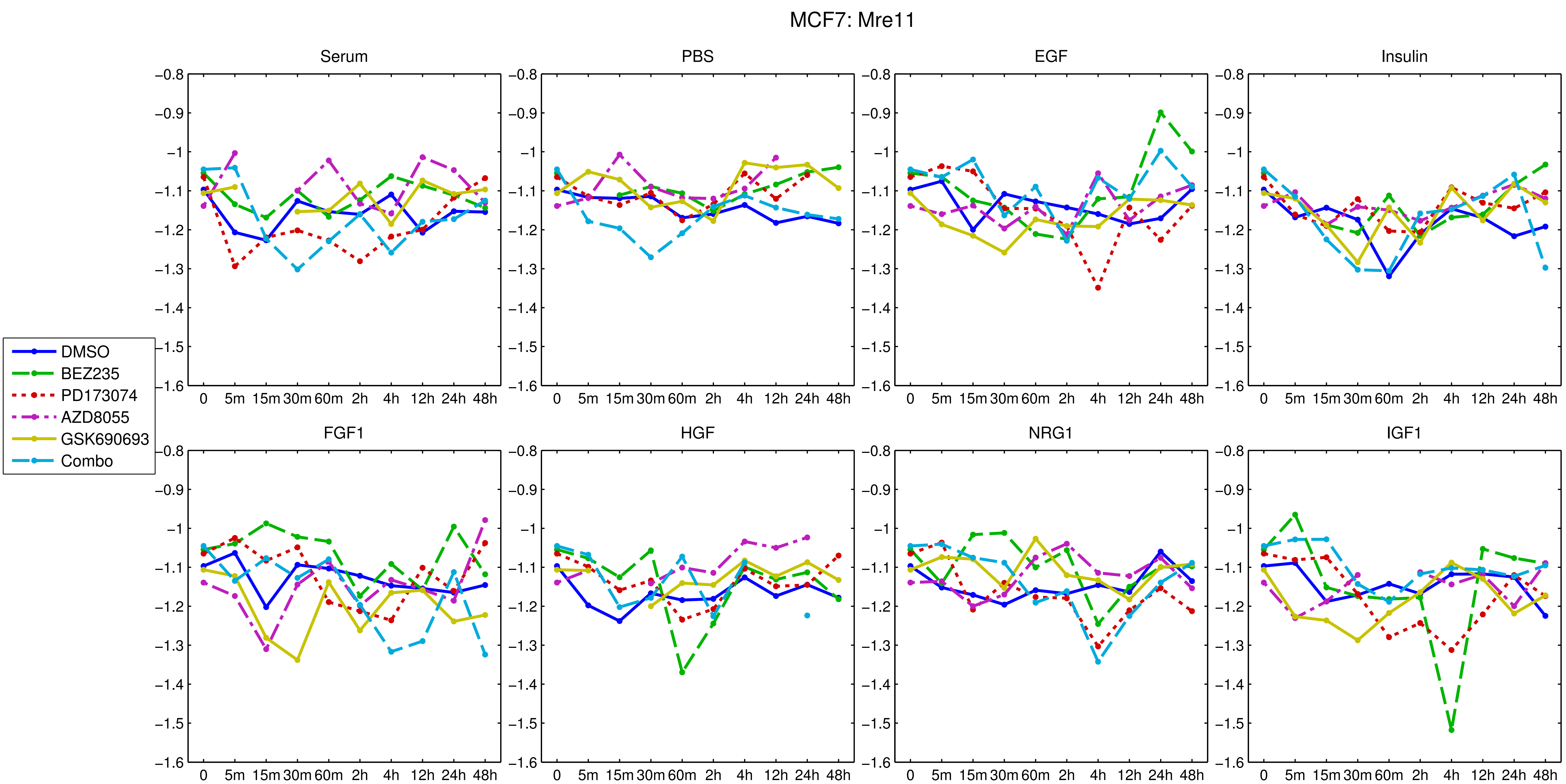
MCF7: Mre11
Serum
PBS
EGF
Insulin
FGF1
HGF
NRG1
IGF1
DMSO
BEZ235
PD173074
AZD8055
GSK690693
Combo
−0.8
−0.9
−1
−1.1
−1.2
−1.3
−1.4
−1.5
−1.6
0
5m
15m
30m
60m
2h
4h
12h
24h
48h

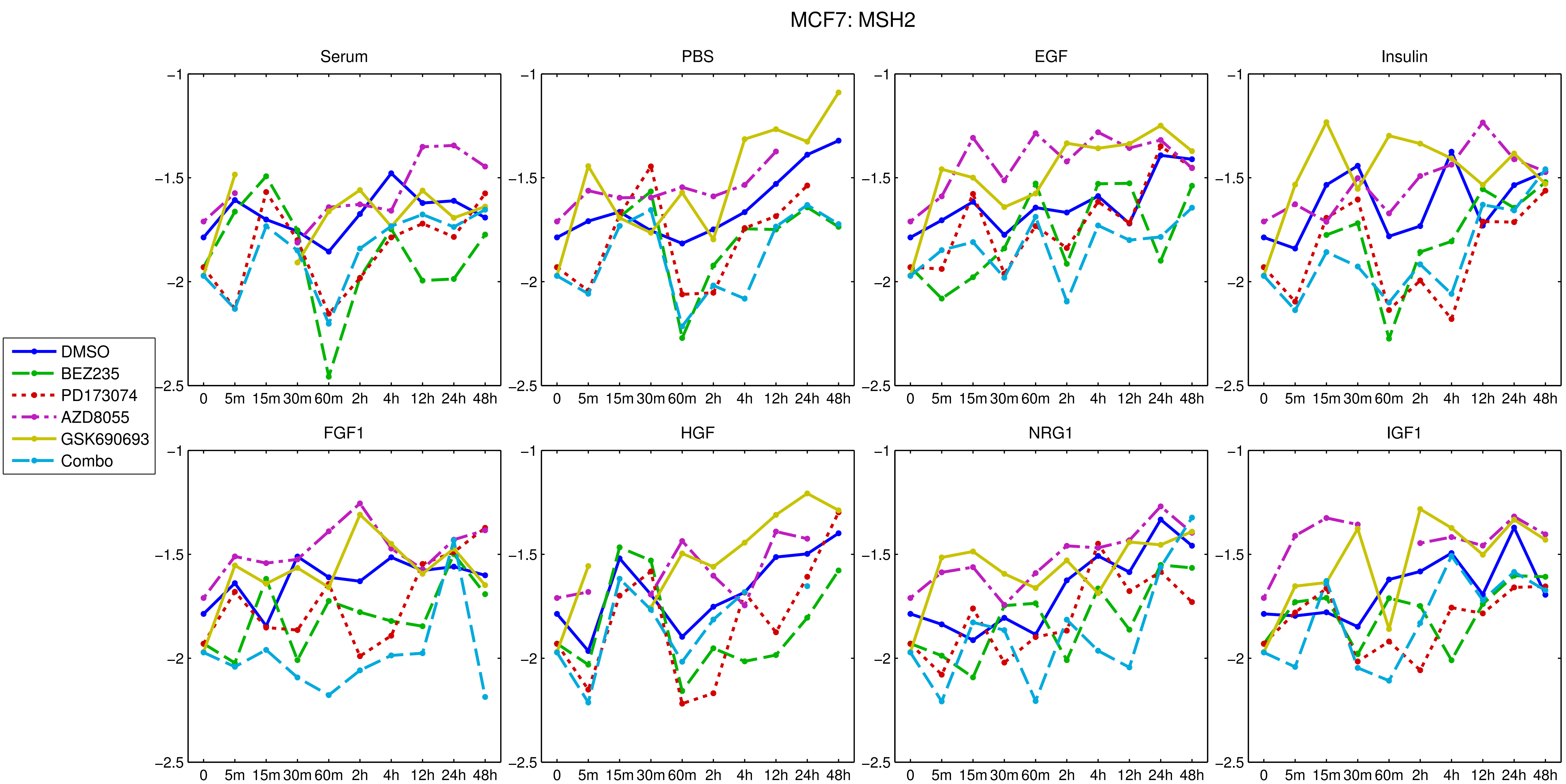
MCF7: MSH2
Serum
PBS
EGF
Insulin
FGF1
HGF
NRG1
IGF1
DMSO
BEZ235
PD173074
AZD8055
GSK690693
Combo
−1
−1.5
−2
−2.5
0
5m
15m
30m
60m
2h
4h
12h
24h
48h

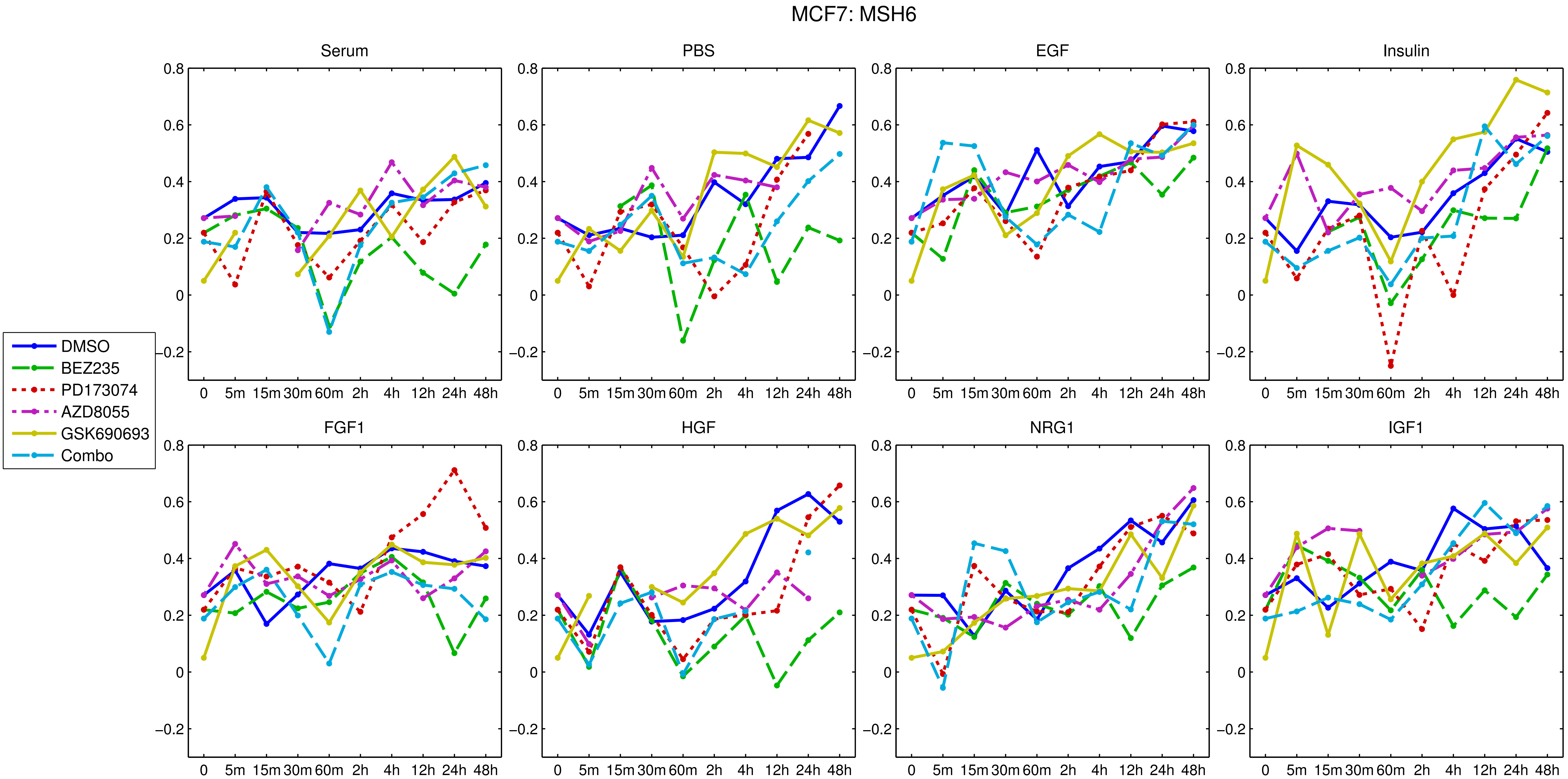
MCF7: MSH6
Serum
PBS
EGF
Insulin
FGF1
HGF
NRG1
IGF1
DMSO
BEZ235
PD173074
AZD8055
GSK690693
Combo
0.8
0.6
0.4
0.2
0
−0.2
0
5m
15m
30m
60m
2h
4h
12h
24h
48h

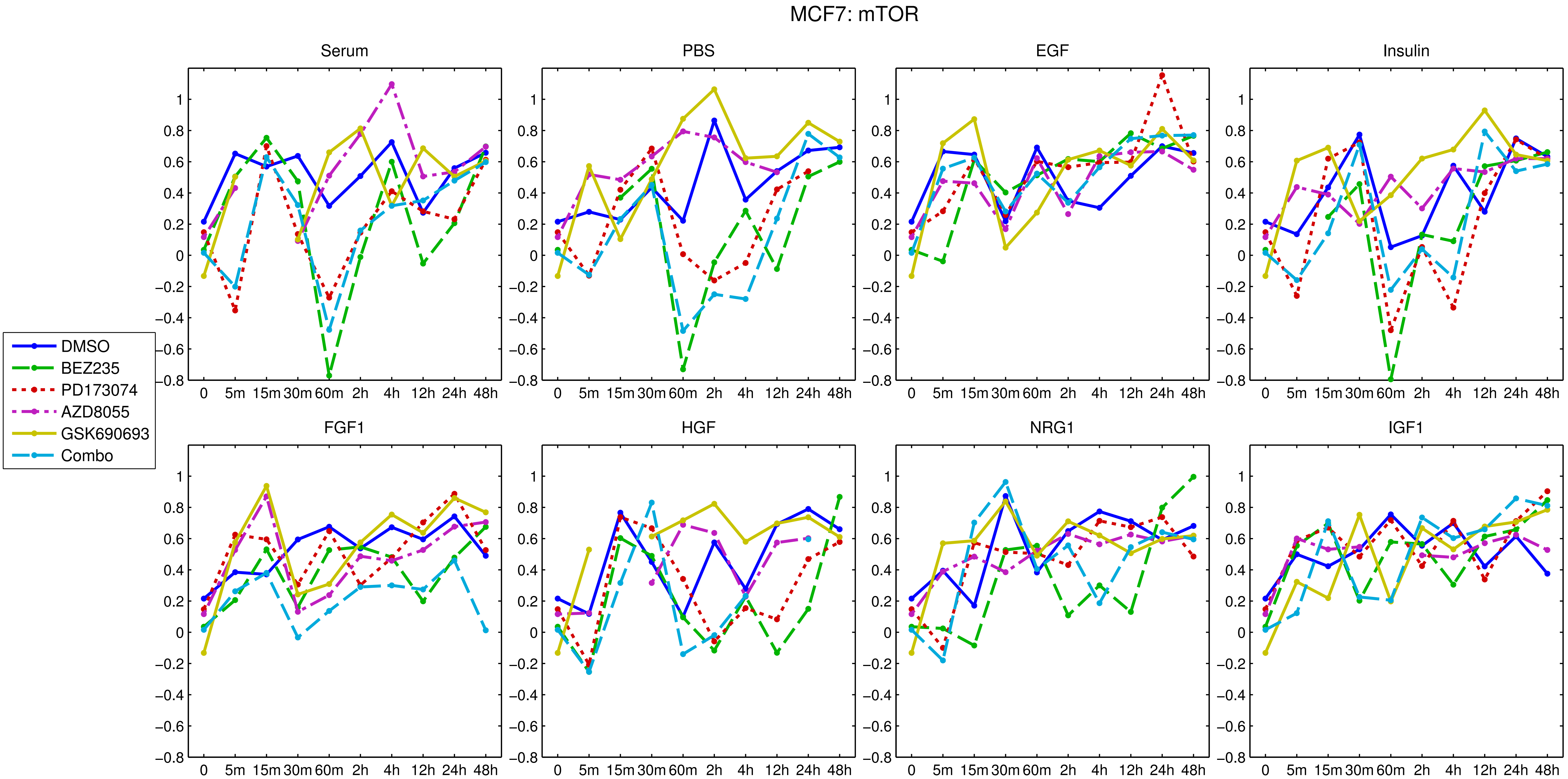
MCF7: mTOR
Serum
PBS
EGF
Insulin
FGF1
HGF
NRG1
IGF1
DMSO
BEZ235
PD173074
AZD8055
GSK690693
Combo
0
5m
15m
30m
60m
2h
4h
12h
24h
48h

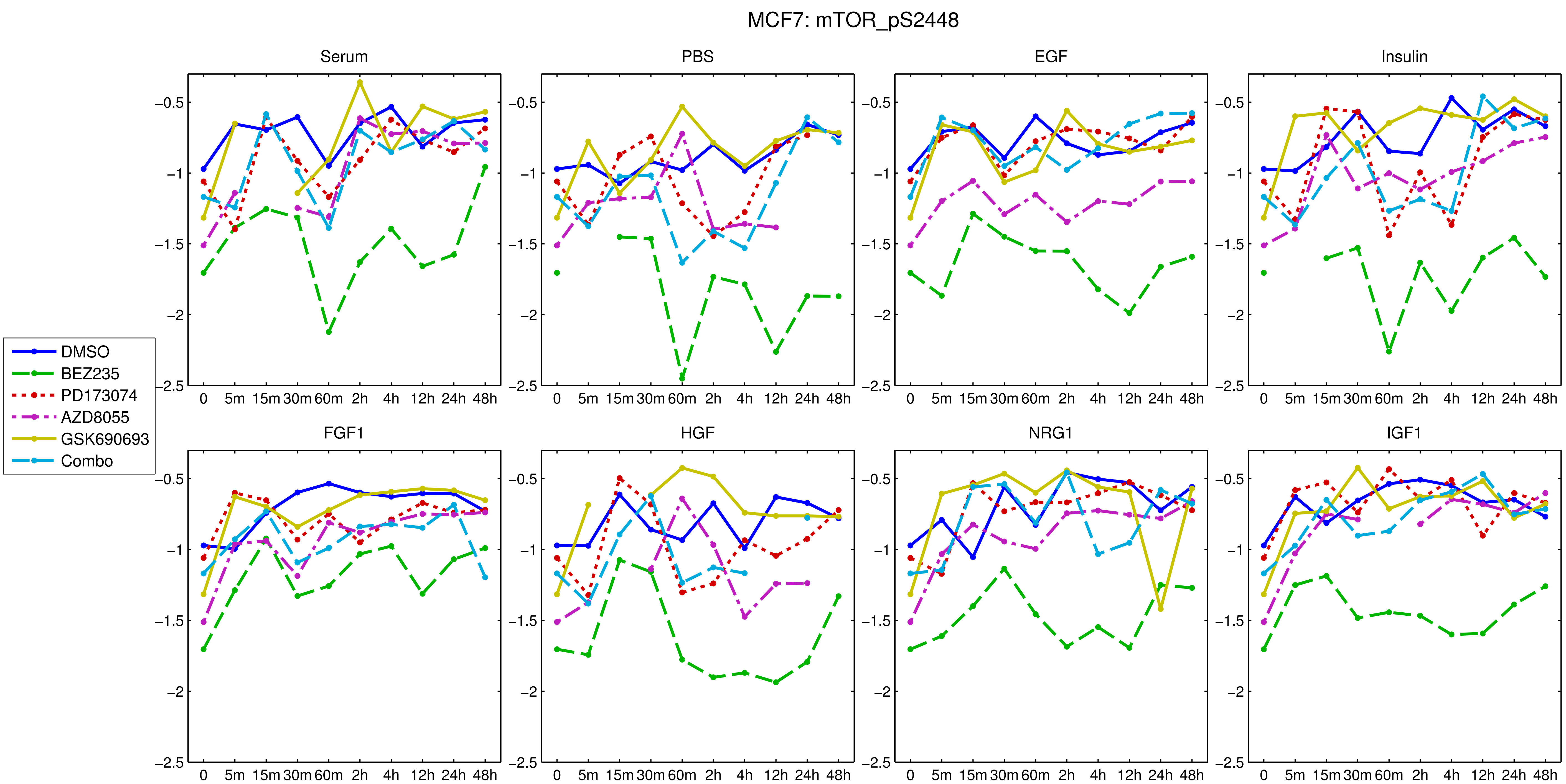
MCF7: mTOR_pS2448
Serum
PBS
EGF
Insulin
FGF1
HGF
NRG1
IGF1
DMSO
BEZ235
PD173074
AZD8055
GSK690693
Combo
−0.5
−1
−1.5
−2
−2.5
0
5m
15m
30m
60m
2h
4h
12h
24h
48h

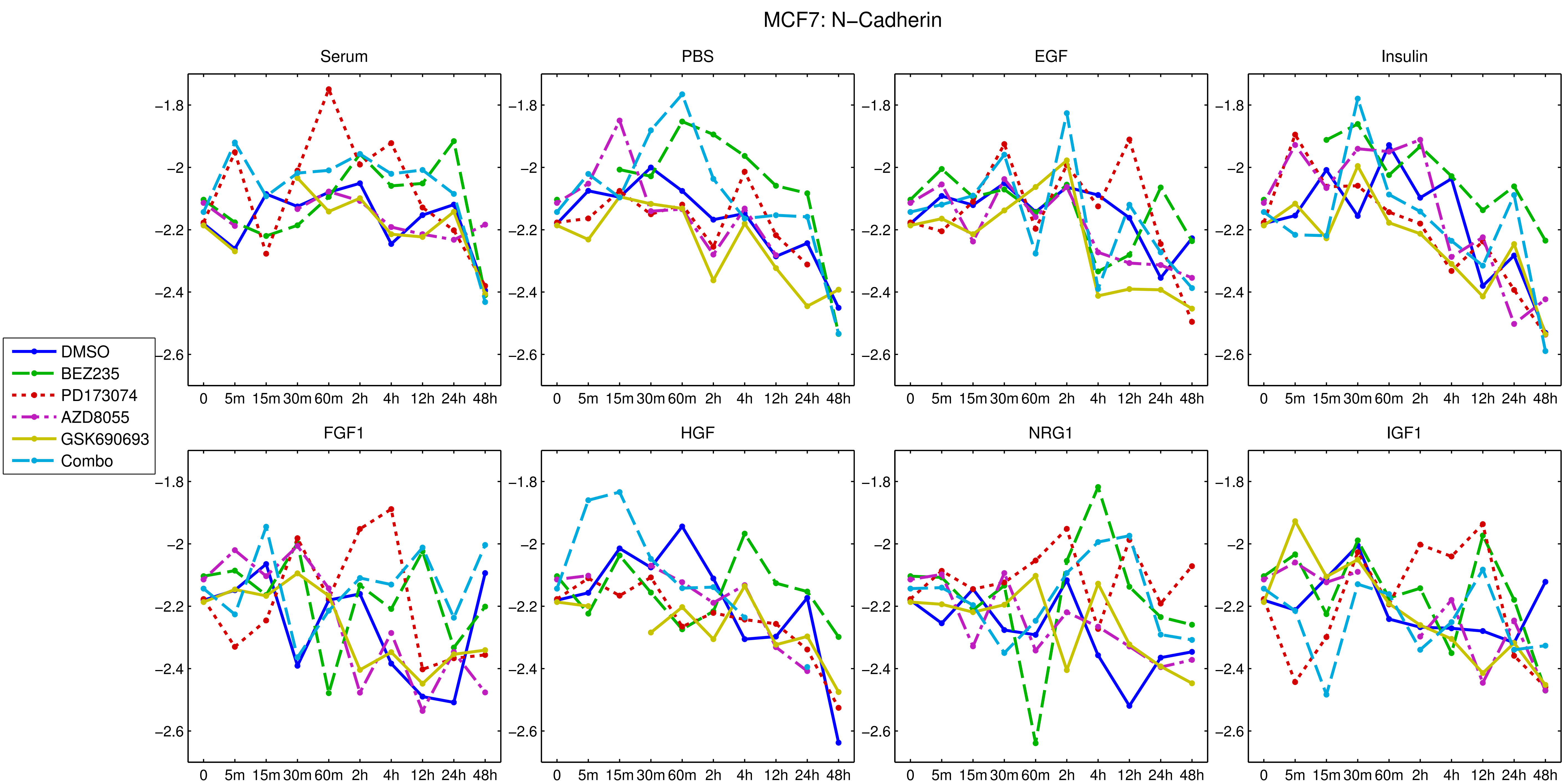
MCF7: N–Cadherin
Serum
PBS
EGF
Insulin
FGF1
HGF
NRG1
IGF1
DMSO
BEZ235
PD173074
AZD8055
GSK690693
Combo
−1.8
−2
−2.2
−2.4
−2.6
0
5m
15m
30m
60m
2h
4h
12h
24h
48h

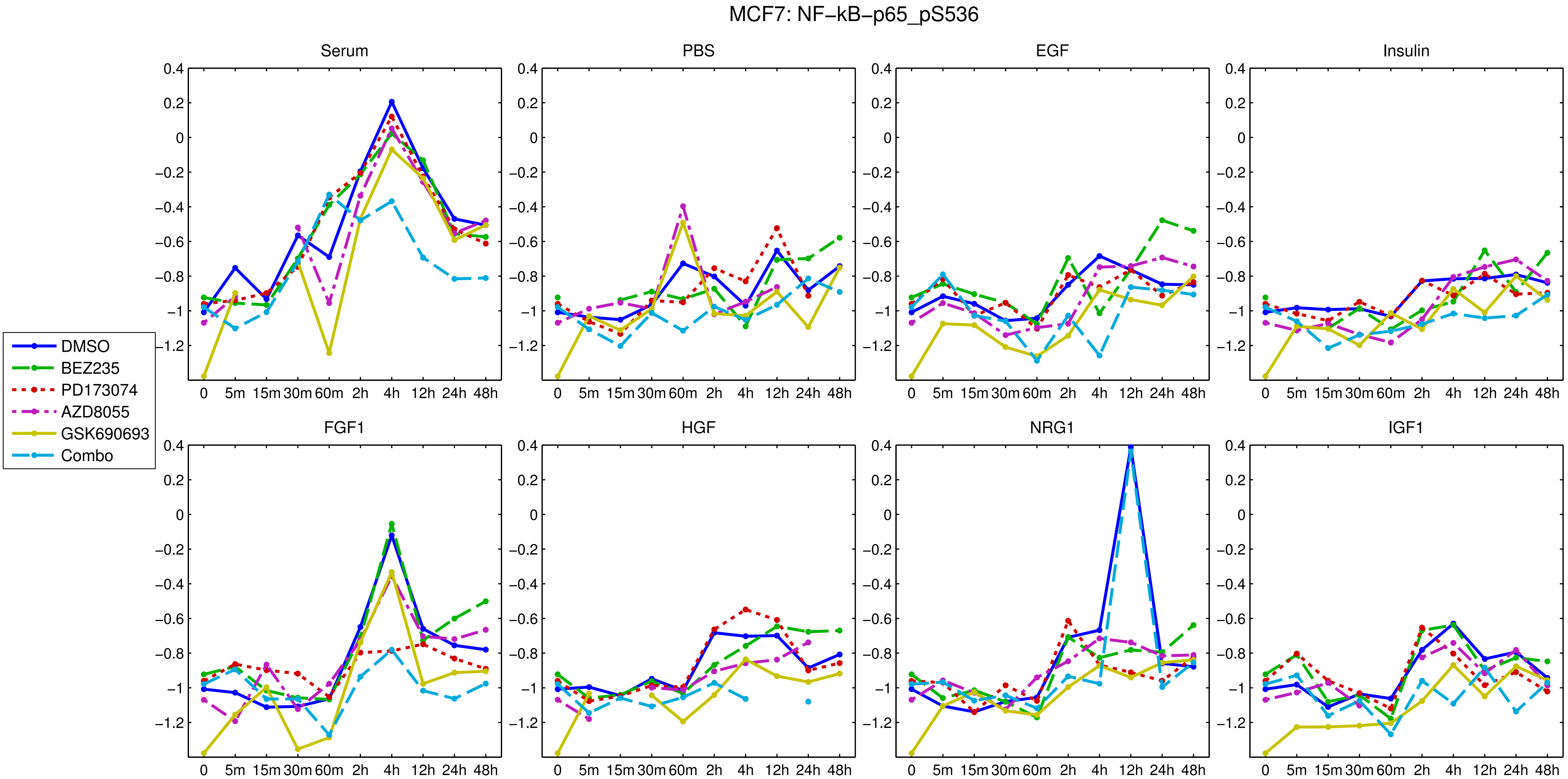
MCF7: NF−kB−p65_pS536
Serum
PBS
EGF
Insulin
FGF1
HGF
NRG1
IGF1
DMSO
BEZ235
PD173074
AZD8055
GSK690693
Combo
0.4
0.2
0
−0.2
−0.4
−0.6
−0.8
−1
−1.2
0
5m
15m
30m
60m
2h
4h
12h
24h
48h

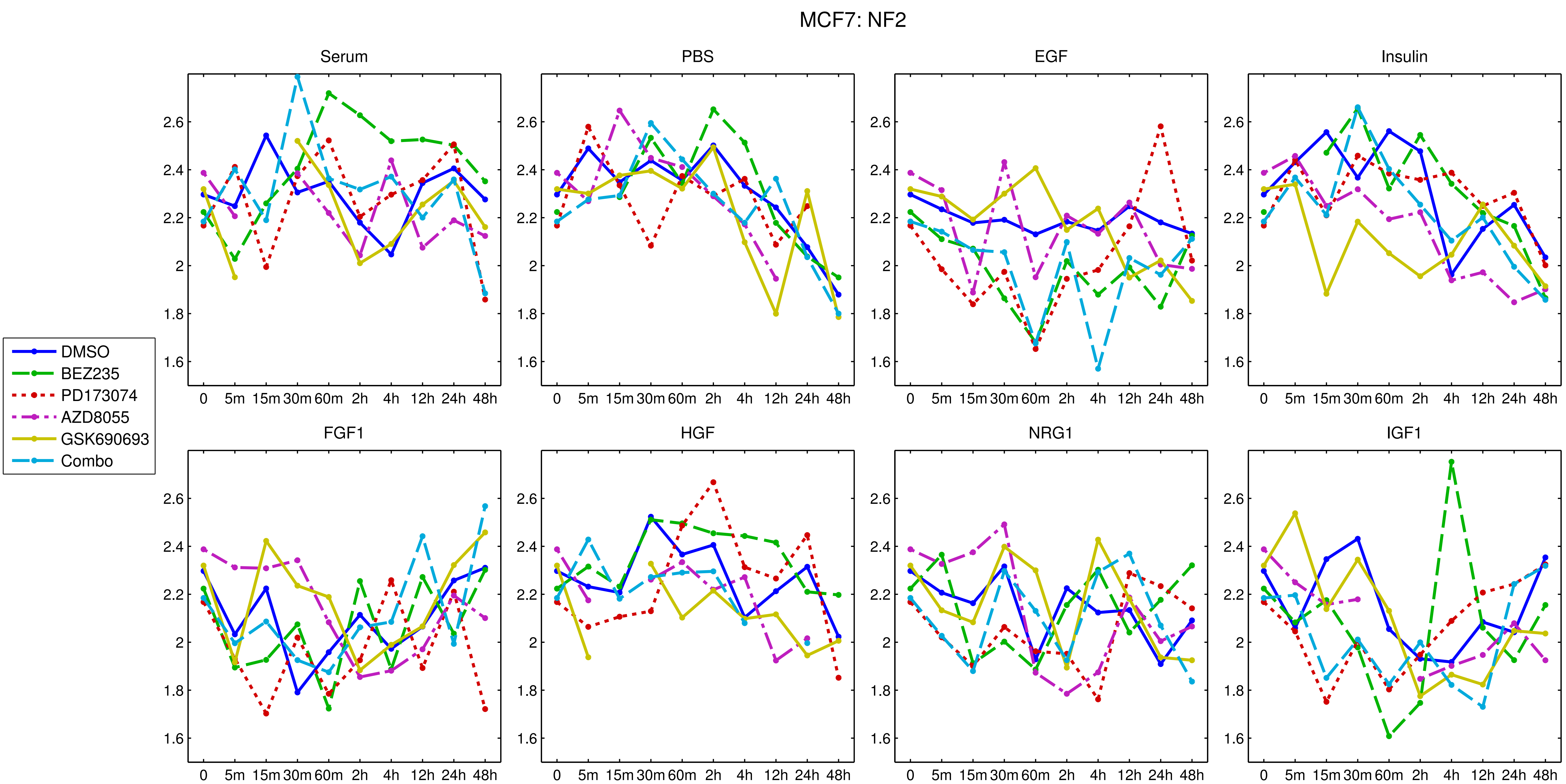
MCF7: NF2
Serum
PBS
EGF
Insulin
FGF1
HGF
NRG1
IGF1
DMSO
BEZ235
PD173074
AZD8055
GSK690693
Combo
2.6
2.4
2.2
2
1.8
1.6
0
5m
15m
30m
60m
2h
4h
12h
24h
48h

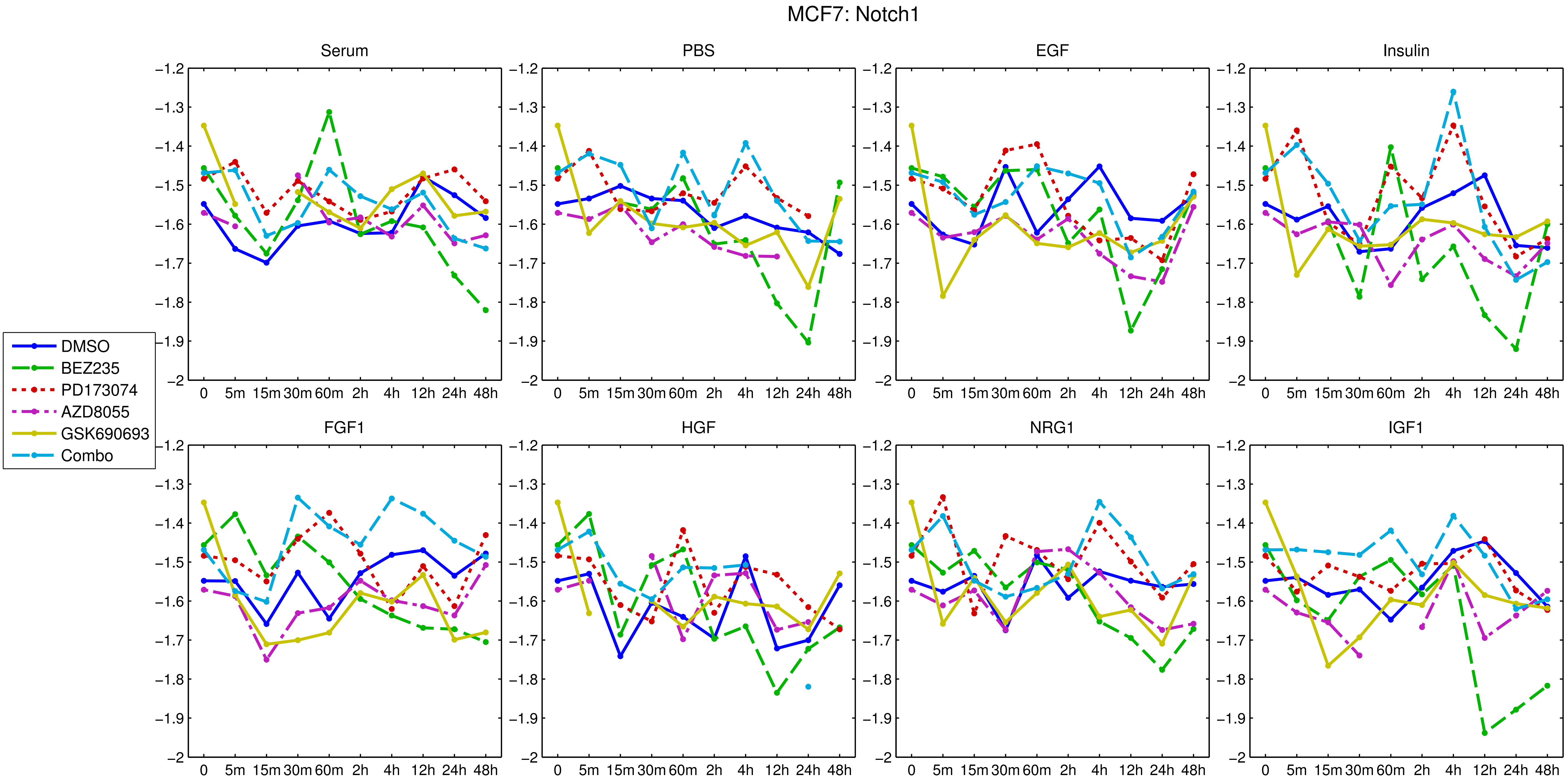
MCF7: Notch1
Serum
PBS
EGF
Insulin
FGF1
HGF
NRG1
IGF1
DMSO
BEZ235
PD173074
AZD8055
GSK690693
Combo
−1.2
−1.3
−1.4
−1.5
−1.6
−1.7
−1.8
−1.9
−2
0 5m 15m 30m 60m 2h 4h 12h 24h 48h

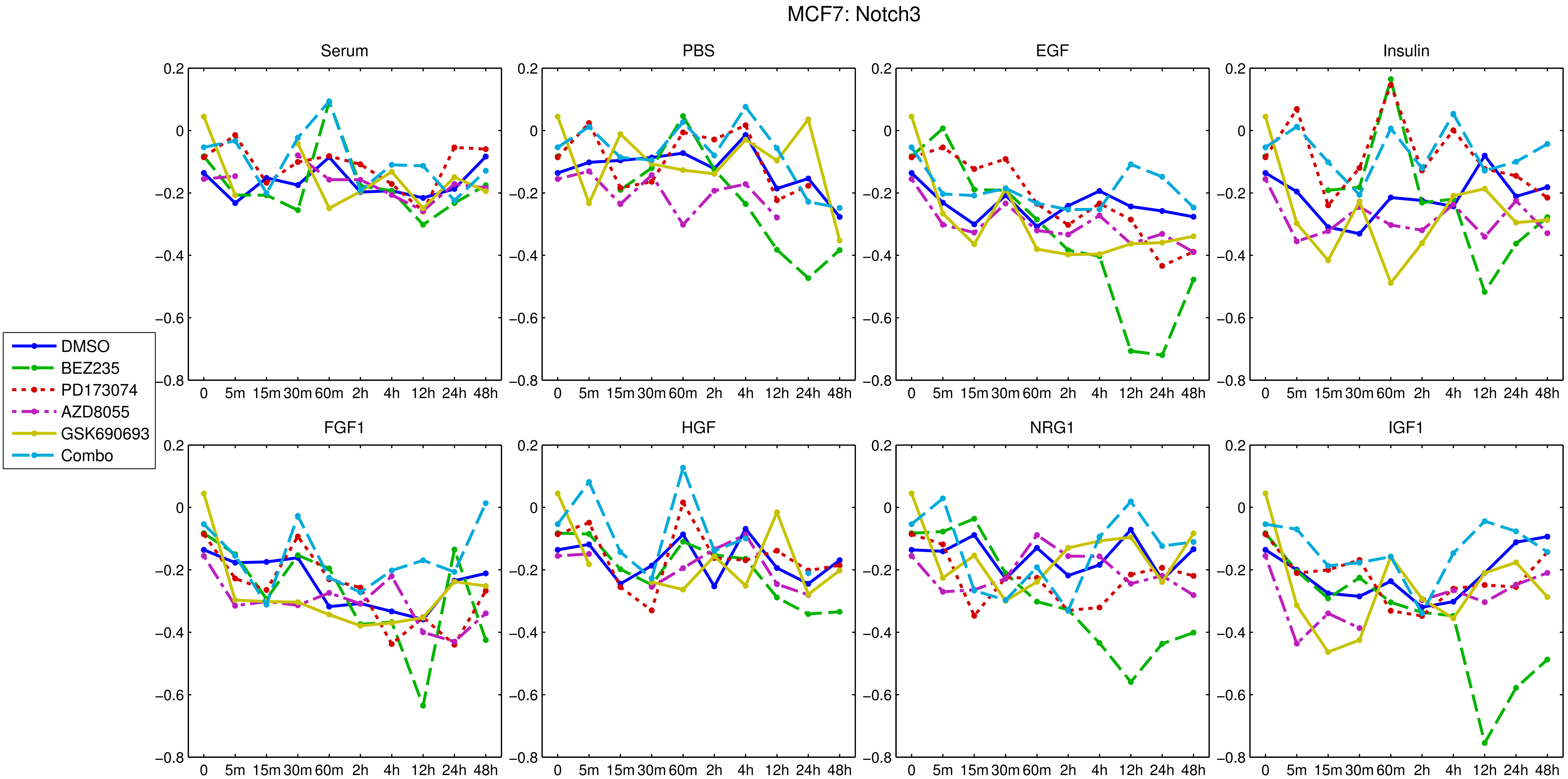
MCF7: Notch3
Serum
PBS
EGF
Insulin
FGF1
HGF
NRG1
IGF1
DMSO
BEZ235
PD173074
AZD8055
GSK690693
Combo
0.2
0
−0.2
−0.4
−0.6
−0.8
0
5m
15m
30m
60m
2h
4h
12h
24h
48h

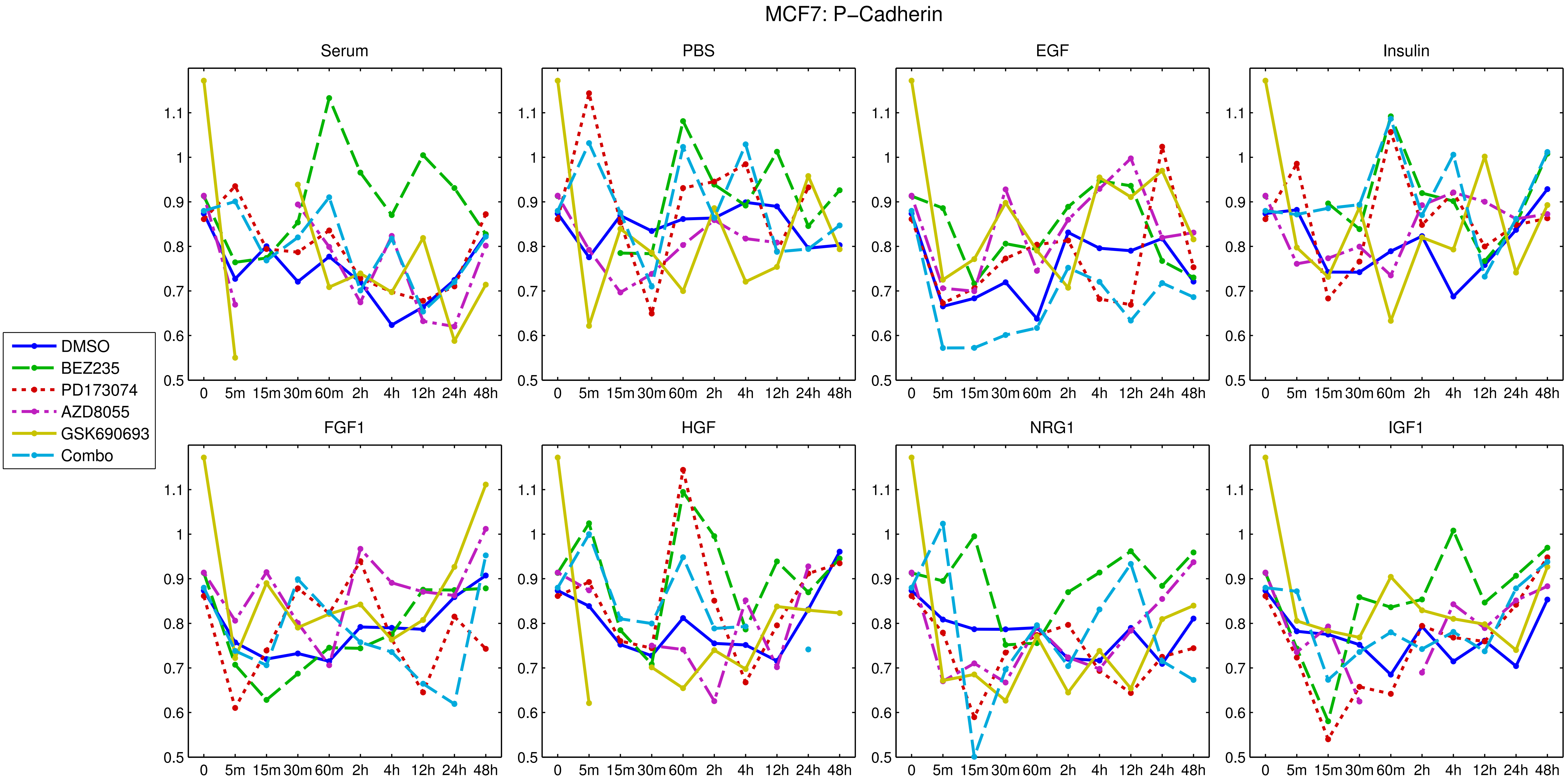

MCF7: P−Cadherin
Serum
PBS
EGF
Insulin
FGF1
HGF
NRG1
IGF1
DMSO
BEZ235
PD173074
AZD8055
GSK690693
Combo
0.5
0.6
0.7
0.8
0.9
1
1.1
0
5m
15m
30m
60m
2h
4h
12h
24h
48h

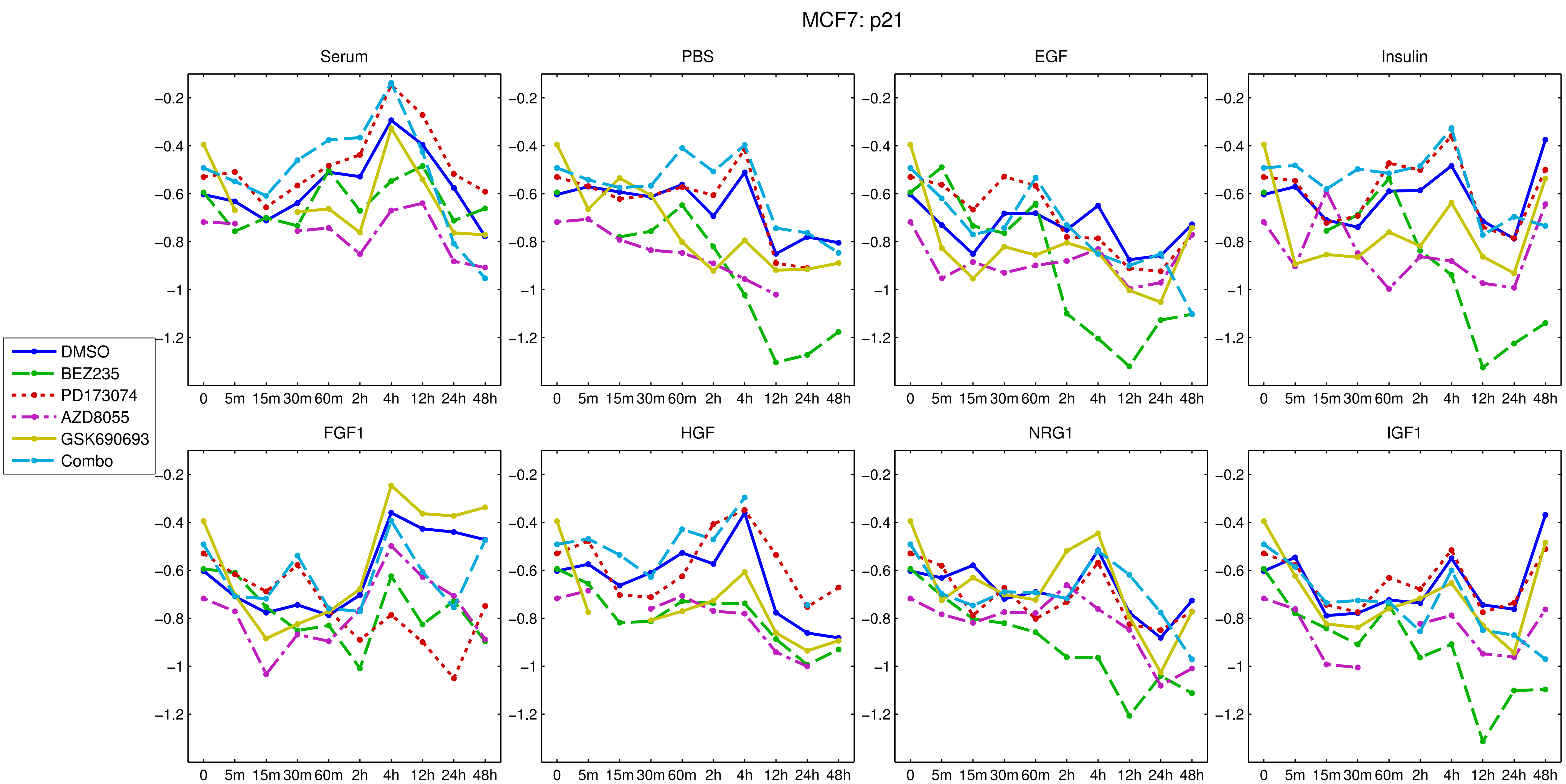

MCF7: p21
Serum
PBS
EGF
Insulin
FGF1
HGF
NRG1
IGF1
DMSO
BEZ235
PD173074
AZD8055
GSK690693
Combo
−0.2
−0.4
−0.6
−0.8
−1
−1.2
0
5m
15m
30m
60m
2h
4h
12h
24h
48h

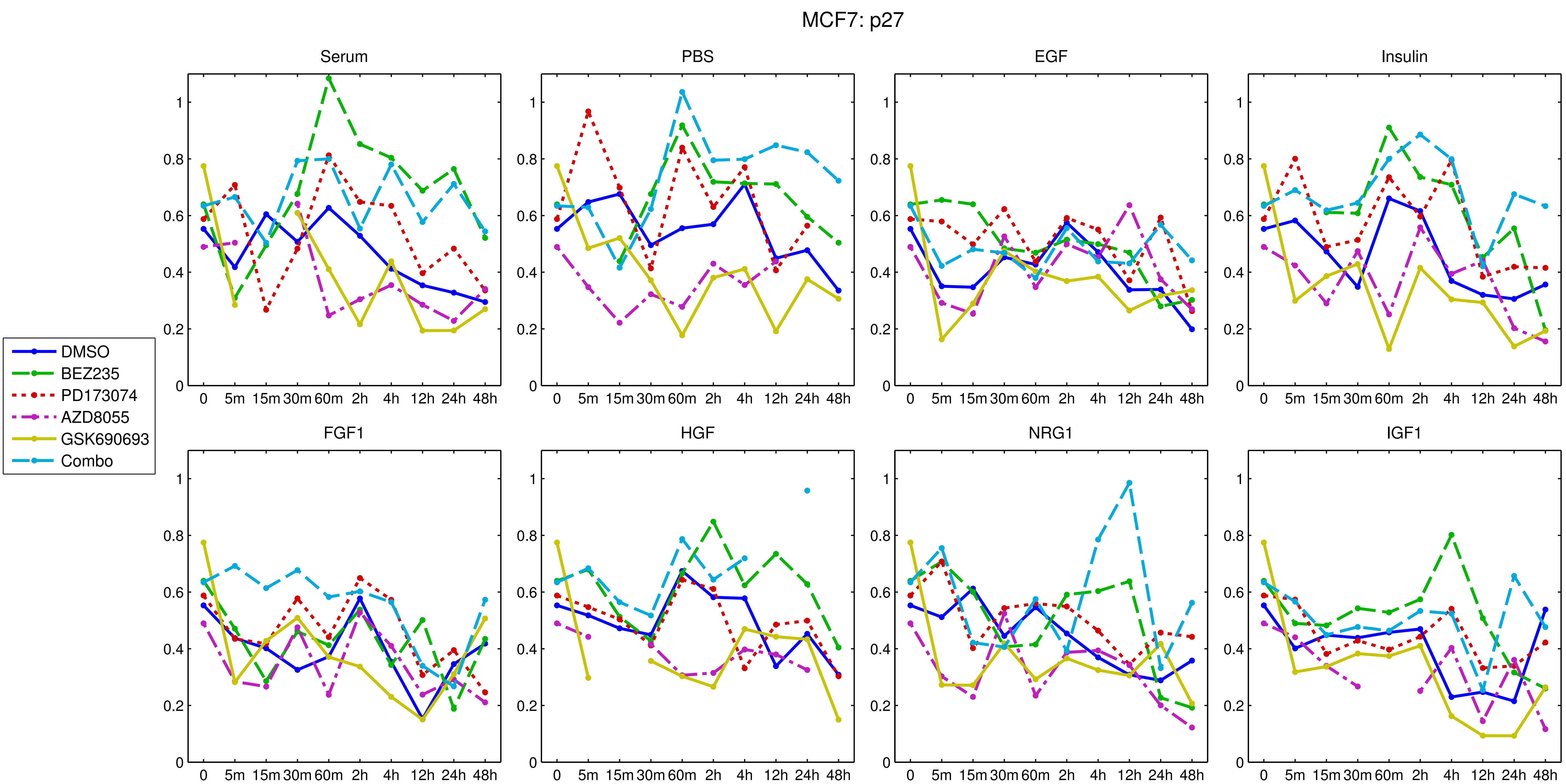

MCF7: p27
Serum
PBS
EGF
Insulin
FGF1
HGF
NRG1
IGF1
DMSO
BEZ235
PD173074
AZD8055
GSK690693
Combo
0
0.2
0.4
0.6
0.8
1
0 5m 15m 30m 60m 2h 4h 12h 24h 48h

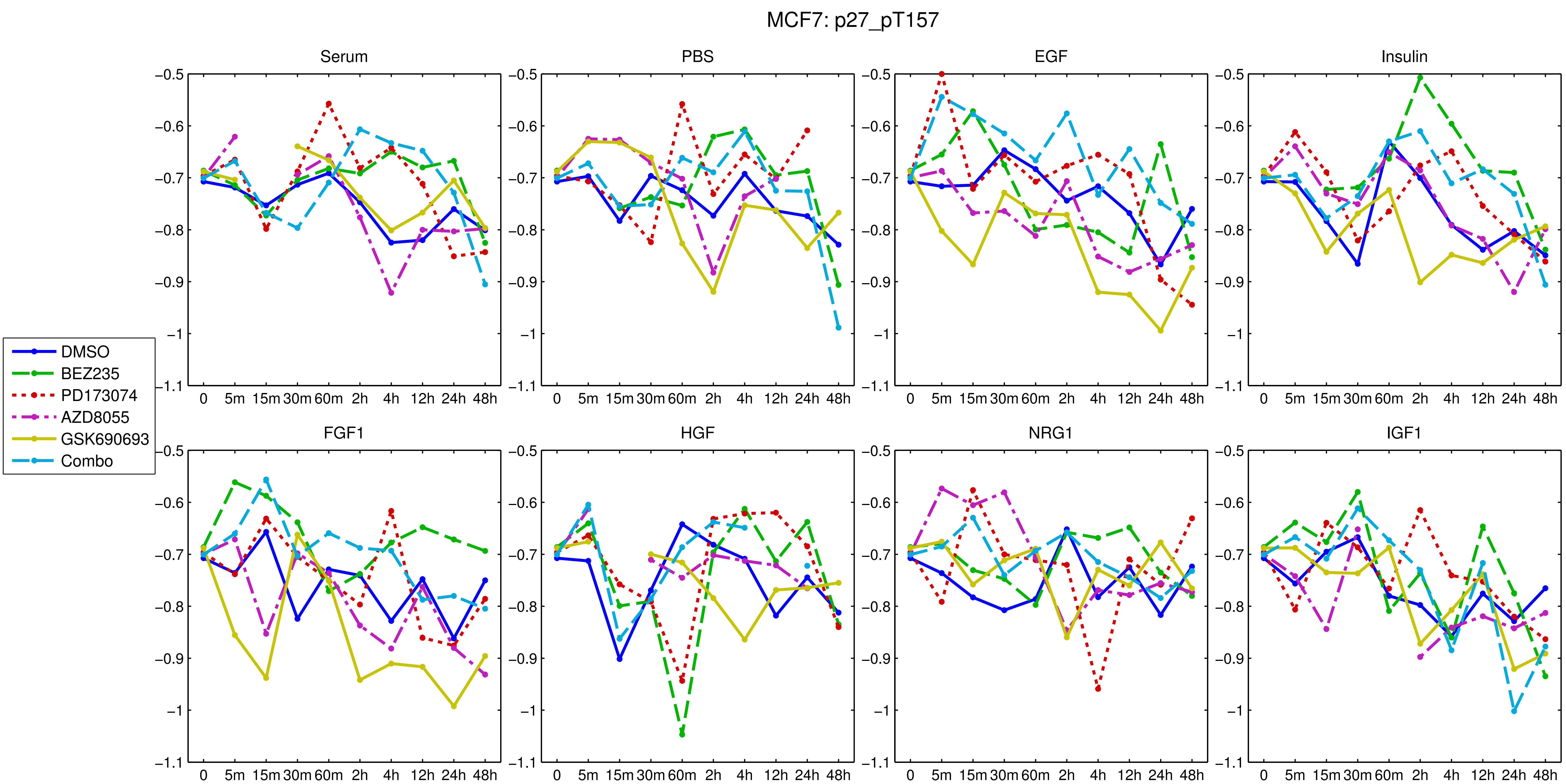
MCF7: p27_pT157
Serum
PBS
EGF
Insulin
FGF1
HGF
NRG1
IGF1
DMSO
BEZ235
PD173074
AZD8055
GSK690693
Combo
−0.5
−0.6
−0.7
−0.8
−0.9
−1
−1.1
0 5m 15m 30m 60m 2h 4h 12h 24h 48h

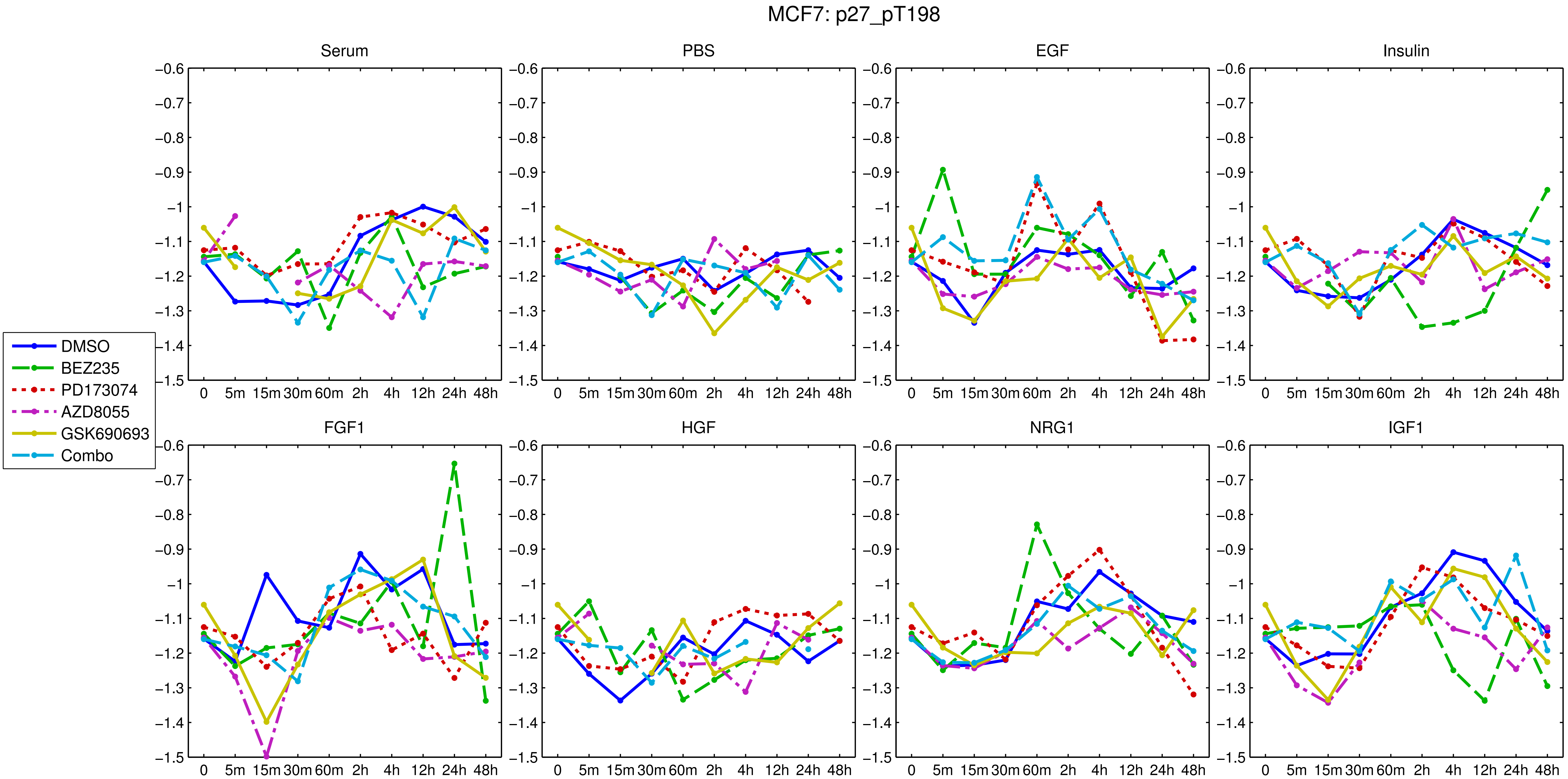
MCF7: p27_pT198
Serum
PBS
EGF
Insulin
FGF1
HGF
NRG1
IGF1
DMSO
BEZ235
PD173074
AZD8055
GSK690693
Combo
−0.6
−0.7
−0.8
−0.9
−1
−1.1
−1.2
−1.3
−1.4
−1.5
0
5m
15m
30m
60m
2h
4h
12h
24h
48h

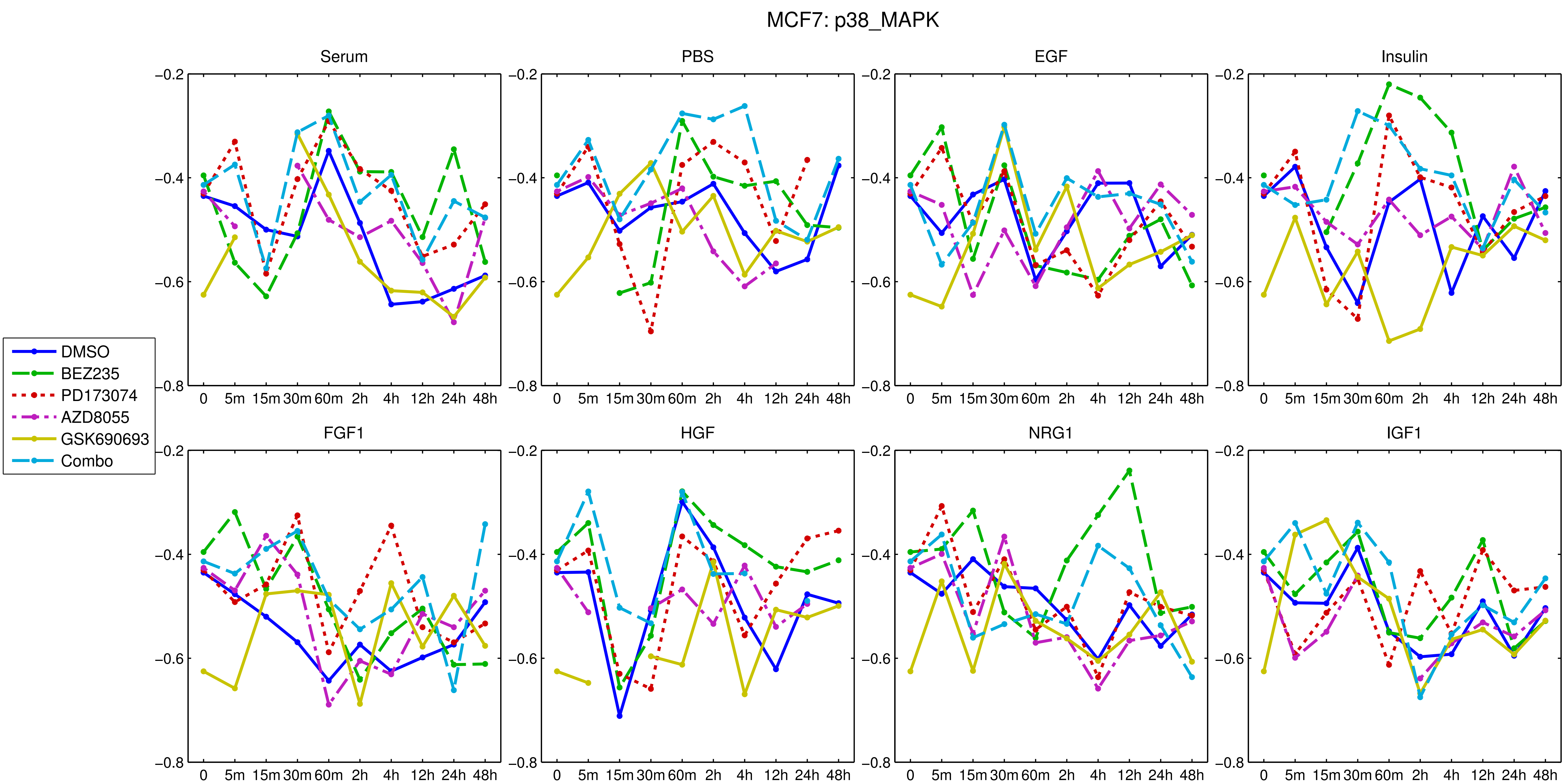

MCF7: p38_MAPK
Serum
PBS
EGF
Insulin
FGF1
HGF
NRG1
IGF1
−0.2
−0.4
−0.6
−0.8
0
5m
15m
30m
60m
2h
4h
12h
24h
48h
DMSO
BEZ235
PD173074
AZD8055
GSK690693
Combo

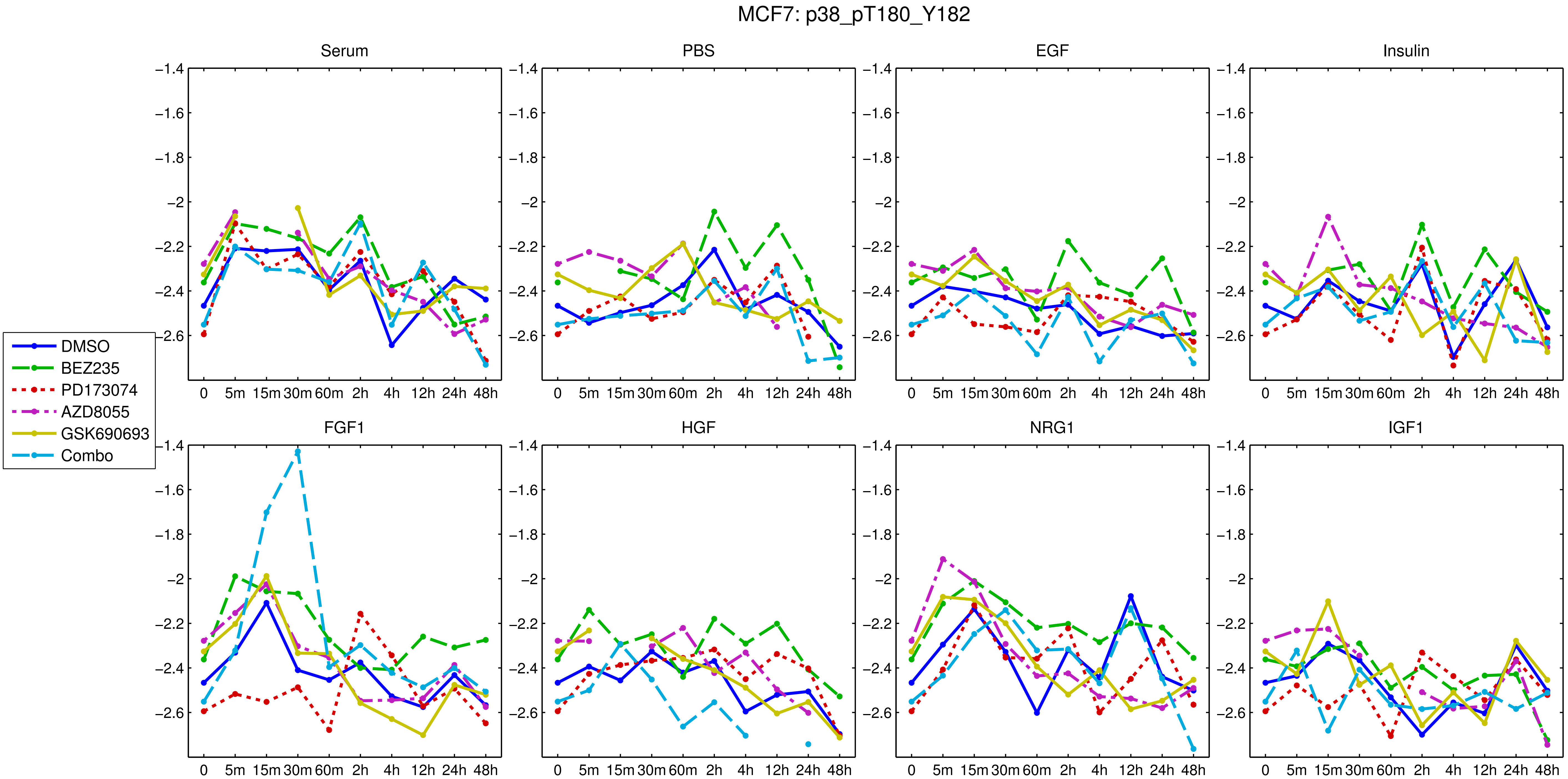
MCF7: p38_pT180_Y182
Serum
PBS
EGF
Insulin
FGF1
HGF
NRG1
IGF1
DMSO
BEZ235
PD173074
AZD8055
GSK690693
Combo
−1.4
−1.6
−1.8
−2
−2.2
−2.4
−2.6
0
5m
15m
30m
60m
2h
4h
12h
24h
48h

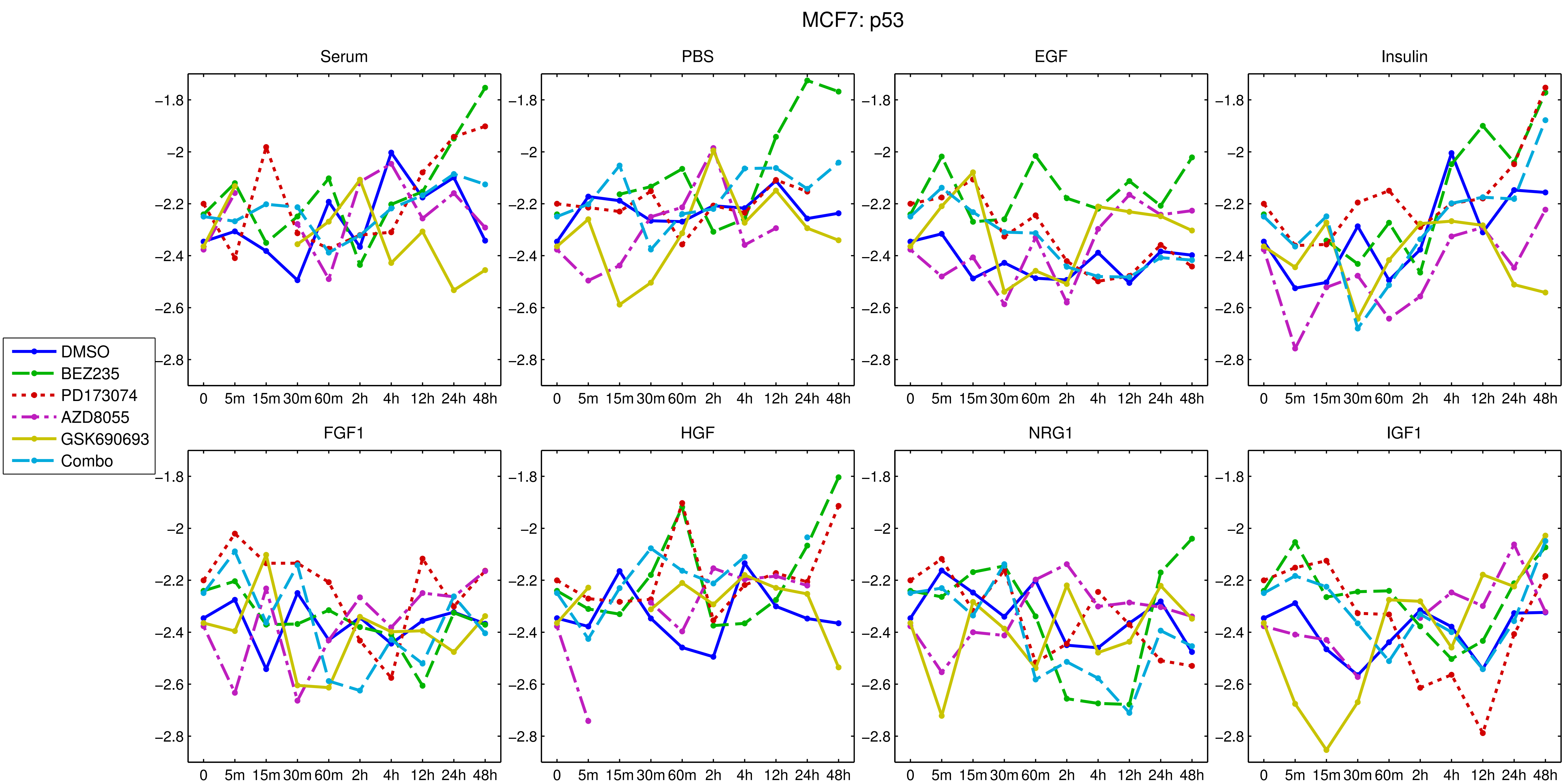

MCF7: p53
Serum
PBS
EGF
Insulin
FGF1
HGF
NRG1
IGF1
DMSO
BEZ235
PD173074
AZD8055
GSK690693
Combo
−1.8
−2
−2.2
−2.4
−2.6
−2.8
0
5m
15m
30m
60m
2h
4h
12h
24h
48h

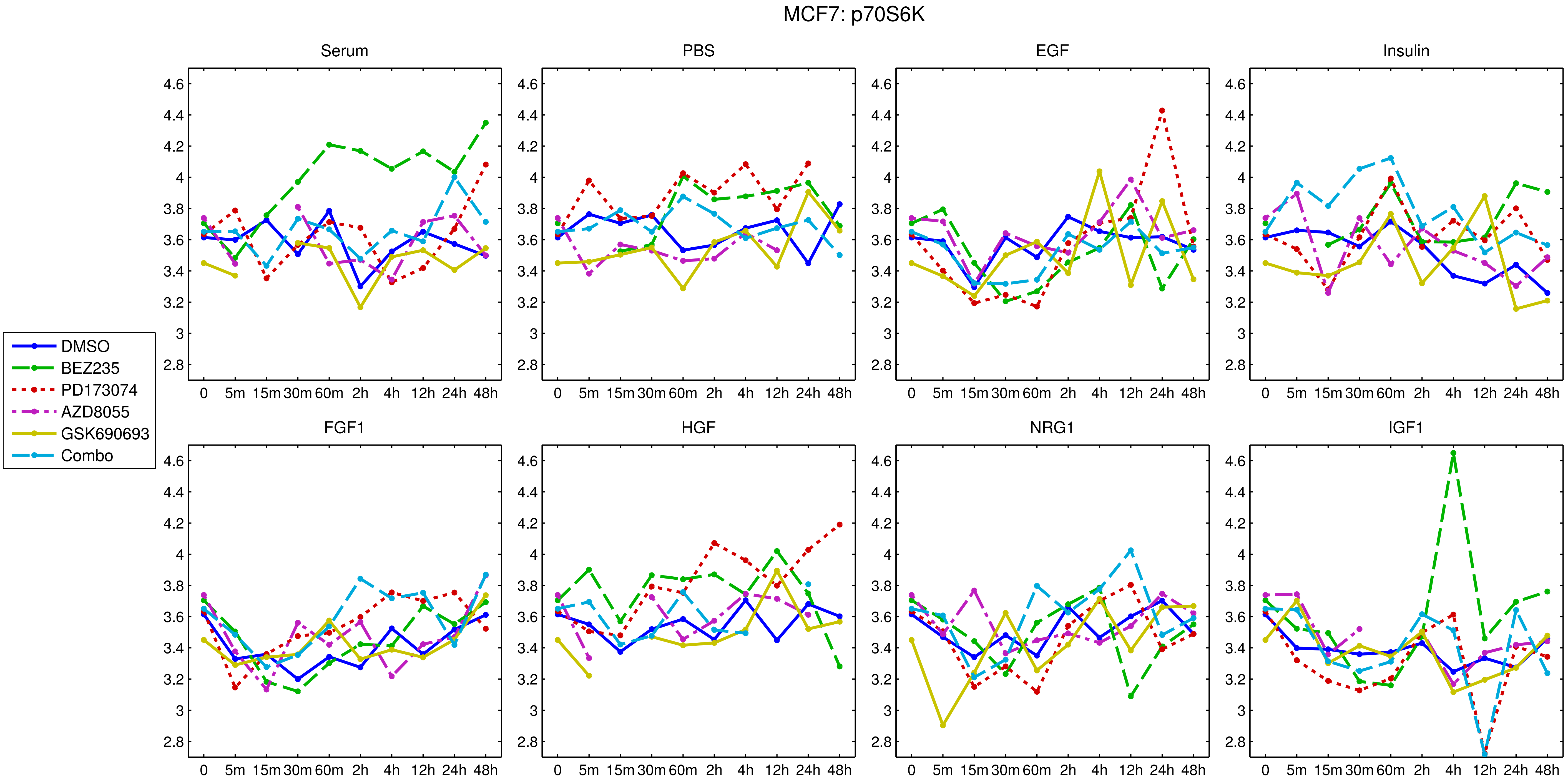

MCF7: p70S6K
Serum
PBS
EGF
Insulin
FGF1
HGF
NRG1
IGF1
DMSO
BEZ235
PD173074
AZD8055
GSK690693
Combo
4.6
4.4
4.2
4
3.8
3.6
3.4
3.2
3
2.8
0
5m
15m
30m
60m
2h
4h
12h
24h
48h

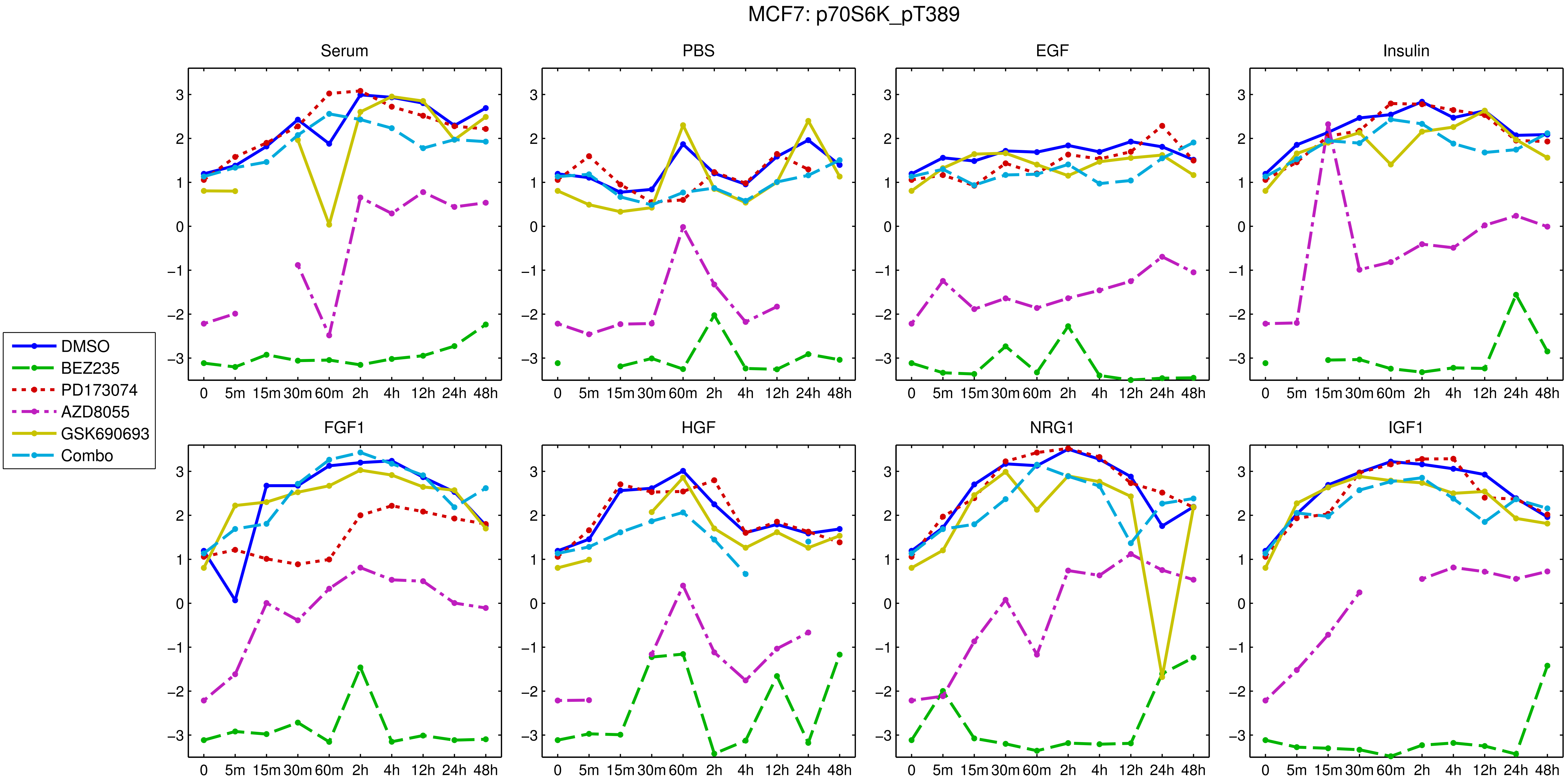

MCF7: p70S6K_pT389
Serum
PBS
EGF
Insulin
FGF1
HGF
NRG1
IGF1
DMSO
BEZ235
PD173074
AZD8055
GSK690693
Combo
3
2
1
0
−1
−2
−3
0
5m
15m
30m
60m
2h
4h
12h
24h
48h

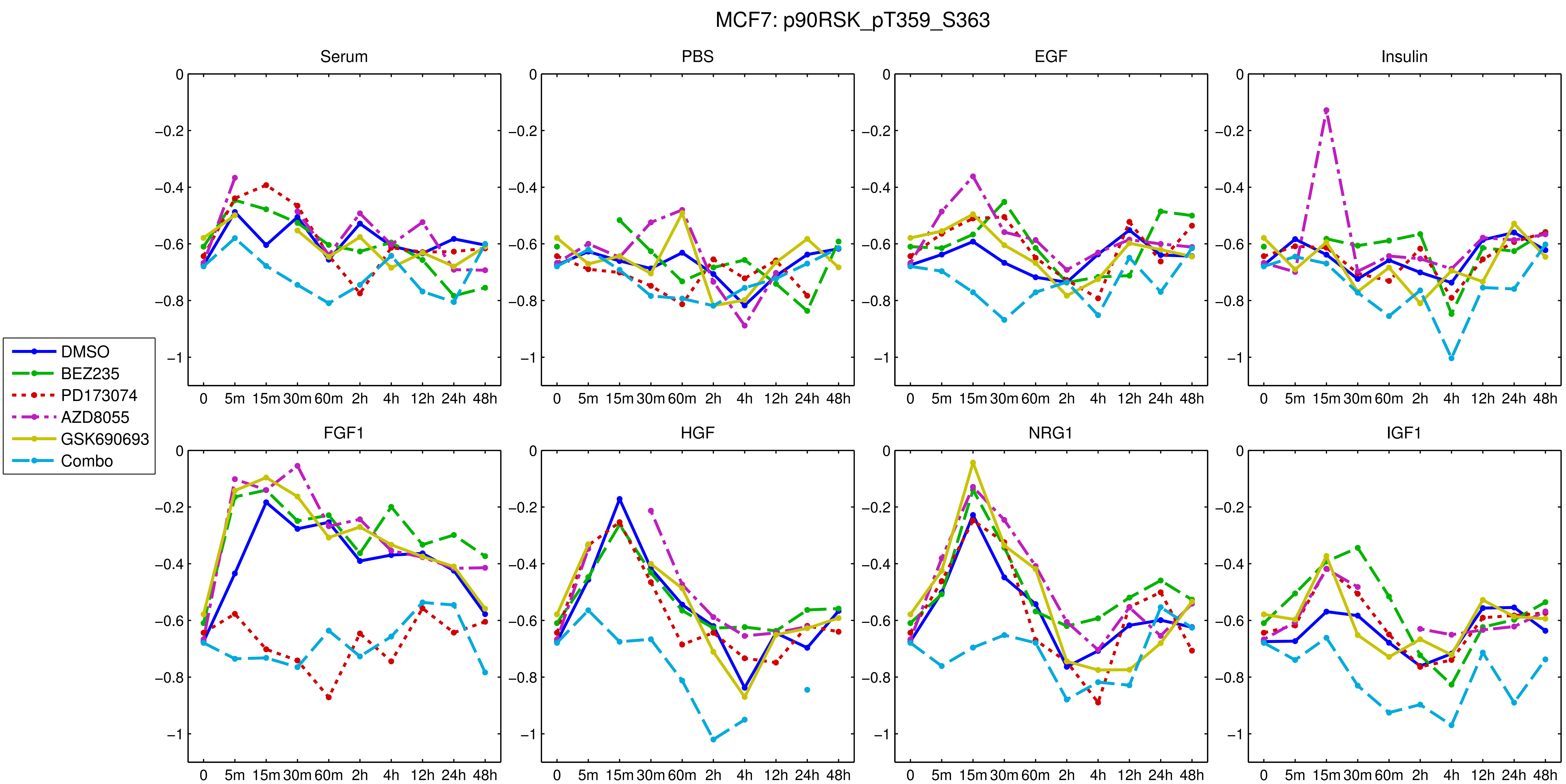
MCF7: p90RSK_pT359_S363
Serum
PBS
EGF
Insulin
FGF1
HGF
NRG1
IGF1
DMSO
BEZ235
PD173074
AZD8055
GSK690693
Combo
0
−0.2
−0.4
−0.6
−0.8
−1
0 5m 15m 30m 60m 2h 4h 12h 24h 48h

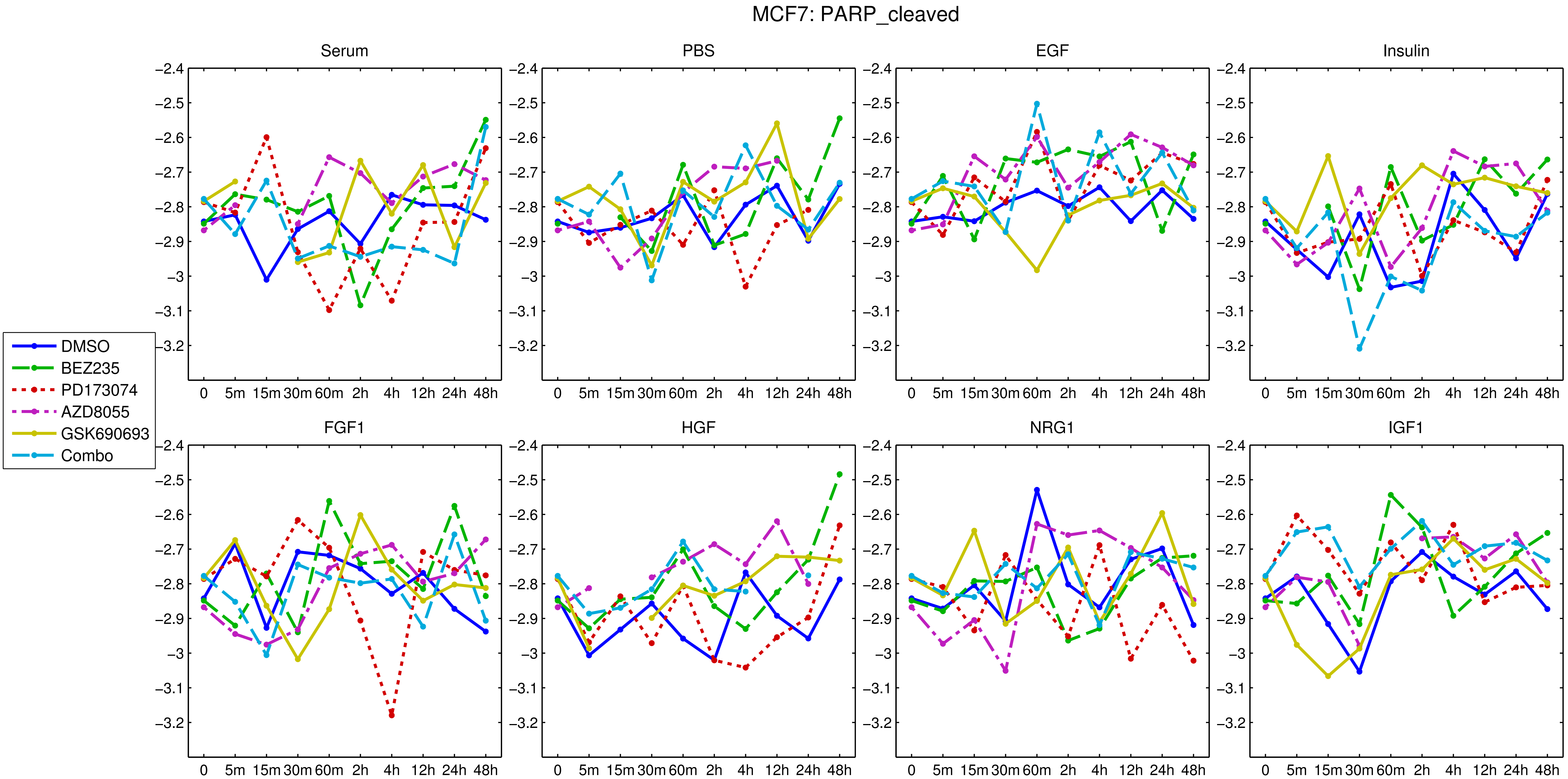
MCF7: PARP_cleaved
Serum
PBS
EGF
Insulin
FGF1
HGF
NRG1
IGF1
DMSO
BEZ235
PD173074
AZD8055
GSK690693
Combo
−2.4
−2.5
−2.6
−2.7
−2.8
−2.9
−3
−3.1
−3.2
0
5m
15m
30m
60m
2h
4h
12h
24h
48h

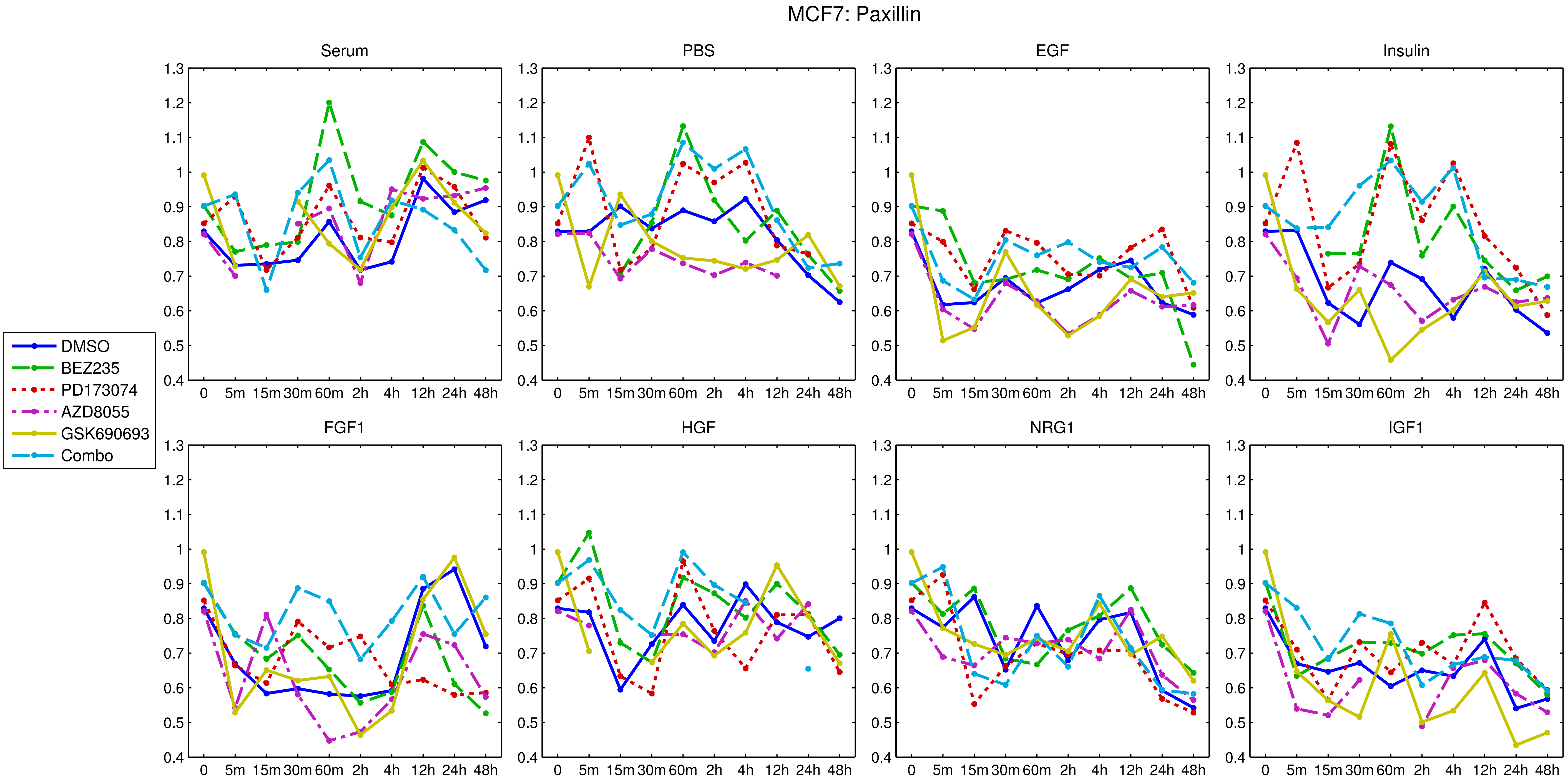
MCF7: Paxillin
Serum
PBS
EGF
Insulin
FGF1
HGF
NRG1
IGF1
DMSO
BEZ235
PD173074
AZD8055
GSK690693
Combo
0 5m 15m 30m 60m 2h 4h 12h 24h 48h
0.4 0.5 0.6 0.7 0.8 0.9 1 1.1 1.2 1.3

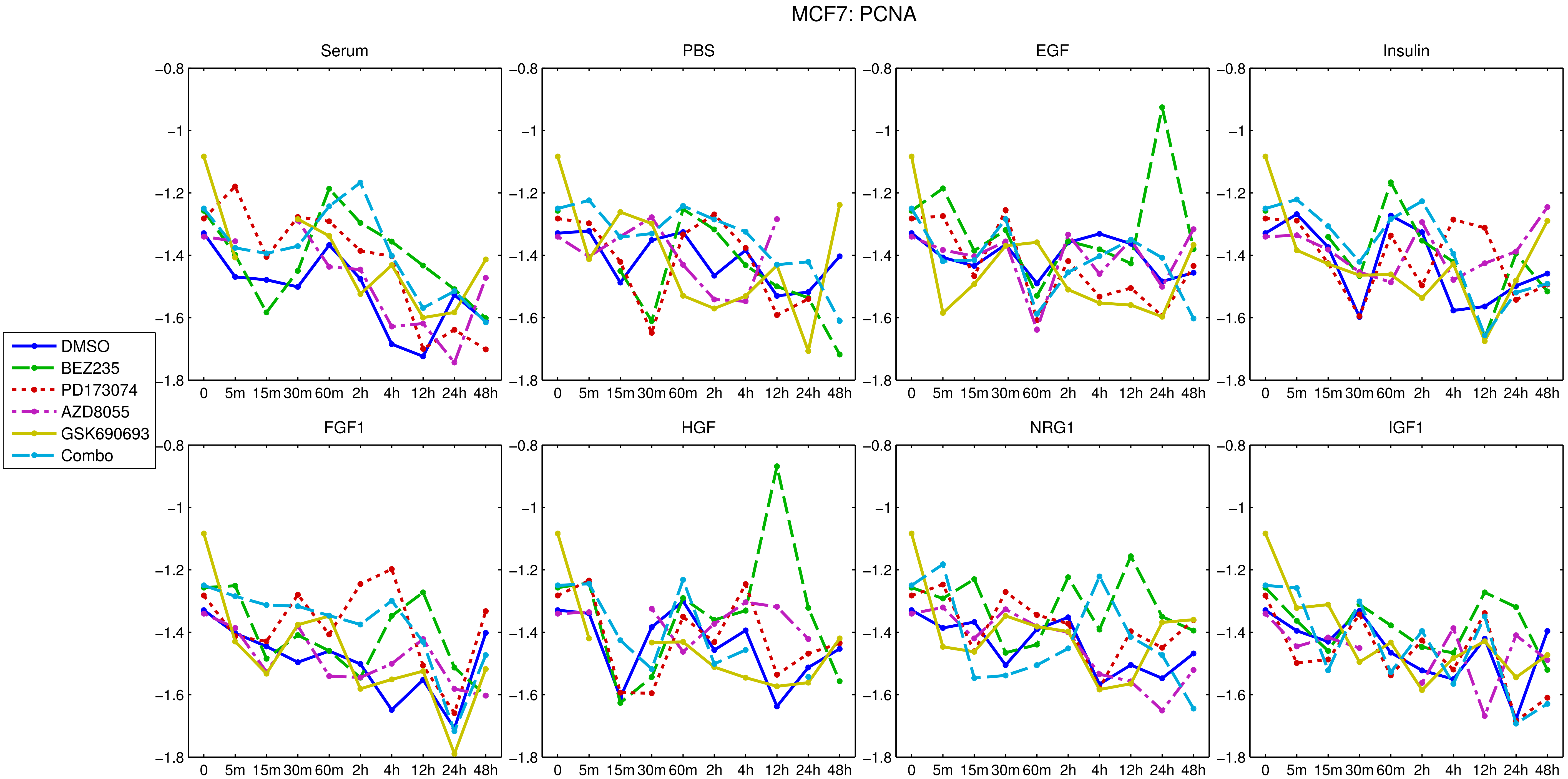

MCF7: PCNA
Serum
PBS
EGF
Insulin
FGF1
HGF
NRG1
IGF1
−0.8
−1
−1.2
−1.4
−1.6
−1.8
0 5m 15m 30m 60m 2h 4h 12h 24h 48h
DMSO
BEZ235
PD173074
AZD8055
GSK690693
Combo

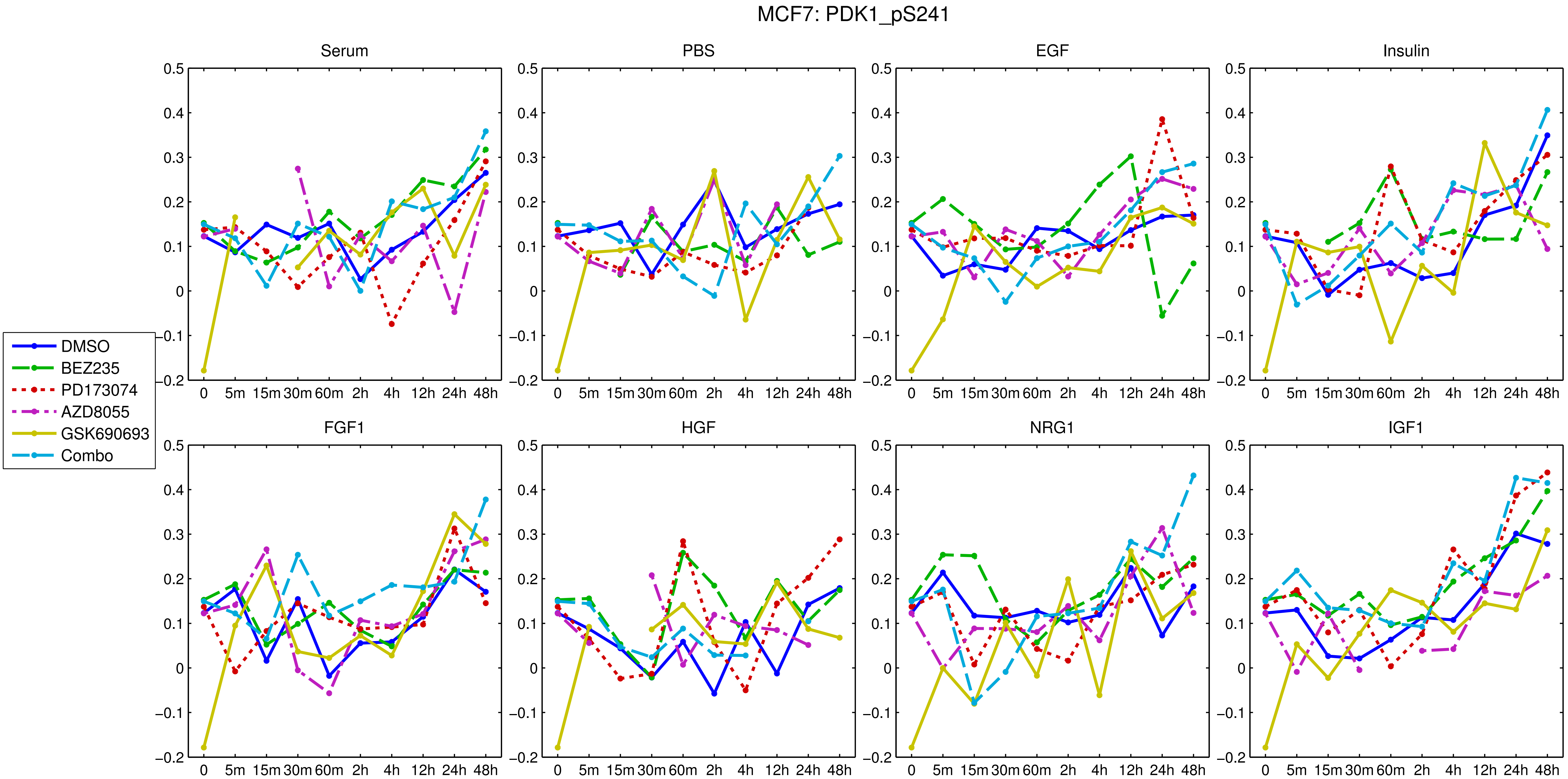

MCF7: PDK1_pS241
Serum
PBS
EGF
Insulin
FGF1
HGF
NRG1
IGF1
DMSO
BEZ235
PD173074
AZD8055
GSK690693
Combo
0.5
0.4
0.3
0.2
0.1
0
−0.1
−0.2
0 5m 15m 30m 60m 2h 4h 12h 24h 48h

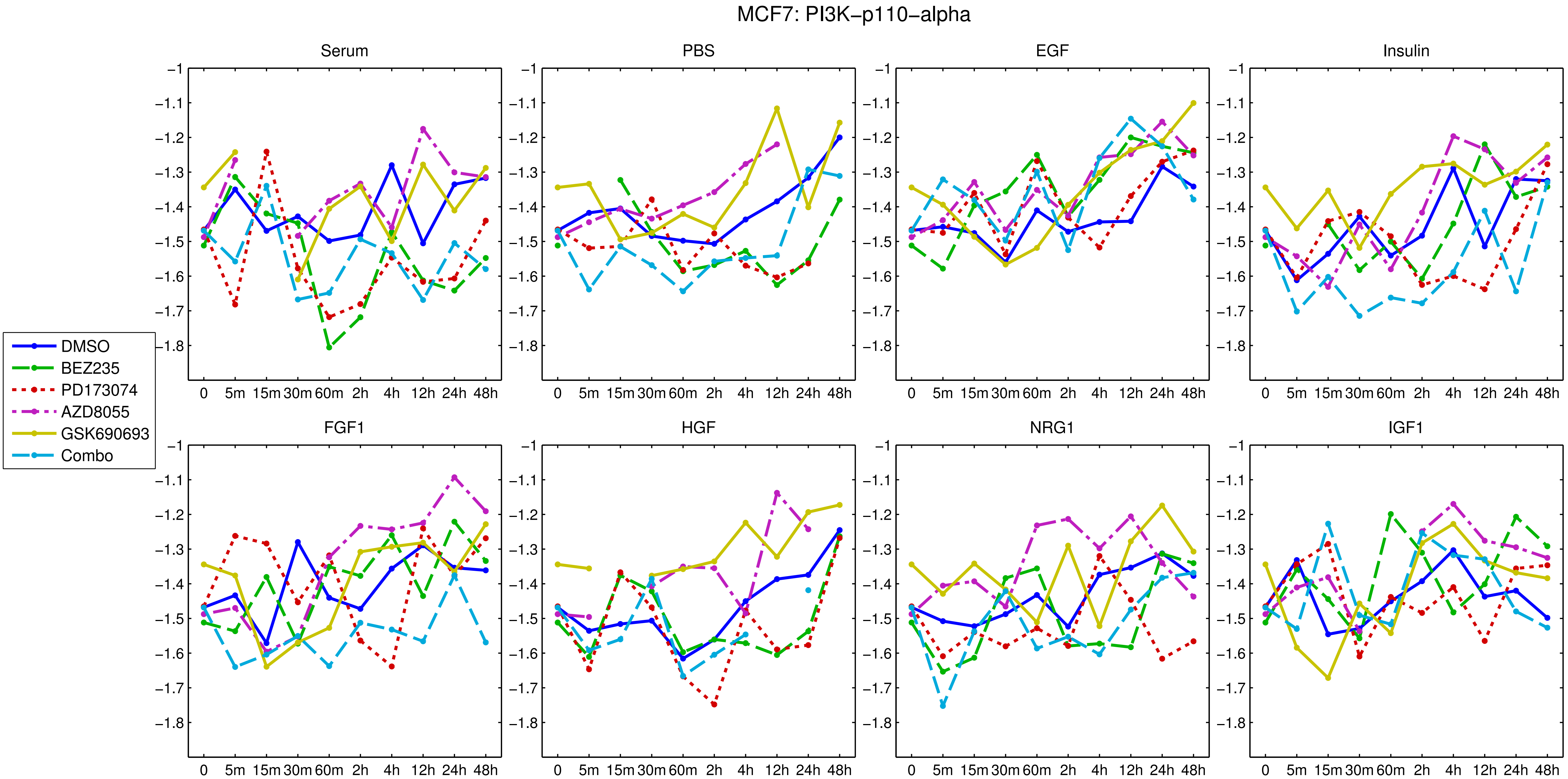

MCF7: PI3K−p110−alpha
Serum
PBS
EGF
Insulin
FGF1
HGF
NRG1
IGF1
DMSO
BEZ235
PD173074
AZD8055
GSK690693
Combo
−1
−1.1
−1.2
−1.3
−1.4
−1.5
−1.6
−1.7
−1.8
0
5m
15m
30m
60m
2h
4h
12h
24h
48h

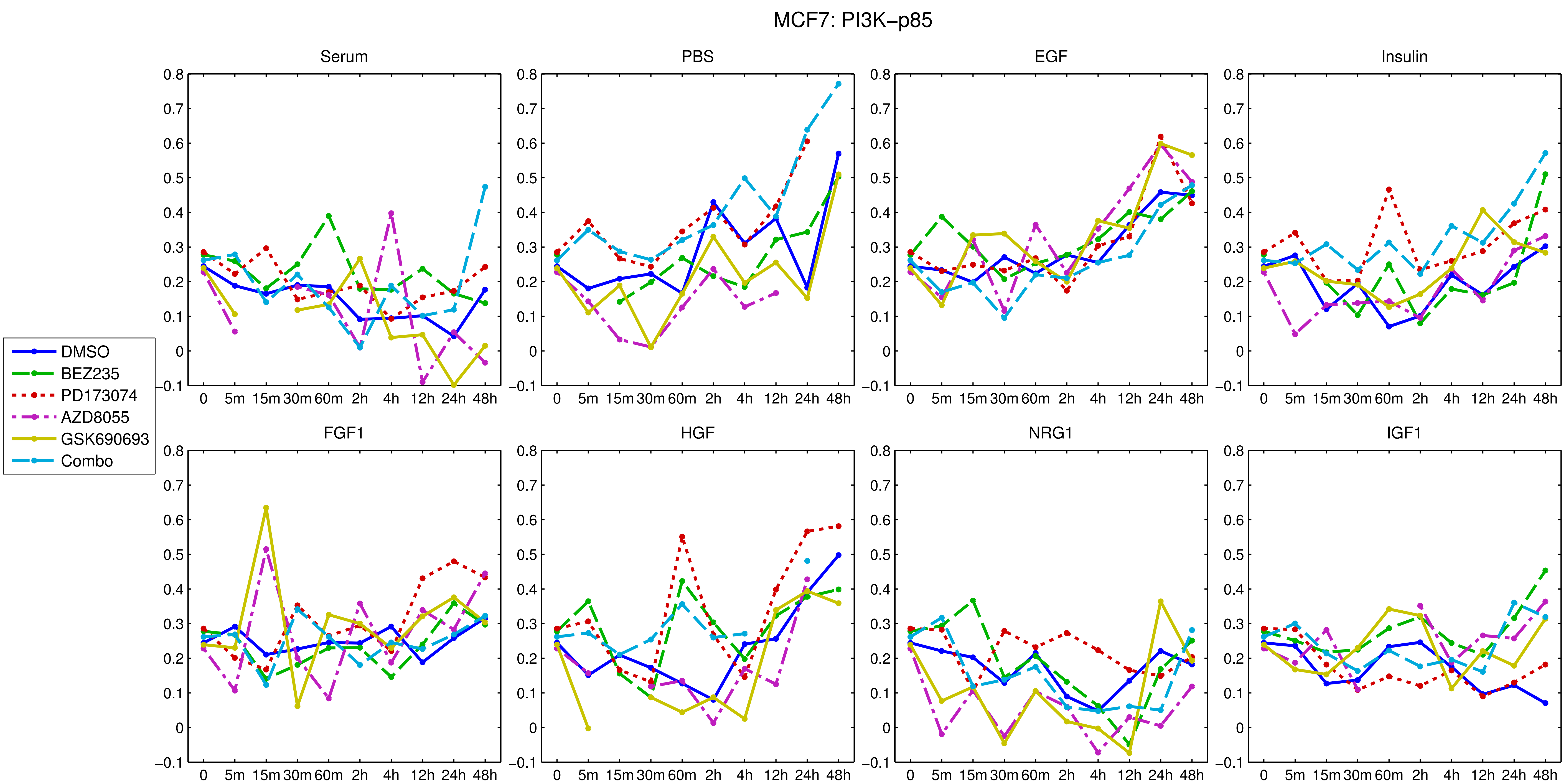
MCF7: PI3K–p85
Serum
PBS
EGF
Insulin
FGF1
HGF
NRG1
IGF1
0.8
0.7
0.6
0.5
0.4
0.3
0.2
0.1
0
−0.1
0 5m 15m 30m 60m 2h 4h 12h 24h 48h
DMSO
BEZ235
PD173074
AZD8055
GSK690693
Combo

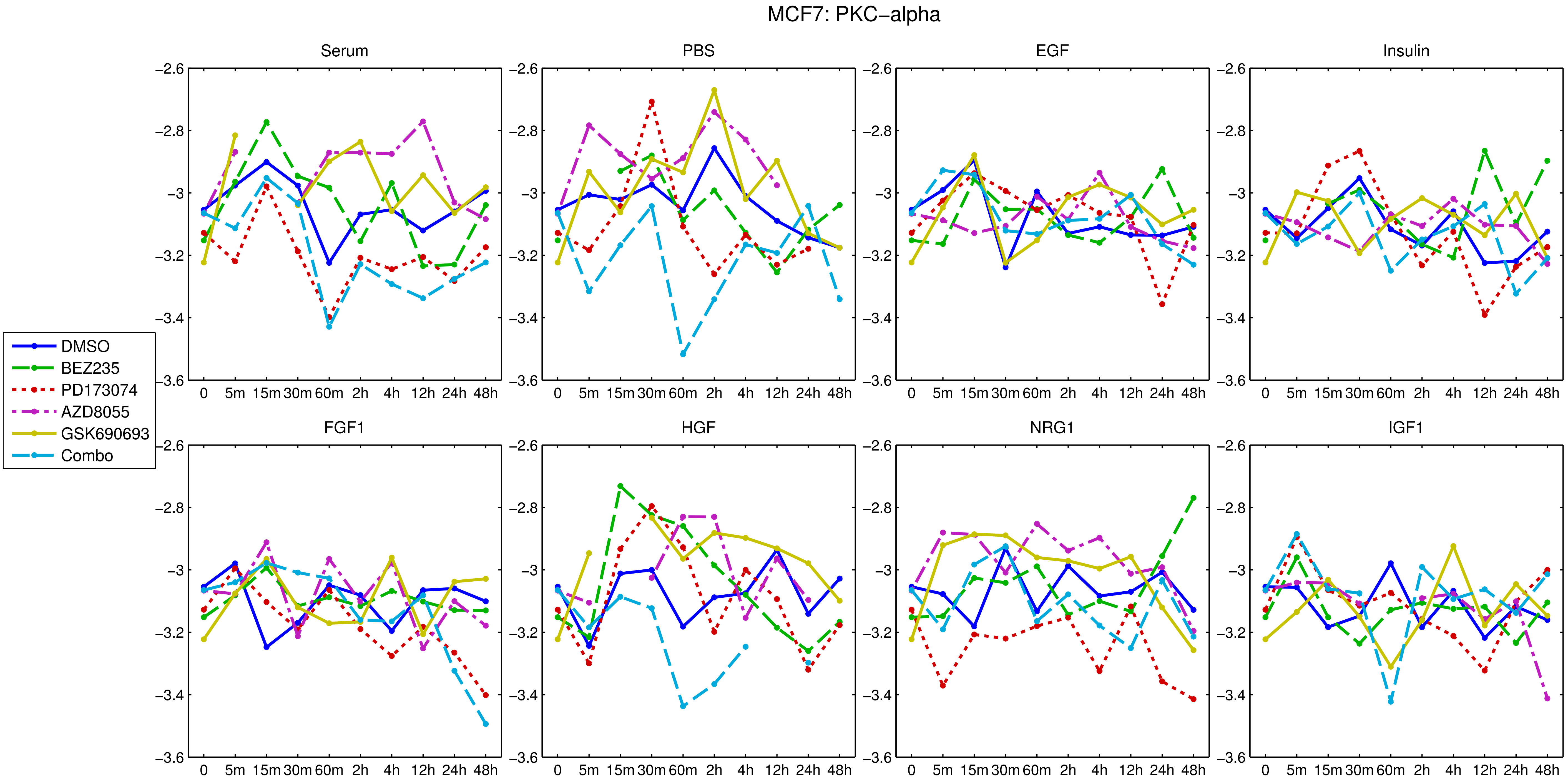
MCF7: PKC−alpha
Serum
PBS
EGF
Insulin
FGF1
HGF
NRG1
IGF1
−2.6
−2.8
−3
−3.2
−3.4
−3.6
0
5m
15m
30m
60m
2h
4h
12h
24h
48h
DMSO
BEZ235
PD173074
AZD8055
GSK690693
Combo

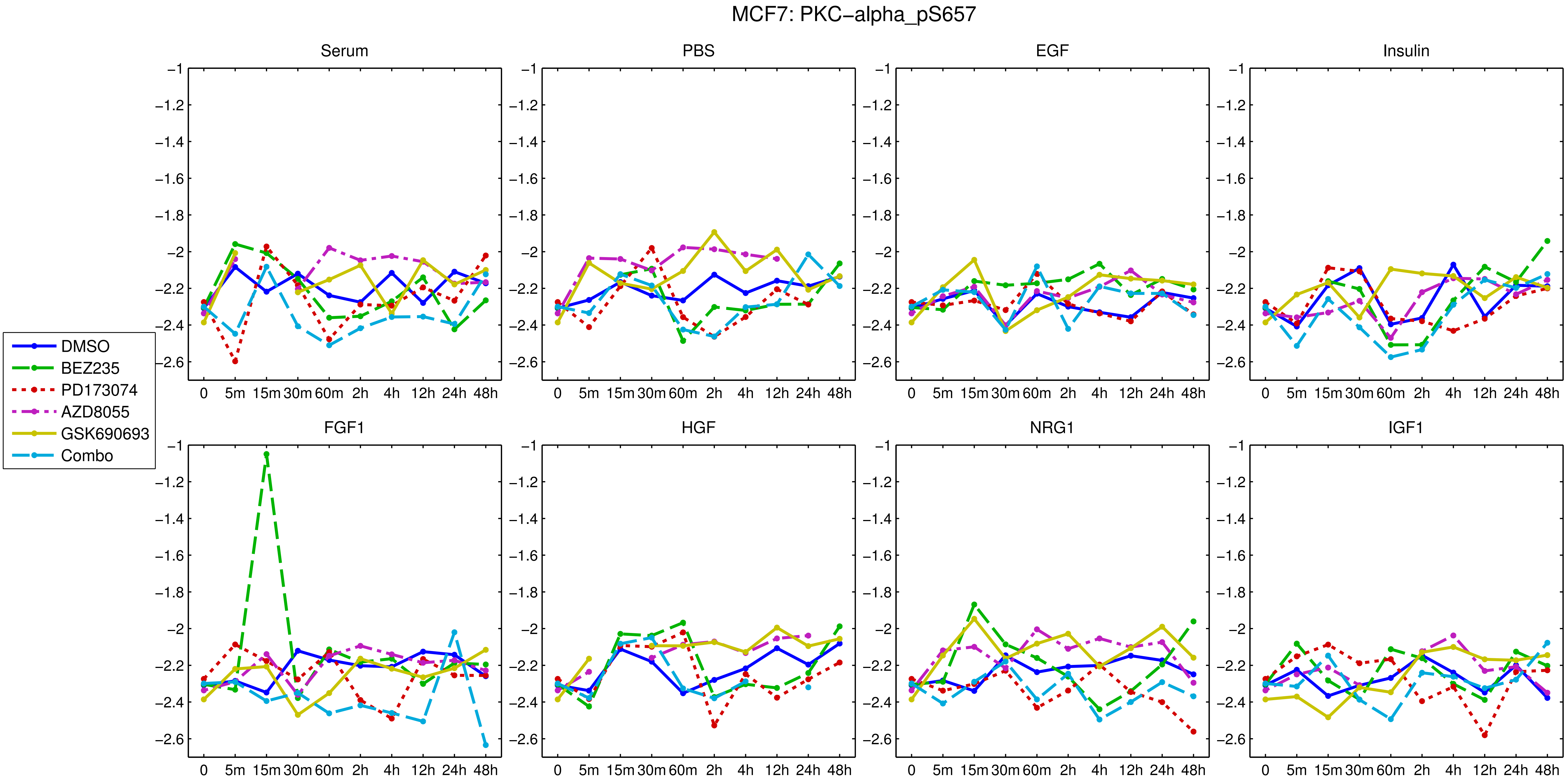

MCF7: PKC−alpha_pS657
Serum
PBS
EGF
Insulin
FGF1
HGF
NRG1
IGF1
DMSO
BEZ235
PD173074
AZD8055
GSK690693
Combo
−1
−1.2
−1.4
−1.6
−1.8
−2
−2.2
−2.4
−2.6
0 5m 15m 30m 60m 2h 4h 12h 24h 48h

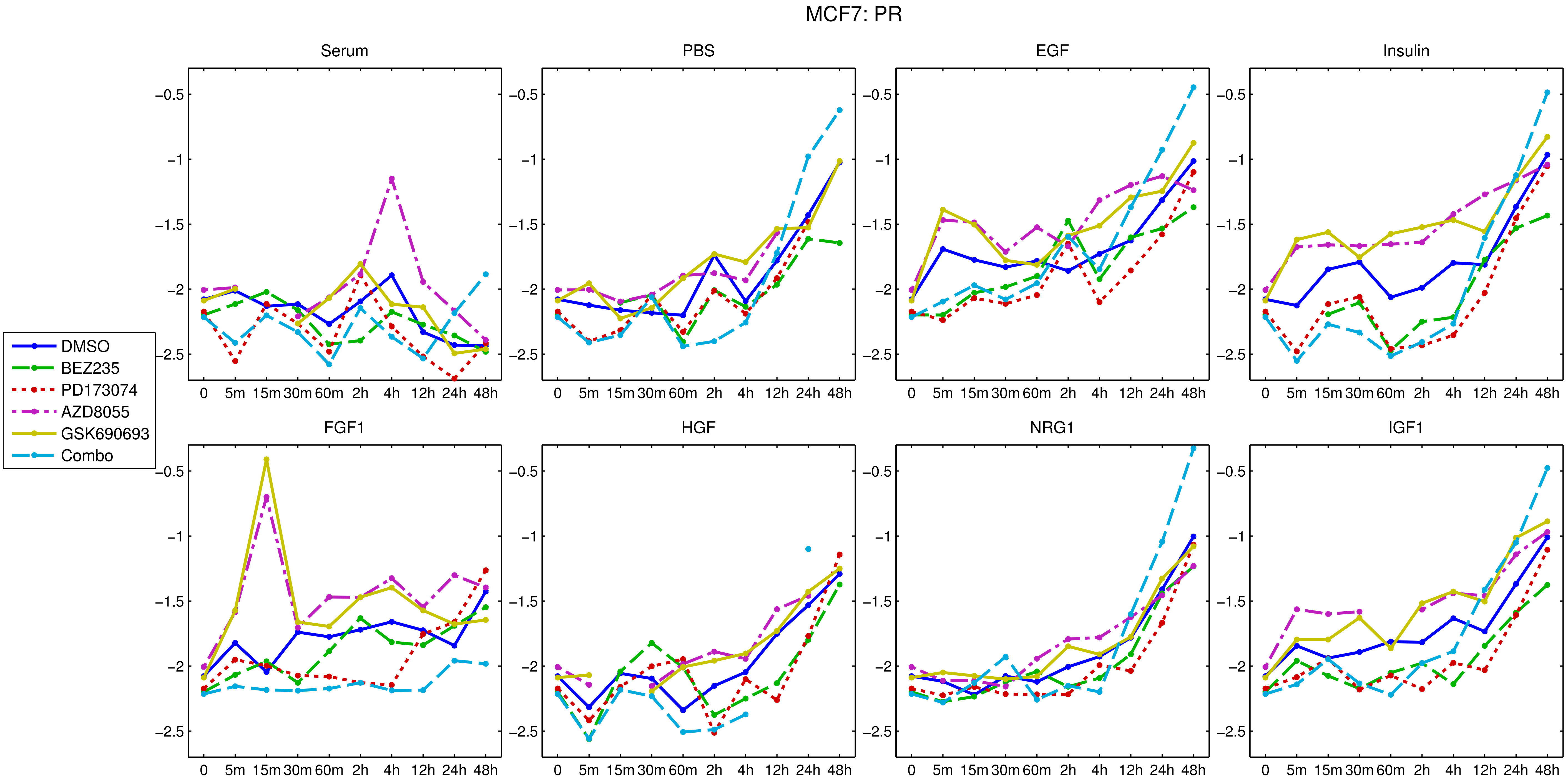
MCF7: PR
Serum
PBS
EGF
Insulin
FGF1
HGF
NRG1
IGF1
−0.5
−1
−1.5
−2
−2.5
0
5m
15m
30m
60m
2h
4h
12h
24h
48h
DMSO
BEZ235
PD173074
AZD8055
GSK690693
Combo

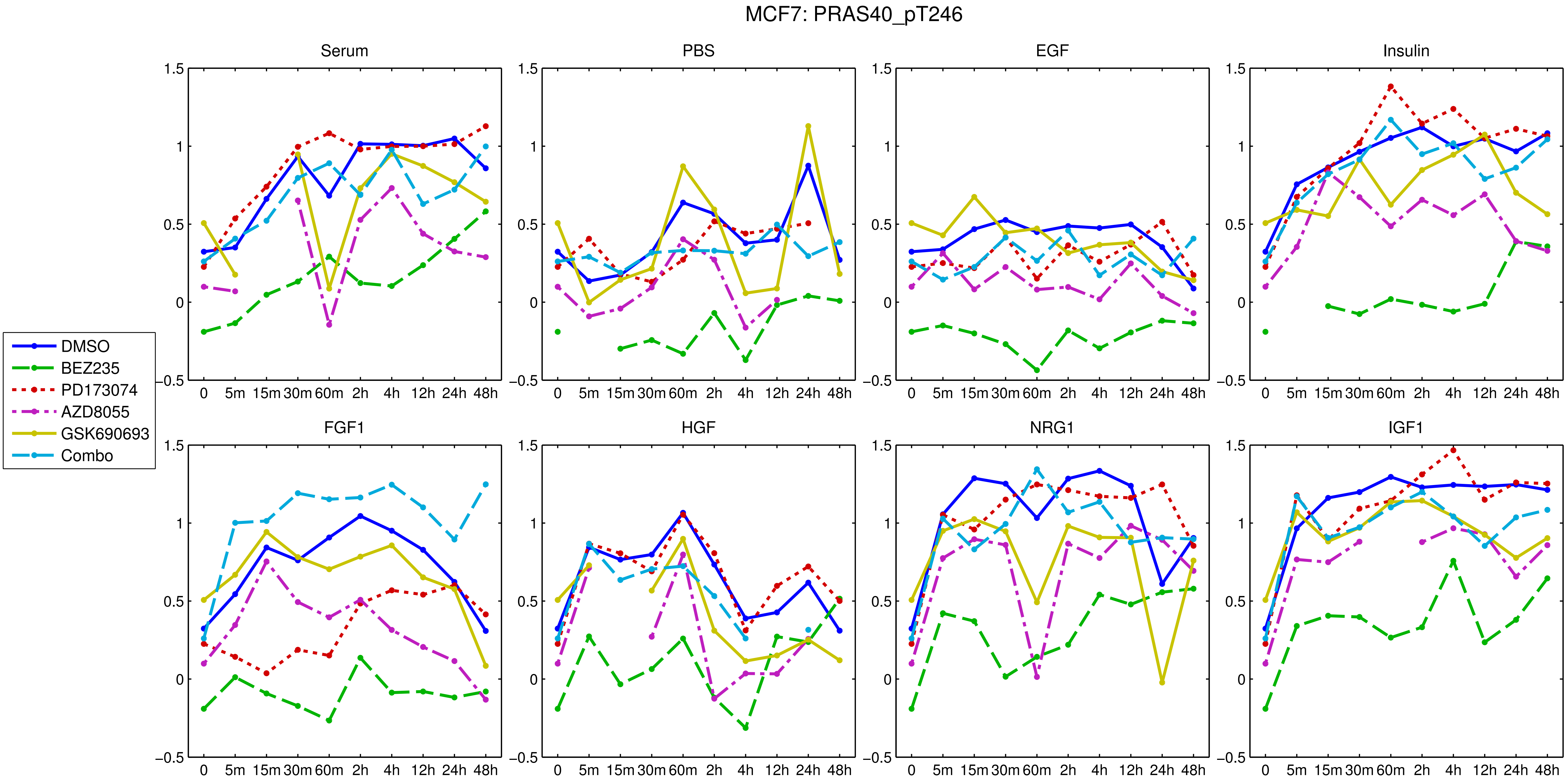
MCF7: PRAS40_pT246
Serum
PBS
EGF
Insulin
FGF1
HGF
NRG1
IGF1
DMSO
BEZ235
PD173074
AZD8055
GSK690693
Combo
1.5
1
0.5
0
−0.5
0
5m
15m
30m
60m
2h
4h
12h
24h
48h

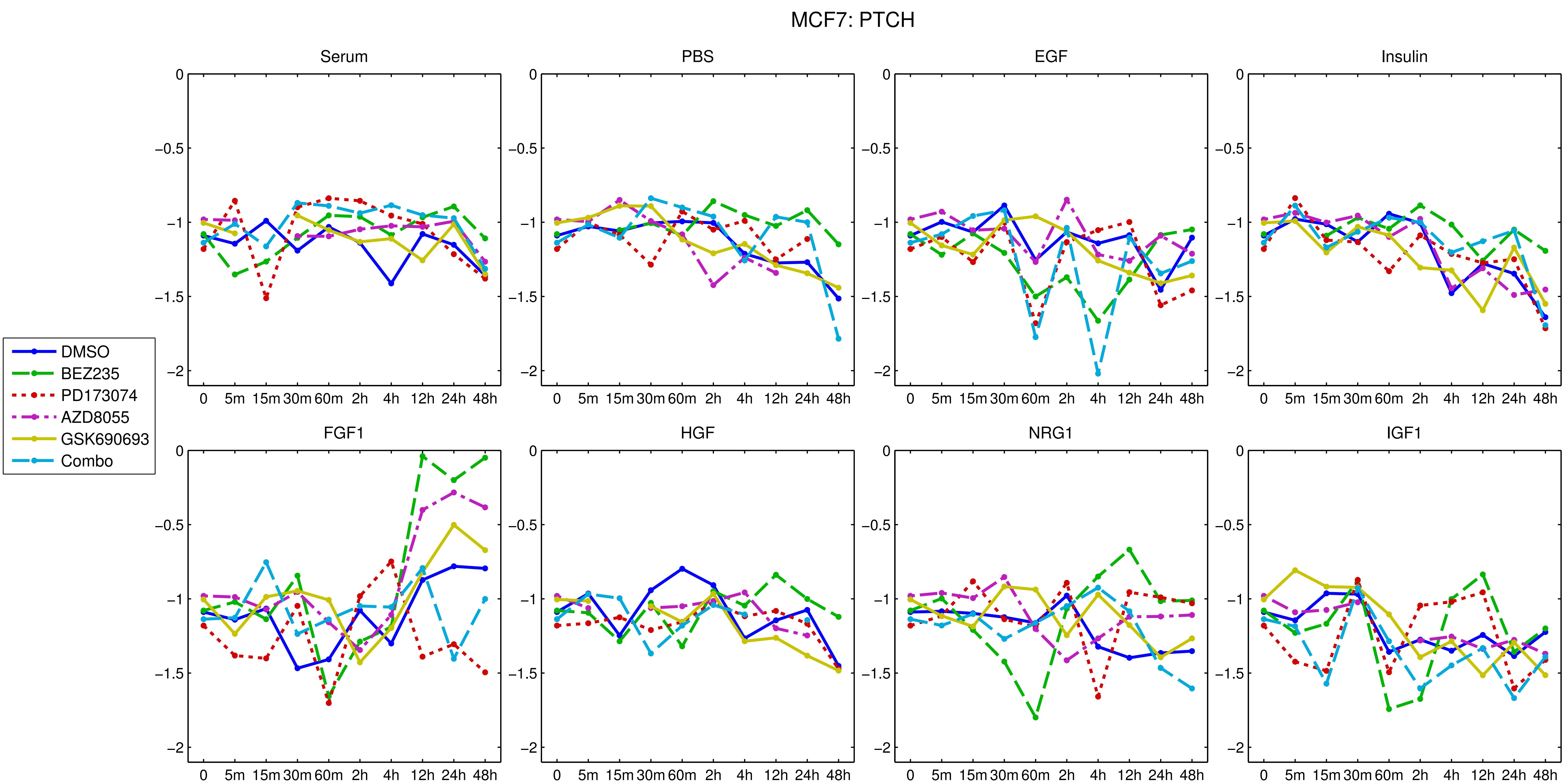

MCF7: PTCH
Serum
PBS
EGF
Insulin
FGF1
HGF
NRG1
IGF1
0
−0.5
−1
−1.5
−2
0
5m
15m
30m
60m
2h
4h
12h
24h
48h
DMSO
BEZ235
PD173074
AZD8055
GSK690693
Combo

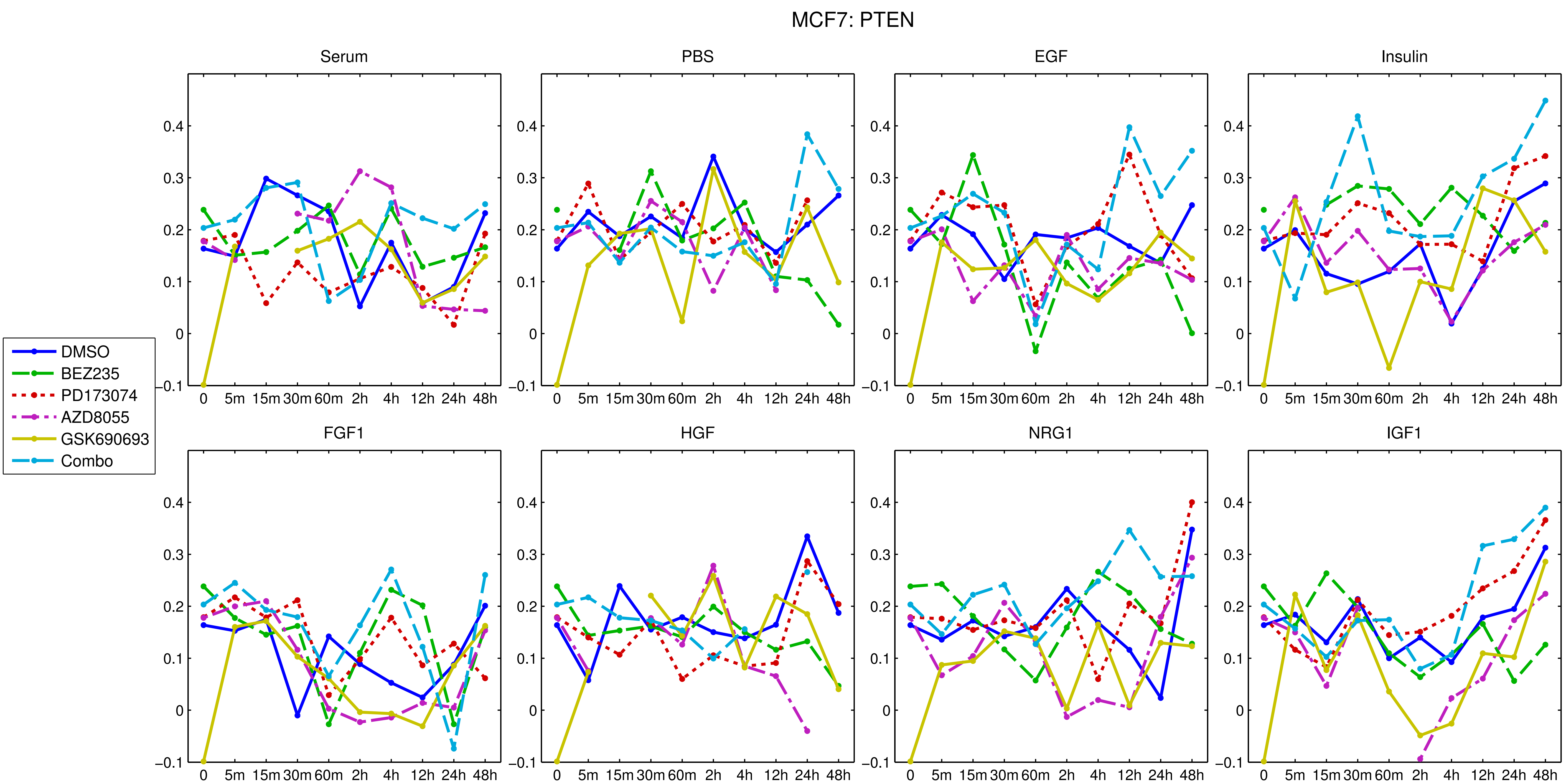
MCF7: PTEN
Serum
PBS
EGF
Insulin
FGF1
HGF
NRG1
IGF1
DMSO
BEZ235
PD173074
AZD8055
GSK690693
Combo
0.4
0.3
0.2
0.1
0
−0.1
0
5m
15m
30m
60m
2h
4h
12h
24h
48h

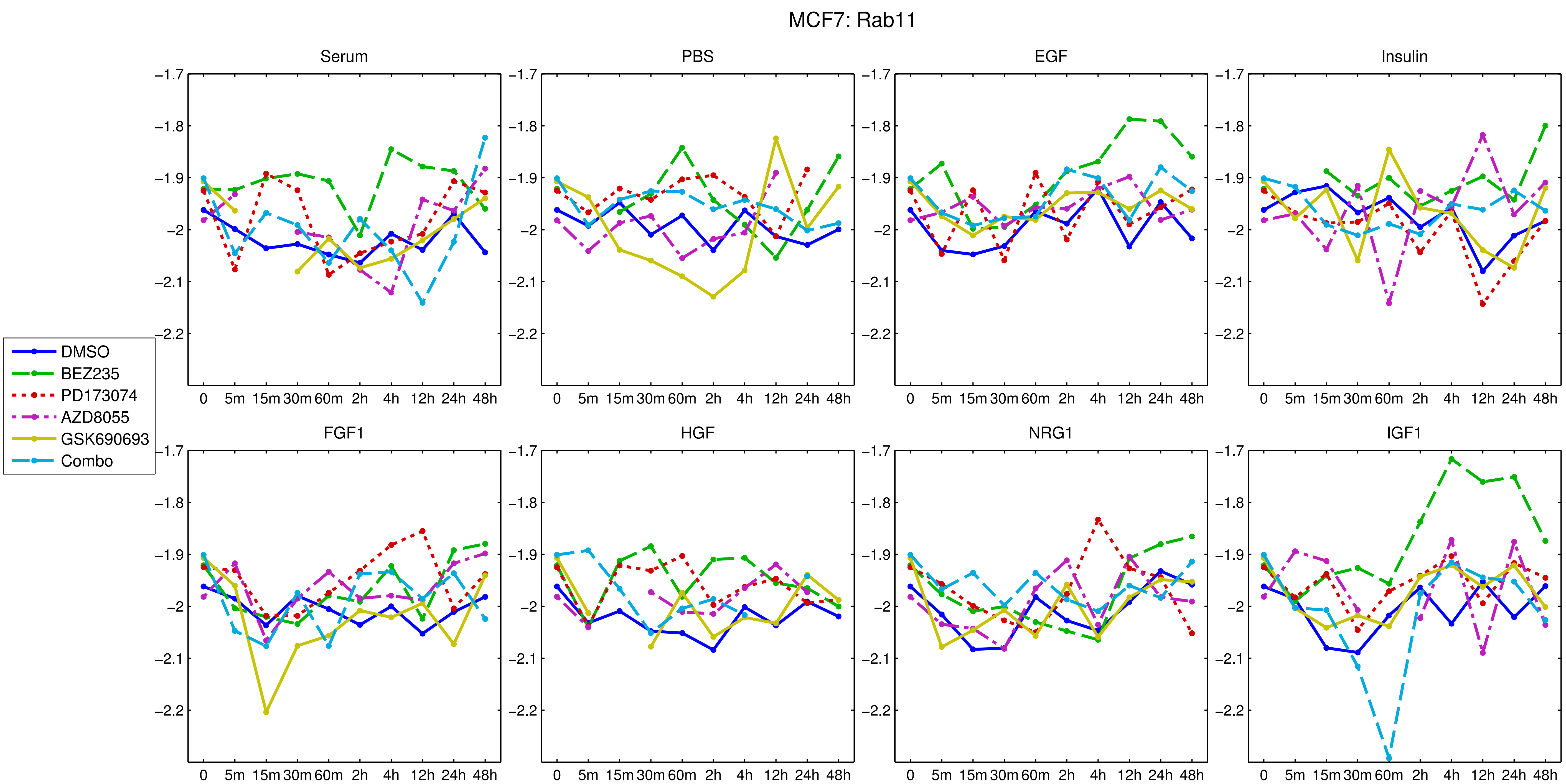
MCF7: Rab11
Serum
PBS
EGF
Insulin
FGF1
HGF
NRG1
IGF1
DMSO
BEZ235
PD173074
AZD8055
GSK690693
Combo
−1.7
−1.8
−1.9
−2
−2.1
−2.2
0
5m
15m
30m
60m
2h
4h
12h
24h
48h

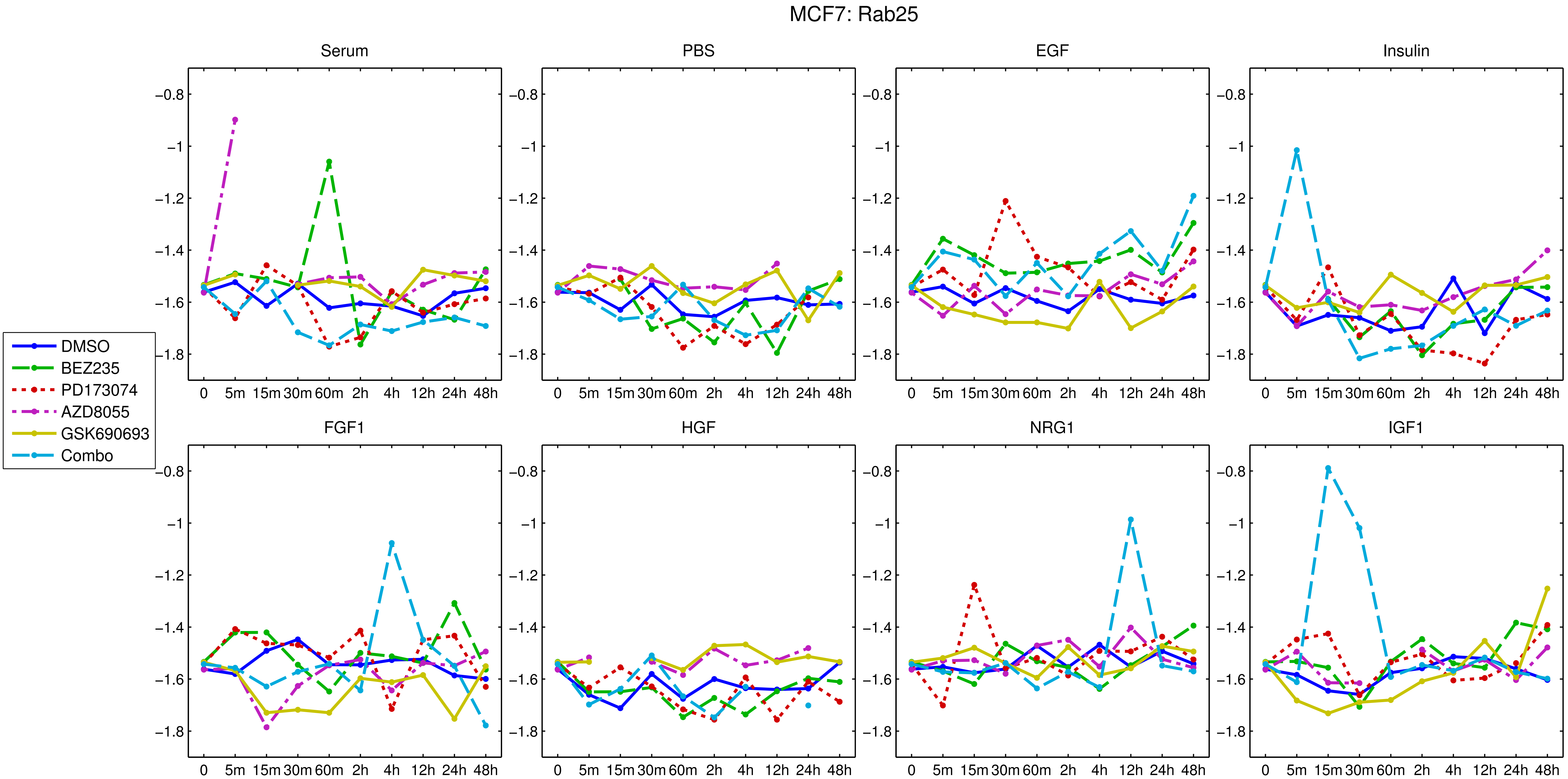
MCF7: Rab25
Serum
PBS
EGF
Insulin
FGF1
HGF
NRG1
IGF1
DMSO
BEZ235
PD173074
AZD8055
GSK690693
Combo
−0.8
−1
−1.2
−1.4
−1.6
−1.8
0
5m
15m
30m
60m
2h
4h
12h
24h
48h

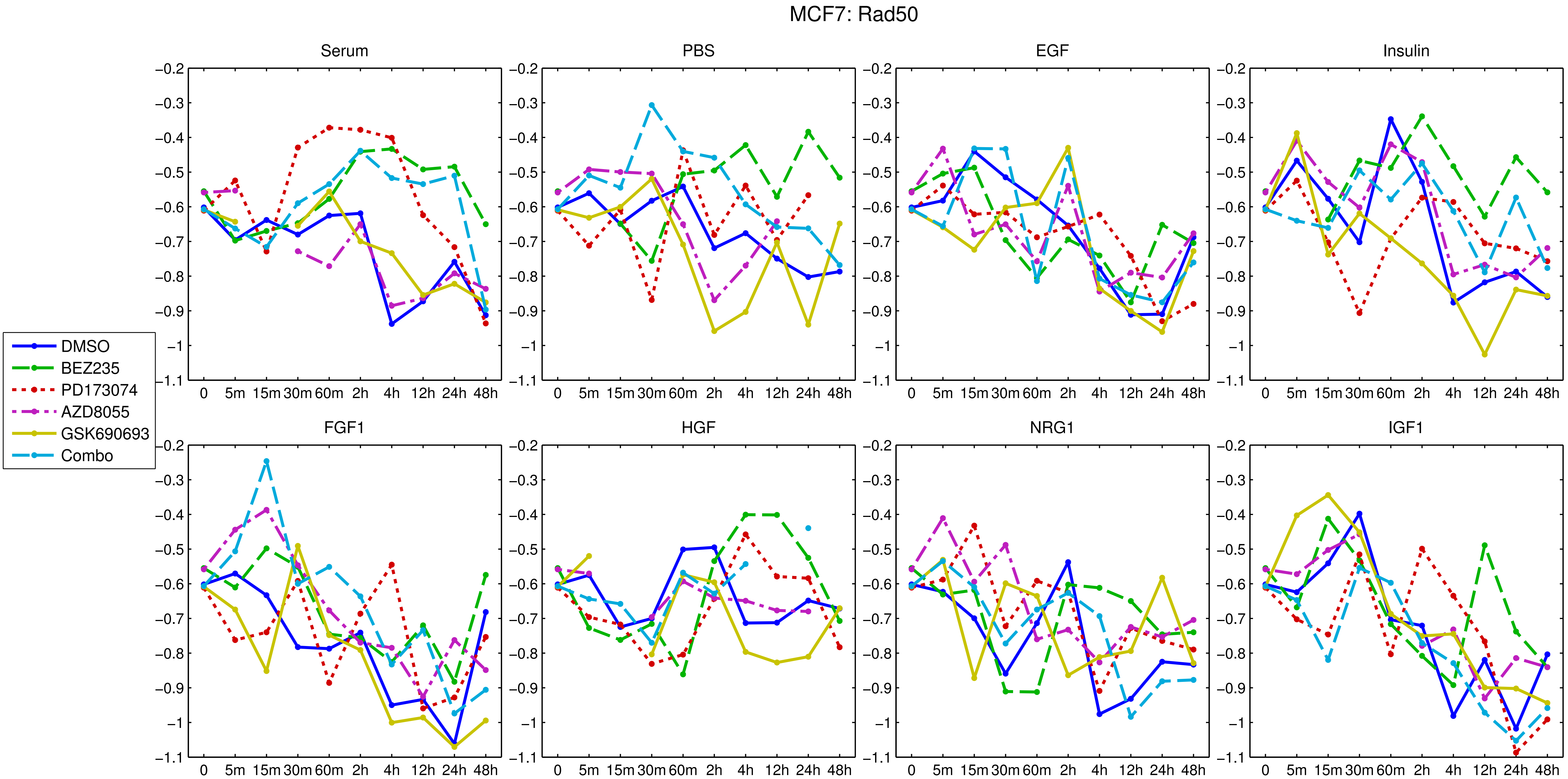

MCF7: Rad50
Serum
PBS
EGF
Insulin
FGF1
HGF
NRG1
IGF1
DMSO
BEZ235
PD173074
AZD8055
GSK690693
Combo
−0.2
−0.3
−0.4
−0.5
−0.6
−0.7
−0.8
−0.9
−1
−1.1
0
5m
15m
30m
60m
2h
4h
12h
24h
48h

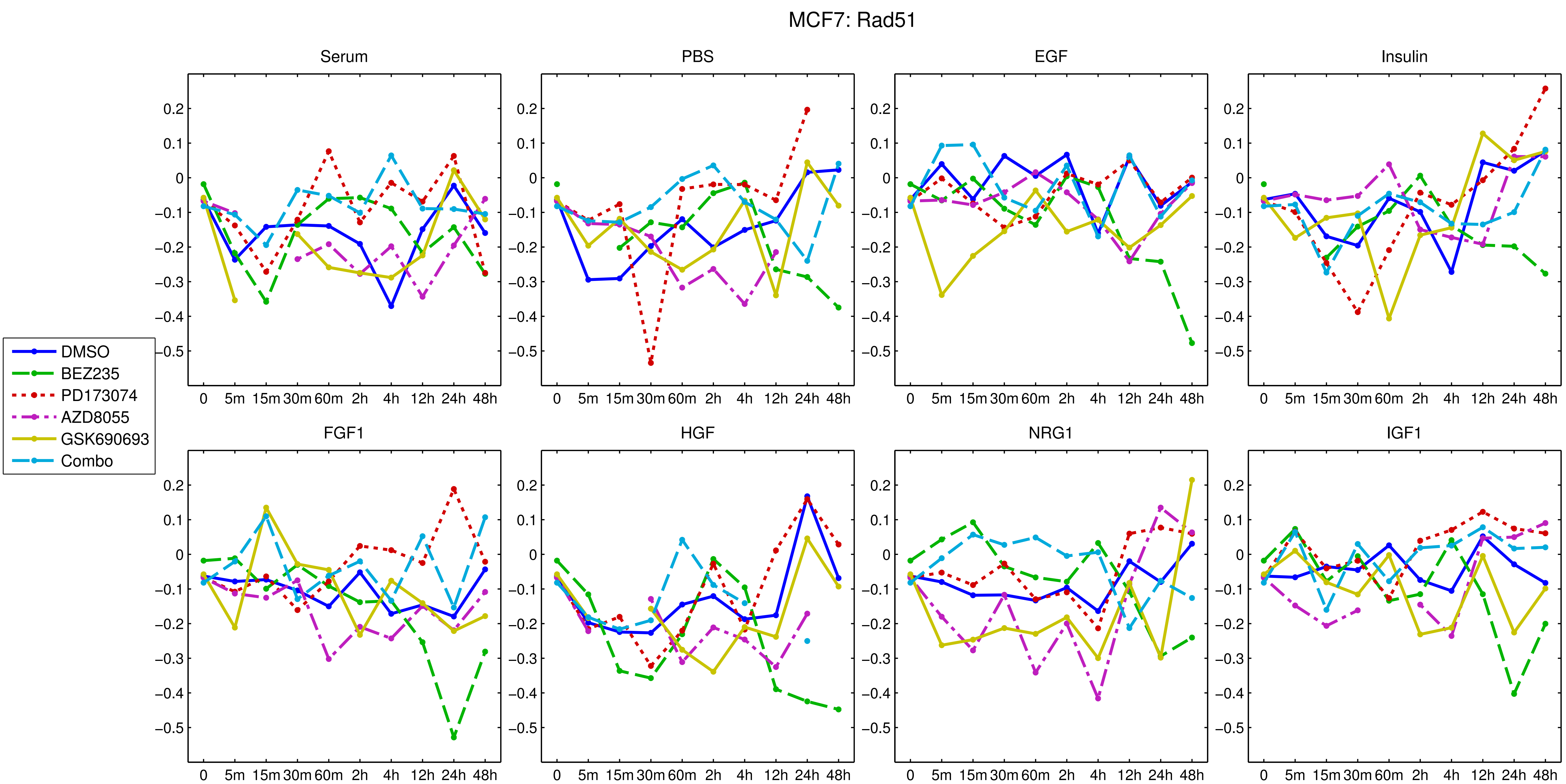
MCF7: Rad51
Serum
PBS
EGF
Insulin
FGF1
HGF
NRG1
IGF1
DMSO
BEZ235
PD173074
AZD8055
GSK690693
Combo
0.2
0.1
0
−0.1
−0.2
−0.3
−0.4
−0.5
0 5m 15m 30m 60m 2h 4h 12h 24h 48h

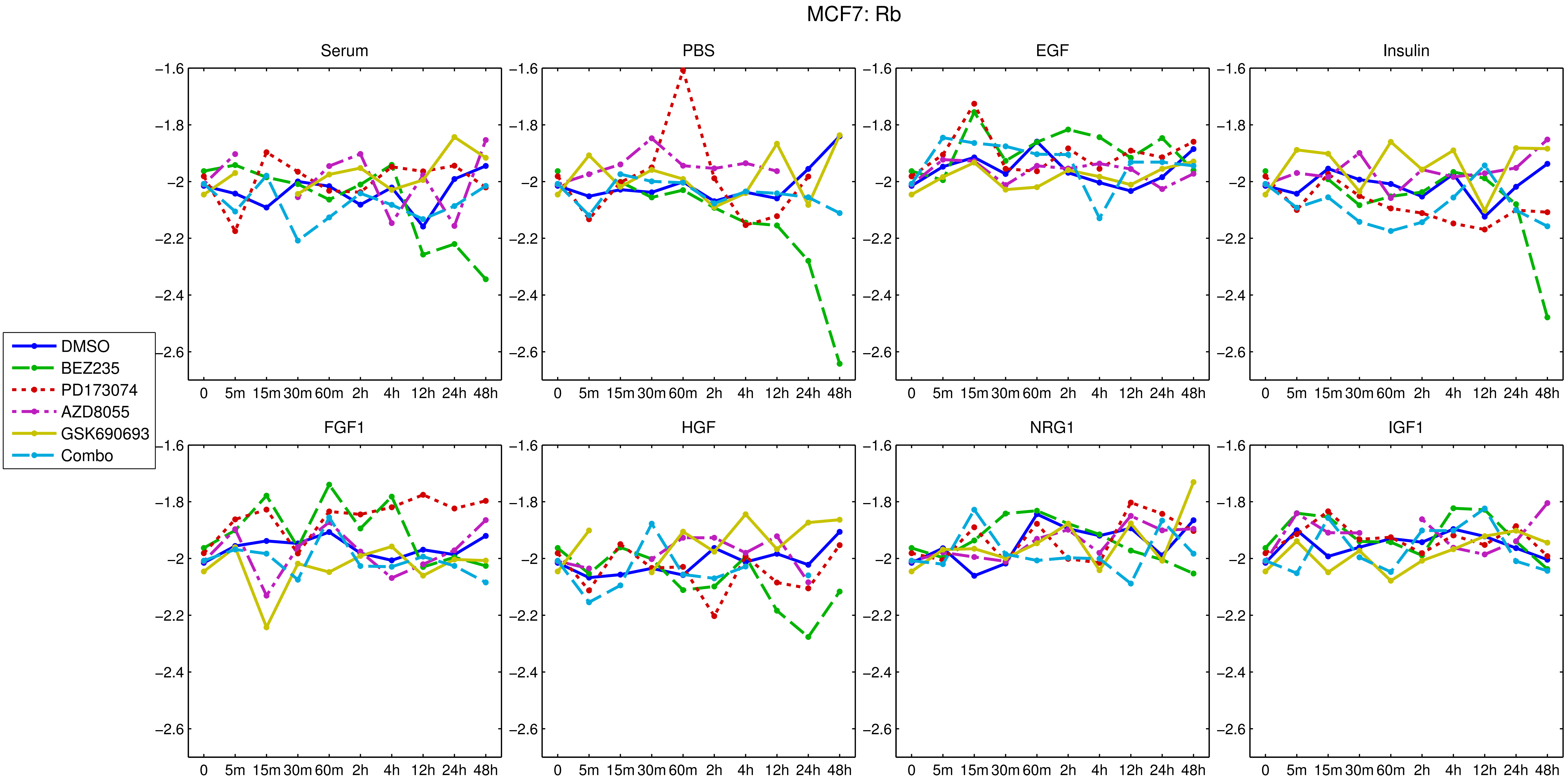

MCF7: Rb
Serum
PBS
EGF
Insulin
FGF1
HGF
NRG1
IGF1
−1.6
−1.8
−2
−2.2
−2.4
−2.6
0
5m
15m
30m
60m
2h
4h
12h
24h
48h
DMSO
BEZ235
PD173074
AZD8055
GSK690693
Combo

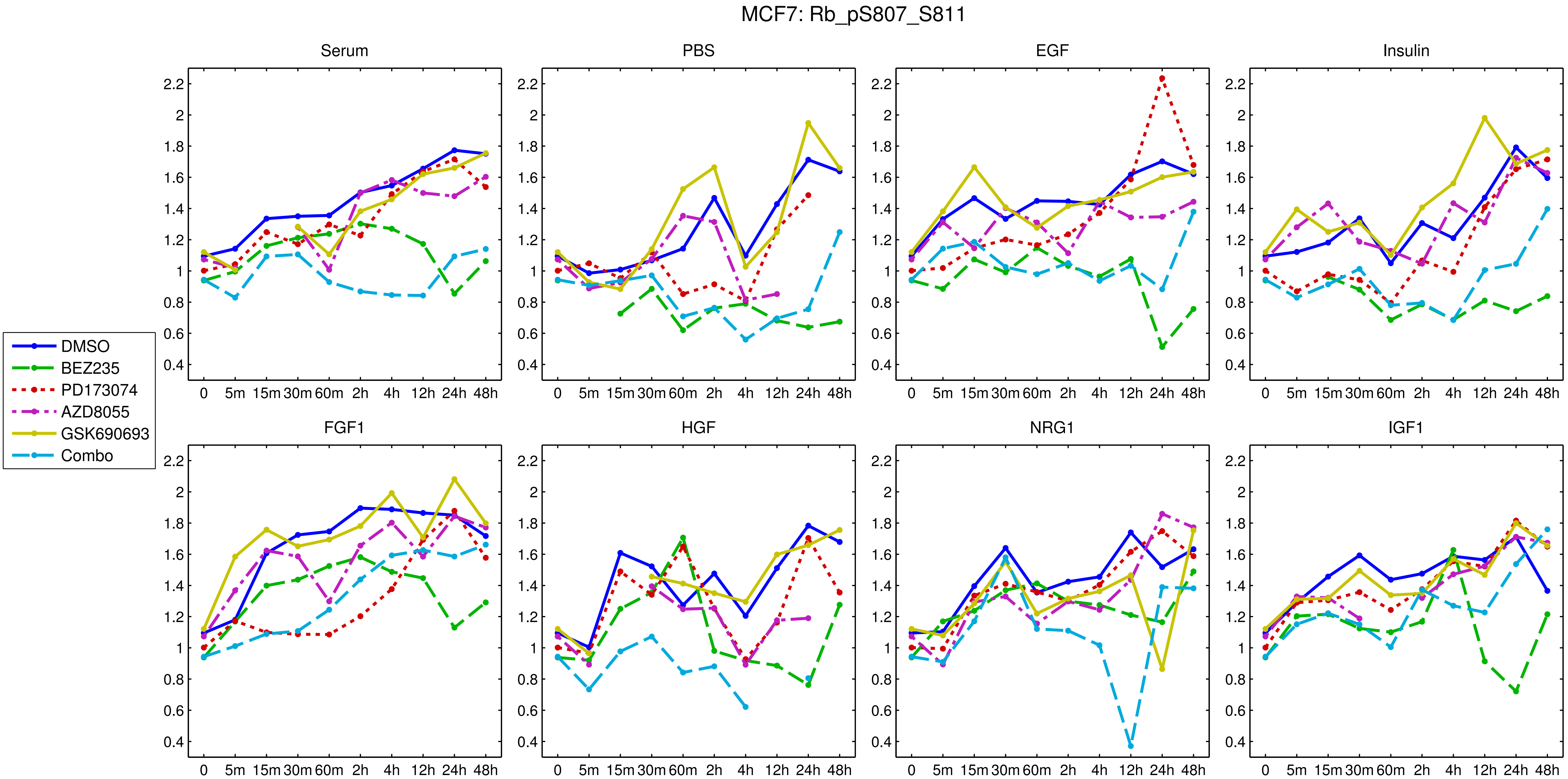

MCF7: Rb_pS807_S811
Serum
PBS
EGF
Insulin
FGF1
HGF
NRG1
IGF1
DMSO
BEZ235
PD173074
AZD8055
GSK690693
Combo
2.2
2
1.8
1.6
1.4
1.2
1
0.8
0.6
0.4
0
5m
15m
30m
60m
2h
4h
12h
24h
48h

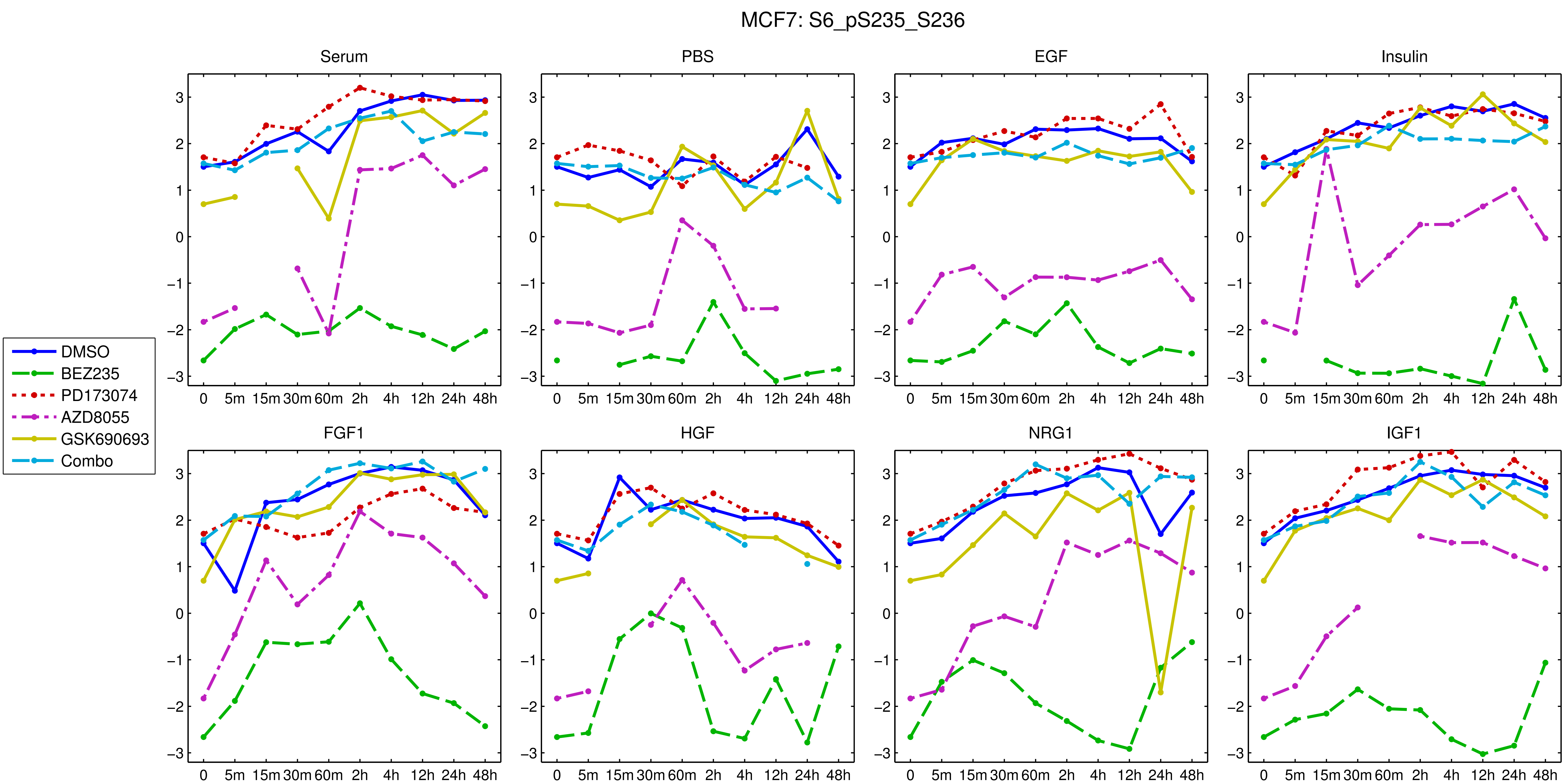
MCF7: S6_pS235_S236
Serum
PBS
EGF
Insulin
FGF1
HGF
NRG1
IGF1
DMSO
BEZ235
PD173074
AZD8055
GSK690693
Combo
0
5m
15m
30m
60m
2h
4h
12h
24h
48h

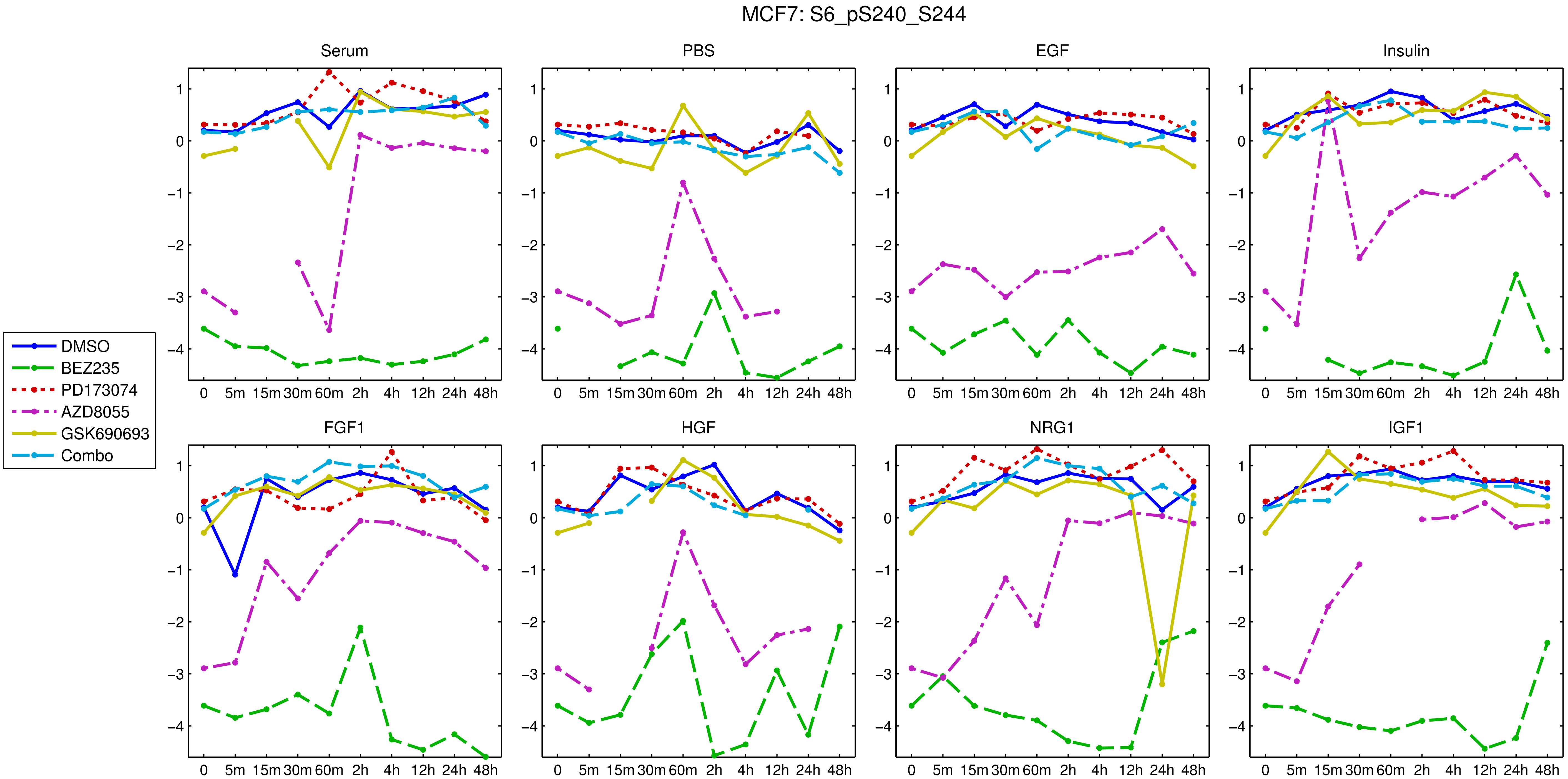
MCF7: S6_pS240_S244
Serum
PBS
EGF
Insulin
FGF1
HGF
NRG1
IGF1
DMSO
BEZ235
PD173074
AZD8055
GSK690693
Combo
0
5m
15m
30m
60m
2h
4h
12h
24h
48h
1
0
−1
−2
−3
−4

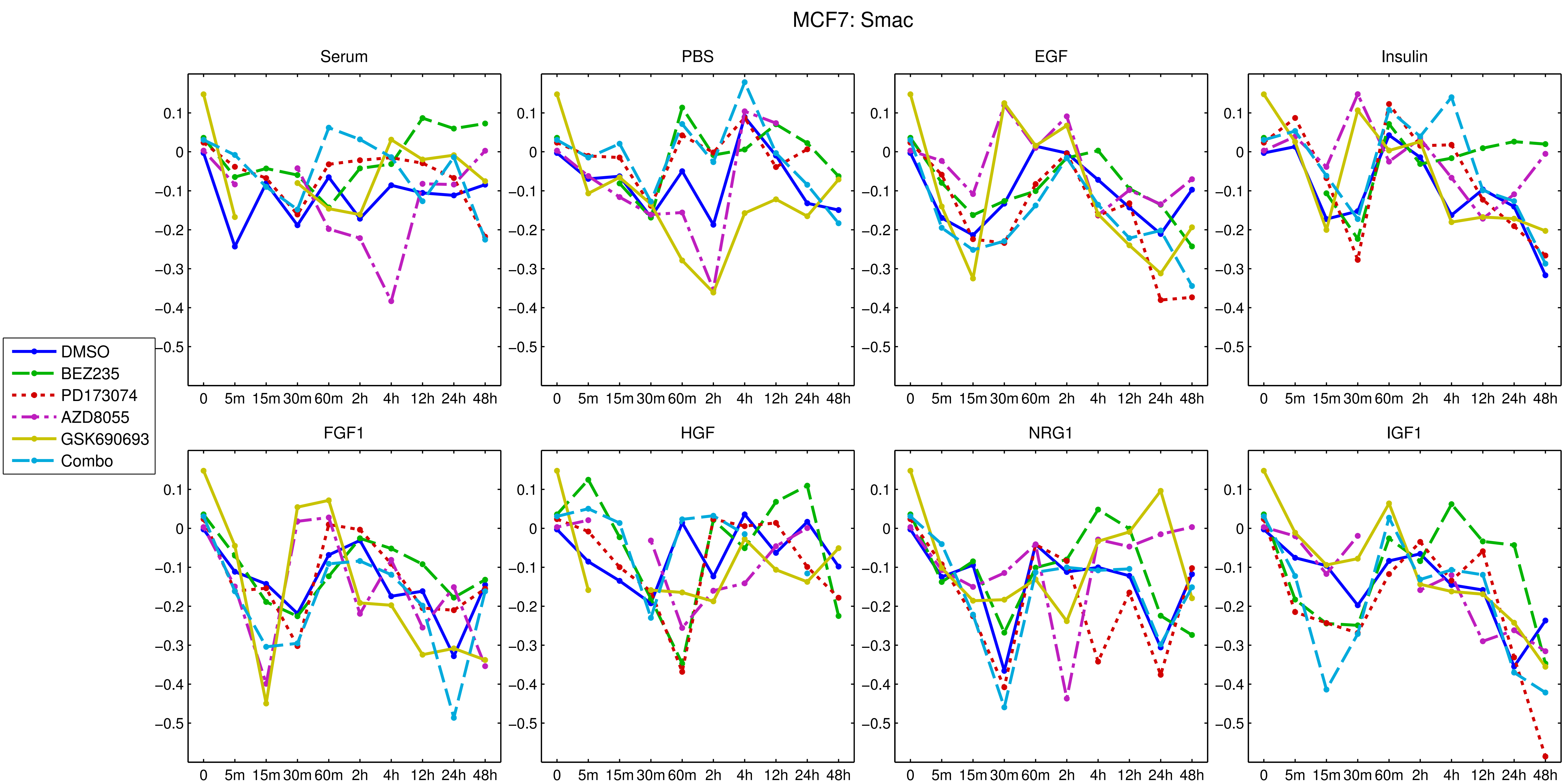

MCF7: Smac
Serum
PBS
EGF
Insulin
FGF1
HGF
NRG1
IGF1
0.1
0
−0.1
−0.2
−0.3
−0.4
−0.5
0
5m
15m
30m
60m
2h
4h
12h
24h
48h
DMSO
BEZ235
PD173074
AZD8055
GSK690693
Combo

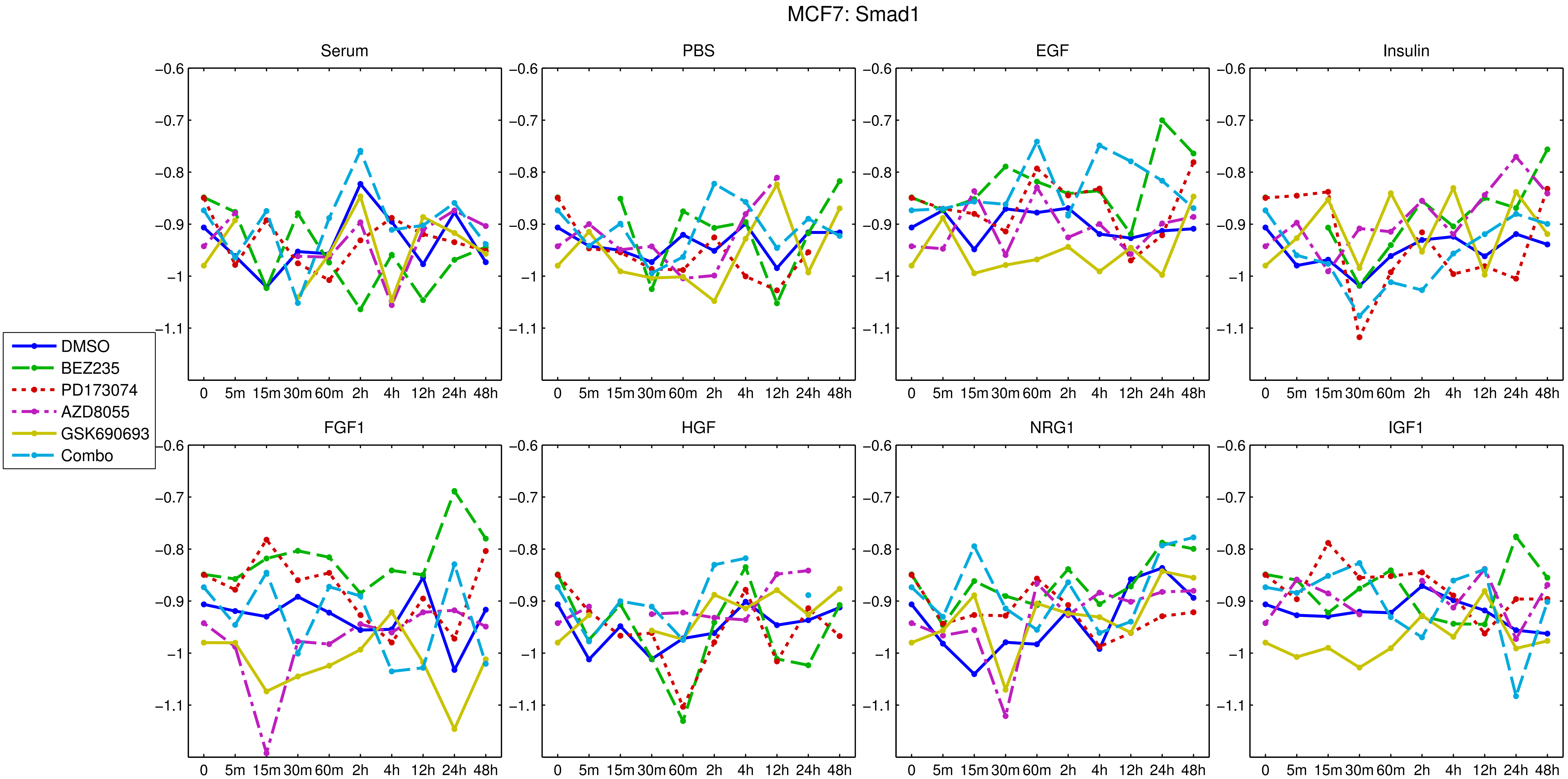
MCF7: Smad1
Serum
PBS
EGF
Insulin
FGF1
HGF
NRG1
IGF1
DMSO
BEZ235
PD173074
AZD8055
GSK690693
Combo
−0.6
−0.7
−0.8
−0.9
−1
−1.1
0
5m
15m
30m
60m
2h
4h
12h
24h
48h

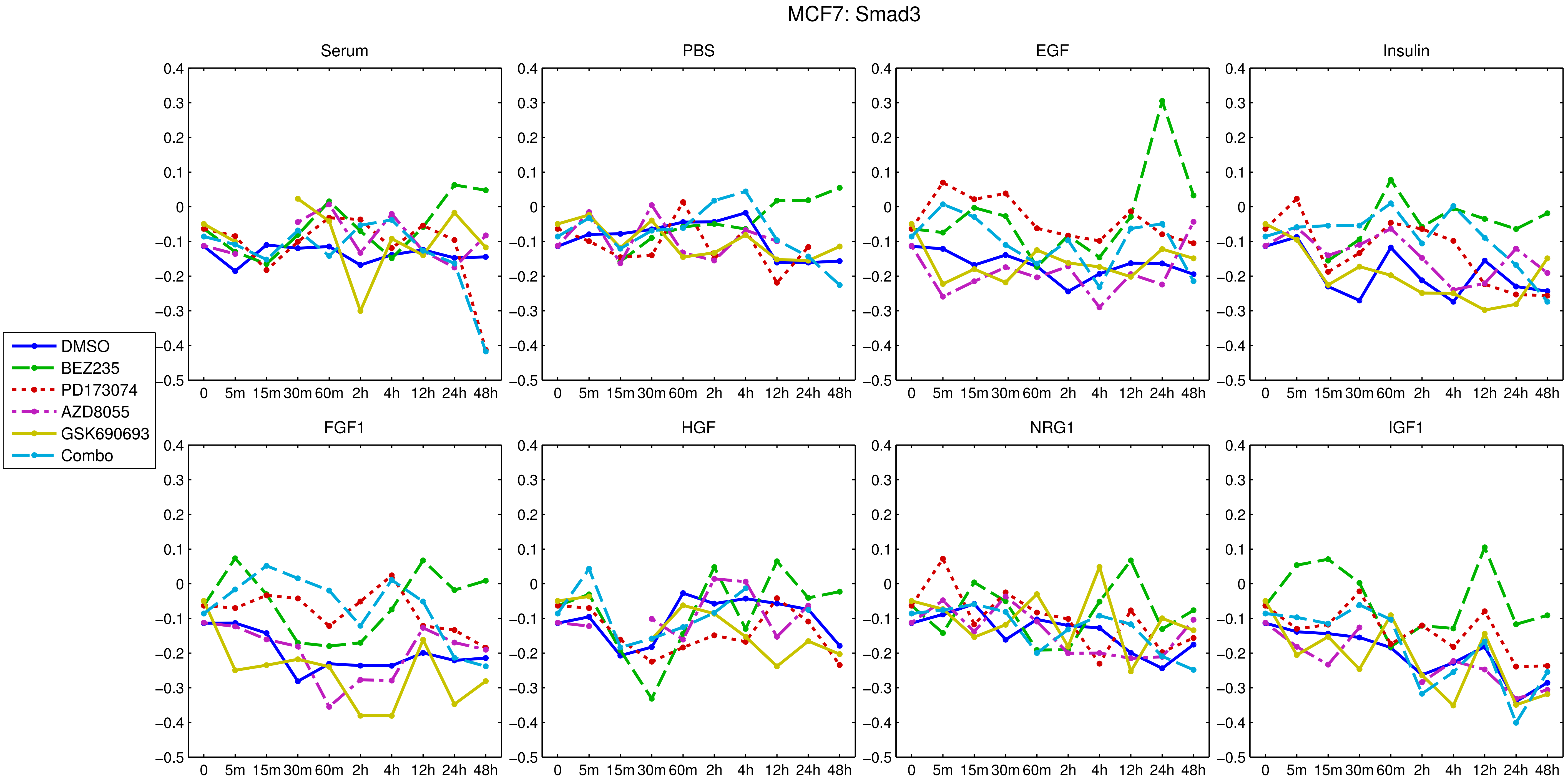

MCF7: Smad3
Serum
PBS
EGF
Insulin
FGF1
HGF
NRG1
IGF1
DMSO
BEZ235
PD173074
AZD8055
GSK690693
Combo
0.4
0.3
0.2
0.1
0
−0.1
−0.2
−0.3
−0.4
−0.5
0
5m
15m
30m
60m
2h
4h
12h
24h
48h

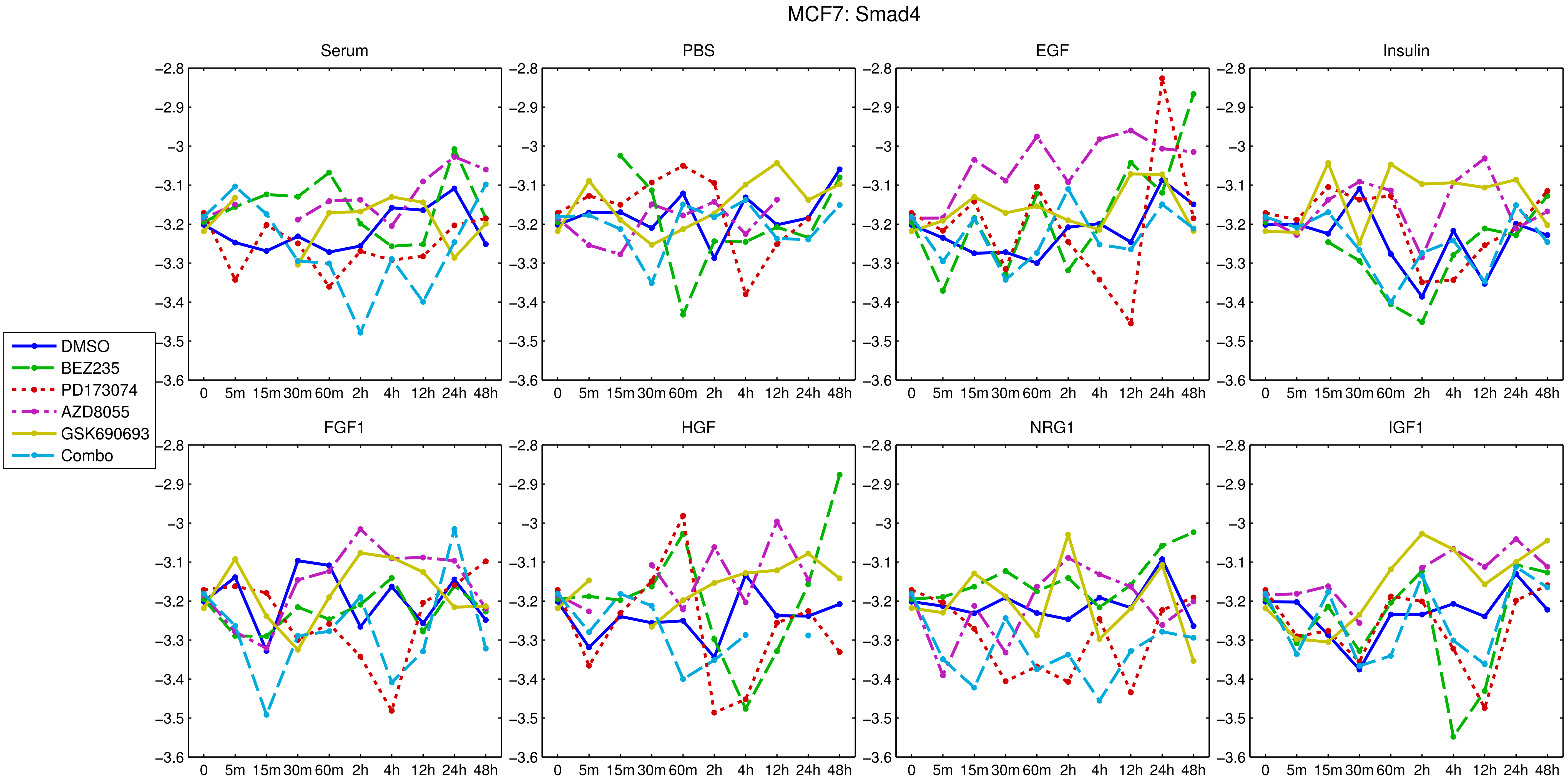

MCF7: Smad4
Serum
PBS
EGF
Insulin
FGF1
HGF
NRG1
IGF1
DMSO
BEZ235
PD173074
AZD8055
GSK690693
Combo
−2.8
−2.9
−3
−3.1
−3.2
−3.3
−3.4
−3.5
−3.6
0
5m
15m
30m
60m
2h
4h
12h
24h
48h

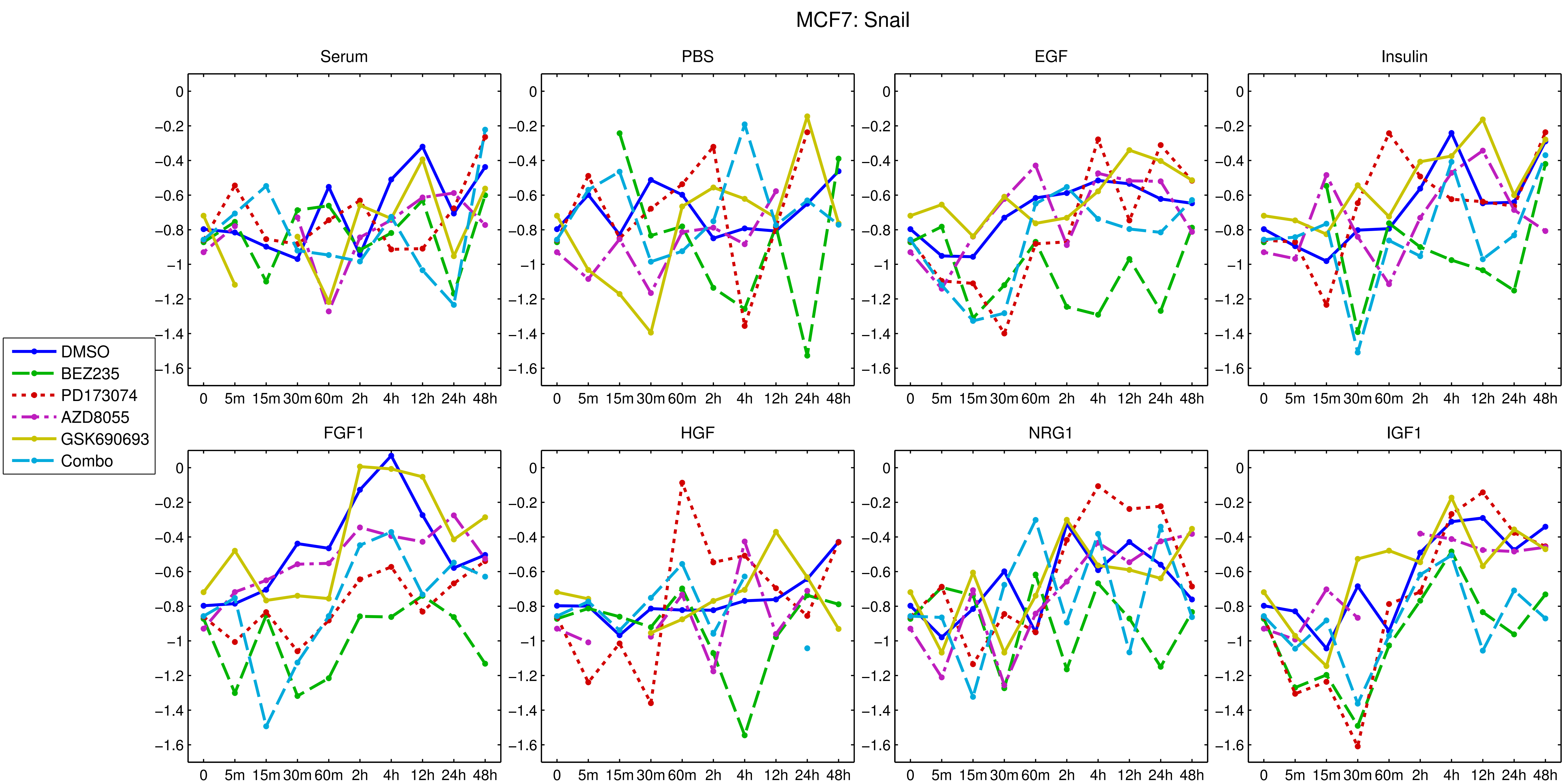

MCF7: Snail
Serum
PBS
EGF
Insulin
FGF1
HGF
NRG1
IGF1
DMSO
BEZ235
PD173074
AZD8055
GSK690693
Combo
0
−0.2
−0.4
−0.6
−0.8
−1
−1.2
−1.4
−1.6
0 5m 15m 30m 60m 2h 4h 12h 24h 48h

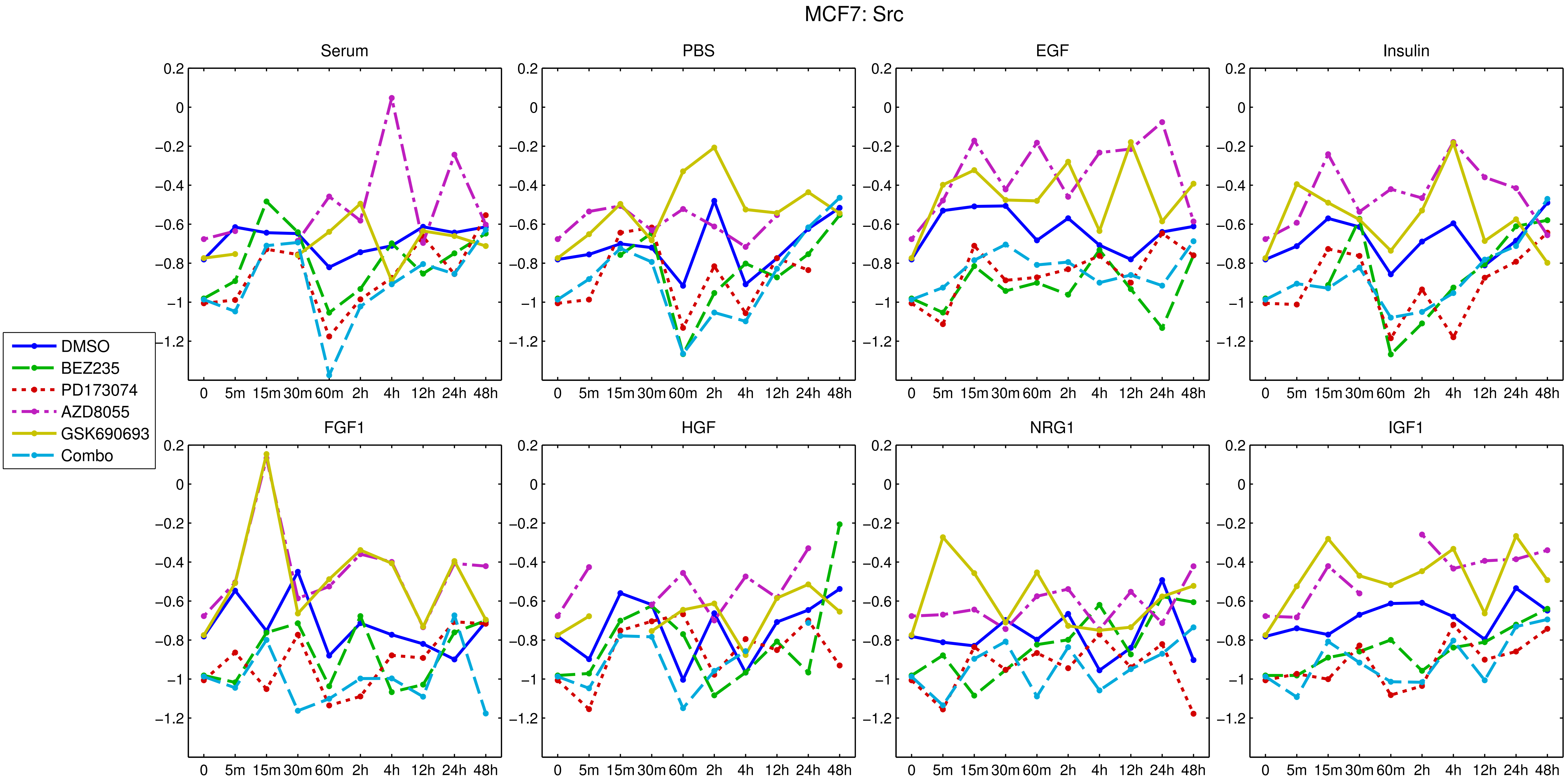
MCF7: Src
Serum
PBS
EGF
Insulin
FGF1
HGF
NRG1
IGF1
0.2
0
−0.2
−0.4
−0.6
−0.8
−1
−1.2
0
5m
15m
30m
60m
2h
4h
12h
24h
48h
DMSO
BEZ235
PD173074
AZD8055
GSK690693
Combo

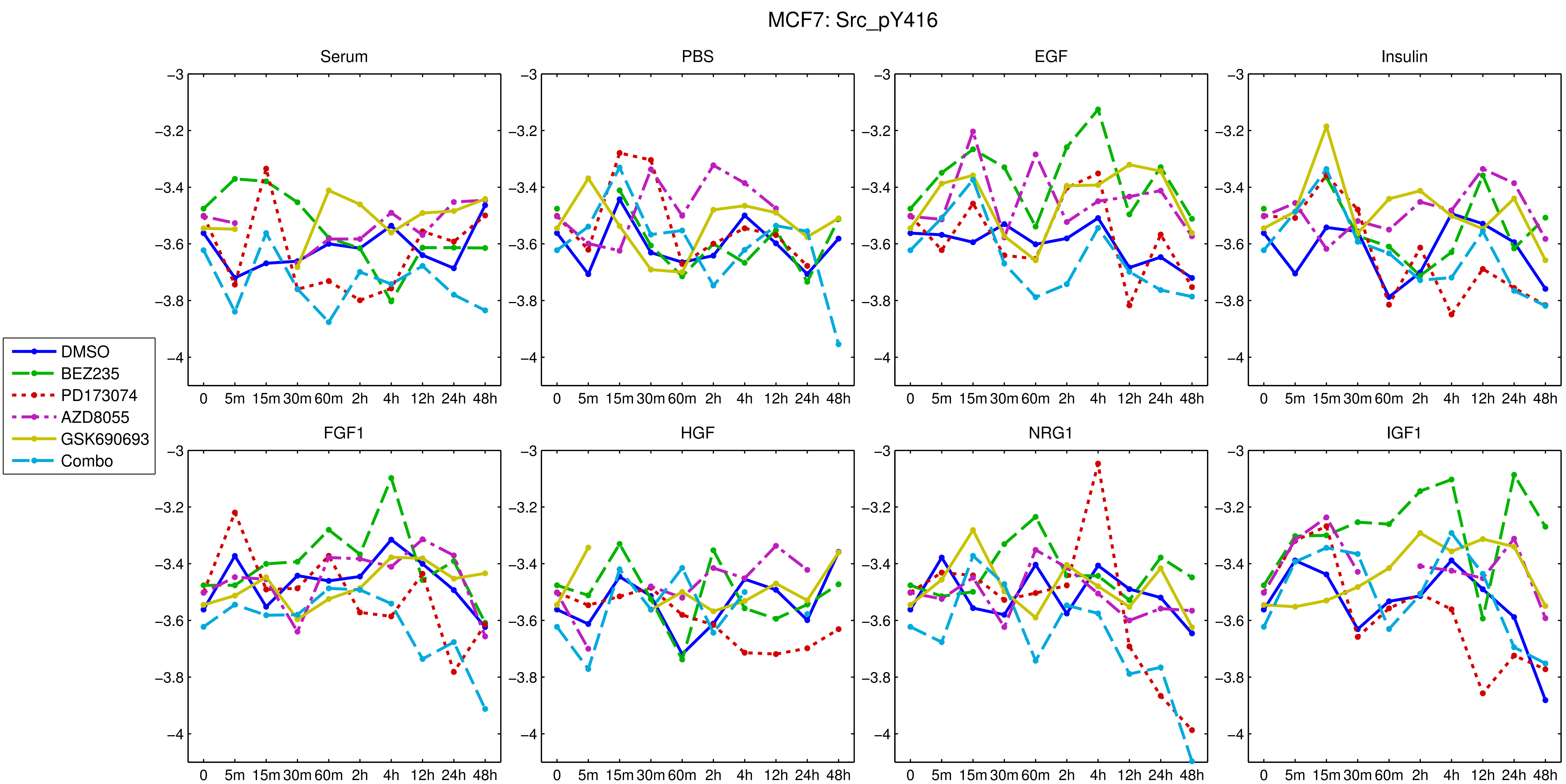

MCF7: Src_pY416
Serum
PBS
EGF
Insulin
FGF1
HGF
NRG1
IGF1
−3
−3.2
−3.4
−3.6
−3.8
−4
0
5m
15m
30m
60m
2h
4h
12h
24h
48h
DMSO
BEZ235
PD173074
AZD8055
GSK690693
Combo

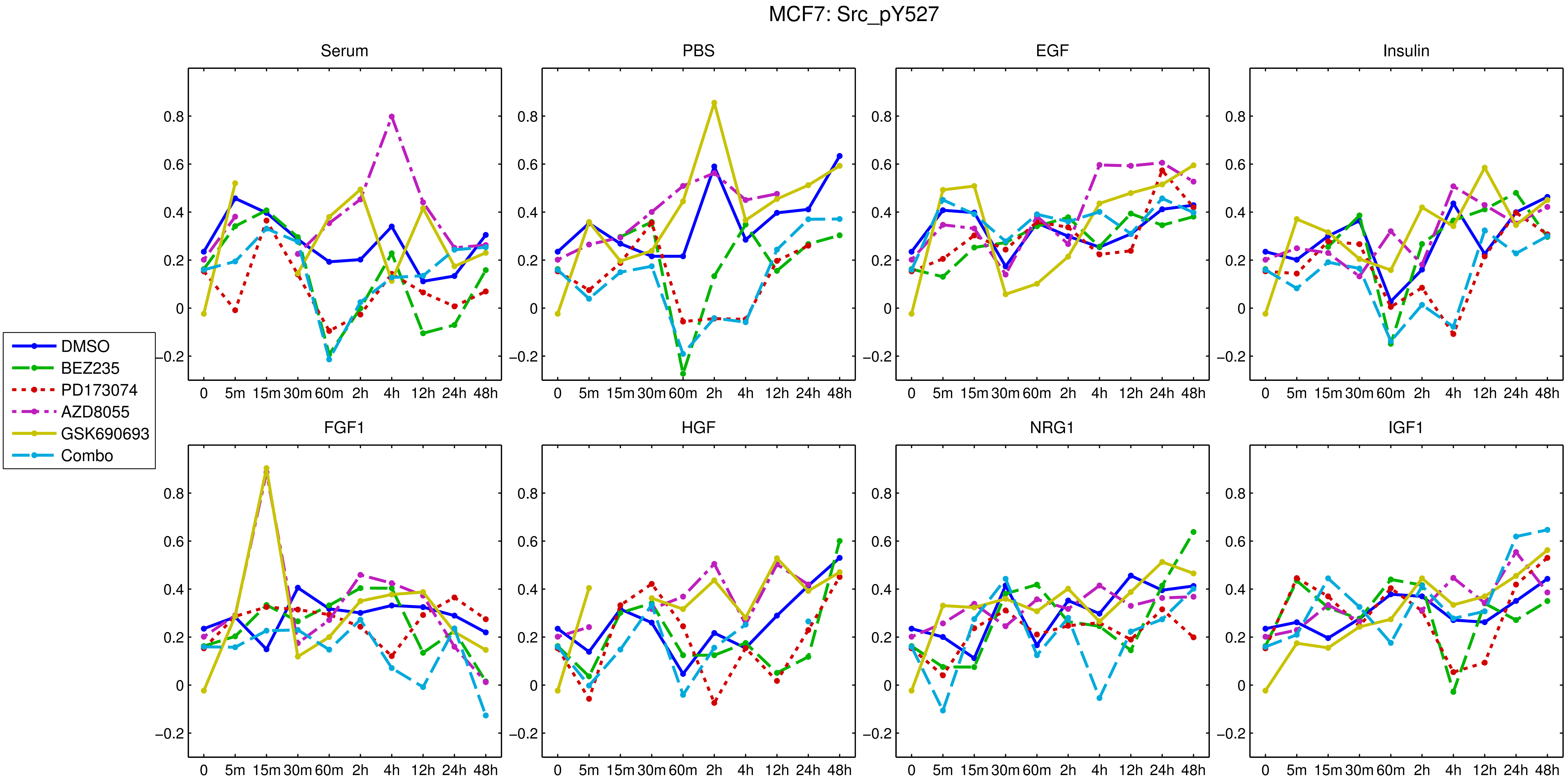
MCF7: Src_pY527
Serum
PBS
EGF
Insulin
FGF1
HGF
NRG1
IGF1
DMSO
BEZ235
PD173074
AZD8055
GSK690693
Combo
0.8
0.6
0.4
0.2
0
−0.2
0
5m
15m
30m
60m
2h
4h
12h
24h
48h

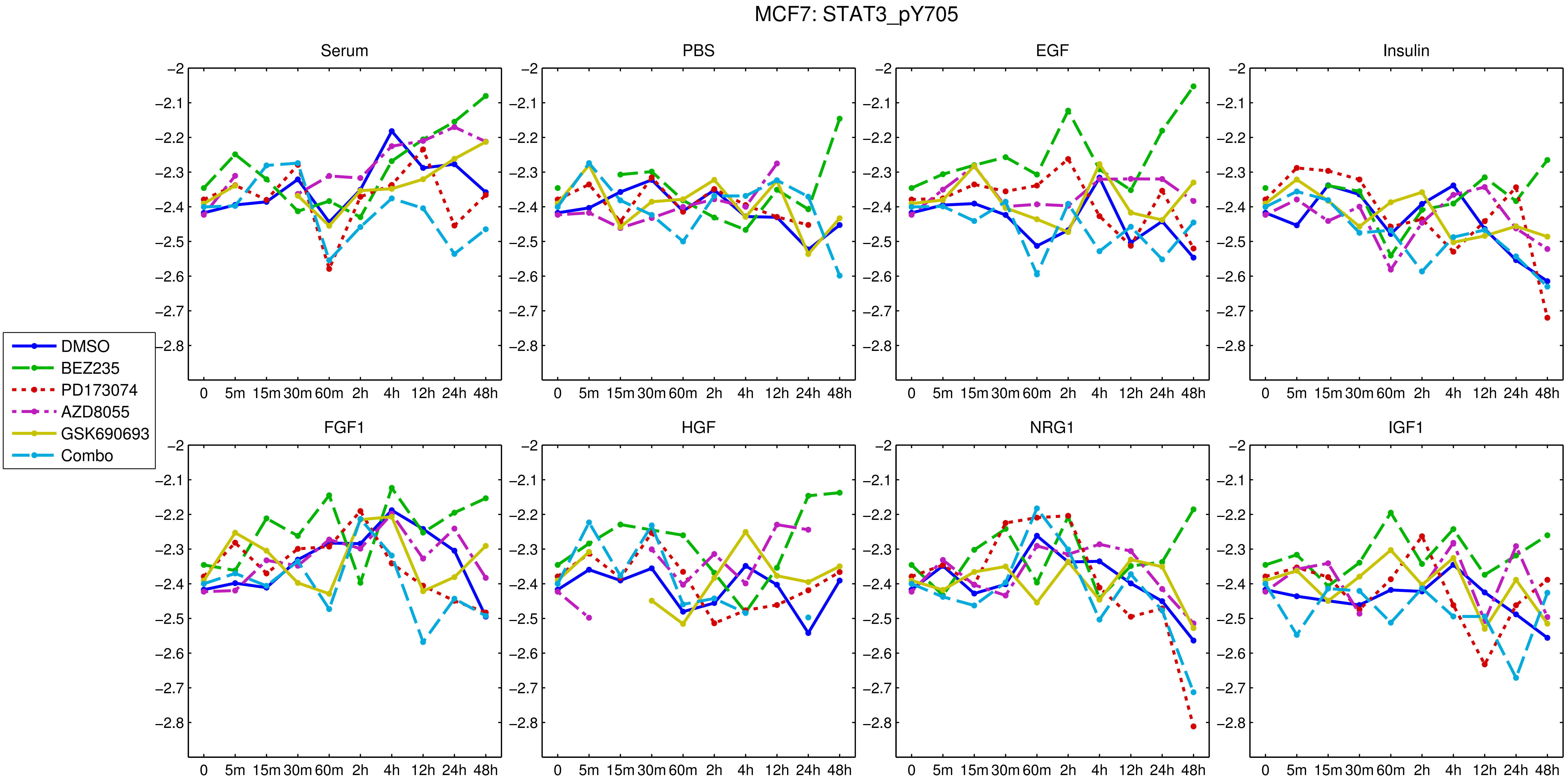

MCF7: STAT3_pY705
Serum
PBS
EGF
Insulin
FGF1
HGF
NRG1
IGF1
−2
−2.1
−2.2
−2.3
−2.4
−2.5
−2.6
−2.7
−2.8
0
5m
15m
30m
60m
2h
4h
12h
24h
48h
DMSO
BEZ235
PD173074
AZD8055
GSK690693
Combo

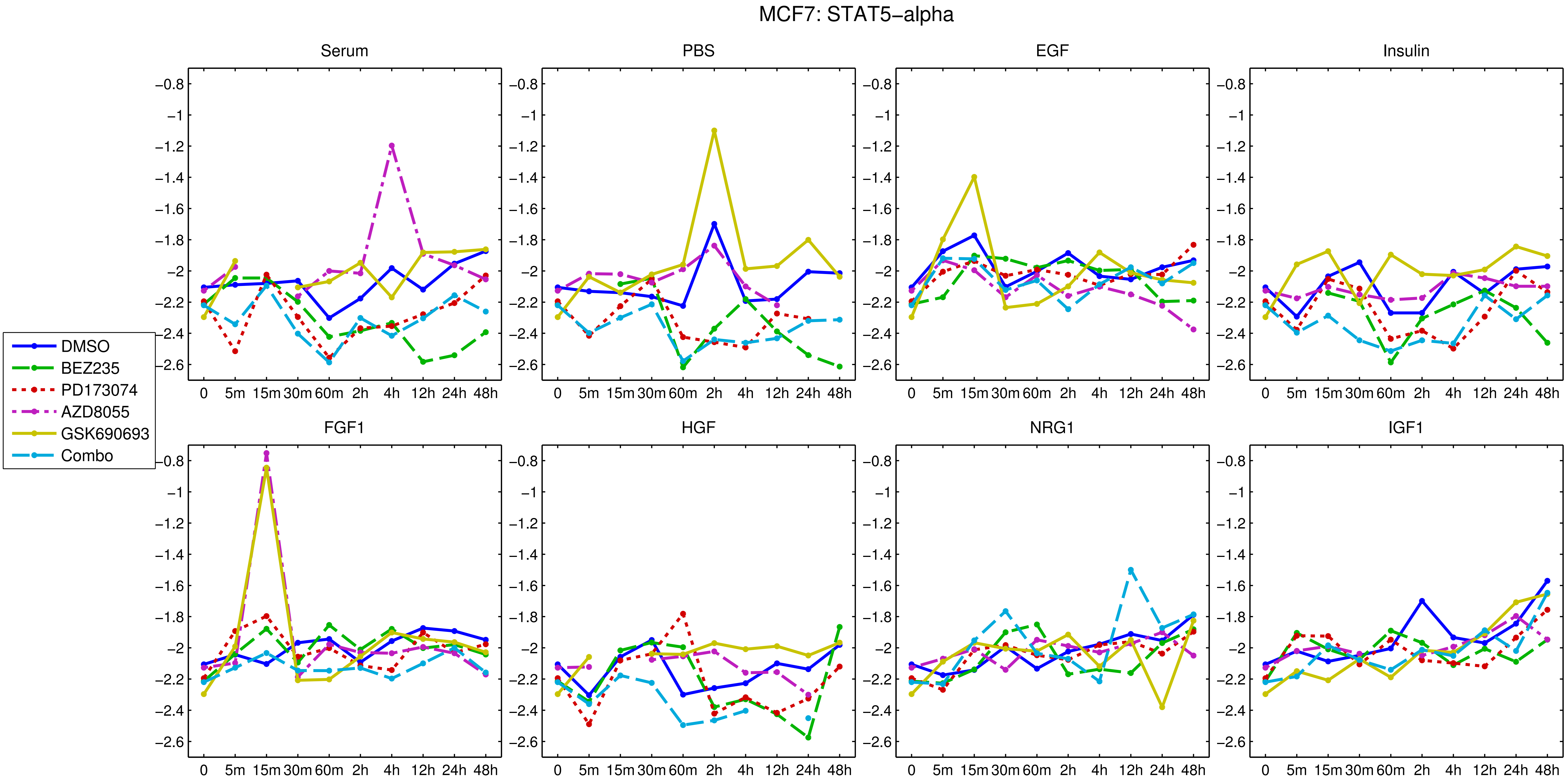
MCF7: STAT5−alpha
Serum
PBS
EGF
Insulin
FGF1
HGF
NRG1
IGF1
−0.8
−1
−1.2
−1.4
−1.6
−1.8
−2
−2.2
−2.4
−2.6
0
5m
15m
30m
60m
2h
4h
12h
24h
48h
DMSO
BEZ235
PD173074
AZD8055
GSK690693
Combo

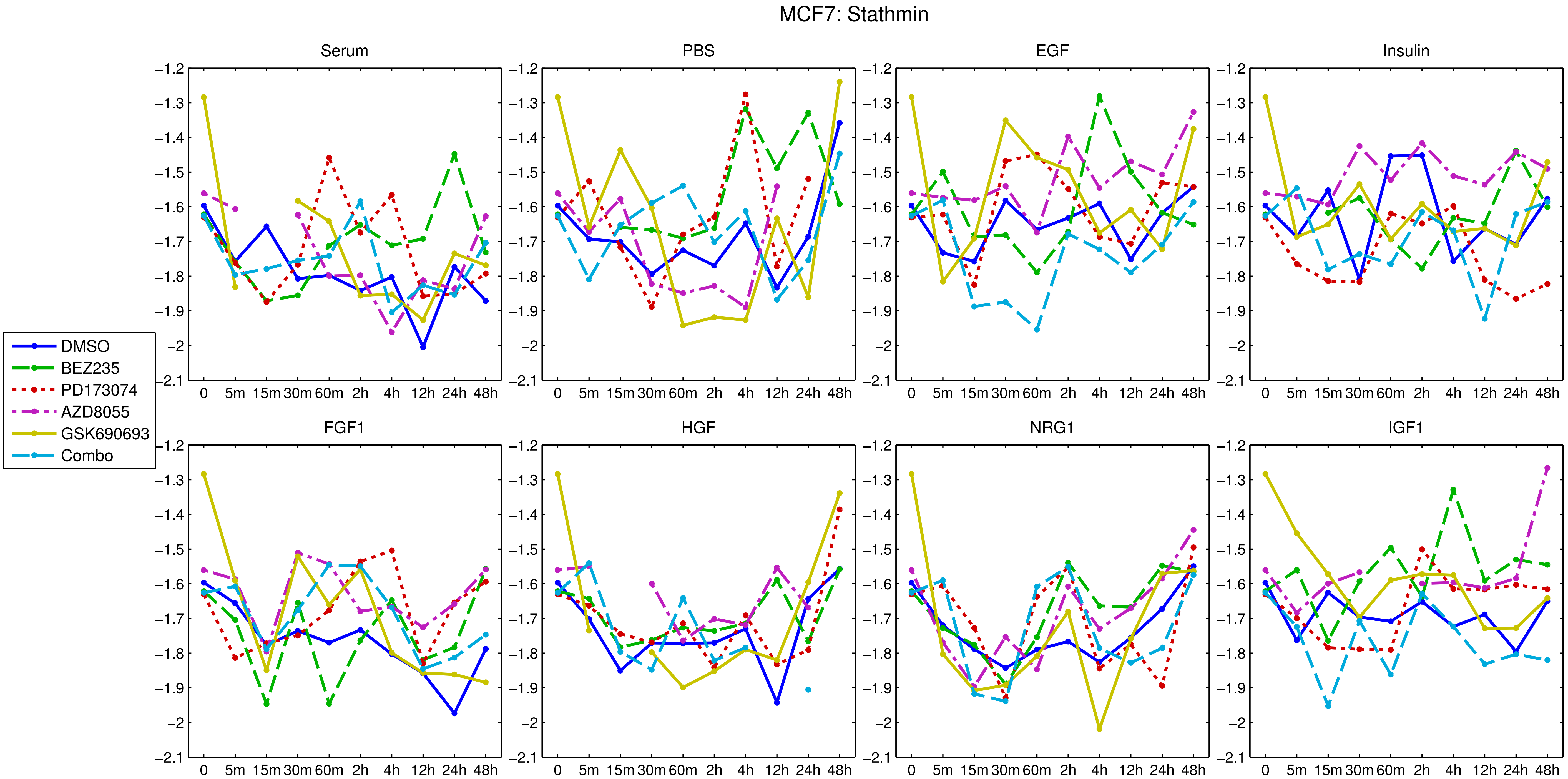
MCF7: Stathmin
Serum
PBS
EGF
Insulin
FGF1
HGF
NRG1
IGF1
DMSO
BEZ235
PD173074
AZD8055
GSK690693
Combo
0 5m 15m 30m 60m 2h 4h 12h 24h 48h
−1.2 −1.3 −1.4 −1.5 −1.6 −1.7 −1.8 −1.9 −2 −2.1

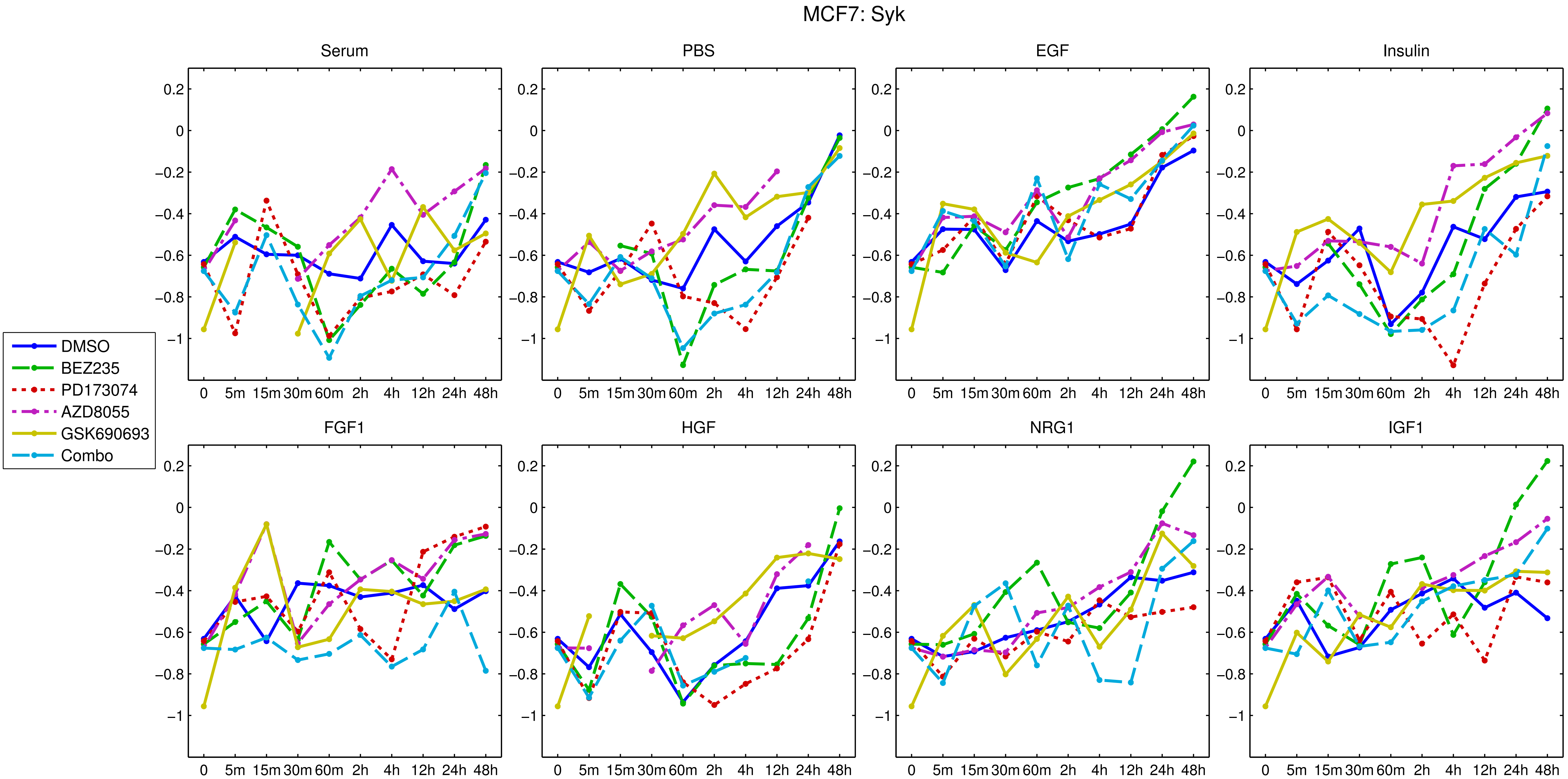

MCF7: Syk
Serum
PBS
EGF
Insulin
FGF1
HGF
NRG1
IGF1
0.2
0
−0.2
−0.4
−0.6
−0.8
−1
0 5m 15m 30m 60m 2h 4h 12h 24h 48h
DMSO
BEZ235
PD173074
AZD8055
GSK690693
Combo

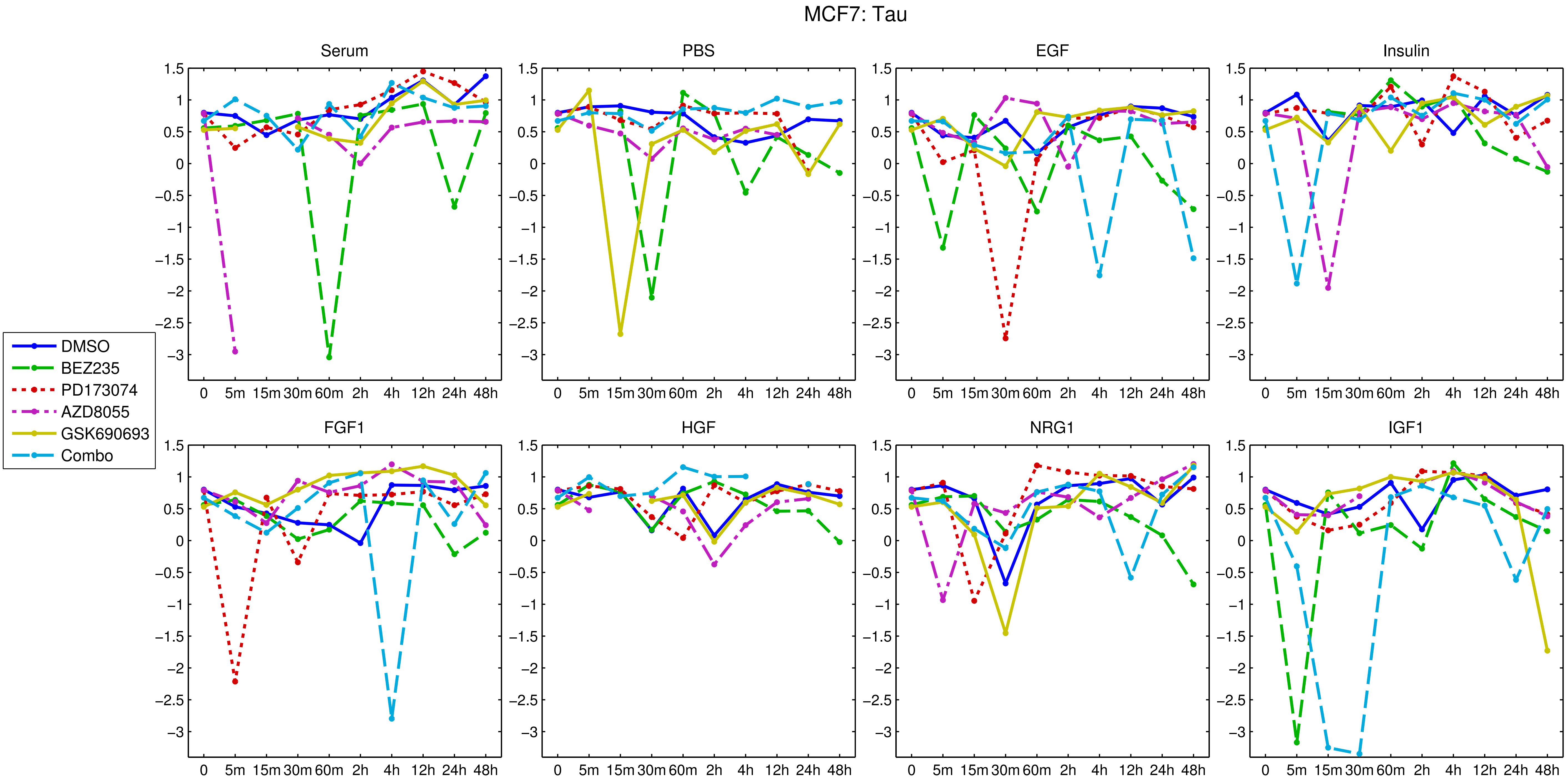
MCF7: Tau
Serum
PBS
EGF
Insulin
FGF1
HGF
NRG1
IGF1
1.5
1
0.5
0
−0.5
−1
−1.5
−2
−2.5
−3
0 5m 15m 30m 60m 2h 4h 12h 24h 48h
DMSO
BEZ235
PD173074
AZD8055
GSK690693
Combo

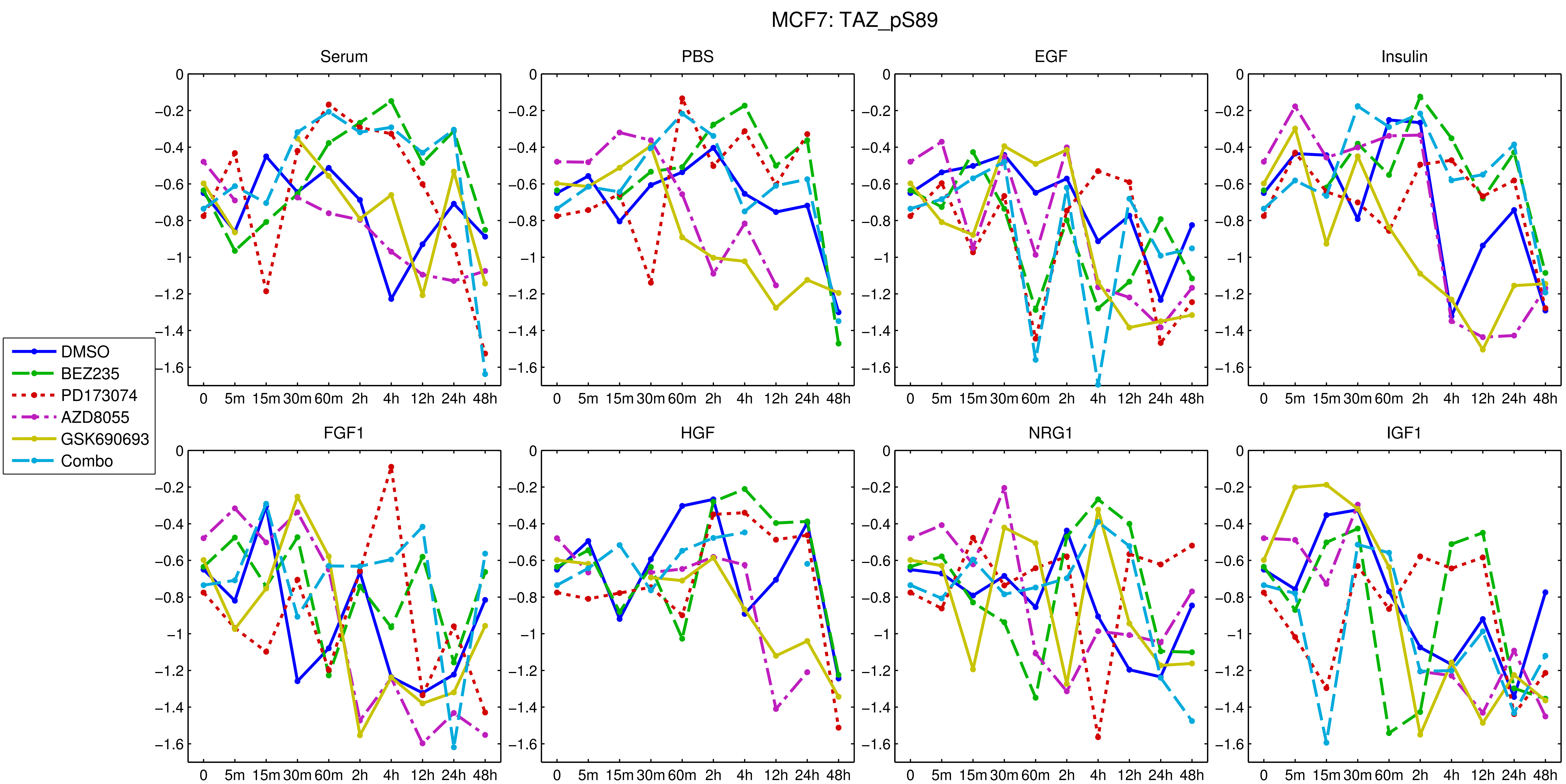
MCF7: TAZ_pS89
Serum
PBS
EGF
Insulin
FGF1
HGF
NRG1
IGF1
0
−0.2
−0.4
−0.6
−0.8
−1
−1.2
−1.4
−1.6
0 5m 15m 30m 60m 2h 4h 12h 24h 48h
DMSO
BEZ235
PD173074
AZD8055
GSK690693
Combo

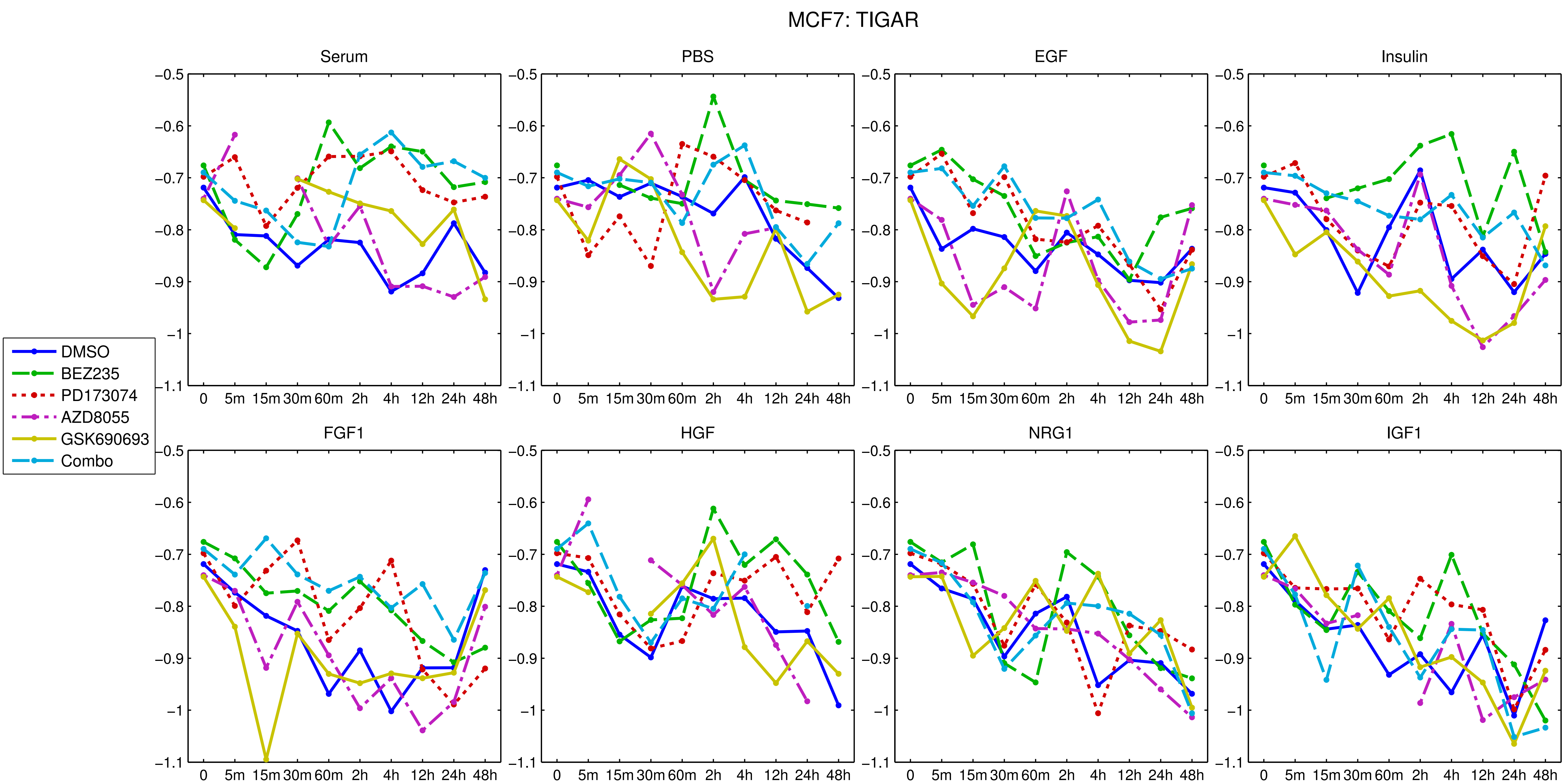
MCF7: TIGAR
Serum
PBS
EGF
Insulin
FGF1
HGF
NRG1
IGF1
DMSO
BEZ235
PD173074
AZD8055
GSK690693
Combo
−0.5
−0.6
−0.7
−0.8
−0.9
−1
−1.1
0
5m
15m
30m
60m
2h
4h
12h
24h
48h

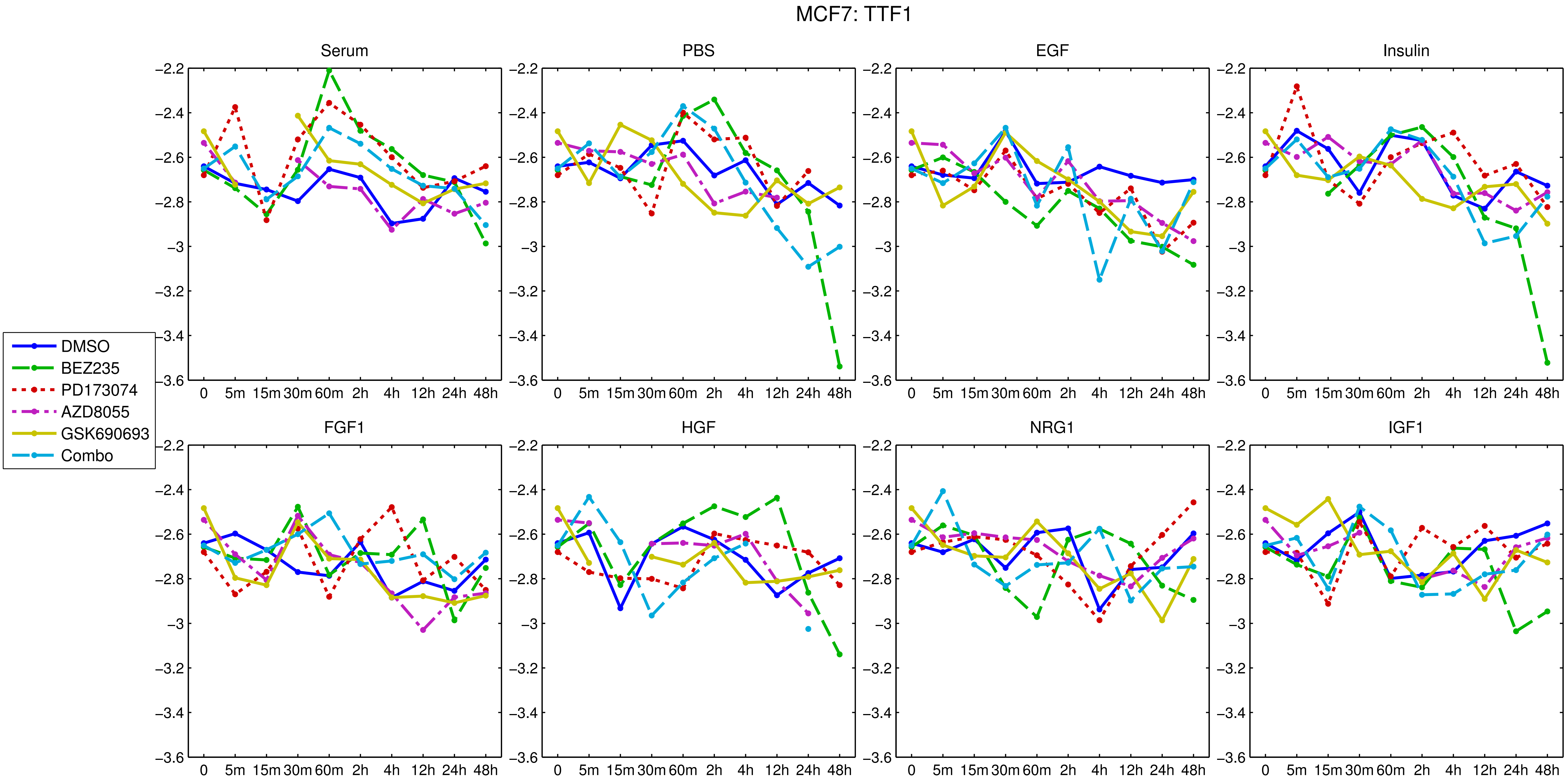

MCF7: TTF1
Serum
PBS
EGF
Insulin
FGF1
HGF
NRG1
IGF1
DMSO
BEZ235
PD173074
AZD8055
GSK690693
Combo
−2.2
−2.4
−2.6
−2.8
−3
−3.2
−3.4
−3.6
0
5m
15m
30m
60m
2h
4h
12h
24h
48h

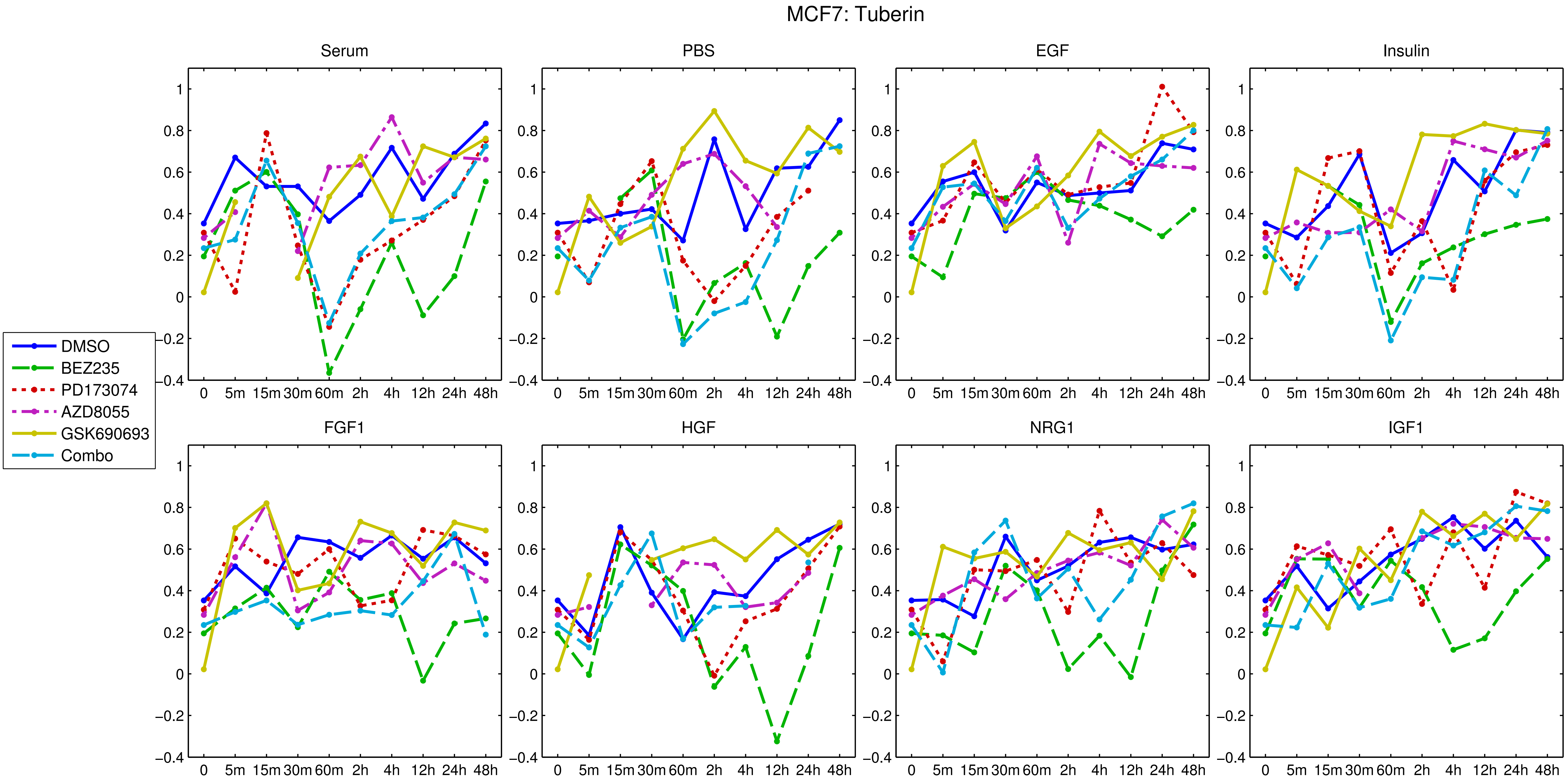

MCF7: Tuberin
Serum
PBS
EGF
Insulin
FGF1
HGF
NRG1
IGF1
DMSO
BEZ235
PD173074
AZD8055
GSK690693
Combo
0
5m
15m
30m
60m
2h
4h
12h
24h
48h
1
0.8
0.6
0.4
0.2
0
−0.2
−0.4

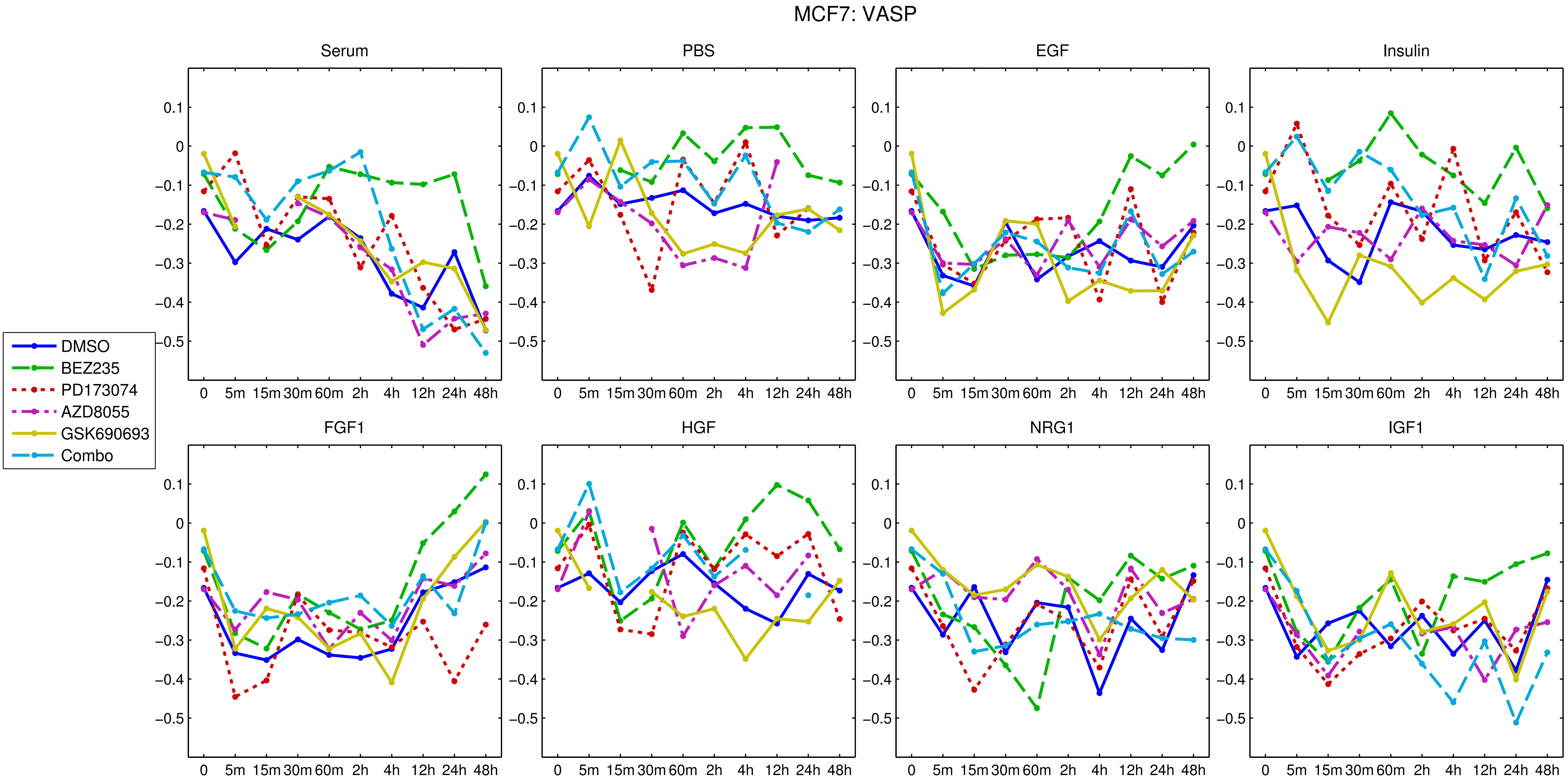
MCF7: VASP
Serum
PBS
EGF
Insulin
FGF1
HGF
NRG1
IGF1
0.1
0
−0.1
−0.2
−0.3
−0.4
−0.5
0
5m
15m
30m
60m
2h
4h
12h
24h
48h
DMSO
BEZ235
PD173074
AZD8055
GSK690693
Combo

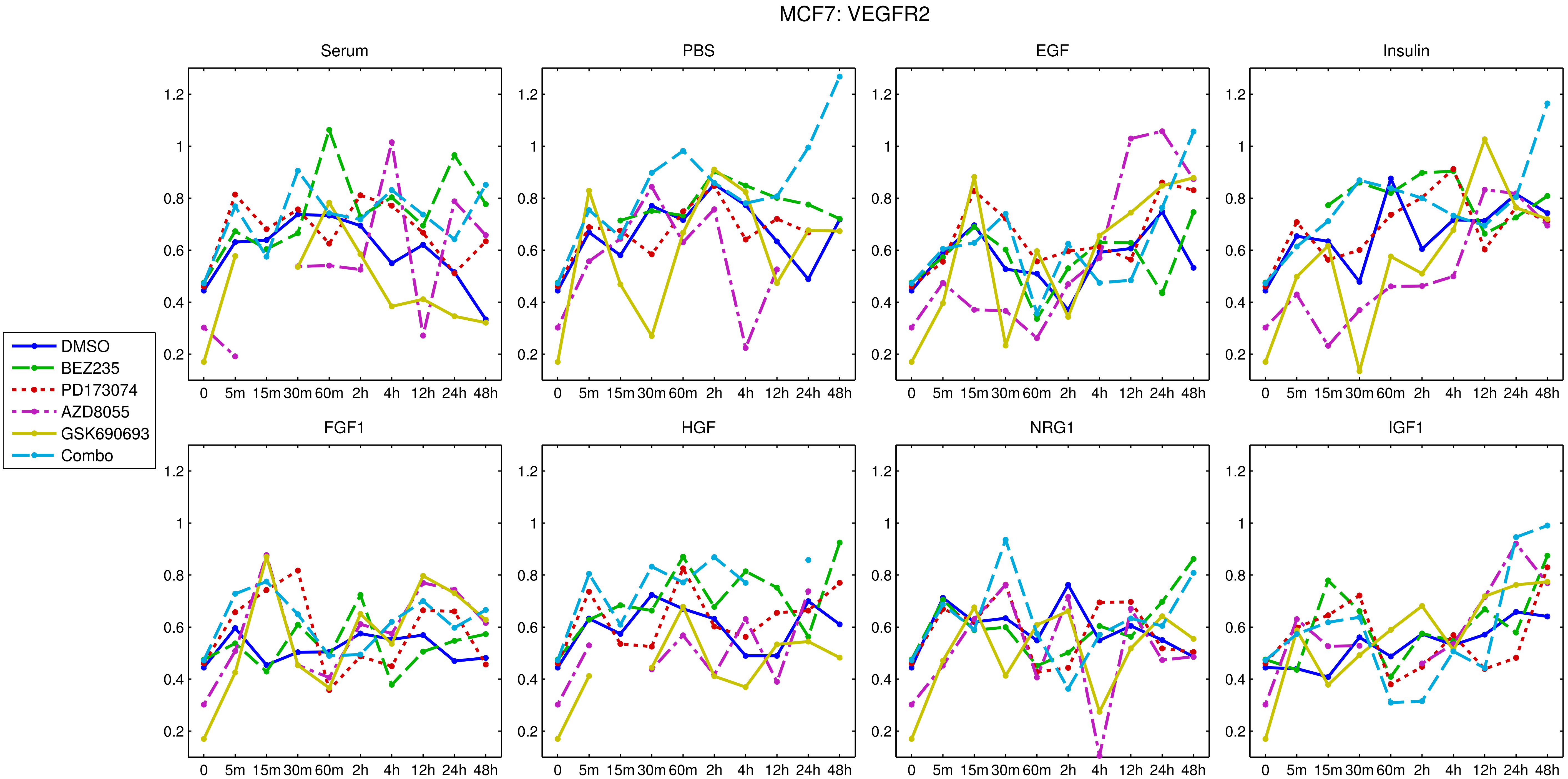
MCF7: VEGFR2
Serum
PBS
EGF
Insulin
FGF1
HGF
NRG1
IGF1
DMSO
BEZ235
PD173074
AZD8055
GSK690693
Combo
0.2
0.4
0.6
0.8
1
1.2
0
5m
15m
30m
60m
2h
4h
12h
24h
48h

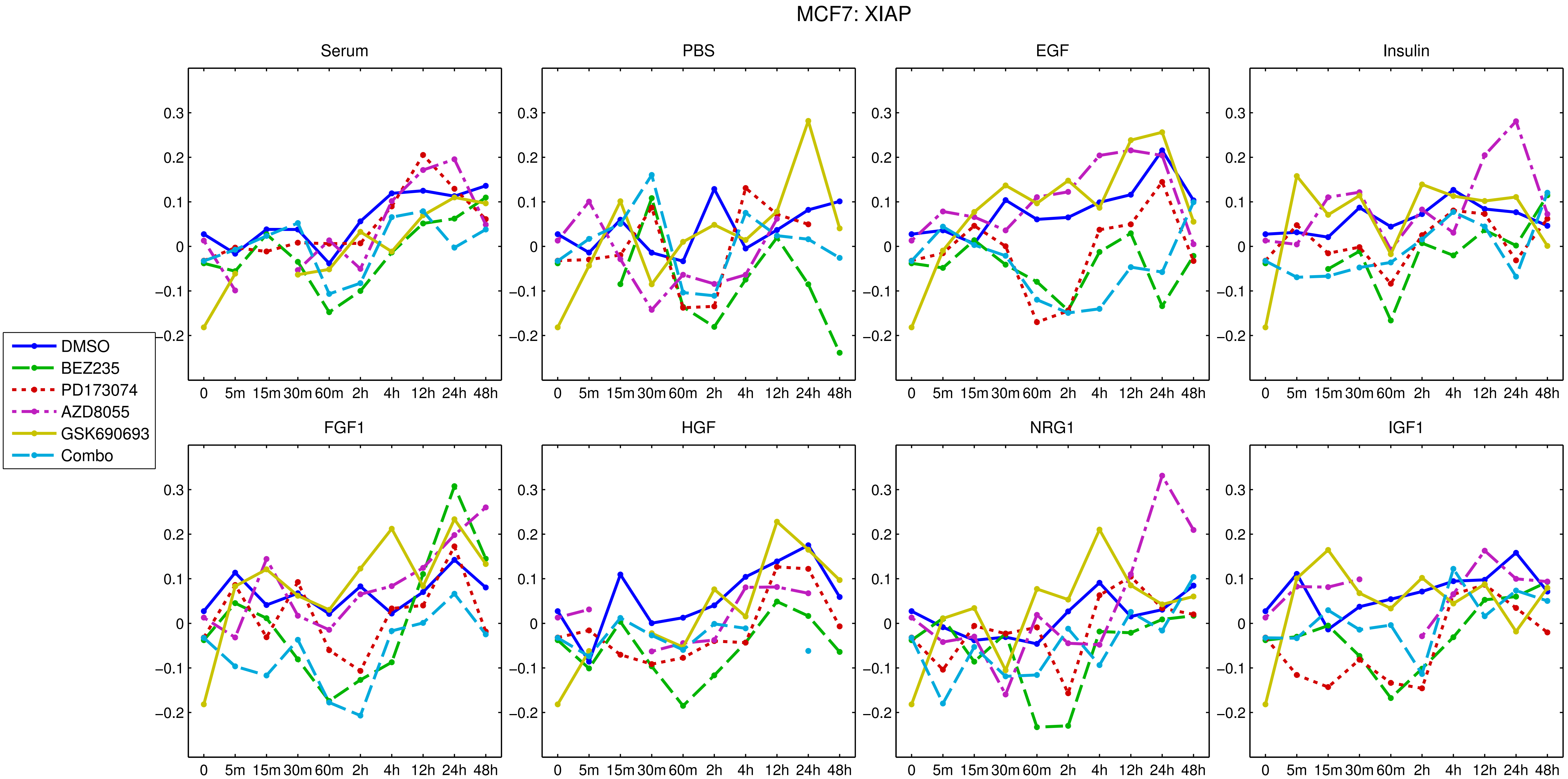

MCF7: XIAP
Serum
PBS
EGF
Insulin
FGF1
HGF
NRG1
IGF1
DMSO
BEZ235
PD173074
AZD8055
GSK690693
Combo
0.3
0.2
0.1
0
−0.1
−0.2
0
5m
15m
30m
60m
2h
4h
12h
24h
48h

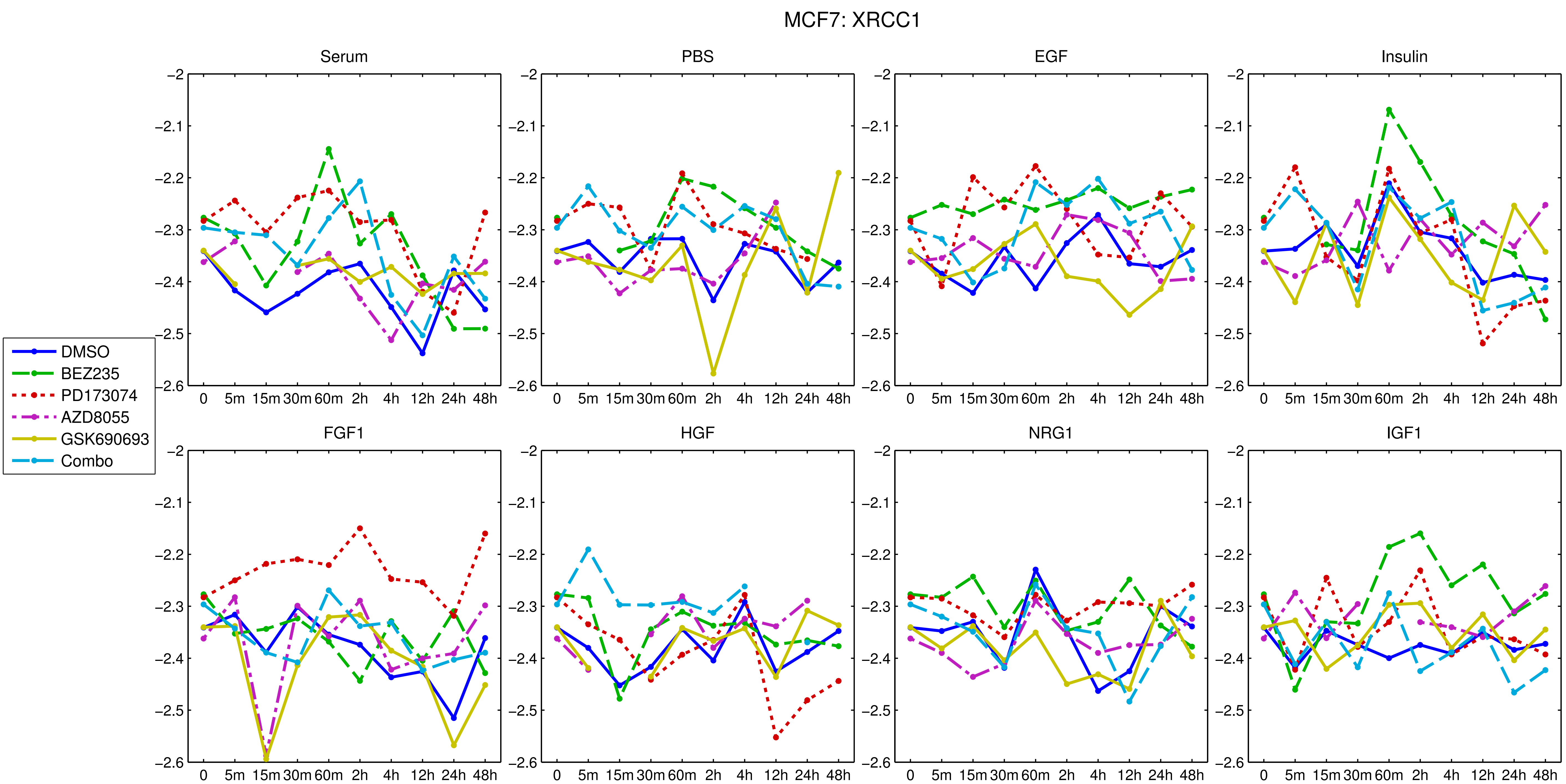
MCF7: XRCC1
Serum
PBS
EGF
Insulin
FGF1
HGF
NRG1
IGF1
−2
−2.1
−2.2
−2.3
−2.4
−2.5
−2.6
0
5m
15m
30m
60m
2h
4h
12h
24h
48h
DMSO
BEZ235
PD173074
AZD8055
GSK690693
Combo

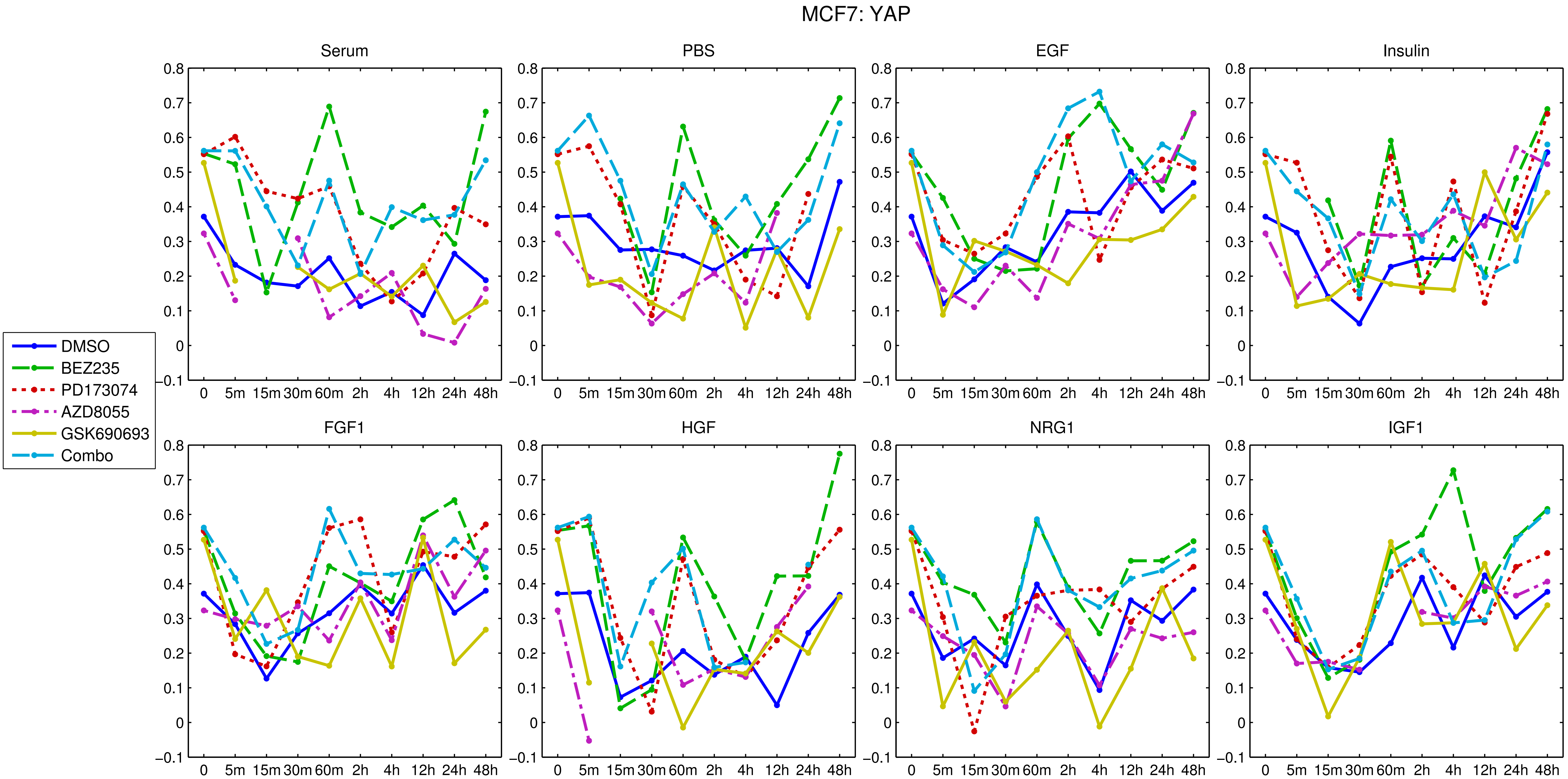
MCF7: YAP
Serum
PBS
EGF
Insulin
FGF1
HGF
NRG1
IGF1
DMSO
BEZ235
PD173074
AZD8055
GSK690693
Combo
0.8
0.7
0.6
0.5
0.4
0.3
0.2
0.1
0
−0.1
0
5m
15m
30m
60m
2h
4h
12h
24h
48h

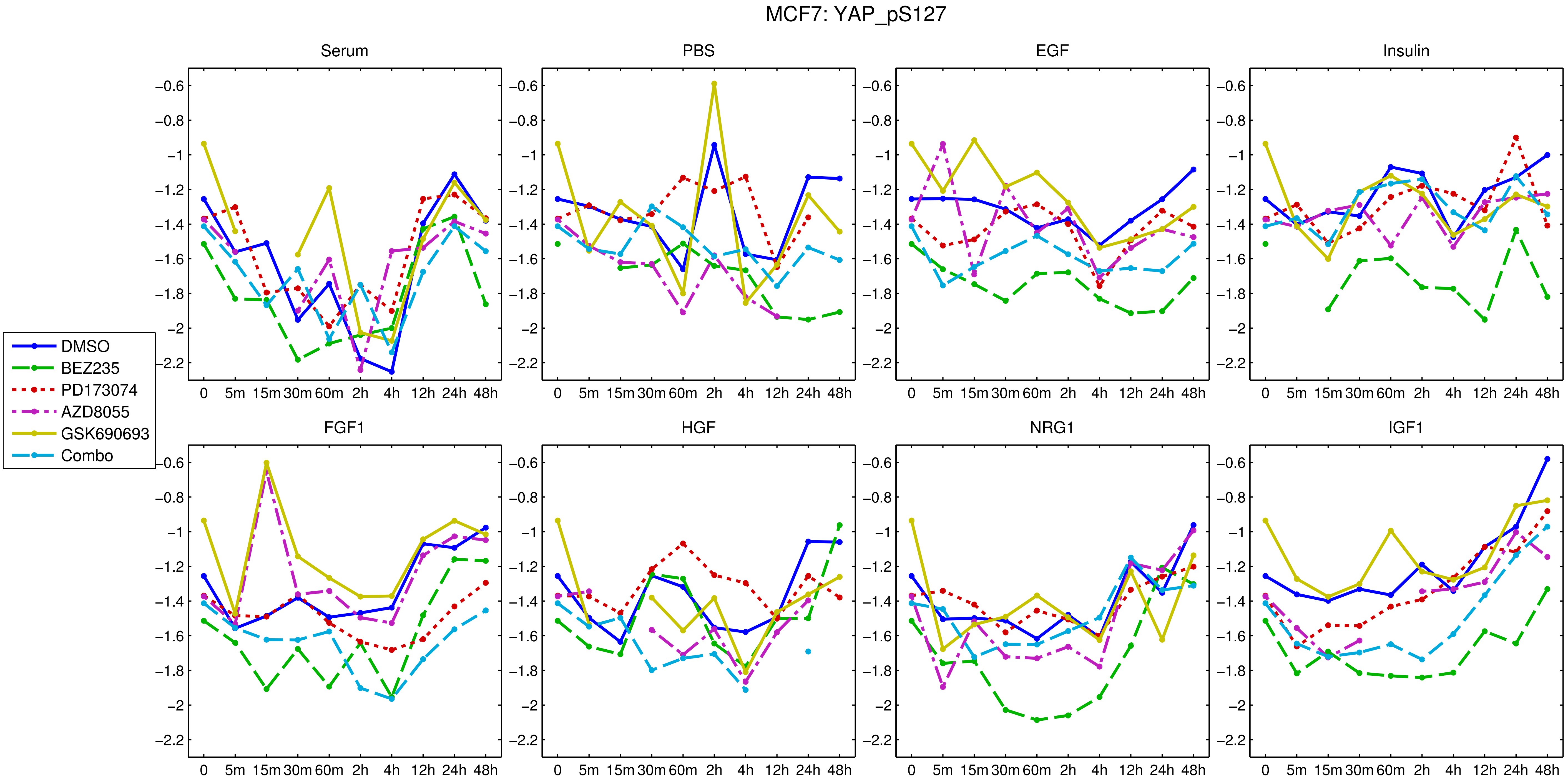
MCF7: YAP_pS127
Serum
PBS
EGF
Insulin
FGF1
HGF
NRG1
IGF1
DMSO
BEZ235
PD173074
AZD8055
GSK690693
Combo
−0.6
−0.8
−1
−1.2
−1.4
−1.6
−1.8
−2
−2.2
0
5m
15m
30m
60m
2h
4h
12h
24h
48h

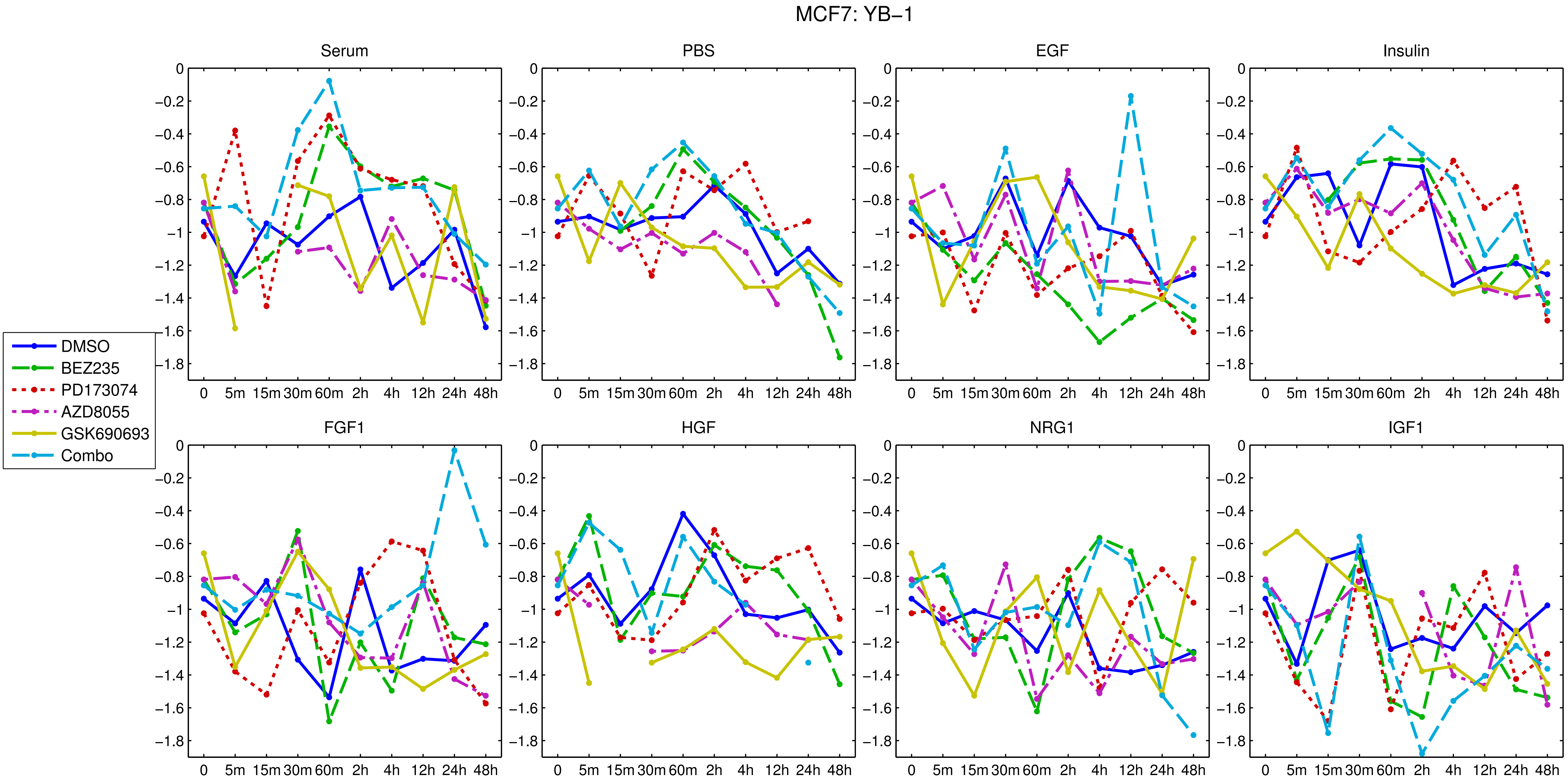
MCF7: YB−1
Serum
PBS
EGF
Insulin
FGF1
HGF
NRG1
IGF1
DMSO
BEZ235
PD173074
AZD8055
GSK690693
Combo
0
−0.2
−0.4
−0.6
−0.8
−1
−1.2
−1.4
−1.6
−1.8
0
5m
15m
30m
60m
2h
4h
12h
24h
48h

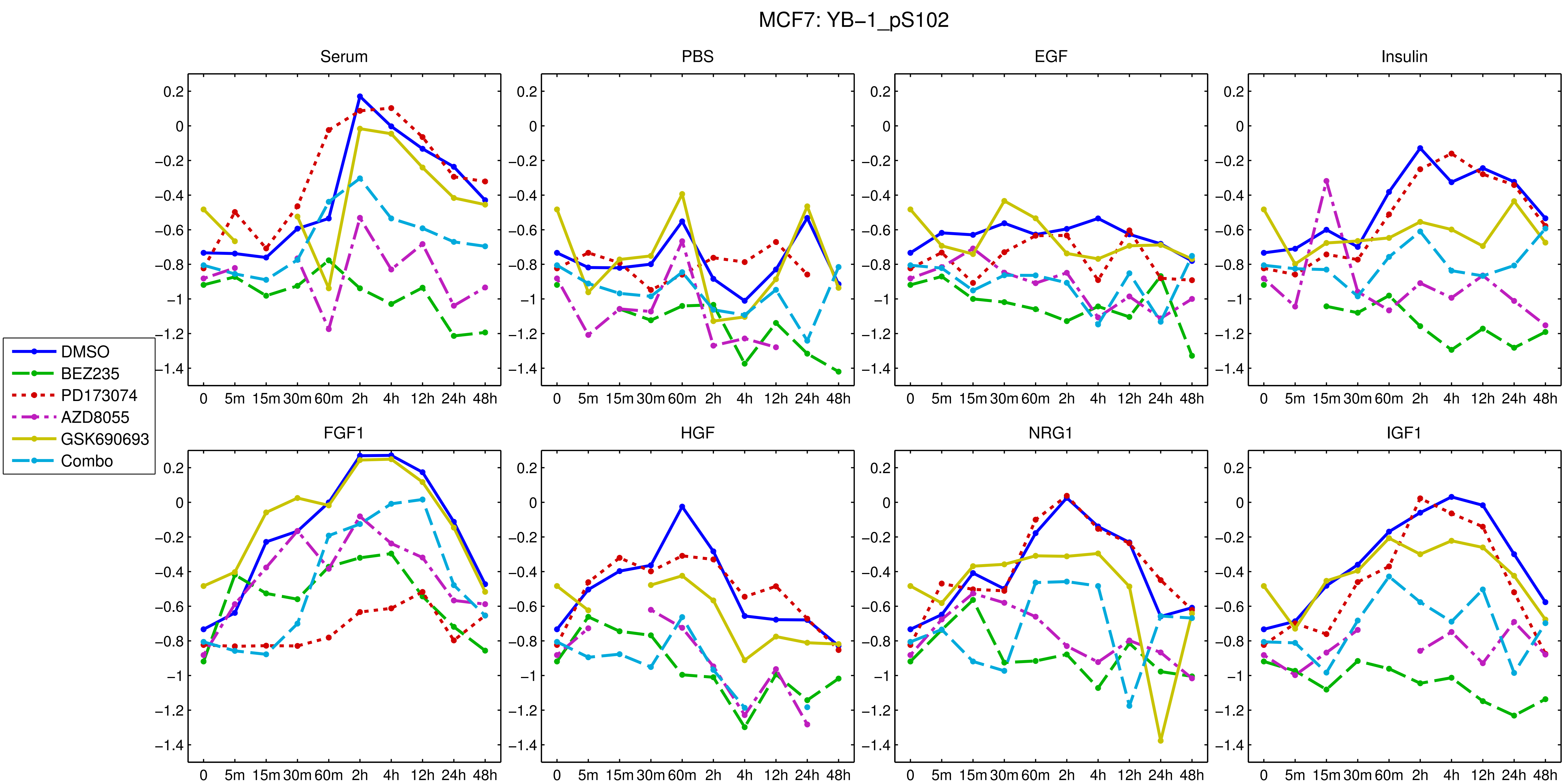
MCF7: YB−1_pS102
Serum
PBS
EGF
Insulin
FGF1
HGF
NRG1
IGF1
DMSO
BEZ235
PD173074
AZD8055
GSK690693
Combo
0.2
0
−0.2
−0.4
−0.6
−0.8
−1
−1.2
−1.4
0
5m
15m
30m
60m
2h
4h
12h
24h
48h
