# Supplementary material for: Context Specificity in Causal Signaling Networks Revealed by Phosphoprotein Profiling
Source: Cell Syst. 2017 Jan 25;4(1):73–83.e10. doi: 10.1016/j.cels.2016.11.013 (PMC5279869; doi:10.1016/j.cels.2016.11.013)
Supplement: Document S2. Article plus Supplemental Information [file mmc7.pdf]

Cell Systems

Article

Context Specificity in Causal Signaling Networks Revealed by Phosphoprotein Profiling

Graphical Abstract

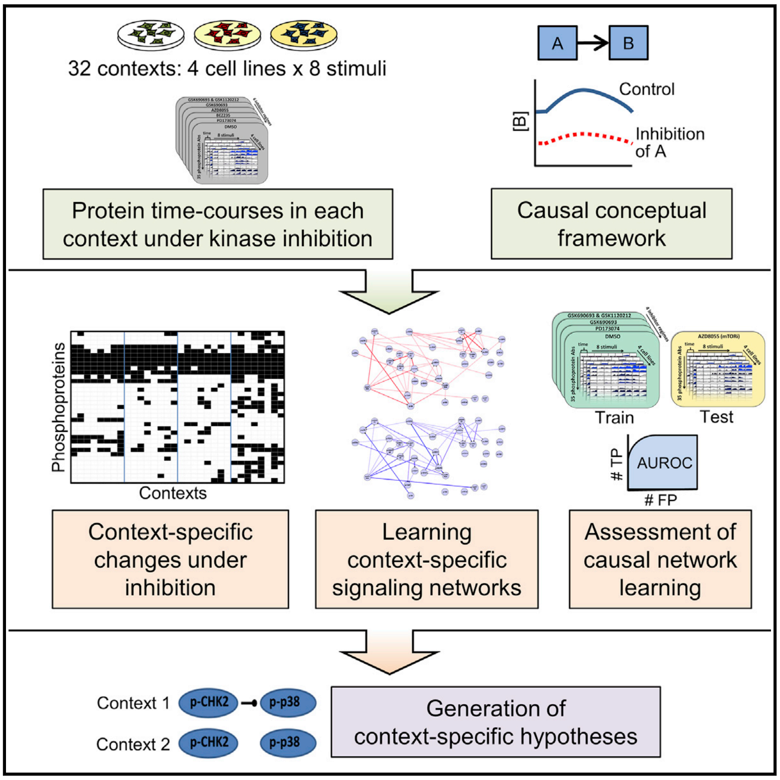

Authors

Steven M. Hill, Nicole K. Nesser, Katie Johnson-Camacho, ..., Gordon B. Mills, Sach Mukherjee, Paul T. Spellman

Correspondence

sach.mukherjee@dzne.de (S.M.), spellmap@ohsu.edu (P.T.S.)

In Brief

Time-course assays under kinase inhibition are used to investigate protein signaling networks in cancer cell lines within a causal conceptual framework. This reveals that patterns of causal influence between signaling proteins depend on the biological context. The data provide a resource for signaling biology and computational methods development.

Highlights

- Time-course assays of signaling proteins in cancer cell lines under kinase inhibition
- Causal conceptual framework for network analysis
- Data shed light on causal protein networks that are specific to biological context
- Resource for signaling biology and for benchmarking computational methods

Hill et al., 2017, Cell Systems 4, 73–83
January 25, 2017 © 2016 The Authors. Published by Elsevier Inc.
http://dx.doi.org/10.1016/j.cels.2016.11.013

Cell Systems
Article

OPEN ACCESS
CellPress

Context Specificity in Causal Signaling Networks Revealed by Phosphoprotein Profiling

Steven M. Hill,[1,12] Nicole K. Nesser,[2,12] Katie Johnson-Camacho,[2] Mara Jeffress,[3] Aimee Johnson,[4] Chris Boniface,[2] Simon E.F. Spencer,[5] Yiling Lu,[6] Laura M. Heiser,[7] Yancey Lawrence,[2,13] Nupur T. Pande,[8,9] James E. Korkola,[7] Joe W. Gray,[7,9,10] Gordon B. Mills,[6] Sach Mukherjee,[1,11,14,*] and Paul T. Spellman[2,15,*]
[1]MRC Biostatistics Unit, University of Cambridge, Cambridge CB2 0SR, UK
[2]Department of Molecular and Medical Genetics, Oregon Health and Science University, Portland, OR 97201, USA
[3]Kantar Health, Foster City, CA 94404, USA
[4]Bayer Healthcare North America, Berkeley, CA 94710, USA
[5]Department of Statistics, University of Warwick, Coventry CV4 7AL, UK
[6]Department of Systems Biology, MD Anderson Cancer Center, Houston, TX 77030, USA
[7]Department of Biomedical Engineering, Oregon Health and Science University, Portland, OR 97201, USA
[8]Department of Obstetrics and Gynecology, Women's Health Research Unit, Oregon Health and Science University, Portland, OR 97239, USA
[9]Knight Cancer Institute, Oregon Health and Science University, Portland, OR 97239, USA
[10]Center for Spatial Systems Biomedicine, Oregon Health and Science University, Portland, OR 97239, USA
[11]German Center for Neurodegenerative Diseases (DZNE), Bonn 53127, Germany
[12]Co-first author
[13]Present address: New York Medical College, Valhalla, NY 10595, USA
[14]Present address: German Center for Neurodegenerative Diseases (DZNE), Bonn 53127, Germany
[15]Lead Contact
*Correspondence: sach.mukherjee@dzne.de (S.M.), spellmap@ohsu.edu (P.T.S.)
http://dx.doi.org/10.1016/j.cels.2016.11.013

SUMMARY

Signaling networks downstream of receptor tyrosine kinases are among the most extensively studied biological networks, but new approaches are needed to elucidate causal relationships between network components and understand how such relationships are influenced by biological context and disease. Here, we investigate the context specificity of signaling networks within a causal conceptual framework using reverse-phase protein array time-course assays and network analysis approaches. We focus on a well-defined set of signaling proteins profiled under inhibition with five kinase inhibitors in 32 contexts: four breast cancer cell lines (MCF7, UACC812, BT20, and BT549) under eight stimulus conditions. The data, spanning multiple pathways and comprising ~70,000 phosphoprotein and ~260,000 protein measurements, provide a wealth of testable, context-specific hypotheses, several of which we experimentally validate. Furthermore, the data provide a unique resource for computational methods development, permitting empirical assessment of causal network learning in a complex, mammalian setting.

INTRODUCTION

The complexity of mammalian receptor tyrosine kinase (RTK) signaling continues to pose challenges for the understanding of physiological processes and aberrations that are relevant to disease. Networks, comprising nodes and linking directed edges, are widely used to summarize and reason about signaling. Obviously, signaling systems depend on the concentration and localization of their component molecules, so signaling events may be influenced by genetic and epigenetic context (Saez-Rodriguez et al., 2011; Good et al., 2009; Zalatan et al., 2012). In disease biology, and cancer in particular, an improved understanding of signaling in specific contexts may have implications for precision medicine by helping to explain variation in disease phenotypes or therapeutic response.

Genomic heterogeneity in disease has been well studied, notably in cancer, and heterogeneity is also manifested at the level of differential expression of components of signaling pathways downstream of RTKs (Akbani et al., 2014; Gerlinger and Swanton, 2010; Nickel et al., 2012; Szerlip et al., 2012). However, differences in average protein abundance (as captured in differential expression or gene set analyses) are conceptually distinct from differences in the edge structure of signaling networks, with the latter implying a change in the ability of nodes to causally influence each other. Causal relationships are also fundamentally distinct from statistical correlations: if there is a causal edge from node *A* to node *B*, then the abundance of *B* may be changed by inhibition of *A*, but *A* and *B* can be correlated with no causal edge linking them (see below for an illustrative example). For this reason, standard concepts from multivariate statistics (that in turn underpin many network analyses in bioinformatics) may not be sufficient for causal analyses (Pearl, 2009).

Canonical signaling pathways and networks (as described, for example, in textbooks and online resources) typically summarize evidence from multiple experiments, conducted in different cell types and growth conditions, and therefore, such networks are not specific to a particular context. Many well-known links in

CrossMark

This is an open access article under the CC BY license (http://creativecommons.org/licenses/by/4.0/).

such networks most likely hold widely, and so canonical networks remain a valuable source of insights. However, if causal signaling depends on context, then using canonical networks alone will neglect context-specific changes, with implications for reasoning, modeling, and prediction. A large literature has focused on the question of inferring molecular networks from data (for reviews, see De Smet and Marchal, 2010; Marbach et al., 2010). The potential for molecular networks to depend on context has motivated efforts to tailor network models in a data-driven manner (Marbach et al., 2016; Petsalaki et al., 2015; Will and Helms, 2016). Our approach is in this vein but with an emphasis on interventional data and a principled causal framework. Unbiased "interactome" approaches (e.g., Rolland et al., 2014) expand our view of the space of possible signaling interactions. However, due to the nature of genetic, epigenetic, and environmental influences, such approaches cannot in general identify signaling events specific to biological context (e.g., specific to a certain cell type under defined conditions).

We study context-specific signaling using human cancer cell lines. The data span 32 contexts, each defined by the combination of (epi)genetics (breast cancer cell lines MCF7, UACC812, BT20, and BT549) and stimuli. In each of the 32 (*cell line*, *stimulus*) contexts, we carried out time-course experiments using kinase inhibitors as interventions (note that as used here, the inhibitors do not contribute to defining the context). Reverse-phase protein arrays (RPPAs; Tibes et al., 2006) were then used to interrogate signaling downstream of RTKs. We used more than 150 high-quality antibodies targeting mainly total and phosphorylated proteins (see Table S1).

The inhibitors applied in each context allowed elucidation of context-specific causal influences between inhibited and downstream phosphoproteins. The extent of context specificity seen can be summarized as follows: on average, across all kinase inhibitors and pairs of contexts in the study, approximately one in five phosphoproteins show changes in abundance under inhibition in one context that are not seen in the other. We also modeled the data using recently developed methods rooted in probabilistic graphical models to reconstruct context-specific networks intended to capture causal interplay between all measured phosphoproteins (and not just interplay related to inhibited nodes).

Thus, we show that causal signaling networks depend on context, with the pattern of changes under inhibition dependent on biological background. This is supported by independent validation experiments. Furthermore, we advance a conceptual view of signaling networks as causal networks (Pearl, 2009). In addition, this paper adds to available resources in two ways. First, it provides a rich data resource, spanning all combinations of context, inhibitor, and time and allowing for a very wide range of analyses, including, but not limited to, analyses of the kind presented here. The data complement available patient datasets (see, for example, Akbani et al., 2014) by providing interventional readouts under defined conditions and provide a wealth of testable hypotheses regarding potentially novel and context-specific signaling links. Second, the data serve as a resource for computational biology benchmarking. Network reconstruction has long been a core topic in computational biology, but performance with respect to learning of causal links has mainly been benchmarked using simulated data that may not adequately reflect the challenges of real data and relevant biology. A previous study established a small, five-node synthetic network in yeast that was valuable to the computational biology community, as it provided a gold-standard network in a biological model (Cantone et al., 2009). The design of our experiments allows for systematic testing of causal network learning in a complex mammalian setting and provides a unique resource for development of computational biology methods. The data presented here were used in the recent HPN-DREAM (Heritage Provider Network-Dialogue for Reverse Engineering Assessment and Methods) network inference challenge. The challenge focused on causal networks, and the data were used to score more than 2,000 submitted networks (full details of the challenge are described in Hill et al., 2016).

RESULTS

Causal Molecular Networks and Context Specificity

We first define causal molecular networks at a conceptual level. Consider a specific cell line grown under defined conditions. We refer to the complete biological setting (including genetic/epigenetic background and growth/environmental conditions) as the context c. If, in this setting, we observe a change in molecule B under inhibition of molecule A, we can conclude that there exists a causal pathway (i.e., a sequence of mechanistic events, possibly involving additional molecular species) between A and B in context c. Conceptually, performing all possible inhibition experiments on a set of molecules (including in combinations) would allow construction of a directed network G_c, with nodes corresponding to the molecules and edges encoding causal relationships between nodes. Specifically, an edge in G_c indicates that in context c, inhibition of the parent node can lead to a change in the child node that is not mediated via any other node in the network. We refer to G_c as the context-specific causal network and to edges therein as causal edges (Figure 1A).

Due to the large number of potentially relevant molecular species, it is likely that in any specific study, there will be variables that are unmeasured but that nonetheless have a causal influence on one or more measured variables. Suppose there is no causal pathway between A and B, but the nodes are correlated due to co-regulation by an unobserved node C that is not represented in the graph (Figure 1B). Then, since inhibition of A would not be capable of changing B, an edge from A to B would not be contained in the ground truth network G_c as defined above, regardless of the strength of any correlation or statistical dependence between A and B (Figure 1C). A contrasting case is that of a missing variable that is intermediate in a causal pathway, e.g., if A influences B via an unmeasured molecule C. Then, using the definition above, we would consider the edge $A \rightarrow B$ to be a correct representation of the causal influence. However, if C were observed, the correct model would be $A \rightarrow C \rightarrow B$ (Figure 1C). Thus, the definition we use is compatible with missing variables while correctly encoding the effect of interventions on observed nodes, but the edges are not intended to encode physically direct influences only. We note that there are many subtle and still open aspects of the epistemology of interventions and causation; for a wider discussion, see Woodward (2016).

The definition of causal molecular networks above is rooted in changes under inhibition but is not restricted to any particular

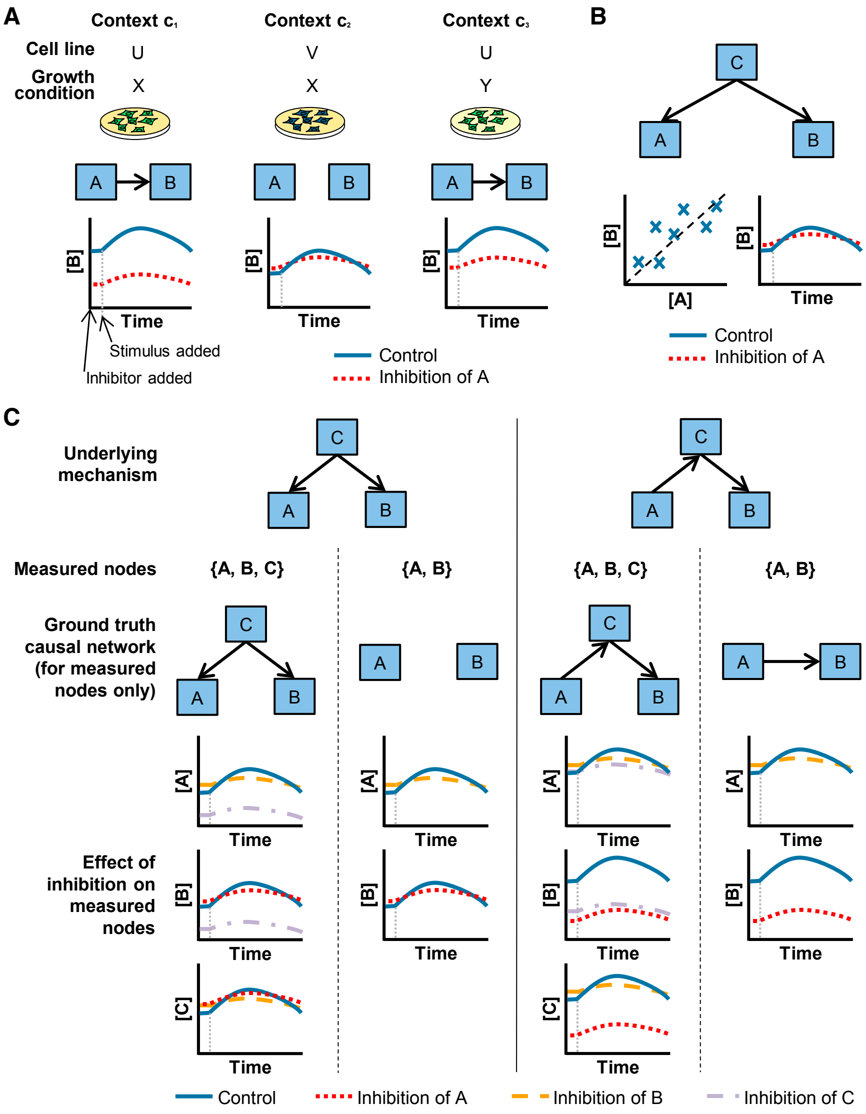

Figure 1. Context-Specific Causal Networks

(A) Context-specific causal influences. Node *A* has a causal influence on node *B* in contexts c_1 and c_3, but not c_2, encoded by the presence of a causal edge between *A* and *B* in c_1 and c_3 only. This reflects the outcome of experiments where *A* is inhibited. Here, each context is defined by the combination of cell line and growth condition.

(B) Correlation and causation. The abundance of node *A* is correlated with that of node *B* due to regulation by the same node *C*. However, as there is no causal influence (direct or indirect) of *A* on *B*, inhibition of *A* does not result in a change in the abundance of *B*, no matter how strong the correlation or statistical dependence.

(C) Causal networks and missing nodes. In the first example, node *C* regulates both nodes *A* and *B* (as in panel B). In the formulation used here, if *C* is not observed and not included in the network, but *A* and *B* are, we would regard the network with no edge between *A* and *B* in either direction as the correct or ground truth causal network, in line with the results of experimental inhibition of these nodes, as shown. In the second example, the underlying mechanism is that *A* influences *C*, and *C* in turn influences *B*. In the formulation used here, if *C* is not measured and not included in the network, an edge from *A* to *B* would be regarded as correct, in line with the results of experimental inhibition of the nodes. However, if all three nodes were included, the correct network would match the underlying mechanism. Although abundance of *B* changes under inhibition of *A*, an edge from *A* to *B* would be regarded as incorrect here because the influence of *A* on *B* is fully mediated via another network node (i.e., *C*). See text for further details of the causal formulation and its interpretation.

mechanism. We focus on kinase inhibitors, phosphoprotein nodes, and relatively short-term changes (up to 4 hr after inhibition), and to that extent, our focus is on signaling, but we note that changes seen in our data could be due to a number of mechanisms, including transcription, translation, or protein stability. In considering causal influences, it is important to specify a relevant time frame, because under the same intervention, different changes may occur over different time periods (see also Discussion). Note also that even if one assumes a very large sample size and neglects statistical issues entirely, a notion of magnitude (of change under inhibition) remains implicit in the network definition itself and influences the sparsity of the ground truth network.

Overview of Approach

We sought to investigate causal signaling networks in specific biological contexts. We considered four breast cancer cell lines (MCF7, UACC812, BT20, and BT549) derived from distinct epigenetic states and harboring different genomic aberrations (these cell lines have been extensively characterized; see Barretina et al., 2012; Garnett et al., 2012; Heiser et al., 2012; Neve et al., 2006). Each cell line was serum starved for 24 hr and then at time t = 0 min stimulated with one of eight different stimuli (Figure 2A). For each (*cell line*, *stimulus*) context, we carried out RPPA time-course assays comprising a total of seven time points spanning 4 hr and under five different kinase inhibitors plus DMSO as a control (Figure 2A; STAR Methods; the assays included additional, later time points that were not used in our analyses but are available in Data S1). To ensure that targets of the kinase inhibitors were effectively blocked, cells were treated with inhibitors for 2 hr before stimulus. Low concentrations of each inhibitor were used to minimize off-target effects (see STAR Methods). Due to the functional significance of phosphorylation, the analyses presented below focus on the 35 phosphoproteins that were measured in all cell lines (see STAR Methods and Table S1; Data S1 contains measurements for all antibodies). Context-specific changes under intervention were summarized as "causal descendancy matrices" (Figure 2B; see below). Machine learning methods were used to integrate the interventional data with known biology to reconstruct context-specific signaling networks (Figure 2C).

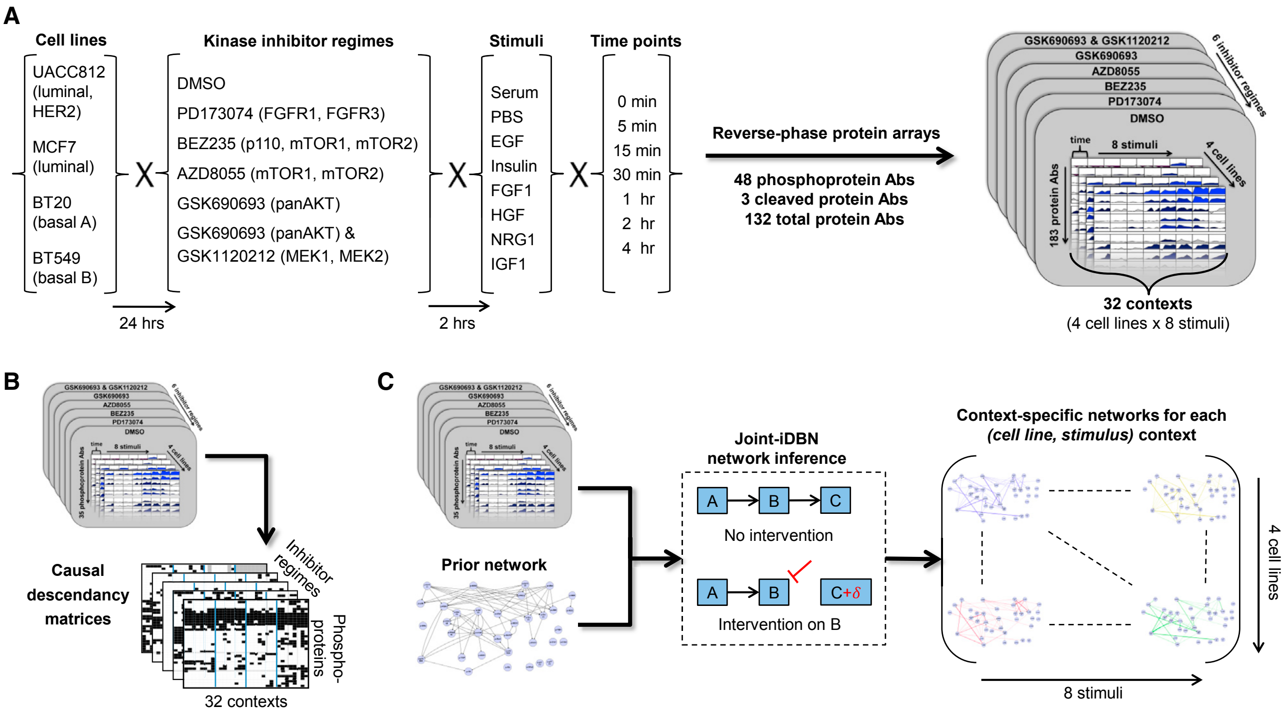

Figure 2. Data-Driven Reconstruction of Context-Specific Causal Signaling Networks

(A) Overview of experimental approach. Reverse-phase protein arrays (RPPAs) were used to investigate protein signaling in four human breast cancer cell lines under eight different stimuli. The combinations of cell line and stimulus defined 32 (*cell line*, *stimulus*) contexts. Prior to stimulus, cell lines were serum starved and treated with kinase inhibitors or DMSO control. RPPA assays were performed for each context at multiple time points post-stimulus, using more than 150 high-quality antibodies to target specific proteins, including ~40 phosphoproteins (the precise number of antibodies varies across cell lines; see STAR Methods and Table S1).

(B) Causal descendancy matrices (CDMs). CDMs summarizing changes under intervention across all contexts were constructed for each intervention (see text for details).

(C) Overview of causal network learning procedure. Interventional time-course data for each context were combined with existing biological knowledge in the form of a prior network to learn context-specific phosphoprotein signaling networks. Networks were learned using a variant of dynamic Bayesian networks designed for use with interventional data and that allowed joint learning over all 32 contexts at once (see STAR Methods).

Interventional Time-Course Data Specific to Biological Context

Comparing time-course data between inhibitor and control (DMSO) experiments allowed us to detect changes to phosphoprotein nodes caused by kinase inhibition (see STAR Methods for details). These changes are visualized in a global manner for cell line MCF7 in Figure 3B, with DMSO time courses shown in Figure 3A. In Figure 3B, the color coding indicates direction of effect (see examples in Figure 3C): green indicates a decrease under inhibition relative to control (consistent with positive regulation) and red an increase under inhibition (consistent with negative regulation). Corresponding visualizations for UACC812, BT20, and BT549 are shown in Figure S1.

Many effects, including many classical ones, are not stimulus dependent. For example, phospho-p70S6K is reduced relative to control under mTOR inhibition (inhibitor AZD8055; Figure 3C), in line with the known causal role of mTOR in regulating phosphorylation of p70S6K. Since mTOR signaling is already active in serum starved cells, the reduction in phospho-p70S6K under mTOR inhibition is seen at all time points, including t = 0 min (recall that the inhibitor is applied prior to stimulus). However, some changes under intervention are specific to individual stimuli. Some of these effects can be readily explained, such as the reduction in abundance of several phosphoproteins in the AKT and mitogen-activated protein kinase (MAPK) pathways under fibroblast growth factor receptor (FGFR) inhibition (inhibitor PD173074) for cell line MCF7 stimulated with FGF1. Other stimulus-specific changes are less expected, including the decrease in abundance of phospho-AKT (phosphorylated at threonine 308) in cell line MCF7 under inhibition of mTOR and phosphatidylinositol 3-kinase (PI3K) (inhibitor BEZ235) that is observed in only four of the stimuli.

Causal Descendancy Matrices Summarize Changes under Intervention across Multiple Contexts

Changes seen under inhibition of mTOR (catalytic inhibitor AZD8055) are summarized in Figure 4A (with phosphoproteins in rows and the 32 contexts in columns). Here, a filled-in box for phosphoprotein p in context c indicates a salient change under mTOR inhibition (see STAR Methods), consistent with a causal influence of mTOR on phosphoprotein p in context c. This could occur via a causal pathway involving other (measured or unmeasured) nodes. In other words, an entry in location (p,c) in the matrix indicates that phosphoprotein p is a *descendant* of mTOR in

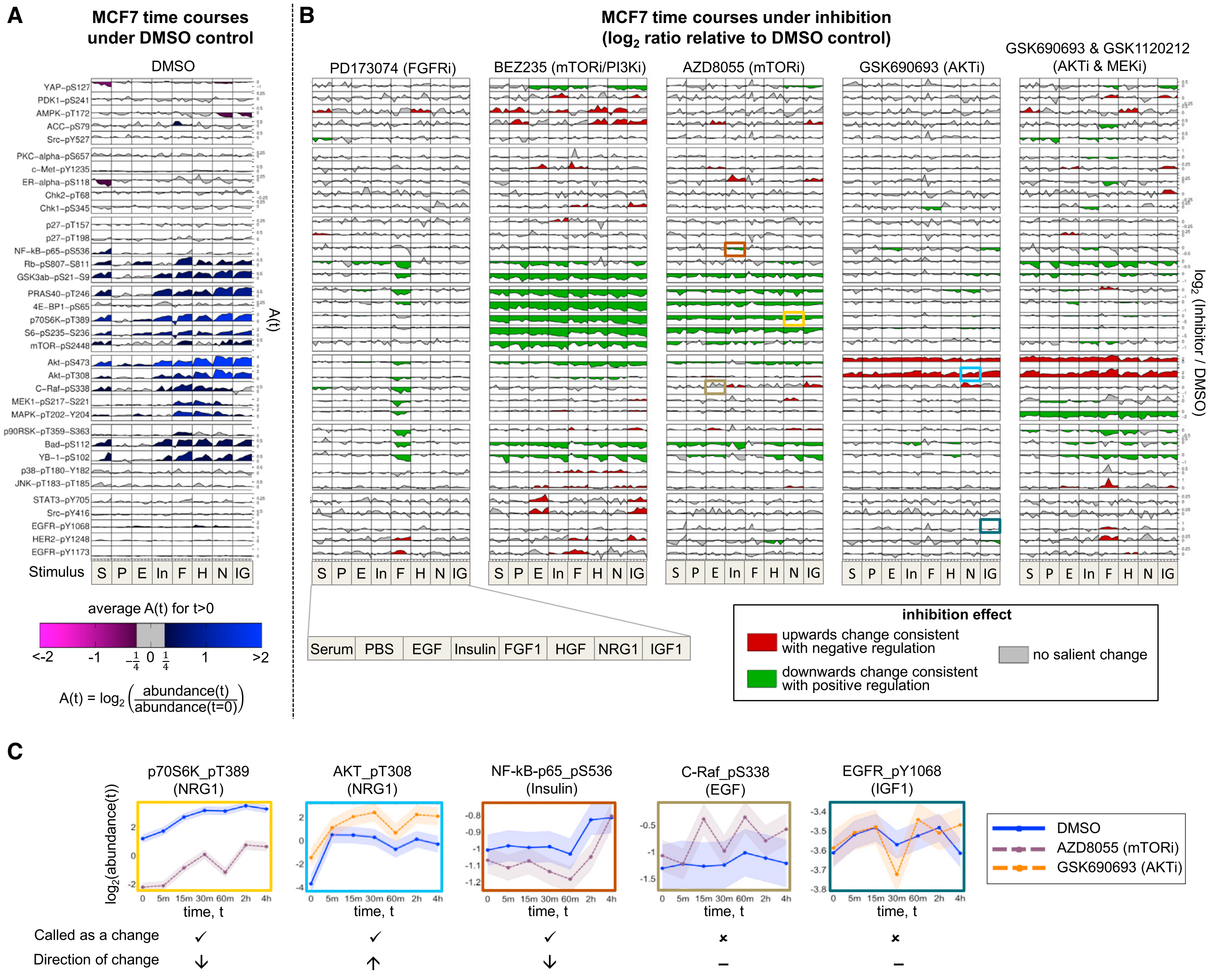

Figure 3. Phosphoprotein Time-Course Data and Context-Specific Changes under Inhibition for Breast Cancer Cell Line MCF7

(A) Phosphoprotein time courses under DMSO control. Rows correspond to 35 phosphoproteins (a subset of the full set of 48; see STAR Methods for details) and columns correspond to the eight stimuli. Each time course shows $\log_2$ ratios of phosphoprotein abundance relative to abundance at $t = 0$. Shading represents average $\log_2$ ratio for $t > 0$.

(B) Phosphoprotein time courses under kinase inhibition. Each of the five vertical blocks corresponds to a different inhibition regime. Within each block, rows and columns are as in (A). Each time course shows $\log_2$ ratios of phosphoprotein abundance under inhibition relative to abundance under DMSO control. Shading represents direction of changes in abundance due to inhibitor: Green denotes a decrease in abundance, red denotes an increase and gray denotes no salient change (see examples in C). See STAR Methods for details of statistical analysis. For both (A) and (B), plots were generated using a modified version of the DataRail software (Saez-Rodriguez et al., 2008). Each phosphoprotein is plotted on its own scale, and phosphoproteins are ordered by hierarchical clustering of all data. See Figure S1 for corresponding plots for cell lines UACC812, BT20, and BT549.

(C) Selected examples from (B) showing control (DMSO) and inhibitor time courses separately; box color identifies the source cell in (B). Examples are shown for (from left to right) a clear decrease in abundance, a clear increase in abundance, a decrease in abundance that is borderline under the criteria we use, a borderline case called negative (i.e., called as no change), and a clear negative case. Shaded region indicates time-averaged replicate SD. See also Figure S2.

the causal network G_c for context c; we therefore refer to this matrix as a causal descendancy matrix for mTOR. For comparison, an additional column shows proteins that are descendants of mTOR according to a canonical signaling network (Figure 4B; STAR Methods). Many classical signaling links are conserved across cell lines and stimuli, but there are also many examples of influences that are both non-canonical and context-specific. For example, phospho-p38 is elevated in UACC812 cells treated with the mTOR inhibitor AZD8055 under serum stimulation, whereas there is no change in BT549 cells under the same conditions. Similarly, we obtained causal descendancy matrices for each of the other inhibitors in our study (Figure S3). On average across all kinase inhibitors and pairs of contexts, 8 out of 35 phosphoproteins show salient changes under inhibition in one context that are not seen in the other (mean number of differences = 8.14). Considering only pairs of cell lines under the same stimulus, the mean number of differences is 8.58, while considering pairs of stimuli for the same cell line, the corresponding value is 6.38. This suggests that the differences in (epi)genetic background between the cell lines have a relatively pronounced effect.

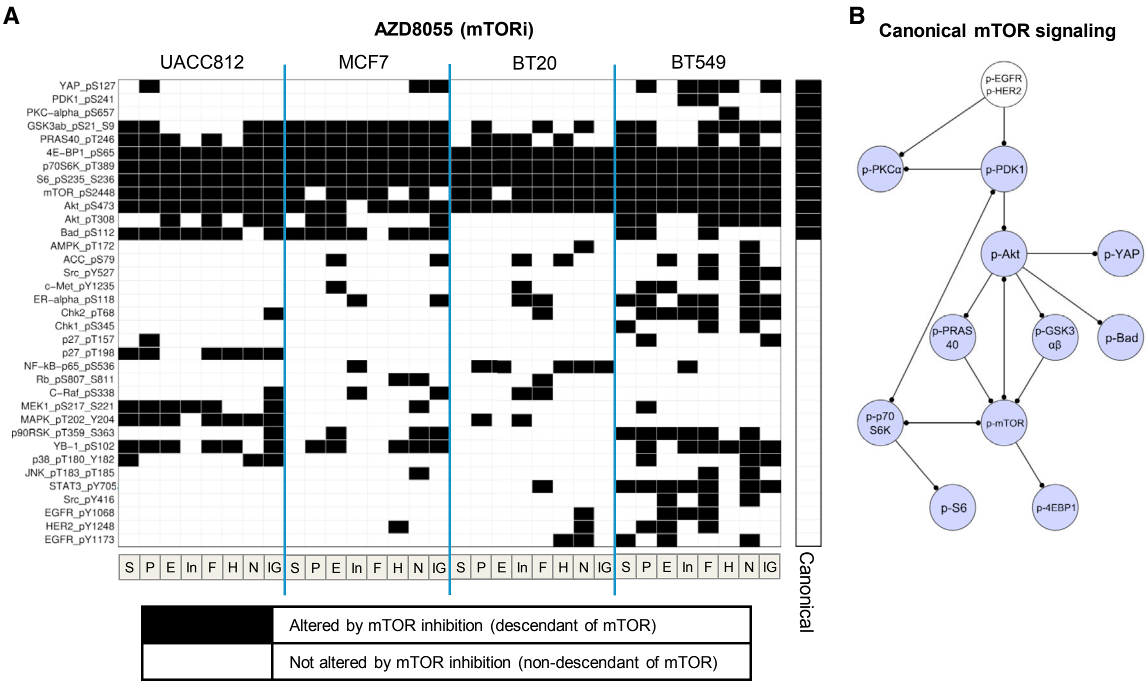

C

Cell Lines	Inhibitors	AZD8055 (mTORi)			BEZ235 (mTORi/PI3Ki)				GSK690693 (AKTi)			PD173074 (FGFRi)				Totals for each cell line: Validated / Tested (% Validated)
	Stimulus	HGF	NRG1	Serum	HGF	Insulin	NRG1	Serum	EGF	NRG1	Serum	FGF	HGF	NRG1	Serum	
UACC812	Validated	0	1	2	1	1	1	4	0	1	4	0	4	3	3	25/31 (81%)
	Tested	1	2	2	2	1	2	5	0	1	5	0	4	3	3	
BT20	Validated	0	0	0	4	1	3	5	1	1	3	0	6	4	2	30/40 (75%)
	Tested	0	0	0	4	1	5	8	1	2	4	1	6	5	3	
MCF7	Validated	0	1	0	4	1	5	7	0	0	3	1	1	4	1	28/32 (88%)
	Tested	0	2	0	4	1	5	7	1	1	3	1	1	5	1	
BT549	Validated	1	1	1	2	1	4	4	0	1	3	0	1	0	2	21/31 (68%)
	Tested	1	2	2	3	1	4	6	1	2	4	0	1	2	2	
Totals for each inhibitor: Validated / Tested (% Validated)		7/12 (58%)			48/59 (81%)				17/25 (68%)			32/38 (84%)				Overall total: 104/134 (78%)

Figure 4. Non-canonical and Context-Specific Signaling

(A) Causal descendancy matrix showing causal effects observed under mTOR inhibitor AZD8055 in each of the 32 (cell line, stimulus) contexts. Rows represent phosphoproteins and columns represent contexts (see Figure 3). Black boxes indicate phosphoproteins that show a salient change under mTOR inhibition in a given context (see STAR Methods) and can therefore be regarded as causal descendants of mTOR in the signaling network for that context. The final column on the right indicates phosphoproteins that are descendants of mTOR in the canonical mTOR signaling pathway shown in (B). Phosphoproteins are ordered first by canonical column and then by hierarchical clustering of all data. See Figure S3 for causal descendancy matrices for the other inhibitor regimes.

(B) Canonical mTOR signaling pathway. Blue nodes are descendants of mTOR in the network, and white nodes are non-descendants. The pathway shown is a subnetwork of the prior network used within the network inference procedure (Figure S4). Full nodes names, including phosphorylation sites, are provided in Table S4.

(C) Summary of western blot validations of causal effects observed in RPPA data. A number of observations from the causal descendancy matrices were chosen for validation via western blot analysis. The number of phosphoprotein validations attempted (“Tested”) and the number of these that successfully validated (“Validated”) are presented for various (*cell line*, *stimulus*, *inhibitor*) combinations. Summary totals are also presented for each cell line, each inhibitor, and across all validation experiments. See also Table S2.

We sought to validate some of the observed causal effects by western blot analysis (STAR Methods). Observations were selected for validation across both inhibitors and antibodies, and included instances of increase and decrease under inhibition, as well as instances where no effect was observed (Table S2). A summary of the number of observations tested for each cell line and inhibitor regime and of validation success rate in independent experiments (i.e., new lysates) is shown in Figure 4C. Overall, we validated 78% of observations tested (104 out of 134 observations). There were 25 (*antibody*, *inhibitor*) combinations that for the same stimulus showed differing effects across cell lines in the RPPA data (and which were also tested by western blotting); 17 of these instances of heterogeneity across cell lines validated (68%). The corresponding validation rate for (*antibody*, *inhibitor*) combinations that for the same cell line showed differing effects across stimuli was only 3 out of 13 (23%). Failures to validate could represent biological variability, differential sensitivity between RPPA and western blotting, use of different antibodies, or other technical issues.

Machine Learning of Signaling Networks

We used dynamic Bayesian networks to learn context-specific causal networks over all measured phosphoprotein nodes (including those not intervened upon). To do so, we exploited

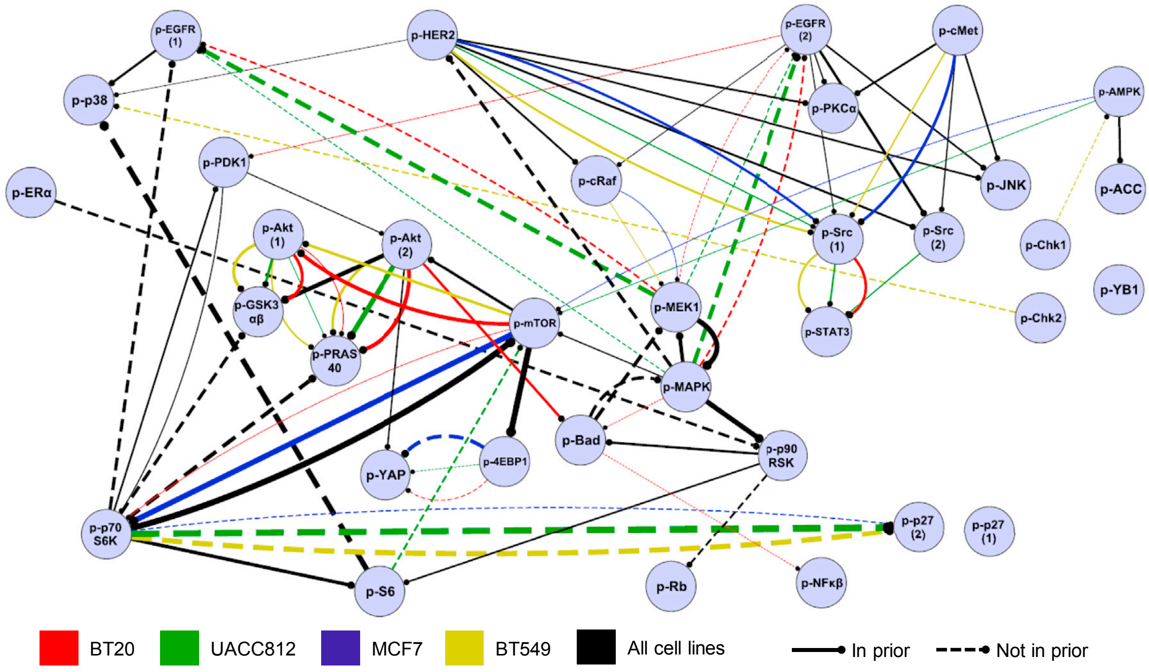

Figure 5. Context-Specific Signaling Networks Reconstructed Using a Machine Learning Approach

Data for 35 phosphoproteins were analyzed using a machine learning approach based on interventional dynamic Bayesian networks, integrating also known biology in the form of a prior network (Figure S4). This gave a set of scores (edge probabilities) for each possible edge in each (*cell line*, *stimulus*) context (see STAR Methods). For each cell line, a summary network was obtained by averaging edge probability scores across the eight stimulus-specific networks for that cell line. Edge color denotes cell line. Only edges with average probabilities greater than 0.2 are shown. A black edge indicates an edge that appears (i.e., is above the 0.2 threshold) in all four cell lines. Edge thickness is proportional to the average edge probability (average taken across all 32 contexts for black edges). Solid or dashed edges were present or not present in the prior network, respectively. Edges are directed with the child node indicated by a circle. Edge signs are not reported; the modeling approach does not distinguish between excitatory and inhibitory causal effects. Full node names, including phosphorylation sites, are provided in Table S4. Network visualized using Cytoscape (Shannon et al., 2003). See also Table S3.

several recent methodological advances that allow integration of interventional data and simultaneous network learning across multiple related problem instances (here, contexts; see STAR Methods and references therein for details). Known biology was incorporated using a prior network (Figure S4).

Figure 5 summarizes networks across all contexts by averaging across the eight stimulus-specific networks for each of the four cell lines. We see that while many edges, including several classical ones, are near universal, others are cell line specific, mirroring, via a global analysis, the inhibition data reported above (Figure 4A). The networks contained edges included in the prior network as well as many edges that were not. Across the 32 contexts, networks contained an average of 49 edges (at a threshold of 0.2 applied to the edge probabilities that are the output of the learning procedure) and, on average, 40% of edges in each network were not in the prior network (Table S3). We discuss potentially novel edges that were not in the prior below. As discussed in Hill et al. (2016), the challenging nature of causal network learning means that empirical performance assessment is important. We used an extended variant of the train-and-test procedure described in Hill et al. (2016) to systematically assess causal network learning (see STAR Methods). We found that the models were able to achieve significant agreement with unseen test interventional data in most of the contexts (Figure S5). However, we note that empirical assessment is a frontier topic in causal inference, and the assessment procedure used here is subject to a number of caveats (see Discussion).

Validation of Context-Specific Signaling Hypotheses

We identified 235 edges in the inferred networks that were not in the prior network. These potentially novel edges shared 35 parent proteins, 4 of which were inhibited in the original dataset. Five edges with parent nodes not among those inhibited in the original RPPA data were selected for validation by western blot. Edge selection was done on the basis of biological interest and availability of sufficiently specific inhibitors for the parent nodes (Figure 6). We note that our computational approach predicts presence/absence of each (directed) edge, but not sign (activating or inhibiting).

For each of the five edges, we tested contexts in which the edge was predicted as well as those in which the edge was not predicted. We inhibited the parent node and observed whether this altered abundance of the predicted child node. We found evidence supporting each of the five predicted causal edges, but with often-complex context dependence. These results, and their agreement and disagreement with context-specific predictions from network modeling, are summarized in Figures 6F and 6G.

An edge from Chk2_pT68 to p38_pT180/Y182 (for phosphoproteins, we give the protein name before an underscore, which is followed by the phosphorylation site or sites) was predicted

OPEN
ACCESS
CellPress

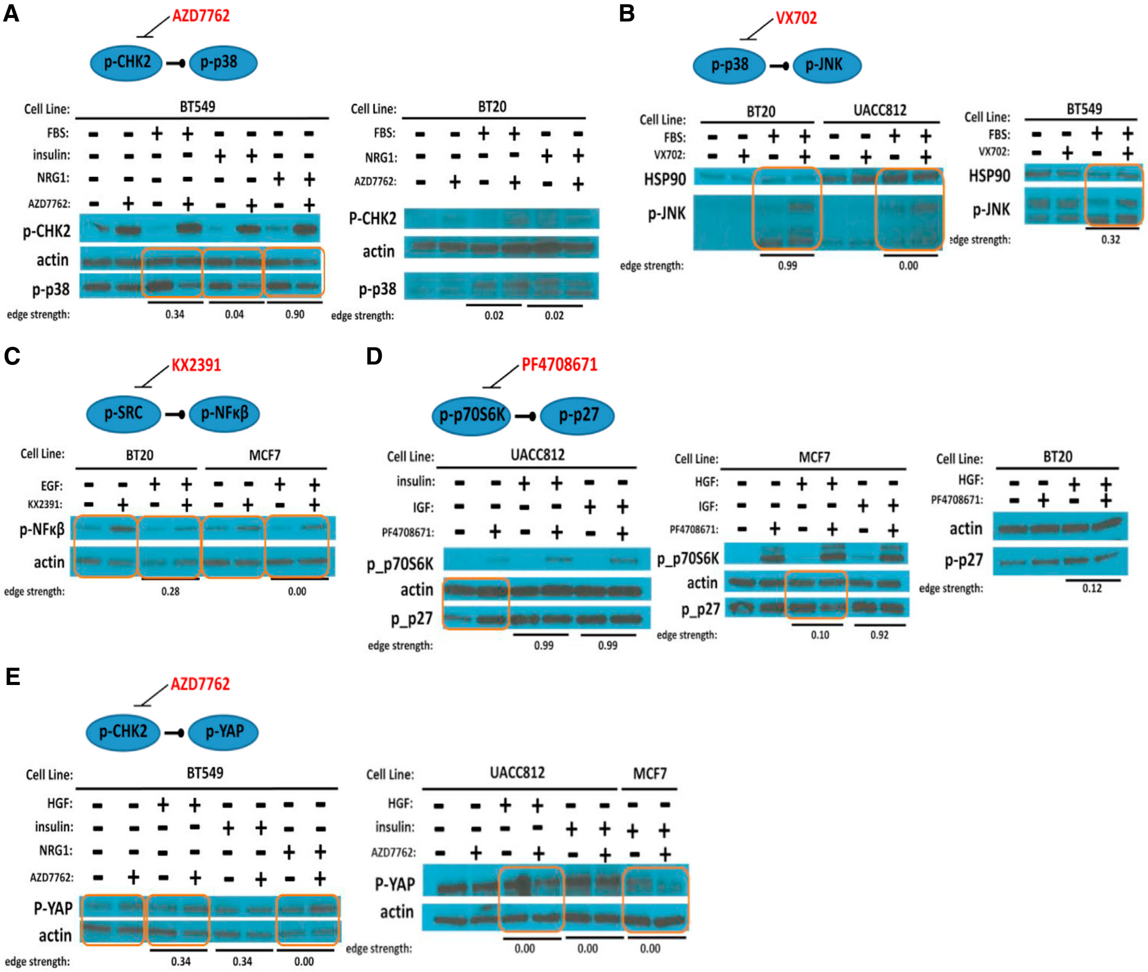

F

Predicted edge		BT20		BT549		MCF7		UACC812	
Parent node	Child node	- stimuli	+ stimuli	- stimuli	+ stimuli	- stimuli	+ stimuli	- stimuli	+ stimuli
p-Chk2	p-p38	NE	NE	NE	→	NA	NA	NA	NA
p-p38	p-JNK	NA	⊣	NE	⊣	NA	NA	NE	⊣
p-Src	p-NFκB	⊣	⊣	NA	NA	⊣	⊣	NA	NA
p-p70S6K	p-p27	NE	NE	NA	NA	NE	→	⊣	NE
p-Chk2	p-YAP	NA	NA	⊣	⊣	NA	→	NE	→

Symbols are colored orange in "+ stimuli" settings where an edge was predicted by our networks

G

	Predicted edge				
Parent node:	p-Chk2	p-p38	p-Src	p-p70S6K	p-Chk2
Child node:	p-p38	p-JNK	p-NFκB	p-p27	p-YAP
Edge validated in at least one predicted context	✓	✓	✓	✗	✓
Validation experiment shows cell line-specificity of edge	✓	✗	✗	✓	✓
Validation experiment shows stimulus-specificity of edge	✗	n/a	n/a	✓	✓

Figure 6. Validation of Novel Network Edges

Western blot analysis of selected context-specific network edges that were not in the prior network. (A–E) Edges tested were (A) phospho-Chk2 to phospho-p38, (B) phospho-p38 to phospho-JNK, (C) phospho-Src to phospho-NF-κB, (D) phospho-p70S6K to phospho-p27, and (E) phospho-Chk2 to phospho-YAP. Orange boxed areas indicate observed changes in abundance of the predicted child node under inhibition of the parent node in a single *(cell line, stimulus)* context (changes in abundance are determined by visual inspection of the bands). Edge probabilities output by the network learning procedure are shown for each context tested ("edge strength").

(F) A summary of the validation experiments. NA denotes not applicable (the experiment was not run), and NE denotes no effect (there was no change in child node abundance upon inhibition of the parent node). An arrow indicates results consistent with an activating parent node. A stunted line represents results consistent with an inhibitory edge. Symbols are colored orange to indicate that an edge was predicted for the corresponding cell line under one of the stimuli tested.

(legend continued on next page)

only in cell line BT549 (Figure 5). We inhibited Chk2 with AZD7762 in BT549 cells and saw decreases in phospho-p38 under serum (fetal bovine serum [FBS]) and neuregulin (NRG)1, where the edge was predicted, as well as under insulin, where the edge was not predicted (Figure 6A). In contrast, there is no change in phospho-p38 in BT20 cells under AZD7762 treatment, consistent with the absence of the edge in the BT20 networks. Here, we see that the edge validates in a cell line-specific, but not stimulus-specific, manner. However, it is important to note that AZD7762 inhibits Chk1 and Chk2 with equal potency and also demonstrates activity, albeit lower, against other kinases.

The networks predicted an edge from p38_pT180/Y182 to JNK_pT183/T185 in BT549 and BT20 cells under stimulus with FBS. We inhibited p38 with VX702 in BT549, BT20, and UACC812 cells stimulated with FBS. In line with network predictions, we observed an increase in phospho-JNK in BT549 and BT20 cells (Figure 6B), but we also observed a modest increase in phospho-JNK in UACC812 cells, where the edge was not predicted.

An edge from Src_p416 to nuclear factor (NF)-κB-p65_pS536 was predicted only in BT20 cells stimulated with epidermal growth factor (EGF). Upon inhibition of Src with KX2391 both before and after stimulation with EGF, an increase in the abundance of phospho-NF-κB was observed in BT20 cells, consistent with the presence of a causal link (Figure 6C). The connection between phospho-Src and phospho-NF-κB was also observed in MCF7, where the edge was not predicted.

An edge from p70S6K_pT389 to p27_pT198 was predicted in all of the UACC812 and BT549 networks. The edge was also predicted in MCF7 networks for PBS, insulin, FGF, NRG1, and insulin-like growth factor (IGF)1 and in the BT20 NRG1 network. When p70S6K was inhibited in UACC812 cells with PF4708671, a change in phospho-p27 was observed only at the zero time point before stimulus was added (Figure 6D). In MCF7 cells stimulated with hepatocyte growth factor (HGF), phospho-p27 decreased in abundance under p70S6K inhibition; however, the edge was not predicted in this context. When PF4708671-treated MCF7 cells were stimulated with IGF, a context in which the edge was predicted with high probability, no change in phospho-p27 was observed. Similarly, there was no change in phospho-p27 in BT20 cells that had been treated with PF4708671 and stimulated with HGF.

In BT549, an edge was predicted from Chk2_pT68 to YAP_pS127 under HGF and insulin. BT549 cells treated with the Chk2 inhibitor AZD7762 exhibit an increase in phospho-YAP (Figure 6E). This edge was not predicted in any other cell line tested. However, in both UACC812 and MCF7 cells treated with AZD7762, a decrease in the abundance of phospho-YAP is observed. Active Chk2 appears to decrease phospho-YAP in BT549 cells (where the edge was predicted) and increase phospho-YAP in UACC812 and MCF7 cells (where the edge was not predicted). These results are consistent with the existence of a causal influence of phospho-Chk2 on phospho-YAP in all of these cell lines and not just in BT549 as predicted.

DISCUSSION

The data and analyses presented here support the notion that causal molecular networks can depend on context. We focused on signaling proteins and breast cancer cell lines. The cell lines represent contexts that are genetically perturbed but with a shared cancer type. The heterogeneity that we observed in causal networks suggests that substantial differences could exist between, for example, samples from different tissue types or divergent environmental conditions. This strongly argues for a need to refine existing regulatory models for specific contexts, not least in disease biology.

Given the range of potentially relevant contexts—spanning combinations of multiple factors, including genetic, epigenetic, and environmental—we do not believe that characterization of causal networks across multiple contexts can feasibly be done using classical approaches in a protein-by-protein manner. Rather, it will require high-throughput data acquisition and computational analysis. Such a program of research requires an appropriate conceptual framework rich enough to capture regulatory relationships but still tractable enough for large-scale investigation. Furthermore, for practical application, such an approach needs to be sufficiently robust to missing or unknown variables. Causal models of the kind we discussed may provide an appropriate framework because, unlike purely correlational models, they allow for reasoning about change under intervention and are, to a certain extent, robust to missing variables. In particular, causal descendancy matrices (Figures 4A and S3) are robust to missing variables in the sense that addition of a protein (row) to the matrix would not change the existing entries. We expect that a systematic program of investigation into context-specific causal networks will be important in many disease areas, and perhaps especially those that have to date not been well represented in the literature.

Our results extend the well-established notion of genomic intertumoral heterogeneity in cancer to the level of signaling phenotype. We found that cell line-specific findings were more reliable than stimulus-specific findings. This may be because the magnitude of epigenetic and genetic differences between cell lines is more marked than differences between stimuli, all of which activate closely related cell-surface receptors.

Our approach relied on inhibitor specificity, but we note that even at relatively low concentrations, off-target effects cannot be entirely ruled out. However, if the inhibitors were highly non-specific, the relatively good results seen in the train-and-test analysis would likely not be possible, since the analysis relies on assumed inhibitor targets. In the future, it may be relevant to consider models that allow uncertainty in the inhibitor targets themselves.

We highlighted the need to specify a relevant time frame in defining a causal graph. Indeed, an inhibitor may in the short to

(G) Summary of agreement and disagreement between predicted edges and validation experiments. The first row indicates whether validation experiments showed evidence for the edge in a (*cell line*, *stimulus*) context in which it was predicted. The second and third rows concern the cell line and stimulus specificity of each edge, respectively. A green tick denotes specificity in (partial) agreement with predictions from inferred networks; an orange tick denotes specificity, but not in agreement with predictions in terms of the precise contexts in which effects were seen; and a red cross indicates that specificity was not observed in the validation experiments, despite being predicted by the networks.

medium term induce changes to specific molecules, but over the longer term, the same inhibitor might induce adaptive changes to the cells themselves, e.g., via changes to epigenetic state (Duncan et al., 2012; Lee et al., 2012). We did not consider such "rewiring" in this article but note that the methods we discussed could be used to study rewiring (e.g., by comparing networks before and after adaptation).

In common with most protein profiling studies, including both low- and high-throughput techniques, our experiments were based on bulk assays and can therefore only elucidate signaling heterogeneity at the level of cell populations; we did not consider cell-to-cell heterogeneity, tumor stromal interactions, or the spatial heterogeneity of tumors that plays an important role in vivo (Bedard et al., 2013; González-García et al., 2002). However, our data have implications for inter- and intra-tumoral heterogeneity, because they suggest the possibility that in vivo causal signaling networks, and in turn the cell fates and disease progression events that they influence, may depend on the local micro-environment. Further work will be needed to elucidate such dependence and draw out its implications.

In the future, causal molecular networks may start to play a role in precision medicine, for example by helping to inform rational assignment of targeted therapies. An implication of the context specificity we report is that such analyses may require models that are learned, or at least modified, for individual samples (or subsets of samples). Although causal models are in some ways simpler than fully dynamical ones, causal inference remains fundamentally challenging and is very much an open area of research. For this reason, alongside advances in relevant assays, a personalized, network-based approach will require suitable empirical diagnostics. Hill et al. (2016) used the data presented here to score, in an automated manner, over 2,000 networks (~70 methods each applied to infer 32 context-specific networks) submitted to the HPN-DREAM network inference challenge, and we used an extended version of this assessment procedure here. Such assessment procedures might allow for automated quality control, for example rejecting networks not sufficiently consistent with unseen interventional readouts (e.g., we did not obtain statistically significant performance under any test inhibitor for the [*BT549*, *EGF*] context; see Figure S5). However, as discussed in Hill et al. (2016) the assessment procedure remains limited in several ways, and this argues for caution in interpreting the relatively good performance reported here. Of particular relevance to context specificity, we note that the procedure focuses on global agreement with held-out interventional data and not specifically on identification of differences between contexts. Indeed, our validation experiments showed that although all novel edges that were tested validated in one or more contexts, network predictions were not accurate with respect to the precise context(s) in which changes were seen.

Recently, Carvunis and Ideker (2014) proposed a view of cellular function involving hierarchies of elements and processes and not just networks. Building detailed dynamical or biophysical models over hierarchies spanning multiple time and spatial scales may prove infeasible. A more tractable approach may be to extend coarser causal models of the kind used here in a hierarchical direction, for example by allowing causal links to cross scales and subsystems. Thus, the approach we pursued—of causal models based on context-specific interventional data—could in the future be used to populate models over biological hierarchies.

STAR★METHODS

Detailed methods are provided in the online version of this paper and include the following:

- KEY RESOURCES TABLE
- CONTACT FOR REAGENT AND RESOURCE SHARING
- EXPERIMENTAL MODEL DETAILS
- METHOD DETAILS
  - Preparation of Cells for RPPA Analysis
  - RPPA Methodology
  - Western Blot Analysis
  - Quality Control and Preprocessing of RPPA Data
  - Identification of Changes Under Kinase Inhibition
  - Network Learning
  - Assessing Performance of Causal Network Learning
- QUANTIFICATION AND STATISTICAL ANALYSIS
- DATA AND SOFTWARE AVAILABILITY
  - Software
  - Data Resources
- ADDITIONAL RESOURCES

SUPPLEMENTAL INFORMATION

Supplemental Information includes six figures, six tables, and two data files and can be found with this article online at http://dx.doi.org/10.1016/j.cels.2016.11.013.

AUTHOR CONTRIBUTIONS

S.M.H. carried out analyses. N.K.N., K.J.-C., M.J., A.J., C.B., Y.L., and N.T.P. carried out the cell culture and inhibition experiments. S.E.F.S. contributed to analyses. Y.L. generated the RPPA data under the supervision of G.B.M. L.M.H. provided technical analyses. J.E.K. and J.W.G. provided experimental design. S.M.H., S.M., and P.T.S. designed analyses. G.B.M., S.M., and P.T.S. conceived and led the study. S.M.H., N.K.N., G.B.M., S.M., and P.T.S. wrote the paper.

ACKNOWLEDGMENTS

This work was supported by the National Institutes of Health National Cancer Institute (grant U54 CA112970 to J.W.G., G.B.M., S.M., and P.T.S.). S.M.H. and S.M. were supported by the UK Medical Research Council (unit program numbers MC_UP_1302/1 and MC_UP_1302/3). S.M. was a recipient of a Royal Society Wolfson Research Merit Award. The MD Anderson Cancer Center RPPA Core Facility is funded by the National Institutes of Health National Cancer Institute (Cancer Center Core Grant CA16672).

Received: November 2, 2015
Revised: August 6, 2016
Accepted: November 23, 2016
Published: December 22, 2016

REFERENCES

Akbani, R., Ng, P.K.S., Werner, H.M.J., Shahmoradgoli, M., Zhang, F., Ju, Z., Liu, W., Yang, J.-Y., Yoshihara, K., Li, J., et al. (2014). A pan-cancer proteomic perspective on The Cancer Genome Atlas. Nat. Commun. *5*, 3887.

Barretina, J., Caponigro, G., Stransky, N., Venkatesan, K., Margolin, A.A., Kim, S., Wilson, C.J., Lehár, J., Kryukov, G.V., Sonkin, D., et al. (2012). The Cancer Cell Line Encyclopedia enables predictive modelling of anticancer drug sensitivity. Nature *483*, 603–607.

Bedard, P.L., Hansen, A.R., Ratain, M.J., and Siu, L.L. (2013). Tumour heterogeneity in the clinic. Nature *501*, 355–364.

Benjamini, Y., Krieger, A.M., and Yekutieli, D. (2006). Adaptive linear step-up procedures that control the false discovery rate. Biometrika *93*, 491–507.

Cantone, I., Marucci, L., Iorio, F., Ricci, M.A., Belcastro, V., Bansal, M., Santini, S., di Bernardo, M., di Bernardo, D., and Cosma, M.P. (2009). A yeast synthetic network for in vivo assessment of reverse-engineering and modeling approaches. Cell *137*, 172–181.

Carvunis, A.-R., and Ideker, T. (2014). Siri of the cell: what biology could learn from the iPhone. Cell *157*, 534–538.

Coombes, K.R., Neeley, S., Joy, C., Hu, J., Baggerly, K., and Roebuck, P. (2012). SuperCurve: SuperCurve R Package. http://bioinformatics.mdanderson.org/Software/supercurve/.

De Smet, R., and Marchal, K. (2010). Advantages and limitations of current network inference methods. Nat. Rev. Microbiol. *8*, 717–729.

Duncan, J.S., Whittle, M.C., Nakamura, K., Abell, A.N., Midland, A.A., Zawistowski, J.S., Johnson, N.L., Granger, D.A., Jordan, N.V., Darr, D.B., et al. (2012). Dynamic reprogramming of the kinome in response to targeted MEK inhibition in triple-negative breast cancer. Cell *149*, 307–321.

Garnett, M.J., Edelman, E.J., Heidorn, S.J., Greenman, C.D., Dastur, A., Lau, K.W., Greninger, P., Thompson, I.R., Luo, X., Soares, J., et al. (2012). Systematic identification of genomic markers of drug sensitivity in cancer cells. Nature *483*, 570–575.

Gerlinger, M., and Swanton, C. (2010). How Darwinian models inform therapeutic failure initiated by clonal heterogeneity in cancer medicine. Br. J. Cancer *103*, 1139–1143.

González-García, I., Solé, R.V., and Costa, J. (2002). Metapopulation dynamics and spatial heterogeneity in cancer. Proc. Natl. Acad. Sci. USA *99*, 13085–13089.

Good, M., Tang, G., Singleton, J., Reményi, A., and Lim, W.A. (2009). The Ste5 scaffold directs mating signaling by catalytically unlocking the Fus3 MAP kinase for activation. Cell *136*, 1085–1097.

Heiser, L.M., Sadanandam, A., Kuo, W.L., Benz, S.C., Goldstein, T.C., Ng, S., Gibb, W.J., Wang, N.J., Ziyad, S., Tong, F., et al. (2012). Subtype and pathway specific responses to anticancer compounds in breast cancer. Proc. Natl. Acad. Sci. USA *109*, 2724–2729.

Hennessy, B.T., Lu, Y., Gonzalez-Angulo, A.M., Carey, M.S., Myhre, S., Ju, Z., Davies, M.A., Liu, W., Coombes, K., Meric-Bernstam, F., et al. (2010). A technical assessment of the utility of reverse phase protein arrays for the study of the functional proteome in non-microdissected human breast cancers. Clin. Proteomics *6*, 129–151.

Hill, S.M., Lu, Y., Molina, J., Heiser, L.M., Spellman, P.T., Speed, T.P., Gray, J.W., Mills, G.B., and Mukherjee, S. (2012). Bayesian inference of signaling network topology in a cancer cell line. Bioinformatics *28*, 2804–2810.

Hill, S.M., Heiser, L.M., Cokelaer, T., Unger, M., Nesser, N.K., Carlin, D.E., Zhang, Y., Sokolov, A., Paull, E.O., Wong, C.K., et al.; HPN-DREAM Consortium (2016). Inferring causal molecular networks: empirical assessment through a community-based effort. Nat. Methods *13*, 310–318.

Hu, J., He, X., Baggerly, K.A., Coombes, K.R., Hennessy, B.T.J., and Mills, G.B. (2007). Non-parametric quantification of protein lysate arrays. Bioinformatics *23*, 1986–1994.

Husmeier, D. (2003). Sensitivity and specificity of inferring genetic regulatory interactions from microarray experiments with dynamic Bayesian networks. Bioinformatics *19*, 2271–2282.

Lee, M.J., Ye, A.S., Gardino, A.K., Heijink, A.M., Sorger, P.K., MacBeath, G., and Yaffe, M.B. (2012). Sequential application of anticancer drugs enhances cell death by rewiring apoptotic signaling networks. Cell *149*, 780–794.

Li, J., Lu, Y., Akbani, R., Ju, Z., Roebuck, P.L., Liu, W., Yang, J.-Y., Broom, B.M., Verhaak, R.G.W., Kane, D.W., et al. (2013). TCPA: a resource for cancer functional proteomics data. Nat. Methods *10*, 1046–1047.

Liu, W., Ju, Z., Lu, Y., Mills, G.B., and Akbani, R. (2014). A comprehensive comparison of normalization methods for loading control and variance stabilization of reverse-phase protein array data. Cancer Inform. *13*, 109–117.

Marbach, D., Prill, R.J., Schaffter, T., Mattiussi, C., Floreano, D., and Stolovitzky, G. (2010). Revealing strengths and weaknesses of methods for gene network inference. Proc. Natl. Acad. Sci. USA *107*, 6286–6291.

Marbach, D., Lamparter, D., Quon, G., Kellis, M., Kutalik, Z., and Bergmann, S. (2016). Tissue-specific regulatory circuits reveal variable modular perturbations across complex diseases. Nat. Methods *13*, 366–370.

Murphy, K. (2002). Dynamic Bayesian Networks: Representation, Inference and Learning (University of California).

Neve, R.M., Chin, K., Fridlyand, J., Yeh, J., Baehner, F.L., Fevr, T., Clark, L., Bayani, N., Coppe, J.-P., Tong, F., et al. (2006). A collection of breast cancer cell lines for the study of functionally distinct cancer subtypes. Cancer Cell *10*, 515–527.

Nickel, G.C., Barnholtz-Sloan, J., Gould, M.P., McMahon, S., Cohen, A., Adams, M.D., Guda, K., Cohen, M., Sloan, A.E., and LaFramboise, T. (2012). Characterizing mutational heterogeneity in a glioblastoma patient with double recurrence. PLoS ONE *7*, e35262.

Oates, C.J., Korkola, J., Gray, J.W., and Mukherjee, S. (2014). Joint estimation of multiple related biological networks. Ann. Appl. Stat. *8*, 1892–1919.

Pearl, J. (2009). Causality: Models, Reasoning, and Inference (Cambridge University Press).

Petsalaki, E., Helbig, A.O., Gopal, A., Pasculescu, A., Roth, F.P., and Pawson, T. (2015). SELPHI: correlation-based identification of kinase-associated networks from global phospho-proteomics data sets. Nucleic Acids Res. *43* (W1), W276–W282.

Rolland, T., Taşan, M., Charloteaux, B., Pevzner, S.J., Zhong, Q., Sahni, N., Yi, S., Lemmens, I., Fontanillo, C., Mosca, R., et al. (2014). A proteome-scale map of the human interactome network. Cell *159*, 1212–1226.

Saez-Rodriguez, J., Goldsipe, A., Muhlich, J., Alexopoulos, L.G., Millard, B., Lauffenburger, D.A., and Sorger, P.K. (2008). Flexible informatics for linking experimental data to mathematical models via DataRail. Bioinformatics *24*, 840–847.

Saez-Rodriguez, J., Alexopoulos, L.G., Zhang, M., Morris, M.K., Lauffenburger, D.A., and Sorger, P.K. (2011). Comparing signaling networks between normal and transformed hepatocytes using discrete logical models. Cancer Res. *71*, 5400–5411.

Shannon, P., Markiel, A., Ozier, O., Baliga, N.S., Wang, J.T., Ramage, D., Amin, N., Schwikowski, B., and Ideker, T. (2003). Cytoscape: a software environment for integrated models of biomolecular interaction networks. Genome Res. *13*, 2498–2504.

Spencer, S.E.F., Hill, S.M., and Mukherjee, S. (2015). Inferring network structure from interventional time-course experiments. Ann. Appl. Stat. *9*, 507–524.

Spirtes, P., Glymour, C.N., and Scheines, R. (2000). Causation, Prediction, and Search (MIT Press).

Szerlip, N.J., Pedraza, A., Chakravarty, D., Azim, M., McGuire, J., Fang, Y., Ozawa, T., Holland, E.C., Huse, J.T., Jhanwar, S., et al. (2012). Intratumoral heterogeneity of receptor tyrosine kinases EGFR and PDGFRA amplification in glioblastoma defines subpopulations with distinct growth factor response. Proc. Natl. Acad. Sci. USA *109*, 3041–3046.

Tibes, R., Qiu, Y., Lu, Y., Hennessy, B., Andreeff, M., Mills, G.B., and Kornblau, S.M. (2006). Reverse phase protein array: validation of a novel proteomic technology and utility for analysis of primary leukemia specimens and hematopoietic stem cells. Mol. Cancer Ther. *5*, 2512–2521.

Weinberg, R. (2013). The Biology of Cancer (Garland Science).

Will, T., and Helms, V. (2016). PPIXpress: construction of condition-specific protein interaction networks based on transcript expression. Bioinformatics *32*, 571–578.

Woodward, J. (2016). Causation and manipulability. In The Stanford Encyclopedia of Philosophy, E.N. Zalta, ed. (Metaphysics Research Lab, Stanford University). https://plato.stanford.edu/archives/fall2016/entries/causation-mani/.

Zalatan, J.G., Coyle, S.M., Rajan, S., Sidhu, S.S., and Lim, W.A. (2012). Conformational control of the Ste5 scaffold protein insulates against MAP kinase misactivation. Science *337*, 1218–1222.

STAR★METHODS

KEY RESOURCES TABLE

REAGENT or RESOURCE	SOURCE	IDENTIFIER
Antibodies		
Rabbit polyclonal anti-14-3-3-beta (clone C-20)	Santa Cruz	Cat#sc-628; RRID: AB_630818
Mouse monoclonal anti-14-3-3-epsilon (clone 8C3)	Santa Cruz	Cat#sc-23957; RRID: AB_626619
Rabbit polyclonal anti-14-3-3-zeta (clone C-16)	Santa Cruz	Cat#sc-1019; RRID: AB_2218378
Rabbit polyclonal anti-4E-BP1	Cell Signaling Technology	Cat#9452; RRID: AB_331692
Rabbit monoclonal anti-phospho-4E-BP1 (Ser65) (clone 174A9)	Cell Signaling Technology	Cat#9456; RRID: AB_823413
Rabbit polyclonal anti-phospho-4E-BP1 (Thr37/46)	Cell Signaling Technology	Cat#9459; RRID: AB_2262165
Rabbit polyclonal anti-53BP1	Cell Signaling Technology	Cat#4937; RRID: AB_10694558
Rabbit polyclonal anti-phospho-ACC (Ser79)	Cell Signaling Technology	Cat#3661; RRID: AB_330337
Rabbit monoclonal anti-ACC1 (clone EP687Y)	Epitomics	Cat#1768-1; RRID: AB_598134
Rabbit monoclonal anti-ACVRL1 (clone EPR4074)	Epitomics	Cat#2940-1; RRID: AB_2222593
Mouse monoclonal anti-AIB1 (clone 34)	BD Biosciences	Cat#611105; RRID: AB_2151198
Rabbit monoclonal anti-Akt (pan) (clone C67E7)	Cell Signaling Technology	Cat#4691; RRID: AB_915783
Rabbit polyclonal anti-Akt	Cell Signaling Technology	Cat#9272; RRID: AB_329827
Rabbit polyclonal anti-phospho-Akt (Ser473)	Cell Signaling Technology	Cat#9271; RRID: AB_329825
Rabbit polyclonal anti-phospho-Akt (Thr308)	Cell Signaling Technology	Cat#9275; RRID: AB_329828
Mouse monoclonal anti-alpha-catenin (clone 1G5)	Calbiochem	Cat#CA1030; RRID: AB_2243846
Rabbit polyclonal anti-AMPK-alpha	Cell Signaling Technology	Cat#2532; RRID: AB_330331
Rabbit monoclonal anti-phospho-AMPK-alpha (Thr172) (clone 40H9)	Cell Signaling Technology	Cat#2535; RRID: AB_331250
Rabbit polyclonal anti-Annexin-I	Invitrogen	Cat#71-3400; RRID: AB_2533983
Mouse monoclonal anti-Annexin-VII (clone 5)	BD Biosciences	Cat#610668; RRID: AB_397995
Rabbit monoclonal anti-AR (clone EP670Y)	Epitomics	Cat#1852-1; RRID: AB_764443
Mouse monoclonal anti-B-Raf (clone F-7)	Santa Cruz	Cat#sc-5284; RRID: AB_626760
Rabbit polyclonal anti-phospho-Bad (Ser112)	Cell Signaling Technology	Cat#9291; RRID: AB_331418
Rabbit monoclonal anti-BAK (clone Y164)	Epitomics	Cat#1542-1; RRID: AB_562051
Rabbit polyclonal anti-BAX	Cell Signaling Technology	Cat#2772; RRID: AB_10695870
Mouse monoclonal anti-BCL-2	Dako	Cat#M0887; RRID: AB_2064429
Rabbit monoclonal anti-Bcl-X (clone E18)	Epitomics	Cat#1018-1; RRID: AB_289586
Rabbit polyclonal anti-Bcl-xL	Cell Signaling Technology	Cat#2762; RRID: AB_10694844
Goat polyclonal anti-Beclin (clone D-18)	Santa Cruz	Cat#sc-10086; RRID: AB_2259076
Rabbit polyclonal anti-beta-Catenin	Cell Signaling Technology	Cat#9562; RRID: AB_331149
Rabbit monoclonal anti-BID (clone Y8)	Epitomics	Cat#1008-1; RRID: AB_365478
Rabbit monoclonal anti-BIM (clone Y36)	Epitomics	Cat#1036-1; RRID: AB_347632
Rabbit polyclonal anti-phospho-c-Jun (Ser73)	Cell Signaling Technology	Cat#9164; RRID: AB_330893
Rabbit monoclonal anti-c-KIT (clone YR145)	Epitomics	Cat#1522-1; RRID: AB_731513
Mouse monoclonal anti-c-Met (clone 25H2)	Cell Signaling Technology	Cat#3127; RRID: AB_2181554
Rabbit monoclonal anti-phospho-c-Met (Tyr1234/1235) (clone 3D7)	Cell Signaling Technology	Cat#3129; RRID: AB_561175
Rabbit polyclonal anti-c-Myc	Cell Signaling Technology	Cat#9402; RRID: AB_10693752
Rabbit polyclonal anti-c-Myc (clone N-262)	Santa Cruz	Cat#sc-764; RRID: AB_631276
Rabbit monoclonal anti-c-Raf (clone AM223)	Millipore	Cat#05-739; RRID: AB_309953
Rabbit monoclonal anti-phospho-c-Raf (Ser338) (clone 56A6)	Cell Signaling Technology	Cat#9427; RRID: AB_2067317

(*Continued on next page*)

Continued

REAGENT or RESOURCE	SOURCE	IDENTIFIER
Rabbit monoclonal anti-Caspase-3- (Active) (clone E83-77)	Epitomics	Cat#1476-1; RRID: AB_562063
Rabbit polyclonal anti-Caspase-7 (cleaved D198)	Cell Signaling Technology	Cat#9491; RRID: AB_2068144
Rabbit polyclonal anti-Caspase-9 (cleaved D330)	Cell Signaling Technology	Cat#9501; RRID: AB_331424
Rabbit polyclonal anti-Caveolin-1	Cell Signaling Technology	Cat#3238; RRID: AB_10699017
Mouse monoclonal anti-CD31	Dako	Cat#M0823; RRID: AB_2114471
Mouse monoclonal anti-CD49B (clone 2)	BD Biosciences	Cat#611016; RRID: AB_398329
Rabbit polyclonal anti-CDK1	Cell Signaling Technology	Cat#9112; RRID: AB_10693432
Rabbit polyclonal anti-Chk1	Cell Signaling Technology	Cat#2345; RRID: AB_10693648
Rabbit monoclonal anti-phospho-Chk1 (Ser345) (clone 133D3)	Cell Signaling Technology	Cat#2348; RRID: AB_2080326
Mouse monoclonal anti-Chk2 (clone 1C12)	Cell Signaling Technology	Cat#3440; RRID: AB_2229490
Rabbit monoclonal anti-phospho-Chk2 (Thr68) (clone C13C1)	Cell Signaling Technology	Cat#2197; RRID: AB_2080501
Rabbit polyclonal anti-cIAP-1/HIAP-2	Millipore	Cat#07-759; RRID: AB_11212879
Rabbit polyclonal anti-Claudin-7	Novus Biologicals	Cat#NB100-91714; RRID: AB_1216502
Rabbit polyclonal anti-Collagen-VI (clone H-200)	Santa Cruz	Cat#SC-20649; RRID: AB_2083098
Rabbit monoclonal anti-COX-2 (clone EP1978Y)	Epitomics	Cat#2169-1; RRID: AB_991710
Rabbit monoclonal anti-Cyclin-B1 (clone Y106)	Epitomics	Cat#1495-1; RRID: AB_562272
Rabbit polyclonal anti-Cyclin-D1 (clone M-20)	Santa Cruz	Cat#sc-718; RRID: AB_2070436
Mouse monoclonal anti-Cyclin-E1 (clone HE12)	Santa Cruz	Cat#sc-247; RRID: AB_627357
Rabbit monoclonal anti-DJ-1/PARK7 (clone EP2815Y)	Abcam	Cat#ab76008; RRID: AB_1310549
Rabbit polyclonal anti-Dvl3	Cell Signaling Technology	Cat#3218; RRID: AB_10694060
Rabbit polyclonal anti-E-Cadherin	Cell Signaling Technology	Cat#4065; RRID: AB_2076803
Rabbit monoclonal anti-E-Cadherin (clone 24E10)	Cell Signaling Technology	Cat#3195; RRID: AB_10694492
Rabbit polyclonal anti-eEF2	Cell Signaling Technology	Cat#2332; RRID: AB_10693546
Rabbit polyclonal anti-eEF2K	Cell Signaling Technology	Cat#3692; RRID: AB_10694413
Rabbit polyclonal anti-EGFR (clone 1005)	Santa Cruz	Cat#SC-03; RRID: AB_631420
Rabbit polyclonal anti-EGFR	Cell Signaling Technology	Cat#2232; RRID: AB_823483
Rabbit polyclonal anti-phospho-EGFR (Tyr1068)	Cell Signaling Technology	Cat#2234; RRID: AB_331701
Rabbit monoclonal anti-phospho-EGFR (Tyr1173) (E124)	Epitomics	Cat#1124; RRID: AB_344895
Rabbit polyclonal anti-phospho-EGFR_pY992	Cell Signaling Technology	Cat#2235; RRID: AB_331709
Rabbit polyclonal anti-eIF4E	Cell Signaling Technology	Cat#9742; RRID: AB_823488
Rabbit polyclonal anti-eIF4G	Cell Signaling Technology	Cat#2498; RRID: AB_10692643
Rabbit monoclonal anti-ER-alpha (clone SP1)	Lab Vision	Cat#RM-9101-S; RRID: AB_149901
Rabbit monoclonal anti-phospho-ER-alpha (Ser118) (clone E91)	Epitomics	Cat#1091-1; RRID: AB_562111
Mouse monoclonal anti-ERCC1 (clone 8F1)	Lab Vision	Cat#MS-671-P0; RRID: AB_143360
Rabbit monoclonal anti-FAK (clone EP695Y)	Epitomics	Cat#1700-1; RRID: AB_562113
Rabbit monoclonal anti-Fibronectin (clone F14)	Epitomics	Cat#1574-1; RRID: AB_562115
Rabbit monoclonal anti-FoxM1 (clone D12D5)	Cell Signaling Technology	Cat#5436; RRID: AB_10692483
Rabbit polyclonal anti-FOXO3a	Cell Signaling Technology	Cat#9467; RRID: AB_10693643
Rabbit monoclonal anti-FOXO3a (clone 75D8)	Cell Signaling Technology	Cat#2497; RRID: AB_836876
Rabbit polyclonal anti-phospho-FOXO3a (Ser318/321)	Cell Signaling Technology	Cat#9465; RRID: AB_2106498
Rabbit monoclonal anti-Gab2 (clone 26B6)	Cell Signaling Technology	Cat#3239; RRID: AB_10698601
Mouse monoclonal anti-GATA3 (clone L50-823)	BD Biosciences	Cat#558686; RRID: AB_2108590
Mouse monoclonal anti-GSK3-alpha-beta (clone 0011-A)	Santa Cruz	Cat#sc-7291; RRID: AB_2279451
Rabbit polyclonal anti-phospho-GSK3-alpha-beta (Ser21/9)	Cell Signaling Technology	Cat#9331; RRID: AB_329830
Rabbit polyclonal anti-phospho-GSK3 (Ser9)	Cell Signaling Technology	Cat#9336; RRID: AB_331405

(*Continued on next page*)

Continued

REAGENT or RESOURCE	SOURCE	IDENTIFIER
Mouse monoclonal anti-HER2	Lab Vision	Cat#MS-325-P1; RRID: AB_61444
Rabbit polyclonal anti-phospho-HER2 (Tyr1248)	Upstate (Millipore)	Cat#06-229; RRID: AB_310076
Rabbit polyclonal anti-HER3 (clone C-17)	Santa Cruz	Cat#sc-285; RRID: AB_2099723
Rabbit monoclonal anti-phospho-HER3 (Tyr1289) (clone 21D3)	Cell Signaling Technology	Cat#4791; RRID: AB_2099708
Rabbit polyclonal anti-IGF-1R-beta	Cell Signaling Technology	Cat#3027; RRID: AB_2122378
Rabbit polyclonal anti-IGFBP2	Cell Signaling Technology	Cat#3922; RRID: AB_10691844
Goat polyclonal anti-INPP4B (clone N-20)	Santa Cruz	Cat#SC-12318; RRID: AB_2126126
Rabbit polyclonal anti-IRS1	Millipore	Cat#06-248; RRID: AB_2127890
Rabbit monoclonal anti-phospho-JNK/SAPK (Thr183/Tyr185) (clone 81E11)	Cell Signaling Technology	Cat#4668; RRID: AB_823588
Rabbit polyclonal anti-JNK2	Cell Signaling Technology	Cat#4672; RRID: AB_10695599
Mouse monoclonal anti-k-Ras (clone F234)	Santa Cruz	Cat#sc-30; RRID: AB_627865
Rabbit polyclonal anti-Lck	Cell Signaling Technology	Cat#2752; RRID: AB_10691548
Rabbit monoclonal anti-phospho-p44/42 MAPK (Erk1/2) (Thr202/Tyr204) (clone 197G2)	Cell Signaling Technology	Cat#4377; RRID: AB_331775
Rabbit monoclonal anti-MEK1 (clone Y77)	Epitomics	Cat#1235-1; RRID: AB_562310
Rabbit monoclonal anti-phospho-MEK1 (Ser217/221) (clone 41G9)	Cell Signaling Technology	Cat#9154; RRID: AB_2138017
Mouse monoclonal anti-MGMT (clone MT3.1)	Millipore	Cat#MAB16200; RRID: AB_2281919
Mouse monoclonal anti-MIG-6	Sigma-Aldrich	Cat#WH0054206M1; RRID: AB_1841511
Rabbit monoclonal anti-Mre11 (clone 31H4)	Cell Signaling Technology	Cat#4847; RRID: AB_10693469
Mouse monoclonal anti-MSH2 (clone 3A2)	Cell Signaling Technology	Cat#2850; RRID: AB_2144797
Rabbit polyclonal anti-MSH6	Novus Biologicals	Cat#22030002; RRID: AB_2266534
Rabbit monoclonal anti-mTOR (clone 7C10)	Cell Signaling Technology	Cat#2983; RRID: AB_2105622
Rabbit polyclonal anti-phospho-mTOR (Ser2448)	Cell Signaling Technology	Cat#2971; RRID: AB_330970
Rabbit polyclonal anti-MYH11	Novus Biologicals	Cat#21370002; RRID: AB_2147162
Rabbit polyclonal anti-N-Cadherin	Cell Signaling Technology	Cat#4061; RRID: AB_10694647
Mouse monoclonal anti-N-Ras (clone F155)	Santa Cruz	Cat#sc-31; RRID: AB_628041
Rabbit polyclonal anti-phospho-NDRG1 (Thr346)	Cell Signaling Technology	Cat#3217; RRID: AB_2150174
Rabbit monoclonal anti-phospho-NF-kB-p65 (Ser536)	Cell Signaling Technology	Cat#3033; RRID: AB_331284
Rabbit polyclonal anti-NF2	SDI / Novus	Cat#2271.00.02; RRID: AB_2298264
Rabbit monoclonal anti-Notch1 (clone C44H11)	Cell Signaling Technology	Cat#3268; RRID: AB_1264224
Rabbit polyclonal anti-Notch3 (clone M-134)	Santa Cruz	Cat#sc-5593; RRID: AB_2151246
Rabbit polyclonal anti-P-Cadherin	Cell Signaling Technology	Cat#2130; RRID: AB_10693468
Rabbit polyclonal anti-p21 (clone C-19)	Santa Cruz	Cat#sc-397; RRID: AB_632126
Rabbit monoclonal anti-p27/Kip1 (clone Y236)	Epitomics	Cat#1591-1; RRID: AB_562357
Rabbit polyclonal anti-phospho-p27/Kip1 (Thr157)	R&D Systems	Cat#AF1555; RRID: AB_354857
Rabbit polyclonal anti-phospho-p27/KIP 1 (Thr198)	Abcam	Cat#ab64949; RRID: AB_1142099
Rabbit polyclonal anti-p38 MAPK	Cell Signaling Technology	Cat#9212; RRID: AB_330713
Rabbit polyclonal anti-phospho-p38 MAPK (Thr180/Tyr182)	Cell Signaling Technology	Cat#9211; RRID: AB_331641
Rabbit polyclonal anti-p53	Cell Signaling Technology	Cat#9282; RRID: AB_10693944
Rabbit monoclonal anti-p70S6K (clone E343)	Epitomics	Cat#1494-1; RRID: AB_562325
Rabbit polyclonal anti-phospho-p70S6K (Thr389)	Cell Signaling Technology	Cat#9205; RRID: AB_330944
Rabbit polyclonal anti-phospho-p90RSK (Thr359/Ser363)	Cell Signaling Technology	Cat#9344; RRID: AB_331650
Mouse monoclonal anti-PARP (cleaved D214) (clone 19F4)	Cell Signaling Technology	Cat#9546; RRID: AB_2160593
Rabbit monoclonal anti-Paxillin (clone Y113)	Epitomics	Cat#1500-1; RRID: AB_562188
Mouse monoclonal anti-PCNA (clone PC10)	Abcam	Cat#ab29; RRID: AB_303394

(*Continued on next page*)

Continued

REAGENT or RESOURCE	SOURCE	IDENTIFIER
Rabbit polyclonal anti-PDCD4	Rockland	Cat#600-401-965; RRID: AB_828370
Rabbit polyclonal anti-PDK1	Cell Signaling Technology	Cat#3062; RRID: AB_10695863
Rabbit polyclonal anti-phospho-PDK1 (Ser241)	Cell Signaling Technology	Cat#3061; RRID: AB_2161311
Rabbit polyclonal anti-PEA15	Cell Signaling Technology	Cat#2780; RRID: AB_2268149
Rabbit polyclonal anti-phospho-PEA15 (Ser116)	Invitrogen	Cat#44-836G; RRID: AB_2533775
Rabbit polyclonal anti-PI3K-p110-alpha	Cell Signaling Technology	Cat#4255; RRID: AB_10695395
Rabbit polyclonal anti-PI3K_p85	Millipore	Cat#06-195; RRID: AB_310069
Mouse monoclonal anti-PKC-alpha (clone M4)	Millipore	Cat#05-154; RRID: AB_2284233
Rabbit polyclonal anti-phospho-PKC-alpha (Ser657)	Millipore	Cat#06-822; RRID: AB_310258
Rabbit polyclonal anti-phospho-PKC-delta (Ser664)	Millipore	Cat#07-875; RRID: AB_568868
Rabbit polyclonal anti-phospho-PKC-pan-betaII (Ser660)	Cell Signaling Technology	Cat#9371; RRID: AB_2168219
Rabbit monoclonal anti-PR (clone YR85)	Epitomics	Cat#1483-1; RRID: AB_562201
Rabbit polyclonal anti-phospho-PRAS40 (Thr246)	Biosource	Cat#441100G; RRID: AB_2533573
Rabbit polyclonal anti-PTCH	SDI	Cat#21130002; RRID: AB_876276
Rabbit polyclonal anti-PTEN	Cell Signaling Technology	Cat#9552; RRID: AB_10694066
Rabbit polyclonal anti-Rab11	Cell Signaling Technology	Cat#3539; RRID: AB_2253210
Rabbit polyclonal anti-Rab25	Covance (custom antibody services)	N/A
Mouse monoclonal anti-Rad50 (clone 13B3/2C6)	Millipore	Cat#05-525; RRID: AB_309782
Mouse polyclonal anti-Rad51	Chem Biotech	Cat#na 71
Rabbit monoclonal anti-Raptor (24C12)	Cell Signaling Technology	Cat#2280; RRID: AB_10694695
Mouse monoclonal anti-Rb (clone 4H1)	Cell Signaling Technology	Cat#9309; RRID: AB_823629
Rabbit polyclonal anti-phospho-Rb (Ser807/811)	Cell Signaling Technology	Cat#9308; RRID: AB_331472
Rabbit polyclonal anti-RBM15	Novus Biologicals	Cat#21390002; RRID: AB_2175759
Rabbit monoclonal anti-Rictor (clone 53A2)	Cell Signaling Technology	Cat#2114; RRID: AB_10694641
Rabbit monoclonal anti-phospho-Rictor (Thr1135) (clone D30A3)	Cell Signaling Technology	Cat#3806; RRID: AB_10557237
Rabbit polyclonal anti-phospho-S6 (Ser235/236)	Cell Signaling Technology	Cat#2211; RRID: AB_331679
Rabbit polyclonal anti-phospho-S6 (Ser240/244)	Cell Signaling Technology	Cat#2215; RRID: AB_331682
Mouse monoclonal anti-SCD1 (clone CD.E10)	Santa Cruz	Cat#sc-58420; RRID: AB_785599
Mouse monoclonal anti-SF2 (clone 96)	Invitrogen	Cat#32-4500; RRID: AB_2533079
Mouse monoclonal anti-Smac/Diablo	Cell Signaling Technology	Cat#2954; RRID: AB_10694396
Rabbit monoclonal anti-Smad1 (clone EP565Y)	Epitomics	Cat#1649-1; RRID: AB_562224
Rabbit monoclonal anti-Smad3 (clone EP568Y)	Epitomics	Cat#1735-1; RRID: AB_598188
Mouse polyclonal anti-Smad4 (clone B-8)	Santa Cruz	Cat#sc-7966; RRID: AB_627905
Mouse polyclonal anti-Snail (clone L70G2)	Cell Signaling Technology	Cat#3895; RRID: AB_2191759
Mouse monoclonal anti-Src (clone GD11)	Millipore	Cat#05-184; RRID: AB_2302631
Rabbit polyclonal anti-phospho-Src (Tyr416)	Cell Signaling Technology	Cat#2101; RRID: AB_331697
Rabbit polyclonal anti-phospho-Src (Tyr527)	Cell Signaling Technology	Cat#2105; RRID: AB_331034
Rabbit polyclonal anti-phospho-STAT3 (Tyr705)	Cell Signaling Technology	Cat#9131; RRID: AB_331586
Rabbit monoclonal anti-STAT5-alpha (E289)	Epitomics	Cat#1289-1; RRID: AB_562347
Rabbit monoclonal anti-Stathmin (clone EP1573Y)	Epitomics	Cat#1972-1; RRID: AB_991829
Mouse monoclonal anti-Syk (clone 4D10)	Santa Cruz	Cat#sc-1240; RRID: AB_628308
Mouse monoclonal anti-Tau (clone 5E2)	Millipore	Cat#05-348; RRID: AB_309687
Rabbit polyclonal anti-TAZ	Cell Signaling Technology	Cat#2149; RRID: AB_823657
Rabbit polyclonal anti-phospho-TAZ (Ser89)	Santa Cruz	Cat#sc-17610; RRID: AB_671263
Rabbit polyclonal anti-TIGAR	Epitomics	Cat#S1711; RRID: AB_10638379
Mouse monoclonal anti-Transglutaminase II	Lab Vision	Cat#MS-224-P1; RRID: AB_62205

(*Continued on next page*)

Continued

REAGENT or RESOURCE	SOURCE	IDENTIFIER
Rabbit polyclonal anti-TFRC	Novus Biologicals	Cat#22500002; RRID: AB_10004660
Rabbit polyclonal anti-TSC1	Cell Signaling Technology	Cat#4906; RRID: AB_10695257
Rabbit monoclonal anti-TTF1 (clone EP1584Y)	Epitomics	Cat#2044-1; RRID: AB_1267367
Rabbit monoclonal anti-Tuberin (clone Y320)	Epitomics	Cat#1613-1; RRID: AB_562354
Rabbit polyclonal anti-VASP	Cell Signaling Technology	Cat#3112; RRID: AB_10693778
Rabbit monoclonal anti-VEGFR2 (clone 55B11)	Cell Signaling Technology	Cat#2479; RRID: AB_2212507
Mouse monoclonal anti-VHL (clone Ig32)	BD Biosciences	Cat#556347; RRID: AB_396376
Rabbit polyclonal anti-XIAP	Cell Signaling Technology	Cat#2042; RRID: AB_2214868
Rabbit polyclonal anti-XRCC1	Cell Signaling Technology	Cat#2735; RRID: AB_2218471
Rabbit polyclonal anti-YAP (clone H-125)	Santa Cruz	Cat#sc-15407; RRID: AB_2273277
Rabbit polyclonal anti-phospho-YAP (Ser127)	Cell Signaling Technology	Cat#4911; RRID: AB_2218913
Rabbit polyclonal anti-YB-1	SDI / Novus	Cat#1725.00.02; RRID: AB_936227
Rabbit monoclonal anti-phospho-YB-1 (Ser102) (clone C34A2)	Cell Signaling Technology	Cat#2900; RRID: AB_2219273
Mouse monoclonal anti-beta-Actin (clone C4)	Santa Cruz	Cat#SC-47778; RRID: AB_626632
Mouse monoclonal alpha-Tubulin (clone B-5-1-2)	Invitrogen	Cat#322500; RRID: AB_2533071
Mouse monoclonal anti-Hsp90 (clone 68)	Transduction Laboratories	Cat#H38220; RRID: AB_397798
HRP Donkey polyclonal anti-mouse	Jackson Immunoresearch	Cat#715-035-150; RRID: AB_2340770
HRP Donkey polyclonal anti-rabbit	Jackson Immunoresearch	Cat#711-035-152; RRID: AB_10015282
Chemicals, Peptides, and Recombinant Proteins		
AZD8055	Selleck	Cat#S1555
GSK690693	Selleck	Cat#S1113
BEZ235	Selleck	Cat#S1009
PD173074	Selleck	Cat#S1264
GSK1120212	Selleck	Cat#S2673
AZD7762	Selleck	Cat#S1532
KX2-391	Selleck	Cat#S2700
PF4708671	Selleck	Cat#S2163
VX-702	Selleck	Cat#S6005
DMEM	GIBCO	Cat#11965-118
RPMI	GIBCO	Cat#11875-119
Fetal Bovine Serum	GIBCO	Cat#10437
HGF	GIBCO	Cat#PHG0254
FGF-alpha	GIBCO	Cat#PHG0014
Insulin	Sigma	Cat#IO516-5ML
IGF	GIBCO	Cat#PHG0078
NRG-1	R&D Systems	Cat#5898-R
EGF	GIBCO	Cat#PHG0311
cOmplete protease inhibitor cocktail	Roche Applied Science	Cat# 4693116001
phosSTOP phosphatase inhibitor cocktail	Roche Applied Science	Cat# 4906845001
Critical Commercial Assays		
SuperSignal West Pico Chemiluminescent HRP Substrate Kit	Thermofisher Scientific	Cat#34080
Pierce BCA Protein Assay	Thermofisher Scientific	Cat#23225
Deposited Data		
RPPA data	This paper	Data S1 and S2
Experimental Models: Cell Lines		
Human: MCF7 cells	ATCC: HTB-22	Cat#HTB-22; RRID:CVCL_0031
Human: UACC812 cells	ATCC: CRL-1897	Cat#CRL-1897; RRID:CVCL_1781

(*Continued on next page*)

Continued

REAGENT or RESOURCE	SOURCE	IDENTIFIER
Human: BT20 cells	ATCC: HTB-19	Cat#HTB-19; RRID:CVCL_0178
Human: BT549 cells	ATCC: HTB-122	Cat#HTB-122; RRID:CVCL_1092
Software and Algorithms		
Scripts for: identification of changes under kinase inhibition, network learning and assessment of network learning performance	This paper (see STAR Methods for details)	https://github.com/Steven-M-Hill/causal-signaling-networks-CellSystems2016
MATLAB R2012a	MathWorks, Inc.	http://www.mathworks.com
DataRail	Saez-Rodriguez et al., 2008	https://code.google.com/archive/p/sbpipeline/
Cytoscape	Shannon et al., 2003	http://www.cytoscape.org/
Joint Network Inference (modified version used in this paper; script available on github, see above)	Oates et al., 2014	http://dx.doi.org/10.1214/14-AOAS761
Supercurve	Coombes et al., 2012	http://bioinformatics.mdanderson.org/Software/supercurve/

CONTACT FOR REAGENT AND RESOURCE SHARING

Further information and requests for reagents and resources may be directed to, and will be fulfilled by, the Lead Contact Paul T. Spellman (spellmap@ohsu.edu).

EXPERIMENTAL MODEL DETAILS

Breast epithelial cells in log-phase of growth were harvested, diluted in the appropriate media (DMEM (with phenol red) for UACC812, BT20 and MCF7; RPMI (with phenol red) for BT549) containing 10% fetal bovine serum, and then seeded into 6 well plates at an optimized cell density (to give 60%–75% confluence at time of lysis). BT20 cells were plated at 230,000 cells/well; BT549 cells were plated at 175,000 cells/well; MCF7 cells were plated at 215,000 cells/well; and UACC812 cells were plated at 510,000 cells/well. After 24 hr of growth at 37°C and 5% CO2 in complete medium, cells were synchronized by incubating with serum-free medium for an additional 24 hr (serum starvation was also necessary to control the presence of stimuli in the medium). The medium was then exchanged with fresh serum-free medium containing either: 15nM AZD8055, 50nM GSK690693, 50nM BEZ235, 150nM PD173074, 10nM GSK1120212 in combination with 50nM GSK690693, or vehicle alone (0.05% DMSO) and incubated for two hours prior to stimulation. Cells were then either harvested (0 time point) or stimulated by addition of 200 μL per well of 10X stimulus (either PBS, fetal bovine serum, 100 ng/mL EGF, 200ng/mL IGF1, 100nM insulin, 200ng/mL FGF1, 1 μg/mL NRG1, or 500 ng/mL HGF) for 0, 5, 15, 30 or 60 min, or 2, 4, 12, 24, 48 or 72 hr prior to protein harvest.

All cell lines have been authenticated by performing STR analysis and matching to reference STR profiles at 15 different loci. STR analysis was performed by Genetica Cell Line Testing.

METHOD DETAILS

Preparation of Cells for RPPA Analysis

Cells were grown as described above, then washed twice with PBS and lysed by adding lysis buffer obtained from MD Anderson Functional Proteomics RPPA Core Facility (Houston, Texas; lysis buffer comprised 1% Triton X-100, 50mM HEPES, pH 7.4, 150mM NaCl, 1.5mM MgCl2, 1mM EGTA, 100mM NaF, 10mM Na pyrophosphate, 1mM Na3VO4, 10% glycerol; protease and phosphatase inhibitors were freshly added on the day of the experiment). Volume of lysis buffer used was optimized for each cell line (to ensure lysates were not too dense for the BCA assay; see below) and varied between 50 μL and 100 μL. Lysates were collected by scraping after 20 min incubation on ice. Lysates were spun at 4°C in a tabletop centrifuge at 15,000 RPM for 10 min and soluble proteins contained in the supernatant were collected. Protein concentration was determined by the Pierce BCA Protein Assay according to manufacturer's protocol. Protein was then diluted to 1 mg/mL and 30 μL of the diluted lysate was mixed with 10 μL 4X SDS sample buffer (obtained from MD Anderson Functional Proteomics RPPA Core Facility; comprised 40% Glycerol, 8% SDS, 0.25M Tris-HCL, pH 6.8; 10% v/v 2-mercaptoethanol was added fresh) and boiled for 5 min prior to freezing and shipment to MD Anderson Cancer Center Functional Proteomics RPPA Core Facility for RPPA analysis (Tibes et al., 2006).

RPPA Methodology

RPPA methodology has been described previously (see e.g., Akbani et al., 2014); an outline is also provided below. Lysates were diluted in five two-fold serial dilutions with lysis buffer. An Aushon Biosystems 2470 arrayer (Burlington, MA) was used to print 1056 samples and control lysates on nitrocellulose-coated slides (Grace Bio-Labs). Each slide was probed with a primary antibody and a biotin-conjugated secondary antibody. Antibodies go through a validation process as previously described (Hennessy et al., 2010) to assess specificity, quantification and dynamic range. Each of the 183 primary antibodies was assigned a label based on this validation process (at the time the assay was performed): "validated," "use with caution" or "under evaluation" (see Table S1). Samples were split across three batches and some antibodies were used only in a subset of these batches (Table S1). A DakoCytomation-catalyzed system and DAB colorimetric reaction was used to capture signal. Following scanning of slides, spot intensities were analyzed and quantified using Microvigene software (VigeneTech). The EC_{50} values of the proteins in each dilution series were estimated using the SuperCurve software (Coombes et al., 2012), available at http://bioinformatics.mdanderson.org/Software/supercurve/. This uses the non-parametric, monotone increasing B-spline model (Hu et al., 2007) to fit a single curve ("supercurve") using all samples on a slide, with signal intensity as the response variable and dilution step as the independent variable. The fitted curve is plotted with the signal intensities on the y axis and the $\log_2$ protein concentrations on the x axis for diagnostic purposes. A quality control (QC) metric, between zero and one was calculated for each slide (Coombes et al., 2012) and slides with values less than 0.8 were excluded. Within each batch, measurements were normalized for protein loading by median centering across antibodies (Liu et al., 2014; Li et al., 2013). This normalization process, performed on $\log_2$ concentrations, comprised the following steps:

1. For each antibody, calculate the median across samples and subtract from each value (i.e., median-center each antibody).
2. For each sample, calculate the median across antibodies to obtain a correction factor (CF).
3. For each sample, take the original $\log_2$ concentration values and subtract the corresponding CF (from step 2).

Normalized values, on a linear scale, are provided in Data S1.

Western Blot Analysis

Cells were grown as described above. For the novel edge validations in Figure 6, additional inhibitors were used to generate lysates following the protocol laid out above. The inhibitors, all used at 1 μM, were AZD7762, KX2-391, PF4708671, and VX-702 (see Figure 6 for targets). In experiments designed to test the range of concentrations that were effective, 1 μM was able to trigger changes in the phosphorylation of proteins downstream of the inhibitor targets. Lysates were harvested 15 min after stimulation and protein concentrations quantified as described above. Denatured lysates were separated by PAGE on 4%–12% Bis-Tris gradient gels (Invitrogen) along with Precision Plus Protein Standards (Bio-Rad) using MOPS SDS NuPAGE Running Buffer (Invitrogen) and NuPAGE LDS Sample Buffer (Invitrogen) on ice at 200 V for 45 min. Gels were transferred to immobilin-FL PVDF membranes (Millipore) using NuPAGE Transfer Buffer (Invitrogen) on ice at 30 V for 1.5 hr before being washed 3x 5 min. with 5% Tween-TBS (TTBS, Amresco & Invitrogen) at room temperature (RT) with agitation and blocked with 5% BSA (Sigma) in TTBS for 1 hr at RT with agitation. Blots were again washed 3x 5 min in TTBS at RT with agitation before being incubated in primary antibody in 5% BSA in TTBS overnight at 4C with agitation. Blots were washed 3x for 5 min at RT with agitation and then transferred to HRP-conjugated secondary antibody in 5% BSA in TTBS and incubated at RT for 1.5 hr. See Table S5 for primary antibodies and HRP-conjugated secondary antibodies used in western blot validations. Blots were washed again as previously described and visualized using SuperSignal West Pico Chemiluminescent HRP Substrate Kit (Thermo Scientific) and CL-X Posure Film (Thermo Scientific) and changes in protein abundance under inhibition were determined by visual inspection of exposed film.

Quality Control and Preprocessing of RPPA Data

Batch Normalization Procedure for Cell Line UACC812

The UACC812 data were split across two RPPA experiments with each batch containing different inhibitors (BEZ235, PD173074 and GSK690693&GSK1120212 in one batch; AZD8055 and GSK690693 in the other). DMSO control samples were common to both batches. The two batches were combined and normalized to obtain a single dataset for UACC812.

The steps of the batch normalization procedure were as follows:

1. Any antibodies not included in both batches were removed.

For each antibody, perform steps 2 and 3 below.

2. Using $\log_2$-transformed data (after normalization for protein loading; see above), the mean and standard deviation of the DMSO samples in each batch were calculated, giving values (μ_1, σ_1) and (μ_2, σ_2) for batch 1 and batch 2 respectively. Note that, for each batch, there are 16 replicates for DMSO, 0min samples (all other DMSO conditions consist of a single replicate). These 16 replicates were averaged prior to calculation of mean values and standard deviations.

3. All samples in batch 2 (for the given antibody) were then scaled and centered so that the mean and standard deviation of the batch 2 DMSO samples agreed with the corresponding batch 1 quantities (μ_1, σ_1). That is, a sample in batch 2 with value x became

$$\mu_1 + \frac{\sigma_1(x - \mu_2)}{\sigma_2}.$$

This scaling and centering was applied to each individual replicate and not to replicate-averaged data.

4. The two batches were then combined to get a single dataset for UACC812.

Data for the two batches and the final normalized dataset are provided in Data S1 on a linear scale.

Samples Excluded from Analyses

Samples identified as outliers were excluded from our analyses. These samples were identified using the following criteria:

- Normalization for protein loading resulted in a correction factor (CF) for each sample (see above). Samples with CF > 2.5 or CF < 0.25 were regarded as outliers.
- Variance across all antibodies was calculated for each sample. Values greater than 40 were regarded as outliers.
- We used the replicates at time $t = 0$ to calculate the signal-to-noise ratio (SNR) for each cell line and phosphoprotein antibody under each inhibitor (mean of replicates divided by standard deviation of replicates). The mean across all calculated SNRs was 10.68 (s.d. = 5.8). SNR values less than 1 were investigated further to determine whether the poor SNR was caused by outlier replicates.

For cell line UACC812, these criteria were applied to the batch-normalized dataset.

In addition to the above, data for the combination of inhibitors GSK690693 & GSK1120212 (AKTi & MEKi) for cell lines BT549 (all stimuli) and BT20 (PBS and NRG1 stimuli only) were excluded since none of the expected effects of MEKi were observed in these samples.

All samples excluded from analyses are shown in Table S6 and also indicated in the data files in Data S1.

Antibodies Included in Analyses

To facilitate comparisons between cell lines, the analyses presented here focused on the set of phosphoprotein antibodies common to all four lines. This set contained two highly correlated pairs of antibodies ($r > 0.9$ for all cell lines), consisting of phosphoforms of the same protein: GSK3αβ_pS21_pS9, GSK3_pS9 and S6_pS235_S236, S6_pS240_S244. Since highly correlated variables can lead to a reduction in the utility of network inference results, only one antibody out of each pair was included in analyses, resulting in a final set of 35 phosphoprotein antibodies. A full list of antibodies can be found in Table S1, where the 35 phosphoproteins included in the analyses are also indicated.

Final Preprocessing Steps

Data were $\log_2$ transformed and replicates (only present for $t = 0$ samples and some DMSO samples) were averaged. Prior to input into our network inference pipeline, imputation was performed for missing data by linear interpolation of adjacent time points.

Identification of Changes Under Kinase Inhibition

We used a procedure centered on paired t tests to determine which phosphoproteins show a salient change in abundance under each kinase inhibitor. Details are described in Hill et al. (2016), but also outlined below for completeness.

For each phosphoprotein, inhibitor regime and *(cell line, stimulus)* context, a paired t test was used to assess whether mean phosphoprotein abundance under DMSO control is significantly different to mean abundance under the inhibitor regime (mean values calculated over seven time points). As discussed above, some phosphoproteins show a clear response to the stimulus under DMSO control, with abundance increasing and then decreasing over time (a "peak" shape), while others show a less clear response due to signal already being present prior to stimulus. For phosphoproteins falling into the former category (according to a heuristic), paired t tests were repeated, but this time restricted to intermediate time points within the peak. This focuses on the portion of the time course where an inhibition effect, if present, should be seen. The *p-value* from the repeated test was retained if smaller than the original *p-value*. For each *(cell line, stimulus)* context and inhibitor regime, the resulting set of *p-value*s (one *p-value* for each phosphoprotein) were corrected for multiple testing using the adaptive linear step-up procedure for controlling the FDR (Benjamini et al., 2006).

For each *(cell line, stimulus)* context, a phosphoprotein was deemed to show a salient change under a given inhibitor regime if two conditions were satisfied. First, the corresponding FDR value had to be less than 5% and, second, the effect size ($\log_2$ ratio between DMSO control and inhibitor conditions) had to be sufficiently large relative to replicate variation (see Figure S2). The latter condition is an additional filter to remove small effects. Replicate variation was quantified by calculating the pooled replicate standard deviation at each time point of the DMSO and inhibitor time courses, and then averaging these values across time points. The phosphoproteins satisfying these criteria are depicted in Figures 3B, 4A, and S1–S3. We note that the overall procedure is heuristic and that the FDR values should not be interpreted formally.

A phosphoprotein p showing a salient change under an inhibitor is consistent with a node targeted by the inhibitor having a causal effect on the phosphoprotein. Since this effect can be direct or indirect, phosphoprotein p can be regarded as a descendant of the inhibitor target node in the underlying signaling network. That is, there exists a directed path starting from the node targeted by the inhibitor and ending at phosphoprotein p.

Network Learning

Networks were learned for each of the 32 *(cell line, stimulus)* contexts using dynamic Bayesian networks (DBNs), a type of probabilistic graphical model for time-course data (see e.g., Hill et al., 2012; Husmeier, 2003; Murphy, 2002). Specifically we used a recently proposed variant called *interventional DBNs* or iDBNs (Spencer et al., 2015), that uses ideas from causal inference (Pearl, 2009; Spirtes et al., 2000) to model interventions and thereby improve ability to infer causal relationships; model specification followed Spencer et al. (2015). Although interested in learning context-specific networks, we expect a good proportion of agreement between contexts. Therefore, rather than learn networks for each context separately, we used a recently developed joint learning approach to solve all the problem instances together (Oates et al., 2014). A prior network was used (Figure S4); this was curated manually with input from literature (Weinberg, 2013) and online resources. The extent to which context-specific networks are encouraged to agree with each other and with the prior network is controlled by two parameters, λ and η respectively, as described in detail in Oates et al. (2014). These parameters were set (to $\lambda = 3$ and $\eta = 15$) by considering a grid of possible values and selecting an option that provides a reasonable, but conservative amount of agreement, allowing for discovery of context-specific edges that are not in the canonical prior network. The network learning approach resulted in a score (edge probability) for each possible edge in each context-specific network. The network estimates were robust to moderate data deletion and precise specification of the biological prior network and its strength (Figure S6). Furthermore, the analyses were computationally efficient, requiring approximately 30 min to learn all 32 context-specific networks using serial computation on a standard personal computer (Intel i7-2640M 2.80GHz processor, 8GB RAM).

Assessing Performance of Causal Network Learning

The ability of our network learning approach to estimate context-specific causal networks was systematically assessed using a train and test scheme proposed by Hill et al. (2016) in the context of the HPN-DREAM network inference challenge associated with the RPPA data presented here. Due to factors specific to the challenge setting, Hill et al. (2016) used only a single iteration of train and test. In contrast, we were able to perform several iterations, as described below.

In each iteration, the data were divided into two sets: (i) a test dataset, consisting of time courses for all 32 *(cell line, stimulus)* contexts under a *single* inhibitor regime, and (ii) a training dataset, consisting of time courses (again for all 32 contexts) for a subset of the remaining five inhibitor regimes (Figure S5A). We refer to the single inhibitor regime in the test data as the *test inhibitor* (although note that one regime contains more than one kinase inhibitor: GSK690693 & GSK1120212). Thirty-two context-specific networks were learned on the training dataset and then each network was assessed as to how well it agreed with changes observed, for the same context, under the test inhibitor (in the test dataset). For each test inhibitor, the set of phosphoproteins that show, for a given context, a salient change in abundance were determined as described above (and shown in Figures 4A and S3), resulting in context-specific "gold-standard" descendant sets. We then compared, for each context, predicted descendants of the test inhibitor target node(s) according to the network inferred from training data, against the corresponding "gold-standard" descendant set. This resulted in a number of correctly predicted descendants (true positives, TPs) and a number of incorrectly predicted descendants (false positives, FPs). Our network learning approach outputs edge probabilities, from which a network can be obtained using a threshold value. The TP and FP values were therefore a function of this threshold value, resulting in an ROC (receiver operating characteristic) curve. Our final assessment metric was then the area under this curve (AUROC), which we calculated for each context and test inhibitor (Figure S5B). The statistical significance of the AUROC scores was determined using an empirical null distribution, generated by calculating AUROC scores for sets of uniformly random edge probabilities.

The assessment procedure requires that nodes targeted by the test inhibitor are present in the network model so that their descendants can be determined. Also, it is important that the training data only contains inhibitor regimes that target nodes which are not also targeted by the test inhibitor. There were three train and test data splits that satisfied these criteria (while also maximizing the sample size of the training dataset), and we assessed performance for all three (Figure S5B).

QUANTIFICATION AND STATISTICAL ANALYSIS

Replicates were averaged prior to carrying out statistical analysis and the time courses shown in Figures 3 and S1 were plotted using replicate-averaged data.

The number of replicates were as follows: ~16 replicates for samples at $t = 0$, except for UACC812, BT20 and MCF7 DMSO $t = 0$ samples which were replicated ~32 times; 2 replicates for the majority of DMSO samples at $t > 0$, except for BT20 DMSO samples; all other samples had a single replicate.

Details of statistical procedures are provided in the methods section above or in figure legends. Analyses were performed using MATLAB R2012a software.

DATA AND SOFTWARE AVAILABILITY

Software

Scripts for the computational and statistical analyses presented here are available at https://github.com/Steven-M-Hill/causal-signaling-networks-CellSystems2016. These scripts include identification of changes under kinase inhibition, network learning, and assessment of network learning performance.

Data Resources

RPPA data, including additional time points and antibodies that were not used in the analyses presented here, are provided in Data S1. Time-course plots for all of the antibodies are provided in Data S2.

ADDITIONAL RESOURCES

HPN-DREAM network inference challenge associated with the RPPA data presented here: https://www.synapse.org/HPN_DREAM_Network_Challenge.

Cell Systems, Volume *4*

Supplemental Information

Context Specificity in Causal Signaling Networks

Revealed by Phosphoprotein Profiling

Steven M. Hill, Nicole K. Nesser, Katie Johnson-Camacho, Mara Jeffress, Aimee Johnson, Chris Boniface, Simon E.F. Spencer, Yiling Lu, Laura M. Heiser, Yancey Lawrence, Nupur T. Pande, James E. Korkola, Joe W. Gray, Gordon B. Mills, Sach Mukherjee, and Paul T. Spellman

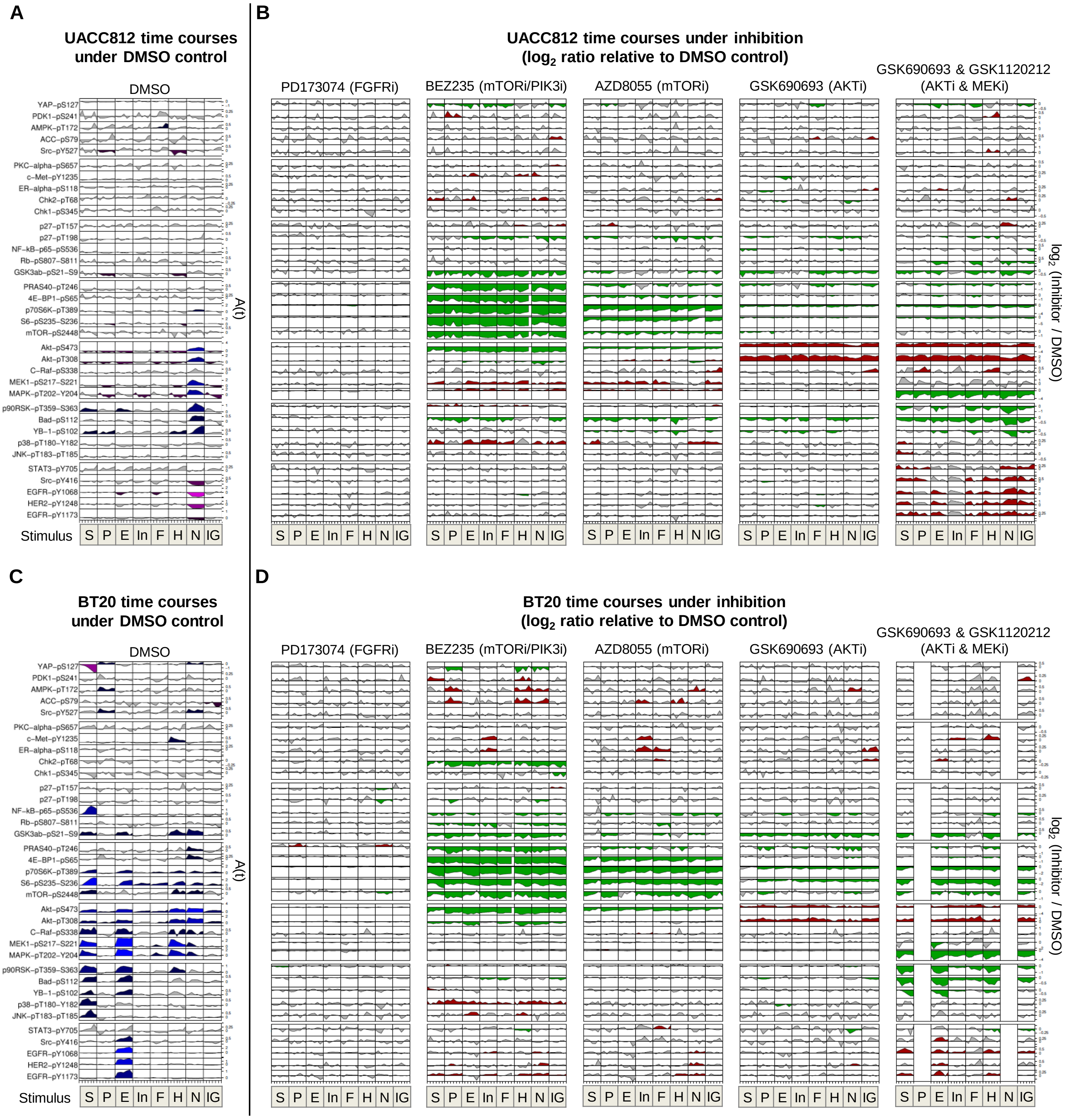

Figure S1 (continued on next page)

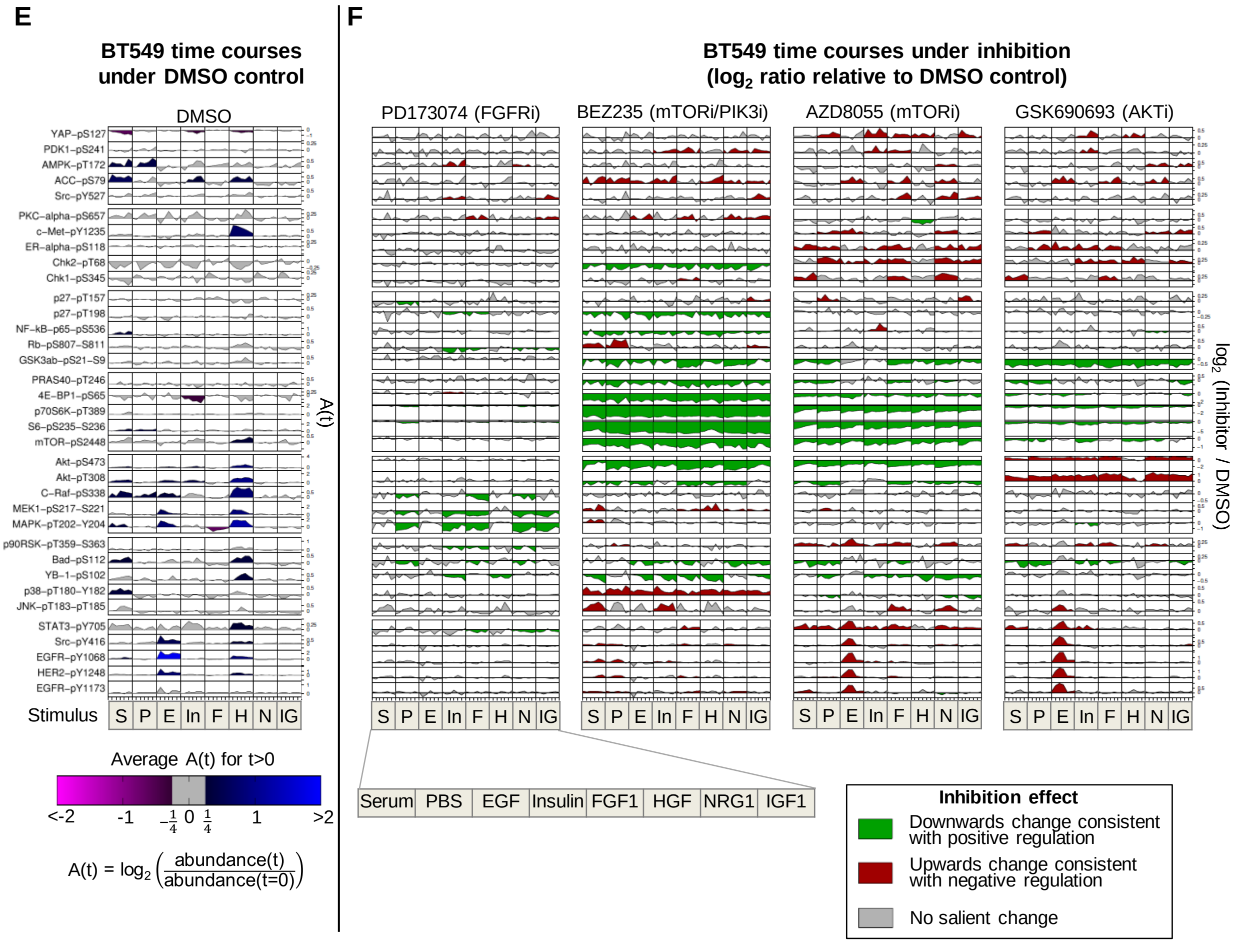

Figure S1. Phosphoprotein Time Course Data and Context-Specific Changes Under Inhibition for Breast Cancer Cell Lines UACC812, BT20 and BT549. Related to Figure 3.

(A,C,E) Phosphoprotein time courses under DMSO control for cell lines UACC812 (A), BT20 (C) and BT549 (E). Rows correspond to 35 phosphoproteins and columns correspond to the eight stimuli. Each time course shows $\log_2$ ratios of phosphoprotein abundance relative to abundance at $t = 0$. Shading represents average $\log_2$ ratio for $t > 0$. (B,D,F) Phosphoprotein time-courses under kinase inhibition for cell lines UACC812 (B), BT20 (D) and BT549 (F). Each of the five vertical blocks corresponds to a different inhibition regime. Within each block, rows and columns are as in (A,C,E). Each time course shows $\log_2$ ratios of phosphoprotein abundance under inhibition relative to abundance under DMSO control. Shading represents direction of changes in abundance due to inhibitor: Green denotes a decrease in abundance, red denotes an increase and gray denotes no salient change. See STAR Methods for details of statistical analysis. There is no data available for the AKTi & MEKi inhibition regime for the following contexts: *(BT549, all stimuli)* and *(BT20, PBS & NRG1)*. Plots were generated using a modified version of the DataRail software (Saez-Rodriguez, 2008). Each phosphoprotein is plotted on its own scale and phosphoproteins are ordered by hierarchical clustering of all data. See Figure 3 for corresponding plots for cell line MCF7.

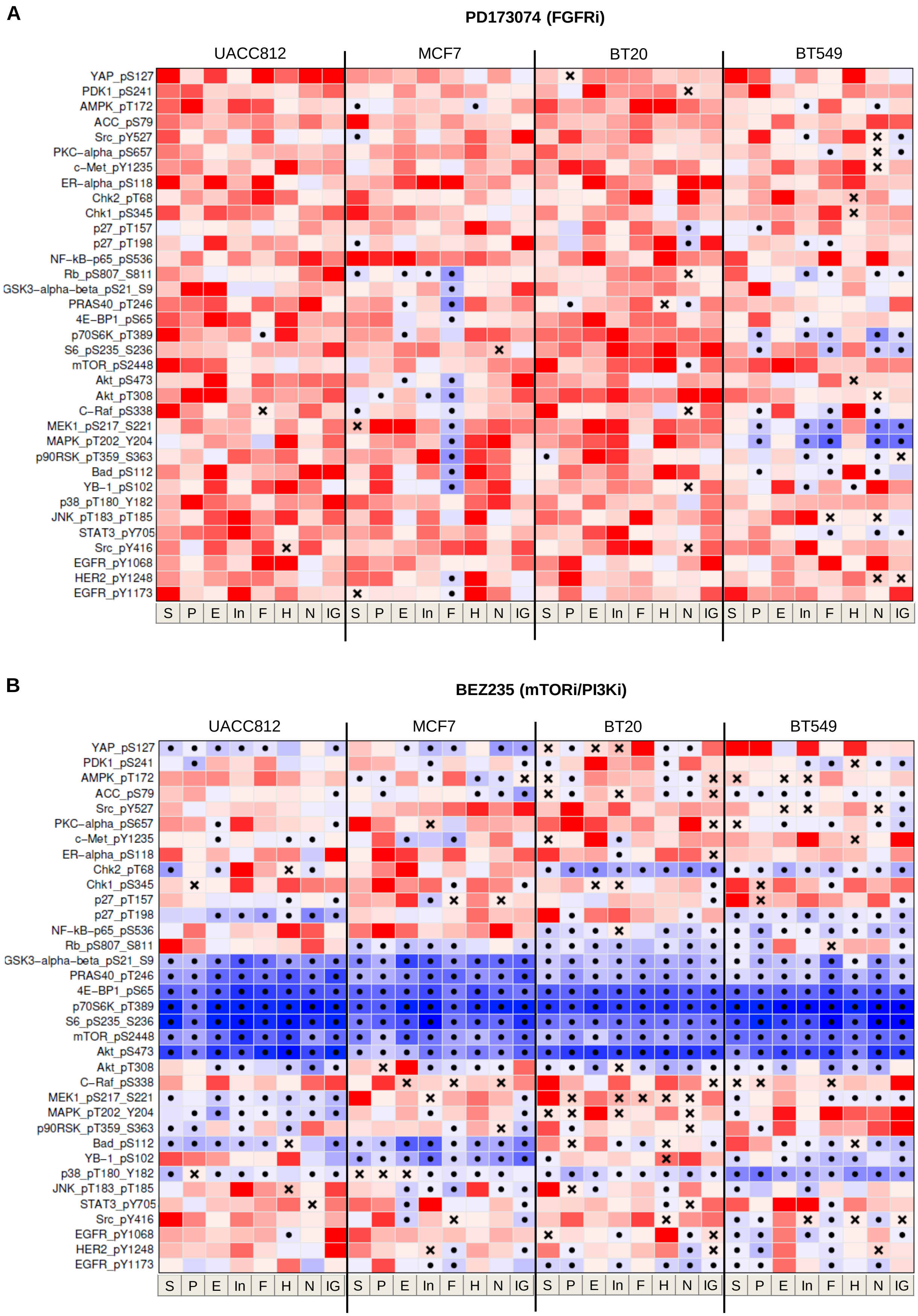

Figure S2 (continued on next page)

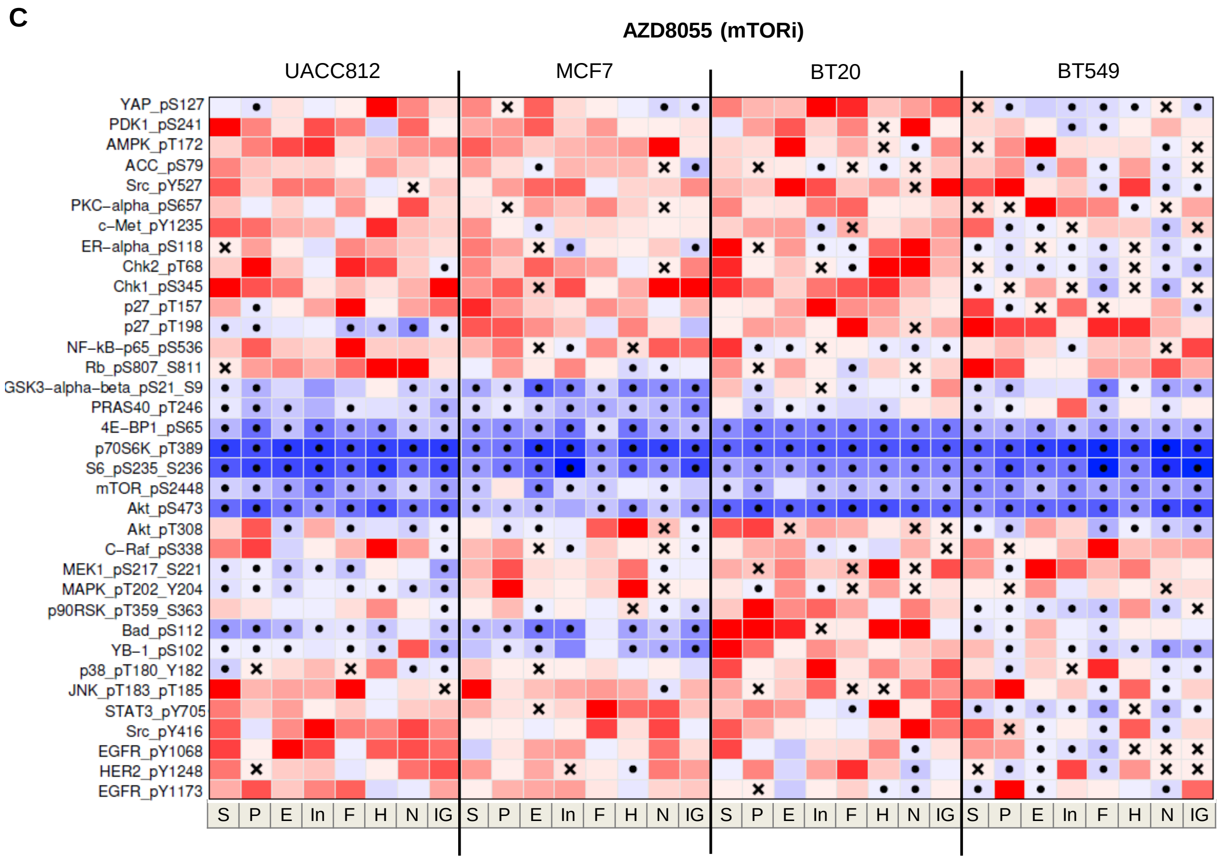

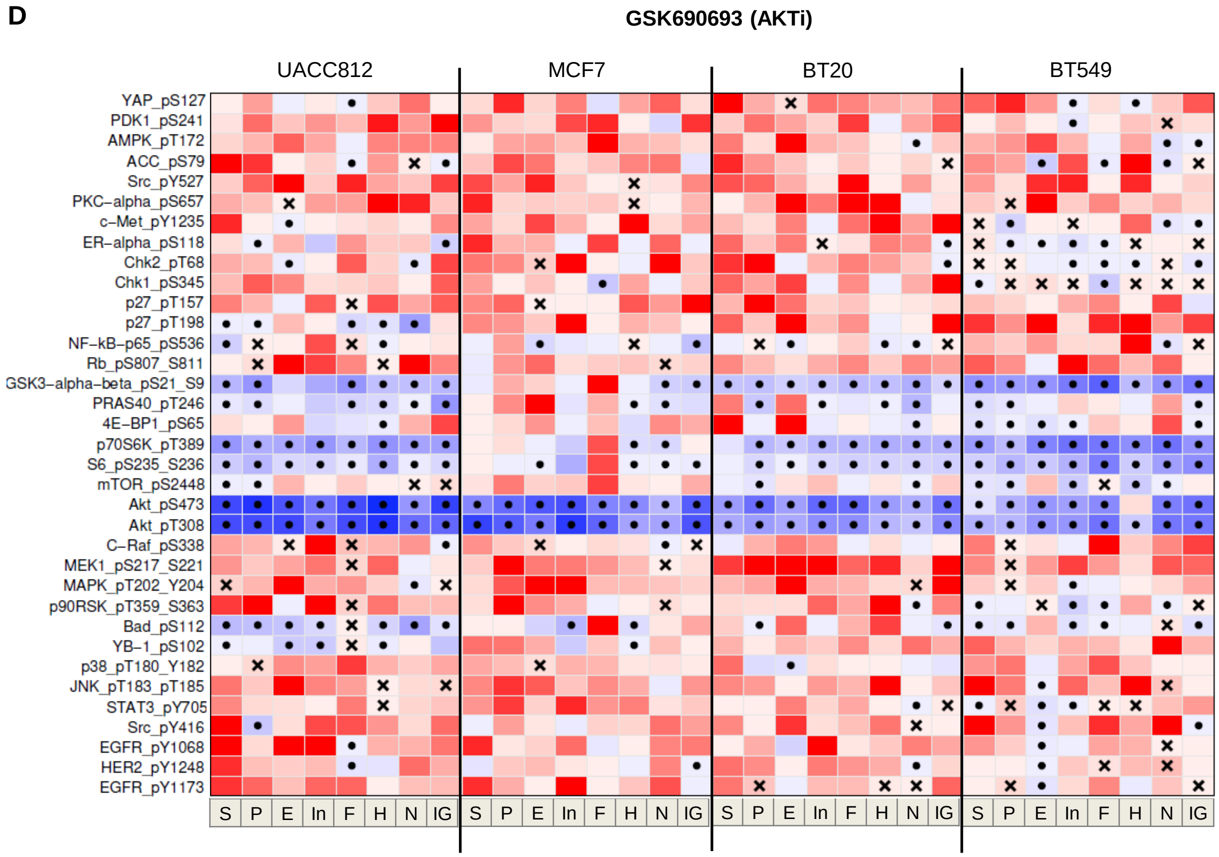

Figure S2 (continued on next page)

E

GSK690693 & GSK1120212 (AKTi & MEKi)

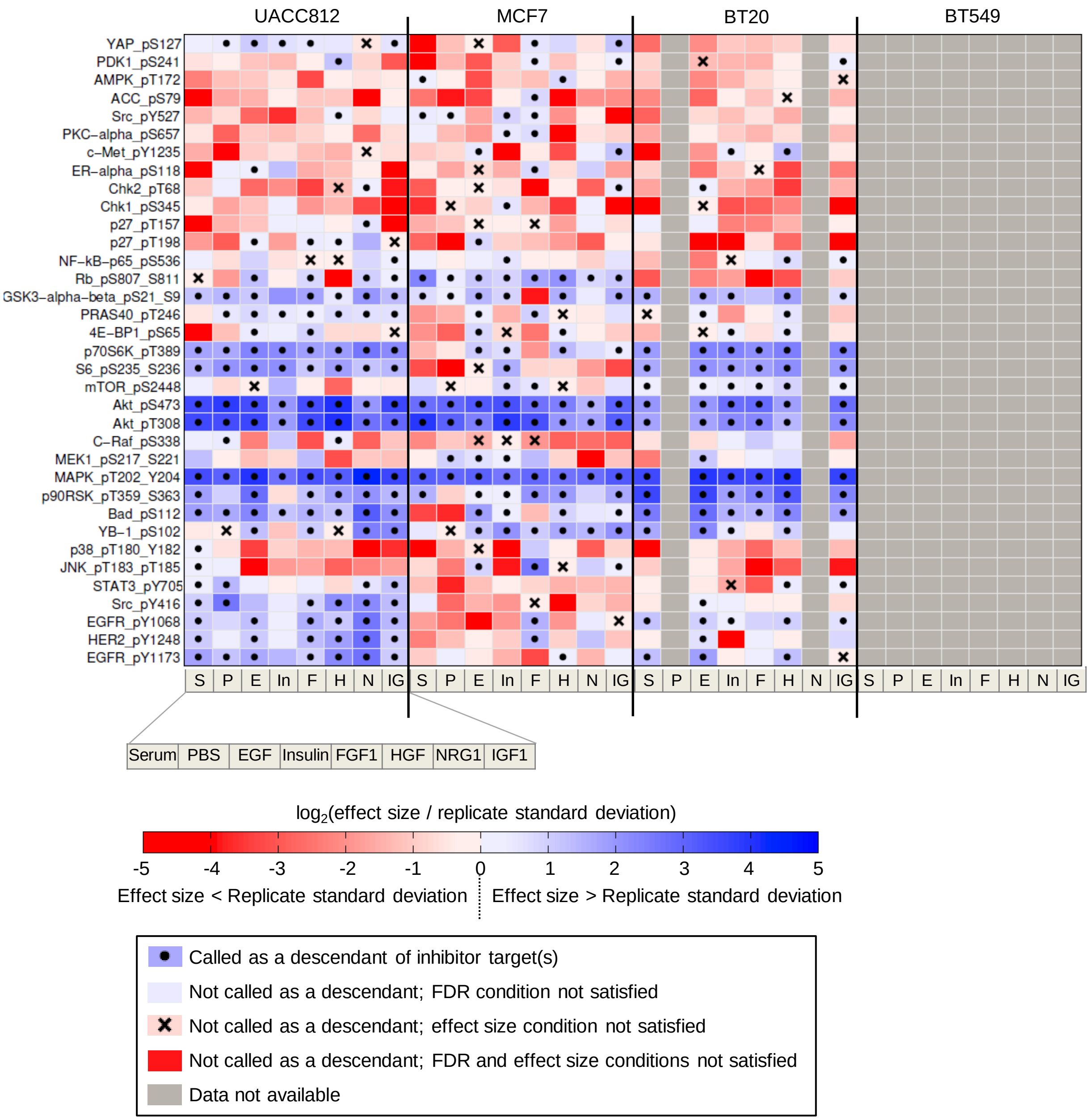

Figure S2. Change under inhibition effect sizes relative to replicate variation. Related to Figure 3. Log_2 ratio of change under inhibition effect size relative to replicate variation for each phosphoprotein in each of the 32 *(cell line, stimulus)* contexts under inhibition of: (A) FGFR (PD173074); (B) mTOR and PI3K (BEZ235); (C) mTOR (AZD8055); (D) AKT (GSK690693); (E) AKT and MEK (GSK690693 and GSK1120212). Rows corresponds to phosphoproteins and columns correspond to contexts. For a given context, a phosphoprotein was deemed to show a salient change under inhibition if two conditions were satisfied (see STAR Methods): (i) FDR < 5% (from paired *t*-test comparing DMSO and inhibitor time courses) and (ii) inhibition effect size > replicate standard deviation. Cells shaded blue indicate that condition (ii) is satisfied. Blue cells with black dots denote phosphoproteins that also satisfy condition (i) and are therefore called as causal descendants of (one of) the inhibitor target(s) in the signaling network for that context. Red cells with black crosses indicate phosphoproteins where condition (i) is satisfied only. Phosphoproteins ordered by output of hierarchical clustering of all data.

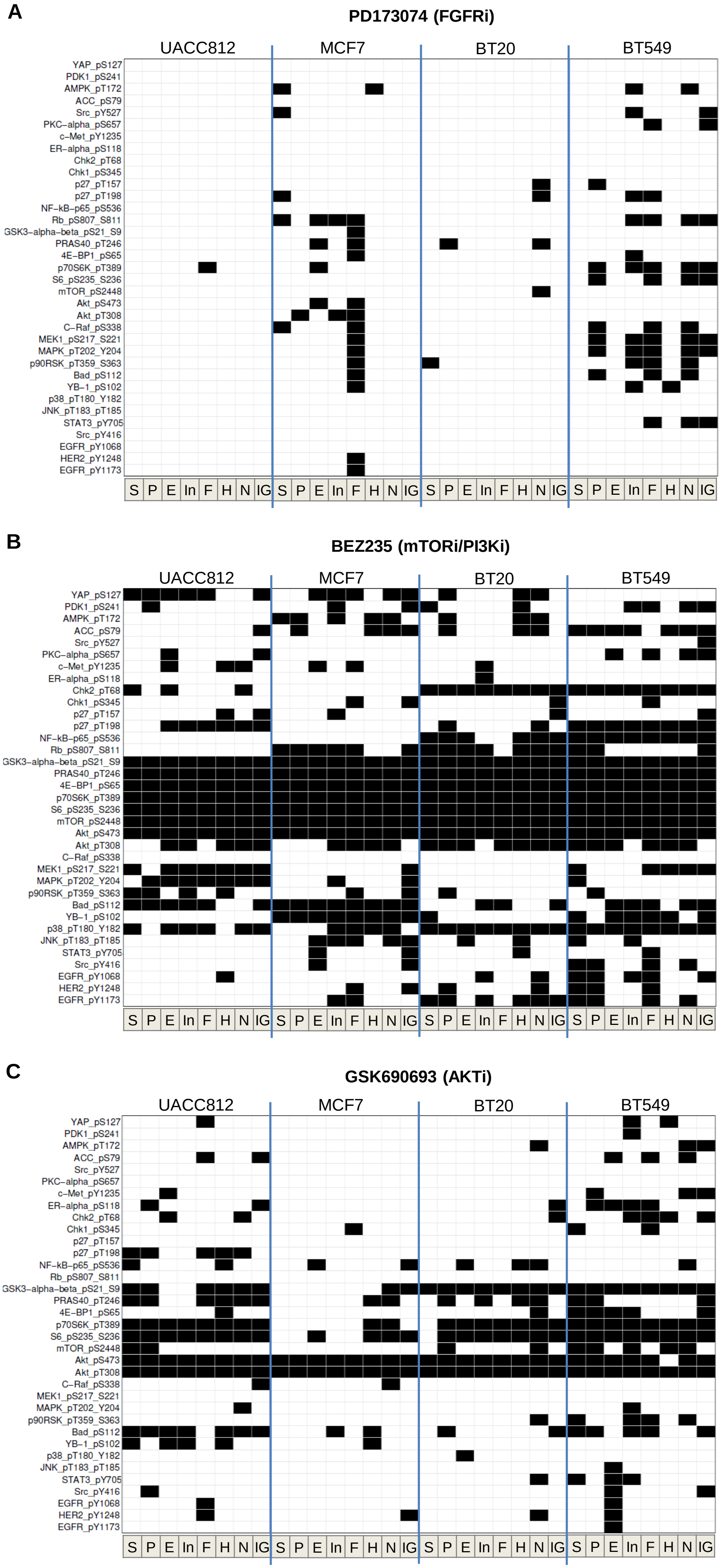

Figure S3 (continued on next page)

D

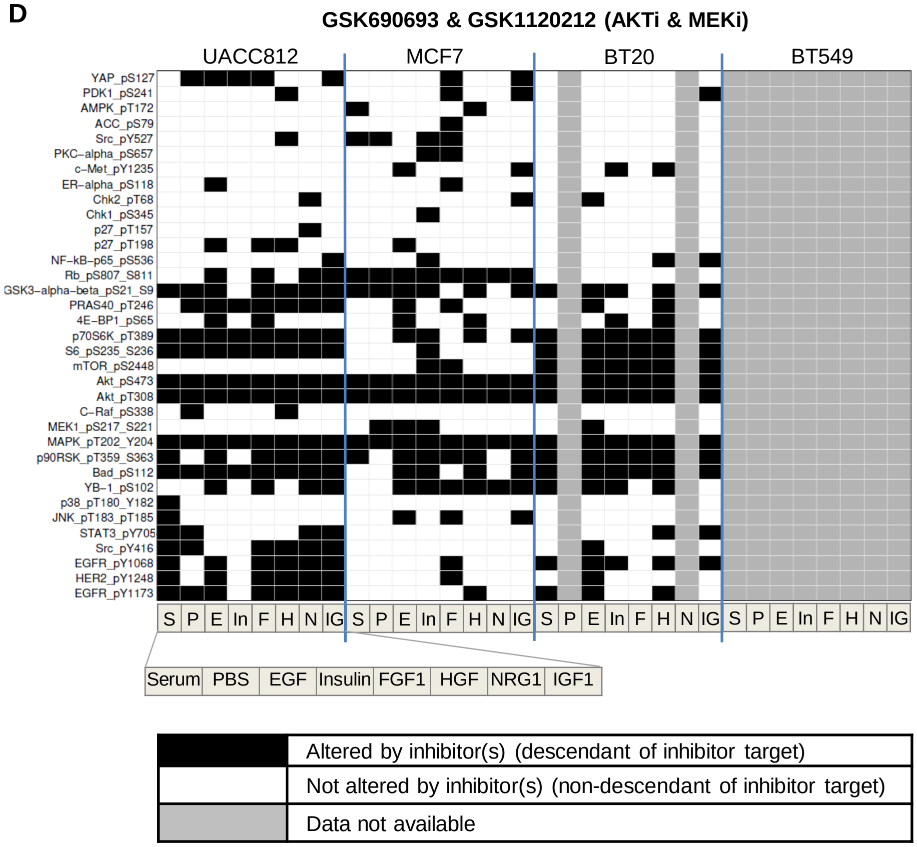

Figure S3. Context-Specific Signaling Revealed by Kinase Inhibition. Related to Figure 4.
Causal descendancy matrices showing causal effects observed in each of the 32 *(cell line, stimulus)* contexts under inhibition of: (A) FGFR (PD173074); (B) mTOR and PI3K (BEZ235); (C) AKT (GSK690693); (D) AKT and MEK (GSK690693 and GSK1120212). Rows correspond to phosphoproteins and columns correspond to contexts. Black cells indicate phosphoproteins that show a salient change under inhibition in a given context (see STAR Methods) and can therefore be regarded as causal descendants of (one of) the inhibitor target(s) in the signaling network for that context. Phosphoproteins ordered by output of hierarchical clustering of all data. Note that black cells correspond to blue cells with black dots in Figure S2. See Figure 4 for the causal descendancy matrix for mTOR inhibitor AZD8055.

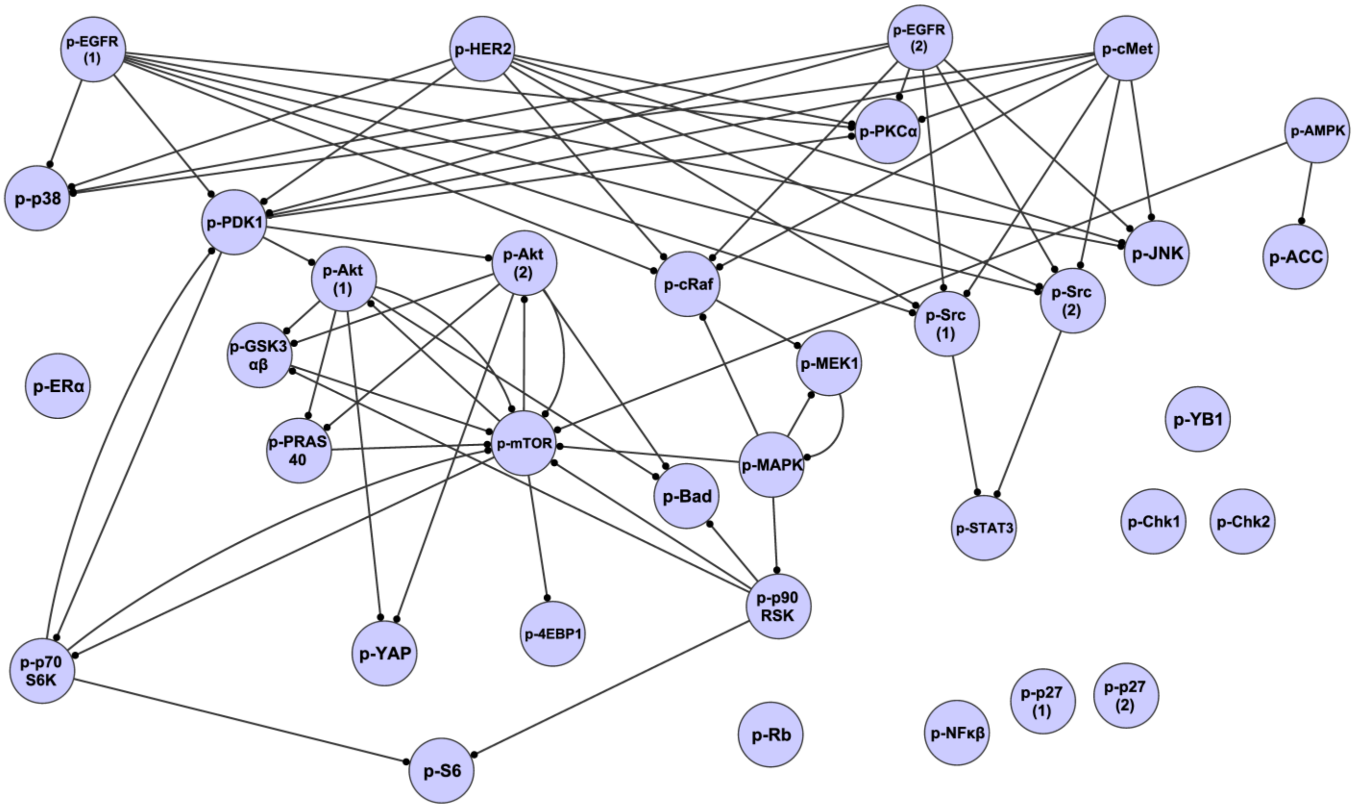

Figure S4. Prior Network Incorporated Within Network Learning. Related to Figure 5.
Known biology was incorporated into network learning using the prior network shown here. The same prior network was used for all contexts and contains canonical information manually curated with input from literature and online resources. Edges are directed with the child node indicated by a circle. The network includes edges describing indirect causal effects mediated via signaling components not included in our study. Full nodes names, including phosphorylation sites, are provided in Table S4.

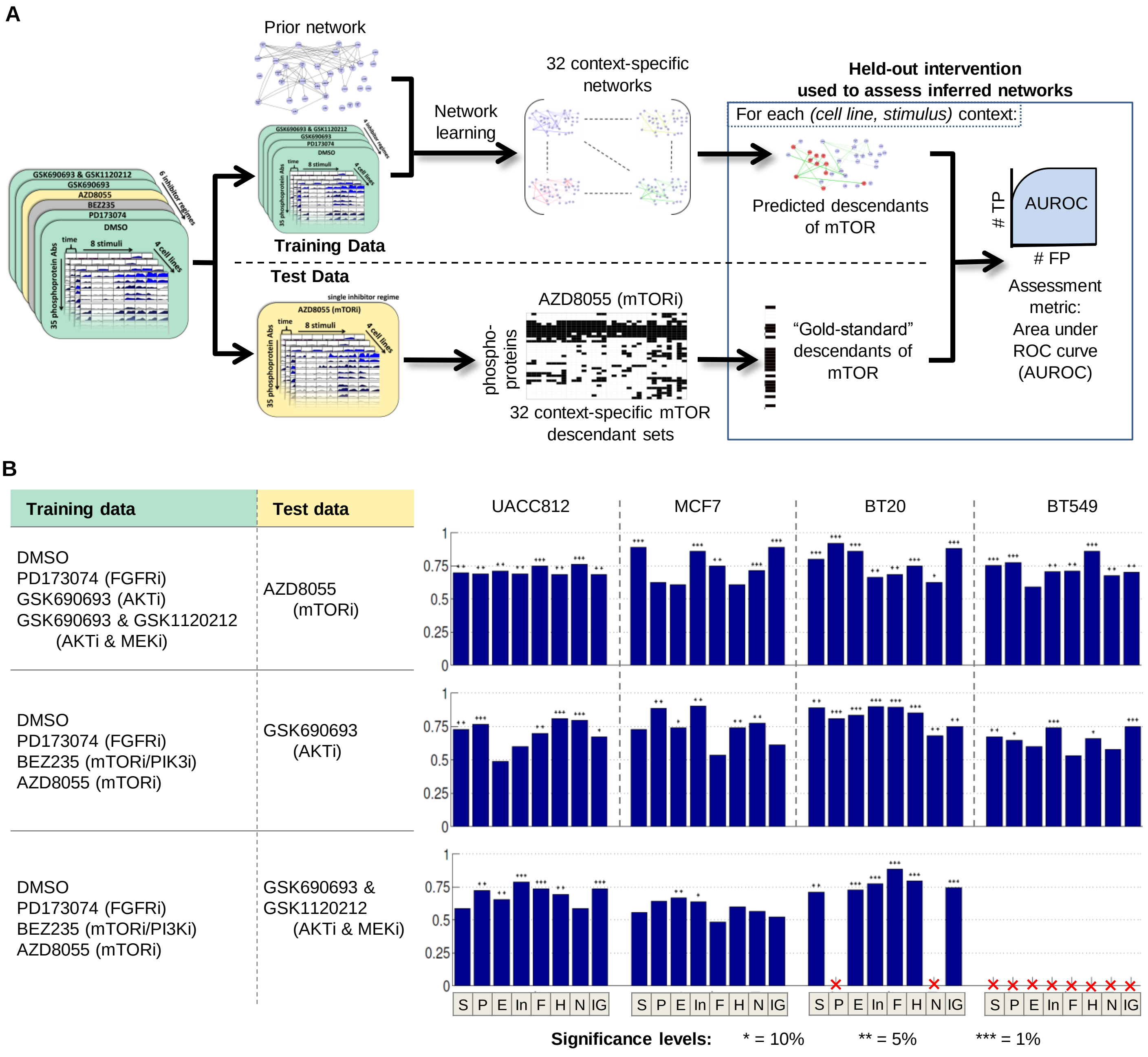

Figure S5. Assessment of Causal Network Learning. Related to STAR Methods.

(A) The ability of our approach to learn causal networks was assessed using an interventional train-and-test approach, following Hill et al. (2016). At each iteration, the data was split into a test set consisting of all data for a single inhibitor regime, and a training set comprising data for all other inhibition regimes (except those that target the same protein(s) as the test inhibitor). The example shown has AZD8055 (mTORi) as the test inhibitor. Thirty-two context-specific networks were inferred by applying the network learning procedure to the training data. The held-out test data were used to determine, for each context, descendants of the test set inhibitor target(s). These "gold-standard" descendant sets were then compared with descendant sets predicted by the inferred context-specific networks, resulting in an AUROC (area under the ROC curve) score for each context. See STAR Methods and Hill et al. (2016) for further details. (B) The procedure in (A) was repeated for three different train/test splits as shown in the table. The bar plots show the corresponding AUROC scores for the 32 contexts. Stars above the bars denote statistical significance of AUROC scores at the 10% (1 star), 5% (2 stars) and 1% (3 stars) level, with respect to a null distribution based on random edge scores. Red crosses indicate contexts where AUROC scores are unavailable due to missing data for the AKTi & MEKi inhibition regime.

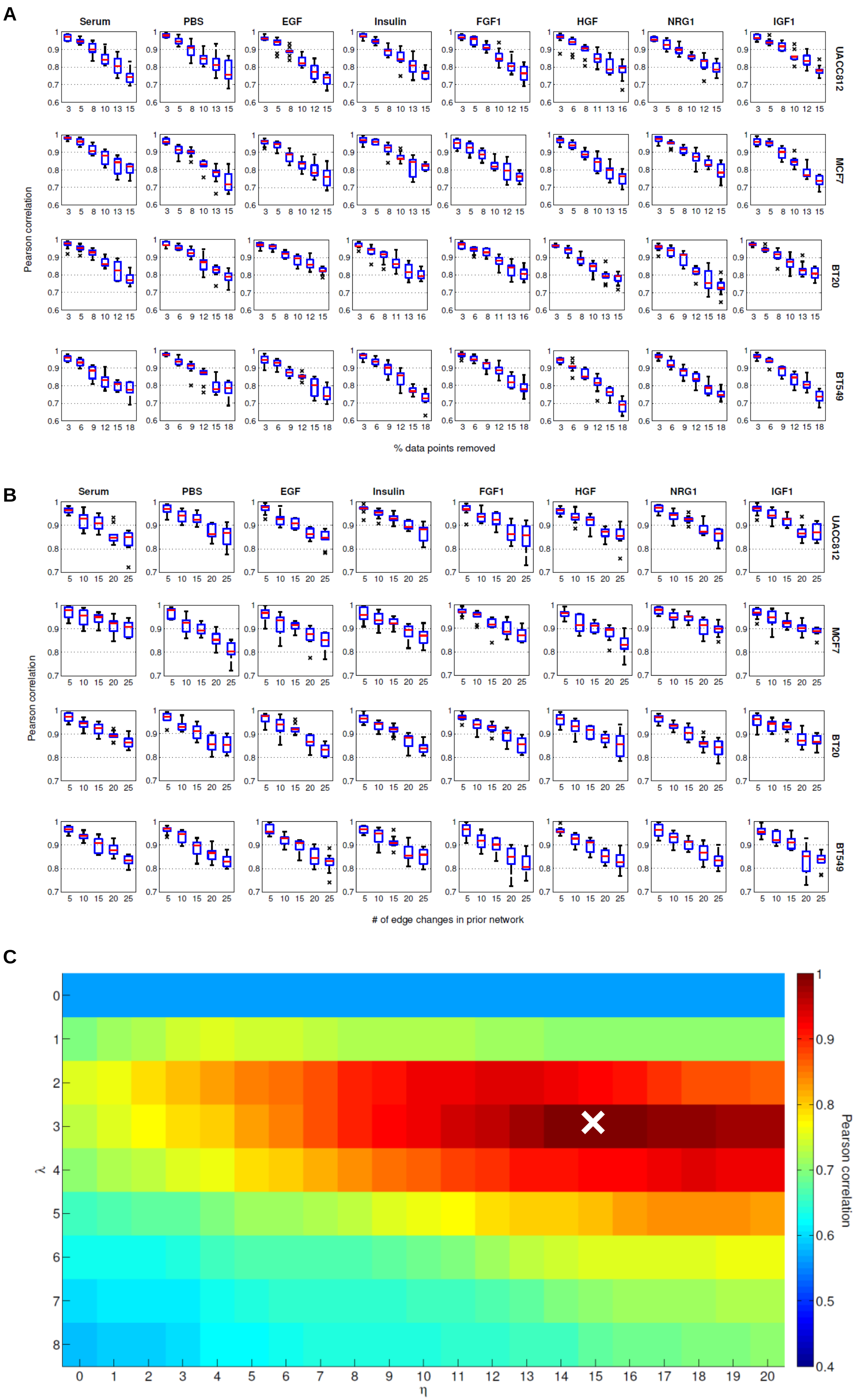

Figure S6 (legend on next page)

Figure S6. Sensitivity of Network Learning to Data Deletion and Prior Network Specification. Related to STAR Methods.

(A) Sensitivity to data deletion. For each *(cell line, stimulus)* context, data were deleted for all 35 phosphoproteins for between one and six time points, selected at random from the time courses for all available inhibitor regimes. Missing data were then imputed by linear interpolation using adjacent time points (for this reason the random selection for deletion excluded the first and last time points in each time series). For each context, Pearson correlation coefficients are shown between posterior edge probabilities inferred using the new, perturbed data sets and those obtained using the original data set. The *x*-axes show the percentage of data points removed. Results shown are over 10 iterations. (B) Sensitivity to prior network edge structure. The prior network (Figure S4) was perturbed by making random edge changes; an edge change consists of removing a randomly selected directed edge and then adding in (a different) randomly selected directed edge. Therefore the overall number of edges remains constant (65 edges). Edge probabilities obtained by incorporating the perturbed prior networks within network learning were compared with those obtained using the original, unperturbed prior network. For each context, Pearson correlations are shown as a function of number of edge changes, each for 10 iterations. (C) Sensitivity to prior strength parameters. Joint learning was done using the formulation in Oates et al. (2014). Parameters λ and η controlled the extent to which context-specific networks agreed with each other and with the prior network respectively. The heatmap shows average Pearson correlations between posterior edge probabilities reported in Main Text, obtained using $(\lambda = 3, \eta = 15)$ and those obtained using alternative (λ, η) values. Averages are calculated over the 32 *(cell line, stimulus)* contexts. The heatmap entry for $(\lambda = 3, \eta = 15)$ therefore has a value of unity and is indicated by a white cross. For $\eta = 0$, the prior network plays no role, while $\lambda = 0$ provides a flat prior distribution over networks (prior network plays no role and no agreement encouraged across contexts).

Table S1. RPPA Antibody List. Related to Figure 2 and STAR Methods.
Details of antibodies contained in each RPPA dataset. The presented analyses focused on phosphoprotein antibodies that were present across all three RPPA datasets, as indicated in the table.

(Supplied as an Excel file)

Table S2. Western Blot Validations of Context-Specific Changes Under Intervention. Related to Figures 4 and S3.
Western blots were carried out to validate a subset of the context-specific changes observed under kinase inhibition in the RPPA data (Figures 4A and S3). Each row in the table reports, for each of the four cell lines under a given stimulus, the change in abundance (relative to DMSO control) of a single phosphoprotein under a given inhibitor. Shown are: (i) changes in abundance determined using the RPPA time-course data (see STAR Methods), with green/red cells denoting a downwards/upwards effect under inhibition respectively and "NE" denotes no observed effect, and (ii) results of attempts to validate the RPPA observations by Western blot (see STAR Methods), with blue cells denoting observations that successfully validated and yellow cells denoting results that were inconsistent with the RPPA data (blank cells denote untested observations).

(Supplied as an Excel file)

Table S3. List of Directed Edges in Inferred Context-Specific Networks. Related to Figures 5 and 6.
A network specific to each of the 32 *(cell line, stimulus)* contexts is obtained by placing a threshold of 0.2 on the posterior edge probabilities that are the output of the network learning procedure. The table contains all directed edges that appear in at least one of these context-specific networks, together with edge probabilities for each context. Edges are sorted into three groups: (i) edges that are not in the prior network and are not self-edges (edges where the parent node is the same as the child node); (ii) edges that are in the prior network; (iii) self-edges (note that the prior network does not contain any self-edges). Within each of these groups, edges are sorted by average edge probability across all contexts. Also indicated are the edges appearing in the cell line-specific summary networks in Figure 5 and associated average edge probabilities (see columns with headings shaded blue). Gray cells denote (average) edge probabilities below a value of 0.2. Edges for which validation was attempted by Western blot (Figure 6) are highlighted in red. Summary counts for the number of edges in each context-specific network and percentage of "novel" edges (not in the prior network) are provided at the bottom of the table.

(Supplied as an Excel file)

Table S4. Full Antibody Names for Nodes in Network Visualizations. Related to Figures 4B, 5 and S4.

The networks shown in Figures 4B, 5 and S4 use abbreviated antibody names for node labels. The corresponding full antibody names are provided in this table.

Network node label	Full antibody name
p-4EBP1	4E-BP1_pS65
p-ACC	ACC_pS79
p-Akt (1)	Akt_pT308
p-Akt (2)	Akt_pS473
p-AMPK	AMPK_pT172
p-Bad	Bad_pS112
p-Chk1	Chk1_pS345
p-Chk2	Chk2_pT68
p-cMet	c-Met_pY1235
p-cRaf	C-Raf_pS338
p-EGFR (1)	EGFR_pY1173
p-EGFR (2)	EGFR_pY1068
p-ERα	ER-alpha_pS118
p-GSK3αβ	GSK3-alpha-beta_pS21_S9
p-HER2	HER2_pY1248
p-JNK	JNK_pT183_pT185
p-MAPK	MAPK_pT202_Y204
p-MEK1	MEK1_pS217_S221
p-mTOR	mTOR_pS2448
p-NFκβ	NF-kB-p65_pS536
p-p27 (1)	p27_pT157
p-p27 (2)	p27_pT198
p-p38	p38_pT180_Y182
p-p70 S6K	p70S6K_pT389
p-p90 RSK	p90RSK_pT359_S363
p-PDK1	PDK1_pS241
p-PKCα	PKC-alpha_pS657
p-PRAS 40	PRAS40_pT246
p-Rb	Rb_pS807_S811
p-S6	S6_pS235_S236
p-Src (1)	Src_pY527
p-Src (2)	Src_pY416
p-STAT3	STAT3_pY705
p-YAP	YAP_pS127
p-YB1	YB-1_pS102

Table S5. Antibody List for Western Blot Validations of Potentially Novel Edges. Related to Figure 6 and STAR Methods.
Details of the antibodies used in the western blot validations of potentially novel edges.

Desired Target	Company	Catalogue Number	Dilution Used
p27_pT198	Abcam	ab64949	1:500
p38_pT180/Y182	CST	9211	1:500
p70S6K_pT389	CST	9205	1:2,000
B-actin	Santa Cruz	sc-47778	1:30,000
Chk2_pT68	CST	2197	1:1,000
HSP90	BD Trans Labs	610419	1:20,000
JNK_pT183/T185	CST	4668	1:500
NFκβp65_pS653	CST	3033	1:500
YAP_pS127	CST	4911	1:500

Table S6. Samples Excluded from Analyses. Related to STAR Methods.
The table shows the samples excluded from analyses due to not passing quality control. For each sample, a justification for removal is provided (see STAR Methods for details). These samples are also highlighted in the data files in Data S1.

Cell line	Inhibitor	Stimulus	Time point	# replicates removed	Justification
MCF7	GSK690693	n/a	0	2 out of 16 replicates	Outlier (CF score)
UACC812	PD173074	n/a	0	1 out of 16 replicates	Outlier (CF score)
UACC812	GSK690693 & GSK1120212	Insulin	15min	1 out of 1 replicate	Outlier (variance)
UACC812	BEZ235	n/a	0	1 out of 13 replicates	Outlier (SNR analysis)
BT549	GSK690693 & GSK1120212	All	All	All	None of the expected effects of GSK1120212 (MEKi) were observed
BT20	GSK690693 & GSK1120212	PBS, NRG1	All	All	
BT20	GSK690693 & GSK1120212	n/a	0	4 out of 16 replicates	

Data S1. RPPA data. Related to STAR Methods.
A zip archive containing the reverse-phase protein array data generated in this study. See the README file included in the zip archive for further details.

Data S2. RPPA data time-course plots. Related to STAR Methods.
A zip archive containing time-course plots of the reverse-phase protein array data generated in this study. See the README file included in the zip archive for further details.
